# The BRD4 Inhibitor dBET57 Exerts Anticancer Effects by Targeting Superenhancer-Related Genes in Neuroblastoma

**DOI:** 10.1155/2022/7945884

**Published:** 2022-11-16

**Authors:** Si-Qi Jia, Ran Zhuo, Zi-Mu Zhang, Yang Yang, Yan-Fang Tao, Jian-Wei Wang, Xiao-Lu Li, Yi Xie, Gen Li, Di Wu, Yan-Ling Chen, Juan-Juan Yu, Chen-xi Feng, Zhi-Heng Li, Rong-Fang Zhou, Ran-Dong Yang, Peng-Cheng Yang, Bi Zhou, Xiao-Mei Wan, Yu-Meng Wu, Wan-Yan Jiao, Ni-Na Zhou, Fang Fang, Jian Pan

**Affiliations:** ^1^Institute of Pediatric Research, Children's Hospital of Soochow University, Suzhou 215003, China; ^2^School of Basic Medicine and Biological Sciences, Soochow University, Suzhou 215003, China; ^3^Department of Pediatric Surgery, Children's Hospital of Soochow University, Suzhou, Jiangsu 215025, China

## Abstract

Neuroblastoma (NB) is the most common solid tumor of the neural crest cell origin in children and has a poor prognosis in high-risk patients. The oncogene MYCN was found to be amplified at extremely high levels in approximately 20% of neuroblastoma cases. In recent years, research on the targeted hydrolysis of BRD4 to indirectly inhibit the transcription of the MYCN created by proteolysis targeting chimaera (PROTAC) technology has become very popular. dBET57 (S0137, Selleck, TX, USA) is a novel and potent heterobifunctional small molecule degrader based on PROTAC technology. The purpose of this study was to investigate the therapeutic effect of dBET57 in NB and its potential mechanism. In this study, we found that dBET57 can target BRD4 ubiquitination and disrupt the proliferation ability of NB cells. At the same time, dBET57 can also induce apoptosis, cell cycle arrest, and decrease migration. Furthermore, dBET57 also has a strong antiproliferation function in xenograft tumor models *in vivo*. In terms of mechanism, dBET57 targets the BET protein family and the MYCN protein family by associating with CRBN and destroys the SE landscape of NB cells. Combined with RNA-seq and ChIP-seq public database analysis, we identified the superenhancer-related genes TBX3 and ZMYND8 in NB as potential downstream targets of dBET57 and experimentally verified that they play an important role in the occurrence and development of NB. In conclusion, these results suggest that dBET57 may be an effective new therapeutic drug for the treatment of NB.

## 1. Introduction

Neuroblastoma (NB) is one of the most common neuroectodermal solid tumors in children [1, 2]. It occurs in infancy and early childhood and is derived from primitive cells, mainly neural crest cells. In the past five years, the prognosis of the low-risk group has been good, with a survival rate of more than 95%, whereas that of high-risk patients is only 40% [3]. Studies have found that MYCN oncogene is an extremely high-level amplification observed in approximately 20% of neuroblastoma cases [4]. Hence, a comprehensive understanding of the mechanisms of proliferation and differentiation may contribute to better targeting to modulate the molecular pathogenesis of neuroblastoma, obtaining the maximum benefit from treatment [5].

MYCN belongs to the MYC protooncogene family [6]. The family and its extended protein network play very important roles in regulating several processes, including cellular self-renewal, apoptotic resistance and metabolic flexibility. In neuroblastoma, abnormal expression of MYCN is the genetic aberration most consistent with treatment failure and poor prognosis [7]. However, the inherently disordered structure of MYC proteins and an incomplete understanding of how MYC acts as a master regulator of the transcriptome to drive tumorigenesis impede traditional drug discovery [8]. Therefore, alternative strategies have been developed to regulate MYC by epigenetic regulation of its transcription or modification of its target genes [9]. For example, BET bromodomain inhibition can target MYC transcription and activate downstream transcription programs, and it shows promise as a new strategy for drug discovery and development.

Bromodomain and ectodomain (BET) proteins function as epigenetic readers to regulate gene expression and are characterized by two tandem bromodomains (BD1 and BD2), a C-terminal domain, and an extraterrestrial domain [10]. This family includes BRD2, BRD3, BRD4 [11], and testis-restricted BRDTs. Among them, the BRD4 protein is anchored in the promoter region and is responsible for recruiting P-TEFb. It recognizes side-chain acetylated lysines on superenhancer open chromatin [12] and is involved in regulating gene transcription, affecting cancer pathogenesis. More specifically, targeted therapy of BRD4 opens up new possibilities for strategies with potent antitumor activity. In the past few years, dozens of BRD4 inhibitors have been clinically tested. The results have shown that the limited and positive benefits of single-drug activity were offset by high-drug toxicity. Therefore, it is necessary to develop new hemichemical sensitizers or new-generation compounds that are expected to enhance the therapeutic value of targeting BET proteins.

Proteolysis-targeting chimaera (PROTAC) [13] is heterobifunctional molecules that can not only bind to the target protein but also to E3 ubiquitin (E3) ligase. Mechanistically, PROTACs induce ubiquitination and degradation of target proteins by recruiting E3 ligases to target proteins [14]. In contrast to small molecule inhibitors, PROTACs are distinguished by low toxicity, targeting nondruggable proteins and specific degradation of target proteins. dBET1, developed by PROTAC technology, is an inhibitor with phthalimide and JQ1 [15] as ligands [16]. Precise targeting of BET proteins can induce BRD2 and BRD3 degradation. It has been reported that the inhibitor has a good effect on killing tumor cells in a variety of cancers (such as colon cancer, lung cancer, ovarian cancer, and breast cancer), but it has not yet achieved the purpose of maximizing the drug benefit [17].

dBET57 is a novel and potent heterobifunctional small molecule degrader based on PROTAC technology. It recruits the proteasome system to target the BRD4BD1 protein for degradation through ligand-dependent CRBN/BRD4 interactions [18]. However, thus far, the efficacy of dBET57 against neuroblastoma remains uncertain. Therefore, this study is aimed at exploring the antitumor activity of dBET57 on neuroblastoma, and further study of the mechanism is warranted to determine the therapeutic value of this inhibitor.

## 2. Materials and Methods

### 2.1. Cell Culture

Human NB cell lines, including SK-N-BE(2), IMR-32, and SH-SY5Y, and normal cell lines, including HPAEC and HCAEC (vascular endothelial cell), 293T (human embryonic renal cells), and MT22 (neuron cell), were purchased from the Chinese Academy of Sciences cell Bank in recent years and identified by short tandem repeat analysis within 3 years. DMEM, MEM, and RPMI 1640 (Biological Industries, Israel) were supplemented with 10% fetal bovine serum (Dongling Biotech, Soochow, China) and 1% penicillin-streptomycin (Millipore Sigma, Darmstadt, Germany) to become complete medium. NB cells were cultured in a humidified incubator with an atmosphere of 5% CO2 at 37°C, and the routine detection did not contain mycoplasma.

### 2.2. Vector Construction and Infection

In lentivirus infection, the envelope and packaging plasmids pMD2.g (Cat: 12259, Addgene) and psPAX2 (Cat: 12260, Addgene) and constructed target plasmids include CRBN, TBX3, and ZMYND8. 293FT cells were cotransfected with the help of PEI (linear MW25,000 Da, 5 mg/mL, pH 7.0). After transfection to 6 h, fresh complete medium was changed and incubated for 48 h, and the virus supernatant was isolated and filtered through a 0.45 mm filter. The virus was infected with SK-N-BE(2) at an appropriate amount. After 24 h, the medium was removed, and positive cells were screened with a complete medium containing 10 *μ*g/mL purinomycin to obtain stable cell lines. The targeted shRNA sequences of human CRBN, TBX3, and ZMYND8 are listed in Table S1.

### 2.3. Cell Viability Assay

NB cells were divided into 1 × 10^4^ cells, which were inoculated into 96 well plates, incubated overnight in an incubator at 37°C, and then, dBET57 was added to each well according to the concentration gradient. After 72 hours of drug treatment, add CCK8 (B34304, Bimake, TX, USA) reagent to each well based on the instructions. The microplate reader read the absorbance at 450 nm. Each concentration was examined and replicated at least three times independently, then calculated the IC50 values of dBET57 by using GraphPad Prism 8.4.3.

### 2.4. EdU Assay

NB cells incubated with different concentrations of dBET57 were inoculated into 6-well plates, and the prepared EdU (C0071S, Beyotime, Beijing, China) working solution was added to label the cells and then incubated for 2 h. Then, the cells were fixed with 4% paraformaldehyde in humidified incubator, and the prepared solution was added after removing the fixative. The cells were incubated with the permeabilizer for 10 minutes before washing. Then, Click reaction solution and Hoechst 33342 were added to cover the surface of NB cells and incubated against light to complete the staining of proliferating cells and nuclei. Finally, the excitation wavelength was adjusted by an optical microscope for color development observation.

### 2.5. Cell Cycle Analysis

NB cells treated with dBET57 for 48 h were collected. The floating color was removed with cold PBS and resuspended with 70% ethanol overnight. On the basis of manufacturing instructions, the samples were incubated in an incubator at 37°C with the affiliation of 25 *μ*g/mL RNaseA and 1.5 *μ*mol/L PI. The cell cycle of the samples was subjected to cell cycle detection using a Beckman Galios™ Flow Cytometer, and the data were analyzed by means of AVDNA analysis software.

### 2.6. Cell Apoptosis Assay

The detection of apoptosis was estimated as described in the previous protocol. NB cells were treated with dBET57 at indicated concentrations for 48 h, digested with trypsin, washed with PBS, and collected. In conformity with the instructions of FITC-Annexin V apoptosis detection kit (556547, BD, NJ, USA), PI and Annexin V-FITC as members of mixture were put into samples which were incubated in incubator for 15 minutes. The samples were then assayed for apoptosis using a Beckman Galios™ Flow Cytometer, and the experimental data were analyzed.

### 2.7. Cell Adhesion Assay

SK-N-BE(2), IMR-32, and SH-SY5Y cells treated with different concentrations of dBET57 were seeded at 3 × 10^5^ cells per well into 24-well plates precoated with cell adhesion agent (Apricot, China). Each treatment group contained 3 replicate wells. After incubating in a 37°C incubator for 2 h, the floating color and unattached cells were cleaned with PBS. It was then fixed with 4% formaldehyde for 10 min and stained with Wright's-Giemsa. Observation and counting were performed with an optical microscope, and analysis was performed with GraphPad Prism 8.3.0.

### 2.8. Wound Healing Assay

NB cells incubated with different concentrations of dBET57 were inoculated in 6-well plates. When the growth density was close to 85 under microscope, wounds were created with the tip of a sterile 10 *μ*L pipette tip. After washing away cell debris with PBS, culture was continued. The wound healing degree at 0 h, 24 h, and 48 h was recorded by optical microscope, and the statistics were made by GraphPadPrism8.3.0.

### 2.9. Western Blot Analysis

NB cells treated with dBET57 gradient concentration for 48 h were harvested, add RIPA buffer (C1053, APPLYGEN, Beijing, China) to completely lyse cells, and then, heated at 100°C for 10 min to obtain total protein. The proteins were electrophoresed and transferred to membranes by sodium dodecyl sulfate polyacrylamide gel electrophoresis (SDS-PAGE) and electrotransfer method, and the primary antibody was added to incubate at 4°C overnight. As mentioned earlier, the following antibodies were applied: anti-BRD2 (5848S, CST, MA, USA), anti-BRD3 (11859-1-AP, Proteintech, IL, USA), anti-BRD4 (13440S, CST, MA, USA), anti-CRBN (HPA045910, Sigma-Aldrich, USA), anti-c-Myc (9402S, CST, MA, USA), anti-Bcl-2 (ab32124, Abcam, UK), anti-CDK9 (2316S, CST, MA, USA), anti-Cyclin D1 (2978S, CST, MA, USA), anti-PARP (9542S, CST, MA, USA), anti-TBX3 (42-4800, Thermo Fisher Scientific, USA), anti-ZMYND8 (EPR16924, Abcam, UK), and anti-GAPDH (AP0063, Bioworld Technology Inc.). The next day, PVDF membranes were mixed with goat anti-rabbit IgG (H+L, 111-035-003) or anti-mouse IgG (H+L, 115035-003) HRP-conjugated secondary antibodies (Jackson ImmunoResearch Laboratories, PA, USA) at 1 : 3000 formulations which were coincubated for 1 h and then immersed membranes into ECL ultrasensitive luminescent fluid (Thermo Fisher Waltham, MA, US). The LAS 4010 (GE Healthcare Life Sciences, Little Chalfont, UK Cytiva) imaging system was used to monitor the expression of NB cell protein levels with GAPDH as a reference protein.

### 2.10. Xenograft Preparation and dBET57 Treatment in Nude Mice

12 nude mice aged 3-4 weeks were purchased from the Lingchang Biotechnology Co., Ltd.; 5 million SK-N-BE(2) cells with luciferase marker were injected into their armpits and then randomly divided into control group and treatment groups. Body weight and tumor size of nude mice were measured every 3 days. When the size of the tumor to be transplanted grew to 100 mm^3^, 150 *μ*L of dBET57 at 7.5 mg/kg and blank solvent (5% Coliflo® HS15) were intraperitoneally injected into the control group and the treatment group every day. Tumor size was measured periodically, and tumor fluorescence signal was detected using a Berthold (Germany) in vivo imaging system (LB987). At the end of the experiment, the nude mice were sacrificed, and the tumors were removed for IHC staining. All experimental and research procedures were approved by and in accordance with relevant guidelines and regulations. All animal procedures in this study have been approved and licensed by the Animal Care and Use Committee of Children's Hospital of Soochow University (CAM-SU-AP #: JP-2018-1).

### 2.11. RNA Preparation and Real-Time PCR Expression Analysis

The detection of RT-QPCR was described in the previous protocol. NB cells treated with dBET57 gradient concentration for 48 h were collected. According to the manufacturer's instructions, the total RNA was purified by RNeasy Mini kit (74104, Qiagen GmbH, MD, Germany). Then, using a two-step method, 2 *μ*g of total RNA was placed into a gradient PCR apparatus to reverse into cDNA. Quantitative real-time PCR analysis was carried out using a LightCycler® 480 (Roche, Mannheim, Germany), using human GAPDH as a reference and 2^−ΔΔCT^ calculation to monitor the expression of RNA levels in NB cells. PCR primer sequences are shown in Table S2.

### 2.12. RNA-Seq and Data Analysis

RNA-seq was implemented using the protocol provided by Novogene (Novogene Co., Ltd., Beijing, China). Total RNA was obtained by lysing NB cells using Trizol reagent (Want Sogenrethermo Fisher, Waltham, MA, USA). SK-N-BE(2) cells treated with 1200 nM dBET57 for 48 h or DMSO were sequenced. In view of the RNA-seq raw data results using the DESeq2 analysis [19] to identify differentially expressed genes (∣log2FoldChange | >1 and *p* (adjusted *p* values) < 0.05), adjusted *p* values were determined by false discovery rate (FDR). GSEA was detected using cluster analyzer software package 4.0.5 analysis in R4.1.1. RNA-seq original data have been submitted to the Gene Expression Omnibus (GEO) database (GSE208184).

### 2.13. Statistical Analysis

All experiments were repeated three times independently. Experimental data were analyzed and graphed using GraphPadPrism8.3.0 (GraphPad Software, Inc., San Diego, CA, USA). Values for each group were shown as mean ± standard deviation (SD), and differences between the two groups were assessed for statistical significance using the *T*-test. *p* < 0.05 was considered statistically significant (^∗^*p* < 0.05, ^∗∗^*p* < 0.01, and ^∗∗∗^*p* < 0.001).

## 3. Results

### 3.1. dBET57 Impairs the Proliferation of NB Cells

**Figure 1:**
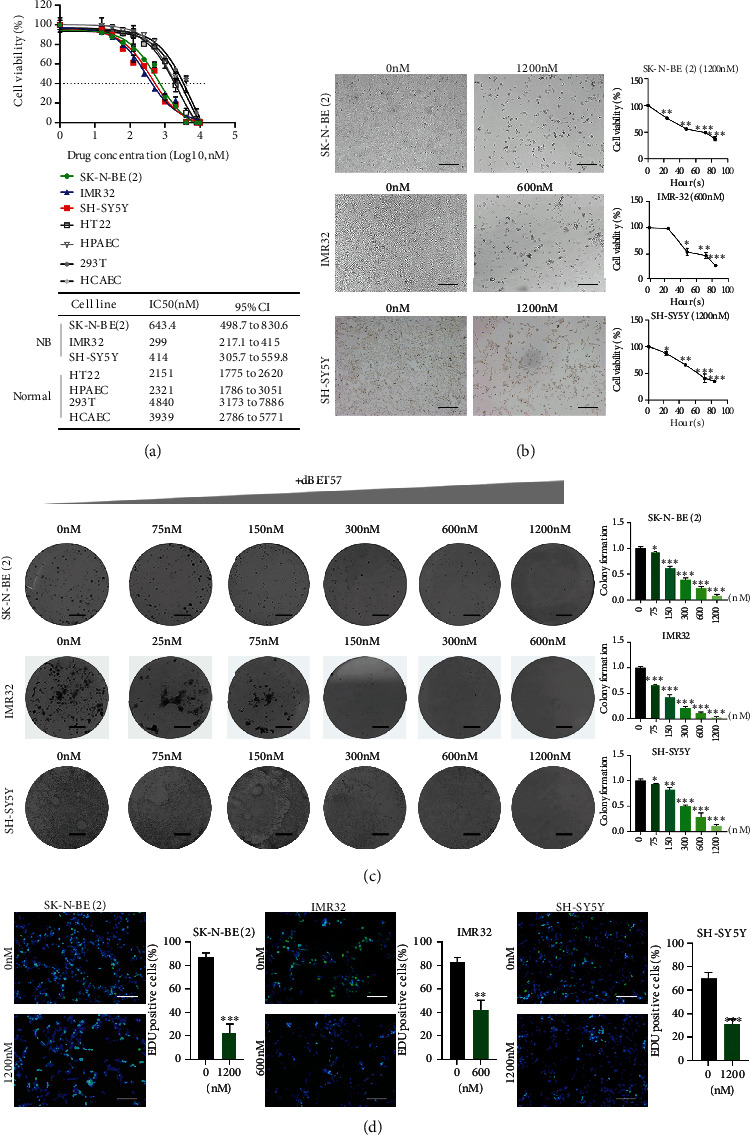
dBET57 impaired the proliferation of NB cells. (a) NB cells and 4 normal cells were treated with gradient concentration of dBET57 for 72 h to analyze the cell viability curve and calculate the value of semi-inhibitory concentration. (b) Morphological changes and cell viability curves (with time as reference) of NB cells treated with dBET57 gradient concentration for 48 h. Scale bars: 100 *μ*m. (c) Colony assay was used to compare the cloning ability of NB cells treated with dBET57 for 48 h. Scale bars: 4 mm. (d) EdU experiment proved the proliferation ability of NB cells incubated with dBET57 for 48 h. Scale bars: 100 *μ*m. The experimental groups (different concentration gradients of dBET57) were compared with the control group (0 nM). ^∗^*p* < 0.05, ^∗∗^*p* < 0.01, and ^∗∗∗^*p* < 0.001; ns: not significant.

PROTACs are heterobifunctional molecules that can ubiquitinate and degrade target proteins by recruiting E3 ligases to target proteins. It precisely targets BRD4 proteins, induces the degradation of BRD2 and BRD3, and has a strong antitumor effect on neuroblastoma [20]. In order to evaluate the anti-NB effect of dBET57, NB cells and 4 normal cells were treated in a concentration gradient for 72 hours. CCK8 assays exhibited that after treatment with dBET57, NB-cell viability declined in a dose-dependent manner (Figure 1(a)) (SK-N-BE(2) IC50 643.4 nM, IMR-32 IC50 299 nM, SH-SY5Y IC50 414 nM, HT22 IC50 2151 nM, HPAEC IC50 2321 nM, 293T IC50 4840 nM, and HCAEC IC50 3939 nM). Similarly, under the effective concentration of dBET57, the cell morphology gradually began to shrink until death occurred, and the cell viability gradually decreased with time (Figure 1(b)). Furthermore, the effects of dBET57 on the long-term proliferation of NB cells were evaluated by clonal formation and EdU assays. It was predicted that the number of clones and positive EdU cells in the dBET57-treated group was significantly lower than that in the control group (Figures 1(c) and 1(d)). Taken together, these findings demonstrate that dBET57 exhibits a robust antitumor effect on NB-cell lines *in vitro*.

### 3.2. dBET57 Induces Apoptosis and Inhibits Normal Cell Cycle and Migration Ability of NB Cells

**Figure 2:**
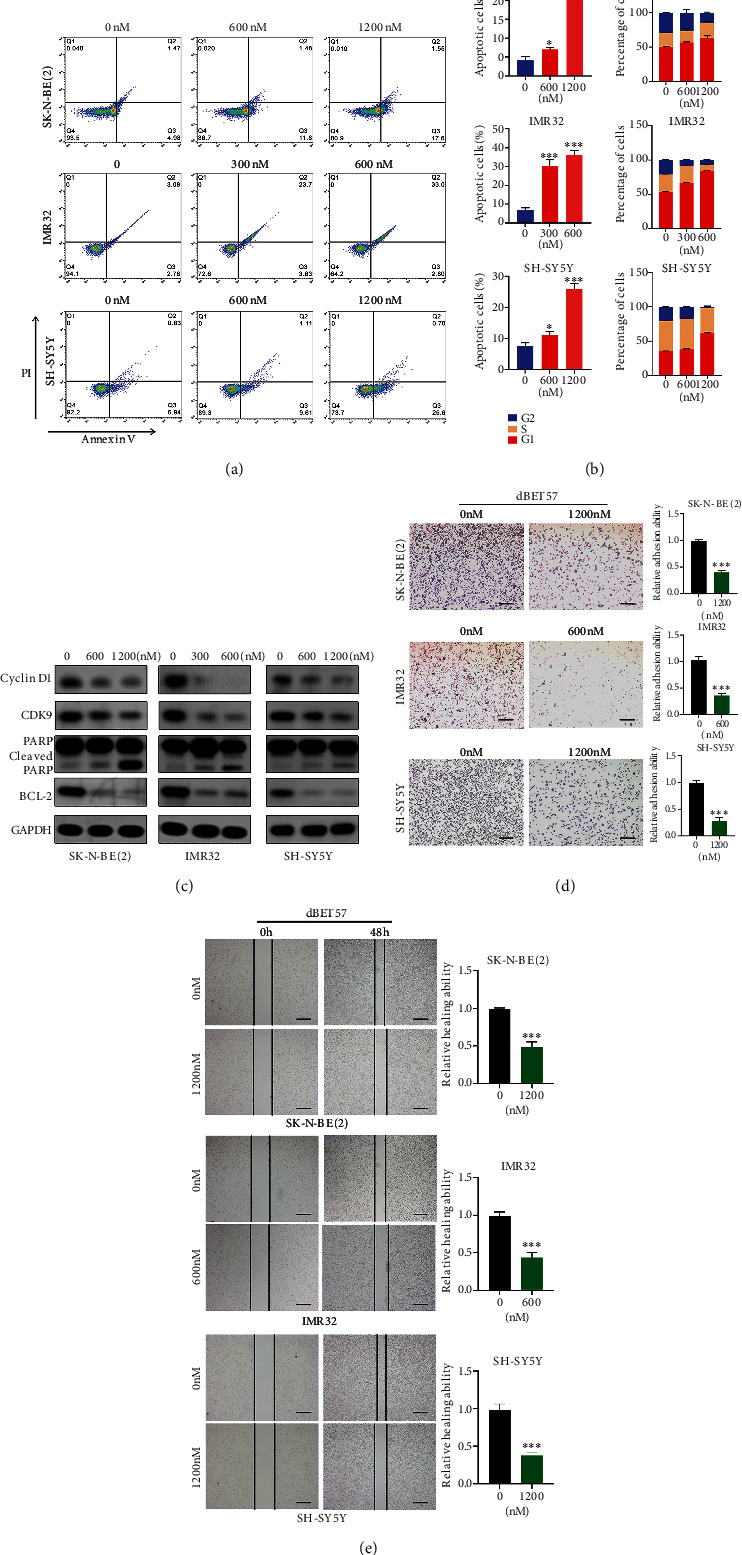
dBET57 induced apoptosis, inhibit normal cell cycle and migration ability of NB cells. NB cells were treated with different concentration gradients of dBET57 for 48 h. (a) Flow cytometry and Annexin V/PI staining were used to analyze the degree of apoptosis of NB cells. (b) Flow cytometry and PI staining were used to analyze the cell cycle distribution of NB cells. (c) Western blotting showed the protein level expression of Cyclin D1, CDK9, Bcl-2, and cleaved PARP in NB cells. (d) Adhesion assay to evaluate the adhesion ability of NB cells. Scale bars: 200 *μ*m. (e) Scratch experiment evaluate the migration ability of NB cells. Scale bars: 100 *μ*m. The experimental groups (different concentration gradients of dBET57) were compared with the control group (0 nM). ^∗^*p* < 0.05, ^∗∗^*p* < 0.01, and ^∗∗∗^*p* < 0.001; ns: not significant.

We explored the effect of dBET57 on NB-cell apoptosis by Annexin V/PI staining. Apoptosis analysis was performed after SK-N-BE(2), IMR-32, and SH-SY5Y cells were treated with dBET57 in a concentration gradient for 48 h. The proportion of apoptotic NB cells in all treatment groups increased significantly in a dose-dependent manner (Figure 2(a)). At the same time, we detected the expression of PARP and Bcl-2 in NB cells treated with different concentrations. Western blotting showed that the activation of PARP increased in a dose-dependent manner, while the expression of Bcl-2 decreased gradually (Figure 2(c)). We also investigated the effect of dBET57 on the NB-cell cycle by PI staining. Cell cycle analysis was carried out after NB cells were treated with dBET57 for 48 h. The proportion of NB cells in G1 phase in all treatment groups increased, while the proportion of cells in G2 and S phases decreased (Figure 2(b)). In addition, CyclinD1 and CDK9 [21], which are cell cycle marker proteins, were downregulated with increasing dBET57 concentrations (Figure 2(c)). Furthermore, cell adhesion assays (Figure 2(d)) and wound healing assays (Figure 2(e)) were conducted to verify the effects of dBET57 on the migration and adhesion abilities of NB cells, respectively. The results were in line with expectations that dBET57 attenuated the migratory and adhesion abilities of NB cells. Taken together, dBET57 can promote apoptosis and inhibit the normal cell cycle and migration ability of NB cells.

### 3.3. dBET57 Downregulates the Expression of BET Family Proteins and MYC Family Proteins in NB Cells

**Figure 3:**
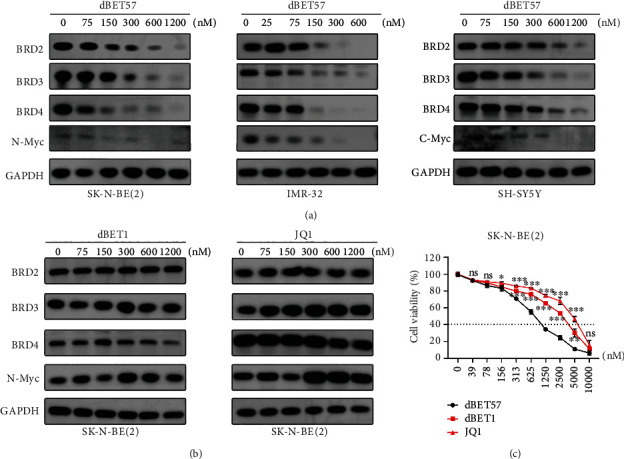
dBET57 reduces BET family proteins and MYC family proteins in NB cells. (a) Western blot quantitative analysis of the protein expression levels of BRD4, BRD3, BRD2, and n-Myc (or c-Myc). (b) Western blot analysis of the protein expression levels of BRD4, BRD3, BRD2, and n-Myc after SK-N-BE(2) cells were treated with different concentrations of dBET1 and JQ1 for 48 h. (c) The survival curve of SK-N-BE(2) cells treated with the same concentration of dBET1, dBET57, and JQ1 for 72 hours. The experimental groups (dBET1 and JQ1) were compared with the control group (dBET57). ^∗^*p* < 0.05, ^∗∗^*p* < 0.01, and ^∗∗∗^*p* < 0.001; ns: not significant.

It has been reported that the occurrence and development of cancer depends in part on the mechanism by which BRD4 can specifically recognize and anchor the side chain acetylated lysine on superenhancer open chromatin to promote the transcription of the MYCN gene [22]. The dBET57 inhibitor we studied is a novel, potent, and selective degrader of BRD4 via heterobifunctional small molecules based on PROTAC technology. Therefore, to explore the ability of dBET57 to degrade BET proteins in NB cells, we determined the expression levels of BRD4, BRD2, and BRD3 in NB cells treated with dBET57 for 48 h. We revealed that the expression of BET proteins in NB cells was downregulated in a concentration-dependent manner. As expected, n-Myc (or c-Myc) protein levels decreased indirectly with the degradation of BET proteins (Figure 3(a)). Furthermore, to confirm the high potency of dBET57, we compared it with a first-generation inhibitor of BRD4 (dBET1 and JQ1). The results showed that dBET1 and JQ1 were significantly weaker than dBET57 in terms of degrading BET and MYC protein families and their ability to kill NB cells at the same concentration (Figures 3(b) and 3(c)). Taken together, these data suggest that dBET57 is a potent protein inhibitor.

### 3.4. CRBN Is Indispensable for the Sensitivity of dBET57

**Figure 4:**
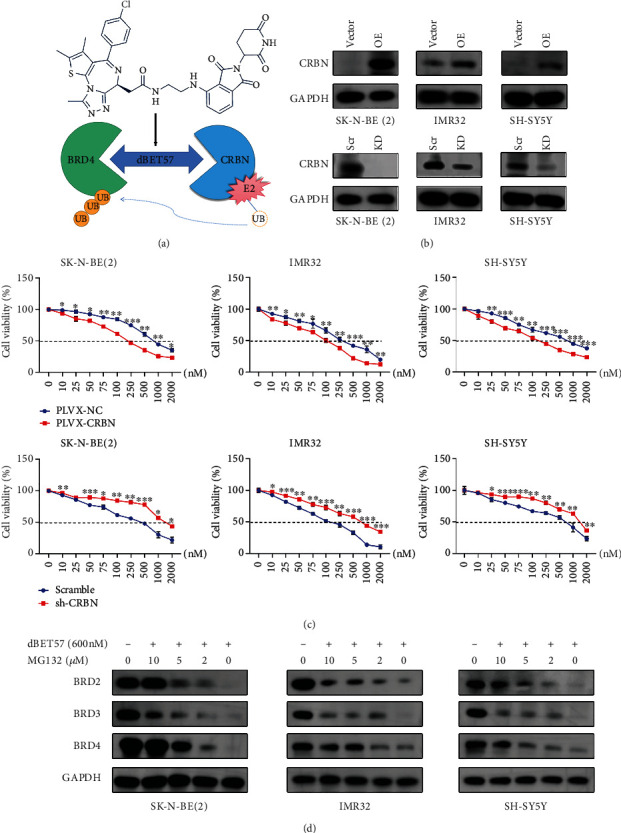
CRBN is indispensable for the sensitivity of dBET57. (a) Chemical formula and reaction diagram of dBET57. (b) Western blotting showed the protein expression levels of CRBN in NB cells transfected with lentiviral sh-CRBN (or PLVX-CRBN). Scr: scramble; KD: knockdown; OE: overexpression. (c) The sensitivity of NB cells transfected with sh-CRBN (or PLVX-CRBN) to dBET57 was compared with NB cells transfected with empty vector alone. (d) Western blot analysis showed that MG132 and dBET57 cotreated NB cells 24 h, the expression level of BET proteins. ^∗^*p* < 0.05, ^∗∗^*p* < 0.01, and ^∗∗∗^*p* < 0.001; ns: not significant.

dBET57 was designed with PROTAC technology and contains a CRBN recruitment moiety connected by a connector (Figure 4(a)). To elucidate the role of CRBN in dBET57-induced BRD4 degradation, we successfully constructed CRBN overexpression and CRBN knockdown systems and transfected them into SK-N-BE(2), IMR-32, and SH-SY5Y cells. The construction of the NB-cell line with CRBN overexpression or knockdown was verified by Western blotting (Figure 4(b)). The CCK-8 experiment revealed that excessive CRBN significantly increased the sensitivity of NB cells to dBET57. Conversely, downregulation of CRBN increased the IC50 of dBET57 in NB cells (Figure 4(c)). In addition, to explore the mechanism of CRBN's ubiquitin-degrading function in NB cells, we treated NB cells with the proteasome inhibitor MG132 (M8699, SIGMA, Darmstadt, GER) [23]. As expected, the content of BET family proteins degraded by dBET57 was restored in a dose-dependent manner by MG132 (Figure 4(d)). In conclusion, these observations suggest that dBET57-mediated alterations in the proliferative activity of NB cells are closely related to a CRBN-mediated mechanism.

### 3.5. dBET57 Exhibits a Tumor Growth Inhibition Effect in a Xenograft Tumor Model

**Figure 5:**
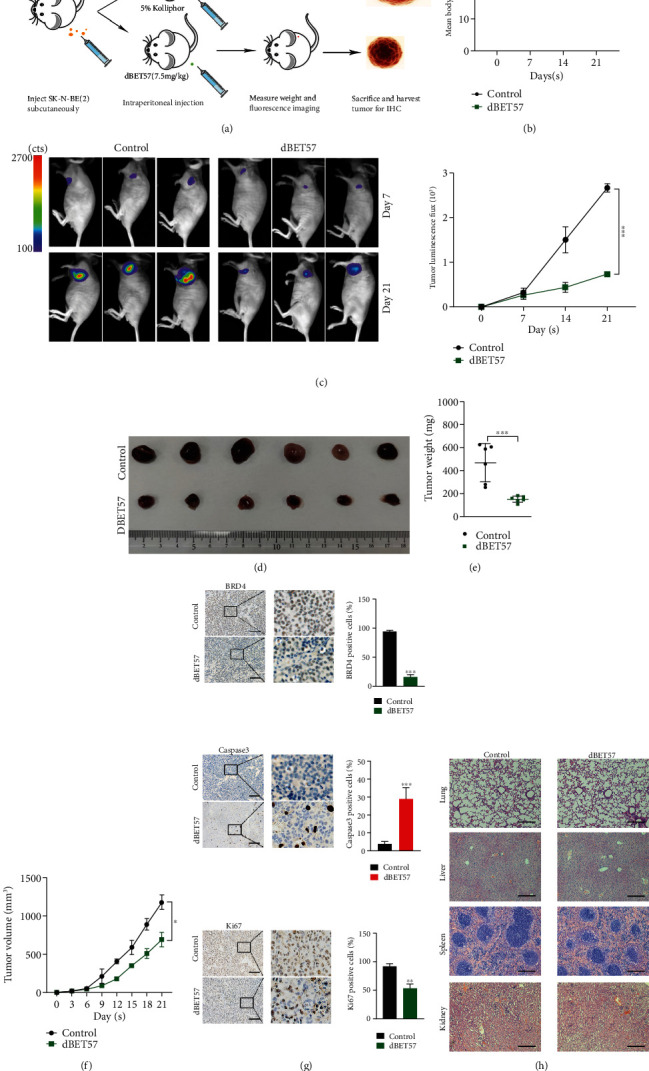
dBET57 exhibits tumor growth inhibition effect in xenograft tumor model. (a) Flow chart of the xenograft tumor model. (b) Body weight curve of nude mice surviving 21 days. (c) Tumor signal values in nude mice. (d) Measurement of tumor size in control and experimental groups. (e) Statistics of the average weight of the two groups of tumors. (f) Tumor growth curves of experimental group and control group. (g) Immunohistochemistry of BRD4, Ki67, and Caspase3. Scale bars: 100 *μ*m. (h) Pathological sections of the kidney, liver, spleen, and lung. Scale bars: 100 *μ*m. The experimental group (intraperitoneal injection of dBET57) was compared with the control group. ^∗^*p* < 0.05, ^∗∗^*p* < 0.01, ^∗∗∗^*p* < 0.001; ns: not significant.

We aimed to evaluate the antitumor ability and safety of dBET57 *in vivo*. A xenograft model based on the SK-N-BE(2) cell line with a luciferase marker was initially established. The abovementioned SK-N-BE(2) cells were subcutaneously inoculated into the armpits of nude mice, and the sizes of the tumors were measured daily. When the tumor volume reached 100 mm^3^, an intraperitoneal injection of 7.5 mg/kg dBET57 or vehicle alone (5% Kolliphor®HS15 (42966, SIGMA, Darmstadt, GER)) was given every day. The body weight of nude mice was measured every other week, and tumor signals of the two groups of nude mice were systematically monitored using a live animal bioluminescence imaging system (Figure 5(a)). After two weeks of treatment, all the nude mice in the two groups survived, and there was no significant difference in the body weight of the nude mice (Figure 5(b)). Compared with the control group, the tumor signals and volume of the dBET57-treated group were significantly reduced at the end time point (Figures 5(c)–5(f)). Immunohistochemical analysis showed the efficacy of dBET57 in reducing BDR4 in subcutaneous xenograft tumors and revealed that the proportion of Ki-67-positive cells [24] decreased and the proportion of caspase3-positive cells [25] increased after dBET57 treatment (Figure 5(g)). In terms of the safety of dBET57, the HE results of each organ of the two groups of nude mice showed that dBET57 had no obvious organ toxicity (Figure 5(h)). In conclusion, dBET57 is effective and feasible for the treatment of NB cells *in vivo*.

### 3.6. dBET57 Treatment Downregulates the Superenhancer-Related Gene ZMYND8 in NB

**Figure 6:**
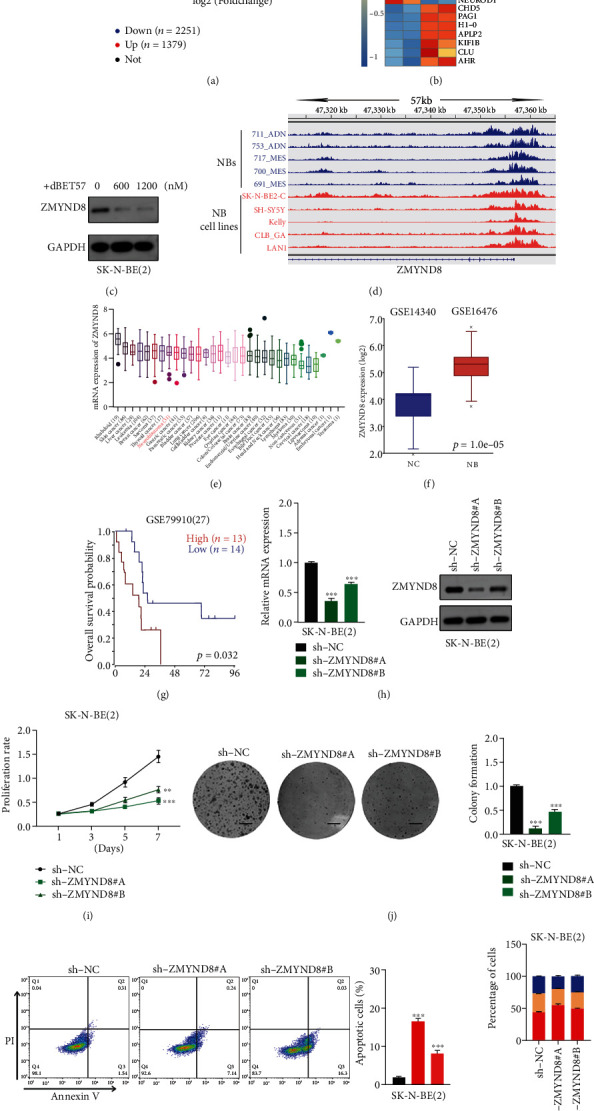
dBET57 treatment downregulates the superenhancer-related gene ZMYND8 in NB. (a) RNA-seq volcanic map of dBET57. (b) Heat map of differentially expressed genes (∣log2FoldChange | >0.5, *p* (adjusted *p* values) < 0.05) in SK-N-BE(2) cells treated with 1200 nM dBET57, each column represents a sample, each row represents a gene, blue represents gene downregulation, and red represents gene upregulation. (c) ZMYND8 protein level decreased in a gradient-dependent manner with dBET57 concentration. (d) The IGV diagram shows the CHIP-seq spectrum of downstream indicator genes. (e) Tumor profile of ZMYND8-specific expression (CCLE). (f) Expression of ZMYND8 in NB and NC cells. *p* value was determined by unpaired two-sided *t*-test. (g) The prognosis of ZMYND8. *p* value was determined by log-rank test. (h) ZMYND8 mRNA and protein expression level in SK-N-BE(2) cell. (i) Cell proliferation ability of sh-NC and sh-ZMYND8. (j) Clonogenic ability of sh-NC and sh-ZMYND8. Scale bars: 4 mm. (k) Apoptosis ability of sh-NC and sh-ZMYND8. (l) Cellular distribution of sh-NC and sh-ZMYND8. Each sh-ZMYND8 group was compared with the sh-NC group. ^∗^*p* < 0.05, ^∗∗^*p* < 0.01, and ^∗∗∗^*p* < 0.001; ns: not significant.

To clarify the potential mechanism by which dBET57 functions in NB cells, RNA-seq was used to screen and mine potential downstream genes. RNA-seq raw data analysis showed that under conditions of ∣log2FoldChange | >1 and *p* (adjusted *p* values) < 0.05, a total of 2251 genes were downregulated and 1,379 genes were upregulated in the 1200 nM dBET57 treatment group (Figure 6(a), Table S3). By taking important genes with specificity in NB as entry points, the scope of downregulated candidate genes was narrowed down, and it included PHOX2B [26, 27], MYCN [28], ZMYND8, XPOT, LIN28B [29], TERT [30], and other important genes (Figure 6(b)). Next, we focused on ZMYND8, also known as RACK7, which is a multifunctional transcription factor. It has been reported that ZMYND8 possesses conserved chromatin-binding modules and can anchor-activated protein-kinase-C- (PKC-) binding proteins to phosphorylation or dephosphorylation [31], thereby controlling the occurrence of malignant tumors. To investigate its relationship with dBET57 and its role in NB, we first confirmed the changes in its protein level in NB cells after dBET57 treatment. The results confirmed that the protein levels of ZMYND8 were dose-dependently decreased after dBET57 treatment in NB cells (Figure 6(c)). Next, ChIP-seq analysis of H3K27Ac was performed in 5 NB cases and 5 NB-cell lines. The Integrative Genomics Viewer (IGV) results showed that H3K27Ac in the ZMYND8 locus had a high accumulation of spacers (Figure 6(d)), indicating the presence of SE; in other words, ZMYND8 is a superenhancer-related gene in NB. Analysis of various tumor cell lines in the CCLE database showed that the expression level of ZMYND8 in NB cells was significantly higher (Figure 6(e), Table S4). Analysis of the GSE14340 and GSE16476 datasets according to the R2 database revealed that ZMYND8 was overexpressed in NB tissues compared to NCs (Figure 6(f)). Furthermore, GSE79910 showed a poor prognosis with high ZMYND8 expression (Figure 6(g)).

To investigate the function of ZMYND8 in NB cells. We infected SK-N-BE(2) cells with sh-NC and sh-ZMYND8 lentiviruses, and we successfully constructed a ZMYND8 knockdown system and empty vector system by Western blotting and qPCR analysis (Figure 6(h)). Then, we carried out CCK8 (Figure 6(i)) and clone formation assays (Figure 6(j)) to confirm the proliferation ability of ZMYND8. Furthermore, we used flow cytometry to explore the changes in apoptosis and cell cycle progression in NB cells induced by ZMYND8. According to the analysis of experimental data, the apoptosis of sh-ZMYND8 cells was obvious, and cell cycle progression was significantly limited (Figures 6(k) and 6(l)). This is consistent with the dBET57-treated phenotype. In summary, these results indicate that ZMYND8 plays a key role in the genesis and development of NB.

### 3.7. As a Potential Downstream Target of dBET57, TBX3 Plays an Important Role in NB Cells

**Figure 7:**
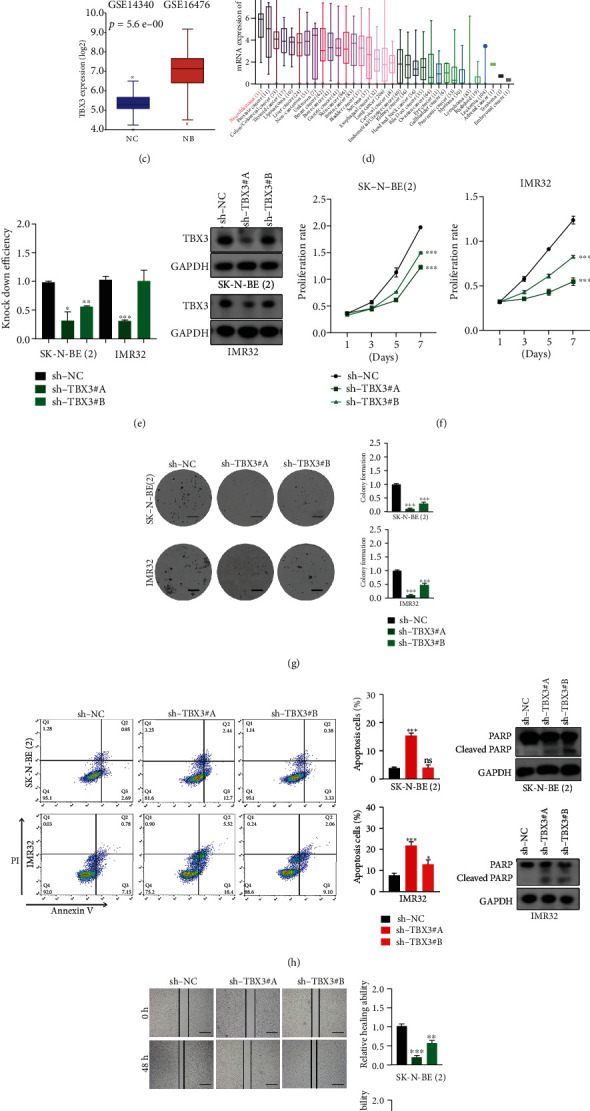
As a potential downstream target of dBET57, TBX3 plays an important role in NB cells. (a) TBX3 protein level decreased in a gradient-dependent manner with dBET57 concentration. (b) The IGV diagram shows the CHIP-seq spectrum of TBX3. (c) Expression of TBX3 in NB and NC cells. *p* value was determined by unpaired two-sided *t*-test. (d) Tumor profile of TBX3-specific expression (CCLE). In SK-N-BE(2) and IMR-32 cells. (e) TBX3 mRNA and protein expression level. (f) Cell proliferation ability of control group and experimental group. (g) Clonogenic ability of control and experimental groups. Scale bars: 4 mm. (h) Apoptosis ability of control group and experimental group. (i) Migration ability of control and experimental groups. Scale bars: 100 *μ*m. Each sh-TBX3 group was compared with the sh-NC group. ^∗^*p* < 0.05, ^∗∗^*p* < 0.01, and ^∗∗∗^*p* < 0.001; ns: not significant.

TBX3, also known as UMS, is a multifunctional transcription factor that belongs to the T-box family of transcription factors and plays a crucial role in embryonic development [32]. Among them, TBX2 is an important member of the core transcriptional regulatory network in NB [33]. Current studies have shown that abnormal expression of TBX3 affects EMT, tissue integrity, and cell differentiation, thereby controlling the occurrence of malignant tumors. However, its specific mechanism in NB is still unclear. Therefore, we explored its relationship with dBET57 and its role in NB. In dBET57-treated NB cells, the protein level of TBX3 was decreased in a dose-dependent manner (Figure 7(a)), suggesting that TBX3 is a potential downstream target of dBET57. Similarly, ChIP-seq analysis of H3K27Ac in 5 NB cases and 5 NB-cell lines showed that TBX3 is also a superenhancer-related gene in NB (Figure 7(b)). Analysis of the GSE14340 and GSE16476 datasets according to the R2 database revealed that TBX3 was overexpressed in NB tissues compared to NCs (Figure 7(c)). The mRNA expression level of TBX3 was high, presenting specific expression characteristics of tumors (CCLE) (Figure 7(d), Table S5). To explore the function of TBX3 in NB cells, we constructed SK-N-BE(2) and IMR-32 cell lines with stable knockdown of TBX3 (Figure 7(e)). To confirm that TBX3 plays an important role in NB-cell proliferation, we performed clonogenic assays and CCK8 assays. Consistent with our prediction, the proliferative capacity of sh-TBX3 cells was lower than that of sh-NC (Figures 7(f) and 7(g)). Furthermore, NB cells after TBX3 knockdown showed obvious signs of apoptosis, and the changes in the apoptosis-related protein PARP were in line with expectations (Figure 7(h)). Additionally, knockdown of TBX3 restricted the migratory ability of NB cells (Figure 7(i)). Taken together, these results indicate that TBX3 plays an integral role in the genesis and development of NB *in vitro*.

**Figure 8:**
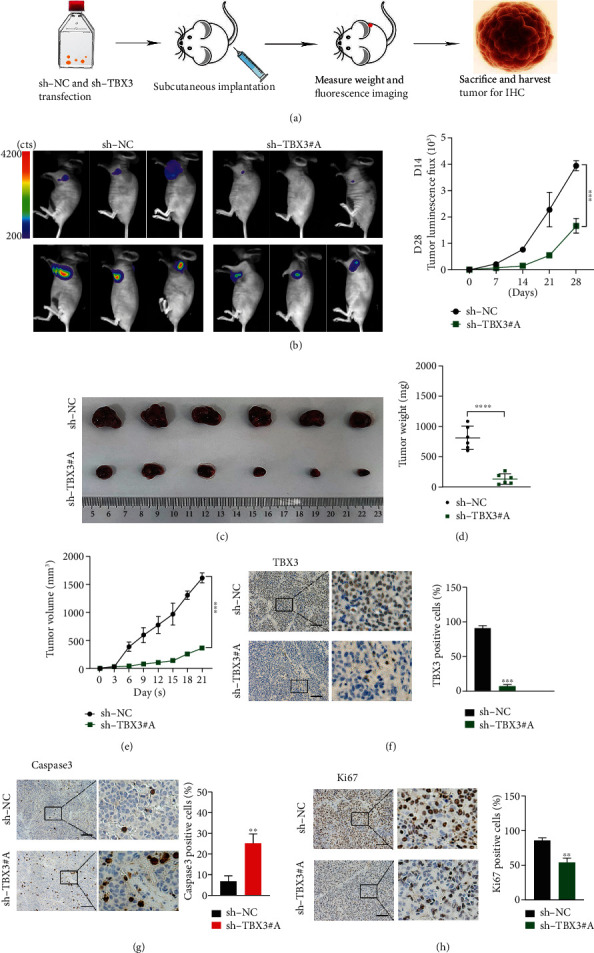
sh-TBX3 exhibits tumor growth inhibition effect in xenograft tumor model. (a) Flow chart of the xenograft tumor model. (b) Tumor signal values in nude mice. (c) Measurement of tumor size in control and experimental groups. (d) Statistics of the average weight of the two groups of tumors. (e) Tumor growth curves of experimental group and control group. (f–h) Immunohistochemistry of TBX3, Ki67, and Caspase3. Scale bars: 100 *μ*m. Each sh-TBX3 group was compared with the sh-NC group. ^∗^*p* < 0.05, ^∗∗^*p* < 0.01, ^∗∗∗^*p* < 0.001; ns: not significant.

We also evaluated the anti-NB effect of TBX3 *in vivo*. The xenograft tumor model is based on sh-NC and sh-TBX3 stably transformed into the SK-N-BE(2) cell line with a luciferase marker. The body weight and tumor signal values of the nude mice were detected weekly (Figure 8(a)). As predicted, the tumor signal and size of nude mice in the TBX3 knockdown group were significantly lower than those in the control group (Figures 8(b)–8(e)). Immunohistochemistry showed that tumor cells had decreased proliferative capacity and increased apoptosis after TBX3 knockdown (Figures 8(f)–8(h)). In conclusion, the growth of NBs was significantly inhibited after TBX3 knockdown *in vivo*.

## 4. Discussion

Neuroblastoma is a cancer of the sympathetic nervous system whose susceptibility to recurrence and drug resistance remains a major treatment challenge [34]. At present, the research and development of more effective and safer new therapies has become an important key goal to improve the prognosis of patients [35]. Studies have shown that the survival rate of invasive NB patients with MYCN gene upregulation is less than 50%, and the recurrence rate is high [36]. Therefore, regulation of the specific expression of MYCN can be used as a potential therapeutic strategy to identify novel MYCN-dependent oncogenic pathways [37]. However, MYCN itself has a short half-life and complex protein structure, so it is difficult to directly target. Fortunately, we can identify the molecules that directly interact with MYCN by monitoring the MYCN network and applying control signals to achieve indirect regulation, which is expected to improve the disease state [38].

BRD4 belongs to the epigenetic reader proteins of the BET family [39]. By recruiting the scaffold of P-TEFb, it localizes to the promoter, selectively binds to acetylated lysine residues, and drives the transcriptional activation of MYCN. More specifically, BRD4 inhibitors targeting ubiquitin-mediated hydrolysis of MYCN to treat NB have important significance and application prospects. In recent years, clinical trials of BRD4 inhibitors have shown that early acquired resistance limits their therapeutic benefits [20]. In this study, we investigated dBET57, a heterobifunctional molecule inhibitor that binds the target protein at one end and the E3 ubiquitin ligase (CRBN binding fragment) at the other end. Mechanistically, BRD4 indirectly binds to the CRBN domain as a target protein and inhibits the transcription of MYCN by ubiquitination of BRD4. Compared with traditional small molecule inhibitors, dBET57 has lower toxicity, longer reaction duration, more selective sensitivity, and higher potency [40].

In this study, we determined whether dBET57 inhibitors can exhibit antitumor activity in NB and accumulated preclinical data. The results showed that *in vitro*, dBET57 inhibited NB proliferation in a dose-dependent manner and induced cell cycle arrest and cell apoptosis. In addition, knockdown or overexpression of proteasome CRBN could correspondingly reduce or improve the hydrolysis efficiency of BRD4 and change the sensitivity to NB. *In vivo*, the tumor weight and size of the nude mice treated with dBET57 were significantly lower than those of the NB group, showing a strong antiproliferation effect. In summary, our results reveal the effectiveness and feasibility of dBET57 in the treatment of NB both *in vivo* and *in vitro*, which has potential guiding significance for clinical treatment.

We then further explored the underlying mechanisms of dBET57's antitumor properties. We detected the changes in total NB transcripts after 48 h of dBET57 treatment by RNA-seq and found thousands of upregulated and downregulated genes. We used the *p* value and the close correlation of NB as the entry point to screen the differentially expressed downregulated genes and narrow down the number of candidate genes. Except for the downregulation of well-known key oncogenes in NB, such as “LIN28B”, “TERT,” and “PHOX2B,” some genes had not been reported in NB. Based on the protein decrease after dBET57 treatment, the analysis of clinical data, and the analysis of ChIP-seq, this study focused on ZMYND8 and TBX3. According to the reported literature, ZMYND8, also known as RACK7, is a multifunctional transcription factor. It contains a PWWP chromatin-binding domain, a BRD, a PHD-type zinc finger, and a MYND domain for protein–protein interactions. Mechanistically, ZMYND8 can also anchor activated PKC-binding proteins for phosphorylation or dephosphorylation, hereby controlling the occurrence of malignant tumors. TBX3, also known as UMS, is a multifunctional transcription factor that plays a crucial role in embryogenesis. The dysregulated expression of TBX3 affects EMT, tissue integrity, and cell differentiation, thereby controlling the occurrence of malignant tumors. In the present study, knockdown of ZMYND8 and TBX3 had a strong inhibitory effect on the proliferation and promotion of apoptosis of NB cells *in vivo* and *in vitro*, thus limiting the occurrence and development of NB. This is consistent with the treatment of dBET57, suggesting that the superenhancer-related genes ZMYND8 and TBX3 play a crucial role as the downstream targets of dBET57 in the treatment of NB.

## 5. Conclusion

Our findings demonstrate that the BRD4 inhibitor dBET57 exhibits potent antitumor activity in both *in vivo* and *in vitro* experiments by targeting the superenhancer-related genes ZMYND8 and TBX3 in NB. However, our experiments lacked an in-depth exploration of the functional mechanism of dBET57 and studies in clinical samples. Therefore, further studies are needed to confirm our conclusions to pave the way for this drug to enter clinical trials and be effective in improving patient outcomes.

## Data Availability

The datasets used and/or analyzed during the current study are available from the corresponding authors on reasonable request. RNA-seq and original data have been submitted to the GEO database with accession number GSE208184 and R2 database with accession numbers GSE14340, GSE79910, and GSE16476.

## Supplementary Materials

Supplementary 1 Table S1: information for the expression vectors used in this study.

Supplementary 2 Table S2: information for the primer in this study.

Supplementary 3 Table S3: information for the RNA-seq of dBET57 in this study.

Supplementary 4 Table S4: information for the CCLE of ZMYND8 in this study.

Supplementary 5 Table S5: information for the CCLE of TBX3 in this study.
