# Supplementary material for: The BRD4 Inhibitor dBET57 Exerts Anticancer Effects by Targeting Superenhancer-Related Genes in Neuroblastoma
Source: J Immunol Res. 2022 Nov 16;2022:7945884. doi: 10.1155/2022/7945884 (PMC9691391; doi:10.1155/2022/7945884)
Supplement: Supplementary 1 — Table S1: information for the expression vectors used in this study. [file 7945884.f1.docx]

| **Table S1. shRNA sequence** | |
| --- | --- |
| **Gene name Sequence** | |
| sh-ZMYND8-A | 5’CCGGGCAGCATCCTGAATCTTAACCCTCGAGGGTTAAGATTCAGGATGCTGCTTTTTGAATT3’ |
| sh-ZMYND8-B | 5’CCGGGGACAGATGCATTCCAGAAGCCTCGAGGCTTCTGGAATGCATCTGTCCTTTTTGAATT3’ |
| sh-TBX3-A | 5’CCGGGCGAATGTTTCCTCCATTTAACTCGAGTTAAATGGAGGAAACATTCGCTTTTTTGAATT3’ |
| sh-TBX3-B | 5’CCGGCCAAGCCGATCATGGATCAATCTCGAGATTGATCCATGATCGGCTTGGTTTTTGAATT3’ |
| sh-CRBN | 5’CCGGGCCCACGAATAGTTGTCATTTCTCGAGAAATGA  CAACTATTCG3’ |
