# Supplementary material for: The BRD4 Inhibitor dBET57 Exerts Anticancer Effects by Targeting Superenhancer-Related Genes in Neuroblastoma
Source: J Immunol Res. 2022 Nov 16;2022:7945884. doi: 10.1155/2022/7945884 (PMC9691391; doi:10.1155/2022/7945884)
Supplement: Supplementary 2 — Table S2: information for the primer in this study. [file 7945884.f2.docx]

| **Table S2. Primer sequence** | |
| --- | --- |
| **Gene name Sequence** | |
| ZMYND8 | 5’GGGTTTATCACGCTAAGTGTCTG-3’(Forward)  5′- GGCTTTACTCTGGGTCTCGATG-3′ (Reverse) |
| TBX3 | 5’GAGGCTAAAGAACTTTGGGATCA-3’ (Forward)  5′-CATTTCGGGGTCGGCCTTA -3′ (Reverse) |
| GAPDH | 5′ATCATCCCTGCCTCTACTGG-3′(Forward)  5′CCCTCCGACGCCTGCTTCAC-3′ (Reverse) |
