# Supplementary material for: The BRD4 Inhibitor dBET57 Exerts Anticancer Effects by Targeting Superenhancer-Related Genes in Neuroblastoma
Source: J Immunol Res. 2022 Nov 16;2022:7945884. doi: 10.1155/2022/7945884 (PMC9691391; doi:10.1155/2022/7945884)
Supplement: Supplementary 3 — Table S3: information for the RNA-seq of dBET57 in this study. [file 7945884.f3.pdf]

Table S3. Differentially expressed genes in dBET57 treatment SK-N-BE(2) cells

ensembl_gene_id	log2FoldChange	pvalue	padj	gene_name	gene_biotype	description	chromosome	start_position	end_position
ENSG0000000819	-2.281546487	0	0	TFAP2B	protein_coding	transcription	6	50818723	50847619
ENSG0000003451	1.687138117	0	0	TMSB10	protein_coding	thymosin be	2	84905656	84906671
ENSG0000004457	-1.95159457	0	0	HSPA5	protein_coding	heat shock p	9	125234853	125241343
ENSG0000005110	-2.181906395	0	0	HERPUD1	protein_coding	homocystein	16	56932142	56944864
ENSG0000008423	1.500182149	0	0	APLP2	protein_coding	amyloid bet	11	130068147	130144811
ENSG0000010029	-4.370926119	0	0	HMOX1	protein_coding	heme oxyge	22	35380361	35394207
ENSG0000010125	-3.662381324	0	0	TRIB3	protein_coding	tribbles pse	20	362835	397559
ENSG0000010219	2.100208572	0	0	GPR50	protein_coding	G protein-c	X	151176584	151181465
ENSG0000011271	-2.238342804	0	0	VEGFA	protein_coding	vascular enc	6	43770184	43786487
ENSG0000011293	-4.968278815	0	0	C7	protein_coding	complemen	5	40909497	40984643
ENSG0000011373	-3.825955057	0	0	STC2	protein_coding	stanniocalci	5	173314713	173329503
ENSG0000012157	-4.958100248	0	0	POPDC2	protein_coding	popeye dom	3	119636457	119665324
ENSG0000012341	1.484339564	0	0	TUBA1B	protein_coding	tubulin alph	12	49127782	49131397
ENSG0000012596	2.863708246	0	0	ID1	protein_coding	inhibitor of	20	31605283	31606515
ENSG0000012856	1.992005841	0	0	VGF	protein_coding	VGF nerve g	7	101162509	101165569
ENSG0000013060	-6.087822548	0	0	H19	lncRNA	H19 imprint	11	1995176	2001470
ENSG0000013238	-5.341245672	0	0	SERPINF1	protein_coding	serpin family	17	1762029	1777565
ENSG0000013247	1.687313835	0	0	H3-3B	protein_coding	H3.3 histone	17	75776434	75785893
ENSG0000013696	-1.97239339	0	0	ENPP2	protein_coding	ectonucleot	8	119557086	119673453
ENSG0000013929	-2.40095717	0	0	LGR5	protein_coding	leucine rich	12	71439798	71586310
ENSG0000013932	-3.527003267	0	0	LUM	protein_coding	lumican [So	12	91102629	91111494
ENSG0000016625	-3.103258965	0	0	CLMP	protein_coding	CXADR like	11	123069872	123195248
ENSG0000016800	-1.765739953	0	0	SLC3A2	protein_coding	solute carrie	11	62856102	62888875
ENSG0000018457	-1.594986209	0	0	XPOT	protein_coding	exportin for	12	64404392	64451125
ENSG0000018555	-5.788612421	0	0	DLK1	protein_coding	delta like no	14	100725705	100738224
ENSG0000018763	3.952067858	0	0	SAMD11	protein_coding	sterile alpha	1	923928	944581
ENSG0000018906	1.760483564	0	0	H1-0	protein_coding	H1.0 linker h	22	37805093	37807436
ENSG0000019840	2.580958316	0	0	OGA	protein_coding	O-GlcNAca	10	101784443	101818465
ENSG0000019883	2.088161229	0	0	HMGN2	protein_coding	high mobilit	1	26472440	26476642
ENSG0000021328	-1.661202662	0	0	NRAS	protein_coding	NRAS proto	1	114704469	114716771
ENSG0000021454	-1.747314733	0	0	MEG3	lncRNA	maternally e	14	100779410	100861031
ENSG0000022376	4.957378047	0	0	LINC02593	lncRNA	long interge	1	916865	921016
ENSG0000026171	3.298377054	0	0	H2BC20P	transcribed_un	H2B clustere	1	149844498	149850937
ENSG0000027027	3.886290921	0	0	H4C15	protein_coding	H4 clustered	1	149854045	149861210
ENSG0000027088	3.880649706	0	0	H4C14	protein_coding	H4 clustered	1	149832659	149839767
ENSG0000016755	1.321868255	####	###	TUBA1A	protein_coding	tubulin alph	12	49184795	49189080
ENSG0000010325	-1.598610066	####	###	SLC7A5	protein_coding	solute carrie	16	87830023	87869507
ENSG0000018822	1.924465074	####	###	TUBB4B	protein_coding	tubulin beta	9	137241287	137243707
ENSG0000007727	-2.192265885	####	###	DCX	protein_coding	doublecorti	X	111293779	111412429
ENSG0000014333	-3.126349782	####	###	RGS16	protein_coding	regulator of	1	182598623	182604389
ENSG0000017054	1.636874133	####	###	ARL6IP1	protein_coding	ADP ribosyl	16	18791669	18801572
ENSG0000016877	-3.457250626	####	###	SHOX2	protein_coding	short stature	3	158095954	158106503
ENSG0000014332	-4.784196461	####	###	CRABP2	protein_coding	cellular retir	1	156699606	156705816
ENSG0000024553	1.761422856	####	###	NEAT1	lncRNA	nuclear para	11	65422774	65445540
ENSG0000013429	-1.344490589	####	###	SLC38A2	protein_coding	solute carrie	12	46358188	46372773
ENSG0000014884	-3.089346595	####	###	ADAM12	protein_coding	ADAM meta	10	126012381	126388455
ENSG0000013506	-1.551037968	####	###	PSAT1	protein_coding	phosphoser	9	78297125	78330093
ENSG0000010121	-2.375444419	####	###	EEF1A2	protein_coding	eukaryotic t	20	63488013	63499239
ENSG0000013876	1.54130211	####	###	SCARB2	protein_coding	scavenger re	4	76158737	76234536
ENSG0000008919	-1.501243375	####	###	CHGB	protein_coding	chromogran	20	5911510	5925353
ENSG0000012517	1.556535149	####	###	DOK4	protein_coding	docking pro	16	57471922	57487327
ENSG0000013928	-2.746755197	####	###	PHLDA1	protein_coding	pleckstrin ho	12	76025447	76033932
ENSG0000014387	-1.558523559	####	###	PDIA6	protein_coding	protein disu	2	10783391	10837977
ENSG0000001146	-5.869201927	####	###	DCN	protein_coding	decorin [Sou	12	91140484	91183217
ENSG0000017688	1.438019829	####	###	SOX11	protein_coding	SRY-box tra	2	5692384	5701385
ENSG0000026597	3.351221582	####	###	TXNIP	protein_coding	thioredoxin	1	145992435	145996579

ENSG0000011372	-2.007515819	####	###	PDGFRB	protein_coding	platelet deri	5	150113839	150155872
ENSG0000014319	-7.065571043	####	###	DPT	protein_coding	dermatopor	1	168695468	168729206
ENSG0000019692	1.27389489	####	###	FLNA	protein_coding	filamin A [Sc	X	154348524	154374638
ENSG0000014925	-1.77806656	####	###	SERPINH1	protein_coding	serpin family	11	75562056	75572783
ENSG0000014387	1.60963187	####	###	RHOB	protein_coding	ras homolog	2	20447074	20449440
ENSG0000015101	-1.364283321	####	###	SLC7A11	protein_coding	solute carrie	4	138164097	138242349
ENSG0000012106	2.022342669	####	###	TBX2	protein_coding	T-box trans	17	61399843	61409466
ENSG0000010633	-2.341205315	####	###	PCOLCE	protein_coding	procollagen	7	100602363	100608175
ENSG0000020558	1.398966158	####	###	HMGN1	protein_coding	high mobilit	21	39342315	39349647
ENSG0000008471	3.374677634	####	###	EFR3B	protein_coding	EFR3 homol	2	25042076	25159135
ENSG0000017184	1.754867985	####	###	RRM2	protein_coding	ribonucleoti	2	10120698	10211725
ENSG0000016273	-1.268553604	####	###	DDR2	protein_coding	discoidin dc	1	162631373	162787405
ENSG0000018316	-3.726146901	####	###	TMEM119	protein_coding	transmembr	12	108589851	108598320
ENSG0000019745	1.72907162	####	###	STMN3	protein_coding	stathmin 3 [	20	63639705	63657682
ENSG0000010034	1.303349453	####	###	MYH9	protein_coding	myosin heav	22	36281280	36387967
ENSG0000022578	-3.128765314	####	###	MIAT	lncRNA	myocardial	22	26646411	26676475
ENSG0000011666	-1.554847957	####	###	C1orf21	protein_coding	chromosom	1	184387029	184629019
ENSG0000011137	-1.193178091	####	###	SLC38A1	protein_coding	solute carrie	12	46183063	46270017
ENSG0000017921	-1.043705524	####	###	CALR	protein_coding	calreticulin [	19	12938578	12944489
ENSG0000014332	-1.294247703	####	###	HDGF	protein_coding	heparin binc	1	156742109	156766925
ENSG0000012258	-3.327406724	####	###	NPY	protein_coding	neuropeptic	7	24284188	24291862
ENSG0000012073	1.745918748	####	###	EGR1	protein_coding	early growtl	5	138465479	138469303
ENSG0000006476	-1.956969331	####	###	FAR2	protein_coding	fatty acyl-C	12	29149103	29340980
ENSG0000013951	-1.526689817	####	###	SLC7A1	protein_coding	solute carrie	13	29509414	29595688
ENSG0000016552	-1.566601926	####	###	EML5	protein_coding	EMAP like 5	14	88612431	88792752
ENSG0000011254	-2.101026761	####	###	PDE10A	protein_coding	phosphodie	6	165327287	165988078
ENSG0000009962	1.122719534	####	###	CIRBP	protein_coding	cold induciŀ	19	1259384	1274880
ENSG0000021459	-1.808204748	####	###	EML6	protein_coding	EMAP like 6	2	54723499	54972025
ENSG0000014385	-2.10825895	####	###	PLEKHA6	protein_coding	pleckstrin hc	1	204218853	204377665
ENSG0000016659	-0.916546827	####	###	HSP90B1	protein_coding	heat shock	12	103930107	103953645
ENSG0000014942	-1.10737305	####	###	HYOU1	protein_coding	hypoxia up-	11	119044188	119057227
ENSG0000006795	1.627961066	####	###	CBFB	protein_coding	core-bindin	16	67028984	67101058
ENSG0000004944	-1.839124006	####	###	RCN1	protein_coding	reticulocalbi	11	32091074	32105722
ENSG0000022897	-4.175866772	####	###	LINC02607	lncRNA	long interge	1	95510059	95782342
ENSG0000013808	-1.671408545	####	###	EMILIN1	protein_coding	elastin micr	2	27078615	27086403
ENSG0000009262	-1.26792468	####	###	PHGDH	protein_coding	phosphogly	1	119648411	119744218
ENSG0000015751	-1.994641973	####	###	TSC22D3	protein_coding	TSC22 dom	X	107713221	107777342
ENSG0000014873	1.444607543	####	###	EIF4EBP2	protein_coding	eukaryotic t	10	70404145	70428618
ENSG0000014026	-1.224839395	####	###	TCF12	protein_coding	transcriptior	15	56918623	57299281
ENSG0000017275	1.179874603	####	###	CFL1	protein_coding	cofilin 1 [So	11	65823022	65862026
ENSG0000013243	-1.79468443	####	###	DDC	protein_coding	dopa decarl	7	50458436	50565405
ENSG0000009142	-3.462065492	####	###	RAPGEF4	protein_coding	Rap guanine	2	172735274	173052893
ENSG0000013931	-2.51749207	####	###	DUSP6	protein_coding	dual specific	12	89347235	89352501
ENSG0000015196	-1.95915106	####	###	SCHIP1	protein_coding	schwannom	3	159839861	159897360
ENSG0000018631	1.365095513	####	###	BACE1	protein_coding	beta-secret	11	117285207	117316259
ENSG0000019651	-4.587064465	####	###	SLC6A9	protein_coding	solute carrie	1	43991500	44031467
ENSG0000013531	-2.33831727	####	###	NT5E	protein_coding	5'-nucleotic	6	85449584	85495791
ENSG0000006580	-1.473752342	####	###	FAM107B	protein_coding	family with	10	14518557	14774897
ENSG0000013877	-2.800482563	####	###	SHROOM3	protein_coding	shroom fam	4	76435229	76783253
ENSG0000011658	-1.810451263	####	###	ARHGEF2	protein_coding	Rho/Rac gu	1	155946851	156007070
ENSG0000012349	-2.644479705	####	###	IL13RA2	protein_coding	interleukin 1	X	115003975	115019977
ENSG0000010610	-1.176679488	####	###	GARS1	protein_coding	glycyl-tRNA	7	30580533	30634033
ENSG0000019769	1.001940102	####	###	SPTAN1	protein_coding	spectrin alpl	9	128552558	128633662
ENSG0000006591	-1.280325567	####	###	MTHFD2	protein_coding	methylenete	2	74198610	74217565
ENSG0000016925	1.937208419	####	###	GPRIN1	protein_coding	G protein re	5	176595802	176610156
ENSG0000020385	4.193018648	####	###	H3C15	protein_coding	H3 clusterec	1	149852619	149854274
ENSG0000025569	-3.080051094	####	###	TRIL	protein_coding	TLR4 interac	7	28953358	28958330
ENSG0000011770	-1.400887289	####	###	PROX1	protein_coding	prospero hc	1	213983181	214041510

ENSG0000016820	-1.402379505	####	###	DDIT4	protein_coding	DNA damag	10	72273924	72276036
ENSG0000008215	1.257735012	####	###	BZW1	protein_coding	basic leucin	2	200810594	200827338
ENSG0000016154	-2.230724676	####	###	CYGB	protein_coding	cytoglobin [	17	76527356	76551175
ENSG0000020381	4.162024247	####	###	H3C14	protein_coding	H3 clustere	1	149839538	149841193
ENSG0000015371	-4.298671412	####	###	LURAP1L	protein_coding	leucine rich	9	12775020	12823060
ENSG0000010104	-1.90860419	####	###	ZMYND8	protein_coding	zinc finger M	20	47209214	47356889
ENSG0000010096	-2.945800525	####	###	NFATC4	protein_coding	nuclear fact	14	24365673	24379604
ENSG0000015486	-3.855467815	####	###	PIEZO2	protein_coding	piezo type r	18	10666483	11149569
ENSG0000013070	-1.798577254	####	###	ASS1	protein_coding	argininosuc	9	130444961	130501274
ENSG0000003332	-1.477412924	####	###	GAB2	protein_coding	GRB2 associ	11	78215293	78418348
ENSG0000016612	-1.462194836	####	###	GPT2	protein_coding	glutamic--p	16	46884362	46931289
ENSG0000002999	1.515647154	####	###	HMGB3	protein_coding	high mobilit X		150980509	150990771
ENSG0000011763	0.992812558	####	###	STMN1	protein_coding	stathmin 1 [	1	25884181	25906991
ENSG0000014254	-0.826031735	####	###	RPL13A	protein_coding	ribosomal p	19	49487554	49492308
ENSG0000016290	-1.670919783	####	###	CAPN2	protein_coding	calpain 2 [S	1	223701593	223776018
ENSG0000007562	0.98003332	####	###	ACTB	protein_coding	actin beta [	7	5526409	5563902
ENSG0000013387	-3.080833803	####	###	DUSP26	protein_coding	dual specific	8	33591330	33600023
ENSG0000018467	2.956116557	####	###	H2BC21	protein_coding	H2B cluster	1	149884459	149886652
ENSG0000021014	1.188896915	####	###	MT-TC	Mt_tRNA	mitochondr MT		5761	5826
ENSG0000011800	-2.141861101	####	###	COLEC11	protein_coding	collectin sub	2	3594832	3644644
ENSG0000006304	-0.933210732	####	###	EIF4B	protein_coding	eukaryotic t	12	53006282	53042215
ENSG0000022186	2.522096053	####	###	PLXNA4	protein_coding	plexin A4 [S	7	132123332	132648688
ENSG0000019878	0.759055611	####	###	MT-ND5	protein_coding	mitochondr MT		12337	14148
ENSG0000011701	-1.320410364	####	###	RIMS3	protein_coding	regulating s	1	40620680	40665682
ENSG0000026003	0.939038712	####	###	NORAD	lncRNA	non-coding	20	36045618	36051018
ENSG0000011573	1.352065908	####	###	ID2	protein_coding	inhibitor of	2	8678845	8684461
ENSG0000021394	-1.863786138	####	###	ITGA1	protein_coding	integrin sub	5	52787916	52959209
ENSG0000012621	-2.346087525	####	###	MCF2L	protein_coding	MCF.2 cell li	13	112894378	113099742
ENSG0000000510	-1.405721683	####	###	THSD7A	protein_coding	thrombospo	7	11370365	11832198
ENSG0000021013	1.201529833	####	###	MT-TN	Mt_tRNA	mitochondr MT		5657	5729
ENSG0000016068	-2.660192157	####	###	ZBTB7B	protein_coding	zinc finger a	1	155002630	155018522
ENSG0000010748	1.216866957	####	###	GATA3	protein_coding	GATA bindi	10	8045378	8075198
ENSG0000018249	-1.585490467	####	###	BGN	protein_coding	biglycan [Sc X		153494980	153509546
ENSG0000013051	-8.101690187	####	###	GDF15	protein_coding	growth diffe	19	18374731	18389176
ENSG0000014445	-5.502341229	####	###	ABCA12	protein_coding	ATP binding	2	214931542	215138626
ENSG0000002650	-1.999437302	####	###	CD44	protein_coding	CD44 molec	11	35138882	35232402
ENSG0000015150	1.188016764	####	###	NCAPD3	protein_coding	non-SMC co	11	134150113	134225454
ENSG0000018848	1.723192868	####	###	H2AX	protein_coding	H2A.X varia	11	119093854	119095467
ENSG0000018918	-2.161066225	####	###	PCDH18	protein_coding	protocadhe	4	137518918	137532494
ENSG0000019891	1.010497872	####	###	L1CAM	protein_coding	L1 cell adhe X		153861514	153886173
ENSG0000019795	-2.809603506	####	###	S100A6	protein_coding	S100 calciur	1	153534599	153536244
ENSG0000006673	1.471399547	####	###	KIF26A	protein_coding	kinesin fami	14	104138723	104180894
ENSG0000019615	-4.557885846	####	###	S100A4	protein_coding	S100 calciur	1	153543613	153550136
ENSG0000015584	-2.451839465	####	###	ELMO1	protein_coding	engulfment	7	36854361	37449249
ENSG0000019842	1.194088808	####	###	TCAF1	protein_coding	TRPM8 char	7	143851375	143902198
ENSG0000000660	1.951101527	####	###	FARP2	protein_coding	FERM, ARH	2	241356285	241494841
ENSG0000018489	1.399271016	####	###	H1-10	protein_coding	H1.10 linker	3	129314771	129316286
ENSG0000011301	-0.917794023	####	###	HSPA9	protein_coding	heat shock p	5	138553756	138575416
ENSG0000014505	-1.678937018	####	###	MANF	protein_coding	mesenceph	3	51385291	51389397
ENSG0000007153	-1.177874299	####	###	SEL1L	protein_coding	SEL1L adap	14	81471547	81533853
ENSG0000014010	-1.230408785	####	###	WARS1	protein_coding	tryptophany	14	100333790	100376805
ENSG0000009086	-1.143096178	####	###	AARS1	protein_coding	alanyl-tRNA	16	70251983	70289707
ENSG0000013310	-2.778719617	####	###	SPART	protein_coding	spartin [Sou	13	36301638	36370180
ENSG0000017678	1.39350745	####	###	BASP1	protein_coding	brain abunc	5	17065598	17276843
ENSG0000012202	-1.078030807	####	###	RPL21	protein_coding	ribosomal p	13	27251309	27256691
ENSG0000016854	-7.156623925	####	###	COL3A1	protein_coding	collagen typ	2	188974373	189012746
ENSG0000018101	-1.960386802	####	###	NQO1	protein_coding	NAD(P)H qu	16	69706996	69726668
ENSG0000014536	-1.52759375	####	###	ANK2	protein_coding	ankyrin 2 [S	4	112818032	113384221

ENSG0000018423	-2.910100226	####	###	OAF	protein_coding	out at first h	11	120211032	120230334
ENSG0000021008	0.647676081	####	###	MT-RNR2	Mt_rRNA	mitochondr	MT	1671	3229
ENSG0000009606	1.310761722	####	###	FKBP5	protein_coding	FKBP prolyl	6	35573585	35728583
ENSG0000011731	1.551328749	####	###	ID3	protein_coding	inhibitor of	1	23557926	23559501
ENSG0000018767	-1.414713547	####	###	SPRY4	protein_coding	sprouty RTK	5	142310427	142326455
ENSG0000010042	1.332241471	####	###	CERK	protein_coding	ceramide ki	22	46684410	46738252
ENSG0000001142	1.416019119	####	###	ANLN	protein_coding	anillin actin	7	36389821	36453791
ENSG0000006098	-0.850089569	####	###	BCAT1	protein_coding	branched ch	12	24810024	24949101
ENSG0000012859	-2.980395823	####	###	CCDC136	protein_coding	coiled-coil c	7	128790757	128822132
ENSG0000014673	-2.094384439	####	###	PSPH	protein_coding	phosphoser	7	56011051	56051604
ENSG0000012865	-1.67648662	####	###	CHN1	protein_coding	chimerin 1 [	2	174798809	175005381
ENSG0000019636	-1.37693225	####	###	LONP1	protein_coding	lon peptida	19	5691834	5720572
ENSG0000013233	-3.955421658	####	###	PTPRE	protein_coding	protein tyro	10	127907061	128085855
ENSG0000019866	0.90672513	####	###	CALM1	protein_coding	calmodulin	14	90396502	90408268
ENSG0000018514	-2.655203786	####	###	NPY2R	protein_coding	neuropeptic	4	155208636	155217078
ENSG0000012827	-0.865822097	####	###	ATF4	protein_coding	activating tr	22	39519672	39522683
ENSG0000013174	0.92222126	####	###	TOP2A	protein_coding	DNA topois	17	40388525	40417896
ENSG0000005040	-1.493687469	####	###	LIMA1	protein_coding	LIM domain	12	50175788	50283546
ENSG0000016311	-1.396023278	####	###	PDLIM5	protein_coding	PDZ and LIM	4	94451857	94668227
ENSG0000018301	-1.574438776	####	###	PYCR1	protein_coding	pyrroline-5-	17	81932384	81942412
ENSG0000013370	-1.082702204	####	###	LARS1	protein_coding	leucyl-tRNA	5	146110566	146182696
ENSG0000011381	1.021669858	####	###	SMC4	protein_coding	structural m	3	160399274	160434954
ENSG0000026436	1.348621799	####	###	DYNLL2	protein_coding	dynein light	17	58083419	58095542
ENSG0000012865	-5.105948424	####	###	PDE11A	protein_coding	phosphodie	2	177623244	178072777
ENSG0000027495	-5.867292727	####	###	RPS9	protein_coding	ribosomal p	CHR_HSCHR	54201424	54249604
ENSG0000007157	-1.282986288	####	###	TRIB2	protein_coding	tribbles pse	2	12716889	12742734
ENSG0000013261	-1.575546717	####	###	MTSS2	protein_coding	MTSS I-BAR	16	70661204	70686053
ENSG0000004874	-4.132118822	####	###	CELF2	protein_coding	CUGBP Elav	10	10798397	11336675
ENSG0000016510	-3.714501691	####	###	RASEF	protein_coding	RAS and EF-	9	82979590	83063177
ENSG0000010588	0.907684217	####	###	MTPN	protein_coding	myotrophin	7	135926760	135977359
ENSG0000017519	-1.708630244	####	###	DDIT3	protein_coding	DNA damag	12	57516588	57521737
ENSG0000019888	0.619039411	####	###	MT-ND4	protein_coding	mitochondr	MT	10760	12137
ENSG0000012584	-3.210277076	####	###	FLRT3	protein_coding	fibronectin l	20	14322985	14337614
ENSG0000006422	-1.767110217	####	###	ST3GAL6	protein_coding	ST3 beta-ga	3	98732236	98821201
ENSG0000016698	-1.023165121	####	###	MARS1	protein_coding	methionyl-t	12	57475445	57517569
ENSG0000007546	-1.754858166	####	###	CACNG4	protein_coding	calcium volt	17	66964707	67033398
ENSG0000011674	-1.867409446	####	###	RGS2	protein_coding	regulator of	1	192809039	192812275
ENSG0000014921	0.849992699	####	###	SESN3	protein_coding	sestrin 3 [Sc	11	95165513	95232541
ENSG0000017697	-3.021143825	####	###	DPP7	protein_coding	dipeptidyl p	9	137110546	137115177
ENSG0000007277	1.961537812	####	###	ACADVL	protein_coding	acyl-CoA de	17	7217125	7225266
ENSG0000018377	-2.194186081	####	###	KCTD16	protein_coding	potassium c	5	144170873	144485686
ENSG0000010581	-0.893904755	####	###	CDK6	protein_coding	cyclin deper	7	92604921	92836573
ENSG0000016410	0.811873403	####	###	HAND2	protein_coding	heart and ne	4	173524969	173530229
ENSG0000017333	-2.655680291	####	###	TRIB1	protein_coding	tribbles pse	8	125430358	125438403
ENSG0000010496	-1.166495896	####	###	TLE5	protein_coding	TLE family n	19	3052910	3063107
ENSG0000015757	-2.126632253	####	###	TSPAN18	protein_coding	tetraspanin	11	44726465	44932423
ENSG0000013082	1.665947826	####	###	PLXNA3	protein_coding	plexin A3 [S	X	154458281	154477779
ENSG0000016468	-1.01720251	####	###	ZNF704	protein_coding	zinc finger p	8	80628451	80874781
ENSG0000011529	1.579277183	####	###	TLX2	protein_coding	T cell leuker	2	74513463	74517148
ENSG0000012338	-1.649686338	####	###	LRP1	protein_coding	LDL recepto	12	57128483	57213361
ENSG0000013781	1.181603527	####	###	KNL1	protein_coding	kinetochore	15	40594020	40664342
ENSG0000016069	-1.032330863	####	###	SHC1	protein_coding	SHC adapto	1	154962298	154974395
ENSG0000008082	0.711017074	####	###	HSP90AA1	protein_coding	heat shock p	14	102080742	102139699
ENSG0000011191	-1.179125804	####	###	NCOA7	protein_coding	nuclear rece	6	125781161	125932034
ENSG0000012025	-1.698183551	####	###	MTHFD1L	protein_coding	methylenete	6	150865549	151101887
ENSG0000026602	1.761138431	####	###	SRGAP2	protein_coding	SLIT-ROBO	1	206203345	206464443
ENSG0000010463	-1.109623199	####	###	SLC39A14	protein_coding	solute carrie	8	22367249	22434129
ENSG0000013278	0.940134475	####	###	NASP	protein_coding	nuclear auto	1	45583846	45618904

ENSG0000014315	-1.310126922	####	###	ATP1B1	protein_codinc	ATPase Na+	1	169105697	169132722
ENSG0000016410	0.930854874	####	###	HMGB2	protein_codinc	high mobilit	4	173331376	173334432
ENSG0000013100	-1.818283932	####	###	TXLNGY	transcribed_un	taxilin gamr	Y	19567313	19606274
ENSG0000025327	-2.131573632	####	###	CCDC71L	protein_codinc	coiled-coil c	7	106654360	106661158
ENSG0000016428	-1.55855361	####	###	GRPEL2	protein_codinc	GrpE like 2,	5	149345430	149354583
ENSG0000011575	1.101397275	####	###	ODC1	protein_codinc	ornithine de	2	10439968	10448327
ENSG0000015465	-1.674626225	####	###	NCAM2	protein_codinc	neural cell a	21	20998409	21543329
ENSG0000013459	1.865451677	####	###	RTL8C	protein_codinc	retrotranspc	X	135032355	135033546
ENSG0000015579	-2.513645651	####	###	DEPTOR	protein_codinc	DEP domair	8	119873717	120050918
ENSG0000011167	-1.926983869	####	###	C12orf57	protein_codinc	chromosom	12	6942978	6946003
ENSG0000010528	-1.170044499	####	###	SLC1A5	protein_codinc	solute carrie	19	46774883	46788594
ENSG0000014045	2.661373443	####	###	ARRDC4	protein_codinc	arrestin don	15	97960703	97973833
ENSG0000014877	0.882744705	####	###	MKI67	protein_codinc	marker of p	10	128096659	128126423
ENSG0000017887	2.315182971	####	###	APOLD1	protein_codinc	apolipoprot	12	12725917	12829975
ENSG0000019738	1.296320548	####	###	HTT	protein_codinc	huntingtin [	4	3041422	3243960
ENSG0000018377	-2.990156247	####	###	B3GALT5	protein_codinc	beta-1,3-ga	21	39556442	39673137
ENSG0000015323	-1.548799421	####	###	PTPRR	protein_codinc	protein tyro	12	70638073	70920738
ENSG0000009920	-1.018995839	####	###	ABLIM1	protein_codinc	actin bindin	10	114431113	114768061
ENSG0000007204	-1.642813657	####	###	SLC6A15	protein_codinc	solute carrie	12	84859491	84913615
ENSG0000017221	-2.049461188	####	###	CEBPB	protein_codinc	CCAAT enha	20	50190830	50192668
ENSG0000016245	-3.602858271	####	###	FBLIM1	protein_codinc	filamin bind	1	15756607	15786594
ENSG0000016724	-4.250293384	####	###	IGF2	protein_codinc	insulin like c	11	2129112	2141238
ENSG0000013301	-4.056648258	####	###	CHRM3	protein_codinc	cholinergic r	1	239386565	239915452
ENSG0000014588	2.143304954	####	###	PCYOX1L	protein_codinc	prenylcystei	5	149358037	149369653
ENSG0000017506	-0.835831841	####	###	SNHG29	lncRNA	small nucleo	17	16438767	16478678
ENSG0000013869	-1.687303565	####	###	BMPR1B	protein_codinc	bone morph	4	94757955	95158448
ENSG0000011759	-4.999038504	####	###	IRF6	protein_codinc	interferon re	1	209785617	209806175
ENSG0000017515	-2.004843152	####	###	YPEL2	protein_codinc	yippee like 2	17	59331655	59401729
ENSG0000000828	-2.062908148	####	###	CYB561	protein_codinc	cytochrome	17	63432304	63446354
ENSG0000014580	-1.566186984	####	###	ADAMTS19	protein_codinc	ADAM meta	5	129460281	129738683
ENSG0000018017	-1.690619582	####	###	TH	protein_codinc	tyrosine hyc	11	2163929	2171815
ENSG0000017215	-2.398078709	####	###	FRMD3	protein_codinc	FERM doma	9	83242990	83538546
ENSG0000008661	-3.497241361	####	###	ERO1B	protein_codinc	endoplasmi	1	236215101	236281958
ENSG0000021014	1.216404225	####	###	MT-TY	Mt_tRNA	mitochondr	MT	5826	5891
ENSG0000027219	3.482195385	####	###	H2AC19	protein_codinc	H2A cluster	1	149851061	149851624
ENSG0000019869	0.833032855	####	###	MT-ND6	protein_codinc	mitochondr	MT	14149	14673
ENSG0000016967	1.327014857	####	###	BUB1	protein_codinc	BUB1 mitoti	2	110637528	110678063
ENSG0000016625	1.154609518	####	###	SCN3B	protein_codinc	sodium volt	11	123629187	123655244
ENSG0000013552	-2.516764562	####	###	MAP7	protein_codinc	microtubule	6	136342281	136550819
ENSG0000017320	2.140623562	####	###	ABCD2	protein_codinc	ATP binding	12	39550033	39619803
ENSG0000017604	-5.198952939	####	###	NUPR1	protein_codinc	nuclear prot	16	28532708	28539008
ENSG0000015665	-1.585168833	####	###	KAT6B	protein_codinc	lysine acetyl	10	74824927	75032624
ENSG0000027635	-6.074536011	####	###	AC004556.	misc_RNA		KI270721.1	52666	52734
ENSG0000015387	-1.48718543	####	###	CEBPG	protein_codinc	CCAAT enha	19	33373685	33382686
ENSG0000016461	1.346267912	####	###	PTTG1	protein_codinc	PTTG1 regu	5	160421855	160428739
ENSG0000018513	-5.051424158	####	###	INPP5J	protein_codinc	inositol poly	22	31122731	31134697
ENSG0000013684	-1.020711139	####	###	TMOD1	protein_codinc	tropomodul	9	97501180	97601743
ENSG0000018707	0.848477102	####	###	TEAD1	protein_codinc	TEA domair	11	12674421	12944737
ENSG0000025530	1.109206943	####	###	EID1	protein_codinc	EP300 intera	15	48878134	48880173
ENSG0000020381	3.484781904	####	###	H2AC18	protein_codinc	H2A cluster	1	149842188	149842736
ENSG0000026091	-1.380612406	####	###	CCPG1	protein_codinc	cell cycle pr	15	55340032	55408510
ENSG0000013730	-1.309467075	####	###	HMGA1	protein_codinc	high mobilit	6	34236873	34246231
ENSG0000015388	-1.760994268	####	###	KCTD15	protein_codinc	potassium c	19	33795933	33815763
ENSG0000016494	-4.730651789	####	###	FREM1	protein_codinc	FRAS1 relat	9	14734666	14910995
ENSG0000027824	-5.976725622	####	###	NA	NA	NA	NA	NA	NA
ENSG0000018219	-1.104478977	####	###	SHMT2	protein_codinc	serine hydrc	12	57229573	57234935
ENSG0000012586	-2.808933423	####	###	LAMP5	protein_codinc	lysosomal a	20	9514358	9530524
ENSG0000018283	-1.346502209	####	###	PLCXD3	protein_codinc	phosphatidy	5	41306952	41510628

ENSG0000019774	0.724718337	####	###	PSAP	protein_coding	prosaposin	10	71816298	71851325
ENSG0000010109	-1.719220759	####	###	RIMS4	protein_coding	regulating s	20	44751808	44810546
ENSG0000017001	-0.842612042	####	###	ALCAM	protein_coding	activated le	3	105366909	105576900
ENSG0000016910	-1.054487945	####	###	SLC25A6	protein_coding	solute carrie	X	1386152	1392113
ENSG0000013755	-4.778236265	####	###	PI15	protein_coding	peptidase ir	8	74824534	74855029
ENSG0000011340	-0.937750424	####	###	TARS1	protein_coding	threonyl-tR	5	33440696	33468091
ENSG0000014361	-0.927664768	####	###	C1orf43	protein_coding	chromosom	1	154206696	154220637
ENSG0000012277	-1.00359155	####	###	KIAA1549	protein_coding	KIAA1549 [S	7	138831381	138981389
ENSG0000013877	1.081124091	####	###	CENPE	protein_coding	centromere	4	103105349	103198445
ENSG0000003108	-1.385816196	####	###	ARHGAP31	protein_coding	Rho GTPase	3	119294383	119420714
ENSG0000013816	1.114995728	####	###	KIF11	protein_coding	kinesin fami	10	92593130	92655395
ENSG0000016580	-3.748313178	####	###	ZNF219	protein_coding	zinc finger p	14	21090077	21104722
ENSG0000016488	-1.070152714	####	###	SLC4A2	protein_coding	solute carrie	7	151057210	151076526
ENSG0000019630	-0.855240858	####	###	IARS1	protein_coding	isoleucyl-tR	9	92210207	92293756
ENSG0000016268	-1.80362495	####	###	KCNT2	protein_coding	potassium s	1	196225779	196609225
ENSG0000013450	-2.543305114	####	###	CABLES1	protein_coding	Cdk5 and A	18	23134564	23260467
ENSG0000022189	2.327741965	####	###	NPTXR	protein_coding	neuronal pe	22	38818452	38844028
ENSG0000014785	-1.477443687	####	###	VLDLR	protein_coding	very low de	9	2621787	2660056
ENSG0000015550	-0.789036354	####	###	LARP1	protein_coding	La ribonucle	5	154712843	154817605
ENSG0000012816	-3.161491823	####	###	ADM2	protein_coding	adrenomed	22	50481543	50486440
ENSG0000014667	-3.445885823	####	###	IGFBP3	protein_coding	insulin like g	7	45912245	45921874
ENSG0000014119	-3.310589717	####	###	TOM1L1	protein_coding	target of my	17	54899387	54961956
ENSG0000016409	-0.836756241	####	###	PRSS12	protein_coding	serine prote	4	118280038	118353003
ENSG0000014287	-1.202659391	####	###	PRKACB	protein_coding	protein kina	1	84078062	84238498
ENSG0000010694	-2.203763718	####	###	AKNA	protein_coding	AT-hook tra	9	114334156	114394405
ENSG0000010798	-1.651432382	####	###	DKK1	protein_coding	dickkopf WI	10	52314281	52318042
ENSG0000018421	-1.32563818	####	###	IRAK1	protein_coding	interleukin 1	X	154010506	154019902
ENSG0000006704	-1.499206406	####	###	DDX3Y	protein_coding	DEAD-box I	Y	12904108	12920478
ENSG0000011990	-1.729459232	####	###	OGFRL1	protein_coding	opioid grow	6	71288811	71309059
ENSG0000007664	2.570332243	####	###	PAG1	protein_coding	phosphopro	8	80967810	81112068
ENSG0000018749	-2.366591009	####	###	COL4A1	protein_coding	collagen typ	13	110148963	110307157
ENSG0000020536	-3.870361458	####	###	INSYN1	protein_coding	inhibitory sy	15	73735431	73752747
ENSG0000018216	0.773650608	####	###	UNC5C	protein_coding	unc-5 netrir	4	95162504	95549206
ENSG0000012087	-1.131625611	####	###	DUSP4	protein_coding	dual specific	8	29333064	29350684
ENSG0000002018	-2.014054977	####	###	ADGRA2	protein_coding	adhesion G	8	37784191	37844896
ENSG0000011061	-1.543504166	####	###	CARS1	protein_coding	cysteinyl-tR	11	3000922	3057613
ENSG0000001381	1.232139499	####	###	TACC3	protein_coding	transforming	4	1712891	1745171
ENSG0000013072	0.762037524	####	###	PRRC2B	protein_coding	proline rich	9	131373636	131500197
ENSG0000003842	-0.829444423	####	###	VCAN	protein_coding	versican [So	5	83471618	83582303
ENSG0000006627	0.816576159	####	###	ASPM	protein_coding	assembly fa	1	197084127	197146694
ENSG0000017601	1.920334289	####	###	TUBB6	protein_coding	tubulin beta	18	12307669	12344320
ENSG0000011712	-3.77817266	####	###	MFAP2	protein_coding	microfibril a	1	16974502	16980632
ENSG0000016973	1.819046307	####	###	DCXR	protein_coding	dicarbonyl a	17	82035136	82037709
ENSG0000015115	-1.687484617	####	###	ANK3	protein_coding	ankyrin 3 [S	10	60026298	60733490
ENSG0000012264	1.532044312	####	###	FKBP9	protein_coding	FKBP prolyl	7	32957404	33006930
ENSG0000019874	-1.288304949	####	###	SLC5A3	protein_coding	solute carrie	21	34073578	34106260
ENSG0000011759	-1.510298537	####	###	UTP25	protein_coding	UTP25 smal	1	209827972	209857565
ENSG0000010278	-1.389780216	####	###	INTS6	protein_coding	integrator c	13	51354077	51454264
ENSG0000012174	-1.246142527	####	###	ZMYM2	protein_coding	zinc finger N	13	19958670	20091829
ENSG0000013771	1.517379141	####	###	PPP2R1B	protein_coding	protein pho	11	111726908	111766389
ENSG0000013284	-1.917933873	####	###	ZBED3	protein_coding	zinc finger E	5	77072072	77087285
ENSG0000014753	1.980783425	####	###	GINS4	protein_coding	GINS compl	8	41529218	41545030
ENSG0000009919	-0.731245993	####	###	SCD	protein_coding	stearoyl-Co	10	100347233	100364826
ENSG0000010258	-1.245580643	####	###	DNAJC3	protein_coding	DnaJ heat sl	13	95677139	95794988
ENSG0000015172	1.370969993	####	###	CENPU	protein_coding	centromere	4	184694085	184734130
ENSG0000019662	-0.814051209	####	###	TCF4	protein_coding	transcription	18	55222185	55664787
ENSG0000016752	-0.835873035	####	###	ANKRD11	protein_coding	ankyrin repe	16	89267630	89490561
ENSG0000018815	-2.304451976	####	###	NHS	protein_coding	NHS actin re	X	17375200	17735994

ENSG0000018444	1.125192014	####	###	KNTC1	protein_coding	kinetochore		12	122527246	122626396
ENSG0000007124	-1.635455958	####	###	VASH1	protein_coding	vasohibin 1		14	76761468	76783015
ENSG0000013780	1.059691139	####	###	NUSAP1	protein_coding	nucleolar ar		15	41320794	41381050
ENSG0000021012	1.214057651	####	###	MT-TA	Mt_tRNA	mitochondr	MT		5587	5655
ENSG0000009285	1.938435327	####	###	CLSPN	protein_coding	claspin [Sou		1	35720218	35769978
ENSG0000009284	0.852985366	####	###	MYL6	protein_coding	myosin light		12	56158346	56163496
ENSG0000000740	-2.524595025	####	###	CACNA2D2	protein_coding	calcium volt		3	50362799	50504244
ENSG0000012982	-1.654356424	####	###	RPS4Y1	protein_coding	ribosomal p	Y		2841602	2932000
ENSG0000012597	1.073603207	####	###	RALY	protein_coding	RALY heterc		20	33993646	34108308
ENSG0000017092	-1.136364869	####	###	TANC2	protein_coding	tetratricope		17	63009556	63427699
ENSG0000016387	4.631792104	####	###	GRIK3	protein_coding	glutamate ic		1	36795527	37034515
ENSG0000007910	-2.615731491	####	###	RUNX1T1	protein_coding	RUNX1 part		8	91954967	92103286
ENSG0000017877	-4.195090803	####	###	CPNE7	protein_coding	copine 7 [Sc		16	89575758	89597246
ENSG0000018248	0.891921773	####	###	KPNA2	protein_coding	karyopherin		17	68035708	68046854
ENSG0000018926	1.069245615	####	###	PNRC2	protein_coding	proline rich		1	23956839	23963462
ENSG0000021158	-2.087370209	####	###	SLC48A1	protein_coding	solute carrie		12	47753916	47782751
ENSG0000016273	1.028591633	####	###	PEA15	protein_coding	proliferatior		1	160205380	160215376
ENSG0000015382	-4.818331846	####	###	PID1	protein_coding	phosphotyro		2	228850526	229271287
ENSG0000014599	-1.899517774	####	###	CDKAL1	protein_coding	CDK5 regula		6	20534457	21232404
ENSG0000014572	-7.550565687	####	###	LIX1	protein_coding	limb and CN		5	97091867	97142753
ENSG0000000665	-1.180762383	####	###	IFRD1	protein_coding	interferon re		7	112422887	112481017
ENSG0000007257	1.238056454	####	###	HMMR	protein_coding	hyaluronan		5	163460203	163491941
ENSG0000014567	-0.961394832	####	###	PIK3R1	protein_coding	phosphoino		5	68215756	68301821
ENSG0000011009	0.666463686	####	###	CCND1	protein_coding	cyclin D1 [S		11	69641156	69654474
ENSG0000007933	-4.392784001	####	###	RAPGEF3	protein_coding	Rap guanine		12	47734363	47771040
ENSG0000018271	0.788984949	####	###	ANXA2	protein_coding	annexin A2		15	60347134	60402883
ENSG0000020381	3.44952131	####	###	NA	NA	NA	NA	NA	NA	
ENSG0000010716	-8.469993958	####	###	TYRP1	protein_coding	tyrosinase re		9	12685439	12710285
ENSG0000015861	-0.947841113	####	###	PPP1R15B	protein_coding	protein pho		1	204403381	204411817
ENSG0000015566	-0.852340033	####	###	PDIA4	protein_coding	protein disu		7	149003062	149028662
ENSG0000014393	0.705266042	####	###	CALM2	protein_coding	calmodulin		2	47160083	47176921
ENSG0000007041	1.41917634	####	###	DGCR2	protein_coding	DiGeorge sy		22	19036282	19122454
ENSG0000012070	-2.543282244	####	###	TGFBI	protein_coding	transforming		5	136028988	136063818
ENSG0000012259	2.720038283	####	###	FAM126A	protein_coding	family with s		7	22889371	23014130
ENSG0000026999	1.872307979	####	###	KC877982.1	lncRNA	novel transc	X		151182386	151182855
ENSG0000017172	-2.523697397	####	###	TMEM51	protein_coding	transmembr		1	15152532	15220478
ENSG0000025477	-0.566195829	####	###	EEF1G	protein_coding	eukaryotic t		11	62559596	62574086
ENSG0000005435	1.599919239	####	###	PTPRN	protein_coding	protein tyro		2	219289623	219309648
ENSG0000015885	-3.330375351	####	###	ADAMTS4	protein_coding	ADAM meta		1	161184302	161199054
ENSG0000017055	-0.86235495	####	###	CDH2	protein_coding	cadherin 2 [		18	27932879	28177946
ENSG0000016840	-3.339686917	####	###	CMAHP	transcribed_un	cytidine mo		6	25061626	25452263
ENSG0000012621	0.943824716	####	###	KLC1	protein_coding	kinesin light		14	103561896	103714249
ENSG0000013410	-1.389406873	####	###	EDEM1	protein_coding	ER degrada		3	5187646	5219958
ENSG0000012859	-1.5417589	####	###	DNAJB9	protein_coding	DnaJ heat sl		7	108569867	108574850
ENSG0000016493	1.470147574	####	###	TP53INP1	protein_coding	tumor prote		8	94925972	94949378
ENSG0000020462	-0.596268885	####	###	RACK1	protein_coding	receptor for		5	181236897	181248096
ENSG0000010408	1.682001835	####	###	BMF	protein_coding	Bcl2 modify		15	40087890	40108892
ENSG0000010596	1.062402628	####	###	H2AZ2	protein_coding	H2A.Z varia		7	44826791	44848087
ENSG0000017149	-0.790811766	####	###	RSL1D1	protein_coding	ribosomal L		16	11833850	11851580
ENSG0000013432	-0.61306823	####	###	MYCN	protein_coding	MYCN proto		2	15940550	15947007
ENSG0000016349	-2.248445003	####	###	FEV	protein_coding	FEV transcri		2	218981087	218985184
ENSG0000019653	-0.677371185	####	###	NACA	protein_coding	nascent poly		12	56712427	56731628
ENSG0000019880	0.575448805	####	###	MT-CO1	protein_coding	mitochondr	MT		5904	7445
ENSG0000013611	-1.330789858	####	###	TBC1D4	protein_coding	TBC1 doma		13	75283503	75482169
ENSG0000015304	1.32091837	####	###	CARHSP1	protein_coding	calcium regu		16	8852942	8869012
ENSG0000011034	0.956893328	####	###	UBE4A	protein_coding	ubiquitinatic		11	118359600	118399211
ENSG0000010980	0.979931903	####	###	NCAPG	protein_coding	non-SMC cc		4	17810979	17844865
ENSG0000014879	0.913375393	####	###	INA	protein_coding	internexin n		10	103277138	103290346

ENSG0000013772	0.916625264	####	###	FXYD6	protein_coding	FXYD doma	11	117836976	117877486
ENSG0000015680	0.963863293	####	###	ATAD2	protein_coding	ATPase fam	8	123319850	123416350
ENSG0000014642	1.07464923	####	###	DYNLT1	protein_coding	dynein light	6	158636474	158644743
ENSG0000015501	-4.947993051	####	###	DKK2	protein_coding	dickkopf W	4	106921802	107283806
ENSG0000017195	-0.797917875	####	###	SCG2	protein_coding	secretogran	2	223596940	223602361
ENSG0000017031	1.102356196	####	###	CDK1	protein_coding	cyclin deper	10	60778331	60794852
ENSG0000016573	-0.757887451	####	###	DDX21	protein_coding	DExD-box h	10	68956170	68985068
ENSG0000010144	-1.361548901	####	###	PPP1R16B	protein_coding	protein pho	20	38805697	38923024
ENSG0000011728	-3.163141697	####	###	RAB29	protein_coding	RAB29, mer	1	205767986	205775482
ENSG0000018648	-2.075175375	####	###	MYT1L	protein_coding	myelin trans	2	1789113	2331664
ENSG0000023795	-2.127789492	####	###	AL445218.1	lncRNA	novel transc	1	95356229	95381000
ENSG0000015464	-2.049095778	####	###	CHODL	protein_coding	chondrolect	21	17901263	18267373
ENSG0000016629	-3.924253509	####	###	TMEM100	protein_coding	transmemb	17	55719627	55732121
ENSG0000019857	-3.29528254	####	###	RD3	protein_coding	retinal dege	1	211476522	211492162
ENSG0000021532	-1.623227328	####	###	HSPA1A	protein_coding	heat shock	CHR_HSCHR	31805699	31808181
ENSG0000007391	-1.421839384	####	###	FRY	protein_coding	FRY microtu	13	31846713	32299125
ENSG0000016597	-4.043275371	####	###	NELL1	protein_coding	neural EGFL	11	20669551	21575686
ENSG0000000533	-1.064316993	####	###	CREBBP	protein_coding	CREB bindir	16	3725054	3880713
ENSG0000019895	2.121827396	####	###	NAGA	protein_coding	alpha-N-ac	22	42058334	42070842
ENSG0000005062	-2.157121965	####	###	PTGER3	protein_coding	prostagland	1	70852353	71047808
ENSG0000013681	1.445732414	####	###	ODF2	protein_coding	outer dense	9	128455186	128501292
ENSG0000016393	-0.915840111	####	###	TKT	protein_coding	transketolas	3	53224712	53256052
ENSG0000017311	-1.12734167	####	###	LRRN3	protein_coding	leucine rich	7	111091006	111125454
ENSG0000018821	-1.90767728	####	###	NCR3LG1	protein_coding	natural kille	11	17351800	17377341
ENSG0000010623	2.005614742	####	###	NPTX2	protein_coding	neuronal pe	7	98617285	98629869
ENSG0000017899	-1.278602202	####	###	SNX18	protein_coding	sorting nexi	5	54517759	54546586
ENSG0000019882	1.006822551	####	###	INPP5F	protein_coding	inositol poly	10	119726042	119829147
ENSG0000001344	-0.966988241	####	###	CLK1	protein_coding	CDC like kin	2	200853009	200864744
ENSG0000014052	1.268394074	####	###	POLG	protein_coding	DNA polym	15	89305198	89334861
ENSG0000017000	-1.107202598	####	###	CHD3	protein_coding	chromodom	17	7884796	7912760
ENSG0000013726	1.016836484	####	###	TUBB2A	protein_coding	tubulin beta	6	3153666	3157544
ENSG0000007854	2.129202834	####	###	ADCYAP1R	protein_coding	ADCYAP rec	7	31052308	31111479
ENSG0000013935	1.505848917	####	###	GAS2L3	protein_coding	growth arre	12	100573683	100628288
ENSG0000016273	-1.424743827	####	###	VANGL2	protein_coding	VANGL plar	1	160400564	160428670
ENSG0000015680	-2.971813747	####	###	FBXO32	protein_coding	F-box prote	8	123497889	123541206
ENSG0000013662	-0.802726511	####	###	EPRS1	protein_coding	glutamyl-pr	1	219968600	220046530
ENSG0000010348	-1.844226273	####	###	QPRT	protein_coding	quinolinate	16	29663279	29698699
ENSG0000014691	1.096994531	####	###	NCAPG2	protein_coding	non-SMC c	7	158631169	158704804
ENSG0000012418	0.897249008	####	###	PLCG1	protein_coding	phospholipa	20	41136960	41196801
ENSG0000003511	-1.743189978	####	###	SH3YL1	protein_coding	SH3 and SY	2	217730	266398
ENSG0000019678	3.279892419	####	###	H2AC11	protein_coding	H2A cluster	6	27133042	27135291
ENSG0000020413	-2.426667432	####	###	NHSL2	protein_coding	NHS like 2 [	X	71910818	72161750
ENSG0000014322	-1.814937675	####	###	UFC1	protein_coding	ubiquitin-fc	1	161152776	161158856
ENSG0000012952	2.313882042	####	###	EGLN3	protein_coding	egl-9 family	14	33924227	34462774
ENSG0000024226	0.696338055	####	###	PEG10	protein_coding	paternally e	7	94656325	94669695
ENSG0000014539	-1.039935855	####	###	SETD7	protein_coding	SET domain	4	139495941	139606699
ENSG0000016542	2.137017828	####	###	ZCCHC24	protein_coding	zinc finger C	10	79382325	79445624
ENSG0000016567	-0.960140844	####	###	NSD1	protein_coding	nuclear rece	5	177133025	177300213
ENSG0000015837	2.019149162	####	###	H2BC5	protein_coding	H2B cluster	6	26158146	26171349
ENSG0000014524	1.049279218	####	###	ATP10D	protein_coding	ATPase pho	4	47485275	47593486
ENSG0000017669	-4.008863464	####	###	BDNF	protein_coding	brain derive	11	27654893	27722058
ENSG0000006506	-1.083804591	####	###	UHRF1BP1	protein_coding	UHRF1 bind	6	34792015	34883138
ENSG0000017100	-4.258566751	####	###	HS6ST2	protein_coding	heparan sul	X	132626016	132961395
ENSG0000012830	-1.273098504	####	###	MPST	protein_coding	mercaptopy	22	37019635	37029822
ENSG0000021494	1.132622235	####	###	ARHGEF28	protein_coding	Rho guanin	5	73626158	73941993
ENSG0000017614	-1.434027093	####	###	TMEM39A	protein_coding	transmemb	3	119428949	119468830
ENSG0000012295	1.35988242	####	###	ZWINT	protein_coding	ZW10 intera	10	56357227	56361273
ENSG0000017516	0.813417305	####	###	PSMD2	protein_coding	proteasome	3	184299198	184309050

ENSG0000010081	-1.058997401	####	###	CCNB1IP1	protein_codinc	cyclin B1 int	14	20311368	20333312
ENSG0000016492	0.695029557	####	###	YWHAZ	protein_codinc	tyrosine 3-r	8	100916523	100953388
ENSG0000025335	0.861801577	####	###	TUG1	protein_codinc	taurine up-r	22	30969245	30979395
ENSG0000016700	-0.81015356	####	###	PDIA3	protein_codinc	protein disu	15	43746410	43773278
ENSG0000023712	0.632603965	####	###	HAND2-AS	lncRNA	HAND2 anti	4	173527270	173659696
ENSG0000011990	1.273245686	####	###	SLF2	protein_codinc	SMC5-SMC	10	100912963	100965134
ENSG0000012031	-1.776634416	####	###	ARAP3	protein_codinc	ArfGAP with	5	141653401	141682230
ENSG0000010024	1.16402386	####	###	SBF1	protein_codinc	SET binding	22	50445000	50475035
ENSG0000009928	-1.094330716	####	###	MACROH2	protein_codinc	macroH2A.2	10	70052846	70112282
ENSG0000009052	-0.962294202	####	###	DNAJB11	protein_codinc	DnaJ heat sl	3	186567403	186585800
ENSG0000008306	-5.892023871	####	###	TRPM3	protein_codinc	transient rec	9	70529063	71446904
ENSG0000011590	-1.166197397	####	###	SLC1A4	protein_codinc	solute carrie	2	64988477	65023865
ENSG0000013926	-7.013857749	####	###	INHBE	protein_codinc	inhibin subu	12	57452323	57459280
ENSG0000005729	-2.703427496	####	###	PKP2	protein_codinc	plakophilin	12	32790755	32896777
ENSG0000009205	-1.73972314	####	###	JPH4	protein_codinc	junctophilin	14	23568035	23578790
ENSG0000014155	1.339439752	####	###	TBCD	protein_codinc	tubulin foldi	17	82752065	82945914
ENSG0000019732	-1.518210125	####	###	SVIL	protein_codinc	supervillin [	10	29457338	29736959
ENSG0000013615	-1.013367838	####	###	ITM2B	protein_codinc	integral mer	13	48232612	48270357
ENSG0000019712	-1.923710896	####	###	SRC	protein_codinc	SRC proto-c	20	37344685	37406050
ENSG0000000398	1.037741971	####	###	SLC7A2	protein_codinc	solute carrie	8	17497088	17570573
ENSG0000007206	1.420372687	####	###	PRKACA	protein_codinc	protein kina	19	14091688	14118084
ENSG0000015237	-2.320671732	####	###	SPOCK1	protein_codinc	SPARC (oste	5	136975298	137598379
ENSG0000016308	-2.597298963	####	###	SGPP2	protein_codinc	sphingosine	2	222424543	222562621
ENSG0000012822	-2.751977657	####	###	SDF2L1	protein_codinc	stromal cell	22	21642302	21644299
ENSG0000010626	0.801960407	####	###	ZKSCAN1	protein_codinc	zinc finger v	7	100015572	100041689
ENSG0000022705	0.847565319	####	###	C14orf132	protein_codinc	chromosom	14	96039324	96093889
ENSG0000001999	-2.907100097	####	###	HGF	protein_codinc	hepatocyte	7	81699010	81770438
ENSG0000013101	0.783850818	####	###	AKAP12	protein_codinc	A-kinase an	6	151239967	151358559
ENSG0000016861	1.032590266	####	###	STAT3	protein_codinc	signal transc	17	42313324	42388482
ENSG0000018723	1.041175104	####	###	FNBP1	protein_codinc	formin bind	9	129887187	130043194
ENSG0000013511	0.793230864	####	###	TBX3	protein_codinc	T-box trans	12	114670255	114684175
ENSG0000019862	-1.39990263	####	###	MDM4	protein_codinc	MDM4 regu	1	204516379	204558120
ENSG0000013459	-3.525191434	####	###	RAB33A	protein_codinc	RAB33A, me	X	130171962	130184870
ENSG0000012045	1.025692487	####	###	SNX19	protein_codinc	sorting nexi	11	130875436	130916509
ENSG0000016950	-2.892563762	####	###	SLC38A11	protein_codinc	solute carrie	2	164896186	164955525
ENSG0000017186	-0.592972205	####	###	RPS7	protein_codinc	ribosomal p	2	3575260	3580920
ENSG0000017731	-1.678226414	####	###	ZBTB38	protein_codinc	zinc finger a	3	141324213	141449792
ENSG0000012296	1.202292019	####	###	CIT	protein_codinc	citron rho-ir	12	119685791	119877320
ENSG0000016411	-1.05244891	####	###	FBXO8	protein_codinc	F-box prote	4	174236658	174283667
ENSG0000025156	0.692200804	####	###	MALAT1	lncRNA	metastasis a	11	65497688	65506516
ENSG0000010031	-0.635701977	####	###	RPL3	protein_codinc	ribosomal p	22	39312882	39320389
ENSG0000010133	-1.04127631	####	###	PLCB4	protein_codinc	phospholipa	20	9068763	9481242
ENSG0000017869	-0.93340996	####	###	KCTD12	protein_codinc	potassium c	13	76880175	76886405
ENSG0000015793	1.110110856	####	###	SKI	protein_codinc	SKI proto-o	1	2228319	2310213
ENSG0000017891	0.976001664	####	###	TAF7	protein_codinc	TATA-box b	5	141260225	141320784
ENSG0000000447	-0.929073813	####	###	FKBP4	protein_codinc	FKBP prolyl	12	2794970	2805423
ENSG0000013648	0.738956841	####	###	DCAF7	protein_codinc	DDB1 and C	17	63550477	63594279
ENSG0000022032	3.379329031	####	###	H2BC19P	transcribed_un	H2B clustere	1	149842875	149846486
ENSG0000013818	1.11464821	####	###	KIF20B	protein_codinc	kinesin fami	10	89701610	89774939
ENSG0000001102	1.48069624	####	###	MRC2	protein_codinc	mannose re	17	62627670	62693597
ENSG0000010501	1.815060793	####	###	ASF1B	protein_codinc	anti-silencir	19	14119512	14136613
ENSG0000006430	0.899616892	####	###	CDON	protein_codinc	cell adhesio	11	125955796	126063335
ENSG0000007869	-1.236013205	####	###	CBFA2T2	protein_codinc	CBFA2/RUN	20	33490075	33650036
ENSG0000007522	-1.26638286	####	###	SEMA3C	protein_codinc	semaphorin	7	80742538	80922359
ENSG0000013548	-0.552805618	####	###	HNRNPA1	protein_codinc	heterogene	12	54280193	54287088
ENSG0000017423	-1.964284979	####	###	ADCY6	protein_codinc	adenylate cy	12	48766194	48789037
ENSG0000013787	-0.853038605	####	###	RSL24D1	protein_codinc	ribosomal L	15	55180806	55197049
ENSG0000017389	-1.328639384	####	###	SPTBN2	protein_codinc	spectrin bet	11	66682497	66729226

ENSG0000016854	2.322019362	####	###	GFRA2	protein_coding	GDNF family	8	21690398	21812357
ENSG0000014354	0.738457641	####	###	TPM3	protein_coding	tropomyosin	1	154155304	154194648
ENSG0000013470	-3.229647392	####	###	HOOK1	protein_coding	hook microt	1	59814786	59876322
ENSG0000010537	-0.850200707	####	###	RPS19	protein_coding	ribosomal p	19	41860255	41872925
ENSG0000018885	-3.607137366	####	###	FAM78B	protein_coding	family with s	1	166057426	166167001
ENSG0000017741	-1.05214645	####	###	ZFAS1	lncRNA	ZNFX1 antis	20	49278178	49299600
ENSG0000017545	0.797946879	####	###	CCDC14	protein_coding	coiled-coil c	3	123897305	123961408
ENSG0000017521	0.714997041	####	###	CKAP5	protein_coding	cytoskeleton	11	46743048	46846308
ENSG0000017494	1.959618807	####	###	KCTD13	protein_coding	potassium c	16	29905012	29926236
ENSG0000009112	-0.806102119	####	###	NRCAM	protein_coding	neuronal ce	7	108147623	108456717
ENSG0000007392	0.782148339	####	###	PICALM	protein_coding	phosphatidy	11	85957175	86069882
ENSG0000023303	6.06890578	####	###	AC243772.2	lncRNA	novel transc	1	149785659	149793020
ENSG0000018561	-1.854959522	####	###	PDIA2	protein_coding	protein disu	16	283164	287215
ENSG0000010009	1.274381447	####	###	SEZ6L	protein_coding	seizure relat	22	26169462	26383597
ENSG0000021290	0.662110296	####	###	MT-ND4L	protein_coding	mitochondr	MT	10470	10766
ENSG0000009053	-4.668784417	####	###	P3H2	protein_coding	prolyl 3-hyc	3	189956728	190122437
ENSG0000025894	0.785531227	####	###	TUBB3	protein_coding	tubulin beta	16	89921392	89938761
ENSG0000019888	0.496092506	####	###	MT-ND1	protein_coding	mitochondr	MT	3307	4262
ENSG0000016207	2.05155141	####	###	PAQR4	protein_coding	progestin ar	16	2969270	2973484
ENSG0000012345	-4.399879205	####	###	DBH	protein_coding	dopamine b	9	133636363	133659329
ENSG0000015009	0.696141236	####	###	ITGB1	protein_coding	integrin sub	10	32900318	33005792
ENSG0000020387	-0.92104995	####	###	SNHG5	lncRNA	small nucleo	6	85650491	85678932
ENSG0000015394	-0.804229658	####	###	MSI2	protein_coding	musashi RN	17	57255851	57684689
ENSG0000011450	0.998415002	####	###	NCBP2	protein_coding	nuclear cap	3	196935402	196942594
ENSG0000016430	-2.00904176	####	###	ERAP1	protein_coding	endoplasmi	5	96760810	96808100
ENSG0000013430	0.699577191	####	###	YWHAQ	protein_coding	tyrosine 3-r	2	9583967	9630997
ENSG0000013074	-0.799810432	####	###	EIF2S3	protein_coding	eukaryotic t	X	24054946	24078810
ENSG0000019858	-0.818949333	####	###	LRBA	protein_coding	LPS respons	4	150264435	151015727
ENSG0000013681	-1.015375012	####	###	ECPAS	protein_coding	Ecm29 prote	9	111360692	111484745
ENSG0000017120	-1.457805591	####	###	TRIM8	protein_coding	tripartite mo	10	102644479	102658318
ENSG0000016353	1.141022609	####	###	SGO2	protein_coding	shugoshin 2	2	200510008	200584096
ENSG0000016992	1.18776373	####	###	KLF13	protein_coding	Kruppel like	15	31326835	31435665
ENSG0000018777	-1.633632032	####	###	LIN28B	protein_coding	lin-28 homo	6	104936616	105083332
ENSG0000014664	1.161087208	####	###	EGFR	protein_coding	epidermal g	7	55019017	55211628
ENSG0000027664	-3.418857546	####	###	DACH1	protein_coding	dachshund	13	71437966	71867204
ENSG0000012476	0.783473742	####	###	GLO1	protein_coding	glyoxalase I	6	38675925	38703145
ENSG0000010583	-1.259537743	####	###	NAMPT	protein_coding	nicotinamid	7	106248298	106286326
ENSG0000018309	-3.534578747	####	###	BEGAIN	protein_coding	brain enrich	14	100537147	100587413
ENSG0000018466	1.374824876	####	###	CDCA2	protein_coding	cell division	8	25459199	25507911
ENSG0000018227	-2.3874196	####	###	B4GALNT4	protein_coding	beta-1,4-N	11	369499	382117
ENSG0000012597	-0.739111286	####	###	EIF2S2	protein_coding	eukaryotic t	20	34088309	34112243
ENSG0000010975	-1.056707478	####	###	RAPGEF2	protein_coding	Rap guanine	4	159103013	159360174
ENSG0000015258	-6.565347482	####	###	SPARCL1	protein_coding	SPARC like 1	4	87473335	87531061
ENSG0000011648	0.908739452	####	###	CAPZA1	protein_coding	capping act	1	112619805	112671616
ENSG0000011215	-0.823175849	####	###	MDN1	protein_coding	midasin AA	6	89642498	89819794
ENSG0000019893	0.432530521	####	###	MT-CO3	protein_coding	mitochondr	MT	9207	9990
ENSG0000019866	0.991516737	####	###	C6orf89	protein_coding	chromosom	6	36871870	36928964
ENSG0000014110	-1.278965677	####	###	NOB1	protein_coding	NIN1 (RPN1	16	69741871	69754926
ENSG0000019814	-0.856848781	####	###	ZNF770	protein_coding	zinc finger p	15	34978341	34988287
ENSG0000016254	-3.151415458	####	###	CAMK2N1	protein_coding	calcium/calr	1	20482391	20486210
ENSG0000014294	-1.911221431	####	###	PTPRF	protein_coding	protein tyro	1	43525187	43623666
ENSG0000017467	-2.685448852	####	###	BRSK2	protein_coding	BR serine/th	11	1389899	1462689
ENSG0000019774	1.959745595	####	###	S100A10	protein_coding	S100 calciur	1	151982915	151993859
ENSG0000018057	1.542173318	####	###	H2AC6	protein_coding	H2A cluster	6	26124145	26139116
ENSG0000019620	-0.584306887	####	###	EEF1A1P5	processed_pse	eukaryotic t	9	133019486	133020874
ENSG0000014926	1.992096723	####	###	CAPN5	protein_coding	calpain 5 [S	11	77066961	77126155
ENSG0000010981	-1.176736208	####	###	UGDH	protein_coding	UDP-glucos	4	39498755	39528311
ENSG0000027574	1.189690712	####	###	KLF13	protein_coding	Kruppel like	CHR_HSCHR	31478417	31529455

ENSG0000016620	0.902374045	####	###	GABRB3	protein_coding	gamma-am	15	26543546	26939539
ENSG0000014573	-0.927318599	####	###	PAM	protein_coding	peptidylglyc	5	102753981	103031105
ENSG0000017443	-0.638610709	####	###	ATP2A2	protein_coding	ATPase sarc	12	110280756	110351093
ENSG0000027583	-2.137289253	####	###	ARHGAP23	protein_coding	Rho GTPase	17	38419280	38512385
ENSG0000008289	0.606258607	####	###	XPO1	protein_coding	exportin 1 [S	2	61477849	61538626
ENSG0000006948	-1.37666273	####	###	GAL	protein_coding	galanin and	11	68683779	68691175
ENSG0000017413	-1.266783626	####	###	RGMB	protein_coding	repulsive gu	5	98768650	98798643
ENSG0000015325	-1.658379597	####	###	SCN3A	protein_coding	sodium volt	2	165087526	165204050
ENSG0000016001	1.051739939	####	###	CALM3	protein_coding	calmodulin	19	46601074	46610782
ENSG0000019785	1.348864493	####	###	GPAA1	protein_coding	glycosylpho	8	144082590	144086216
ENSG0000016343	1.067969556	####	###	FSTL1	protein_coding	follistatin lik	3	120392293	120450993
ENSG0000019871	0.442522507	####	###	MT-CO2	protein_coding	mitochondr MT		7586	8269
ENSG0000018225	0.904728961	####	###	SYNM	protein_coding	synemin [So	15	99098217	99135593
ENSG0000005507	0.77251168	####	###	SZRD1	protein_coding	SUZ RNA bi	1	16352575	16398145
ENSG0000011555	-2.412111738	####	###	PLCD4	protein_coding	phospholipa	2	218607855	218637184
ENSG0000006927	0.56409111	####	###	NUCKS1	protein_coding	nuclear case	1	205712822	205750182
ENSG0000019881	-1.630907229	####	###	ZNF358	protein_coding	zinc finger p	19	7515292	7521025
ENSG0000013268	-1.090653251	####	###	NES	protein_coding	nestin [Sour	1	156668763	156677407
ENSG0000011972	-1.013415809	####	###	RHOQ	protein_coding	ras homolog	2	46541806	46584688
ENSG0000012275	1.375482012	####	###	CNTFR	protein_coding	ciliary neuro	9	34551432	34590140
ENSG0000003169	-0.668298844	####	###	SARS1	protein_coding	seryl-tRNA	1	109213918	109238182
ENSG0000022450	-1.542915561	####	###	HSPA1B	protein_coding	heat shock p CHR_HSCHR		31815107	31817630
ENSG0000011931	1.094080711	####	###	PTBP3	protein_coding	polypyrimid	9	112217716	112333664
ENSG0000027088	-2.12753875	####	###	RASL10B	protein_coding	RAS like fan	17	35731639	35743521
ENSG0000017716	1.176481075	####	###	ULK1	protein_coding	unc-51 like	12	131894622	131923150
ENSG0000011683	1.413536517	####	###	TTF2	protein_coding	transcription	1	117060326	117107453
ENSG0000013672	-1.151420101	####	###	HS6ST1	protein_coding	heparan sul	2	128236716	128318868
ENSG0000000116	0.919450948	####	###	NFYA	protein_coding	nuclear tran	6	41072945	41099976
ENSG0000012397	1.028940761	####	###	CKS2	protein_coding	CDC28 prot	9	89311195	89316703
ENSG0000017130	-1.554937449	####	###	KCNK3	protein_coding	potassium t	2	26692690	26733420
ENSG0000022345	1.328301072	####	###	TCAF1P1	unprocessed_p	TRPM8 chan	7	143598039	143604839
ENSG0000011687	-1.872351023	####	###	WARS2	protein_coding	tryptophany	1	119031216	119140654
ENSG0000011435	1.402430885	####	###	GNAI2	protein_coding	G protein su	3	50226292	50259362
ENSG0000007207	-1.037258113	####	###	ADGRL1	protein_coding	adhesion G	19	14147743	14206187
ENSG0000004314	-2.399011761	####	###	JADE2	protein_coding	jade family	5	134524312	134583230
ENSG0000014929	0.69994906	####	###	NCAM1	protein_coding	neural cell a	11	112961247	113278436
ENSG0000001223	0.984012022	####	###	EXTL3	protein_coding	exostosin lik	8	28600469	28755599
ENSG0000013802	-2.685281335	####	###	CGREF1	protein_coding	cell growth	2	27098889	27119128
ENSG0000016960	1.182069109	####	###	CKAP2L	protein_coding	cytoskeletor	2	112736349	112764664
ENSG0000000941	-0.891778063	####	###	REV3L	protein_coding	REV3 like, D	6	111299028	111483715
ENSG0000018483	-2.633804196	####	###	PRR16	protein_coding	proline rich	5	120464300	120687332
ENSG0000018297	-0.996182424	####	###	MTA1	protein_coding	metastasis a	14	105419820	105470729
ENSG0000016644	-0.54172131	####	###	RPL27A	protein_coding	ribosomal p	11	8682788	8714759
ENSG0000015281	-2.455767998	####	###	UTRN	protein_coding	utrophin [So	6	144285701	144853034
ENSG0000014992	-2.049832865	####	###	TLCD3B	protein_coding	TLC domain	16	30024427	30052978
ENSG0000013063	1.792074718	####	###	COL5A1	protein_coding	collagen typ	9	134641803	134844843
ENSG0000012129	-2.303152601	####	###	TSHZ3	protein_coding	teashirt zinc	19	31149979	31349436
ENSG0000012702	-0.566577455	####	###	CANX	protein_coding	calnexin [So	5	179678628	179731641
ENSG0000017149	-1.171903288	####	###	LRRC8D	protein_coding	leucine rich	1	89821014	89936611
ENSG0000016332	-1.841742926	####	###	GPR155	protein_coding	G protein-c	2	174431571	174487094
ENSG0000016688	1.05779365	####	###	VPS39	protein_coding	VPS39 subu	15	42158701	42208307
ENSG0000017849	-1.569747146	####	###	DTX3	protein_coding	deltex E3 ub	12	57604622	57609804
ENSG0000000831	1.782551492	####	###	AASS	protein_coding	aminoadipa	7	122073549	122144255
ENSG0000016729	0.949837226	####	###	TBC1D16	protein_coding	TBC1 doma	17	79932343	80035872
ENSG0000014357	-1.106566413	####	###	HAX1	protein_coding	HCLS1 asso	1	154272589	154275875
ENSG0000018246	-1.849070889	####	###	TSHZ2	protein_coding	teashirt zinc	20	52972358	53495330
ENSG0000007231	-2.262406318	####	###	SREBF1	protein_coding	sterol regula	17	17810399	17837011
ENSG0000013786	-2.284499063	####	###	STRA6	protein_coding	signaling re	15	74179466	74212267

ENSG0000016497	1.316619754	####	###	FAM219A	protein_codinç	family with s	9	34398184	34458570
ENSG0000015816	1.917677465	####	###	TMSB15A	protein_codinç	thymosin be	X	102513682	102516739
ENSG0000013794	0.97493571	####	###	FNBP1L	protein_codinç	formin bind	1	93448131	93554661
ENSG0000021402	-1.072788682	####	###	REPIN1	protein_codinç	replication i	7	150368189	150374044
ENSG0000009936	-1.553852845	####	###	FBXL19	protein_codinç	F-box and l	16	30923055	30948783
ENSG0000003312	-2.525870288	####	###	LRRC7	protein_codinç	leucine rich	1	69567922	70151945
ENSG0000016338	-1.284696645	####	###	POGLUT1	protein_codinç	protein O-g	3	119468963	119494708
ENSG0000011759	-1.962253832	####	###	PLPPR5	protein_codinç	phospholipi	1	98890245	99005032
ENSG0000018141	-2.664613149	####	###	DDN	protein_codinç	dendrin [So	12	48995149	48999375
ENSG0000015240	-0.969077733	####	###	JMY	protein_codinç	junction me	5	79236131	79327211
ENSG0000023301	-1.432791165	####	###	SNHG7	lncRNA	small nucleo	9	136721366	136728184
ENSG0000011276	-3.54791285	####	###	LAMA4	protein_codinç	laminin sub	6	112107931	112254939
ENSG0000013615	-1.403249137	####	###	SPRY2	protein_codinç	sprouty RTK	13	80335976	80341126
ENSG0000019883	-0.988314945	####	###	UBE2J1	protein_codinç	ubiquitin co	6	89326625	89352722
ENSG0000015250	2.322841729	####	###	TRIM36	protein_codinç	tripartite mo	5	115124762	115180546
ENSG0000012314	-1.180056532	####	###	PKN1	protein_codinç	protein kina	19	14433053	14471867
ENSG0000011825	-1.280161244	####	###	NRP2	protein_codinç	neuropilin 2	2	205681990	205798133
ENSG0000018897	0.952197196	####	###	NOC2L	protein_codinç	NOC2 like n	1	944203	959309
ENSG0000015847	0.837169245	####	###	B4GALT5	protein_codinç	beta-1,4-ga	20	49632945	49713878
ENSG0000013914	-0.915436495	####	###	SINHCAF	protein_codinç	SIN3-HDAC	12	31280584	31327058
ENSG0000027378	-2.092024409	####	###	ARHGAP23	protein_codinç	Rho GTPase	CHR_HSCHR	38229196	38313162
ENSG0000015647	2.714391353	####	###	PPP2R2B	protein_codinç	protein pho	5	146581146	147084784
ENSG0000025408	-1.034313801	####	###	LYN	protein_codinç	LYN proto-o	8	55879835	56014169
ENSG0000015721	0.895222306	####	###	SSBP3	protein_codinç	single stranc	1	54225432	54413479
ENSG0000016799	-3.011445314	####	###	RAB3IL1	protein_codinç	RAB3A inter	11	61897301	61920269
ENSG0000022494	-2.03093089	####	###	PRRT4	protein_codinç	proline rich	7	128350325	128361685
ENSG0000023447	-1.706250866	####	###	HSPA1A	protein_codinç	heat shock p	CHR_HSCHR	31797650	31800132
ENSG0000000646	-1.038846373	####	###	ETV1	protein_codinç	ETS variant	7	13891229	13991425
ENSG0000016450	-1.239786939	####	###	STXBP5	protein_codinç	syntaxin bin	6	147204425	147390476
ENSG0000007655	-0.991056734	####	###	TPD52	protein_codinç	tumor prote	8	80034745	80231232
ENSG0000013964	1.051476628	####	###	ANKRD52	protein_codinç	ankyrin repe	12	56237807	56258384
ENSG0000020438	-1.732425856	####	###	HSPA1A	protein_codinç	heat shock p	6	31815543	31817946
ENSG0000027819	-1.446535481	####	###	CARS1	protein_codinç	cysteinyl-tR	CHR_HSCHR	2999797	3056458
ENSG0000014044	-0.807187107	####	###	IGF1R	protein_codinç	insulin like g	15	98648539	98964530
ENSG0000015650	-0.509277996	####	###	EEF1A1	protein_codinç	eukaryotic t	6	73515750	73523797
ENSG0000013807	0.622700706	####	###	ACTR2	protein_codinç	actin relatec	2	65227788	65271253
ENSG0000008991	0.92167209	####	###	GPATCH2L	protein_codinç	G-patch do	14	76151916	76254342
ENSG0000017257	-1.549378093	####	###	PDE3A	protein_codinç	phosphodie	12	20368537	20688583
ENSG0000012732	-2.535563954	####	###	PTPRB	protein_codinç	protein tyro	12	70515866	70637440
ENSG0000001823	-0.7568183	####	###	CNTN1	protein_codinç	contactin 1	12	40692439	41072415
ENSG0000016848	-0.781743062	####	###	ATXN2L	protein_codinç	ataxin 2 like	16	28823035	28837237
ENSG0000017770	1.581630482	####	###	POLR2L	protein_codinç	RNA polyme	11	837356	842529
ENSG0000000314	-1.030934249	####	###	ICA1	protein_codinç	islet cell aut	7	8113184	8262687
ENSG0000013264	0.968549227	####	###	PCNA	protein_codinç	proliferating	20	5114953	5126626
ENSG0000023772	-1.671526453	####	###	HSPA1A	protein_codinç	heat shock p	CHR_HSCHR	31802834	31805316
ENSG0000016549	1.346474502	####	###	DDIAS	protein_codinç	DNA damag	11	82899975	82958277
ENSG0000015808	-1.639703975	####	###	GALNT14	protein_codinç	polypeptide	2	30910467	31155202
ENSG0000014404	1.495187131	####	###	DQX1	protein_codinç	DEAQ-box	2	74518131	74526281
ENSG0000014608	-0.913118618	####	###	RNF44	protein_codinç	ring finger p	5	176526712	176538025
ENSG0000016935	-1.07921814	####	###	SLC33A1	protein_codinç	solute carrie	3	155821024	155854456
ENSG0000012478	-1.436448486	####	###	ATXN1	protein_codinç	ataxin 1 [So	6	16299112	16761491
ENSG0000017273	1.789276029	####	###	LRRC20	protein_codinç	leucine rich	10	70298970	70382650
ENSG0000004093	1.12128233	####	###	INPP4A	protein_codinç	inositol poly	2	98444854	98594392
ENSG0000011928	-0.729658433	####	###	TRIM67	protein_codinç	tripartite mo	1	231162112	231221556
ENSG0000019864	-1.064057679	####	###	NCOA6	protein_codinç	nuclear rece	20	34689097	34825651
ENSG0000013626	-2.045935988	####	###	DGKB	protein_codinç	diacylglycer	7	14145049	14974777
ENSG0000018081	-0.772771029	####	###	PPA1	protein_codinç	inorganic py	10	70202835	70233911
ENSG0000012083	-1.728346588	####	###	SOCS2	protein_codinç	suppressor c	12	93569814	93583487

ENSG0000024440	-0.995163949	####	###	ETV5	protein_coding	ETS variant	3	186046314	186110318
ENSG0000009159	2.500493288	####	###	NLRP1	protein_coding	NLR family	17	5499427	5619424
ENSG0000014473	-1.682311453	####	###	IL17RD	protein_coding	interleukin 1	3	57089982	57170306
ENSG0000015979	1.276131771	####	###	PSKH1	protein_coding	protein serir	16	67893254	67929676
ENSG0000011314	0.963571408	####	###	SPARC	protein_coding	secreted pro	5	151661096	151686975
ENSG0000011339	-2.563726801	####	###	SLC27A6	protein_coding	solute carrie	5	128538013	129033642
ENSG0000004206	-5.488987064	####	###	RIPOR3	protein_coding	RIPOR famil	20	50586108	50691542
ENSG0000023155	-1.553831854	####	###	HSPA1B	protein_coding	heat shock	CHR_HSCHR	31809921	31812441
ENSG0000017319	-2.614043148	####	###	PARP14	protein_coding	poly(ADP-ri	3	122680839	122730840
ENSG0000016363	-1.161981042	####	###	ADAMTS9	protein_coding	ADAM meta	3	64515654	64688000
ENSG0000015096	-0.753572196	####	###	SEC24D	protein_coding	SEC24 hom	4	118722823	118838683
ENSG0000020438	-1.60563497	####	###	HSPA1B	protein_coding	heat shock	6	31827738	31830254
ENSG0000017322	-2.014020422	####	###	GLRX	protein_coding	glutaredoxir	5	95751319	95822726
ENSG0000014253	-0.626996729	####	###	RPS11	protein_coding	ribosomal p	19	49496365	49499708
ENSG0000020593	0.723218804	####	###	RNPS1	protein_coding	RNA binding	16	2253116	2268397
ENSG0000012535	-0.721479121	####	###	SEPTIN6	protein_coding	septin 6 [So	X	119615724	119693370
ENSG0000010773	0.645504908	####	###	UNC5B	protein_coding	unc-5 netrir	10	71212570	71302864
ENSG0000001482	0.77376974	####	###	SLC30A9	protein_coding	solute carrie	4	41990502	42090461
ENSG0000013497	-0.854086317	####	###	TMED7	protein_coding	transmembr	5	115613210	115632992
ENSG0000015372	-1.380152189	####	###	CNKSR3	protein_coding	CNKSR fami	6	154387515	154510685
ENSG0000016432	-0.957784268	####	###	CFAP97	protein_coding	cilia and flag	4	185159665	185209504
ENSG0000017328	-2.455591515	####	###	PPP1R3B	protein_coding	protein pho	8	9136255	9151574
ENSG0000017092	-7.540541256	####	###	PKHD1	protein_coding	PKHD1 ciliar	6	51615299	52087613
ENSG0000014489	-0.837946394	####	###	EIF2A	protein_coding	eukaryotic t	3	150546678	150586016
ENSG0000017364	-3.754658673	####	###	HSPB7	protein_coding	heat shock	1	16014028	16019594
ENSG0000011359	-0.995435614	####	###	LIFR	protein_coding	LIF receptor	5	38474668	38608354
ENSG0000012308	1.014214686	####	###	CDKN2C	protein_coding	cyclin deper	1	50960745	50974634
ENSG0000012201	-1.603852148	####	###	SV2C	protein_coding	synaptic ves	5	76083383	76353939
ENSG0000014812	-1.2089713	####	###	AOPEP	protein_coding	aminopeptic	9	94726701	95087218
ENSG0000021286	-1.593688949	####	###	HSPA1B	protein_coding	heat shock	CHR_HSCHR	31817974	31820498
ENSG0000012553	1.779350405	####	###	PPDPF	protein_coding	pancreatic p	20	63520765	63522206
ENSG0000019645	-1.392102836	####	###	ZNF605	protein_coding	zinc finger p	12	132918306	132956306
ENSG0000000324	-2.518702943	####	###	DBNDD1	protein_coding	dysbindin d	16	90004871	90020128
ENSG0000010065	0.704976563	####	###	SRSF5	protein_coding	serine and a	14	69726900	69772005
ENSG0000017015	-0.657135902	####	###	RNF150	protein_coding	ring finger p	4	140859807	141212877
ENSG0000016240	-1.070041749	####	###	USP24	protein_coding	ubiquitin sp	1	55066359	55215364
ENSG0000010253	-1.190857322	####	###	FNDC3A	protein_coding	fibronectin t	13	48975912	49209779
ENSG0000016849	-0.956632675	####	###	POLR3D	protein_coding	RNA polyme	8	22245133	22254601
ENSG0000013653	-2.491251314	####	###	SCN2A	protein_coding	sodium volt	2	165194993	165392310
ENSG0000023594	-1.656974134	####	###	HSPA1A	protein_coding	heat shock	CHR_HSCHR	31882493	31884975
ENSG0000012744	1.015084951	####	###	PIN1	protein_coding	peptidylprol	19	9835257	9849689
ENSG0000013601	-0.96308358	####	###	ALDH1L2	protein_coding	aldehyde de	12	105019784	105107643
ENSG0000012965	-0.848989257	####	###	SEC14L1	protein_coding	SEC14 like li	17	77088749	77217101
ENSG0000016029	1.098754397	####	###	VAV2	protein_coding	vav guanine	9	133761894	133992604
ENSG0000010472	0.90998604	####	###	TUSC3	protein_coding	tumor supp	8	15417215	15766649
ENSG0000016696	0.844915627	####	###	MAP1A	protein_coding	microtubule	15	43510958	43531620
ENSG0000018420	-1.385283237	####	###	TSPYL2	protein_coding	TSPY like 2	X	53082367	53088540
ENSG0000017756	-0.835724423	####	###	TBL1XR1	protein_coding	TBL1X recep	3	177019340	177228000
ENSG0000013030	0.984279691	####	###	COLGALT1	protein_coding	collagen be	19	17555649	17583162
ENSG0000017542	2.045889927	####	###	PCSK1	protein_coding	proprotein c	5	96390333	96434143
ENSG0000023930	1.126927004	####	###	RBM14	protein_coding	RNA binding	11	66616626	66629934
ENSG0000000340	-1.607253441	####	###	CFLAR	protein_coding	CASP8 and	2	201116154	201176687
ENSG0000011995	-1.208322832	####	###	MXI1	protein_coding	MAX interac	10	110207605	110287365
ENSG0000011746	0.843433176	####	###	PIK3R3	protein_coding	phosphoino	1	46040140	46133036
ENSG0000017207	-1.321090762	####	###	EIF2AK3	protein_coding	eukaryotic t	2	88556741	88627464
ENSG0000017513	0.779901788	####	###	MARCKSL1	protein_coding	MARCKS like	1	32333839	32336233
ENSG0000003303	-1.267712895	####	###	ZCCHC8	protein_coding	zinc finger C	12	122471599	122501073
ENSG0000014392	-0.594794665	####	###	EML4	protein_coding	EMAP like 4	2	42169353	42332548

ENSG0000018458	-3.459487687	####	###	PDE4B	protein_coding	phosphodie	1	65792514	66374579
ENSG0000014524	-2.806869393	####	###	CORIN	protein_coding	corin, serine	4	47593999	47838106
ENSG0000012508	-3.188386885	####	###	SH3TC1	protein_coding	SH3 domain	4	8182072	8241803
ENSG0000017448	1.660446012	####	###	BBS1	protein_coding	Bardet-Biec	11	66510606	66533613
ENSG0000010246	-1.840978197	####	###	FGF14	protein_coding	fibroblast gr	13	101710804	102402457
ENSG0000005511	-0.8616697	####	###	KCNH2	protein_coding	potassium v	7	150944961	150978321
ENSG0000026056	1.695794812	####	###	ERVK13-1	lncRNA	endogenous	16	2660348	2682379
ENSG0000016746	-0.663680686	####	###	TPM4	protein_coding	tropomyosin	19	16067021	16103002
ENSG0000010569	-1.526464327	####	###	USF2	protein_coding	upstream tr	19	35268962	35279821
ENSG0000016765	0.808061409	####	###	ATCAY	protein_coding	ATCAY kine	19	3879864	3928082
ENSG0000012678	0.96761021	####	###	DLGAP5	protein_coding	DLG associa	14	55148112	55191608
ENSG0000017357	-0.759917008	####	###	CHD2	protein_coding	chromodom	15	92900189	93027996
ENSG0000023280	-1.621252788	####	###	HSPA1B	protein_coding	heat shock p	CHR_HSCHR	31894767	31897281
ENSG0000005016	-2.30088639	####	###	DKK3	protein_coding	dickkopf WI	11	11956207	12009769
ENSG0000022685	-1.989579503	####	###	NA	NA	NA	NA	NA	NA
ENSG0000015464	0.97587403	####	###	C21orf91	protein_coding	chromosom	21	17788974	17819386
ENSG0000014063	0.78623641	####	###	GLYR1	protein_coding	glyoxylate re	16	4803203	4847288
ENSG0000016563	-3.105385599	####	###	VSTM4	protein_coding	V-set and tr	10	49014236	49115522
ENSG0000010766	-1.176882885	####	###	ATE1	protein_coding	arginyltrans	10	121740421	121928801
ENSG0000015150	0.799240777	####	###	VPS26B	protein_coding	VPS26, retro	11	134224671	134247788
ENSG0000008420	0.583369964	####	###	GSTP1	protein_coding	glutathione	11	67583742	67586656
ENSG0000005867	-0.570342491	####	###	ZC3H11A	protein_coding	zinc finger C	1	203795654	203854999
ENSG0000004932	-2.003468108	####	###	LTBP1	protein_coding	latent transf	2	32946953	33399509
ENSG0000018396	1.776612852	####	###	SMTN	protein_coding	smoothelin	22	31064105	31104757
ENSG0000011851	-1.668763942	####	###	SGK1	protein_coding	serum/gluco	6	134169246	134318112
ENSG0000016405	-6.296289821	####	###	SPRY1	protein_coding	sprouty RTK	4	123396795	123403760
ENSG0000015077	1.145117348	####	###	TIMM8B	protein_coding	translocase	11	112084800	112086798
ENSG0000010300	1.75231059	####	###	USB1	protein_coding	U6 snRNA b	16	57999546	58021618
ENSG0000014175	-0.82199731	####	###	FKBP10	protein_coding	FKBP prolyl	17	41812680	41823213
ENSG0000013098	0.629828828	####	###	UBA1	protein_coding	ubiquitin like	X	47190861	47215128
ENSG0000017690	1.017787643	####	###	PNMA1	protein_coding	PNMA famil	14	73711783	73714384
ENSG0000010002	2.810437427	####	###	YPEL1	protein_coding	yippee like 1	22	21697536	21735794
ENSG0000010490	0.677478104	####	###	OAZ1	protein_coding	ornithine de	19	2269509	2273490
ENSG0000013777	-0.77769999	####	###	SLTM	protein_coding	SAFB like tra	15	58879050	58933679
ENSG0000018618	-1.173716927	####	###	POLR1D	protein_coding	RNA polyme	13	27620742	27744237
ENSG0000004834	-1.578907555	####	###	CC2D2A	protein_coding	coiled-coil a	4	15469865	15601552
ENSG0000016441	-1.04101228	####	###	GRIK2	protein_coding	glutamate io	6	101181257	102070083
ENSG0000010678	1.103456059	####	###	MEGF9	protein_coding	multiple EG	9	120600811	120714470
ENSG0000024416	-2.363032161	####	###	P2RY11	protein_coding	purinergic re	19	10111693	10115372
ENSG0000013591	-0.67928386	####	###	ITM2C	protein_coding	integral mer	2	230864639	230879248
ENSG0000006902	-1.517043446	####	###	MAST4	protein_coding	microtubule	5	66596361	67169595
ENSG0000014725	-0.959173857	####	###	DOCK11	protein_coding	dedicator of	X	118495898	118686163
ENSG0000016763	-0.733962608	####	###	ZNF146	protein_coding	zinc finger p	19	36214602	36238774
ENSG0000008102	0.965004975	####	###	MAGI3	protein_coding	membrane a	1	113390515	113685923
ENSG0000013248	0.901150405	####	###	ZRANB2	protein_coding	zinc finger R	1	71063291	71081289
ENSG0000012237	-0.801641807	####	###	PRXL2A	protein_coding	peroxiredox	10	80407829	80437115
ENSG0000016004	0.990068287	####	###	DFFA	protein_coding	DNA fragme	1	10456522	10472529
ENSG0000018915	0.806747352	####	###	JPT1	protein_coding	Jupiter micr	17	75135248	75168281
ENSG0000013984	-1.031523197	####	###	CUL4A	protein_coding	cullin 4A [So	13	113208193	113267108
ENSG0000016237	-1.206177261	####	###	COA7	protein_coding	cytochrome	1	52684449	52698347
ENSG0000024967	2.101984766	####	###	NOP14-AS	lncRNA	NOP14 anti	4	2934882	2961738
ENSG0000021833	-0.808709579	####	###	TENM3	protein_coding	teneurin tra	4	182143987	182803024
ENSG0000010349	-0.614572357	####	###	MAZ	protein_coding	MYC associa	16	29806106	29811164
ENSG0000001152	1.079094864	####	###	CEP68	protein_coding	centrosoma	2	65056366	65087004
ENSG0000016560	-1.695891188	####	###	DRGX	protein_coding	dorsal root	10	49364181	49396016
ENSG0000021436	1.257696045	####	###	HAUS3	protein_coding	HAUS augm	4	2078998	2242276
ENSG0000014262	-4.138513749	####	###	EPHA2	protein_coding	EPH recepto	1	16124337	16156069
ENSG0000019654	-3.736406794	####	###	SPTSSB	protein_coding	serine palmi	3	161344792	161372880

ENSG0000016618	0.681369484	####	###	API5	protein_coding	apoptosis ir	11	43311963	43344529
ENSG0000016270	0.741547437	####	###	ARPC5	protein_coding	actin relatec	1	183620846	183635783
ENSG0000017370	1.568092039	####	###	SUSD5	protein_coding	sushi domai	3	33150043	33218810
ENSG0000015164	1.435446131	####	###	DPYSL4	protein_coding	dihydropyri	10	132186948	132205759
ENSG0000010520	-0.908034481	####	###	FBL	protein_coding	fibrillarin [Sc	19	39834458	39846379
ENSG0000013424	0.862066851	####	###	SORT1	protein_coding	sortilin 1 [Sc	1	109309568	109397918
ENSG0000002655	-1.402060366	####	###	KCNG1	protein_coding	potassium v	20	51003656	51023107
ENSG0000019665	1.296499542	####	###	TRAPPC4	protein_coding	trafficking p	11	119018763	119025454
ENSG0000008264	-0.61063884	####	###	NFE2L1	protein_coding	nuclear fact	17	48048329	48061545
ENSG0000013489	1.390509066	####	###	ERCC5	protein_coding	ERCC excisi	13	102845831	102875995
ENSG0000014925	-1.449533167	####	###	TENM4	protein_coding	teneurin tra	11	78652829	79441030
ENSG0000015798	-0.904845556	####	###	AGAP1	protein_coding	ArfGAP with	2	235494043	236131800
ENSG0000016717	1.857165744	####	###	ISLR2	protein_coding	immunoglol	15	74100311	74138540
ENSG0000008671	-2.972779643	####	###	PPEF1	protein_coding	protein pho X		18675909	18827921
ENSG0000007826	-1.663695393	####	###	SYNJ2	protein_coding	synaptojanii	6	157981863	158099176
ENSG0000014742	-0.9591948	####	###	HMBOX1	protein_coding	homeobox (	8	28890395	29064764
ENSG0000013533	-3.944843246	####	###	EPHA7	protein_coding	EPH recepto	6	93240020	93419559
ENSG0000012880	-4.077729728	####	###	ARHGAP22	protein_coding	Rho GTPase	10	48446036	48656265
ENSG0000014486	-1.05370791	####	###	TMEM108	protein_coding	transmembı	3	133038391	133397792
ENSG0000008968	0.863687674	####	###	BIRC5	protein_coding	baculoviral I	17	78214186	78225636
ENSG0000010522	-0.921163766	####	###	GPI	protein_coding	glucose-6-ı	19	34359480	34402413
ENSG0000010564	-0.498850637	####	###	RPL18A	protein_coding	ribosomal p	19	17859910	17864153
ENSG0000012348	1.090409437	####	###	HJURP	protein_coding	Holliday jun	2	233833416	233854566
ENSG0000014921	1.163941653	####	###	ENDOD1	protein_coding	endonuclea	11	95089846	95132645
ENSG0000012457	-2.243412804	####	###	SERPINB6	protein_coding	serpin famil	6	2948159	2972165
ENSG0000012310	-1.287318845	####	###	ITPR2	protein_coding	inositol 1,4,!	12	26335352	26833194
ENSG0000011361	-1.113863242	####	###	SEC24A	protein_coding	SEC24 hom	5	134648785	134727909
ENSG0000015052	-1.612607905	####	###	MIA2	protein_coding	MIA SH3 dc	14	39230231	39388513
ENSG0000016608	0.734750318	####	###	JAM3	protein_coding	junctional a	11	134069071	134152001
ENSG0000024315	0.88841626	####	###	MICAL3	protein_coding	microtubule	22	17787649	18024561
ENSG0000014334	-1.128910099	####	###	FAM163A	protein_coding	family with	1	179743163	179816198
ENSG0000014337	-0.988802555	####	###	SETDB1	protein_coding	SET domain	1	150926263	150964744
ENSG0000011510	0.95461934	####	###	EPB41L5	protein_coding	erythrocyte	2	120013077	120179119
ENSG0000013163	-2.70871147	####	###	TMEM204	protein_coding	transmembı	16	1528688	1555580
ENSG0000019861	0.814667339	####	###	COPS8	protein_coding	COP9 signa	2	237085882	237100474
ENSG0000012478	-1.043625132	####	###	RIOK1	protein_coding	RIO kinase 1	6	7389793	7418037
ENSG0000010052	1.38595169	####	###	CDKN3	protein_coding	cyclin deper	14	54396849	54420218
ENSG0000010081	-0.714660468	####	###	YY1	protein_coding	YY1 transcri	14	100238298	100282788
ENSG0000013015	-1.292130977	####	###	ECSIT	protein_coding	ECSIT signal	19	11505929	11529172
ENSG0000007811	-2.967292824	####	###	NEBL	protein_coding	nebulette [S	10	20779973	21293011
ENSG0000007880	1.268243721	####	###	TP53INP2	protein_coding	tumor prote	20	34704339	34713439
ENSG0000013964	0.68494731	####	###	TMBIM6	protein_coding	transmembı	12	49707725	49764934
ENSG0000010986	-1.310211987	####	###	CTSC	protein_coding	cathepsin C	11	88293592	88337761
ENSG0000011066	-1.310158054	####	###	SLC35F2	protein_coding	solute carrie	11	107790991	107928293
ENSG0000016802	-0.532204688	####	###	RPSA	protein_coding	ribosomal p	3	39406716	39412542
ENSG0000014538	0.846466264	####	###	CCNA2	protein_coding	cyclin A2 [Sc	4	121816444	121823883
ENSG0000016488	1.027649489	####	###	INTS1	protein_coding	integrator c	7	1470277	1504389
ENSG0000024838	1.697268731	####	###	PCDHAC1	protein_coding	protocadhe	5	140926299	141012347
ENSG0000016474	-1.020235918	####	###	DLC1	protein_coding	DLC1 Rho G	8	13083361	13604610
ENSG0000018211	-1.330293234	####	###	FAM89A	protein_coding	family with	1	231018958	231040254
ENSG0000014764	-0.648349024	####	###	MTDH	protein_coding	metadherin	8	97644184	97730260
ENSG0000011494	-0.617996688	####	###	EEF1B2	protein_coding	eukaryotic t	2	206159585	206162928
ENSG0000015765	-1.091575796	####	###	ZNF618	protein_coding	zinc finger p	9	113876282	114056591
ENSG0000016303	-2.835467204	####	###	VSNL1	protein_coding	visinin like 1	2	17539126	17657018
ENSG0000018425	-4.604263905	####	###	ALDH1A3	protein_coding	aldehyde de	15	100877714	100916626
ENSG0000011588	-1.547318057	####	###	SDC1	protein_coding	syndecan 1	2	20200797	20225433
ENSG0000015321	1.4751237	####	###	TMEM87B	protein_coding	transmembı	2	112055269	112119318
ENSG0000012326	-1.445448727	####	###	ATF1	protein_coding	activating tr	12	50763710	50821162

ENSG0000006966	-1.757652762	####	###	RORA	protein_coding	RAR related	15	60488284	61229302
ENSG0000013444	-0.756652884	####	###	NARS1	protein_coding	asparaginyl-	18	57600656	57622213
ENSG0000017163	-4.294601544	####	###	P2RY6	protein_coding	pyrimidinerg	11	73264505	73298625
ENSG0000014436	-0.764066197	####	###	GULP1	protein_coding	GULP PTB d	2	188291669	188595931
ENSG0000013837	1.545318714	####	###	BARD1	protein_coding	BRCA1 asso	2	214725646	214809683
ENSG0000010411	0.656851051	####	###	SCG3	protein_coding	secretogran	15	51681492	51721026
ENSG0000019639	1.04352302	####	###	PTPN1	protein_coding	protein tyro	20	50510321	50585241
ENSG0000016368	1.143367953	####	###	SMIM14	protein_coding	small integr	4	39546336	39638902
ENSG0000015871	-0.772013444	####	###	TAGLN2	protein_coding	transgelin 2	1	159918107	159925507
ENSG0000014727	-0.583232148	####	###	RBMX	protein_coding	RNA binding	X	136848004	136880764
ENSG0000006764	-2.3665397	####	###	ZFY	protein_coding	zinc finger p	Y	2935281	2982506
ENSG0000013364	-1.134680747	####	###	C12orf29	protein_coding	chromosom	12	88033846	88050160
ENSG0000015313	1.058194268	####	###	SCOC	protein_coding	short coiled	4	140257286	140385726
ENSG0000017369	0.762286401	####	###	PSMD1	protein_coding	proteasome	2	231056864	231172827
ENSG0000013118	-3.596062387	####	###	F12	protein_coding	coagulation	5	177402141	177409564
ENSG0000013158	1.10480872	####	###	ACAP3	protein_coding	ArfGAP with	1	1292390	1309609
ENSG0000013532	-2.155832624	####	###	MRAP2	protein_coding	melanocorti	6	84033772	84090881
ENSG0000010112	-0.671184481	####	###	ADNP	protein_coding	activity dep	20	50888916	50931437
ENSG0000016728	0.714172271	####	###	ATP5MG	protein_coding	ATP synthas	11	118401606	118431496
ENSG0000013810	0.74954485	####	###	ACTR1A	protein_coding	actin relatec	10	102461881	102502712
ENSG0000017179	-0.924205562	####	###	BCL2	protein_coding	BCL2 apopt	18	63123346	63320128
ENSG0000017391	-5.045701641	####	###	C1QTNF1	protein_coding	C1q and TN	17	79022814	79049788
ENSG0000018456	-1.943328844	####	###	SLITRK6	protein_coding	SLIT and NT	13	85792790	85806683
ENSG0000011196	-1.926356479	####	###	SASH1	protein_coding	SAM and SH	6	148272304	148552048
ENSG0000017708	0.847188607	####	###	POLE	protein_coding	DNA polym	12	132623753	132687376
ENSG0000018773	-0.697664773	####	###	TCEA1	protein_coding	transcription	8	53966552	54022456
ENSG0000004764	-2.918924446	####	###	ARHGAP6	protein_coding	Rho GTPase	X	11117651	11665920
ENSG0000013818	1.032901183	####	###	CEP55	protein_coding	centrosoma	10	93496612	93529092
ENSG0000016099	-1.413450759	####	###	ORAI2	protein_coding	ORAI calciu	7	102433106	102456825
ENSG0000015257	-1.06231394	####	###	GRIA4	protein_coding	glutamate io	11	105609994	105982092
ENSG0000008345	-4.563212699	####	###	P2RX5	protein_coding	purinergic r	17	3672199	3696240
ENSG0000018088	-2.590567701	####	###	ZNF792	protein_coding	zinc finger p	19	34956354	34964229
ENSG0000017199	-1.919980214	####	###	SYNPO	protein_coding	synaptopod	5	150601080	150659207
ENSG0000011031	1.34639129	####	###	RNF141	protein_coding	ring finger p	11	10511673	10541230
ENSG0000010044	0.866019302	####	###	FKBP3	protein_coding	FKBP prolyl	14	45115599	45135319
ENSG0000011090	-2.525532721	####	###	TSPAN11	protein_coding	tetraspanin	12	30926428	30996602
ENSG0000003540	0.855087096	####	###	VCL	protein_coding	vinculin [So	10	73995193	74121363
ENSG0000021465	1.105144681	####	###	ZSWIM8	protein_coding	zinc finger S	10	73785606	73801797
ENSG0000026092	-1.733076555	####	###	AL031985.3	lncRNA	novel transc	1	40464319	40466767
ENSG0000019824	-0.487287504	####	###	RPL23A	protein_coding	ribosomal p	17	28719985	28724359
ENSG0000027637	-1.051300798	####	###	SINHCAF	protein_coding	SIN3-HDAC	CHR_HSCHR	31282857	31329331
ENSG0000013311	-0.534138333	####	###	TPT1	protein_coding	tumor prote	13	45333471	45341284
ENSG0000010372	1.320043666	####	###	AP3B2	protein_coding	adaptor rela	15	82659281	82709946
ENSG0000010082	-0.592599302	####	###	APEX1	protein_coding	apurinic/apy	14	20455191	20457772
ENSG0000020410	2.524545372	####	###	MAFB	protein_coding	MAF bZIP tr	20	40685848	40689236
ENSG0000016786	0.868714149	####	###	ATP5PD	protein_coding	ATP synthas	17	75038863	75046985
ENSG0000018631	2.394544116	####	###	NAP1L3	protein_coding	nucleosome	X	93670930	93673578
ENSG0000012422	0.660448501	####	###	STX16	protein_coding	syntaxin 16	20	58651253	58679526
ENSG0000011336	0.774251476	####	###	LMNB1	protein_coding	lamin B1 [So	5	126776623	126837020
ENSG0000015852	-2.661372005	####	###	PPP1R9A	protein_coding	protein pho	7	94907202	95296415
ENSG0000019812	-1.131670539	####	###	LPAR1	protein_coding	lysophospha	9	110873263	111038458
ENSG0000012053	0.81349752	####	###	ENY2	protein_coding	ENY2 transc	8	109334324	109345954
ENSG0000000794	-1.745293171	####	###	MYLIP	protein_coding	myosin regu	6	16129086	16148248
ENSG0000010116	-0.758754083	####	###	PRELID3B	protein_coding	PRELI doma	20	59033145	59042809
ENSG0000015536	1.389740625	####	###	RHOC	protein_coding	ras homolog	1	112701127	112707434
ENSG0000027147	1.960732268	####	###	AC106881.1	lncRNA	novel transc	4	95549129	95552457
ENSG0000010789	-1.032698296	####	###	ACBD5	protein_coding	acyl-CoA bi	10	27195214	27242130
ENSG0000019704	-0.88733256	####	###	ANXA6	protein_coding	annexin A6	5	151100706	151157785

ENSG0000013704	0.988868428	####	###	RANBP6	protein_coding	RAN binding	9	6011025	6015625
ENSG0000010443	-0.458449631	####	###	STMN2	protein_coding	stathmin 2 [	8	79611117	79666158
ENSG0000019613	-1.233684188	####	###	AKR1C3	protein_coding	aldo-keto re	10	5035354	5107686
ENSG0000013541	0.715393257	####	###	GDF11	protein_coding	growth diffe	12	55743122	55757264
ENSG0000017773	-1.092339467	####	###	SOX12	protein_coding	SRY-box tra	20	325595	330224
ENSG0000012430	2.462471813	####	###	CHST8	protein_coding	carbohydrat	19	33621953	33773509
ENSG0000021318	1.103252237	####	###	TRIM59	protein_coding	tripartite mo	3	160432445	160485773
ENSG0000016458	-0.485823584	####	###	RPS14	protein_coding	ribosomal p	5	150442635	150449739
ENSG0000018304	-1.322483924	####	###	CAMK1D	protein_coding	calcium/calr	10	12349547	12835545
ENSG0000014747	1.179487613	####	###	ERLIN2	protein_coding	ER lipid raft	8	37736601	37758422
ENSG0000011191	-1.265085218	####	###	HINT3	protein_coding	histidine tria	6	125956770	125980244
ENSG0000018918	-1.032246724	####	###	ZNF33A	protein_coding	zinc finger p	10	38010650	38065088
ENSG0000024331	0.95947326	####	###	STMP1	protein_coding	short transm	7	135662496	135693418
ENSG0000018884	-0.582589719	####	###	RPL14	protein_coding	ribosomal p	3	40457292	40468587
ENSG0000008898	0.738358125	####	###	DYNLL1	protein_coding	dynein light	12	120469850	120498493
ENSG0000014646	-10.99616177	####	###	VIP	protein_coding	vasoactive i	6	152750797	152759765
ENSG0000012189	-0.591554764	####	###	PDS5A	protein_coding	PDS5 cohes	4	39822863	39977956
ENSG0000009088	0.878061915	####	###	KIF4A	protein_coding	kinesin fami	X	70290104	70420886
ENSG0000015414	2.23700366	####	###	NRGN	protein_coding	neurogranin	11	124739942	124747210
ENSG0000007801	-0.653527837	####	###	MAP2	protein_coding	microtubule	2	209424058	209734118
ENSG0000016793	0.967516949	####	###	FAM234A	protein_coding	family with s	16	234521	272183
ENSG0000016407	-1.651420255	####	###	CAMKV	protein_coding	CaM kinase	3	49857988	49869935
ENSG0000024125	-1.054251467	####	###	CRCP	protein_coding	CGRP recep	7	66114604	66154568
ENSG0000014833	-0.968767395	####	###	CIZ1	protein_coding	CDKN1A int	9	128161251	128204383
ENSG0000016975	-1.726263177	####	###	RAC3	protein_coding	Rac family s	17	82031678	82034204
ENSG0000011132	-0.810407584	####	###	CDK2AP1	protein_coding	cyclin deper	12	123250112	123272334
ENSG0000018718	0.97479796	####	###	TSPYL4	protein_coding	TSPY like 4	6	116249964	116254075
ENSG0000024392	-1.325564552	####	###	MRPS6	protein_coding	mitochondr	21	34073224	34143034
ENSG0000016237	-0.695262991	####	###	ELAVL4	protein_coding	ELAV like RN	1	50024029	50203772
ENSG0000011576	-0.595774643	####	###	BIRC6	protein_coding	baculoviral I	2	32357028	32618899
ENSG0000016101	-0.715326795	####	###	SQSTM1	protein_coding	sequestosor	5	179806398	179838078
ENSG0000024217	-3.646531625	####	###	ARHGDIG	protein_coding	Rho GDP di	16	280450	283010
ENSG0000017198	-0.68443976	####	###	JMJD1C	protein_coding	jumonji don	10	63167221	63521850
ENSG0000018581	-2.781611777	####	###	NAT8L	protein_coding	N-acetyltrar	4	2059327	2069089
ENSG0000013860	1.124914599	####	###	GLCE	protein_coding	glucuronic a	15	69160584	69272217
ENSG0000010238	1.598791718	####	###	CENPI	protein_coding	centromere	X	101098218	101163681
ENSG0000015331	-0.831735702	####	###	CYRIB	protein_coding	CYFIP relate	8	129839593	130017129
ENSG0000016421	-0.663239012	####	###	STARD4	protein_coding	StAR relatec	5	111496033	111512590
ENSG0000011640	-0.654213701	####	###	EDEM3	protein_coding	ER degradat	1	184690237	184754907
ENSG0000015565	1.500213797	####	###	TTN	protein_coding	titin [Source	2	178525989	178830802
ENSG0000027556	0.861651844	####	###	ARHGAP11	protein_coding	Rho GTPase	CHR_HSCHR	32695437	32720246
ENSG0000017530	1.849150727	####	###	CCNE2	protein_coding	cyclin E2 [Sc	8	94879770	94896678
ENSG0000001851	-0.787531877	####	###	AGPS	protein_coding	alkylglycero	2	177392757	177559299
ENSG0000015497	-4.793772629	####	###	CA10	protein_coding	carbonic an	17	51630313	52160017
ENSG0000016586	-1.690434211	####	###	HSPA12A	protein_coding	heat shock p	10	116671192	116850251
ENSG0000017473	-1.033942224	####	###	NR1D2	protein_coding	nuclear rece	3	23945286	23980617
ENSG0000009113	-0.550922252	####	###	LAMB1	protein_coding	laminin subu	7	107923799	108003187
ENSG0000008736	0.54676356	####	###	SF3B2	protein_coding	splicing fact	11	66050729	66069308
ENSG0000014637	-2.612282138	####	###	RNF217	protein_coding	ring finger p	6	124962545	125092633
ENSG0000012409	1.703642807	####	###	FAM210B	protein_coding	family with s	20	56358974	56368663
ENSG0000001029	0.617761062	####	###	NCAPD2	protein_coding	non-SMC co	12	6493356	6531955
ENSG0000010904	0.997507316	####	###	WSB1	protein_coding	WD repeat a	17	27294076	27315926
ENSG0000010359	1.059131791	####	###	AAGAB	protein_coding	alpha and g	15	67200667	67255195
ENSG0000018857	-2.122030792	####	###	FBLL1	protein_coding	fibrillarin like	5	168529305	168530634
ENSG0000024890	-1.152599695	####	###	FMN1	protein_coding	formin 1 [Sc	15	32765544	33194714
ENSG0000024731	-1.122634469	####	###	ZCCHC3	protein_coding	zinc finger C	20	297570	300321
ENSG0000010844	-1.499539269	####	###	TRIM16L	protein_coding	tripartite mo	17	18697998	18736118
ENSG0000016541	1.023562618	####	###	CFL2	protein_coding	cofilin 2 [So	14	34709113	34714823

ENSG0000018385	0.99785896	####	###	IQGAP3	protein_coding	IQ motif cor	1	156525405	156572604
ENSG0000006646	-2.635756328	####	###	FGFR2	protein_coding	fibroblast gr	10	121478332	121598458
ENSG0000021145	0.445788896	####	###	MT-RNR1	Mt_rRNA	mitochondr	MT	648	1601
ENSG0000017014	0.836431975	####	###	SIK2	protein_coding	salt inducibl	11	111602449	111730855
ENSG0000025976	2.394104096	####	###	AC004943.2	lncRNA	novel transc	16	72665123	72822781
ENSG0000011275	-1.12937829	####	###	SLC29A1	protein_coding	solute carrie	6	44219553	44234142
ENSG0000019745	0.591401609	####	###	HNRNPAB	protein_coding	heterogene	5	178204533	178211163
ENSG0000015136	0.725903324	####	###	NDUFC2	protein_coding	NADH:ubiqu	11	78068297	78079862
ENSG0000025472	-0.583423522	####	###	MEX3A	protein_coding	mex-3 RNA	1	156072013	156082465
ENSG0000021357	-3.838155527	####	###	CPLX3	protein_coding	complexin 3	15	74826627	74831802
ENSG0000012645	-0.803442037	####	###	PRMT1	protein_coding	protein argi	19	49675786	49689029
ENSG0000015562	-3.989300845	####	###	PIK3AP1	protein_coding	phosphoino	10	96593315	96720514
ENSG0000017292	0.900753618	####	###	RNASEH2C	protein_coding	ribonuclease	11	65714005	65720818
ENSG0000001547	-1.420171882	####	###	BID	protein_coding	BH3 interac	22	17734138	17774770
ENSG0000014600	0.864294705	####	###	ZMAT2	protein_coding	zinc finger r	5	140698680	140706686
ENSG0000010587	2.005331422	####	###	WDR91	protein_coding	WD repeat d	7	135183839	135211534
ENSG0000007061	-0.950726723	####	###	NDST1	protein_coding	N-deacetyla	5	150485818	150558211
ENSG0000011510	-2.319749574	####	###	STEAP3	protein_coding	STEAP3 met	2	119223831	119265652
ENSG0000017526	-3.090470228	####	###	CHST1	protein_coding	carbohydrat	11	45647689	45665622
ENSG0000013313	-1.886037977	####	###	BEX2	protein_coding	brain expres	X	103309346	103311007
ENSG0000012106	-1.158899348	####	###	SCPEP1	protein_coding	serine carbo	17	56978129	57006768
ENSG0000014787	-1.400412976	####	###	PLIN2	protein_coding	perilipin 2 [S	9	19108375	19149290
ENSG0000016381	-0.736394474	####	###	WDR43	protein_coding	WD repeat d	2	28894667	28948219
ENSG0000017291	-0.842260516	####	###	NBEA	protein_coding	neurobeach	13	34942287	35673022
ENSG0000013232	-2.176295755	####	###	RAMP1	protein_coding	receptor act	2	237858893	237912106
ENSG0000016229	-1.101384671	####	###	SYVN1	protein_coding	synoviolin 1	11	65121780	65134532
ENSG0000014057	0.697096744	####	###	IQGAP1	protein_coding	IQ motif cor	15	90388242	90502239
ENSG0000013251	-1.003361192	####	###	KDM6B	protein_coding	lysine deme	17	7839904	7854796
ENSG0000016737	-1.953697135	####	###	PRRT2	protein_coding	proline rich	16	29811382	29815892
ENSG0000016423	-1.868003605	####	###	CMBL	protein_coding	carboxymet	5	10275875	10307902
ENSG0000022871	0.833124492	####	###	DHFR	protein_coding	dihydrofolat	5	80626226	80654983
ENSG0000005498	-1.89956287	####	###	GALC	protein_coding	galactosylce	14	87837820	87993665
ENSG0000027566	-6.146500506	####	###	AC004556.2	misc_RNA		KI270721.1	52895	53010
ENSG0000013880	-0.678479502	####	###	PAPSS1	protein_coding	3'-phospho	4	107590276	107720234
ENSG0000015117	1.540681899	####	###	PLBD2	protein_coding	phospholipa	12	113358566	113391629
ENSG0000017345	0.939863322	####	###	RNF26	protein_coding	ring finger p	11	119334527	119337309
ENSG0000016544	-0.71033945	####	###	PHYHIPL	protein_coding	phytanoyl-C	10	59176643	59247774
ENSG0000022403	-1.209501985	####	###	EPB41L4A-	lncRNA	EPB41L4A a	5	112160526	112164818
ENSG0000008883	-0.800685857	####	###	FKBP1A	protein_coding	FKBP prolyl	20	1368978	1393172
ENSG0000012059	-3.706573088	####	###	PLXDC2	protein_coding	plexin doma	10	19816239	20289856
ENSG0000010981	-2.329768579	####	###	PPARGC1A	protein_coding	PPARG coac	4	23755041	23904089
ENSG0000014142	-0.911548644	####	###	SLC39A6	protein_coding	solute carrie	18	36108531	36129385
ENSG0000013197	-1.175954384	####	###	GCH1	protein_coding	GTP cyclohy	14	54842008	54902826
ENSG0000011980	-0.868381116	####	###	YPEL5	protein_coding	yippee like 5	2	30146941	30160533
ENSG0000027239	0.524170377	####	###	CD24	protein_coding	CD24 molec	6	106969831	106975627
ENSG0000014884	-0.913258733	####	###	PPRC1	protein_coding	PPARG relat	10	102132994	102150333
ENSG0000015291	-6.308732743	####	###	CNTNAP4	protein_coding	contactin as	16	76277278	76560757
ENSG0000011499	0.582775761	####	###	TTL	protein_coding	tubulin tyros	2	112482156	112541739
ENSG0000016495	1.171347773	####	###	PDP1	protein_coding	pyruvate de	8	93857807	93926068
ENSG0000013908	-1.517232678	####	###	ETV6	protein_coding	ETS variant	12	11649674	11895377
ENSG0000017281	-2.221449692	####	###	RARG	protein_coding	retinoic acic	12	53210567	53232980
ENSG0000018122	0.650552001	####	###	POLR2A	protein_coding	RNA polyme	17	7484366	7514616
ENSG0000010433	0.694934355	####	###	BPNT2	protein_coding	3'(2'), 5'-bis	8	56957931	56993867
ENSG0000013710	1.100684488	####	###	DCTN3	protein_coding	dynactin sul	9	34613545	34620523
ENSG0000026708	3.086403291	####	###	ASB16-AS1	lncRNA	ASB16 antis	17	44175968	44186723
ENSG0000012911	-1.087854544	####	###	PALLD	protein_coding	palladin, cyt	4	168497052	168928457
ENSG0000011752	1.034982059	####	###	ABCD3	protein_coding	ATP binding	1	94418389	94518666
ENSG0000014169	-1.420229455	####	###	P3H4	protein_coding	prolyl 3-hyc	17	41801947	41812604

ENSG0000016278	1.121246089	####	###	IER5	protein_coding	immediate e	1	181088700	181092900
ENSG0000010118	-2.72151197	####	###	SLCO4A1	protein_coding	solute carrie	20	62642503	62685785
ENSG0000010365	0.715969924	####	###	HERC1	protein_coding	HECT and R	15	63608618	63833948
ENSG0000011541	1.083161949	####	###	STAT1	protein_coding	signal transc	2	190908460	191020960
ENSG0000013771	0.636439018	####	###	RDX	protein_coding	radixin [Sou	11	109864295	110296712
ENSG0000010817	-1.38141142	####	###	DNAJC12	protein_coding	DnaJ heat sl	10	67796669	67838188
ENSG0000010024	0.913841875	####	###	CYB5R3	protein_coding	cytochrome	22	42617840	42649392
ENSG0000007859	-2.318160769	####	###	ITM2A	protein_coding	integral mei X		79360384	79367667
ENSG0000007213	-0.852995648	####	###	RPS6KA6	protein_coding	ribosomal p X		84058346	84188199
ENSG0000014053	1.31428488	####	###	TICRR	protein_coding	TOPBP1 inte	15	89575469	89631056
ENSG0000010865	0.417769289	####	###	DDX5	protein_coding	DEAD-box l	17	64498254	64508199
ENSG0000013783	1.583444364	####	###	SMAD6	protein_coding	SMAD famil	15	66702236	66782849
ENSG0000010086	4.404313998	####	###	DHRS2	protein_coding	dehydroger	14	23630115	23645639
ENSG0000018527	-1.195341657	####	###	ZBTB37	protein_coding	zinc finger a	1	173868082	173903549
ENSG0000015391	-0.601875224	####	###	SREK1	protein_coding	splicing regu	5	66139971	66183615
ENSG0000011970	0.5599471	####	###	RBM25	protein_coding	RNA binding	14	73058532	73123899
ENSG0000010050	0.787707697	####	###	NIN	protein_coding	ninein [Sour	14	50719763	50831162
ENSG0000018576	-1.109858455	####	###	KCNQ5	protein_coding	potassium v	6	72621792	73198851
ENSG0000003568	-0.964256777	####	###	NSMAF	protein_coding	neutral sphi	8	58583508	58659853
ENSG0000016527	3.33631223	####	###	AQP3	protein_coding	aquaporin 3	9	33441156	33447596
ENSG0000014554	1.143239289	####	###	SRD5A1	protein_coding	steroid 5 alp	5	6633427	6674386
ENSG0000010519	-0.560731531	####	###	RPS16	protein_coding	ribosomal p	19	39433137	39435949
ENSG0000017470	1.25523552	####	###	SH3PXD2B	protein_coding	SH3 and PX	5	172325000	172454525
ENSG0000010707	-1.546148427	####	###	KDM4C	protein_coding	lysine deme	9	6720863	7175648
ENSG0000016410	0.777885138	####	###	MAD2L1	protein_coding	mitotic arres	4	120055623	120066858
ENSG0000014993	0.914722367	####	###	TAOK2	protein_coding	TAO kinase	16	29973868	29992261
ENSG0000026346	-0.962755802	####	###	SRSF8	protein_coding	serine and a	11	95067197	95071224
ENSG0000015769	-1.614275075	####	###	TMEM268	protein_coding	transmembr	9	114611206	114646422
ENSG0000015745	0.829915506	####	###	CCNB2	protein_coding	cyclin B2 [So	15	59105126	59125045
ENSG0000016684	0.664144308	####	###	DCTN5	protein_coding	dynactin sul	16	23641466	23677472
ENSG0000017474	-1.800027336	####	###	FAM241A	protein_coding	family with s	4	112145454	112195256
ENSG0000012525	-1.238027706	####	###	ABCC4	protein_coding	ATP binding	13	95019835	95301475
ENSG0000013780	0.884966219	####	###	KIF23	protein_coding	kinesin fami	15	69414246	69448427
ENSG0000006318	-3.788339542	####	###	CA11	protein_coding	carbonic an	19	48637946	48646187
ENSG0000017041	-2.4841996	####	###	VSTM2A	protein_coding	V-set and tr	7	54542325	54571080
ENSG0000020381	2.992886491	####	###	H2BC18	protein_coding	H2B clustere	1	149782689	149812373
ENSG0000013883	0.794924966	####	###	MAPK8IP3	protein_coding	mitogen-ac	16	1706183	1770351
ENSG0000011868	-0.614167647	####	###	FOXO3	protein_coding	forkhead bc	6	108559835	108684774
ENSG0000017527	1.706327249	####	###	TP53I11	protein_coding	tumor prote	11	44885903	44951306
ENSG0000012636	-2.326822357	####	###	NR1D1	protein_coding	nuclear rece	17	40092793	40100589
ENSG0000011167	-0.904063997	####	###	ATN1	protein_coding	atrophin 1 [	12	6924463	6942321
ENSG0000018760	-0.603181871	####	###	TET3	protein_coding	tet methylcy	2	73986404	74108176
ENSG0000010537	-0.887379328	####	###	NOP53	protein_coding	NOP53 ribo	19	47745546	47757058
ENSG0000010436	-2.539367196	####	###	PLAT	protein_coding	plasminoge	8	42174718	42207676
ENSG0000022746	-1.883789	####	###	SYNGAP1	protein_coding	synaptic Ras CHR_HSCHR		33560260	33593877
ENSG0000014748	-6.647267515	####	###	PXDNL	protein_coding	peroxidasin	8	51319577	51809445
ENSG0000009198	-1.790521433	####	###	CCDC80	protein_coding	coiled-coil c	3	112596797	112649530
ENSG0000011092	0.877669316	####	###	CSRNP2	protein_coding	cysteine anc	12	51061205	51083664
ENSG0000013509	-2.379779651	####	###	MSI1	protein_coding	musashi RN	12	120341330	120369164
ENSG0000021453	-2.175955953	####	###	STARD10	protein_coding	StAR relatec	11	72754729	72793681
ENSG0000012731	0.805604804	####	###	RAP1B	protein_coding	RAP1B, mer	12	68610855	68671901
ENSG0000012824	0.763693532	####	###	YWHAH	protein_coding	tyrosine 3-r	22	31944522	31957603
ENSG0000011144	1.089689007	####	###	RFC5	protein_coding	replication f	12	118013588	118033130
ENSG0000009165	0.936115022	####	###	ORC6	protein_coding	origin recog	16	46689643	46698394
ENSG0000011870	-1.138516602	####	###	TGIF2	protein_coding	TGFB induce	20	36573488	36593950
ENSG0000018815	2.030525723	####	###	AGRN	protein_coding	agrin [Sourc	1	1020120	1056118
ENSG0000001134	-3.344062182	####	###	SYT7	protein_coding	synaptotagr	11	61513714	61581148
ENSG0000016223	0.80558585	####	###	NXF1	protein_coding	nuclear RNA	11	62792123	62806302

ENSG0000017161	1.20966171	####	###	ENC1	protein_coding	ectodermal-	5	74627406	74641424
ENSG0000013816	-0.923418756	####	###	TACC2	protein_coding	transforming	10	121989163	122254545
ENSG0000013957	-1.178927768	####	###	NABP2	protein_coding	nucleic acid	12	56222015	56229854
ENSG0000018147	-1.249893941	####	###	ZBTB2	protein_coding	zinc finger a	6	151364115	151391559
ENSG0000008720	-1.171632586	####	###	UIMC1	protein_coding	ubiquitin int	5	176905005	177022633
ENSG0000019855	1.043338194	####	###	WDHD1	protein_coding	WD repeat a	14	54938949	55027105
ENSG0000017332	-0.872841771	####	###	STOX2	protein_coding	storkhead b	4	183797692	184023526
ENSG0000016746	0.789777515	####	###	GPX4	protein_coding	glutathione	19	1103926	1106791
ENSG0000014472	-2.068021065	####	###	PTPRG	protein_coding	protein tyro	3	61561569	62297609
ENSG0000011546	-1.122295282	####	###	IGFBP5	protein_coding	insulin like g	2	216672105	216695549
ENSG0000010062	-2.774173049	####	###	SIX4	protein_coding	SIX homeob	14	60709539	60724351
ENSG0000012337	1.159101763	####	###	CDK2	protein_coding	cyclin deper	12	55966781	55972789
ENSG0000015255	-0.754373429	####	###	PFKM	protein_coding	phosphofru	12	48105139	48146404
ENSG0000019706	-0.840231922	####	###	MAFG	protein_coding	MAF bZIP tr	17	81918270	81927735
ENSG0000000670	-1.177186825	####	###	GTF2IRD1	protein_coding	GTF2I repea	7	74453790	74602604
ENSG0000015538	0.544122982	####	###	SLC16A1	protein_coding	solute carrie	1	112911847	112957013
ENSG0000016755	1.204821687	####	###	TUBA1C	protein_coding	tubulin alph	12	49188736	49274600
ENSG0000016021	0.822569346	####	###	G6PD	protein_coding	glucose-6-p X		154531391	154547572
ENSG0000020518	1.711207391	####	###	LINC00654	lncRNA	long interge	20	5482736	5526709
ENSG0000017145	-0.717939383	####	###	ASXL1	protein_coding	ASXL transc	20	32358330	32439319
ENSG0000019779	1.181607945	####	###	FAM118B	protein_coding	family with s	11	126211724	126262984
ENSG0000015251	-1.10658615	####	###	ZFP36L2	protein_coding	ZFP36 ring f	2	43222402	43226606
ENSG0000016346	-0.526363763	####	###	CCT3	protein_coding	chaperonin	1	156308968	156367873
ENSG0000006513	0.661954006	####	###	GNAI3	protein_coding	G protein su	1	109548615	109600195
ENSG0000019879	0.68455839	####	###	MTOR	protein_coding	mechanistic	1	11106535	11262551
ENSG0000023267	-1.458075905	####	###	LINC00665	lncRNA	long interge	19	36259540	36331770
ENSG0000028018	3.229961707	####	###	AC022107.1	TEC	TEC	5	69186359	69189452
ENSG0000007353	-1.319894781	####	###	NLE1	protein_coding	notchless hc	17	35128730	35142304
ENSG0000016694	-1.428853616	####	###	SMAD3	protein_coding	SMAD famil	15	67063763	67195169
ENSG0000010840	-0.682247957	####	###	DHX40	protein_coding	DEAH-box l	17	59565558	59608345
ENSG0000006716	-0.674273426	####	###	TRAM1	protein_coding	translocatio	8	70573218	70608416
ENSG0000016692	-2.893674591	####	###	NYAP1	protein_coding	neuronal tyr	7	100483927	100494802
ENSG0000018783	1.723570128	####	###	H1-2	protein_coding	H1.2 linker h	6	26055740	26056470
ENSG0000013482	-0.617622093	####	###	FADS2	protein_coding	fatty acid de	11	61792980	61867354
ENSG0000017327	-1.018153366	####	###	ZBTB21	protein_coding	zinc finger a	21	41986831	42010387
ENSG0000017096	-2.893875922	####	###	PDGFD	protein_coding	platelet deri	11	103907189	104164379
ENSG0000019725	1.449792178	####	###	KANK2	protein_coding	KN motif an	19	11164267	11197791
ENSG0000013529	-2.187411478	####	###	ANKRD6	protein_coding	ankyrin repe	6	89433152	89633834
ENSG0000017089	0.964181347	####	###	GSTA4	protein_coding	glutathione	6	52977948	52995304
ENSG0000013308	-1.138532101	####	###	DCLK1	protein_coding	doublecorti	13	35768652	36131382
ENSG0000007778	0.705477068	####	###	FGFR1	protein_coding	fibroblast gr	8	38400215	38468834
ENSG0000013200	-0.756655609	####	###	DNAJB1	protein_coding	DnaJ heat sl	19	14514769	14529770
ENSG0000016589	1.639065505	####	###	E2F7	protein_coding	E2F transcrip	12	77021248	77065569
ENSG0000011213	-1.593100552	####	###	PHACTR1	protein_coding	phosphatase	6	12716554	13287843
ENSG0000019760	0.673162641	####	###	FAR1	protein_coding	fatty acyl-C	11	13668668	13732346
ENSG0000011127	-0.617101324	####	###	CDKN1B	protein_coding	cyclin deper	12	12715058	12722369
ENSG0000012025	1.123740501	####	###	LRP11	protein_coding	LDL recepto	6	149818757	149864359
ENSG0000013627	0.990349741	####	###	DBNL	protein_coding	drebrin like	7	44044640	44069456
ENSG0000021407	0.707899911	####	###	CPNE1	protein_coding	copine 1 [Sc	20	35626031	35664956
ENSG0000012935	2.276945501	####	###	CDKN2D	protein_coding	cyclin deper	19	10566460	10569059
ENSG0000016356	-0.954357947	####	###	IFI16	protein_coding	interferon g	1	158999968	159055155
ENSG0000013487	-1.08081576	####	###	COL4A2	protein_coding	collagen typ	13	110305812	110513209
ENSG0000012741	1.81816055	####	###	TMEM175	protein_coding	transmembr	4	932387	958656
ENSG0000014489	-2.479194475	####	###	AGTR1	protein_coding	angiotensin	3	148697784	148743008
ENSG0000009638	-0.353902189	####	###	HSP90AB1	protein_coding	heat shock p	6	44246166	44253888
ENSG0000015908	0.672893879	####	###	PAXBP1	protein_coding	PAX3 and P	21	32733899	32771792
ENSG0000013917	-2.204282506	####	###	PRICKLE1	protein_coding	prickle plan	12	42456757	42590355
ENSG0000021397	1.871377842	####	###	TAX1BP3	protein_coding	Tax1 bindin	17	3662895	3668679

ENSG0000012842	-6.76896856	####	###	KRT17	protein_coding	keratin 17 [S	17	41619442	41624842
ENSG0000016109	1.218603	####	###	MFSD12	protein_coding	major facilit	19	3538261	3574290
ENSG0000011567	-0.465290901	####	###	HDLBP	protein_coding	high density	2	241227264	241317061
ENSG0000013340	-2.54135497	####	###	PDZD2	protein_coding	PDZ domain	5	31639131	32110932
ENSG0000018780	-1.641882583	####	###	ZFP69B	protein_coding	ZFP69 zinc f	1	40450102	40463718
ENSG0000003405	-1.300401847	####	###	APBA2	protein_coding	amyloid bet	15	28884483	29118315
ENSG0000019694	0.80803958	####	###	NOP9	protein_coding	NOP9 nucle	14	24299850	24309124
ENSG0000014052	0.691023174	####	###	ABHD2	protein_coding	abhydrolase	15	89087459	89202355
ENSG0000018723	1.279259644	####	###	SESTD1	protein_coding	SEC14 and s	2	179101678	179264832
ENSG0000016779	0.692683973	####	###	NDUFV1	protein_coding	NADH:ubiqu	11	67605653	67612554
ENSG0000013883	-1.200151744	####	###	RGS3	protein_coding	regulator of	9	113444731	113597743
ENSG0000026024	-1.358509575	####	###	AC104083.1	lncRNA	novel transc	4	155734448	155737062
ENSG0000014190	-0.883919172	####	###	NFIC	protein_coding	nuclear fact	19	3314403	3469217
ENSG0000023063	-1.552280734	####	###	DNM3OS	lncRNA	DNM3 oppo	1	172138397	172144840
ENSG0000011442	-0.845598188	####	###	CBLB	protein_coding	Cbl proto-o	3	105655461	105869552
ENSG0000007071	0.853292406	####	###	AP3M2	protein_coding	adaptor rela	8	42152946	42171673
ENSG0000017385	0.944287238	####	###	DPY19L1	protein_coding	dpy-19 like	7	34928876	35038271
ENSG0000011564	-1.463251016	####	###	FHL2	protein_coding	four and a h	2	105357712	105438513
ENSG0000013050	-0.557544402	####	###	PXDN	protein_coding	peroxidasin	2	1631887	1744852
ENSG0000012313	-0.948221956	####	###	DDX39A	protein_coding	DExD-box h	19	14408798	14419383
ENSG0000016765	-0.377554629	####	###	EEF2	protein_coding	eukaryotic t	19	3976056	3985463
ENSG0000011127	1.307950518	####	###	ACAD10	protein_coding	acyl-CoA de	12	111686053	111757107
ENSG0000011187	-2.073850689	####	###	FAM184A	protein_coding	family with s	6	118959763	119149387
ENSG0000015145	0.768405698	####	###	ANKRD50	protein_coding	ankyrin repe	4	124664048	124712732
ENSG0000010338	-1.464738032	####	###	CPPED1	protein_coding	calcineurin l	16	12659799	12803887
ENSG0000010147	0.864558753	####	###	APMAP	protein_coding	adipocyte p	20	24962925	24992751
ENSG0000010040	0.60490643	####	###	RANGAP1	protein_coding	Ran GTPase	22	41244779	41286187
ENSG0000017132	1.214963138	####	###	ESCO2	protein_coding	establishme	8	27771949	27812640
ENSG0000000486	-1.180854807	####	###	ST7	protein_coding	suppression	7	116953238	117230103
ENSG0000018313	1.485726703	####	###	CEP57L1	protein_coding	centrosoma	6	109095110	109163932
ENSG0000011730	1.808082165	####	###	HMGCL	protein_coding	3-hydroxy-3	1	23801885	23838620
ENSG0000010810	0.661346724	####	###	UBE2S	protein_coding	ubiquitin co	19	55399745	55407788
ENSG0000004278	-4.074165821	####	###	USH2A	protein_coding	usherin [Sou	1	215622891	216423448
ENSG0000016591	-2.680018725	####	###	PACSIN3	protein_coding	protein kina	11	47177522	47186443
ENSG0000014219	-0.417516466	####	###	APP	protein_coding	amyloid bet	21	25880550	26171128
ENSG0000016754	-0.607574995	####	###	KMT2D	protein_coding	lysine methy	12	49018978	49060794
ENSG0000014955	-1.577506772	####	###	FEZ1	protein_coding	fasciculation	11	125442881	125592568
ENSG0000016698	-1.141612014	####	###	MBD6	protein_coding	methyl-CpG	12	57520710	57530148
ENSG0000013253	-1.969674102	####	###	DLG4	protein_coding	discs large M	17	7187187	7219841
ENSG0000014377	0.842707783	####	###	GUK1	protein_coding	guanylate ki	1	228139962	228148984
ENSG0000018668	0.949403875	####	###	LYRM7	protein_coding	LYR motif co	5	131170944	131205428
ENSG0000004923	1.236146989	####	###	H6PD	protein_coding	hexose-6-p	1	9234774	9271337
ENSG0000007715	0.950983371	####	###	UBE2T	protein_coding	ubiquitin co	1	202331657	202341980
ENSG0000010926	-0.702399332	####	###	CRACD	protein_coding	capping pro	4	56049073	56328625
ENSG0000010060	1.255930948	####	###	LGMN	protein_coding	legumain [S	14	92703807	92748679
ENSG0000011604	-0.641122066	####	###	NFE2L2	protein_coding	nuclear fact	2	177227595	177392697
ENSG0000017720	-0.645330465	####	###	CHD9	protein_coding	chromodom	16	53055033	53329150
ENSG0000016777	1.897785764	####	###	SPRYD3	protein_coding	SPRY domai	12	53064316	53079404
ENSG0000016095	1.151376219	####	###	RECQL4	protein_coding	RecQ like he	8	144511288	144517845
ENSG0000011479	-0.620682092	####	###	KLHL24	protein_coding	kelch like fa	3	183635610	183684519
ENSG0000007794	-0.800960551	####	###	FBLN1	protein_coding	fibulin 1 [So	22	45502238	45601135
ENSG0000010825	-0.611314892	####	###	NUFIP2	protein_coding	nuclear FMF	17	29255839	29294148
ENSG0000017181	-2.603506622	####	###	PWWP2B	protein_coding	PWWP dom	10	132397168	132417859
ENSG0000025966	1.312162835	####	###	AC010478.1	lncRNA	novel transc	5	51372736	51383332
ENSG0000011940	-1.121959375	####	###	PHF19	protein_coding	PHD finger	9	120855651	120894896
ENSG0000018905	1.384557251	####	###	FAM111B	protein_coding	family with s	11	59107185	59127412
ENSG0000016224	-0.479596935	####	###	RPL29	protein_coding	ribosomal p	3	51993522	51995895
ENSG0000016755	1.655384225	####	###	ZNF528	protein_coding	zinc finger p	19	52397849	52418412

ENSG0000012826	-2.635631474	####	###	MGAT3	protein_coding	beta-1,4-m	22	39457012	39492194
ENSG0000007088	-0.755153122	####	###	OSBPL3	protein_coding	oxysterol bi	7	24796540	24981634
ENSG0000006613	-0.756391698	####	###	KDM4A	protein_coding	lysine deme	1	43650149	43705518
ENSG0000006070	-1.034443269	####	###	RIMBP2	protein_coding	RIMS bindin	12	130396137	130716281
ENSG0000011593	-0.845436835	####	###	WIPF1	protein_coding	WAS/WASL	2	174559572	174682916
ENSG0000006541	-0.837398657	####	###	ANKRD44	protein_coding	ankyrin repe	2	196967017	197311173
ENSG0000018518	1.912360925	####	###	NRBP2	protein_coding	nuclear rece	8	143833583	143840973
ENSG0000014821	1.373512164	####	###	ALAD	protein_coding	aminolevuli	9	113386312	113401290
ENSG0000010099	0.584861344	####	###	PYGB	protein_coding	glycogen ph	20	25248085	25298012
ENSG0000007049	-1.283076312	####	###	JMJD6	protein_coding	jumonji dom	17	76712832	76726799
ENSG0000018391	-3.196818605	####	###	DNAH2	protein_coding	dynein axor	17	7717354	7833744
ENSG0000000851	-1.279358457	####	###	ST3GAL1	protein_coding	ST3 beta-ga	8	133454848	133571940
ENSG0000010126	-0.699216937	####	###	CSNK2A1	protein_coding	casein kinas	20	472498	543835
ENSG0000018434	-0.816815569	####	###	SLIT3	protein_coding	slit guidance	5	168661733	169301139
ENSG0000016616	0.629283786	####	###	CKB	protein_coding	creatine kin	14	103519667	103522833
ENSG0000014432	0.721147224	####	###	LNPK	protein_coding	lunapark, EF	2	175923882	176002839
ENSG0000016164	-2.297328657	####	###	ZNF385A	protein_coding	zinc finger p	12	54369133	54391298
ENSG0000016518	-5.213883614	####	###	PTCHD1	protein_coding	patched dor	X	23334849	23404374
ENSG0000018256	-0.829280982	####	###	SATB1	protein_coding	SATB home	3	18345377	18445588
ENSG0000007768	-0.881353572	####	###	JADE1	protein_coding	jade family	4	128809700	128875224
ENSG0000016491	-0.786338459	####	###	FOXK1	protein_coding	forkhead bc	7	4682295	4771442
ENSG0000007540	-0.889353445	####	###	ZNF37A	protein_coding	zinc finger p	10	38094334	38150293
ENSG0000011227	-0.973142119	####	###	BVES	protein_coding	blood vesse	6	105096822	105137157
ENSG0000013570	1.809197391	####	###	KIAA0513	protein_coding	KIAA0513 [S	16	85027751	85094230
ENSG0000016913	-1.845892942	####	###	ATF5	protein_coding	activating tr	19	49928702	49933935
ENSG0000018213	1.052294303	####	###	TDRKH	protein_coding	tudor and K	1	151770107	151791416
ENSG0000019864	0.941137656	####	###	KLHL9	protein_coding	kelch like fa	9	21329665	21335404
ENSG0000018262	0.755631564	####	###	SKA2	protein_coding	spindle and	17	59109857	59155260
ENSG0000013976	0.988481022	####	###	SRRM4	protein_coding	serine/argin	12	118981541	119163051
ENSG0000017306	-4.405325234	####	###	BNC2	protein_coding	basonuclin 2	9	16409503	16870843
ENSG0000005452	0.542988409	####	###	KIF1B	protein_coding	kinesin fami	1	10210570	10381603
ENSG0000017014	-1.057229877	####	###	UBE2E1	protein_coding	ubiquitin co	3	23805955	23891640
ENSG0000017039	-1.05808618	####	###	ZNF804A	protein_coding	zinc finger p	2	184598529	184939492
ENSG0000015063	-2.874900823	####	###	VEGFC	protein_coding	vascular enc	4	176683538	176792922
ENSG0000010032	-1.7915484	####	###	SYNGR1	protein_coding	synaptogyri	22	39349925	39385575
ENSG0000018724	-1.178317381	####	###	DYNC2H1	protein_coding	dynein cyto	11	103109410	103479863
ENSG0000013150	0.628188366	####	###	DIAPH1	protein_coding	diaphanous	5	141515016	141619055
ENSG0000011994	-2.623718128	####	###	CNNM1	protein_coding	cyclin and C	10	99329356	99394330
ENSG0000016640	-0.578429615	####	###	TUB	protein_coding	TUB bipartit	11	8019244	8106243
ENSG0000016668	-4.812938511	####	###	PLEKHA7	protein_coding	pleckstrin h	11	16777297	17014414
ENSG0000010175	0.808317901	####	###	MIB1	protein_coding	mindbomb	18	21704957	21870957
ENSG0000015249	-0.490365737	####	###	CAMK4	protein_coding	calcium/caln	5	111223653	111494886
ENSG0000017011	-0.887129371	####	###	NIPA1	protein_coding	NIPA magn	15	22773063	22829789
ENSG0000023474	-0.587473942	####	###	GAS5	lncRNA	growth arre	1	173858559	173868882
ENSG0000018448	-2.093900602	####	###	FOXO4	protein_coding	forkhead bc	X	71095851	71103532
ENSG0000010135	0.837090948	####	###	KIF3B	protein_coding	kinesin fami	20	32277651	32335011
ENSG0000012758	1.221376827	####	###	CHTF18	protein_coding	chromosom	16	788046	800737
ENSG0000017518	1.880243789	####	###	CSRP2	protein_coding	cysteine anc	12	76858709	76879023
ENSG0000018209	1.295555065	####	###	GET1	protein_coding	guided entr	21	39377698	39428528
ENSG0000000723	-2.133791774	####	###	GAS7	protein_coding	growth arre	17	9910609	10198551
ENSG0000013629	0.620911481	####	###	TTYH3	protein_coding	tweety fami	7	2631986	2664802
ENSG0000008118	-2.186652581	####	###	ARG2	protein_coding	arginase 2 [	14	67619920	67651708
ENSG0000016921	-2.511273322	####	###	RAB3B	protein_coding	RAB3B, mer	1	51907956	51990700
ENSG0000017266	-0.927632414	####	###	WASHC2C	protein_coding	WASH comp	10	45727200	45792964
ENSG0000000871	0.585703813	####	###	PKD1	protein_coding	polycystin 1	16	2088708	2135898
ENSG0000020638	-2.881525329	####	###	COL6A6	protein_coding	collagen typ	3	130560334	130678155
ENSG0000010021	1.308033253	####	###	CBY1	protein_coding	chibby fami	22	38656636	38673854
ENSG0000022192	-1.118684708	####	###	TRIM16	protein_coding	tripartite mc	17	15627960	15684311

ENSG0000006545	1.04102523	####	###	ADAT1	protein_coding	adenosine c	16	75596981	75623300
ENSG0000026316	-1.62357595	####	###	TMEM50B	protein_coding	transmembı	CHR_HSCHR	33432485	33489568
ENSG0000017703	-1.194941643	####	###	DEAF1	protein_coding	DEAF1 trans	11	644233	706715
ENSG0000016913	0.634637296	####	###	UBE2V2	protein_coding	ubiquitin co	8	48008415	48064708
ENSG0000007635	-1.49420249	####	###	PLXNA2	protein_coding	plexin A2 [S	1	208022242	208244384
ENSG0000004814	1.253064677	####	###	TSPAN17	protein_coding	tetraspanin	5	176647387	176659054
ENSG0000007600	0.74339294	####	###	MCM6	protein_coding	minichromo	2	135839626	135876443
ENSG0000016880	0.744808091	####	###	CHTF8	protein_coding	chromosom	16	69118010	69132588
ENSG0000001125	-0.713658264	####	###	MBTD1	protein_coding	mbt domair	17	51177425	51260163
ENSG0000017444	-0.401791513	####	###	RPL4	protein_coding	ribosomal p	15	66498015	66524532
ENSG0000007421	-1.811171776	####	###	PPP2R2C	protein_coding	protein pho	4	6320578	6563600
ENSG0000016361	-1.266790743	####	###	CADPS	protein_coding	calcium dep	3	62398346	62875416
ENSG0000015157	-2.574455412	####	###	ANO4	protein_coding	anoctamin 4	12	100717526	101128641
ENSG0000013147	0.958818896	####	###	VPS25	protein_coding	vacuolar prc	17	42773449	42779599
ENSG0000015629	-1.157780246	####	###	TSPAN7	protein_coding	tetraspanin	X	38561542	38688920
ENSG0000016490	0.716237358	####	###	ALDH7A1	protein_coding	aldehyde de	5	126531200	126595362
ENSG0000016017	-3.094190858	####	###	ABCG1	protein_coding	ATP binding	21	42199689	42297244
ENSG0000013781	0.770084888	####	###	RTF1	protein_coding	RTF1 homol	15	41408408	41483563
ENSG0000012406	1.751409998	####	###	SLC12A4	protein_coding	solute carrie	16	67943474	67969601
ENSG0000014218	-1.626009488	####	###	TMEM50B	protein_coding	transmembı	21	33432485	33479974
ENSG0000021391	1.142820169	####	###	DNASE1	protein_coding	deoxyribonu	16	3611728	3680143
ENSG0000016971	-0.484797936	####	###	CNBP	protein_coding	CCHC-type	3	129167827	129183922
ENSG0000015149	0.971424318	####	###	PTPRO	protein_coding	protein tyro	12	15322257	15602175
ENSG0000006515	0.732936077	####	###	OAT	protein_coding	ornithine an	10	124397303	124418976
ENSG0000018500	-1.305769915	####	###	ROBO2	protein_coding	roundabout	3	75906695	77649964
ENSG0000013784	0.653735003	####	###	ADAM10	protein_coding	ADAM meta	15	58588809	58749791
ENSG0000019658	0.990552953	####	###	XRCC2	protein_coding	X-ray repair	7	152644776	152676141
ENSG0000015648	-0.498142945	####	###	RPL30	protein_coding	ribosomal p	8	98024851	98046469
ENSG0000017928	-7.216366426	####	###	DAND5	protein_coding	DAN domai	19	12965159	12974762
ENSG0000014040	1.015877411	####	###	MAN2C1	protein_coding	mannosidas	15	75355207	75368612
ENSG0000025097	-1.762497373	####	###	AC022905.1	unprocessed_p	transient rec	8	72202751	72251565
ENSG0000021414	-1.82328651	####	###	PRCD	protein_coding	photorecep	17	76527586	76553578
ENSG0000027112	2.169489971	####	###	AC018647.2	lncRNA	novel transc	7	35695214	35699413
ENSG0000012596	1.20353676	####	###	MMP24	protein_coding	matrix meta	20	35226690	35276998
ENSG0000017858	1.705541821	####	###	CTNNBIP1	protein_coding	catenin beta	1	9848276	9910336
ENSG0000000470	0.912033707	####	###	RECQL	protein_coding	RecQ like he	12	21468910	21501669
ENSG0000010028	0.830505836	####	###	AP1B1	protein_coding	adaptor rela	22	29327680	29423179
ENSG0000003058	0.800676866	####	###	GRN	protein_coding	granulin pre	17	44345246	44353106
ENSG0000011434	0.66723915	####	###	ECT2	protein_coding	epithelial ce	3	172750682	172821474
ENSG0000019678	-1.000277076	####	###	MAML3	protein_coding	mastermind	4	139716753	140154184
ENSG0000018205	0.718711039	####	###	IDH2	protein_coding	isocitrate de	15	90083045	90102477
ENSG0000016799	-1.113584699	####	###	BEST1	protein_coding	bestrophin 1	11	61950063	61965515
ENSG0000014811	0.955316121	####	###	MFSD14B	protein_coding	major facilit	9	94374569	94461042
ENSG0000010679	0.868335977	####	###	TGFBR1	protein_coding	transforming	9	99104038	99154192
ENSG0000001281	-1.788974505	####	###	KDM5D	protein_coding	lysine deme	Y	19703865	19744939
ENSG0000010685	1.284992391	####	###	PTGR1	protein_coding	prostagland	9	111549722	111599893
ENSG0000022370	1.329963408	####	###	NSUN5P1	transcribed_un	NSUN5 pseu	7	75410322	75416787
ENSG0000012896	-1.033580545	####	###	CHAC1	protein_coding	ChaC glutat	15	40952962	40956512
ENSG0000016643	1.444609971	####	###	TRIM66	protein_coding	tripartite mo	11	8612037	8682694
ENSG0000017142	-1.837866288	####	###	ZNF581	protein_coding	zinc finger p	19	55635459	55645623
ENSG0000011715	0.967791217	####	###	KLHL12	protein_coding	kelch like fa	1	202891116	202928636
ENSG0000013550	0.5918929	####	###	OS9	protein_coding	OS9 endopl	12	57693955	57721557
ENSG0000019728	-1.743057695	####	###	SYNGAP1	protein_coding	synaptic Ras	6	33419661	33453689
ENSG0000017589	-2.576209867	####	###	NA	NA	NA	NA	NA	NA
ENSG0000022911	-0.354532916	####	###	RPL41	protein_coding	ribosomal p	12	56116590	56117967
ENSG0000012194	1.078897159	####	###	CLCC1	protein_coding	chloride cha	1	108927361	108963504
ENSG0000011493	-1.205563189	####	###	INO80D	protein_coding	INO80 comp	2	205993721	206086303
ENSG0000011027	0.835630354	####	###	CEP164	protein_coding	centrosoma	11	117314557	117413266

ENSG0000008082	0.762674663	####	###	CLDND1	protein_codinc	claudin dom	3	98497912	98523066
ENSG0000012870	-2.15657511	####	###	HOXD9	protein_codinc	homeobox I	2	176122719	176124937
ENSG0000013287	-1.169342999	####	###	SYT4	protein_codinc	synaptotagr	18	43267892	43277535
ENSG0000027263	-2.507899846	####	###	DOC2B	protein_codinc	double C2 c	17	142789	181650
ENSG0000018695	-1.238670414	####	###	PPARA	protein_codinc	peroxisome	22	46150521	46243756
ENSG0000006460	0.610316805	####	###	SUGP2	protein_codinc	SURP and G	19	18990888	19034023
ENSG0000014314	-1.317002332	####	###	GPR161	protein_codinc	G protein-c	1	168079543	168137667
ENSG0000016797	-2.895371326	####	###	CASKIN1	protein_codinc	CASK intera	16	2177180	2196605
ENSG0000006515	-0.528882358	####	###	IPO5	protein_codinc	importin 5 [	13	97953658	98024296
ENSG0000014324	-0.360981844	####	###	RGS5	protein_codinc	regulator of	1	163111121	163321791
ENSG0000013363	-0.728989727	####	###	BTG1	protein_codinc	BTG anti-pr	12	92140278	92145846
ENSG0000018222	0.77217906	####	###	ATP6AP2	protein_codinc	ATPase H+	X	40579372	40606848
ENSG0000015785	-0.723193712	####	###	DPYSL5	protein_codinc	dihydropyri	2	26847747	26950351
ENSG0000004839	1.118469281	####	###	RRM2B	protein_codinc	ribonucleoti	8	102204502	102238961
ENSG0000017876	-3.380220291	####	###	ZHX2	protein_codinc	zinc fingers	8	122781655	122974510
ENSG0000025074	-1.472972714	####	###	LINC02381	lncRNA	long interge	12	54126082	54147485
ENSG0000019620	-0.822563734	####	###	GREB1	protein_codinc	growth regu	2	11482341	11642788
ENSG0000019870	0.842181849	####	###	CEP290	protein_codinc	centrosoma	12	88049016	88142099
ENSG0000012815	1.055111677	####	###	TUBGCP6	protein_codinc	tubulin gam	22	50217689	50245023
ENSG0000012478	-0.594814872	####	###	NUP153	protein_codinc	nucleoporin	6	17615035	17706925
ENSG0000016797	-0.686667622	####	###	ABCA3	protein_codinc	ATP binding	16	2275881	2340746
ENSG0000015768	-0.655600019	####	###	DGKI	protein_codinc	diacylglycer	7	137381037	137847092
ENSG0000016732	0.644326832	####	###	RRM1	protein_codinc	ribonucleoti	11	4094707	4138932
ENSG0000011365	0.597464648	####	###	SMAD5	protein_codinc	SMAD famil	5	136132845	136188747
ENSG0000016243	0.718034272	####	###	JAK1	protein_codinc	Janus kinase	1	64833229	65067754
ENSG0000016790	1.222611853	####	###	TK1	protein_codinc	thymidine k	17	78174091	78187233
ENSG0000016943	-1.426853963	####	###	COL22A1	protein_codinc	collagen typ	8	138588235	138914041
ENSG0000006669	-1.046823911	####	###	MSANTD3	protein_codinc	Myb/SANT	9	100427143	100451734
ENSG0000007283	0.502900613	####	###	CRMP1	protein_codinc	collapsin res	4	5748084	5893086
ENSG0000001421	-1.354587993	####	###	CAPN1	protein_codinc	calpain 1 [S	11	65180566	65212006
ENSG0000012269	1.400040574	####	###	GLIPR2	protein_codinc	GLI pathoge	9	36136536	36163913
ENSG0000016433	0.93306957	####	###	UBLCP1	protein_codinc	ubiquitin lik	5	159263290	159286036
ENSG0000011545	-0.65011911	####	###	IGFBP2	protein_codinc	insulin like g	2	216632828	216664436
ENSG0000006888	0.808202358	####	###	IFT80	protein_codinc	intraflagella	3	160256986	160399880
ENSG0000016688	0.710366991	####	###	NEMP1	protein_codinc	nuclear env	12	57055643	57088063
ENSG0000017634	0.716593813	####	###	COX8A	protein_codinc	cytochrome	11	63974620	63976543
ENSG0000018502	-2.389448475	####	###	MAFF	protein_codinc	MAF bZIP tr	22	38200767	38216507
ENSG0000010090	-1.21834412	####	###	NFKBIA	protein_codinc	NFKB inhibi	14	35401513	35404749
ENSG0000000838	1.76979279	####	###	MPND	protein_codinc	MPN domai	19	4343527	4360086
ENSG0000008905	0.567419005	####	###	ANAPC5	protein_codinc	anaphase pr	12	121308245	121399896
ENSG0000014142	1.185303235	####	###	GALNT1	protein_codinc	polypeptide	18	35581117	35711834
ENSG0000017050	-0.590625334	####	###	LONRF2	protein_codinc	LON peptid	2	100271875	100322501
ENSG0000011355	0.594098251	####	###	SKP1	protein_codinc	S-phase kin	5	134148935	134176964
ENSG0000008725	0.981156499	####	###	LPCAT2	protein_codinc	lysophospha	16	55509072	55586666
ENSG0000012947	-2.162970547	####	###	AJUBA	protein_codinc	ajuba LIM p	14	22971177	22982551
ENSG0000011007	0.535834537	####	###	PPP6R3	protein_codinc	protein pho	11	68460731	68615334
ENSG0000016425	0.853863402	####	###	AGGF1	protein_codinc	angiogenic	5	77029251	77065234
ENSG0000018121	-1.357328716	####	###	H2AW	protein_codinc	H2A.W histc	1	228456979	228457873
ENSG0000018247	0.69374897	####	###	EXOC7	protein_codinc	exocyst con	17	76081016	76121576
ENSG0000017246	-1.663698711	####	###	FUT9	protein_codinc	fucosyltrans	6	96015974	96215612
ENSG0000006807	4.93749808	####	###	FGFR3	protein_codinc	fibroblast gr	4	1793293	1808872
ENSG0000013813	-1.38382367	####	###	STAMBPL1	protein_codinc	STAM bindi	10	88879734	88975153
ENSG0000013716	-1.131233762	####	###	CNPY3	protein_codinc	canopy FGF	6	42929192	42939294
ENSG0000009996	-0.645508075	####	###	BCL2L13	protein_codinc	BCL2 like 13	22	17628855	17730855
ENSG0000016431	-4.699488296	####	###	EGFLAM	protein_codinc	EGF like, fib	5	38258409	38465480
ENSG0000013764	-0.933843592	####	###	SORL1	protein_codinc	sortilin relat	11	121452314	121633763
ENSG0000016263	0.904745811	####	###	FAM102B	protein_codinc	family with s	1	108560089	108644900
ENSG0000012890	-0.943259837	####	###	INO80	protein_codinc	INO80 comp	15	40978880	41116280

ENSG0000018266	-2.088950503	####	###	NTM	protein_codinc	neurotrimin	11	131370478	132336822
ENSG0000024333	-1.430958512	####	###	KCTD7	protein_codinc	potassium c	7	66628767	66649067
ENSG0000017211	0.523604967	####	###	CYCS	protein_codinc	cytochrome	7	25118656	25125260
ENSG0000014435	0.831419177	####	###	CDCA7	protein_codinc	cell division	2	173354820	173368997
ENSG0000019894	-3.230832383	####	###	DMD	protein_codinc	dystrophin [	X	31097677	33339441
ENSG0000016571	-2.044041037	####	###	DIPK1B	protein_codinc	divergent pı	9	136712572	136724742
ENSG0000007219	-0.983315327	####	###	SPEG	protein_codinc	striated mus	2	219434843	219493629
ENSG0000010498	-0.806622457	####	###	TIMM44	protein_codinc	translocase	19	7926718	7943667
ENSG0000013660	-0.763065386	####	###	SKIL	protein_codinc	SKI like prot	3	170357678	170396835
ENSG0000017870	1.570162197	####	###	DHFR2	protein_codinc	dihydrofolat	3	94047836	94063389
ENSG0000016600	0.840035576	####	###	CEP295	protein_codinc	centrosoma	11	93661682	93730358
ENSG0000011762	-0.824380125	####	###	RCOR3	protein_codinc	REST corepı	1	211258377	211316385
ENSG0000004135	0.598537147	####	###	PSMA4	protein_codinc	proteasome	15	78540405	78552417
ENSG0000016029	-0.760755921	####	###	PCNT	protein_codinc	pericentrin [	21	46324141	46445769
ENSG0000019869	-1.762305405	####	###	EIF1AY	protein_codinc	eukaryotic t	Y	20575776	20593154
ENSG0000010957	-0.515517142	####	###	CLCN3	protein_codinc	chloride vol	4	169612633	169723673
ENSG0000015313	1.571620505	####	###	CLGN	protein_codinc	calmegin [S	4	140388453	140427661
ENSG0000011241	-2.598743951	####	###	ADGRG6	protein_codinc	adhesion G	6	142301854	142446266
ENSG0000016020	-0.786538762	####	###	CBS	protein_codinc	cystathionin	21	43053191	43076943
ENSG0000013076	-0.944311843	####	###	SESN2	protein_codinc	sestrin 2 [Sc	1	28259518	28282491
ENSG0000007441	-3.863606713	####	###	MGLL	protein_codinc	monoglycer	3	127689062	128052190
ENSG0000004335	1.93900294	####	###	ZIC2	protein_codinc	Zic family m	13	99981784	99986765
ENSG0000016388	-1.889372122	####	###	KLF15	protein_codinc	Kruppel like	3	126342635	126357408
ENSG0000010867	-0.726168712	####	###	LGALS3BP	protein_codinc	galectin 3 b	17	78971238	78979947
ENSG0000019715	1.253089498	####	###	ABCB8	protein_codinc	ATP bindinc	7	151028422	151047782
ENSG0000004135	-2.081810222	####	###	RAB27B	protein_codinc	RAB27B, me	18	54717860	54895516
ENSG0000014706	-0.525728586	####	###	MSN	protein_codinc	moesin [Sou	X	65588377	65741931
ENSG0000013146	0.809090199	####	###	TUBG1	protein_codinc	tubulin gam	17	42609683	42615238
ENSG0000024069	-0.607627337	####	###	PNMA2	protein_codinc	PNMA famil	8	26504701	26514092
ENSG0000025371	0.592986804	####	###	ATXN7L3B	protein_codinc	ataxin 7 like	12	74537835	74545430
ENSG0000017079	2.105068525	####	###	CHCHD7	protein_codinc	coiled-coil-	8	56211686	56218809
ENSG0000005162	-1.381226139	####	###	HEBP2	protein_codinc	heme bindiı	6	138403531	138422197
ENSG0000007465	-0.938593684	####	###	ZNF532	protein_codinc	zinc finger p	18	58862600	58986480
ENSG0000013123	-0.817264007	####	###	PPT1	protein_codinc	palmitoyl-p	1	40072710	40097260
ENSG0000010050	1.086290719	####	###	TRIM9	protein_codinc	tripartite mc	14	50975262	51096061
ENSG0000016380	1.074244056	####	###	KIF15	protein_codinc	kinesin fami	3	44761721	44873376
ENSG0000017237	0.886038516	####	###	ARNT2	protein_codinc	aryl hydroca	15	80404350	80597933
ENSG0000013865	0.980049317	####	###	ZGRF1	protein_codinc	zinc finger C	4	112539333	112636995
ENSG0000012804	1.526606216	####	###	RASL11B	protein_codinc	RAS like fam	4	52862317	52866835
ENSG0000018930	1.019978895	####	###	RRP7A	protein_codinc	ribosomal R	22	42508344	42519796
ENSG0000018446	1.190200975	####	###	WDR27	protein_codinc	WD repeat c	6	169457212	169702067
ENSG0000014395	-0.801598697	####	###	VPS54	protein_codinc	VPS54 subu	2	63892146	64019428
ENSG0000016871	0.606585639	####	###	AHCYL1	protein_codinc	adenosylhoı	1	109984765	110023742
ENSG0000019635	-1.597337907	####	###	CD55	protein_codinc	CD55 molec	1	207321532	207386804
ENSG0000013999	-2.085067499	####	###	RAB15	protein_codinc	RAB15, mer	14	64945814	64973226
ENSG0000018893	1.124956539	####	###	FAM120AC	protein_codinc	family with s	9	93431441	93453581
ENSG0000017115	1.52702681	####	###	C9orf16	protein_codinc	chromosom	9	128160265	128163924
ENSG0000016446	-1.26341106	####	###	CREBRF	protein_codinc	CREB3 regu	5	173056352	173139284
ENSG0000014485	-1.295356984	####	###	BOC	protein_codinc	BOC cell ad	3	113211003	113287459
ENSG0000019722	-0.948971546	####	###	TBC1D9B	protein_codinc	TBC1 doma	5	179862066	179907859
ENSG0000014054	0.976453432	####	###	MFGE8	protein_codinc	milk fat glol	15	88898683	88913381
ENSG0000013101	-0.972804039	####	###	SYNE1	protein_codinc	spectrin rep	6	152121684	152637801
ENSG0000016345	-3.306961733	####	###	IGFBP7	protein_codinc	insulin like g	4	57030773	57110385
ENSG0000014068	0.977003444	####	###	RUSF1	protein_codinc	RUS family ı	16	31489471	31509309
ENSG0000010144	0.977066539	####	###	FAM83D	protein_codinc	family with s	20	38926312	38953106
ENSG0000016382	-2.979123042	####	###	LRRC2	protein_codinc	leucine rich	3	46515385	46580099
ENSG0000008506	1.020619788	####	###	CD59	protein_codinc	CD59 molec	11	33703010	33736479
ENSG0000010482	-1.975695561	####	###	NFKBIB	protein_codinc	NFKB inhibit	19	38899700	38908893

ENSG0000025716	2.536722629	####	###	TMPO-AS1	lncRNA	TMPO antis	12	98512973	98516422
ENSG0000009704	-0.791060716	####	###	CDC7	protein_coding	cell division	1	91500851	91525764
ENSG0000017191	0.9928144	####	###	TLN2	protein_coding	talin 2 [Sour	15	62390526	62844631
ENSG0000017896	0.914532643	####	###	RMI1	protein_coding	RecQ media	9	83980798	84004074
ENSG0000013431	0.521222831	####	###	KIDINS220	protein_coding	kinase D int	2	8721081	8837630
ENSG0000005100	1.453518087	####	###	FAM160A2	protein_coding	family with s	11	6211345	6234711
ENSG0000008647	-0.594078958	####	###	SEPHS1	protein_coding	selenophosp	10	13317428	13348298
ENSG0000019884	-1.19104888	####	###	TOX	protein_coding	thymocyte s	8	58805412	59119147
ENSG0000018664	0.912017555	####	###	PDE2A	protein_coding	phosphodie	11	72576141	72674591
ENSG0000013123	0.595587556	####	###	CAP1	protein_coding	cyclase assc	1	40040233	40072649
ENSG0000014456	0.769104959	####	###	VGLL4	protein_coding	vestigial like	3	11556069	11771350
ENSG0000007727	-4.274540624	####	###	CAPN6	protein_coding	calpain 6 [S	X	111245099	111270483
ENSG0000011798	-0.764226403	####	###	CTSD	protein_coding	cathepsin D	11	1752755	1764573
ENSG0000017109	1.264314333	####	###	ALK	protein_coding	ALK recepto	2	29192774	29921586
ENSG0000027649	-1.273463523	####	###	APBA2	protein_coding	amyloid bet	CHR_HSCHR	29060035	29259075
ENSG0000013480	-1.796217812	####	###	SLC43A3	protein_coding	solute carrie	11	57406954	57427580
ENSG0000012420	0.605648955	####	###	CSE1L	protein_coding	chromosom	20	49046246	49096960
ENSG0000020460	2.067519605	####	###	ZNF468	protein_coding	zinc finger p	19	52838010	52857649
ENSG0000014519	-2.407420678	####	###	VWA5B2	protein_coding	von Willebra	3	184230429	184242329
ENSG0000016280	1.361579139	####	###	SNED1	protein_coding	sushi, nidog	2	240998618	241095568
ENSG0000023744	1.617402079	####	###	ZNF737	protein_coding	zinc finger p	19	20535825	20565809
ENSG0000008726	-0.804354467	####	###	NOP14	protein_coding	NOP14 nucl	4	2937933	2963406
ENSG0000017981	-1.560331416	####	###	PCBP1-AS1	lncRNA	PCBP1 antis	2	69962263	70103220
ENSG0000018652	0.853405938	####	###	SEPTIN10	protein_coding	septin 10 [S	2	109542799	109614143
ENSG0000014817	-1.091560022	####	###	STOM	protein_coding	stomatin [So	9	121338987	121370304
ENSG0000017351	1.241733145	####	###	PEAK1	protein_coding	pseudopodi	15	77100656	77420144
ENSG0000006324	-1.664178999	####	###	ISOC2	protein_coding	isochorisma	19	55452985	55462343
ENSG0000015336	1.880394661	####	###	LINC00467	lncRNA	long interge	1	211382736	211435570
ENSG0000012458	1.153684737	####	###	NQO2	protein_coding	N-ribosyldih	6	2987987	3021850
ENSG0000017646	-1.297243404	####	###	SLCO3A1	protein_coding	solute carrie	15	91853708	92172435
ENSG0000010134	1.062471502	####	###	SAMHD1	protein_coding	SAM and HI	20	36890229	36951843
ENSG0000016617	-1.131757971	####	###	LARP6	protein_coding	La ribonucle	15	70829130	70854157
ENSG0000018861	1.305870155	####	###	FAM72B	protein_coding	family with s	1	121167646	121185539
ENSG0000013102	-0.696804394	####	###	LATS1	protein_coding	large tumor	6	149658153	149718105
ENSG0000018832	1.406296206	####	###	ZNF559	protein_coding	zinc finger p	19	9323772	9351162
ENSG0000010290	-0.64490616	####	###	NFAT5	protein_coding	nuclear facto	16	69565094	69704666
ENSG0000014340	-0.523392672	####	###	ANP32E	protein_coding	acidic nucle	1	150218417	150236156
ENSG0000011529	-1.147306729	####	###	CLIP4	protein_coding	CAP-Gly do	2	29097705	29189643
ENSG0000013058	1.64174775	####	###	HELZ2	protein_coding	helicase witl	20	63558086	63574239
ENSG0000018202	1.532254673	####	###	CHST15	protein_coding	carbohydrat	10	124007668	124093598
ENSG0000016370	-1.871932143	####	###	CRELD1	protein_coding	cysteine rich	3	9933816	9945406
ENSG0000013988	-0.843049866	####	###	CDH24	protein_coding	cadherin 24	14	23047062	23057538
ENSG0000013202	-1.405506663	####	###	CC2D1A	protein_coding	coiled-coil a	19	13906201	13930879
ENSG0000005325	-0.521911121	####	###	FOXN3	protein_coding	forkhead bo	14	89124871	89619149
ENSG0000010542	0.590790776	####	###	PTPRS	protein_coding	protein tyro	19	5158495	5340803
ENSG0000024450	-2.997753263	####	###	APOBEC3C	protein_coding	apolipoprot	22	39014257	39020352
ENSG0000011324	-1.048339736	####	###	CLK4	protein_coding	CDC like kin	5	178602664	178630615
ENSG0000013051	2.160895366	####	###	IQCN	protein_coding	IQ motif cor	19	18257097	18274509
ENSG0000010646	0.648946718	####	###	EZH2	protein_coding	enhancer of	7	148807383	148884321
ENSG0000015939	-0.641008031	####	###	HK2	protein_coding	hexokinase	2	74834127	74893359
ENSG0000000589	0.679903183	####	###	LAMP2	protein_coding	lysosomal a	X	120426148	120469365
ENSG0000014329	-0.611569889	####	###	PRCC	protein_coding	proline rich	1	156750610	156800815
ENSG0000014982	-0.876035566	####	###	VPS51	protein_coding	VPS51 subu	11	65089324	65111862
ENSG0000015172	0.785752151	####	###	ACSL1	protein_coding	acyl-CoA sy	4	184755595	184826818
ENSG0000014461	-1.991452985	####	###	CNTN4	protein_coding	contactin 4	3	2098813	3057959
ENSG0000013386	1.027504171	####	###	TEX15	protein_coding	testis expres	8	30831544	30913008
ENSG0000020638	-1.084813757	####	###	SNHG32	protein_coding	small nucle	CHR_HSCHR	31824855	31830009
ENSG0000013410	-1.969678087	####	###	BHLHE40	protein_coding	basic helix-l	3	4979437	4985323

ENSG0000009972	-2.315724992	####	###	PRKY	protein_codinc	protein kina	Y	7273972	7381548
ENSG0000018535	-0.848065002	####	###	HS6ST3	protein_codinc	heparan sul	13	96090839	96839562
ENSG0000011033	0.72509519	####	###	BIRC2	protein_codinc	baculoviral I	11	102347211	102378670
ENSG0000016867	-3.680382178	####	###	LRATD2	protein_codinc	LRAT doma	8	126552443	126558478
ENSG0000018155	-0.690253159	####	###	SETD2	protein_codinc	SET domain	3	47016429	47164113
ENSG0000027601	-1.684482983	####	###	ABR	protein_codinc	ABR activatc	CHR_HSCHR	1003519	1116206
ENSG0000017110	0.791546448	####	###	MFN1	protein_codinc	mitofusin 1	3	179347709	179394936
ENSG0000009501	-0.647128396	####	###	MAP3K1	protein_codinc	mitogen-ac	5	56815549	56896152
ENSG0000010288	-1.778256518	####	###	MAPK3	protein_codinc	mitogen-ac	16	30114105	30123506
ENSG0000012363	-0.782939563	####	###	BAZ2B	protein_codinc	bromodoma	2	159318979	159616569
ENSG0000018659	-0.562296173	####	###	UBE2H	protein_codinc	ubiquitin co	7	129830732	129952960
ENSG0000016546	0.649011221	####	###	PHOX2A	protein_codinc	paired like h	11	72239077	72245664
ENSG0000016240	-3.612037626	####	###	PLPP3	protein_codinc	phospholipi	1	56494761	56645301
ENSG0000024446	0.662420143	####	###	RBM12	protein_codinc	RNA bindin	20	35648925	35664956
ENSG0000010085	-0.570128755	####	###	ARHGAP5	protein_codinc	Rho GTPase	14	32076114	32159728
ENSG0000016779	-1.297669107	####	###	CDK2AP2	protein_codinc	cyclin deper	11	67506497	67508649
ENSG0000013105	-0.482210257	####	###	RBM39	protein_codinc	RNA bindin	20	35701347	35742312
ENSG0000013965	-0.86327712	####	###	ZNF740	protein_codinc	zinc finger p	12	53180704	53195142
ENSG0000008117	-1.249994104	####	###	EXD2	protein_codinc	exonuclease	14	69191511	69244020
ENSG0000016390	-0.602946392	####	###	RPN1	protein_codinc	ribophorin I	3	128619969	128681075
ENSG0000016465	2.099364094	####	###	ELAPOR2	protein_codinc	endosome-	7	86876906	87059654
ENSG0000015934	1.49821468	####	###	CYB5R1	protein_codinc	cytochrome	1	202961873	202967275
ENSG0000022546	-2.048809389	####	###	RFPL1S	lncRNA	RFPL1 antise	22	29436534	29478868
ENSG0000011237	-5.425219975	####	###	ARFGEF3	protein_codinc	ARFGEF fam	6	138161939	138344663
ENSG0000022930	-1.095154481	####	###	SNHG32	protein_codinc	small nuclec	CHR_HSCHR	31816794	31821951
ENSG0000006496	1.081725359	####	###	HMG20B	protein_codinc	high mobilit	19	3572777	3579088
ENSG0000012100	-0.902009288	####	###	CRISPLD1	protein_codinc	cysteine rich	8	74984505	75034558
ENSG0000010570	-0.721212583	####	###	FKBP8	protein_codinc	FKBP prolyl	19	18531751	18544077
ENSG0000019838	-0.633338233	####	###	GFPT1	protein_codinc	glutamine--	2	69319780	69387250
ENSG0000008727	0.595944509	####	###	ADD1	protein_codinc	adducin 1 [S	4	2843857	2930076
ENSG0000012400	-0.990644061	####	###	OBSL1	protein_codinc	obscurin like	2	219550728	219571859
ENSG0000017279	0.634419728	####	###	DCP2	protein_codinc	decapping r	5	112976702	113022195
ENSG0000013728	0.50607642	####	###	TUBB2B	protein_codinc	tubulin beta	6	3224277	3231730
ENSG0000009995	-0.91072732	####	###	CECR2	protein_codinc	CECR2 histc	22	17359949	17558149
ENSG0000022671	-1.096784275	####	###	SNHG32	protein_codinc	small nuclec	CHR_HSCHR	31821974	31827129
ENSG0000016411	-0.823063873	####	###	GUCY1A1	protein_codinc	guanylate c	4	155666726	155737059
ENSG0000016943	-0.644653199	####	###	SCN9A	protein_codinc	sodium volt	2	166195185	166376001
ENSG0000014294	0.669132748	####	###	KIF2C	protein_codinc	kinesin fami	1	44739818	44767767
ENSG0000011632	-2.168267628	####	###	OPRD1	protein_codinc	opioid recep	1	28812142	28871267
ENSG0000015697	0.611176075	####	###	BUB1B	protein_codinc	BUB1 mitoti	15	40161023	40221123
ENSG0000005765	-6.804926879	####	###	PRDM1	protein_codinc	PR/SET dom	6	105993463	106109939
ENSG0000011352	-0.593407517	####	###	RAD50	protein_codinc	RAD50 doul	5	132556019	132646349
ENSG0000020387	0.564317074	####	###	GDI1	protein_codinc	GDP dissoci	X	154436913	154443467
ENSG0000017131	0.940671821	####	###	CHST11	protein_codinc	carbohydrat	12	104455295	104762014
ENSG0000010621	0.832865463	####	###	HSPB1	protein_codinc	heat shock p	7	76302673	76304295
ENSG0000018416	-1.317982119	####	###	CRELD2	protein_codinc	cysteine rich	22	49918167	49927540
ENSG0000012910	0.641872702	####	###	SUMF2	protein_codinc	sulfatase mc	7	56064002	56080670
ENSG0000007817	-0.716997628	####	###	N4BP2	protein_codinc	NEDD4 binc	4	40056850	40158252
ENSG0000011977	-0.506453206	####	###	DNMT3A	protein_codinc	DNA methy	2	25227855	25342590
ENSG0000013757	0.53215969	####	###	SDCBP	protein_codinc	syndecan bi	8	58552924	58582859
ENSG0000013828	0.898703422	####	###	FAM149B1	protein_codinc	family with s	10	73168119	73244504
ENSG0000011972	-1.265561504	####	###	NRDE2	protein_codinc	NRDE-2, ne	14	90267860	90331969
ENSG0000023472	-1.093977897	####	###	SNHG32	protein_codinc	small nuclec	CHR_HSCHR	31901630	31906788
ENSG0000013268	-0.703372418	####	###	KHDC4	protein_codinc	KH domain	1	155913045	155934413
ENSG0000012870	0.972558006	####	###	HAT1	protein_codinc	histone acet	2	171922448	171983686
ENSG0000010714	-2.03779579	####	###	KCNT1	protein_codinc	potassium s	9	135702185	135795508
ENSG0000010141	1.498342744	####	###	PXMP4	protein_codinc	peroxisoma	20	33702758	33720319
ENSG0000018438	-1.295102971	####	###	MAML2	protein_codinc	mastermind	11	95976598	96343195

ENSG0000012412	1.008690386	####	###	TTPAL	protein_coding	alpha tocop	20	44475874	44494603
ENSG0000016524	-2.115859864	####	###	NLGN4Y	protein_coding	neuroligin 4 Y		14522573	14845650
ENSG0000012335	-1.589795899	####	###	NR4A1	protein_coding	nuclear rece	12	52022832	52059507
ENSG0000011589	1.769701021	####	###	PLCL1	protein_coding	phospholipa	2	197804593	198572581
ENSG0000016805	-1.161563886	####	###	LTBP3	protein_coding	latent transf	11	65538805	65558930
ENSG0000015114	0.895360067	####	###	UBE3B	protein_coding	ubiquitin pr	12	109477402	109536705
ENSG0000016978	-3.539120732	####	###	LINGO1	protein_coding	leucine rich	15	77613027	77820900
ENSG0000011076	-0.77478425	####	###	GTF2H1	protein_coding	general tran	11	18322295	18367045
ENSG0000011713	-0.543915336	####	###	KDM5B	protein_coding	lysine deme	1	202724495	202808487
ENSG0000011198	-1.808722951	####	###	ULBP1	protein_coding	UL16 bindin	6	149963943	149973715
ENSG0000006812	-0.787724121	####	###	COASY	protein_coding	Coenzyme A	17	42561467	42566277
ENSG0000014151	-0.900652022	####	###	TP53	protein_coding	tumor prote	17	7661779	7687538
ENSG0000019667	1.20193701	####	###	ERI2	protein_coding	ERI1 exoribo	16	20780193	20900349
ENSG0000013962	-1.686224124	####	###	GALNT6	protein_coding	polypeptide	12	51351247	51392867
ENSG0000011006	-0.739134875	####	###	KMT5B	protein_coding	lysine methy	11	68154863	68213828
ENSG0000016237	0.719985654	####	###	ZYG11B	protein_coding	zyg-11 fam	1	52726453	52827336
ENSG0000009220	0.446936806	####	###	SUPT16H	protein_coding	SPT16 homo	14	21351476	21384019
ENSG0000014094	0.579555994	####	###	MAP1LC3B	protein_coding	microtubule	16	87383953	87404779
ENSG0000018882	1.237675241	####	###	SLX4	protein_coding	SLX4 structu	16	3581181	3611606
ENSG0000010764	0.624094259	####	###	MAPK8	protein_coding	mitogen-ac	10	48306639	48439360
ENSG0000010779	-2.480641728	####	###	ACTA2	protein_coding	actin alpha 2	10	88935074	88991339
ENSG0000008784	-1.159781973	####	###	PIR	protein_coding	pirin [Sourc X		15384799	15493564
ENSG0000016359	-0.532620364	####	###	SNHG16	lncRNA	small nucleo	17	76557764	76565348
ENSG0000014156	1.284361101	####	###	FN3KRP	protein_coding	fructosamin	17	82716706	82730328
ENSG0000010205	0.623590894	####	###	RBBP7	protein_coding	RB binding p X		16839283	16870362
ENSG0000011364	0.483714267	####	###	MACROH2A	protein_coding	macroH2A.1	5	135333900	135399914
ENSG0000010306	1.907164797	####	###	PLA2G15	protein_coding	phospholipa	16	68245304	68261058
ENSG0000014381	1.30061608	####	###	EPHX1	protein_coding	epoxide hyd	1	225810092	225845563
ENSG0000013271	0.440424344	####	###	SYT11	protein_coding	synaptotagr	1	155859567	155885199
ENSG0000010586	1.131699261	####	###	SP4	protein_coding	Sp4 transcri	7	21428043	21514822
ENSG0000011675	-0.854063987	####	###	BCAS2	protein_coding	BCAS2 pre-	1	114567557	114581629
ENSG0000017769	0.939700531	####	###	CD151	protein_coding	CD151 mole	11	832887	839831
ENSG0000026983	1.835701301	####	###	IPO5P1	transcribed_pr	importin 5 p	19	23255053	23257939
ENSG0000011344	-0.725056867	####	###	LNPEP	protein_coding	leucyl and c	5	96935394	97037513
ENSG0000011939	0.88777277	####	###	CNTRL	protein_coding	centriolin [S	9	121074863	121177610
ENSG0000005272	0.651888924	####	###	SIKE1	protein_coding	suppressor o	1	114769479	114780685
ENSG0000016310	-0.551197947	####	###	SMARCAD1	protein_coding	SWI/SNF-re	4	94207611	94291292
ENSG0000008671	-0.842343298	####	###	TXLNG	protein_coding	taxilin gamr X		16786432	16844519
ENSG0000013415	0.933276959	####	###	KATNBL1	protein_coding	katanin regu	15	34140674	34210096
ENSG0000020438	-1.062098154	####	###	SNHG32	lncRNA	small nucleo	6	31834608	31839766
ENSG0000010733	-1.077093309	####	###	ABCA2	protein_coding	ATP binding	9	137007234	137028922
ENSG0000015299	0.618961763	####	###	ADGRA3	protein_coding	adhesion G	4	22345071	22516066
ENSG0000016786	-2.740902298	####	###	HID1	protein_coding	HID1 domai	17	74950742	74973166
ENSG0000015465	-2.769224053	####	###	L3MBTL4	protein_coding	L3MBTL hist	18	5954706	6415237
ENSG0000014394	-0.468424112	####	###	RPS27A	protein_coding	ribosomal p	2	55231903	55235853
ENSG0000022566	0.908556735	####	###	MCRIP1	protein_coding	MAPK regul	17	81822361	81833302
ENSG0000011071	-1.128517814	####	###	AIP	protein_coding	aryl hydroca	11	67483026	67491103
ENSG0000026941	1.704697851	####	###	LINC01224	lncRNA	long interge	19	23399233	23416075
ENSG0000014344	-0.534634952	####	###	POGZ	protein_coding	pogo transp	1	151402724	151459494
ENSG0000013744	-1.414313176	####	###	CPEB2	protein_coding	cytoplasmic	4	15002674	15070153
ENSG0000007441	-2.498248977	####	###	CA12	protein_coding	carbonic an	15	63321378	63381846
ENSG0000010003	0.602211558	####	###	MAPK1	protein_coding	mitogen-ac	22	21759657	21867680
ENSG0000024448	1.269985293	####	###	SCARF2	protein_coding	scavenger re	22	20424584	20437826
ENSG0000014152	0.509431086	####	###	ARHGDIA	protein_coding	Rho GDP dis	17	81867721	81871378
ENSG0000021475	0.448973108	####	###	HNRNPUL2	protein_coding	heterogene	11	62712630	62727457
ENSG0000010466	-0.603930563	####	###	LEPROTL1	protein_coding	leptin recep	8	30095408	30177208
ENSG0000013453	-2.900211889	####	###	SOX5	protein_coding	SRY-box tra	12	23529504	24562544
ENSG0000010560	1.086978585	####	###	GCDH	protein_coding	glutaryl-Co/	19	12891160	12914207

ENSG0000011336	0.869973572	####	###	ARRDC3	protein_coding	arrestin don	5	91368631	91383317
ENSG0000018493	0.866081692	####	###	ZFP90	protein_coding	ZFP90 zinc f	16	68530090	68576072
ENSG0000013435	-0.494347276	####	###	IL6ST	protein_coding	interleukin 6	5	55935095	55995022
ENSG0000000933	0.520069072	####	###	UBE3C	protein_coding	ubiquitin pr	7	157138916	157269370
ENSG0000013242	-1.338964096	####	###	POPDC3	protein_coding	popeye don	6	105157900	105180014
ENSG0000013599	-0.918589737	####	###	EPC2	protein_coding	enhancer of	2	148644440	148787569
ENSG0000011023	0.84766611	####	###	ARHGEF17	protein_coding	Rho guanine	11	73308276	73369388
ENSG0000011667	1.487927239	####	###	MAD2L2	protein_coding	mitotic arres	1	11674480	11691650
ENSG0000013095	1.334485383	####	###	HABP4	protein_coding	hyaluronan	9	96450169	96491336
ENSG0000013529	-1.160394556	####	###	MTO1	protein_coding	mitochondr	6	73461578	73509236
ENSG0000018372	0.818610761	####	###	TMEM50A	protein_coding	transmembr	1	25338317	25362361
ENSG0000013543	-3.05245606	####	###	AGAP2	protein_coding	ArfGAP with	12	57723761	57742157
ENSG0000002579	-0.498363496	####	###	SEC63	protein_coding	SEC63 hom	6	107867756	107958208
ENSG0000015047	-2.373781324	####	###	ADGRL3	protein_coding	adhesion G	4	61201258	62078335
ENSG0000004759	-1.870974614	####	###	XK	protein_coding	X-linked Kx	X	37685791	37732130
ENSG0000015189	-5.460832477	####	###	GFRA1	protein_coding	GDNF family	10	116056925	116273467
ENSG0000027382	-3.998147741	####	###	FP700111.2	unprocessed_p	otopetrin 1	1	144754572	144786606
ENSG0000016692	-0.666109569	####	###	GREM1	protein_coding	gremlin 1, D	15	32718004	32745106
ENSG0000018302	-0.855814442	####	###	AP2A2	protein_coding	adaptor rela	11	924894	1012245
ENSG0000017229	0.466237819	####	###	CERS6	protein_coding	ceramide sy	2	168456249	168775134
ENSG0000006708	0.634596038	####	###	KLF6	protein_coding	Kruppel like	10	3775996	3785281
ENSG0000017002	0.387996617	####	###	YWHAG	protein_coding	tyrosine 3-r	7	76326799	76358991
ENSG0000017566	1.176385637	####	###	TOM1L2	protein_coding	target of my	17	17843511	17972422
ENSG0000011546	-0.526243102	####	###	USP34	protein_coding	ubiquitin sp	2	61187463	61471087
ENSG0000011606	0.656026748	####	###	MSH6	protein_coding	mutS homo	2	47695530	47810063
ENSG0000011623	0.665456694	####	###	ICMT	protein_coding	isoprenylcys	1	6221193	6235972
ENSG0000017354	0.667645759	####	###	MOB1B	protein_coding	MOB kinase	4	70902326	71022449
ENSG0000011590	-0.479211782	####	###	SOS1	protein_coding	SOS Ras/Ra	2	38981549	39124345
ENSG0000013469	0.840896979	####	###	CDCA8	protein_coding	cell division	1	37692481	37709719
ENSG0000011576	0.624481011	####	###	PLEKHB2	protein_coding	pleckstrin ho	2	131104847	131353709
ENSG0000004793	0.596482335	####	###	GOPC	protein_coding	golgi associ	6	117560269	117602542
ENSG0000000612	0.463739368	####	###	AP2B1	protein_coding	adaptor rela	17	35578046	35726413
ENSG0000010572	-0.950051843	####	###	ERF	protein_coding	ETS2 repres	19	42247569	42255128
ENSG0000012364	1.199103458	####	###	SLC36A1	protein_coding	solute carrie	5	151437046	151492379
ENSG0000015207	1.357636169	####	###	CCDC74B	protein_coding	coiled-coil c	2	130139287	130145134
ENSG0000011939	0.926856623	####	###	GLE1	protein_coding	GLE1 RNA e	9	128504719	128542288
ENSG0000015086	0.761539588	####	###	PIP4K2A	protein_coding	phosphatidy	10	22534854	22714578
ENSG0000017075	0.398257598	####	###	KIF5B	protein_coding	kinesin fami	10	32009015	32056425
ENSG0000015708	-2.292275443	####	###	ATP2B2	protein_coding	ATPase plas	3	10324023	10708007
ENSG0000012061	-0.781644042	####	###	EPC1	protein_coding	enhancer of	10	32267751	32378798
ENSG0000012270	1.307106784	####	###	RECK	protein_coding	reversion in	9	36036913	36124455
ENSG0000016494	0.856378769	####	###	INTS8	protein_coding	integrator c	8	94813311	94881746
ENSG0000019693	0.821340341	####	###	FAM3C	protein_coding	FAM3 meta	7	121348878	121396364
ENSG0000015455	-3.039150814	####	###	SORBS2	protein_coding	sorbin and S	4	185585444	185956652
ENSG0000015106	-2.739990118	####	###	CACNA1C	protein_coding	calcium volt	12	1970786	2697950
ENSG0000011166	1.094111736	####	###	CDCA3	protein_coding	cell division	12	6844793	6852066
ENSG0000018237	-1.748507136	####	###	PLCXD1	protein_coding	phosphatidy	X	276322	303356
ENSG0000014481	-0.685898106	####	###	NXPE3	protein_coding	neurexophil	3	101779202	101828231
ENSG0000018907	-0.661095929	####	###	ARID2	protein_coding	AT-rich inte	12	45729706	45908040
ENSG0000016813	-0.66357984	####	###	SETD5	protein_coding	SET domain	3	9397615	9479240
ENSG0000027845	-1.083637336	####	###	KANSL1	protein_coding	KAT8 regula	CHR_HSCHR	46041525	46237041
ENSG0000017677	-4.012097643	####	###	NCKAP5	protein_coding	NCK associa	2	132671788	133568463
ENSG0000010786	0.668744323	####	###	GBF1	protein_coding	golgi brefeld	10	102245532	102382899
ENSG0000007840	-0.774222791	####	###	MLLT10	protein_coding	MLLT10 hist	10	21524646	21743630
ENSG0000016849	0.719125376	####	###	FEN1	protein_coding	flap structur	11	61792911	61797238
ENSG0000016602	0.439285083	####	###	AMOTL1	protein_coding	angiomotin	11	94706431	94876748
ENSG0000013256	-1.546583677	####	###	REEP2	protein_coding	receptor acc	5	138439057	138446969
ENSG0000020476	1.681378357	####	###	RANBP17	protein_coding	RAN binding	5	170861870	171300015

ENSG0000016298	-4.861845608	####	###	KCNJ3	protein_coding	potassium in	2	154697855	154858354
ENSG0000010677	0.661808329	####	###	TMEM245	protein_coding	transmembr	9	109015135	109119947
ENSG0000016080	-0.752862877	####	###	UBQLN4	protein_coding	ubiquilin 4 [	1	156035299	156053798
ENSG0000014929	1.780691238	####	###	TTC12	protein_coding	tetratricope	11	113314579	113383544
ENSG0000008543	0.844092242	####	###	WDR47	protein_coding	WD repeat c	1	108970214	109042113
ENSG0000017601	-0.89298023	####	###	LYSMD3	protein_coding	LysM doma	5	90515611	90529584
ENSG0000027422	-0.747483309	####	###	SOCS7	protein_coding	suppressor c	CHR_HSCHR	38152644	38206380
ENSG0000013776	-1.180399775	####	###	ALKBH8	protein_coding	alkB homolo	11	107502727	107565742
ENSG0000013428	0.592917935	####	###	ARF3	protein_coding	ADP ribosyl	12	48935723	48957487
ENSG0000027688	-0.685673057	####	###	GREM1	protein_coding	gremlin 1, D	CHR_HSCHR	32798311	32814964
ENSG0000007008	-1.114920735	####	###	NUCB2	protein_coding	nucleobindi	11	17208153	17349980
ENSG0000008924	-0.689377735	####	###	ERP29	protein_coding	endoplasmi	12	112013348	112023449
ENSG0000027707	2.095923683	####	###	STAG3L2	transcribed_un	stromal anti	7	74843754	74890610
ENSG0000018491	-2.72882532	####	###	JAG2	protein_coding	jagged canc	14	105140982	105168824
ENSG0000018400	0.346833807	####	###	ACTG1	protein_coding	actin gamm	17	81509971	81523847
ENSG0000016346	-1.451836095	####	###	TRIM46	protein_coding	tripartite mo	1	155173787	155184971
ENSG0000019657	0.629318897	####	###	PLXNB2	protein_coding	plexin B2 [S	22	50274979	50307646
ENSG0000017465	0.92824549	####	###	ZNF266	protein_coding	zinc finger p	19	9412461	9435571
ENSG0000001329	-0.805025553	####	###	SLC7A14	protein_coding	solute carrie	3	170459548	170586075
ENSG0000010449	-1.538085493	####	###	SNX16	protein_coding	sorting nexi	8	81799581	81842866
ENSG0000015423	-3.120544581	####	###	LRRK1	protein_coding	leucine rich	15	100919327	101078257
ENSG0000017289	1.208096733	####	###	NADSYN1	protein_coding	NAD synthe	11	71453109	71524107
ENSG0000010320	-0.9352437	####	###	NME4	protein_coding	NME/NM23	16	396725	410367
ENSG0000005279	-0.81015379	####	###	FNIP2	protein_coding	folliculin int	4	158769026	158908050
ENSG0000003549	0.930403236	####	###	DEPDC1B	protein_coding	DEP domair	5	60596912	60700190
ENSG0000009086	0.595028698	####	###	GLG1	protein_coding	golgi glycop	16	74447427	74607144
ENSG0000021578	1.348689995	####	###	FAM72D	protein_coding	family with s	1	145095974	145112696
ENSG0000013149	0.966177962	####	###	NDUFA2	protein_coding	NADH:ubiqu	5	140638740	140647771
ENSG0000018178	-0.960562706	####	###	SIAH2	protein_coding	siah E3 ubic	3	150741125	150763477
ENSG0000019846	1.3358049	####	###	ZNF480	protein_coding	zinc finger p	19	52297169	52325922
ENSG0000001330	0.658780736	####	###	SLC25A39	protein_coding	solute carrie	17	44319625	44324870
ENSG0000018022	0.741681507	####	###	PRKRA	protein_coding	protein activ	2	178431414	178451512
ENSG0000014519	-1.534148331	####	###	ECE2	protein_coding	endothelin c	3	184276011	184293031
ENSG0000012919	1.009981501	####	###	PIMREG	protein_coding	PICALM inte	17	6444441	6451469
ENSG0000016834	0.423930114	####	###	INSM2	protein_coding	INSM transc	14	35534164	35537054
ENSG0000018807	-0.845192335	####	###	C11orf95	protein_coding	chromosom	11	63759892	63768775
ENSG0000023977	0.983603709	####	###	WBP1	protein_coding	WW domair	2	74458400	74460891
ENSG0000015146	1.20026806	####	###	CCDC3	protein_coding	coiled-coil c	10	12896625	13099652
ENSG0000017358	-3.18905521	####	###	CCDC106	protein_coding	coiled-coil c	19	55641062	55653161
ENSG0000026908	-3.757966324	####	###	AC008555.2	lncRNA	novel transc	19	34837889	34872479
ENSG0000013721	1.066630316	####	###	TMEM14B	protein_coding	transmembr	6	10747759	10852753
ENSG0000003656	-5.936523508	####	###	SLC18A1	protein_coding	solute carrie	8	20144855	20183206
ENSG0000011358	0.799054172	####	###	C5orf15	protein_coding	chromosom	5	133955510	133968674
ENSG0000018543	0.761932887	####	###	METTL7A	protein_coding	methyltrans	12	50923472	50932510
ENSG0000010476	-2.305371064	####	###	FGL1	protein_coding	fibrinogen li	8	17864380	17910365
ENSG0000017001	-2.704875032	####	###	MYRIP	protein_coding	myosin VIIA	3	39808914	40260321
ENSG0000024676	-3.178506959	####	###	RGMB-AS1	lncRNA	RGMB antis	5	98769618	98773469
ENSG0000016440	0.793421764	####	###	UQCRQ	protein_coding	ubiquinol-c	5	132866630	132868847
ENSG0000007571	-0.80254825	####	###	DLG1	protein_coding	discs large M	3	197042560	197299330
ENSG0000011485	-0.486560586	####	###	SSR3	protein_coding	signal seque	3	156539553	156555149
ENSG0000025623	-2.607426277	####	###	SMIM3	protein_coding	small integr	5	150777946	150796734
ENSG0000011327	1.187489666	####	###	ARSB	protein_coding	arylsulfatase	5	78777209	78986087
ENSG0000015978	0.986928667	####	###	RGS12	protein_coding	regulator of	4	3293028	3439913
ENSG0000014736	-0.876398928	####	###	FBXO25	protein_coding	F-box prote	8	406428	477967
ENSG0000012449	-1.553847737	####	###	TRERF1	protein_coding	transcriptior	6	42224931	42452051
ENSG0000014332	-0.724451729	####	###	ABL2	protein_coding	ABL proto-c	1	179099330	179229684
ENSG0000017077	0.961535094	####	###	CDCA4	protein_coding	cell division	14	105009573	105021083
ENSG0000015447	0.483358858	####	###	BUB3	protein_coding	BUB3 mitoti	10	123154402	123170467

ENSG0000018274	-1.831945769	####	###	PAQR7	protein_coding	progestin ar	1	25861484	25875708
ENSG0000018378	-1.972058701	####	###	SLC35F3	protein_coding	solute carrie	1	233904676	234324511
ENSG0000008354	-1.378349378	####	###	TDRD3	protein_coding	tudor doma	13	60396457	60573878
ENSG0000010979	-0.810110716	####	###	KLHL5	protein_coding	kelch like fa	4	39045039	39126857
ENSG0000016955	-1.010133194	####	###	ZEB2	protein_coding	zinc finger E	2	144364364	144521057
ENSG0000006724	0.694680062	####	###	DHX29	protein_coding	DExH-box h	5	55256055	55307694
ENSG0000024169	0.709221919	####	###	TMEFF1	protein_coding	transmembr	9	100473149	100577636
ENSG0000015149	1.10699989	####	###	ACAD8	protein_coding	acyl-CoA de	11	134253548	134265855
ENSG0000010440	-0.437025549	####	###	EIF3E	protein_coding	eukaryotic t	8	108201216	108435333
ENSG0000018784	-0.621068503	####	###	EIF4EBP1	protein_coding	eukaryotic t	8	38030534	38060365
ENSG0000010661	-1.310258043	####	###	PRKAG2	protein_coding	protein kina	7	151556124	151877125
ENSG0000018687	1.309195137	####	###	ERCC6L	protein_coding	ERCC excisio	X	72204657	72239027
ENSG0000015183	-0.50994533	####	###	SACS	protein_coding	sacsin mole	13	23328826	23433740
ENSG0000023119	-5.225087175	####	###	NA	NA	NA	NA	NA	NA
ENSG0000008469	-1.004955124	####	###	AGBL5	protein_coding	ATP/GTP bi	2	27042364	27070622
ENSG0000019875	0.540348806	####	###	CDC42BPB	protein_coding	CDC42 binc	14	102932380	103057549
ENSG0000015067	-2.82919165	####	###	DLG2	protein_coding	discs large M	11	83455012	85627922
ENSG0000018116	-0.354200892	####	###	NPM1	protein_coding	nucleophos	5	171387116	171411137
ENSG0000000930	0.401332448	####	###	CSDE1	protein_coding	cold shock d	1	114716913	114758676
ENSG0000010222	0.641281575	####	###	USP11	protein_coding	ubiquitin sp	X	47232866	47248328
ENSG0000000675	1.06338115	####	###	ARSD	protein_coding	arylsulfatase	X	2903972	2929349
ENSG0000017250	3.209786522	####	###	CARNS1	protein_coding	carnosine sy	11	67414968	67425607
ENSG0000015650	1.114434909	####	###	FAM122B	protein_coding	family with s	X	134769566	134797232
ENSG0000013707	0.741766343	####	###	UBAP2	protein_coding	ubiquitin as	9	33921693	34048949
ENSG0000010561	-1.401094575	####	###	DNASE2	protein_coding	deoxyribonu	19	12875209	12881466
ENSG0000010354	-1.003139134	####	###	VPS35L	protein_coding	VPS35 endo	16	19555240	19706793
ENSG0000010473	0.490770707	####	###	MCM4	protein_coding	minichromo	8	47960185	47978160
ENSG0000006865	-1.139865948	####	###	ATP11A	protein_coding	ATPase pho	13	112690329	112887168
ENSG0000009938	-0.843885855	####	###	SETD1A	protein_coding	SET domain	16	30957754	30984664
ENSG0000010236	-1.794537251	####	###	SYTL4	protein_coding	synaptotagr	X	100674491	100732123
ENSG0000008599	1.332269623	####	###	RAD54L	protein_coding	RAD54 like	1	46246461	46278480
ENSG0000005793	-0.743107185	####	###	MTA3	protein_coding	metastasis a	2	42494569	42756947
ENSG0000016815	-0.590187989	####	###	RNF187	protein_coding	ring finger p	1	228487382	228496188
ENSG0000016301	-1.591240513	####	###	FBXO41	protein_coding	F-box prote	2	73254682	73284431
ENSG0000010140	0.886858571	####	###	TTI1	protein_coding	TELO2 inter	20	37983007	38033461
ENSG0000023628	-3.518767225	####	###	GACAT3	lncRNA	gastric canc	2	16013928	16087201
ENSG0000010774	-2.385921001	####	###	SPOCK2	protein_coding	SPARC (oste	10	72059034	72089032
ENSG0000026351	1.342316942	####	###	FAM72C	protein_coding	family with s	1	143955364	143971965
ENSG0000017186	-0.674231184	####	###	PRNP	protein_coding	prion protei	20	4686350	4701590
ENSG0000021334	1.634232853	####	###	MXD3	protein_coding	MAX dimeri	5	177301461	177312757
ENSG0000017435	1.734709472	####	###	STAG3L3	transcribed_un	stromal anti	7	72969696	73005922
ENSG0000017587	-1.431109686	####	###	HOXD8	protein_coding	homeobox D	2	176129694	176132695
ENSG0000016372	0.76452033	####	###	TTC14	protein_coding	tetratricope	3	180602163	180617828
ENSG0000026300	-0.473560319	####	###	GTF2I	protein_coding	general trar	7	74650231	74760692
ENSG0000013096	1.313009446	####	###	PRRG1	protein_coding	proline rich	X	37349309	37457292
ENSG0000015493	-3.030795674	####	###	ACSS1	protein_coding	acyl-CoA sy	20	25006230	25058980
ENSG0000016317	-0.407324358	####	###	CDC42EP3	protein_coding	CDC42 effe	2	37641882	37738468
ENSG0000014354	-0.713765375	####	###	JTB	protein_coding	jumping tra	1	153974269	153977674
ENSG0000014374	0.41865461	####	###	SRP9	protein_coding	signal recog	1	225777813	225790468
ENSG0000016475	0.389445819	####	###	RAD21	protein_coding	RAD21 cohe	8	116845934	116874776
ENSG0000021539	-2.861727363	####	###	SCRT2	protein_coding	scratch fami	20	661596	675802
ENSG0000010136	-0.883872055	####	###	IDH3B	protein_coding	isocitrate de	20	2658395	2664219
ENSG0000010851	-2.576732969	####	###	ENO3	protein_coding	enolase 3 [S	17	4948092	4957131
ENSG0000011485	0.666093733	####	###	NKTR	protein_coding	natural killer	3	42600655	42648735
ENSG0000012707	-10.0485889	####	###	RGS13	protein_coding	regulator of	1	192636138	192660306
ENSG0000011963	0.562410737	####	###	NEK9	protein_coding	NIMA relate	14	75079353	75127344
ENSG0000010886	-0.950743222	####	###	DUSP3	protein_coding	dual specific	17	43766125	43778977
ENSG0000020570	1.394796017	####	###	ETFRF1	protein_coding	electron trai	12	25195216	25209645

ENSG0000015418	-5.911348431	####	###	ANGPT1	protein_codinc	angiopoietir	8	107249482	107498055
ENSG0000014093	-2.199615897	####	###	CDH11	protein_codinc	cadherin 11	16	64943753	65126112
ENSG0000026971	-0.937034571	####	###	NBPF9	protein_codinc	NBPF memb	1	149054027	149103561
ENSG0000014377	-0.537075546	####	###	CDC42BPA	protein_codinc	CDC42 binc	1	226989865	227318474
ENSG0000014574	-0.418455075	####	###	BTF3	protein_codinc	basic transc	5	73498408	73505635
ENSG0000027576	-3.96668193	####	###	AC244015.2	unprocessed_p	otopetrin 1	1	144179777	144211729
ENSG0000011759	0.776011716	####	###	DARS2	protein_codinc	aspartyl-tRN	1	173824653	173858808
ENSG0000012105	-0.79459017	####	###	AKAP1	protein_codinc	A-kinase an	17	57085092	57121346
ENSG0000027421	-0.751432671	####	###	SOCS7	protein_codinc	suppressor c	17	38351844	38405593
ENSG0000001567	0.755143753	####	###	NUDCD3	protein_codinc	NudC doma	7	44379119	44490658
ENSG0000010043	-4.010469095	####	###	KCNK10	protein_codinc	potassium t	14	88180103	88326907
ENSG0000015916	1.874052882	####	###	STC1	protein_codinc	stanniocalci	8	23841929	23854806
ENSG0000015611	-3.956447376	####	###	KCNMA1	protein_codinc	potassium c	10	76869601	77638369
ENSG0000016498	0.459610294	####	###	PSIP1	protein_codinc	PC4 and SFI	9	15464066	15510995
ENSG0000010846	0.667337265	####	###	CDK5RAP3	protein_codinc	CDK5 regula	17	47967810	47981781
ENSG0000014786	0.487413809	####	###	NFIB	protein_codinc	nuclear fact	9	14081843	14398983
ENSG0000016647	-0.749708308	####	###	TMEM41B	protein_codinc	transmembr	11	9280654	9314636
ENSG0000025731	-1.014676478	####	###	ZBED6	protein_codinc	zinc finger E	1	203795714	203854999
ENSG0000012526	-1.145582803	####	###	EFNB2	protein_codinc	ephrin B2 [S	13	106489745	106535662
ENSG0000011615	-1.335105405	####	###	GPX7	protein_codinc	glutathione	1	52602371	52609051
ENSG0000015174	-0.703685879	####	###	BICD1	protein_codinc	BICD cargo	12	32106835	32383633
ENSG0000008573	0.479929602	####	###	CTTN	protein_codinc	cortactin [Sc	11	70398404	70436584
ENSG0000012741	2.420231202	####	###	FGFRL1	protein_codinc	fibroblast gr	4	1009936	1026898
ENSG0000010318	1.291821146	####	###	COTL1	protein_codinc	coactosin lik	16	84565596	84618078
ENSG0000011010	-0.854608994	####	###	CCDC86	protein_codinc	coiled-coil c	11	60842113	60851081
ENSG0000008409	0.490203947	####	###	STARD7	protein_codinc	StAR relatec	2	96184859	96208825
ENSG0000016433	-1.007791991	####	###	EBF1	protein_codinc	EBF transcri	5	158695916	159099916
ENSG0000026157	-2.099685291	####	###	AP003119.3	lncRNA	novel transc	11	76800364	76804555
ENSG0000019617	1.625495308	####	###	ZNF681	protein_codinc	zinc finger p	19	23739195	23758891
ENSG0000011042	0.934261648	####	###	FBXO3	protein_codinc	F-box prote	11	33740939	33774543
ENSG0000011947	1.048404527	####	###	HSDL2	protein_codinc	hydroxyster	9	112380080	112472405
ENSG0000011167	-0.813521494	####	###	ENO2	protein_codinc	enolase 2 [S	12	6913745	6923698
ENSG0000019655	1.329603985	####	###	FAM72A	protein_codinc	family with s	1	206186179	206204414
ENSG0000011123	1.112024391	####	###	GPN3	protein_codinc	GPN-loop G	12	110452486	110469268
ENSG0000010119	0.719911859	####	###	ARFGAP1	protein_codinc	ADP ribosyl	20	63272785	63289790
ENSG0000016573	0.553958792	####	###	BMS1	protein_codinc	BMS1 ribosc	10	42782795	42834937
ENSG0000019889	0.287201162	####	###	MT-ATP6	protein_codinc	mitochondr	MT	8527	9207
ENSG0000018686	-1.091214395	####	###	MAPT	protein_codinc	microtubule	17	45894551	46028334
ENSG0000017399	1.457999395	####	###	CCS	protein_codinc	copper chap	11	66593153	66606019
ENSG0000014099	0.642877522	####	###	PDPK1	protein_codinc	3-phosphoi	16	2537979	2603188
ENSG0000011772	0.362318571	####	###	CENPF	protein_codinc	centromere	1	214603195	214664571
ENSG0000010114	-2.256545254	####	###	BMP7	protein_codinc	bone morph	20	57168753	57266641
ENSG0000010221	1.080886212	####	###	RP2	protein_codinc	RP2 activatc	X	46837043	46882358
ENSG0000012437	1.708539291	####	###	PAIP2B	protein_codinc	poly(A) binc	2	71182738	71227103
ENSG0000014345	-1.117403174	####	###	GOLPH3L	protein_codinc	golgi phosp	1	150646230	150697154
ENSG0000016302	0.888791947	####	###	WDCP	protein_codinc	WD repeat a	2	24029347	24049575
ENSG0000011274	0.726826609	####	###	TTK	protein_codinc	TTK protein	6	80003887	80042527
ENSG0000012929	0.595602146	####	###	PHF20L1	protein_codinc	PHD finger	8	132775358	132848807
ENSG0000016530	0.741922759	####	###	MELK	protein_codinc	maternal en	9	36572862	36677683
ENSG0000017363	-1.483715128	####	###	SLC19A1	protein_codinc	solute carrie	21	45493572	45573365
ENSG0000019646	-0.982893049	####	###	MYL6B	protein_codinc	myosin light	12	56152256	56159647
ENSG0000017689	0.955877343	####	###	TYMS	protein_codinc	thymidylate	18	657653	673578
ENSG0000015992	1.078752634	####	###	GNE	protein_codinc	glucosamine	9	36214441	36277056
ENSG0000011269	0.660247015	####	###	COX7A2	protein_codinc	cytochrome	6	75237675	75250323
ENSG0000011565	0.82608992	####	###	UXS1	protein_codinc	UDP-glucur	2	106093308	106194301
ENSG0000016509	0.952890636	####	###	KDM1B	protein_codinc	lysine deme	6	18155329	18223854
ENSG0000018387	2.343312101	####	###	SCN5A	protein_codinc	sodium volt	3	38548057	38649687
ENSG0000022649	-1.000911144	####	###	CUTA	protein_codinc	cutA divaler	CHR_HSCHR	33556632	33558507

ENSG0000006419	1.47919523	####	###	SPA17	protein_coding	sperm autoa	11	124673844	124697518
ENSG0000019692	1.03536437	####	###	PDLIM7	protein_coding	PDZ and LIM	5	177483394	177497606
ENSG0000016799	0.326398899	####	###	FTH1	protein_coding	ferritin heav	11	61959718	61967634
ENSG0000011164	-0.81666804	####	###	NOP2	protein_coding	NOP2 nucle	12	6556863	6568691
ENSG0000015442	0.732974303	####	###	CCSAP	protein_coding	centriole, cil	1	229321011	229343294
ENSG0000016071	-0.614913161	####	###	UBE2Q1	protein_coding	ubiquitin co	1	154548577	154559028
ENSG0000023242	0.504861227	####	###	TUBB	protein_coding	tubulin beta	CHR_HSCHR	30712452	30717677
ENSG0000011671	0.912870771	####	###	GADD45A	protein_coding	growth arre	1	67685201	67688334
ENSG0000018192	0.909429762	####	###	PRKAG1	protein_coding	protein kina	12	49002274	49018807
ENSG0000013072	0.517377216	####	###	TRIM28	protein_coding	tripartite mo	19	58544064	58550722
ENSG0000018601	-1.295235761	####	###	ZNF566	protein_coding	zinc finger p	19	36445119	36489902
ENSG0000016492	-2.150004307	####	###	BAALC	protein_coding	BAALC bind	8	103140713	103230305
ENSG0000013297	-0.919578911	####	###	WASF3	protein_coding	WASP family	13	26557683	26688948
ENSG0000016523	-1.556208061	####	###	WNK2	protein_coding	WNK lysine	9	93184916	93320572
ENSG0000015718	1.306125454	####	###	CPT2	protein_coding	carnitine pa	1	53196792	53214197
ENSG0000019727	1.069486469	####	###	RAD54B	protein_coding	RAD54 hom	8	94371960	94475115
ENSG0000016645	0.95631737	####	###	CENPN	protein_coding	centromere	16	81006498	81033114
ENSG0000000827	0.479086186	####	###	ADAM22	protein_coding	ADAM meta	7	87934143	88202889
ENSG0000016807	-1.232856773	####	###	SCARA3	protein_coding	scavenger re	8	27633868	27676776
ENSG0000010002	0.879526727	####	###	PPIL2	protein_coding	peptidylprol	22	21666009	21700015
ENSG0000014268	0.912400841	####	###	KIAA0319L	protein_coding	KIAA0319 lil	1	35433492	35557950
ENSG0000010098	1.000825585	####	###	GSS	protein_coding	glutathione	20	34928432	34956027
ENSG0000006943	-2.756399556	####	###	ABCC9	protein_coding	ATP binding	12	21797401	21942529
ENSG0000012419	-1.357147048	####	###	GDAP1L1	protein_coding	ganglioside	20	44247099	44280947
ENSG0000012697	1.375376993	####	###	ZC4H2	protein_coding	zinc finger C	X	64915802	65034713
ENSG0000011344	-2.282579173	####	###	PDE4D	protein_coding	phosphodie	5	58969038	60522120
ENSG0000024849	-2.114636955	####	###	ASNSP1	transcribed_un	asparagine s	8	46579213	46638886
ENSG0000016061	0.800096303	####	###	PCSK7	protein_coding	proprotein c	11	117204337	117232525
ENSG0000024475	-0.64459608	####	###	N4BP2L2	protein_coding	NEDD4 binc	13	32432417	32538885
ENSG0000017437	0.796469326	####	###	EXO1	protein_coding	exonuclease	1	241847967	241895148
ENSG0000006532	0.804115562	####	###	MCM10	protein_coding	minichromo	10	13161554	13211104
ENSG0000014740	1.17545462	####	###	CETN2	protein_coding	centrin 2 [Sc	X	152826994	152830757
ENSG0000015492	1.305588613	####	###	EME1	protein_coding	essential me	17	50373220	50381483
ENSG0000015707	-0.888187349	####	###	ZFYVE9	protein_coding	zinc finger F	1	52142094	52346686
ENSG0000011992	-0.557386822	####	###	GPAM	protein_coding	glycerol-3-p	10	112149865	112215377
ENSG0000013671	-0.715579527	####	###	BIN1	protein_coding	bridging int	2	127048027	127107288
ENSG0000010740	-0.931240523	####	###	DVL1	protein_coding	dishevelled	1	1335276	1349418
ENSG0000022847	0.687948846	####	###	OST4	protein_coding	oligosaccha	2	27070472	27071654
ENSG0000027018	-13.67230221	####	###	BIVM-ERCC	protein_coding	BIVM-ERCC	13	102799110	102875994
ENSG0000027874	-1.579821135	####	###	ABR	protein_coding	ABR activato	CHR_HSCHR	1003519	1192547
ENSG0000017892	1.123625727	####	###	CYBC1	protein_coding	cytochrome	17	82442586	82450829
ENSG0000014045	1.165987805	####	###	PIF1	protein_coding	PIF1 5'-to-3	15	64815632	64825668
ENSG0000009980	-0.771550867	####	###	TIMM13	protein_coding	translocase	19	2425625	2427586
ENSG0000025571	-0.493853385	####	###	SNHG1	lncRNA	small nucleo	11	62851984	62855953
ENSG0000017367	0.535450545	####	###	EIF1AX	protein_coding	eukaryotic t	X	20124525	20141838
ENSG0000012007	-0.991144847	####	###	KANSL1	protein_coding	KAT8 regula	17	46029916	46225389
ENSG0000013331	-0.88238152	####	###	WDR74	protein_coding	WD repeat c	11	62832342	62841809
ENSG0000010297	0.678104953	####	###	POLR2C	protein_coding	RNA polyme	16	57462660	57472009
ENSG0000018572	0.692423096	####	###	ANKFY1	protein_coding	ankyrin repe	17	4163821	4263995
ENSG0000010012	-0.443850357	####	###	EIF3L	protein_coding	eukaryotic t	22	37848868	37889407
ENSG0000014931	-0.435989401	####	###	ATM	protein_coding	ATM serine/	11	108222832	108369102
ENSG0000018922	-2.334739801	####	###	MAOA	protein_coding	monoamine	X	43654907	43746824
ENSG0000019669	-0.829011412	####	###	ZNF33B	protein_coding	zinc finger p	10	42574185	42638570
ENSG0000014186	-0.586263837	####	###	BRD4	protein_coding	bromodoma	19	15235519	15332545
ENSG0000022968	0.520895844	####	###	TUBB	protein_coding	tubulin beta	CHR_HSCHR	30798683	30803908
ENSG0000010756	-0.789209242	####	###	RAB11FIP2	protein_coding	RAB11 fami	10	118004916	118046941
ENSG0000010834	-0.515200842	####	###	CASC3	protein_coding	CASC3 exon	17	40140318	40172171
ENSG0000012942	-0.549914409	####	###	MTUS1	protein_coding	microtubule	8	17643795	17800917

ENSG0000013583	-1.667807616	####	###	KIAA1614	protein_coding	KIAA1614 [S	1	180912897	180951614
ENSG0000015376	1.147137322	####	###	GTF2E1	protein_coding	general tran	3	120742637	120783069
ENSG0000012360	0.802448172	####	###	TTC21B	protein_coding	tetratricope	2	165857475	165953816
ENSG0000014482	-1.685513013	####	###	MYH15	protein_coding	myosin heav	3	108380368	108529322
ENSG0000019699	-3.169894338	####	###	FAM163B	protein_coding	family with s	9	133577081	133609389
ENSG0000009578	-0.443207483	####	###	WAC	protein_coding	WW domain	10	28532493	28623112
ENSG0000025907	-9.72723496	####	###	LINC00639	lncRNA	long interge	14	38738155	38948273
ENSG0000002092	0.65274092	####	###	MRE11	protein_coding	MRE11 hom	11	94415570	94493885
ENSG0000011256	-2.516222094	####	###	TFEB	protein_coding	transcription	6	41683978	41736259
ENSG0000016057	-1.695797299	####	###	DEDD2	protein_coding	death effect	19	42198598	42220140
ENSG0000010119	-0.580105328	####	###	DIDO1	protein_coding	death induc	20	62877738	62937952
ENSG0000012587	-0.785454489	####	###	TBC1D20	protein_coding	TBC1 doma	20	435480	462543
ENSG0000007105	0.390373136	####	###	MAP4K4	protein_coding	mitogen-ac	2	101696850	101894689
ENSG0000010771	-1.544544854	####	###	PALD1	protein_coding	phosphatase	10	70478767	70568450
ENSG0000017834	-1.686928694	####	###	SHISA3	protein_coding	shisa family	4	42397488	42402487
ENSG0000013020	-2.968427569	####	###	NECTIN2	protein_coding	nectin cell a	19	44846175	44889223
ENSG0000017483	1.033841998	####	###	DENND6A	protein_coding	DENN doma	3	57625454	57693077
ENSG0000018334	1.313814307	####	###	JRKL	protein_coding	JRK like [Sou	11	96389989	96507574
ENSG0000019872	1.18047552	####	###	ECI2	protein_coding	enoyl-CoA	6	4115689	4135597
ENSG0000007083	0.48530484	####	###	CDC42	protein_coding	cell division	1	22025511	22101360
ENSG0000026041	-1.419233976	####	###	AL353746.1	lncRNA	novel transc	9	27937617	27944497
ENSG0000010024	0.619128732	####	###	SUN2	protein_coding	Sad1 and U	22	38734725	38794143
ENSG0000015229	0.497392779	####	###	TGOLN2	protein_coding	trans-golgi	2	85318020	85328296
ENSG0000017738	-1.608158542	####	###	PPFIA3	protein_coding	PTPRF intera	19	49119544	49151026
ENSG0000011241	1.498198438	####	###	PHACTR2	protein_coding	phosphatase	6	143536845	143831185
ENSG0000011653	-0.529175342	####	###	ASH1L	protein_coding	ASH1 like hi	1	155335268	155562807
ENSG0000015815	1.328776559	####	###	CNNM4	protein_coding	cyclin and C	2	96760902	96811874
ENSG0000018780	-3.005024476	####	###	PEAR1	protein_coding	platelet end	1	156893698	156916434
ENSG0000015920	0.64934613	####	###	UBE2Z	protein_coding	ubiquitin co	17	48908407	48929056
ENSG0000011600	0.701708295	####	###	PCYOX1	protein_coding	prenylcystei	2	70257386	70281185
ENSG0000012887	0.49364318	####	###	TMOD2	protein_coding	tropomodul	15	51751597	51816363
ENSG0000002529	0.697173409	####	###	PHF20	protein_coding	PHD finger	20	35771974	35950370
ENSG0000013964	0.599953157	####	###	ESYT1	protein_coding	extended sy	12	56118250	56144674
ENSG0000017506	0.679727216	####	###	UBE2C	protein_coding	ubiquitin co	20	45812576	45816957
ENSG0000016825	-0.658047055	####	###	DNAJC7	protein_coding	DnaJ heat sl	17	41976421	42021376
ENSG0000017163	-0.479154779	####	###	BPTF	protein_coding	bromodoma	17	67825503	67984378
ENSG0000010525	-0.992858222	####	###	FSD1	protein_coding	fibronectin t	19	4304598	4323843
ENSG0000007250	0.432815459	####	###	SMC1A	protein_coding	structural m	X	53374149	53422728
ENSG0000015191	-0.430112013	####	###	DST	protein_coding	dystonin [So	6	56457987	56954649
ENSG0000015925	1.352029867	####	###	CHAF1B	protein_coding	chromatin a	21	36385392	36419015
ENSG0000011141	0.702150262	####	###	C12orf49	protein_coding	chromosom	12	116710171	116738070
ENSG0000019843	0.364893743	####	###	TXNRD1	protein_coding	thioredoxin	12	104215779	104350307
ENSG0000022516	0.784138249	####	###	PRRC2A	protein_coding	proline rich	CHR_HSCHR	31608127	31625177
ENSG0000027267	0.983974955	####	###	PCDHB16	protein_coding	protocadhe	5	141181399	141186399
ENSG0000010030	-1.210886838	####	###	TSPO	protein_coding	translocator	22	43151547	43163242
ENSG0000012853	-2.245312031	####	###	CDHR3	protein_coding	cadherin rel	7	105876796	106036432
ENSG0000010122	0.584955745	####	###	CDC25B	protein_coding	cell division	20	3786772	3806121
ENSG0000005427	1.399098003	####	###	OPN3	protein_coding	opsin 3 [Sou	1	241590102	241677376
ENSG0000007738	0.553330281	####	###	DYNC1I2	protein_coding	dynein cyto	2	171687409	171750158
ENSG0000014722	0.779725846	####	###	PRPS1	protein_coding	phosphoribo	X	107628428	107651993
ENSG0000016444	-1.625699353	####	###	CITED2	protein_coding	Cbp/p300 in	6	139371807	139374648
ENSG0000017640	-0.719463947	####	###	KCMF1	protein_coding	potassium c	2	84971093	85059472
ENSG0000011065	0.600431256	####	###	CD81	protein_coding	CD81 molec	11	2376177	2397397
ENSG0000012121	0.895300874	####	###	TMEM131L	protein_coding	transmembr	4	153466346	153636711
ENSG0000009925	1.31907918	####	###	NRP1	protein_coding	neuropilin 1	10	33177492	33336262
ENSG0000017106	0.972606468	####	###	C11orf24	protein_coding	chromosom	11	68261338	68272001
ENSG0000013422	0.955772433	####	###	PSRC1	protein_coding	proline and	1	109279556	109283186
ENSG0000025855	-2.009365646	####	###	SPECC1L-A	protein_coding	SPECC1L-A	22	24270898	24442356

ENSG0000008278	-1.076525397	####	###	ITGB5	protein_coding	integrin sub	3	124761948	124901418
ENSG0000017820	0.703785543	####	###	PLEC	protein_coding	plectin [Sou	8	143915147	143976734
ENSG0000022084	-0.982437568	####	###	RPL21P16	processed_pse	ribosomal p	10	120354701	120355183
ENSG0000013509	-0.766439945	####	###	TAOK3	protein_coding	TAO kinase	12	118149801	118372907
ENSG0000011673	0.67715602	####	###	PRDM2	protein_coding	PR/SET dom	1	13700198	13825079
ENSG0000013624	-0.545682678	####	###	KDELR2	protein_coding	KDEL endop	7	6445953	6484190
ENSG0000015840	1.242458726	####	###	CDC25C	protein_coding	cell division	5	138285265	138338355
ENSG0000011105	-3.750353363	####	###	LIN7A	protein_coding	lin-7 homol	12	80792520	80937925
ENSG0000010829	-0.380834698	####	###	RPL19	protein_coding	ribosomal p	17	39200283	39204732
ENSG0000011221	1.171077974	####	###	RAB23	protein_coding	RAB23, mer	6	57186992	57222307
ENSG0000010661	0.631395453	####	###	RHEB	protein_coding	Ras homolo	7	151466012	151520120
ENSG0000010532	1.849696478	####	###	TGFB1	protein_coding	transforming	19	41301587	41353922
ENSG0000014158	-0.941120956	####	###	CBX4	protein_coding	chromobox	17	79833156	79839440
ENSG0000017502	-0.529006733	####	###	CTBP2	protein_coding	C-terminal I	10	124984317	125161170
ENSG0000011516	0.833140923	####	###	CENPA	protein_coding	centromere	2	26764289	26801067
ENSG0000027380	3.041686485	####	###	H2BC8	protein_coding	H2B clustere	6	26215159	26216692
ENSG0000017103	0.514397458	####	###	PKIA	protein_coding	cAMP-depe	8	78516340	78605267
ENSG0000015051	-1.39494591	####	###	FAM124A	protein_coding	family with s	13	51222334	51284239
ENSG0000027327	2.252336915	####	###	AC090114.2	lncRNA	novel transc	7	128524016	128531069
ENSG0000008897	-0.737761809	####	###	KIZ	protein_coding	kizuna centr	20	21125983	21246622
ENSG0000010838	-0.552647276	####	###	MTMR4	protein_coding	myotubulari	17	58489529	58517905
ENSG0000013562	1.573790145	####	###	SEMA4F	protein_coding	ssemaphori	2	74654228	74683853
ENSG0000017924	-4.825810661	####	###	CDH4	protein_coding	cadherin 4 [	20	61252261	61940617
ENSG0000019667	1.028974296	####	###	ZFP62	protein_coding	ZFP62 zinc f	5	180847611	180861285
ENSG0000014701	-1.068939756	####	###	SH3KBP1	protein_coding	SH3 domain	X	19533977	19887600
ENSG0000026244	1.108747594	####	###	GBA	protein_coding	glucosylcera	CHR_HSCHR	155249852	155260266
ENSG0000016807	0.670446643	####	###	PBK	protein_coding	PDZ binding	8	27809624	27838082
ENSG0000008316	0.466103925	####	###	KAT6A	protein_coding	lysine acetyl	8	41929479	42051994
ENSG0000018709	1.208410117	####	###	ENTPD5	protein_coding	ectonucleos	14	73958010	74019399
ENSG0000015946	0.910751249	####	###	AMFR	protein_coding	autocrine m	16	56361452	56425545
ENSG0000015509	0.917855427	####	###	KLF10	protein_coding	Kruppel like	8	102648784	102655725
ENSG0000010947	-0.548451464	####	###	RPL34	protein_coding	ribosomal p	4	108620566	108630412
ENSG0000018460	1.081974829	####	###	SNN	protein_coding	stannin [Sou	16	11668455	11679152
ENSG0000017697	-3.536868432	####	###	FIBIN	protein_coding	fin bud initi	11	26994112	26997087
ENSG0000014935	0.807186761	####	###	LAMTOR1	protein_coding	late endoso	11	72085895	72103297
ENSG0000014667	0.675317373	####	###	CDCA5	protein_coding	cell division	11	65066300	65084164
ENSG0000012752	2.8531536	####	###	KLF2	protein_coding	Kruppel like	19	16324826	16328685
ENSG0000024168	0.709222857	####	###	ARPC1A	protein_coding	actin relatec	7	99325898	99366262
ENSG0000012014	0.97475872	####	###	MSX2	protein_coding	msh homeo	5	174724582	174730896
ENSG0000016468	0.806414282	####	###	FABP5	protein_coding	fatty acid bi	8	81280536	81284777
ENSG0000027744	0.402379091	####	###	MARCKS	protein_coding	myristoylate	6	113857345	113863475
ENSG0000014918	0.764090215	####	###	ARFGAP2	protein_coding	ADP ribosyl	11	47164299	47177125
ENSG0000013365	0.452346695	####	###	ATP13A3	protein_coding	ATPase 13A	3	194402672	194498364
ENSG0000015626	-0.449523251	####	###	CCT8	protein_coding	chaperonin	21	29055805	29073797
ENSG0000018463	-0.788993867	####	###	MED12	protein_coding	mediator co	X	71118556	71142454
ENSG0000012096	0.815439193	####	###	ZNF706	protein_coding	zinc finger p	8	101177878	101206193
ENSG0000015916	-0.644745556	####	###	SV2A	protein_coding	synaptic ves	1	149903318	149917844
ENSG0000011435	-0.60066035	####	###	TFG	protein_coding	trafficking fr	3	100709295	100748964
ENSG0000011920	0.554200576	####	###	CPSF3	protein_coding	cleavage an	2	9423651	9473101
ENSG0000010025	1.207020437	####	###	LMF2	protein_coding	lipase matu	22	50502949	50507702
ENSG0000018324	-3.114499965	####	###	PRR36	protein_coding	proline rich	19	7868719	7874390
ENSG0000017661	0.507026119	####	###	LMNB2	protein_coding	lamin B2 [Sc	19	2427638	2456959
ENSG0000014416	-0.970562242	####	###	ZC3H8	protein_coding	zinc finger C	2	112211529	112255136
ENSG0000005806	0.779168439	####	###	ATP11B	protein_coding	ATPase pho	3	182793503	182921629
ENSG0000016839	0.679499002	####	###	ATG4B	protein_coding	autophagy r	2	241637213	241673857
ENSG0000014214	-1.458268397	####	###	HUNK	protein_coding	hormonally	21	31873020	32044633
ENSG0000008725	0.516190666	####	###	GNAO1	protein_coding	G protein su	16	56191390	56357444
ENSG0000017924	1.349610559	####	###	GVQW3	protein_coding	GVQW mot	11	76381303	76414619

ENSG0000010442	0.687272121	####	###	ZC2HC1A	protein_coding	zinc finger C		8	78666089	78719765
ENSG0000014752	0.952136635	####	###	TACC1	protein_coding	transforming		8	38728186	38853028
ENSG0000019735	-1.086466963	####	###	UAP1L1	protein_coding	UDP-N-ace		9	137077517	137084539
ENSG0000016684	1.100046872	####	###	C18orf54	protein_coding	chromosom		18	54357906	54385218
ENSG0000016076	-1.30797669	####	###	FAM189B	protein_coding	family with s		1	155247205	155255483
ENSG0000013651	0.596286129	####	###	ACTL6A	protein_coding	actin like 6A		3	179562886	179588407
ENSG0000010595	0.710089463	####	###	OGDH	protein_coding	oxoglutarate		7	44606572	44709066
ENSG0000017685	0.537492373	####	###	FAM91A1	protein_coding	family with s		8	123768439	123815452
ENSG0000013490	-0.55625856	####	###	TPP2	protein_coding	tripeptidyl p		13	102596986	102679958
ENSG0000007933	0.510848116	####	###	SAR1A	protein_coding	secretion as		10	70147289	70170523
ENSG0000026811	1.489952229	####	###	AC010615.2	lncRNA	novel transc		19	21444241	21463908
ENSG0000016513	-2.103174689	####	###	ANKS6	protein_coding	ankyrin repe		9	98731329	98796965
ENSG0000011375	-0.411100059	####	###	DBN1	protein_coding	drebrin 1 [S		5	177456608	177474401
ENSG0000017220	-1.72978562	####	###	GPR22	protein_coding	G protein-c		7	107470018	107475684
ENSG0000023345	1.018945753	####	###	KIFC1	protein_coding	kinesin fami	CHR_HSCHR		33531696	33550087
ENSG0000002057	-1.65258009	####	###	SAMD4A	protein_coding	sterile alpha		14	54566117	54793315
ENSG0000000739	0.567475787	####	###	LUC7L	protein_coding	LUC7 like [S		16	188969	229463
ENSG0000016904	-1.435951997	####	###	IRS1	protein_coding	insulin recep		2	226731317	226799759
ENSG0000026420	3.314114638	####	###	AC239868.1	lncRNA	novel transc		1	149861271	149862504
ENSG0000008959	0.407224088	####	###	GANAB	protein_coding	glucosidase		11	62624826	62646726
ENSG0000014643	0.499230195	####	###	TMEM181	protein_coding	transmembr		6	158536436	158635428
ENSG0000008060	-0.501410846	####	###	SRCAP	protein_coding	Snf2 related		16	30698209	30741409
ENSG0000018253	0.483828125	####	###	MXRA7	protein_coding	matrix remo		17	76672551	76711016
ENSG0000011273	-0.447476924	####	###	PRPF4B	protein_coding	pre-mRNA		6	4021267	4064983
ENSG0000014607	0.75777252	####	###	TNFRSF21	protein_coding	TNF recepto		6	47231532	47309905
ENSG0000017457	-0.726089382	####	###	MSL2	protein_coding	MSL comple		3	136148917	136197241
ENSG0000010327	0.492571931	####	###	UBE2I	protein_coding	ubiquitin co		16	1308880	1327018
ENSG0000015276	0.509606066	####	###	FARP1	protein_coding	FERM, ARH/		13	98142562	98455176
ENSG0000013836	-0.589668789	####	###	ATIC	protein_coding	5-aminoimi		2	215311956	215349773
ENSG0000015818	-1.905215084	####	###	MRAS	protein_coding	muscle RAS		3	138347648	138405534
ENSG0000016461	-0.820418824	####	###	CAMLG	protein_coding	calcium mo		5	134738548	134752157
ENSG0000011088	-1.895343612	####	###	ASIC1	protein_coding	acid sensing		12	50057548	50083611
ENSG0000024773	3.509039198	####	###	AC120114.1	lncRNA	novel transc		16	29926836	29928933
ENSG0000027267	-2.667333852	####	###	DOC2B	protein_coding	double C2 c	CHR_HSCHR		142789	180568
ENSG0000017640	-0.931971572	####	###	RIMS2	protein_coding	regulating s		8	103500696	104256094
ENSG0000018852	-2.436041275	####	###	FAM83G	protein_coding	family with s		17	18968789	19004764
ENSG0000011733	0.497787975	####	###	CD46	protein_coding	CD46 molec		1	207752054	207795513
ENSG0000011913	-0.912324377	####	###	KLF9	protein_coding	Kruppel like		9	70384597	70414624
ENSG0000012139	-0.719124663	####	###	PSPC1	protein_coding	paraspeckle		13	19674752	19783019
ENSG0000020476	-5.643049882	####	###	INSYN2B	protein_coding	inhibitory sy		5	169861303	169980495
ENSG0000012295	0.620672556	####	###	VPS26A	protein_coding	VPS26, retro		10	69123512	69174412
ENSG0000012600	-0.891729611	####	###	PLAGL2	protein_coding	PLAG1 like z		20	32192504	32207743
ENSG0000014521	2.107118259	####	###	DGKQ	protein_coding	diacylglycer		4	958887	986895
ENSG0000013051	-2.064694793	####	###	PGPEP1	protein_coding	pyroglutamy		19	18340598	18369950
ENSG0000023967	-0.686059864	####	###	NME1	protein_coding	NME/NM23		17	51153559	51162428
ENSG0000016434	0.785008838	####	###	GFM2	protein_coding	GTP depenc		5	74721206	74767147
ENSG0000017077	0.489714438	####	###	AKAP13	protein_coding	A-kinase an		15	85380571	85749358
ENSG0000010399	0.866912673	####	###	CEP152	protein_coding	centrosoma		15	48712928	48811146
ENSG0000010047	-1.609567149	####	###	COCH	protein_coding	cochlin [Sou		14	30874514	30895065
ENSG0000019699	-1.079487031	####	###	WDR45	protein_coding	WD repeat (	X		49074433	49101170
ENSG0000010022	0.620473291	####	###	GTPBP1	protein_coding	GTP binding		22	38705742	38738299
ENSG0000020446	0.638275424	####	###	PRRC2A	protein_coding	proline rich		6	31620715	31637771
ENSG0000009301	-0.968542729	####	###	COMT	protein_coding	catechol-O-		22	19941733	19969975
ENSG0000010532	-0.463629881	####	###	HNRNPUL1	protein_coding	heterogene		19	41262496	41307787
ENSG0000007521	-1.646761867	####	###	SEMA3A	protein_coding	semaphorin		7	83955777	84492724
ENSG0000011486	-0.91320594	####	###	FOXP1	protein_coding	forkhead bo		3	70952817	71583989
ENSG0000014824	-0.480550027	####	###	SURF4	protein_coding	surfeit 4 [So		9	133361450	133376166
ENSG0000013780	1.036503378	####	###	MAPKBP1	protein_coding	mitogen-ac		15	41774434	41827855

ENSG0000013709	0.843827002	####	###	DNAJB5	protein_coding	DnaJ heat sl	9	34989641	34998900
ENSG0000017355	1.56734581	####	###	NABP1	protein_coding	nucleic acid	2	191678068	191741097
ENSG0000024367	-0.485178807	####	###	NME2	protein_coding	NME/NM23	17	51165435	51171744
ENSG0000013596	-1.043888043	####	###	TGFBRAP1	protein_coding	transforming	2	105264391	105329735
ENSG0000005479	-0.675458949	####	###	ATP9A	protein_coding	ATPase pho	20	51596514	51768390
ENSG0000013627	1.107771546	####	###	NACAD	protein_coding	NAC alpha	7	45080437	45088969
ENSG0000006874	-0.661582063	####	###	IP6K2	protein_coding	inositol hex	3	48688003	48740353
ENSG0000019731	0.634033846	####	###	DDI2	protein_coding	DNA damag	1	15617458	15669044
ENSG0000013707	1.366819909	####	###	IL11RA	protein_coding	interleukin 1	9	34650702	34661902
ENSG0000000663	0.71402273	####	###	DBF4	protein_coding	DBF4 zinc fi	7	87876216	87909553
ENSG0000010129	0.614900154	####	###	CDS2	protein_coding	CDP-diacylg	20	5126879	5197887
ENSG0000026177	-2.816852236	####	###	AC006504.	lncRNA	novel transc	19	27757184	27760849
ENSG0000012229	-0.648756967	####	###	ZC3H7A	protein_coding	zinc finger C	16	11750586	11797258
ENSG0000017994	1.076661203	####	###	BBS10	protein_coding	Bardet-Biec	12	76344474	76348415
ENSG0000013844	0.458043922	####	###	ABI2	protein_coding	abl interacto	2	203328280	203447728
ENSG0000018751	0.277043828	####	###	PTMA	protein_coding	prothymosir	2	231706895	231713541
ENSG0000018442	-1.051662797	####	###	TOP1MT	protein_coding	DNA topois	8	143304384	143359979
ENSG0000017094	1.576794436	####	###	ZNF160	protein_coding	zinc finger p	19	53066606	53103436
ENSG0000020494	-1.543474606	####	###	ZNF783	protein_coding	zinc finger f	7	149262171	149297302
ENSG0000010806	0.648218843	####	###	SHOC2	protein_coding	SHOC2 leuc	10	110919593	111013666
ENSG0000008249	-0.92424639	####	###	SERTAD4	protein_coding	SERTA dom	1	210232796	210246631
ENSG0000014381	0.550704332	####	###	LBR	protein_coding	lamin B rece	1	225401502	225428925
ENSG0000020399	1.449028486	####	###	ARRDC1-A	lncRNA	ARRDC1 an	9	137615332	137618906
ENSG0000012435	0.958655803	####	###	NAGK	protein_coding	N-acetylglu	2	71064344	71079808
ENSG0000017689	1.737809217	####	###	PXMP2	protein_coding	peroxisoma	12	132687587	132704985
ENSG0000014467	-0.602736446	####	###	GOLGA4	protein_coding	golgin A4 [S	3	37243177	37366751
ENSG0000010306	-0.725670159	####	###	SLC7A6	protein_coding	solute carrie	16	68264516	68301823
ENSG0000016325	0.684740572	####	###	DCAF16	protein_coding	DDB1 and C	4	17800655	17810758
ENSG0000012674	-0.713219048	####	###	ZNF384	protein_coding	zinc finger p	12	6666477	6689572
ENSG0000018308	-1.936949212	####	###	GAS6	protein_coding	growth arre	13	113820549	113864076
ENSG0000011123	0.888559179	####	###	VPS29	protein_coding	VPS29 retro	12	110491083	110502111
ENSG0000000413	0.673502173	####	###	SARM1	protein_coding	sterile alpha	17	28364356	28404049
ENSG0000008990	0.570556297	####	###	RCOR1	protein_coding	REST corepr	14	102592649	102730561
ENSG0000016838	-0.364746265	####	###	SEPTIN2	protein_coding	septin 2 [So	2	241315100	241354027
ENSG0000010464	0.683954307	####	###	MTMR9	protein_coding	myotubulari	8	11284816	11328146
ENSG0000016319	-0.869502308	####	###	S100A11	protein_coding	S100 calciur	1	152032506	152047907
ENSG0000016586	1.102883922	####	###	ZFYVE1	protein_coding	zinc finger F	14	72969451	73027131
ENSG0000017909	-1.235358001	####	###	PER1	protein_coding	period circa	17	8140472	8156506
ENSG0000027795	-0.946117158	####	###	MAPT	protein_coding	microtubule	CHR_HSCHR	45906010	46039943
ENSG0000010915	-5.049859885	####	###	GABRA4	protein_coding	gamma-am	4	46918900	46993581
ENSG0000012748	0.391584591	####	###	HP1BP3	protein_coding	heterochror	1	20740266	20787323
ENSG0000010016	-0.785932158	####	###	SEPTIN3	protein_coding	septin 3 [So	22	41969475	41998221
ENSG0000010293	0.801947901	####	###	ARL2BP	protein_coding	ADP ribosyl	16	57245259	57253635
ENSG0000012176	0.956313858	####	###	ZCCHC17	protein_coding	zinc finger C	1	31296982	31364953
ENSG0000016395	0.696601538	####	###	SLBP	protein_coding	stem-loop b	4	1692731	1712344
ENSG0000007503	-4.178666824	####	###	WSCD2	protein_coding	WSC domai	12	108129288	108250537
ENSG0000007980	0.483790911	####	###	DNM2	protein_coding	dynamin 2 [	19	10718079	10833488
ENSG0000008900	0.536444577	####	###	SNX5	protein_coding	sorting nexi	20	17941597	17968980
ENSG0000013794	0.67288452	####	###	KYAT3	protein_coding	kynurenine	1	88935773	88992953
ENSG0000018282	-0.701764273	####	###	ACBD3	protein_coding	acyl-CoA bi	1	226144679	226186741
ENSG0000015295	-1.127439253	####	###	NRSN1	protein_coding	neurensin 1	6	24126186	24154900
ENSG0000012660	-0.553642271	####	###	TRAP1	protein_coding	TNF recepto	16	3651639	3717553
ENSG0000015033	5.373711105	####	###	FCGR1A	protein_coding	Fc fragment	1	149782671	149792518
ENSG0000017101	-0.866452204	####	###	PYGO1	protein_coding	pygopus far	15	55538890	55588947
ENSG0000014169	-0.701023956	####	###	NT5C3B	protein_coding	5'-nucleotic	17	41825057	41836260
ENSG0000014162	-0.821058208	####	###	DYM	protein_coding	dymeclin [S	18	49036387	49461347
ENSG0000026989	-0.807999975	####	###	SNHG8	lncRNA	small nucle	4	118278709	118285316
ENSG0000016549	-0.515771603	####	###	PCF11	protein_coding	PCF11 cleav	11	83156988	83187451

ENSG0000013623	-0.772124	####	###	RAPGEF5	protein_coding	Rap guanine	7	22118236	22357154
ENSG0000014175	0.674948568	####	###	IGFBP4	protein_coding	insulin like g	17	40443450	40457725
ENSG0000011847	-1.800148558	####	###	SGIP1	protein_coding	SH3GL inter	1	66533383	66751139
ENSG0000013069	0.755877165	####	###	CEP85	protein_coding	centrosoma	1	26234200	26278808
ENSG0000027645	1.183158819	####	###	LENG8	protein_coding	leukocyte re	CHR_HSCHR	54380864	54394163
ENSG0000006179	-0.710350575	####	###	MRPS35	protein_coding	mitochondr	12	27710822	27756295
ENSG0000013620	-3.800829008	####	###	TNS3	protein_coding	tensin 3 [So	7	47275154	47582558
ENSG0000018676	0.945495513	####	###	SPIN4	protein_coding	spindlin fam	X	63347228	63351332
ENSG0000011826	-0.483447996	####	###	KLF7	protein_coding	Kruppel like	2	207074137	207167267
ENSG0000010573	-1.271893922	####	###	GRIK5	protein_coding	glutamate io	19	41998321	42069498
ENSG0000019835	-0.815398531	####	###	PIM3	protein_coding	Pim-3 proto	22	49960768	49964072
ENSG0000019681	-1.551583834	####	###	MVB12B	protein_coding	multivesicul	9	126326829	126507041
ENSG0000010041	1.217272916	####	###	PMM1	protein_coding	phosphoma	22	41576900	41589871
ENSG0000016350	0.693832338	####	###	CIP2A	protein_coding	cellular inhil	3	108549864	108589644
ENSG0000018450	-1.30218694	####	###	PROS1	protein_coding	protein S [S	3	93873051	93980003
ENSG0000022620	2.165524591	####	###	SGMS1-AS	lncRNA	SGMS1 anti	10	50623466	50641451
ENSG0000001266	0.42047019	####	###	ELOVL5	protein_coding	ELOVL fatty	6	53267398	53349179
ENSG0000007156	-0.610140528	####	###	TCF3	protein_coding	transcription	19	1609290	1652615
ENSG0000016679	-0.406449816	####	###	PPIB	protein_coding	peptidylprol	15	64155817	64163022
ENSG0000025373	0.839264238	####	###	OTUD6B-A	lncRNA	OTUD6B an	8	91059318	91070583
ENSG0000019775	-1.123522476	####	###	HOXC6	protein_coding	homeobox C	12	53990624	54030823
ENSG0000016251	-0.995146129	####	###	PEF1	protein_coding	penta-EF-h	1	31629866	31644896
ENSG0000018144	-3.158864307	####	###	ZNF467	protein_coding	zinc finger p	7	149764182	149773588
ENSG0000018905	1.587885516	####	###	RNFT1	protein_coding	ring finger p	17	59952240	59964761
ENSG0000000580	-0.646800726	####	###	ZNF195	protein_coding	zinc finger p	11	3339261	3379222
ENSG0000018547	-2.013992294	####	###	GPRIN3	protein_coding	GPRIN famil	4	89236383	89307800
ENSG0000016271	-0.658175696	####	###	ZNF496	protein_coding	zinc finger p	1	247297412	247331846
ENSG0000014379	0.502734163	####	###	MBOAT2	protein_coding	membrane b	2	8852690	9003709
ENSG0000015783	-2.085825314	####	###	GAREM2	protein_coding	GRB2 associ	2	26173088	26189663
ENSG0000024670	2.003835809	####	###	H2AJ	protein_coding	H2A.J histor	12	14774383	14778002
ENSG0000011208	-0.388043444	####	###	SRSF3	protein_coding	serine and a	6	36594353	36605600
ENSG0000013997	2.927207822	####	###	ARMH4	protein_coding	armadillo lik	14	57999735	58298139
ENSG0000012248	-0.620652063	####	###	ZNF644	protein_coding	zinc finger p	1	90915298	91022272
ENSG0000012476	-0.975905987	####	###	CDKN1A	protein_coding	cyclin deper	6	36676460	36687337
ENSG0000027115	2.283718142	####	###	AL161729.1	lncRNA	novel transc	9	95506235	95507636
ENSG0000013693	-0.368175207	####	###	ANP32B	protein_coding	acidic nucle	9	97983341	98015943
ENSG0000014219	1.495012056	####	###	DOP1B	protein_coding	DOP1 leucir	21	36156782	36294274
ENSG0000013437	-0.623551721	####	###	CDC73	protein_coding	cell division	1	193121983	193254815
ENSG0000002953	-2.315404726	####	###	ANK1	protein_coding	ankyrin 1 [S	8	41653220	41896762
ENSG0000016332	0.432023828	####	###	CGGBP1	protein_coding	CGG triplet	3	88051944	88149885
ENSG0000006717	-0.967464882	####	###	PHKA1	protein_coding	phosphoryla	X	72578814	72714319
ENSG0000010290	0.637970092	####	###	NUP93	protein_coding	nucleoporin	16	56730118	56850286
ENSG0000017039	-0.793512709	####	###	DCLK2	protein_coding	doublecortir	4	150078445	150257438
ENSG0000022199	3.139404265	####	###	EXOC3-AS:	lncRNA	EXOC3 antis	5	441498	443160
ENSG0000023477	-1.468442737	####	###	SLC25A25-	lncRNA	SLC25A25 a	9	128108581	128118693
ENSG0000012310	-1.050763402	####	###	CCDC91	protein_coding	coiled-coil c	12	28133249	28581511
ENSG0000007153	0.888840445	####	###	TRIP13	protein_coding	thyroid horr	5	892884	919357
ENSG0000013468	-0.516819436	####	###	YARS1	protein_coding	tyrosyl-tRN/	1	32775237	32818153
ENSG0000023764	1.001844533	####	###	KIFC1	protein_coding	kinesin fami	6	33391823	33409896
ENSG0000004063	-0.994167919	####	###	PHF23	protein_coding	PHD finger	17	7235029	7239722
ENSG0000019656	-0.951422563	####	###	SULF2	protein_coding	sulfatase 2 [	20	47656348	47786616
ENSG0000006753	-0.839532377	####	###	RRP15	protein_coding	ribosomal R	1	218285293	218337983
ENSG0000016803	0.512235581	####	###	CTNNB1	protein_coding	catenin beta	3	41194741	41260096
ENSG0000019636	0.779217533	####	###	SRGAP2B	protein_coding	SLIT-ROBO	1	144887265	145095528
ENSG0000010217	-0.527331419	####	###	SMS	protein_coding	spermine sy	X	21940709	21994837
ENSG0000027427	-0.914393268	####	###	CBSL	protein_coding	cystathionin	21	6444869	6468040
ENSG0000008707	-0.757224064	####	###	PPP1R15A	protein_coding	protein pho	19	48872421	48876058
ENSG0000018611	0.910990494	####	###	PIP5K1C	protein_coding	phosphatidy	19	3630183	3700468

ENSG0000019825	0.72772441	####	###	UBL5	protein_codinç	ubiquitin lik	19	9827892	9830115
ENSG0000024945	-1.146909148	####	###	ZNF286B	transcribed_un	zinc finger p	17	18658429	18682228
ENSG0000008467	0.844720592	####	###	NCOA1	protein_codinç	nuclear rece	2	24491914	24770702
ENSG0000010682	0.477786502	####	###	TLE4	protein_codinç	TLE family n	9	79571773	79726882
ENSG0000000537	-2.918863694	####	###	TSPOAP1	protein_codinç	TSPO associ	17	58301228	58328795
ENSG0000020419	1.020921198	####	###	KIFC1	protein_codinç	kinesin fami	CHR_HSCHR	33320351	33338717
ENSG0000014309	0.793920391	####	###	STRIP1	protein_codinç	striatin inter	1	110031577	110074641
ENSG0000010052	-0.57395086	####	###	GNPNAT1	protein_codinç	glucosamin	14	52775193	52791668
ENSG0000011824	-1.344485712	####	###	MREG	protein_codinç	melanoregu	2	215942584	216034096
ENSG0000012237	-0.709290946	####	###	SHLD2	protein_codinç	shieldin con	10	87094161	87191468
ENSG0000016949	0.977097672	####	###	TM2D2	protein_codinç	TM2 domai	8	38988808	38996824
ENSG0000018559	-0.493243515	####	###	SP1	protein_codinç	Sp1 transcri	12	53380176	53416446
ENSG0000005770	-1.781975886	####	###	TMCC3	protein_codinç	transmemb	12	94567122	94650557
ENSG0000025533	0.33523188	####	###	AL096711.2	protein_codinç	novel prote	6	127438406	127519001
ENSG0000018287	0.651144414	####	###	RBM10	protein_codinç	RNA binding	X	47145221	47186813
ENSG0000023182	0.711666526	####	###	PRRC2A	protein_codinç	proline rich	CHR_HSCHR	31611467	31628517
ENSG0000002577	0.791563709	####	###	NCAPH2	protein_codinç	non-SMC c	22	50508224	50524780
ENSG0000012828	-4.123134711	####	###	CDC42EP1	protein_codinç	CDC42 effe	22	37560480	37569405
ENSG0000001043	-1.082531844	####	###	PRSS3	protein_codinç	serine prote	9	33750466	33799231
ENSG0000019789	0.515784566	####	###	ADH5	protein_codinç	alcohol deh	4	99070978	99088801
ENSG0000024892	-3.924062524	####	###	AC114284.1	lncRNA	novel transc	5	120781218	120790778
ENSG0000006571	-2.204411061	####	###	TLE2	protein_codinç	TLE family n	19	2997639	3047635
ENSG0000013987	-3.080279864	####	###	SSTR1	protein_codinç	somatostati	14	38207904	38213067
ENSG0000018666	-0.49284971	####	###	ZFP91	protein_codinç	ZFP91 zinc f	11	58579063	58621550
ENSG0000004027	0.700388323	####	###	SPDL1	protein_codinç	spindle app	5	169583636	169604778
ENSG0000008726	-0.91841288	####	###	SH3BP2	protein_codinç	SH3 domair	4	2793071	2841098
ENSG0000014039	0.853499644	####	###	WDR61	protein_codinç	WD repeat	15	78277835	78299703
ENSG0000016964	0.900305205	####	###	LUZP1	protein_codinç	leucine zipp	1	23084023	23177808
ENSG0000013177	0.685169649	####	###	CHD1L	protein_codinç	chromodom	1	147242654	147295765
ENSG0000015279	-0.363374101	####	###	HNRNPDL	protein_codinç	heterogene	4	82422564	82430408
ENSG0000021363	-0.386679726	####	###	PPP1CB	protein_codinç	protein pho	2	28751640	28802940
ENSG0000027861	1.071797194	####	###	C11orf98	protein_codinç	chromosom	11	62662817	62665210
ENSG0000009933	0.59693876	####	###	MYO9B	protein_codinç	myosin IXB	19	17075781	17214537
ENSG0000017354	-1.839321354	####	###	SNX33	protein_codinç	sorting nexi	15	75647912	75662301
ENSG0000016638	-1.406793634	####	###	PPFIBP2	protein_codinç	PPFIA bindi	11	7513298	7657127
ENSG0000014814	-0.716436744	####	###	ZNF462	protein_codinç	zinc finger p	9	106863166	107013634
ENSG0000010525	-0.974207977	####	###	SHD	protein_codinç	Src homolo	19	4279266	4290722
ENSG0000013114	-0.437008331	####	###	GSE1	protein_codinç	Gse1 coiled	16	85169525	85676204
ENSG0000009201	-1.0094562	####	###	PSME1	protein_codinç	proteasome	14	24136163	24138967
ENSG0000017899	0.947632243	####	###	AURKB	protein_codinç	aurora kinas	17	8204733	8210600
ENSG0000020484	0.41400189	####	###	DCTN1	protein_codinç	dynactin sul	2	74361154	74392087
ENSG0000010522	-0.792200721	####	###	AKT2	protein_codinç	AKT serine/t	19	40230317	40285536
ENSG0000026189	1.026955944	####	###	CLK2	protein_codinç	CDC like kin	CHR_HSCHR	155278272	155293895
ENSG0000014397	-0.554329949	####	###	ASXL2	protein_codinç	ASXL transc	2	25733753	25878487
ENSG0000012162	0.73511814	####	###	KIF18A	protein_codinç	kinesin fami	11	28020619	28108156
ENSG0000025907	-4.116497999	####	###	POC1B-GA	protein_codinç	POC1B-GAI	12	89519408	89526262
ENSG0000001413	0.948372265	####	###	POLA2	protein_codinç	DNA polym	11	65261920	65305589
ENSG0000012818	1.110642921	####	###	DGCR6L	protein_codinç	DiGeorge sy	22	20314238	20320080
ENSG0000010230	0.996858236	####	###	PIN4	protein_codinç	peptidylpro	X	72181353	72302926
ENSG0000015587	0.721031046	####	###	RRAGA	protein_codinç	Ras related	9	19049427	19051025
ENSG0000024273	0.464574444	####	###	RTL5	protein_codinç	retrotransp	X	72127110	72131901
ENSG0000013974	-0.597576117	####	###	RBM26	protein_codinç	RNA binding	13	79311824	79406477
ENSG0000011744	-0.771597372	####	###	AKR1A1	protein_codinç	aldo-keto re	1	45550543	45570049
ENSG0000019890	0.668241694	####	###	MAP3K3	protein_codinç	mitogen-ac	17	63622415	63696305
ENSG0000025987	2.942690234	####	###	AC009113.1	lncRNA	novel transc	16	89215211	89217653
ENSG0000014351	-0.542244009	####	###	TP53BP2	protein_codinç	tumor prote	1	223779893	223845954
ENSG0000019705	0.903739946	####	###	ZMYM1	protein_codinç	zinc finger M	1	35032172	35115859
ENSG0000016747	0.611628557	####	###	MIDN	protein_codinç	midnolin [Sc	19	1248553	1259140

ENSG0000027812	-1.122491611	####	###	ZNF8	protein_coding	zinc finger p	19	58278955	58302791
ENSG0000000801	0.509559114	####	###	PSMB1	protein_coding	proteasome	6	170535120	170553307
ENSG0000011580	0.61051053	####	###	STRN	protein_coding	striatin [Sou	2	36837698	36966536
ENSG0000002503	-1.37818807	####	###	RRAGD	protein_coding	Ras related	6	89364616	89412273
ENSG0000010370	1.553513495	####	###	MTFMT	protein_coding	mitochondr	15	65001512	65029639
ENSG0000014403	0.895010875	####	###	TPRKB	protein_coding	TP53RK binc	2	73729104	73737400
ENSG0000013918	-1.144424126	####	###	CLSTN3	protein_coding	calsyntenin	12	7129698	7158945
ENSG0000017295	-0.584325393	####	###	LCLAT1	protein_coding	lysocardiolip	2	30447226	30644225
ENSG0000014940	-4.064720676	####	###	GRIK4	protein_coding	glutamate io	11	120511746	120988906
ENSG0000010758	-0.294870653	####	###	EIF3A	protein_coding	eukaryotic t	10	119033670	119080823
ENSG0000016514	-5.653663453	####	###	FBP1	protein_coding	fructose-bis	9	94603133	94640249
ENSG0000018889	-0.493412403	####	###	MSL1	protein_coding	MSL comple	17	40121971	40136917
ENSG0000017556	1.12932291	####	###	UCP2	protein_coding	uncoupling	11	73974672	73983246
ENSG0000015740	-2.52499269	####	###	KIT	protein_coding	KIT proto-o	4	54657918	54740715
ENSG0000000588	-1.821450754	####	###	PDK2	protein_coding	pyruvate de	17	50094737	50112152
ENSG0000016154	0.416829649	####	###	SRSF2	protein_coding	serine and a	17	76734115	76737333
ENSG0000015317	-0.762655606	####	###	RASSF3	protein_coding	Ras associat	12	64507001	64697564
ENSG0000015304	0.77510905	####	###	CENPH	protein_coding	centromere	5	69189574	69210357
ENSG0000009098	0.729998467	####	###	EXOC1	protein_coding	exocyst com	4	55853648	55905086
ENSG0000021471	-1.530311348	####	###	ZBED1	protein_coding	zinc finger B	X	2486414	2500976
ENSG0000013471	-0.588522058	####	###	BTF3L4	protein_coding	basic transc	1	52056199	52090716
ENSG0000012065	1.123982285	####	###	TAF12	protein_coding	TATA-box b	1	28589323	28643085
ENSG0000008226	-0.86939324	####	###	FAM135A	protein_coding	family with s	6	70412941	70561174
ENSG0000014965	0.597993288	####	###	LSM14B	protein_coding	LSM family r	20	62122461	62135374
ENSG0000010469	0.565538844	####	###	PPP2CB	protein_coding	protein pho	8	30774457	30814314
ENSG0000017359	-0.547800439	####	###	PC	protein_coding	pyruvate ca	11	66848417	66958386
ENSG0000010105	0.54978543	####	###	MYBL2	protein_coding	MYB proto-	20	43667019	43716495
ENSG0000010050	1.468629577	####	###	PYGL	protein_coding	glycogen ph	14	50857891	50944483
ENSG0000010567	-0.8473624	####	###	ARMC6	protein_coding	armadillo re	19	19033575	19060311
ENSG0000015390	-0.692151973	####	###	DDAH1	protein_coding	dimethylarg	1	85318481	85578363
ENSG0000023640	-1.981858392	####	###	VLDLR-AS1	lncRNA	VLDLR antis	9	2421597	2643359
ENSG0000011090	0.60204544	####	###	KCTD10	protein_coding	potassium c	12	109448655	109477359
ENSG0000023445	-1.147979782	####	###	MAGI2-AS3	lncRNA	MAGI2 antis	7	79452877	79471208
ENSG0000012643	-0.566408979	####	###	PRDX5	protein_coding	peroxiredox	11	64318121	64321811
ENSG0000016393	0.720544593	####	###	BAP1	protein_coding	BRCA1 asso	3	52401008	52410008
ENSG0000013966	-0.807483923	####	###	WDFY2	protein_coding	WD repeat a	13	51584455	51767709
ENSG0000023302	4.736088672	####	###	AC244453.2	lncRNA	novel transc	1	121090289	121097655
ENSG0000010193	-0.821698569	####	###	AMMECR1	protein_coding	AMMECR nu	X	110194186	110440233
ENSG0000011095	0.382941238	####	###	PTGES3	protein_coding	prostagland	12	56663341	56688408
ENSG0000007993	-2.138256286	####	###	MOXD1	protein_coding	monooxyge	6	132296055	132401475
ENSG0000005697	-2.551667547	####	###	TRAF3IP2	protein_coding	TRAF3 intera	6	111555381	111606906
ENSG0000011841	0.75769827	####	###	HMGN3	protein_coding	high mobilit	6	79201245	79234738
ENSG0000019818	-0.554574399	####	###	HSD17B11	protein_coding	hydroxystero	4	87336515	87391188
ENSG0000013997	0.593101749	####	###	RTN1	protein_coding	reticulon 1 [	14	59595976	59870776
ENSG0000010184	0.899389785	####	###	PSMD10	protein_coding	proteasome	X	108084207	108091549
ENSG0000010699	-1.629373827	####	###	ENG	protein_coding	endoglin [So	9	127815013	127854658
ENSG0000014144	3.800641629	####	###	GATA6	protein_coding	GATA bindi	18	22169589	22202528
ENSG0000017495	-4.015604518	####	###	FUT1	protein_coding	fucosyltrans	19	48748011	48755390
ENSG0000011350	-2.956051443	####	###	SLC12A7	protein_coding	solute carrie	5	1050384	1112063
ENSG0000024557	2.335784977	####	###	BDNF-AS	lncRNA	BDNF antise	11	27506830	27698231
ENSG0000005725	0.744207486	####	###	SOAT1	protein_coding	sterol O-acy	1	179293714	179358680
ENSG0000000149	0.712869594	####	###	LAS1L	protein_coding	LAS1 like rib	X	65512582	65534787
ENSG0000010968	0.766589754	####	###	STIM2	protein_coding	stromal inte	4	26857601	27025381
ENSG0000000524	0.636218992	####	###	PRKAR2B	protein_coding	protein kina	7	107044705	107161811
ENSG0000027330	-4.224811823	####	###	AC016717.2	lncRNA	novel transc	2	225698514	225703654
ENSG0000013491	-0.38594594	####	###	STT3A	protein_coding	STT3 oligosa	11	125591712	125625215
ENSG0000011209	0.62855252	####	###	SOD2	protein_coding	superoxide d	6	159669069	159762529
ENSG0000017268	1.435534368	####	###	ZNF738	protein_coding	zinc finger p	19	21358930	21379302

ENSG000001474	0.693259947	####	###	DOCK5	protein_coding	dedicator o		8	25184689	25418082
ENSG000001974	-0.865190658	####	###	RPF2	protein_coding	ribosome p		6	110982015	111028263
ENSG000000346	0.700309317	####	###	RNF19A	protein_coding	ring finger		8	100257060	100336218
ENSG000001113	0.812992999	####	###	GTF2H3	protein_coding	general trar		12	123633739	123662604
ENSG000001478	0.722311428	####	###	C9orf72	protein_coding	C9orf72-SN		9	27535640	27573866
ENSG000002758	-0.588339865	####	###	MLLT6	protein_coding	MLLT6, PHD	CHR_HSCHR		38506329	38530590
ENSG000001810	-0.864732919	####	###	AEN	protein_coding	apoptosis e		15	88621337	88632281
ENSG000001399	-0.651725276	####	###	DCAF5	protein_coding	DDB1 and C		14	69050881	69153150
ENSG000000646	-2.014315206	####	###	CNN2	protein_coding	calponin 2 [		19	1026586	1039068
ENSG000001180	-0.416294453	####	###	KMT2A	protein_coding	lysine meth		11	118436490	118526832
ENSG000002485	4.03938704	####	###	STIMATE-M	protein_coding	STIMATE-M		3	52833121	52897562
ENSG000001969	0.87402734	####	###	AP2A1	protein_coding	adaptor rela		19	49767001	49807114
ENSG000000910	0.471672399	####	###	OSBPL8	protein_coding	oxysterol bi		12	76351797	76559809
ENSG000002678	1.022855914	####	###	NDUFA7	protein_coding	NADH:ubiqu		19	8308768	8321375
ENSG000000266	-0.65708526	####	###	AGPAT4	protein_coding	1-acylglycer		6	161129967	161274061
ENSG000001088	-1.429401012	####	###	COL1A1	protein_coding	collagen typ		17	50184101	50201632
ENSG000000401	1.085195323	####	###	PHLPP2	protein_coding	PH domain		16	71637835	71724701
ENSG000001174	-0.949171724	####	###	NSUN4	protein_coding	NOP2/Sun		1	46340177	46365152
ENSG000001274	0.531690176	####	###	EMC1	protein_coding	ER membra		1	19215660	19251527
ENSG000000822	0.803896489	####	###	C5orf22	protein_coding	chromosom		5	31532287	31555053
ENSG000001698	0.427200023	####	###	ROBO1	protein_coding	roundabout		3	78597239	79767998
ENSG000001295	0.610345964	####	###	DAD1	protein_coding	defender ag		14	22564907	22589224
ENSG000001403	0.597891653	####	###	GTF2A2	protein_coding	general trar		15	59638062	59657541
ENSG000001697	-1.066687119	####	###	ZNF32	protein_coding	zinc finger		10	43643860	43648881
ENSG000001768	-2.585307028	####	###	GRIN1	protein_coding	glutamate i		9	137139154	137168756
ENSG000001709	-0.630148123	####	###	PAQR8	protein_coding	progestin a		6	52361421	52407777
ENSG000001434	1.696481037	####	###	SEMA6C	protein_coding	semaphorin		1	151131685	151146664
ENSG000001973	-1.167082326	####	###	LRP10	protein_coding	LDL recepto		14	22871740	22881713
ENSG000001162	0.495570486	####	###	PARK7	protein_coding	Parkinsonisr		1	7954291	7985505
ENSG000001342	1.264116767	####	###	NOTCH2	protein_coding	notch recep		1	119911553	120100779
ENSG000001683	-1.887902231	####	###	MFSD2A	protein_coding	major facilit		1	39955112	39969968
ENSG000001667	0.439287939	####	###	B2M	protein_coding	beta-2-mic		15	44711487	44718877
ENSG000000772	-1.894386822	####	###	IL4R	protein_coding	interleukin 4		16	27313668	27364778
ENSG000000552	-0.495259112	####	###	TAB2	protein_coding	TGF-beta a		6	149218641	149411613
ENSG000001061	1.780344956	####	###	NSUN5P2	transcribed_un	NSUN5 pse		7	72947581	72954790
ENSG000002315	2.78722402	####	###	FAHD2CP	transcribed_un	fumarylacet		2	96010551	96023380
ENSG000001543	-0.840009149	####	###	TNIK	protein_coding	TRAF2 and		3	171058414	171460408
ENSG000001100	1.121592287	####	###	SIAE	protein_coding	sialic acid a		11	124633113	124695707
ENSG000002254	-2.702101068	####	###	BX119917.1	transcribed_pr	tumor prote	X		72159845	72160346
ENSG000001973	1.30972392	####	###	GATA3-AS	lncRNA	GATA3 antis		10	8050450	8053484
ENSG000000602	0.365960652	####	###	WNK1	protein_coding	WNK lysine		12	752579	911452
ENSG000001971	0.680971839	####	###	PCNX3	protein_coding	pecanex 3 [		11	65615773	65637439
ENSG000001785	0.833271109	####	###	EPM2AIP1	protein_coding	EPM2A inte		3	36985043	36993131
ENSG000000805	1.197021419	####	###	SESN1	protein_coding	sestrin 1 [Sc		6	108986437	109094819
ENSG000000078	-1.926027687	####	###	TEAD3	protein_coding	TEA domain		6	35473597	35497079
ENSG000001064	0.542643933	####	###	GLCCI1	protein_coding	glucocortico		7	7968796	8094272
ENSG000002277	0.378246439	####	###	TUBB	protein_coding	tubulin beta	CHR_HSCHR		30710059	30715284
ENSG000001689	-0.310381073	####	###	MAT2A	protein_coding	methionine		2	85539168	85545281
ENSG000001979	2.060111105	####	###	ZNF347	protein_coding	zinc finger		19	53124072	53159075
ENSG000001257	1.268941946	####	###	GPCPD1	protein_coding	glycerophos		20	5544439	5611006
ENSG000001609	0.59747731	####	###	PWWP3A	protein_coding	PWWP dom		19	1354711	1378431
ENSG000001374	0.522337348	####	###	MTCH1	protein_coding	mitochondr		6	36968141	36986298
ENSG000001443	0.514561987	####	###	UBR3	protein_coding	ubiquitin pr		2	169827454	170084131
ENSG000002510	0.580325421	####	###	THAP9-AS	lncRNA	THAP9 antis		4	82893009	82900960
ENSG000001672	1.473787573	####	###	POP5	protein_coding	POP5 homo		12	120578764	120581402
ENSG000001751	-2.192795494	####	###	TMEM51-A	lncRNA	TMEM51 an		1	15111815	15153618
ENSG000001522	-1.423937107	####	###	SETBP1	protein_coding	SET binding		18	44680173	45068510
ENSG000001572	-0.677905108	####	###	MMP14	protein_coding	matrix meta		14	22836560	22849027

ENSG0000016626	-0.876507033	####	###	ZNF202	protein_coding	zinc finger p	11	123723914	123741675
ENSG0000011976	-0.51069163	####	###	SUPT7L	protein_coding	SPT7 like, S	2	27650809	27663840
ENSG0000018521	0.770427327	####	###	ZNF445	protein_coding	zinc finger p	3	44431705	44477670
ENSG0000016690	-1.837188556	####	###	STX3	protein_coding	syntaxin 3 [S	11	59713456	59805882
ENSG0000016631	1.442536429	####	###	SMPD1	protein_coding	sphingomye	11	6390440	6394998
ENSG0000020362	-1.728491757	####	###	MRPS18B	protein_coding	mitochondr	CHR_HSCHR	30607204	30615890
ENSG0000026728	1.866358836	####	###	TBX2-AS1	lncRNA	TBX2 antise	17	61393456	61411555
ENSG0000016567	0.458597147	####	###	PRDX3	protein_coding	peroxiredox	10	119167720	119178812
ENSG0000016335	-1.943565077	####	###	COL6A3	protein_coding	collagen typ	2	237324003	237414207
ENSG0000016928	-2.303383375	####	###	KCNAB1	protein_coding	potassium v	3	156037701	156539138
ENSG0000013514	0.79651676	####	###	TRAFD1	protein_coding	TRAF-type z	12	112125538	112153604
ENSG0000010163	0.887431923	####	###	CEP192	protein_coding	centrosoma	18	12991362	13125052
ENSG0000016334	0.642908088	####	###	PYGO2	protein_coding	pygopus far	1	154957026	154963853
ENSG0000004924	0.607595764	####	###	VAMP3	protein_coding	vesicle asso	1	7771296	7781432
ENSG0000006848	0.576625466	####	###	PRR11	protein_coding	proline rich	17	59155732	59206709
ENSG0000010312	1.011759277	####	###	CMC2	protein_coding	C-X9-C mo	16	80966448	81020270
ENSG0000010469	-1.612521652	####	###	UBXN8	protein_coding	UBX domair	8	30732247	30767006
ENSG0000014336	1.202512862	####	###	TUFT1	protein_coding	tuftelin 1 [S	1	151540305	151583583
ENSG0000016299	-1.559866804	####	###	FRZB	protein_coding	frizzled relat	2	182833275	182866637
ENSG0000018819	-1.935883781	####	###	PRKAR1B	protein_coding	protein kina	7	549197	727650
ENSG0000015123	0.853035488	####	###	GXYLT1	protein_coding	glucoside xy	12	42081845	42144874
ENSG0000018250	0.675036961	####	###	CEP97	protein_coding	centrosoma	3	101724593	101770562
ENSG0000013681	0.593437308	####	###	TXN	protein_coding	thioredoxin	9	110243810	110256507
ENSG0000012680	1.678115704	####	###	HSPA2	protein_coding	heat shock p	14	64535905	64546173
ENSG0000012448	0.38913315	####	###	USP9X	protein_coding	ubiquitin sp	X	41085445	41236579
ENSG0000018284	1.373602201	####	###	RRP7BP	transcribed_un	ribosomal R	22	42555223	42582038
ENSG0000010639	0.874055848	####	###	RPA3	protein_coding	replication p	7	7636518	7718607
ENSG0000014347	0.62300983	####	###	DTL	protein_coding	denticleless	1	212035553	212107400
ENSG0000015932	0.756754714	####	###	ADPGK	protein_coding	ADP depend	15	72751369	72785846
ENSG0000010543	-0.661026906	####	###	KDELR1	protein_coding	KDEL endop	19	48382575	48391551
ENSG0000019708	0.38054906	####	###	IGF2R	protein_coding	insulin like g	6	159969082	160113507
ENSG0000010702	1.63673238	####	###	PLGRKT	protein_coding	plasminoge	9	5357971	5437925
ENSG0000018104	-1.416592286	####	###	SLC26A11	protein_coding	solute carrie	17	80219699	80253500
ENSG0000010432	-2.073827875	####	###	TRPA1	protein_coding	transient rec	8	72019917	72075584
ENSG0000015505	-3.143181737	####	###	CNTNAP5	protein_coding	contactin as	2	124025287	124915287
ENSG0000014860	-2.423788125	####	###	CDHR1	protein_coding	cadherin rel	10	84194537	84219621
ENSG0000012167	1.172740635	####	###	CRY2	protein_coding	cryptochron	11	45847118	45883248
ENSG0000012115	0.675517705	####	###	NCAPH	protein_coding	non-SMC co	2	96335766	96377091
ENSG0000018097	0.84814424	####	###	LRRC57	protein_coding	leucine rich	15	42537820	42548802
ENSG0000016358	-1.274817646	####	###	RPL22L1	protein_coding	ribosomal p	3	170864875	170870208
ENSG0000015742	1.014112475	####	###	AASDH	protein_coding	aminoadipa	4	56338287	56387508
ENSG0000015146	-0.590574851	####	###	UPF2	protein_coding	UPF2 regula	10	11920022	12043170
ENSG0000004854	-2.391433404	####	###	LMO3	protein_coding	LIM domain	12	16548372	16610594
ENSG0000016295	-1.386481072	####	###	LRRTM1	protein_coding	leucine rich	2	80288351	80304752
ENSG0000011967	-1.155342248	####	###	ACOT2	protein_coding	acyl-CoA th	14	73567620	73575658
ENSG0000006553	0.910643917	####	###	MYLK	protein_coding	myosin light	3	123610049	123884332
ENSG0000014911	-0.586067685	####	###	TNKS1BP1	protein_coding	tankyrase 1	11	57299638	57324952
ENSG0000016297	3.217381703	####	###	SLC66A3	protein_coding	solute carrie	2	11155198	11178870
ENSG0000008830	1.451650454	####	###	DNMT3B	protein_coding	DNA methy	20	32762385	32809356
ENSG0000008020	-0.59104681	####	###	CRYBG3	protein_coding	crystallin be	3	97822011	97944984
ENSG0000013827	0.677561167	####	###	ANXA7	protein_coding	annexin A7	10	73375101	73414076
ENSG0000014052	0.483007933	####	###	FANCI	protein_coding	FA complem	15	89243945	89317261
ENSG0000014954	0.468744641	####	###	EI24	protein_coding	EI24 autoph	11	125569280	125584684
ENSG0000013604	-2.615530063	####	###	DRAM1	protein_coding	DNA damag	12	101877580	102012130
ENSG0000018411	-0.481039579	####	###	NIPSNAP1	protein_coding	nipsnap hor	22	29554808	29581327
ENSG0000013052	0.512987893	####	###	LSM4	protein_coding	LSM4 homo	19	18306236	18323112
ENSG0000019645	0.742031999	####	###	PIK3R4	protein_coding	phosphoino	3	130678934	130746829
ENSG0000014307	0.664249741	####	###	CTTNBP2N	protein_coding	CTTNBP2 N	1	112396214	112463456

ENSG0000019876	0.245268297	####	###	MT-ND2	protein_coding	mitochondr	MT	4470	5511
ENSG0000018619	0.757969101	####	###	SAPCD2	protein_coding	suppressor	9	137062127	137070557
ENSG0000003514	-0.810909232	####	###	FAM136A	protein_coding	family with	2	70295975	70302090
ENSG0000012278	-0.428521451	####	###	CALD1	protein_coding	caldesmon 1	7	134744252	134970729
ENSG0000011439	-0.316319811	####	###	RPL24	protein_coding	ribosomal p	3	101681091	101686718
ENSG0000014155	0.785454932	####	###	ANAPC11	protein_coding	anaphase p	17	81890790	81900991
ENSG0000014157	0.920163125	####	###	CEP131	protein_coding	centrosoma	17	81189593	81222999
ENSG0000010851	-0.408875486	####	###	MED13	protein_coding	mediator cc	17	61942605	62065278
ENSG0000000548	-0.550698436	####	###	KMT2E	protein_coding	lysine methy	7	105014190	105115019
ENSG0000005605	0.869207867	####	###	HPF1	protein_coding	histone PAR	4	169729470	169757944
ENSG0000015381	-0.8432451	####	###	JAZF1	protein_coding	JAZF zinc fir	7	27830573	28180795
ENSG0000011040	0.55060061	####	###	NECTIN1	protein_coding	nectin cell a	11	119623408	119729200
ENSG0000015984	-1.357998813	####	###	ABR	protein_coding	ABR activato	17	1003518	1229738
ENSG0000021822	-1.773107036	####	###	TATDN2P2	processed_pse	TatD DNase	6	158609706	158621636
ENSG0000016105	0.540559735	####	###	PSMC2	protein_coding	proteasome	7	103344254	103369395
ENSG0000017351	-1.488295773	####	###	VEGFB	protein_coding	vascular enc	11	64234538	64238793
ENSG0000028000	2.156477438	####	###	AC078925.4	TEC	novel transc	12	130988312	130990028
ENSG0000010520	-1.357333726	####	###	DYRK1B	protein_coding	dual specific	19	39825350	39834201
ENSG0000017311	-3.172101516	####	###	HSPA6	protein_coding	heat shock p	1	161524540	161526894
ENSG0000018306	1.867147737	####	###	LYSMD4	protein_coding	LysM doma	15	99715697	99733561
ENSG0000007751	0.905816452	####	###	POLD3	protein_coding	DNA polym	11	74493851	74669117
ENSG0000014560	-0.446889442	####	###	SKP2	protein_coding	S-phase kin	5	36151989	36184319
ENSG0000013926	-1.368828681	####	###	MARCHF9	protein_coding	membrane a	12	57755103	57760411
ENSG0000010076	0.445068271	####	###	PSMC1	protein_coding	proteasome	14	90256527	90275429
ENSG0000014815	0.747389644	####	###	INIP	protein_coding	INTS3 and N	9	112683926	112718149
ENSG0000013760	0.553464647	####	###	NEK1	protein_coding	NIMA relate	4	169392809	169612627
ENSG0000011158	0.524344186	####	###	NUP107	protein_coding	nucleoporin	12	68686951	68745809
ENSG0000019612	0.940641329	####	###	KIAA0895L	protein_coding	KIAA0895 lil	16	67175599	67184040
ENSG0000011887	-0.522637524	####	###	RAB3GAP2	protein_coding	RAB3 GTPas	1	220148293	220272453
ENSG0000017038	-0.716572765	####	###	SLC30A1	protein_coding	solute carrie	1	211571568	211579161
ENSG0000025101	-4.794608092	####	###	TMEM108-	lncRNA	TMEM108 a	3	133245603	133257207
ENSG0000026040	-1.893749285	####	###	AL513534.2	lncRNA	novel transc	10	68698500	68700794
ENSG0000014215	-0.859048076	####	###	COL6A1	protein_coding	collagen typ	21	45981769	46005050
ENSG0000013561	1.695862453	####	###	PRADC1	protein_coding	protease as	2	73228010	73233239
ENSG0000015473	-1.50336921	####	###	ADAMTS1	protein_coding	ADAM meta	21	26835755	26845409
ENSG0000013137	-1.362877428	####	###	RFTN1	protein_coding	raftlin, lipid	3	16313574	16514026
ENSG0000019853	1.623631953	####	###	ZNF28	protein_coding	zinc finger p	19	52797408	52857600
ENSG0000011736	-0.517527279	####	###	PRPF3	protein_coding	pre-mRNA	1	150321479	150353233
ENSG0000016396	0.867300805	####	###	PIGX	protein_coding	phosphatidy	3	196639775	196736007
ENSG0000018647	-1.995218263	####	###	BTN3A2	protein_coding	butyrophilin	6	26365159	26378320
ENSG0000011492	-3.410883407	####	###	SLC4A3	protein_coding	solute carrie	2	219627394	219641980
ENSG0000018019	-1.693092731	####	###	TDRP	protein_coding	testis develo	8	489792	545781
ENSG0000008556	2.182628602	####	###	ABCB1	protein_coding	ATP binding	7	87503017	87713323
ENSG0000018681	0.795477764	####	###	ZNF397	protein_coding	zinc finger p	18	35241030	35267133
ENSG0000017289	-0.655308938	####	###	DHCR7	protein_coding	7-dehydroc	11	71428193	71452868
ENSG0000017805	1.443204376	####	###	NDUFAF3	protein_coding	NADH:ubiqu	3	49020459	49023495
ENSG0000012074	-0.523881901	####	###	SERP1	protein_coding	stress assoc	3	150541998	150603228
ENSG0000027793	-0.787327124	####	###	MRPL45	protein_coding	mitochondr	CHR_HSCHR	38097522	38123634
ENSG0000016904	0.264061228	####	###	HNRNPH1	protein_coding	heterogene	5	179614178	179634784
ENSG0000018569	1.368036803	####	###	MYBL1	protein_coding	MYB proto-	8	66562175	66614247
ENSG0000020467	-0.997527331	####	###	AKT1S1	protein_coding	AKT1 substr	19	49869033	49878459
ENSG0000011881	0.340904614	####	###	CCNI	protein_coding	cyclin I [Sou	4	77047155	77076309
ENSG0000015782	0.647337878	####	###	AP3S2	protein_coding	adaptor rela	15	89830599	89894638
ENSG0000014362	-0.641776434	####	###	INTS3	protein_coding	integrator c	1	153728050	153774808
ENSG0000015937	0.421328627	####	###	PSMB4	protein_coding	proteasome	1	151399560	151401937
ENSG0000017755	1.473072477	####	###	ATOX1	protein_coding	antioxidant	5	151742316	151772532
ENSG0000012564	-1.023008117	####	###	SLC25A23	protein_coding	solute carrie	19	6436079	6465203
ENSG0000018386	-0.685307024	####	###	TOB2	protein_coding	transducer c	22	41433494	41446801

ENSG0000011672	-0.962076943	####	###	WLS	protein_coding	Wnt ligand	1	68098473	68233120
ENSG0000016882	0.529664579	####	###	NSG1	protein_coding	neuronal ve	4	4348140	4419058
ENSG0000027319	8.514432107	####	###	AP000692.2	lncRNA	novel transc	21	36319792	36320670
ENSG0000021889	-1.596475771	####	###	ZNF579	protein_coding	zinc finger p	19	55576774	55580848
ENSG0000017327	1.218452063	####	###	ZNF449	protein_coding	zinc finger p	X	135344796	135363413
ENSG0000014142	-0.503088767	####	###	RPRD1A	protein_coding	regulation c	18	35984387	36067576
ENSG0000013567	-0.51054339	####	###	MDM2	protein_coding	MDM2 prot	12	68808177	68850686
ENSG0000018145	-2.057729953	####	###	TMEM45A	protein_coding	transmembr	3	100492619	100577444
ENSG0000008832	0.384877323	####	###	TPX2	protein_coding	TPX2 microt	20	31739271	31801805
ENSG0000016880	1.02600854	####	###	LCMT2	protein_coding	leucine carb	15	43323649	43330582
ENSG0000016353	-0.569438623	####	###	CLASP2	protein_coding	cytoplasmic	3	33496245	33718356
ENSG0000012370	-4.863619258	####	###	KCNJ2	protein_coding	potassium ir	17	70168673	70180044
ENSG0000003724	0.940508943	####	###	RPL26L1	protein_coding	ribosomal p	5	172958729	172969771
ENSG0000012277	-0.407528325	####	###	TRIM24	protein_coding	tripartite mo	7	138460259	138589996
ENSG0000018360	-1.197795671	####	###	SFXN4	protein_coding	sideroflexin	10	119140767	119165714
ENSG0000013875	-0.476244691	####	###	FRAS1	protein_coding	Fraser extra	4	78057323	78544269
ENSG0000016117	-1.420576169	####	###	YDJC	protein_coding	YdjC chitool	22	21628089	21630064
ENSG0000013547	-0.762023953	####	###	FAIM2	protein_coding	Fas apoptot	12	49866896	49904217
ENSG0000017743	0.943458701	####	###	NAP1L5	protein_coding	nucleosome	4	88695913	88697829
ENSG0000016538	0.7304243	####	###	SPTSSA	protein_coding	serine palmi	14	34432788	34462240
ENSG0000010409	0.438002481	####	###	DMXL2	protein_coding	Dmx like 2 [	15	51447711	51622833
ENSG0000008098	0.824849725	####	###	NDC80	protein_coding	NDC80 kine	18	2571557	2616635
ENSG0000016528	-0.596128886	####	###	BRWD3	protein_coding	bromodoma	X	80669503	80809877
ENSG0000025615	2.158273046	####	###	ADGRD1-A	lncRNA	ADGRD1 an	12	130990138	130993976
ENSG0000013793	-1.306028874	####	###	BCAR3	protein_coding	BCAR3 adap	1	93561741	93847150
ENSG0000026006	5.434324809	####	###	AL512408.1	lncRNA	novel transc	1	26692132	26694131
ENSG0000015664	0.634845811	####	###	NPTN	protein_coding	neuroplastir	15	73560014	73634134
ENSG0000017988	-0.74996672	####	###	PDXDC1	protein_coding	pyridoxal de	16	14974591	15139339
ENSG0000015697	0.934231821	####	###	PDE6D	protein_coding	phosphodie	2	231732433	231786272
ENSG0000008081	0.642739547	####	###	PSEN1	protein_coding	presenilin 1	14	73136418	73223691
ENSG0000012225	-2.162980705	####	###	HS3ST2	protein_coding	heparan sul	16	22814162	22916338
ENSG0000010895	0.499197748	####	###	YWHAE	protein_coding	tyrosine 3-r	17	1344275	1400222
ENSG0000016756	2.374099245	####	###	ZNF701	protein_coding	zinc finger p	19	52555457	52587174
ENSG0000025565	-2.577036714	####	###	FAM222A-	lncRNA	FAM222A a	12	109734166	109773508
ENSG0000012892	0.652558203	####	###	IVD	protein_coding	isovaleryl-C	15	40405485	40435947
ENSG0000004961	-0.497618215	####	###	ARID1B	protein_coding	AT-rich inte	6	156776020	157210779
ENSG0000011698	-2.144739764	####	###	HPCAL4	protein_coding	hippocalcin	1	39678648	39691485
ENSG0000018705	-0.878905883	####	###	RPS19BP1	protein_coding	ribosomal p	22	39529093	39532761
ENSG0000010305	0.592709356	####	###	COG4	protein_coding	component	16	70480568	70523560
ENSG0000000632	2.150078112	####	###	TNFRSF12A	protein_coding	TNF recepto	16	3018445	3022383
ENSG0000009990	0.446406819	####	###	RANBP1	protein_coding	RAN binding	22	20115938	20127355
ENSG0000013736	1.095159744	####	###	TPMT	protein_coding	thiopurine S	6	18128311	18155077
ENSG0000017085	-0.681294296	####	###	RIOX2	protein_coding	ribosomal o	3	97941818	97972457
ENSG0000019875	-2.13215969	####	###	COLGALT2	protein_coding	collagen be	1	183929854	184037729
ENSG0000014640	-1.522373681	####	###	SLC18B1	protein_coding	solute carrie	6	132769370	132813339
ENSG0000010585	-0.665162539	####	###	HBP1	protein_coding	HMG-box tr	7	107168961	107202522
ENSG0000015127	-0.77877525	####	###	MAGI1	protein_coding	membrane a	3	65353525	66038834
ENSG0000017629	1.89578238	####	###	ZNF135	protein_coding	zinc finger p	19	58059239	58086310
ENSG0000010287	1.812630933	####	###	CORO1A	protein_coding	coronin 1A	16	30182827	30189076
ENSG0000008251	-0.742342804	####	###	TRAF5	protein_coding	TNF recepto	1	211326615	211374946
ENSG0000017057	-3.078329681	####	###	DLGAP1	protein_coding	DLG associa	18	3496032	4455307
ENSG0000025980	3.320750899	####	###	AC012640.2	lncRNA	novel transc	5	10352701	10353601
ENSG0000000653	0.872894243	####	###	AGK	protein_coding	acylglycerol	7	141551278	141655244
ENSG0000021339	0.710074204	####	###	ARHGAP19	protein_coding	Rho GTPase	10	97222173	97292673
ENSG0000006722	1.389335416	####	###	STOML1	protein_coding	stomatin like	15	73978926	73994622
ENSG0000026568	-0.335862638	####	###	RPL17	protein_coding	ribosomal p	18	49488453	49492523
ENSG0000012256	0.239609104	####	###	HNRNPA2B	protein_coding	heterogene	7	26173057	26201529
ENSG0000014216	0.501252643	####	###	SOD1	protein_coding	superoxide	21	31659666	31668931

ENSG0000014871	-0.930486927	####	###	DNAJB12	protein_codinc	DnaJ heat sl	10	72332830	72355149
ENSG0000010607	2.326901154	####	###	ABHD11	protein_codinc	abhydrolase	7	73736094	73738867
ENSG0000016591	-1.235882729	####	###	TTC7B	protein_codinc	tetratricope	14	90524564	90816479
ENSG0000017009	-0.355583472	####	###	NSG2	protein_codinc	neuronal ve	5	174045706	174243501
ENSG0000025120	-1.409412075	####	###	TMED7-TIC	protein_codinc	TMED7-TIC	5	115578642	115626161
ENSG0000012588	0.739680765	####	###	MCM8	protein_codinc	minichromo	20	5950652	5998977
ENSG0000014055	0.678178214	####	###	ST8SIA2	protein_codinc	ST8 alpha-N	15	92393881	92468728
ENSG0000016266	0.549032206	####	###	ZNF326	protein_codinc	zinc finger p	1	89995110	90035533
ENSG0000019872	-0.302926603	####	###	MT-CYB	protein_codinc	mitochondr MT		14747	15887
ENSG0000011231	0.745573176	####	###	GMNN	protein_codinc	geminin DN	6	24774931	24786099
ENSG0000017278	0.744632624	####	###	CBWD1	protein_codinc	COBW dom	9	121038	179147
ENSG0000013687	0.591490821	####	###	PRPF4	protein_codinc	pre-mRNA	9	113275642	113294009
ENSG0000017644	0.978499528	####	###	CLK2	protein_codinc	CDC like kin	1	155262868	155278491
ENSG0000014954	1.443169832	####	###	CCDC15	protein_codinc	coiled-coil c	11	124954121	125041489
ENSG0000019895	-3.421974803	####	###	TGM2	protein_codinc	transglutam	20	38127385	38166578
ENSG0000004805	-0.492754305	####	###	HDAC9	protein_codinc	histone dea	7	18086949	19002416
ENSG0000011554	0.576727465	####	###	KDM3A	protein_codinc	lysine deme	2	86440647	86492716
ENSG0000010434	-0.372817383	####	###	LAPTM4B	protein_codinc	lysosomal p	8	97775057	97853013
ENSG0000013601	-2.787673771	####	###	USP44	protein_codinc	ubiquitin sp	12	95516560	95551476
ENSG0000013048	-4.608866993	####	###	KLHDC7B	protein_codinc	kelch doma	22	50545899	50551023
ENSG0000010774	0.568658177	####	###	MICU1	protein_codinc	mitochondr	10	72367327	72626191
ENSG0000015911	1.43646797	####	###	IFNAR2	protein_codinc	interferon a	21	33229901	33265675
ENSG0000010860	-0.628821594	####	###	SMARCD2	protein_codinc	SWI/SNF rel	17	63832081	63843065
ENSG0000006552	-0.478443684	####	###	SPEN	protein_codinc	spen family	1	15836095	15940456
ENSG0000011233	0.54608677	####	###	HBS1L	protein_codinc	HBS1 like tr	6	134960378	135103056
ENSG0000018530	1.081429436	####	###	ARL15	protein_codinc	ADP ribosyl	5	53883942	54310582
ENSG0000015111	1.684534183	####	###	TMEM86A	protein_codinc	transmembr	11	18693122	18704785
ENSG0000010485	0.880878216	####	###	CLPTM1	protein_codinc	CLPTM1 reg	19	44954585	44993341
ENSG0000016093	1.19798325	####	###	LY6E	protein_codinc	lymphocyte	8	143017982	143023832
ENSG0000025903	2.564711574	####	###	FPGT-TNNI	protein_codinc	FPGT-TNNI	1	74198235	74544393
ENSG0000016645	-1.002464283	####	###	PRTG	protein_codinc	protogenin	15	55611544	55743152
ENSG0000006718	-1.364379181	####	###	TNFRSF1A	protein_codinc	TNF recepto	12	6328757	6342114
ENSG0000012868	-4.452907653	####	###	GAD1	protein_codinc	glutamate c	2	170813213	170861151
ENSG0000019803	-0.283414911	####	###	RPS4X	protein_codinc	ribosomal p X		72255679	72277248
ENSG0000013034	-0.670441258	####	###	SNX9	protein_codinc	sorting nexi	6	157700387	157945077
ENSG0000000146	-1.570219317	####	###	NIPAL3	protein_codinc	NIPA like do	1	24415802	24472976
ENSG0000017938	0.704379016	####	###	ELMOD2	protein_codinc	ELMO doma	4	140524168	140553770
ENSG0000014057	-0.834487778	####	###	CRTC3	protein_codinc	CREB regula	15	90529923	90645345
ENSG0000017829	0.517716372	####	###	GEN1	protein_codinc	GEN1 Hollic	2	17753858	17788946
ENSG0000015947	-1.063045083	####	###	MED8	protein_codinc	mediator cc	1	43383917	43389808
ENSG0000010287	-0.63270856	####	###	ZNF629	protein_codinc	zinc finger p	16	30778456	30787205
ENSG0000016887	0.981323483	####	###	ANKRD49	protein_codinc	ankyrin repe	11	94493979	94499578
ENSG0000010100	0.806358801	####	###	NINL	protein_codinc	ninein like [S	20	25452697	25585531
ENSG0000016359	0.513368643	####	###	PPM1L	protein_codinc	protein pho	3	160755602	161078902
ENSG0000018280	0.836871267	####	###	CRIP2	protein_codinc	cysteine rich	14	105472962	105480162
ENSG0000013962	0.936200989	####	###	CERS5	protein_codinc	ceramide sy	12	50129289	50167533
ENSG0000020405	-0.815102545	####	###	LINC00963	lncRNA	long interge	9	129476946	129513687
ENSG0000008331	-0.281300185	####	###	TNPO1	protein_codinc	transportin	5	72816312	72916733
ENSG0000011336	0.550913288	####	###	DROSHA	protein_codinc	drosha ribo	5	31400497	31532196
ENSG0000011521	0.533392643	####	###	NRBP1	protein_codinc	nuclear rece	2	27427790	27442259
ENSG0000009148	0.620470886	####	###	FH	protein_codinc	fumarate hy	1	241497603	241519755
ENSG0000015438	0.30762284	####	###	ENAH	protein_codinc	ENAH actin	1	225486835	225653142
ENSG0000012068	-0.640065178	####	###	PROSER1	protein_codinc	proline and	13	39009865	39038089
ENSG0000016528	0.370470394	####	###	VCP	protein_codinc	valosin cont	9	35056064	35072627
ENSG0000017643	-1.174160683	####	###	SYNE3	protein_codinc	spectrin rep	14	95407266	95475836
ENSG0000017140	-3.744918608	####	###	PDE7B	protein_codinc	phosphodie	6	135851701	136195574
ENSG0000011765	0.764554824	####	###	NEK2	protein_codinc	NIMA relate	1	211658657	211675630
ENSG0000011971	-3.98546723	####	###	GPR68	protein_codinc	G protein-c	14	91232532	91253925

ENSG0000000844	-0.607311861	####	###	NFIX	protein_coding	nuclear fact	19	12995608	13098796
ENSG0000017751	-0.750674947	####	###	ST8SIA3	protein_coding	ST8 alpha-N	18	57352557	57371731
ENSG0000013757	-0.928965553	####	###	SLCO5A1	protein_coding	solute carrie	8	69667046	69834978
ENSG0000015972	0.69088219	####	###	ATP6V0D1	protein_coding	ATPase H+	16	67438014	67481181
ENSG0000011992	-3.997932944	####	###	IFIT2	protein_coding	interferon ir	10	89283694	89309271
ENSG0000009571	4.160376027	####	###	CRTAC1	protein_coding	cartilage aci	10	97865000	98030828
ENSG0000015511	-0.961106127	####	###	GTF3C6	protein_coding	general tran	6	110958706	110967890
ENSG0000012064	1.995212739	####	###	IQSEC3	protein_coding	IQ motif anc	12	66767	178455
ENSG0000006784	-3.00727316	####	###	ATP2B3	protein_coding	ATPase plas	X	153517676	153582939
ENSG0000011739	0.557248282	####	###	CDC20	protein_coding	cell division	1	43358981	43363203
ENSG0000014534	0.625178471	####	###	CAMK2D	protein_coding	calcium/calr	4	113451032	113761927
ENSG0000018042	1.910101976	####	###	C11orf71	protein_coding	chromosom	11	114391443	114400511
ENSG0000009700	-0.503404242	####	###	ABL1	protein_coding	ABL proto-c	9	130713016	130887675
ENSG0000022416	3.63462177	####	###	DNAJC27-A	lncRNA	DNAJC27 ar	2	24971390	25039716
ENSG0000016641	0.752626372	####	###	IDH3A	protein_coding	isocitrate de	15	78131498	78171945
ENSG0000016516	0.982429901	####	###	DYNLT3	protein_coding	dynein light	X	37836757	37847571
ENSG0000017026	-0.658651985	####	###	ZNF282	protein_coding	zinc finger p	7	149195546	149226238
ENSG0000017044	-0.735055224	####	###	NFXL1	protein_coding	nuclear tran	4	47847233	47914667
ENSG0000018773	-1.087115719	####	###	NHEJ1	protein_coding	non-homolo	2	219069357	219160865
ENSG0000021873	0.721324751	####	###	CEBPZOS	protein_coding	CEBPZ oppo	2	37196488	37216193
ENSG0000016613	0.590325159	####	###	HIF1AN	protein_coding	hypoxia indu	10	100529072	100559998
ENSG0000013044	0.546514709	####	###	ZSWIM6	protein_coding	zinc finger S	5	61332258	61546172
ENSG0000016568	-0.673619751	####	###	ENTR1	protein_coding	endosome a	9	136401922	136410614
ENSG0000015188	-1.248320133	####	###	PARP8	protein_coding	poly(ADP-ri	5	50665899	50846519
ENSG0000022862	-6.452681448	####	###	AC245100.3	unprocessed_p	novel pseuc	1	148288001	148288951
ENSG0000011371	-0.612601152	####	###	ERGIC1	protein_coding	endoplasmi	5	172834251	172952683
ENSG0000018504	1.238171412	####	###	CIB1	protein_coding	calcium and	15	90229975	90234047
ENSG0000012094	0.416806661	####	###	TARDBP	protein_coding	TAR DNA bi	1	11012344	11025739
ENSG0000017960	-0.867144681	####	###	CDC42EP4	protein_coding	CDC42 effec	17	73283624	73312005
ENSG0000018103	1.009886732	####	###	METTL23	protein_coding	methyltrans	17	76726830	76733936
ENSG0000022675	0.661479404	####	###	CUTALP	transcribed_un	cutA divaler	9	120824828	120854385
ENSG0000011584	2.60093405	####	###	DLX2	protein_coding	distal-less h	2	172099438	172102900
ENSG0000012128	-1.422626807	####	###	ADCY7	protein_coding	adenylate cy	16	50246137	50318135
ENSG0000013835	-2.971621021	####	###	AOX1	protein_coding	aldehyde ox	2	200586014	200677064
ENSG0000005439	-1.249120404	####	###	HHAT	protein_coding	hedgehog a	1	210328252	210676296
ENSG0000019805	0.75717651	####	###	GRK6	protein_coding	G protein-c	5	177403204	177442901
ENSG0000016078	0.377983659	####	###	LMNA	protein_coding	lamin A/C [S	1	156082573	156140089
ENSG0000013844	-0.556456743	####	###	WDR12	protein_coding	WD repeat c	2	202874261	203014798
ENSG0000018656	-0.443822063	####	###	GPATCH8	protein_coding	G-patch do	17	44395281	44503430
ENSG0000017313	2.204917929	####	###	ADCK5	protein_coding	aarF domair	8	144373101	144393242
ENSG0000015791	0.574578129	####	###	RER1	protein_coding	retention in	1	2391775	2405442
ENSG0000015597	0.665777303	####	###	VPS37A	protein_coding	VPS37A sub	8	17246931	17302427
ENSG0000019626	0.25705719	####	###	PPIA	protein_coding	peptidylprol	7	44796680	44824564
ENSG0000016755	2.899828848	####	###	ZNF610	protein_coding	zinc finger p	19	52336243	52367778
ENSG0000011652	1.942992513	####	###	TRIM62	protein_coding	tripartite mo	1	33145399	33182059
ENSG0000008601	0.666849068	####	###	MAST2	protein_coding	microtubule	1	45786987	46036122
ENSG0000016348	-2.968640697	####	###	ADORA1	protein_coding	adenosine A	1	203090654	203167405
ENSG0000013962	-0.96296165	####	###	MAP3K12	protein_coding	mitogen-ac	12	53479669	53500063
ENSG0000010540	0.458003567	####	###	CDC37	protein_coding	cell division	19	10391133	10420121
ENSG0000013868	-1.13055142	####	###	FGF2	protein_coding	fibroblast gr	4	122826708	122898236
ENSG0000016021	-0.850661255	####	###	AGPAT3	protein_coding	1-acylglycer	21	43865223	43987592
ENSG0000025121	-1.097650477	####	###	AC106895.2	lncRNA	novel transc	4	173924207	173991024
ENSG0000012136	-0.806343864	####	###	KCNJ8	protein_coding	potassium ir	12	21764955	21775600
ENSG0000016459	-2.447404013	####	###	MYOZ3	protein_coding	myozenin 3	5	150660882	150679368
ENSG0000016760	-4.129549188	####	###	CYP2S1	protein_coding	cytochrome	19	41193210	41207539
ENSG0000012680	-0.584428813	####	###	ZBTB1	protein_coding	zinc finger a	14	64503712	64533690
ENSG0000009607	-0.592981826	####	###	BRPF3	protein_coding	bromodoma	6	36196744	36232790
ENSG0000016891	-0.665514719	####	###	ZNF608	protein_coding	zinc finger p	5	124636913	124748807

ENSG0000008724	0.33478044	####	###	MMP2	protein_coding	matrix meta	16	55389700	55506691
ENSG0000011338	-0.492704906	####	###	GOLPH3	protein_coding	golgi phosp	5	32124716	32174319
ENSG0000003835	0.61375557	####	###	EDC4	protein_coding	enhancer of	16	67873052	67884499
ENSG0000018854	-1.722966225	####	###	CCDC9B	protein_coding	coiled-coil c	15	40331452	40340967
ENSG0000015340	-1.683819522	####	###	PLEKHG4B	protein_coding	pleckstrin hc	5	92151	189972
ENSG0000026019	-2.000319168	####	###	AC124798.1	lncRNA	antisense to	11	17380649	17383531
ENSG0000010348	-1.204996345	####	###	XYLT1	protein_coding	xylosyltransf	16	17101769	17470960
ENSG0000024058	-2.970263444	####	###	AQP1	protein_coding	aquaporin 1	7	30911853	30925517
ENSG0000014574	-0.508922106	####	###	SLC30A5	protein_coding	solute carrie	5	69093949	69131069
ENSG0000014467	-0.923945256	####	###	CTDSPL	protein_coding	CTD small p	3	37861880	37984469
ENSG0000015344	-1.25061252	####	###	UBALD1	protein_coding	UBA like do	16	4608883	4615027
ENSG0000009703	0.54090526	####	###	SH3GLB1	protein_coding	SH3 domain	1	86704570	86748184
ENSG0000020423	1.619137805	####	###	OXLD1	protein_coding	oxidoreduct	17	81665036	81666635
ENSG0000010059	0.746187354	####	###	DAAM1	protein_coding	dishevelled	14	59188646	59371405
ENSG0000016944	0.595806613	####	###	MMGT1	protein_coding	membrane	X	135962070	135974063
ENSG0000021388	1.729704085	####	###	LINC01521	lncRNA	long interge	22	31346777	31348719
ENSG0000011180	-2.401911702	####	###	BTN3A3	protein_coding	butyrophilin	6	26440472	26453415
ENSG0000016552	0.504360141	####	###	ARF6	protein_coding	ADP ribosyla	14	49893082	49897054
ENSG0000018606	0.60152793	####	###	AIDA	protein_coding	axin interact	1	222668013	222713210
ENSG0000013615	-1.41795221	####	###	LMO7	protein_coding	LIM domain	13	75620434	75859870
ENSG0000012043	0.334806692	####	###	TCP1	protein_coding	t-complex 1	6	159778498	159789703
ENSG0000014635	0.563326746	####	###	CLVS2	protein_coding	clavesin 2 [S	6	122996235	123072925
ENSG0000013795	0.54325529	####	###	RABGGTB	protein_coding	Rab geranyl	1	75786197	75795086
ENSG0000016796	-0.839343497	####	###	MLST8	protein_coding	MTOR assoc	16	2204248	2209453
ENSG0000019760	0.686649261	####	###	CPLANE1	protein_coding	ciliogenesis	5	37106228	37249421
ENSG0000008917	-1.450212605	####	###	KIF16B	protein_coding	kinesin fami	20	16272104	16573448
ENSG0000010877	-0.827835828	####	###	KAT2A	protein_coding	lysine acetyl	17	42113111	42121367
ENSG0000019857	0.891005809	####	###	ARC	protein_coding	activity regu	8	142611049	142614479
ENSG0000008555	-1.160888105	####	###	IGSF9	protein_coding	immunoglob	1	159927039	159945613
ENSG0000012791	-0.483869793	####	###	AKAP9	protein_coding	A-kinase an	7	91940862	92110673
ENSG0000015378	0.746095559	####	###	ZDHHC7	protein_coding	zinc finger D	16	84974175	85011535
ENSG0000012053	0.607022427	####	###	MASTL	protein_coding	microtubule	10	27154824	27187953
ENSG0000019677	0.508832989	####	###	CD47	protein_coding	CD47 molec	3	108043091	108091862
ENSG0000010029	-0.602579314	####	###	THOC5	protein_coding	THO comple	22	29505879	29555216
ENSG0000010482	-1.343562568	####	###	ECH1	protein_coding	enoyl-CoA	19	38815422	38831841
ENSG0000019698	1.4155383	####	###	WDR5B	protein_coding	WD repeat c	3	122411846	122416062
ENSG0000011633	-0.614592448	####	###	AMPD2	protein_coding	adenosine m	1	109616104	109632053
ENSG0000010476	0.566994547	####	###	BNIP3L	protein_coding	BCL2 intera	8	26383054	26505636
ENSG0000018628	-1.097914102	####	###	TOR3A	protein_coding	torsin family	1	179082070	179098023
ENSG0000018860	-0.977039431	####	###	CLN3	protein_coding	CLN3 lysoso	16	28474111	28495575
ENSG0000012291	1.311033418	####	###	SLC25A16	protein_coding	solute carrie	10	68477998	68527523
ENSG0000013586	0.357952849	####	###	LAMC1	protein_coding	laminin subu	1	183023420	183145592
ENSG0000001080	-0.757787643	####	###	SCMH1	protein_coding	Scm polycor	1	41027200	41242154
ENSG0000016841	0.524931652	####	###	RFWD3	protein_coding	ring finger a	16	74621399	74666877
ENSG0000015627	-0.803377637	####	###	BACH1	protein_coding	BTB domain	21	29194071	29630751
ENSG0000013677	0.770527154	####	###	DNAJC1	protein_coding	DnaJ heat sh	10	21756548	22003769
ENSG0000008888	-2.158273533	####	###	EBF4	protein_coding	EBF family m	20	2692874	2760108
ENSG0000017131	-0.466096682	####	###	CHD7	protein_coding	chromodom	8	60678740	60868028
ENSG0000017563	0.718856738	####	###	RPS6KB2	protein_coding	ribosomal p	11	67428460	67435401
ENSG0000016907	-0.766726113	####	###	ROR2	protein_coding	receptor tyr	9	91563091	91950228
ENSG0000014156	-0.521112089	####	###	FOXK2	protein_coding	forkhead bo	17	82519713	82644662
ENSG0000015938	-3.998423539	####	###	IRX6	protein_coding	iroquois hor	16	55324203	55330756
ENSG0000027368	0.517346177	####	###	B2M	protein_coding	beta-2-mic	CHR_HSCHR	44711489	44718888
ENSG0000010009	0.602099577	####	###	LGALS1	protein_coding	galectin 1 [S	22	37675636	37679802
ENSG0000010297	-0.463513652	####	###	CTCF	protein_coding	CCCTC-bind	16	67562467	67639177
ENSG0000026286	-0.658443886	####	###	LSM14A	protein_coding	LSM14A mR	CHR_HSCHR	34172447	34229322
ENSG0000017959	-1.412648631	####	###	PLD6	protein_coding	phospholipa	17	17200995	17206333
ENSG0000021501	0.839349379	####	###	RTL10	protein_coding	retrotranspo	22	19846138	19854896

ENSG0000024713	2.05072415	####	###	AP000873.2	lncRNA	novel transc	11	83184491	83193794
ENSG0000016683	-0.443452266	####	###	NAV2	protein_coding	neuron navi	11	19350724	20121601
ENSG0000011512	0.441256468	####	###	SF3B6	protein_coding	splicing fact	2	24067586	24076373
ENSG0000015294	0.823644512	####	###	MED21	protein_coding	mediator cc	12	27022546	27066343
ENSG0000012086	0.710440685	####	###	APAF1	protein_coding	apoptotic pe	12	98645290	98735433
ENSG0000017348	-2.173808472	####	###	PTPRM	protein_coding	protein tyro	18	7566782	8406861
ENSG0000009500	0.464239291	####	###	MSH2	protein_coding	mutS homo	2	47403067	47663146
ENSG0000013803	0.543881897	####	###	PPM1B	protein_coding	protein pho	2	44167969	44244384
ENSG0000017810	0.632657266	####	###	PDE4DIP	protein_coding	phosphodie	1	148808181	149048286
ENSG0000007521	0.54908644	####	###	GTSE1	protein_coding	G2 and S-p	22	46296870	46330810
ENSG0000013653	0.428597037	####	###	MARCHF7	protein_coding	membrane a	2	159712457	159771027
ENSG0000015614	-0.714935899	####	###	ADAMTS3	protein_coding	ADAM meta	4	72280969	72569221
ENSG0000019895	0.68171154	####	###	KIFBP	protein_coding	kinesin fami	10	68988803	69043544
ENSG0000000805	0.895228122	####	###	SYN1	protein_coding	synapsin I [S	X	47571901	47619857
ENSG0000016264	-5.085038341	####	###	AKNAD1	protein_coding	AKNA doma	1	108815898	108963484
ENSG0000008050	-0.750688139	####	###	SMARCA2	protein_coding	SWI/SNF rel	9	1980290	2193624
ENSG0000019852	0.890529284	####	###	ZNF43	protein_coding	zinc finger p	19	21804946	21852125
ENSG0000009488	0.593052669	####	###	CDC23	protein_coding	cell division	5	138187650	138213343
ENSG0000002317	-1.061374787	####	###	GRAMD1B	protein_coding	GRAM dom	11	123358428	123627774
ENSG0000012012	-0.863054941	####	###	DUSP1	protein_coding	dual specific	5	172768096	172771195
ENSG0000011726	-2.272145032	####	###	CDK18	protein_coding	cyclin deper	1	205504595	205532793
ENSG0000021443	1.117203698	####	###	AS3MT	protein_coding	arsenite me	10	102869470	102901899
ENSG0000011698	-4.072640302	####	###	NT5C1A	protein_coding	5'-nucleotic	1	39659121	39672038
ENSG0000021470	-1.119713228	####	###	IFRD2	protein_coding	interferon re	3	50287732	50292918
ENSG0000007496	-1.401802089	####	###	ARHGEF10I	protein_coding	Rho guanine	1	17539698	17697874
ENSG0000009165	-0.521390835	####	###	ZFHX4	protein_coding	zinc finger h	8	76681239	76867281
ENSG0000013882	-1.180732999	####	###	SLC39A8	protein_coding	solute carrie	4	102251041	102431258
ENSG0000027427	1.684305392	####	###	AC069281.2	lncRNA	novel transc	7	100572232	100578700
ENSG0000015646	-6.312999898	####	###	GDF6	protein_coding	growth diffe	8	96142333	96160806
ENSG0000014157	-0.9071738	####	###	CBX8	protein_coding	chromobox	17	79792132	79801683
ENSG0000015625	-0.698420651	####	###	USP16	protein_coding	ubiquitin sp	21	29024629	29054488
ENSG0000017915	0.572069465	####	###	EDC3	protein_coding	enhancer of	15	74630558	74696292
ENSG0000017557	1.228081432	####	###	PAAF1	protein_coding	proteasoma	11	73876699	73931114
ENSG0000018263	2.000965257	####	###	CCNYL2	transcribed_un	cyclin Y like	10	42408168	42475349
ENSG0000012694	-0.785820195	####	###	ARMCX1	protein_coding	armadillo re	X	101550547	101554700
ENSG0000027125	-0.827574251	####	###	AC240274.1	protein_coding	neuroblasto	KI270711.1	4612	29626
ENSG0000011007	0.8009547	####	###	FOXRED1	protein_coding	FAD depend	11	126269055	126278131
ENSG0000010039	-0.406362901	####	###	EP300	protein_coding	E1A binding	22	41092592	41180077
ENSG0000013628	1.026132461	####	###	CCM2	protein_coding	CCM2 scaffo	7	44999475	45076469
ENSG0000019873	0.547064378	####	###	CTR9	protein_coding	CTR9 homo	11	10751246	10801625
ENSG0000020605	0.486067096	####	###	JPT2	protein_coding	Jupiter micr	16	1678256	1702280
ENSG0000010947	0.437113963	####	###	CPE	protein_coding	carboxypep	4	165361194	165498547
ENSG0000016648	-1.068717389	####	###	MFAP4	protein_coding	microfibril a	17	19383442	19387190
ENSG0000024147	1.607938389	####	###	PTPRG-AS1	lncRNA	PTPRG antis	3	62221249	62369406
ENSG0000012105	-0.957064564	####	###	COIL	protein_coding	coilin [Sourc	17	56938199	56961050
ENSG0000007240	0.769440855	####	###	UBE2D1	protein_coding	ubiquitin co	10	58334979	58370751
ENSG0000026266	-1.141975773	####	###	FAM189B	protein_coding	family with s	CHR_HSCHR	155262609	155270887
ENSG0000022394	-5.109891371	####	###	ROR1-AS1	lncRNA	ROR1 antise	1	64094379	64171342
ENSG0000017223	-0.524110218	####	###	PAIP1	protein_coding	poly(A) binc	5	43526267	43557758
ENSG0000013780	-1.958537544	####	###	THBS1	protein_coding	thrombospo	15	39581079	39599466
ENSG0000013051	-0.903988817	####	###	SSBP4	protein_coding	single strand	19	18418864	18434562
ENSG0000016771	-1.178861343	####	###	WDR81	protein_coding	WD repeat c	17	1716523	1738599
ENSG0000011130	-0.618536164	####	###	NAA25	protein_coding	N-alpha-ac	12	112026689	112108796
ENSG0000013615	-0.800819551	####	###	COG3	protein_coding	component	13	45464898	45536701
ENSG0000022750	0.781896134	####	###	SCAMP4	protein_coding	secretory ca	19	1905372	1926013
ENSG0000021386	-1.028197808	####	###	RPL21P75	processed_pse	ribosomal p	7	20002765	20003247
ENSG0000017522	0.859039162	####	###	ARHGAP1	protein_coding	Rho GTPase	11	46677080	46700619
ENSG0000017277	0.493042309	####	###	PSME3IP1	protein_coding	proteasome	16	57152466	57186116

ENSG0000015395	-0.422710461	####	###	CACNA2D1	protein_codinc	calcium volt	7	81946444	82443777
ENSG0000016655	-0.704902794	####	###	TMED3	protein_codinc	transmembı	15	79311112	79427432
ENSG0000016337	-0.499424714	####	###	YY1AP1	protein_codinc	YY1 associa	1	155659443	155689000
ENSG0000015113	-2.246267488	####	###	BTBD11	protein_codinc	BTB domain	12	107318421	107659642
ENSG0000015879	-0.7324287	####	###	DEDD	protein_codinc	death effect	1	161120974	161132688
ENSG0000010551	-1.808881796	####	###	DBP	protein_codinc	D-box bind	19	48630030	48637379
ENSG0000017807	0.947925364	####	###	C2orf69	protein_codinc	chromosom	2	199911293	199955935
ENSG0000013591	-3.542668416	####	###	HTR2B	protein_codinc	5-hydroxytr	2	231108230	231125042
ENSG0000008900	-0.270289355	####	###	RPL6	protein_codinc	ribosomal p	12	112405190	112418838
ENSG0000010662	0.579636597	####	###	POLD2	protein_codinc	DNA polym	7	44114681	44124358
ENSG0000021015	0.566289132	####	###	MT-TS1	Mt_tRNA	mitochondr	MT	7446	7514
ENSG0000019621	1.154241629	####	###	ZNF766	protein_codinc	zinc finger p	19	52269587	52296046
ENSG0000019622	0.813964256	####	###	FAM217B	protein_codinc	family with s	20	59933764	59948680
ENSG0000015268	-0.742214361	####	###	PELO	protein_codinc	pelota mRN	5	52787916	52804044
ENSG0000013426	1.092043834	####	###	AP4B1	protein_codinc	adaptor rela	1	113894194	113905201
ENSG0000012859	0.338467456	####	###	CALU	protein_codinc	calumenin [	7	128739292	128773400
ENSG0000010247	-0.669035346	####	###	NDFIP2	protein_codinc	Nedd4 fami	13	79481124	79556075
ENSG0000004244	0.856099722	####	###	RETSAT	protein_codinc	retinol satur	2	85341955	85354531
ENSG0000011690	0.649128891	####	###	EXOC8	protein_codinc	exocyst cor	1	231332753	231337852
ENSG0000016035	0.773000322	####	###	ZNF714	protein_codinc	zinc finger p	19	21082159	21125270
ENSG0000006259	0.720584872	####	###	ELMO2	protein_codinc	engulfment	20	46366050	46432985
ENSG0000025710	-0.638647335	####	###	LSM14A	protein_codinc	LSM14A mF	19	34172504	34229515
ENSG0000017027	-1.633281892	####	###	FAXDC2	protein_codinc	fatty acid hy	5	154818492	154859252
ENSG0000018422	0.76176253	####	###	PCDH9	protein_codinc	protocadhe	13	66302834	67230445
ENSG0000016494	-1.050053731	####	###	GEM	protein_codinc	GTP bindinc	8	94249253	94262350
ENSG0000012169	0.957113992	####	###	DEPDC7	protein_codinc	DEP domair	11	33015876	33033582
ENSG0000016498	0.70104903	####	###	TMEM65	protein_codinc	transmembı	8	124306189	124372692
ENSG0000007772	0.622399737	####	###	UBE2A	protein_codinc	ubiquitin co	X	119574536	119591083
ENSG0000013146	0.425484005	####	###	PSME3	protein_codinc	proteasome	17	42824385	42843760
ENSG0000027878	0.724357087	####	###	UBR7	protein_codinc	ubiquitin pr	CHR_HSCHR	93207056	93229215
ENSG0000015886	-0.485163798	####	###	NDUFS2	protein_codinc	NADH:ubiqu	1	161197104	161214395
ENSG0000010997	-0.255957518	####	###	HSPA8	protein_codinc	heat shock p	11	123057489	123063230
ENSG0000000588	-0.641692143	####	###	ZFX	protein_codinc	zinc finger p	X	24149173	24216255
ENSG0000021393	1.111367752	####	###	GALT	protein_codinc	galactose-1	9	34638133	34651035
ENSG0000010074	0.871436412	####	###	VRK1	protein_codinc	VRK serine/t	14	96797382	96931722
ENSG0000015629	-0.611956983	####	###	TIAM1	protein_codinc	TIAM Rac1 a	21	31118416	31559977
ENSG0000018279	1.327230985	####	###	TMEM198B	transcribed_un	transmembı	12	55829608	55836246
ENSG0000000581	-0.442036059	####	###	MYCBP2	protein_codinc	MYC bindin	13	77044657	77327094
ENSG0000027648	-2.971561156	####	###	SLC12A7	protein_codinc	solute carrie	CHR_HSCHR	1050378	1111210
ENSG0000006655	0.773424593	####	###	LRRC40	protein_codinc	leucine rich	1	70144805	70205579
ENSG0000014337	-0.593340398	####	###	SNX27	protein_codinc	sorting nexi	1	151612006	151699091
ENSG0000010842	-0.284743318	####	###	KPNB1	protein_codinc	karyopherin	17	47649476	47685505
ENSG0000014106	-0.955015195	####	###	KSR1	protein_codinc	kinase supp	17	27456470	27626438
ENSG0000006065	-1.402383006	####	###	PTPRU	protein_codinc	protein tyro	1	29236516	29326813
ENSG0000027519	-0.457267249	####	###	AKT3	protein_codinc	AKT serine/t	CHR_HSCHR	243488233	243855434
ENSG0000004847	0.764068098	####	###	SNX29	protein_codinc	sorting nexi	16	11976734	12574287
ENSG0000026617	0.715554917	####	###	STRADA	protein_codinc	STE20 relate	17	63682336	63741986
ENSG0000017460	-5.496995357	####	###	CMKLR1	protein_codinc	chemerin ch	12	108288044	108339317
ENSG0000013545	0.636323981	####	###	TROAP	protein_codinc	trophinin as	12	49323236	49331731
ENSG0000016778	0.822943545	####	###	ZNF558	protein_codinc	zinc finger p	19	8806170	8832314
ENSG0000012069	-0.450669279	####	###	SMAD9	protein_codinc	SMAD famil	13	36844831	36920765
ENSG0000025557	1.029388767	####	###	MIR9-3HG	lncRNA	MIR9-3 hos	15	89361579	89398487
ENSG0000008137	0.935596639	####	###	CDC14B	protein_codinc	cell division	9	96490241	96619843
ENSG0000013531	0.999718843	####	###	CEP162	protein_codinc	centrosoma	6	84124241	84227643
ENSG0000011179	0.559809548	####	###	FGFR1OP2	protein_codinc	FGFR1 oncc	12	26938470	26966650
ENSG0000001726	-0.386431828	####	###	ATP2C1	protein_codinc	ATPase secr	3	130850595	131016712
ENSG0000016219	-0.539419	####	###	UBXN1	protein_codinc	UBX domair	11	62676498	62679117
ENSG0000015646	0.920214297	####	###	MTERF3	protein_codinc	mitochondr	8	96239398	96261610

ENSG0000024261	0.836914107	####	###	GNG10	protein_codinç	G protein su	9	111661605	111670226
ENSG0000013839	-3.846229201	####	###	CDK15	protein_codinç	cyclin deper	2	201790461	201895550
ENSG0000006632	0.824730004	####	###	ELOVL1	protein_codinç	ELOVL fatty	1	43363398	43368074
ENSG0000018861	1.286240253	####	###	NANOS1	protein_codinç	nanos C2HC	10	119029714	119033730
ENSG0000016294	-1.417823863	####	###	DISC1	protein_codinç	DISC1 scaffo	1	231626790	232041272
ENSG0000012626	-3.896097862	####	###	FFAR2	protein_codinç	free fatty ac	19	35443907	35451767
ENSG0000016272	2.552101284	####	###	TRIM58	protein_codinç	tripartite mo	1	247857187	247880138
ENSG0000023703	1.266420434	####	###	ZEB1-AS1	lncRNA	ZEB1 antiser	10	31206278	31320447
ENSG0000013036	1.310904984	####	###	RSPH3	protein_codinç	radial spoke	6	158972871	159000202
ENSG0000003774	0.655808183	####	###	MFAP3	protein_codinç	microfibril a	5	154038906	154220478
ENSG0000003838	-0.319047244	####	###	TRIO	protein_codinç	trio Rho gua	5	14143342	14532128
ENSG0000011269	0.405504147	####	###	TMEM30A	protein_codinç	transmembı	6	75252924	75284948
ENSG0000011741	-0.807968577	####	###	B4GALT2	protein_codinç	beta-1,4-ga	1	43978943	43991170
ENSG0000023457	4.829685088	####	###	H2BP2	transcribed_un	H2B histone	1	143875171	143904650
ENSG0000006832	-0.723488537	####	###	TFE3	protein_codinç	transcriptior	X	49028726	49043410
ENSG0000016482	0.39362377	####	###	SUN1	protein_codinç	Sad1 and U	7	816615	896435
ENSG0000011761	-0.608171519	####	###	SYF2	protein_codinç	SYF2 pre-m	1	25222276	25232502
ENSG0000012282	1.247997657	####	###	NUDT10	protein_codinç	nudix hydro	X	51332231	51337525
ENSG0000014844	0.716891272	####	###	COMMD3	protein_codinç	COMM don	10	22316386	22320306
ENSG0000015845	-2.36364309	####	###	NRG2	protein_codinç	neuregulin 2	5	139846779	140043299
ENSG0000010111	-2.210082995	####	###	SALL4	protein_codinç	spalt like tra	20	51782331	51802521
ENSG0000011961	0.586302271	####	###	FCF1	protein_codinç	FCF1 rRNA-	14	74713144	74738620
ENSG0000002707	-4.303132324	####	###	PRKCH	protein_codinç	protein kina	14	61187559	61550976
ENSG0000013456	-1.171830429	####	###	LRP4	protein_codinç	LDL recepto	11	46856717	46918642
ENSG0000014801	0.60178988	####	###	CEP78	protein_codinç	centrosoma	9	78236062	78279690
ENSG0000018430	-1.887302961	####	###	CCSER1	protein_codinç	coiled-coil s	4	90127535	91601913
ENSG0000011070	-0.340000256	####	###	RPS13	protein_codinç	ribosomal p	11	17074388	17077715
ENSG0000017016	-2.294157186	####	###	HOXD4	protein_codinç	homeobox I	2	176151550	176153226
ENSG0000019801	-0.585873039	####	###	MRPL42	protein_codinç	mitochondr	12	93467488	93516214
ENSG0000013345	-3.245649749	####	###	MYO18B	protein_codinç	myosin XVII	22	25742144	26031045
ENSG0000009242	0.633163349	####	###	SEMA6A	protein_codinç	semaphorin	5	116443555	116574823
ENSG0000011203	-3.730842683	####	###	OPRM1	protein_codinç	opioid recep	6	154010496	154246867
ENSG0000009220	0.531847011	####	###	TOX4	protein_codinç	TOX high m	14	21476597	21499175
ENSG0000006756	0.409192467	####	###	RHOA	protein_codinç	ras homolog	3	49359145	49412998
ENSG0000017286	-0.611132855	####	###	DMXL1	protein_codinç	Dmx like 1 [	5	119037772	119249138
ENSG0000010059	0.541385804	####	###	SPTLC2	protein_codinç	serine palm	14	77505997	77616637
ENSG0000015339	0.697859806	####	###	LPCAT1	protein_codinç	lysophospha	5	1456480	1523962
ENSG0000011251	-0.81733003	####	###	CUTA	protein_codinç	cutA divaler	6	33416442	33418317
ENSG0000000309	-0.443465847	####	###	KLHL13	protein_codinç	kelch like fa	X	117897813	118117340
ENSG0000016839	-1.706065948	####	###	BDKRB2	protein_codinç	bradykinin r	14	96204679	96244166
ENSG0000013710	1.492027909	####	###	TMEM8B	protein_codinç	transmembı	9	35814451	35865518
ENSG0000010431	-1.208088226	####	###	RIPK2	protein_codinç	receptor int	8	89757806	89791064
ENSG0000014851	-0.384528412	####	###	ZEB1	protein_codinç	zinc finger E	10	31318495	31529814
ENSG0000018490	-0.672912758	####	###	SUMO3	protein_codinç	small ubiqui	21	44805617	44818779
ENSG0000011373	0.658735531	####	###	ATP6V0E1	protein_codinç	ATPase H+	5	172983771	173035445
ENSG0000014894	2.944681658	####	###	LRRC4C	protein_codinç	leucine rich	11	40114203	41459773
ENSG0000009995	-0.704307973	####	###	SMARCB1	protein_codinç	SWI/SNF rel	22	23786931	23838009
ENSG0000011255	-1.985513093	####	###	MDFI	protein_codinç	MyoD family	6	41636882	41654246
ENSG0000013859	0.600783902	####	###	SECISBP2L	protein_codinç	SECIS bindir	15	48988476	49046447
ENSG0000013295	-0.67380654	####	###	USPL1	protein_codinç	ubiquitin sp	13	30617693	30660770
ENSG0000017320	0.699297041	####	###	AHSA2P	transcribed_un	activator of	2	61177418	61191203
ENSG0000012857	2.121749647	####	###	STRIP2	protein_codinç	striatin inter	7	129434432	129488399
ENSG0000010336	0.537458614	####	###	GGA2	protein_codinç	golgi associ	16	23463542	23521995
ENSG0000024759	-0.877622114	####	###	TWF2	protein_codinç	twinfilin acti	3	52228612	52239158
ENSG0000016206	0.631591711	####	###	CCNF	protein_codinç	cyclin F [Sou	16	2429394	2458854
ENSG0000012091	-3.475322392	####	###	PDLIM2	protein_codinç	PDZ and LIN	8	22578279	22598025
ENSG0000015789	-2.013872962	####	###	MEGF11	protein_codinç	multiple EGI	15	65895079	66253747
ENSG0000016539	0.637366055	####	###	WRN	protein_codinç	WRN RecQ	8	31033788	31176138

ENSG0000019653	-1.369218364	####	###	MYO18A	protein_coding	myosin XVII	17	29071124	29180412
ENSG0000015076	-0.836481258	####	###	DIXDC1	protein_coding	DIX domain	11	111927144	112022653
ENSG0000001126	-0.585200017	####	###	UTP18	protein_coding	UTP18 smal	17	51260546	51297936
ENSG0000009999	0.522568877	####	###	CABIN1	protein_coding	calcineurin	22	24011192	24178628
ENSG0000012214	-0.792025556	####	###	MRPS2	protein_coding	mitochondr	9	135499984	135504673
ENSG0000016285	-0.889108842	####	###	TFB2M	protein_coding	transcriptior	1	246540561	246566261
ENSG0000025929	2.144952743	####	###	ZNF710-AS	lncRNA	ZNF710 ant	15	90074512	90082207
ENSG0000018059	2.643260516	####	###	H2BC4	protein_coding	H2B clustere	6	26114873	26123926
ENSG0000021322	1.365111323	####	###	NOC2LP1	transcribed_pr	NOC2 like r	2	130229379	130232106
ENSG0000010582	-0.618451087	####	###	DNAJC2	protein_coding	DnaJ heat sl	7	103312289	103344830
ENSG0000013198	1.241915407	####	###	LGALS3	protein_coding	galectin 3 [S	14	55124110	55145423
ENSG0000019692	0.655263455	####	###	ZNF252P	transcribed_un	zinc finger p	8	144973589	145002895
ENSG0000023216	2.177627936	####	###	RAP2C-AS1	lncRNA	RAP2C antis	X	132217053	132432862
ENSG0000007981	1.888775149	####	###	EPB41L2	protein_coding	erythrocyte	6	130839347	131063322
ENSG0000013115	0.878786839	####	###	GINS2	protein_coding	GINS compl	16	85676198	85690073
ENSG0000007734	-1.26031869	####	###	EXOSC5	protein_coding	exosome cc	19	41386374	41397359
ENSG0000017895	0.569740858	####	###	GAK	protein_coding	cyclin G ass	4	849276	932373
ENSG0000023296	1.721705327	####	###	CSNK2B	protein_coding	casein kinas	CHR_HSCHR	31704690	31711736
ENSG0000014339	-0.400940206	####	###	PIP5K1A	protein_coding	phosphatidy	1	151197949	151249536
ENSG0000023137	0.61394579	####	###	PRRC2A	protein_coding	proline rich	CHR_HSCHR	31602891	31619942
ENSG0000016474	-0.992193433	####	###	ADCY1	protein_coding	adenylate cy	7	45574140	45723116
ENSG0000010858	0.572270459	####	###	CPD	protein_coding	carboxypep	17	30378927	30469989
ENSG0000017004	1.044231268	####	###	TRAPPC1	protein_coding	trafficking p	17	7930345	7932123
ENSG0000004766	-2.43802811	####	###	FAM184B	protein_coding	family with s	4	17629306	17781621
ENSG0000013963	0.997742233	####	###	LMBR1L	protein_coding	limb develo	12	49096551	49110900
ENSG0000015656	-4.640175184	####	###	LRFN2	protein_coding	leucine rich	6	40391591	40587364
ENSG0000019641	0.302705667	####	###	XRCC6	protein_coding	X-ray repair	22	41621163	41664048
ENSG0000019700	-0.447947572	####	###	METTL9	protein_coding	methyltrans	16	21597218	21657473
ENSG0000011988	2.053593939	####	###	EPCAM	protein_coding	epithelial ce	2	47345158	47387601
ENSG0000000829	0.335963379	####	###	SPAG9	protein_coding	sperm assoc	17	50962174	51120868
ENSG0000017284	-0.659183814	####	###	PDP2	protein_coding	pyruvate de	16	66878589	66895754
ENSG0000011258	0.967545986	####	###	FAM120B	protein_coding	family with s	6	170290703	170407067
ENSG0000011568	0.948757123	####	###	PASK	protein_coding	PAS domair	2	241106099	241150264
ENSG0000012478	-1.683717925	####	###	RREB1	protein_coding	ras responsi	6	7107597	7251980
ENSG0000019797	-0.904727292	####	###	AKAP17A	protein_coding	A-kinase an	X	1591604	1602520
ENSG0000019807	-2.008184998	####	###	SULT1C4	protein_coding	sulfotransfe	2	108377911	108388989
ENSG0000018774	0.757460382	####	###	FANCA	protein_coding	FA complen	16	89737549	89816657
ENSG0000017695	0.702659634	####	###	NFATC2IP	protein_coding	nuclear fact	16	28950807	28967097
ENSG0000013867	-3.220604603	####	###	GPAT3	protein_coding	glycerol-3-p	4	83535914	83605875
ENSG0000023211	-4.657524021	####	###	AL353751.1	lncRNA	novel transc	10	89283765	89292125
ENSG0000015207	-1.719361125	####	###	TLCD4	protein_coding	TLC domain	1	95117355	95197607
ENSG0000024755	-0.295489426	####	###	OIP5-AS1	lncRNA	OIP5 antiser	15	41283990	41309737
ENSG0000017389	0.549356636	####	###	CBX2	protein_coding	chromobox	17	79778148	79787983
ENSG0000017032	-0.885449688	####	###	PRDM10	protein_coding	PR/SET dom	11	129899706	130002835
ENSG0000016063	-0.35577904	####	###	SAFB	protein_coding	scaffold atta	19	5623035	5668478
ENSG0000010592	-0.672059511	####	###	MPP6	protein_coding	membrane p	7	24573268	24694193
ENSG0000013562	0.300503978	####	###	CCT7	protein_coding	chaperonin	2	73233420	73253021
ENSG0000011485	-3.235642849	####	###	ZBTB47	protein_coding	zinc finger a	3	42653697	42667580
ENSG0000007836	0.305201234	####	###	GNB1	protein_coding	G protein su	1	1785285	1891117
ENSG0000018003	-0.784640134	####	###	ZNF48	protein_coding	zinc finger p	16	30378106	30400108
ENSG0000014240	-1.752200265	####	###	CACNG8	protein_coding	calcium volt	19	53963040	53990215
ENSG0000022340	-1.861426337	####	###	MEG9	lncRNA	maternally e	14	101068283	101072937
ENSG0000014340	-1.193193164	####	###	MINDY1	protein_coding	MINDY lysir	1	150996086	151008375
ENSG0000015511	0.514324096	####	###	CDK19	protein_coding	cyclin deper	6	110609978	110815958
ENSG0000027376	0.572084301	####	###	DDX24	protein_coding	DEAD-box I	CHR_HSCHR	94050920	94081245
ENSG0000010064	0.953336867	####	###	SUSD6	protein_coding	sushi domai	14	69611596	69715144
ENSG0000012089	-1.739536294	####	###	SORBS3	protein_coding	sorbin and S	8	22544986	22575788
ENSG0000010029	1.52789154	####	###	ARSA	protein_coding	arylsulfatase	22	50622754	50628173

ENSG0000013284	-2.705709151	####	###	PATJ	protein_coding	PATJ crumb	1	61742477	62178675
ENSG0000023389	1.859892249	####	###	TCF19	protein_coding	transcriptior	CHR_HSCHR	31152269	31157938
ENSG0000010491	-0.749140173	####	###	STX10	protein_coding	syntaxin 10	19	13144058	13150383
ENSG0000010434	0.562591593	####	###	UBE2W	protein_coding	ubiquitin co	8	73780097	73878910
ENSG0000012254	0.338169333	####	###	SEPTIN7	protein_coding	septin 7 [So	7	35800932	35907105
ENSG0000015414	0.693946019	####	###	TBRG1	protein_coding	transforming	11	124622836	124635926
ENSG0000014220	0.5888804	####	###	URB1	protein_coding	URB1 ribosc	21	32311018	32393012
ENSG0000016078	-0.957669738	####	###	PMF1	protein_coding	polyamine r	1	156212993	156240042
ENSG0000019796	-0.432504745	####	###	VPS13A	protein_coding	vacuolar prc	9	77177445	77421537
ENSG0000017046	-0.902320738	####	###	DNAJC18	protein_coding	DnaJ heat sl	5	139408588	139444491
ENSG0000014245	1.049136697	####	###	EVI5L	protein_coding	ecotropic vi	19	7830233	7864976
ENSG0000011356	0.461284063	####	###	NUP155	protein_coding	nucleoporin	5	37288137	37371106
ENSG0000010913	-0.519287328	####	###	PHOX2B	protein_coding	paired like h	4	41744082	41748725
ENSG0000015964	1.052911963	####	###	ACE	protein_coding	angiotensin	17	63477061	63498380
ENSG0000013979	-0.489328491	####	###	MBNL2	protein_coding	muscleblind	13	97221434	97394120
ENSG0000017003	0.77417921	####	###	CNTROB	protein_coding	centrobin, c	17	7932101	7949920
ENSG0000019674	1.337923799	####	###	GM2A	protein_coding	GM2 gangli	5	151212150	151270440
ENSG0000006588	-0.569000169	####	###	CDK13	protein_coding	cyclin deper	7	39950121	40099580
ENSG0000017105	0.755272772	####	###	FEZ2	protein_coding	fasciculatior	2	36531805	36646087
ENSG0000016925	-0.853596076	####	###	B3GALNT1	protein_coding	beta-1,3-N	3	161083883	161105411
ENSG0000012442	0.330123747	####	###	USP22	protein_coding	ubiquitin sp	17	20999596	21043760
ENSG0000013477	-1.123422813	####	###	FHOD3	protein_coding	formin hom	18	36297714	36780055
ENSG0000008788	1.10845763	####	###	AAMDC	protein_coding	adipogenes	11	77821109	77918432
ENSG0000011327	-0.999452725	####	###	THG1L	protein_coding	tRNA-histid	5	157731420	157741449
ENSG0000006597	0.291933777	####	###	YBX1	protein_coding	Y-box bindi	1	42682418	42703805
ENSG0000013291	-0.397446302	####	###	DCTN4	protein_coding	dynactin sul	5	150708440	150759095
ENSG0000019716	-1.083231078	####	###	ZNF785	protein_coding	zinc finger p	16	30573740	30585769
ENSG0000018562	0.47895716	####	###	PSMD13	protein_coding	proteasome	11	236966	252984
ENSG0000010231	-0.367562483	####	###	RBM3	protein_coding	RNA binding	X	48574449	48581162
ENSG0000015210	0.36534137	####	###	FAM168B	protein_coding	family with s	2	131047876	131093460
ENSG0000014483	-1.239518831	####	###	TAGLN3	protein_coding	transgelin 3	3	111998739	112013887
ENSG0000011760	-0.866786592	####	###	PLPPR4	protein_coding	phospholipi	1	99263953	99309590
ENSG0000010013	-0.91380385	####	###	MICALL1	protein_coding	MICAL like 1	22	37905657	37942822
ENSG0000005805	0.574943577	####	###	USP13	protein_coding	ubiquitin sp	3	179653040	179789401
ENSG0000016240	-2.183117752	####	###	PRKAA2	protein_coding	protein kina	1	56645314	56715335
ENSG0000011457	0.507055653	####	###	ATP6V1A	protein_coding	ATPase H+	3	113747033	113812056
ENSG0000016384	-1.374967726	####	###	DTX3L	protein_coding	deltex E3 ub	3	122564338	122575203
ENSG0000016273	0.622706635	####	###	PEX19	protein_coding	peroxisoma	1	160276807	160286348
ENSG0000016347	-0.39574563	####	###	SSR2	protein_coding	signal seque	1	156009048	156020951
ENSG0000010452	2.390669118	####	###	PYCR3	protein_coding	pyrroline-5-	8	143603210	143609773
ENSG0000020506	0.556393731	####	###	SLC35B4	protein_coding	solute carrie	7	134289332	134316930
ENSG0000011497	0.460904714	####	###	MOB1A	protein_coding	MOB kinase	2	74152528	74178898
ENSG0000013707	0.799454598	####	###	APTX	protein_coding	aprataxin [S	9	32886601	33025130
ENSG0000024833	0.546083832	####	###	CDK11B	protein_coding	cyclin deper	1	1635227	1659012
ENSG0000011625	-0.257803856	####	###	RPL22	protein_coding	ribosomal p	1	6185020	6209389
ENSG0000017586	0.99376449	####	###	BAIAP2	protein_coding	BAR/IMD do	17	81035122	81117432
ENSG0000011069	-0.870144974	####	###	PITPNM1	protein_coding	phosphatidy	11	67491768	67506263
ENSG0000013246	-0.387234899	####	###	ANKRD17	protein_coding	ankyrin repe	4	73073376	73258798
ENSG0000011699	-3.519820115	####	###	MYCL	protein_coding	MYCL proto	1	39895426	39902256
ENSG0000018182	-0.479273965	####	###	RFX7	protein_coding	regulatory fa	15	56087280	56245082
ENSG0000017469	0.417513418	####	###	TMEM167A	protein_coding	transmembr	5	83052846	83077863
ENSG0000002577	0.685636945	####	###	TOMM34	protein_coding	translocase	20	44942130	44960397
ENSG0000007606	-1.081192538	####	###	RBMS2	protein_coding	RNA binding	12	56521820	56596193
ENSG0000013917	-2.422701424	####	###	TMEM117	protein_coding	transmembr	12	43835967	44389762
ENSG0000015866	0.414324852	####	###	GPAT4	protein_coding	glycerol-3-p	8	41577187	41625001
ENSG0000014834	0.813689439	####	###	SH3GLB2	protein_coding	SH3 domair	9	129007036	129028331
ENSG0000027803	1.163938504	####	###	LY6E	protein_coding	lymphocyte	CHR_HSCHR	143017982	143023832
ENSG0000009056	0.839189743	####	###	RAB11FIP3	protein_coding	RAB11 fami	16	425649	523011

ENSG0000010594	1.06795122	####	###	TTC26	protein_coding	tetratricope	7	139133744	139191986
ENSG0000005337	0.943869248	####	###	AKR7A2	protein_coding	aldo-keto re	1	19303965	19312146
ENSG0000010240	0.606297492	####	###	BEX4	protein_coding	brain expres	X	103215108	103217246
ENSG0000012088	0.505457216	####	###	CLU	protein_coding	clusterin [Sc	8	27596917	27614700
ENSG0000011364	0.417927055	####	###	RARS1	protein_coding	arginyl-tRN	5	168486451	168519301
ENSG0000016396	0.579950319	####	###	RNF168	protein_coding	ring finger p	3	196468783	196503768
ENSG0000018905	0.483186786	####	###	RELN	protein_coding	reelin [Sour	7	103471784	103989658
ENSG0000018175	-3.121746899	####	###	AMIGO1	protein_coding	adhesion m	1	109504178	109509738
ENSG0000014267	-0.319090454	####	###	RPL11	protein_coding	ribosomal p	1	23691742	23696835
ENSG0000012537	-3.253121857	####	###	BMP4	protein_coding	bone morph	14	53949736	53958761
ENSG0000026753	-2.495747838	####	###	S1PR2	protein_coding	sphingosine	19	10221433	10231331
ENSG0000027665	2.445450874	####	###	PYCR3	protein_coding	pyrroline-5-	CHR_HSCHR	143603913	143609773
ENSG0000010140	2.027159876	####	###	SNTA1	protein_coding	syntrophin a	20	33407957	33443763
ENSG0000023790	-2.522587088	####	###	ZBTB12	protein_coding	zinc finger a	CHR_HSCHR	31887151	31889536
ENSG0000016817	0.482994032	####	###	HOOK3	protein_coding	hook microt	8	42896946	43030535
ENSG0000011531	-1.376641741	####	###	LOXL3	protein_coding	lysyl oxidase	2	74532258	74555690
ENSG0000016981	-0.35477057	####	###	HNRNPF	protein_coding	heterogene	10	43385617	43409166
ENSG0000011531	0.74441047	####	###	HTRA2	protein_coding	HtrA serine	2	74529725	74533332
ENSG0000017065	0.560305879	####	###	ATF7	protein_coding	activating tr	12	53507856	53626410
ENSG0000018898	1.158679126	####	###	DHFRP1	processed_pse	dihydrofolat	18	26170726	26171284
ENSG0000015825	-1.452018831	####	###	CLSTN2	protein_coding	calsyntenin	3	139935185	140577397
ENSG0000016971	-0.342126387	####	###	FASN	protein_coding	fatty acid sy	17	82078338	82098294
ENSG0000010965	0.387901394	####	###	TRIM2	protein_coding	tripartite mo	4	153152163	153339319
ENSG0000018899	-0.425492006	####	###	ZNF292	protein_coding	zinc finger p	6	87152833	87265943
ENSG0000012254	2.028280882	####	###	EEPD1	protein_coding	endonuclea	7	36153254	36301538
ENSG0000014134	0.73329499	####	###	G6PC3	protein_coding	glucose-6-p	17	44070735	44076344
ENSG0000013592	0.843187427	####	###	DNAJB2	protein_coding	DnaJ heat sl	2	219279342	219286898
ENSG0000022661	0.566252546	####	###	PRRC2A	protein_coding	proline rich	CHR_HSCHR	31660168	31677219
ENSG0000016517	-0.496534073	####	###	MID1IP1	protein_coding	MID1 intera	X	38801432	38806537
ENSG0000023069	2.679755176	####	###	AL645608.2	lncRNA	novel transc	1	911435	914948
ENSG0000011865	0.968380378	####	###	DCLRE1B	protein_coding	DNA cross-	1	113905213	113914086
ENSG0000006258	-0.770469719	####	###	MRPS24	protein_coding	mitochondr	7	43866558	43869893
ENSG0000013394	-1.039307132	####	###	DGLUCY	protein_coding	D-glutamat	14	91060333	91225632
ENSG0000022967	1.937201279	####	###	ZNF492	protein_coding	zinc finger p	19	22634324	22667671
ENSG0000014316	-0.773019753	####	###	CREG1	protein_coding	cellular repr	1	167529117	167553805
ENSG0000016453	0.936415254	####	###	TBX20	protein_coding	T-box trans	7	35202430	35254100
ENSG0000018940	0.293103875	####	###	HMGB1	protein_coding	high mobilit	13	30456704	30617597
ENSG0000014412	1.423115375	####	###	TMEM177	protein_coding	transmembr	2	119679167	119686507
ENSG0000019845	-0.976628139	####	###	ZXDB	protein_coding	zinc finger X	X	57591652	57597545
ENSG0000007357	0.476114008	####	###	SDHA	protein_coding	succinate de	5	218241	257082
ENSG0000016029	1.116685561	####	###	C21orf58	protein_coding	chromosom	21	46300181	46323875
ENSG0000005972	-0.645138005	####	###	MXD1	protein_coding	MAX dimeri	2	69897688	69942945
ENSG0000018039	-0.405665672	####	###	MCFD2	protein_coding	multiple coa	2	46901870	46941855
ENSG0000019785	-3.797260665	####	###	ADAMTSL2	protein_coding	ADAMTS lik	9	133532164	133575519
ENSG0000019611	1.798170506	####	###	ZNF699	protein_coding	zinc finger p	19	9291140	9309838
ENSG0000000984	0.538419464	####	###	VTA1	protein_coding	vesicle traffi	6	142147162	142224685
ENSG0000015476	0.824141646	####	###	XPC	protein_coding	XPC comple	3	14145147	14178621
ENSG0000018275	-2.315235999	####	###	PAPPA	protein_coding	pappalysin 1	9	116153791	116402321
ENSG0000014306	-0.460696443	####	###	IGSF3	protein_coding	immunoglol	1	116574399	116667755
ENSG0000017911	-0.597698599	####	###	SPTY2D1	protein_coding	SPT2 chrom	11	18606403	18634791
ENSG0000014085	-1.600733877	####	###	KIFC3	protein_coding	kinesin fami	16	57758217	57863053
ENSG0000001163	-0.517772129	####	###	CYP51A1	protein_coding	cytochrome	7	92112153	92134803
ENSG0000016997	0.62095613	####	###	SF3B5	protein_coding	splicing fact	6	144094884	144095573
ENSG0000026633	-0.58903719	####	###	NBPF15	protein_coding	NBPF memb	1	144421386	144461674
ENSG0000008928	-0.645576225	####	###	IGBP1	protein_coding	immunoglol	X	70133447	70166324
ENSG0000012994	-2.546625367	####	###	SHC2	protein_coding	SHC adapto	19	416583	460996
ENSG0000013225	-1.291606619	####	###	TRIM5	protein_coding	tripartite mo	11	5663195	5938619
ENSG0000017559	-3.754962787	####	###	FOSL1	protein_coding	FOS like 1, A	11	65892049	65900573

ENSG0000014740	-0.258032653	####	###	RPL10	protein_coding	ribosomal p	X	154389955	154409168
ENSG0000011775	0.67857614	####	###	STX12	protein_coding	syntaxin 12	1	27773219	27824443
ENSG0000017722	0.710062564	####	###	GATD1	protein_coding	glutamine a	11	767220	777488
ENSG0000016391	0.698716777	####	###	RFC4	protein_coding	replication f	3	186789880	186807058
ENSG0000009609	0.968499871	####	###	EFHC1	protein_coding	EF-hand do	6	52362123	52529886
ENSG0000020597	-0.858935034	####	###	NYNRIN	protein_coding	NYN domai	14	24399003	24419283
ENSG0000016772	1.885723858	####	###	SRR	protein_coding	serine racen	17	2303383	2325260
ENSG0000015398	-0.398982488	####	###	NUS1	protein_coding	NUS1 dehyc	6	117675469	117710727
ENSG0000027969	-1.431118006	####	###	AP001273.1	TEC	novel transc	11	93726654	93729805
ENSG0000015318	0.24908825	####	###	HNRNPU	protein_coding	heterogene	1	244840638	244864560
ENSG0000009244	-1.447561523	####	###	TYRO3	protein_coding	TYRO3 prot	15	41557675	41583589
ENSG0000011580	-0.531649726	####	###	GORASP2	protein_coding	golgi reasse	2	170928464	170967130
ENSG0000026023	1.769762088	####	###	KDM7A-DT	lncRNA	KDM7A dive	7	140177184	140179640
ENSG0000020554	0.576762521	####	###	TMSB4X	protein_coding	thymosin be	X	12975110	12977227
ENSG0000013498	-0.334361971	####	###	NREP	protein_coding	neuronal re	5	111662621	111997464
ENSG0000010830	-0.705025958	####	###	RUNDC3A	protein_coding	RUN domai	17	44308413	44318670
ENSG0000013809	0.49299031	####	###	CENPO	protein_coding	centromere	2	24793136	24822376
ENSG0000015163	-1.775714543	####	###	AKR1C2	protein_coding	aldo-keto re	10	4987400	5018031
ENSG0000011473	-1.596891725	####	###	MAPKAPK3	protein_coding	MAPK activa	3	50611520	50649291
ENSG0000015828	-1.572201863	####	###	RNF207	protein_coding	ring finger p	1	6205475	6221299
ENSG0000017788	0.411444095	####	###	UBE2N	protein_coding	ubiquitin co	12	93405673	93441947
ENSG0000018710	-0.264023013	####	###	NAP1L1	protein_coding	nucleosome	12	76036585	76084735
ENSG0000016197	-0.282087648	####	###	RPL26	protein_coding	ribosomal p	17	8377516	8383213
ENSG0000010566	0.514307286	####	###	COPE	protein_coding	COPI coat c	19	18899514	18919387
ENSG0000014397	0.596818762	####	###	SNRPG	protein_coding	small nuclea	2	70281362	70293740
ENSG0000011188	0.621244508	####	###	RNGTT	protein_coding	RNA guanyl	6	88609897	88963618
ENSG0000024034	-0.972853068	####	###	PPIL3	protein_coding	peptidylprol	2	200870907	200889303
ENSG0000019796	0.37898153	####	###	MPZL1	protein_coding	myelin prote	1	167721192	167791919
ENSG0000024155	0.642537144	####	###	ARPC4	protein_coding	actin relatec	3	9792495	9807726
ENSG0000011473	-0.833055339	####	###	ACVR2B	protein_coding	activin A rec	3	38453890	38493142
ENSG0000007938	-0.710730026	####	###	SENP1	protein_coding	SUMO spec	12	48042897	48106079
ENSG0000017179	0.688150857	####	###	RHNO1	protein_coding	RAD9-HUS1	12	2876258	2889524
ENSG0000007284	0.836641989	####	###	EVC	protein_coding	EvC ciliary c	4	5711201	5814305
ENSG0000024591	-0.642313842	####	###	SNHG6	lncRNA	small nucle	8	66921684	66926398
ENSG0000022513	1.363749358	####	###	SLC9A3-AS	lncRNA	SLC9A3 ant	5	473236	480884
ENSG0000011702	-0.430032067	####	###	AKT3	protein_coding	AKT serine/t	1	243488233	243851079
ENSG0000013507	0.562567368	####	###	ISCA1	protein_coding	iron-sulfur c	9	86264546	86283102
ENSG0000011004	1.131373603	####	###	ATG2A	protein_coding	autophagy r	11	64894546	64917211
ENSG0000016761	1.026404613	####	###	LENG8	protein_coding	leukocyte re	19	54448887	54462037
ENSG0000010437	1.104229334	####	###	STK3	protein_coding	serine/threc	8	98371228	98942827
ENSG0000012241	1.169525033	####	###	ODF2L	protein_coding	outer dense	1	86346824	86396342
ENSG0000025573	-1.918429632	####	###	AGAP2-AS1	lncRNA	AGAP2 antis	12	57726271	57728356
ENSG0000009282	0.376505304	####	###	EZR	protein_coding	ezrin [Sourc	6	158765741	158819368
ENSG0000015099	-0.77980315	####	###	DHX37	protein_coding	DEAH-box l	12	124946825	124989131
ENSG0000018884	-0.844798158	####	###	BEND4	protein_coding	BEN domair	4	42110853	42152878
ENSG0000018542	-0.781709177	####	###	SMYD3	protein_coding	SET and MY	1	245749342	246507312
ENSG0000017194	0.773117995	####	###	SRGAP2C	protein_coding	SLIT-ROBO	1	121184811	121392874
ENSG0000006984	-0.42559613	####	###	ATP1B3	protein_coding	ATPase Na+	3	141876124	141926549
ENSG0000021411	1.026235158	####	###	MYCBP	protein_coding	MYC bindin	1	38862493	38873368
ENSG0000018725	-0.524325374	####	###	RSBN1L	protein_coding	round sperr	7	77696459	77783022
ENSG0000020386	-0.448198848	####	###	RBM20	protein_coding	RNA binding	10	110644336	110839468
ENSG0000011396	-0.965850992	####	###	ARL6	protein_coding	ADP ribosyl	3	97764521	97801229
ENSG0000013623	-2.5038304	####	###	GPNMB	protein_coding	glycoprotein	7	23235967	23275108
ENSG0000017083	-1.023929273	####	###	GPR27	protein_coding	G protein-c	3	71753855	71756496
ENSG0000013077	-0.560719248	####	###	CLIP1	protein_coding	CAP-Gly do	12	122271432	122422632
ENSG0000016777	-0.689386281	####	###	CD320	protein_coding	CD320 mole	19	8302127	8308358
ENSG0000013400	0.389808432	####	###	EIF2S1	protein_coding	eukaryotic t	14	67360151	67386516
ENSG0000009233	0.858685133	####	###	TINF2	protein_coding	TERF1 intera	14	24239643	24242674

ENSG0000021519	0.593726281	####	###	PEX26	protein_coding	peroxisoma	22	18077920	18131138
ENSG0000027583	0.832872091	####	###	TUBGCP5	protein_coding	tubulin gam	15	22983192	23039572
ENSG0000017897	-0.655035725	####	###	FBXO34	protein_coding	F-box prote	14	55271421	55361918
ENSG0000014586	-0.514358875	####	###	FBXO38	protein_coding	F-box prote	5	148383935	148442836
ENSG0000016001	-10.44738142	####	###	PTGIR	protein_coding	prostagland	19	46620468	46625089
ENSG0000018556	1.468920925	####	###	AHNAK2	protein_coding	AHNAK nuc	14	104937244	104978374
ENSG0000001120	1.919914097	####	###	ANOS1	protein_coding	anosmin 1 [	X	8528874	8732137
ENSG0000012235	-0.808620924	####	###	ANXA11	protein_coding	annexin A11	10	80150889	80205572
ENSG0000016853	0.597238027	####	###	TRAPPC11	protein_coding	trafficking p	4	183659267	183713594
ENSG0000006970	-2.272593302	####	###	TGFBR3	protein_coding	transforming	1	91680343	91906335
ENSG0000016457	1.536226901	####	###	GALNT10	protein_coding	polypeptide	5	154190730	154420984
ENSG0000018732	0.63471655	####	###	TAF9B	protein_coding	TATA-box b	X	78129748	78139650
ENSG0000027288	-0.583157634	####	###	LINC01578	lncRNA	long interge	15	92819540	92899701
ENSG0000002602	0.291817223	####	###	VIM	protein_coding	vimentin [Sc	10	17228241	17237593
ENSG0000011625	2.233471445	####	###	CHD5	protein_coding	chromodom	1	6101787	6180321
ENSG0000016324	0.804610075	####	###	CCNYL1	protein_coding	cyclin Y like	2	207711540	207761839
ENSG0000009929	-0.508966485	####	###	WASHC2A	protein_coding	WASH comp	10	50067888	50133509
ENSG0000010604	0.872543707	####	###	HIBADH	protein_coding	3-hydroxyis	7	27525442	27662883
ENSG0000017153	0.741176597	####	###	MAP6	protein_coding	microtubule	11	75586918	75669120
ENSG0000012766	-1.887766136	####	###	TICAM1	protein_coding	toll like rece	19	4815932	4831712
ENSG0000023565	2.184403254	####	###	FBXO30-D	lncRNA	FBXO30 div	6	145799409	145887430
ENSG0000017024	0.406576246	####	###	USP47	protein_coding	ubiquitin sp	11	11841423	11961887
ENSG0000007008	0.33008091	####	###	PFN2	protein_coding	profilin 2 [Sc	3	149964904	150050788
ENSG0000010614	0.541932155	####	###	CASP2	protein_coding	caspase 2 [S	7	143288215	143307696
ENSG0000017911	-0.450654086	####	###	FARSA	protein_coding	phenylalany	19	12922479	12934037
ENSG0000019704	1.509713479	####	###	ZNF441	protein_coding	zinc finger p	19	11767000	11784078
ENSG0000019784	-1.082334579	####	###	ZNF181	protein_coding	zinc finger p	19	34734155	34745378
ENSG0000010080	-0.456076981	####	###	PSMB5	protein_coding	proteasome	14	23016543	23035230
ENSG0000018127	-1.181766207	####	###	FRAT2	protein_coding	FRAT regula	10	97332497	97334729
ENSG0000019896	-0.602353108	####	###	RORB	protein_coding	RAR related	9	74497335	74693177
ENSG0000012924	4.410954655	####	###	ATP1B2	protein_coding	ATPase Na+	17	7646627	7657770
ENSG0000013980	1.4982475	####	###	ZIC5	protein_coding	Zic family m	13	99962964	99971909
ENSG0000016360	-0.37085352	####	###	PPP4R2	protein_coding	protein pho	3	72996803	73069198
ENSG0000016429	1.191175963	####	###	TIGD6	protein_coding	tigger trans	5	149993118	150000654
ENSG0000011164	0.209404672	####	###	GAPDH	protein_coding	glyceraldeh	12	6534512	6538374
ENSG0000006613	-0.712265388	####	###	NFYC	protein_coding	nuclear tran	1	40691648	40771603
ENSG0000010626	1.048428996	####	###	NUDT1	protein_coding	nudix hydro	7	2242222	2251146
ENSG0000010834	0.42674403	####	###	PSMD3	protein_coding	proteasome	17	39980807	39997959
ENSG0000009531	0.430639068	####	###	NUP188	protein_coding	nucleoporin	9	128947699	129007096
ENSG0000007995	0.720189845	####	###	STX7	protein_coding	syntaxin 7 [S	6	132445867	132513198
ENSG0000010222	0.484731357	####	###	CDK16	protein_coding	cyclin deper	X	47217860	47229997
ENSG0000013715	-0.221611181	####	###	RPS6	protein_coding	ribosomal p	9	19375715	19380236
ENSG0000008598	0.610762772	####	###	USP40	protein_coding	ubiquitin sp	2	233475520	233566782
ENSG0000011849	3.686658724	####	###	PLAGL1	protein_coding	PLAG1 like z	6	143940300	144064599
ENSG0000012174	-0.596633031	####	###	TBC1D15	protein_coding	TBC1 doma	12	71839707	71927248
ENSG0000010477	0.829134894	####	###	MAN2B1	protein_coding	mannosidas	19	12646511	12666742
ENSG0000018904	0.424497908	####	###	NDUFA4	protein_coding	NDUFA4 mi	7	10931943	10940153
ENSG0000010663	0.416683362	####	###	YKT6	protein_coding	YKT6 v-SNA	7	44200968	44214294
ENSG0000027533	0.868129429	####	###	FAM120B	protein_coding	family with s	CHR_HSCHR	170290703	170416388
ENSG0000017329	1.763718381	####	###	FAM86B3P	transcribed_un	family with s	8	8228595	8244865
ENSG0000020436	-2.508058536	####	###	ZBTB12	protein_coding	zinc finger a	6	31899613	31902086
ENSG0000027668	1.157553824	####	###	LENG8	protein_coding	leukocyte re	CHR_HSCHR	54456812	54469943
ENSG0000015574	0.656423895	####	###	FAM126B	protein_coding	family with s	2	200973718	201071671
ENSG0000012309	-5.0614165	####	###	BHLHE41	protein_coding	basic helix-l	12	26120030	26125037
ENSG0000005826	-0.309970487	####	###	SEC61A1	protein_coding	SEC61 trans	3	128051641	128071683
ENSG0000015473	-2.807141375	####	###	ADAMTS5	protein_coding	ADAM meta	21	26917922	26967088
ENSG0000014268	-0.887262146	####	###	C1orf216	protein_coding	chromosom	1	35713877	35718894
ENSG0000018407	0.676921222	####	###	UQCR10	protein_coding	ubiquinol-c	22	29767369	29770413

ENSG0000018492	-0.864256495	####	###	PTRHD1	protein_coding	peptidyl-tRI	2	24789734	24793382
ENSG0000014815	0.750275806	####	###	SNX30	protein_coding	sorting nexi	9	112750760	112881671
ENSG0000017366	-0.405905978	####	###	UQCRH	protein_coding	ubiquinol-c	1	46303698	46316776
ENSG0000016599	-0.392973666	####	###	ARL5B	protein_coding	ADP ribosyl	10	18659431	18681639
ENSG0000012050	0.837243385	####	###	PDZD11	protein_coding	PDZ domair X		70286595	70290514
ENSG0000015886	0.835679176	####	###	FAM160B2	protein_coding	family with s	8	22089150	22104911
ENSG0000008081	0.784644979	####	###	CPOX	protein_coding	coproporph	3	98579446	98593648
ENSG0000013274	-2.238418019	####	###	TESMIN	protein_coding	testis expres	11	68707440	68751520
ENSG0000014725	-4.15716787	####	###	GPC3	protein_coding	glypican 3 [X		133535745	133985594
ENSG0000016781	-0.431904016	####	###	PRDX2	protein_coding	peroxiredox	19	12796820	12801800
ENSG0000016711	0.787131513	####	###	COQ4	protein_coding	coenzyme C	9	128322544	128334072
ENSG0000022491	1.259191175	####	###	LINC00863	lncRNA	long interge	10	87341685	87357882
ENSG0000017913	0.494548299	####	###	SAMD4B	protein_coding	sterile alpha	19	39342396	39385710
ENSG0000025512	2.943289626	####	###	CCDC84-D	lncRNA	CCDC84 div	11	118994824	118998004
ENSG0000010231	-1.92065295	####	###	PORCN	protein_coding	porcupine C X		48508962	48520814
ENSG0000011376	-0.974480007	####	###	UNC5A	protein_coding	unc-5 netrir	5	176810519	176880898
ENSG0000014624	-1.070056626	####	###	TPBG	protein_coding	trophoblast	6	82363206	82367420
ENSG0000010099	0.667656444	####	###	ABHD12	protein_coding	abhydrolase	20	25294742	25390835
ENSG0000003244	-0.649581484	####	###	PNPLA6	protein_coding	patatin like	19	7534004	7561764
ENSG0000015124	0.471314147	####	###	EIF4E	protein_coding	eukaryotic t	4	98871684	98930637
ENSG0000016046	-1.913266248	####	###	SPTBN4	protein_coding	spectrin bet	19	40466241	40576464
ENSG0000010983	-1.457966719	####	###	DDX25	protein_coding	DEAD-box I	11	125903348	125943702
ENSG0000023055	-0.578293472	####	###	AC021078.	lncRNA	novel transc	5	149494314	149504670
ENSG0000018668	-1.870371988	####	###	CYP27C1	protein_coding	cytochrome	2	127183832	127220313
ENSG0000006714	-0.495757584	####	###	NEO1	protein_coding	neogenin 1	15	73051710	73305205
ENSG0000013246	0.618804366	####	###	UTP3	protein_coding	UTP3 small	4	70688532	70690551
ENSG0000025372	0.265123302	####	###	PRKDC	protein_coding	protein kina	8	47773111	47960178
ENSG0000011986	-0.605598862	####	###	BCL11A	protein_coding	BAF chroma	2	60450520	60554467
ENSG0000014757	-10.32302655	####	###	CRH	protein_coding	corticotropi	8	66176376	66178464
ENSG0000014829	0.907072095	####	###	SURF1	protein_coding	SURF1 cytoc	9	133351758	133356676
ENSG0000007698	-0.557364284	####	###	MAP2K7	protein_coding	mitogen-ac	19	7903843	7914478
ENSG0000013081	-0.644185754	####	###	PPAN	protein_coding	peter pan h	19	10106362	10112012
ENSG0000016252	-0.294852925	####	###	RBBP4	protein_coding	RB binding	1	32651142	32686211
ENSG0000013686	-2.719154233	####	###	TLR4	protein_coding	toll like rece	9	117704175	117724735
ENSG0000014963	0.648373159	####	###	DSN1	protein_coding	DSN1 comp	20	36751791	36773818
ENSG0000018776	-1.016398802	####	###	SEMA4D	protein_coding	semaphorin	9	89360787	89498130
ENSG0000007943	-0.605643709	####	###	CIC	protein_coding	capicua trar	19	42268537	42295797
ENSG0000016304	0.343000098	####	###	H3-3A	protein_coding	H3.3 histone	1	226061851	226073212
ENSG0000018620	-1.135456813	####	###	MTARC1	protein_coding	mitochondr	1	220786913	220819659
ENSG0000013070	0.571145418	####	###	ADRM1	protein_coding	adhesion re	20	62302093	62308862
ENSG0000019718	-0.673811555	####	###	NOL4L	protein_coding	nucleolar pr	20	32443059	32585074
ENSG0000010057	1.102154644	####	###	GSTZ1	protein_coding	glutathione	14	77320996	77331597
ENSG0000015598	-0.648630701	####	###	KIF5A	protein_coding	kinesin fami	12	57546026	57586633
ENSG0000003317	0.523714505	####	###	UBA6	protein_coding	ubiquitin lik	4	67612652	67701155
ENSG0000015300	-0.733010965	####	###	SREK1IP1	protein_coding	SREK1 intera	5	64718148	64768691
ENSG0000014866	-0.60435878	####	###	CAMK2G	protein_coding	calcium/calr	10	73812501	73874591
ENSG0000013267	0.589235195	####	###	PTPRA	protein_coding	protein tyro	20	2864184	3039076
ENSG0000017226	-0.604102038	####	###	DPAGT1	protein_coding	dolichyl-pho	11	119096503	119108331
ENSG0000026307	1.255822001	####	###	ZNF213-AS	lncRNA	ZNF213 ant	16	3100696	3143734
ENSG0000017816	-0.744301805	####	###	ZNF518B	protein_coding	zinc finger p	4	10439880	10457426
ENSG0000019868	0.83556364	####	###	SLC9A6	protein_coding	solute carrie X		135973841	136047269
ENSG0000019851	0.808969785	####	###	ATL1	protein_coding	atlastin GTP	14	50532509	50634017
ENSG0000017684	3.409822504	####	###	MIR7-3HG	lncRNA	MIR7-3 hos	19	4769031	4785077
ENSG0000009026	0.668110781	####	###	NDUFB2	protein_coding	NADH:ubiqu	7	140690777	140722790
ENSG0000009006	0.736833568	####	###	CCNK	protein_coding	cyclin K [Sou	14	99481169	99535044
ENSG0000021369	1.095437754	####	###	S1PR3	protein_coding	sphingosine	9	88990863	89005155
ENSG0000013693	-0.896997897	####	###	RABEPK	protein_coding	Rab9 effecto	9	125200542	125234161
ENSG0000014683	-0.616533394	####	###	MEPCE	protein_coding	methylphos	7	100428790	100434118

ENSG0000011374	-0.47567965	####	###	CPEB4	protein_coding	cytoplasmic	5	173888349	173961980
ENSG0000011626	0.767129096	####	###	QSOX1	protein_coding	quiescin sul	1	180154869	180204030
ENSG0000009563	1.180628337	####	###	SORBS1	protein_coding	sorbin and S	10	95311771	95561414
ENSG0000013510	0.479990093	####	###	FBXO21	protein_coding	F-box prote	12	117141991	117190471
ENSG0000015234	1.127731815	####	###	ATG10	protein_coding	autophagy r	5	81972023	82276857
ENSG0000013504	1.182874768	####	###	CEMIP2	protein_coding	cell migratic	9	71683366	71816690
ENSG0000017172	0.602439799	####	###	HDAC3	protein_coding	histone dea	5	141620876	141636849
ENSG0000010055	0.637746439	####	###	ATP6V1D	protein_coding	ATPase H+	14	67294371	67360265
ENSG0000014138	-1.054996485	####	###	TAF4B	protein_coding	TATA-box b	18	26226445	26391685
ENSG0000025051	-1.953666538	####	###	GPR162	protein_coding	G protein-c	12	6821624	6829972
ENSG0000018302	-0.740852627	####	###	SLC8A1	protein_coding	solute carrie	2	40097270	40611053
ENSG0000014913	-0.325919098	####	###	SSRP1	protein_coding	structure sp	11	57325986	57335892
ENSG0000021502	-0.352461777	####	###	PHB2	protein_coding	prohibitin 2	12	6965327	6970753
ENSG0000014336	-2.756905209	####	###	RORC	protein_coding	RAR related	1	151806071	151831845
ENSG0000017266	1.195762246	####	###	TMEM134	protein_coding	transmembr	11	67461710	67469272
ENSG0000021451	0.483165891	####	###	PPME1	protein_coding	protein pho	11	74171267	74254703
ENSG0000011821	-0.443377997	####	###	ATF6	protein_coding	activating tr	1	161766320	161964070
ENSG0000004723	-0.761045642	####	###	CTPS2	protein_coding	CTP synthas	X	16587999	16712936
ENSG0000015658	-1.386654589	####	###	UBE2L6	protein_coding	ubiquitin co	11	57551656	57568284
ENSG0000012787	-0.833353023	####	###	RNF6	protein_coding	ring finger p	13	26132115	26222314
ENSG0000012581	0.475036326	####	###	CENPB	protein_coding	centromere	20	3783851	3786740
ENSG0000027276	-2.96775376	####	###	NA	NA	NA	NA	NA	NA
ENSG0000019654	-0.653273297	####	###	MAN2A2	protein_coding	mannosidas	15	90902218	90922584
ENSG0000023506	0.36537992	####	###	TUBB	protein_coding	tubulin beta	CHR_HSCHR	30710458	30715683
ENSG0000016524	0.870076317	####	###	ZNF367	protein_coding	zinc finger p	9	96385941	96418370
ENSG0000013799	0.675016707	####	###	DBT	protein_coding	dihydrolipoa	1	100186919	100249834
ENSG0000008906	0.503433552	####	###	TMEM230	protein_coding	transmembr	20	5068232	5113103
ENSG0000012278	0.802041109	####	###	CYREN	protein_coding	cell cycle re	7	135092363	135170795
ENSG0000012538	-0.510790049	####	###	PTGER2	protein_coding	prostagland	14	52314305	52328598
ENSG0000010788	0.750638096	####	###	SUFU	protein_coding	SUFU negat	10	102503972	102633535
ENSG0000013689	-0.516475927	####	###	TEX10	protein_coding	testis expres	9	100302077	100352942
ENSG0000014621	0.696138115	####	###	TTBK1	protein_coding	tau tubulin k	6	43243481	43288258
ENSG0000016351	-0.70567378	####	###	AZI2	protein_coding	5-azacytidir	3	28315003	28349050
ENSG0000012368	-0.405493632	####	###	LPGAT1	protein_coding	lysophospha	1	211743457	211830763
ENSG0000020411	-1.08931216	####	###	CHIC1	protein_coding	cysteine rich	X	73563200	73687102
ENSG0000022994	-10.20967711	####	###	PDE11A-AS	lncRNA	PDE11A ant	2	177653419	177723289
ENSG0000010293	-0.908559604	####	###	ZNF423	protein_coding	zinc finger p	16	49487524	49857919
ENSG0000018499	0.891654817	####	###	SIVA1	protein_coding	SIVA1 apop	14	104753147	104768494
ENSG0000010444	0.558562214	####	###	ARMC1	protein_coding	armadillo re	8	65602458	65634217
ENSG0000017401	0.55919176	####	###	FBXO45	protein_coding	F-box prote	3	196568611	196589059
ENSG0000019695	-4.152934359	####	###	CASP4	protein_coding	caspase 4 [S	11	104942866	104969366
ENSG0000010431	-0.467349175	####	###	EYA1	protein_coding	EYA transcri	8	71197433	71592025
ENSG0000008881	0.444525709	####	###	ATRN	protein_coding	attractin [Sc	20	3471018	3651118
ENSG0000016827	-1.142987731	####	###	COA6	protein_coding	cytochrome	1	234373456	234385080
ENSG0000011601	-1.227647657	####	###	ARID3A	protein_coding	AT-rich inte	19	925781	975939
ENSG0000011892	-0.688342898	####	###	KLF12	protein_coding	Kruppel like	13	73686089	73995056
ENSG0000014342	0.407037942	####	###	ENSA	protein_coding	endosulfine	1	150600851	150629612
ENSG0000011986	-1.067951016	####	###	LGALSL	protein_coding	galectin like	2	64453969	64461381
ENSG0000014099	-0.664538927	####	###	NDUFB10	protein_coding	NADH:ubiqu	16	1959538	1961975
ENSG0000008984	4.04744853	####	###	ANKRD24	protein_coding	ankyrin repe	19	4183354	4224814
ENSG0000014168	-0.736131162	####	###	PMAIP1	protein_coding	phorbol-12	18	59899996	59904305
ENSG0000011166	0.478549155	####	###	USP5	protein_coding	ubiquitin sp	12	6852128	6866632
ENSG0000020631	-1.636178703	####	###	PBX2	protein_coding	PBX homeo	CHR_HSCHR	32142341	32147792
ENSG0000010646	0.398795085	####	###	TMEM106B	protein_coding	transmembr	7	12211270	12243367
ENSG0000016417	0.715995113	####	###	MOCS2	protein_coding	molybdenur	5	53095679	53110063
ENSG0000013081	0.323973209	####	###	DNMT1	protein_coding	DNA methy	19	10133345	10231286
ENSG0000016680	0.479682241	####	###	PCLAF	protein_coding	PCNA clamp	15	64364304	64387687
ENSG0000007162	-0.402864048	####	###	DAZAP1	protein_coding	DAZ associa	19	1407569	1435687

ENSG0000013524	1.04229666	####	###	HILPDA	protein_coding	hypoxia ind	7	128455849	128458418
ENSG0000014960	-0.980715405	####	###	COMMD7	protein_coding	COMM dom	20	32702691	32743997
ENSG0000022447	1.708013899	####	###	TCF19	protein_coding	transcriptior	CHR_HSCHR	31237108	31242737
ENSG0000014486	-0.479633258	####	###	SRPRB	protein_coding	SRP recepto	3	133784023	133825772
ENSG0000016527	0.606149028	####	###	NOL6	protein_coding	nucleolar pr	9	33461353	33473930
ENSG0000000405	0.595470549	####	###	ARF5	protein_coding	ADP ribosyl	7	127588386	127591700
ENSG0000013920	-1.684827103	####	###	PIANP	protein_coding	PILR alpha a	12	6693792	6700800
ENSG0000014365	0.672508492	####	###	SCCPDH	protein_coding	saccharopin	1	246724409	246768137
ENSG0000010855	-0.638132494	####	###	RAI1	protein_coding	retinoic acic	17	17681458	17811453
ENSG0000013200	-1.181787014	####	###	RFX1	protein_coding	regulatory f	19	13961530	14007039
ENSG0000019678	-0.692271067	####	###	TLE1	protein_coding	TLE family n	9	81583683	81689547
ENSG0000024817	-1.937456178	####	###	LINC02268	lncRNA	long interge	4	174091766	174220398
ENSG0000027971	2.464921643	####	###	AC006128.1	TEC	TEC	19	15398696	15400356
ENSG0000013784	-1.857536727	####	###	TMEM62	protein_coding	transmembr	15	43123279	43185144
ENSG0000022182	-0.383738521	####	###	PPP3R1	protein_coding	protein pho	2	68178857	68256237
ENSG0000018924	0.434582842	####	###	TSPYL1	protein_coding	TSPY like 1	6	116267760	116279930
ENSG0000008963	-1.403322655	####	###	GMIP	protein_coding	GEM interac	19	19629476	19643657
ENSG0000021302	1.689196953	####	###	ZNF611	protein_coding	zinc finger p	19	52702813	52735073
ENSG0000017471	0.68344947	####	###	RESF1	protein_coding	retroelemer	12	31959370	31993107
ENSG0000018244	0.433744402	####	###	NPLOC4	protein_coding	NPL4 homo	17	81556887	81648465
ENSG0000023065	3.493645135	####	###	KLHL7-DT	lncRNA	KLHL7 diver	7	23100214	23105703
ENSG0000015984	-0.709909648	####	###	ZYX	protein_coding	zyxin [Sourc	7	143381295	143391111
ENSG0000011736	-0.44459117	####	###	APH1A	protein_coding	aph-1 homo	1	150265399	150269580
ENSG0000016908	-1.298714513	####	###	HSPBAP1	protein_coding	HSPB1 asso	3	122739999	122793831
ENSG0000014715	-0.7496851	####	###	EBP	protein_coding	EBP cholest	X	48521799	48528716
ENSG0000019891	0.674430002	####	###	SPOUT1	protein_coding	SPOUT dom	9	128819651	128829794
ENSG0000024327	0.795493513	####	###	PRAF2	protein_coding	PRA1 doma	X	49071161	49074002
ENSG0000008935	-0.711624671	####	###	GRAMD1A	protein_coding	GRAM dom	19	34994784	35026471
ENSG0000000339	0.648940881	####	###	ALS2	protein_coding	alsin Rho gu	2	201700267	201780956
ENSG0000025792	-0.467120653	####	###	CUX1	protein_coding	cut like hom	7	101815904	102283958
ENSG0000019801	-1.031919269	####	###	ENTPD7	protein_coding	ectonucleos	10	99659509	99711241
ENSG0000016346	0.417021304	####	###	ARPC2	protein_coding	actin relatec	2	218217141	218254356
ENSG0000016405	-1.189884695	####	###	PLXNB1	protein_coding	plexin B1 [S	3	48403854	48430086
ENSG0000013287	-2.179198851	####	###	FBXO44	protein_coding	F-box prote	1	11654375	11663327
ENSG0000023819	1.709112733	####	###	PAXBP1-AS	lncRNA	PAXBP1 ant	21	32728097	32747065
ENSG0000025721	0.603708993	####	###	GATC	protein_coding	glutamyl-tR	12	120446444	120463749
ENSG0000016019	-2.291439317	####	###	PDE9A	protein_coding	phosphodie	21	42653621	42775509
ENSG0000007999	-0.559469994	####	###	KEAP1	protein_coding	kelch like EC	19	10486125	10503558
ENSG0000013428	-1.182988739	####	###	FKBP11	protein_coding	FKBP prolyl	12	48921518	48926474
ENSG0000019649	-0.38954236	####	###	NCOR2	protein_coding	nuclear rece	12	124324415	124567589
ENSG0000027023	-1.039241594	####	###	NBPF8	transcribed_un	NBPF memb	1	120419850	120467739
ENSG0000019886	-0.549812717	####	###	TSEN15	protein_coding	tRNA splicin	1	184051651	184123978
ENSG0000015314	0.803134763	####	###	CETN3	protein_coding	centrin 3 [So	5	90392257	90409766
ENSG0000010710	0.528587131	####	###	ELAVL2	protein_coding	ELAV like RI	9	23690104	23826337
ENSG0000002452	0.505009375	####	###	DEPDC1	protein_coding	DEP domair	1	68474152	68497221
ENSG0000023199	0.840751566	####	###	ANXA2P2	processed_pse	annexin A2	9	33624274	33625293
ENSG0000017234	-0.798497939	####	###	SUCLG2	protein_coding	succinate-C	3	67360460	67654612
ENSG0000007866	0.428804403	####	###	VDAC3	protein_coding	voltage dep	8	42391624	42405937
ENSG0000012106	-0.627735053	####	###	TRIM25	protein_coding	tripartite mo	17	56836387	56914080
ENSG0000016622	-0.971724385	####	###	PCBD1	protein_coding	pterin-4 alp	10	70882280	70888565
ENSG0000017561	1.333976298	####	###	LINC00476	lncRNA	long interge	9	95759231	95876049
ENSG0000023257	0.347267456	####	###	TUBB	protein_coding	tubulin beta	CHR_HSCHR	30764859	30770084
ENSG0000017460	-0.624018889	####	###	ANGEL2	protein_coding	angel homo	1	212992182	213015867
ENSG0000011739	0.623575639	####	###	EBNA1BP2	protein_coding	EBNA1 bind	1	43164175	43270936
ENSG0000027430	1.152238155	####	###	LENG8	protein_coding	leukocyte re	CHR_HSCHR	54379038	54392337
ENSG0000008229	-2.522146022	####	###	COL19A1	protein_coding	collagen typ	6	69866556	70212468
ENSG0000011233	-0.506208722	####	###	SNX3	protein_coding	sorting nexi	6	108211222	108261246
ENSG0000015678	0.823804187	####	###	TBC1D31	protein_coding	TBC1 doma	8	123041968	123152153

ENSG0000019650	-2.850542238	####	###	ARL9	protein_codinç	ADP ribosyl	4	56505209	56524959
ENSG0000016020	-0.645464504	####	###	PDXK	protein_codinç	pyridoxal ki	21	43719094	43762307
ENSG0000016776	1.419313839	####	###	ZNF83	protein_codinç	zinc finger p	19	52594060	52690496
ENSG0000016096	0.905686232	####	###	ZNF333	protein_codinç	zinc finger p	19	14689801	14733746
ENSG0000014240	-1.097054475	####	###	ZNF787	protein_codinç	zinc finger p	19	56087366	56121295
ENSG0000018464	-0.329353933	####	###	SEPTIN9	protein_codinç	septin 9 [So	17	77280569	77500596
ENSG0000006097	1.20802367	####	###	ACAA1	protein_codinç	acetyl-CoA	3	38103129	38137242
ENSG0000012550	0.824117973	####	###	PPP1R12C	protein_codinç	protein pho	19	55090914	55117637
ENSG0000015153	0.694331432	####	###	VTI1A	protein_codinç	vesicle trans	10	112446998	112818744
ENSG0000021549	-0.605940205	####	###	HNRNPA1F	processed_pse	heterogene	18	32412214	32413176
ENSG0000017287	-1.204073226	####	###	METAP1D	protein_codinç	methionyl a	2	171999943	172082430
ENSG0000007036	0.39336026	####	###	EXOC5	protein_codinç	exocyst com	14	57200507	57268905
ENSG0000008407	0.791638824	####	###	SMAP2	protein_codinç	small ArfGA	1	40344850	40423326
ENSG0000025485	0.903746488	####	###	MPV17L2	protein_codinç	MPV17 mitc	19	18193218	18196948
ENSG0000017299	0.721110409	####	###	DCAKD	protein_codinç	dephospho-	17	45023340	45061109
ENSG0000016719	-0.532462056	####	###	FBXO22	protein_codinç	F-box prote	15	75903876	75942511
ENSG0000012069	-0.85419652	####	###	ALG5	protein_codinç	ALG5 dolich	13	36949738	37000261
ENSG0000016036	-0.557013439	####	###	GPSM1	protein_codinç	G protein si	9	136327476	136359605
ENSG0000014339	-0.521926245	####	###	RFX5	protein_codinç	regulatory f	1	151340640	151347357
ENSG0000014254	-1.857204952	####	###	IGLON5	protein_codinç	IgLON famil	19	51311848	51330354
ENSG0000011084	-0.418036528	####	###	PPFIBP1	protein_codinç	PPFIA bindi	12	27523431	27695564
ENSG0000004228	-1.434250161	####	###	AIFM2	protein_codinç	apoptosis ir	10	70098223	70132934
ENSG0000005496	1.150166148	####	###	RELT	protein_codinç	RELT TNF re	11	73376399	73397474
ENSG0000010335	-0.693671407	####	###	EARS2	protein_codinç	glutamyl-tR	16	23520754	23557731
ENSG0000010775	0.514433059	####	###	PPP3CB	protein_codinç	protein pho	10	73436433	73496024
ENSG0000012596	1.15006957	####	###	NECAB3	protein_codinç	N-terminal	20	33657087	33674463
ENSG0000015228	-1.837484817	####	###	TCF7L1	protein_codinç	transcriptior	2	85133392	85310387
ENSG0000015249	-0.453105519	####	###	CCDC50	protein_codinç	coiled-coil c	3	191329085	191398659
ENSG0000018081	-1.820025412	####	###	HOXC10	protein_codinç	homeobox (	12	53985065	53990279
ENSG0000017933	0.552929131	####	###	CLK3	protein_codinç	CDC like kin	15	74598500	74645414
ENSG0000013481	-2.948801312	####	###	APLNR	protein_codinç	apelin recep	11	57233577	57237235
ENSG0000019671	-0.707198704	####	###	NF1	protein_codinç	neurofibron	17	31094927	31382116
ENSG0000014399	0.646318927	####	###	MEIS1	protein_codinç	Meis homec	2	66433452	66573869
ENSG0000013657	-1.916920246	####	###	GATA4	protein_codinç	GATA bindi	8	11676959	11760002
ENSG0000013594	-0.553622323	####	###	REV1	protein_codinç	REV1 DNA c	2	99400475	99490035
ENSG0000022887	-4.198314112	####	###	AC010745.2	lncRNA	novel transc	2	16224047	16333978
ENSG0000014687	-0.495804771	####	###	TLK2	protein_codinç	tousled like	17	62458658	62615481
ENSG0000023160	1.430797587	####	###	DLEU2	lncRNA	deleted in ly	13	49956670	50125720
ENSG0000020558	1.789321223	####	###	STAG3L1	transcribed_un	stromal anti	7	75359194	75395383
ENSG0000014805	-10.00578541	####	###	NTRK2	protein_codinç	neurotrophi	9	84668551	85027070
ENSG0000011298	0.401329129	####	###	KIF20A	protein_codinç	kinesin fami	5	138178719	138187723
ENSG0000018287	-1.68181034	####	###	COL18A1	protein_codinç	collagen typ	21	45405165	45513720
ENSG0000013055	0.367212575	####	###	OLFM1	protein_codinç	olfactomedi	9	135075422	135121180
ENSG0000017898	-0.605561418	####	###	EIF3K	protein_codinç	eukaryotic t	19	38619082	38636955
ENSG0000014997	-0.779920329	####	###	CNKSR2	protein_codinç	connector e	X	21372801	21654695
ENSG0000016826	-0.378040775	####	###	IRF2BP2	protein_codinç	interferon re	1	234604269	234609525
ENSG0000017304	-2.624259632	####	###	EVC2	protein_codinç	EvC ciliary c	4	5542772	5709548
ENSG0000001217	0.5736483	####	###	MBTPS2	protein_codinç	membrane	X	21839617	21885423
ENSG0000016800	0.611377438	####	###	POLR2G	protein_codinç	RNA polyme	11	62761565	62766710
ENSG0000007037	2.146461774	####	###	CLTCL1	protein_codinç	clathrin hea	22	19179473	19291719
ENSG0000015278	-0.651823479	####	###	PRDM8	protein_codinç	PR/SET dom	4	80183879	80204329
ENSG0000010196	-0.496941146	####	###	XIAP	protein_codinç	X-linked inh	X	123859724	123913976
ENSG0000015306	-0.959432599	####	###	TXNDC11	protein_codinç	thioredoxin	16	11679080	11742878
ENSG0000016696	0.878107696	####	###	RCCD1	protein_codinç	RCC1 doma	15	90954870	90963125
ENSG0000024606	1.361454491	####	###	RAB30-DT	lncRNA	RAB30 diver	11	83072052	83107789
ENSG0000010932	-0.84378999	####	###	NFKB1	protein_codinç	nuclear fact	4	102501331	102617302
ENSG0000016999	2.004041576	####	###	IFFO2	protein_codinç	intermediat	1	18904280	18956676
ENSG0000008022	-4.034039703	####	###	EPHA6	protein_codinç	EPH recepto	3	96814581	97752460

ENSG0000018311	-1.354225477	####	###	FAM43B	protein_coding	family with s	1	20552573	20555020
ENSG0000011978	1.288573073	####	###	FKBP1B	protein_coding	FKBP prolyl	2	24049701	24063681
ENSG0000024809	-0.805257532	####	###	BCKDHA	protein_coding	branched ch	19	41397808	41425002
ENSG0000010781	-0.801358792	####	###	LZTS2	protein_coding	leucine zipp	10	100996618	101007836
ENSG0000018325	0.520300182	####	###	DDX41	protein_coding	DEAD-box l	5	177511577	177516961
ENSG0000011612	0.418622339	####	###	FARSB	protein_coding	phenylalany	2	222566899	222656092
ENSG0000011265	-0.391370262	####	###	PTK7	protein_coding	protein tyro	6	43076307	43161719
ENSG0000010564	-1.766725073	####	###	ARRDC2	protein_coding	arrestin don	19	18001132	18014102
ENSG0000016591	0.400823138	####	###	PSMC3	protein_coding	proteasome	11	47418769	47426473
ENSG0000010857	0.776964051	####	###	BLMH	protein_coding	bleomycin h	17	30248203	30292056
ENSG0000012583	-0.415326854	####	###	SNRPB	protein_coding	small nuclea	20	2461634	2470853
ENSG0000000611	0.402129927	####	###	TMEM132A	protein_coding	transmembr	11	60924460	60937159
ENSG0000013590	-1.838724407	####	###	DOCK10	protein_coding	dedicator of	2	224765090	225042445
ENSG0000017115	-0.528568609	####	###	SOCS5	protein_coding	suppressor c	2	46698952	46780245
ENSG0000014322	1.158308976	####	###	PPOX	protein_coding	protoporphy	1	161166056	161178013
ENSG0000010093	-0.420383635	####	###	SEC23A	protein_coding	Sec23 homo	14	39031919	39109646
ENSG0000022863	-2.668916195	####	###	HOTAIR	lncRNA	HOX transcr	12	53962308	53974956
ENSG0000013521	1.202501123	####	###	TMEM60	protein_coding	transmembr	7	77793728	77798434
ENSG0000021578	-2.750704631	####	###	TNFRSF25	protein_coding	TNF recepto	1	6460786	6466175
ENSG0000010413	0.572519628	####	###	SPG11	protein_coding	SPG11 vesic	15	44562696	44663678
ENSG0000012786	-3.045179835	####	###	TNFRSF19	protein_coding	TNF recepto	13	23570370	23676104
ENSG0000010494	-1.260320745	####	###	TBC1D17	protein_coding	TBC1 doma	19	49877425	49888750
ENSG0000013685	-0.902818994	####	###	ANGPTL2	protein_coding	angiopoietin	9	127087348	127122635
ENSG0000008888	0.426160208	####	###	MAVS	protein_coding	mitochondr	20	3846799	3876123
ENSG0000019742	1.149593642	####	###	IPP	protein_coding	intracisterna	1	45694324	45750653
ENSG0000014599	-0.628243631	####	###	GFOD1	protein_coding	glucose-fru	6	13357830	13487662
ENSG0000005533	-0.410129945	####	###	EIF2AK2	protein_coding	eukaryotic t	2	37099210	37157065
ENSG0000016570	0.702841917	####	###	HPRT1	protein_coding	hypoxanthir	X	134460165	134520513
ENSG0000012670	-0.790229976	####	###	AHDC1	protein_coding	AT-hook DN	1	27534035	27604431
ENSG0000019889	-0.739257071	####	###	CIPC	protein_coding	CLOCK inter	14	77098126	77117287
ENSG0000017390	0.355403834	####	###	GOLIM4	protein_coding	golgi integra	3	168008689	168095924
ENSG0000016767	-0.619674881	####	###	UBXN6	protein_coding	UBX domair	19	4444999	4457794
ENSG0000012335	1.006004174	####	###	ORMDL2	protein_coding	ORMDL sph	12	55818041	55821879
ENSG0000013233	-0.638154372	####	###	SCLY	protein_coding	selenocystei	2	238060924	238099413
ENSG0000012054	-2.211019372	####	###	KIAA1217	protein_coding	KIAA1217 [S	10	23694746	24547848
ENSG0000019856	0.315384223	####	###	CTNND1	protein_coding	catenin delt	11	57753243	57819546
ENSG0000001032	0.59204885	####	###	NISCH	protein_coding	nischarin [Sc	3	52455118	52493068
ENSG0000019887	-0.674745369	####	###	SFMBT2	protein_coding	Scm like wit	10	7158624	7411486
ENSG0000018359	1.351049147	####	###	TANGO2	protein_coding	transport ar	22	20017014	20067164
ENSG0000002012	0.596050822	####	###	NCDN	protein_coding	neurochonc	1	35557473	35567274
ENSG0000026090	0.749777166	####	###	XKR7	protein_coding	XK related 7	20	31968151	32003387
ENSG0000012248	0.994869386	####	###	CCDC18	protein_coding	coiled-coil c	1	93179919	93278730
ENSG0000014892	-1.691871934	####	###	ADM	protein_coding	adrenomed	11	10305073	10307397
ENSG0000019642	-0.806767316	####	###	PPP1R26	protein_coding	protein pho	9	135479079	135488893
ENSG0000014031	0.403459189	####	###	SRP14	protein_coding	signal recog	15	40035690	40039181
ENSG0000016360	-3.525419802	####	###	CD200R1	protein_coding	CD200 rece	3	112921205	112975103
ENSG0000012963	0.546388132	####	###	ITFG1	protein_coding	integrin alpl	16	47154387	47464149
ENSG0000011616	-0.406096908	####	###	CACYBP	protein_coding	calcyclin bir	1	174999163	175012027
ENSG0000010601	-2.950094833	####	###	VIPR2	protein_coding	vasoactive ir	7	159028175	159144867
ENSG0000016101	-0.621186601	####	###	MGAT4B	protein_coding	alpha-1,3-m	5	179797597	179806952
ENSG0000014923	0.457001344	####	###	CCDC82	protein_coding	coiled-coil c	11	96352769	96389956
ENSG0000000161	-1.29217933	####	###	SEMA3F	protein_coding	semaphorin	3	50155045	50189075
ENSG0000016688	0.583310358	####	###	NAB2	protein_coding	NGFI-A binc	12	57089043	57095476
ENSG0000016580	0.359185275	####	###	NSMF	protein_coding	NMDA rece	9	137447570	137459334
ENSG0000010354	0.394675693	####	###	RNF40	protein_coding	ring finger p	16	30761745	30776307
ENSG0000002915	2.555886665	####	###	ARNTL2	protein_coding	aryl hydroca	12	27332854	27425289
ENSG0000015238	-0.807559393	####	###	TADA1	protein_coding	transcriptior	1	166856510	166876264
ENSG0000016611	1.190077908	####	###	SVOP	protein_coding	SV2 related	12	108907741	109021068

ENSG0000016305	-0.614555573	####	###	COQ8A	protein_codinc	coenzyme C	1	226897536	226987545
ENSG0000025365	-3.166240672	####	###	AC138646.:	lncRNA	novel transc	8	79259402	79314951
ENSG0000014627	-0.480391747	####	###	PNRC1	protein_codinc	proline rich	6	89080751	89085160
ENSG0000013577	-0.533530355	####	###	NTPCR	protein_codinc	nucleoside-	1	232950605	232983882
ENSG0000003654	-0.499208254	####	###	ZZZ3	protein_codinc	zinc finger Z	1	77562416	77683419
ENSG0000014173	1.855230002	####	###	ERBB2	protein_codinc	erb-b2 rece	17	39687914	39730426
ENSG0000006682	-1.366195806	####	###	ZFAT	protein_codinc	zinc finger a	8	134477788	134713049
ENSG0000011647	0.602976006	####	###	RAP1A	protein_codinc	RAP1A, mer	1	111542218	111716691
ENSG0000014413	-0.471910549	####	###	SLC20A1	protein_codinc	solute carrie	2	112645939	112663825
ENSG0000011440	0.763229546	####	###	C3orf14	protein_codinc	chromosom	3	62319015	62336213
ENSG0000001948	-1.082859053	####	###	PRDM11	protein_codinc	PR/SET dom	11	45095806	45235110
ENSG0000012766	0.468919028	####	###	KDM4B	protein_codinc	lysine deme	19	4969113	5153598
ENSG0000011600	0.331688825	####	###	TIA1	protein_codinc	TIA1 cytoto:	2	70209444	70248660
ENSG0000010454	0.374253592	####	###	SQLE	protein_codinc	squalene ep	8	124998497	125022283
ENSG0000005152	-2.099804793	####	###	CYBA	protein_codinc	cytochrome	16	88643289	88651054
ENSG0000027353	-5.728505592	####	###	AC004556.:	misc_RNA		KI270721.1	51722	51792
ENSG0000018099	0.819656489	####	###	GPR137C	protein_codinc	G protein-c	14	52552836	52637713
ENSG0000016792	-1.236008854	####	###	GHDC	protein_codinc	GH3 domair	17	42188799	42194532
ENSG0000017130	0.575727959	####	###	CANT1	protein_codinc	calcium acti	17	78991716	79009867
ENSG0000020590	-0.589852493	####	###	ZNF316	protein_codinc	zinc finger p	7	6637318	6658279
ENSG0000010438	0.488163237	####	###	GDAP1	protein_codinc	ganglioside	8	74320613	74518007
ENSG0000014915	-1.591018406	####	###	SLC43A1	protein_codinc	solute carrie	11	57484534	57515780
ENSG0000013716	-0.559476945	####	###	PPIL1	protein_codinc	peptidylprol	6	36854827	36874803
ENSG0000027725	-0.996965001	####	###	PCGF2	protein_codinc	polycomb g	17	38733898	38749817
ENSG0000017489	-0.610915682	####	###	RSRC1	protein_codinc	arginine anc	3	158105855	158545730
ENSG0000016825	0.463595351	####	###	NKIRAS2	protein_codinc	NFKB inhibi	17	42011382	42025644
ENSG0000016241	-0.784719001	####	###	KLHL21	protein_codinc	kelch like fa	1	6590724	6614607
ENSG0000009093	-2.186795371	####	###	DLL3	protein_codinc	delta like ca	19	39498895	39508481
ENSG0000014198	0.454807695	####	###	SH3GL1	protein_codinc	SH3 domair	19	4360370	4400547
ENSG0000013014	0.489637543	####	###	SH3BP4	protein_codinc	SH3 domair	2	234952017	235055714
ENSG0000012565	-0.412409435	####	###	GTF2F1	protein_codinc	general trar	19	6379572	6393981
ENSG0000010639	-0.757342376	####	###	PLOD3	protein_codinc	procollagen	7	101205977	101218420
ENSG0000023485	-2.415098213	####	###	ZBTB12	protein_codinc	zinc finger a	CHR_HSCHR	31881788	31884173
ENSG0000023419	-2.522461097	####	###	ZBTB12	protein_codinc	zinc finger a	CHR_HSCHR	31976089	31978474
ENSG0000002542	-2.240249978	####	###	HSD17B6	protein_codinc	hydroxyster	12	56752161	56787790
ENSG0000012238	-1.198840645	####	###	ZNF205	protein_codinc	zinc finger p	16	3112560	3120517
ENSG0000012966	-1.921017038	####	###	RHBDF2	protein_codinc	rhomboid 5	17	76470891	76501790
ENSG0000022825	0.266951185	####	###	MT-ATP8	protein_codinc	mitochondr	MT	8366	8572
ENSG0000022415	0.332319107	####	###	TUBB	protein_codinc	tubulin beta	CHR_HSCHR	30784111	30789336
ENSG0000017175	-1.817963292	####	###	LRRC34	protein_codinc	leucine rich	3	169793003	169812986
ENSG0000010967	0.863776796	####	###	NEIL3	protein_codinc	nei like DN/	4	177309874	177362936
ENSG0000010088	0.460051455	####	###	SRP54	protein_codinc	signal recog	14	34981957	35029567
ENSG0000019878	-1.416146902	####	###	FAM169A	protein_codinc	family with s	5	74777574	74866966
ENSG0000012732	-4.123661717	####	###	TSPAN8	protein_codinc	tetraspanin	12	71125085	71441898
ENSG0000012464	0.755354791	####	###	MED20	protein_codinc	mediator cc	6	41905354	41921139
ENSG0000011126	-1.328774035	####	###	MANSC1	protein_codinc	MANSC dor	12	12326056	12350242
ENSG0000019791	0.605302136	####	###	SPG7	protein_codinc	SPG7 matrix	16	89490719	89557766
ENSG0000014152	-2.521529533	####	###	TMC6	protein_codinc	transmembi	17	78107397	78132407
ENSG0000019787	-1.169971699	####	###	MYO1C	protein_codinc	myosin IC [S	17	1464186	1492686
ENSG0000013754	-0.520409852	####	###	MRPL15	protein_codinc	mitochondr	8	54135241	54148514
ENSG0000018025	2.358426139	####	###	ZNF816	protein_codinc	zinc finger p	19	52949379	52962911
ENSG0000018616	0.789676697	####	###	CCDC84	protein_codinc	coiled-coil c	11	118998138	119015793
ENSG0000013295	-0.589945407	####	###	XPO4	protein_codinc	exportin 4 [!	13	20777329	20903048
ENSG0000010856	-0.448430249	####	###	C1QBP	protein_codinc	complemen	17	5432777	5448830
ENSG0000025336	1.681876136	####	###	TRNP1	protein_codinc	TMF1 regula	1	26993692	27000886
ENSG0000013453	-1.860300917	####	###	RERG	protein_codinc	RAS like est	12	15107783	15348675
ENSG0000016458	0.820630606	####	###	HCN1	protein_codinc	hyperpolariz	5	45254948	45696498
ENSG0000015431	-0.364777451	####	###	FAM167A	protein_codinc	family with s	8	11421476	11475908

ENSG0000015166	0.826331268	####	###	PIGF	protein_coding	phosphatidy	2	46580937	46617055
ENSG0000018331	0.328676872	####	###	TUBB	protein_coding	tubulin beta	CHR_HSCHR	30709720	30714946
ENSG0000013943	-0.534045571	####	###	FAM222A	protein_coding	family with s	12	109713825	109770495
ENSG0000011932	0.474399152	####	###	CTNNAL1	protein_coding	catenin alph	9	108942569	109013522
ENSG0000012798	0.640741579	####	###	PEX1	protein_coding	peroxisoma	7	92487020	92528520
ENSG0000014133	-1.380002323	####	###	ARSG	protein_coding	arylsulfatase	17	68259182	68422731
ENSG0000008817	-0.601032971	####	###	PTPN4	protein_coding	protein tyro	2	119759922	119984899
ENSG0000022563	0.27290612	####	###	MTND2P28	unprocessed_p	MT-ND2 ps	1	629640	630683
ENSG0000013485	-0.616848654	####	###	CLOCK	protein_coding	clock circad	4	55427903	55546909
ENSG0000026080	-7.222996354	####	###	SERTM2	protein_coding	serine rich a	X	111511662	111522399
ENSG0000013342	0.473148289	####	###	MORC2	protein_coding	MORC fami	22	30925130	30968774
ENSG0000014543	-0.581261598	####	###	CBR4	protein_coding	carbonyl rec	4	168863770	169010275
ENSG0000013832	-0.213329001	####	###	RPS24	protein_coding	ribosomal p	10	78033760	78056813
ENSG0000018179	-2.06107662	####	###	ADGRB1	protein_coding	adhesion G	8	142449430	142545009
ENSG0000027478	0.705751067	####	###	PHRF1	protein_coding	PHD and rir	CHR_HSCHR	576501	612474
ENSG0000012354	-0.695102504	####	###	NDUFAF4	protein_coding	NADH:ubiqu	6	96889315	96897891
ENSG0000007534	-0.379661267	####	###	ADD2	protein_coding	adducin 2 [S	2	70607618	70768225
ENSG0000013235	-0.453737536	####	###	RAP1GAP2	protein_coding	RAP1 GTPas	17	2755705	3037741
ENSG0000013643	-0.514246244	####	###	CALCOCO2	protein_coding	calcium binc	17	48831018	48866522
ENSG0000019675	-0.92970956	####	###	SNHG17	lncRNA	small nuclec	20	38419638	38435409
ENSG0000016838	-0.785034912	####	###	FILIP1L	protein_coding	filamin A int	3	99830141	100114513
ENSG0000012567	-1.76733637	####	###	GRIA3	protein_coding	glutamate ic	X	123184153	123490915
ENSG0000027422	2.680603375	####	###	AC009163.7	lncRNA	novel transc	16	75433836	75436392
ENSG0000015763	0.636075294	####	###	SLC38A10	protein_coding	solute carrie	17	81244811	81295547
ENSG0000015582	0.497254152	####	###	RNF20	protein_coding	ring finger p	9	101533853	101563344
ENSG0000008549	0.54841458	####	###	SLC25A24	protein_coding	solute carrie	1	108134043	108200849
ENSG0000019729	0.680886139	####	###	BLM	protein_coding	BLM RecQ l	15	90717346	90816166
ENSG0000010644	-0.414414668	####	###	PHF14	protein_coding	PHD finger	7	10973336	11169623
ENSG0000014482	0.700755021	####	###	ABHD10	protein_coding	abhydrolase	3	111979010	111993368
ENSG0000015277	0.60004048	####	###	IFIT5	protein_coding	interferon ir	10	89414568	89420997
ENSG0000014029	-0.377675093	####	###	BNIP2	protein_coding	BCL2 intera	15	59659146	59689534
ENSG0000012687	-2.189420437	####	###	AIF1L	protein_coding	allograft infl	9	131096476	131123152
ENSG0000010086	0.821567236	####	###	CINP	protein_coding	cyclin deper	14	102341102	102362916
ENSG0000021416	-0.667718596	####	###	ALG3	protein_coding	ALG3 alpha	3	184242301	184249548
ENSG0000016680	-1.595449532	####	###	LDHAL6A	protein_coding	lactate dehy	11	18455824	18479601
ENSG0000016446	0.446801917	####	###	SFXN1	protein_coding	sideroflexin	5	175477062	175529742
ENSG0000010093	-0.701246625	####	###	GMPR2	protein_coding	guanosine r	14	24232422	24239242
ENSG0000016407	-1.555775731	####	###	MON1A	protein_coding	MON1 hom	3	49907160	49930173
ENSG0000014536	-1.944737624	####	###	TIFA	protein_coding	TRAF intera	4	112274537	112285904
ENSG0000011518	-0.508706409	####	###	TANC1	protein_coding	tetratricope	2	158968640	159232659
ENSG0000019889	0.565256076	####	###	PRMT6	protein_coding	protein argi	1	107056674	107067636
ENSG0000011924	0.765474993	####	###	CCDC92	protein_coding	coiled-coil c	12	123918660	123972831
ENSG0000010302	-1.24041456	####	###	NME3	protein_coding	NME/NM23	16	1770286	1771730
ENSG0000017993	0.730199311	####	###	C14orf119	protein_coding	chromosom	14	23095505	23100456
ENSG0000019872	-0.724287046	####	###	ANKRD13B	protein_coding	ankyrin repe	17	29589769	29614761
ENSG0000021521	-1.179856318	####	###	UBE2QL1	protein_coding	ubiquitin co	5	6448859	6496723
ENSG0000003853	0.569457135	####	###	CLEC16A	protein_coding	C-type lecti	16	10944564	11182186
ENSG0000010144	-0.328785505	####	###	AHCY	protein_coding	adenosylhoı	20	34280268	34311802
ENSG0000010879	-0.82691078	####	###	CNTNAP1	protein_coding	contactin as	17	42682531	42699993
ENSG0000008268	-3.355829182	####	###	SEMA5B	protein_coding	semaphorin	3	122909082	123028605
ENSG0000016850	-0.639471146	####	###	MTCL1	protein_coding	microtubule	18	8705661	8832778
ENSG0000016184	0.533568617	####	###	RAVER1	protein_coding	ribonucleop	19	10316212	10333638
ENSG0000010605	0.391254421	####	###	TAX1BP1	protein_coding	Tax1 bindin	7	27739331	27844564
ENSG0000015412	0.554830851	####	###	OTULIN	protein_coding	OTU deubic	5	14664664	14699850
ENSG0000013862	1.339043038	####	###	PPCDC	protein_coding	phosphopar	15	75023586	75117462
ENSG0000016395	0.607155465	####	###	LRPAP1	protein_coding	LDL recepto	4	3503612	3532446
ENSG0000020416	-9.69124999	####	###	TMEM273	protein_coding	transmembı	10	49154725	49188585
ENSG0000006693	0.491026618	####	###	MYO9A	protein_coding	myosin IXA	15	71822291	72118577

ENSG0000017218	-4.525741874	####	###	ISG20	protein_coding	interferon st	15	88636153	88656483
ENSG0000010529	0.579496087	####	###	APLP1	protein_coding	amyloid bet	19	35867899	35879792
ENSG0000013216	-3.563131499	####	###	SLC6A11	protein_coding	solute carrie	3	10816201	10940714
ENSG0000015161	-1.245022869	####	###	EDNRA	protein_coding	endothelin r	4	147480917	147544954
ENSG0000014094	0.301565938	####	###	ZCCHC14	protein_coding	zinc finger C	16	87406246	87493024
ENSG0000002695	-1.517858417	####	###	BTN3A1	protein_coding	butyrophilin	6	26402237	26415208
ENSG0000015989	-1.163520357	####	###	NPR2	protein_coding	natriuretic p	9	35791591	35809732
ENSG0000011610	-2.155795454	####	###	EPHA4	protein_coding	EPH recepto	2	221418027	221574202
ENSG0000007508	0.708264726	####	###	ACTR6	protein_coding	actin relatec	12	100199122	100241865
ENSG0000016152	0.467894397	####	###	SAP30BP	protein_coding	SAP30 bind	17	75667251	75708062
ENSG0000011827	-1.292351981	####	###	B4GALT6	protein_coding	beta-1,4-ga	18	31622247	31685836
ENSG0000016210	-0.958403198	####	###	SHANK2	protein_coding	SH3 and mu	11	70467856	71252577
ENSG0000016924	-1.622262833	####	###	EFNA1	protein_coding	ephrin A1 [S	1	155127876	155134899
ENSG0000017735	1.720178778	####	###	CCDC71	protein_coding	coiled-coil c	3	49162535	49166331
ENSG0000017400	-4.629091505	####	###	NRROS	protein_coding	negative reg	3	196639694	196662004
ENSG0000022418	-0.755722606	####	###	HAGLR	lncRNA	HOXD antis	2	176164051	176188958
ENSG0000018898	0.545594855	####	###	NELFB	protein_coding	negative elc	9	137255327	137273542
ENSG0000016701	-3.3892808	####	###	NAT16	protein_coding	N-acetyltrar	7	101170496	101180293
ENSG0000021342	1.021017563	####	###	GPC2	protein_coding	glypican 2 [S	7	100169606	100177381
ENSG0000025952	4.515897052	####	###	AC051619.4	lncRNA	novel transc	15	45235930	45279251
ENSG0000014362	-0.282044885	####	###	ILF2	protein_coding	interleukin e	1	153661788	153671028
ENSG0000016427	-5.268820432	####	###	HTR4	protein_coding	5-hydroxytr	5	148451032	148677235
ENSG0000015959	0.424945437	####	###	NAE1	protein_coding	NEDD8 activ	16	66802875	66873256
ENSG0000014614	0.835275553	####	###	PRIM2	protein_coding	DNA primas	6	57314805	57646850
ENSG0000017315	-0.651226416	####	###	ESRRA	protein_coding	estrogen rel	11	64305497	64316743
ENSG0000014273	0.529769987	####	###	PLK4	protein_coding	polo like kin	4	127880893	127899224
ENSG0000017058	0.871912173	####	###	STAT2	protein_coding	signal transc	12	56341597	56360167
ENSG0000017991	-0.585724428	####	###	SEPHS2	protein_coding	selenophosp	16	30443631	30445874
ENSG0000013554	-0.642963412	####	###	AHI1	protein_coding	Abelson hel	6	135283532	135497765
ENSG0000001357	0.666140038	####	###	DDX11	protein_coding	DEAD/H-bc	12	31073860	31104799
ENSG0000011515	-0.731463974	####	###	GPD2	protein_coding	glycerol-3-p	2	156435290	156613735
ENSG0000002177	0.371355848	####	###	AQR	protein_coding	aquarius int	15	34851782	34969742
ENSG0000022845	1.961677219	####	###	SDAD1P1	transcribed_pr	SDA1 doma	8	26375913	26382953
ENSG0000022862	-1.178934014	####	###	ZNF883	transcribed_un	zinc finger p	9	112957722	113012227
ENSG0000015325	0.395931848	####	###	RBMS1	protein_coding	RNA binding	2	160272151	160493807
ENSG0000023402	2.748427639	####	###	EIF2AK3-DT	lncRNA	EIF2AK3 div	2	88627539	88631821
ENSG0000017528	1.029849506	####	###	DOLK	protein_coding	dolichol kina	9	128945530	128947603
ENSG0000021456	1.43178856	####	###	NUTM2D	protein_coding	NUT family	10	87357668	87370695
ENSG0000000375	0.444370088	####	###	RBM5	protein_coding	RNA binding	3	50088919	50119021
ENSG0000015965	0.578303027	####	###	EFCAB14	protein_coding	EF-hand cal	1	46674659	46719146
ENSG0000027502	-0.457208781	####	###	MLLT6	protein_coding	MLLT6, PHD	17	38705273	38729795
ENSG0000006658	-0.401753486	####	###	ISOC1	protein_coding	isochorisma	5	129094749	129114028
ENSG0000007196	-2.289427694	####	###	CYBRD1	protein_coding	cytochrome	2	171522247	171558129
ENSG0000017488	0.488318752	####	###	NDUFA11	protein_coding	NADH:ubiqu	19	5891276	5904006
ENSG0000015393	0.463699701	####	###	HS2ST1	protein_coding	heparan sul	1	86914635	87109982
ENSG0000018867	3.59387764	####	###	RHCE	protein_coding	Rh blood gr	1	25362249	25430192
ENSG0000018369	-2.407889726	####	###	UPP1	protein_coding	uridine phos	7	48088628	48108736
ENSG0000011136	0.487889043	####	###	EIF2B1	protein_coding	eukaryotic t	12	123620406	123633766
ENSG0000023685	1.395955633	####	###	NIFK-AS1	lncRNA	NIFK antiser	2	121649320	121728563
ENSG0000010060	-0.286799845	####	###	CHGA	protein_coding	chromograr	14	92923150	92935285
ENSG0000027790	1.006287796	####	###	SPC25	protein_coding	SPC25 comp	CHR_HSCHR	168834132	168930360
ENSG0000014524	-0.962244524	####	###	OCIAD2	protein_coding	OCIA doma	4	48885019	48906937
ENSG0000017063	-1.27067724	####	###	ZNF16	protein_coding	zinc finger p	8	144930358	144950888
ENSG0000008243	-1.380263131	####	###	COBLL1	protein_coding	cordon-bleu	2	164653624	164843679
ENSG0000020376	-0.970479192	####	###	MSTO2P	unprocessed_p	misato fami	1	155745829	155750137
ENSG0000013536	-0.427337524	####	###	PHF21A	protein_coding	PHD finger	11	45929319	46121454
ENSG0000008744	-0.469739439	####	###	KLHL42	protein_coding	kelch like fa	12	27780048	27803040
ENSG0000015040	0.547958693	####	###	TMCO3	protein_coding	transmembr	13	113491021	113554590

ENSG0000026100	2.345193904	####	###	LINC01572	lncRNA	long interge	16	72236281	72665014
ENSG0000019647	-1.428298946	####	###	C20orf96	protein_codinc	chromosom	20	270863	290778
ENSG0000027096	3.272720725	####	###	AC016355.:	lncRNA	novel transc	15	67541072	67542604
ENSG0000022197	0.330129293	####	###	CCNL2	protein_codinc	cyclin L2 [Sc	1	1385711	1399335
ENSG0000001040	0.263340085	####	###	IDS	protein_codinc	iduronate 2 X		149476988	149521096
ENSG0000016474	-4.800540101	####	###	HNF4G	protein_codinc	hepatocyte	8	75407914	75566834
ENSG0000010606	-1.493041377	####	###	CHN2	protein_codinc	chimerin 2 [	7	29146569	29514328
ENSG0000014004	-0.9966526	####	###	JDP2	protein_codinc	Jun dimeriz	14	75427716	75474111
ENSG0000017393	-4.760160534	####	###	SLCO4C1	protein_codinc	solute carrie	5	102233986	102296284
ENSG0000008583	-0.506787056	####	###	EPS15	protein_codinc	epidermal g	1	51354263	51519266
ENSG0000010131	-2.866649697	####	###	FERMT1	protein_codinc	fermitin fam	20	6074845	6123030
ENSG0000009988	0.558574938	####	###	ARVCF	protein_codinc	ARVCF delta	22	19969896	20016823
ENSG0000010092	-0.673171337	####	###	TM9SF1	protein_codinc	transmembı	14	24189149	24195687
ENSG0000019805	-3.075481537	####	###	SIRPA	protein_codinc	signal regul	20	1894167	1940592
ENSG0000011987	0.606764889	####	###	CRIPT	protein_codinc	CXXC repea	2	46616416	46630176
ENSG0000015188	-1.003112355	####	###	TMEM267	protein_codinc	transmembı	5	43444252	43483893
ENSG0000025545	1.113359412	####	###	AP003486.:	lncRNA	novel transc	11	130866254	130870247
ENSG0000016339	-2.885781159	####	###	SLC22A15	protein_codinc	solute carrie	1	115976513	116070054
ENSG0000018516	-0.468857953	####	###	NOMO2	protein_codinc	NODAL mo	16	18417325	18562211
ENSG0000012461	-1.982274006	####	###	ZNF391	protein_codinc	zinc finger p	6	27374615	27403904
ENSG0000014404	0.524332714	####	###	TEX261	protein_codinc	testis expres	2	70985942	70994873
ENSG0000014032	0.840696186	####	###	CDAN1	protein_codinc	codanin 1 [S	15	42723544	42737128
ENSG0000005465	0.352904269	####	###	SYNE2	protein_codinc	spectrin rep	14	63761899	64226433
ENSG0000014350	-1.107090016	####	###	DUSP10	protein_codinc	dual specific	1	221701424	221742089
ENSG0000011185	1.118465237	####	###	NEDD9	protein_codinc	neural precu	6	11183298	11382348
ENSG0000010074	0.75955923	####	###	GSKIP	protein_codinc	GSK3B inter	14	96363452	96387288
ENSG0000016756	-0.608812922	####	###	NCKAP5L	protein_codinc	NCK associa	12	49791146	49828750
ENSG0000013748	-0.582038979	####	###	ARRB1	protein_codinc	arrestin beta	11	75260122	75351705
ENSG0000015398	-1.08758083	####	###	GDPD1	protein_codinc	glycerophos	17	59220467	59275970
ENSG0000017656	3.114209159	####	###	CNTD1	protein_codinc	cyclin N-ter	17	42798800	42811587
ENSG0000016033	1.356658272	####	###	ZNF761	protein_codinc	zinc finger p	19	53445001	53458261
ENSG0000011968	0.460636344	####	###	AREL1	protein_codinc	apoptosis re	14	74653437	74713108
ENSG0000017576	0.612215608	####	###	TOMM5	protein_codinc	translocase	9	37582646	37592604
ENSG0000024403	-0.346898585	####	###	DDOST	protein_codinc	dolichyl-dip	1	20651767	20661544
ENSG0000009674	-0.278427073	####	###	HNRNPH3	protein_codinc	heterogene	10	68331174	68343191
ENSG0000010858	-0.588579909	####	###	GOSR1	protein_codinc	golgi SNAP	17	30477362	30527592
ENSG0000011114	-0.330584844	####	###	METAP2	protein_codinc	methionyl a	12	95473520	95515839
ENSG0000019881	-0.424743873	####	###	FOXJ3	protein_codinc	forkhead bo	1	42176539	42335877
ENSG0000014164	1.572932599	####	###	ELAC1	protein_codinc	elaC ribonu	18	50967991	50988121
ENSG0000015075	0.901248536	####	###	ATPSCKMT	protein_codinc	ATP synthas	5	10225507	10249897
ENSG0000012851	-0.500181408	####	###	DOCK4	protein_codinc	dedicator o	7	111726110	112206407
ENSG0000010015	-0.971891093	####	###	PICK1	protein_codinc	protein inte	22	38056311	38075701
ENSG0000010136	0.3710063	####	###	MAPRE1	protein_codinc	microtubule	20	32819954	32850405
ENSG0000013162	0.443941881	####	###	PPFIA1	protein_codinc	PTPRF intera	11	70270700	70385312
ENSG0000015437	-0.585254038	####	###	TRIM11	protein_codinc	tripartite mc	1	228393673	228406835
ENSG0000014743	1.043961689	####	###	BIN3	protein_codinc	bridging int	8	22620418	22669148
ENSG0000011797	1.019769475	####	###	CHRNB4	protein_codinc	cholinergic	15	78624111	78727754
ENSG0000010572	-0.592067719	####	###	GSK3A	protein_codinc	glycogen sy	19	42230190	42242625
ENSG0000010079	-0.370937609	####	###	PPP4R3A	protein_codinc	protein pho	14	91457611	91510554
ENSG0000016881	-0.548989911	####	###	ZNF507	protein_codinc	zinc finger p	19	32345594	32387667
ENSG0000019737	-1.798995169	####	###	SLC22A5	protein_codinc	solute carrie	5	132369710	132395613
ENSG0000010210	-0.587435208	####	###	PQBP1	protein_codinc	polyglutami X		48890197	48903143
ENSG0000014346	-0.379770846	####	###	SYT14	protein_codinc	synaptotagr	1	209900923	210171389
ENSG0000016906	0.450462802	####	###	UPF3A	protein_codinc	UPF3A regu	13	114281601	114305817
ENSG0000012860	-2.300005398	####	###	LRRC17	protein_codinc	leucine rich	7	102913000	102945111
ENSG0000013593	-0.731480074	####	###	ARMC9	protein_codinc	armadillo re	2	231198546	231376848
ENSG0000022737	0.595637948	####	###	TP73-AS1	transcribed_un	TP73 antiser	1	3735511	3747373
ENSG0000005937	-1.744637583	####	###	PARP12	protein_codinc	poly(ADP-ri	7	140023749	140062951

ENSG0000024225	0.83372922	####	###	C22orf39	protein_coding	chromosom	22	19351368	19448232
ENSG0000013610	0.343612215	####	###	CKAP2	protein_coding	cytoskeletor	13	52455429	52476628
ENSG0000014108	0.686277378	####	###	RANBP10	protein_coding	RAN binding	16	67723070	67806652
ENSG0000011611	-2.746277532	####	###	PARD3B	protein_coding	par-3 family	2	204545475	205620162
ENSG0000010010	-0.482009294	####	###	PATZ1	protein_coding	POZ/BTB ar	22	31325804	31346346
ENSG0000010488	0.763997534	####	###	PLEKHJ1	protein_coding	pleckstrin hc	19	2230084	2237704
ENSG0000015920	-1.675224105	####	###	CIART	protein_coding	circadian as	1	150282543	150287093
ENSG0000010532	-2.075948987	####	###	BBC3	protein_coding	BCL2 bindin	19	47220822	47232766
ENSG0000017220	1.396914534	####	###	ID4	protein_coding	inhibitor of	6	19837370	19842197
ENSG0000027966	-2.891781484	####	###	AL603839.4	TEC	TEC	1	40473055	40474059
ENSG0000018264	1.295379198	####	###	LINC01006	lncRNA	long interge	7	156388922	156640654
ENSG0000016682	-3.339933568	####	###	ANPEP	protein_coding	alanyl aminc	15	89784895	89815401
ENSG0000013614	-2.778932582	####	###	PHF11	protein_coding	PHD finger	13	49495610	49528981
ENSG0000011535	0.336190949	####	###	CCDC88A	protein_coding	coiled-coil c	2	55287842	55419895
ENSG0000007459	-1.266915724	####	###	NUAK1	protein_coding	NUAK family	12	106063340	106140033
ENSG0000011319	0.379913452	####	###	FAF2	protein_coding	Fas associat	5	176447628	176510074
ENSG0000014403	0.857381386	####	###	EXOC6B	protein_coding	exocyst com	2	72175984	72826041
ENSG0000016414	-2.328324479	####	###	FAM160A1	protein_coding	family with s	4	151409176	151670503
ENSG0000019886	-0.503989095	####	###	LTN1	protein_coding	listerin E3 ul	21	28928144	28992956
ENSG0000014337	-1.723442124	####	###	CGN	protein_coding	cingulin [So	1	151510510	151538692
ENSG0000010014	0.684156243	####	###	POLR2F	protein_coding	RNA polyme	22	37952607	38041915
ENSG0000011520	0.541046143	####	###	MPV17	protein_coding	mitochondr	2	27309492	27325680
ENSG0000011751	-0.358851446	####	###	CNN3	protein_coding	calponin 3 [	1	94896949	94927223
ENSG0000018387	-1.330582184	####	###	UTY	protein_coding	ubiquitously Y		13248379	13480673
ENSG0000018887	1.486132598	####	###	FBF1	protein_coding	Fas binding	17	75909574	75941140
ENSG0000014842	-0.656730117	####	###	USP6NL	protein_coding	USP6 N-terr	10	11453946	11611754
ENSG0000018293	0.365389376	####	###	SRPRA	protein_coding	SRP recepto	11	126262938	126269144
ENSG0000006228	-1.603921556	####	###	DGAT2	protein_coding	diacylglycer	11	75759512	75801535
ENSG0000017762	0.729400687	####	###	GBA	protein_coding	glucosylcera	1	155234452	155244699
ENSG0000018422	-1.403733478	####	###	ACOT1	protein_coding	acyl-CoA th	14	73537143	73543796
ENSG0000014341	-0.335633329	####	###	CERS2	protein_coding	ceramide sy	1	150960583	150975003
ENSG0000010035	0.495961056	####	###	FOXRED2	protein_coding	FAD depenc	22	36487190	36507101
ENSG0000014542	-0.215498447	####	###	RPS3A	protein_coding	ribosomal p	4	151099624	151104642
ENSG0000016468	-0.654850784	####	###	HEY1	protein_coding	hes related	8	79762371	79767857
ENSG0000013777	0.388941411	####	###	CTDSPL2	protein_coding	CTD small p	15	44427622	44529038
ENSG0000027709	-4.69673937	####	###	AC242852.2	unprocessed_p	otopetrin 1	1	143354191	143386122
ENSG0000010599	0.3730735	####	###	DNAJB6	protein_coding	DnaJ heat sl	7	157335381	157417439
ENSG0000013132	-0.665089787	####	###	TRAF3	protein_coding	TNF recepto	14	102777449	102911500
ENSG0000011790	0.380603153	####	###	RCN2	protein_coding	reticulocalbi	15	76931738	76954393
ENSG0000015935	0.320027386	####	###	PSMD4	protein_coding	proteasome	1	151254709	151267479
ENSG0000013240	0.730676627	####	###	TMEM128	protein_coding	transmembr	4	4235542	4248223
ENSG0000011463	-0.546344196	####	###	PODXL2	protein_coding	podocalyxin	3	127629185	127672802
ENSG0000013243	-0.930922265	####	###	LANCL2	protein_coding	LanC like 2	7	55365448	55433742
ENSG0000010920	-7.003465762	####	###	ODAM	protein_coding	odontogeni	4	70196496	70204576
ENSG0000023261	1.598269408	####	###	AL683813.2	lncRNA	novel transc X		136840931	136847797
ENSG0000010677	0.472382866	####	###	PRUNE2	protein_coding	prune homc	9	76611376	76906114
ENSG0000013727	0.720827697	####	###	BPHL	protein_coding	biphenyl hy	6	3118374	3153578
ENSG0000019701	1.65569818	####	###	SERTAD1	protein_coding	SERTA dom	19	40421589	40425992
ENSG0000001954	0.77209184	####	###	SNAI2	protein_coding	snail family	8	48917598	48921740
ENSG0000017547	-2.598897621	####	###	MCTP1	protein_coding	multiple C2	5	94703741	95284575
ENSG0000006191	0.74059564	####	###	GUCY1B1	protein_coding	guanylate c	4	155758992	155807811
ENSG0000011114	-0.498109135	####	###	ELK3	protein_coding	ETS transcri	12	96194375	96269824
ENSG0000013649	0.600478138	####	###	BRIP1	protein_coding	BRCA1 inter	17	61679139	61863528
ENSG0000012317	-0.739518054	####	###	EBPL	protein_coding	EBP like [So	13	49660674	49691486
ENSG0000012069	-0.346429968	####	###	HSPH1	protein_coding	heat shock p	13	31134973	31162388
ENSG0000011997	0.749645903	####	###	DENND10	protein_coding	DENN doma	10	119104086	119137984
ENSG0000013331	0.266818294	####	###	RTN3	protein_coding	reticulon 3 [	11	63681446	63759891
ENSG0000013844	0.345539874	####	###	ITGAV	protein_coding	integrin sub	2	186590056	186680901

ENSG0000016040	-0.924487397	####	###	ST6GALNA	protein_coding	ST6 N-acety	9	127885321	127905408
ENSG0000025791	-2.352591962	####	###	DDN-AS1	lncRNA	DDN and PI	12	48998367	49019235
ENSG0000013687	0.789152052	####	###	STX17	protein_coding	syntaxin 17	9	99906654	99974534
ENSG0000014129	-0.491814528	####	###	SSH2	protein_coding	slingshot pr	17	29625938	29930276
ENSG0000012069	-0.564025278	####	###	EXOSC8	protein_coding	exosome cc	13	36998816	37009614
ENSG0000013784	-2.747676587	####	###	PLCB2	protein_coding	phospholipa	15	40278176	40307935
ENSG0000017168	-1.556696222	####	###	PLEKHG5	protein_coding	pleckstrin hc	1	6467122	6520074
ENSG0000010064	-0.300144443	####	###	HIF1A	protein_coding	hypoxia indu	14	61695513	61748259
ENSG0000015122	0.979899929	####	###	SLC2A13	protein_coding	solute carrie	12	39755025	40106089
ENSG0000023012	-0.501352658	####	###	ACBD6	protein_coding	acyl-CoA bi	1	180269653	180502954
ENSG0000022574	-2.452739674	####	###	MEG8	lncRNA	maternally e	14	100894770	101038859
ENSG0000012795	0.825362954	####	###	STYXL1	protein_coding	serine/threc	7	75996338	76048004
ENSG0000014427	-0.58596791	####	###	GALNT13	protein_coding	polypeptide	2	153871922	154453979
ENSG0000014357	-0.80409677	####	###	CREB3L4	protein_coding	cAMP respc	1	153967534	153974361
ENSG0000001950	-4.296275907	####	###	SYT13	protein_coding	synaptotagr	11	45240302	45286341
ENSG0000010290	0.643019934	####	###	CENPT	protein_coding	centromere	16	67828157	67847811
ENSG0000020653	-1.150559782	####	###	CFAP44	protein_coding	cilia and flag	3	113286930	113441610
ENSG0000001358	1.945187182	####	###	HEBP1	protein_coding	heme bindir	12	12974870	13000265
ENSG0000010218	0.692089714	####	###	CD99L2	protein_coding	CD99 molec	X	150766336	150898816
ENSG0000017314	0.593077948	####	###	NOC3L	protein_coding	NOC3 like D	10	94333226	94362959
ENSG0000018383	-4.056160747	####	###	PNMA3	protein_coding	PNMA famil	X	153056409	153060467
ENSG0000011118	-2.900110011	####	###	WNT5B	protein_coding	Wnt family r	12	1529891	1647212
ENSG0000024212	-0.604691405	####	###	SNHG3	lncRNA	small nuclec	1	28505980	28510892
ENSG0000027876	1.168862323	####	###	CORO7-PA	protein_coding	CORO7-PA	CHR_HSCHR	4340251	4422453
ENSG0000027507	1.857071194	####	###	NUDT18	protein_coding	nudix hydro	8	22106874	22109419
ENSG0000017372	-0.278289854	####	###	TOMM20	protein_coding	translocase	1	235109341	235128837
ENSG0000001513	-0.76082323	####	###	CCDC88C	protein_coding	coiled-coil c	14	91271323	91417844
ENSG0000016916	-1.028762636	####	###	CPT1C	protein_coding	carnitine pa	19	49690898	49713731
ENSG0000016394	-0.934805889	####	###	ARHGEF3	protein_coding	Rho guanine	3	56727418	57079329
ENSG0000016011	-0.913863936	####	###	NR2F6	protein_coding	nuclear rece	19	17231883	17245940
ENSG0000008037	0.435152707	####	###	RAB21	protein_coding	RAB21, mer	12	71754863	71800286
ENSG0000019792	0.927650255	####	###	ZNF677	protein_coding	zinc finger p	19	53235381	53254898
ENSG0000010007	-0.353811159	####	###	GRK3	protein_coding	G protein-c	22	25564675	25729294
ENSG0000011890	0.40602017	####	###	UBN1	protein_coding	ubinuclein 1	16	4846665	4882401
ENSG0000016657	3.227276754	####	###	IQCD	protein_coding	IQ motif cor	12	113195441	113221094
ENSG0000013450	-0.960269649	####	###	KCTD1	protein_coding	potassium c	18	26454910	26657401
ENSG0000015208	-0.654501108	####	###	MZT2B	protein_coding	mitotic spin	2	130181737	130190729
ENSG0000016166	2.750140078	####	###	ASB16	protein_coding	ankyrin repe	17	44170447	44179084
ENSG0000014315	-0.326637831	####	###	POGK	protein_coding	pogo transp	1	166839447	166856359
ENSG0000013782	-0.748642843	####	###	LRRC49	protein_coding	leucine rich	15	70853239	71053658
ENSG0000011264	0.446025512	####	###	PPP2R5D	protein_coding	protein pho	6	42984553	43012342
ENSG0000016268	-0.551945445	####	###	AGL	protein_coding	amylo-alph	1	99850361	99924023
ENSG0000016351	-0.688731034	####	###	CWC22	protein_coding	CWC22 splic	2	179944876	180007297
ENSG0000017799	-1.477292496	####	###	DPY19L2	protein_coding	dpy-19 like	12	63558913	63668939
ENSG0000011298	0.467227886	####	###	BRD8	protein_coding	bromodoma	5	138139770	138178953
ENSG0000014098	0.541339856	####	###	RHOT2	protein_coding	ras homolog	16	668105	674174
ENSG0000015225	0.993466334	####	###	SPC25	protein_coding	SPC25 comp	2	168834132	168913371
ENSG0000017766	0.884615575	####	###	PNPLA2	protein_coding	patatin like	11	818914	825573
ENSG0000013405	0.329186201	####	###	CCNB1	protein_coding	cyclin B1 [Sc	5	69167135	69178245
ENSG0000010462	0.585566421	####	###	ERI1	protein_coding	exoribonucl	8	9002147	9116746
ENSG0000016925	-0.4379461	####	###	NMD3	protein_coding	NMD3 ribos	3	161104696	161253532
ENSG0000025511	0.633583953	####	###	CHMP1B	protein_coding	charged mu	18	11851413	11854444
ENSG0000020636	-2.421962912	####	###	ZBTB12	protein_coding	zinc finger a	CHR_HSCHR	31889987	31892374
ENSG0000012574	-3.906650583	####	###	FOSB	protein_coding	FosB proto-	19	45467995	45475179
ENSG0000007946	-0.775077209	####	###	PAFAH1B3	protein_coding	platelet acti	19	42297033	42303546
ENSG0000013963	1.08432298	####	###	CSAD	protein_coding	cysteine sulf	12	53157663	53180909
ENSG0000016732	0.680339114	####	###	STIM1	protein_coding	stromal inte	11	3854527	4093210
ENSG0000017297	-0.698528316	####	###	KAT5	protein_coding	lysine acetyl	11	65711996	65719604

ENSG0000013404	-0.500450073	####	###	IER3IP1	protein_coding	immediate e	18	47152834	47176364
ENSG0000005411	0.308371655	####	###	THRAP3	protein_coding	thyroid horr	1	36224432	36305357
ENSG0000013302	0.287073794	####	###	MYH10	protein_coding	myosin heav	17	8474207	8630761
ENSG0000010608	0.49343458	####	###	PLEKHA8	protein_coding	pleckstrin h	7	30027404	30130483
ENSG0000010022	-0.402734628	####	###	FBXO7	protein_coding	F-box prote	22	32474676	32498829
ENSG0000019640	0.436067019	####	###	EVL	protein_coding	Enah/Vasp-	14	99971449	100144236
ENSG0000010483	-1.230187113	####	###	SARS2	protein_coding	seryl-tRNA	19	38915266	38930763
ENSG0000024241	0.924231542	####	###	PCDHGC4	protein_coding	protocadhe	5	141484997	141512979
ENSG0000013081	-3.610110888	####	###	SHFL	protein_coding	shiftless ant	19	10086122	10093252
ENSG0000014359	-2.788463463	####	###	EFNA3	protein_coding	ephrin A3 [S	1	155078837	155087538
ENSG0000010487	-1.530823359	####	###	FCGRT	protein_coding	Fc fragment	19	49506816	49526428
ENSG0000017415	1.137472908	####	###	CYB561D1	protein_coding	cytochrome	1	109494052	109502932
ENSG0000012969	0.50172945	####	###	ASH2L	protein_coding	ASH2 like, h	8	38105493	38144076
ENSG0000013147	-3.008824157	####	###	RAMP2	protein_coding	receptor act	17	42758447	42763041
ENSG0000024427	-2.563814616	####	###	DBNDD2	protein_coding	dysbindin d	20	45406057	45410610
ENSG0000007771	-1.718379858	####	###	SLC25A43	protein_coding	solute carrie	X	119399336	119454478
ENSG0000016788	-0.401081937	####	###	SRP68	protein_coding	signal recog	17	76038775	76072517
ENSG0000010584	0.651364486	####	###	TWISTNB	protein_coding	TWIST neigl	7	19695461	19709037
ENSG0000016031	-0.559597948	####	###	PRMT2	protein_coding	protein argi	21	46635595	46665124
ENSG0000019664	0.337438181	####	###	RABL6	protein_coding	RAB, memb	9	136807943	136841187
ENSG0000015638	1.272419364	####	###	SFR1	protein_coding	SWI5 deper	10	104122058	104126385
ENSG0000008628	0.912525195	####	###	EPDR1	protein_coding	ependymin	7	37683843	37951936
ENSG0000015879	0.84776686	####	###	NIT1	protein_coding	nitrilase 1 [S	1	161118086	161125445
ENSG0000005459	1.486132009	####	###	FOXC1	protein_coding	forkhead bc	6	1609915	1613897
ENSG0000007638	0.492715213	####	###	SPAG5	protein_coding	sperm assoc	17	28577565	28599025
ENSG0000016046	-0.932130208	####	###	BRSK1	protein_coding	BR serine/th	19	55282072	55312562
ENSG0000027252	0.679630685	####	###	NA	NA	NA	NA	NA	NA
ENSG0000013312	-1.715879224	####	###	STARD13	protein_coding	StAR relatec	13	33103137	33350630
ENSG0000016540	-1.13495692	####	###	MARCHF8	protein_coding	membrane	10	45454585	45594906
ENSG0000013276	-0.9439495	####	###	MMACHC	protein_coding	metabolism	1	45500300	45513382
ENSG0000011316	0.321792533	####	###	HMGCR	protein_coding	3-hydroxy-	5	75336329	75362101
ENSG0000010894	-1.341529023	####	###	EFNB3	protein_coding	ephrin B3 [S	17	7705202	7711372
ENSG0000010041	0.391645987	####	###	ACO2	protein_coding	aconitase 2	22	41469117	41528989
ENSG0000009702	0.484487428	####	###	ACOT7	protein_coding	acyl-CoA th	1	6264269	6394391
ENSG0000025534	-5.99259202	####	###	NOX5	protein_coding	NADPH oxic	15	68930525	69062762
ENSG0000011940	0.3630048	####	###	FBXW2	protein_coding	F-box and \	9	120751978	120793416
ENSG0000014289	0.585919432	####	###	PIGK	protein_coding	phosphatidy	1	77088989	77219430
ENSG0000025839	-2.536399549	####	###	AL117190.1	lncRNA	novel transc	14	100836269	100947194
ENSG0000015144	0.637034795	####	###	VIPAS39	protein_coding	VPS33B inte	14	77426675	77457952
ENSG0000016777	-0.750462132	####	###	RCOR2	protein_coding	REST corepr	11	63911230	63917164
ENSG0000017773	0.267029021	####	###	HNRNPA0	protein_coding	heterogene	5	137745651	137754363
ENSG0000013015	-1.837857824	####	###	DOCK6	protein_coding	dedicator of	19	11199295	11262524
ENSG0000027311	2.114601989	####	###	AC144652.	lncRNA	novel transc	7	155295918	155297541
ENSG0000018447	-0.865296257	####	###	TXNRD2	protein_coding	thioredoxin	22	19875517	19941820
ENSG0000016784	-1.061298217	####	###	ZNF232	protein_coding	zinc finger p	17	5105541	5123116
ENSG0000015854	0.415129605	####	###	ZC3H18	protein_coding	zinc finger C	16	88570403	88631964
ENSG0000022515	-1.708480798	####	###	AC012354.	lncRNA	novel transc	2	44954664	44968762
ENSG0000019713	1.919730674	####	###	ZNF257	protein_coding	zinc finger p	19	22052430	22091480
ENSG0000005514	0.67757942	####	###	FAM114A2	protein_coding	family with s	5	153990148	154038936
ENSG0000017182	-1.135114011	####	###	FBXL14	protein_coding	F-box and l	12	1565993	1594581
ENSG0000006662	0.708465405	####	###	EML1	protein_coding	EMAP like 1	14	99737693	99942060
ENSG0000018082	0.922795394	####	###	PSMG4	protein_coding	proteasome	6	3231403	3303373
ENSG0000019670	0.521076518	####	###	AMZ2	protein_coding	archaelysin	17	68206076	68257712
ENSG0000021397	1.475861699	####	###	NA	NA	NA	NA	NA	NA
ENSG0000011868	0.540221719	####	###	MYL12B	protein_coding	myosin light	18	3261479	3278461
ENSG0000027956	-3.093874162	####	###	AC020763.	TEC	novel transc	16	70661667	70665836
ENSG0000018651	3.748607581	####	###	ARHGAP30	protein_coding	Rho GTPase	1	161046946	161069970
ENSG0000013756	0.541131734	####	###	GGH	protein_coding	gamma-glu	8	63015079	63038806

ENSG0000013261	0.37771133	####	###	VPS4A	protein_coding	vacuolar prc	16	69311350	69326939
ENSG0000007961	0.433802162	####	###	KIF22	protein_coding	kinesin fami	16	29790719	29805384
ENSG0000016329	0.431774965	####	###	PAQR3	protein_coding	progestin ar	4	78887127	78939438
ENSG0000027204	0.903550609	####	###	GTF2H5	protein_coding	general trar	6	158168350	158199344
ENSG0000016027	-0.392384124	####	###	RALGDS	protein_coding	ral guanine	9	133097720	133149334
ENSG0000021274	0.852158465	####	###	RTL8B	protein_coding	retrotranspc	X	135020513	135022542
ENSG0000013911	-0.256424806	####	###	KIF21A	protein_coding	kinesin fami	12	39293228	39443390
ENSG0000006548	-1.144736855	####	###	PDIA5	protein_coding	protein disu	3	123067025	123225227
ENSG0000022437	1.65850712	####	###	TCF19	protein_coding	transcriptior	CHR_HSCHR	31151089	31156758
ENSG0000019864	-0.450570024	####	###	STK39	protein_coding	serine/threc	2	167954020	168247595
ENSG0000006638	-1.543841024	####	###	MPPED2	protein_coding	metallophos	11	30384493	30586872
ENSG0000011696	-0.739286816	####	###	NID1	protein_coding	nidogen 1 [	1	235975830	236065109
ENSG0000014285	0.688916832	####	###	ITGB3BP	protein_coding	integrin sub	1	63440770	63593721
ENSG0000027705	-0.327950518	####	###	GTF2IP1	transcribed_un	general trar	7	75185385	75237696
ENSG0000013526	-0.997427343	####	###	TES	protein_coding	testin LIM d	7	116210506	116258783
ENSG0000013228	0.600599035	####	###	TIMM10B	protein_coding	translocase	11	6481501	6484681
ENSG0000025031	1.339007382	####	###	ZNF718	protein_coding	zinc finger p	4	124501	202303
ENSG0000013873	-1.510080478	####	###	PDE5A	protein_coding	phosphodie	4	119494397	119628804
ENSG0000015323	-1.72435664	####	###	NR4A2	protein_coding	nuclear rece	2	156324437	156342348
ENSG0000011541	0.314918988	####	###	GLS	protein_coding	glutaminase	2	190880821	190965552
ENSG0000013652	0.278316482	####	###	TRA2B	protein_coding	transformer	3	185914558	185938103
ENSG0000001081	-0.658624132	####	###	HIVEP2	protein_coding	HIVEP zinc f	6	142751469	142956698
ENSG0000014926	-0.661553656	####	###	PAK1	protein_coding	p21 (RAC1)	11	77322017	77474635
ENSG0000017179	0.383879481	####	###	CTPS1	protein_coding	CTP synthas	1	40979688	41012565
ENSG0000012141	1.201582982	####	###	ZNF211	protein_coding	zinc finger p	19	57630395	57644041
ENSG0000016788	-0.884415384	####	###	MGAT5B	protein_coding	alpha-1,6-n	17	76868404	76950393
ENSG0000009115	0.759018954	####	###	WDR7	protein_coding	WD repeat c	18	56651343	57036606
ENSG0000024146	0.445758238	####	###	ATP5MF	protein_coding	ATP synthas	7	99448475	99466186
ENSG0000013805	-0.797837556	####	###	THUMPD2	protein_coding	THUMP dor	2	39736060	39779267
ENSG0000025717	-2.449899509	####	###	AC009318.1	lncRNA	novel transc	12	29277955	29317848
ENSG0000019871	-1.16391926	####	###	GLMP	protein_coding	glycosylatec	1	156290089	156295689
ENSG0000011362	-0.667771383	####	###	TXNDC15	protein_coding	thioredoxin	5	134874371	134901635
ENSG0000012626	-0.364117019	####	###	UBA2	protein_coding	ubiquitin lik	19	34428352	34471251
ENSG0000016933	-1.583072941	####	###	MINAR1	protein_coding	membrane i	15	79432336	79472304
ENSG0000014312	-0.701128802	####	###	CELSR2	protein_coding	cadherin EG	1	109249539	109275751
ENSG0000020637	-0.955089639	####	###	EHMT2	protein_coding	euchromatic	CHR_HSCHR	31870139	31888078
ENSG0000023131	1.628625881	####	###	MAP4K3-D	lncRNA	MAP4K3 div	2	39436530	39665343
ENSG0000027763	-5.955939762	####	###	IGHG1	IG_C_gene	immunoglol	CHR_HSCHR	105737114	105743842
ENSG0000017694	-0.640781655	####	###	THAP4	protein_coding	THAP doma	2	241584405	241637158
ENSG0000002580	0.381886124	####	###	KPNA6	protein_coding	karyopherin	1	32108056	32176563
ENSG0000014848	0.577455354	####	###	MINDY3	protein_coding	MINDY lysir	10	15778170	15860507
ENSG0000011612	0.558366679	####	###	ALMS1	protein_coding	ALMS1 cent	2	73385758	73625166
ENSG0000013232	-1.170820704	####	###	PER2	protein_coding	period circa	2	238244044	238290102
ENSG0000016180	0.347361652	####	###	RACGAP1	protein_coding	Rac GTPase	12	49976923	50033136
ENSG0000013498	-0.387688515	####	###	APC	protein_coding	APC regulat	5	112707498	112846239
ENSG0000010781	-0.625521919	####	###	TWNK	protein_coding	twinkle mtD	10	100987367	100994403
ENSG0000013218	-0.723499808	####	###	NUP210	protein_coding	nucleoporin	3	13316235	13420322
ENSG0000018634	-2.688343639	####	###	THBS2	protein_coding	thrombospc	6	169215780	169254044
ENSG0000012568	-0.312567243	####	###	MED1	protein_coding	mediator cc	17	39404285	39451272
ENSG0000012311	1.021054229	####	###	NECAB1	protein_coding	N-terminal	8	90791741	90959393
ENSG0000016213	0.629098505	####	###	NEU3	protein_coding	neuraminida	11	74988279	75018893
ENSG0000018779	-1.133439763	####	###	CARD9	protein_coding	caspase recr	9	136361903	136373681
ENSG0000012658	0.532482573	####	###	BECN1	protein_coding	beclin 1 [So	17	42810134	42833350
ENSG0000013175	0.820688932	####	###	RARA	protein_coding	retinoic acic	17	40309180	40357643
ENSG0000015435	-0.55231004	####	###	OBSCN	protein_coding	obscurin, cy	1	228208063	228378876
ENSG0000016353	-0.90328218	####	###	NFASC	protein_coding	neurofascin	1	204828651	205022822
ENSG0000019834	1.905494084	####	###	ZNF813	protein_coding	zinc finger p	19	53467733	53496255
ENSG0000018415	1.218103667	####	###	LRTOMT	protein_coding	leucine rich	11	72080337	72096895

ENSG0000019618	-1.487572443	####	###	TMEM63A	protein_coding	transmembr	1	225845536	225882380
ENSG0000016360	-0.420695274	####	###	NEPRO	protein_coding	nucleolus ar	3	113002444	113019861
ENSG0000017783	0.592334607	####	###	CHID1	protein_coding	chitinase dc	11	867859	915058
ENSG0000015755	-9.235948225	####	###	ERG	protein_coding	ETS transcri	21	38380027	38661780
ENSG0000017574	-1.275847687	####	###	NR2F1	protein_coding	nuclear rece	5	93583222	93594611
ENSG0000011444	0.754158154	####	###	IFT57	protein_coding	intraflagella	3	108160812	108222435
ENSG0000027244	1.196553441	####	###	AL135925.1	lncRNA	novel transc	10	79825902	79827602
ENSG0000026091	-3.194823562	####	###	AC107398.3	lncRNA	novel transc	4	47431960	47438959
ENSG0000018215	0.821651172	####	###	MRPL41	protein_coding	mitochondr	9	137551879	137552555
ENSG0000013787	-0.540147753	####	###	SEMA6D	protein_coding	semaphorin	15	47184101	47774228
ENSG0000008411	-0.452475602	####	###	SSH1	protein_coding	slingshot pr	12	108778191	108857590
ENSG0000010030	0.434057983	####	###	TTLL12	protein_coding	tubulin tyros	22	43166622	43187134
ENSG0000011161	-0.440607176	####	###	KRR1	protein_coding	KRR1 small	12	75490863	75511636
ENSG0000016219	-1.15780808	####	###	LBHD1	protein_coding	LBH domain	11	62662817	62672255
ENSG0000018479	-1.900342684	####	###	OSBP2	protein_coding	oxysterol bi	22	30693782	30907824
ENSG0000013071	0.494618269	####	###	EXOSC2	protein_coding	exosome cc	9	130693721	130704894
ENSG0000023521	1.339226037	####	###	TSPY26P	transcribed_pr	testis specifi	20	32186477	32190527
ENSG0000013680	0.381570171	####	###	LRRC8A	protein_coding	leucine rich	9	128882133	128918039
ENSG0000024197	0.326326577	####	###	PI4KA	protein_coding	phosphatidy	22	20707691	20859417
ENSG0000008904	-1.258815078	####	###	P2RX7	protein_coding	purinergic re	12	121132819	121188032
ENSG0000015399	-1.33483164	####	###	SEMA3D	protein_coding	semaphorin	7	84995553	85186855
ENSG0000016360	-0.684454737	####	###	GTPBP8	protein_coding	GTP binding	3	112990984	113015060
ENSG0000022539	-0.875479504	####	###	NHEG1	lncRNA	neuroblasto	6	136982165	136993234
ENSG0000010072	0.717379674	####	###	TELO2	protein_coding	telomere ma	16	1493344	1510457
ENSG0000013992	-0.452950245	####	###	FRMD6	protein_coding	FERM doma	14	51489100	51730727
ENSG0000023422	-1.964631916	####	###	TMEM229A	protein_coding	transmembr	7	124030921	124033067
ENSG0000010570	0.547578088	####	###	SUGP1	protein_coding	SURP and G	19	19276018	19320509
ENSG0000012080	0.322044241	####	###	TMPO	protein_coding	thymopoieti	12	98515579	98550351
ENSG0000006932	0.378194862	####	###	VPS35	protein_coding	VPS35 retro	16	46656132	46689518
ENSG0000010490	-0.573043983	####	###	TRMT1	protein_coding	tRNA methy	19	13104902	13117567
ENSG0000015137	-1.618224056	####	###	ME3	protein_coding	malic enzym	11	86441108	86672636
ENSG0000021392	0.299303495	####	###	CSNK1E	protein_coding	casein kinas	22	38290691	38318084
ENSG0000020386	2.718055655	####	###	ATP1A1-AS	lncRNA	ATP1A1 ant	1	116378437	116421301
ENSG0000012398	0.358970681	####	###	ACSL3	protein_coding	acyl-CoA sy	2	222860942	222944639
ENSG0000006271	0.456826306	####	###	VMP1	protein_coding	vacuole mer	17	59707192	59842255
ENSG0000006673	0.370961757	####	###	ATG2B	protein_coding	autophagy r	14	96279195	96363341
ENSG0000023635	-1.532265762	####	###	PBX2	protein_coding	PBX homeo	CHR_HSCHR	32261167	32266609
ENSG0000014528	-0.88338732	####	###	SCD5	protein_coding	stearoyl-Co	4	82629539	82798796
ENSG0000019778	0.739513166	####	###	TAF13	protein_coding	TATA-box b	1	109062486	109076003
ENSG0000021527	1.08541339	####	###	HOMEZ	protein_coding	homeobox a	14	23272422	23299447
ENSG0000020430	-1.164207165	####	###	PBX2	protein_coding	PBX homeo	6	32184733	32190202
ENSG0000018077	-0.470697954	####	###	SLC36A4	protein_coding	solute carrie	11	93144174	93197991
ENSG0000019882	-3.65966771	####	###	CD247	protein_coding	CD247 mole	1	167430640	167518610
ENSG0000014948	-0.313250134	####	###	FADS1	protein_coding	fatty acid de	11	61799627	61829318
ENSG0000027036	2.47743703	####	###	HMGN3-AS	lncRNA	HMGN3 ant	6	79233718	79236797
ENSG0000008828	1.03390928	####	###	ASAP3	protein_coding	ArfGAP with	1	23428563	23484568
ENSG0000027301	0.699519769	####	###	AC008124.1	lncRNA	novel transc	12	45718046	45727775
ENSG0000010542	0.497192834	####	###	MEGF8	protein_coding	multiple EG	19	42325609	42378769
ENSG0000014440	-0.86132903	####	###	METTL21A	protein_coding	methyltrans	2	207580631	207625928
ENSG0000018906	-0.861708641	####	###	LITAF	protein_coding	lipopolysacc	16	11547722	11636381
ENSG0000010174	-0.536504011	####	###	ANKRD12	protein_coding	ankyrin repe	18	9136228	9285985
ENSG0000013713	0.912951903	####	###	ARHGEF39	protein_coding	Rho guanin	9	35658875	35675866
ENSG0000011468	-0.379568364	####	###	MRPL3	protein_coding	mitochondr	3	131462212	131502983
ENSG0000010319	0.436291598	####	###	TSC2	protein_coding	TSC comple	16	2047967	2089491
ENSG0000001296	0.615552903	####	###	UBR7	protein_coding	ubiquitin pr	14	93207241	93229215
ENSG0000012799	-0.50842246	####	###	CASD1	protein_coding	CAS1 doma	7	94509219	94557019
ENSG0000026053	0.837999119	####	###	NA	NA	NA	NA	NA	NA
ENSG0000016403	0.272245311	####	###	H2AZ1	protein_coding	H2A.Z varia	4	99948086	99950355

ENSG0000027974	2.94297469	####	###	AC132938.(	TEC	TEC	17	82462601	82464255
ENSG0000010728	0.581886869	####	###	NPDC1	protein_coding	neural proli	9	137039463	137046179
ENSG0000021507	0.444727754	####	###	BRD2	protein_coding	bromodoma	CHR_HSCHR	32897392	32910230
ENSG0000016500	-0.526969565	####	###	UBAP1	protein_coding	ubiquitin as	9	34179005	34252523
ENSG0000013282	-0.58813704	####	###	OSER1	protein_coding	oxidative st	20	44195939	44210771
ENSG0000018367	-4.258677042	####	###	GPR1	protein_coding	G protein-c	2	206175316	206218047
ENSG0000014931	0.322494246	####	###	AASDHPPT	protein_coding	aminoadipa	11	106075501	106098699
ENSG0000018221	2.146098131	####	###	HHIPL1	protein_coding	HHIP like 1	14	99645110	99680569
ENSG0000007260	0.665140767	####	###	CHFR	protein_coding	checkpoint	12	132822187	132956304
ENSG0000017446	2.678636233	####	###	ZCCHC12	protein_coding	zinc finger (	X	118823824	118826968
ENSG0000005701	-0.388166425	####	###	DCBLD2	protein_coding	discoidin, C	3	98795941	98901695
ENSG0000013808	-0.420975203	####	###	FBXO11	protein_coding	F-box prote	2	47789316	47906498
ENSG0000016095	0.546016743	####	###	LRRC14	protein_coding	leucine rich	8	144517992	144525172
ENSG0000011645	0.327154081	####	###	ATP5PB	protein_coding	ATP synthas	1	111448864	111462773
ENSG0000014322	0.382325492	####	###	NUF2	protein_coding	NUF2 comp	1	163266576	163355764
ENSG0000019893	0.761549148	####	###	CCDC167	protein_coding	coiled-coil	6	37482938	37499893
ENSG0000014100	0.390395715	####	###	TCF25	protein_coding	transcriptior	16	89873570	89913627
ENSG0000025025	-0.848819016	####	###	PKD1P6	transcribed_un	polycystin 1	16	15125242	15154564
ENSG0000024741	1.67209342	####	###	AP000802.1	lncRNA	novel transc	11	112959279	112963460
ENSG0000026033	-0.919855728	####	###	NA	NA	NA	NA	NA	NA
ENSG0000018504	-0.649654248	####	###	NELFA	protein_coding	negative elc	4	1982717	2041903
ENSG0000025465	-5.150737839	####	###	RTL1	protein_coding	retrotranspc	14	100879753	100903722
ENSG0000010680	0.842794788	####	###	C5	protein_coding	complemen	9	120952335	121050275
ENSG0000011495	0.489251566	####	###	DGUOK	protein_coding	deoxyguanc	2	73926826	73958961
ENSG0000014104	1.372887813	####	###	ZNF287	protein_coding	zinc finger p	17	16546954	16569204
ENSG0000022495	-1.33314684	####	###	PBX2	protein_coding	PBX homeo	CHR_HSCHR	32192405	32197856
ENSG0000002936	-0.269844451	####	###	BCLAF1	protein_coding	BCL2 associ	6	136256627	136289851
ENSG0000018026	0.548452398	####	###	FGD6	protein_coding	FYVE, RhoG	12	95076749	95217482
ENSG0000018315	0.815421547	####	###	RABIF	protein_coding	RAB interac	1	202878282	202889149
ENSG0000014123	-0.622616313	####	###	TOB1	protein_coding	transducer c	17	50862223	50867978
ENSG0000017901	0.257108267	####	###	MRFAP1	protein_coding	Morf4 family	4	6640091	6642745
ENSG0000013017	0.306647728	####	###	PRKCSH	protein_coding	protein kina	19	11435288	11450968
ENSG0000015311	-0.46274261	####	###	CAST	protein_coding	calpastatin [	5	96525267	96779595
ENSG0000026710	1.051630532	####	###	ILF3-DT	lncRNA	ILF3 diverge	19	10651862	10653844
ENSG0000018590	-0.727446618	####	###	KLHDC8B	protein_coding	kelch doma	3	49171598	49176486
ENSG0000007088	-4.113445913	####	###	EPHA8	protein_coding	EPH recepto	1	22563489	22603595
ENSG0000014955	0.365328678	####	###	CHEK1	protein_coding	checkpoint	11	125625665	125676255
ENSG0000010299	1.068750036	####	###	MMP15	protein_coding	matrix meta	16	58025754	58046901
ENSG0000023007	2.559865067	####	###	AL162231.2	lncRNA	novel transc	9	34665607	34681981
ENSG0000014474	-0.927645729	####	###	SLC25A26	protein_coding	solute carrie	3	66133610	66388116
ENSG0000007124	0.385853217	####	###	RPS6KA2	protein_coding	ribosomal p	6	166409364	166906451
ENSG0000016989	0.476260058	####	###	SYAP1	protein_coding	synapse ass	X	16719612	16765340
ENSG0000008905	0.438651115	####	###	SLC23A2	protein_coding	solute carrie	20	4852356	5010293
ENSG0000012256	0.234410898	####	###	CBX3	protein_coding	chromobox	7	26201162	26213607
ENSG0000013064	-3.208749967	####	###	CALY	protein_coding	calcyon neu	10	133324072	133336935
ENSG0000016847	0.592054413	####	###	REEP4	protein_coding	receptor acc	8	22138020	22141951
ENSG0000022940	-1.369010432	####	###	AL359853.1	lncRNA	novel transc	1	179816184	179818191
ENSG0000001129	0.64647292	####	###	TTC19	protein_coding	tetratricope	17	15999784	16045015
ENSG0000014524	0.725082562	####	###	CENPC	protein_coding	centromere	4	67468762	67545503
ENSG0000007867	0.23179877	####	###	PCM1	protein_coding	pericentriola	8	17922840	18027975
ENSG0000011218	-1.060255642	####	###	BACH2	protein_coding	BTB domain	6	89926528	90296908
ENSG0000013119	-2.800577719	####	###	NFATC1	protein_coding	nuclear fact	18	79395856	79529325
ENSG0000010596	-2.201817504	####	###	ADAP1	protein_coding	ArfGAP with	7	897900	955407
ENSG0000009048	-0.527357138	####	###	SPG21	protein_coding	SPG21 abhy	15	64963022	64990310
ENSG0000013720	0.434532145	####	###	CMTR1	protein_coding	cap methylt	6	37433219	37482827
ENSG0000010652	0.819598907	####	###	ANKMY2	protein_coding	ankyrin repe	7	16599779	16645817
ENSG0000011819	-0.783578554	####	###	DDX59	protein_coding	DEAD-box	1	200623896	200669907
ENSG0000013671	-0.484504946	####	###	IMP4	protein_coding	IMP U3 sma	2	130342877	130347967

ENSG0000005039	-0.569964869	####	###	MCUR1	protein_coding	mitochondr	6	13786557	13814568
ENSG0000007579	0.519830793	####	###	BCAP29	protein_coding	B cell recept	7	107579977	107629170
ENSG0000022474	-1.135347888	####	###	FLOT1	protein_coding	flotillin 1 [Sc	CHR_HSCHR	30806191	30820021
ENSG0000006677	-0.401923702	####	###	ARFGEF1	protein_coding	ADP ribosyl	8	67173511	67343781
ENSG0000023161	-1.537032493	####	###	PPT2	protein_coding	palmitoyl-p	CHR_HSCHR	32229863	32248406
ENSG0000008409	-0.531329355	####	###	NOA1	protein_coding	nitric oxide	4	56963350	56977606
ENSG0000014690	0.512419564	####	###	NOM1	protein_coding	nucleolar pr	7	156949712	156973176
ENSG0000009058	0.740804081	####	###	GNPTG	protein_coding	N-acetylglu	16	1351931	1364113
ENSG0000018182	1.692695616	####	###	RELL1	protein_coding	RELT like 1 [	4	37590800	37686376
ENSG0000017982	-1.070490175	####	###	MYADM	protein_coding	myeloid ass	19	53864763	53876435
ENSG0000013140	-1.651554553	####	###	LRRC4B	protein_coding	leucine rich	19	50516892	50568435
ENSG0000016005	0.479661164	####	###	BSDC1	protein_coding	BSD domair	1	32364633	32394731
ENSG0000010517	0.740507647	####	###	POP4	protein_coding	POP4 homc	19	29606283	29617237
ENSG0000014992	-0.287797787	####	###	ALDOA	protein_coding	aldolase, fru	16	30064164	30070457
ENSG0000011159	-0.411579444	####	###	CNOT2	protein_coding	CCR4-NOT	12	70242994	70355257
ENSG0000012597	0.407332087	####	###	DYNLRB1	protein_coding	dynein light	20	34516409	34540958
ENSG0000005591	-0.272323622	####	###	PUM2	protein_coding	pumilio RN/	2	20248691	20352234
ENSG0000021297	1.08227975	####	###	AC016747.1	lncRNA	novel transc	2	61141592	61144969
ENSG0000024633	2.758706072	####	###	EXTL3-AS1	lncRNA	EXTL3 antise	8	28690215	28702030
ENSG0000011939	0.348220321	####	###	RAB14	protein_coding	RAB14, mer	9	121178133	121223014
ENSG0000003221	-0.478107805	####	###	ARID4A	protein_coding	AT-rich inte	14	58298504	58373887
ENSG0000012955	0.43899054	####	###	NEDD8	protein_coding	NEDD8 ubic	14	24216857	24232367
ENSG0000025119	-9.085353471	####	###	LINC00589	lncRNA	long interge	8	29673922	29748109
ENSG0000011505	0.20580407	####	###	NCL	protein_coding	nucleolin [S	2	231453531	231483641
ENSG0000018666	0.906003238	####	###	BCDIN3D	protein_coding	BCDIN3 dor	12	49836043	49843106
ENSG0000014704	0.510119496	####	###	CASK	protein_coding	calcium/calr	X	41514934	41923517
ENSG0000015874	-1.085014833	####	###	NBL1	protein_coding	NBL1, DAN	1	19596979	19658456
ENSG0000016761	-1.648738132	####	###	TMEM145	protein_coding	transmemb	19	42313309	42325064
ENSG0000009116	-0.498777825	####	###	TXNL1	protein_coding	thioredoxin	18	56597209	56651600
ENSG0000010862	-9.081805094	####	###	ICAM2	protein_coding	intercellular	17	64002594	64020634
ENSG0000017459	1.572621727	####	###	TRAM1L1	protein_coding	translocatio	4	117083554	117085576
ENSG0000013169	0.746603036	####	###	NPHP4	protein_coding	nephrocysti	1	5862811	5992473
ENSG0000012991	-0.58062679	####	###	KLF16	protein_coding	Kruppel like	19	1852399	1863579
ENSG0000016344	0.740967657	####	###	TMEM169	protein_coding	transmemb	2	216081866	216102783
ENSG0000017479	0.847472725	####	###	THAP6	protein_coding	THAP doma	4	75513946	75550473
ENSG0000012360	-2.470737234	####	###	NMI	protein_coding	N-myc and	2	151270470	151289894
ENSG0000013441	-0.26853496	####	###	RPS15A	protein_coding	ribosomal p	16	18781295	18790383
ENSG0000013424	-1.818221348	####	###	WNT2B	protein_coding	Wnt family	1	112466541	112530165
ENSG0000025975	-0.372802374	####	###	NA	NA	NA	NA	NA	NA
ENSG0000017073	0.702921653	####	###	POLH	protein_coding	DNA polym	6	43576185	43620523
ENSG0000024685	0.769825779	####	###	STARD4-AS	lncRNA	STARD4 ant	5	111510396	111739726
ENSG0000006784	0.631105479	####	###	PDZD4	protein_coding	PDZ domair	X	153802166	153830565
ENSG0000017170	-1.882190795	####	###	RGS19	protein_coding	regulator of	20	64073181	64079988
ENSG0000012083	0.802173811	####	###	MTERF2	protein_coding	mitochondr	12	106977277	106987160
ENSG0000016673	0.351449442	####	###	GOLM2	protein_coding	golgi memb	15	44288719	44415758
ENSG0000016768	-1.008691798	####	###	ZNF444	protein_coding	zinc finger p	19	56132599	56160893
ENSG0000015129	0.394669095	####	###	CSNK1G3	protein_coding	casein kinas	5	123512099	123617045
ENSG0000010488	0.674778454	####	###	RNASEH2A	protein_coding	ribonuclease	19	12806584	12813640
ENSG0000017760	0.364452281	####	###	JUN	protein_coding	Jun proto-c	1	58780791	58784047
ENSG0000014757	-5.590063453	####	###	TRIM55	protein_coding	tripartite mc	8	66126896	66175485
ENSG0000016519	-0.754497855	####	###	PIGA	protein_coding	phosphatidy	X	15319452	15335554
ENSG0000018630	-0.844176801	####	###	ZNF555	protein_coding	zinc finger p	19	2841437	2860471
ENSG0000018440	0.42855057	####	###	SS18L1	protein_coding	SS18L1 sub	20	62143769	62182514
ENSG0000017602	-0.722705299	####	###	B3GALT6	protein_coding	beta-1,3-ga	1	1232237	1235041
ENSG0000020645	1.59366657	####	###	TCF19	protein_coding	transcriptior	CHR_HSCHR	31150981	31156655
ENSG0000010869	-6.676910256	####	###	CCL2	protein_coding	C-C motif c	17	34255218	34257203
ENSG0000010522	-0.582877349	####	###	PIAS4	protein_coding	protein inhil	19	4007736	4039386
ENSG0000010303	0.368600883	####	###	PSMD7	protein_coding	proteasome	16	74296814	74306288

ENSG0000005118	0.828484344	####	###	RAD51	protein_codinç	RAD51 reco	15	40694774	40732340
ENSG0000013614	-0.639375621	####	###	LRCH1	protein_codinç	leucine rich	13	46553168	46753040
ENSG0000015424	3.010161104	####	###	CEP112	protein_codinç	centrosoma	17	65635537	66192133
ENSG0000011923	0.453242167	####	###	SENP5	protein_codinç	SUMO spec	3	196867856	196934714
ENSG0000006518	0.394737158	####	###	WDR3	protein_codinç	WD repeat	1	117929720	117966543
ENSG0000003775	0.753191652	####	###	MRI1	protein_codinç	methylthiori	19	13764522	13774282
ENSG0000013487	0.384998707	####	###	DZIP1	protein_codinç	DAZ interac	13	95578202	95644706
ENSG0000018534	1.080991768	####	###	TEDC1	protein_codinç	tubulin epsi	14	105489855	105499575
ENSG0000019615	0.789324325	####	###	FAT4	protein_codinç	FAT atypica	4	125314955	125492932
ENSG0000017250	0.5653862	####	###	FIBP	protein_codinç	FGF1 intrace	11	65883740	65888531
ENSG0000013875	0.82254277	####	###	BMP2K	protein_codinç	BMP2 induc	4	78776342	78916372
ENSG0000009938	0.971459685	####	###	BCL7C	protein_codinç	BAF chroma	16	30833626	30894960
ENSG0000015115	-0.528007533	####	###	IPMK	protein_codinç	inositol poly	10	58191517	58267894
ENSG0000013243	0.515737544	####	###	FIGNL1	protein_codinç	fidgetin like	7	50444128	50542535
ENSG0000007426	0.621984885	####	###	EED	protein_codinç	embryonic e	11	86244753	86278813
ENSG0000027442	-0.42393753	####	###	DLG5	protein_codinç	discs large I	CHR_HSCHR	77790791	77926526
ENSG0000013342	1.919059635	####	###	LARGE1	protein_codinç	LARGE xylos	22	33162226	33922841
ENSG0000015663	-0.394148796	####	###	ZFAND3	protein_codinç	zinc finger A	6	37819727	38154624
ENSG0000011126	-0.396298483	####	###	DUSP16	protein_codinç	dual specific	12	12473282	12562863
ENSG0000013686	-1.129166225	####	###	ZFP37	protein_codinç	ZFP37 zinc f	9	113038377	113056759
ENSG0000010888	0.355007949	####	###	EFTUD2	protein_codinç	elongation f	17	44849948	44899445
ENSG0000016990	-0.650489699	####	###	TPST1	protein_codinç	tyrosylprote	7	66205317	66420543
ENSG0000018086	-0.840204475	####	###	PDIA3P1	transcribed_pr	protein disu	1	147172744	147179622
ENSG0000018219	0.568904628	####	###	EXT1	protein_codinç	exostosin gl	8	117794490	118111826
ENSG0000023467	1.538656827	####	###	TCF19	protein_codinç	transcriptior	CHR_HSCHR	31153422	31159096
ENSG0000026820	-1.155094279	####	###	AC005261.1	lncRNA	novel transc	19	57304305	57308562
ENSG0000014628	0.610582077	####	###	RARS2	protein_codinç	arginyl-tRN	6	87513938	87589987
ENSG0000016006	-0.766627071	####	###	ZBTB8A	protein_codinç	zinc finger a	1	32539427	32605941
ENSG0000021286	-2.481616465	####	###	RNF208	protein_codinç	ring finger p	9	137220247	137221581
ENSG0000016460	-1.249904764	####	###	GPR85	protein_codinç	G protein-c	7	113078331	113087778
ENSG0000018501	1.957520108	####	###	F8	protein_codinç	coagulation X		154835788	155026940
ENSG0000010123	0.429303141	####	###	RNF24	protein_codinç	ring finger p	20	3927309	4015558
ENSG0000016406	0.71510522	####	###	APEH	protein_codinç	acylaminoac	3	49674014	49683971
ENSG0000010896	-0.75329217	####	###	DPH1	protein_codinç	diphthamide	17	2030110	2043430
ENSG0000011552	0.208486393	####	###	SF3B1	protein_codinç	splicing fact	2	197388515	197435079
ENSG0000008082	-1.392077326	####	###	MOK	protein_codinç	MOK protei	14	102224500	102305190
ENSG0000017539	-0.298503559	####	###	EIF3F	protein_codinç	eukaryotic t	11	7970251	8001862
ENSG0000010588	-2.572126805	####	###	DLX5	protein_codinç	distal-less h	7	97020396	97024950
ENSG0000014950	0.443553236	####	###	INCENP	protein_codinç	inner centrc	11	62123998	62153169
ENSG0000010441	0.596341195	####	###	EMC2	protein_codinç	ER membra	8	108443601	108489196
ENSG0000014045	-0.494272918	####	###	USP3	protein_codinç	ubiquitin sp	15	63504511	63594640
ENSG0000022395	-8.994047057	####	###	C1QTNF5	protein_codinç	C1q and TN	11	119338942	119340940
ENSG0000027370	2.738523041	####	###	AC091271.1	lncRNA	novel transc	17	59618553	59619714
ENSG0000000305	0.515416225	####	###	M6PR	protein_codinç	mannose-6	12	8940361	8949761
ENSG0000013139	1.442023076	####	###	KCNC3	protein_codinç	potassium v	19	50311937	50333515
ENSG0000005516	-0.611946764	####	###	CYFIP2	protein_codinç	cytoplasmic	5	157266079	157395595
ENSG0000026246	2.272063334	####	###	LINC01569	lncRNA	long interge	16	4243943	4253817
ENSG0000011667	-0.775749479	####	###	LEPR	protein_codinç	leptin recep	1	65420652	65641559
ENSG0000015031	0.41955946	####	###	CWC15	protein_codinç	CWC15 splic	11	94962620	94973586
ENSG0000018080	-1.46983344	####	###	ARSJ	protein_codinç	arylsulfatase	4	113900284	113979727
ENSG0000011412	0.29342764	####	###	SLC25A36	protein_codinç	solute carrie	3	140941830	140980995
ENSG0000005182	0.393395877	####	###	MPHOSPH	protein_codinç	M-phase ph	12	123152320	123244014
ENSG0000016408	-0.907105634	####	###	DUSP7	protein_codinç	dual specific	3	52048919	52056571
ENSG0000024991	0.485411118	####	###	PDCD6	protein_codinç	programme	5	271621	314974
ENSG0000023628	0.396632592	####	###	ZBED5	protein_codinç	zinc finger E	11	10812074	10858796
ENSG0000012135	-0.758573192	####	###	PYROXD1	protein_codinç	pyridine nuc	12	21437615	21471250
ENSG0000015522	-0.463605494	####	###	MMS19	protein_codinç	MMS19 hor	10	97458324	97498794
ENSG0000006441	0.363049276	####	###	TNPO3	protein_codinç	transportin	7	128954180	129055173

ENSG0000015150	0.674519299	####	###	THYN1	protein_coding	thymocyte r	11	134248279	134253370
ENSG0000014590	0.25255714	####	###	G3BP1	protein_coding	G3BP stress	5	151771045	151812785
ENSG0000014262	-2.922325443	####	###	FHAD1	protein_coding	forkhead as	1	15247272	15400283
ENSG0000008132	0.645101089	####	###	STK17B	protein_coding	serine/threc	2	196133583	196176503
ENSG0000011968	0.343034992	####	###	DLST	protein_coding	dihydrolipoa	14	74881891	74903743
ENSG0000016640	-1.725031034	####	###	LMO1	protein_coding	LIM domain	11	8224309	8268716
ENSG0000012072	0.346790959	####	###	PAIP2	protein_coding	poly(A) binc	5	139341587	139369720
ENSG0000019858	-0.463017529	####	###	TLK1	protein_coding	tousled like	2	170990823	171231314
ENSG0000011088	0.667290286	####	###	CAPRIN2	protein_coding	caprin famil	12	30709552	30754951
ENSG0000024323	0.494822959	####	###	PCDHAC2	protein_coding	protocadhe	5	140966470	141012347
ENSG0000011268	0.538120936	####	###	EXOC2	protein_coding	exocyst com	6	485154	693139
ENSG0000017770	-0.907856429	####	###	FAM20C	protein_coding	FAM20C go	7	192571	260772
ENSG0000011445	0.535893217	####	###	GNB4	protein_coding	G protein su	3	179396088	179451476
ENSG0000026178	1.042625273	####	###	AC006058.1	lncRNA	novel transc	3	44117299	44122365
ENSG0000011596	-0.329133771	####	###	ATF2	protein_coding	activating tr	2	175072250	175168382
ENSG0000012848	-1.330906589	####	###	RNF112	protein_coding	ring finger p	17	19411125	19417276
ENSG0000014065	-0.689342647	####	###	PMM2	protein_coding	phosphoma	16	8788823	8849325
ENSG0000027740	0.81270292	####	###	TJP1	protein_coding	tight junctio	CHR_HSCHR	29850002	29972346
ENSG0000016483	-0.735540416	####	###	OXR1	protein_coding	oxidation re	8	106359476	106752694
ENSG0000019881	1.051871951	####	###	GK	protein_coding	glycerol kin	X	30653359	30731456
ENSG0000017274	1.155797572	####	###	ZNF596	protein_coding	zinc finger p	8	232137	264703
ENSG0000018298	0.783166628	####	###	ZNF320	protein_coding	zinc finger p	19	52860851	52897693
ENSG0000018768	3.392983531	####	###	KRT18P59	transcribed_pr	keratin 18 p	11	125113161	125126781
ENSG0000016454	-0.36573976	####	###	TRA2A	protein_coding	transformer	7	23504780	23532041
ENSG0000012565	-0.72316454	####	###	CLPP	protein_coding	caseinolytic	19	6361531	6370242
ENSG0000001328	1.052656976	####	###	MAN2B2	protein_coding	mannosidas	4	6575189	6623362
ENSG0000017240	-0.258836021	####	###	SYNPO2	protein_coding	synaptopod	4	118850688	119061247
ENSG0000017361	1.150504913	####	###	NMNAT1	protein_coding	nicotinamid	1	9943428	9985501
ENSG0000009573	0.978263876	####	###	BAMBI	protein_coding	BMP and ac	10	28677510	28682932
ENSG0000017048	-2.788708127	####	###	NPAS2	protein_coding	neuronal PA	2	100820139	100996829
ENSG0000027678	-0.266595915	####	###	CHGA	protein_coding	chromograr	CHR_HSCHR	92923080	92935293
ENSG0000010512	-0.54216681	####	###	AKAP8	protein_coding	A-kinase an	19	15353385	15379798
ENSG0000014760	0.584012251	####	###	TERF1	protein_coding	telomeric re	8	73008864	73048123
ENSG0000019626	0.91942967	####	###	ZNF493	protein_coding	zinc finger p	19	21397119	21427577
ENSG0000010534	-0.719728717	####	###	DMAC2	protein_coding	distal memb	19	41431318	41440717
ENSG0000016126	-1.066471823	####	###	BDH1	protein_coding	3-hydroxyb	3	197509783	197573323
ENSG0000018470	-0.669415728	####	###	SEPTIN5	protein_coding	septin 5 [So	22	19714503	19724224
ENSG0000010961	0.70558501	####	###	SEPSECS	protein_coding	Sep (O-pho	4	25120014	25160449
ENSG0000014154	0.42669615	####	###	EIF4A3	protein_coding	eukaryotic t	17	80134369	80147151
ENSG0000019793	-0.395920305	####	###	ERO1A	protein_coding	endoplasmi	14	52639915	52695900
ENSG0000012107	-0.446784474	####	###	SLC35B1	protein_coding	solute carrie	17	49700934	49709014
ENSG0000012314	-1.260830047	####	###	ADGRE5	protein_coding	adhesion G	19	14380501	14408725
ENSG0000023634	-2.062457491	####	###	NFKBIL1	protein_coding	NFKB inhibi	CHR_HSCHR	31537619	31549575
ENSG0000010565	-0.629192901	####	###	ISYNA1	protein_coding	inositol-3-p	19	18434388	18438167
ENSG0000019846	-1.572898428	####	###	TPM2	protein_coding	tropomyosi	9	35681992	35690056
ENSG0000016275	1.16337519	####	###	C1orf74	protein_coding	chromosom	1	209779208	209784559
ENSG0000010217	-0.713710881	####	###	UBL4A	protein_coding	ubiquitin lik	X	154483717	154486615
ENSG0000002025	-0.731630563	####	###	ZFP64	protein_coding	ZFP64 zinc f	20	52051663	52204308
ENSG0000014444	0.661988777	####	###	KANSL1L	protein_coding	KAT8 regula	2	210021421	210171409
ENSG0000002820	0.366674973	####	###	VEZT	protein_coding	vezatin, adh	12	95217746	95302799
ENSG0000018657	1.261201255	####	###	SMIM29	protein_coding	small integr	6	34246381	34249108
ENSG0000022415	2.321326516	####	###	AC009506.1	lncRNA	novel transc	2	159615296	159617082
ENSG0000009520	-1.81569946	####	###	EPB41L4B	protein_coding	erythrocyte	9	109171975	109320964
ENSG0000022375	0.721735622	####	###	TSSC2	transcribed_un	tumor supp	11	3380961	3408978
ENSG0000027960	3.233457744	####	###	AC109326.1	TEC	TEC	17	43360041	43361361
ENSG0000012240	-0.216503367	####	###	RPL5	protein_coding	ribosomal p	1	92832013	92841924
ENSG0000019808	0.828494553	####	###	SFI1	protein_coding	SFI1 centrin	22	31488688	31618588
ENSG0000019882	0.456812175	####	###	CHAMP1	protein_coding	chromosom	13	114314482	114337626

ENSG0000019647(	-0.573251998	####	###	SIAH1	protein_codinç	siah E3 ubic	16	48356364	48448402
ENSG0000007830	-0.323078562	####	###	PPP2R5C	protein_codinç	protein pho	14	101761798	101927989
ENSG0000000830	0.429388893	####	###	CELSR3	protein_codinç	cadherin EG	3	48636463	48662886
ENSG0000007112	0.376876605	####	###	WDR1	protein_codinç	WD repeat	4	10074339	10116949
ENSG0000010601	-0.728551657	####	###	IQCE	protein_codinç	IQ motif cor	7	2558972	2614733
ENSG0000007407	-0.470090635	####	###	MRPS34	protein_codinç	mitochondr	16	1771890	1773150
ENSG0000017010	0.619247466	####	###	ZNF778	protein_codinç	zinc finger p	16	89217703	89237071
ENSG0000013712	0.825453707	####	###	ALDH1B1	protein_codinç	aldehyde de	9	38392702	38398661
ENSG0000022190	0.817649385	####	###	FAM200A	protein_codinç	family with s	7	99546300	99558536
ENSG0000017466	-1.12018675	####	###	SLC29A2	protein_codinç	solute carrie	11	66362521	66372214
ENSG0000021145	-0.496947786	####	###	SACM1L	protein_codinç	SAC1 like pl	3	45689056	45745409
ENSG0000016893	0.625047448	####	###	TMEM129	protein_codinç	transmembı	4	1715952	1721358
ENSG0000027519	3.522066321	####	###	AL512791.2	lncRNA	novel transc	14	90383365	90387973
ENSG0000011043	-2.168541372	####	###	SLC1A2	protein_codinç	solute carrie	11	35251205	35420063
ENSG0000025422	1.503160316	####	###	PCDHGB1	protein_codinç	protocadhe	5	141350102	141512979
ENSG0000020520	0.474964198	####	###	C4orf46	protein_codinç	chromosom	4	158666675	158672255
ENSG0000011338	0.292670957	####	###	SUB1	protein_codinç	SUB1 regula	5	32531633	32604079
ENSG0000018799	-1.893390705	####	###	RINL	protein_codinç	Ras and Rat	19	38867830	38878275
ENSG0000009463	0.539059849	####	###	HDAC6	protein_codinç	histone dea X		48801377	48824982
ENSG0000027399	-0.722328747	####	###	PTPRK	protein_codinç	protein tyro CHR_HSCHR		127968779	128524810
ENSG0000018817	0.801791971	####	###	ZC3H6	protein_codinç	zinc finger C	2	112275597	112340063
ENSG0000017246	-1.152013565	####	###	TCEAL1	protein_codinç	transcriptior X		103628704	103630953
ENSG0000008108	0.606935281	####	###	OSTM1	protein_codinç	osteoclasto	6	108041409	108165854
ENSG0000016941	-1.821633507	####	###	NPR1	protein_codinç	natriuretic p	1	153678688	153693992
ENSG0000011750	0.402239194	####	###	DR1	protein_codinç	down-regul	1	93345907	93369493
ENSG0000024999	-1.986345087	####	###	TMEM158	protein_codinç	transmembı	3	45224466	45226287
ENSG0000014103	-0.8949673	####	###	GID4	protein_codinç	GID comple	17	18039408	18068405
ENSG0000016985	-1.351518778	####	###	PCDH7	protein_codinç	protocadhe	4	30720415	31146805
ENSG0000006878	0.569709326	####	###	SRBD1	protein_codinç	S1 RNA bin	2	45388680	45612165
ENSG0000025468	1.039332176	####	###	FPGT	protein_codinç	fucose-1-pl	1	74198212	74234086
ENSG0000011381	-0.739685908	####	###	SELENOK	protein_codinç	selenoprote	3	53884417	53891962
ENSG0000020462	1.064916748	####	###	DISP3	protein_codinç	dispatched	1	11479155	11537551
ENSG0000024394	-0.334639599	####	###	ZNF512	protein_codinç	zinc finger p	2	27582969	27623217
ENSG0000018831	0.581030324	####	###	CENPP	protein_codinç	centromere	9	92325953	92620529
ENSG0000014404	1.069890591	####	###	SFXN5	protein_codinç	sideroflexin	2	72942036	73075619
ENSG0000023070	-1.59410141	####	###	CSNK2B	protein_codinç	casein kinas CHR_HSCHR		31647414	31654461
ENSG0000022622	-0.294429739	####	###	RPS18	protein_codinç	ribosomal p CHR_HSCHR		33442379	33446883
ENSG0000023172	-1.128588395	####	###	LINC-PINT	lncRNA	long interge	7	130938963	131110176
ENSG0000027359	0.353303646	####	###	EEF1D	protein_codinç	eukaryotic t CHR_HSCHR		143579697	143597744
ENSG0000013374	-0.659993874	####	###	E2F5	protein_codinç	E2F transcri	8	85177154	85217158
ENSG0000014474	-1.480190393	####	###	LRIG1	protein_codinç	leucine rich	3	66378797	66501263
ENSG0000011270	-0.325946309	####	###	SENP6	protein_codinç	SUMO spec	6	75601509	75718281
ENSG0000011836	0.790294616	####	###	USP35	protein_codinç	ubiquitin sp	11	78188812	78215232
ENSG0000018486	-0.947487006	####	###	ARMCX2	protein_codinç	armadillo re X		101655281	101659850
ENSG0000025058	-1.732931069	####	###	NA	NA	NA	NA	NA	NA
ENSG0000017697	0.620424984	####	###	FAM89B	protein_codinç	family with s	11	65572349	65574198
ENSG0000027288	-0.591745194	####	###	DCP1A	protein_codinç	decapping r	3	53283429	53347586
ENSG0000021418	-1.904662082	####	###	XPOTP1	processed_pse	exportin for	20	34213495	34215892
ENSG0000010846	0.270727937	####	###	CBX1	protein_codinç	chromobox	17	48070052	48101478
ENSG0000012417	-1.392497255	####	###	PARD6B	protein_codinç	par-6 family	20	50731580	50756795
ENSG0000015100	2.028628	####	###	PRSS53	protein_codinç	serine prote	16	31083425	31089628
ENSG0000018646	-0.527384701	####	###	RPS23	protein_codinç	ribosomal p	5	82273320	82278396
ENSG0000013693	0.345282574	####	###	NCBP1	protein_codinç	nuclear cap	9	97633668	97673748
ENSG0000025936	-1.061871119	####	###	AC108449.2	lncRNA	novel transc	8	29055935	29056685
ENSG0000014818	-0.589454906	####	###	MRRF	protein_codinç	mitochondr	9	122264603	122331337
ENSG0000021851	0.926358405	####	###	LINC00339	lncRNA	long interge	1	22024558	22031223
ENSG0000011994	2.095008195	####	###	PYROXD2	protein_codinç	pyridine nuc	10	98383565	98415182
ENSG0000012586	-0.500096783	####	###	MKKS	protein_codinç	McKusick-K	20	10401009	10434222

ENSG0000010315	1.256260168	####	###	MLYCD	protein_coding	malonyl-Co	16	83899115	83951445
ENSG0000011218	-1.217965333	####	###	CAP2	protein_coding	cyclase asso	6	17393505	17557790
ENSG0000000548	0.484856641	####	###	RHBDD2	protein_coding	rhomboid d	7	75842602	75888926
ENSG0000016865	0.38217596	####	###	NDUFS5	protein_coding	NADH:ubiqu	1	39026318	39034636
ENSG0000014388	0.376228205	####	###	HNRNPLL	protein_coding	heterogene	2	38561969	38603586
ENSG0000010231	-0.380619524	####	###	MAGED2	protein_coding	MAGE famil	X	54807599	54816015
ENSG0000027591	0.885025907	####	###	NDE1	protein_coding	nudE neuro	CHR_HSCHR	15728421	15811507
ENSG0000012203	-0.969501558	####	###	MTIF3	protein_coding	mitochondr	13	27435643	27450591
ENSG0000026788	1.756444852	####	###	AC074135.1	lncRNA	novel transc	19	23075201	23100361
ENSG0000011756	-0.637122895	####	###	PTBP2	protein_coding	polypyrimid	1	96721665	96823738
ENSG0000018427	-1.463377457	####	###	POU6F1	protein_coding	POU class 6	12	51186936	51217708
ENSG0000016312	-0.38480984	####	###	RPRD2	protein_coding	regulation c	1	150363091	150476566
ENSG0000013039	0.345203892	####	###	AFDN	protein_coding	afadin, adhe	6	167826922	167972023
ENSG0000013022	0.303910175	####	###	XPO7	protein_coding	exportin 7 [	8	21919662	22006585
ENSG0000027334	1.258903651	####	###	PAXIP1-AS	lncRNA	PAXIP1 anti	7	155003448	155005703
ENSG0000011745	0.28476493	####	###	PRDX1	protein_coding	peroxiredox	1	45511036	45523047
ENSG0000011172	0.588312834	####	###	CMAS	protein_coding	cytidine mo	12	22046218	22065674
ENSG0000015933	0.255518311	####	###	PTMS	protein_coding	parathymos	12	6765516	6770952
ENSG0000018719	2.211398205	####	###	MT1X	protein_coding	metallothio	16	56682470	56684196
ENSG0000017235	0.355256902	####	###	GNB2	protein_coding	G protein su	7	100673567	100679174
ENSG0000018565	-1.057206908	####	###	ZFP36L1	protein_coding	ZFP36 ring f	14	68787660	68796253
ENSG0000020388	-0.444142602	####	###	PCMTD2	protein_coding	protein-L-is	20	64255695	64287821
ENSG0000011397	0.628507421	####	###	NPHP3	protein_coding	nephrocysti	3	132680609	132722414
ENSG0000025490	0.886669822	####	###	BORCS8	protein_coding	BLOC-1 rela	19	19176903	19192591
ENSG0000017194	0.476609591	####	###	ZNF217	protein_coding	zinc finger p	20	53567065	53609907
ENSG0000008407	-0.451636563	####	###	ZMPSTE24	protein_coding	zinc metallo	1	40258041	40294180
ENSG0000010632	-2.052685246	####	###	TFR2	protein_coding	transferrin r	7	100620416	100642779
ENSG0000028012	1.732879043	####	###	NA	NA	NA	NA	NA	NA
ENSG0000015585	-0.814191643	####	###	LSM11	protein_coding	LSM11, U7 s	5	157743712	157760709
ENSG0000013143	0.439607776	####	###	KIF3A	protein_coding	kinesin fami	5	132692628	132737638
ENSG0000027586	-0.991897393	####	###	KANSL1	protein_coding	KAT8 regula	CHR_HSCHR	45871009	46068204
ENSG0000017155	-0.631341074	####	###	BCL2L1	protein_coding	BCL2 like 1	20	31664452	31723989
ENSG0000017276	-0.595447669	####	###	NAA16	protein_coding	N-alpha-ac	13	41311267	41377030
ENSG0000027534	1.364157259	####	###	PRAG1	protein_coding	PEAK1 relat	8	8317736	8386439
ENSG0000014741	0.409842968	####	###	CCDC25	protein_coding	coiled-coil	8	27733316	27772653
ENSG0000027773	-8.790660303	####	###	TRAC	TR_C_gene	T cell recept	14	22547506	22552156
ENSG0000011512	0.561263922	####	###	TP53I3	protein_coding	tumor prote	2	24077433	24085861
ENSG0000019650	0.76213502	####	###	GDAP2	protein_coding	ganglioside	1	117863485	117929621
ENSG0000027911	-0.701976251	####	###	AP001972.5	TEC	TEC	11	75260129	75262466
ENSG0000015670	-0.554514031	####	###	AIFM1	protein_coding	apoptosis in	X	130124666	130165879
ENSG0000015869	-0.588307779	####	###	ZSCAN12	protein_coding	zinc finger a	6	28378955	28399734
ENSG0000014083	-0.407671307	####	###	ZFHX3	protein_coding	zinc finger h	16	72782885	73891871
ENSG0000004840	0.648331303	####	###	ZNF800	protein_coding	zinc finger p	7	127346790	127431924
ENSG0000015510	0.598967117	####	###	OTUD6B	protein_coding	OTU deubic	8	91070196	91087095
ENSG0000013770	-8.774755037	####	###	POU2F3	protein_coding	POU class 2	11	120236640	120319945
ENSG0000016751	-0.562979305	####	###	CDT1	protein_coding	chromatin li	16	88803789	88809258
ENSG0000015556	0.29141556	####	###	NUP205	protein_coding	nucleoporin	7	135557917	135648757
ENSG0000022613	1.444547408	####	###	BAIAP2-DT	lncRNA	BAIAP2 dive	17	81029130	81034881
ENSG0000024757	1.291075364	####	###	CKMT2-AS	lncRNA	CKMT2 anti	5	81201341	81301565
ENSG0000023051	1.643709953	####	###	THAP7-AS1	lncRNA	THAP7 antis	22	21001886	21010342
ENSG0000008462	-0.664945049	####	###	NKAIN1	protein_coding	sodium/pot	1	31179745	31239887
ENSG0000022879	0.736165275	####	###	LINC01128	lncRNA	long interge	1	825138	859446
ENSG0000010306	0.876818215	####	###	SLC7A6OS	protein_coding	solute carrie	16	68284503	68310946
ENSG0000014448	-3.424814599	####	###	TRPM8	protein_coding	transient re	2	233917373	234019522
ENSG0000007278	-0.66205782	####	###	STK10	protein_coding	serine/thre	5	172042079	172188224
ENSG0000017698	0.330993288	####	###	SEC24C	protein_coding	SEC24 hom	10	73744372	73772161
ENSG0000016336	-1.911562601	####	###	LINC01116	lncRNA	long interge	2	176629572	176637931
ENSG0000013110	0.37751412	####	###	ATP6V1E1	protein_coding	ATPase H+	22	17592136	17628749

ENSG0000016489	0.542970481	####	###	FASTK	protein_coding	Fas activate	7	151076593	151080866
ENSG0000018351	0.63312191	####	###	COA5	protein_coding	cytochrome	2	98599314	98608515
ENSG0000007341	-0.787868377	####	###	PDE8A	protein_coding	phosphodie	15	84980440	85139145
ENSG0000006665	-0.732503202	####	###	TRMT11	protein_coding	tRNA methy	6	125986479	126203817
ENSG0000023442	-1.842310724	####	###	LINC01250	lncRNA	long interge	2	2895048	3126026
ENSG0000007311	0.340470661	####	###	MCM2	protein_coding	minichromo	3	127598410	127622436
ENSG0000010933	-1.219336727	####	###	MAPK10	protein_coding	mitogen-ac	4	85990007	86594625
ENSG0000008803	1.032334853	####	###	ALG6	protein_coding	ALG6 alpha	1	63367575	63438553
ENSG0000017321	0.80092802	####	###	VANGL1	protein_coding	VANGL plan	1	115641970	115698224
ENSG0000025780	1.280806672	####	###	FNBP1P1	processed_pse	formin bind	2	74120680	74123218
ENSG0000013436	-0.441262166	####	###	NAV1	protein_coding	neuron navi	1	201622885	201826969
ENSG0000006430	-1.590949992	####	###	NGFR	protein_coding	nerve growt	17	49495293	49515008
ENSG0000006127	-0.495739791	####	###	HDAC7	protein_coding	histone dea	12	47782722	47833132
ENSG0000010141	0.483556624	####	###	E2F1	protein_coding	E2F transcri	20	33675477	33686385
ENSG0000027143	-0.905320186	####	###	NA	NA	NA	NA	NA	NA
ENSG0000012480	-0.545403662	####	###	EEF1E1	protein_coding	eukaryotic t	6	8073360	8102559
ENSG0000015819	0.327881849	####	###	WASF2	protein_coding	WASP family	1	27404230	27490167
ENSG0000007493	-0.455123205	####	###	TUBE1	protein_coding	tubulin epsi	6	112070663	112087529
ENSG0000008176	0.58583414	####	###	AACS	protein_coding	acetoacetyl-	12	125065434	125143333
ENSG0000024609	2.371652719	####	###	AP002026.1	lncRNA	novel transc	4	99088805	99301356
ENSG0000017838	-1.113713964	####	###	ZFAND2A	protein_coding	zinc finger A	7	1152071	1160759
ENSG0000016671	-0.489156672	####	###	ZNF592	protein_coding	zinc finger p	15	84748592	84806445
ENSG0000007761	0.547310068	####	###	NAALAD2	protein_coding	N-acetylate	11	90131515	90192894
ENSG0000026046	-3.549221481	####	###	INSYN1-AS	lncRNA	INSYN1 anti	15	73752317	73770613
ENSG0000027425	1.171912184	####	###	AC138649.1	lncRNA	novel transc	15	22757841	22778741
ENSG0000012545	-0.532986208	####	###	MSTO1	protein_coding	misato mito	1	155610205	155614951
ENSG0000017231	0.646145782	####	###	B3GALT1	protein_coding	beta-1,3-ga	2	167868948	167874041
ENSG0000014854	-1.038484193	####	###	FAM13C	protein_coding	family with s	10	59246130	59363181
ENSG0000022494	1.475225102	####	###	TCF19	protein_coding	transcription	CHR_HSCHR	31203494	31209123
ENSG0000016329	-0.327510586	####	###	ANTXR2	protein_coding	ANTXR cell	4	79901146	80125454
ENSG0000007677	-0.665813377	####	###	MBNL3	protein_coding	muscleblind	X	132369317	132489968
ENSG0000020526	0.484134022	####	###	PDE7A	protein_coding	phosphodie	8	65714334	65842322
ENSG0000005866	0.691163526	####	###	ATP2B4	protein_coding	ATPase plas	1	203626787	203744081
ENSG0000010468	0.388675481	####	###	GSR	protein_coding	glutathione-	8	30678066	30727846
ENSG0000014223	0.367766862	####	###	SAE1	protein_coding	SUMO1 acti	19	47113274	47210636
ENSG0000015921	0.463113052	####	###	SNF8	protein_coding	SNF8 subun	17	48929316	48944842
ENSG0000007221	0.636574935	####	###	ALDH3A2	protein_coding	aldehyde de	17	19648136	19685760
ENSG0000026972	0.675310426	####	###	NA	NA	NA	NA	NA	NA
ENSG0000010010	0.705793111	####	###	TRIOBP	protein_coding	TRIO and F-	22	37697048	37776556
ENSG0000017673	0.750816045	####	###	RBIS	protein_coding	ribosomal b	8	85214048	85220421
ENSG0000016368	-0.274458717	####	###	RPL9	protein_coding	ribosomal p	4	39452587	39458931
ENSG0000006583	-0.557219503	####	###	ME1	protein_coding	malic enzym	6	83210402	83431051
ENSG0000009994	-0.281282961	####	###	CRKL	protein_coding	CRK like pro	22	20917407	20953747
ENSG0000018364	1.887185526	####	###	ZNF530	protein_coding	zinc finger p	19	57599885	57612722
ENSG0000013605	0.359501161	####	###	WASHC4	protein_coding	WASH comp	12	105107324	105169130
ENSG0000007384	0.366762926	####	###	ST6GAL1	protein_coding	ST6 beta-ga	3	186930502	187078553
ENSG0000019648	-0.376908042	####	###	ESRRG	protein_coding	estrogen rel	1	216503246	217137755
ENSG0000007634	-2.489884684	####	###	RGS11	protein_coding	regulator of	16	268301	275980
ENSG0000011206	0.438871937	####	###	MAPK14	protein_coding	mitogen-ac	6	36027677	36111236
ENSG0000022357	-2.214411567	####	###	TINCR	protein_coding	TINCR ubiqu	19	5558167	5578349
ENSG0000015660	0.645546966	####	###	MED19	protein_coding	mediator co	11	57703714	57712323
ENSG0000011649	-0.528171264	####	###	S100PBP	protein_coding	S100P bindi	1	32816767	32858875
ENSG0000015309	0.398977321	####	###	BCL2L11	protein_coding	BCL2 like 11	2	111119378	111168445
ENSG0000013546	-1.140406345	####	###	COQ10A	protein_coding	coenzyme Q	12	56266858	56270966
ENSG0000015266	-2.546936996	####	###	GJA1	protein_coding	gap junction	6	121435595	121449727
ENSG0000021677	0.645247218	####	###	AL109918.1	transcribed_un	heterogene	6	52664366	52669155
ENSG0000006542	0.28369931	####	###	KARS1	protein_coding	lysyl-tRNA s	16	75627474	75648643
ENSG0000011842	-0.997965929	####	###	UBE3D	protein_coding	ubiquitin pr	6	82892390	83065841

ENSG0000016503	1.189755011	####	###	NFIL3	protein_coding	nuclear fact	9	91409045	91423832
ENSG0000018090	0.677637687	####	###	SCRIB	protein_coding	scribble plar	8	143790920	143815773
ENSG0000013954	-0.781347649	####	###	TARBP2	protein_coding	TARBP2 sub	12	53500921	53506431
ENSG0000020642	0.587190595	####	###	PRRC2A	protein_coding	proline rich	CHR_HSCHR	31610949	31627996
ENSG0000015227	0.447763523	####	###	PDE3B	protein_coding	phosphodie	11	14643804	14872044
ENSG0000011009	0.607898608	####	###	CPT1A	protein_coding	carnitine pa	11	68754620	68844410
ENSG0000026115	-5.198867017	####	###	AC109597.1	lncRNA	novel transc	16	12759282	12761162
ENSG0000010023	0.525308012	####	###	PPP6R2	protein_coding	protein pho	22	50343304	50445090
ENSG0000011290	-0.639954222	####	###	SEMA5A	protein_coding	semaphorin	5	9035033	9546075
ENSG0000015431	2.622895923	####	###	TDH	transcribed_un	L-threonine	8	11339637	11368452
ENSG0000010973	-0.703892464	####	###	MFSD10	protein_coding	major facilit	4	2930561	2934834
ENSG0000011706	-1.181759522	####	###	ST6GALNA	protein_coding	ST6 N-acety	1	76867480	77067546
ENSG0000015924	-2.651043069	####	###	GJD2	protein_coding	gap junctior	15	34751032	34754998
ENSG0000013750	0.472352866	####	###	CCDC90B	protein_coding	coiled-coil c	11	83259097	83286407
ENSG0000013077	2.243867149	####	###	THEMIS2	protein_coding	thymocyte s	1	27872543	27886685
ENSG0000018939	1.596859881	####	###	OR7E12P	unprocessed_p	olfactory rec	11	3390780	3391752
ENSG0000022503	-1.043099314	####	###	EIF4BP7	processed_pse	eukaryotic t	X	111619677	111621489
ENSG0000024724	1.625149722	####	###	UBL7-AS1	lncRNA	UBL7 antise	15	74461265	74513636
ENSG0000010751	-0.48180561	####	###	ATRNL1	protein_coding	attractin like	10	115093365	115948999
ENSG0000014568	-0.635531194	####	###	LHFPL2	protein_coding	LHFPL tetra:	5	78485215	78770021
ENSG0000011841	0.442072151	####	###	CASP8AP2	protein_coding	caspase 8 a:	6	89829894	89874436
ENSG0000016686	-0.554547014	####	###	ZBTB39	protein_coding	zinc finger a	12	56998836	57006546
ENSG0000021355	0.438976148	####	###	DNAJC9	protein_coding	DnaJ heat sl	10	73183362	73247255
ENSG0000010309	-0.551521078	####	###	WDR59	protein_coding	WD repeat c	16	74871362	75000173
ENSG0000024698	-1.669175153	####	###	SOCS2-AS1	lncRNA	SOCS2 antis	12	93542022	93571768
ENSG0000020481	-1.484118576	####	###	TTC25	protein_coding	tetratricope	17	41930617	41966503
ENSG0000025409	0.62309897	####	###	PINX1	protein_coding	PIN2 (TERF1	8	10764961	10839884
ENSG0000015754	-0.449305445	####	###	DYRK1A	protein_coding	dual specific	21	37365573	37526358
ENSG0000018400	0.305411862	####	###	PTP4A2	protein_coding	protein tyro	1	31906421	31944856
ENSG0000016044	-1.225660109	####	###	PKN3	protein_coding	protein kina	9	128702503	128720916
ENSG0000014868	0.563957596	####	###	RPP30	protein_coding	ribonuclease	10	90871952	90908553
ENSG0000011088	0.266158653	####	###	CORO1C	protein_coding	coronin 1C	12	108645109	108731526
ENSG0000010129	-0.334771061	####	###	HM13	protein_coding	histocompa	20	31514410	31577923
ENSG0000018589	-8.700967416	####	###	FFAR3	protein_coding	free fatty ac	19	35358460	35360489
ENSG0000012595	-0.412595103	####	###	MAX	protein_coding	MYC associa	14	65006174	65102695
ENSG0000014708	-8.666569327	####	###	AKAP4	protein_coding	A-kinase an	X	50190777	50201007
ENSG0000018498	0.761068692	####	###	NDUFA6	protein_coding	NADH:ubiqu	22	42085526	42090884
ENSG0000016829	4.604992293	####	###	H1-4	protein_coding	H1.4 linker l	6	26156329	26157115
ENSG0000007582	1.074587744	####	###	SEC31B	protein_coding	SEC31 hom	10	100486646	100519864
ENSG0000016307	-2.640317202	####	###	SPATA18	protein_coding	spermatoge	4	52051304	52097299
ENSG0000010046	-0.364090716	####	###	PRMT5	protein_coding	protein argi	14	22920525	22929408
ENSG0000015848	1.130410803	####	###	FAM86C1P	transcribed_un	family with s	11	71787510	71801237
ENSG0000011004	-0.391689531	####	###	OSBP	protein_coding	oxysterol bi	11	59574398	59615774
ENSG0000013944	-3.049377486	####	###	FOXN4	protein_coding	forkhead bc	12	109277978	109309284
ENSG0000014206	-1.018606754	####	###	ZFP14	protein_coding	ZFP14 zinc f	19	36334453	36379201
ENSG0000002322	-0.300211289	####	###	NDUFS1	protein_coding	NADH:ubiqu	2	206114817	206159509
ENSG0000013583	-0.331939301	####	###	CEP350	protein_coding	centrosoma	1	179954773	180114875
ENSG0000013106	0.641977938	####	###	GGT7	protein_coding	gamma-glu	20	34844720	34872856
ENSG0000026939	1.995071859	####	###	AC011503.1	lncRNA	novel transc	19	23927788	23929287
ENSG0000019654	-6.402835709	####	###	MME	protein_coding	membrane r	3	155024124	155183704
ENSG0000017014	-0.183085729	####	###	HNRNPA3	protein_coding	heterogene	2	177212563	177223958
ENSG0000027339	0.764037902	####	###	NDUFA6	protein_coding	NADH:ubiqu	CHR_HSCHR	42085525	42090955
ENSG0000019831	-0.365137044	####	###	ZKSCAN8	protein_coding	zinc finger v	6	28141883	28159460
ENSG0000016801	-1.698043572	####	###	TRANK1	protein_coding	tetratricope	3	36826819	36945057
ENSG0000019795	1.136517362	####	###	DNM3	protein_coding	dynamin 3 [	1	171841498	172418466
ENSG0000023347	-0.583867587	####	###	EEF1A1P6	processed_pse	eukaryotic t	7	22510616	22511999
ENSG0000017544	1.857196563	####	###	RFESD	protein_coding	Rieske Fe-S	5	95646754	95684773
ENSG0000012420	-4.594804443	####	###	EDN3	protein_coding	endothelin 3	20	59300443	59325992

ENSG0000010795	-0.558930082	####	###	MTPAP	protein_coding	mitochondr	10	30309801	30374448
ENSG0000010292	0.864067983	####	###	CBLN1	protein_coding	cerebellin 1	16	49277917	49281838
ENSG0000016068	-0.420047849	####	###	FLAD1	protein_coding	flavin adeni	1	154983338	154993111
ENSG0000021334	0.434323021	####	###	CHUK	protein_coding	component	10	100188300	100229596
ENSG0000013485	-0.423905292	####	###	TMEM165	protein_coding	transmembr	4	55395957	55453397
ENSG0000010057	-0.600751757	####	###	TIMM9	protein_coding	translocase	14	58408495	58427531
ENSG0000012879	0.77597333	####	###	TWSG1	protein_coding	twisted gast	18	9334767	9402420
ENSG0000014245	-0.458840319	####	###	CARM1	protein_coding	coactivator	19	10871553	10923075
ENSG0000014239	0.593287448	####	###	ERVK3-1	protein_coding	endogenous	19	58305319	58315663
ENSG0000010508	-1.409121098	####	###	OLFM2	protein_coding	olfactomedi	19	9853718	9936552
ENSG0000015776	-0.407471355	####	###	BRAF	protein_coding	B-Raf proto	7	140719327	140924929
ENSG0000017617	-2.379413922	####	###	SPHK1	protein_coding	sphingosine	17	76376584	76387860
ENSG0000010154	0.920642688	####	###	RBFA	protein_coding	ribosome bi	18	80034389	80050651
ENSG0000008225	0.37201119	####	###	CCNT2	protein_coding	cyclin T2 [So	2	134918235	134959342
ENSG0000024139	1.917899063	####	###	CD302	protein_coding	CD302 mole	2	159768628	159798255
ENSG0000015807	-0.587502408	####	###	PTPDC1	protein_coding	protein tyro	9	94030794	94109856
ENSG0000013548	0.811358878	####	###	ZC3H10	protein_coding	zinc finger C	12	56118260	56127514
ENSG0000015168	-1.058126382	####	###	INPP1	protein_coding	inositol poly	2	190343570	190371665
ENSG0000012935	0.874097558	####	###	SLC44A2	protein_coding	solute carrie	19	10602457	10644557
ENSG0000024077	-0.834834649	####	###	ARHGEF25	protein_coding	Rho guanin	12	57610180	57617245
ENSG0000007948	-2.328524634	####	###	OPHN1	protein_coding	oligophreni	X	68042344	68433913
ENSG0000008711	0.825748156	####	###	ADAMTS2	protein_coding	ADAM meta	5	179110853	179345461
ENSG0000017259	-1.60848783	####	###	SMPDL3A	protein_coding	sphingomye	6	122789049	122809720
ENSG0000009643	-1.887879495	####	###	ITPR3	protein_coding	inositol 1,4,5	6	33620365	33696574
ENSG0000024645	1.396201046	####	###	AL049840.2	lncRNA	novel transc	14	103682362	103684015
ENSG0000010184	0.960329645	####	###	STS	protein_coding	steroid sulfa	X	7147237	7804358
ENSG0000021344	-0.501307209	####	###	RPL18AP3	processed_pse	ribosomal p	12	104265309	104265836
ENSG0000017184	0.607341647	####	###	MLLT3	protein_coding	MLLT3 supe	9	20341669	20622499
ENSG0000006167	-0.253626319	####	###	NCKAP1	protein_coding	NCK associa	2	182909115	183038858
ENSG0000025772	-0.461431591	####	###	CNPY2	protein_coding	canopy FGF	12	56309842	56316119
ENSG0000017131	0.328584121	####	###	PGAM1	protein_coding	phosphogly	10	97426191	97433444
ENSG0000014331	0.682214245	####	###	PIGM	protein_coding	phosphatidy	1	160024953	160031990
ENSG0000009143	-0.363501313	####	###	MAP3K20	protein_coding	mitogen-ac	2	173075435	173268015
ENSG0000015432	0.818014331	####	###	NEIL2	protein_coding	nei like DNA	8	11769639	11787345
ENSG0000021271	0.790006561	####	###	LINC02693	lncRNA	long interge	17	21428263	21574517
ENSG0000013935	0.6425517	####	###	NEDD1	protein_coding	NEDD1 gam	12	96907224	96953780
ENSG0000017437	0.380152569	####	###	RALGAPA1	protein_coding	Ral GTPase	14	35538352	35809304
ENSG0000017988	-1.086558695	####	###	TIGD5	protein_coding	tigger trans	8	143597831	143603224
ENSG0000014056	-1.963883822	####	###	MCTP2	protein_coding	multiple C2	15	94231538	94483952
ENSG0000020484	-0.45926257	####	###	ATXN2	protein_coding	ataxin 2 [So	12	111443485	111599676
ENSG0000013807	-0.272813262	####	###	PREPL	protein_coding	prolyl endo	2	44316281	44361862
ENSG0000012600	0.882428819	####	###	MMP24OS	protein_coding	MMP24 opp	20	35201745	35278131
ENSG0000016971	0.445799663	####	###	DUS1L	protein_coding	dihydrourid	17	82057506	82065887
ENSG0000010038	-0.24106607	####	###	ST13	protein_coding	ST13 Hsp70	22	40824535	40856639
ENSG0000014464	-0.4912206	####	###	RBMS3	protein_coding	RNA binding	3	28574791	30010391
ENSG0000026175	-1.145450094	####	###	AC008555.	lncRNA	novel transc	19	34811589	34814345
ENSG0000018587	0.775302617	####	###	THNSL1	protein_coding	threonine sy	10	25016612	25026664
ENSG0000010944	0.423926105	####	###	ZNF330	protein_coding	zinc finger p	4	141220887	141234697
ENSG0000019796	-0.399776263	####	###	ZNF121	protein_coding	zinc finger p	19	9560353	9584533
ENSG0000016198	0.718456287	####	###	SNRNP25	protein_coding	small nuclea	16	53828	57669
ENSG0000027715	-2.055668938	####	###	TOMM40P1	processed_pse	TOMM40 ps	14	19267115	19268164
ENSG0000017266	0.597985841	####	###	ZMAT3	protein_coding	zinc finger m	3	178960121	179072215
ENSG0000018512	0.685209156	####	###	C6orf120	protein_coding	chromosom	6	169702190	169704856
ENSG0000018034	1.277371301	####	###	TIGD2	protein_coding	tigger trans	4	89111500	89114899
ENSG0000014349	0.6913965	####	###	VASH2	protein_coding	vasohibin 2	1	212950520	212992037
ENSG0000023565	-0.28772645	####	###	RPS18	protein_coding	ribosomal p	CHR_HSCHR	33193610	33198110
ENSG0000006783	1.187634653	####	###	ROGDI	protein_coding	rogdi atypic	16	4796968	4802880
ENSG0000017665	2.66677981	####	###	MYO1D	protein_coding	myosin ID [S	17	32492522	32877177

ENSG0000013834	0.49309116	####	###	DNA2	protein_coding	DNA replica	10	68414064	68472121
ENSG0000013817	-0.89762822	####	###	CALHM2	protein_coding	calcium hor	10	103446786	103452402
ENSG0000010662	-0.829560559	####	###	AEBP1	protein_coding	AE binding	7	44104345	44114562
ENSG0000027276	0.745133487	####	###	NDUFA6	protein_coding	NADH:ubiq	CHR_HSCHR	42085525	42090955
ENSG0000027736	0.75399336	####	###	NDUFA6	protein_coding	NADH:ubiq	CHR_HSCHR	42085525	42090955
ENSG0000014366	-0.55505008	####	###	LYST	protein_coding	lysosomal tr	1	235661041	235883640
ENSG0000016821	0.275283927	####	###	RBPJ	protein_coding	recombinati	4	26163455	26435131
ENSG0000013849	-1.882547642	####	###	PARP9	protein_coding	poly(ADP-ri	3	122527924	122564577
ENSG0000005860	-0.536837936	####	###	POLR3E	protein_coding	RNA polyme	16	22297375	22335101
ENSG0000008029	-0.410275084	####	###	RFX3	protein_coding	regulatory fa	9	3218297	3526004
ENSG0000017534	0.730706614	####	###	TMEM9B	protein_coding	TMEM9 dor	11	8947202	8965011
ENSG0000009077	0.880010042	####	###	EFNB1	protein_coding	ephrin B1 [S	X	68829021	68842160
ENSG0000009984	2.630961039	####	###	IZUMO4	protein_coding	IZUMO fam	19	2096429	2099593
ENSG0000022677	-3.752202577	####	###	FAM30A	lncRNA	family with s	14	105917979	105932642
ENSG0000011849	-0.354503738	####	###	FBXO30	protein_coding	F-box prote	6	145793502	145814795
ENSG0000010118	0.293030314	####	###	PSMA7	protein_coding	proteasome	20	62136733	62143440
ENSG0000011391	-1.272573611	####	###	BCL6	protein_coding	BCL6 transc	3	187721377	187745725
ENSG0000011997	0.593204796	####	###	TCTN3	protein_coding	tectonic fam	10	95663396	95694143
ENSG0000016341	-0.635307034	####	###	EIF4E3	protein_coding	eukaryotic t	3	71675414	71754773
ENSG0000020366	0.793906535	####	###	COX20	protein_coding	cytochrome	1	244835616	244845057
ENSG0000022740	-0.672432649	####	###	SLC39A7	protein_coding	solute carrie	CHR_HSCHR	33354325	33358319
ENSG0000013612	1.240889125	####	###	BORA	protein_coding	BORA auror	13	72727749	72756198
ENSG0000010624	0.309516581	####	###	PDAP1	protein_coding	PDGFA asso	7	99392048	99408597
ENSG0000018385	1.182591349	####	###	ZNF730	protein_coding	zinc finger p	19	23075210	23147221
ENSG0000010058	0.409312449	####	###	TMED8	protein_coding	transmembr	14	77335029	77377094
ENSG0000027329	-5.185699018	####	###	C1QTNF3-A	protein_coding	C1QTNF3-A	5	33987174	34124528
ENSG0000011155	0.851909555	####	###	MDM1	protein_coding	Mdm1 nucle	12	68272443	68332381
ENSG0000024985	-1.212086399	####	###	PVT1	lncRNA	Pvt1 oncoge	8	127794526	128187101
ENSG0000017148	-0.553294933	####	###	LRRC8C	protein_coding	leucine rich	1	89633072	89769903
ENSG0000007924	0.238844026	####	###	XRCC5	protein_coding	X-ray repair	2	216107464	216206303
ENSG0000012733	-0.32229349	####	###	DYRK2	protein_coding	dual specific	12	67648338	67665406
ENSG0000014039	-0.396806446	####	###	NCOA2	protein_coding	nuclear rece	8	70109782	70403808
ENSG0000018856	0.991790429	####	###	NDOR1	protein_coding	NADPH dep	9	137205685	137217009
ENSG0000013687	0.643934188	####	###	FPGS	protein_coding	folylpolyglu	9	127794597	127814327
ENSG0000017820	0.581586903	####	###	POGLUT3	protein_coding	protein O-g	11	108472112	108498384
ENSG0000012473	0.558235338	####	###	MEA1	protein_coding	male-enhar	6	43011143	43016868
ENSG0000013879	0.519063253	####	###	HADH	protein_coding	hydroxyacyl	4	107989714	108035174
ENSG0000008729	0.679151127	####	###	L2HGDH	protein_coding	L-2-hydroxy	14	50237563	50312229
ENSG0000013516	0.317998138	####	###	DMTF1	protein_coding	cyclin D bin	7	87152409	87196337
ENSG0000010777	-0.339180234	####	###	CCSER2	protein_coding	coiled-coil s	10	84328586	84518521
ENSG0000013750	0.27219189	####	###	CREBZF	protein_coding	CREB/ATF b	11	85659708	85682908
ENSG0000009300	0.621011379	####	###	CDC45	protein_coding	cell division	22	19479457	19520612
ENSG0000016956	-0.299673457	####	###	PCBP1	protein_coding	poly(rC) bin	2	70087477	70089203
ENSG0000010041	-0.43865402	####	###	POLR3H	protein_coding	RNA polyme	22	41525799	41544606
ENSG0000019833	0.892575054	####	###	HYLS1	protein_coding	HYLS1 centr	11	125883614	125900646
ENSG0000016120	-0.316843871	####	###	DVL3	protein_coding	dishevelled	3	184155377	184173614
ENSG0000014026	-0.393003095	####	###	SORD	protein_coding	sorbitol deh	15	45023147	45077185
ENSG0000012650	-1.467381472	####	###	FLRT1	protein_coding	fibronectin l	11	64103188	64119173
ENSG0000016091	0.597703368	####	###	CPSF4	protein_coding	cleavage an	7	99438922	99457373
ENSG0000017664	-0.691075207	####	###	RNF152	protein_coding	ring finger p	18	61808067	61894247
ENSG0000015451	0.331595843	####	###	ATP5MC3	protein_coding	ATP synthas	2	175176258	175184607
ENSG0000018795	0.51406144	####	###	CYHR1	protein_coding	cysteine and	8	144449582	144465677
ENSG0000022997	-3.145759129	####	###	AC007128.1	lncRNA	novel transc	7	8262264	8344516
ENSG0000007305	-0.661779592	####	###	XRCC1	protein_coding	X-ray repair	19	43543311	43580473
ENSG0000007050	0.596542671	####	###	POLB	protein_coding	DNA polym	8	42338454	42371808
ENSG0000008707	-3.085845137	####	###	HSD17B14	protein_coding	hydroxyster	19	48813018	48836510
ENSG0000000505	1.462601603	####	###	MCUB	protein_coding	mitochondr	4	109560209	109688719
ENSG0000013841	-0.819975402	####	###	HECW2	protein_coding	HECT, C2 ar	2	196189099	196593684

ENSG0000010796	0.871166584	####	###	STN1	protein_coding	STN1 subur	10	103877569	103918184
ENSG0000013242	0.251898602	####	###	PNISR	protein_coding	PNN interac	6	99398050	99425331
ENSG0000013381	-0.392150136	####	###	SBF2	protein_coding	SET binding	11	9778667	10294219
ENSG0000014144	-0.697647151	####	###	ESCO1	protein_coding	establishme	18	21529284	21600884
ENSG0000018260	-0.587198516	####	###	TRAK1	protein_coding	trafficking k	3	42013802	42225890
ENSG0000010201	-8.521469443	####	###	BMX	protein_coding	BMX non-re	X	15464246	15556529
ENSG0000011527	-0.749653109	####	###	INO80B	protein_coding	INO80 com	2	74455087	74457944
ENSG0000011124	0.537425918	####	###	RAD51AP1	protein_coding	RAD51 asso	12	4538798	4560048
ENSG0000007686	-1.633566461	####	###	RAP1GAP	protein_coding	RAP1 GTPas	1	21596215	21669363
ENSG0000017994	-1.001158634	####	###	FIZ1	protein_coding	FLT3 interac	19	55591376	55601970
ENSG0000019879	-0.391273115	####	###	SCAMP5	protein_coding	secretory ca	15	74957219	75021495
ENSG0000007108	-0.21227996	####	###	RPL31	protein_coding	ribosomal p	2	101002229	101024032
ENSG0000010081	-0.236927115	####	###	ACIN1	protein_coding	apoptotic cl	14	23058564	23095614
ENSG0000011675	0.236863777	####	###	SRSF11	protein_coding	serine and a	1	70205682	70253052
ENSG0000011568	0.486536247	####	###	PPP1R7	protein_coding	protein pho	2	241149576	241183652
ENSG0000007668	0.475259317	####	###	NT5C2	protein_coding	5'-nucleotic	10	103087185	103277605
ENSG0000012669	0.334540008	####	###	DNAJC8	protein_coding	DnaJ heat sl	1	28199456	28233029
ENSG0000014101	-0.930158803	####	###	GAS8	protein_coding	growth arre	16	90019629	90044975
ENSG0000018798	1.286645759	####	###	ZSCAN23	protein_coding	zinc finger a	6	28431930	28443502
ENSG0000000477	1.276397714	####	###	ARHGAP33	protein_coding	Rho GTPase	19	35774532	35788822
ENSG0000006360	-0.657078898	####	###	MTMR1	protein_coding	myotubulari	X	150692971	150765108
ENSG0000013529	-0.698292403	####	###	ADGRB3	protein_coding	adhesion G	6	68635282	69389506
ENSG0000022859	-1.881781574	####	###	FNDC10	protein_coding	fibronectin t	1	1598012	1600135
ENSG0000002831	0.478922312	####	###	BRD9	protein_coding	bromodoma	5	850291	892801
ENSG0000015842	1.341277678	####	###	TMSB15B	protein_coding	thymosin be	X	103918896	103966712
ENSG0000012591	0.462902522	####	###	NCLN	protein_coding	nicalin [Sou	19	3185563	3209575
ENSG0000013310	-2.377683122	####	###	CCNA1	protein_coding	cyclin A1 [S	13	36431520	36442870
ENSG0000018365	-0.444418057	####	###	MARCHF11	protein_coding	membrane a	5	16067139	16180762
ENSG0000026132	1.808160689	####	###	AC010168.2	lncRNA	novel transc	12	14762504	14767931
ENSG0000009615	-0.284989162	####	###	RPS18	protein_coding	ribosomal p	CHR_HSCHR	33200834	33205337
ENSG0000022334	2.481097343	####	###	H2BP1	transcribed_un	H2B histone	1	121108210	121117257
ENSG0000023150	-0.282919113	####	###	RPS18	protein_coding	ribosomal p	6	33272075	33276511
ENSG0000018048	0.678333266	####	###	MIGA1	protein_coding	mitoguardir	1	77779624	77879539
ENSG0000026411	-0.660394439	####	###	AC015813.1	lncRNA	novel transc	17	57989039	57994850
ENSG0000006706	0.369568993	####	###	IDI1	protein_coding	isopentenyl-	10	1039152	1049170
ENSG0000010038	0.459973365	####	###	RBX1	protein_coding	ring-box 1 [	22	40951347	40973309
ENSG0000011443	-0.357513802	####	###	BBX	protein_coding	BBX high m	3	107522936	107811339
ENSG0000016725	-0.562846908	####	###	RNF214	protein_coding	ring finger p	11	117232625	117286454
ENSG0000022598	-1.206698033	####	###	PBX2	protein_coding	PBX homeo	CHR_HSCHR	32251742	32257192
ENSG0000012288	-0.552600291	####	###	P4HA1	protein_coding	prolyl 4-hyd	10	73007217	73096974
ENSG0000013116	0.514140565	####	###	CHMP1A	protein_coding	charged mu	16	89640816	89657738
ENSG0000014947	0.608210803	####	###	TKFC	protein_coding	triokinase a	11	61333210	61353295
ENSG0000017761	0.709197459	####	###	PGBD5	protein_coding	piggyBac tra	1	230314490	230426332
ENSG0000015645	-0.49304926	####	###	PCDH1	protein_coding	protocadhe	5	141853111	141879246
ENSG0000013269	-0.403801955	####	###	ARHGEF11	protein_coding	Rho guanin	1	156934840	157045742
ENSG0000021478	1.438936755	####	###	POLR2J4	lncRNA	RNA polyme	7	43940895	44019175
ENSG0000011163	0.395314955	####	###	MRPL51	protein_coding	mitochondr	12	6491886	6493841
ENSG0000022754	2.131762849	####	###	DNAJC9-AS	lncRNA	DNAJC9 ant	10	73252791	73254349
ENSG0000017233	0.702654101	####	###	BPGM	protein_coding	bisphospho	7	134646811	134679816
ENSG0000018506	2.877384016	####	###	AC000068.1	lncRNA	novel transc	22	19447893	19450105
ENSG0000013200	0.561543899	####	###	ZSWIM4	protein_coding	zinc finger S	19	13795460	13832230
ENSG0000015392	-0.313564003	####	###	CHD1	protein_coding	chromodom	5	98853985	98929007
ENSG0000013752	0.661523272	####	###	RNF121	protein_coding	ring finger p	11	71929018	71997597
ENSG0000016974	-1.010383018	####	###	LDB2	protein_coding	LIM domain	4	16501541	16898678
ENSG0000001145	-0.42789902	####	###	WIZ	protein_coding	WIZ zinc fin	19	15419978	15449956
ENSG0000016101	0.634772055	####	###	MRNIP	protein_coding	MRN compl	5	179835133	179862173
ENSG0000021402	1.099634444	####	###	TTLL3	protein_coding	tubulin tyro	3	9808086	9855138
ENSG0000019802	1.421391335	####	###	ZNF560	protein_coding	zinc finger p	19	9466355	9498616

ENSG0000014465	-0.798332036	####	###	SLC25A38	protein_coding	solute carrie	3	39383370	39397351
ENSG0000017396	0.476749391	####	###	UBXN2A	protein_coding	UBX domair	2	23927285	24004909
ENSG0000015748	1.100014496	####	###	MYO1E	protein_coding	myosin IE [S	15	59132434	59372871
ENSG0000013380	-2.780649984	####	###	AMPD3	protein_coding	adenosine r	11	10308313	10507579
ENSG0000027500	-1.071717217	####	###	DDT	protein_coding	D-dopachro	CHR_HSCHR	23971367	23980473
ENSG0000013681	0.415957262	####	###	C9orf78	protein_coding	chromosom	9	129827290	129835863
ENSG0000011224	0.308130432	####	###	PTP4A1	protein_coding	protein tyro	6	63521746	63583436
ENSG0000019732	0.32355808	####	###	TRIM33	protein_coding	tripartite mc	1	114392790	114511203
ENSG0000011357	-3.369453094	####	###	FGF1	protein_coding	fibroblast gı	5	142592178	142698070
ENSG0000018544	-1.68524101	####	###	FAM174B	protein_coding	family with s	15	92617448	92809884
ENSG0000010653	0.433388075	####	###	TSPAN13	protein_coding	tetraspanin	7	16753755	16784536
ENSG0000012897	-0.498164185	####	###	CLN6	protein_coding	CLN6 transr	15	68206992	68257211
ENSG0000025053	-2.473047075	####	###	AC104407.1	lncRNA	novel transc	4	155206529	155209027
ENSG0000018835	0.390560042	####	###	FOCAD	protein_coding	focadhesin [	9	20658309	20995955
ENSG0000014471	-0.239893044	####	###	RPL32	protein_coding	ribosomal p	3	12834485	12841582
ENSG0000027164	2.716296155	####	###	AC099343.3	lncRNA	novel transc	4	184474802	184477304
ENSG0000023307	1.147777083	####	###	DDAH2	protein_coding	dimethylarg	CHR_HSCHR	31766492	31770071
ENSG0000010035	0.438430346	####	###	SGSM3	protein_coding	small G prot	22	40370591	40410289
ENSG0000017145	-0.554673124	####	###	POLR1C	protein_coding	RNA polyme	6	43509702	43562419
ENSG0000011601	-2.875992241	####	###	EPAS1	protein_coding	endothelial	2	46293667	46386697
ENSG0000018771	0.669776612	####	###	TMEM203	protein_coding	transmembı	9	137204082	137205648
ENSG0000016589	0.905318894	####	###	ISCA2	protein_coding	iron-sulfur c	14	74493756	74497106
ENSG0000027696	3.021817134	####	###	H4C5	protein_coding	H4 clusterec	6	26204552	26206038
ENSG0000016722	0.662590508	####	###	HDHD2	protein_coding	haloacid de	18	47107408	47150500
ENSG0000018855	0.556094526	####	###	RALGAPA2	protein_coding	Ral GTPase	20	20389530	20712644
ENSG0000010047	-1.206578637	####	###	AP4S1	protein_coding	adaptor rela	14	31025106	31130996
ENSG0000001031	2.231480902	####	###	PHF7	protein_coding	PHD finger	3	52410660	52423641
ENSG0000018921	-1.62027423	####	###	DPY19L2P1	transcribed_un	DPY19L2 ps	7	35079989	35186041
ENSG0000017442	-0.631372003	####	###	GTF2IRD2B	protein_coding	GTF2I repea	7	75092573	75149817
ENSG0000000448	0.26240829	####	###	KDM1A	protein_coding	lysine deme	1	23019443	23083689
ENSG0000027447	0.315609406	####	###	YWHAE	protein_coding	tyrosine 3-r	CHR_HSCHR	1247054	1303157
ENSG0000027006	-1.816150623	####	###	NA	NA	NA	NA	NA	NA
ENSG0000010354	0.441187898	####	###	SLC6A2	protein_coding	solute carrie	16	55655604	55706192
ENSG0000011441	0.270875163	####	###	FXR1	protein_coding	FMR1 autos	3	180868141	180982753
ENSG0000010669	0.439658437	####	###	FKTN	protein_coding	fukutin [Sou	9	105558122	105653820
ENSG0000015245	0.510537426	####	###	SUV39H2	protein_coding	suppressor c	10	14878820	14904315
ENSG0000011277	-1.340777978	####	###	TENT5A	protein_coding	terminal nuc	6	81491439	81752774
ENSG0000011133	-1.039774979	####	###	OAS3	protein_coding	2'-5'-oligoa	12	112938444	112973251
ENSG0000007709	0.291410552	####	###	TOP2B	protein_coding	DNA topois	3	25597905	25664907
ENSG0000015938	-1.113859179	####	###	BTG2	protein_coding	BTG anti-pr	1	203305491	203309602
ENSG0000016208	0.605380071	####	###	ZNF75A	protein_coding	zinc finger p	16	3305406	3318852
ENSG0000014085	-2.264832321	####	###	NLRC5	protein_coding	NLR family (	16	56989485	57083531
ENSG0000006719	-1.009345745	####	###	CACNB1	protein_coding	calcium volt	17	39173453	39197703
ENSG0000013919	1.39096059	####	###	VAMP1	protein_coding	vesicle asso	12	6462237	6470987
ENSG0000013147	0.232430984	####	###	ACLY	protein_coding	ATP citrate l	17	41866917	41930542
ENSG0000018408	0.815021071	####	###	FAM120C	protein_coding	family with s	X	54068324	54183281
ENSG0000022380	-1.370199966	####	###	CERS1	protein_coding	ceramide sy	19	18868545	18896727
ENSG0000017761	0.398947174	####	###	CSTF2T	protein_coding	cleavage sti	10	51695486	51699595
ENSG0000016543	-0.47359776	####	###	PGM2L1	protein_coding	phosphoglu	11	74330316	74398433
ENSG0000009928	-1.350182269	####	###	TSPAN15	protein_coding	tetraspanin	10	69451465	69507666
ENSG0000026509	-6.234902032	####	###	C1QTNF1-A	lncRNA	C1QTNF1 a	17	79018131	79027673
ENSG0000019634	1.041765682	####	###	ZKSCAN7	protein_coding	zinc finger v	3	44555193	44594173
ENSG0000012373	0.443913158	####	###	EXOSC9	protein_coding	exosome cc	4	121801318	121817021
ENSG0000010654	1.36245475	####	###	AHR	protein_coding	aryl hydroca	7	16916359	17346152
ENSG0000011181	-0.51216106	####	###	DSE	protein_coding	dermatan su	6	116254173	116444861
ENSG0000010978	-0.427044851	####	###	KLF3	protein_coding	Kruppel like	4	38664197	38701517
ENSG0000016375	-0.794038692	####	###	GYG1	protein_coding	glycogenin	3	148991408	149031775
ENSG0000027705	-3.695042524	####	###	FAM30A	lncRNA	family with s	CHR_HSCHR	105918750	105933413

ENSG0000016497	0.307035957	####	###	SNAPC3	protein_coding	small nuclea	9	15422704	15465953
ENSG0000010541	0.807753453	####	###	MEIS3	protein_coding	Meis homec	19	47403124	47419527
ENSG0000014795	0.386334158	####	###	SIGMAR1	protein_coding	sigma non-	9	34634722	34637809
ENSG0000011576	-0.423604088	####	###	NOL10	protein_coding	nucleolar pr	2	10570754	10689987
ENSG0000017423	0.592798775	####	###	PITPNA	protein_coding	phosphatidy	17	1517718	1562792
ENSG0000017505	0.427911933	####	###	ATR	protein_coding	ATR serine/t	3	142449007	142578733
ENSG0000010010	0.719940883	####	###	SRRD	protein_coding	SRR1 doma	22	26483877	26494658
ENSG0000026282	-0.481613483	####	###	INTS3	protein_coding	integrator c	CHR_HSCHR	153745298	153792039
ENSG0000014568	0.424612374	####	###	SSBP2	protein_coding	single stranc	5	81413021	81751797
ENSG0000017620	0.644445184	####	###	SMIM19	protein_coding	small integr	8	42541155	42555195
ENSG0000013429	0.511474878	####	###	TMEM106C	protein_coding	transmembi	12	47963569	47968878
ENSG0000014102	-0.323856405	####	###	NCOR1	protein_coding	nuclear rece	17	16029157	16218185
ENSG0000006603	-0.390248876	####	###	CTNNA2	protein_coding	catenin alph	2	79185231	80648861
ENSG0000018230	0.385335367	####	###	C8orf33	protein_coding	chromosom	8	145052465	145066685
ENSG0000017146	-0.426228577	####	###	ZNF561	protein_coding	zinc finger p	19	9604680	9621236
ENSG0000016403	-0.911556616	####	###	BDH2	protein_coding	3-hydroxyb	4	103077592	103099870
ENSG0000026913	0.656660116	####	###	NA	NA	NA	NA	NA	NA
ENSG0000027639	-8.424102409	####	###	FLJ36000	lncRNA	uncharacter	17	22406019	22413744
ENSG0000013913	-0.754434401	####	###	YARS2	protein_coding	tyrosyl-tRN	12	32727490	32755897
ENSG0000013508	-0.947001405	####	###	CCNJL	protein_coding	cyclin J like	5	160249083	160345396
ENSG0000025508	2.297014548	####	###	AF186192.2	transcribed_un	zinc finger p	8	144700353	144708517
ENSG0000013595	0.479345671	####	###	TMEM127	protein_coding	transmembi	2	96248514	96265997
ENSG0000011968	-1.834488341	####	###	LTBP2	protein_coding	latent transf	14	74498183	74612378
ENSG0000009209	0.754643021	####	###	RNF31	protein_coding	ring finger p	14	24146683	24160660
ENSG0000011619	-0.394413492	####	###	FAM20B	protein_coding	FAM20B gly	1	179025804	179076567
ENSG0000022336	-0.279667291	####	###	RPS18	protein_coding	ribosomal p	CHR_HSCHR	33249931	33254431
ENSG0000007541	-0.201419039	####	###	SLC25A3	protein_coding	solute carrie	12	98593591	98606379
ENSG0000010374	-1.936773677	####	###	IGDCC4	protein_coding	immunoglol	15	65381484	65422947
ENSG0000025098	-4.046497782	####	###	LINC02600	lncRNA	long interge	4	3758748	3763390
ENSG0000016656	-0.84121274	####	###	SEC11C	protein_coding	SEC11 hom	18	59139866	59158832
ENSG0000010336	0.493301246	####	###	ELOB	protein_coding	elongin B [S	16	2771414	2777280
ENSG0000018869	0.505128895	####	###	UROS	protein_coding	uroporphyri	10	125784980	125823258
ENSG0000011889	0.97475326	####	###	EEF2KMT	protein_coding	eukaryotic e	16	5084284	5097795
ENSG0000002390	-0.472151439	####	###	GCLM	protein_coding	glutamate-c	1	93885199	93909456
ENSG0000012996	0.471277889	####	###	ABHD17A	protein_coding	abhydrolase	19	1876810	1885496
ENSG0000027707	2.911202337	####	###	H2AC8	protein_coding	H2A cluster	6	26216975	26217483
ENSG0000025354	2.258249411	####	###	FAM86HP	transcribed_un	family with s	3	130099092	130111472
ENSG0000019650	0.239550703	####	###	PRPF40A	protein_coding	pre-mRNA	2	152651593	152717997
ENSG0000013429	1.232575692	####	###	PLEKHA8P1	transcribed_pr	pleckstrin hc	12	45173064	45216041
ENSG0000014841	-1.569004175	####	###	NACC2	protein_coding	NACC family	9	136006537	136095289
ENSG0000004757	0.632483432	####	###	KIAA0556	protein_coding	KIAA0556 [S	16	27550133	27780369
ENSG0000012574	0.393659689	####	###	SNRPD2	protein_coding	small nuclea	19	45687454	45692569
ENSG0000013860	-0.495660511	####	###	SHF	protein_coding	Src homolog	15	45167214	45201175
ENSG0000017293	-8.394451305	####	###	MRGPRF	protein_coding	MAS relatec	11	69004395	69013382
ENSG0000011870	0.270839573	####	###	RPN2	protein_coding	ribophorin I	20	37178410	37241619
ENSG0000012478	-0.261821102	####	###	SSR1	protein_coding	signal seque	6	7268306	7347446
ENSG0000020648	-0.838034319	####	###	FLOT1	protein_coding	flotillin 1 [Sc	CHR_HSCHR	30717229	30732250
ENSG0000027293	-1.114804555	####	###	AL391121.1	lncRNA	novel transc	10	102642792	102644140
ENSG0000014037	0.466997226	####	###	ETFA	protein_coding	electron trai	15	76215353	76311472
ENSG0000019887	0.621990998	####	###	GRK5	protein_coding	G protein-c	10	119207571	119459745
ENSG0000016405	-0.566277946	####	###	SHISA5	protein_coding	shisa family	3	48467798	48504826
ENSG0000011401	-2.271311621	####	###	AMOTL2	protein_coding	angiomotin	3	134355874	134375479
ENSG0000007286	0.758192729	####	###	NDE1	protein_coding	nudE neuro	16	15643267	15734691
ENSG0000012249	1.025958621	####	###	SLC66A2	protein_coding	solute carrie	18	79902420	79951657
ENSG0000016265	-1.159168664	####	###	ATXN7L2	protein_coding	ataxin 7 like	1	109483479	109492804
ENSG0000027192	-2.534582308	####	###	NA	NA	NA	NA	NA	NA
ENSG0000017866	-0.940701802	####	###	ZNF713	protein_coding	zinc finger p	7	55887456	55942530
ENSG0000000671	-0.47004138	####	###	VPS41	protein_coding	VPS41 subu	7	38722974	38932394

ENSG0000017742	-0.888354756	####	###	TGIF1	protein_coding	TGFB induce	18	3411608	3459978
ENSG0000017304	0.797271576	####	###	ZNF680	protein_coding	zinc finger p	7	64519878	64563075
ENSG0000013398	0.560246139	####	###	COX16	protein_coding	cytochrome	14	70325081	70416984
ENSG0000012148	-0.622117496	####	###	RNF2	protein_coding	ring finger p	1	185045526	185102603
ENSG0000023257	-0.652062898	####	###	RPL3P4	processed_pse	ribosomal p	14	98972879	98973301
ENSG0000013859	-0.33586789	####	###	USP8	protein_coding	ubiquitin sp	15	50424380	50514421
ENSG0000018748	-5.094751042	####	###	KCNJ11	protein_coding	potassium in	11	17385859	17389331
ENSG0000008172	-0.544190318	####	###	DUSP12	protein_coding	dual specific	1	161749758	161757238
ENSG0000014882	0.503033455	####	###	MTG1	protein_coding	mitochondr	10	133394094	133422520
ENSG0000018385	-0.796884823	####	###	KIRREL1	protein_coding	kirre like ne	1	157993273	158100262
ENSG0000011211	0.479726818	####	###	MRPL18	protein_coding	mitochondr	6	159789812	159798436
ENSG0000006598	-2.229388667	####	###	PDE4A	protein_coding	phosphodie	19	10416773	10469630
ENSG0000017860	-0.55045513	####	###	ERN1	protein_coding	endoplasmi	17	64039142	64130819
ENSG0000001301	-0.809610371	####	###	EHD3	protein_coding	EH domain	2	31234152	31269451
ENSG0000024085	-0.712800697	####	###	RDH14	protein_coding	retinol dehy	2	18554723	18560679
ENSG0000013539	0.427730021	####	###	DNAJC14	protein_coding	DnaJ heat sl	12	55820960	55830824
ENSG0000017410	0.965449578	####	###	C16orf91	protein_coding	chromosom	16	1419752	1420756
ENSG0000023469	2.489446039	####	###	GPR50-AS1	lncRNA	GPR50 antis	X	151175192	151177836
ENSG0000013731	1.505054849	####	###	TCF19	protein_coding	transcription	6	31158547	31167159
ENSG0000016968	0.633351281	####	###	CHRNA5	protein_coding	cholinergic r	15	78565520	78595269
ENSG0000010071	0.786573388	####	###	ZFYVE21	protein_coding	zinc finger F	14	103715730	103733668
ENSG0000015686	-0.439636535	####	###	FBRS	protein_coding	fibrosin [Sou	16	30658431	30670810
ENSG0000007188	0.891793407	####	###	FAM3A	protein_coding	FAM3 meta	X	154506159	154516242
ENSG0000023386	-1.095714855	####	###	NA	NA	NA	NA	NA	NA
ENSG0000024587	-1.413652316	####	###	LINC00682	lncRNA	long interge	4	41872747	41882955
ENSG0000016168	-1.40454726	####	###	SHANK1	protein_coding	SH3 and mu	19	50661827	50719450
ENSG0000017152	-2.929576214	####	###	PTGER4	protein_coding	prostagland	5	40679915	40693735
ENSG0000025724	-7.550840464	####	###	LINC01619	lncRNA	long interge	12	91984976	92142914
ENSG0000015639	1.021720137	####	###	SFXN2	protein_coding	sideroflexin	10	102714538	102743492
ENSG0000018087	-3.393908361	####	###	GREM2	protein_coding	gremlin 2, D	1	240489573	240612155
ENSG0000027012	-1.597589785	####	###	AC027020.2	lncRNA	novel transc	15	100547765	100550153
ENSG0000012805	-0.241871346	####	###	PAICS	protein_coding	phosphoribo	4	56435741	56464578
ENSG0000023991	-2.131393735	####	###	PRKAG2-AS	lncRNA	PRKAG2 ant	7	151877042	151879223
ENSG0000010863	-1.080308607	####	###	SYNGR2	protein_coding	synaptogyri	17	78168558	78173527
ENSG0000019641	1.171804757	####	###	ZNF765	protein_coding	zinc finger p	19	53389793	53430413
ENSG0000016995	-0.459428363	####	###	ZNF768	protein_coding	zinc finger p	16	30524004	30526821
ENSG0000027590	0.530953201	####	###	PSMB3	protein_coding	proteasome	CHR_HSCHR	38553523	38565018
ENSG0000016362	-0.34110106	####	###	WDFY3	protein_coding	WD repeat a	4	84669597	84966690
ENSG0000010062	0.67330708	####	###	CEP128	protein_coding	centrosoma	14	80476983	80959517
ENSG0000011553	-0.735417216	####	###	PDCL3	protein_coding	phosducin li	2	100562993	100576739
ENSG0000013698	0.719755611	####	###	DSCC1	protein_coding	DNA replica	8	119833976	119855894
ENSG0000012745	0.858300994	####	###	FBXL12	protein_coding	F-box and l	19	9810267	9827816
ENSG0000017217	0.528250348	####	###	MRPL13	protein_coding	mitochondr	8	120380761	120445402
ENSG0000016985	-1.118124449	####	###	AVEN	protein_coding	apoptosis ar	15	33858782	34075155
ENSG0000013022	-0.627147637	####	###	DPP6	protein_coding	dipeptidyl p	7	153887097	154894285
ENSG0000010733	0.766728681	####	###	SHB	protein_coding	SH2 domain	9	37915898	38069227
ENSG0000015685	-0.825418465	####	###	ZNF689	protein_coding	zinc finger p	16	30602558	30624012
ENSG0000018023	0.858172233	####	###	ZNRF2	protein_coding	zinc and rin	7	30284597	30367689
ENSG0000018586	-0.94946113	####	###	ZNF829	protein_coding	zinc finger p	19	36888124	36916291
ENSG0000015136	-5.089956582	####	###	ALLC	protein_coding	allantoicase	2	3658200	3702671
ENSG0000010795	-2.383580839	####	###	NEURL1	protein_coding	neuralized E	10	103493979	103592552
ENSG0000028038	-0.839994847	####	###	AP000648.4	TEC	novel transc	11	90193614	90198120
ENSG0000011120	0.284031169	####	###	FOXM1	protein_coding	forkhead bo	12	2857680	2877174
ENSG0000019689	1.529205302	####	###	H2BU1	protein_coding	H2B.U histo	1	228458107	228460470
ENSG0000014349	-0.480034148	####	###	SMYD2	protein_coding	SET and MY	1	214281102	214337131
ENSG0000004802	0.463789361	####	###	USP28	protein_coding	ubiquitin sp	11	113797874	113875570
ENSG0000013875	0.275690176	####	###	G3BP2	protein_coding	G3BP stress	4	75642782	75724525
ENSG0000002852	0.315363891	####	###	SNX1	protein_coding	sorting nexi	15	64094123	64146090

ENSG0000015894	0.317338677	####	###	CCAR2	protein_coding	cell cycle an	8	22604632	22621514
ENSG0000017654	-0.705638331	####	###	USF3	protein_coding	upstream tr	3	113648385	113696646
ENSG0000004445	-0.549321169	####	###	CNTLN	protein_coding	centlein [So	9	17134982	17503923
ENSG0000019894	-1.063379455	####	###	L3MBTL3	protein_coding	L3MBTL hist	6	130013699	130141449
ENSG0000016254	-2.600149696	####	###	TMCO4	protein_coding	transmembr	1	19682213	19799945
ENSG0000022461	4.046358018	####	###	RTCA-AS1	lncRNA	RTCA antise	1	100251528	100266179
ENSG0000013063	0.31360195	####	###	ATXN10	protein_coding	ataxin 10 [S	22	45671798	45845307
ENSG0000010022	-0.321208475	####	###	POLDIP3	protein_coding	DNA polym	22	42583721	42614962
ENSG0000017381	-0.230239226	####	###	EIF1	protein_coding	eukaryotic t	17	41688885	41692668
ENSG0000011081	-0.740118747	####	###	P3H3	protein_coding	prolyl 3-hyc	12	6828407	6839847
ENSG0000015310	-0.32158352	####	###	ANAPC1	protein_coding	anaphase pr	2	111611639	111884690
ENSG0000027891	2.779233678	####	###	CEP83-DT	lncRNA	CEP83 diver	12	94460003	94462484
ENSG0000015724	0.867707148	####	###	FZD1	protein_coding	frizzled class	7	91264433	91271326
ENSG0000012799	-0.471104925	####	###	SGCE	protein_coding	sarcoglycan	7	94524204	94656572
ENSG0000016618	1.033022916	####	###	ZNF319	protein_coding	zinc finger p	16	57994668	58000453
ENSG0000002972	0.391915634	####	###	RABEP1	protein_coding	rabaptin, RA	17	5282265	5386340
ENSG0000010906	0.599988292	####	###	TMEM104	protein_coding	transmembr	17	74776499	74839753
ENSG0000011977	-0.416750643	####	###	TMEM214	protein_coding	transmembr	2	27032910	27041694
ENSG0000016489	-0.849154035	####	###	TMUB1	protein_coding	transmembr	7	151081085	151083493
ENSG0000027951	-2.186088721	####	###	AC083843.3	TEC	TEC	8	134783694	134785879
ENSG0000013310	-0.66716336	####	###	COG6	protein_coding	component	13	39655627	39791665
ENSG0000007561	0.275935346	####	###	FSCN1	protein_coding	fascin actin-	7	5592816	5606655
ENSG0000011931	-0.225014597	####	###	RAD23B	protein_coding	RAD23 hom	9	107283137	107332192
ENSG0000017622	0.595897408	####	###	RTTN	protein_coding	rotatin [Sou	18	70003031	70205726
ENSG0000021379	1.132122138	####	###	ZNF845	protein_coding	zinc finger p	19	53333749	53356906
ENSG0000020437	0.398162993	####	###	SDHD	protein_coding	succinate de	11	112086824	112120016
ENSG0000025989	1.531441264	####	###	AC107375.1	lncRNA	novel transc	8	140505813	140508043
ENSG0000013295	-0.813223476	####	###	ZMYM5	protein_coding	zinc finger M	13	19823482	19863649
ENSG0000011042	-1.088966432	####	###	KIAA1549L	protein_coding	KIAA1549 lil	11	33376083	33674102
ENSG0000012429	0.458985462	####	###	PEPD	protein_coding	peptidase D	19	33386950	33521791
ENSG0000012639	0.706338201	####	###	FRMD8	protein_coding	FERM doma	11	65386621	65413525
ENSG0000010057	0.440635796	####	###	KIAA0586	protein_coding	KIAA0586 [S	14	58427385	58551297
ENSG0000003931	0.313112263	####	###	ZFYVE16	protein_coding	zinc finger F	5	80408013	80483379
ENSG0000019693	-0.456515006	####	###	SRGAP1	protein_coding	SLIT-ROBO	12	63844700	64162217
ENSG0000010490	-2.803477043	####	###	LYL1	protein_coding	LYL1 basic h	19	13099033	13103161
ENSG0000023549	-2.263818024	####	###	AC073046.1	lncRNA	novel transc	2	73985132	73986343
ENSG0000027425	2.742985956	####	###	NA	NA	NA	NA	NA	NA
ENSG0000027604	0.454245108	####	###	UHRF1	protein_coding	ubiquitin like	19	4903080	4962154
ENSG0000016733	1.232444469	####	###	TRIM68	protein_coding	tripartite mo	11	4598672	4608231
ENSG0000014423	0.473052455	####	###	POLR2D	protein_coding	RNA polyme	2	127843553	127858155
ENSG0000010878	-0.38806015	####	###	MLX	protein_coding	MAX dimeri	17	42567068	42573239
ENSG0000013644	3.070216685	####	###	MYCBPAP	protein_coding	MYCBP asso	17	50508384	50531501
ENSG0000016198	0.784586684	####	###	POLR3K	protein_coding	RNA polyme	16	46407	53608
ENSG0000012476	-0.247359163	####	###	SOX4	protein_coding	SRY-box tra	6	21593751	21598619
ENSG0000016482	0.438969524	####	###	OSGIN2	protein_coding	oxidative str	8	89901849	89927888
ENSG0000017419	-0.316742622	####	###	MGA	protein_coding	MAX dimeri	15	41621224	41773081
ENSG0000017662	0.532190905	####	###	RMDN1	protein_coding	regulator of	8	86468257	86514357
ENSG0000004854	0.408084835	####	###	MRPS10	protein_coding	mitochondr	6	42206807	42217861
ENSG0000012573	-0.961140084	####	###	TRIP10	protein_coding	thyroid horr	19	6737925	6751530
ENSG0000011266	-1.0840087	####	###	DNPH1	protein_coding	2'-deoxynu	6	43225629	43229481
ENSG0000009002	0.726002193	####	###	SLC9A1	protein_coding	solute carrie	1	27098809	27166981
ENSG0000019835	-0.63551192	####	###	HOXC4	protein_coding	homeobox C	12	54016931	54056030
ENSG0000018893	-1.913691153	####	###	USP32P1	transcribed_un	ubiquitin sp	17	16786489	16804455
ENSG0000004864	-0.249376053	####	###	RSF1	protein_coding	remodeling	11	77659996	77821017
ENSG0000018145	0.699003624	####	###	ZNF678	protein_coding	zinc finger p	1	227563543	227677443
ENSG0000015722	-0.455367022	####	###	CLDN12	protein_coding	claudin 12 [	7	90383721	90513402
ENSG0000016700	0.225590555	####	###	NUDT21	protein_coding	nudix hydro	16	56429133	56452199
ENSG0000015146	0.505217741	####	###	SCLT1	protein_coding	sodium chai	4	128864921	129093600

ENSG0000013865	-1.708224512	####	###	PCDH10	protein_coding	protocadhe	4	133149294	133208606
ENSG0000003586	0.294432201	####	###	TIMP2	protein_coding	TIMP metall	17	78852977	78925387
ENSG0000001491	0.401664849	####	###	COX15	protein_coding	cytochrome	10	99710868	99732127
ENSG0000010276	-0.548580503	####	###	VWA8	protein_coding	von Willebra	13	41566835	41961120
ENSG0000018353	0.322122161	####	###	PRR14L	protein_coding	proline rich	22	31676256	31750140
ENSG0000016374	-0.653660509	####	###	RCHY1	protein_coding	ring finger a	4	75479037	75514764
ENSG0000015914	-0.209689488	####	###	SON	protein_coding	SON DNA a	21	33543038	33577481
ENSG0000014574	-0.758851531	####	###	FBXL17	protein_coding	F-box and l	5	107859035	108382098
ENSG0000016020	-0.494148447	####	###	U2AF1	protein_coding	U2 small nu	21	43092956	43107570
ENSG0000013499	1.007795761	####	###	OSTF1	protein_coding	osteoclast s	9	75088514	75147265
ENSG0000015356	-0.267600678	####	###	RMND5A	protein_coding	required for	2	86720291	86778041
ENSG0000027533	0.776439581	####	###	RAB11FIP3	protein_coding	RAB11 fami	CHR_HSCHR	425619	526533
ENSG0000017710	-2.702773407	####	###	DSCAML1	protein_coding	DS cell adhe	11	117427772	117817525
ENSG0000008384	-0.235606052	####	###	RPS5	protein_coding	ribosomal p	19	58386400	58394806
ENSG0000006397	0.401293245	####	###	RNF4	protein_coding	ring finger p	4	2462220	2625320
ENSG0000016897	1.521689857	####	###	JMJD7-PLA	protein_coding	JMJD7-PLA	15	41828095	41848155
ENSG0000018035	-0.349112714	####	###	ZNF609	protein_coding	zinc finger p	15	64460742	64686068
ENSG0000019704	0.30855748	####	###	GMFB	protein_coding	glia maturat	14	54474484	54489026
ENSG0000015204	-8.297659256	####	###	KCNE4	protein_coding	potassium v	2	223051814	223198399
ENSG0000010110	0.705334297	####	###	PABPC1L	protein_coding	poly(A) binc	20	44910060	44959035
ENSG0000016363	0.349139833	####	###	PSMD6	protein_coding	proteasome	3	64010550	64024010
ENSG0000016589	0.697499267	####	###	ARHGAP42	protein_coding	Rho GTPase	11	100687288	100993941
ENSG0000010537	0.722407284	####	###	ETFB	protein_coding	electron tra	19	51345169	51366388
ENSG0000014364	0.321141587	####	###	GALNT2	protein_coding	polypeptide	1	230057990	230282122
ENSG0000027837	-0.590428463	####	###	MYO19	protein_coding	myosin XIX	CHR_HSCHR	36496005	36543807
ENSG0000018575	0.712442133	####	###	CXorf38	protein_coding	chromosom	X	40626921	40647561
ENSG0000022374	1.87009455	####	###	MIR503HG	lncRNA	MIR503 hos	X	134543119	134546642
ENSG0000013916	0.42370813	####	###	ZCRB1	protein_coding	zinc finger C	12	42312086	42326118
ENSG0000016533	0.464662819	####	###	HECTD2	protein_coding	HECT doma	10	91409280	91514829
ENSG0000013377	-0.512487649	####	###	CCDC59	protein_coding	coiled-coil c	12	82223681	82358805
ENSG0000011531	0.243150449	####	###	RTN4	protein_coding	reticulon 4 [	2	54972187	55112621
ENSG0000010072	-0.27487034	####	###	ZC3H14	protein_coding	zinc finger C	14	88562970	88627596
ENSG0000013694	0.262702452	####	###	RPL35	protein_coding	ribosomal p	9	124857880	124861981
ENSG0000010073	-0.33819067	####	###	PCNX1	protein_coding	pecanex 1 [	14	70907405	71115382
ENSG0000025504	4.366115761	####	###	AC069185.1	lncRNA	novel transc	8	11797928	11802568
ENSG0000001640	-1.741716091	####	###	IL20RA	protein_coding	interleukin 2	6	136999971	137045180
ENSG0000013687	0.576972076	####	###	USP20	protein_coding	ubiquitin sp	9	129834698	129881828
ENSG0000021310	-1.39156331	####	###	AL365223.1	transcribed_pr	ribosomal p	6	136900233	136901529
ENSG0000006836	-0.403685578	####	###	ACSL4	protein_coding	acyl-CoA sy	X	109624244	109733403
ENSG0000016375	0.715779335	####	###	HPS3	protein_coding	HPS3 bioge	3	149129638	149173732
ENSG0000010406	0.758697843	####	###	TJP1	protein_coding	tight junctio	15	29699367	29968865
ENSG0000016564	-2.554253002	####	###	COMTD1	protein_coding	catechol-O-	10	75233641	75236030
ENSG0000019790	-0.718879094	####	###	H2BC12	protein_coding	H2B clustere	6	27146361	27146855
ENSG0000014504	-0.554676179	####	###	DCAF1	protein_coding	DDB1 and C	3	51395867	51500002
ENSG0000010438	0.338779112	####	###	RAB2A	protein_coding	RAB2A, mer	8	60516936	60623644
ENSG0000010589	-0.276829886	####	###	PTN	protein_coding	pleiotrophir	7	137227341	137343774
ENSG0000016195	-8.25734772	####	###	FGF11	protein_coding	fibroblast gi	17	7438273	7444937
ENSG0000016074	-0.562256032	####	###	CRTC2	protein_coding	CREB regula	1	153947669	153958615
ENSG0000011701	-4.740216042	####	###	KCNQ4	protein_coding	potassium v	1	40783787	40840452
ENSG0000027428	0.689820587	####	###	SCRIB	protein_coding	scribble plai	CHR_HSCHR	143777927	143802386
ENSG0000011092	-0.701870885	####	###	MVK	protein_coding	mevalonate	12	109573255	109598125
ENSG0000025166	1.533932258	####	###	PCDHA12	protein_coding	protocadhe	5	140875302	141012347
ENSG0000014597	0.651598292	####	###	TBC1D7	protein_coding	TBC1 doma	6	13266542	13328583
ENSG0000016282	-0.655653117	####	###	NBPF20	protein_coding	NBPF memb	1	145289900	145405778
ENSG0000010008	-0.451091194	####	###	HIRA	protein_coding	histone cell	22	19330698	19447450
ENSG0000011379	1.228208286	####	###	EHHADH	protein_coding	enoyl-CoA I	3	185190624	185281990
ENSG0000007921	-6.095242183	####	###	SLC1A3	protein_coding	solute carrie	5	36606355	36688334
ENSG0000011364	-1.667155645	####	###	WWC1	protein_coding	WW and C2	5	168291645	168472303

ENSG0000010932	0.810484334	####	###	MANBA	protein_coding	mannosidas	4	102630770	102760994
ENSG0000014766	0.496430177	####	###	POLR2K	protein_coding	RNA polyme	8	100150623	100154003
ENSG0000003301	0.648620747	####	###	ALG1	protein_coding	ALG1 chitob	16	5033923	5087379
ENSG0000011669	-0.297303834	####	###	SMG7	protein_coding	SMG7 nonse	1	183472216	183598246
ENSG0000014333	-0.396287662	####	###	TOR1AIP1	protein_coding	torsin 1A int	1	179882042	179925000
ENSG0000018774	0.389143622	####	###	SECISBP2	protein_coding	SECIS bindir	9	89318500	89359663
ENSG0000019845	-0.953376394	####	###	ZNF568	protein_coding	zinc finger p	19	36916329	36998700
ENSG0000018044	-3.574092484	####	###	SERTM1	protein_coding	serine rich a	13	36674020	36697839
ENSG0000010325	-1.489087427	####	###	HAGHL	protein_coding	hydroxyacyl	16	726936	735525
ENSG0000010818	0.921498537	####	###	PBLD	protein_coding	phenazine b	10	68282660	68333049
ENSG0000014036	0.628486857	####	###	COMMD4	protein_coding	COMM dom	15	75336020	75343224
ENSG0000013682	-3.030132362	####	###	KLF4	protein_coding	Kruppel like	9	107484852	107490482
ENSG0000016723	0.552098709	####	###	ZNF91	protein_coding	zinc finger p	19	23304991	23395471
ENSG0000022513	-1.251966139	####	###	NA	NA	NA	NA	NA	NA
ENSG0000016259	-2.291158334	####	###	MEGF6	protein_coding	multiple EGI	1	3487951	3611508
ENSG0000006431	0.345056051	####	###	TAF2	protein_coding	TATA-box b	8	119730774	119832841
ENSG0000027854	-1.136722562	####	###	MARCHF8	protein_coding	membrane	CHR_HSCHR	45470650	45480390
ENSG0000008719	0.289350201	####	###	PSMC5	protein_coding	proteasome	17	63827152	63832026
ENSG0000010992	-0.298792793	####	###	FNBP4	protein_coding	formin bind	11	47716494	47767443
ENSG0000018053	-0.406321292	####	###	NRIP1	protein_coding	nuclear rece	21	14961235	15065936
ENSG0000026300	2.313243917	####	###	AC007114.1	lncRNA	novel transc	17	57071814	57085024
ENSG0000011455	0.288360434	####	###	PLXNA1	protein_coding	plexin A1 [S	3	126988594	127037392
ENSG0000010040	-0.301356223	####	###	ZC3H7B	protein_coding	zinc finger C	22	41301525	41360147
ENSG0000027655	0.347287622	####	###	HERC2P2	transcribed_un	hect domair	15	22495570	22590815
ENSG0000014353	-0.547302693	####	###	ADAM15	protein_coding	ADAM meta	1	155050566	155062775
ENSG0000016381	-0.896436252	####	###	LZTFL1	protein_coding	leucine zipp	3	45823316	45916042
ENSG0000017052	-0.308364561	####	###	PFKFB3	protein_coding	6-phosphof	10	6144934	6254644
ENSG0000021571	0.60514336	####	###	TMEM242	protein_coding	transmembı	6	157289025	157323601
ENSG0000016292	0.259019249	####	###	WDR26	protein_coding	WD repeat c	1	224385143	224437033
ENSG0000017974	1.432089961	####	###	FLJ37453	lncRNA	uncharacter	1	15834474	15848147
ENSG0000014978	0.513671831	####	###	PLCB3	protein_coding	phospholipa	11	64251530	64269398
ENSG0000007725	0.410324468	####	###	USP33	protein_coding	ubiquitin sp	1	77695987	77759852
ENSG0000011331	1.839532332	####	###	RASGRF2	protein_coding	Ras protein	5	80960363	81230162
ENSG0000015940	-2.858353564	####	###	C1R	protein_coding	complemen	12	7080214	7092540
ENSG0000011873	-0.599439531	####	###	OLFM3	protein_coding	olfactomedi	1	101802560	101996926
ENSG0000019729	-3.001261634	####	###	RAMP2-AS	lncRNA	RAMP2 anti	17	42753914	42761257
ENSG0000010837	-2.263949307	####	###	RGS9	protein_coding	regulator of	17	65100812	65227703
ENSG0000005521	0.595014342	####	###	GINM1	protein_coding	glycoprotein	6	149566294	149591748
ENSG0000020485	0.726439725	####	###	TCTN1	protein_coding	tectonic fam	12	110614027	110649430
ENSG0000025502	2.663473383	####	###	AF131216.3	lncRNA	novel transc	8	11345748	11347502
ENSG0000026732	1.950155396	####	###	LINC01415	lncRNA	long interge	18	55776727	55781722
ENSG0000027398	2.791138457	####	###	H3C8	protein_coding	H3 clusterec	6	26269405	26271815
ENSG0000018933	-0.811418202	####	###	KAZN	protein_coding	kazrin, perip	1	13892792	15118043
ENSG0000010467	-0.483956674	####	###	DCTN6	protein_coding	dynactin sul	8	30156319	30183639
ENSG0000016994	0.805519849	####	###	ZFPM2	protein_coding	zinc finger p	8	104590733	105804539
ENSG0000011374	-4.156861616	####	###	HRH2	protein_coding	histamine re	5	175658030	175710756
ENSG0000007682	-2.789650767	####	###	CAMSAP3	protein_coding	calmodulin	19	7595863	7618304
ENSG0000014822	0.316080492	####	###	POLE3	protein_coding	DNA polym	9	113407235	113410675
ENSG0000017983	0.416661358	####	###	SERTAD2	protein_coding	SERTA dom	2	64631621	64751005
ENSG0000010843	0.459938243	####	###	GOSR2	protein_coding	golgi SNAP	17	46923075	46975524
ENSG0000015243	1.571930619	####	###	ZNF773	protein_coding	zinc finger p	19	57499915	57518404
ENSG0000017796	0.393831231	####	###	RIC8A	protein_coding	RIC8 guanir	11	207511	215113
ENSG0000017192	-0.619819558	####	###	TVP23B	protein_coding	trans-golgi	17	18781111	18806714
ENSG0000013686	0.309143575	####	###	CDK5RAP2	protein_coding	CDK5 regula	9	120388869	120580170
ENSG0000016491	0.323826643	####	###	COX6C	protein_coding	cytochrome	8	99873200	99893707
ENSG0000010280	-0.92594942	####	###	CLN5	protein_coding	CLN5 intrac	13	76990660	77019143
ENSG0000016168	0.646441311	####	###	FAM171A2	protein_coding	family with s	17	44353215	44363853
ENSG0000011177	0.262135037	####	###	COX6A1	protein_coding	cytochrome	12	120438090	120440737

ENSG0000023608	1.180229956	####	###	COX10-AS	lncRNA	COX10 antis	17	13755574	14069495
ENSG0000016352	-0.318202083	####	###	STT3B	protein_coding	STT3 oligos	3	31532638	31637616
ENSG0000006986	-0.42356728	####	###	NEDD4	protein_coding	NEDD4 E3 u	15	55826922	55993746
ENSG0000013645	-0.307567869	####	###	VEZF1	protein_coding	vascular enc	17	57971547	57988259
ENSG0000018200	-0.392559748	####	###	SNRPE	protein_coding	small nuclea	1	203861599	203871152
ENSG0000018475	0.472216363	####	###	NDUFA12	protein_coding	NADH:ubiqu	12	94897055	95003748
ENSG0000012514	1.560060135	####	###	MT2A	protein_coding	metallothior	16	56608584	56609497
ENSG0000012438	0.480937591	####	###	SNRNP27	protein_coding	small nuclea	2	69893956	69905575
ENSG0000001124	0.341941569	####	###	AKAP8L	protein_coding	A-kinase an	19	15380050	15419141
ENSG0000027549	-0.588842603	####	###	PDXDC1	protein_coding	pyridoxal de	CHR_HSCHR	15058498	15122225
ENSG0000015174	0.530455827	####	###	SAV1	protein_coding	salvador far	14	50632058	50668306
ENSG0000013796	0.992651541	####	###	ARHGAP29	protein_coding	Rho GTPase	1	94148988	94275068
ENSG0000021306	0.405542561	####	###	SFT2D2	protein_coding	SFT2 domai	1	168225938	168253021
ENSG0000016441	0.618745105	####	###	SLC35A1	protein_coding	solute carrie	6	87470623	87512336
ENSG0000010257	-0.39365727	####	###	STK24	protein_coding	serine/threc	13	98445185	98577940
ENSG0000015525	0.527339474	####	###	ZFYVE27	protein_coding	zinc finger F	10	97737121	97760907
ENSG0000023544	-2.910589591	####	###	LURAP1L-A	lncRNA	LURAP1L ar	9	12698554	12814377
ENSG0000016541	0.302585256	####	###	GTF2A1	protein_coding	general trar	14	81175452	81221377
ENSG0000010211	0.43971534	####	###	EMD	protein_coding	emerin [Sou	X	154379273	154381523
ENSG0000010598	0.344959805	####	###	LMBR1	protein_coding	limb develo	7	156668946	156893216
ENSG0000018568	-1.409389576	####	###	PRAME	protein_coding	preferentiall	22	22547701	22559361
ENSG0000015934	0.361417584	####	###	ADIPOR1	protein_coding	adiponectin	1	202940826	202958572
ENSG0000016796	-0.451788781	####	###	ZNF598	protein_coding	zinc finger p	16	1997654	2009821
ENSG0000018463	0.801198984	####	###	ZNF93	protein_coding	zinc finger p	19	19900913	19963464
ENSG0000024418	0.816119501	####	###	TMEM141	protein_coding	transmembr	9	136791344	136793317
ENSG0000013424	-0.847863372	####	###	PTGFRN	protein_coding	prostagland	1	116909916	116990353
ENSG0000011786	0.372933048	####	###	ESYT2	protein_coding	extended sy	7	158730995	158830253
ENSG0000015697	-0.212943248	####	###	EIF4A2	protein_coding	eukaryotic t	3	186783205	186789897
ENSG0000027898	-2.988876006	####	###	AP001148.1	TEC	novel transc	11	86434924	86437282
ENSG0000009513	0.232517486	####	###	ARCN1	protein_coding	archain 1 [S	11	118572390	118603033
ENSG0000007969	-0.715972649	####	###	CARMIL1	protein_coding	capping prc	6	25279078	25620530
ENSG0000017647	-0.829681281	####	###	SGF29	protein_coding	SAGA comp	16	28553915	28591790
ENSG0000022547	0.81698201	####	###	JPX	lncRNA	JPX transcri	X	73944182	74070408
ENSG0000016690	-0.67829365	####	###	PIP4K2C	protein_coding	phosphatidy	12	57591174	57603418
ENSG0000018561	-2.652760239	####	###	INKA1	protein_coding	inka box act	3	49803261	49805030
ENSG0000011762	0.597127787	####	###	SLC35A3	protein_coding	solute carrie	1	99969351	100035634
ENSG0000017754	0.746691939	####	###	SLC25A22	protein_coding	solute carrie	11	790475	798281
ENSG0000017750	-2.365942394	####	###	IRX3	protein_coding	iroquois hor	16	54283304	54286787
ENSG0000023177	1.848398009	####	###	TMEM44-A	lncRNA	TMEM44 an	3	194584004	194590260
ENSG0000016881	-3.498828837	####	###	IL12A	protein_coding	interleukin 1	3	159988835	159996019
ENSG0000016406	0.565041462	####	###	BSN	protein_coding	bassoon pre	3	49554477	49671549
ENSG0000017492	-2.358749335	####	###	C3orf33	protein_coding	chromosom	3	155762617	155806278
ENSG0000018862	1.649050551	####	###	ZNF177	protein_coding	zinc finger p	19	9363020	9382617
ENSG0000018488	0.64876718	####	###	BTBD6	protein_coding	BTB domain	14	105248490	105251093
ENSG0000015590	0.614516624	####	###	RASA2	protein_coding	RAS p21 prc	3	141487027	141615344
ENSG0000023485	-0.405745167	####	###	HNRNPUL2	protein_coding	HNRNPUL2	11	62690275	62727384
ENSG0000010011	0.779972463	####	###	GCAT	protein_coding	glycine C-ac	22	37807905	37817176
ENSG0000025441	-1.120247569	####	###	CHKB-CPT1	protein_coding	CHKB-CPT1	22	50568869	50582965
ENSG0000008187	0.929880006	####	###	HSPB11	protein_coding	heat shock p	1	53916574	53945929
ENSG0000010666	0.468099594	####	###	CLIP2	protein_coding	CAP-Gly do	7	74289407	74405935
ENSG0000007604	-0.398587463	####	###	REXO2	protein_coding	RNA exonuc	11	114439435	114450279
ENSG0000017844	-7.533314849	####	###	GLDC	protein_coding	glycine deca	9	6532467	6645729
ENSG0000021189	-7.518875728	####	###	IGHM	IG_C_gene	immunoglol	14	105851705	105856218
ENSG0000016348	-0.669532681	####	###	STK36	protein_coding	serine/threc	2	218672069	218702716
ENSG0000015435	-0.87087709	####	###	LONRF1	protein_coding	LON peptid	8	12721906	12756073
ENSG0000021362	0.618734937	####	###	LEPROT	protein_coding	leptin recep	1	65420587	65436007
ENSG0000018030	0.365783563	####	###	OAZ2	protein_coding	ornithine de	15	64687573	64703281
ENSG0000018074	-0.539352156	####	###	SMG1P3	transcribed_un	SMG1 pseu	16	21446683	21520444

ENSG0000012264	-1.194809106	####	###	ARL4A	protein_coding	ADP ribosyl	7	12686856	12690958
ENSG0000025531	-1.34688876	####	###	AF131215.5	lncRNA	novel transc	8	11107788	11109726
ENSG0000017105	-1.489315841	####	###	SOX7	protein_coding	SRY-box tra	8	10723768	10730511
ENSG0000015711	-1.642889543	####	###	RBPMS	protein_coding	RNA binding	8	30384511	30572256
ENSG0000007179	0.300616052	####	###	HLTF	protein_coding	helicase like	3	149030127	149086554
ENSG0000012923	0.836935503	####	###	TXNDC17	protein_coding	thioredoxin	17	6640985	6644541
ENSG0000010968	0.241885615	####	###	NSD2	protein_coding	nuclear rece	4	1871393	1982207
ENSG0000006771	0.255205367	####	###	SYT1	protein_coding	synaptotagr	12	78863993	79452008
ENSG0000014402	0.366392955	####	###	CIAO1	protein_coding	cytosolic iro	2	96266159	96274173
ENSG0000017534	-0.770852576	####	###	CHRNA7	protein_coding	cholinergic	15	31923438	32173018
ENSG0000012025	0.361555495	####	###	NUP43	protein_coding	nucleoporin	6	149724315	149749665
ENSG0000022708	-0.354758973	####	###	AC005912.1	processed_pse	ribosomal p	12	3211663	3211917
ENSG0000014601	-2.676337026	####	###	GFRA3	protein_coding	GDNF family	5	138252380	138274621
ENSG0000019615	-2.233387421	####	###	PLEKHG4	protein_coding	pleckstrin h	16	67277510	67289499
ENSG0000021541	0.971090202	####	###	MIR17HG	lncRNA	miR-17-92a	13	91347820	91354579
ENSG0000025556	1.205673533	####	###	FDXACB1	protein_coding	ferredoxin-f	11	111874056	111881243
ENSG0000023655	-0.637659746	####	###	RPL13AP5	processed_pse	ribosomal p	10	96750288	96750899
ENSG0000019641	-0.65442189	####	###	EPHB4	protein_coding	EPH recepto	7	100802565	100827523
ENSG0000006861	1.5427385	####	###	REEP1	protein_coding	receptor acc	2	86213993	86338083
ENSG0000017027	-0.369424135	####	###	CRTAP	protein_coding	cartilage ass	3	33114014	33147773
ENSG0000016969	-0.704806859	####	###	ASPSCR1	protein_coding	ASPSCR1 te	17	81976807	82017406
ENSG0000004954	-1.089288901	####	###	ELN	protein_coding	elastin [Sou	7	74027789	74069907
ENSG0000020451	0.962690179	####	###	ZNF551	protein_coding	zinc finger p	19	57681969	57717301
ENSG0000015586	1.015082733	####	###	MED7	protein_coding	mediator cc	5	157137424	157159019
ENSG0000019858	0.465421805	####	###	NUDT16	protein_coding	nudix hydro	3	131381671	131388830
ENSG0000005274	-0.459147199	####	###	RRP12	protein_coding	ribosomal R	10	97356358	97426076
ENSG0000004924	-0.837904055	####	###	PER3	protein_coding	period circa	1	7784320	7845177
ENSG0000013932	-0.553012753	####	###	POC1B	protein_coding	POC1 centri	12	89419718	89526047
ENSG0000016475	0.464885935	####	###	PEX2	protein_coding	peroxisoma	8	76980258	77001044
ENSG0000017508	-0.611863405	####	###	PDIK1L	protein_coding	PDLIM1 inte	1	26111165	26125555
ENSG0000019726	-2.192295918	####	###	C6orf141	protein_coding	chromosom	6	49550646	49561907
ENSG0000014394	1.077664841	####	###	CHAC2	protein_coding	ChaC glutat	2	53767804	53775196
ENSG0000016873	-0.931984841	####	###	PKIG	protein_coding	cAMP-depe	20	44531785	44624247
ENSG0000025048	2.062111551	####	###	FAM218A	lncRNA	family with	4	164956948	164959122
ENSG0000019825	0.518516104	####	###	STYX	protein_coding	serine/threc	14	52730166	52774989
ENSG0000010608	0.699678163	####	###	STX1A	protein_coding	syntaxin 1A	7	73699206	73719672
ENSG0000018663	0.460783832	####	###	ARAP1	protein_coding	ArfGAP with	11	72685069	72793599
ENSG0000018400	0.610879433	####	###	ST6GALNAC	protein_coding	ST6 N-acety	1	76074746	76634603
ENSG0000009926	-1.454804313	####	###	PALMD	protein_coding	palmdelphin	1	99646113	99694541
ENSG0000005343	0.35753513	####	###	NNAT	protein_coding	neuronatin	20	37521206	37523690
ENSG0000014046	0.919743011	####	###	BBS4	protein_coding	Bardet-Biec	15	72686179	72738475
ENSG0000019655	-0.710810975	####	###	CACNA1H	protein_coding	calcium volt	16	1153106	1221771
ENSG0000019893	-0.77309884	####	###	TBKBP1	protein_coding	TBK1 bindin	17	47694081	47712050
ENSG0000012196	-0.763419653	####	###	GTDC1	protein_coding	glycosyltran	2	143938068	144332568
ENSG0000019879	0.565307706	####	###	TMEM184B	protein_coding	transmembr	22	38219291	38273010
ENSG0000006498	-2.948283474	####	###	CALCRL	protein_coding	calcitonin re	2	187341964	187448460
ENSG0000019838	-0.519357724	####	###	UVRAG	protein_coding	UV radiation	11	75815210	76144232
ENSG0000013330	0.690059176	####	###	SLF1	protein_coding	SMC5-SMC	5	94618669	94739436
ENSG0000015509	-0.399378999	####	###	ATP6V1C1	protein_coding	ATPase H+	8	103021063	103073051
ENSG0000017359	-0.310057594	####	###	NUDT4	protein_coding	nudix hydro	12	93377883	93408146
ENSG0000025805	1.908236879	####	###	BCDIN3D-A	lncRNA	BCDIN3D ar	12	49827913	49841143
ENSG0000011299	-0.450946955	####	###	MRPS30	protein_coding	mitochondr	5	44808947	44820428
ENSG0000011901	0.511063619	####	###	NDUFB3	protein_coding	NADH:ubiqu	2	201071433	201085750
ENSG0000016262	0.484568493	####	###	SNX7	protein_coding	sorting nexi	1	98661701	98760500
ENSG0000023048	1.419724488	####	###	PSMG3-AS	lncRNA	PSMG3 anti	7	1570073	1589626
ENSG0000020426	-1.715691423	####	###	TAP2	protein_coding	transporter	6	32821833	32838770
ENSG0000006720	0.689407755	####	###	EVI5	protein_coding	ecotropic vi	1	92508696	92792404
ENSG0000010805	0.223154988	####	###	SMC3	protein_coding	structural m	10	110567695	110606048

ENSG0000018226	-0.563026676	####	###	FIGN	protein_coding	fidgetin, mic	2	163593396	163736012
ENSG0000015147	0.766843822	####	###	C4orf33	protein_coding	chromosom	4	129093317	129116640
ENSG0000015695	-0.76040367	####	###	GALK2	protein_coding	galactokina	15	49155656	49367869
ENSG0000016937	-0.828800839	####	###	SNUPN	protein_coding	snurportin 1	15	75598083	75626469
ENSG0000024447	-3.502495375	####	###	ERVFRD-1	protein_coding	endogenous	6	11102489	11111725
ENSG0000019734	0.510374378	####	###	MRPL21	protein_coding	mitochondr	11	68891276	68903835
ENSG0000018364	0.750369501	####	###	NDUFB1	protein_coding	NADH:ubiqu	14	92116122	92121917
ENSG0000020418	-0.352786708	####	###	ZDBF2	protein_coding	zinc finger D	2	206274663	206314427
ENSG0000013196	0.406286463	####	###	ACTR10	protein_coding	actin relatec	14	58200080	58235636
ENSG0000010355	0.44985398	####	###	KNOP1	protein_coding	lysine rich n	16	19701937	19718235
ENSG0000015147	-1.325854105	####	###	FRMD4A	protein_coding	FERM doma	10	13643706	14462142
ENSG0000010718	-0.310263288	####	###	MPDZ	protein_coding	multiple PD	9	13105704	13279590
ENSG0000013138	0.533873183	####	###	RBSN	protein_coding	rabenosyn,	3	15070073	15099163
ENSG0000010566	-0.726189753	####	###	CRTC1	protein_coding	CREB regula	19	18683678	18782333
ENSG0000014626	-0.574847234	####	###	FAXC	protein_coding	failed axon	6	99271168	99350062
ENSG0000016809	0.396150435	####	###	COPS6	protein_coding	COP9 signa	7	100088969	100092187
ENSG0000024595	0.736556962	####	###	AC093752.1	transcribed_un	septin 4 (sep	4	119454791	119552025
ENSG0000008353	-0.68837106	####	###	PIBF1	protein_coding	progesteror	13	72782133	73016461
ENSG0000014082	0.355140153	####	###	DHX38	protein_coding	DEAH-box l	16	72093613	72112912
ENSG0000012795	0.42695868	####	###	GNAI1	protein_coding	G protein su	7	79768028	80226181
ENSG0000008583	-2.501629427	####	###	TTC39A	protein_coding	tetratricope	1	51287258	51345116
ENSG0000008977	0.567663999	####	###	ZBTB25	protein_coding	zinc finger a	14	64449106	64505213
ENSG0000016325	1.825921295	####	###	FZD5	protein_coding	frizzled clas	2	207762598	207769906
ENSG0000019749	2.923521991	####	###	ZNF665	protein_coding	zinc finger p	19	53159213	53193386
ENSG0000012419	-0.345530806	####	###	ARFGEF2	protein_coding	ADP ribosyl	20	48921711	49036693
ENSG0000027825	-0.62362407	####	###	MYO19	protein_coding	myosin XIX	17	36495633	36543435
ENSG0000016810	0.703216779	####	###	NUDT16L1	protein_coding	nudix hydro	16	4693694	4695859
ENSG0000017672	-2.218139813	####	###	BOK	protein_coding	BCL2 family	2	241551424	241574131
ENSG0000013514	-3.263014064	####	###	DTX1	protein_coding	deltex E3 ub	12	113056709	113098028
ENSG0000000518	1.291589763	####	###	REXO5	protein_coding	RNA exonuc	16	20806429	20849668
ENSG0000014501	-0.680954605	####	###	LPP	protein_coding	LIM domain	3	188153284	188890671
ENSG0000007185	0.450550331	####	###	FAM50A	protein_coding	family with s	X	154444141	154450654
ENSG0000022543	-0.874627307	####	###	BOLA3-AS1	lncRNA	BOLA3 dive	2	74148007	74151952
ENSG0000007794	-2.557728853	####	###	ITGA8	protein_coding	integrin sub	10	15513954	15719922
ENSG0000011211	0.253627541	####	###	MCM3	protein_coding	minichromo	6	52264014	52284881
ENSG0000023033	2.33855314	####	###	HMGN2P3	processed_pse	high mobilit	16	26032539	26032811
ENSG0000024779	1.838707447	####	###	AC008966.1	lncRNA	novel transc	5	53109816	53127673
ENSG0000018042	-5.921855506	####	###	LINC00304	lncRNA	long interge	16	89159146	89164245
ENSG0000016677	1.080939627	####	###	ZNF667-AS	lncRNA	ZNF667 ant	19	56477250	56504362
ENSG0000014338	1.795364167	####	###	ADAMTSL4	protein_coding	ADAMTS lik	1	150549369	150560937
ENSG0000018802	-1.124029124	####	###	RILPL1	protein_coding	Rab interact	12	123470054	123533719
ENSG0000027990	-2.731036208	####	###	AC063926.1	TEC	novel transc	12	130425004	130430661
ENSG0000020591	3.376682681	####	###	SRRM2-AS	lncRNA	SRRM2 antis	16	2737076	2752600
ENSG0000027058	-0.97460184	####	###	PKD1P6-NI	lncRNA	PKD1P6-NP	16	15104723	15131601
ENSG0000023551	-2.095388421	####	###	L3MBTL2-A	lncRNA	L3MBTL2 ar	22	41207592	41228500
ENSG0000012335	-0.406653793	####	###	SPATS2	protein_coding	spermatoge	12	49366584	49527425
ENSG0000026232	0.81679517	####	###	AGK	protein_coding	acylglycerol	CHR_HSCHR	141633901	141655244
ENSG0000018349	0.290979681	####	###	EP400	protein_coding	E1A binding	12	131949942	132080460
ENSG0000016406	0.668155767	####	###	RNF123	protein_coding	ring finger p	3	49689538	49721529
ENSG0000013582	0.240955664	####	###	GLUL	protein_coding	glutamate-a	1	182378098	182392206
ENSG0000015213	-2.04193182	####	###	HSPB8	protein_coding	heat shock p	12	119171555	119224855
ENSG0000012341	0.75693717	####	###	SMUG1	protein_coding	single-stran	12	54121277	54189008
ENSG0000025161	-1.578768435	####	###	AC104825.1	lncRNA	novel transc	4	8355090	8358338
ENSG0000023596	-2.637751599	####	###	PNMA6A	protein_coding	PNMA famil	X	153072454	153075019
ENSG0000015921	-3.106487644	####	###	CCDC24	protein_coding	coiled-coil	1	43991359	43996528
ENSG0000026696	0.615130958	####	###	AARSD1	protein_coding	alanyl-tRNA	17	42950526	42964498
ENSG0000010298	-1.60262623	####	###	PARD6A	protein_coding	par-6 family	16	67660946	67662778
ENSG0000012864	0.205232295	####	###	MYO1B	protein_coding	myosin IB [S	2	191245185	191425389

ENSG0000017572	0.314386595	####	###	MLXIP	protein_codinç	MLX interac	12	122078756	122147344
ENSG0000013488	0.306924792	####	###	ARGLU1	protein_codinç	arginine anc	13	106541673	106568137
ENSG0000017535	3.031587789	####	###	NRIP3	protein_codinç	nuclear rece	11	8980576	9004049
ENSG0000015530	-1.783183048	####	###	SAMSN1	protein_codinç	SAM domai	21	14485228	14658821
ENSG0000024506	-7.992983064	####	###	IGFBP7-AS	lncRNA	IGFBP7 antis	4	57109762	57207459
ENSG0000010555	-4.259516255	####	###	FGF21	protein_codinç	fibroblast gı	19	48755524	48758333
ENSG0000016957	-1.251997787	####	###	DTWD2	protein_codinç	DTW domai	5	118836074	118988547
ENSG0000015908	0.468305617	####	###	SYNJ1	protein_codinç	synaptojanii	21	32628759	32728040
ENSG0000013016	-0.489145266	####	###	LDLR	protein_codinç	low density	19	11089462	11133820
ENSG0000013020	-1.463996959	####	###	APOE	protein_codinç	apolipoprot	19	44905791	44909393
ENSG0000009203	-0.707163501	####	###	HAUS4	protein_codinç	HAUS augm	14	22946228	22957161
ENSG0000026922	1.060694419	####	###	TMSB15B	protein_codinç	thymosin beX		104063871	104076212
ENSG0000008981	0.437506573	####	###	NECAP1	protein_codinç	NECAP end	12	8076939	8097881
ENSG0000014199	-0.613395389	####	###	DUS3L	protein_codinç	dihydrourid	19	5784832	5791225
ENSG0000017914	-5.962464185	####	###	MTUS2-AS	lncRNA	MTUS2 anti:	13	29476515	29490105
ENSG0000008064	0.367542733	####	###	CHRNA3	protein_codinç	cholinergic	15	78593052	78621295
ENSG0000013437	-0.308108125	####	###	TIMM17A	protein_codinç	translocase	1	201955503	201970664
ENSG0000014948	-0.272880036	####	###	MTA2	protein_codinç	metastasis a	11	62593214	62601865
ENSG0000012682	0.554670674	####	###	SGPP1	protein_codinç	sphingosine	14	63684216	63728065
ENSG0000013833	-0.535708071	####	###	TET1	protein_codinç	tet methylc	10	68560337	68694487
ENSG0000015171	-0.468365891	####	###	WWC2	protein_codinç	WW and C2	4	183099257	183320777
ENSG0000010385	0.948239947	####	###	TTC23	protein_codinç	tetratricope	15	99136323	99251223
ENSG0000015757	1.8823381	####	###	LCA5L	protein_codinç	lebercilin LC	21	39405844	39445805
ENSG0000017337	-3.782906476	####	###	NDNF	protein_codinç	neuron deri	4	121035613	121073021
ENSG0000018766	-5.92491244	####	###	HAPLN4	protein_codinç	hyaluronan	19	19254756	19262804
ENSG0000020425	0.321506893	####	###	BRD2	protein_codinç	bromodoma	6	32968594	32981505
ENSG0000015233	0.297656616	####	###	UHMK1	protein_codinç	U2AF homo	1	162497251	162529631
ENSG0000007542	-0.328934615	####	###	FNDC3B	protein_codinç	fibronectin t	3	172039578	172401669
ENSG0000019620	0.756794718	####	###	RNF216P1	lncRNA	ring finger p	7	4973988	5040675
ENSG0000012964	1.153493022	####	###	QRICH2	protein_codinç	glutamine ri	17	76274049	76307998
ENSG0000016918	0.304241496	####	###	XPO6	protein_codinç	exportin 6 [	16	28097976	28211965
ENSG0000011125	-0.441977311	####	###	SH2B3	protein_codinç	SH2B adapt	12	111405923	111451623
ENSG0000015595	0.372404215	####	###	VBP1	protein_codinç	VHL bindinçX		155197007	155239841
ENSG0000010119	0.337582342	####	###	GID8	protein_codinç	GID comple	20	62938147	62948475
ENSG0000007890	-2.984347385	####	###	TP73	protein_codinç	tumor prote	1	3652516	3736201
ENSG0000011928	-0.291503067	####	###	HEATR1	protein_codinç	HEAT repea	1	236549005	236604516
ENSG0000023058	7.965883671	####	###	GTF2IRD1P	transcribed_un	GTF2I repea	7	66809993	66844882
ENSG0000010452	0.286864986	####	###	EEF1D	protein_codinç	eukaryotic t	8	143579697	143599541
ENSG0000018405	0.896429435	####	###	VPS33B	protein_codinç	VPS33B late	15	90998673	91022603
ENSG0000001204	0.383530051	####	###	BRCA1	protein_codinç	BRCA1 DNA	17	43044295	43170245
ENSG0000013145	2.212440239	####	###	GFPT2	protein_codinç	glutamine-f	5	180300698	180353336
ENSG0000010540	0.463409763	####	###	NAPA	protein_codinç	NSF attachr	19	47487637	47515091
ENSG0000019729	0.723021028	####	###	FITM2	protein_codinç	fat storage i	20	44302840	44311202
ENSG0000017882	3.632221745	####	###	TMEM139	protein_codinç	transmembı	7	143279957	143288048
ENSG0000018588	0.734236798	####	###	TRIM69	protein_codinç	tripartite mc	15	44728988	44767829
ENSG0000010624	-0.524039438	####	###	PTCD1	protein_codinç	pentatricop	7	99416739	99466163
ENSG0000000671	-0.545541846	####	###	PAF1	protein_codinç	PAF1 homo	19	39385629	39391154
ENSG0000010118	-0.721479145	####	###	MRGBP	protein_codinç	MRG domai	20	62796473	62801729
ENSG0000010668	0.210693971	####	###	EIF4H	protein_codinç	eukaryotic t	7	74174245	74197101
ENSG0000013863	-3.479472979	####	###	ARHGAP24	protein_codinç	Rho GTPase	4	85475150	86002668
ENSG0000012862	-0.875982974	####	###	MRPS12	protein_codinç	mitochondr	19	38930548	38933162
ENSG0000010297	0.656266265	####	###	ACD	protein_codinç	ACD shelter	16	67657512	67660815
ENSG0000017122	0.390809905	####	###	JUNB	protein_codinç	JunB proto-	19	12791486	12793315
ENSG0000012198	0.729467673	####	###	ACVR2A	protein_codinç	activin A rec	2	147844517	147930826
ENSG0000016824	0.315893632	####	###	GNG4	protein_codinç	G protein su	1	235547685	235650754
ENSG0000016263	-0.87821016	####	###	NTNG1	protein_codinç	netrin G1 [S	1	107140007	107483458
ENSG0000010656	-3.112079625	####	###	TMEM176B	protein_codinç	transmembı	7	150791285	150801360
ENSG0000015827	-3.301572135	####	###	COLEC12	protein_codinç	collectin sub	18	316737	500722

ENSG0000025057	1.123456471	####	###	GLI4	protein_coding	GLI family z	8	143267433	143276931
ENSG0000018419	-0.544095014	####	###	GPR173	protein_coding	G protein-c	X	53048789	53080615
ENSG0000010852	0.495354449	####	###	SLC25A11	protein_coding	solute carrie	17	4937130	4940053
ENSG0000027602	-1.129266442	####	###	WDR81	protein_coding	WD repeat	CHR_HSCHR	1716523	1738610
ENSG0000018582	0.388690294	####	###	BCAP31	protein_coding	B cell recep	X	153700492	153724565
ENSG0000017332	0.518607574	####	###	MAP3K11	protein_coding	mitogen-ac	11	65597756	65615382
ENSG0000023200	-1.012097349	####	###	PBX2	protein_coding	PBX homeo	CHR_HSCHR	32224129	32229571
ENSG0000024211	-0.904486596	####	###	MTFP1	protein_coding	mitochondr	22	30425623	30429054
ENSG0000016516	-2.226060056	####	###	CYBB	protein_coding	cytochrome	X	37780059	37813461
ENSG0000011189	0.228048597	####	###	SERINC1	protein_coding	serine incor	6	122443351	122471807
ENSG0000007077	-0.466473789	####	###	PTPN21	protein_coding	protein tyro	14	88465778	88555007
ENSG0000015885	-0.437109436	####	###	B4GALT3	protein_coding	beta-1,4-ga	1	161171310	161177968
ENSG0000017493	0.397722598	####	###	SEZ6L2	protein_coding	seizure relat	16	29871159	29899547
ENSG0000013741	-0.551866812	####	###	TAF8	protein_coding	TATA-box b	6	42050513	42087461
ENSG0000010035	-0.383543906	####	###	TNRC6B	protein_coding	trinucleotide	22	40044817	40335808
ENSG0000014703	-1.731154158	####	###	LANCL3	protein_coding	LanC like 3	X	37571569	37684463
ENSG0000013750	-4.245592429	####	###	LRRC32	protein_coding	leucine rich	11	76657524	76670747
ENSG0000013534	0.350048659	####	###	MAP3K7	protein_coding	mitogen-ac	6	90513573	90587072
ENSG0000007915	0.525375448	####	###	OSBPL6	protein_coding	oxysterol bi	2	178194481	178402893
ENSG0000015601	-0.312914879	####	###	PSD3	protein_coding	pleckstrin ar	8	18527303	19084730
ENSG0000018571	-0.779006337	####	###	SMG1P4	transcribed_un	SMG1 pseu	16	21879338	21919156
ENSG0000022396	0.574977198	####	###	CHROMR	lncRNA	cholesterol	2	178413635	178440243
ENSG0000024398	-1.058010972	####	###	ACY1	protein_coding	aminoacylas	3	51983340	51989197
ENSG0000007723	0.300105177	####	###	GTF3C1	protein_coding	general tran	16	27459555	27549913
ENSG0000027233	-0.525420883	####	###	KMT2B	protein_coding	lysine methy	19	35717973	35738878
ENSG0000027214	2.252670678	####	###	FGF14-AS2	lncRNA	FGF14 antis	13	102394630	102395703
ENSG0000017327	-0.844390092	####	###	MZT2A	protein_coding	mitotic spin	2	131464900	131492743
ENSG0000014032	0.782750385	####	###	DISP2	protein_coding	dispatched	15	40358219	40378621
ENSG0000025513	1.193059464	####	###	AP002360.1	lncRNA	novel transc	11	76435559	76444687
ENSG0000015262	-0.585705654	####	###	NADK2	protein_coding	NAD kinase	5	36192589	36242279
ENSG0000017448	-0.732597682	####	###	LINGO2	protein_coding	leucine rich	9	27948078	28670286
ENSG0000013165	-0.651349235	####	###	THOC6	protein_coding	THO comple	16	3024027	3027755
ENSG0000007484	-0.402604789	####	###	MYDGF	protein_coding	myeloid der	19	4641374	4670362
ENSG0000016391	0.586763311	####	###	IFT122	protein_coding	intraflagella	3	129440036	129520507
ENSG0000017624	0.502100273	####	###	ANAPC2	protein_coding	anaphase pr	9	137174784	137188560
ENSG0000017136	1.171491381	####	###	TPPP	protein_coding	tubulin poly	5	659862	693352
ENSG0000018636	-1.647130074	####	###	NUDT17	protein_coding	nudix hydro	1	145845630	145848954
ENSG0000015692	0.675417855	####	###	MALSU1	protein_coding	mitochondr	7	23298739	23311729
ENSG0000016769	-0.944602312	####	###	TLCD3A	protein_coding	TLC domain	17	732412	742968
ENSG0000014357	-0.375537211	####	###	SLC39A1	protein_coding	solute carrie	1	153959099	153968184
ENSG0000026260	2.132164106	####	###	IQSEC3	protein_coding	IQ motif an	CHR_HSCHR	70780	175481
ENSG0000016269	0.411865665	####	###	EXTL2	protein_coding	exostosin lik	1	100872372	100895179
ENSG0000022821	0.99037408	####	###	NA	NA	NA	NA	NA	NA
ENSG0000026085	-0.984913831	####	###	FBXL19-AS	lncRNA	FBXL19 anti	16	30919319	30923269
ENSG0000016287	-1.314920167	####	###	PKDCC	protein_coding	protein kina	2	42048021	42058517
ENSG0000023751	-2.041664009	####	###	DGCR5	transcribed_un	DiGeorge sy	22	18985836	18994501
ENSG0000019660	1.731754739	####	###	ZNF846	protein_coding	zinc finger p	19	9751993	9793180
ENSG0000014533	0.354859003	####	###	KLHL8	protein_coding	kelch like fa	4	87160103	87240314
ENSG0000014341	-2.197934327	####	###	ANXA9	protein_coding	annexin A9	1	150982249	150995634
ENSG0000019608	0.934086083	####	###	ZNF724	protein_coding	zinc finger p	19	23221599	23250394
ENSG0000016633	0.510532305	####	###	TAF10	protein_coding	TATA-box b	11	6606294	6612539
ENSG0000012131	-0.992132798	####	###	ECHDC2	protein_coding	enoyl-CoA	1	52895910	52927212
ENSG0000023371	-0.414490058	####	###	MYCNOS	lncRNA	MYCN oppo	2	15918350	15942249
ENSG0000011005	-1.61259414	####	###	UNC93B1	protein_coding	unc-93 hom	11	67991100	68004982
ENSG0000024136	0.460366803	####	###	PDXP	protein_coding	pyridoxal ph	22	37658723	37666932
ENSG0000017249	0.292562232	####	###	AFF1	protein_coding	AF4/FMR2 f	4	86935002	87141054
ENSG0000021920	0.793781871	####	###	AC138392.1	processed_pse	novel pseuc	1	77810861	77811781
ENSG0000017110	1.258078389	####	###	MTM1	protein_coding	myotubulari	X	150568621	150673143

ENSG0000017585	1.038144798	####	###	SWI5	protein_coding	SWI5 homo	9	128275379	128316123
ENSG0000017803	0.279488719	####	###	IMPDH2	protein_coding	inosine mor	3	49024325	49029408
ENSG0000021442	-1.083423674	####	###	LRRC37A4P	transcribed_un	leucine rich	17	45506741	45551537
ENSG0000013590	-0.473024723	####	###	MRPL44	protein_coding	mitochondr	2	223957463	223967714
ENSG0000013593	0.346080572	####	###	EIF4E2	protein_coding	eukaryotic t	2	232550674	232583644
ENSG0000018864	0.500522921	####	###	DPYD	protein_coding	dihydropyri	1	97077743	97995000
ENSG0000016610	-2.37989571	####	###	ADAMTS15	protein_coding	ADAM meta	11	130448974	130476641
ENSG0000012539	-1.098876314	####	###	SOX9	protein_coding	SRY-box tra	17	72121020	72126416
ENSG0000019687	0.692751092	####	###	CBWD3	protein_coding	COBW dom	9	68232003	68300035
ENSG0000017108	1.976028824	####	###	FAM86JP	transcribed_un	family with s	3	125916620	125930024
ENSG0000018380	0.354397819	####	###	RBM12B	protein_coding	RNA binding	8	93729356	93741017
ENSG0000014028	-0.865026843	####	###	LYSMD2	protein_coding	LysM doma	15	51723011	51751585
ENSG0000017428	-0.712433681	####	###	ZBTB4	protein_coding	zinc finger a	17	7459366	7484263
ENSG0000019683	-1.800393214	####	###	ADA	protein_coding	adenosine c	20	44619522	44652233
ENSG0000018163	0.691244904	####	###	ZFP41	protein_coding	ZFP41 zinc f	8	143246821	143262705
ENSG0000010892	-0.959540225	####	###	HLF	protein_coding	HLF transcri	17	55264960	55325187
ENSG0000016579	-1.034713764	####	###	NDRG2	protein_coding	NDRG famil	14	21016763	21070872
ENSG0000011402	-0.850023429	####	###	FAM162A	protein_coding	family with s	3	122384176	122412334
ENSG0000016670	0.890450897	####	###	ZNF606	protein_coding	zinc finger p	19	57977053	58003349
ENSG0000005013	0.425315854	####	###	JKAMP	protein_coding	JNK1/MAPK	14	59484443	59505410
ENSG0000014908	-0.573842438	####	###	APIP	protein_coding	APAF1 inter	11	34853094	34916379
ENSG0000011125	2.692552461	####	###	AKAP3	protein_coding	A-kinase an	12	4615508	4649051
ENSG0000024366	0.708299671	####	###	WDR92	protein_coding	WD repeat c	2	68122936	68157549
ENSG0000016626	-0.474324563	####	###	STXBP4	protein_coding	syntaxin bin	17	54968727	55173632
ENSG0000018827	-2.130677693	####	###	C15orf62	protein_coding	chromosom	15	40769980	40772449
ENSG0000007997	0.69038655	####	###	RABL2B	protein_coding	RAB, memb	22	50767501	50783663
ENSG0000018526	-0.453879943	####	###	UBALD2	protein_coding	UBA like do	17	76265348	76271298
ENSG0000023930	0.511741234	####	###	RNF103	protein_coding	ring finger p	2	86603398	86623866
ENSG0000012895	0.309487641	####	###	DUT	protein_coding	deoxyuridin	15	48331011	48343373
ENSG0000011490	0.548074317	####	###	SPCS1	protein_coding	signal pepti	3	52704955	52711148
ENSG0000014138	0.438317248	####	###	AFG3L2	protein_coding	AFG3 like m	18	12328944	12377227
ENSG0000013252	0.54565236	####	###	GPS2	protein_coding	G protein pa	17	7311324	7315564
ENSG0000018240	0.375136461	####	###	TRAPPC6B	protein_coding	trafficking p	14	39147811	39170532
ENSG0000020644	-1.503419634	####	###	NFKBIL1	protein_coding	NFKB inhibi	CHR_HSCHR	31537129	31549080
ENSG0000013849	0.543074538	####	###	COX17	protein_coding	cytochrome	3	119654513	119677454
ENSG0000018262	-0.379942151	####	###	PLCB1	protein_coding	phospholipa	20	8077251	8968360
ENSG0000014913	-3.894447673	####	###	SERPING1	protein_coding	serpin family	11	57597387	57614848
ENSG0000013638	0.232360124	####	###	IREB2	protein_coding	iron respons	15	78437431	78501453
ENSG0000020465	-0.847706153	####	###	LINC02210	transcribed_un	long interge	17	45620328	45655156
ENSG0000018526	-2.696884529	####	###	NOTUM	protein_coding	notum, paln	17	81952507	81961840
ENSG0000010032	0.450506876	####	###	ASCC2	protein_coding	activating si	22	29788609	29838304
ENSG0000024825	4.528575214	####	###	AC107398.2	lncRNA	novel transc	4	47556731	47560259
ENSG0000018608	-4.751498059	####	###	GSAP	protein_coding	gamma-sec	7	77310751	77416349
ENSG0000008928	0.175924699	####	###	FUS	protein_coding	FUS RNA bi	16	31180138	31191605
ENSG0000006493	-0.797219003	####	###	SBNO2	protein_coding	strawberry r	19	1107637	1174268
ENSG0000015584	-1.002017892	####	###	PPARGC1B	protein_coding	PPARG coac	5	149730298	149855022
ENSG0000013580	-0.455762507	####	###	TAF5L	protein_coding	TATA-box b	1	229593111	229626047
ENSG0000009979	0.4167111	####	###	TECR	protein_coding	trans-2,3-er	19	14517085	14565980
ENSG0000009005	0.380915766	####	###	SPTLC1	protein_coding	serine palm	9	92000087	92115413
ENSG0000015252	-0.556614174	####	###	PAN3	protein_coding	poly(A) spec	13	28138506	28295335
ENSG0000019874	-0.571695495	####	###	ZNF652	protein_coding	zinc finger p	17	49289206	49362473
ENSG0000021316	-0.331269927	####	###	KLHL23	protein_coding	kelch like fa	2	169694488	169776989
ENSG0000000502	-0.226774548	####	###	SLC25A5	protein_coding	solute carrie	X	119468422	119471396
ENSG0000013539	-0.333872234	####	###	ATP5MC2	protein_coding	ATP synthas	12	53632726	53677408
ENSG0000016413	0.272236969	####	###	NAA15	protein_coding	N-alpha-ac	4	139301505	139420033
ENSG0000019762	0.267002193	####	###	CDC42SE1	protein_coding	CDC42 sma	1	151050971	151070325
ENSG0000013212	-0.390993114	####	###	LRRC41	protein_coding	leucine rich	1	46261196	46303608
ENSG0000010021	-2.699522627	####	###	XBP1	protein_coding	X-box bindi	22	28794555	28800597

ENSG0000013837	-0.643827229	####	###	SMARCAL1	protein_coding	SWI/SNF rel	2	216412414	216483053
ENSG0000008587	-1.214064116	####	###	MGST2	protein_coding	microsomal	4	139665768	139740745
ENSG0000023336	-0.282771075	####	###	GTF2IP4	transcribed_pr	general tran	7	73154938	73207283
ENSG0000022661	-0.689615781	####	###	SLC39A7	protein_coding	solute carrie	CHR_HSCHR	33122069	33126063
ENSG0000010203	-0.381447823	####	###	SMARCA1	protein_coding	SWI/SNF rel	X	129446501	129523500
ENSG0000019883	-0.399713101	####	###	DENND4B	protein_coding	DENN doma	1	153929501	153946718
ENSG0000020657	0.819006984	####	###	THUMPD3-	lncRNA	THUMPD3 a	3	9349689	9398579
ENSG0000024370	0.914327932	####	###	DUBR	lncRNA	DPPA2 upst	3	107220744	107348464
ENSG0000014218	0.479682182	####	###	SCYL1	protein_coding	SCY1 like ps	11	65525077	65538704
ENSG0000001515	-0.666862724	####	###	YAF2	protein_coding	YY1 associa	12	42157104	42238349
ENSG0000015651	-0.251559076	####	###	HK1	protein_coding	hexokinase	10	69269984	69401884
ENSG0000012503	0.619796099	####	###	EMC3	protein_coding	ER membra	3	9962537	10011116
ENSG0000016363	-0.525966586	####	###	ATXN7	protein_coding	ataxin 7 [So	3	63863155	64003462
ENSG0000008659	-0.256458772	####	###	TMED2	protein_coding	transmembr	12	123584533	123598582
ENSG0000018420	0.56474424	####	###	PGP	protein_coding	phosphogly	16	2211593	2214840
ENSG0000013682	-0.469316587	####	###	TOR1A	protein_coding	torsin family	9	129812942	129824244
ENSG0000025395	1.629821583	####	###	HMGN1P38	processed_pse	high mobilit	15	92711808	92712110
ENSG0000023460	0.682935637	####	###	MAPKAPK5	lncRNA	MAPKAPK5	12	111839764	111842902
ENSG0000015043	0.647601324	####	###	TMEM218	protein_coding	transmembr	11	125096545	125111763
ENSG0000019760	0.957700645	####	###	ZNF841	protein_coding	zinc finger p	19	52064466	52095765
ENSG0000013602	0.321656076	####	###	SCYL2	protein_coding	SCY1 like ps	12	100267140	100341715
ENSG0000013034	-0.458975483	####	###	QRSL1	protein_coding	glutaminyl-	6	106629578	106668417
ENSG0000021374	1.348583347	####	###	ZNF337-AS	lncRNA	ZNF337 ant	20	25624045	25689032
ENSG0000003386	0.505382668	####	###	SLC4A7	protein_coding	solute carrie	3	27372721	27484420
ENSG0000018215	-0.247387137	####	###	CREB3L2	protein_coding	cAMP respo	7	137874979	138002086
ENSG0000025863	0.545536145	####	###	AL160006.1	lncRNA	novel transc	1	110058340	110062555
ENSG0000000683	0.300633807	####	###	ADIPOR2	protein_coding	adiponectin	12	1688574	1788674
ENSG0000012993	-0.787541596	####	###	DOHH	protein_coding	deoxyhypus	19	3490821	3500674
ENSG0000009538	-0.459308064	####	###	NANS	protein_coding	N-acetylneu	9	98056732	98083077
ENSG0000015650	1.463000081	####	###	FAM122C	protein_coding	family with s	X	134796395	134854835
ENSG0000017327	0.235588103	####	###	TNKS	protein_coding	tankyrase [S	8	9555912	9782346
ENSG0000014684	0.46221814	####	###	TMEM209	protein_coding	transmembr	7	130164713	130207770
ENSG0000025908	2.772552281	####	###	AL137779.2	lncRNA	novel transc	14	101796555	101810321
ENSG0000027364	-0.642732486	####	###	KBTBD11	protein_coding	kelch repeat	CHR_HSCHR	1973878	2006936
ENSG0000026879	-2.993517362	####	###	AC027307.3	lncRNA	novel transc	19	1440839	1441938
ENSG0000013533	1.036099656	####	###	LCA5	protein_coding	lebercilin LC	6	79484991	79537458
ENSG0000027294	-1.808578838	####	###	NA	NA	NA	NA	NA	NA
ENSG0000023453	-1.671188229	####	###	NFKBIL1	protein_coding	NFKB inhibit	CHR_HSCHR	31534315	31546261
ENSG0000010228	3.25796131	####	###	GABRE	protein_coding	gamma-am	X	151953124	151974680
ENSG0000010159	0.355345033	####	###	SMCHD1	protein_coding	structural m	18	2655726	2805017
ENSG0000016580	0.537158657	####	###	ARHGEF40	protein_coding	Rho guanine	14	21070273	21090248
ENSG0000014459	-0.63941004	####	###	GMPPA	protein_coding	GDP-manno	2	219498884	219506989
ENSG0000015458	0.397943729	####	###	ELOC	protein_coding	elongin C [S	8	73939169	73972287
ENSG0000013783	1.358188071	####	###	UACA	protein_coding	uveal autoa	15	70654554	70763558
ENSG0000017705	0.549685544	####	###	ZDHHC13	protein_coding	zinc finger D	11	19117099	19176422
ENSG0000016011	-1.799651776	####	###	ANKLE1	protein_coding	ankyrin repe	19	17281645	17287646
ENSG0000016949	-1.312213643	####	###	PLEKHA2	protein_coding	pleckstrin ho	8	38901235	38973912
ENSG0000018330	0.893256101	####	###	TMEM121B	protein_coding	transmembr	22	17116297	17121367
ENSG0000017242	0.570559356	####	###	COPS9	protein_coding	COP9 signa	2	240126563	240136807
ENSG0000018080	-0.897619308	####	###	HOXC9	protein_coding	homeobox C	12	53994895	54003337
ENSG0000018578	0.212365192	####	###	MORF4L1	protein_coding	mortality fac	15	78810487	78898139
ENSG0000014040	0.524043567	####	###	TLNRD1	protein_coding	talin rod do	15	81000923	81005788
ENSG0000008477	-0.284044343	####	###	CAD	protein_coding	carbamoyl-	2	27217369	27243943
ENSG0000019848	1.409851908	####	###	ZNF808	protein_coding	zinc finger p	19	52527652	52564464
ENSG0000015509	-0.427834081	####	###	PIP4P2	protein_coding	phosphatidy	8	90993802	91040872
ENSG0000018929	0.955579322	####	###	ZKSCAN3	protein_coding	zinc finger w	6	28349947	28369172
ENSG0000016888	0.499909695	####	###	C2orf68	protein_coding	chromosom	2	85605254	85612066
ENSG0000002176	-0.945793542	####	###	OSBPL5	protein_coding	oxysterol bi	11	3087107	3166739

ENSG0000018702	1.40698199	####	###	PTRH1	protein_coding	peptidyl-tRI	9	127690348	127724873
ENSG0000012803	0.688835434	####	###	SRD5A3	protein_coding	steroid 5 alp	4	55346242	55373100
ENSG0000010302	1.400623953	####	###	CCDC113	protein_coding	coiled-coil c	16	58231157	58283836
ENSG0000010131	-0.338976878	####	###	SEC23B	protein_coding	SEC23 hom	20	18507520	18561415
ENSG0000019840	0.900180935	####	###	NTRK1	protein_coding	neurotrophi	1	156815640	156881850
ENSG0000014468	-0.329756125	####	###	STAC	protein_coding	SH3 and cys	3	36380344	36548007
ENSG0000019896	-0.557078228	####	###	ARMCX6	protein_coding	armadillo re	X	101615118	101618001
ENSG0000017911	-5.828800283	####	###	HES7	protein_coding	hes family b	17	8120592	8124106
ENSG0000017795	0.546948799	####	###	BET1L	protein_coding	Bet1 golgi v	11	167784	207428
ENSG0000016056	0.485228658	####	###	MED27	protein_coding	mediator cc	9	131852928	132079867
ENSG0000023322	-1.863861903	####	###	AC016876.2	lncRNA	novel transc	17	7581964	7584086
ENSG0000025964	1.044978972	####	###	ST20-AS1	lncRNA	ST20 antiser	15	79922771	79926993
ENSG0000014446	-3.123386376	####	###	NYAP2	protein_coding	neuronal tyr	2	225399710	225654018
ENSG0000013239	0.593570506	####	###	EEFSEC	protein_coding	eukaryotic e	3	128153481	128408646
ENSG0000018251	0.442347002	####	###	GLRX5	protein_coding	glutaredoxir	14	95533503	95544724
ENSG0000011032	0.172304912	####	###	EIF4G2	protein_coding	eukaryotic t	11	10797050	10808940
ENSG0000011938	-0.328838347	####	###	PTPA	protein_coding	protein pho	9	129110950	129148946
ENSG0000015189	0.28147354	####	###	CACUL1	protein_coding	CDK2 assoc	10	118674167	118755249
ENSG0000018907	-0.939559123	####	###	TMEM120A	protein_coding	transmembr	7	75986831	75994656
ENSG0000027607	3.636863593	####	###	NA	NA	NA	NA	NA	NA
ENSG0000012088	-0.75172245	####	###	TNFRSF10B	protein_coding	TNF recepto	8	23020133	23069031
ENSG0000024101	1.838748657	####	###	TPM3P9	transcribed_pr	tropomyosir	19	53431984	53444670
ENSG0000014675	0.600883396	####	###	ZNF92	protein_coding	zinc finger p	7	65373799	65401136
ENSG0000019685	0.379296646	####	###	PPTC7	protein_coding	protein pho	12	110533245	110583318
ENSG0000013668	0.4173895	####	###	CBWD2	protein_coding	COBW dom	2	113437691	113496204
ENSG0000014004	0.577790455	####	###	PTGR2	protein_coding	prostagland	14	73851844	73886827
ENSG0000015397	-1.489669366	####	###	HS3ST3A1	protein_coding	heparan sul	17	13494032	13601929
ENSG0000016075	-0.278058437	####	###	FDPS	protein_coding	farnesyl dipl	1	155308748	155320666
ENSG0000018941	-3.82114094	####	###	SH2D5	protein_coding	SH2 domair	1	20719731	20732837
ENSG0000015134	0.36693565	####	###	EXT2	protein_coding	exostosin gl	11	44095673	44251962
ENSG0000027471	-1.549759807	####	###	AC005332.4	transcribed_un	bromodoma	17	68205489	68207493
ENSG0000010633	0.945137589	####	###	MOSPD3	protein_coding	motile sperr	7	100612102	100615384
ENSG0000011876	0.371110912	####	###	PKD2	protein_coding	polycystin 2	4	88007635	88077777
ENSG0000016556	1.352014958	####	###	AKR1E2	protein_coding	aldo-keto re	10	4786629	4848062
ENSG0000018831	-1.321740784	####	###	C3orf62	protein_coding	chromosom	3	49268596	49277232
ENSG0000014808	-0.621017186	####	###	SHC3	protein_coding	SHC adaptc	9	89005771	89178818
ENSG0000012121	0.787430641	####	###	MND1	protein_coding	meiotic nuc	4	153344649	153415118
ENSG0000017226	0.593967836	####	###	NEGR1	protein_coding	neuronal gr	1	71395943	72282539
ENSG0000018110	-0.632964859	####	###	F2R	protein_coding	coagulation	5	76716126	76735770
ENSG0000024022	0.787228134	####	###	ZNF542P	transcribed_un	zinc finger p	19	56368099	56379828
ENSG0000025434	-2.421889898	####	###	AC091563.1	lncRNA	novel transc	8	120052180	120056201
ENSG0000011705	0.435919506	####	###	ACADM	protein_coding	acyl-CoA de	1	75724347	75787575
ENSG0000008836	0.562280415	####	###	EPB41L1	protein_coding	erythrocyte	20	36091504	36232799
ENSG0000027728	1.523507906	####	###	AC004812.2	lncRNA	novel transc	12	120116907	120119000
ENSG0000013137	0.652097534	####	###	SH3BP5	protein_coding	SH3 domair	3	15254353	15341368
ENSG0000014386	-0.534853968	####	###	ARL8A	protein_coding	ADP ribosyl.	1	202133404	202144743
ENSG0000016692	-0.749748441	####	###	SCG5	protein_coding	secretogran	15	32641676	32697098
ENSG0000011627	-0.561946854	####	###	PHF13	protein_coding	PHD finger	1	6613731	6624030
ENSG0000011615	1.125399891	####	###	MORN1	protein_coding	MORN repe	1	2321253	2391707
ENSG0000010882	-4.017303161	####	###	PTGES3L-A	protein_coding	PTGES3L-A	17	42950526	42980528
ENSG0000027557	-0.865752681	####	###	PTP4A3	protein_coding	protein tyro	CHR_HSCHR	141391991	141438356
ENSG0000008206	0.480797964	####	###	WDR70	protein_coding	WD repeat c	5	37379285	37753435
ENSG0000023509	1.947257038	####	###	ID2-AS1	lncRNA	ID2 antisens	2	8666636	8681864
ENSG0000018425	-1.524344709	####	###	NA	NA	NA	NA	NA	NA
ENSG0000027883	2.175989782	####	###	AC073508.3	lncRNA	novel transc	17	40648300	40649718
ENSG0000013913	-2.052088183	####	###	FGD4	protein_coding	FYVE, RhoG	12	32399529	32646050
ENSG0000010702	0.554583415	####	###	TBC1D13	protein_coding	TBC1 doma	9	128787253	128810430
ENSG0000004816	-0.430644086	####	###	NOP16	protein_coding	NOP16 nucl	5	176383938	176388927

ENSG0000012869	-0.735883694	####	###	EIF2S2P4	processed_pse	eukaryotic t	2	170751805	170752788
ENSG0000013547	0.442480925	####	###	PAN2	protein_coding	poly(A) spec	12	56316223	56334053
ENSG0000016679	-0.504301444	####	###	CIAO2A	protein_coding	cytosolic iro	15	64072565	64093857
ENSG0000015733	-3.431803087	####	###	C1orf158	protein_coding	chromosom	1	12746200	12763699
ENSG0000014375	-0.363993548	####	###	FBXO28	protein_coding	F-box prote	1	224114111	224162047
ENSG0000024030	0.56757987	####	###	ACAD11	protein_coding	acyl-CoA de	3	132558138	132660082
ENSG0000025978	0.392056685	####	###	HMGB1P6	processed_pse	high mobilit	15	71164770	71165415
ENSG0000012719	-0.663475601	####	###	TRAF2	protein_coding	TNF recepto	9	136881912	136926607
ENSG0000010071	-0.265713841	####	###	MTHFD1	protein_coding	methylenete	14	64388031	64463457
ENSG0000023499	2.400459036	####	###	AC098934.	transcribed_pr	actin, gamm	1	202861754	202875241
ENSG0000014075	0.417658909	####	###	ARHGAP17	protein_coding	Rho GTPase	16	24919389	25015666
ENSG0000010184	0.956021621	####	###	ATG4A	protein_coding	autophagy r	X	108091668	108154671
ENSG0000016571	0.717047357	####	###	BORCS5	protein_coding	BLOC-1 rela	12	12357078	12471233
ENSG0000019813	0.653695439	####	###	ZNF544	protein_coding	zinc finger p	19	58228594	58277495
ENSG0000013919	-0.457531031	####	###	PEX5	protein_coding	peroxisoma	12	7188685	7218574
ENSG0000019618	-0.378738858	####	###	STK40	protein_coding	serine/threo	1	36339624	36385924
ENSG0000019891	-0.206031013	####	###	SREBF2	protein_coding	sterol regula	22	41833079	41907307
ENSG0000007968	-0.581039559	####	###	SCGN	protein_coding	secretagogi	6	25652201	25701783
ENSG0000022840	-0.918491357	####	###	CCT6P1	transcribed_un	chaperonin	7	65751142	65763354
ENSG0000007001	-0.28923427	####	###	LRP6	protein_coding	LDL recepto	12	12116025	12267044
ENSG0000016520	-0.419275178	####	###	STRBP	protein_coding	spermatid p	9	123109500	123268586
ENSG0000010059	-3.092122588	####	###	RIN3	protein_coding	Ras and Rab	14	92513781	92688994
ENSG0000028043	1.619649148	####	###	AC006058.	TEC	TEC	3	44115818	44117487
ENSG0000014361	-0.366638787	####	###	GATAD2B	protein_coding	GATA zinc f	1	153789030	153923360
ENSG0000023392	-1.636778686	####	###	LINC01694	lncRNA	long interge	21	45593654	45603088
ENSG0000017354	-0.466181595	####	###	ZNF622	protein_coding	zinc finger p	5	16451519	16465800
ENSG0000011729	0.324160641	####	###	ECE1	protein_coding	endothelin c	1	21217247	21345572
ENSG0000018577	-1.172170696	####	###	KCNIP4	protein_coding	potassium v	4	20728606	21948772
ENSG0000016017	1.211566117	####	###	FAM86C2P	transcribed_un	family with s	11	67791648	67805336
ENSG0000011494	0.538545989	####	###	ADAM23	protein_coding	ADAM meta	2	206443532	206621127
ENSG0000013878	0.433009247	####	###	GSTCD	protein_coding	glutathione	4	105708778	105847725
ENSG0000000103	-0.636025846	####	###	FUCA2	protein_coding	alpha-L-fuc	6	143494812	143511720
ENSG0000014148	-0.848337591	####	###	ARRB2	protein_coding	arrestin beta	17	4710596	4721499
ENSG0000014413	0.987386912	####	###	RABL2A	protein_coding	RAB, memb	2	113627229	113643396
ENSG0000016648	0.401379528	####	###	WEE1	protein_coding	WEE1 G2 ch	11	9573670	9593457
ENSG0000012355	0.521734685	####	###	USP45	protein_coding	ubiquitin sp	6	99432325	99521728
ENSG0000025003	-1.608968224	####	###	SLC7A11-A	lncRNA	SLC7A11 an	4	138057464	138178177
ENSG0000016526	0.453932058	####	###	NDUFB6	protein_coding	NADH:ubiqu	9	32553001	32573184
ENSG0000025967	-1.064652725	####	###	IQCH-AS1	lncRNA	IQCH antise	15	67290636	67521844
ENSG0000020344	-7.743671741	####	###	AC004988.	lncRNA	novel transc	7	40538127	40546928
ENSG0000001102	-0.462170109	####	###	CLCN6	protein_coding	chloride vol	1	11806096	11848079
ENSG0000014156	0.397805553	####	###	NARF	protein_coding	nuclear prel	17	82458180	82490537
ENSG0000016683	-2.293149068	####	###	RBPMS2	protein_coding	RNA binding	15	64739891	64775589
ENSG0000017537	0.413533643	####	###	EIF1AD	protein_coding	eukaryotic t	11	65996545	66002176
ENSG0000014954	0.498192666	####	###	B3GAT3	protein_coding	beta-1,3-gl	11	62615296	62622154
ENSG0000014222	-1.771402121	####	###	EMP3	protein_coding	epithelial m	19	48321509	48330553
ENSG0000014754	-0.235425252	####	###	NSD3	protein_coding	nuclear rece	8	38269704	38382272
ENSG0000017474	0.467763041	####	###	BRMS1	protein_coding	BRMS1 tran	11	66337333	66345125
ENSG0000017659	-0.643509538	####	###	KBTBD11	protein_coding	kelch repea	8	1973677	2006936
ENSG0000011666	-5.773689958	####	###	FBXO2	protein_coding	F-box prote	1	11637018	11655785
ENSG0000018066	-0.476949418	####	###	YOD1	protein_coding	YOD1 deub	1	207043849	207052980
ENSG0000007518	0.565287361	####	###	NUP37	protein_coding	nucleoporin	12	102073103	102120120
ENSG0000016667	-1.66609464	####	###	TVP23A	protein_coding	trans-golgi	16	10760919	10818794
ENSG0000009209	0.592405978	####	###	OSGEP	protein_coding	O-sialoglyco	14	20446401	20455089
ENSG0000008893	0.233370219	####	###	XRN2	protein_coding	5'-3' exorib	20	21303331	21389825
ENSG0000017555	0.460687701	####	###	DRAP1	protein_coding	DR1 associa	11	65919274	65921563
ENSG0000019753	0.673480706	####	###	MIB2	protein_coding	mindbomb	1	1615415	1630610
ENSG0000000507	0.564530221	####	###	POLR2J	protein_coding	RNA polyme	7	102473118	102478907

ENSG0000017788	0.324639569	####	###	GRB2	protein_coding	growth facto	17	75318076	75405709
ENSG0000008915	0.493011576	####	###	PXN	protein_coding	paxillin [Sou	12	120210439	120265771
ENSG0000021302	-2.782610051	####	###	SYT3	protein_coding	synaptotagr	19	50621307	50639881
ENSG0000025983	-3.084598168	####	###	AL365361.1	lncRNA	novel transc	1	110653560	110657040
ENSG0000017767	0.473087387	####	###	SRRM3	protein_coding	serine/argin	7	76201896	76287288
ENSG0000019817	-0.589148015	####	###	DDRGK1	protein_coding	DDRGK don	20	3190350	3204685
ENSG0000010155	0.337161092	####	###	USP14	protein_coding	ubiquitin sp	18	158383	214629
ENSG0000008522	-0.206427076	####	###	ATRX	protein_coding	ATRX chrom	X	77504880	77786233
ENSG0000013743	2.264233588	####	###	C6orf52	protein_coding	chromosom	6	10671418	10694797
ENSG0000010992	0.281388572	####	###	SC5D	protein_coding	sterol-C5-d	11	121292681	121313410
ENSG0000012777	0.934122574	####	###	EMC6	protein_coding	ER membra	17	3668812	3669668
ENSG0000019769	-1.917059935	####	###	NMB	protein_coding	neuromedir	15	84655129	84658563
ENSG0000017370	0.464603422	####	###	HEG1	protein_coding	heart develo	3	124965710	125055997
ENSG0000016258	0.615382076	####	###	FAAP20	protein_coding	FA core con	1	2184461	2212720
ENSG0000027356	0.821749534	####	###	NAP1L4	protein_coding	nucleosome	CHR_HSCHR	2943292	2991260
ENSG0000010678	-0.509812497	####	###	CORO2A	protein_coding	coronin 2A	9	98120975	98192637
ENSG0000023556	1.592970992	####	###	AC002310.1	lncRNA	novel transc	16	30534752	30537149
ENSG0000019736	0.971892404	####	###	ZNF517	protein_coding	zinc finger p	8	144798876	144811169
ENSG0000019755	-0.365552867	####	###	SIPA1L1	protein_coding	signal induc	14	71320449	71741229
ENSG0000010319	0.292063828	####	###	USP10	protein_coding	ubiquitin sp	16	84699986	84779922
ENSG0000024430	-0.435257617	####	###	DUXAP10	transcribed_pr	double hom	14	19268853	19337730
ENSG0000013266	-1.711264139	####	###	RIN2	protein_coding	Ras and Rab	20	19757606	20002459
ENSG0000018834	0.496930855	####	###	CIBAR1	protein_coding	CBY1 intera	8	93698561	93731527
ENSG0000013713	0.769112722	####	###	HINT2	protein_coding	histidine tria	9	35812960	35815354
ENSG0000017226	-0.375301475	####	###	ZNF131	protein_coding	zinc finger p	5	43065176	43192021
ENSG0000021408	0.542503921	####	###	ARL16	protein_coding	ADP ribosyl	17	81681174	81683924
ENSG0000027583	-0.420238557	####	###	SMARCB1	protein_coding	SWI/SNF rel	CHR_HSCHR	23786963	23834516
ENSG0000018635	0.557581403	####	###	RXRA	protein_coding	retinoid X re	9	134317098	134440585
ENSG0000010319	2.498895766	####	###	CRISPLD2	protein_coding	cysteine rich	16	84819985	84920768
ENSG0000011517	-0.426095089	####	###	ACVR1	protein_coding	activin A rec	2	157736444	157876330
ENSG0000009187	-0.880147893	####	###	ANGPT2	protein_coding	angiopoietir	8	6499632	6563409
ENSG0000013019	-0.699808319	####	###	THEM6	protein_coding	thioesterase	8	142727223	142736927
ENSG0000010022	0.353225204	####	###	JOSD1	protein_coding	Josephin do	22	38685543	38701556
ENSG0000023510	0.708441126	####	###	BRD3OS	protein_coding	BRD3 oppos	9	134025481	134034666
ENSG0000027720	0.942834698	####	###	F8A1	protein_coding	coagulation	X	154886349	154888061
ENSG0000010524	-0.742391586	####	###	YJU2	protein_coding	YJU2 splicin	19	4247080	4269088
ENSG0000013220	1.388756145	####	###	SLX1A	protein_coding	SLX1 homol	16	30193875	30197561
ENSG0000018428	-0.515774041	####	###	TSSC4	protein_coding	tumor supp	11	2400488	2403878
ENSG0000011971	-0.470506908	####	###	EIF2B2	protein_coding	eukaryotic t	14	75002921	75012366
ENSG0000016876	-1.294302611	####	###	GSTM4	protein_coding	glutathione	1	109656099	109674836
ENSG0000012819	0.367736372	####	###	DGCR8	protein_coding	DGCR8 micr	22	20080232	20111877
ENSG0000017234	-1.664311189	####	###	CSDC2	protein_coding	cold shock c	22	41561010	41577741
ENSG0000016905	-0.313755789	####	###	MECP2	protein_coding	methyl-CpG	X	154021573	154137103
ENSG0000012820	-1.729691412	####	###	ASPHD2	protein_coding	aspartate be	22	26429260	26445015
ENSG0000018451	0.50595131	####	###	ZFP1	protein_coding	ZFP1 zinc fir	16	75148494	75172236
ENSG0000019801	7.238281181	####	###	FCGR1B	protein_coding	Fc fragment	1	121087345	121097161
ENSG0000018589	0.251700083	####	###	LAMP1	protein_coding	lysosomal a	13	113297239	113323672
ENSG0000016044	1.084834306	####	###	ZDHHC12	protein_coding	zinc finger D	9	128720870	128724127
ENSG0000018285	-0.616006308	####	###	ALG12	protein_coding	ALG12 alpha	22	49900229	49918438
ENSG0000010629	0.428770227	####	###	TAF6	protein_coding	TATA-box b	7	100107070	100119841
ENSG0000011165	0.569437014	####	###	COPS7A	protein_coding	COP9 signa	12	6724014	6731875
ENSG0000007477	-4.476219283	####	###	NOX3	protein_coding	NADPH oxid	6	155395368	155455839
ENSG0000013717	-0.603864373	####	###	KIF13A	protein_coding	kinesin fami	6	17759183	17987635
ENSG0000017185	-0.361283297	####	###	RPS21	protein_coding	ribosomal p	20	62387103	62388520
ENSG0000000657	0.30138916	####	###	PHTF2	protein_coding	putative hor	7	77798792	77957503
ENSG0000012168	0.802414437	####	###	PEX16	protein_coding	peroxisoma	11	45909663	45918812
ENSG0000023390	-5.064452111	####	###	LINC01761	lncRNA	long interge	1	95474737	95479356
ENSG0000024886	2.462517302	####	###	USP46-DT	lncRNA	USP46 diver	4	52659406	52661668

ENSG0000016857	-0.437480118	####	###	SLC20A2	protein_coding	solute carrie	8	42416475	42541926
ENSG0000010305	0.775984152	####	###	SMPD3	protein_coding	sphingomye	16	68358327	68448508
ENSG0000016413	-1.40726465	####	###	IL15	protein_coding	interleukin 1	4	141636583	141733987
ENSG0000014768	-0.303140549	####	###	NDUFB9	protein_coding	NADH:ubiqu	8	124539101	124580648
ENSG0000022880	1.301320057	####	###	AC064807.1	lncRNA	novel transc	8	51899268	51949874
ENSG0000012372	-0.435209248	####	###	RAP2C	protein_coding	RAP2C, mer	X	132203024	132219480
ENSG0000009044	-0.730815935	####	###	TFAP4	protein_coding	transcription	16	4257186	4273075
ENSG0000007692	-0.617445893	####	###	ARHGEF1	protein_coding	Rho guanin	19	41883173	41930150
ENSG0000027141	-2.792213722	####	###	NA	NA	NA	NA	NA	NA
ENSG0000016691	0.206861883	####	###	YWHAB	protein_coding	tyrosine 3-r	20	44885702	44908532
ENSG0000005658	0.220157317	####	###	RC3H2	protein_coding	ring finger a	9	122844556	122905359
ENSG0000027087	0.832750527	####	###	SRGAP2D	unprocessed_p	SLIT-ROBO	1	143975087	144068350
ENSG0000007036	0.604390006	####	###	SMG6	protein_coding	SMG6 nonse	17	2059839	2303785
ENSG0000010907	0.533008519	####	###	TNFAIP1	protein_coding	TNF alpha in	17	28335602	28347009
ENSG0000014268	-0.793304011	####	###	ZNF593	protein_coding	zinc finger p	1	26169908	26170873
ENSG0000015413	-1.45388737	####	###	ROBO3	protein_coding	roundabout	11	124865432	124881471
ENSG0000004954	0.531544471	####	###	RFC2	protein_coding	replication f	7	74231499	74254458
ENSG0000017519	0.676849146	####	###	PCCA	protein_coding	propionyl-C	13	100089015	100530437
ENSG0000016393	-0.31882065	####	###	GNL3	protein_coding	G protein nu	3	52681156	52694497
ENSG0000007808	-2.216702045	####	###	LAMP3	protein_coding	lysosomal a	3	183122215	183163839
ENSG0000011523	0.350787366	####	###	PSMD14	protein_coding	proteasome	2	161308425	161411717
ENSG0000025857	-7.662557573	####	###	AL163195.2	lncRNA	novel transc	14	20587644	20607221
ENSG0000022198	-1.056208862	####	###	PPT2	protein_coding	palmitoyl-p	6	32153441	32163678
ENSG0000018822	-0.689501171	####	###	ZNF793	protein_coding	zinc finger p	19	37506939	37548762
ENSG0000010061	0.462484072	####	###	DHRS7	protein_coding	dehydroger	14	60144119	60169856
ENSG0000020535	0.596199361	####	###	PRR13	protein_coding	proline rich	12	53441678	53446645
ENSG0000017895	-0.633559816	####	###	ZBTB7A	protein_coding	zinc finger a	19	4043303	4066899
ENSG0000010991	0.312084431	####	###	MTCH2	protein_coding	mitochondr	11	47617315	47642607
ENSG0000013637	0.437422649	####	###	ADAMTS7	protein_coding	ADAM meta	15	78759206	78811464
ENSG0000011214	0.397130443	####	###	FBXO9	protein_coding	F-box prote	6	53051991	53100873
ENSG0000006272	-0.315714955	####	###	APPBP2	protein_coding	amyloid bet	17	60443158	60526242
ENSG0000025759	1.654885164	####	###	GALNT4	protein_coding	polypeptide	12	89519412	89524796
ENSG0000017246	-0.291081489	####	###	ZNF24	protein_coding	zinc finger p	18	35332227	35345482
ENSG0000027360	-0.802756752	####	###	EPOP	protein_coding	elongin BC a	17	38671703	38674957
ENSG0000014609	-1.296321597	####	###	DOK3	protein_coding	docking pro	5	177501907	177511274
ENSG0000026997	-1.067991256	####	###	AC010969.2	lncRNA	novel transc	2	9936360	9939590
ENSG0000019805	0.49290422	####	###	PRIM1	protein_coding	DNA primas	12	56731296	56752374
ENSG0000010634	-0.504241247	####	###	IMPDH1	protein_coding	inosine mor	7	128392277	128410252
ENSG0000027676	-0.721197436	####	###	CWC25	protein_coding	CWC25 splic	CHR_HSCHR	38601221	38626268
ENSG0000018307	7.668077351	####	###	NKX2-5	protein_coding	NK2 homeo	5	173232109	173235311
ENSG0000017018	0.346098532	####	###	USP38	protein_coding	ubiquitin sp	4	143184917	143223874
ENSG0000014397	0.450771271	####	###	ETAA1	protein_coding	ETAA1 activ	2	67397322	67412089
ENSG0000019671	0.377314626	####	###	VKORC1L1	protein_coding	vitamin K ep	7	65873074	65959563
ENSG0000012656	-2.459121015	####	###	STAT5A	protein_coding	signal transc	17	42287547	42311943
ENSG0000018274	-1.194648134	####	###	SLC35D3	protein_coding	solute carrie	6	136922301	136925660
ENSG0000010110	0.32057869	####	###	STK4	protein_coding	serine/threo	20	44966479	45080021
ENSG0000006597	0.551002462	####	###	FOXJ2	protein_coding	forkhead bo	12	8032716	8055517
ENSG0000008527	-0.447871053	####	###	MYNN	protein_coding	myoneurin [	3	169772831	169789716
ENSG0000013843	0.442402604	####	###	FAM117B	protein_coding	family with s	2	202634969	202769757
ENSG0000017768	-0.357819724	####	###	THAP5	protein_coding	THAP doma	7	108554543	108569750
ENSG0000027256	-5.037722978	####	###	AC005162.2	lncRNA	novel transc	7	28979967	29013367
ENSG0000013681	0.546385466	####	###	TOR1B	protein_coding	torsin family	9	129803157	129811281
ENSG0000025107	2.311251851	####	###	BMS1P2	transcribed_un	BMS1 pseud	10	47538377	47551954
ENSG0000012524	0.289324272	####	###	RAP2A	protein_coding	RAP2A, mer	13	97434169	97469128
ENSG0000018547	0.695170772	####	###	TMEM179B	protein_coding	transmembr	11	62787402	62790400
ENSG0000016387	0.241917833	####	###	YEATS2	protein_coding	YEATS dom	3	183697797	183812624
ENSG0000016547	0.309258883	####	###	REEP3	protein_coding	receptor acc	10	63521401	63625128
ENSG0000018744	-0.308277817	####	###	CHP1	protein_coding	calcineurin l	15	41230839	41281887

ENSG0000007284	-0.510561081	####	###	DERL2	protein_coding	derlin 2 [Sou	17	5471254	5486811
ENSG0000014193	-0.704332405	####	###	TPGS1	protein_coding	tubulin poly	19	507497	519654
ENSG0000017773	0.530701244	####	###	FLII	protein_coding	FLII actin re	17	18244815	18258738
ENSG0000014577	1.238710788	####	###	TNFAIP8	protein_coding	TNF alpha i	5	119268692	119399688
ENSG0000022350	1.291932101	####	###	RPL23AP53	transcribed_pr	ribosomal p	8	213186	232231
ENSG0000012788	0.455980853	####	###	ECHS1	protein_coding	enoyl-CoA	10	133362485	133373354
ENSG0000016763	0.645209794	####	###	TRAPPC9	protein_coding	trafficking p	8	139727725	140458579
ENSG0000007973	-0.790391185	####	###	PGM1	protein_coding	phosphoglu	1	63593411	63660245
ENSG0000027730	-0.772502063	####	###	EPOP	protein_coding	elongin BC	CHR_HSCHR	38472490	38476208
ENSG0000016332	0.542814241	####	###	ABRAXAS1	protein_coding	abraxas 1, B	4	83459517	83523348
ENSG0000006465	0.416475841	####	###	SLC12A2	protein_coding	solute carrie	5	128083766	128189677
ENSG0000011402	0.851745657	####	###	OGG1	protein_coding	8-oxoguani	3	9749944	9788219
ENSG0000019636	0.405622505	####	###	NUDT11	protein_coding	nudix hydro	X	51490011	51496592
ENSG0000005352	-1.062766623	####	###	MCF2L2	protein_coding	MCF.2 cell li	3	183178043	183428778
ENSG0000021353	0.861209974	####	###	STIMATE	protein_coding	STIM activa	3	52836219	52897548
ENSG0000022695	-0.412783839	####	###	DANCR	lncRNA	differentiati	4	52712325	52723623
ENSG0000010918	-0.269625964	####	###	OCIAD1	protein_coding	OCIA doma	4	48805212	48861817
ENSG0000010866	-0.347847843	####	###	CYTH1	protein_coding	cytohesin 1	17	78674048	78782297
ENSG0000011114	-0.281968232	####	###	LTA4H	protein_coding	leukotriene	12	96000753	96043520
ENSG0000012492	-0.687947232	####	###	MYRF	protein_coding	myelin regu	11	61752636	61788518
ENSG0000016991	0.53098663	####	###	GUSB	protein_coding	glucuronida	7	65960684	65982215
ENSG0000019721	0.315593838	####	###	ENTPD4	protein_coding	ectonucleos	8	23385783	23457695
ENSG0000010316	0.427911079	####	###	HSDL1	protein_coding	hydroxyster	16	84122141	84145192
ENSG0000019691	-0.595149103	####	###	KPNA5	protein_coding	karyopherin	6	116681187	116741867
ENSG0000013293	-1.425110075	####	###	MTUS2	protein_coding	microtubule	13	28820348	29505947
ENSG0000013943	0.397075713	####	###	GIT2	protein_coding	GIT ArfGAP	12	109929802	109996389
ENSG0000016482	-7.653557895	####	###	DEFB1	protein_coding	defensin be	8	6870592	6877936
ENSG0000017306	0.260647237	####	###	HECTD4	protein_coding	HECT doma	12	112160188	112382439
ENSG0000025447	-2.207598948	####	###	AP5B1	protein_coding	adaptor rela	11	65773898	65780976
ENSG0000008344	-0.346192229	####	###	PLOD1	protein_coding	procollagen	1	11934205	11975538
ENSG0000010488	0.706309041	####	###	ARHGEF18	protein_coding	Rho/Rac gu	19	7395113	7472484
ENSG0000010722	0.299353334	####	###	EDF1	protein_coding	endothelial	9	136862119	136866308
ENSG0000022904	2.069784571	####	###	AC091729.	lncRNA	novel transc	7	1160374	1165607
ENSG0000010883	-1.308866919	####	###	RND2	protein_coding	Rho family	17	43025231	43032041
ENSG0000027615	-1.670462098	####	###	MAPT	protein_coding	microtubule	CHR_HSCHR	46069784	46203150
ENSG0000015874	-4.317980326	####	###	HTR6	protein_coding	5-hydroxytr	1	19664875	19680966
ENSG0000015536	0.721066362	####	###	MOV10	protein_coding	Mov10 RISC	1	112673141	112700746
ENSG0000015533	0.525321415	####	###	C16orf87	protein_coding	chromosom	16	46796603	46831180
ENSG0000024646	1.542496064	####	###	AC138904.	lncRNA	novel transc	16	28284885	28292064
ENSG0000011508	0.499105116	####	###	SLC35F5	protein_coding	solute carrie	2	113705011	113756693
ENSG0000026224	0.909280949	####	###	CORO7	protein_coding	coronin 7 [S	16	4354542	4425705
ENSG0000016688	-0.373583734	####	###	PATL1	protein_coding	PAT1 homo	11	59636716	59669037
ENSG0000019872	0.274248633	####	###	LDB1	protein_coding	LIM domain	10	102106489	102120368
ENSG0000017610	0.332833003	####	###	CSTF3	protein_coding	cleavage sti	11	33077188	33162371
ENSG0000023516	0.670668885	####	###	C12orf75	protein_coding	chromosom	12	105235290	105396097
ENSG0000016022	-1.178040237	####	###	CFAP410	protein_coding	cilia and fla	21	44328944	44339402
ENSG0000014039	0.253079055	####	###	TSPAN3	protein_coding	tetraspanin	15	77041404	77083984
ENSG0000017787	0.421223404	####	###	AP3S1	protein_coding	adaptor rela	5	115841592	115914081
ENSG0000026983	1.195392752	####	###	ZNF528-AS	lncRNA	ZNF528 ant	19	52388842	52397783
ENSG0000019836	-0.265450901	####	###	SPRED2	protein_coding	sprouty rela	2	65310851	65432637
ENSG0000019636	0.405211303	####	###	WDR5	protein_coding	WD repeat	9	134135365	134159968
ENSG0000010629	0.280219133	####	###	WASL	protein_coding	WASP like a	7	123681943	123749003
ENSG0000010817	-0.209183149	####	###	ZMIZ1	protein_coding	zinc finger	10	79068966	79316528
ENSG0000005627	0.556098194	####	###	ZNF280C	protein_coding	zinc finger p	X	130202707	130268899
ENSG0000026580	0.258351013	####	###	SEC22B	protein_coding	SEC22 hom	1	120150898	120176520
ENSG0000020652	-0.376872258	####	###	HACD2	protein_coding	3-hydroxya	3	123490820	123585053
ENSG0000026937	0.597478894	####	###	AC022149.	processed_pse	integrin bet	19	14621634	14622242
ENSG0000011612	0.465335869	####	###	BCL9	protein_coding	BCL9 transc	1	147541501	147626216

ENSG0000010630	-0.434740769	####	###	AIMP2	protein_coding	aminoacyl t	7	6009255	6023834
ENSG0000019887	-0.298049185	####	###	DCAF12	protein_coding	DDB1 and C	9	34086387	34127399
ENSG0000016627	0.628752927	####	###	WBP1L	protein_coding	WW domair	10	102743948	102834516
ENSG0000004411	0.201043689	####	###	CTNNA1	protein_coding	catenin alph	5	138610967	138935034
ENSG0000011021	0.419219401	####	###	PANX1	protein_coding	pannexin 1	11	94128841	94181968
ENSG0000014710	0.89507826	####	###	SLC16A2	protein_coding	solute carrie	X	74421493	74533917
ENSG0000021537	1.403121758	####	###	MYL5	protein_coding	myosin light	4	673580	682033
ENSG0000012203	-0.458415208	####	###	GTF3A	protein_coding	general trar	13	27424619	27435823
ENSG0000018201	-0.579875047	####	###	PNMA8A	protein_coding	PNMA famil	19	46466491	46471563
ENSG0000013477	0.424445957	####	###	TPGS2	protein_coding	tubulin poly	18	36777647	36829216
ENSG0000013003	-2.920520285	####	###	CRACR2A	protein_coding	calcium rele	12	3606633	3764819
ENSG0000018565	0.267272709	####	###	UBE2L3	protein_coding	ubiquitin co	22	21549447	21624034
ENSG0000015852	0.486650925	####	###	TSR2	protein_coding	TSR2 riboso	X	54440404	54448032
ENSG0000015213	0.377494305	####	###	GPATCH11	protein_coding	G-patch do	2	37084451	37099244
ENSG0000010134	0.336818964	####	###	POFUT1	protein_coding	protein O-fu	20	32207880	32238658
ENSG0000017087	-0.350246311	####	###	KIAA0232	protein_coding	KIAA0232 [S	4	6781375	6884170
ENSG0000007078	0.511707838	####	###	EIF2B3	protein_coding	eukaryotic t	1	44850522	44986722
ENSG0000010327	-0.750339533	####	###	NUBP1	protein_coding	nucleotide b	16	10743786	10769351
ENSG0000010200	-0.375284776	####	###	SYP	protein_coding	synaptophy	X	49187815	49200199
ENSG0000014953	0.907650108	####	###	FRG1BP	unprocessed_p	FSHD regior	20	30377372	30399257
ENSG0000010877	0.358982248	####	###	RAB5C	protein_coding	RAB5C, mer	17	42124978	42155044
ENSG0000011217	-1.026792495	####	###	BMP5	protein_coding	bone morph	6	55753653	55875590
ENSG0000013627	0.545572724	####	###	HUS1	protein_coding	HUS1 check	7	47963288	47979615
ENSG0000022374	1.264044206	####	###	CCDC18-A	lncRNA	CCDC18 an	1	93262186	93346025
ENSG0000009595	-0.668924376	####	###	HIVEP1	protein_coding	HIVEP zinc f	6	12008762	12164999
ENSG0000018333	0.308118257	####	###	BCOR	protein_coding	BCL6 corep	X	40049815	40177329
ENSG0000013059	-1.666787693	####	###	SAMD10	protein_coding	sterile alpha	20	63974116	63980008
ENSG0000023136	-0.663374156	####	###	WARS2-AS	lncRNA	WARS2 anti	1	119140391	119275973
ENSG0000013524	0.376117622	####	###	PNPLA8	protein_coding	patatin like	7	108470417	108569666
ENSG0000022964	-1.286204754	####	###	NAMPTP1	processed_pse	nicotinamid	10	36521721	36524234
ENSG0000010056	0.319293797	####	###	PSMA3	protein_coding	proteasome	14	58244843	58272012
ENSG0000010056	0.379327056	####	###	VTI1B	protein_coding	vesicle trans	14	67647085	67674820
ENSG0000008546	-1.224864857	####	###	OVGP1	protein_coding	oviductal gl	1	111414319	111427735
ENSG0000017898	0.784659981	####	###	SELENOW	protein_coding	selenoprote	19	47778585	47784686
ENSG0000014836	0.801562452	####	###	PAXX	protein_coding	PAXX non-h	9	136992422	136993984
ENSG0000017919	0.178833058	####	###	ZNF664	protein_coding	zinc finger p	12	123971845	124015439
ENSG0000010376	0.269982839	####	###	RAB11A	protein_coding	RAB11A, me	15	65726054	65891989
ENSG0000017788	0.359537167	####	###	ZBTB41	protein_coding	zinc finger a	1	197153682	197201293
ENSG0000013644	-0.53792652	####	###	RSAD1	protein_coding	radical S-ac	17	50478860	50485974
ENSG0000011559	-0.904902368	####	###	WNT6	protein_coding	Wnt family r	2	218859805	218874233
ENSG0000011714	0.438366215	####	###	UAP1	protein_coding	UDP-N-ace	1	162561506	162599842
ENSG0000012148	0.509085933	####	###	TRMT1L	protein_coding	tRNA methy	1	185118101	185157072
ENSG0000010551	1.080504841	####	###	CAPS	protein_coding	calcyphosin	19	5912339	5916211
ENSG0000027210	2.218464419	####	###	AC243587.	processed_pseudogene		CHR_HSCHR	29880557	29880829
ENSG0000013407	-2.218758494	####	###	IRAK2	protein_coding	interleukin 1	3	10164919	10243745
ENSG0000013807	-0.364252862	####	###	SLC5A6	protein_coding	solute carrie	2	27199587	27212958
ENSG0000013572	-0.911318363	####	###	FHOD1	protein_coding	formin hom	16	67229387	67247481
ENSG0000019623	0.282305183	####	###	TUBB	protein_coding	tubulin beta	6	30720352	30725426
ENSG0000018154	0.840336023	####	###	FANCB	protein_coding	FA complen	X	14835961	14873069
ENSG0000014570	-1.694644599	####	###	IQGAP2	protein_coding	IQ motif cor	5	76403285	76708132
ENSG0000019668	0.401450602	####	###	TOMM7	protein_coding	translocase	7	22812628	22822852
ENSG0000015914	-0.65401019	####	###	DONSON	protein_coding	DNA replica	21	33559542	33588706
ENSG0000026136	2.624677162	####	###	AC010491.	lncRNA	novel transc	5	14661808	14664604
ENSG0000014372	-0.241613315	####	###	ACP1	protein_coding	acid phosph	2	264140	278283
ENSG0000017513	-0.391616789	####	###	SH3BP5L	protein_coding	SH3 binding	1	248810446	248825915
ENSG0000010037	0.456083926	####	###	SLC25A17	protein_coding	solute carrie	22	40769630	40819399
ENSG0000008876	-0.42312412	####	###	CRLS1	protein_coding	cardiolipin s	20	6006093	6040053
ENSG0000009981	0.909848492	####	###	CEP170B	protein_coding	centrosoma	14	104865268	104896770

ENSG0000014367	-1.336031651	####	###	MAP3K21	protein_coding	mitogen-ac	1	233327724	233385148
ENSG0000015801	-0.496019043	####	###	BABAM2	protein_coding	BRISC and E	2	27889941	28338901
ENSG0000018855	-0.236207205	####	###	NBR1	protein_coding	NBR1 autop	17	43170481	43211689
ENSG0000014591	-0.298379487	####	###	BOD1	protein_coding	biorientation	5	173607145	173616659
ENSG0000016519	-2.264174511	####	###	VEGFD	protein_coding	vascular end	X	15345596	15384413
ENSG0000019654	-0.932043091	####	###	BORCS6	protein_coding	BLOC-1 rela	17	8188345	8190180
ENSG0000012154	0.628920077	####	###	SEC22A	protein_coding	SEC22 hom	3	123201927	123274136
ENSG0000010218	0.442991354	####	###	EEA1	protein_coding	early endos	12	92770637	92929331
ENSG0000016901	-0.444601502	####	###	E2F6	protein_coding	E2F transcri	2	11444375	11466177
ENSG0000023271	-7.542350473	####	###	GAPDHP48	processed_pse	glyceraldeh	2	3688021	3688981
ENSG0000025113	-1.461207719	####	###	AF117829.1	lncRNA	novel transc	8	89585872	89757812
ENSG0000017452	1.021098587	####	###	TTC9B	protein_coding	tetratricope	19	40216058	40218399
ENSG0000023969	-7.541380191	####	###	TNFSF12	protein_coding	TNF superfa	17	7549058	7557890
ENSG0000012463	1.560879706	####	###	H2BC11	protein_coding	H2B cluster	6	27125897	27132795
ENSG0000012013	-0.17486513	####	###	PANK3	protein_coding	pantothena	5	168548495	168579368
ENSG0000007371	1.361303339	####	###	PPP2R3A	protein_coding	protein pho	3	135965728	136147894
ENSG0000007524	-0.486082955	####	###	GRAMD4	protein_coding	GRAM dom	22	46576012	46679790
ENSG0000011202	0.355801372	####	###	FBXO5	protein_coding	F-box prote	6	152970519	152983579
ENSG0000012594	-0.468794935	####	###	ZNF436	protein_coding	zinc finger p	1	23359448	23369836
ENSG0000016996	1.250747232	####	###	TMEM42	protein_coding	transmemb	3	44861904	44865670
ENSG0000003380	0.31898687	####	###	PIAS1	protein_coding	protein inhi	15	68054309	68198603
ENSG0000011645	0.385121535	####	###	WDR77	protein_coding	WD repeat	1	111439890	111449256
ENSG0000008571	0.307749254	####	###	CPNE3	protein_coding	copine 3 [S	8	86514435	86561498
ENSG0000016711	0.554899994	####	###	SLC27A4	protein_coding	solute carrie	9	128340527	128361470
ENSG0000026728	-7.574434519	####	###	AC138150.2	lncRNA	novel transc	17	45168800	45171584
ENSG0000010600	1.925689671	####	###	LFNG	protein_coding	LFNG O-fuc	7	2512529	2529177
ENSG0000018121	-2.570169188	####	###	C4orf50	protein_coding	chromosom	4	5897373	6200555
ENSG0000014047	-1.802196175	####	###	PCSK6	protein_coding	proprotein	15	101297142	101525202
ENSG0000026113	3.017847903	####	###	AL137802.2	lncRNA	novel transc	1	16514645	16515754
ENSG0000008955	-1.280146076	####	###	KCNH4	protein_coding	potassium v	17	42156891	42181142
ENSG0000008407	0.435874329	####	###	PPIE	protein_coding	peptidylpro	1	39692182	39763914
ENSG0000011433	0.291493909	####	###	ACAP2	protein_coding	ArfGAP with	3	195274745	195443044
ENSG0000017063	-0.508860394	####	###	TRABD	protein_coding	TraB domai	22	50185915	50199598
ENSG0000025805	1.572126379	####	###	AC009779.2	lncRNA	novel transc	12	55729104	55730852
ENSG0000016365	0.49872767	####	###	TIPARP	protein_coding	TCDD induc	3	156673235	156706770
ENSG0000017424	0.260000328	####	###	DDX23	protein_coding	DEAD-box	12	48829756	48852842
ENSG0000018129	2.742535954	####	###	TMEM132E	protein_coding	transmemb	17	34579487	34639318
ENSG0000018683	-7.524421572	####	###	KRT16	protein_coding	keratin 16 [S	17	41609778	41615899
ENSG0000015503	0.678985092	####	###	FBXL18	protein_coding	F-box and l	7	5431335	5513809
ENSG0000007124	-0.643051373	####	###	ING3	protein_coding	inhibitor of	7	120950763	120977216
ENSG0000012450	-0.902899837	####	###	BTN2A2	protein_coding	butyrophilin	6	26383096	26394874
ENSG0000021339	0.723629564	####	###	HAUS7	protein_coding	HAUS augm	X	153447666	153495516
ENSG0000006883	-4.185344813	####	###	RASGRP2	protein_coding	RAS guanyl	11	64726911	64745456
ENSG0000015917	-0.680852598	####	###	CSRP1	protein_coding	cysteine and	1	201483530	201509456
ENSG0000016493	-0.519839559	####	###	FZD6	protein_coding	frizzled clas	8	103298433	103332866
ENSG0000018420	0.381058175	####	###	PPP1R2	protein_coding	protein pho	3	195514428	195543386
ENSG0000015683	-0.545768556	####	###	NSMCE2	protein_coding	NSE2 (MMS	8	125091679	125367120
ENSG0000013095	-1.206528879	####	###	SLC35D2	protein_coding	solute carrie	9	96313444	96383711
ENSG0000022438	-4.925252927	####	###	PRR29	protein_coding	proline rich	17	63998351	64004305
ENSG0000012479	0.218433973	####	###	DEK	protein_coding	DEK proto-	6	18223860	18264548
ENSG0000013682	0.292688211	####	###	SMC2	protein_coding	structural m	9	104094260	104141419
ENSG0000021399	0.482450775	####	###	NAXD	protein_coding	NAD(P)HX d	13	110615460	110639993
ENSG0000027227	-2.837969614	####	###	AC092687.3	lncRNA	novel transc	2	10767875	10770058
ENSG0000021019	0.718152644	####	###	MT-TE	Mt_tRNA	mitochondr	MT	14674	14742
ENSG0000014317	-0.28046994	####	###	UCK2	protein_coding	uridine-cyti	1	165827614	165911618
ENSG0000016291	0.732271049	####	###	MRPL55	protein_coding	mitochondr	1	228106679	228109312
ENSG0000014197	-0.60428104	####	###	MVB12A	protein_coding	multivesicul	19	17405722	17433724
ENSG0000027017	0.83081946	####	###	NCBP2AS2	protein_coding	NCBP2 antis	3	196942674	196943543

ENSG0000022184	1.366925487	####	###	C2orf16	protein_coding	chromosom	2	27537386	27582721
ENSG0000011918	0.320575282	####	###	ITGB1BP1	protein_coding	integrin sub	2	9403475	9423528
ENSG0000016436	-2.29875907	####	###	TERT	protein_coding	telomerase	5	1253147	1295068
ENSG0000017243	-0.409262696	####	###	GTPBP2	protein_coding	GTP binding	6	43605316	43629264
ENSG0000022564	-7.50028092	####	###	AC064875.1	lncRNA	novel transc	2	12780593	13007029
ENSG0000016281	-0.41146163	####	###	BROX	protein_coding	BRO1 doma	1	222712553	222735196
ENSG0000013609	-1.776345004	####	###	PCDH8	protein_coding	protocadhe	13	52842889	52848641
ENSG0000027938	1.864895631	####	###	AC018665.1	TEC	TEC	17	76090308	76091669
ENSG0000014356	-0.206409997	####	###	UBAP2L	protein_coding	ubiquitin as	1	154220179	154271510
ENSG0000010052	-0.256007681	####	###	CNIH1	protein_coding	cornichon fa	14	54423561	54441391
ENSG0000012068	-0.36659006	####	###	UFM1	protein_coding	ubiquitin fol	13	38349849	38363619
ENSG0000004188	-1.551766771	####	###	PARP3	protein_coding	poly(ADP-ri	3	51942345	51948867
ENSG0000011190	-0.602124554	####	###	HDDC2	protein_coding	HD domain	6	125219962	125302078
ENSG0000013870	-0.453971097	####	###	LARP1B	protein_coding	La ribonucle	4	128061286	128222931
ENSG0000022696	0.622630351	####	###	RHEBP2	processed_pse	RHEB pseuc	10	47706203	47706802
ENSG0000025031	0.63488687	####	###	SMIM20	protein_coding	small integr	4	25861830	25929874
ENSG0000021943	-0.89570819	####	###	TAFA5	protein_coding	TAFA chem	22	48489553	48850912
ENSG0000027503	0.689856456	####	###	CCDC92	protein_coding	coiled-coil c	CHR_HSCHR	123918723	123946006
ENSG0000011031	-1.077792091	####	###	CEP126	protein_coding	centrosoma	11	101915010	102001062
ENSG0000016317	0.931449937	####	###	BOLA3	protein_coding	bolA family	2	74135400	74147912
ENSG0000021527	-1.633385784	####	###	TOMM40P2	processed_pse	TOMM40 ps	22	15854195	15855243
ENSG0000010573	-0.81526934	####	###	ZNF574	protein_coding	zinc finger p	19	42068477	42081552
ENSG0000018786	-1.39229244	####	###	PALM3	protein_coding	paralemmin	19	14053363	14062076
ENSG0000024364	1.02007276	####	###	IL10RB	protein_coding	interleukin 1	21	33266367	33310187
ENSG0000016719	0.419386938	####	###	CRK	protein_coding	CRK proto-c	17	1420689	1463162
ENSG0000009609	0.685541517	####	###	TMEM14A	protein_coding	transmembr	6	52671113	52686588
ENSG0000016525	-0.628220133	####	###	HDX	protein_coding	highly diver	X	84317874	84502479
ENSG0000027463	-1.280194121	####	###	GLIS2	protein_coding	GLIS family	CHR_HSCHR	4314761	4339597
ENSG0000010207	0.963203461	####	###	SLC25A14	protein_coding	solute carrie	X	130339888	130373361
ENSG0000021376	0.66508986	####	###	ZNF134	protein_coding	zinc finger p	19	57614233	57624724
ENSG0000019894	-0.694127665	####	###	MFAP3L	protein_coding	microfibril a	4	169986597	170033031
ENSG0000012165	0.333966144	####	###	MAPK8IP1	protein_coding	mitogen-ac	11	45885651	45906465
ENSG0000010414	0.522291195	####	###	VPS18	protein_coding	VPS18 core	15	40894450	40903975
ENSG0000007823	0.850749597	####	###	TIGAR	protein_coding	TP53 induce	12	4307763	4360028
ENSG0000018361	-0.733881617	####	###	MRPL54	protein_coding	mitochondr	19	3762682	3768575
ENSG0000010301	0.304038826	####	###	CYB5B	protein_coding	cytochrome	16	69424619	69466264
ENSG0000017538	-0.374776389	####	###	SMAD2	protein_coding	SMAD famil	18	47808957	47931146
ENSG0000016418	0.451478603	####	###	TMEM161B	protein_coding	transmembr	5	88189633	88269476
ENSG0000009284	0.332292732	####	###	AGO1	protein_coding	argonaute R	1	35869808	35930532
ENSG0000013862	0.430986522	####	###	UBL7	protein_coding	ubiquitin lik	15	74445977	74461182
ENSG0000025004	-7.533324889	####	###	AC114316.1	lncRNA	novel transc	5	92082597	92479426
ENSG0000008730	-2.607742971	####	###	NID2	protein_coding	nidogen 2 [	14	52004803	52069228
ENSG0000016434	-0.279690783	####	###	NSA2	protein_coding	NSA2 riboso	5	74766991	74780113
ENSG0000016878	0.420621337	####	###	TSPAN5	protein_coding	tetraspanin	4	98470367	98658611
ENSG0000005991	0.49174615	####	###	PSD	protein_coding	pleckstrin ar	10	102402617	102421539
ENSG0000016510	0.464553268	####	###	HGSNAT	protein_coding	heparan-alp	8	43140464	43202855
ENSG0000023810	-7.473698313	####	###	LINC02806	lncRNA	long interge	1	148295180	148297556
ENSG0000011613	0.271441013	####	###	DHCR24	protein_coding	24-dehydro	1	54849627	54887195
ENSG0000017186	0.363654865	####	###	RNASEH1	protein_coding	ribonuclease	2	3541430	3558333
ENSG0000016643	1.705348078	####	###	ZMAT1	protein_coding	zinc finger r	X	101882288	101932079
ENSG0000020426	1.570049011	####	###	COL5A2	protein_coding	collagen typ	2	189031898	189225312
ENSG0000024506	1.272920602	####	###	LINC00847	lncRNA	long interge	5	180830326	180839742
ENSG0000016032	0.933233245	####	###	ADAMTS13	protein_coding	ADAM meta	9	133414358	133459402
ENSG0000014833	-0.463579405	####	###	PTGES2	protein_coding	prostagland	9	128120693	128128462
ENSG0000013776	-0.585668763	####	###	MAP2K5	protein_coding	mitogen-ac	15	67542703	67807117
ENSG0000017812	0.477225515	####	###	NDUFV2	protein_coding	NADH:ubiqu	18	9102630	9134345
ENSG0000018255	-0.376951475	####	###	ADI1	protein_coding	acireducton	2	3497366	3519531
ENSG0000010481	0.78520348	####	###	GYS1	protein_coding	glycogen sy	19	48968130	48993310

ENSG0000013553	-1.336876094	####	###	AFG1L	protein_coding	AFG1 like AT	6	108294991	108526796
ENSG0000010991	0.676219809	####	###	ELP4	protein_coding	elongator a	11	31509755	31790324
ENSG0000016169	0.547576625	####	###	DBF4B	protein_coding	DBF4 zinc fi	17	44708608	44752264
ENSG0000002784	0.817241435	####	###	B4GALT7	protein_coding	beta-1,4-ga	5	177600132	177610330
ENSG0000014606	-0.482629386	####	###	HIGD2A	protein_coding	HIG1 hypox	5	176388751	176389761
ENSG0000017236	-1.166029316	####	###	MCRIP2	protein_coding	MAPK regul	16	636817	648474
ENSG0000025958	-2.766343159	####	###	AC015712.2	lncRNA	novel transc	15	100892343	100919391
ENSG0000017509	-4.893098503	####	###	RAG2	protein_coding	recombinati	11	36575574	36598279
ENSG0000010576	-1.403989366	####	###	CADM4	protein_coding	cell adhesio	19	43622368	43639850
ENSG0000018876	0.618563554	####	###	TMEM198	protein_coding	transmembr	2	219543663	219550595
ENSG0000016504	0.82556821	####	###	LETM2	protein_coding	leucine zipp	8	38386207	38409527
ENSG0000005082	-0.460067773	####	###	BCAR1	protein_coding	BCAR1 scaff	16	75228181	75268053
ENSG0000020395	0.573034766	####	###	RTL8A	protein_coding	retrotranspo	X	135050932	135052196
ENSG0000027958	-3.27787222	####	###	AC006963.1	TEC	TEC	X	117894632	117895683
ENSG0000007523	0.305379553	####	###	ACAT1	protein_coding	acetyl-CoA	11	108116695	108147603
ENSG0000015753	-0.419347367	####	###	VPS26C	protein_coding	VPS26 endo	21	37223420	37267919
ENSG0000027501	-1.131640378	####	###	PRAME	protein_coding	preferentiall	CHR_HSCHR	22556806	22568466
ENSG0000012655	-7.455917981	####	###	HTN1	protein_coding	histatin 1 [S	4	70050438	70058848
ENSG0000016619	1.045574843	####	###	SENP8	protein_coding	SUMO pept	15	72114258	72143692
ENSG0000014049	0.482006529	####	###	SCAMP2	protein_coding	secretory ca	15	74843730	74873365
ENSG0000013831	-1.82542078	####	###	ADAMTS14	protein_coding	ADAM meta	10	70672506	70762441
ENSG0000018276	-0.240076002	####	###	NGRN	protein_coding	neugrin, neu	15	90265659	90275778
ENSG0000002764	0.98878069	####	###	INSRR	protein_coding	insulin recep	1	156840063	156859117
ENSG0000010115	-0.310407878	####	###	NELFCD	protein_coding	negative elo	20	58981208	58995133
ENSG0000012454	0.453854875	####	###	RRP36	protein_coding	ribosomal R	6	43021623	43034156
ENSG0000011552	-0.561692174	####	###	CHST10	protein_coding	carbohydrat	2	100391860	100417668
ENSG0000006942	-0.566877541	####	###	KCNAB2	protein_coding	potassium v	1	5990927	6101193
ENSG0000014359	-5.559077754	####	###	AQP10	protein_coding	aquaporin 1	1	154321090	154325325
ENSG0000016334	-0.479194404	####	###	PMVK	protein_coding	phosphome	1	154924740	154936719
ENSG0000008684	0.410803854	####	###	ALG9	protein_coding	ALG9 alpha	11	111782195	111871581
ENSG0000011971	0.661862081	####	###	ALDH6A1	protein_coding	aldehyde de	14	74056847	74084492
ENSG0000000041	0.382554953	####	###	DPM1	protein_coding	dolichyl-pho	20	50934867	50958555
ENSG0000006439	0.279351116	####	###	HIPK2	protein_coding	homeodom	7	139561570	139777998
ENSG0000011635	-0.307109876	####	###	SRSF4	protein_coding	serine and a	1	29147743	29181900
ENSG0000022457	-0.692390144	####	###	HNRNPA1P	protein_coding	heterogene	16	51553436	51647132
ENSG0000018562	-0.18203169	####	###	P4HB	protein_coding	prolyl 4-hyd	17	81843161	81860624
ENSG0000021510	1.250329979	####	###	TTC3P1	processed_pse	tetratricope	X	75740831	75746911
ENSG0000016167	0.350239938	####	###	EMC10	protein_coding	ER membra	19	50476400	50490871
ENSG0000010289	-0.266818427	####	###	PHKB	protein_coding	phosphoryla	16	47461123	47701523
ENSG0000012516	-0.285260053	####	###	GOT2	protein_coding	glutamic-ox	16	58707131	58734342
ENSG0000013262	-1.46015282	####	###	ANKEF1	protein_coding	ankyrin repe	20	9986126	10058303
ENSG0000023667	-2.552109846	####	###	PRKG1-AS1	lncRNA	PRKG1 antis	10	52230398	52314507
ENSG0000015780	0.353168738	####	###	SLC37A3	protein_coding	solute carrie	7	140293693	140404433
ENSG0000013626	0.266466843	####	###	BZW2	protein_coding	basic leucin	7	16646131	16706523
ENSG0000026039	-1.521524322	####	###	AC068700.1	lncRNA	novel transc	8	78605952	78609705
ENSG0000018201	0.393450322	####	###	RTKN2	protein_coding	rhotekin 2 [S	10	62183035	62268844
ENSG0000016644	-1.382509532	####	###	TMEM130	protein_coding	transmembr	7	98846488	98870771
ENSG0000008880	0.269897492	####	###	PPP1R13B	protein_coding	protein pho	14	103733195	103847575
ENSG0000017730	-0.633193977	####	###	CASKIN2	protein_coding	CASK intera	17	75500261	75515537
ENSG0000024850	1.208290448	####	###	SRP14-AS1	lncRNA	SRP14 antis	15	40039242	40076539
ENSG0000008666	0.431786052	####	###	ZFAND6	protein_coding	zinc finger A	15	80059568	80138393
ENSG0000010026	-0.635478395	####	###	RHBDD3	protein_coding	rhomboid d	22	29259872	29268209
ENSG0000012092	-0.573716165	####	###	RNF170	protein_coding	ring finger p	8	42849637	42897290
ENSG0000013147	0.538450786	####	###	PSMC3IP	protein_coding	PSMC3 inte	17	42572310	42577831
ENSG0000016277	-0.406288123	####	###	ATF3	protein_coding	activating tr	1	212565334	212620777
ENSG0000012220	0.324769069	####	###	KIAA1191	protein_coding	KIAA1191 [S	5	176346062	176361807
ENSG0000017605	-0.666716909	####	###	TPRN	protein_coding	taperin [Sou	9	137191617	137204193
ENSG0000018657	0.437065399	####	###	NF2	protein_coding	neurofibron	22	29603556	29698598

ENSG0000026079	1.833380052	####	###	AC003102.1	lncRNA	novel transc	17	44221401	44223710
ENSG0000016747	1.622648788	####	###	JSRP1	protein_coding	junctional sa	19	2252252	2269759
ENSG0000005760	-0.211560277	####	###	GDI2	protein_coding	GDP dissoci	10	5765223	5842132
ENSG0000012390	-0.436867627	####	###	AGO2	protein_coding	argonaute R	8	140520156	140635633
ENSG0000019733	1.192793335	####	###	AC008543.1	lncRNA	novel transc	19	11639754	11686569
ENSG0000007295	-1.965913835	####	###	IRAG1	protein_coding	inositol 1,4,5	11	10573091	10693988
ENSG0000017340	0.43588601	####	###	DAG1	protein_coding	dystroglycan	3	49468703	49535618
ENSG0000018018	0.52216958	####	###	FAHD1	protein_coding	fumarylacet	16	1826941	1840207
ENSG0000009930	0.886889918	####	###	MAST3	protein_coding	microtubule	19	18097793	18151692
ENSG0000022564	-0.648629705	####	###	SBDSP1	transcribed_un	SBDS pseuc	7	72829425	72836701
ENSG0000006505	-0.741753225	####	###	NTHL1	protein_coding	nth like DNA	16	2039815	2047866
ENSG0000016801	0.354067353	####	###	C2CD3	protein_coding	C2 domain c	11	74012714	74171210
ENSG0000010422	0.490566424	####	###	TRIM35	protein_coding	tripartite mo	8	27284886	27311272
ENSG0000019813	0.528149453	####	###	HIBCH	protein_coding	3-hydroxyis	2	190189735	190344193
ENSG0000016681	0.592621267	####	###	KIF7	protein_coding	kinesin fami	15	89608789	89655467
ENSG0000016811	0.401673929	####	###	KIAA1586	protein_coding	KIAA1586 [S	6	57046532	57055239
ENSG0000027604	0.952310718	####	###	ORAI1	protein_coding	ORAI calciu	12	121626550	121642677
ENSG0000012892	-0.433671285	####	###	MINDY2	protein_coding	MINDY lysin	15	58771192	58861900
ENSG0000010884	0.31247808	####	###	HDAC5	protein_coding	histone dea	17	44076746	44123702
ENSG0000015097	-1.178972429	####	###	RILPL2	protein_coding	Rab interact	12	123410683	123436717
ENSG0000013319	-0.634717621	####	###	SLC39A11	protein_coding	solute carrie	17	72645949	73092712
ENSG0000019887	-0.493058202	####	###	TYW1	protein_coding	tRNA-yW sy	7	66995173	67239519
ENSG0000026277	0.479242476	####	###	SSBP1	protein_coding	single strand	CHR_HSCHR	141744329	141752534
ENSG0000010192	0.585958457	####	###	MOSPD1	protein_coding	motile sperm	X	134887632	134915257
ENSG0000026039	-4.335763537	####	###	AL360014.1	lncRNA	novel transc	9	27829276	27844481
ENSG0000016302	0.258652348	####	###	SMC6	protein_coding	structural m	2	17663812	17800242
ENSG0000009209	0.681291426	####	###	SLC22A17	protein_coding	solute carrie	14	23346306	23352912
ENSG0000000146	-2.053940504	####	###	STPG1	protein_coding	sperm tail P	1	24356999	24416934
ENSG0000027232	-0.251324585	####	###	NUDT3	protein_coding	nudix hydro	6	34279679	34392669
ENSG0000012212	0.322296976	####	###	OCRL	protein_coding	OCRL inosit	X	129539849	129592561
ENSG0000014948	0.642798015	####	###	TMEM138	protein_coding	transmembr	11	61362344	61369509
ENSG0000000708	0.377973436	####	###	CCDC124	protein_coding	coiled-coil c	19	17933015	17943991
ENSG0000004908	-0.877239248	####	###	COL9A2	protein_coding	collagen typ	1	40300489	40317813
ENSG0000014829	0.434830069	####	###	MED22	protein_coding	mediator co	9	133338312	133348131
ENSG0000009197	-0.825457245	####	###	CD200	protein_coding	CD200 mole	3	112332347	112362812
ENSG0000013685	0.857874913	####	###	SLC2A8	protein_coding	solute carrie	9	127397138	127408424
ENSG0000026808	1.327899888	####	###	GABRQ	protein_coding	gamma-am	X	152637895	152657542
ENSG0000011358	-0.70546661	####	###	NR3C1	protein_coding	nuclear rece	5	143277931	143435512
ENSG0000012581	0.399702523	####	###	NAPB	protein_coding	NSF attachm	20	23374519	23421519
ENSG0000011276	-0.464637303	####	###	BTN2A1	protein_coding	butyrophilin	6	26457904	26476621
ENSG0000020417	0.705278671	####	###	BMS1P1	transcribed_un	BMS1 pseuc	10	46786674	46811989
ENSG0000007514	0.438740789	####	###	SRI	protein_coding	sorcin [Sour	7	88205115	88226993
ENSG0000023285	1.72646962	####	###	LYRM9	protein_coding	LYR motif co	17	27878314	27894752
ENSG0000000754	0.593042488	####	###	PIGQ	protein_coding	phosphatidy	16	566995	584109
ENSG0000010203	-1.662695319	####	###	ELF4	protein_coding	E74 like ETS	X	130063955	130110716
ENSG0000027442	1.47676074	####	###	AC114271.1	lncRNA	novel transc	19	10333436	10336248
ENSG0000014489	-1.07070573	####	###	MED12L	protein_coding	mediator co	3	151085697	151437072
ENSG0000023449	-0.606333069	####	###	TRIM27	protein_coding	tripartite mo	CHR_HSCHR	28902993	28923981
ENSG0000010271	-0.369699701	####	###	SUPT20H	protein_coding	SPT20 homo	13	37009312	37059713
ENSG0000016212	-0.365597713	####	###	CLPB	protein_coding	caseinolytic	11	72285495	72434680
ENSG0000013616	0.8579213	####	###	RCBTB2	protein_coding	RCC1 and B	13	48488959	48533256
ENSG0000013081	-0.282749935	####	###	EIF3G	protein_coding	eukaryotic t	19	10115014	10119918
ENSG0000011449	0.422872504	####	###	UMPS	protein_coding	uridine mon	3	124730433	124749273
ENSG0000019645	-0.64354279	####	###	ZNF777	protein_coding	zinc finger p	7	149431363	149461062
ENSG0000017560	-1.897028039	####	###	SUGCT	protein_coding	succinyl-Co	7	40135005	40860763
ENSG0000009085	-0.284149365	####	###	PDPR	protein_coding	pyruvate de	16	70114332	70162537
ENSG0000012894	0.354693402	####	###	KNSTRN	protein_coding	kinetochore	15	40382721	40394246
ENSG0000017480	1.061041481	####	###	FZD4	protein_coding	frizzled class	11	86945679	86955395

ENSG0000007105	0.405954043	####	###	NCK2	protein_coding	NCK adapto	2	105744912	105894274
ENSG0000010564	-0.29540247	####	###	PIK3R2	protein_coding	phosphoino	19	18153163	18170532
ENSG0000018378	-7.418672264	####	###	TUBA8	protein_coding	tubulin alph	22	18110331	18146554
ENSG0000016517	2.467756219	####	###	METTL27	protein_coding	methyltrans	7	73834590	73842516
ENSG0000016899	-1.739897039	####	###	PXDC1	protein_coding	PX domain	6	3722614	3751713
ENSG0000017546	0.270043708	####	###	SART1	protein_coding	spliceosome	11	65961728	65980137
ENSG0000027956	0.710074933	####	###	ZNF43	protein_coding	zinc finger p	CHR_HSCHR	21814429	21818511
ENSG0000012565	-0.829195898	####	###	ALKBH7	protein_coding	alkB homolo	19	6372794	6375250
ENSG0000007081	-0.324954463	####	###	TCOF1	protein_coding	treacle ribos	5	150357629	150400308
ENSG0000010718	0.305352227	####	###	RGP1	protein_coding	RGP1 homo	9	35749287	35758585
ENSG0000007241	0.458371343	####	###	MPP5	protein_coding	membrane	14	67241342	67336061
ENSG0000016029	-0.276286808	####	###	MCM3AP	protein_coding	minichromo	21	46235133	46286297
ENSG0000010033	-0.267978961	####	###	MIEF1	protein_coding	mitochondr	22	39499432	39518132
ENSG0000005775	0.472147816	####	###	PITHD1	protein_coding	PITH domai	1	23778418	23788232
ENSG0000015860	0.326525345	####	###	TMED4	protein_coding	transmembr	7	44577894	44582287
ENSG0000010210	0.684470301	####	###	PCSK1N	protein_coding	proprotein c	X	48831096	48835610
ENSG0000017764	0.447628666	####	###	ACAD9	protein_coding	acyl-CoA de	3	128879596	128916067
ENSG0000010128	-2.152081151	####	###	RSPO4	protein_coding	R-spondin 4	20	958452	1002311
ENSG0000018622	0.744123938	####	###	BLOC1S4	protein_coding	biogenesis c	4	6716174	6717664
ENSG0000018873	-1.475844742	####	###	FAM221A	protein_coding	family with s	7	23680130	23703249
ENSG0000010896	0.335317745	####	###	MMD	protein_coding	monocyte to	17	55392622	55421924
ENSG0000022389	0.918582001	####	###	OSER1-DT	lncRNA	OSER1 diver	20	44210907	44226027
ENSG0000019627	-0.771872414	####	###	GTF2IRD2	protein_coding	GTF2I repea	7	74796144	74851551
ENSG0000013649	0.490553326	####	###	LIMD2	protein_coding	LIM domain	17	63695888	63701172
ENSG0000013647	0.377158245	####	###	TEX2	protein_coding	testis expres	17	64147227	64263260
ENSG0000018034	-0.878232395	####	###	FZD2	protein_coding	frizzled clas	17	44557484	44561262
ENSG0000024253	0.692662874	####	###	AC007620.2	lncRNA	novel transc	3	179396961	179399191
ENSG0000023171	2.138160854	####	###	LINC00899	lncRNA	long interge	22	46039907	46044853
ENSG0000019875	-1.540532154	####	###	PLXNB3	protein_coding	plexin B3 [S	X	153764196	153779346
ENSG0000017582	-0.415900707	####	###	CTDNEP1	protein_coding	CTD nuclear	17	7243591	7252491
ENSG0000014563	-0.618952947	####	###	PLK2	protein_coding	polo like kin	5	58453982	58460139
ENSG0000026199	-0.351655382	####	###	GATAD2B	protein_coding	GATA zinc f	CHR_HSCHR	153821956	153845579
ENSG0000010028	0.666673807	####	###	CHKB	protein_coding	choline kina	22	50578959	50601455
ENSG0000017623	-2.792037008	####	###	RPP38-DT	lncRNA	RPP38 diver	10	15095385	15097319
ENSG0000016632	1.792737252	####	###	C11orf65	protein_coding	chromosom	11	108308519	108467531
ENSG0000014208	0.876179063	####	###	SIRT3	protein_coding	sirtuin 3 [So	11	215030	236931
ENSG0000013563	-0.612938834	####	###	RAB11FIP5	protein_coding	RAB11 fami	2	73073382	73156721
ENSG0000014423	-0.333544728	####	###	AMMECR1L	protein_coding	AMMECR1 l	2	127861630	127885956
ENSG0000022945	-7.35957725	####	###	AC010157.1	lncRNA	novel transc	10	82224213	82229179
ENSG0000013864	-0.595520989	####	###	FAM13A	protein_coding	family with s	4	88725955	89111398
ENSG0000018192	0.397124676	####	###	COA4	protein_coding	cytochrome	11	73872667	73876901
ENSG0000016809	-0.670106241	####	###	ANKS3	protein_coding	ankyrin repe	16	4696510	4734378
ENSG0000016206	0.868549689	####	###	TEDC2	protein_coding	tubulin epsi	16	2460086	2464963
ENSG0000011101	-1.649884795	####	###	CYP27B1	protein_coding	cytochrome	12	57762334	57768986
ENSG0000012189	-5.461886728	####	###	TMEM156	protein_coding	transmembr	4	38966744	39032922
ENSG0000012169	0.470248755	####	###	CAT	protein_coding	catalase [So	11	34438934	34472060
ENSG0000017898	0.266912418	####	###	MRFAP1L1	protein_coding	Morf4 family	4	6707701	6709865
ENSG0000018492	-2.355238269	####	###	FMNL1	protein_coding	formin like 1	17	45221444	45247319
ENSG0000010838	0.506822452	####	###	RAD51C	protein_coding	RAD51 para	17	58692573	58735611
ENSG0000001024	0.210102621	####	###	ZNF207	protein_coding	zinc finger p	17	32350117	32381886
ENSG0000007001	0.3545813	####	###	UFD1	protein_coding	ubiquitin rec	22	19449911	19479202
ENSG0000007731	-0.34076983	####	###	SNRPA	protein_coding	small nuclea	19	40750637	40765389
ENSG0000015472	-0.378606679	####	###	GABPA	protein_coding	GA binding	21	25734570	25772460
ENSG0000002256	0.718183797	####	###	SLC45A4	protein_coding	solute carrie	8	141207166	141308305
ENSG0000015407	0.915911699	####	###	SDHAF4	protein_coding	succinate de	6	70566917	70589569
ENSG0000012069	-0.602970636	####	###	KBTBD7	protein_coding	kelch repeat	13	41189834	41194569
ENSG0000006897	-0.749885666	####	###	PPP2R5B	protein_coding	protein pho	11	64917553	64934475
ENSG0000027809	0.797890823	####	###	ZNF85	protein_coding	zinc finger p	CHR_HSCHR	20929638	20957113

ENSG0000010002	0.319222498	####	###	SNRPD3	protein_codinç	small nuclea	22	24555958	24582052
ENSG0000016241	-0.623959431	####	###	GMEB1	protein_codinç	glucocorticc	1	28668732	28719353
ENSG0000020449	-2.27705538	####	###	NFKBIL1	protein_codinç	NFKB inhibi	6	31546870	31558829
ENSG0000005055	-1.714064897	####	###	LAMC3	protein_codinç	laminin subı	9	131009174	131094473
ENSG0000026764	0.92772322	####	###	AC105052.:	protein_codinç	novel protei	7	102637049	102671641
ENSG0000010433	0.190241523	####	###	SFRP1	protein_codinç	secreted friz	8	41261962	41309473
ENSG0000017671	1.028271965	####	###	CCDC121	protein_codinç	coiled-coil (	2	27625639	27629012
ENSG0000009936	-1.578313809	####	###	STX1B	protein_codinç	syntaxin 1B	16	30989256	31010638
ENSG0000011526	0.213137144	####	###	RPS15	protein_codinç	ribosomal p	19	1438358	1440495
ENSG0000014578	-0.550235468	####	###	COMMD10	protein_codinç	COMM don	5	116085016	116412762
ENSG0000021422	-0.597486078	####	###	HNRNPA1F	processed_pse	heterogene	19	11666069	11667030
ENSG0000015454	0.566476301	####	###	SRSF12	protein_codinç	serine and a	6	89095959	89118071
ENSG0000014731	-0.545999637	####	###	MCPH1	protein_codinç	microcepha	8	6406596	6648508
ENSG0000015912	-0.74193367	####	###	IFNGR2	protein_codinç	interferon g	21	33403413	33479348
ENSG0000008166	0.459214472	####	###	ZNF506	protein_codinç	zinc finger p	19	19785839	19821751
ENSG0000012457	2.967986708	####	###	H1-3	protein_codinç	H1.3 linker l	6	26234212	26234987
ENSG0000024402	0.978552194	####	###	FAM86DP	transcribed_un	family with s	3	75421552	75435110
ENSG0000021212	-2.000073002	####	###	PRR22	protein_codinç	proline rich	19	5782960	5784746
ENSG0000017098	-3.299125861	####	###	S1PR1	protein_codinç	sphingosine	1	101236865	101243713
ENSG0000016453	0.616921639	####	###	DAGLB	protein_codinç	diacylglycer	7	6409126	6484190
ENSG0000013383	-0.276378348	####	###	HSD17B4	protein_codinç	hydroxyster	5	119452473	119637199
ENSG0000013747	0.299265397	####	###	FCHSD2	protein_codinç	FCH and do	11	72836745	73142318
ENSG0000015409	0.223826484	####	###	THY1	protein_codinç	Thy-1 cell sı	11	119415476	119424985
ENSG0000026776	-1.293521094	####	###	LINC01801	lncRNA	long interge	19	34788527	34832869
ENSG0000017526	-0.416926662	####	###	GOLGA8A	protein_codinç	golgin A8 fa	15	34379068	34437466
ENSG0000013072	-0.435777118	####	###	UBE2M	protein_codinç	ubiquitin co	19	58555712	58558954
ENSG0000023798	1.163026551	####	###	PTENP1	transcribed_pr	phosphatas	9	33673504	33677499
ENSG0000000601	0.751134177	####	###	REX1BD	protein_codinç	required for	19	18588685	18592336
ENSG0000008973	0.360497006	####	###	DDX24	protein_codinç	DEAD-box l	14	94048287	94081212
ENSG0000021515	-0.839985128	####	###	AC138409.:	transcribed_un	Putative bet	5	34164698	34244796
ENSG0000006843	-0.481626735	####	###	FTSJ1	protein_codinç	FtsJ RNA 2'-	X	48476021	48486364
ENSG0000013564	1.40090286	####	###	KCNMB4	protein_codinç	potassium c	12	70366290	70434292
ENSG0000015696	-2.983984353	####	###	B3GNT7	protein_codinç	UDP-GlcNA	2	231395710	231401164
ENSG0000010394	-0.621238608	####	###	HOMER2	protein_codinç	homer scaff	15	82836946	82986153
ENSG0000011016	2.611123125	####	###	HPX	protein_codinç	hemopexin	11	6431049	6442617
ENSG0000010020	-1.018998006	####	###	HSCB	protein_codinç	HscB mitoch	22	28742039	28757515
ENSG0000011996	0.3821976	####	###	HELLS	protein_codinç	helicase, lyn	10	94501434	94613905
ENSG0000025712	-1.585396025	####	###	RRN3P3	transcribed_un	RRN3 pseuc	16	22418672	22437715
ENSG0000014790	-0.368615183	####	###	ZCCHC7	protein_codinç	zinc finger (	9	37120574	37358149
ENSG0000017554	0.461556882	####	###	ALG10B	protein_codinç	ALG10 alph	12	38316762	38329721
ENSG0000017499	0.35057485	####	###	KLC2	protein_codinç	kinesin light	11	66257294	66267860
ENSG0000014533	-1.485431087	####	###	SNCA	protein_codinç	synuclein al	4	89700345	89838315
ENSG0000021307	-0.295148459	####	###	SCAF8	protein_codinç	SR-related (	6	154733378	154834244
ENSG0000015601	0.753146383	####	###	CARNMT1	protein_codinç	carnosine N	9	74980790	75028423
ENSG0000026279	-0.812535837	####	###	IFNGR2	protein_codinç	interferon g	CHR_HSCHR	33410836	33488905
ENSG0000010447	-0.471751899	####	###	CHRAC1	protein_codinç	chromatin a	8	140511311	140517154
ENSG0000011536	-0.403690949	####	###	MRPL19	protein_codinç	mitochondr	2	75646783	75690851
ENSG0000024300	-1.210666166	####	###	AC005062.:	lncRNA	novel transc	7	19918981	20140453
ENSG0000011952	-0.511622967	####	###	DENND1A	protein_codinç	DENN dom	9	123379654	123930152
ENSG0000016053	-1.110863828	####	###	PLPP7	protein_codinç	phospholipi	9	131289459	131359022
ENSG0000015856	0.694896479	####	###	DYNC1I1	protein_codinç	dynein cyto	7	95772506	96110322
ENSG0000007042	-0.456126901	####	###	RNF126	protein_codinç	ring finger p	19	647526	663233
ENSG0000008364	-0.355241995	####	###	PDS5B	protein_codinç	PDS5 cohes	13	32586452	32778019
ENSG0000008658	-0.324911701	####	###	RBM22	protein_codinç	RNA binding	5	150690792	150701077
ENSG0000007930	-0.422148868	####	###	TNS1	protein_codinç	tensin 1 [So	2	217799588	218033982
ENSG0000027949	-4.585980237	####	###	NA	NA	NA	NA	NA	NA
ENSG0000013293	-1.724485222	####	###	ATP8A2	protein_codinç	ATPase pho	13	25371974	26025851
ENSG0000020372	1.722755811	####	###	SAMD5	protein_codinç	sterile alpha	6	147508690	147737547

ENSG0000014154	-1.201597844	####	###	TTYH2	protein_coding	tweety fami	17		74213571	74262020
ENSG0000013994	-0.524188606	####	###	PELI2	protein_coding	pellino E3 u	14		56117814	56301524
ENSG0000023247	-0.659795554	####	###	EEF1B2P3	processed_pse	eukaryotic t	X		24788392	24789069
ENSG0000018047	-1.27878571	####	###	ZNF571	protein_coding	zinc finger p	19		37554782	37594792
ENSG0000012993	-0.327667606	####	###	MAU2	protein_coding	MAU2 sister	19		19320829	19358754
ENSG0000008564	0.713197066	####	###	ZNF213	protein_coding	zinc finger p	16		3129777	3142804
ENSG0000018798	-3.13819777	####	###	ANKRD19P	transcribed_un	ankyrin repe	9		92809568	92838457
ENSG0000019837	-0.520113855	####	###	WWP2	protein_coding	WW domain	16		69762306	69941741
ENSG0000014162	-0.660246479	####	###	RNF165	protein_coding	ring finger p	18		46326809	46463140
ENSG0000008912	-3.527769191	####	###	OAS1	protein_coding	2'-5'-oligoa	12		112906783	112933222
ENSG0000027335	-5.385919473	####	###	LINC02019	lncRNA	long interge	3		50669989	50672048
ENSG0000022958	1.633881515	####	###	ACVR2B-AS	lncRNA	ACVR2B ant	3		38451027	38454820
ENSG0000018486	-0.258625939	####	###	RBM33	protein_coding	RNA binding	7		155644451	155781485
ENSG0000012751	0.422691433	####	###	SIN3B	protein_coding	SIN3 transcr	19		16829400	16880353
ENSG0000003800	0.530123992	####	###	AGA	protein_coding	aspartylgluc	4		177430774	177442437
ENSG0000014025	0.360797143	####	###	MFAP1	protein_coding	microfibril a	15		43804492	43824690
ENSG0000018053	-5.433799295	####	###	BHLHA15	protein_coding	basic helix-l	7		98211427	98212979
ENSG0000007346	-0.648203165	####	###	CLCN4	protein_coding	chloride vol	X		10156975	10237660
ENSG0000010202	-0.350920196	####	###	PLS3	protein_coding	plastin 3 [Sc	X		115561174	115650861
ENSG0000023773	-1.007587356	####	###	CT75	transcribed_un	cancer/testis	2		222318275	222352989
ENSG0000014325	0.264461195	####	###	SDHC	protein_coding	succinate de	1		161314381	161371964
ENSG0000019686	0.928548686	####	###	ZFP28	protein_coding	ZFP28 zinc f	19		56538948	56556808
ENSG0000013935	-0.356317849	####	###	ASCL1	protein_coding	achaete-scu	12		102957674	102960513
ENSG0000017504	0.773568171	####	###	CHST2	protein_coding	carbohydrat	3		143119771	143124014
ENSG0000013610	-0.4590974	####	###	VPS36	protein_coding	vacuolar pro	13		52412602	52450634
ENSG0000013749	-0.177797001	####	###	NUMA1	protein_coding	nuclear mito	11		72002864	72080693
ENSG0000014830	-0.147122184	####	###	RPL7A	protein_coding	ribosomal p	9		133348218	133351426
ENSG0000016400	0.675744063	####	###	EXO5	protein_coding	exonuclease	1		40508741	40516556
ENSG0000027290	2.456366909	####	###	TBC1D8-AS	lncRNA	TBC1D8 ant	2		101151660	101155412
ENSG0000009924	0.296521325	####	###	RAB18	protein_coding	RAB18, mer	10		27504174	27542239
ENSG0000015820	0.959046889	####	###	ABHD3	protein_coding	abhydrolase	18		21650901	21704780
ENSG0000010037	0.521557196	####	###	KCTD17	protein_coding	potassium c	22		37051736	37063390
ENSG0000013572	0.188299889	####	###	DYNC1LI2	protein_coding	dynein cyto	16		66720893	66751609
ENSG0000010124	-0.600818007	####	###	NDUFAF5	protein_coding	NADH:ubiqu	20		13785007	13821580
ENSG0000016760	-1.968542703	####	###	AXL	protein_coding	AXL recepto	19		41219223	41261766
ENSG0000017767	-1.159181388	####	###	AGTRAP	protein_coding	angiotensin	1		11736084	11754802
ENSG0000025089	-2.148533929	####	###	AC125807.2	lncRNA	novel transc	12		3041437	3044950
ENSG0000019889	0.272358966	####	###	CAPZA2	protein_coding	capping act	7		116811070	116922049
ENSG0000016316	0.286494359	####	###	ERCC3	protein_coding	ERCC excisio	2		127257290	127294166
ENSG0000002610	-2.17758757	####	###	FAS	protein_coding	Fas cell surf	10		88990531	89017059
ENSG0000015718	-0.360271971	####	###	ODR4	protein_coding	odr-4 GPCR	1		186375838	186421378
ENSG0000011314	0.245349843	####	###	IK	protein_coding	IK cytokine	5		140647058	140662480
ENSG0000011660	-0.399793387	####	###	MEF2D	protein_coding	myocyte en	1		156463727	156500779
ENSG0000018232	0.717387348	####	###	FBXL6	protein_coding	F-box and l	8		144355431	144359376
ENSG0000016337	-0.75167452	####	###	EOGT	protein_coding	EGF domain	3		68975217	69013684
ENSG0000011894	-0.863403905	####	###	PCDH17	protein_coding	protocadhe	13		57631744	57729311
ENSG0000019664	0.692212824	####	###	ZNF136	protein_coding	zinc finger p	19		12163064	12189871
ENSG0000016121	-0.285163722	####	###	PCYT1A	protein_coding	phosphate c	3		196214222	196287957
ENSG0000022515	0.56915161	####	###	GOLGA2P7	transcribed_un	GOLGA2 ps	15		84199311	84230136
ENSG0000023381	-0.860344381	####	###	MRPS18B	protein_coding	mitochondr	CHR_HSCHR		30607946	30616632
ENSG0000014559	-0.44357749	####	###	RPL37	protein_coding	ribosomal p	5		40825262	40835222
ENSG0000011000	-1.607253407	####	###	VWA5A	protein_coding	von Willebra	11		124115404	124147721
ENSG0000021358	0.776906283	####	###	ZBTB9	protein_coding	zinc finger a	6		33453970	33457544
ENSG0000019726	0.483747726	####	###	GTF2E2	protein_coding	general tran	8		30578318	30658236
ENSG0000017288	-0.466013906	####	###	ZNF621	protein_coding	zinc finger p	3		40524878	40574685
ENSG0000012341	-0.886339889	####	###	IKZF4	protein_coding	IKAROS fam	12		56007659	56038435
ENSG0000008682	0.381505971	####	###	ZW10	protein_coding	zw10 kineto	11		113733187	113773735
ENSG0000017164	0.772575049	####	###	ZIK1	protein_coding	zinc finger p	19		57578456	57593890

ENSG0000010116	0.251118723	####	###	PRPF6	protein_coding	pre-mRNA	20	63981132	64033100
ENSG0000013088	-0.521485087	####	###	LRP3	protein_coding	LDL recepto	19	33177603	33208864
ENSG0000026716	1.008354087	####	###	AC022098.1	lncRNA	novel transc	19	14137146	14171267
ENSG0000027161	1.85567479	####	###	ATP2B1-AS	lncRNA	ATP2B1 ant	12	89708959	89712590
ENSG0000027467	0.897670919	####	###	GTF2H2C_2	protein_coding	GTF2H2 fan	CHR_HSCHR	70182128	70214752
ENSG0000001133	0.89705953	####	###	DPF1	protein_coding	double PHD	19	38211006	38229714
ENSG0000018618	0.379326123	####	###	KIF18B	protein_coding	kinesin fami	17	44924709	44947773
ENSG0000016753	-0.730717592	####	###	CACNB3	protein_coding	calcium volt	12	48813794	48828941
ENSG0000009981	0.295961548	####	###	POLR2E	protein_coding	RNA polyme	19	1086574	1095380
ENSG0000016619	-0.607696792	####	###	ALKBH3	protein_coding	alkB homolo	11	43880811	43920274
ENSG0000016726	0.829803209	####	###	DUS2	protein_coding	dihydrourid	16	67987746	68079320
ENSG0000010782	-1.111623775	####	0	KAZALD1	protein_coding	Kazal type s	10	101061989	101068131
ENSG0000009307	-3.07958109	####	0	ADA2	protein_coding	adenosine c	22	17178790	17258235
ENSG0000007295	-1.228363511	####	0	TMEM38A	protein_coding	transmembr	19	16661139	16690023
ENSG0000010107	-0.424093858	####	0	NDRG3	protein_coding	NDRG famil	20	36651766	36746090
ENSG0000014821	-0.27697786	####	0	ASTN2	protein_coding	astrotactin 2	9	116425225	117415070
ENSG0000024524	-1.538150073	####	0	USP2-AS1	lncRNA	USP2 antise	11	119364359	119526664
ENSG0000013749	0.283178115	####	0	THAP12	protein_coding	THAP doma	11	76349956	76381132
ENSG0000019712	0.966766721	####	0	ZNF682	protein_coding	zinc finger p	19	19997058	20039505
ENSG0000023263	1.495547575	####	0	GABBR1	protein_coding	gamma-am	CHR_HSCHR	29552212	29629760
ENSG0000022738	-7.263419889	####	0	AL133410.1	lncRNA	novel transc	9	35772163	35790432
ENSG0000020542	0.583213362	####	0	CNEP1R1	protein_coding	CTD nuclear	16	50024410	50037088
ENSG0000026091	1.670374887	####	0	AL158206.1	lncRNA	novel transc	9	19453209	19455173
ENSG0000011085	-0.392730319	####	0	PRDM4	protein_coding	PR/SET dom	12	107732871	107761272
ENSG0000016019	-0.865437072	####	0	WDR4	protein_coding	WD repeat c	21	42843094	42879568
ENSG0000022611	-1.120231301	####	0	MRPS18B	protein_coding	mitochondr	CHR_HSCHR	30662345	30671031
ENSG0000012688	0.327185107	####	0	NUP214	protein_coding	nucleoporin	9	131125586	131234663
ENSG0000014035	0.200830491	####	0	ANP32A	protein_coding	acidic nucle	15	68778535	68820897
ENSG0000023470	0.398399613	####	0	BRD2	protein_coding	bromodoma	CHR_HSCHR	33122451	33135290
ENSG0000019855	0.506823507	####	0	ZNF627	protein_coding	zinc finger p	19	11559374	11619161
ENSG0000010755	-0.434378536	####	0	DNMBP	protein_coding	dynamin bir	10	99875577	100009947
ENSG0000016657	-0.529242071	####	0	TMEM135	protein_coding	transmembr	11	87037844	87328824
ENSG0000020478	-5.338536618	####	0	ZNF204P	processed_pse	zinc finger p	6	27357825	27360221
ENSG0000025710	-3.194200713	####	0	NHLRC4	protein_coding	NHL repeat	16	567005	569495
ENSG0000018584	-0.694610415	####	0	DNAH14	protein_coding	dynein axon	1	224896262	225399292
ENSG0000016635	-0.558424073	####	0	IFTAP	protein_coding	intraflagella	11	36594493	36659290
ENSG0000016404	0.287042076	####	0	PGRMC2	protein_coding	progesteron	4	128269237	128288829
ENSG0000016837	-0.275693778	####	0	ARF4	protein_coding	ADP ribosyla	3	57571363	57598220
ENSG0000017759	1.237416037	####	0	ZNF491	protein_coding	zinc finger p	19	11797667	11809622
ENSG0000006411	0.428254377	####	0	TM7SF3	protein_coding	transmembr	12	26971579	27014434
ENSG0000008705	-0.258618665	####	0	MTMR2	protein_coding	myotubulari	11	95821766	95925315
ENSG0000013367	0.994020585	####	0	TMEM254	protein_coding	transmembr	10	80078646	80092557
ENSG0000014047	-0.486820499	####	0	ULK3	protein_coding	unc-51 like	15	74836118	74843346
ENSG0000017227	0.165693194	####	0	BSG	protein_coding	basigin (Ok	19	571277	583493
ENSG0000019741	1.04275246	####	0	SHPK	protein_coding	sedoheptulo	17	3608240	3636250
ENSG0000006840	0.362228156	####	0	GRIPAP1	protein_coding	GRIP1 assoc	X	48973720	49002264
ENSG0000013878	0.488590403	####	0	INTS12	protein_coding	integrator c	4	105682627	105895986
ENSG0000012645	0.949806323	####	0	BCL2L12	protein_coding	BCL2 like 12	19	49665142	49673916
ENSG0000018316	0.525986457	####	0	FANCF	protein_coding	FA complem	11	22622533	22625823
ENSG0000007943	-1.567128164	####	0	LIPE	protein_coding	lipase E, hor	19	42401514	42427388
ENSG0000026140	-0.830439991	####	0	TEN1-CDK3	protein_coding	TEN1-CDK3	17	75979231	76005999
ENSG0000016425	0.404618321	####	0	WDR41	protein_coding	WD repeat c	5	77425970	77620611
ENSG0000014026	0.210188856	####	0	SERF2	protein_coding	small EDRK-	15	43777087	43804427
ENSG0000010042	-0.293606462	####	0	BRD1	protein_coding	bromodoma	22	49773283	49827512
ENSG0000008854	-1.022661329	####	0	C3orf18	protein_coding	chromosom	3	50558025	50571027
ENSG0000027897	-3.740870613	####	0	AC091305.1	TEC	TEC	18	70489630	70491205
ENSG0000012548	-0.444481809	####	0	DDX31	protein_coding	DEAD-box h	9	132592997	132670401
ENSG0000025044	1.609248526	####	0	AC092335.1	lncRNA	novel transc	5	16180238	16185585

ENSG0000010810	-0.22348556	####	0	RPL28	protein_coding	ribosomal p	19	55385932	55403250
ENSG0000023394	-0.683593943	####	0	TRIM27	protein_coding	tripartite mc	CHR_HSCHR	28903164	28924151
ENSG0000012546	-0.883456177	####	0	C1orf61	protein_coding	chromosom	1	156404250	156456763
ENSG0000022574	0.453690793	####	0	PRRC2A	protein_coding	proline rich	CHR_HSCHR	31697009	31714058
ENSG0000020421	0.236583278	####	0	BMPR2	protein_coding	bone morph	2	202376327	202567751
ENSG0000017641	0.858694708	####	0	DNAJC30	protein_coding	DnaJ heat sl	7	73680918	73683453
ENSG0000014320	-0.377499898	####	0	COP1	protein_coding	COP1 E3 ub	1	175944831	176207286
ENSG0000001818	-0.278128034	####	0	RUFY3	protein_coding	RUN and FY	4	70704204	70808619
ENSG0000011224	0.298757165	####	0	E2F3	protein_coding	E2F transcri	6	20401879	20493714
ENSG0000016563	0.434145181	####	0	TAF3	protein_coding	TATA-box b	10	7818505	8016631
ENSG0000013396	-0.298608968	####	0	NUMB	protein_coding	NUMB endc	14	73275107	73458617
ENSG0000013074	-0.370078603	####	0	ZC3H4	protein_coding	zinc finger (	19	47064187	47113776
ENSG0000018837	-1.810140563	####	0	ZP3	protein_coding	zona pelluci	7	76397518	76442071
ENSG0000027384	0.451985448	####	0	TAF9	protein_coding	TATA-box b	5	69364743	69370013
ENSG0000010953	-0.414114171	####	0	GAR1	protein_coding	GAR1 ribon	4	109815510	109824740
ENSG0000018100	-0.727325948	####	0	ZFP82	protein_coding	ZFP82 zinc f	19	36383120	36418644
ENSG0000027761	-0.706054342	####	0	SCG5	protein_coding	secretogran	CHR_HSCHR	32721966	32777405
ENSG0000010753	0.674842111	####	0	PHYH	protein_coding	phytanoyl-(	10	13277796	13302412
ENSG0000017129	0.449544209	####	0	GAA	protein_coding	glucosidase	17	80101556	80119881
ENSG0000017045	-2.874685839	####	0	CD14	protein_coding	CD14 molec	5	140631728	140633701
ENSG0000026754	-1.886874872	####	0	AC015802.4	lncRNA	novel transc	17	76545668	76557683
ENSG0000027882	1.518817427	####	0	H3C10	protein_coding	H3 clusterec	6	27810064	27811300
ENSG0000003415	0.920068513	####	0	MAP2K3	protein_coding	mitogen-ac	17	21284672	21315232
ENSG0000026488	-0.717572678	####	0	NA	NA	NA	NA	NA	NA
ENSG0000026258	-1.362292166	####	0	AC087741.1	lncRNA	novel transc	17	80200673	80205949
ENSG0000016474	1.242029043	####	0	C8orf48	protein_coding	chromosom	8	13566869	13568288
ENSG0000017956	-0.444654874	####	0	GCC1	protein_coding	GRIP and cc	7	127580628	127593611
ENSG0000017272	0.813819633	####	0	FUT10	protein_coding	fucosyltrans	8	33370824	33473146
ENSG0000016284	-0.346912943	####	0	KIF26B	protein_coding	kinesin fami	1	245154985	245709432
ENSG0000000486	-0.383730935	####	0	SLC25A13	protein_coding	solute carrie	7	96120220	96322147
ENSG0000017522	-1.566846882	####	0	GAL3ST3	protein_coding	galactose-3	11	66040765	66049161
ENSG0000021479	1.272140643	####	0	TUBAP5	transcribed_un	tubulin alph	1	202851828	202861620
ENSG0000025865	1.602518873	####	0	ARHGAP5-	lncRNA	ARHGAP5 a	14	32074946	32076793
ENSG0000018123	-2.525300988	####	0	TMEM132C	protein_coding	transmembi	12	128267403	128707915
ENSG0000013474	0.298599692	####	0	PRPF38A	protein_coding	pre-mRNA	1	52404602	52420836
ENSG0000016418	0.291223718	####	0	LMBRD2	protein_coding	LMBR1 dom	5	36098407	36151887
ENSG0000010224	0.261888745	####	0	HTATSF1	protein_coding	HIV-1 Tat s	X	136497079	136512346
ENSG0000014208	-2.141975511	####	0	IFITM3	protein_coding	interferon ir	11	319676	327537
ENSG0000011166	0.201898248	####	0	TPI1	protein_coding	triosephosp	12	6867119	6870948
ENSG0000027402	-1.998783484	####	0	FAM27E3	lncRNA	family with s	9	67717411	67719178
ENSG0000022573	-7.210996018	####	0	NRG3-AS1	lncRNA	NRG3 antise	10	82228985	82232920
ENSG0000016244	0.474671387	####	0	LZIC	protein_coding	leucine zipp	1	9922113	9943407
ENSG0000014672	0.319693388	####	0	NIPSNAP2	protein_coding	nipsnap hor	7	55951793	56000181
ENSG0000001412	0.360056588	####	0	UFL1	protein_coding	UFM1 speci	6	96521595	96555276
ENSG0000014830	0.307497671	####	0	GTF3C5	protein_coding	general trar	9	133030675	133058503
ENSG0000015531	-0.339704085	####	0	USP25	protein_coding	ubiquitin sp	21	15730025	15880069
ENSG0000010524	0.629502583	####	0	NUMBL	protein_coding	NUMB like e	19	40665905	40690972
ENSG0000019675	-3.419739835	####	0	S100A2	protein_coding	S100 calciur	1	153561108	153567890
ENSG0000017066	1.363881692	####	0	RASA4B	protein_coding	RAS p21 prc	7	102479976	102517777
ENSG0000011819	-5.382273984	####	0	TNNT2	protein_coding	troponin T2	1	201359008	201377764
ENSG0000011412	-0.419127945	####	0	RNF7	protein_coding	ring finger p	3	141738249	141747560
ENSG0000012524	1.197927001	####	0	CLYBL	protein_coding	citramalyl-(	13	99606669	99897134
ENSG0000027305	-2.495018752	####	0	AL359921.2	lncRNA	novel transc	1	236536162	236536704
ENSG0000014581	0.306964158	####	0	YIPF5	protein_coding	Yip1 domair	5	144158162	144170714
ENSG0000021465	1.37475431	####	0	B3GNT10	protein_coding	UDP-GlcNA	9	120792403	120799918
ENSG0000013248	-1.647439139	####	0	TRIM47	protein_coding	tripartite mc	17	75874164	75878581
ENSG0000024381	3.331183677	####	0	RN7SL832F	lncRNA	RNA, 7SL, c	2	10690344	10692099
ENSG0000008916	-1.550043588	####	0	RPH3A	protein_coding	rabphilin 3A	12	112570380	112898881

ENSG0000012103	-0.717202579	####	0	RDH10	protein_coding	retinol dehy	8	73294602	73325281
ENSG0000007204	0.265038961	####	0	RDH11	protein_coding	retinol dehy	14	67676800	67695793
ENSG0000012070	-0.198119912	####	0	ETF1	protein_coding	eukaryotic t	5	138506095	138543236
ENSG0000019875	-0.157670103	####	0	RPL10A	protein_coding	ribosomal p	6	35468401	35470785
ENSG0000007553	0.254270166	####	0	FRYL	protein_coding	FRY like trar	4	48497357	48780322
ENSG0000017564	0.687007161	####	0	RMI2	protein_coding	RecQ media	16	11249619	11381662
ENSG0000011982	-0.253715228	####	0	YIPF4	protein_coding	Yip1 domair	2	32277904	32316594
ENSG0000026167	0.508116733	####	0	SCRT1	protein_coding	scratch fami	8	144330565	144336482
ENSG0000008309	0.350078651	####	0	LYRM2	protein_coding	LYR motif co	6	89568144	89638749
ENSG0000006800	0.622567558	####	0	HYAL2	protein_coding	hyaluronida	3	50317790	50322782
ENSG0000011676	-0.938137816	####	0	CTH	protein_coding	cystathionin	1	70411218	70439851
ENSG0000016552	-0.368479932	####	0	RPUSD4	protein_coding	RNA pseudo	11	126202096	126211692
ENSG0000017609	-0.38327751	####	0	IP6K1	protein_coding	inositol hexa	3	49724294	49786542
ENSG0000012590	-0.732980984	####	0	MRPS26	protein_coding	mitochondr	20	3046052	3048250
ENSG0000013373	0.380708917	####	0	IMPA1	protein_coding	inositol mor	8	81656914	81686331
ENSG0000013545	0.360431849	####	0	TFCP2	protein_coding	transcriptior	12	51093656	51173135
ENSG0000014958	0.662458388	####	0	TMEM25	protein_coding	transmembr	11	118531041	118547280
ENSG0000016353	0.939747543	####	0	SERPINI1	protein_coding	serpin family	3	167735243	167825569
ENSG0000016386	0.503441599	####	0	SMIM12	protein_coding	small integr	1	34712737	34859755
ENSG0000025166	1.185707855	####	0	FAM86EP	transcribed_un	family with s	4	3941760	3955432
ENSG0000018371	0.510955546	####	0	TRIM52	protein_coding	tripartite mo	5	181254417	181261139
ENSG0000027850	-2.139802803	####	0	NA	NA	NA	NA	NA	NA
ENSG0000015289	-0.55195429	####	0	PTPRK	protein_coding	protein tyro	6	127968779	128520674
ENSG0000017624	0.844612576	####	0	ACBD7	protein_coding	acyl-CoA bi	10	15075475	15088776
ENSG0000014535	-3.696629036	####	0	DDIT4L	protein_coding	DNA damag	4	100185870	100190782
ENSG0000016843	-0.759954574	####	0	COG7	protein_coding	component	16	23388493	23453189
ENSG0000005076	-2.492154425	####	0	COL23A1	protein_coding	collagen typ	5	178237618	178590393
ENSG0000012660	-1.223003536	####	0	GLIS2	protein_coding	GLIS family	16	4314761	4339597
ENSG0000027368	-2.044024032	####	0	AC009318.2	lncRNA	novel transc	12	29332733	29333383
ENSG0000020641	-0.572173016	####	0	RAB12	protein_coding	RAB12, mer	18	8609437	8639382
ENSG0000017533	0.275078009	####	0	BANF1	protein_coding	BAF nuclear	11	66002228	66004149
ENSG0000027266	1.408752614	####	0	AC012306.2	lncRNA	novel transc	2	127886556	127887185
ENSG0000015911	-0.431989601	####	0	MRPL10	protein_coding	mitochondr	17	47823272	47831541
ENSG0000011973	-1.811688144	####	0	GPR75	protein_coding	G protein-c	2	53852912	53859967
ENSG0000016210	0.367142668	####	0	ADCY9	protein_coding	adenylate cy	16	3953387	4116442
ENSG0000016260	0.254031704	####	0	USP1	protein_coding	ubiquitin sp	1	62436297	62451804
ENSG0000014419	0.864522486	####	0	FAHD2B	protein_coding	fumarylacet	2	97083583	97094882
ENSG0000011697	-0.457951058	####	0	LGALS8	protein_coding	galectin 8 [S	1	236518000	236552981
ENSG0000023729	0.952504608	####	0	TTN-AS1	lncRNA	TTN antisen	2	178521183	178779963
ENSG0000025933	1.013665809	####	0	INAFM2	protein_coding	InaF motif c	15	40323692	40326715
ENSG0000011140	-4.062353927	####	0	ENDOU	protein_coding	endonuclea	12	47709734	47725567
ENSG0000018140	-2.258052042	####	0	AATK	protein_coding	apoptosis as	17	81110487	81166221
ENSG0000001029	0.520488867	####	0	IFFO1	protein_coding	intermediat	12	6538375	6556083
ENSG0000016466	0.392256575	####	0	USP49	protein_coding	ubiquitin sp	6	41789896	41895361
ENSG0000017434	-7.174678415	####	0	CHRNA9	protein_coding	cholinergic r	4	40335333	40355217
ENSG0000019815	1.453434961	####	0	ZNF876P	transcribed_un	zinc finger p	4	212610	255985
ENSG0000010197	-0.20196029	####	0	STAG2	protein_coding	stromal anti	X	123960212	124422664
ENSG0000012678	-3.518130642	####	0	RHOJ	protein_coding	ras homolog	14	63204114	63293508
ENSG0000016599	-1.161468944	####	0	CACNB2	protein_coding	calcium volt	10	18140424	18543557
ENSG0000017084	0.47990813	####	0	AC093323.1	protein_coding	Putative MC	4	6663396	6676755
ENSG0000018452	0.651994043	####	0	CEND1	protein_coding	cell cycle ex	11	787115	790113
ENSG0000016595	-0.640830162	####	0	CLMN	protein_coding	calmin [Sou	14	95181940	95319908
ENSG0000022900	-0.584329769	####	0	TRIM27	protein_coding	tripartite mo	CHR_HSCHR	28903869	28924846
ENSG0000010133	-0.278412126	####	0	TM9SF4	protein_coding	transmembr	20	32109714	32167258
ENSG0000022812	1.218006028	####	0	DDAH2	protein_coding	dimethylarg	CHR_HSCHR	31717799	31721378
ENSG0000014156	-0.363608985	####	0	RPTOR	protein_coding	regulatory a	17	80544819	80966371
ENSG0000027243	7.153491214	####	0	AL645608.6	lncRNA	novel transc	1	904834	915976
ENSG0000010968	-0.403279296	####	0	SH3D19	protein_coding	SH3 domair	4	151102751	151325632

ENSG0000023417	-0.688233401	####	0	RNASEH1-/	lncRNA	RNASEH1 a	2	3558474	3564842
ENSG0000011667	0.319788899	####	0	DNAJC6	protein_codinc	DnaJ heat sl	1	65248219	65415871
ENSG0000011940	-2.558602169	####	0	NEK6	protein_codinc	NIMA relate	9	124257606	124353307
ENSG0000015923	1.057535292	####	0	AC005041.1	protein_codinc	novel protei	2	74393836	74421662
ENSG0000018610	0.425817684	####	0	ANKRD46	protein_codinc	ankyrin repe	8	100509752	100559784
ENSG0000010393	0.416566845	####	0	RPAP1	protein_codinc	RNA polyme	15	41517176	41544269
ENSG0000017323	-0.223571354	####	0	GOLGB1	protein_codinc	golgin B1 [S	3	121663199	121749767
ENSG0000009316	0.512099166	####	0	LRRFIP2	protein_codinc	LRR binding	3	37052626	37183689
ENSG0000007539	-0.942699683	####	0	RASAL2	protein_codinc	RAS protein	1	178094104	178484147
ENSG0000012422	-0.269065142	####	0	RNF114	protein_codinc	ring finger p	20	49936336	49953885
ENSG0000017023	-1.214812149	####	0	FABP6	protein_codinc	fatty acid bi	5	160187367	160238735
ENSG0000005980	-0.772573167	####	0	SLC2A3	protein_codinc	solute carrie	12	7919230	7936187
ENSG0000019700	0.684398716	####	0	ZNF138	protein_codinc	zinc finger p	7	64794388	64833681
ENSG0000015881	-7.148962129	####	0	FGF17	protein_codinc	fibroblast gi	8	22042398	22048809
ENSG0000000045	-0.555150974	####	0	SCYL3	protein_codinc	SCY1 like ps	1	169849631	169894267
ENSG0000018237	0.628381634	####	0	CLN8	protein_codinc	CLN8 transr	8	1755778	1801711
ENSG0000006711	0.828322015	####	0	PLPP1	protein_codinc	phospholipi	5	55424854	55534969
ENSG0000010289	0.299549537	####	0	NUTF2	protein_codinc	nuclear tran	16	67846923	67872567
ENSG0000023268	-7.194939497	####	0	AC002511.1	lncRNA	novel transc	19	35432957	35434642
ENSG0000010200	-0.526042856	####	0	PLP2	protein_codinc	proteolipid	X	49171898	49175235
ENSG0000016565	-0.620136776	####	0	ZNF503	protein_codinc	zinc finger p	10	75397830	75401764
ENSG0000016653	0.402021528	####	0	RIMKLB	protein_codinc	ribosomal m	12	8681600	8783095
ENSG0000018779	0.463870458	####	0	FANCM	protein_codinc	FA complen	14	45135930	45200890
ENSG0000027197	1.045613188	####	0	NA	NA	NA	NA	NA	NA
ENSG0000012272	0.661209283	####	0	ACO1	protein_codinc	aconitase 1	9	32384603	32454769
ENSG0000000162	0.267050644	####	0	ANKIB1	protein_codinc	ankyrin repe	7	92245974	92401383
ENSG0000016387	0.453838396	####	0	SNIP1	protein_codinc	Smad nucle	1	37534449	37554293
ENSG0000006049	0.475359582	####	0	OGFR	protein_codinc	opioid grow	20	62804835	62814000
ENSG0000024097	-0.279918127	####	0	MIF	protein_codinc	macrophage	22	23894383	23895227
ENSG0000020348	-0.531456902	####	0	INF2	protein_codinc	inverted for	14	104681146	104722535
ENSG0000025771	2.125646502	####	0	CPNE8-AS1	lncRNA	CPNE8 antis	12	38906451	38909592
ENSG0000017384	-0.272968934	####	0	NET1	protein_codinc	neuroepithe	10	5412557	5459056
ENSG0000014061	-0.253260917	####	0	SEC11A	protein_codinc	SEC11 hom	15	84669538	84716460
ENSG0000014799	0.495321865	####	0	CBWD5	protein_codinc	COBW dom	9	65668805	65734041
ENSG0000007295	0.338470454	####	0	AP1M1	protein_codinc	adaptor rela	19	16197854	16245906
ENSG0000014591	-1.418628861	####	0	N4BP3	protein_codinc	NEDD4 binc	5	178113532	178126081
ENSG0000022350	1.148329861	####	0	AC135983.1	transcribed_pr	WAS protei	15	32519848	32536926
ENSG0000017257	-1.7742115	####	0	RASGRP1	protein_codinc	RAS guanyl	15	38488103	38565575
ENSG0000027878	-0.74971821	####	0	SBNO2	protein_codinc	strawberry r	CHR_HSCHR	1107635	1156979
ENSG0000010575	0.772218874	####	0	ZNF85	protein_codinc	zinc finger p	19	20923222	20950697
ENSG0000013083	0.756633581	####	0	MPP1	protein_codinc	membrane	X	154778684	154821007
ENSG0000027925	2.049786973	####	0	AC087741.1	TEC	TEC	17	80147250	80148596
ENSG0000027940	-1.869110986	####	0	AC007191.1	TEC	TEC	19	45714387	45717381
ENSG0000013645	-0.167030283	####	0	SRSF1	protein_codinc	serine and a	17	58000919	58007346
ENSG0000010028	-0.280481713	####	0	NEFH	protein_codinc	neurofilame	22	29480218	29491390
ENSG0000016179	-2.847136123	####	0	AQP5	protein_codinc	aquaporin 5	12	49961872	49965682
ENSG0000014054	-0.684405653	####	0	DET1	protein_codinc	DET1 partne	15	88494440	88546681
ENSG0000010556	0.244448488	####	0	PPP2R1A	protein_codinc	protein pho	19	52190048	52229518
ENSG0000011768	0.521596568	####	0	DHDDS	protein_codinc	dehydrodoli	1	26432282	26471306
ENSG0000012106	-0.301039398	####	0	SPOP	protein_codinc	speckle type	17	49598884	49678163
ENSG0000027245	-1.382274936	####	0	NA	NA	NA	NA	NA	NA
ENSG0000013413	0.224753499	####	0	MEIS2	protein_codinc	Meis homec	15	36889204	37101299
ENSG0000016622	0.22224851	####	0	CCT2	protein_codinc	chaperonin	12	69585426	69601570
ENSG0000013558	-1.27585968	####	0	SMPD2	protein_codinc	sphingomye	6	109440724	109443919
ENSG0000017578	0.526045718	####	0	SLC35E3	protein_codinc	solute carrie	12	68746125	68793964
ENSG0000019823	0.205490795	####	0	DDX42	protein_codinc	DEAD-box l	17	63773603	63819317
ENSG0000006999	-0.374628875	####	0	HDHD5	protein_codinc	haloacid de	22	17137511	17165287
ENSG0000007890	0.382560463	####	0	TOLLIP	protein_codinc	toll interacti	11	1274371	1309654

ENSG0000016774	0.678523474	####	0	CYB5D2	protein_coding	cytochrome	17	4143168	4187310
ENSG0000021339	2.455511115	####	0	LCAT	protein_coding	lecithin-cho	16	67939750	67944131
ENSG0000012268	0.418478342	####	0	MRM2	protein_coding	mitochondr	7	2234195	2242205
ENSG0000015882	0.528267367	####	0	PINK1	protein_coding	PTEN induc	1	20633458	20651511
ENSG0000012267	-0.342239929	####	0	CCZ1	protein_coding	CCZ1 homc	7	5898725	5926550
ENSG0000027617	0.957634234	####	0	NA	NA	NA	NA	NA	NA
ENSG0000023762	-3.48818204	####	0	OXCT2P1	unprocessed_p	3-oxoacid C	1	39514956	39516490
ENSG0000022746	2.419371695	####	0	LINC01537	lncRNA	long interge	11	72570660	72573229
ENSG0000013576	-0.35630778	####	0	URB2	protein_coding	URB2 riboso	1	229626247	229660200
ENSG0000018193	-0.457907277	####	0	GINS3	protein_coding	GINS compl	16	58295080	58406147
ENSG0000013387	0.18960611	####	0	SARAF	protein_coding	store-opera	8	30063003	30083208
ENSG0000010782	-0.55696759	####	0	FBXW4	protein_coding	F-box and V	10	101610664	101695295
ENSG0000019701	0.777510717	####	0	ZNF470	protein_coding	zinc finger p	19	56567468	56588911
ENSG0000016511	-1.190314477	####	0	GKAP1	protein_coding	G kinase an	9	83739425	83829516
ENSG0000013937	-0.2547212	####	0	TDG	protein_coding	thymine DN	12	103965822	103988874
ENSG0000012610	0.533396337	####	0	HECTD3	protein_coding	HECT doma	1	45002540	45011324
ENSG0000014980	-0.227300883	####	0	FAU	protein_coding	FAU ubiquit	11	65120630	65122177
ENSG0000011039	0.213383982	####	0	CBL	protein_coding	Cbl proto-o	11	119206298	119313926
ENSG0000020446	-0.443377861	####	0	BAG6	protein_coding	BAG cochap	6	31639028	31652705
ENSG0000019847	-0.546173952	####	0	SH3BGRL2	protein_coding	SH3 domair	6	79631329	79703655
ENSG0000020623	-1.701792821	####	0	TAP2	protein_coding	transporter	CHR_HSCHR	32975593	32992541
ENSG0000010317	-0.956020989	####	0	NAGPA	protein_coding	N-acetylglu	16	5024844	5034141
ENSG0000010762	0.305750089	####	0	DDX50	protein_coding	DExD-box h	10	68901286	68946847
ENSG0000013504	-0.263935223	####	0	AGTPBP1	protein_coding	ATP/GTP bi	9	85546539	85742029
ENSG0000017589	-0.356663329	####	0	ZDHHC21	protein_coding	zinc finger D	9	14611071	14693471
ENSG0000011473	0.74775908	####	0	HEMK1	protein_coding	HemK meth	3	50569152	50596168
ENSG0000023466	2.105800147	####	0	HMGN2P5	processed_pse	high mobilit	15	29730713	29730985
ENSG0000018904	-0.467823183	####	0	ALKBH2	protein_coding	alkB homolo	12	109088188	109093631
ENSG0000012185	0.683474515	####	0	POLR3GL	protein_coding	RNA polyme	1	145964690	145978848
ENSG0000014622	-0.204503532	####	0	RPL7L1	protein_coding	ribosomal p	6	42879616	42889925
ENSG0000010396	0.711602611	####	0	EHD4	protein_coding	EH domain	15	41895933	41972557
ENSG0000018175	0.472862999	####	0	MACIR	protein_coding	macrophage	5	103258763	103278660
ENSG0000012563	-0.318562733	####	0	POLR1B	protein_coding	RNA polyme	2	112541915	112579818
ENSG0000018802	0.369074868	####	0	UBQLN2	protein_coding	ubiquilin 2 [	X	56563627	56567868
ENSG0000014325	-0.465746512	####	0	USP21	protein_coding	ubiquitin sp	1	161159450	161165723
ENSG0000027672	-0.378207853	####	0	DNAJA3	protein_coding	DnaJ heat sl	CHR_HSCHR	4427764	4458734
ENSG0000010716	0.282151079	####	0	FUBP3	protein_coding	far upstrean	9	130578965	130638352
ENSG0000018015	2.772667538	####	0	LYNX1	protein_coding	Ly6/neurotc	8	142771197	142777810
ENSG0000016013	0.369619202	####	0	VMA21	protein_coding	vacuolar AT	X	151396515	151409364
ENSG0000016796	0.455100172	####	0	ECI1	protein_coding	enoyl-CoA	16	2239402	2252300
ENSG0000015710	0.534834452	####	0	FCHO2	protein_coding	FCH and mu	5	72956041	73090522
ENSG0000026217	-5.283936506	####	0	AC116025.2	lncRNA	novel transc	17	79991944	79992841
ENSG0000018119	-7.098742959	####	0	PENK	protein_coding	proenkepha	8	56436674	56446671
ENSG0000023113	-7.10138106	####	0	LNCAROD	lncRNA	lncRNA activ	10	52450874	52755507
ENSG0000027207	-1.610307009	####	0	AC124045.1	lncRNA	novel transc	3	44667412	44669364
ENSG0000023091	1.476353751	####	0	AL391807.1	lncRNA	novel transc	6	68627879	68635161
ENSG0000011978	0.240847925	####	0	ATL2	protein_coding	atlastin GTP	2	38293954	38377285
ENSG0000018357	0.789838397	####	0	ZNRF3	protein_coding	zinc and rin	22	28883572	29057488
ENSG0000024183	0.796630511	####	0	PLEKHO2	protein_coding	pleckstrin ho	15	64841883	64868002
ENSG0000013379	-0.600048638	####	0	ARNTL	protein_coding	aryl hydroca	11	13276652	13387266
ENSG0000027178	2.138941636	####	0	AL118558.3	lncRNA	novel transc	14	101948347	101949425
ENSG0000023575	-2.191451811	####	0	KIAA0040	protein_coding	KIAA0040 [S	1	175156986	175192999
ENSG0000019882	0.7533866	####	0	ARHGAP11	protein_coding	Rho GTPase	15	32615144	32639941
ENSG0000018767	-1.079888118	####	0	B3GLCT	protein_coding	beta 3-gluc	13	31199975	31332276
ENSG0000027670	-0.281464617	####	0	MIF	protein_coding	macrophage	CHR_HSCHR	23894004	23895227
ENSG0000016829	0.485762437	####	0	PDHB	protein_coding	pyruvate de	3	58427630	58433857
ENSG0000022756	-1.280476487	####	0	NFKBIL1	protein_coding	NFKB inhibit	CHR_HSCHR	31586326	31598283
ENSG0000021564	-0.605480541	####	0	TRIM27	protein_coding	tripartite mo	CHR_HSCHR	28902991	28923978

ENSG0000027276	1.823188664	####	0	JMJD1C-AS	lncRNA	JMJD1C ant	10	63465229	63466563
ENSG0000023698	-2.342731471	####	0	AC130710.1	lncRNA	novel transc	2	16085222	16105841
ENSG0000026039	-2.979964062	####	0	AC022336.2	lncRNA	novel transc	3	124723788	124726325
ENSG0000016416	-0.307638961	####	0	OTUD4	protein_codinc	OTU deubic	4	145110838	145180589
ENSG0000016870	0.593695326	####	0	TMEM208	protein_codinc	transmembı	16	67227103	67229278
ENSG0000011965	0.775380852	####	0	IFT43	protein_codinc	intraflagella	14	75902136	76084585
ENSG0000016378	0.458460583	####	0	SNRK	protein_codinc	SNF related	3	43286512	43424764
ENSG0000010042	0.259093251	####	0	ZBED4	protein_codinc	zinc finger B	22	49853844	49890080
ENSG0000022983	0.643965921	####	0	PET100	protein_codinc	PET100 cytc	19	7629793	7631956
ENSG0000018579	0.708133534	####	0	WDR53	protein_codinc	WD repeat c	3	196554177	196568674
ENSG0000027822	0.605532872	####	0	CLN8	protein_codinc	CLN8 transr	CHR_HSCHR	1755340	1786132
ENSG0000028015	-2.753309629	####	0	AC009078.1	TEC	TEC	16	75245994	75250077
ENSG0000022441	0.831098207	####	0	HSP90AA2P	processed_pse	heat shock p	11	27888838	27891033
ENSG0000016368	-0.414753131	####	0	SLMAP	protein_codinc	sarcolemma	3	57755450	57930003
ENSG0000026096	-0.860277444	####	0	AP001486.2	lncRNA	novel transc	11	103050687	103055799
ENSG0000009131	0.570447347	####	0	CMTM6	protein_codinc	CKLF like M	3	32481312	32502852
ENSG0000013324	0.2988298	####	0	BTBD2	protein_codinc	BTB domain	19	1985438	2034881
ENSG0000022598	-2.244031279	####	0	LAMP5-AS1	lncRNA	LAMP5 antis	20	9505180	9514998
ENSG0000006505	-1.156785531	####	0	SLC9A3R2	protein_codinc	SLC9A3 reg	16	2025356	2039026
ENSG0000006802	-0.677959673	####	0	RASSF1	protein_codinc	Ras associat	3	50329782	50340980
ENSG0000012601	-0.234124616	####	0	KDM5C	protein_codinc	lysine deme	X	53191321	53225422
ENSG0000013084	-0.53403399	####	0	ZNF331	protein_codinc	zinc finger p	19	53519527	53580269
ENSG0000026738	1.190739066	####	0	AC011447.1	lncRNA	novel transc	19	20122569	20321305
ENSG0000015123	0.293189624	####	0	TWF1	protein_codinc	twinfilin acti	12	43793723	43806328
ENSG0000011950	-1.734949324	####	0	NR4A3	protein_codinc	nuclear rece	9	99821855	99866891
ENSG0000025994	1.173205481	####	0	AL050341.2	lncRNA	novel transc	1	40256427	40257967
ENSG0000016496	1.024889349	####	0	RPP25L	protein_codinc	ribonucleas	9	34610486	34612104
ENSG0000015823	-0.711812044	####	0	FAIM	protein_codinc	Fas apoptot	3	138608606	138633376
ENSG0000025668	1.102698428	####	0	ZNF350	protein_codinc	zinc finger p	19	51964340	51986856
ENSG0000021385	0.477277768	####	0	EMP2	protein_codinc	epithelial m	16	10528422	10580632
ENSG0000009640	-0.291703163	####	0	CDC5L	protein_codinc	cell division	6	44387706	44450425
ENSG0000014087	1.154562396	####	0	NUDT7	protein_codinc	nudix hydro	16	77722492	77742260
ENSG0000022675	2.819992929	####	0	AC007365.1	lncRNA	novel transc	2	64644612	64646698
ENSG0000007978	-0.171843145	####	0	DDX1	protein_codinc	DEAD-box h	2	15591178	15631111
ENSG0000015719	0.408998859	####	0	NECAP2	protein_codinc	NECAP endo	1	16440721	16460078
ENSG0000012599	-0.281004562	####	0	ERGIC3	protein_codinc	ERGIC and g	20	35542038	35557634
ENSG0000019810	1.65547917	####	0	CHSY3	protein_codinc	chondroitin	5	129904465	130186634
ENSG0000017275	-4.439787448	####	0	COL6A5	protein_codinc	collagen typ	3	130345516	130484844
ENSG0000023755	-0.222965088	####	0	AC243919.1	transcribed_pr	ribosomal p	15	82372196	82372912
ENSG0000016238	0.536660252	####	0	CZIB	protein_codinc	CXXC motif	1	53214099	53220634
ENSG0000015421	-0.432707479	####	0	PITPNC1	protein_codinc	phosphatidy	17	67377281	67697261
ENSG0000022354	1.194375218	####	0	LINC00630	lncRNA	long interge	X	102769158	102885406
ENSG0000008018	0.474297617	####	0	SLC35C2	protein_codinc	solute carrie	20	46345980	46364458
ENSG0000020471	-0.547651115	####	0	TRIM27	protein_codinc	tripartite mo	6	28903002	28923988
ENSG0000013968	-0.304070415	####	0	RB1	protein_codinc	RB transcrip	13	48303744	48599436
ENSG0000018304	0.391009465	####	0	ABAT	protein_codinc	4-aminobut	16	8674596	8784575
ENSG0000024864	0.505973864	####	0	RBM14-RBM	protein_codinc	RBM14-RBM	11	66616626	66646469
ENSG0000012891	0.270357027	####	0	ICE2	protein_codinc	interactor o	15	60419609	60479160
ENSG0000024229	0.786482823	####	0	STAG3L5P	transcribed_un	stromal anti	7	100336079	100351900
ENSG0000017927	-0.358492497	####	0	GADD45GII	protein_codinc	GADD45G ir	19	12953119	12957223
ENSG0000018187	0.637124128	####	0	IBA57	protein_codinc	iron-sulfur c	1	228165804	228182257
ENSG0000019610	-1.684279821	####	0	SPOCK3	protein_codinc	SPARC (oste	4	166733384	167234796
ENSG0000012140	0.866471966	####	0	ZNF549	protein_codinc	zinc finger p	19	57527325	57557542
ENSG0000019818	0.708135516	####	0	ZNF334	protein_codinc	zinc finger p	20	46499630	46513559
ENSG0000024852	0.186781924	####	0	MTATP6P1	unprocessed_p	MT-ATP6 ps	1	633696	634376
ENSG0000006665	0.291676717	####	0	THUMPD1	protein_codinc	THUMP dor	16	20702816	20742084
ENSG0000010488	0.583095477	####	0	ERCC2	protein_codinc	ERCC excisio	19	45349837	45370918
ENSG0000016416	0.571400659	####	0	LSM6	protein_codinc	LSM6 homo	4	146175703	146200000

ENSG0000010678	-0.405048692	####	0	TRIM14	protein_coding	tripartite mo	9	98069275	98119222
ENSG0000006560	0.263621669	####	0	SNAP91	protein_coding	synaptosom	6	83552880	83709691
ENSG0000023471	2.01489013	####	0	NPIPB2	protein_coding	nuclear pore	16	11927259	11976643
ENSG0000025012	0.41357571	####	0	PCDHA10	protein_coding	protocadhe	5	140855883	141012347
ENSG0000017620	0.529561384	####	0	ATAD5	protein_coding	ATPase fam	17	30831966	30895869
ENSG0000001356	-0.835622397	####	0	DNASE1L1	protein_coding	deoxyribonu	X	154401236	154412112
ENSG0000002963	-0.703004948	####	0	TFB1M	protein_coding	transcription	6	155256134	155314493
ENSG0000011933	0.444567346	####	0	WDR34	protein_coding	WD repeat	9	128633661	128656787
ENSG0000018677	0.953381804	####	0	ZNF732	protein_coding	zinc finger p	4	270675	305474
ENSG0000011430	0.392558408	####	0	PRKAR2A	protein_coding	protein kina	3	48744597	48847874
ENSG0000018267	0.175912074	####	0	TTC3	protein_coding	tetratricope	21	37073226	37203112
ENSG0000027716	0.623691123	####	0	PIGW	protein_coding	phosphatidy	17	36534987	36539310
ENSG0000018416	-1.190894576	####	0	ADRA2C	protein_coding	adrenocepto	4	3766348	3768526
ENSG0000019798	0.603415981	####	0	C1orf122	protein_coding	chromosom	1	37806979	37809454
ENSG0000018581	0.534148035	####	0	PCYT2	protein_coding	phosphate c	17	81900958	81911432
ENSG0000007515	0.187782633	####	0	EIF4G3	protein_coding	eukaryotic t	1	20806292	21176888
ENSG0000026091	0.661388353	####	0	AL158212.3	lncRNA	novel transc	10	112823490	112827726
ENSG0000012347	0.340476665	####	0	STIL	protein_coding	STIL centrio	1	47250139	47314147
ENSG0000008910	-2.377745051	####	0	CFAP61	protein_coding	cilia and flag	20	20052514	20360703
ENSG0000027367	-0.225256502	####	0	AC011295.1	transcribed_pr	ribosomal p	CHR_HSCHR	82451282	82451992
ENSG0000027558	2.032211737	####	0	AL031670.1	lncRNA	novel transc	20	3921279	3923400
ENSG0000025948	1.260862423	####	0	AC023355.1	lncRNA	novel transc	15	48312353	48331856
ENSG0000014764	-7.084063835	####	0	DPYS	protein_coding	dihydropyri	8	104330324	104467055
ENSG0000022646	0.869045395	####	0	AGPAT1	protein_coding	1-acylglycer	CHR_HSCHR	32116683	32126567
ENSG0000022949	0.424146255	####	0	DDX39B	protein_coding	DExD-box h	CHR_HSCHR	31606569	31622924
ENSG0000011093	0.316032442	####	0	CAMKK2	protein_coding	calcium/calr	12	121237675	121298308
ENSG0000015926	-0.606463808	####	0	HLCS	protein_coding	holocarboxy	21	36748626	36990236
ENSG0000027952	-1.482053831	####	0	NA	NA	NA	NA	NA	NA
ENSG0000018594	0.503432666	####	0	RNPC3	protein_coding	RNA binding	1	103525691	103555239
ENSG0000010451	-0.174565867	####	0	UBR5	protein_coding	ubiquitin pr	8	102252273	102412759
ENSG0000013331	0.456511662	####	0	CNDP2	protein_coding	carnosine di	18	74495816	74523454
ENSG0000018376	0.835567481	####	0	TRAIP	protein_coding	TRAF intera	3	49828601	49856574
ENSG0000012826	-0.537726896	####	0	GNAZ	protein_coding	G protein su	22	23070519	23125032
ENSG0000013143	-0.553278945	####	0	PDLIM4	protein_coding	PDZ and LIM	5	132257696	132273454
ENSG0000011207	0.331790554	####	0	STK38	protein_coding	serine/threo	6	36493892	36547479
ENSG0000016968	0.469680609	####	0	CENPX	protein_coding	centromere	17	82018702	82024107
ENSG0000012601	0.502481353	####	0	AMOT	protein_coding	angiomotin	X	112774503	112840815
ENSG0000010157	-0.797761498	####	0	METTL4	protein_coding	methyltrans	18	2537525	2571509
ENSG0000013031	-0.549538565	####	0	MRPL34	protein_coding	mitochondr	19	17292609	17306843
ENSG0000012334	-0.257906484	####	0	PFDN5	protein_coding	prefoldin su	12	53295291	53299450
ENSG0000018614	-0.373387917	####	0	POLR3C	protein_coding	RNA polyme	1	145824088	145844402
ENSG0000020555	2.823255659	####	0	CHKB-DT	lncRNA	CHKB diverg	22	50583026	50595634
ENSG0000013929	0.398530264	####	0	TMEM19	protein_coding	transmembr	12	71686082	71705047
ENSG0000014835	0.248632785	####	0	GPR107	protein_coding	G protein-c	9	130053426	130140169
ENSG0000013750	3.633920641	####	0	SYTL2	protein_coding	synaptotagr	11	85694224	85811159
ENSG0000010342	-0.405634534	####	0	BFAR	protein_coding	bifunctional	16	14632931	14669236
ENSG0000013577	0.380694405	####	0	ABCB10	protein_coding	ATP binding	1	229516582	229558707
ENSG0000013867	-0.189136624	####	0	SEC31A	protein_coding	SEC31 hom	4	82818509	82901166
ENSG0000018377	-1.414639115	####	0	ZNF703	protein_coding	zinc finger p	8	37695782	37700019
ENSG0000013263	-0.646187537	####	0	PCED1A	protein_coding	PC-esterase	20	2835314	2841190
ENSG0000021396	0.966146613	####	0	AC019080.1	lncRNA	novel transc	2	177283508	177392691
ENSG0000017945	0.328428379	####	0	KLHL28	protein_coding	kelch like fa	14	44924324	45042322
ENSG0000011635	0.670177952	####	0	MECR	protein_coding	mitochondr	1	29192657	29230942
ENSG0000013894	0.345554707	####	0	RNF185	protein_coding	ring finger p	22	31160182	31207019
ENSG0000019804	-0.277975872	####	0	ZNF84	protein_coding	zinc finger p	12	133037292	133063304
ENSG0000013418	-0.2202697	####	0	PRPF38B	protein_coding	pre-mRNA	1	108692310	108702928
ENSG0000006454	-1.652332817	####	0	LPAR2	protein_coding	lysophospha	19	19623655	19628930
ENSG0000013025	0.23678191	####	0	RPL36	protein_coding	ribosomal p	19	5674947	5691875

ENSG0000025119	-0.832507405	####	0	ZNF674	protein_coding	zinc finger p	X	46497727	46545457
ENSG0000015397	0.558306452	####	0	ZUP1	protein_coding	zinc finger c	6	116635618	116668794
ENSG0000011575	-0.401575315	####	0	HPCAL1	protein_coding	hippocalcin	2	10302889	10427617
ENSG0000012296	-0.352434247	####	0	RBM19	protein_coding	RNA binding	12	113816738	113966325
ENSG0000012968	0.49782788	####	0	MAP7D3	protein_coding	MAP7 doma	X	136213220	136256482
ENSG0000014339	0.298825278	####	0	PI4KB	protein_coding	phosphatidy	1	151291797	151327715
ENSG0000012535	-0.433814412	####	0	NDUFA1	protein_coding	NADH:ubiqu	X	119871832	119876662
ENSG0000018042	1.262107696	####	0	HARBI1	protein_coding	harbinger tr	11	46602861	46617909
ENSG0000003568	-0.310505992	####	0	ADSS2	protein_coding	adenylosucc	1	244408494	244451909
ENSG0000010517	1.121344063	####	0	CCNE1	protein_coding	cyclin E1 [Sc	19	29811991	29824312
ENSG0000027426	5.171601886	####	0	NA	NA	NA	NA	NA	NA
ENSG0000022354	0.85431239	####	0	ZNF844	protein_coding	zinc finger p	19	12064731	12081565
ENSG0000022895	-0.895504492	####	0	SATB1-AS1	lncRNA	SATB1 antis	3	18445024	18920401
ENSG0000010275	-0.346913328	####	0	KPNA3	protein_coding	karyopherin	13	49699320	49792682
ENSG0000014757	2.162724466	####	0	ADHFE1	protein_coding	alcohol deh	8	66432492	66468907
ENSG0000015693	0.339701188	####	0	VPS8	protein_coding	VPS8 subun	3	184812143	185052614
ENSG0000015778	-2.666213608	####	0	CABP1	protein_coding	calcium bin	12	120640552	120667324
ENSG0000011502	0.227052834	####	0	PIKFYVE	protein_coding	phosphoino	2	208266255	208358746
ENSG0000011548	0.390641974	####	0	GGCX	protein_coding	gamma-glu	2	85544720	85561547
ENSG0000010634	-0.300350197	####	0	RBM28	protein_coding	RNA binding	7	128297685	128343908
ENSG0000008393	-0.71387013	####	0	CHMP2B	protein_coding	charged mu	3	87227271	87255556
ENSG0000016528	-0.303738631	####	0	STOML2	protein_coding	stomatin like	9	35099776	35103195
ENSG0000010186	0.347951637	####	0	POLA1	protein_coding	DNA polym	X	24693909	24996986
ENSG0000012212	-2.515609415	####	0	XPNPEP2	protein_coding	X-prolyl am	X	129738974	129769536
ENSG0000023651	0.717435485	####	0	ZBTB9	protein_coding	zinc finger a	CHR_HSCHR	33594767	33597736
ENSG0000011306	0.384591561	####	0	PFDN1	protein_coding	prefoldin su	5	140245035	140303113
ENSG0000012435	0.317097889	####	0	STAMBP	protein_coding	STAM bindi	2	73828916	73873659
ENSG0000016434	-4.652219822	####	0	KLKB1	protein_coding	kallikrein B1	4	186208979	186258471
ENSG0000016384	-0.282202691	####	0	ZNF148	protein_coding	zinc finger p	3	125225669	125375325
ENSG0000010314	-0.436955089	####	0	NPRL3	protein_coding	NPR3 like, G	16	84271	138677
ENSG0000027153	4.2649954	####	0	LINC02427	lncRNA	long interge	4	184503271	184537626
ENSG0000010925	0.80814828	####	0	NMU	protein_coding	neuromedin	4	55595229	55636698
ENSG0000011647	0.328072528	####	0	HDAC1	protein_coding	histone dea	1	32292083	32333635
ENSG0000003592	0.240674753	####	0	RFC1	protein_coding	replication f	4	39287456	39366375
ENSG0000027801	-3.00882485	####	0	AL031658.2	lncRNA	novel transc	20	31970181	31970831
ENSG0000013172	1.069656834	####	0	IL13RA1	protein_coding	interleukin 1	X	118727133	118794535
ENSG0000014713	0.307092242	####	0	TAF1	protein_coding	TATA-box b	X	71366264	71532374
ENSG0000023059	-1.209135105	####	0	FTX	lncRNA	FTX transcri	X	73940435	74293574
ENSG0000012712	-1.066896538	####	0	HIVEP3	protein_coding	HIVEP zinc f	1	41506365	42035925
ENSG0000013673	-0.214842423	####	0	UGGT1	protein_coding	UDP-glucos	2	128091200	128195677
ENSG0000010441	0.719896572	####	0	NDRG1	protein_coding	N-myc dow	8	133237175	133302022
ENSG0000017558	-0.235400556	####	0	RAB6A	protein_coding	RAB6A, mer	11	73675638	73761137
ENSG0000015155	0.321792773	####	0	QDPR	protein_coding	quinoid dihy	4	17460261	17512206
ENSG0000008711	0.397894769	####	0	PIGS	protein_coding	phosphatidy	17	28553383	28571794
ENSG0000015215	2.803491881	####	0	TMEM178A	protein_coding	transmembr	2	39664982	39717963
ENSG0000018521	-1.249272107	####	0	TNFAIP2	protein_coding	TNF alpha ir	14	103121476	103137439
ENSG0000016976	-0.385863181	####	0	TAPT1	protein_coding	transmembr	4	16160505	16227410
ENSG0000010027	0.645095797	####	0	TTLL1	protein_coding	tubulin tyros	22	43039516	43089419
ENSG0000023715	-1.176930483	####	0	IER3	protein_coding	immediate e	CHR_HSCHR	30733058	30734413
ENSG0000018660	-0.864347404	####	0	HPDL	protein_coding	4-hydroxyp	1	45326895	45328679
ENSG0000019666	0.451117466	####	0	TECPR2	protein_coding	tectonin bet	14	102362941	102502477
ENSG0000017947	1.374662321	####	0	C14orf28	protein_coding	chromosom	14	44897275	44907257
ENSG0000003471	0.319587582	####	0	GABARAPL	protein_coding	GABA type	16	75566375	75577881
ENSG0000017083	-0.372929359	####	0	PPM1D	protein_coding	protein pho	17	60600183	60666280
ENSG0000016071	-0.162310315	####	0	ADAR	protein_coding	adenosine c	1	154582057	154628013
ENSG0000014890	-0.946464673	####	0	RGS10	protein_coding	regulator of	10	119499817	119542719
ENSG0000015109	0.87597244	####	0	NGLY1	protein_coding	N-glycanase	3	25718944	25790039
ENSG0000025662	1.041623162	####	0	ZBTB11-AS	lncRNA	ZBTB11 anti	3	101676475	101679217

ENSG0000014918	0.201101521	####	0	CELF1	protein_codinç	CUGBP Elav	11	47465933	47565569
ENSG0000017172	0.408740874	####	0	GPHN	protein_codinç	gephyrin [So	14	66507407	67181803
ENSG0000013118	-0.836924273	####	0	PRR7	protein_codinç	proline rich	5	177446445	177456286
ENSG0000027114	0.894124653	####	0	ARMCX5-G	lncRNA	ARMCX5-G	X	102599512	102714671
ENSG0000006419	1.944956435	####	0	DLX3	protein_codinç	distal-less h	17	49990005	49995224
ENSG0000014310	0.3511902	####	0	PSMA5	protein_codinç	proteasome	1	109399042	109426448
ENSG0000012621	0.707525868	####	0	XRCC3	protein_codinç	X-ray repair	14	103697609	103715504
ENSG0000027607	0.736189012	####	0	CU633904.:	lncRNA	uncharacter	21	7430659	7469007
ENSG0000017823	0.365305516	####	0	GALNT11	protein_codinç	polypeptide	7	152025674	152122340
ENSG0000016767	0.308627645	####	0	CHAF1A	protein_codinç	chromatin a	19	4402640	4445018
ENSG0000009023	-0.693716639	####	0	YPEL3	protein_codinç	yippee like :	16	30092314	30096915
ENSG0000011703	-0.298416722	####	0	ETV3	protein_codinç	ETS variant	1	157121191	157138474
ENSG0000017728	2.320170826	####	0	FZD8	protein_codinç	frizzled clas	10	35638249	35642278
ENSG0000023265	2.199353311	####	0	GOLGA8N	protein_codinç	golgin A8 fa	15	32593456	32607310
ENSG0000013619	0.83938853	####	0	C7orf25	protein_codinç	chromosom	7	42908726	42912305
ENSG0000000812	0.467103667	####	0	CDK11A	protein_codinç	cyclin deper	1	1702379	1724357
ENSG0000019697	-0.882549954	####	0	ANXA4	protein_codinç	annexin A4	2	69644425	69827112
ENSG0000019830	0.433357552	####	0	PEG3	protein_codinç	paternally e	19	56810077	56840728
ENSG0000014549	0.161281075	####	0	MARCHF6	protein_codinç	membrane	5	10353695	10440388
ENSG0000019892	1.120328812	####	0	NOS1AP	protein_codinç	nitric oxide	1	162069691	162370475
ENSG0000017553	2.134172976	####	0	LIPT2	protein_codinç	lipoyl(octan	11	74490519	74493724
ENSG0000027324	1.120383414	####	0	AC097376.:	lncRNA	novel transc	4	139411927	139454034
ENSG0000026724	1.86978447	####	0	AC012615.(	lncRNA	novel transc	19	1822089	1824542
ENSG0000005957	-0.232743749	####	0	ALDH18A1	protein_codinç	aldehyde de	10	95605941	95656711
ENSG0000009000	-0.831848292	1E-04	0	LTBP4	protein_codinç	latent transf	19	40592883	40629818
ENSG0000027064	0.255688131	1E-04	0	TAF15	protein_codinç	TATA-box b	17	35713791	35864615
ENSG0000010720	1.069913789	1E-04	0	DDX58	protein_codinç	DExD/H-bo	9	32455302	32526208
ENSG0000016640	-1.059708291	1E-04	0	RIC3	protein_codinç	RIC3 acetylc	11	8106056	8169055
ENSG0000017510	-1.177022849	1E-04	0	TVP23C	protein_codinç	trans-golgi	17	15502264	15563595
ENSG0000012581	-0.350162839	1E-04	0	GZF1	protein_codinç	GDNF induc	20	23362182	23373062
ENSG0000025831	0.889013041	1E-04	0	C17orf49	protein_codinç	chromosom	17	7014495	7017525
ENSG0000008270	0.228184447	1E-04	0	GSK3B	protein_codinç	glycogen sy	3	119821321	120094447
ENSG0000014127	-0.250556148	1E-04	0	NPEPPS	protein_codinç	aminopeptic	17	47522942	47623276
ENSG0000017484	0.590283196	1E-04	0	GLMN	protein_codinç	glomulin, FK	1	92246402	92298987
ENSG0000010088	-0.292221658	1E-04	0	PCK2	protein_codinç	phosphoenc	14	24094053	24110598
ENSG0000005377	-0.30164347	1E-04	0	AP5M1	protein_codinç	adaptor rela	14	57268924	57298742
ENSG0000016021	-0.361657403	1E-04	0	TRAPPC10	protein_codinç	trafficking p	21	44012309	44106552
ENSG0000014402	-0.346656005	1E-04	0	MRPS5	protein_codinç	mitochondr	2	95085369	95149434
ENSG0000016746	0.345496946	1E-04	0	RAB8A	protein_codinç	RAB8A, mer	19	16111889	16134234
ENSG0000011355	-1.485296258	1E-04	0	PCDH12	protein_codinç	protocadhe	5	141943581	141969741
ENSG0000017578	-1.534452597	1E-04	0	PRIMA1	protein_codinç	proline rich	14	93718298	93788485
ENSG0000017823	-1.224044274	1E-04	0	TMEM151B	protein_codinç	transmembı	6	44270450	44307506
ENSG0000013277	-0.625165828	1E-04	0	TOE1	protein_codinç	target of EG	1	45340052	45343973
ENSG0000018131	0.359411176	1E-04	0	ZNF322	protein_codinç	zinc finger p	6	26634383	26659752
ENSG0000015177	-0.262233907	1E-04	0	NBAS	protein_codinç	NBAS subur	2	15166914	15561334
ENSG0000009152	-0.200493627	1E-04	0	CDV3	protein_codinç	CDV3 homc	3	133573686	133590261
ENSG0000000602	0.840300075	1E-04	0	OSBPL7	protein_codinç	oxysterol bi	17	47807372	47821834
ENSG0000017178	-2.194411226	1E-04	0	NHLH1	protein_codinç	nescient hel	1	160367071	160372846
ENSG0000026406	-6.950403483	1E-04	0	AC005291.:	lncRNA	novel transc	17	10320392	10341458
ENSG0000009532	0.837655471	1E-04	0	CRAT	protein_codinç	carnitine O-	9	129094794	129111189
ENSG0000026027	2.362604066	1E-04	0	AC068338.:	lncRNA	novel transc	15	75368155	75369584
ENSG0000015194	-1.949670891	1E-04	0	GLT1D1	protein_codinç	glycosyltran	12	128853427	128984968
ENSG0000027144	-4.532956779	1E-04	0	MMP28	protein_codinç	matrix meta	17	35756249	35795707
ENSG0000000873	-0.342541955	1E-04	0	MAPK8IP2	protein_codinç	mitogen-ac	22	50600793	50613981
ENSG0000013306	-0.772069769	1E-04	0	TMCC2	protein_codinç	transmembı	1	205227946	205285632
ENSG0000027853	1.779440359	1E-04	0	DHRS11	protein_codinç	dehydroger	17	36591879	36600804
ENSG0000000645	-0.247904015	1E-04	0	KDM7A	protein_codinç	lysine deme	7	140084746	140176983
ENSG0000006901	-0.959319249	1E-04	0	PITX1	protein_codinç	paired like h	5	135027734	135034813

ENSG0000010737	0.204281024	1E-04	0	ZFAND5	protein_coding	zinc finger A	9	72351413	72365235
ENSG0000008299	-0.345342228	1E-04	0	RNF13	protein_coding	ring finger p	3	149812770	149962139
ENSG0000007280	-0.248294621	1E-04	0	FBXW11	protein_coding	F-box and W	5	171861549	172006873
ENSG0000012193	-0.389871905	1E-04	0	LRIF1	protein_coding	ligand depe	1	110947190	110963965
ENSG0000018508	0.486881564	1E-04	0	INTS5	protein_coding	integrator c	11	62646848	62653302
ENSG0000007396	0.480537862	1E-04	0	NSF	protein_coding	N-ethylmale	17	46590669	46757464
ENSG0000014154	0.822418501	1E-04	0	RAB40B	protein_coding	RAB40B, me	17	82654973	82698698
ENSG0000025342	-4.171320111	1E-04	0	NA	NA	NA	NA	NA	NA
ENSG0000019626	1.107499304	1E-04	0	ZNF471	protein_coding	zinc finger p	19	56507850	56530221
ENSG0000011287	0.642646898	1E-04	0	NUDT12	protein_coding	nudix hydro	5	103548855	103562790
ENSG0000019725	-1.004546623	1E-04	0	EIF4BP6	processed_pse	eukaryotic t	7	104667749	104669576
ENSG0000012422	0.307586604	1E-04	0	DDX27	protein_coding	DEAD-box I	20	49219295	49244077
ENSG0000014301	0.324301402	1E-04	0	LMO4	protein_coding	LIM domain	1	87328880	87348923
ENSG0000026991	-1.726829567	1E-04	0	AF131215.6	lncRNA	novel transc	8	11104691	11106704
ENSG0000010540	-0.453967147	1E-04	0	RABAC1	protein_coding	Rab accepto	19	41956681	41959321
ENSG0000013859	0.249653087	1E-04	0	TMOD3	protein_coding	tropomodul	15	51829628	51947295
ENSG0000012645	0.759142472	1E-04	0	IRF3	protein_coding	interferon re	19	49659569	49665875
ENSG0000024205	-4.077625028	1E-04	0	RPL10P7	processed_pse	ribosomal p	3	119635526	119636150
ENSG0000023734	-0.902154564	1E-04	0	PBX2	protein_coding	PBX homeo	CHR_HSCHR	32133204	32138654
ENSG0000012051	-0.763549202	1E-04	0	SLC10A7	protein_coding	solute carrie	4	146253975	146521964
ENSG0000022355	-1.611775671	1E-04	0	AC073136.1	processed_pse	exportin, tR	7	56288230	56291425
ENSG0000017583	-0.334995749	1E-04	0	ETV4	protein_coding	ETS variant	17	43527844	43579620
ENSG0000014542	-4.04606124	1E-04	0	RNF175	protein_coding	ring finger p	4	153710160	153760024
ENSG0000010108	0.360430401	1E-04	0	RAB5IF	protein_coding	RAB5 intera	20	36605779	36612557
ENSG0000014815	0.421556458	1E-04	0	UGCG	protein_coding	UDP-glucos	9	111896814	111935369
ENSG0000013680	0.407216395	1E-04	0	CDK9	protein_coding	cyclin deper	9	127785679	127790792
ENSG0000023073	0.624696701	1E-04	0	AC092171.2	lncRNA	novel transc	7	5475804	5479811
ENSG0000012575	-0.356148754	1E-04	0	SYMPK	protein_coding	symplekin [S	19	45815410	45863194
ENSG0000026265	-2.086872846	1E-04	0	SPON1	protein_coding	spondin 1 [S	11	13962723	14268133
ENSG0000001869	-0.486419877	1E-04	0	TTC27	protein_coding	tetratricope	2	32628032	32821051
ENSG0000015602	0.468416575	1E-04	0	MCU	protein_coding	mitochondr	10	72692131	72887694
ENSG0000006580	0.330594812	1E-04	0	ASB1	protein_coding	ankyrin repe	2	238426742	238452250
ENSG0000023729	-0.594557892	1E-04	0	SMG1P1	transcribed_un	SMG1 pseu	16	22437008	22492220
ENSG0000026032	2.044746227	1E-04	0	AC007541.1	lncRNA	novel transc	12	106954029	106955497
ENSG0000021333	-0.437267108	1E-04	0	QTRT1	protein_coding	queuine tRN	19	10701430	10713437
ENSG0000013177	0.571061381	1E-04	0	PEX11B	protein_coding	peroxisoma	1	145911350	145918717
ENSG0000014700	2.544465249	1E-04	0	CLTRN	protein_coding	collectrin, ar	X	15627318	15675012
ENSG0000012251	0.332062687	1E-04	0	ZMIZ2	protein_coding	zinc finger M	7	44748581	44769881
ENSG0000015518	0.238156937	1E-04	0	AGPAT5	protein_coding	1-acylglycer	8	6708642	6761503
ENSG0000012332	-2.802319369	1E-04	0	ARHGAP9	protein_coding	Rho GTPase	12	57472264	57488814
ENSG0000016693	-0.506482752	1E-04	0	DIS3L	protein_coding	DIS3 like ex	15	66293217	66333898
ENSG0000009243	-0.245141206	1E-04	0	TRPM7	protein_coding	transient rec	15	50552473	50686797
ENSG0000006530	-0.362007852	1E-04	0	TRAM2	protein_coding	translocatio	6	52497408	52577060
ENSG0000011265	0.350393539	1E-04	0	SRF	protein_coding	serum respo	6	43171269	43181506
ENSG0000014438	-0.160660105	1E-04	0	HSPD1	protein_coding	heat shock p	2	197486584	197516737
ENSG0000010529	0.427171507	1E-04	0	CACTIN	protein_coding	cactin, splice	19	3610645	3626815
ENSG0000011722	0.327206122	1E-04	0	RBBP5	protein_coding	RB binding p	1	205086142	205122015
ENSG0000017253	-0.591795727	1E-04	0	BANP	protein_coding	BTG3 associ	16	87949244	88077318
ENSG0000010026	-0.287934303	1E-04	0	PACSIN2	protein_coding	protein kina	22	42835412	43015149
ENSG0000017560	0.493587194	1E-04	0	CCDC85B	protein_coding	coiled-coil c	11	65890673	65891635
ENSG0000026146	-1.514113269	1E-04	0	AC009690.2	lncRNA	novel transc	15	72278867	72351794
ENSG0000022858	-1.665032396	1E-04	0	TAP2	protein_coding	transporter	CHR_HSCHR	32744153	32761098
ENSG0000019790	-1.093242632	1E-04	0	TEAD4	protein_coding	TEA domain	12	2959330	3040676
ENSG0000006994	-1.370752188	1E-04	0	PIGB	protein_coding	phosphatidy	15	55318960	55355648
ENSG0000000725	0.847888254	1E-04	0	TRAPPC6A	protein_coding	trafficking p	19	45162928	45178237
ENSG0000012754	0.305380286	1E-04	0	UQCR11	protein_coding	ubiquinol-cy	19	1597169	1605473
ENSG0000016285	-0.317420803	1E-04	0	CNST	protein_coding	consortin, co	1	246566444	246668595
ENSG0000014010	0.875050827	1E-04	0	CLBA1	protein_coding	clathrin binc	14	104985775	105010482

ENSG0000027125	-4.275612592	1E-04	0	AL683887.1	lncRNA	novel transc	1	95743096	95759470
ENSG0000016840	-2.993973207	1E-04	0	MLKL	protein_coding	mixed lineag	16	74671855	74700960
ENSG0000022683	2.388683562	1E-04	0	AC092164.1	lncRNA	novel transc	2	28707511	28751722
ENSG0000011977	-0.269595726	1E-04	0	ATAD2B	protein_coding	ATPase fam	2	23748664	23927123
ENSG0000018471	-2.651683622	1E-04	0	RNLS	protein_coding	renalase, FA	10	88273864	88584530
ENSG0000009506	-0.678146625	1E-04	0	HOOK2	protein_coding	hook microt	19	12763003	12872740
ENSG0000019628	-0.741496033	1E-04	0	SUPT3H	protein_coding	SPT3 homol	6	44809317	45377953
ENSG0000026029	-0.9632425	1E-04	0	AC106820.4	lncRNA	novel transc	16	2476558	2482173
ENSG0000016825	0.726886843	1E-04	0	POLR2J3	protein_coding	RNA polyme	7	102537918	102572653
ENSG0000012350	-0.233789844	1E-04	0	AMD1	protein_coding	adenosylme	6	110874770	110898879
ENSG0000018771	-1.059436133	1E-04	0	SLC18A3	protein_coding	solute carrie	10	49610310	49612720
ENSG0000009300	0.201890456	1E-04	0	NUP50	protein_coding	nucleoporin	22	45163925	45188017
ENSG0000016913	-0.45835736	1E-04	0	ZNF354A	protein_coding	zinc finger p	5	178711512	178730659
ENSG0000013817	0.362611451	1E-04	0	ARL3	protein_coding	ADP ribosyl	10	102673731	102714397
ENSG0000022810	1.741734208	1E-04	0	MELTF-AS1	lncRNA	MELTF antis	3	196999460	197004744
ENSG0000010203	0.365986938	1E-04	0	NAA10	protein_coding	N-alpha-ac	X	153929225	153935080
ENSG0000026188	-2.709297047	1E-04	0	AC108134.2	lncRNA	novel transc	16	3156736	3157483
ENSG0000016292	0.549030977	1E-04	0	KIAA1841	protein_coding	KIAA1841 [S	2	61065871	61138034
ENSG0000016736	-1.618004553	1E-04	0	FN3K	protein_coding	fructosamin	17	82735615	82751196
ENSG0000018467	0.443649645	1E-04	0	AMER1	protein_coding	APC membr	X	64185117	64205708
ENSG0000017759	0.616973522	1E-04	0	PIDD1	protein_coding	p53-induce	11	799179	809753
ENSG0000012609	0.61697527	1E-04	0	ST3GAL3	protein_coding	ST3 beta-ga	1	43705824	43931165
ENSG0000018214	0.575095273	1E-04	0	ZNF708	protein_coding	zinc finger p	19	21291160	21329425
ENSG0000009925	0.373708226	1E-04	0	PRTFDC1	protein_coding	phosphoribo	10	24848614	24952606
ENSG0000017196	-1.52049111	1E-04	0	DRC3	protein_coding	dynein regu	17	17972813	18016889
ENSG0000023709	0.610549942	1E-04	0	MDC1	protein_coding	mediator of	CHR_HSCHR	30778290	30796371
ENSG0000009980	-0.3514461	1E-04	0	CDC34	protein_coding	cell division	19	531760	542092
ENSG0000012373	0.485343474	1E-04	0	PLA2G12A	protein_coding	phospholipa	4	109709989	109730070
ENSG0000027679	1.700109021	1E-04	0	AC092117.1	lncRNA	novel transc	16	2777319	2780568
ENSG0000001528	2.050124809	1E-04	0	WAS	protein_coding	WASP actin	X	48676596	48691427
ENSG0000012831	0.658024928	1E-04	0	TST	protein_coding	thiosulfate s	22	37010859	37020183
ENSG0000011365	0.160140149	1E-04	0	DPYSL3	protein_coding	dihydropyri	5	147390808	147510068
ENSG0000002331	0.301306723	1E-04	0	ERP44	protein_coding	endoplasmi	9	99979185	100099000
ENSG0000014479	0.632878113	1E-04	0	LIMD1	protein_coding	LIM domain	3	45555394	45686341
ENSG0000018550	0.442273894	1E-04	0	FAAP100	protein_coding	FA core con	17	81539885	81553961
ENSG0000012705	0.318274217	1E-04	0	INTS11	protein_coding	integrator c	1	1311585	1324687
ENSG0000010312	-0.457468132	1E-04	0	AXIN1	protein_coding	axin 1 [Sour	16	287440	352723
ENSG0000011521	-0.320616142	1E-04	0	EIF2B4	protein_coding	eukaryotic t	2	27364352	27370457
ENSG0000012548	-0.198888691	1E-04	0	GTF3C4	protein_coding	general trar	9	132670035	132694953
ENSG0000014493	0.530957364	1E-04	0	TRPC1	protein_coding	transient rec	3	142724034	142807888
ENSG0000017272	0.419322281	1E-04	0	CORO1B	protein_coding	coronin 1B [	11	67435510	67443821
ENSG0000016025	-0.53773503	1E-04	0	FAM207A	protein_coding	family with s	21	44940012	44976989
ENSG0000026735	-0.439557319	1E-04	0	NA	NA	NA	NA	NA	NA
ENSG0000026179	-0.732641051	1E-04	0	AC007406.5	lncRNA	novel transc	12	273954	277123
ENSG0000016753	-0.987957298	1E-04	0	DHRS13	protein_coding	dehydrogen	17	28897781	28903079
ENSG0000010826	0.35067613	1E-04	0	GIT1	protein_coding	GIT ArfGAP	17	29573475	29594054
ENSG0000011257	0.492789413	1E-04	0	CCND3	protein_coding	cyclin D3 [S	6	41934934	42050357
ENSG0000018139	0.509967473	1E-04	0	OGFOD3	protein_coding	2-oxoglutar	17	82389210	82418637
ENSG0000016199	0.604787917	1E-04	0	WDR90	protein_coding	WD repeat c	16	649311	667833
ENSG0000022192	1.055366178	1E-04	0	ZNF880	protein_coding	zinc finger p	19	52369917	52385795
ENSG0000012871	-3.843663766	1E-04	0	HOXD10	protein_coding	homeobox D	2	176108790	176119937
ENSG0000013619	-0.177466799	1E-04	0	SCRN1	protein_coding	secernin 1 [S	7	29920104	29990289
ENSG0000023143	-6.902224956	1E-04	0	WASIR2	lncRNA	WASH and I	16	22910	25123
ENSG0000012900	-0.798804454	1E-04	0	CALML4	protein_coding	calmodulin	15	68190705	68206110
ENSG0000018100	1.214059629	1E-04	0	BBS12	protein_coding	Bardet-Bied	4	122732702	122744942
ENSG0000016239	1.055546952	1E-04	0	PARS2	protein_coding	prolyl-tRNA	1	54756898	54764523
ENSG0000012869	0.51096366	1E-04	0	OSGEPL1	protein_coding	O-sialoglyco	2	189746660	189763227
ENSG0000018583	-1.163181382	1E-04	0	GNB1L	protein_coding	G protein su	22	19783223	19854939

ENSG0000012587	-0.275097688	1E-04	0	SNRPB2	protein_coding	small nuclea	20	16729961	16742564
ENSG0000011411	-0.286923268	1E-04	0	RBP1	protein_coding	retinol bindi	3	139517434	139539829
ENSG0000013595	-0.499164893	1E-04	0	MFSD9	protein_coding	major facilit	2	102714630	102736888
ENSG0000016918	0.627162959	2E-04	0	APEX2	protein_coding	apurinic/apy	X	55000363	55009057
ENSG0000027494	-1.554891074	2E-04	0	CNOT3	protein_coding	CCR4-NOT	CHR_HSCHR	54138182	54156191
ENSG0000016583	-0.340746269	2E-04	0	TRUB1	protein_coding	TruB pseudo	10	114938195	114977676
ENSG0000018271	0.986967539	2E-04	0	CMC4	protein_coding	C-X9-C mo	X	155061622	155071136
ENSG0000017016	0.817519103	2E-04	0	AL512625.1	lncRNA	uncharacter	9	62897368	62900104
ENSG0000026058	1.739018246	2E-04	0	STAM-AS1	lncRNA	STAM antise	10	17641284	17643878
ENSG0000015040	0.388396764	2E-04	0	DCUN1D2	protein_coding	defective in	13	113455819	113490951
ENSG0000012783	0.31406677	2E-04	0	AAMP	protein_coding	angio assoc	2	218264129	218270178
ENSG0000014164	0.383653619	2E-04	0	MBD1	protein_coding	methyl-CpG	18	50266882	50281774
ENSG0000018018	0.257667407	2E-04	0	MED14	protein_coding	mediator cc	X	40648305	40735858
ENSG0000013427	0.346561292	2E-04	0	SPIRE1	protein_coding	spire type a	18	12446512	12658134
ENSG0000011620	0.755115542	2E-04	0	TCEANC2	protein_coding	transcriptior	1	54053584	54112519
ENSG0000016929	0.405462811	2E-04	0	PGM2	protein_coding	phosphoglu	4	37826660	37862937
ENSG0000009978	1.062639651	2E-04	0	MARCHF2	protein_coding	membrane	19	8413270	8439017
ENSG0000013921	0.19070779	2E-04	0	SCAF11	protein_coding	SR-related	12	45919131	45992120
ENSG0000011800	-0.307972659	2E-04	0	STAG1	protein_coding	stromal anti	3	136336236	136752403
ENSG0000018760	0.767912863	2E-04	0	MAGEH1	protein_coding	MAGE famil	X	55452127	55453566
ENSG0000017612	-0.627946414	2E-04	0	DLEU1	lncRNA	deleted in ly	13	50082169	50906856
ENSG0000027909	-1.309798715	2E-04	0	AC243964.4	TEC	TEC	19	44664131	44666158
ENSG0000019727	-2.208864506	2E-04	0	ZNF165	protein_coding	zinc finger p	6	28080568	28089563
ENSG0000017042	-1.585790869	2E-04	0	ADORA2B	protein_coding	adenosine A	17	15944917	15975746
ENSG0000013082	-0.212101213	2E-04	0	DKC1	protein_coding	dyskerin pse	X	154762742	154777689
ENSG0000016668	0.426532287	2E-04	0	COG1	protein_coding	component	17	73192632	73208507
ENSG0000026690	1.526346166	2E-04	0	LINC00663	lncRNA	long interge	19	19757366	19776423
ENSG0000016811	-0.34842861	2E-04	0	RAB4A	protein_coding	RAB4A, mer	1	229271062	229305894
ENSG0000016888	0.639284705	2E-04	0	TNIP2	protein_coding	TNFAIP3 int	4	2741648	2756342
ENSG0000010577	-0.51225809	2E-04	0	SMG9	protein_coding	SMG9 nonse	19	43727983	43754962
ENSG0000017733	-2.731809889	2E-04	0	C8orf31	lncRNA	chromosom	8	143039209	143060684
ENSG0000013073	-0.469464706	2E-04	0	YIPF2	protein_coding	Yip1 domair	19	10922185	10928681
ENSG0000023444	-0.427977033	2E-04	0	ZNF736	protein_coding	zinc finger p	7	64307459	64356634
ENSG0000018054	0.23157445	2E-04	0	TSPYL5	protein_coding	TSPY like 5	8	97273488	97277928
ENSG0000010023	1.200531027	2E-04	0	TIMP3	protein_coding	TIMP metall	22	32801705	32863041
ENSG0000010573	-0.624970824	2E-04	0	SIPA1L3	protein_coding	signal induc	19	37907208	38208369
ENSG0000015224	0.434309329	2E-04	0	C18orf25	protein_coding	chromosom	18	46173553	46266992
ENSG0000018817	0.57343231	2E-04	0	ZNF626	protein_coding	zinc finger p	19	20619939	20661596
ENSG0000018663	0.588887562	2E-04	0	KIF24	protein_coding	kinesin fami	9	34252381	34311371
ENSG0000016287	-1.810196915	2E-04	0	KLHDC8A	protein_coding	kelch doma	1	205336061	205357090
ENSG0000027685	0.62520913	2E-04	0	TUBGCP5	protein_coding	tubulin gam	CHR_HSCHR	22983146	23039654
ENSG0000009999	1.169393542	2E-04	0	TBC1D10A	protein_coding	TBC1 doma	22	30291990	30326947
ENSG0000016418	-0.419367344	2E-04	0	NDUFAF2	protein_coding	NADH:ubiqu	5	60945205	61153026
ENSG0000017560	0.406092942	2E-04	0	TMEM70	protein_coding	transmembr	8	73972437	73982783
ENSG0000014930	0.289695092	2E-04	0	NPAT	protein_coding	nuclear prot	11	108157215	108222638
ENSG0000023442	0.305250868	2E-04	0	ZNF37BP	transcribed_pr	zinc finger p	10	42513510	42552822
ENSG0000014971	0.433012145	2E-04	0	LTO1	protein_coding	LTO1 matur	11	69653076	69675416
ENSG0000012858	0.221669132	2E-04	0	MKLN1	protein_coding	muskelin 1	7	131110096	131496632
ENSG0000018158	0.362413533	2E-04	0	MEX3D	protein_coding	mex-3 RNA	19	1554669	1568058
ENSG0000013424	0.323871795	2E-04	0	LAMTOR5	protein_coding	late endoso	1	110401249	110407942
ENSG0000001148	0.332956508	2E-04	0	PPP5C	protein_coding	protein pho	19	46347087	46392981
ENSG0000000530	-0.449521682	2E-04	0	MSL3	protein_coding	MSL comple	X	11758159	11775772
ENSG0000013468	0.263768115	2E-04	0	PHC2	protein_coding	polyhomeot	1	33323623	33431052
ENSG0000027360	-1.419147202	2E-04	0	SRCIN1	protein_coding	SRC kinase	CHR_HSCHR	38330785	38406717
ENSG0000005034	-0.878622695	2E-04	0	NFE2L3	protein_coding	nuclear fact	7	26152198	26187137
ENSG0000017693	2.375621911	2E-04	0	TOB2P1	processed_pse	transducer	6	28217643	28218634
ENSG0000000704	-0.497693186	2E-04	0	MARK4	protein_coding	microtubule	19	45079288	45305284
ENSG0000010364	0.718806964	2E-04	0	LACTB	protein_coding	lactamase b	15	63121833	63142061

ENSG0000018189	0.745341244	2E-04	0	ZNF101	protein_codinç	zinc finger p	19	19668796	19683509
ENSG0000016521	-0.238845506	2E-04	0	GAPVD1	protein_codinç	GTPase acti	9	125261794	125367207
ENSG0000018712	-1.41954959	2E-04	0	SLIT1	protein_codinç	slit guidance	10	96998038	97185959
ENSG0000026145	0.805417948	2E-04	0	LINC01003	lncRNA	long interge	7	152463786	152465549
ENSG0000007624	0.497860477	2E-04	0	MLH1	protein_codinç	mutL homo	3	36993350	37050846
ENSG0000019872	0.293188109	2E-04	0	UNC13B	protein_codinç	unc-13 hom	9	35161992	35405338
ENSG0000016218	-2.051161156	2E-04	0	GNG3	protein_codinç	G protein su	11	62707676	62709201
ENSG0000018765	1.210515327	2E-04	0	VMAC	protein_codinç	vimentin typ	19	5904872	5910853
ENSG0000015913	-0.260553763	2E-04	0	GART	protein_codinç	phosphoribo	21	33503931	33543491
ENSG0000006526	-0.415381065	2E-04	0	WDR18	protein_codinç	WD repeat	19	984332	998438
ENSG0000018165	-1.542746684	2E-04	0	ATG9B	protein_codinç	autophagy r	7	151012209	151024499
ENSG0000017866	-4.439044241	2E-04	0	ARMC10P1	processed_pse	armadillo re	3	94506766	94507620
ENSG0000011172	0.409561259	2E-04	0	PRKAB1	protein_codinç	protein kina	12	119667864	119681624
ENSG0000016342	-2.853214672	2E-04	0	PROK2	protein_codinç	prokineticin	3	71771655	71785206
ENSG0000012030	-0.948714575	2E-04	0	CYSTM1	protein_codinç	cysteine rich	5	140175156	140282052
ENSG0000014828	-1.843463868	2E-04	0	GBGT1	protein_codinç	globoside a	9	133152948	133163933
ENSG0000027303	-1.161383422	2E-04	0	AL365203.2	lncRNA	novel transc	10	32887255	32889311
ENSG0000017267	0.622636114	2E-04	0	ZFAND4	protein_codinç	zinc finger A	10	45615500	45672780
ENSG0000017700	0.45025051	2E-04	0	MTHFR	protein_codinç	methylenete	1	11785723	11806455
ENSG0000018279	1.983428407	2E-04	0	CCDC87	protein_codinç	coiled-coil c	11	66590176	66593063
ENSG0000016404	-0.698688534	2E-04	0	ZNF589	protein_codinç	zinc finger p	3	48241100	48299253
ENSG0000018085	0.902180968	2E-04	0	ZNF443	protein_codinç	zinc finger p	19	12429706	12441021
ENSG0000010002	-0.289935165	2E-04	0	PES1	protein_codinç	pescadillo ri	22	30576625	30607083
ENSG0000024372	0.543353192	2E-04	0	TTC4	protein_codinç	tetratricope	1	54715861	54742657
ENSG0000018342	0.572322414	2E-04	0	NPIPA1	protein_codinç	nuclear pore	16	14922802	14952060
ENSG0000016720	-0.328906041	2E-04	0	TBC1D2B	protein_codinç	TBC1 doma	15	77984036	78077724
ENSG0000013011	-0.464606249	2E-04	0	GNL3L	protein_codinç	G protein nu	X	54530183	54621521
ENSG0000016922	0.314585893	2E-04	0	TBC1D10B	protein_codinç	TBC1 doma	16	30357102	30370494
ENSG0000023244	-1.271022566	2E-04	0	EMSLR	lncRNA	E2F1 mRNA	7	101308270	101314800
ENSG0000014319	0.348915422	2E-04	0	MGST3	protein_codinç	microsomal	1	165631213	165661796
ENSG0000010489	-0.251047904	2E-04	0	SF3A2	protein_codinç	splicing fact	19	2236824	2248655
ENSG0000017038	-1.069459706	2E-04	0	LRRN2	protein_codinç	leucine rich	1	204617170	204685738
ENSG0000007250	0.329441045	2E-04	0	HSD17B10	protein_codinç	hydroxyster	X	53431258	53434370
ENSG0000016909	1.531690204	2E-04	0	ASMTL	protein_codinç	acetylserotc	X	1403139	1453762
ENSG0000025995	-2.002181806	2E-04	0	AL138756.1	lncRNA	novel transc	9	112028570	112039143
ENSG0000012045	0.414389378	2E-04	0	MSANTD2	protein_codinç	Myb/SANT	11	124766498	124800706
ENSG0000007213	0.671413293	2E-04	0	PTPN18	protein_codinç	protein tyro	2	130356045	130375405
ENSG0000013191	-6.866416972	2E-04	0	NR0B2	protein_codinç	nuclear rece	1	26911489	26913975
ENSG0000014760	-0.119065707	2E-04	0	RPL7	protein_codinç	ribosomal p	8	73290242	73295789
ENSG0000011328	-0.215216229	2E-04	0	CLINT1	protein_codinç	clathrin inte	5	157785743	157859145
ENSG0000001027	0.385717926	2E-04	0	STARD3NL	protein_codinç	STARD3 N-	7	38178222	38230671
ENSG0000018804	-0.293080798	2E-04	0	ARL4C	protein_codinç	ADP ribosyl	2	234493041	234497081
ENSG0000016257	1.723535425	2E-04	0	SCNN1D	protein_codinç	sodium char	1	1280436	1292029
ENSG0000011628	0.33625628	2E-04	0	ERRFI1	protein_codinç	ERBB recept	1	8004404	8026309
ENSG0000016191	1.091896456	2E-04	0	ADCY10P1	transcribed_un	ADCY10 pse	6	41101022	41140835
ENSG0000026008	-4.529296493	2E-04	0	AC007611.1	lncRNA	novel transc	16	48623422	48746318
ENSG0000018208	0.267376477	2E-04	0	TMEM259	protein_codinç	transmembr	19	1009648	1021179
ENSG0000027710	0.732246657	2E-04	0	TADA2A	protein_codinç	transcriptior	CHR_HSCHR	37411498	37484368
ENSG0000018782	-1.373408664	2E-04	0	TMEM220	protein_codinç	transmembr	17	10699015	10730023
ENSG0000000840	-0.313322826	2E-04	0	CRY1	protein_codinç	cryptochron	12	106991364	107093549
ENSG0000011355	-0.298001279	2E-04	0	GNPDA1	protein_codinç	glucosamine	5	141991749	142013041
ENSG0000018823	-0.458079961	2E-04	0	AGAP4	protein_codinç	ArfGAP with	10	45825594	45853875
ENSG0000016769	0.362723179	2E-04	0	GLOD4	protein_codinç	glyoxalase c	17	757097	783390
ENSG0000010056	0.673176239	2E-04	0	PIGH	protein_codinç	phosphatidy	14	67581955	67600286
ENSG0000015596	-0.418655326	2E-04	0	RAB39B	protein_codinç	RAB39B, me	X	155258235	155264491
ENSG0000016922	0.247793864	2E-04	0	LMAN2	protein_codinç	lectin, manr	5	177331567	177351668
ENSG0000013868	0.44749919	2E-04	0	BBS7	protein_codinç	Bardet-Biec	4	121824329	121870487
ENSG0000014094	0.215071206	2E-04	0	MBTPS1	protein_codinç	membrane	16	84053761	84116942

ENSG0000013733	0.542324348	2E-04	0	PGBD1	protein_coding	piggyBac tra		6	28281572	28302549
ENSG0000020482	0.458212474	2E-04	0	MRPL53	protein_coding	mitochondr		2	74471982	74472687
ENSG0000010513	-0.386360307	2E-04	0	ILVBL	protein_coding	ilvB acetolac		19	15114984	15125786
ENSG0000027736	-1.198341232	2E-04	0	SRCIN1	protein_coding	SRC kinase s		17	38530031	38605952
ENSG0000000754	-0.31305913	2E-04	0	CRAMP1	protein_coding	cramped ch		16	1612337	1677908
ENSG0000011668	0.397156329	2E-04	0	KIAA2013	protein_coding	KIAA2013 [S		1	11919591	11926428
ENSG0000017719	-0.49610734	2E-04	0	PUS1	protein_coding	pseudouridi		12	131929200	131945896
ENSG0000006566	0.673242659	2E-04	0	SEC61A2	protein_coding	SEC61 trans		10	12129637	12169961
ENSG0000017395	0.406536736	2E-04	0	XXYLT1	protein_coding	xyloside xylo		3	195068284	195271159
ENSG0000016283	-0.812311334	2E-04	0	ACP6	protein_coding	acid phosph		1	147629652	147670524
ENSG0000020612	2.96023148	2E-04	0	GOLGA8O	protein_coding	golgin A8 fa		15	32441914	32455634
ENSG0000017104	-0.839219932	2E-04	0	TSNARE1	protein_coding	t-SNARE dc		8	142212080	142403182
ENSG0000015610	0.234015856	2E-04	0	MMP16	protein_coding	matrix meta		8	88032011	88328025
ENSG0000025829	0.546265433	2E-04	0	NA	NA	NA	NA	NA	NA	
ENSG0000027578	6.792786432	2E-04	0	NA	NA	NA	NA	NA	NA	
ENSG0000018368	-0.999120986	2E-04	0	RFLNB	protein_coding	refilin B [Sou		17	439978	445939
ENSG0000011072	0.291275985	2E-04	0	CHKA	protein_coding	choline kina		11	68052859	68121444
ENSG0000022199	-1.251620209	2E-04	0	ZNF630	protein_coding	zinc finger p	X		47983356	48071658
ENSG0000010485	0.231483133	2E-04	0	SNRNP70	protein_coding	small nuclea		19	49085419	49108605
ENSG0000015527	-0.664235543	2E-04	0	TRMT44	protein_coding	tRNA methy		4	8436140	8493531
ENSG0000011754	-0.32600135	2E-04	0	DPH5	protein_coding	diphthamide		1	100989623	101026088
ENSG0000021469	-1.650184623	2E-04	0	ARHGEF33	protein_coding	Rho guanine		2	38889841	38975449
ENSG0000026754	-1.601908064	2E-04	0	AC015802.3	lncRNA	novel transc		17	76549951	76550826
ENSG0000011304	-0.254733987	2E-04	0	MRPS27	protein_coding	mitochondr		5	72219403	72320646
ENSG0000015762	0.334750796	2E-04	0	TAB3	protein_coding	TGF-beta ac	X		30827442	30975084
ENSG0000014456	0.255009584	2E-04	0	RETREG2	protein_coding	reticulophag		2	219176225	219185475
ENSG0000014286	0.166194847	2E-04	0	SERBP1	protein_coding	SERPINE1 m		1	67407810	67430415
ENSG0000016164	-1.798077574	2E-04	0	MPP3	protein_coding	membrane p		17	43800799	43833170
ENSG0000015063	-1.262292543	2E-04	0	CCDC102B	protein_coding	coiled-coil c		18	68715209	69055189
ENSG0000011091	-0.168885176	2E-04	0	MLEC	protein_coding	malectin [So		12	120687149	120701859
ENSG0000005390	0.423824667	2E-04	0	ANAPC4	protein_coding	anaphase pr		4	25377263	25418498
ENSG0000018573	-2.933656251	2E-04	0	NRG3	protein_coding	neuregulin 3		10	81875194	82987179
ENSG0000012834	-1.777352563	2E-04	0	RAC2	protein_coding	Rac family s		22	37225270	37244448
ENSG0000018376	0.779513403	2E-04	0	KREMEN1	protein_coding	kringle cont		22	29073035	29168333
ENSG0000012801	-1.039886352	2E-04	0	ZFP36	protein_coding	ZFP36 ring f		19	39406847	39409412
ENSG0000010539	0.338312509	2E-04	0	TYK2	protein_coding	tyrosine kina		19	10350529	10380572
ENSG0000027906	1.158683824	2E-04	0	AC015813.5	TEC	novel transc		17	58006674	58008187
ENSG0000023029	1.254508736	2E-04	0	MSH5	protein_coding	mutS homo	CHR_HSCHR		31722164	31744857
ENSG0000018795	-1.929144661	2E-04	0	HS6ST1P1	processed_pse	heparan sul		1	21428303	21429536
ENSG0000022888	1.910172544	2E-04	0	UBAC2-AS1	lncRNA	UBAC2 antis		13	99181223	99200757
ENSG0000013982	0.453131569	2E-04	0	ABHD13	protein_coding	abhydrolase		13	108218392	108234243
ENSG0000014098	0.511135429	2E-04	0	ZSCAN32	protein_coding	zinc finger a		16	3382081	3401065
ENSG0000027474	0.760779608	2E-04	0	ZNF100	protein_coding	zinc finger p	CHR_HSCHR		21732245	21777028
ENSG0000027002	3.91116722	2E-04	0	AC026691.1	lncRNA	novel transc		5	135399280	135401296
ENSG0000013623	0.175038663	2E-04	0	RAC1	protein_coding	Rac family s		7	6374527	6403967
ENSG0000011594	-0.367147161	2E-04	0	ORC2	protein_coding	origin recog		2	200908977	200963680
ENSG0000026885	1.014854786	2E-04	0	AL118506.1	lncRNA	uncharacter		20	63861212	63864293
ENSG0000016875	-0.369997246	2E-04	0	SEMA4C	protein_coding	semaphorin		2	96859718	96870757
ENSG0000014745	0.298328535	2E-04	0	SLC25A37	protein_coding	solute carrie		8	23528956	23575463
ENSG0000013780	0.635898549	2E-04	0	NDUFAF1	protein_coding	NADH:ubiqu		15	41387353	41402519
ENSG0000022873	-0.884809123	2E-04	0	RGL2	protein_coding	ral guanine	CHR_HSCHR		33461997	33469667
ENSG0000014319	-0.337152908	2E-04	0	POU2F1	protein_coding	POU class 2		1	167220876	167427345
ENSG0000013303	0.196283446	2E-04	0	MPRIP	protein_coding	myosin pho		17	17042457	17217679
ENSG0000027789	1.265072928	2E-04	0	GSTT2	protein_coding	glutathione	CHR_HSCHR		23980152	23983919
ENSG0000010364	-0.842940131	2E-04	0	CORO2B	protein_coding	coronin 2B [		15	68578993	68727806
ENSG0000014753	-0.250659949	2E-04	0	GOLGA7	protein_coding	golgin A7 [S		8	41490396	41510980
ENSG0000010205	-1.692858752	2E-04	0	ZC3H12B	protein_coding	zinc finger C	X		65366638	65507887
ENSG0000016853	-1.500214074	2E-04	0	CHRM1	protein_coding	cholinergic r		11	62908679	62921807

ENSG0000010359	1.555091224	2E-04	0	IQCH	protein_coding	IQ motif cor	15	67254786	67502260
ENSG0000015604	1.87993797	2E-04	0	CFAP70	protein_coding	cilia and flag	10	73253759	73358859
ENSG0000015761	-1.244614905	2E-04	0	C2CD2	protein_coding	C2 calcium	21	41885112	41954018
ENSG0000016088	-3.8092472	2E-04	0	HK3	protein_coding	hexokinase	5	176880869	176899346
ENSG0000013047	0.416750344	2E-04	0	MAP1S	protein_coding	microtubule	19	17719242	17734513
ENSG0000011552	0.773352783	2E-04	0	ST3GAL5	protein_coding	ST3 beta-ga	2	85837120	85905199
ENSG0000014102	0.614772896	2E-04	0	MED9	protein_coding	mediator cc	17	17476994	17493221
ENSG0000028010	-1.868046613	2E-04	0	AC008555.6	TEC	TEC	19	34949038	34951205
ENSG0000016828	-0.260920917	2E-04	0	MMADHC	protein_coding	metabolism	2	149569637	149587778
ENSG0000026094	-2.032352708	2E-04	0	LINC00622	lncRNA	long interge	1	119597702	119599271
ENSG0000010094	-0.788175985	2E-04	0	RABGGTA	protein_coding	Rab geranyl	14	24265538	24271739
ENSG0000016718	-0.453915046	2E-04	0	COQ7	protein_coding	coenzyme C	16	19067614	19080095
ENSG0000017791	0.338285501	2E-04	0	ARL6IP6	protein_coding	ADP ribosyl	2	152717893	152762396
ENSG0000013425	1.033265722	2E-04	0	TRIM45	protein_coding	tripartite mo	1	117111060	117122587
ENSG0000016535	0.511901129	2E-04	0	FBXO33	protein_coding	F-box prote	14	39397669	39432500
ENSG0000006722	-0.13688308	2E-04	0	PKM	protein_coding	pyruvate kir	15	72199029	72231822
ENSG0000012512	-0.372153868	2E-04	0	BBS2	protein_coding	Bardet-Biec	16	56466836	56520087
ENSG0000015789	0.486890718	2E-04	0	C12orf43	protein_coding	chromosom	12	121000486	121016502
ENSG0000018656	-1.474513496	2E-04	0	CEACAM19	protein_coding	CEA cell adh	19	44662278	44684359
ENSG0000015472	-0.31123429	2E-04	0	JAM2	protein_coding	junctional a	21	25639258	25717562
ENSG0000012995	-2.57408264	2E-04	0	PLPPR3	protein_coding	phospholipi	19	812488	821955
ENSG0000017509	0.529716736	2E-04	0	SPSB4	protein_coding	splA/ryanoc	3	141051347	141148611
ENSG0000010090	0.273714371	2E-04	0	PSMA6	protein_coding	proteasome	14	35278633	35317493
ENSG0000017246	2.17927831	2E-04	0	PRSS30P	lncRNA	serine prote	16	2839568	2842744
ENSG0000025023	-4.503893039	2E-04	0	AC010273.3	lncRNA	novel transc	5	69038518	69043821
ENSG0000015968	-0.651775746	2E-04	0	CHCHD6	protein_coding	coiled-coil-	3	126704240	126960420
ENSG0000013609	-0.936195798	2E-04	0	NEK3	protein_coding	NIMA relate	13	52132639	52159861
ENSG0000013550	0.30270867	2E-04	0	ACVR1B	protein_coding	activin A re	12	51951699	51997078
ENSG0000011753	-0.38005247	2E-04	0	VAMP4	protein_coding	vesicle asso	1	171700160	171742074
ENSG0000018358	-0.384692368	2E-04	0	FBXL7	protein_coding	F-box and l	5	15500180	15939795
ENSG0000013587	-0.265896732	2E-04	0	RC3H1	protein_coding	ring finger a	1	173931084	174022357
ENSG0000004763	0.455836996	2E-04	0	SCML1	protein_coding	Scm polyco	X	17737449	17754988
ENSG0000005872	-0.336127552	2E-04	0	RIOK2	protein_coding	RIO kinase 2	5	97160867	97183247
ENSG0000008733	0.290099246	2E-04	0	GMCL1	protein_coding	germ cell-le	2	69829660	69881384
ENSG0000015959	-0.2327906	2E-04	0	GPBP1L1	protein_coding	GC-rich pro	1	45627304	45688113
ENSG0000024977	-1.641191253	2E-04	0	AC092647.5	protein_coding	novel zinc fi	7	55887277	55955239
ENSG0000016408	0.749773057	2E-04	0	POC1A	protein_coding	POC1 centri	3	52075226	52154690
ENSG0000023634	-4.201639887	2E-04	0	SCAT8	lncRNA	S-phase car	6	63806836	63822642
ENSG0000027341	-1.396973484	2E-04	0	AC004877.1	lncRNA	novel transc	7	149858400	149862492
ENSG0000013749	0.694745552	2E-04	0	ANKRD42	protein_coding	ankyrin repe	11	83193712	83260694
ENSG0000010957	-0.420477139	2E-04	0	AADAT	protein_coding	aminoadipa	4	170060222	170091699
ENSG0000027323	-1.376722211	2E-04	0	AC102953.2	lncRNA	novel transc	7	1464497	1467522
ENSG0000002227	0.282738776	2E-04	0	RTF2	protein_coding	replication t	20	56468585	56519449
ENSG0000017449	-1.615222407	2E-04	0	IGDCC3	protein_coding	immunoglol	15	65327127	65378002
ENSG0000010835	1.074446945	2E-04	0	RAPGEFL1	protein_coding	Rap guanine	17	40177010	40195656
ENSG0000008523	0.503154897	2E-04	0	AK6	protein_coding	adenylate ki	5	69350984	69370013
ENSG0000013959	-0.928092208	2E-04	0	N4BP2L1	protein_coding	NEDD4 binc	13	32400723	32428311
ENSG0000017413	-1.538442877	2E-04	0	FAM53A	protein_coding	family with s	4	1617915	1684302
ENSG0000024821	-1.604927517	2E-04	0	CICP16	processed_pse	capicua tran	4	118635970	118638782
ENSG0000020413	-0.305188336	2E-04	0	PHACTR4	protein_coding	phosphatase	1	28369582	28500364
ENSG0000027095	2.545641677	2E-04	0	LPP-AS2	lncRNA	LPP antisens	3	188151206	188154057
ENSG0000000570	-0.263486844	2E-04	0	IBTK	protein_coding	inhibitor of	6	82169986	82247754
ENSG0000027560	0.585515419	2E-04	0	PIGW	protein_coding	phosphatidy	CHR_HSCHR	36535392	36539682
ENSG0000026010	2.570023374	2E-04	0	AC012435.2	transcribed_pr	coiled-coil-	15	74478070	74490286
ENSG0000015293	-1.795043086	2E-04	0	PART1	lncRNA	prostate anc	5	60487713	60548813
ENSG0000018650	-0.437218067	2E-04	0	TMEM222	protein_coding	transmembr	1	27322145	27336400
ENSG0000014068	-0.726296262	2E-04	0	TGFB1I1	protein_coding	transforming	16	31471585	31477960
ENSG0000016839	0.349871261	3E-04	0	DTYMK	protein_coding	deoxythymi	2	241675747	241686944

ENSG0000024546	2.740675877	3E-04	0	LINC02447	lncRNA	long interge		4	7093776	7103394
ENSG0000010908	-0.415187604	3E-04	0	CDR2L	protein_coding	cerebellar d		17	74987632	75005800
ENSG0000020468	0.583417681	3E-04	0	MIR1915HC	lncRNA	MIR1915 hc		10	21492658	21497260
ENSG0000018550	1.842152656	3E-04	0	IRF7	protein_coding	interferon re		11	612553	615983
ENSG0000014279	-1.124468698	3E-04	0	NBPF3	protein_coding	NBPF meml		1	21440128	21485005
ENSG0000027706	0.653095148	3E-04	0	CU634019.1	lncRNA	uncharacter		21	7048891	7087229
ENSG0000013749	-1.512219037	3E-04	0	SLCO2B1	protein_coding	solute carrie		11	75100563	75206549
ENSG0000025132	-1.324851809	3E-04	0	SHANK3	protein_coding	SH3 and mu		22	50674415	50733298
ENSG0000025151	-6.736049553	3E-04	0	AC096734.1	lncRNA	novel transc		4	42706107	42707335
ENSG0000017570	0.923903012	3E-04	0	MTLN	protein_coding	mitoregulin		2	110211529	110245420
ENSG0000013177	-0.351739382	3E-04	0	KHDRBS3	protein_coding	KH RNA bin		8	135457456	135656722
ENSG0000016661	0.354015793	3E-04	0	BLCAP	protein_coding	BLCAP apop		20	37492472	37527931
ENSG0000011247	-0.555666822	3E-04	0	SLC39A7	protein_coding	solute carrie		6	33200445	33204439
ENSG0000011664	-0.260417298	3E-04	0	DOCK7	protein_coding	dedicator of		1	62454298	62688386
ENSG0000024989	1.230249664	3E-04	0	MCPH1-AS	lncRNA	MCPH1 anti		8	6617310	6708224
ENSG0000014109	0.418788076	3E-04	0	GFOD2	protein_coding	glucose-fru		16	67674531	67719339
ENSG0000025794	-2.114498809	3E-04	0	TEN1	protein_coding	TEN1 subun		17	75979240	76000586
ENSG0000017598	-2.142533089	3E-04	0	DENND2C	protein_coding	DENN doma		1	114582848	114670422
ENSG0000026993	-2.720887328	3E-04	0	NA	NA	NA	NA	NA	NA	
ENSG0000010737	0.44418151	3E-04	0	EXOSC3	protein_coding	exosome cc		9	37766978	37801437
ENSG0000024430	0.438917365	3E-04	0	GATA2-AS1	lncRNA	GATA2 antis		3	128489212	128502970
ENSG0000018834	-0.476007654	3E-04	0	GTF2F2	protein_coding	general trar		13	45120510	45284893
ENSG0000027886	0.9425884	3E-04	0	AC055811.4	TEC	novel transc		17	17181504	17183257
ENSG0000017936	0.327454687	3E-04	0	PACS2	protein_coding	phosphofuri		14	105300563	105398147
ENSG0000013201	1.037006401	3E-04	0	ZNF20	protein_coding	zinc finger p		19	12092843	12140355
ENSG0000000995	-0.170327917	3E-04	0	BAZ1B	protein_coding	bromodoma		7	73440406	73522293
ENSG0000017321	0.586685651	3E-04	0	MFSD4B	protein_coding	major facilit		6	111259327	111445354
ENSG0000016005	1.069444897	3E-04	0	IQCC	protein_coding	IQ motif cor		1	32205671	32208682
ENSG0000010046	-0.301441569	3E-04	0	RBM23	protein_coding	RNA binding		14	22893204	22919182
ENSG0000010567	-0.483143686	3E-04	0	TMEM147	protein_coding	transmembr		19	35545600	35547526
ENSG0000014363	0.58578636	3E-04	0	C1orf131	protein_coding	chromosom		1	231223763	231241187
ENSG0000019803	0.636365592	3E-04	0	ZNF273	protein_coding	zinc finger p		7	64870172	64930966
ENSG0000027795	5.566290059	3E-04	0	SENP3-EIF4	protein_coding	SENP3-EIF4		17	7563287	7578715
ENSG0000011964	0.679647544	3E-04	0	ACYP1	protein_coding	acylphospha		14	75053237	75069483
ENSG0000013911	0.258207343	3E-04	0	CPNE8	protein_coding	copine 8 [Sc		12	38646822	38907430
ENSG0000013108	0.399937438	3E-04	0	ARHGEF9	protein_coding	Cdc42 guan	X		63634967	63809274
ENSG0000018292	0.472464185	3E-04	0	CEP63	protein_coding	centrosoma		3	134485721	134575017
ENSG0000015783	-0.340166028	3E-04	0	SPPL3	protein_coding	signal pepti		12	120762510	120904358
ENSG0000018299	1.020024624	3E-04	0	C12orf60	protein_coding	chromosom		12	14803666	14906586
ENSG0000017945	-0.252950505	3E-04	0	ZBTB18	protein_coding	zinc finger a		1	244048939	244057476
ENSG0000015699	0.779200503	3E-04	0	RPUSD3	protein_coding	RNA pseudo		3	9837849	9844602
ENSG0000012415	-0.304998518	3E-04	0	NCOA3	protein_coding	nuclear rece		20	47501887	47656877
ENSG0000013193	-0.512992111	3E-04	0	THAP1	protein_coding	THAP doma		8	42836674	42843325
ENSG0000011184	-0.340634658	3E-04	0	TMEM14C	protein_coding	transmembr		6	10722915	10731129
ENSG0000018393	2.004099744	3E-04	0	HTR7P1	transcribed_pr	5-hydroxytr		12	13000420	13004830
ENSG0000007709	-2.470073969	3E-04	0	RARB	protein_coding	retinoic acic		3	25174332	25597932
ENSG0000016400	0.642092029	3E-04	0	C1orf50	protein_coding	chromosom		1	42767249	42779491
ENSG0000016120	0.15864918	3E-04	0	AP2M1	protein_coding	adaptor rela		3	184174689	184184091
ENSG0000011203	-0.377078115	3E-04	0	PPARD	protein_coding	peroxisome		6	35342558	35428191
ENSG0000016316	0.293865072	3E-04	0	IWS1	protein_coding	interacts wit		2	127436207	127526886
ENSG0000013683	0.412613909	3E-04	0	NIBAN2	protein_coding	niban apopt		9	127505339	127578989
ENSG0000025543	-3.36677066	3E-04	0	LINC02735	lncRNA	long interge		11	56848478	56878078
ENSG0000027787	2.061447259	3E-04	0	NA	NA	NA	NA	NA	NA	
ENSG0000000445	-0.241681424	3E-04	0	AK2	protein_coding	adenylate ki		1	33007940	33080996
ENSG0000011966	0.442948099	3E-04	0	DNAL1	protein_coding	dynein axor		14	73644875	73703732
ENSG0000004784	0.220255845	3E-04	0	MAP4	protein_coding	microtubule		3	47850690	48089272
ENSG0000010043	-0.792418617	3E-04	0	ABHD4	protein_coding	abhydrolase		14	22598237	22613215
ENSG0000012458	0.508480488	3E-04	0	PEX6	protein_coding	peroxisoma		6	42963865	42979181

ENSG0000016809	0.195229159	3E-04	0	PAFAH1B2	protein_coding	platelet acti	11	117144284	117176894
ENSG0000019893	1.565787722	3E-04	0	MAGEE1	protein_coding	MAGE famil	X	76427710	76431342
ENSG0000012297	-0.368999548	3E-04	0	IFT81	protein_coding	intraflagella	12	110124335	110218793
ENSG0000011167	1.145635322	3E-04	0	SPSB2	protein_coding	splA/ryanoc	12	6870935	6889358
ENSG0000016737	-0.379857114	3E-04	0	IRGQ	protein_coding	immunity re	19	43584369	43596135
ENSG0000016373	-0.591754265	3E-04	0	MTHFD2L	protein_coding	methylenete	4	74114174	74303099
ENSG0000012760	0.182589577	3E-04	0	MACF1	protein_coding	microtubule	1	39081316	39487177
ENSG0000013547	-0.282092933	3E-04	0	ESPL1	protein_coding	extra spindl	12	53268299	53293638
ENSG0000018664	-0.656105651	3E-04	0	CARMIL3	protein_coding	capping prc	14	24052009	24069729
ENSG0000010205	1.216133384	3E-04	0	KCND1	protein_coding	potassium v	X	48961378	48971844
ENSG0000027884	-1.334387704	3E-04	0	MRPL45	protein_coding	mitochondr	17	38297023	38323217
ENSG0000013271	-0.230159567	3E-04	0	DCAF8	protein_coding	DDB1 and C	1	160215715	160262549
ENSG0000013934	0.317612164	3E-04	0	SNRPF	protein_coding	small nuclea	12	95858928	95903828
ENSG0000012416	-0.295935149	3E-04	0	NCOA5	protein_coding	nuclear rece	20	46060991	46089962
ENSG0000018592	-0.484832187	3E-04	0	NA	NA	NA	NA	NA	NA
ENSG0000027375	3.273250284	3E-04	0	AL117379.1	lncRNA	novel transc	20	62928621	62929297
ENSG0000007315	-2.213455396	3E-04	0	PANX2	protein_coding	pannexin 2	22	50170731	50180295
ENSG0000011771	-0.182910317	3E-04	0	ARID1A	protein_coding	AT-rich inte	1	26693236	26782104
ENSG0000013457	0.651776109	3E-04	0	DDB2	protein_coding	damage spe	11	47214465	47239217
ENSG0000008610	-0.270758664	3E-04	0	NFX1	protein_coding	nuclear tran	9	33290512	33371157
ENSG0000015225	-0.638782231	3E-04	0	PDK1	protein_coding	pyruvate de	2	172555373	172608669
ENSG0000006809	0.448082996	3E-04	0	HEATR6	protein_coding	HEAT repea	17	60041008	60078922
ENSG0000011504	0.625974627	3E-04	0	FAHD2A	protein_coding	fumarylacet	2	95402708	95416616
ENSG0000010906	0.554777026	3E-04	0	SLC9A3R1	protein_coding	SLC9A3 reg	17	74748628	74769353
ENSG0000016831	-0.596292039	3E-04	0	IRF2	protein_coding	interferon re	4	184387729	184474550
ENSG0000027559	0.725023009	3E-04	0	XKR5	protein_coding	XK related 5	8	6808517	6835524
ENSG0000014926	0.296211321	3E-04	0	INTS4	protein_coding	integrator c	11	77878720	77994671
ENSG0000026775	1.427137952	3E-04	0	RUNDC3A-	lncRNA	RUNDC3A a	17	44299574	44315315
ENSG0000018492	0.983040331	3E-04	0	NUTM2A	protein_coding	NUT family	10	87225448	87236908
ENSG0000013781	0.260848441	3E-04	0	PARP6	protein_coding	poly(ADP-ri	15	72241181	72272999
ENSG0000017580	-1.526347798	3E-04	0	MSRA	protein_coding	methionine	8	10054292	10428891
ENSG0000018128	-3.744260229	3E-04	0	TMEM102	protein_coding	transmembr	17	7435435	7437679
ENSG0000025540	0.893444791	3E-04	0	PCDHA3	protein_coding	protocadhe	5	140801028	141012347
ENSG0000013569	0.449561197	3E-04	0	MPHOSPH6	protein_coding	M-phase ph	16	82147798	82170224
ENSG0000004034	0.302419636	3E-04	0	STAU2	protein_coding	staufen dou	8	73420369	73747708
ENSG0000010911	-0.346281438	3E-04	0	PHF12	protein_coding	PHD finger	17	28905250	28951771
ENSG0000016387	0.324300142	3E-04	0	MEAF6	protein_coding	MYST/Esa1	1	37489993	37514766
ENSG0000016710	1.560799597	3E-04	0	PIP5KL1	protein_coding	phosphatidy	9	127920881	127930785
ENSG0000023743	-5.763654857	3E-04	0	LINC02790	lncRNA	long interge	1	95937901	96022890
ENSG0000022796	-6.694088163	3E-04	0	NPM1P22	processed_pse	nucleophos	13	67831876	67832827
ENSG0000014107	-0.328096188	3E-04	0	UTP4	protein_coding	UTP4 small	16	69131291	69231130
ENSG0000010575	-2.559261584	3E-04	0	ETHE1	protein_coding	ETHE1 persu	19	43506719	43527230
ENSG0000011904	-1.89231396	3E-04	0	SATB2	protein_coding	SATB home	2	199269500	199471266
ENSG0000021324	2.400012561	3E-04	0	H3P4	unprocessed_p	H3 histone p	1	121118195	121118610
ENSG0000024261	0.950996567	3E-04	0	DECR2	protein_coding	2,4-dienoyl-	16	401858	412487
ENSG0000012236	-1.612654039	3E-04	0	LDB3	protein_coding	LIM domain	10	86668507	86736072
ENSG0000008915	-0.122525723	3E-04	0	RPLP0	protein_coding	ribosomal p	12	120196699	120201235
ENSG0000023565	0.378156174	3E-04	0	H3P6	processed_pse	H3 histone p	2	174719908	174720318
ENSG0000005043	-0.252407951	3E-04	0	SLC4A8	protein_coding	solute carrie	12	51391317	51515763
ENSG0000013596	-0.314679437	3E-04	0	GCC2	protein_coding	GRIP and co	2	108449191	108509415
ENSG0000012070	-0.271103704	3E-04	0	FAM53C	protein_coding	family with s	5	138331935	138349729
ENSG0000022431	-1.186103568	3E-04	0	DXO	protein_coding	decapping e	CHR_HSCHR	32046253	32048735
ENSG0000010633	2.584393444	3E-04	0	FBXO24	protein_coding	F-box prote	7	100583982	100601117
ENSG0000022569	1.235113665	3E-04	0	SLC26A6	protein_coding	solute carrie	3	48625723	48635493
ENSG0000014048	2.260273546	3E-04	0	CELF6	protein_coding	CUGBP Elav	15	72284727	72320157
ENSG0000006366	0.548550385	3E-04	0	GPC1	protein_coding	glypican 1 [	2	240435663	240468076
ENSG0000027305	-6.692328654	3E-04	0	AL354694.1	lncRNA	novel transc	9	7786105	7786688
ENSG0000025607	1.722558322	3E-04	0	URB1-AS1	lncRNA	URB1 antise	21	32393130	32393960

ENSG0000023966	0.640879618	3E-04	0	AL157392.3	lncRNA	novel transc		10	13631143	13668445
ENSG0000011524	-0.187765551	3E-04	0	PPM1G	protein_coding	protein pho		2	27381195	27409591
ENSG0000014919	0.33160455	3E-04	0	HIKESHI	protein_coding	heat shock		11	86302211	86345943
ENSG0000016617	0.264821625	3E-04	0	BAG5	protein_coding	BAG cochap		14	103556544	103562831
ENSG0000013407	0.403544495	3E-04	0	THUMPD3	protein_coding	THUMP dor		3	9362971	9386791
ENSG0000024183	-0.252831579	3E-04	0	ATP5PO	protein_coding	ATP synthas		21	33903453	33915814
ENSG0000019896	-0.550845968	3E-04	0	SGMS1	protein_coding	sphingomye		10	50305586	50625163
ENSG0000017853	0.949176992	3E-04	0	SLC25A20	protein_coding	solute carrie		3	48856926	48898904
ENSG0000017608	0.303378141	3E-04	0	SLC35A4	protein_coding	solute carrie		5	140564828	140569100
ENSG0000010083	-0.185603746	3E-04	0	PABPN1	protein_coding	poly(A) binc		14	23321289	23326185
ENSG0000010787	-0.546416135	3E-04	0	FBXL15	protein_coding	F-box and l		10	102419189	102423136
ENSG0000014976	0.631596493	3E-04	0	NUDT22	protein_coding	nudix hydro		11	64225941	64230686
ENSG0000005159	0.333846365	3E-04	0	THOC3	protein_coding	THO compl		5	175917873	176034680
ENSG0000017436	-0.754277605	3E-04	0	SNHG11	lncRNA	small nucle		20	38446343	38450940
ENSG0000015045	-0.257204591	3E-04	0	SAP18	protein_coding	Sin3A assoc		13	21140514	21149084
ENSG0000018126	0.323994728	3E-04	0	TLCD5	protein_coding	TLC domain		11	120325296	120333686
ENSG0000023601	-0.967246229	3E-04	0	VPS52	protein_coding	VPS52 subu	CHR_HSCHR		33390441	33412193
ENSG0000025840	1.770955572	3E-04	0	ZNF578	protein_coding	zinc finger p		19	52453553	52516882
ENSG0000019765	-1.083164127	3E-04	0	DNAH10	protein_coding	dynein axor		12	123762188	123935720
ENSG0000013242	0.632633468	3E-04	0	COQ3	protein_coding	coenzyme C		6	99369401	99394195
ENSG0000015171	-1.859590662	3E-04	0	TMEM45B	protein_coding	transmembr		11	129815848	129860003
ENSG0000023544	-1.649590989	3E-04	0	ZNRD1	protein_coding	zinc ribbon	CHR_HSCHR		30051404	30057408
ENSG0000007745	0.466304636	3E-04	0	LRCH4	protein_coding	leucine rich		7	100574011	100586129
ENSG0000026989	1.44927923	4E-04	0	AL513477.1	transcribed_pr	small nuclea		1	2350414	2352820
ENSG0000012570	0.633659747	4E-04	0	ATG4C	protein_coding	autophagy r		1	62784132	62865516
ENSG0000010505	0.663666101	4E-04	0	VRK3	protein_coding	VRK serine/t		19	49976468	50025946
ENSG0000025713	4.065818231	4E-04	0	ODC1-DT	lncRNA	ODC1 diver		2	10448689	10457693
ENSG0000014157	-0.389519313	4E-04	0	RNF157	protein_coding	ring finger p		17	76142465	76240493
ENSG0000017672	-2.755524894	4E-04	0	TTTY14	lncRNA	testis-specif	Y		18772706	19077416
ENSG0000017809	0.793579103	4E-04	0	BOLA1	protein_coding	bolA family		1	149887890	149900798
ENSG0000010743	-1.218938894	4E-04	0	PDLIM1	protein_coding	PDZ and LIN		10	95237572	95291012
ENSG0000026960	-0.877273799	4E-04	0	RPARP-AS1	lncRNA	RPARP antis		10	102449816	102461106
ENSG0000019870	0.16692683	4E-04	0	IPO9	protein_coding	importin 9 [		1	201829149	201884291
ENSG0000023318	0.743882015	4E-04	0	AC093157.1	lncRNA	novel transc		1	101025844	101090513
ENSG0000023454	0.396437298	4E-04	0	FAM133B	protein_coding	family with s		7	92560758	92590393
ENSG0000019710	-0.145395844	4E-04	0	DYNC1H1	protein_coding	dynein cyto		14	101964573	102056443
ENSG0000027157	-4.866912572	4E-04	0	AC078880.3	lncRNA	novel transc		12	115299588	115300708
ENSG0000019607	-1.761591409	4E-04	0	SYCP2	protein_coding	synaptonem		20	59863564	59933655
ENSG0000013846	0.305977979	4E-04	0	SENP7	protein_coding	SUMO spec		3	101324205	101513241
ENSG0000017963	-2.47905057	4E-04	0	LACC1	protein_coding	laccase dom		13	43879284	43893932
ENSG0000013464	-0.183728815	4E-04	0	PUM1	protein_coding	pumilio RNA		1	30931506	31065991
ENSG0000026553	6.644282603	4E-04	0	FCGR1CP	unprocessed_p	Fc fragment		1	143874793	143883575
ENSG0000006499	0.365355208	4E-04	0	TAF11	protein_coding	TATA-box b		6	34877462	34888071
ENSG0000014873	0.41830311	4E-04	0	TCF7L2	protein_coding	transcriptior		10	112950247	113167678
ENSG0000004741	0.147919021	4E-04	0	TPR	protein_coding	translocatec		1	186311652	186375693
ENSG0000027872	-1.09323714	4E-04	0	CNTNAP2	protein_coding	contactin as	CHR_HSCHR		148217288	148419466
ENSG0000012586	-2.584520123	4E-04	0	BFSP1	protein_coding	beaded filar		20	17493905	17569220
ENSG0000026703	-0.550490632	4E-04	0	NA	NA	NA	NA		NA	NA
ENSG0000027626	0.445488549	4E-04	0	NSF	protein_coding	N-ethylmal	CHR_HSCHR		46419843	46586373
ENSG0000027458	-1.356943795	4E-04	0	DGKK	protein_coding	diacylglycer	X		50365409	50470825
ENSG0000006934	-0.245150171	4E-04	0	DNAJA2	protein_coding	DnaJ heat sl		16	46955362	46973674
ENSG0000013020	-3.021943291	4E-04	0	APOC1	protein_coding	apolipoprot		19	44914247	44919349
ENSG0000016044	0.37846125	4E-04	0	ZER1	protein_coding	zyg-11 relat		9	128729786	128772414
ENSG0000011904	-0.323332374	4E-04	0	UBE2B	protein_coding	ubiquitin co		5	134371469	134392108
ENSG0000007469	0.186294763	4E-04	0	HACD3	protein_coding	3-hydroxya		15	65530418	65578349
ENSG0000011383	0.433775247	4E-04	0	TBCCD1	protein_coding	TBCC doma		3	186546067	186570543
ENSG0000025726	0.534425712	4E-04	0	ZNF271P	transcribed_un	zinc finger p		18	35290282	35328390
ENSG0000017468	0.365417221	4E-04	0	B4GAT1	protein_coding	beta-1,4-gl		11	66345374	66347629

ENSG0000019836	-0.248122013	4E-04	0	ASPH	protein_coding	aspartate be	8	61500556	61714640
ENSG0000016315	-0.267525908	4E-04	0	VPS72	protein_coding	vacuolar pro	1	151176304	151195321
ENSG0000024989	-1.364836443	4E-04	0	LINC02495	transcribed_un	long interge	4	6200733	6239937
ENSG0000016292	0.380397495	4E-04	0	REL	protein_coding	REL proto-c	2	60881521	60931612
ENSG0000009293	0.312513826	4E-04	0	MFSD11	protein_coding	major facilit	17	76735865	76781449
ENSG0000017869	-0.503383631	4E-04	0	NSUN3	protein_coding	NOP2/Sun I	3	94062980	94131832
ENSG0000018541	0.507321082	4E-04	0	TARS3	protein_coding	threonyl-tRI	15	101653596	101724473
ENSG0000021157	-1.680650877	4E-04	0	MIR770	miRNA	microRNA 7	14	100852390	100852487
ENSG0000012794	-0.392577051	4E-04	0	POR	protein_coding	cytochrome	7	75899200	75986855
ENSG0000013276	-0.36816855	4E-04	0	DPH2	protein_coding	diphthamide	1	43970000	43973369
ENSG0000023594	-2.667902244	4E-04	0	ZNF815P	transcribed_un	zinc finger p	7	5823160	5854365
ENSG0000016992	0.204935509	4E-04	0	BRD3	protein_coding	bromodoma	9	134030305	134068535
ENSG0000021417	0.674806807	4E-04	0	AMZ2P1	transcribed_un	archaelysin	17	64966550	64975576
ENSG0000027644	-4.299261388	4E-04	0	AC005393.1	lncRNA	novel transc	19	39812972	39813555
ENSG0000016887	0.272764278	4E-04	0	DDX19A	protein_coding	DEAD-box I	16	70346861	70373383
ENSG0000026167	-1.024087507	4E-04	0	NA	NA	NA	NA	NA	NA
ENSG0000010488	-0.240507805	4E-04	0	DOT1L	protein_coding	DOT1 like h	19	2163933	2232578
ENSG0000013187	0.352349902	4E-04	0	SNRPA1	protein_coding	small nuclea	15	101281510	101295282
ENSG0000006500	-0.164958965	4E-04	0	AP3D1	protein_coding	adaptor rela	19	2100988	2164468
ENSG0000027354	1.596920041	4E-04	0	H4C12	protein_coding	H4 clustered	6	27831174	27831560
ENSG0000022663	1.052058509	4E-04	0	DDAH2	protein_coding	dimethylarg	CHR_HSCHR	31714445	31718024
ENSG0000010314	-0.766005956	4E-04	0	HCFC1R1	protein_coding	host cell fac	16	3022625	3024286
ENSG0000023703	1.198323595	4E-04	0	NDUFA6-D	lncRNA	NDUFA6 div	22	42090931	42137742
ENSG0000011523	-1.066828545	4E-04	0	ITGA4	protein_coding	integrin sub	2	181457202	181538940
ENSG0000019712	0.73970853	4E-04	0	ZNF772	protein_coding	zinc finger p	19	57466663	57477570
ENSG0000012176	-1.602586251	4E-04	0	FABP3	protein_coding	fatty acid bi	1	31365253	31376850
ENSG0000014620	-2.864254826	4E-04	0	ANO7	protein_coding	anoctamin 7	2	241188509	241225377
ENSG0000023338	-1.335458437	4E-04	0	AK4P3	processed_pse	adenylate ki	12	31615771	31616439
ENSG0000027127	0.782992835	4E-04	0	TMCC1-AS	lncRNA	TMCC1 anti	3	129893811	129918575
ENSG0000026226	-1.929896618	4E-04	0	U95743.1	lncRNA	novel transc	16	13728654	13779760
ENSG0000017520	-0.224640142	4E-04	0	DCTN2	protein_coding	dynactin sul	12	57530051	57547224
ENSG0000018368	0.203200249	4E-04	0	ALYREF	protein_coding	Aly/REF exp	17	81887835	81891586
ENSG0000026355	-6.622975469	4E-04	0	NA	NA	NA	NA	NA	NA
ENSG0000017403	0.701377421	4E-04	0	SLC25A30	protein_coding	solute carrie	13	45393316	45418455
ENSG0000018936	0.554239398	4E-04	0	KIAA0408	protein_coding	KIAA0408 [S	6	127438406	127459389
ENSG0000021435	0.538077528	4E-04	0	NEURL1B	protein_coding	neuralized E	5	172641263	172691540
ENSG0000019691	0.13627253	4E-04	0	ARHGEF12	protein_coding	Rho guanine	11	120336413	120489937
ENSG0000015925	0.312030417	4E-04	0	MORC3	protein_coding	MORC fami	21	36320189	36386148
ENSG0000018551	0.368149511	4E-04	0	BRCC3	protein_coding	BRCA1/BRC	X	155071420	155123074
ENSG0000012415	0.340709281	4E-04	0	PIGT	protein_coding	phosphatidy	20	45416084	45456934
ENSG0000014015	-0.354046945	4E-04	0	WDR20	protein_coding	WD repeat	14	102139503	102224847
ENSG0000021419	1.263659536	4E-04	0	SH3D21	protein_coding	SH3 domain	1	36306368	36329340
ENSG0000019885	0.481609128	4E-04	0	R3HDM4	protein_coding	R3H domain	19	896503	913245
ENSG0000013126	-0.1931569	4E-04	0	RLIM	protein_coding	ring finger p	X	74582976	74614624
ENSG0000011582	-0.364856385	4E-04	0	DCAF17	protein_coding	DDB1 and C	2	171434217	171485052
ENSG0000024940	-6.632139051	4E-04	0	AC015909.1	lncRNA	novel transc	17	50199876	50215922
ENSG0000017417	0.348664193	4E-04	0	TRMT10C	protein_coding	tRNA methy	3	101561868	101566446
ENSG0000010663	-0.457558116	4E-04	0	BCL7B	protein_coding	BAF chroma	7	73536356	73557690
ENSG0000010847	-1.166406051	4E-04	0	GALK1	protein_coding	galactokinas	17	75751594	75765236
ENSG0000011599	-0.189331509	4E-04	0	TRAK2	protein_coding	trafficking k	2	201377207	201451500
ENSG0000027714	-1.807937189	4E-04	0	LINC00869	transcribed_un	long interge	1	149606334	149705716
ENSG0000015381	0.403668657	4E-04	0	CMIP	protein_coding	c-Maf induc	16	81445170	81711762
ENSG0000017890	-0.259871931	4E-04	0	DPY19L3	protein_coding	dpy-19 like	19	32405543	32485895
ENSG0000010539	0.298696937	4E-04	0	BABAM1	protein_coding	BRISC and E	19	17267376	17281249
ENSG0000015111	0.531716184	4E-04	0	UEVLD	protein_coding	UEV and lac	11	18529609	18588747
ENSG0000011420	-1.459557589	4E-04	0	BCHE	protein_coding	butyrylcholi	3	165772904	165837462
ENSG0000011316	0.253089387	4E-04	0	CERT1	protein_coding	ceramide tra	5	75356345	75512138
ENSG0000012551	-2.141132519	4E-04	0	OPRL1	protein_coding	opioid relat	20	64080082	64100643

ENSG0000018936	0.564204958	4E-04	0	GSPT2	protein_coding	G1 to S pha	X	51743442	51746232
ENSG0000020441	1.088448575	4E-04	0	MSH5	protein_coding	mutS homo	6	31739677	31762676
ENSG0000016190	-0.28289725	4E-04	0	LEMD2	protein_coding	LEM domain	6	33771202	33789130
ENSG0000023990	-0.258739761	4E-04	0	ADSL	protein_coding	adenylosucc	22	40346500	40390463
ENSG0000018713	-0.471864668	4E-04	0	AKR1C1	protein_coding	aldo-keto re	10	4963253	4983283
ENSG0000017091	0.917165891	4E-04	0	NUDT6	protein_coding	nudix hydro	4	122888697	122922968
ENSG0000027408	-1.438861873	4E-04	0	PRIMA1	protein_coding	proline rich	CHR_HSCHR	93718298	93788481
ENSG0000013671	0.352109956	4E-04	0	SAP130	protein_coding	Sin3A assoc	2	127941217	128028120
ENSG0000027778	3.693499258	4E-04	0	AC068870.2	lncRNA	novel transc	15	80999593	80999981
ENSG0000012255	0.424724452	4E-04	0	HERPUD2	protein_coding	HERPUD far	7	35632659	35697459
ENSG0000016067	-0.22172246	4E-04	0	CHTOP	protein_coding	chromatin ta	1	153633982	153646306
ENSG0000007367	-1.25629489	4E-04	0	ADAM11	protein_coding	ADAM meta	17	44758988	44781846
ENSG0000013926	0.82445295	4E-04	0	LRIG3	protein_coding	leucine rich	12	58872149	58920504
ENSG0000014056	-0.450562362	4E-04	0	FURIN	protein_coding	furin, paired	15	90868588	90883458
ENSG0000016432	-0.344267064	4E-04	0	TENT2	protein_coding	terminal nuc	5	79612120	79686648
ENSG0000027277	0.624190154	4E-04	0	AC245060.4	transcribed_un	BMS1, ribos	22	22303224	22310401
ENSG0000016163	-1.908090344	4E-04	0	ITGA5	protein_coding	integrin sub	12	54395261	54419266
ENSG0000014314	0.323111753	4E-04	0	ALDH9A1	protein_coding	aldehyde de	1	165662216	165698863
ENSG0000015647	-0.222349271	4E-04	0	PTDSS1	protein_coding	phosphatidy	8	96261902	96336995
ENSG0000023733	1.593266188	4E-04	0	MSH5	protein_coding	mutS homo	CHR_HSCHR	31730753	31753433
ENSG0000017444	0.278974875	4E-04	0	ZWILCH	protein_coding	zwilch kinet	15	66504959	66550130
ENSG0000027779	0.36422643	4E-04	0	PSMB3	protein_coding	proteasome	17	38752741	38764225
ENSG0000013488	-0.294746502	4E-04	0	UBAC2	protein_coding	UBA domain	13	99200774	99386504
ENSG0000018315	-0.562275396	4E-04	0	GPR19	protein_coding	G protein-c	12	12660890	12696207
ENSG0000010865	0.344487183	4E-04	0	UTP6	protein_coding	UTP6 small	17	31860904	31901708
ENSG0000017489	-4.078206949	4E-04	0	SLC66A1L	transcribed_un	solute carrie	3	157543246	157677749
ENSG0000018019	0.271260247	4E-04	0	RCC1	protein_coding	regulator of	1	28505943	28539300
ENSG0000019841	1.315909884	4E-04	0	ZNF658B	transcribed_un	zinc finger p	9	39443815	39508885
ENSG0000013796	0.943015495	4E-04	0	SLC44A5	protein_coding	solute carrie	1	75202131	75611116
ENSG0000013179	-0.788922226	4E-04	0	CLUHP3	transcribed_un	clustered m	16	31700590	31711986
ENSG0000023627	-0.796004283	4E-04	0	FLOT1	protein_coding	flotillin 1 [So	CHR_HSCHR	30772367	30787391
ENSG0000020560	-0.202040236	4E-04	0	EIF3CL	protein_coding	eukaryotic t	16	28379579	28403841
ENSG0000017689	0.975684755	4E-04	0	TCEANC	protein_coding	transcription	X	13653189	13681964
ENSG0000017283	-1.64340555	4E-04	0	SSH3	protein_coding	slingshot pr	11	67303478	67312607
ENSG0000017298	0.632357093	4E-04	0	GXYLT2	protein_coding	glucoside xy	3	72888046	72998138
ENSG0000024371	-0.439895124	4E-04	0	NPIPB5	protein_coding	nuclear pore	16	22479121	22536521
ENSG0000010909	-0.500617811	5E-04	0	PMP22	protein_coding	peripheral n	17	15229773	15272292
ENSG0000017208	-0.511149378	5E-04	0	MOB3A	protein_coding	MOB kinase	19	2071036	2096673
ENSG0000013582	0.84817337	5E-04	0	RNASEL	protein_coding	ribonuclease	1	182573634	182589256
ENSG0000010052	0.275282606	5E-04	0	DDHD1	protein_coding	DDHD dom	14	53036745	53153323
ENSG0000027435	1.044296371	5E-04	0	ZNF707	protein_coding	zinc finger p	CHR_HSCHR	143671459	143700905
ENSG0000020637	-0.560866098	5E-04	0	FLOT1	protein_coding	flotillin 1 [So	CHR_HSCHR	30717966	30732991
ENSG0000013733	0.605649872	5E-04	0	MDC1	protein_coding	mediator of	6	30699807	30717447
ENSG0000016022	-0.531305139	5E-04	0	GATD3A	protein_coding	glutamine a	21	44133610	44210114
ENSG0000024237	-0.277456483	5E-04	0	EIF6	protein_coding	eukaryotic t	20	35278907	35284985
ENSG0000017793	-0.309483439	5E-04	0	ZNF354C	protein_coding	zinc finger p	5	179060373	179083977
ENSG0000015703	0.952397196	5E-04	0	EXOG	protein_coding	exo/endonu	3	38496127	38542161
ENSG0000023601	2.496677814	5E-04	0	ASMTL-AS1	lncRNA	ASMTL antis	X	1401769	1414028
ENSG0000010136	0.209996567	5E-04	0	NOP56	protein_coding	NOP56 ribo	20	2652593	2658393
ENSG0000023185	1.914273299	5E-04	0	AL162377.1	lncRNA	novel transc	13	51803838	51813832
ENSG0000018543	1.221391136	5E-04	0	SH3BGR	protein_coding	SH3 domain	21	39445855	39515506
ENSG0000012198	-0.466108593	5E-04	0	ZRANB3	protein_coding	zinc finger R	2	135136916	135531218
ENSG0000019787	-1.121798223	5E-04	0	CYRIA	protein_coding	CYFIP relate	2	16549459	16666331
ENSG0000014144	-0.358843397	5E-04	0	OSBPL1A	protein_coding	oxysterol bi	18	24162045	24397880
ENSG0000025709	-0.410437161	5E-04	0	DENND11	protein_coding	DENN doma	7	141656728	141702166
ENSG0000013953	0.80169691	5E-04	0	SUOX	protein_coding	sulfite oxida	12	55997180	56006641
ENSG0000018548	-0.428280666	5E-04	0	ROR1	protein_coding	receptor tyr	1	63774017	64181498
ENSG0000018062	-0.8679969	5E-04	0	PCGF5	protein_coding	polycomb g	10	91163012	91284337

ENSG0000016352	1.242141183	5E-04	0	GLB1L	protein_codin	galactosidas	2	219236598	219245478
ENSG0000021511	-0.262680494	5E-04	0	UBXN2B	protein_codin	UBX domair	8	58411359	58451501
ENSG0000024496	1.624362288	5E-04	0	LIFR-AS1	lncRNA	LIFR antisen	5	38556765	38671216
ENSG0000014758	0.522241932	5E-04	0	MRPS28	protein_codin	mitochondr	8	79918717	80030289
ENSG0000021011	-1.041126228	5E-04	0	MT-TM	Mt_tRNA	mitochondr	MT	4402	4469
ENSG0000013799	0.358380325	5E-04	0	RTCA	protein_codin	RNA 3'-terr	1	100266216	100292769
ENSG0000016261	0.165347584	5E-04	0	FUBP1	protein_codin	far upstrean	1	77944055	77979110
ENSG0000015293	0.230808328	5E-04	0	RAB3C	protein_codin	RAB3C, mer	5	58582221	58859394
ENSG0000016313	0.4552023	5E-04	0	PACRGL	protein_codin	parkin coreg	4	20696282	20752907
ENSG0000018647	-0.282222858	5E-04	0	PCLO	protein_codin	piccolo pres	7	82754012	83162930
ENSG0000018801	-4.877201839	5E-04	0	S100A3	protein_codin	S100 calciur	1	153547329	153549258
ENSG0000023035	3.230671828	5E-04	0	TPI1P2	transcribed_pr	triosephosp	7	129055223	129057239
ENSG0000018482	-1.31502787	5E-04	0	ZBTB7C	protein_codin	zinc finger a	18	48026672	48410752
ENSG0000017298	-1.307569509	5E-04	0	SH3RF3	protein_codin	SH3 domair	2	109129205	109504634
ENSG0000002515	-0.299739574	5E-04	0	HSF2	protein_codin	heat shock t	6	122399551	122433119
ENSG0000014330	0.43535874	5E-04	0	RRNAD1	protein_codin	ribosomal R	1	156728442	156736960
ENSG0000010113	-0.320537207	5E-04	0	PFDN4	protein_codin	prefoldin su	20	54208087	54228052
ENSG0000016610	1.944837616	5E-04	0	GLB1L3	protein_codin	galactosidas	11	134274245	134319564
ENSG0000012794	-0.463810294	5E-04	0	HIP1	protein_codin	huntingtin ir	7	75533298	75738962
ENSG0000018561	0.225570281	5E-04	0	PCGF3	protein_codin	polycomb g	4	705748	770640
ENSG0000014785	0.257789167	5E-04	0	AK3	protein_codin	adenylate ki	9	4709556	4742043
ENSG0000017678	-0.357652636	5E-04	0	RUFY1	protein_codin	RUN and FY	5	179550554	179610012
ENSG0000013346	0.493096838	5E-04	0	C1QTNF6	protein_codin	C1q and TN	22	37180166	37199385
ENSG0000016999	-0.530897685	5E-04	0	NLGN2	protein_codin	neuroligin 2	17	7404874	7419860
ENSG0000026059	-0.976018579	5E-04	0	AC012531.	lncRNA	novel transc	12	54019910	54022589
ENSG0000018454	-0.944510387	5E-04	0	DUSP8	protein_codin	dual specific	11	1554051	1572271
ENSG0000016409	0.197508623	5E-04	0	WDR82	protein_codin	WD repeat	3	52254434	52288020
ENSG0000007556	-0.25898545	5E-04	0	TMEM131	protein_codin	transmembr	2	97756333	97995948
ENSG0000017557	0.544197497	5E-04	0	C11orf68	protein_codin	chromosom	11	65916810	65919062
ENSG0000019636	-0.229740421	5E-04	0	TRRAP	protein_codin	transformat	7	98877933	99050831
ENSG0000017053	-1.908887792	5E-04	0	TMC7	protein_codin	transmembr	16	18983934	19063942
ENSG0000000886	0.259358311	5E-04	0	HEATR5B	protein_codin	HEAT repea	2	36968383	37084372
ENSG0000016861	0.752820989	5E-04	0	ZSWIM1	protein_codin	zinc finger S	20	45881227	45885266
ENSG0000022568	-1.333851609	5E-04	0	DXO	protein_codin	decapping e	CHR_HSCHR	31957356	31959838
ENSG0000012175	-0.452497619	5E-04	0	ADGRB2	protein_codin	adhesion G	1	31727117	31764893
ENSG0000016659	0.396772051	5E-04	0	CIAO2B	protein_codin	cytosolic iro	16	66932065	66934423
ENSG0000016764	-0.640126411	5E-04	0	YIF1B	protein_codin	Yip1 interac	19	38303558	38317273
ENSG0000013148	-1.534078026	5E-04	0	AOC2	protein_codin	amine oxida	17	42844580	42850707
ENSG0000018518	-1.998620561	5E-04	0	SIGIRR	protein_codin	single Ig an	11	405716	417455
ENSG0000007868	0.276339088	5E-04	0	TNRC6C	protein_codin	trinucleotide	17	77959240	78108835
ENSG0000006317	-0.61837258	5E-04	0	SPHK2	protein_codin	sphingosine	19	48619291	48630717
ENSG0000010527	-0.334762359	5E-04	0	CLIP3	protein_codin	CAP-Gly do	19	36014660	36033343
ENSG0000023222	-2.528186022	5E-04	0	NA	NA	NA	NA	NA	NA
ENSG0000011420	0.33292129	5E-04	0	PDCD10	protein_codin	programme	3	167683298	167734939
ENSG0000027823	1.240137332	5E-04	0	RNA5-8SN	rRNA	RNA, 5.8S ri	21	8212572	8212724
ENSG0000008251	0.265726719	5E-04	0	GEMIN5	protein_codin	gem nuclea	5	154887411	154938211
ENSG0000008606	0.159897623	5E-04	0	DNAJA1	protein_codin	DnaJ heat sl	9	33025273	33039907
ENSG0000016602	0.49002563	5E-04	0	R3HCC1L	protein_codin	R3H domair	10	98134624	98244897
ENSG0000023517	0.645043202	5E-04	0	HGH1	protein_codin	HGH1 homo	8	144137774	144140851
ENSG0000016379	-0.288382418	5E-04	0	SLC4A1AP	protein_codin	solute carrie	2	27663471	27694976
ENSG0000012217	-1.531732075	5E-04	0	FMOD	protein_codin	fibromoduli	1	203340628	203351758
ENSG0000016471	-0.444105005	5E-04	0	BRI3	protein_codin	brain protei	7	98252379	98310441
ENSG0000023356	-0.865523693	5E-04	0	DHX16	protein_codin	DEAH-box I	CHR_HSCHR	30642952	30662900
ENSG0000013114	-0.185733208	5E-04	0	COX4I1	protein_codin	cytochrome	16	85798633	85807068
ENSG0000013968	-0.277920178	5E-04	0	ESD	protein_codin	esterase D [	13	46771256	46797420
ENSG0000021346	-2.37002581	5E-04	0	FIRRE	lncRNA	firre interge	X	131688779	131830862
ENSG0000016982	0.280193923	5E-04	0	CSGALNAC	protein_codin	chondroitin	10	43138445	43185302
ENSG0000019806	0.261338434	5E-04	0	MARCHF5	protein_codin	membrane	10	92291167	92353964

ENSG0000024647	1.697930582	5E-04	0	AF131216.1	lncRNA	uncharacter	8	11315859	11325429
ENSG0000014317	1.330471049	5E-04	0	TBX19	protein_coding	T-box trans	1	168280877	168314426
ENSG0000018601	1.112200037	5E-04	0	AC021092.1	lncRNA	novel transc	19	44103007	44113183
ENSG0000016304	0.59572668	5E-04	0	CCDC74A	protein_coding	coiled-coil c	2	131527675	131533666
ENSG0000019852	-0.323794304	5E-04	0	GPN1	protein_coding	GPN-loop G	2	27628247	27651511
ENSG0000018849	-0.868458315	5E-04	0	C19orf54	protein_coding	chromosom	19	40740856	40751553
ENSG0000025153	-2.659573381	5E-04	0	AC005324.3	protein_coding	novel tripart	17	15571491	15651653
ENSG0000017926	0.281036464	5E-04	0	RAD23A	protein_coding	RAD23 hom	19	12945855	12953642
ENSG0000016316	-0.413589927	5E-04	0	RNF149	protein_coding	ring finger p	2	101271219	101308701
ENSG0000008919	-0.323629232	5E-04	0	TRMT6	protein_coding	tRNA methy	20	5937228	5950558
ENSG0000022935	-1.538588829	5E-04	0	DPY19L1P1	unprocessed_p	DPY19L1 ps	7	32580949	32761787
ENSG0000013378	-0.429925935	5E-04	0	SWAP70	protein_coding	switching B	11	9664077	9752993
ENSG0000023000	2.001310195	5E-04	0	ALMS1-IT1	lncRNA	ALMS1 intro	2	73456764	73459482
ENSG0000010063	0.171393231	5E-04	0	ERH	protein_coding	ERH mRNA	14	69380128	69398299
ENSG0000009606	0.190103278	5E-04	0	SRPK1	protein_coding	SRSF protei	6	35832966	35921342
ENSG0000012213	-6.59832232	5E-04	0	OBP2A	protein_coding	odorant bin	9	135546139	135549969
ENSG0000011900	0.432232354	5E-04	0	CYP20A1	protein_coding	cytochrome	2	203238977	203306026
ENSG0000015292	0.388622909	5E-04	0	ZNF117	protein_coding	zinc finger p	7	64971772	65006684
ENSG0000026865	1.174914678	5E-04	0	LINC00664	lncRNA	long interge	19	21483374	21503238
ENSG0000012756	0.479102661	5E-04	0	PKMYT1	protein_coding	protein kina	16	2968024	2980479
ENSG0000027505	-0.182758423	5E-04	0	PPP4R3B	protein_coding	protein pho	2	55547292	55618880
ENSG0000013769	-3.302016991	5E-04	0	TRIM29	protein_coding	tripartite mo	11	120111286	120185529
ENSG0000010513	0.7730562	5E-04	0	ZNF419	protein_coding	zinc finger p	19	57487711	57496098
ENSG0000027379	-0.888253359	5E-04	0	DUSP8	protein_coding	dual specific	CHR_HSCHR	1559754	1577630
ENSG0000027135	2.115472599	6E-04	0	NA	NA	NA	NA	NA	NA
ENSG0000019891	0.248283087	6E-04	0	DZIP3	protein_coding	DAZ interac	3	108589705	108694840
ENSG0000001061	-2.881715531	6E-04	0	CD4	protein_coding	CD4 molecu	12	6786858	6820799
ENSG0000016960	0.348201743	6E-04	0	C15orf40	protein_coding	chromosom	15	82988441	83011641
ENSG0000016749	-0.230318952	6E-04	0	GATAD2A	protein_coding	GATA zinc f	19	19385826	19508931
ENSG0000016930	-1.651219948	6E-04	0	STK32A	protein_coding	serine/threo	5	147234963	147387855
ENSG0000026121	1.97908544	6E-04	0	AL162231.4	lncRNA	novel transc	9	34661903	34666029
ENSG0000022864	-6.540404826	6E-04	0	AC079779.2	lncRNA	novel transc	2	286419	301515
ENSG0000007605	-0.351813586	6E-04	0	RBM7	protein_coding	RNA binding	11	114400030	114414203
ENSG0000010166	0.540907976	6E-04	0	SMAD7	protein_coding	SMAD famil	18	48919853	48952052
ENSG0000011363	-0.439698954	6E-04	0	TTC33	protein_coding	tetratricope	5	40512333	40755963
ENSG0000023707	-0.502729903	6E-04	0	TRIM27	protein_coding	tripartite mo	CHR_HSCHR	28903816	28924807
ENSG0000016632	-0.207426059	6E-04	0	TRIM44	protein_coding	tripartite mo	11	35662775	35818007
ENSG0000012274	-0.26465301	6E-04	0	DCAF10	protein_coding	DDB1 and C	9	37800554	37867666
ENSG0000014591	0.33772063	6E-04	0	RMND5B	protein_coding	required for	5	178130996	178150568
ENSG0000011639	0.889063358	6E-04	0	KCNC4	protein_coding	potassium v	1	110210314	110283100
ENSG0000023204	-0.828219976	6E-04	0	ZBED9	protein_coding	zinc finger B	6	28570535	28616212
ENSG0000014606	-0.304963439	6E-04	0	TRIM41	protein_coding	tripartite mo	5	181222499	181235808
ENSG0000016351	0.32261608	6E-04	0	ANKZF1	protein_coding	ankyrin repe	2	219229783	219236679
ENSG0000017110	-0.37584187	6E-04	0	INSR	protein_coding	insulin recep	19	7112255	7294414
ENSG0000018414	-0.894866829	6E-04	0	CNTN2	protein_coding	contactin 2	1	205042937	205078289
ENSG0000014019	0.355982497	6E-04	0	SLC12A6	protein_coding	solute carrie	15	34229784	34338060
ENSG0000000600	0.269185898	6E-04	0	GDE1	protein_coding	glycerophos	16	19501693	19522123
ENSG0000026696	0.632506416	6E-04	0	AC067852.2	lncRNA	novel transc	17	42552436	42554748
ENSG0000027082	1.612724786	6E-04	0	AC016727.1	lncRNA	novel transc	2	61471188	61484130
ENSG0000025331	-1.337433649	6E-04	0	AC078906.1	lncRNA	novel transc	8	72196334	72202269
ENSG0000017888	-2.057236703	6E-04	0	RFLNA	protein_coding	refilin A [So	12	123973241	124316024
ENSG0000007812	0.325341812	6E-04	0	ACER3	protein_coding	alkaline cera	11	76860867	77026797
ENSG0000026528	-2.32141877	6E-04	0	AC005828.4	lncRNA	novel transc	17	63430468	63432211
ENSG0000026220	2.916385692	6E-04	0	AC007952.4	lncRNA	novel transc	17	19112000	19112636
ENSG0000014870	0.268584264	6E-04	0	ADD3	protein_coding	adducin 3 [S	10	109996368	110135565
ENSG0000023201	-2.502087312	6E-04	0	NA	NA	NA	NA	NA	NA
ENSG0000013030	-3.481243947	6E-04	0	BST2	protein_coding	bone marro	19	17402939	17405630
ENSG0000013173	0.478442394	6E-04	0	ZCCHC9	protein_coding	zinc finger C	5	81301587	81313297

ENSG0000017527	0.710409615	6E-04	0	CENPS	protein_coding	centromere	1	10430433	10442808
ENSG0000026888	-2.54701573	6E-04	0	PNMA6B	unprocessed_p	PNMA famil	X	153075769	153076968
ENSG0000010910	-0.552453431	6E-04	0	ALDOC	protein_coding	aldolase, fru	17	28573115	28576948
ENSG0000012306	-0.328240132	6E-04	0	DDX54	protein_coding	DEAD-box l	12	113157173	113185479
ENSG0000020573	-0.434585306	6E-04	0	ITPRIPL2	protein_coding	ITPRIP like 2	16	19113932	19121629
ENSG0000027110	-1.382028089	6E-04	0	AC008555.4	lncRNA	novel transc	19	34849046	34860747
ENSG0000012387	1.728135078	6E-04	0	ZNF137P	transcribed_un	zinc finger p	19	52588505	52597345
ENSG0000012457	0.437704403	6E-04	0	ABCC10	protein_coding	ATP binding	6	43427366	43450427
ENSG0000024727	1.334714685	6E-04	0	ZBED5-AS1	lncRNA	ZBED5 antis	11	10858179	10908972
ENSG0000027631	-6.53455171	6E-04	0	AL357033.3	lncRNA	novel transc	20	62651272	62652186
ENSG0000026759	6.517386114	6E-04	0	AC011446.2	lncRNA	novel transc	19	13153071	13154193
ENSG0000024593	-0.5584225	6E-04	0	LINC01184	lncRNA	long interge	5	127939152	128083172
ENSG0000015169	-0.263495563	6E-04	0	ASAP2	protein_coding	ArfGAP with	2	9206765	9405683
ENSG0000018631	-0.699250155	6E-04	0	CA5BP1	transcribed_un	carbonic an	X	15674932	15703724
ENSG0000016495	-0.695069724	6E-04	0	TMEM67	protein_coding	transmembr	8	93754844	93819234
ENSG0000010881	-0.274602157	6E-04	0	PPP1R9B	protein_coding	protein pho	17	50133737	50150677
ENSG0000018838	-1.359343932	6E-04	0	JAKMIP3	protein_coding	Janus kinase	10	132036336	132184858
ENSG0000012510	-0.148259631	6E-04	0	CNOT1	protein_coding	CCR4-NOT	16	58519951	58629885
ENSG0000017187	0.551496597	6E-04	0	FRMD5	protein_coding	FERM doma	15	43870761	44195271
ENSG0000011043	-0.361102104	6E-04	0	PDHX	protein_coding	pyruvate de	11	34915829	35020591
ENSG0000021554	2.319114429	6E-04	0	FRG1JP	unprocessed_p	FSHD regio	9	63832127	63859641
ENSG0000010414	0.594565378	6E-04	0	OIP5	protein_coding	Opa interac	15	41309273	41332591
ENSG0000003469	0.535334567	6E-04	0	PEX3	protein_coding	peroxisoma	6	143450805	143490616
ENSG0000020461	0.513534967	6E-04	0	ZNF616	protein_coding	zinc finger p	19	52113091	52139938
ENSG0000023190	2.542005128	6E-04	0	IDH1-AS1	lncRNA	IDH1 antise	2	208255247	208256181
ENSG0000019771	-1.341148595	6E-04	0	ZNF460	protein_coding	zinc finger p	19	57280051	57294069
ENSG0000013240	-0.207773854	6E-04	0	TBC1D14	protein_coding	TBC1 doma	4	6909242	7033118
ENSG0000020628	-0.882593811	6E-04	0	WDR46	protein_coding	WD repeat	CHR_HSCHR	33207935	33218358
ENSG0000016601	0.158788872	6E-04	0	TAF1D	protein_coding	TATA-box b	11	93729948	93784391
ENSG0000021369	1.989119138	6E-04	0	SEC14L1P1	processed_pse	SEC14 like 1	11	43897456	43899636
ENSG0000023553	0.934044475	6E-04	0	MSC-AS1	lncRNA	MSC antise	8	71828167	72118393
ENSG0000023485	-0.713835849	6E-04	0	RPL23AP42	processed_pse	ribosomal p	3	161429127	161429597
ENSG0000011173	0.276703764	6E-04	0	RAB35	protein_coding	RAB35, mer	12	120095099	120117502
ENSG0000018596	0.271138005	6E-04	0	BICD2	protein_coding	BICD cargo	9	92711363	92764833
ENSG0000027849	-0.289196229	6E-04	0	RRN3	protein_coding	RRN3 homo	CHR_HSCHR	15002502	15036801
ENSG0000010347	0.241792935	6E-04	0	RBL2	protein_coding	RB transcrip	16	53433977	53491648
ENSG0000021336	-3.057483082	6E-04	0	AP000593.1	processed_pse	U2 small nu	11	72280151	72281178
ENSG0000013267	-0.207959804	6E-04	0	DAP3	protein_coding	death assoc	1	155687960	155739010
ENSG0000015078	-0.382842176	6E-04	0	PTS	protein_coding	6-pyruvoylt	11	112226367	112269955
ENSG0000021319	0.884900066	6E-04	0	ASIC3	protein_coding	acid sensing	7	151048292	151052756
ENSG0000016315	-0.490445841	6E-04	0	LYSMD1	protein_coding	LysM doma	1	151159748	151165948
ENSG0000016891	-0.375810766	6E-04	0	SLC35G2	protein_coding	solute carrie	3	136818647	136855888
ENSG0000016412	-2.263424453	6E-04	0	GASK1B	protein_coding	golgi associ	4	158124474	158173318
ENSG0000008902	-0.333737361	6E-04	0	MAPKAPK5	protein_coding	MAPK activa	12	111842228	111902222
ENSG0000019885	-2.570360434	6E-04	0	HSD3BP5	transcribed_un	hydroxy-de	1	119601340	119609250
ENSG0000017445	0.789394776	6E-04	0	C12orf76	protein_coding	chromosom	12	110027028	110073634
ENSG0000027174	1.677520448	6E-04	0	AC114490.2	protein_coding	novel transc	1	34981533	35031741
ENSG0000011236	-0.689714148	6E-04	0	FIG4	protein_coding	FIG4 phospl	6	109690609	109878098
ENSG0000026549	-0.301293361	6E-04	0	RNF115	protein_coding	ring finger p	1	145738868	145824095
ENSG0000019814	0.542286417	6E-04	0	SOWAHC	protein_coding	sosondowah	2	109614364	109618990
ENSG0000003747	-0.218230826	6E-04	0	NSUN2	protein_coding	NOP2/Sun F	5	6599239	6633291
ENSG0000005414	0.364424503	7E-04	0	PHPT1	protein_coding	phosphohis	9	136848724	136851027
ENSG0000013296	0.312455168	7E-04	0	HMGB1P5	transcribed_pr	high mobilit	3	22381819	22382929
ENSG0000023043	-2.25279276	7E-04	0	LINC01650	lncRNA	long interge	1	95351251	95352676
ENSG0000011715	-0.352372223	7E-04	0	SSX2IP	protein_coding	SSX family r	1	84643707	84690803
ENSG0000017095	1.276644317	7E-04	0	ZNF415	protein_coding	zinc finger p	19	53107879	53133077
ENSG0000023012	-1.025241796	7E-04	0	IER3	protein_coding	immediate e	CHR_HSCHR	30787857	30789212
ENSG0000012233	0.493440458	7E-04	0	SERAC1	protein_coding	serine active	6	158109519	158168280

ENSG0000023669	0.854103816	7E-04	0	EIF1AXP1	processed_pse	EIF1AX pseu	1	16685621	16686055
ENSG0000022831	-0.74443683	7E-04	0	GUSBP11	lncRNA	GUSB pseuc	22	23638487	23717356
ENSG0000013686	-0.22522095	7E-04	0	SLC31A1	protein_coding	solute carrie	9	113221544	113264492
ENSG0000013165	-0.23985738	7E-04	0	TRAF7	protein_coding	TNF recepto	16	2155698	2178129
ENSG0000027904	2.513409023	7E-04	0	AC080080.1	TEC	tec	7	17940503	17942922
ENSG0000016392	0.597353213	7E-04	0	RPL39L	protein_coding	ribosomal p	3	187120948	187180908
ENSG0000013652	0.259063461	7E-04	0	NDUFB5	protein_coding	NADH:ubiqu	3	179604690	179627647
ENSG0000019826	-0.198227231	7E-04	0	HELZ	protein_coding	helicase witl	17	67070444	67245989
ENSG0000022204	-1.046783328	7E-04	0	CYTOR	lncRNA	cytoskeletor	2	87454781	87636740
ENSG0000011528	-0.362167436	7E-04	0	NDUFS7	protein_coding	NADH:ubiqu	19	1383527	1395589
ENSG0000001608	-0.245396098	7E-04	0	ISL1	protein_coding	ISL LIM hom	5	51383448	51394730
ENSG0000014096	-0.865143217	7E-04	0	OSGIN1	protein_coding	oxidative str	16	83931311	83966332
ENSG0000017506	0.636228524	7E-04	0	GK5	protein_coding	glycerol kina	3	142157527	142225592
ENSG0000013640	-2.52193785	7E-04	0	TM6SF1	protein_coding	transmembr	15	83107572	83144854
ENSG0000016627	-0.497082132	7E-04	0	BORCS7	protein_coding	BLOC-1 rela	10	102854259	102864961
ENSG0000008239	-0.289802816	7E-04	0	EPB41L3	protein_coding	erythrocyte	18	5392381	5630700
ENSG0000014380	-0.970277034	7E-04	0	PSEN2	protein_coding	presenilin 2	1	226870184	226896105
ENSG0000012755	-0.47599803	7E-04	0	GFER	protein_coding	growth facto	16	1984193	1987749
ENSG0000013762	-1.739794218	7E-04	0	DDX60	protein_coding	DExD/H-bo	4	168216294	168318804
ENSG0000024018	0.744205705	7E-04	0	PCDHGC3	protein_coding	protocadhe	5	141475947	141512977
ENSG0000010795	0.349473432	7E-04	0	PITRM1	protein_coding	pitrilysin me	10	3137728	3172841
ENSG0000016430	0.463687119	7E-04	0	PRIMPOL	protein_coding	primase anc	4	184649667	184694963
ENSG0000013875	0.166150741	7E-04	0	SEPTIN11	protein_coding	septin 11 [S	4	76949703	77040384
ENSG0000016222	-0.537468255	7E-04	0	TAF6L	protein_coding	TATA-box b	11	62771357	62787342
ENSG0000017288	-4.296256891	7E-04	0	EGFL7	protein_coding	EGF like dor	9	136658856	136672678
ENSG0000010067	0.830465216	7E-04	0	SLC8A3	protein_coding	solute carrie	14	70044215	70189070
ENSG0000012068	-0.531471274	7E-04	0	WBP4	protein_coding	WW domair	13	41061509	41084006
ENSG0000016657	0.394760566	7E-04	0	NDEL1	protein_coding	nudE neuro	17	8413131	8490411
ENSG0000010003	1.562254036	7E-04	0	GGT1	protein_coding	gamma-glu	22	24594811	24629005
ENSG0000019775	-0.309587561	7E-04	0	RPL37A	protein_coding	ribosomal p	2	216498825	216579180
ENSG0000020647	-1.226120164	7E-04	0	IER3	protein_coding	immediate e	CHR_HSCHR	30732716	30734071
ENSG0000016078	0.270994716	7E-04	0	SLC25A44	protein_coding	solute carrie	1	156193932	156212796
ENSG0000023232	-1.786612919	7E-04	0	TAP2	protein_coding	transporter	CHR_HSCHR	32912700	32929495
ENSG0000011986	0.29903548	7E-04	0	CNRIP1	protein_coding	cannabinoic	2	68284171	68320051
ENSG0000017145	0.444927142	7E-04	0	CDK5R2	protein_coding	cyclin deper	2	218959666	218962155
ENSG0000027934	3.594881301	7E-04	0	AC021945.1	TEC	TEC	8	119838736	119840385
ENSG0000025412	1.150953233	7E-04	0	PCDHGB7	protein_coding	protocadhe	5	141417645	141512975
ENSG0000017170	0.51050294	7E-04	0	TCEA2	protein_coding	transcriptior	20	64049836	64072347
ENSG0000012953	0.283773379	7E-04	0	MIS18BP1	protein_coding	MIS18 bindi	14	45203190	45253540
ENSG0000016887	0.773692938	7E-04	0	ATOH8	protein_coding	atonal bHLH	2	85751344	85791383
ENSG0000015400	-0.188072262	7E-04	0	PPP2R5E	protein_coding	protein pho	14	63371364	63543377
ENSG0000006064	0.709233268	7E-04	0	PIGV	protein_coding	phosphatidy	1	26787054	26800659
ENSG0000013923	0.387013363	7E-04	0	LLPH	protein_coding	LLP homolo	12	66116555	66130750
ENSG0000010879	-0.332389526	7E-04	0	EZH1	protein_coding	enhancer of	17	42700275	42745049
ENSG0000017818	0.843561891	7E-04	0	ZNF454	protein_coding	zinc finger p	5	178941191	178966433
ENSG0000010551	0.526155752	7E-04	0	RAB3D	protein_coding	RAB3D, mer	19	11322068	11346270
ENSG0000010878	-0.501743725	7E-04	0	NAGLU	protein_coding	N-acetyl-al	17	42536241	42544449
ENSG0000022494	0.958700454	7E-04	0	AL353150.1	lncRNA	novel transc	9	89088604	89109934
ENSG0000027697	-1.187315777	7E-04	0	HYDIN2	transcribed_un	HYDIN axor	1	146472566	146914294
ENSG0000012344	-0.508393005	7E-04	0	KBTBD4	protein_coding	kelch repeat	11	47572197	47578976
ENSG0000018416	0.55751342	7E-04	0	NR2C2AP	protein_coding	nuclear rece	19	19201409	19203414
ENSG0000014151	-0.818888172	7E-04	0	CCDC40	protein_coding	coiled-coil c	17	80036632	80100613
ENSG0000023660	-0.825786628	7E-04	0	ZNF853	protein_coding	zinc finger p	7	6615610	6624290
ENSG0000010269	0.310260404	7E-04	0	PARP4	protein_coding	poly(ADP-ri	13	24420931	24512778
ENSG0000022408	0.637466166	7E-04	0	PPM1F-AS1	lncRNA	PPM1F antis	22	21938269	21977632
ENSG0000014232	-0.755469733	7E-04	0	RNPEPL1	protein_coding	arginyl amir	2	240565804	240581372
ENSG0000016032	0.590296402	7E-04	0	CACFD1	protein_coding	calcium cha	9	133459965	133470848
ENSG0000016697	-2.322900074	7E-04	0	EVA1C	protein_coding	eva-1 homo	21	32412006	32515397

ENSG0000000674	-0.34204247	7E-04	0	ELAC2	protein_coding	elaC ribonu	17	12991612	13018065
ENSG0000016990	0.221707327	7E-04	0	TOR1AIP2	protein_coding	torsin 1A int	1	179839967	179877803
ENSG0000027722	2.761106249	7E-04	0	H2BC7	protein_coding	H2B clustere	6	26199520	26200715
ENSG0000026247	-0.704657904	7E-04	0	NA	NA	NA	NA	NA	NA
ENSG0000014578	0.210126625	7E-04	0	ATG12	protein_coding	autophagy r	5	115828200	115841837
ENSG0000018268	0.817335828	7E-04	0	BRICD5	protein_coding	BRICHOS dc	16	2209253	2211950
ENSG0000007075	-0.129478673	7E-04	0	PABPC1	protein_coding	poly(A) binc	8	100685816	100722809
ENSG0000013135	0.512677931	7E-04	0	HAUS8	protein_coding	HAUS augm	19	17049729	17075625
ENSG0000009253	-0.2698913	7E-04	0	SNAP23	protein_coding	synaptosom	15	42491233	42545356
ENSG0000023020	-0.546324317	7E-04	0	AL450405.1	processed_pse	ribosomal p	6	117998975	117999448
ENSG0000014484	0.274769671	7E-04	0	ATG3	protein_coding	autophagy r	3	112532510	112562046
ENSG0000023212	-2.241847618	7E-04	0	HLA-B	protein_coding	major histoc	CHR_HSCHR	31346626	31433637
ENSG0000021306	3.322745425	7E-04	0	Z99572.1	lncRNA	novel transc	1	169486076	169500182
ENSG0000016417	-0.537352006	7E-04	0	ITGA2	protein_coding	integrin sub	5	52989340	53094779
ENSG0000014331	0.310702257	7E-04	0	MRPL24	protein_coding	mitochondr	1	156737303	156741590
ENSG0000020554	1.034328883	7E-04	0	TMEM256	protein_coding	transmembr	17	7402975	7404097
ENSG0000010060	-0.520822895	8E-04	0	ITPK1	protein_coding	inositol-tetr	14	92936914	93116320
ENSG0000012577	-0.317383637	8E-04	0	PANK2	protein_coding	pantothena	20	3888839	3929882
ENSG0000016828	-0.399067796	8E-04	0	THAP11	protein_coding	THAP doma	16	67842320	67844195
ENSG0000014175	-0.841164903	8E-04	0	STAC2	protein_coding	SH3 and cys	17	39210541	39225945
ENSG0000016432	-0.27146772	8E-04	0	RICTOR	protein_coding	RPTOR inde	5	38937920	39074399
ENSG0000017382	1.731381916	8E-04	0	KCNH6	protein_coding	potassium v	17	63523334	63548977
ENSG0000016403	-0.349118074	8E-04	0	SLC9B2	protein_coding	solute carrie	4	103019868	103085829
ENSG0000024911	0.59882846	8E-04	0	HAUS5	protein_coding	HAUS augm	19	35612735	35625355
ENSG0000015245	1.184361345	8E-04	0	ZNF256	protein_coding	zinc finger p	19	57940833	57947706
ENSG0000027338	3.24539282	8E-04	0	AC005005.3	lncRNA	novel transc	22	31082156	31083565
ENSG0000013111	-0.414231271	8E-04	0	ZNF428	protein_coding	zinc finger p	19	43607224	43619629
ENSG0000015840	0.964844683	8E-04	0	H4C8	protein_coding	H4 clustered	6	26277609	26285638
ENSG0000017094	0.410779403	8E-04	0	DNAJC24	protein_coding	DnaJ heat sl	11	31369840	31432835
ENSG0000027633	0.985636008	8E-04	0	AL133243.2	lncRNA	novel transc	2	32521927	32523547
ENSG0000009562	-0.300449671	8E-04	0	TDRD1	protein_coding	tudor doma	10	114179270	114232304
ENSG0000011972	0.620660149	8E-04	0	COQ6	protein_coding	coenzyme C	14	73949926	73963670
ENSG0000010846	0.475512105	8E-04	0	RECQL5	protein_coding	RecQ like he	17	75626845	75667189
ENSG0000011520	-0.192935054	8E-04	0	GTF3C2	protein_coding	general trar	2	27325849	27357034
ENSG0000013041	1.115295985	8E-04	0	STK33	protein_coding	serine/threc	11	8391868	8594289
ENSG0000014164	0.270212773	8E-04	0	SMAD4	protein_coding	SMAD famil	18	51028394	51085045
ENSG0000019709	-5.725133689	8E-04	0	GOLGA6L1	unprocessed_p	golgin A6 fa	Y	25495651	25501958
ENSG0000004734	0.277554733	8E-04	0	FAM214A	protein_coding	family with s	15	52581317	52709817
ENSG0000016780	-1.296824554	8E-04	0	AC011511.1	protein_coding	novel transc	19	10305427	10316009
ENSG0000026137	0.749420778	8E-04	0	PDCD6IPP2	transcribed_un	PDCD6IP ps	15	28789664	28859007
ENSG0000022181	1.272751069	8E-04	0	PPP3CB-AS	lncRNA	PPP3CB ant	10	73495525	73520070
ENSG0000018760	-0.293439626	8E-04	0	ZNF286A	protein_coding	zinc finger p	17	15699577	15720787
ENSG0000010019	-1.276164931	8E-04	0	KDELR3	protein_coding	KDEL endop	22	38468078	38483447
ENSG0000027910	-1.511991696	8E-04	0	AC008537.3	TEC	TEC	19	40840159	40842039
ENSG0000016876	0.365626292	8E-04	0	CNNM3	protein_coding	cyclin and C	2	96816245	96835382
ENSG0000023428	-0.755918732	8E-04	0	ZNF879	protein_coding	zinc finger p	5	179023804	179035064
ENSG0000023728	-0.536511589	8E-04	0	CKMT1B	protein_coding	creatine kin	15	43593054	43604901
ENSG0000010672	0.187374591	8E-04	0	SPIN1	protein_coding	spindlin 1 [S	9	88388430	88478694
ENSG0000010154	0.383539455	8E-04	0	ADNP2	protein_coding	ADNP home	18	80109262	80147523
ENSG0000011840	0.454272689	8E-04	0	ELOVL4	protein_coding	ELOVL fatty	6	79914814	79947553
ENSG0000022723	-1.000611643	8E-04	0	IER3	protein_coding	immediate e	CHR_HSCHR	30733457	30734812
ENSG0000019884	0.482891744	8E-04	0	MT-ND3	protein_coding	mitochondr	MT	10059	10404
ENSG0000025461	-1.614371504	8E-04	0	AC027031.2	lncRNA	novel transc	8	106270144	106272902
ENSG0000006705	-0.281304324	8E-04	0	PFKP	protein_coding	phosphofru	10	3066333	3137718
ENSG0000017642	-3.40522871	8E-04	0	VPS37D	protein_coding	VPS37D sub	7	73667831	73672112
ENSG0000010495	-1.494455987	8E-04	0	TLE6	protein_coding	TLE family n	19	2977538	2995179
ENSG0000027859	-2.843026323	8E-04	0	AL589182.2	processed_pse	translocase	14	19131842	19132890
ENSG0000014683	-0.363114266	8E-04	0	TRIM4	protein_coding	tripartite mc	7	99876958	99919600

ENSG0000011767	0.73381256	8E-04	0	RPS6KA1	protein_coding	ribosomal p		1	26529761	26575030
ENSG0000013282	0.826112795	8E-04	0	PPP1R3D	protein_coding	protein pho		20	59936663	59940305
ENSG0000013373	0.936491852	8E-04	0	LRRCC1	protein_coding	leucine rich		8	85107215	85146080
ENSG0000016668	4.691898798	8E-04	0	TMPRSS5	protein_coding	transmembr		11	113687547	113706373
ENSG0000016699	0.661886649	8E-04	0	CNPY4	protein_coding	canopy FGF		7	100119634	100125508
ENSG0000018298	0.157229335	8E-04	0	CADM1	protein_coding	cell adhesio		11	115169218	115504957
ENSG0000011554	0.198456084	8E-04	0	HSPE1	protein_coding	heat shock p		2	197500140	197503449
ENSG0000012401	-1.712135143	8E-04	0	FAM124B	protein_coding	family with s		2	224378698	224402107
ENSG0000008576	0.303668623	8E-04	0	MTIF2	protein_coding	mitochondr		2	55236595	55269347
ENSG0000027949	1.838967275	8E-04	0	AL928654.4	TEC	novel transc		14	105370418	105372918
ENSG0000005548	-0.259668478	9E-04	0	USP36	protein_coding	ubiquitin sp		17	78787381	78841441
ENSG0000021291	0.643398571	9E-04	0	MAP10	protein_coding	microtubule		1	232804892	232808407
ENSG0000013877	0.353840525	9E-04	0	PPA2	protein_coding	inorganic py		4	105369077	105474067
ENSG0000010157	0.473682449	9E-04	0	LPIN2	protein_coding	lipin 2 [Sour		18	2916994	3013315
ENSG0000016670	0.823437219	9E-04	0	ZCCHC18	protein_coding	zinc finger C	X		104112131	104115842
ENSG0000012383	-0.831106074	9E-04	0	PFKFB2	protein_coding	6-phosphof		1	207034366	207081024
ENSG0000017521	-0.635698035	9E-04	0	ZNF408	protein_coding	zinc finger p		11	46701030	46705912
ENSG0000011049	-0.373469673	9E-04	0	MDK	protein_coding	midkine [So		11	46380756	46383837
ENSG0000026713	2.460450104	9E-04	0	AC005746.2	lncRNA	novel transc		17	61361668	61400243
ENSG0000011715	0.192760657	9E-04	0	RGS4	protein_coding	regulator of		1	163068775	163076802
ENSG0000011559	-0.838700324	9E-04	0	IL1R1	protein_coding	interleukin 1		2	102064544	102179874
ENSG0000022735	1.248289815	9E-04	0	RBM26-AS	lncRNA	RBM26 antis		13	79406293	79427317
ENSG0000014220	0.232353935	9E-04	0	AKT1	protein_coding	AKT serine/t		14	104769349	104795751
ENSG0000016040	0.706707128	9E-04	0	TOR2A	protein_coding	torsin family		9	127731524	127735313
ENSG0000015936	0.449700541	9E-04	0	ATP13A2	protein_coding	ATPase catio		1	16985958	17011928
ENSG0000017147	-0.283123748	9E-04	0	WIPF2	protein_coding	WAS/WASL		17	40219304	40284136
ENSG0000014090	0.349124862	9E-04	0	GCSH	protein_coding	glycine clea		16	81081945	81096395
ENSG0000018508	0.49869701	9E-04	0	RPS27L	protein_coding	ribosomal p		15	63125872	63158021
ENSG0000001352	0.394817122	9E-04	0	ANGEL1	protein_coding	angel homo		14	76786009	76826246
ENSG0000016281	-0.344888111	9E-04	0	BPNT1	protein_coding	3'(2'), 5'-bis		1	220057482	220090462
ENSG0000023809	1.597803474	9E-04	0	ABCA17P	transcribed_un	ATP binding		16	2339150	2426699
ENSG0000013624	0.385934754	9E-04	0	ZDHHC4	protein_coding	zinc finger D		7	6577434	6589374
ENSG0000022854	1.429326664	9E-04	0	CCDC183-A	lncRNA	CCDC183 an		9	136803927	136808848
ENSG0000017086	0.362916792	9E-04	0	LSM3	protein_coding	LSM3 homo		3	14178817	14201122
ENSG0000019771	-0.641444895	9E-04	0	FAM114A1	protein_coding	family with s		4	38867677	38945739
ENSG0000008885	0.425493314	9E-04	0	C20orf194	protein_coding	chromosom		20	3249305	3407625
ENSG0000003821	0.397679361	9E-04	0	PI4K2B	protein_coding	phosphatidy		4	25160663	25279204
ENSG0000004705	0.424387355	9E-04	0	WDR37	protein_coding	WD repeat d		10	1049538	1132384
ENSG0000014456	-0.3061513	9E-04	0	RAB5A	protein_coding	RAB5A, mer		3	19947097	19985175
ENSG0000015888	-2.300522913	9E-04	0	MPZ	protein_coding	myelin prote		1	161304735	161309968
ENSG0000019756	0.54507545	9E-04	0	RAB40C	protein_coding	RAB40C, me		16	589357	629272
ENSG0000027237	1.509412951	9E-04	0	AC009102.2	lncRNA	novel transc		16	56351886	56353524
ENSG0000017151	0.454917264	9E-04	0	LPAR3	protein_coding	lysophospha		1	84811602	84893206
ENSG0000017303	-0.316181973	9E-04	0	RELA	protein_coding	RELA proto-		11	65653597	65663090
ENSG0000015590	0.344195698	9E-04	0	RMND1	protein_coding	required for		6	151404762	151452158
ENSG0000023614	-0.543413138	9E-04	0	ABCF1	protein_coding	ATP binding	CHR_HSCHR		30561217	30587022
ENSG0000017050	-0.335275113	9E-04	0	NUDT9	protein_coding	nudix hydro		4	87422573	87459455
ENSG0000013684	-0.906199162	9E-04	0	ST6GALNA	protein_coding	ST6 N-acety		9	127907886	127917041
ENSG0000016481	-0.311034044	9E-04	0	DNAAF5	protein_coding	dynein axon		7	726699	786475
ENSG0000008726	-0.231251635	9E-04	0	OGFOD1	protein_coding	2-oxoglutar		16	56451521	56479104
ENSG0000027514	0.489241911	9E-04	0	FRG1	protein_coding	FSHD regior	CHR_HSCHR		189944765	189967182
ENSG0000015047	0.79128178	9E-04	0	KIAA1328	protein_coding	KIAA1328 [S		18	36829106	37232172
ENSG0000026326	-0.537613555	9E-04	0	RPS7P1	processed_pse	ribosomal p		17	28467822	28468406
ENSG0000014347	-0.96633606	9E-04	0	KCNH1	protein_coding	potassium v		1	210676823	211134165
ENSG0000016855	0.847424468	9E-04	0	ING2	protein_coding	inhibitor of		4	183505058	183512429
ENSG0000025912	0.970422896	9E-04	0	LINC00648	lncRNA	long interge		14	47764954	47795302
ENSG0000016680	0.967953938	9E-04	0	FAM111A	protein_coding	family with s		11	59142748	59155039
ENSG0000011145	0.252925443	9E-04	0	STX2	protein_coding	syntaxin 2 [S		12	130789600	130839266

ENSG0000016861	0.224989556	1E-03	0	ADAM9	protein_coding	ADAM meta	8	38996869	39105261
ENSG0000026518	2.445963771	1E-03	0	SNORD3B-	snoRNA	small nucleo	17	19061912	19062669
ENSG0000014391	-0.597622691	1E-03	0	CAMKMT	protein_coding	calmodulin-	2	44361947	44772592
ENSG0000017963	-0.262145274	1E-03	0	MAF1	protein_coding	MAF1 homo	8	144104461	144107611
ENSG0000014586	-0.222440132	1E-03	0	RNF145	protein_coding	ring finger p	5	159157409	159210053
ENSG0000019872	-2.828970761	1E-03	0	TEX45	protein_coding	testis expres	19	7492976	7508450
ENSG0000012461	-0.815575718	1E-03	0	MOCS1	protein_coding	molybdenum	6	39899578	39934551
ENSG0000027624	1.295813191	1E-03	0	AL442125.1	lncRNA	novel transc	13	113527260	113530621
ENSG0000006939	-1.496396048	1E-03	0	BCL3	protein_coding	BCL3 transc	19	44747705	44760044
ENSG0000006760	-0.642211948	1E-03	0	PRKCZ	protein_coding	protein kina	1	2050411	2185395
ENSG0000019798	0.468130556	1E-03	0	SNHG12	lncRNA	small nucleo	1	28578538	28583132
ENSG0000011840	-1.245061138	1E-03	0	FILIP1	protein_coding	filamin A int	6	75291859	75493800
ENSG0000016489	-0.492220872	1E-03	0	FMC1	protein_coding	formation o	7	139339457	139346328
ENSG0000011164	0.276529265	1E-03	0	UHRF1BP1L	protein_coding	UHRF1 bind	12	100028455	100142874
ENSG0000013172	0.354266007	1E-03	0	WDR44	protein_coding	WD repeat (	X	118346073	118449961
ENSG0000023305	-2.461726135	1E-03	0	ERVH48-1	lncRNA	endogenous	21	42916803	42925646
ENSG0000023028	1.945006958	1E-03	0	HNRNPA1P	processed_pse	heterogene	1	202911812	202912729
ENSG0000006608	0.180229089	1E-03	0	DIP2B	protein_coding	disco intera	12	50504985	50748657
ENSG0000013524	0.369078533	1E-03	0	RINT1	protein_coding	RAD50 inter	7	105532169	105567677
ENSG0000007004	0.417782149	1E-03	0	PHRF1	protein_coding	PHD and rir	11	576470	612222
ENSG0000014573	0.886217564	1E-03	0	GTF2H2	protein_coding	general tran	5	71032670	71067689
ENSG0000007189	0.225041718	1E-03	0	CPSF1	protein_coding	cleavage an	8	144393229	144409335
ENSG0000011008	-0.285688226	1E-03	0	ST3GAL4	protein_coding	ST3 beta-ga	11	126355640	126440344
ENSG0000018772	-0.731841075	1E-03	0	THSD4	protein_coding	thrombospo	15	71096952	71783383
ENSG0000021869	6.390137246	1E-03	0	H2AC10P	transcribed_un	H2A cluster	6	26272021	26272701
ENSG0000013637	0.498620842	1E-03	0	ABHD17C	protein_coding	abhydrolase	15	80679684	80755621
ENSG0000024020	-3.257627999	0.001	0	SMKR1	protein_coding	small lysine	7	129502531	129512918
ENSG0000011036	0.130143381	0.001	0	DDX6	protein_coding	DEAD-box l	11	118747763	118791164
ENSG0000006869	-0.166921895	0.001	0	LAPTM4A	protein_coding	lysosomal p	2	20032650	20051628
ENSG0000013720	-0.252524345	0.001	0	YIPF3	protein_coding	Yip1 domain	6	43511832	43516985
ENSG0000010333	-0.399922823	0.001	0	PIEZO1	protein_coding	piezo type r	16	88715338	88785220
ENSG0000020452	-1.487937573	0.001	0	HLA-C	protein_coding	major histo	6	31268749	31272130
ENSG0000013688	-0.239582631	0.001	0	ATP6V1G1	protein_coding	ATPase H+	9	114587769	114598879
ENSG0000026381	2.720390327	0.001	0	RDM1P5	transcribed_pr	RDM1 pseu	17	39057019	39113190
ENSG0000023502	-2.66224029	0.001	0	AC068580.3	lncRNA	novel transc	11	1760348	1762486
ENSG0000027280	-0.708687269	0.001	0	AC015712.6	lncRNA	uncharacter	15	100849831	100876836
ENSG0000016797	0.287470129	0.001	0	KCTD5	protein_coding	potassium c	16	2682523	2709030
ENSG0000002369	0.395010779	0.001	0	DERA	protein_coding	deoxyribose	12	15911302	16037381
ENSG0000018809	0.509249728	0.001	0	GPR89B	protein_coding	G protein-c	1	147928393	147993592
ENSG0000021482	0.641092411	0.001	0	DDX12P	unprocessed_p	DEAD/H-bo	12	9418673	9448229
ENSG0000012924	-0.265983675	0.001	0	FXR2	protein_coding	FMR1 autos	17	7591230	7614897
ENSG0000004444	-0.45194602	0.001	0	PHKA2	protein_coding	phosphoryla	X	18892298	18984114
ENSG0000016846	-0.360790173	0.001	0	RAB31	protein_coding	RAB31, mer	18	9708275	9862551
ENSG0000025118	1.392695278	0.001	0	AC079140.3	processed_pse	novel zinc fi	4	268982	269531
ENSG0000012298	-2.035731823	0.001	0	HVCN1	protein_coding	hydrogen vo	12	110627841	110704950
ENSG0000023646	-4.611072043	0.001	0	KCNMA1-A	lncRNA	KCNMA1 ar	10	76888044	76980624
ENSG0000014084	-0.640277141	0.001	0	CPNE2	protein_coding	copine 2 [So	16	57092583	57148369
ENSG0000017422	0.279723717	0.001	0	PIGG	protein_coding	phosphatidy	4	499210	540200
ENSG0000004231	0.590515321	0.001	0	SPATA7	protein_coding	spermatoge	14	88384924	88470350
ENSG0000027260	0.525438277	0.001	0	ZNF595	protein_coding	zinc finger p	4	53286	88208
ENSG0000004309	0.263084258	0.001	0	DCUN1D1	protein_coding	defective in	3	182938074	182985953
ENSG0000011586	-0.20263429	0.001	0	DARS1	protein_coding	aspartyl-tRN	2	135905881	135986100
ENSG0000013215	0.245488094	0.001	0	RAF1	protein_coding	Raf-1 proto	3	12583601	12664226
ENSG0000016573	-0.191868731	0.001	0	RET	protein_coding	ret proto-o	10	43077064	43130351
ENSG0000013600	-0.257470118	0.001	0	ISCU	protein_coding	iron-sulfur c	12	108562582	108569368
ENSG0000025080	-0.778161473	0.001	0	ZBED3-AS1	lncRNA	ZBED3 antis	5	77086688	77166909
ENSG0000023765	-6.368370384	0.001	0	AC073834.1	lncRNA	novel transc	2	177603089	177618572
ENSG0000012171	0.700244335	0.001	0	PILRB	protein_coding	paired immu	7	100352176	100367733

ENSG0000015822	-1.360097292	0.001	0	ESYT3	protein_coding	extended sy	3	138434586	138481686
ENSG0000018681	0.416811193	0.001	0	ZSCAN30	protein_coding	zinc finger a	18	35251058	35290245
ENSG0000012321	0.375722833	0.001	0	CENPK	protein_coding	centromere	5	65517766	65563168
ENSG0000019777	1.242940223	0.001	0	KLHDC1	protein_coding	kelch doma	14	49693105	49753150
ENSG0000012421	0.17166852	0.001	0	STAU1	protein_coding	staufen dou	20	49113339	49188367
ENSG0000013005	-1.247292805	0.001	0	STARD8	protein_coding	StAR relatec	X	68647666	68725842
ENSG0000022992	0.776338565	0.001	0	RHEBP1	processed_pse	RHEB pseuc	10	46634911	46635466
ENSG0000017245	0.92540004	0.001	0	FGGY	protein_coding	FGGY carbo	1	59296638	59810647
ENSG0000010686	-0.727106777	0.001	0	SUSD1	protein_coding	sushi domai	9	112040783	112175297
ENSG0000016387	-0.873998486	0.001	0	DNALI1	protein_coding	dynein axor	1	37556919	37566857
ENSG0000022912	1.637896122	0.001	0	VIM-AS1	lncRNA	VIM antisen	10	17214239	17229985
ENSG0000016243	-0.974933266	0.001	0	AK4	protein_coding	adenylate ki	1	65147549	65232145
ENSG0000010285	0.264930258	0.001	0	MGRN1	protein_coding	mahogunin	16	4616493	4690974
ENSG0000020485	0.548712134	0.001	0	ZBTB48	protein_coding	zinc finger a	1	6579994	6589280
ENSG0000012903	-1.312243794	0.001	0	LOXL1	protein_coding	lysyl oxidase	15	73925989	73952137
ENSG0000012033	-3.535074882	0.001	0	TNFSF18	protein_coding	TNF superfa	1	173039960	173050963
ENSG0000002383	-1.0471121	0.001	0	ABCC2	protein_coding	ATP binding	10	99782640	99852594
ENSG0000010789	0.327388538	0.001	0	ANKRD26	protein_coding	ankyrin repe	10	26973793	27100494
ENSG0000011856	0.34517021	0.001	0	FBXL5	protein_coding	F-box and l	4	15604381	15681679
ENSG0000014439	-0.51308121	0.001	0	CCDC150	protein_coding	coiled-coil c	2	196639554	196763490
ENSG0000023168	-1.037276913	0.001	0	ANKRD11P	processed_pse	ANKRD11 p	X	146619597	146620584
ENSG0000017046	0.500969326	0.001	0	RIOX1	protein_coding	ribosomal o	14	73490933	73493394
ENSG0000014351	-0.222845319	0.001	0	ATP8B2	protein_coding	ATPase pho	1	154325553	154351304
ENSG0000010051	0.201297262	0.001	0	PSMC6	protein_coding	proteasome	14	52707178	52728590
ENSG0000004180	0.21959269	0.001	0	LSG1	protein_coding	large 60S su	3	194640791	194672463
ENSG0000011095	-0.135016427	0.001	0	ATP5F1B	protein_coding	ATP synthas	12	56638175	56645984
ENSG0000018449	1.001159397	0.001	0	NEU1	protein_coding	neuraminida	CHR_HSCHR	31848038	31853285
ENSG0000006252	1.988680349	0.001	0	LTK	protein_coding	leukocyte re	15	41503637	41513887
ENSG0000012607	-0.279985053	0.001	0	AGO3	protein_coding	argonaute R	1	35930718	36072500
ENSG0000027753	-0.881362044	0.001	0	AC007996.	lncRNA	novel transc	18	26542971	26545791
ENSG0000020567	-0.765016875	0.001	0	SMIM11A	protein_coding	small integr	21	34375480	34416961
ENSG0000019702	-0.332184722	0.001	0	ZNF398	protein_coding	zinc finger p	7	149126416	149183042
ENSG0000013765	0.367984325	0.001	0	BUD13	protein_coding	BUD13 hom	11	116748170	116772987
ENSG0000010350	0.284019654	0.001	0	CDIPT	protein_coding	CDP-diacylg	16	29858357	29863414
ENSG0000015634	-0.773205564	0.001	0	CDK20	protein_coding	cyclin deper	9	87966441	87974753
ENSG0000025606	1.104651924	0.001	0	TRAPPC2B	protein_coding	trafficking p	19	57363551	57365405
ENSG0000015669	-0.292977147	0.001	0	UTP14A	protein_coding	UTP14A sm	X	129906121	129929761
ENSG0000016005	1.056342483	0.001	0	TMEM234	protein_coding	transmembr	1	32214472	32222359
ENSG0000010181	0.395372895	0.001	0	CSTF2	protein_coding	cleavage sti	X	100820359	100841520
ENSG0000023211	0.437997728	0.001	0	TMA7	protein_coding	translation r	3	48440257	48444208
ENSG0000010001	0.186292873	0.001	0	SPECC1L	protein_coding	sperm antig	22	24270817	24417739
ENSG0000014484	-0.98046673	0.001	0	ADPRH	protein_coding	ADP-ribosyl	3	119579268	119589945
ENSG0000010555	-0.474266427	0.001	0	MIER2	protein_coding	MIER family	19	301444	344815
ENSG0000014032	0.278992947	0.001	0	BAHD1	protein_coding	bromo adja	15	40439721	40468236
ENSG0000011145	1.14651219	0.001	0	ADGRD1	protein_coding	adhesion G	12	130953907	131141469
ENSG0000011007	-0.613424334	0.001	0	NRXN2	protein_coding	neurexin 2 [	11	64606174	64723197
ENSG0000016959	-0.673867554	0.001	0	DFFB	protein_coding	DNA fragme	1	3857267	3885429
ENSG0000018338	-0.737879202	0.001	0	FHL3	protein_coding	four and a h	1	37996770	38005606
ENSG0000011541	0.393799065	0.001	0	FN1	protein_coding	fibronectin 1	2	215360440	215436073
ENSG0000027691	0.788996785	0.001	0	GTF2H2	protein_coding	general trar	CHR_HSCHR	70376388	70411404
ENSG0000023555	-0.389211293	0.001	0	RPL6P27	transcribed_pr	ribosomal p	18	6462144	6463015
ENSG0000026524	0.207109103	0.001	0	RBM8A	protein_coding	RNA binding	1	145917714	145927678
ENSG0000011153	0.163225067	0.001	0	CAND1	protein_coding	cullin associ	12	67269358	67319953
ENSG0000011120	0.379163608	0.001	0	ITFG2	protein_coding	integrin alpl	12	2812622	2859791
ENSG0000023209	0.863685768	0.001	0	AC012313.	lncRNA	novel transc	19	58404238	58408484
ENSG0000001137	-0.508733813	0.001	0	LARS2	protein_coding	leucyl-tRNA	3	45388561	45554726
ENSG0000016462	-0.750976352	0.001	0	RELL2	protein_coding	RELT like 2 [	5	141636950	141641064
ENSG0000017343	0.319136242	0.001	0	MICOS10	protein_coding	mitochondr	1	19484403	19629821

ENSG0000013972	-0.325690206	0.001	0	VPS37B	protein_coding	VPS37B sub		12	122865330	122896127
ENSG0000011933	0.13565773	0.001	0	SET	protein_coding	SET nuclear		9	128683424	128696400
ENSG0000017420	0.686318052	0.001	0	C12orf66	protein_coding	chromosom		12	64186316	64222296
ENSG0000025951	-2.536710661	0.001	0	LINC01583	lncRNA	long interge		15	82088569	82097694
ENSG0000011739	-0.259891279	0.001	0	SLC2A1	protein_coding	solute carrie		1	42925353	42958893
ENSG0000008130	0.253669587	0.001	0	UBA5	protein_coding	ubiquitin lik		3	132654446	132678498
ENSG0000018088	-1.751883291	0.001	0	CAPS2	protein_coding	calcyphosin		12	75275979	75390928
ENSG0000020513	0.33143192	0.001	0	TRIQK	protein_coding	triple QxxK/		8	92883532	93017673
ENSG0000014767	-0.141734961	0.001	0	EIF3H	protein_coding	eukaryotic t		8	116642130	116766925
ENSG0000026123	0.224926288	0.001	0	BOP1	protein_coding	BOP1 riboso		8	144262045	144291438
ENSG0000010015	0.488092977	0.001	0	DEPDC5	protein_coding	DEP domain		22	31753867	31908033
ENSG0000018643	0.192804824	0.001	0	KPNA4	protein_coding	karyopherin		3	160495007	160565571
ENSG0000015737	1.006918861	0.001	0	DHRS1	protein_coding	dehydrogen		14	24290598	24299780
ENSG0000016565	0.212855888	0.001	0	PDZD8	protein_coding	PDZ domain		10	117277274	117375440
ENSG0000016725	0.202250856	0.001	0	CDK12	protein_coding	cyclin depe		17	39461486	39564907
ENSG0000012064	0.306829443	0.001	0	CCDC77	protein_coding	coiled-coil c		12	389273	442642
ENSG0000022080	-1.023996889	0.001	0	LINC01881	transcribed_un	long interge		2	242088633	242160153
ENSG0000013017	0.259184665	0.001	0	CDC16	protein_coding	cell division		13	114234887	114272723
ENSG0000026988	2.487849905	0.001	0	NA	NA	NA	NA	NA	NA	
ENSG0000016272	0.823331592	0.001	0	KCNJ9	protein_coding	potassium in		1	160081538	160090563
ENSG0000012186	-0.280924435	0.001	0	ZNF639	protein_coding	zinc finger p		3	179323031	179338583
ENSG0000021004	0.952662858	0.001	0	MT-TF	Mt_tRNA	mitochondr	MT		577	647
ENSG0000013282	0.206106438	0.001	0	SERINC3	protein_coding	serine incor		20	44496221	44522085
ENSG0000014653	0.26320616	0.001	0	GNA12	protein_coding	G protein su		7	2728105	2844308
ENSG0000015526	-1.224657626	0.001	0	GOLGA7B	protein_coding	golgin A7 fa		10	97849843	97871580
ENSG0000008747	0.169109018	0.001	0	DNM1L	protein_coding	dynamin 1 l		12	32679200	32745650
ENSG0000025150	1.394826669	0.001	0	CENPS-CO	protein_coding	CENPS-COR		1	10430102	10452153
ENSG0000016616	-0.503336698	0.001	0	TRMT61A	protein_coding	tRNA methy		14	103529196	103537073
ENSG0000027621	4.117621135	0.001	0	AC245014.3	lncRNA	novel transc		1	145281116	145281462
ENSG0000016654	-0.740091654	0.001	0	TK2	protein_coding	thymidine k		16	66508003	66552544
ENSG0000013565	0.21141875	0.001	0	USP15	protein_coding	ubiquitin sp		12	62260338	62417431
ENSG0000013533	0.274860283	0.001	0	ORC3	protein_coding	origin recog		6	87590067	87667453
ENSG0000010097	0.565607009	0.001	0	PLTP	protein_coding	phospholipi		20	45898621	45912155
ENSG0000018254	0.484607854	0.001	0	LIMK2	protein_coding	LIM domain		22	31212239	31280080
ENSG0000014374	-0.327431317	0.001	0	NVL	protein_coding	nuclear VCP		1	224227334	224330189
ENSG0000004731	0.157499654	0.001	0	POLR2B	protein_coding	RNA polyme		4	56977722	57031158
ENSG0000006404	-0.16947098	0.001	0	LIMCH1	protein_coding	LIM and cal		4	41359607	41700044
ENSG0000020533	-1.851727637	0.001	0	ADGRG1	protein_coding	adhesion G		16	57610652	57665580
ENSG0000006560	-0.387669144	0.001	0	PACC1	protein_coding	proton activ		1	212363928	212414901
ENSG0000012575	-0.531571183	0.001	0	VASP	protein_coding	vasodilator s		19	45506579	45526983
ENSG0000022202	6.302263112	0.001	0	HDAC4-AS	lncRNA	HDAC4 anti		2	239401436	239402364
ENSG0000026026	1.582786784	0.001	0	AC124944.2	transcribed_un	programme		3	195913078	195954485
ENSG0000016796	0.4107389	0.001	0	E4F1	protein_coding	E4F transcri		16	2223580	2235742
ENSG0000011267	-0.655486442	0.001	0	DUSP22	protein_coding	dual specific		6	291630	351355
ENSG0000019891	-0.179879092	0.001	0	RPL39	protein_coding	ribosomal p	X		119786504	119791630
ENSG0000025652	0.403999169	0.001	0	POLG2	protein_coding	DNA polym		17	64477785	64497054
ENSG0000012258	-0.82443385	0.001	0	NXPH1	protein_coding	neurexophil		7	8433609	8752961
ENSG0000017036	-0.343994962	0.001	0	SMAD1	protein_coding	SMAD famil		4	145481194	145559176
ENSG0000003169	0.488005926	0.001	0	CENPQ	protein_coding	centromere		6	49463370	49493107
ENSG0000023690	-0.761774885	0.001	0	MIR600HG	lncRNA	MIR600 hos		9	123109494	123115477
ENSG0000014446	-0.334262872	0.001	0	RHBDD1	protein_coding	rhomboid d		2	226835581	226999215
ENSG0000018899	-1.827282762	0.001	0	LRRC66	protein_coding	leucine rich		4	51993702	52017620
ENSG0000018078	0.481277491	0.001	0	ZFP3	protein_coding	ZFP3 zinc fi		17	5078467	5096374
ENSG0000020434	-1.107181959	0.001	0	DXO	protein_coding	decapping e		6	31969810	31972290
ENSG0000017924	0.27743125	0.001	0	LDLRAD3	protein_coding	low density		11	35943981	36232136
ENSG0000025997	-1.68139767	0.001	0	AC093010.2	lncRNA	novel transc		3	114314501	114329714
ENSG0000025881	-1.184952519	0.001	0	RNASE4	protein_coding	ribonucleas		14	20684560	20701216
ENSG0000015562	0.457518442	0.001	0	C9orf85	protein_coding	chromosom		9	71911510	71986054

ENSG0000016991	-0.330893378	0.001	0	OTUD3	protein_coding	OTU deubic	1	19882395	19912945
ENSG0000022865	-6.325379734	0.001	0	NA	NA	NA	NA	NA	NA
ENSG0000014646	-0.215818563	0.001	0	ZMYM4	protein_coding	zinc finger M	1	35268709	35422058
ENSG0000013716	0.454656295	0.001	0	FOXP4	protein_coding	forkhead bo	6	41546426	41602384
ENSG0000024935	-0.360147731	0.001	0	NPM1P27	processed_pse	nucleophos	5	93682838	93683667
ENSG0000010660	-0.326201874	0.001	0	URGCP	protein_coding	upregulator	7	43875894	43926411
ENSG0000016713	0.538609701	0.001	0	ENDOG	protein_coding	endonuclea	9	128818500	128822676
ENSG0000019730	0.374017023	0.001	0	ZNF720	protein_coding	zinc finger p	16	31713229	31794869
ENSG0000006790	0.25697722	0.001	0	ROCK1	protein_coding	Rho associa	18	20946906	21111813
ENSG0000026974	0.833390216	0.001	0	SLC25A53	protein_coding	solute carrie	X	104099214	104157009
ENSG0000022731	0.95688392	0.001	0	MSH5	protein_coding	mutS homo	CHR_HSCHR	31816274	31839440
ENSG0000024983	0.487055508	0.001	0	AC011330.1	unprocessed_p	histidine aci	15	43663654	43684339
ENSG0000027313	-0.550477099	0.001	0	NBPF26	protein_coding	NBPF memb	1	120723949	120842110
ENSG0000019670	-0.251925573	0.001	0	ZNF512B	protein_coding	zinc finger p	20	63956704	63969930
ENSG0000025769	0.95291527	0.001	0	GIHCG	lncRNA	GIHCG inhib	12	57930115	57936345
ENSG0000016674	0.213269931	0.001	0	AP1G1	protein_coding	adaptor rela	16	71729000	71809201
ENSG0000010024	0.486582901	0.001	0	DNAL4	protein_coding	dynein axon	22	38778508	38794198
ENSG0000015177	-1.286409391	0.001	0	SERP2	protein_coding	stress assoc	13	44373665	44397714
ENSG0000016401	-0.675447015	0.001	0	ERMAP	protein_coding	erythroblast	1	42817122	42844991
ENSG0000012339	-0.364195176	0.001	0	ATG101	protein_coding	autophagy r	12	52069246	52077494
ENSG0000016995	-0.507477166	0.001	0	ZNF747	protein_coding	zinc finger p	16	30530367	30535347
ENSG0000010475	0.323383787	0.001	0	KCTD9	protein_coding	potassium c	8	25427847	25458476
ENSG0000010648	-2.170248637	0.001	0	MEST	protein_coding	mesoderm s	7	130486171	130506465
ENSG0000020407	-0.778796095	0.001	0	ARMCX7P	transcribed_un	armadillo re	X	101597510	101598671
ENSG0000010381	-2.315079024	0.001	0	CTSH	protein_coding	cathepsin H	15	78921058	78949574
ENSG0000016094	0.313225039	0.001	0	VPS28	protein_coding	VPS28 subu	8	144423601	144428563
ENSG0000026519	1.438600776	0.001	0	AL359922.2	lncRNA	novel transc	9	21858910	21861926
ENSG0000016760	-1.669311578	0.001	0	NFKBID	protein_coding	NFKB inhibit	19	35887653	35902303
ENSG0000010985	-0.349456125	0.001	0	HTATIP2	protein_coding	HIV-1 Tat in	11	20363685	20383783
ENSG0000016300	0.411772399	0.001	0	NUP35	protein_coding	nucleoporin	2	183117513	183161680
ENSG0000016099	-0.621171435	0.001	0	ALKBH4	protein_coding	alkB homolo	7	102456238	102464863
ENSG0000010913	-0.172552338	0.001	0	TMEM33	protein_coding	transmembr	4	41935129	41960803
ENSG0000012915	0.40183595	0.001	0	KCNC1	protein_coding	potassium v	11	17734774	17856804
ENSG0000016557	-0.319875092	0.001	0	KBTBD6	protein_coding	kelch repeat	13	41127569	41132802
ENSG0000008352	-0.22666166	0.001	0	DIS3	protein_coding	DIS3 homol	13	72752169	72782096
ENSG0000018498	-1.508515871	0.001	0	SORCS2	protein_coding	sortilin relat	4	7192538	7742836
ENSG0000027094	-0.920691103	0.002	0	NA	NA	NA	NA	NA	NA
ENSG0000015563	0.55642218	0.002	0	RBM45	protein_coding	RNA binding	2	178112424	178139011
ENSG0000018381	-0.360925662	0.002	0	LIN9	protein_coding	lin-9 DREAM	1	226231149	226309869
ENSG0000022992	-1.887881641	0.002	0	KIF25-AS1	lncRNA	KIF25 antise	6	167992822	167997110
ENSG0000017839	0.436644007	0.002	0	FAM220A	protein_coding	family with s	7	6329411	6348981
ENSG0000016617	0.604903177	0.002	0	DPCD	protein_coding	deleted in p	10	101570560	101609662
ENSG0000003953	-2.682256324	0.002	0	C6	protein_coding	complemen	5	41142116	41261438
ENSG0000005609	0.167757174	0.002	0	ZFR	protein_coding	zinc finger R	5	32354350	32444740
ENSG0000006472	0.197971254	0.002	0	BTBD1	protein_coding	BTB domain	15	83016423	83067252
ENSG0000019858	2.082897429	0.002	0	AC073343.1	unprocessed_p	zinc finger p	7	6673494	6676366
ENSG0000027308	-6.26459546	0.002	0	AL713999.1	protein_coding	novel protei	1	155169409	155187272
ENSG0000007824	0.275125624	0.002	0	TULP3	protein_coding	TUB like pro	12	2877223	2941138
ENSG0000016470	1.151509283	0.002	0	SLC13A4	protein_coding	solute carrie	7	135681237	135729258
ENSG0000026731	1.174764474	0.002	0	AC027307.2	lncRNA	novel transc	19	1457670	1458580
ENSG0000027817	0.409880263	0.002	0	NSF	protein_coding	N-ethylmale	CHR_HSCHR	46886236	47052838
ENSG0000011525	1.482291154	0.002	0	REEP6	protein_coding	receptor acc	19	1491166	1497927
ENSG0000008708	-0.142217157	0.002	0	FTL	protein_coding	ferritin light	19	48965309	48966879
ENSG0000013038	0.20044259	0.002	0	MLLT1	protein_coding	MLLT1 supe	19	6210381	6279975
ENSG0000016848	0.355502396	0.002	0	BMP1	protein_coding	bone morph	8	22165140	22212326
ENSG0000012033	0.283849633	0.002	0	MRPS14	protein_coding	mitochondr	1	175010789	175023425
ENSG0000018872	-0.239643073	0.002	0	SMIM15	protein_coding	small integr	5	61157704	61162468
ENSG0000021545	-1.893617419	0.002	0	ZNF663P	transcribed_un	zinc finger p	20	46414228	46459276

ENSG0000016988	2.763903218	0.002	0	CALML6	protein_coding	calmodulin		1	1915108	1917296
ENSG0000010365	-0.288748262	0.002	0	CSK	protein_coding	C-terminal		15	74782057	74803198
ENSG0000011569	0.219428574	0.002	0	STK25	protein_coding	serine/threo		2	241492670	241509730
ENSG0000014884	0.168161584	0.002	0	PDCD11	protein_coding	programme		10	103396626	103446294
ENSG0000011294	-0.22218805	0.002	0	TENT4A	protein_coding	terminal nu		5	6713432	6757044
ENSG0000019669	-0.666078116	0.002	0	PDXDC2P-I	lncRNA	nuclear pore		16	69976297	70065948
ENSG0000026900	4.499896697	0.002	0	AC092070.2	transcribed_un	zinc finger p		19	53197111	53214522
ENSG0000027454	-0.636573387	0.002	0	AC243734.1	protein_coding	cholinergic	CHR_HSCHR		32181324	32319966
ENSG0000013444	0.428263382	0.002	0	RELCH	protein_coding	RAB11 bind		18	62187255	62310249
ENSG0000014590	-0.381061352	0.002	0	TNIP1	protein_coding	TNFAIP3 int		5	151029945	151093577
ENSG0000013174	0.362591327	0.002	0	STARD3	protein_coding	StAR relatec		17	39637090	39664201
ENSG0000018113	0.875757703	0.002	0	ZNF707	protein_coding	zinc finger p		8	143684452	143713898
ENSG0000019702	0.582050868	0.002	0	ZNF100	protein_coding	zinc finger p		19	21722771	21767579
ENSG0000009920	-0.539885919	0.002	0	TMED1	protein_coding	transmembr		19	10832067	10836318
ENSG0000027299	-0.826261066	0.002	0	AC012360.3	lncRNA	novel transc		2	105334027	105337475
ENSG0000016752	1.372136476	0.002	0	RSKR	protein_coding	ribosomal p		17	28601827	28614197
ENSG0000016731	0.289496094	0.002	0	ACAA2	protein_coding	acetyl-CoA		18	49782164	49813953
ENSG0000021397	-0.940341468	0.002	0	AC010615.1	unprocessed_p	zinc finger p		19	21382865	21387177
ENSG0000011098	-0.255996167	0.002	0	BCL7A	protein_coding	BAF chroma		12	122019422	122062044
ENSG0000018089	-0.425315629	0.002	0	CUEDC1	protein_coding	CUE domair		17	57861243	57955412
ENSG0000026160	0.347165731	0.002	0	GAN	protein_coding	gigaxonin [S		16	81314944	81390809
ENSG0000012967	-0.861048798	0.002	0	ARHGEF6	protein_coding	Rac/Cdc42 g	X		136665547	136780932
ENSG0000022604	-1.772729467	0.002	0	AC010745.1	lncRNA	novel transc		2	16202430	16204226
ENSG0000025878	2.122206692	0.002	0	AL162171.1	lncRNA	novel transc		14	88551597	88552493
ENSG0000010349	0.381881014	0.002	0	RPGRIP1L	protein_coding	RPGRIP1 like		16	53598153	53703938
ENSG0000011122	0.821956277	0.002	0	PARP11	protein_coding	poly(ADP-ri		12	3791047	3873448
ENSG0000018435	2.586952554	0.002	0	H1-5	protein_coding	H1.5 linker		6	27866792	27867588
ENSG0000000796	-0.403083829	0.002	0	E2F2	protein_coding	E2F transcri		1	23506438	23531233
ENSG0000017797	0.366659964	0.002	0	IMP3	protein_coding	IMP U3 sma		15	75639085	75648706
ENSG0000010615	0.160668614	0.002	0	CHCHD2	protein_coding	coiled-coil-		7	56101573	56106476
ENSG0000024669	0.857264062	0.002	0	RASSF8-AS	lncRNA	RASSF8 anti		12	25936683	25959765
ENSG0000027690	6.248459902	0.002	0	H2AC16	protein_coding	H2A cluster		6	27865317	27865798
ENSG0000015178	-1.291766299	0.002	0	ZNF385D	protein_coding	zinc finger p		3	21412218	22373321
ENSG0000027802	1.817975421	0.002	0	RDM1	protein_coding	RAD52 mot		17	35918066	35930773
ENSG0000018568	0.403520192	0.002	0	EP400P1	transcribed_un	EP400 pseu		12	132084283	132131639
ENSG0000011816	-2.062417523	0.002	0	SLC8A2	protein_coding	solute carrie		19	47428017	47471893
ENSG0000019679	-0.63078335	0.002	0	ZNF239	protein_coding	zinc finger p		10	43556344	43574618
ENSG0000010668	-2.670491455	0.002	0	SPATA6L	protein_coding	spermatoge		9	4553386	4666674
ENSG0000024421	2.616023817	0.002	0	TMEM225B	protein_coding	transmembr		7	99598267	99611045
ENSG0000026457	1.371742918	0.002	0	LINC00526	lncRNA	long interge		18	5236724	5238598
ENSG0000023797	0.473125534	0.002	0	MTCO1P12	unprocessed_p	MT-CO1 ps		1	631074	632616
ENSG0000000619	-0.306034051	0.002	0	ZNF263	protein_coding	zinc finger p		16	3263800	3301401
ENSG0000012420	-0.355747902	0.002	0	ZNFX1	protein_coding	zinc finger N		20	49237946	49278426
ENSG0000018562	0.731013668	0.002	0	LMLN	protein_coding	leishmanoly		3	197960200	198043720
ENSG0000017245	-2.067986529	0.002	0	IL17D	protein_coding	interleukin 1		13	20702127	20723098
ENSG0000011774	0.295873401	0.002	0	RPA2	protein_coding	replication p		1	27891524	27914746
ENSG0000019778	0.43063103	0.002	0	ZNF780A	protein_coding	zinc finger p		19	40069152	40090938
ENSG0000018374	0.231866031	0.002	0	CBX6	protein_coding	chromobox		22	38861422	38872249
ENSG0000016711	-0.213124139	0.002	0	GOLGA2	protein_coding	golgin A2 [S		9	128255829	128275995
ENSG0000015161	0.727098645	0.002	0	MMAA	protein_coding	metabolism		4	145599042	145660033
ENSG0000025459	-0.559844771	0.002	0.01	CSNK2A3	protein_coding	casein kinas		11	11351942	11353250
ENSG0000015863	0.24710476	0.002	0.01	EMSY	protein_coding	EMSY transc		11	76444923	76553025
ENSG0000014839	0.177812041	0.002	0.01	SEC16A	protein_coding	SEC16 hom		9	136440096	136483759
ENSG0000006588	0.265926025	0.002	0.01	TBC1D1	protein_coding	TBC1 doma		4	37891084	38139175
ENSG0000015525	-0.230503852	0.002	0.01	PI4K2A	protein_coding	phosphatidy		10	97640686	97676434
ENSG0000014463	0.287041289	0.002	0.01	DYNC1LI1	protein_coding	dynein cyto		3	32525974	32570858
ENSG0000014547	-0.541890656	0.002	0.01	CYP4V2	protein_coding	cytochrome		4	186191567	186213463
ENSG0000016352	-0.823814292	0.002	0.01	CHCHD4	protein_coding	coiled-coil-		3	14112077	14124870

ENSG0000018753	-0.400861634	0.002	0.01	IFT140	protein_coding	intraflagella	16	1510427	1612072
ENSG0000013166	-0.752357702	0.002	0.01	NINJ1	protein_coding	ninjurin 1 [S	9	93121496	93134251
ENSG0000016411	0.190497694	0.002	0.01	ANXA5	protein_coding	annexin A5	4	121667946	121696995
ENSG0000021952	-2.478991312	0.002	0.01	AP000580.1	processed_pse	ribosomal p	11	77813319	77813676
ENSG0000006838	0.477148618	0.002	0.01	INPP5A	protein_coding	inositol poly	10	132537787	132783480
ENSG0000023687	0.661057695	0.002	0.01	AGPAT1	protein_coding	1-acylglycer	CHR_HSCHR	32235221	32245105
ENSG0000023687	2.666842366	0.002	0.01	LINC00106	lncRNA	long interge	X	1396427	1399402
ENSG0000024157	-6.250370956	0.002	0.01	PAQR9-AS1	lncRNA	PAQR9 antis	3	142960650	143001559
ENSG0000010124	0.4300844	0.002	0.01	ARFRP1	protein_coding	ADP ribosyla	20	63698642	63708025
ENSG0000013823	0.357088701	0.002	0.01	DBR1	protein_coding	debranching	3	138160988	138174921
ENSG0000020426	-4.009222397	0.002	0.01	PSMB8	protein_coding	proteasome	6	32840717	32844679
ENSG0000015101	-0.613987198	0.002	0.01	NOCT	protein_coding	nocturnin [S	4	139015781	139045939
ENSG0000025400	-0.308956252	0.002	0.01	ZNF260	protein_coding	zinc finger p	19	36510687	36528271
ENSG0000013705	-0.236999351	0.002	0.01	PLAA	protein_coding	phospholipa	9	26903372	26947242
ENSG0000023382	0.598019914	0.002	0.01	H2BC15	protein_coding	H2B clustere	6	27837760	27865798
ENSG0000023512	-0.985193194	0.002	0.01	NFKBIL1	protein_coding	NFKB inhibit	CHR_HSCHR	31623212	31635161
ENSG0000027338	-4.10100436	0.002	0.01	AC005291.2	lncRNA	novel transc	17	10291820	10317926
ENSG0000014303	0.27227999	0.002	0.01	MTF2	protein_coding	metal respo	1	93079235	93139079
ENSG0000004762	0.339523274	0.002	0.01	C12orf4	protein_coding	chromosom	12	4487735	4538508
ENSG0000018627	1.0960349	0.002	0.01	ZNF17	protein_coding	zinc finger p	19	57411163	57421939
ENSG0000011230	-0.402164733	0.002	0.01	SMAP1	protein_coding	small ArfGA	6	70667776	70862011
ENSG0000018651	-2.295816998	0.002	0.01	CLCNKA	protein_coding	chloride vol	1	16018875	16034050
ENSG0000012574	-0.517694022	0.002	0.01	OPA3	protein_coding	outer mitoc	19	45527427	45602212
ENSG0000015653	0.172584188	0.002	0.01	PHF6	protein_coding	PHD finger	X	134373312	134428791
ENSG0000023334	1.201417237	0.002	0.01	MSH5	protein_coding	mutS homo	CHR_HSCHR	31823611	31830163
ENSG0000012569	-0.110712475	0.002	0.01	RPL23	protein_coding	ribosomal p	17	38847860	38853764
ENSG0000012127	0.286423327	0.002	0.01	TENT4B	protein_coding	terminal nuc	16	50152911	50235310
ENSG0000014657	-0.258466651	0.002	0.01	CCZ1B	protein_coding	CCZ1 homo	7	6794134	6826770
ENSG0000027476	-0.636795156	0.002	0.01	CISD3	protein_coding	CDGSH iron	CHR_HSCHR	38531022	38535831
ENSG0000020574	1.738071316	0.002	0.01	AL359878.1	lncRNA	uncharacter	10	971146	988341
ENSG0000013769	-2.508658198	0.002	0.01	CFAP300	protein_coding	cilia and flag	11	102047437	102084554
ENSG0000019843	0.961127832	0.002	0.01	NRARP	protein_coding	NOTCH reg	9	137299631	137302271
ENSG0000018928	-1.727155571	0.002	0.01	FHIT	protein_coding	fragile histic	3	59747277	61251459
ENSG0000014054	-0.486519226	0.002	0.01	ZNF710	protein_coding	zinc finger p	15	90001324	90082206
ENSG0000014840	-0.375111354	0.002	0.01	CACNA1B	protein_coding	calcium volt	9	137877782	138124624
ENSG0000012882	0.284076701	0.002	0.01	EIF2AK4	protein_coding	eukaryotic t	15	39934115	40035591
ENSG0000010850	-0.545579965	0.002	0.01	CAMTA2	protein_coding	calmodulin	17	4967992	4987675
ENSG0000021336	0.536632069	0.002	0.01	GSTM2	protein_coding	glutathione	1	109668022	109709551
ENSG0000004976	-0.575621858	0.002	0.01	PPP1R3F	protein_coding	protein pho	X	49269793	49301461
ENSG0000026127	-1.275786414	0.002	0.01	AC012181.2	lncRNA	novel transc	16	56940278	56941342
ENSG0000021689	1.230997703	0.002	0.01	AC009403.1	lncRNA	novel transc	7	155611231	155645205
ENSG0000022618	-2.450933463	0.002	0.01	AC010536.1	lncRNA	novel transc	16	87693537	87696147
ENSG0000027435	-3.910293414	0.002	0.01	NDUFA3	protein_coding	NADH:ubiq	CHR_HSCHR	54102906	54107026
ENSG0000014571	0.269437207	0.002	0.01	RASA1	protein_coding	RAS p21 pro	5	87267883	87391931
ENSG0000021332	-0.544143181	0.002	0.01	RPS7P11	processed_pse	ribosomal p	17	46721582	46722167
ENSG0000021649	-1.12775037	0.002	0.01	IFI30	protein_coding	IFI30 lysoso	19	18173162	18178117
ENSG0000011091	-0.279754435	0.002	0.01	SLC11A2	protein_coding	solute carrie	12	50979401	51028566
ENSG0000026151	-6.215878176	0.002	0.01	LINC01976	lncRNA	long interge	17	43938363	43938959
ENSG0000016337	-0.677205261	0.002	0.01	KBTBD8	protein_coding	kelch repeat	3	66998307	67011210
ENSG0000026000	-2.187321061	0.002	0.01	AC107871.1	protein_coding	novel protei	15	68184032	68229718
ENSG0000011530	0.113788788	0.002	0.01	SPTBN1	protein_coding	spectrin bet	2	54456317	54671446
ENSG0000010136	0.367026967	0.002	0.01	MANBAL	protein_coding	mannosidas	20	37289638	37317260
ENSG0000022493	1.444543673	0.002	0.01	AL391684.1	lncRNA	novel transc	10	99430919	99463259
ENSG0000019880	-0.216629966	0.002	0.01	PNP	protein_coding	purine nucle	14	20468954	20477089
ENSG0000011218	-2.408774855	0.002	0.01	RBM24	protein_coding	RNA binding	6	17281361	17293871
ENSG0000021764	6.199561564	0.002	0.01	H2BC16P	processed_pse	H2B clustere	6	27864062	27864401
ENSG0000022915	0.64157248	0.002	0.01	ANKRD10-	lncRNA	ANKRD10 ir	13	110894639	110899172
ENSG0000008518	-0.525042557	0.002	0.01	BCORL1	protein_coding	BCL6 corepi	X	129981107	130058083

ENSG0000013325	-0.671507716	0.002	0.01	ZNF414	protein_coding	zinc finger p	19	8509678	8514167
ENSG0000012420	0.27355887	0.002	0.01	RAB22A	protein_coding	RAB22A, me	20	58309715	58367507
ENSG0000015059	0.177481984	0.002	0.01	PDCD4	protein_coding	programme	10	110871795	110900006
ENSG0000012859	0.378973171	0.002	0.01	FLNC	protein_coding	filamin C [So	7	128830377	128859274
ENSG0000018219	0.263441012	0.002	0.01	ARL6IP4	protein_coding	ADP ribosyla	12	122980060	122982913
ENSG0000016881	-0.326225514	0.002	0.01	STX18	protein_coding	syntaxin 18	4	4415742	4542346
ENSG0000016937	-0.72929084	0.002	0.01	CRADD	protein_coding	CASP2 and	12	93677375	93894840
ENSG0000027378	1.643743519	0.002	0.01	AL136040.1	lncRNA	novel transc	14	81221218	81222460
ENSG0000014709	-0.42667229	0.002	0.01	HDAC8	protein_coding	histone dea	X	72329516	72573101
ENSG0000022857	0.502322217	0.002	0.01	MDC1	protein_coding	mediator of	CHR_HSCHR	30744466	30762547
ENSG0000024719	-2.188904793	0.002	0.01	AC011346.1	lncRNA	novel transc	5	148088125	148383907
ENSG0000008700	0.576518778	0.002	0.01	ACOX3	protein_coding	acyl-CoA ox	4	8366282	8440723
ENSG0000018057	-0.960441975	0.002	0.01	EIF2S3B	protein_coding	eukaryotic t	12	10505602	10523135
ENSG0000023099	-0.388658922	0.002	0.01	PPP1R10	protein_coding	protein pho	CHR_HSCHR	30590243	30608455
ENSG0000022878	-0.798197752	0.002	0.01	MRPL45P2	transcribed_un	mitochondr	17	47450568	47492492
ENSG0000014046	-2.21535912	0.002	0.01	CYP1A1	protein_coding	cytochrome	15	74719542	74725536
ENSG0000024957	6.188175769	0.002	0.01	AC034231.1	lncRNA	novel transc	5	33424025	33440619
ENSG0000013184	-1.184493597	0.002	0.01	ZSCAN5A	protein_coding	zinc finger a	19	56219670	56368383
ENSG0000020496	0.665730643	0.002	0.01	PCDHA8	protein_coding	protocadhe	5	140841187	141012347
ENSG0000011761	0.229718338	0.002	0.01	RSRP1	protein_coding	arginine and	1	25242249	25338213
ENSG0000016365	-0.203920836	0.002	0.01	GMPS	protein_coding	guanine mo	3	155870650	155944020
ENSG0000027213	2.369749936	0.002	0.01	LINC01607	lncRNA	long interge	8	79768110	79802842
ENSG0000005975	0.290946636	0.002	0.01	CDK17	protein_coding	cyclin deper	12	96278261	96400480
ENSG0000027576	0.743302874	0.002	0.01	AC092747.4	lncRNA	novel transc	12	27037100	27038960
ENSG0000026078	-3.089261288	0.002	0.01	ARHGAP23	transcribed_un	Rho GTPase	16	33907419	33937167
ENSG0000010028	0.409523918	0.002	0.01	TOM1	protein_coding	target of my	22	35299275	35347992
ENSG0000011190	-5.443037556	0.002	0.01	TPD52L1	protein_coding	TPD52 like 1	6	125119049	125264407
ENSG0000023978	0.483923784	0.002	0.01	MRPS17	protein_coding	mitochondr	7	55951877	55956500
ENSG0000010287	0.935264455	0.002	0.01	TRADD	protein_coding	TNFRSF1A a	16	67154185	67159909
ENSG0000005074	0.252762448	0.002	0.01	MAPK9	protein_coding	mitogen-ac	5	180233143	180292099
ENSG0000022703	-1.369920599	0.002	0.01	LINC00511	lncRNA	long interge	17	72290091	72640472
ENSG0000019624	0.42471579	0.002	0.01	ZNF107	protein_coding	zinc finger p	7	64666083	64711582
ENSG0000007513	0.493079461	0.002	0.01	TIPIN	protein_coding	TIMELESS in	15	66336191	66386746
ENSG0000017995	-0.3374045	0.002	0.01	DCTPP1	protein_coding	dCTP pyrop	16	30423615	30430030
ENSG0000027130	0.325842923	0.002	0.01	SRXN1	protein_coding	sulfiredoxin	20	646615	653200
ENSG0000017253	0.176063217	0.002	0.01	PPP1CA	protein_coding	protein pho	11	67398183	67421183
ENSG0000019695	0.208540243	0.002	0.01	SLC39A10	protein_coding	solute carrie	2	195575977	195737702
ENSG0000021314	-0.937751234	0.002	0.01	CRIP1	protein_coding	cysteine rich	14	105486317	105488947
ENSG0000010100	0.3245266	0.002	0.01	GINS1	protein_coding	GINS compl	20	25407673	25452628
ENSG0000016371	0.149478103	0.002	0.01	U2SURP	protein_coding	U2 snRNP a	3	142964497	143060725
ENSG0000011536	0.185121106	0.002	0.01	LANCL1	protein_coding	LanC like 1	2	210431249	210477652
ENSG0000014474	0.214086687	0.002	0.01	UBA3	protein_coding	ubiquitin like	3	69054730	69080408
ENSG0000013749	0.581094579	0.002	0.01	IL18BP	protein_coding	interleukin 1	11	71998613	72005715
ENSG0000026726	3.234511967	0.002	0.01	NA	NA	NA	NA	NA	NA
ENSG0000019663	0.56487811	0.002	0.01	SDHAF3	protein_coding	succinate de	7	97117698	97181763
ENSG0000016666	-0.910857166	0.002	0.01	CHRFAM7A	protein_coding	CHRNA7 (ex	15	30360566	30393849
ENSG0000010726	-0.289702439	0.002	0.01	BAG1	protein_coding	BAG cochap	9	33247820	33264720
ENSG0000015554	-0.209703761	0.002	0.01	MIER3	protein_coding	MIER family	5	56919602	56971675
ENSG0000017052	-0.234872082	0.002	0.01	ELOVL6	protein_coding	ELOVL fatty	4	110045846	110199199
ENSG0000027873	-0.320672817	0.002	0.01	AC005332.6	lncRNA	novel transc	17	68126666	68129586
ENSG0000011970	0.394240227	0.002	0.01	SLIRP	protein_coding	SRA stem-lo	14	77708071	77761104
ENSG0000007076	0.304965747	0.002	0.01	CFAP20	protein_coding	cilia and flag	16	58113592	58129381
ENSG0000011297	0.180193365	0.002	0.01	HMGCS1	protein_coding	3-hydroxy-3	5	43287470	43313512
ENSG0000017023	-0.268036783	0.002	0.01	PWWP2A	protein_coding	PWWP dom	5	160061801	160119450
ENSG0000013085	-0.473989093	0.002	0.01	ZNF236	protein_coding	zinc finger p	18	76822607	76970727
ENSG0000016396	0.166512238	0.002	0.01	UBXN7	protein_coding	UBX domain	3	196347662	196432430
ENSG0000020614	0.368269145	0.002	0.01	HERC2P9	transcribed_un	hect domain	15	28589492	28685264
ENSG0000012086	0.473616016	0.002	0.01	WASHC3	protein_coding	WASH comp	12	102012840	102062149

ENSG0000017446	-0.963644182	0.002	0.01	CNTNAP2	protein_coding	contactin as	7	146116002	148420998
ENSG0000021418	0.899497632	0.002	0.01	ZNF788P	transcribed_un	zinc finger f	19	12092263	12137235
ENSG0000015650	-0.266405458	0.002	0.01	SUPV3L1	protein_coding	Suv3 like RN	10	69180234	69209099
ENSG0000013071	-0.434881003	0.002	0.01	POMT1	protein_coding	protein O-m	9	131502902	131523806
ENSG0000010569	-1.066928274	0.002	0.01	TMEM59L	protein_coding	transmembr	19	18607430	18621039
ENSG0000017767	0.647317534	0.002	0.01	CD163L1	protein_coding	CD163 mole	12	7346685	7479897
ENSG0000014957	0.489949721	0.002	0.01	SIDT2	protein_coding	SID1 transm	11	117178736	117197445
ENSG0000019658	0.350445981	0.002	0.01	MRTFA	protein_coding	myocardin r	22	40410281	40636719
ENSG0000018436	-0.803521535	0.002	0.01	MAP7D2	protein_coding	MAP7 doma	X	20006713	20116907
ENSG0000013410	0.28112162	0.002	0.01	ARL8B	protein_coding	ADP ribosyl	3	5122249	5180911
ENSG0000026259	-0.374994687	0.002	0.01	DENND11	protein_coding	DENN doma	CHR_HSCHR	141656728	141702156
ENSG0000012608	-0.420220473	0.002	0.01	UROD	protein_coding	uroporphyri	1	45010950	45015575
ENSG0000009548	0.371071253	0.002	0.01	CWF19L1	protein_coding	CWF19 like	10	100232298	100267680
ENSG0000025977	2.417042398	0.002	0.01	AL138976.2	lncRNA	novel transc	14	103331674	103332367
ENSG0000013785	-0.837941867	0.002	0.01	DUOX1	protein_coding	dual oxidase	15	45129933	45165576
ENSG0000014265	-0.521624751	0.002	0.01	PEX14	protein_coding	peroxisoma	1	10472288	10630758
ENSG0000026858	2.455053811	0.002	0.01	AC073389.1	lncRNA	novel transc	10	73625996	73626790
ENSG0000018164	1.689187442	0.002	0.01	PHLDA2	protein_coding	pleckstrin ho	11	2928273	2929420
ENSG0000015536	0.254255908	0.002	0.01	DBI	protein_coding	diazepam b	2	119366921	119372560
ENSG0000010280	0.216846488	0.002	0.01	TSC22D1	protein_coding	TSC22 dom	13	44432143	44577147
ENSG0000016234	0.589451787	0.002	0.01	TPCN2	protein_coding	two pore se	11	69048932	69136316
ENSG0000017860	-0.497226893	0.002	0.01	GTPBP6	protein_coding	GTP binding	X	304529	318819
ENSG0000010624	-0.232961162	0.002	0.01	BUD31	protein_coding	BUD31 hom	7	99408641	99419616
ENSG0000014788	1.636686435	0.002	0.01	CDKN2B	protein_coding	cyclin deper	9	22002903	22009305
ENSG0000027453	1.679520467	0.002	0.01	PHLDA2	protein_coding	pleckstrin ho	CHR_HSCHR	2928201	2929383
ENSG0000011175	0.34296544	0.002	0.01	PHC1	protein_coding	polyhomeot	12	8913896	8941467
ENSG0000013950	-0.253841293	0.002	0.01	MTMR6	protein_coding	myotubulari	13	25246201	25288009
ENSG0000013838	0.240882998	0.002	0.01	NAB1	protein_coding	NGFI-A bin	2	190646746	190692766
ENSG0000017417	0.540168877	0.002	0.01	CTU2	protein_coding	cytosolic thi	16	88706483	88715396
ENSG0000022867	-1.523137961	0.002	0.01	PROB1	protein_coding	proline rich	5	139390592	139395104
ENSG0000025147	0.527190116	0.002	0.01	RPL32P3	transcribed_un	ribosomal p	3	129382922	129399655
ENSG0000013689	-0.401679382	0.002	0.01	GARNL3	protein_coding	GTPase activ	9	127224265	127393660
ENSG0000022182	1.24632304	0.002	0.01	C6orf226	protein_coding	chromosom	6	42890265	42890821
ENSG0000017296	-0.777888275	0.002	0.01	MIR4435-2	lncRNA	MIR4435-2	2	111006015	111523376
ENSG0000014355	0.382001525	0.002	0.01	SNAPIN	protein_coding	SNAP assoc	1	153658703	153661852
ENSG0000017160	-0.34004893	0.002	0.01	CXXC5	protein_coding	CXXC finger	5	139647299	139683882
ENSG0000012918	-0.283625605	0.002	0.01	DCTD	protein_coding	dCMP deam	4	182890060	182917936
ENSG0000022456	-2.962387832	0.002	0.01	LINC01886	lncRNA	long interge	2	107529292	107556499
ENSG0000010115	0.183827403	0.002	0.01	TPD52L2	protein_coding	TPD52 like 2	20	63865228	63891545
ENSG0000013992	-0.230393181	0.002	0.01	TMX1	protein_coding	thioredoxin	14	51240162	51257655
ENSG0000009066	-1.843454721	0.002	0.01	CERS4	protein_coding	ceramide sy	19	8206736	8262421
ENSG0000015616	0.234994585	0.002	0.01	DPY19L4	protein_coding	dpy-19 like	8	94719703	94793836
ENSG0000027331	1.140540234	0.002	0.01	AC005229.4	lncRNA	novel transc	7	148696467	148698664
ENSG0000021417	0.620385898	0.002	0.01	PLEKHM1P1	transcribed_un	pleckstrin ho	17	64779259	64837154
ENSG0000013942	-0.370485597	0.002	0.01	MMAB	protein_coding	metabolism	12	109553715	109573580
ENSG0000026116	-1.096528122	0.002	0.01	AC107027.3	lncRNA	novel transc	3	131455126	131458598
ENSG0000015169	-0.292688204	0.002	0.01	MFSD6	protein_coding	major facilit	2	190408355	190509205
ENSG0000015478	-0.461069369	0.002	0.01	CCDC174	protein_coding	coiled-coil c	3	14651746	14672659
ENSG0000026492	1.436983097	0.002	0.01	AC018521.5	lncRNA	novel transc	17	47891229	47895812
ENSG0000027690	2.087388979	0.002	0.01	AC023157.2	lncRNA	novel transc	12	31729117	31731204
ENSG0000025463	0.384804337	0.002	0.01	WAC-AS1	lncRNA	WAC antise	10	28522652	28533066
ENSG0000026026	0.644372478	0.002	0.01	AC026471.1	lncRNA	novel transc	16	31456711	31459736
ENSG0000011231	0.247003032	0.002	0.01	EYA4	protein_coding	EYA transcri	6	133240598	133532128
ENSG0000013573	-0.980221391	0.002	0.01	CCDC102A	protein_coding	coiled-coil c	16	57512181	57536571
ENSG0000019696	-0.515928489	0.002	0.01	FUT11	protein_coding	fucosyltrans	10	73772276	73780251
ENSG0000022331	-6.152780583	0.002	0.01	RNA5SP11	rRNA_pseudog	RNA, 5S ribo	2	164895677	164895777
ENSG0000014611	0.482673541	0.002	0.01	PPP1R18	protein_coding	protein pho	6	30676389	30687895
ENSG0000015240	0.330631489	0.002	0.01	CWF19L2	protein_coding	CWF19 like	11	107326345	107457844

ENSG0000015679	0.495943774	0.002	0.01	NTAQ1	protein_coding	N-terminal	8	123416726	123470028
ENSG0000022707	-6.162932707	0.002	0.01	LINC02094	lncRNA	long interge	17	19419035	19424357
ENSG0000013873	-0.743854922	0.002	0.01	PRDM5	protein_coding	PR/SET dom	4	120684919	120922870
ENSG0000013420	0.31609382	0.002	0.01	GSTM3	protein_coding	glutathione	1	109733932	109741038
ENSG0000012345	-1.431816389	0.002	0.01	SARDH	protein_coding	sarcosine de	9	133663560	133739955
ENSG0000007655	0.524162242	0.002	0.01	ACACB	protein_coding	acetyl-CoA	12	109116595	109268226
ENSG0000013279	0.258465585	0.002	0.01	CTNNBL1	protein_coding	catenin beta	20	37693955	37872129
ENSG0000018062	0.508618904	0.002	0.01	ZNF594	protein_coding	zinc finger p	17	5179535	5191868
ENSG0000024462	1.065220628	0.002	0.01	TPTEP2	transcribed_un	TPTE pseud	22	38335762	38398929
ENSG0000026170	4.467786272	0.002	0.01	AC092134.1	lncRNA	novel transc	16	77741468	77743000
ENSG0000027784	-1.244201499	0.002	0.01	MEGF11	protein_coding	multiple EG	CHR_HSCHR	65909030	66265884
ENSG0000016264	0.40713076	0.002	0.01	C1orf52	protein_coding	chromosom	1	85249953	85259672
ENSG0000023565	0.999236627	0.002	0.01	ATAT1	protein_coding	alpha tubuli	CHR_HSCHR	30671478	30691459
ENSG0000016941	0.219571111	0.002	0.01	PTPN9	protein_coding	protein tyro	15	75463251	75579315
ENSG0000016108	-1.315106277	0.002	0.01	CELF5	protein_coding	CUGBP Elav	19	3224661	3297076
ENSG0000013886	0.332324102	0.002	0.01	GUCD1	protein_coding	guanylyl cyc	22	24540423	24555935
ENSG0000027536	-0.725413019	0.002	0.01	MMP11	protein_coding	matrix meta	CHR_HSCHR	23768226	23784316
ENSG0000017869	-0.25091018	0.002	0.01	SUZ12	protein_coding	SUZ12 poly	17	31937007	32001038
ENSG0000024249	-0.496450334	0.002	0.01	ARPIN	protein_coding	actin relatec	15	89895006	89912952
ENSG0000012860	0.21847091	0.002	0.01	NDUFA5	protein_coding	NADH:ubiqu	7	123536997	123557904
ENSG0000014718	0.164546633	0.002	0.01	ZNF711	protein_coding	zinc finger p	X	85243991	85273362
ENSG0000023022	2.153767974	0.002	0.01	PHBP9	processed_pse	prohibitin p	10	100248271	100249095
ENSG0000010519	-0.327063306	0.002	0.01	TIMM50	protein_coding	translocase	19	39480412	39493785
ENSG0000011836	-0.205776779	0.002	0.01	SPCS2	protein_coding	signal pepti	11	74949247	74979033
ENSG0000014129	-0.775908673	0.002	0.01	SCRN2	protein_coding	secernin 2 [	17	47837692	47841289
ENSG0000018091	0.316725248	0.002	0.01	CMTR2	protein_coding	cap methylt	16	71281389	71289715
ENSG0000025898	-4.821617887	0.002	0.01	UBE2F-SCL	protein_coding	UBE2F-SCL	2	237967014	238099412
ENSG0000014457	-0.344579687	0.002	0.01	CTDSP1	protein_coding	CTD small p	2	218398256	218405941
ENSG0000019816	0.182540343	0.002	0.01	MAN1A2	protein_coding	mannosidas	1	117367449	117528872
ENSG0000010031	0.553989981	0.002	0.01	ZMAT5	protein_coding	zinc finger r	22	29730956	29767011
ENSG0000014026	-0.245005237	0.002	0.01	ZSCAN29	protein_coding	zinc finger a	15	43358172	43371025
ENSG0000014606	0.329963668	0.002	0.01	FAM193B	protein_coding	family with s	5	177519789	177554563
ENSG0000013238	-0.323649025	0.002	0.01	MYBBP1A	protein_coding	MYB binding	17	4538897	4555386
ENSG0000014583	-0.165205519	0.002	0.01	DDX46	protein_coding	DEAD-box l	5	134758771	134855133
ENSG0000026182	-0.415679853	0.002	0.01	LINC00662	lncRNA	long interge	19	27681072	27794005
ENSG0000016511	-0.116805864	0.002	0.01	HNRNPK	protein_coding	heterogene	9	83968083	83980616
ENSG0000013040	0.207621968	0.002	0.01	ACTN4	protein_coding	actinin alph	19	38647649	38731589
ENSG0000006549	-0.473374654	0.002	0.01	TBC1D22B	protein_coding	TBC1 doma	6	37257772	37332970
ENSG0000014101	0.538677181	0.002	0.01	GALNS	protein_coding	galactosami	16	88813734	88856970
ENSG0000014834	-1.880235483	0.002	0.01	PTGES	protein_coding	prostagland	9	129738331	129753042
ENSG0000021828	0.229551776	0.002	0.01	MORF4L1P	processed_pse	mortality fac	1	220253570	220254536
ENSG0000027816	-0.881099424	0.002	0.01	DUSP8	protein_coding	dual specific	CHR_HSCHR	1554044	1571920
ENSG0000027009	-2.170918767	0.002	0.01	NA	NA	NA	NA	NA	NA
ENSG0000026215	2.642812181	0.002	0.01	LINC02175	lncRNA	long interge	16	25066937	25088618
ENSG0000016021	-0.383439808	0.002	0.01	RRP1	protein_coding	ribosomal R	21	43789513	43805293
ENSG0000015404	0.954056859	0.002	0.01	CABYR	protein_coding	calcium binc	18	24138956	24161603
ENSG0000015430	-0.170045538	0.002	0.01	MIA3	protein_coding	MIA SH3 dc	1	222618097	222668007
ENSG0000018712	1.330010488	0.002	0.01	LYPD6	protein_coding	LY6/PLAUR	2	149329985	149474138
ENSG0000026452	0.248130534	0.002	0.01	OTUD7B	protein_coding	OTU deubic	1	149937812	150010726
ENSG0000014349	-0.208491049	0.002	0.01	INTS7	protein_coding	integrator c	1	211940399	212035557
ENSG0000006645	-0.271285755	0.002	0.01	GOLGA5	protein_coding	golgin A5 [S	14	92794305	92839947
ENSG0000013264	-0.282691677	0.002	0.01	BTBD3	protein_coding	BTB domain	20	11890723	11926609
ENSG0000016986	1.047372143	0.002	0.01	CTNND2	protein_coding	catenin delt	5	10971836	11904446
ENSG0000026704	0.724487138	0.002	0.01	ZNF850	protein_coding	zinc finger p	19	36714383	36772825
ENSG0000015412	-0.233510389	0.002	0.01	ANKH	protein_coding	ANKH inorg	5	14704800	14871778
ENSG0000016005	-0.877128871	0.002	0.01	CCDC28B	protein_coding	coiled-coil c	1	32200595	32205387
ENSG0000008615	-1.716291371	0.002	0.01	AQP6	protein_coding	aquaporin 6	12	49967194	49977139
ENSG0000022428	1.586011595	0.002	0.01	SLC25A5-A	lncRNA	SLC25A5 an	X	119465986	119469128

ENSG0000019859	-0.345455994	0.002	0.01	ZNF536	protein_coding	zinc finger p	19	30228290	30713538
ENSG0000003453	0.666714303	0.002	0.01	ASTE1	protein_coding	asteroid hor	3	131013875	131027649
ENSG0000010821	0.27908235	0.003	0.01	TSPAN14	protein_coding	tetraspanin	10	80454166	80533124
ENSG0000022568	-1.174413774	0.003	0.01	FAM225B	lncRNA	family with s	9	113102117	113111543
ENSG0000027938	1.138071932	0.003	0.01	GARRE1	protein_coding	granule asso	CHR_HSCHR	34261710	34308123
ENSG0000022194	2.092375217	0.003	0.01	LINC01465	lncRNA	long interge	12	62601751	62603690
ENSG0000013302	-0.767736504	0.003	0.01	PEMT	protein_coding	phosphatidy	17	17505563	17591708
ENSG0000013269	-1.161791519	0.003	0.01	BCAN	protein_coding	brevican [Sc	1	156641390	156659532
ENSG0000011410	0.399180486	0.003	0.01	CEP70	protein_coding	centrosoma	3	138494344	138594538
ENSG0000019885	-0.243038787	0.003	0.01	OSTC	protein_coding	oligosaccha	4	108650585	108667820
ENSG0000022198	-0.118105685	0.003	0.01	UBA52	protein_coding	ubiquitin A-	19	18571730	18577550
ENSG0000008673	-1.724473392	0.003	0.01	LAT2	protein_coding	linker for ac	7	74199652	74229834
ENSG0000026747	-1.739016669	0.003	0.01	NA	NA	NA	NA	NA	NA
ENSG0000016563	0.349378054	0.003	0.01	PRPF18	protein_coding	pre-mRNA	10	13586939	13630859
ENSG0000011171	-0.126955986	0.003	0.01	LDHB	protein_coding	lactate dehy	12	21635342	21757857
ENSG0000014600	0.724013415	0.003	0.01	PCDHB18P	transcribed_un	protocadhe	5	141234333	141237529
ENSG0000007597	0.350766356	0.003	0.01	MKRN2	protein_coding	makorin ring	3	12557087	12583713
ENSG0000016335	2.304402575	0.003	0.01	DCST2	protein_coding	DC-STAMP	1	155018520	155033781
ENSG0000026375	0.354584163	0.003	0.01	LINC00667	lncRNA	long interge	18	5237826	5290608
ENSG0000027715	3.445582598	0.003	0.01	H4C4	protein_coding	H4 clustered	6	26188710	26189112
ENSG0000013956	-1.221834431	0.003	0.01	ACVRL1	protein_coding	activin A rec	12	51906908	51923361
ENSG0000018220	-0.402414686	0.003	0.01	MOB2	protein_coding	MOB kinase	11	1469457	1501247
ENSG0000020458	4.438338091	0.003	0.01	ACOXL-AS	lncRNA	ACOXL antis	2	111098345	111116407
ENSG0000018529	0.355486326	0.003	0.01	CCDC137	protein_coding	coiled-coil c	17	81666737	81673904
ENSG0000025995	-0.595812878	0.003	0.01	AC107068.1	lncRNA	novel transc	4	47840122	47844339
ENSG0000008844	-0.212546959	0.003	0.01	ANKRD10	protein_coding	ankyrin repe	13	110878540	110915069
ENSG0000021542	0.331337772	0.003	0.01	DDX39B	protein_coding	DExD-box h	CHR_HSCHR	31520488	31536841
ENSG0000018307	-0.516505322	0.003	0.01	AFMID	protein_coding	arylformami	17	78187317	78207701
ENSG0000014041	0.219165468	0.003	0.01	TPM1	protein_coding	tropomyosin	15	63042632	63071915
ENSG0000026025	2.431012042	0.003	0.01	AC084262.1	lncRNA	novel transc	8	29352420	29353170
ENSG0000022402	1.026720159	0.003	0.01	EDRF1-DT	lncRNA	EDRF1 diver	10	125700436	125719566
ENSG0000027711	-1.49373847	0.003	0.01	CNOT3	protein_coding	CCR4-NOT	CHR_HSCHR	54138182	54156190
ENSG0000013819	0.368496338	0.003	0.01	EXOC6	protein_coding	exocyst com	10	92826831	93059493
ENSG0000008675	-0.13207946	0.003	0.01	HUWE1	protein_coding	HECT, UBA	X	53532096	53686728
ENSG0000010560	-0.763353568	0.003	0.01	CACNG7	protein_coding	calcium volt	19	53909335	53943941
ENSG0000013137	-0.229434738	0.003	0.01	TBC1D5	protein_coding	TBC1 doma	3	17157162	18444817
ENSG0000017548	0.732990538	0.003	0.01	POLD4	protein_coding	DNA polym	11	67350772	67356972
ENSG0000017840	-0.510744845	0.003	0.01	BEND3	protein_coding	BEN domain	6	107065182	107115515
ENSG0000012626	-0.284805417	0.003	0.01	COX6B1	protein_coding	cytochrome	19	35648323	35658782
ENSG0000013504	-0.395689928	0.003	0.01	CTSL	protein_coding	cathepsin L	9	87726109	87731469
ENSG0000027561	-0.328271982	0.003	0.01	BFAR	protein_coding	bifunctional	CHR_HSCHR	14632814	14669237
ENSG0000017699	-0.429211396	0.003	0.01	SMCR8	protein_coding	SMCR8-C9o	17	18315293	18328056
ENSG0000014759	0.581334578	0.003	0.01	LACTB2	protein_coding	lactamase b	8	70635318	70669185
ENSG0000024044	-0.646960117	0.003	0.01	FOXO3B	protein_coding	forkhead bo	17	18663505	18682262
ENSG0000027433	0.627869706	0.003	0.01	CU633967.1	lncRNA	uncharacter	21	5553637	5614880
ENSG0000027903	1.70971307	0.003	0.01	AC011447.(	TEC	TEC	19	20207192	20209789
ENSG0000027359	-0.727153204	0.003	0.01	SMIM11B	protein_coding	small integr	21	7744962	7777853
ENSG0000016379	-0.362677343	0.003	0.01	ZNF513	protein_coding	zinc finger p	2	27377235	27380790
ENSG0000018366	-0.555844377	0.003	0.01	GUSBP1	transcribed_un	GUSB pseuc	5	21341833	21589372
ENSG0000005280	0.240142038	0.003	0.01	MSMO1	protein_coding	methylstero	4	165327667	165343164
ENSG0000014712	-0.389991828	0.003	0.01	ZNF41	protein_coding	zinc finger p	X	47445879	47482946
ENSG0000013838	-0.406742603	0.003	0.01	METTL5	protein_coding	methyltrans	2	169810081	169824931
ENSG0000016074	0.600128392	0.003	0.01	ANO10	protein_coding	anoctamin 1	3	43354859	43691594
ENSG0000016322	-6.102697618	0.003	0.01	S100A9	protein_coding	S100 calciur	1	153357854	153361023
ENSG0000016338	0.360637084	0.003	0.01	NAXE	protein_coding	NAD(P)HX e	1	156591762	156594299
ENSG0000013554	-1.1032247	0.003	0.01	PKIB	protein_coding	cAMP-depe	6	122471917	122726373
ENSG0000025453	-0.579590843	0.003	0.01	PABPC4L	protein_coding	poly(A) binc	4	134196333	134201789
ENSG0000018230	-1.783761686	0.003	0.01	DCAF4L1	protein_coding	DDB1 and C	4	41981756	41986465

ENSG0000027279	-3.731417314	0.003	0.01	AL008721.1	lncRNA	novel transc	22	25436312	25436915
ENSG0000017671	-0.42271774	0.003	0.01	ACSF3	protein_coding	acyl-CoA sy	16	89088375	89164121
ENSG0000017372	1.108498769	0.003	0.01	AP000769.1	transcribed_un	Finkel-Biskis	11	65455258	65466720
ENSG0000014714	-0.657995157	0.003	0.01	CCDC120	protein_coding	coiled-coil c	X	49053572	49069857
ENSG0000027138	3.347047187	0.003	0.01	AL445228.3	lncRNA	novel transc	1	184385753	184386704
ENSG0000010873	-0.562333226	0.003	0.01	PEX12	protein_coding	peroxisoma	17	35574795	35578863
ENSG0000017910	0.210807781	0.003	0.01	TMTC2	protein_coding	transmembı	12	82686880	83134870
ENSG0000023407	-0.549205259	0.003	0.01	AC074117.1	lncRNA	novel transc	2	27356246	27367622
ENSG0000025546	1.296376854	0.003	0.01	AP001107.9	lncRNA	novel transc	11	66347950	66364804
ENSG0000016784	-0.326305901	0.003	0.01	MIS12	protein_coding	MIS12 kinet	17	5486285	5490814
ENSG0000027388	1.937764934	0.003	0.01	FRMD6-AS	lncRNA	FRMD6 anti	14	51649516	51651744
ENSG0000027629	0.236194237	0.003	0.01	PIP4K2B	protein_coding	phosphatidy	17	38765689	38800126
ENSG0000000283	0.241490305	0.003	0.01	LASP1	protein_coding	LIM and SH	17	38869859	38921770
ENSG0000017892	-0.936620566	0.003	0.01	HYI	protein_coding	hydroxypyru	1	43451003	43453989
ENSG0000021793	-0.527363955	0.003	0.01	PAM16	protein_coding	presequenc	16	4331549	4355607
ENSG0000023948	-2.23663519	0.003	0.01	AC091390.3	unprocessed_p	pseudogene	7	102380465	102382737
ENSG0000011242	-0.462446373	0.003	0.01	EPM2A	protein_coding	EPM2A gluc	6	145382535	145736023
ENSG0000014349	-0.510578991	0.003	0.01	TAF1A	protein_coding	TATA-box b	1	222557902	222589933
ENSG0000019883	-1.105570145	0.003	0.01	SELENOM	protein_coding	selenoprote	22	31104772	31120069
ENSG0000012688	-1.611684627	0.003	0.01	FAM78A	protein_coding	family with s	9	131258076	131276510
ENSG0000017120	0.274084569	0.003	0.01	TMEM126B	protein_coding	transmembı	11	85628573	85636539
ENSG0000012026	0.197196248	0.003	0.01	PCMT1	protein_coding	protein-L-is	6	149749443	149811420
ENSG0000011371	0.231267087	0.003	0.01	HMGXB3	protein_coding	HMG-box c	5	150000046	150053142
ENSG0000018445	-1.529028737	0.003	0.01	CCR10	protein_coding	C-C motif c	17	42678889	42683917
ENSG0000012424	-1.120642586	0.003	0.01	BCAS4	protein_coding	breast carci	20	50794894	50882676
ENSG0000012646	0.325276156	0.003	0.01	SCAF1	protein_coding	SR-related (	19	49642209	49658642
ENSG0000010005	-0.431107985	0.003	0.01	CRYBB2P1	transcribed_un	crystallin be	22	25448105	25520854
ENSG0000024234	-1.81405201	0.003	0.01	NA	NA	NA	NA	NA	NA
ENSG0000011164	1.541900129	0.003	0.01	ACRBP	protein_coding	acrosin binc	12	6638075	6647433
ENSG0000020802	-2.72614204	0.003	0.01	MIR199A2	miRNA	microRNA 1	1	172144535	172144644
ENSG0000025619	3.682835564	0.003	0.01	AP003721.1	lncRNA	novel transc	11	60916339	60925397
ENSG0000010010	0.289888381	0.003	0.01	TFIP11	protein_coding	tuftelin inter	22	26491225	26512505
ENSG0000008312	0.32663153	0.003	0.01	BCKDHB	protein_coding	branched ch	6	80106647	80346270
ENSG0000024984	2.917711199	0.003	0.01	LINC02021	lncRNA	long interge	3	130111669	130201336
ENSG0000023594	-6.092115984	0.003	0.01	NA	NA	NA	NA	NA	NA
ENSG0000027095	3.589286632	0.003	0.01	AC009948.2	lncRNA	novel transc	2	178541125	178541799
ENSG0000017204	0.297318116	0.003	0.01	USP19	protein_coding	ubiquitin sp	3	49108046	49120938
ENSG0000020399	-0.61010721	0.003	0.01	ZYG11A	protein_coding	zyg-11 fam	1	52842511	52894998
ENSG0000012606	-0.388950137	0.003	0.01	TMEM115	protein_coding	transmembı	3	50354750	50359521
ENSG0000013030	1.097426327	0.003	0.01	SLC27A1	protein_coding	solute carrie	19	17468769	17506168
ENSG0000010506	0.266806788	0.003	0.01	PPP6R1	protein_coding	protein pho	19	55229779	55259017
ENSG0000017754	-0.814867156	0.003	0.01	RABEP2	protein_coding	rabaptin, RA	16	28904421	28936526
ENSG0000026955	0.486054155	0.003	0.01	TMEM185A	protein_coding	transmembı	X	149596556	149631912
ENSG0000018818	-0.876820055	0.003	0.01	LINC00265	lncRNA	long interge	7	39733430	39793092
ENSG0000027656	1.447800724	0.003	0.01	IRF7	protein_coding	interferon re	CHR_HSCHR	612805	616257
ENSG0000027214	1.401485359	0.003	0.01	AC022400.4	lncRNA	novel transc	10	73703735	73713581
ENSG0000027464	-0.248869967	0.003	0.01	RPS9	protein_coding	ribosomal p	CHR_HSCHR	54201241	54249371
ENSG0000012599	0.499764397	0.003	0.01	ROMO1	protein_coding	reactive oxy	20	35699272	35700984
ENSG0000014071	0.249484348	0.003	0.01	FTO	protein_coding	FTO alpha-l	16	53701692	54158512
ENSG0000027897	-2.502042792	0.003	0.01	NA	NA	NA	NA	NA	NA
ENSG0000012417	0.458505537	0.003	0.01	ATP5F1E	protein_coding	ATP synthas	20	59025475	59032345
ENSG0000027643	1.798575011	0.003	0.01	RDM1	protein_coding	RAD52 mot	CHR_HSCHR	35918066	35930773
ENSG0000014927	-0.103669907	0.003	0.01	RPS3	protein_coding	ribosomal p	11	75399515	75422280
ENSG0000016328	-1.941371729	0.003	0.01	GABRB1	protein_coding	gamma-am	4	46993723	47426447
ENSG0000002319	-0.36393324	0.003	0.01	RNH1	protein_coding	ribonucleas	11	494512	507300
ENSG0000012535	-0.468941258	0.003	0.01	RNF113A	protein_coding	ring finger p	X	119870475	119871733
ENSG0000013404	-0.459029679	0.003	0.01	MBD2	protein_coding	methyl-CpG	18	54151606	54224669
ENSG0000000477	0.242137703	0.003	0.01	NDUFAB1	protein_coding	NADH:ubiqu	16	23581014	23596316

ENSG0000017927	-1.780672308	0.003	0.01	MEIS3P1	processed_pse	Meis homec	17	15786618	15787575
ENSG0000015745	-0.212225007	0.003	0.01	RNF111	protein_coding	ring finger p	15	58865175	59097419
ENSG0000015241	0.420844536	0.003	0.01	HOMER1	protein_coding	homer scaff	5	79372636	79514134
ENSG0000016524	0.205616508	0.003	0.01	ATP7A	protein_coding	ATPase cop	X	77910656	78050395
ENSG0000008566	-0.17920469	0.003	0.01	AKR1B1	protein_coding	aldo-keto re	7	134442356	134459284
ENSG0000013480	-0.382209972	0.003	0.01	TIMM10	protein_coding	translocase	11	57528464	57530803
ENSG0000014145	0.394951137	0.003	0.01	NPC1	protein_coding	NPC intrace	18	23506184	23586506
ENSG0000013106	-0.3847483	0.003	0.01	ACSS2	protein_coding	acyl-CoA sy	20	34872146	34927962
ENSG0000014641	0.745294393	0.003	0.01	MTFR2	protein_coding	mitochondr	6	136231024	136250335
ENSG0000017375	-0.325203946	0.003	0.01	STAT5B	protein_coding	signal trans	17	42199177	42276707
ENSG0000006237	-0.650358607	0.003	0.01	ZNF112	protein_coding	zinc finger p	19	44326555	44367217
ENSG0000013811	-0.553217881	0.003	0.01	MFSD13A	protein_coding	major facilit	10	102461395	102477045
ENSG0000007300	-0.46176809	0.003	0.01	PVR	protein_coding	PVR cell adh	19	44643798	44666162
ENSG0000022691	-0.566653939	0.003	0.01	WDR46	protein_coding	WD repeat (	CHR_HSCHR	33257029	33267452
ENSG0000023542	-0.602125535	0.003	0.01	NA	NA	NA	NA	NA	NA
ENSG0000016566	0.22608174	0.003	0.01	QSOX2	protein_coding	quiescin sul	9	136206333	136245812
ENSG0000001464	0.204995774	0.003	0.01	MDH1	protein_coding	malate dehy	2	63588609	63607197
ENSG0000022585	-2.122360384	0.003	0.01	LINC02816	lncRNA	long interge	1	180906651	180909166
ENSG0000014047	-0.394486917	0.003	0.01	ADAMTS17	protein_coding	ADAM meta	15	99971437	100342005
ENSG0000027347	6.105563498	0.003	0.01	BX649601.1	lncRNA	novel transc	9	134168769	134169340
ENSG0000014137	-0.347784495	0.003	0.01	PTRH2	protein_coding	peptidyl-tRI	17	59674636	59707626
ENSG0000024815	0.921860542	0.003	0.01	ANK2-AS1	lncRNA	ANK2 antise	4	112973272	113071962
ENSG0000010561	-0.356536814	0.003	0.01	MAST1	protein_coding	microtubule	19	12833951	12874952
ENSG0000015871	-0.240487702	0.003	0.01	ELK4	protein_coding	ETS transcri	1	205597556	205632011
ENSG0000017044	0.209510548	0.003	0.01	HARS1	protein_coding	histidyl-tRN	5	140673035	140691537
ENSG0000025749	-2.679142511	0.003	0.01	AC121761.1	lncRNA	novel transc	12	75483454	75489820
ENSG0000021716	0.666789051	0.003	0.01	ANKRD18E	processed_pse	ankyrin repe	6	39110321	39112952
ENSG0000020635	0.807711671	0.003	0.01	CCHCR1	protein_coding	coiled-coil a	CHR_HSCHR	31136168	31151940
ENSG0000024101	1.486442086	0.003	0.01	AC114490.1	transcribed_un	novel platel	1	34975699	34978706
ENSG0000011656	0.105275944	0.003	0.01	SFPQ	protein_coding	splicing fact	1	35176378	35193145
ENSG0000012856	-0.288895101	0.003	0.01	PRKRIP1	protein_coding	PRKR intera	7	102363872	102426676
ENSG0000015142	-0.214220233	0.003	0.01	FER	protein_coding	FER tyrosine	5	108747841	109196841
ENSG0000016990	-2.66354935	0.003	0.01	TM4SF4	protein_coding	transmembr	3	149474697	149503394
ENSG0000023642	-1.997806373	0.003	0.01	LINC01134	lncRNA	long interge	1	3900352	3917225
ENSG0000010413	0.155349531	0.003	0.01	EIF3J	protein_coding	eukaryotic t	15	44537125	44563029
ENSG0000017673	2.22004239	0.003	0.01	PFN4	protein_coding	profilin fami	2	24114809	24123464
ENSG0000004665	-0.407699555	0.003	0.01	GPM6B	protein_coding	glycoprotein	X	13770939	13938638
ENSG0000024268	-1.148911381	0.003	0.01	CNTF	protein_coding	ciliary neurc	11	58622665	58625733
ENSG0000016397	0.355047532	0.003	0.01	MELTF	protein_coding	melanotrans	3	196988621	197029817
ENSG0000001081	0.175325831	0.003	0.01	FYN	protein_coding	FYN proto-c	6	111660332	111873452
ENSG0000021346	0.267828379	0.003	0.01	SYNJ2BP	protein_coding	synaptojanin	14	70366499	70417090
ENSG0000016706	0.872339173	0.003	0.01	DUSP18	protein_coding	dual specific	22	30652051	30667890
ENSG0000016618	0.61180697	0.003	0.01	HPS6	protein_coding	HPS6 bioge	10	102065349	102068036
ENSG0000001053	0.541239181	0.003	0.01	ZNF200	protein_coding	zinc finger p	16	3222325	3236221
ENSG0000002328	-0.146268867	0.003	0.01	RB1CC1	protein_coding	RB1 inducib	8	52622458	52745843
ENSG0000018704	-0.594457739	0.003	0.01	TMEM216	protein_coding	transmembr	11	61392360	61398863
ENSG0000016889	-0.829052969	0.003	0.01	TMEM150A	protein_coding	transmembr	2	85598547	85603196
ENSG0000019659	0.555057194	0.003	0.01	ZNF782	protein_coding	zinc finger p	9	96816269	96875623
ENSG0000026025	-1.175938604	0.003	0.01	AL035071.1	lncRNA	novel transc	20	32856621	32858751
ENSG0000019659	0.127035402	0.003	0.01	HDAC2	protein_coding	histone dea	6	113933028	114011308
ENSG0000009297	0.315241468	0.003	0.01	GPATCH2	protein_coding	G-patch do	1	217426992	217631090
ENSG0000008887	-0.562084448	0.003	0.01	ZNF343	protein_coding	zinc finger p	20	2481817	2524702
ENSG0000013553	0.149648173	0.003	0.01	CD164	protein_coding	CD164 mole	6	109366514	109382467
ENSG0000027429	0.829086533	0.003	0.01	DECR2	protein_coding	2,4-dienoyl	CHR_HSCHR	401826	412487
ENSG0000027001	1.054616119	0.003	0.01	ZNF559-ZN	protein_coding	ZNF559-ZN	19	9324174	9382617
ENSG0000008094	0.778874194	0.003	0.01	CROCCP3	transcribed_un	CROCC pse	1	16467436	16499257
ENSG0000010091	-0.336485995	0.003	0.01	PSME2	protein_coding	proteasome	14	24143362	24147570
ENSG0000017116	-0.670904954	0.003	0.01	NAIF1	protein_coding	nuclear apo	9	128061233	128068206

ENSG0000016440	0.982032532	0.003	0.01	LEAP2	protein_coding	liver enriche	5	132873444	132875046
ENSG0000020417	-0.796859987	0.003	0.01	PTPN20	protein_coding	protein tyro	10	46911396	47002488
ENSG0000016408	-0.551443284	0.003	0.01	TEX264	protein_coding	testis expres	3	51662693	51704323
ENSG0000010640	-0.726533332	0.003	0.01	CLDN15	protein_coding	claudin 15 [	7	101232092	101238820
ENSG0000025538	-0.800816601	0.003	0.01	Z97989.1	lncRNA	chromosom	6	111599875	111602295
ENSG0000016987	-0.528951395	0.003	0.01	TRIM56	protein_coding	tripartite mo	7	101085481	101097967
ENSG0000027951	2.631235834	0.003	0.01	AL157902.2	TEC	TEC	1	117493515	117495006
ENSG0000012416	0.178450305	0.003	0.01	VAPB	protein_coding	VAMP assoc	20	58389229	58451101
ENSG0000001336	-1.087455718	0.003	0.01	MVP	protein_coding	major vault	16	29820394	29848039
ENSG0000011409	0.28874646	0.003	0.01	ARMC8	protein_coding	armadillo re	3	138187248	138298384
ENSG0000023238	-0.445006607	0.003	0.01	SMIM26	protein_coding	small integr	20	18567347	18569563
ENSG0000018933	-0.181987099	0.003	0.01	SLC35E2B	protein_coding	solute carrie	1	1659529	1692795
ENSG0000026564	2.608927899	0.003	0.01	TUFMP1	processed_pse	Tu translatic	17	27082690	27084036
ENSG0000014376	-0.134475881	0.003	0.01	ARF1	protein_coding	ADP ribosyl	1	228082660	228099212
ENSG0000001999	0.27071246	0.003	0.01	ZRANB1	protein_coding	zinc finger R	10	124942123	124988189
ENSG0000017024	0.234098196	0.003	0.01	PDCD6IP	protein_coding	programme	3	33798571	33869707
ENSG0000014422	-0.337362172	0.003	0.01	SPOPL	protein_coding	speckle type	2	138501770	138573547
ENSG0000018161	0.804978749	0.003	0.01	GPR135	protein_coding	G protein-c	14	59429022	59465342
ENSG0000022886	1.332162745	0.003	0.01	NRM	protein_coding	nurim [Sour	CHR_HSCHR	30732707	30736080
ENSG0000011594	0.212129505	0.003	0.01	ORC4	protein_coding	origin recog	2	147930396	148021604
ENSG0000018401	-0.172504898	0.003	0.01	DENND5A	protein_coding	DENN doma	11	9138825	9265350
ENSG0000012780	-0.295046328	0.003	0.01	METTL16	protein_coding	methyltrans	17	2405562	2511891
ENSG0000022407	-0.192300148	0.003	0.01	SNHG14	lncRNA	small nuclec	15	24978583	25420336
ENSG0000010340	-0.296357212	0.003	0.01	USP31	protein_coding	ubiquitin sp	16	23061406	23149270
ENSG0000023653	-2.174152115	0.003	0.01	LINC01695	lncRNA	long interge	21	28116094	28228667
ENSG0000008169	0.510793061	0.003	0.01	JMJD4	protein_coding	jumonji don	1	227730425	227735411
ENSG0000022948	-1.661028162	0.003	0.01	NA	NA	NA	NA	NA	NA
ENSG0000011785	-0.218816012	0.003	0.01	OSBPL9	protein_coding	oxysterol bi	1	51577179	51798427
ENSG0000010728	0.940518815	0.003	0.01	APBA1	protein_coding	amyloid bet	9	69427532	69672371
ENSG0000021333	0.583904116	0.003	0.01	ANKRD39	protein_coding	ankyrin repe	2	96836611	96858016
ENSG0000022079	-1.105738773	0.003	0.01	RPL21P119	processed_pse	ribosomal p	16	9156402	9156881
ENSG0000027314	1.406558854	0.003	0.01	LINC00653	lncRNA	long interge	20	18794529	18796067
ENSG0000010254	-0.789413323	0.003	0.01	CAB39L	protein_coding	calcium binc	13	49308650	49444064
ENSG0000011528	-0.354326039	0.003	0.01	PCGF1	protein_coding	polycomb g	2	74505043	74507695
ENSG0000022458	0.512751512	0.003	0.01	MDC1	protein_coding	mediator of	CHR_HSCHR	30689666	30707748
ENSG0000009097	-0.395640037	0.003	0.01	NAT14	protein_coding	N-acetyltrar	19	55485188	55487568
ENSG0000015427	-0.116723538	0.003	0.01	UCHL1	protein_coding	ubiquitin C-	4	41256413	41268455
ENSG0000014354	-6.03709038	0.003	0.01	S100A8	protein_coding	S100 calciur	1	153390032	153391073
ENSG0000020394	-1.211633811	0.003	0.01	SAMD13	protein_coding	sterile alpha	1	84298366	84389957
ENSG0000015862	0.384895828	0.003	0.01	COPG2	protein_coding	COPI coat c	7	130506238	130668748
ENSG0000013594	0.285204902	0.003	0.01	COX5B	protein_coding	cytochrome	2	97646062	97648383
ENSG0000016645	0.504673716	0.003	0.01	AKIP1	protein_coding	A-kinase int	11	8911139	8920084
ENSG0000007199	-0.232379248	0.003	0.01	PDCD2	protein_coding	programme	6	170575295	170584692
ENSG0000023037	-1.177754086	0.003	0.01	GOLGA6L5	transcribed_un	golgin A6 fa	15	84507885	84516814
ENSG0000017929	-1.118232269	0.003	0.01	TMEM151A	protein_coding	transmembi	11	66291894	66296664
ENSG0000015702	-0.288306777	0.003	0.01	SEC13	protein_coding	SEC13 hom	3	10293131	10321112
ENSG0000014083	0.348862286	0.003	0.01	TXNL4B	protein_coding	thioredoxin	16	72044289	72094431
ENSG0000016408	-0.754193285	0.003	0.01	PPM1M	protein_coding	protein pho	3	52245759	52250599
ENSG0000024790	0.789174472	0.003	0.01	AC024896.1	lncRNA	novel transc	12	26971586	26979582
ENSG0000013178	0.352146378	0.003	0.01	PIAS3	protein_coding	protein inhil	1	145848522	145859836
ENSG0000016675	0.599880908	0.003	0.01	SLFN5	protein_coding	schlafen fan	17	35243071	35273655
ENSG0000016535	-0.468117932	0.003	0.01	INTS6L	protein_coding	integrator c	X	135520643	135582510
ENSG0000013866	0.260765413	0.003	0.01	COPS4	protein_coding	COP9 signa	4	83034447	83075818
ENSG0000018522	-0.592498338	0.003	0.01	PGBD2	protein_coding	piggyBac tra	1	248906196	248919946
ENSG0000012829	0.740868784	0.003	0.01	TPST2	protein_coding	tyrosylprote	22	26521996	26596717
ENSG0000007120	-0.552738501	0.003	0.01	ARHGAP10	protein_coding	Rho GTPase	4	147732063	148072776
ENSG0000017144	0.234694659	0.003	0.01	MCC	protein_coding	MCC regula	5	113022099	113488823
ENSG0000017200	-0.357899541	0.003	0.01	RAB33B	protein_coding	RAB33B, me	4	139453232	139476609

ENSG0000010042	0.886315674	0.003	0.01	HDAC10	protein_coding	histone dea	22	50245183	50251405
ENSG0000006872	0.488033535	0.003	0.01	TTC7A	protein_coding	tetratricope	2	46916157	47076137
ENSG0000015710	-0.152736724	0.003	0.01	SMG1	protein_coding	SMG1 nons	16	18804860	18926408
ENSG0000019869	0.360053328	0.003	0.01	FAN1	protein_coding	FANCD2 an	15	30890559	30943108
ENSG0000017907	1.980731632	0.003	0.01	CCDC89	protein_coding	coiled-coil d	11	85683848	85686195
ENSG0000018372	-0.327225634	0.003	0.01	CMTM4	protein_coding	CKLF like M	16	66614750	66696743
ENSG0000024211	0.640123535	0.003	0.01	AMACR	protein_coding	alpha-meth	5	33986165	34008104
ENSG0000017130	0.348381705	0.003	0.01	ZDHHC16	protein_coding	zinc finger D	10	97446170	97457370
ENSG0000019883	0.176268155	0.003	0.01	OPA1	protein_coding	OPA1 mitoc	3	193593144	193697811
ENSG0000013717	-0.363583543	0.004	0.01	KLC4	protein_coding	kinesin light	6	43040777	43075095
ENSG0000027606	3.115502922	0.004	0.01	NDUFA3	protein_coding	NADH:ubiqu	CHR_HSCHR	54102782	54108779
ENSG0000008730	-0.219739735	0.004	0.01	TXNDC16	protein_coding	thioredoxin	14	52430596	52552522
ENSG0000011017	0.544200045	0.004	0.01	TRIM3	protein_coding	tripartite mo	11	6448613	6474459
ENSG0000008380	-1.438364284	0.004	0.01	SLC27A5	protein_coding	solute carrie	19	58479512	58512413
ENSG0000013685	0.178660023	0.004	0.01	STXBP1	protein_coding	syntaxin bin	9	127579370	127696027
ENSG0000000737	-1.245335106	0.004	0.01	PAX6	protein_coding	paired box 6	11	31784779	31817961
ENSG0000024344	-1.326570778	0.004	0.01	NA	NA	NA	NA	NA	NA
ENSG0000027237	-3.123507281	0.004	0.01	AC026979.3	lncRNA	novel transc	8	30197404	30198048
ENSG0000020496	0.759225932	0.004	0.01	PCDHA2	protein_coding	protocadhe	5	140794852	141012347
ENSG0000027626	-2.693788255	0.004	0.01	TFPT	protein_coding	TCF3 fusion	CHR_HSCHR	54107066	54115801
ENSG0000019888	0.223576049	0.004	0.01	SMC5	protein_coding	structural m	9	70258978	70354873
ENSG0000024608	0.906206621	0.004	0.01	AC016065.1	lncRNA	novel transc	8	6403551	6407142
ENSG0000027715	1.788016038	0.004	0.01	AL139384.2	lncRNA	novel transc	13	112602828	112606417
ENSG0000016580	0.529789725	0.004	0.01	CASP7	protein_coding	caspase 7 [S	10	113679162	113730907
ENSG0000024262	1.282692384	0.004	0.01	AC092910.3	lncRNA	novel transc	3	120094895	120136783
ENSG0000028012	-0.906990091	0.004	0.01	AL662795.2	TEC	TEC	6	30282349	30286054
ENSG0000020640	-0.729167376	0.004	0.01	GPANK1	protein_coding	G-patch do	CHR_HSCHR	31651454	31656514
ENSG0000020422	-0.557745742	0.004	0.01	WDR46	protein_coding	WD repeat d	CHR_HSCHR	33200708	33211131
ENSG0000017662	-0.205427558	0.004	0.01	MEX3C	protein_coding	mex-3 RNA	18	51174550	51218333
ENSG0000015559	0.270607666	0.004	0.01	ZKSCAN2	protein_coding	zinc finger w	16	25236001	25257845
ENSG0000018500	0.430125682	0.004	0.01	DGAT1	protein_coding	diacylglycer	8	144314584	144326910
ENSG0000000581	-0.272267116	0.004	0.01	FBXL3	protein_coding	F-box and l	13	76992598	77027195
ENSG0000027226	-6.028776073	0.004	0.01	AC009686.2	lncRNA	novel transc	8	80032724	80033300
ENSG0000027671	0.627358478	0.004	0.01	CSPG4P10	transcribed_un	chondroitin	15	82459472	82477258
ENSG0000020553	-0.479475738	0.004	0.01	SMG1P2	transcribed_un	SMG1 pseu	16	29527568	29594966
ENSG0000011667	-0.153618959	0.004	0.01	IVNS1ABP	protein_coding	influenza vir	1	185296388	185317273
ENSG0000017952	0.64923095	0.004	0.01	EIF3J-DT	lncRNA	EIF3J diverg	15	44527257	44537046
ENSG0000023717	0.648492344	0.004	0.01	B3GNT9	protein_coding	UDP-GlcNA	16	67148104	67150998
ENSG0000022820	-0.504742248	0.004	0.01	RNF144A-A	lncRNA	RNF144A ar	2	6911754	6918734
ENSG0000000476	-0.382060562	0.004	0.01	VPS50	protein_coding	VPS50 subu	7	93232340	93361123
ENSG0000027950	0.413187075	0.004	0.01	NA	NA	NA	NA	NA	NA
ENSG0000025004	-2.401252425	0.004	0.01	AC106895.1	lncRNA	novel transc	4	173877471	173913252
ENSG0000018629	-0.145958977	0.004	0.01	PPP1CC	protein_coding	protein pho	12	110719680	110742939
ENSG0000008715	-0.212353903	0.004	0.01	ATXN7L3	protein_coding	ataxin 7 like	17	44191805	44200113
ENSG0000016752	1.62749159	0.004	0.01	PROCA1	protein_coding	protein inte	17	28703197	28711854
ENSG0000022842	-0.723810391	0.004	0.01	VPS52	protein_coding	VPS52 subu	CHR_HSCHR	33228191	33249968
ENSG0000020443	-0.846445241	0.004	0.01	GPANK1	protein_coding	G-patch do	6	31661228	31666283
ENSG0000016929	-1.532126737	0.004	0.01	NR0B1	protein_coding	nuclear rece	X	30304206	30309390
ENSG0000016000	-0.198428955	0.004	0.01	ARHGAP35	protein_coding	Rho GTPase	19	46860997	47005077
ENSG0000019673	-1.320544238	0.004	0.01	COL27A1	protein_coding	collagen typ	9	114155537	114312511
ENSG0000016639	0.400914548	0.004	0.01	GARRE1	protein_coding	granule asso	19	34254552	34355566
ENSG0000017952	0.693486727	0.004	0.01	LBX2	protein_coding	ladybird hor	2	74497517	74503316
ENSG0000016239	-0.983318069	0.004	0.01	ACOT11	protein_coding	acyl-CoA th	1	54542257	54639192
ENSG0000027772	-3.549676011	0.004	0.01	NDUFA3	protein_coding	NADH:ubiqu	CHR_HSCHR	54102906	54107026
ENSG0000012949	0.297074596	0.004	0.01	HEATR5A	protein_coding	HEAT repea	14	31291788	31420550
ENSG0000025586	-2.720633664	0.004	0.01	NA	NA	NA	NA	NA	NA
ENSG0000020417	-0.220637729	0.004	0.01	MACO1	protein_coding	macoilin 1 [	1	25430858	25500209
ENSG0000025040	1.570392109	0.004	0.01	NA	NA	NA	NA	NA	NA

ENSG0000018093	0.708837615	0.004	0.01	ZNF572	protein_coding	zinc finger p	8	124973295	124979389
ENSG0000001559	0.399681243	0.004	0.01	STMN4	protein_coding	stathmin 4 [	8	27235323	27258420
ENSG0000023153	5.998115575	0.004	0.01	AL157932.1	lncRNA	novel transc	13	42043727	42044247
ENSG0000019849	-0.170460994	0.004	0.01	YTHDF2	protein_coding	YTH N6-me	1	28736621	28769775
ENSG0000027429	2.796044126	0.004	0.01	H2BC6	protein_coding	H2B clustere	6	26172059	26184655
ENSG0000019820	-0.921303991	0.004	0.01	ZXDA	protein_coding	zinc finger X	X	57906708	57910820
ENSG0000016361	0.397692961	0.004	0.01	SPICE1	protein_coding	spindle and	3	113442718	113515187
ENSG0000022191	0.217722586	0.004	0.01	PPP2R2A	protein_coding	protein pho	8	26291508	26372680
ENSG0000009671	-0.227070923	0.004	0.01	SIRT1	protein_coding	sirtuin 1 [So	10	67884656	67918390
ENSG0000018154	-0.120705916	0.004	0.01	MAB21L2	protein_coding	mab-21 like	4	150582151	150584693
ENSG0000026161	-0.589695907	0.004	0.01	AC093525.(	lncRNA	novel transc	16	2554060	2556060
ENSG0000016255	-1.551238091	0.004	0.01	ALPL	protein_coding	alkaline phc	1	21509397	21578410
ENSG0000012621	-0.251443155	0.004	0.01	TUBGCP3	protein_coding	tubulin gam	13	112485011	112588205
ENSG0000014036	-0.310472816	0.004	0.01	UBE2Q2	protein_coding	ubiquitin co	15	75843307	75901078
ENSG0000016548	-0.352391515	0.004	0.01	MICU2	protein_coding	mitochondr	13	21492691	21604181
ENSG0000023269	1.090685964	0.004	0.01	ZNRD1ASP	lncRNA	zinc ribbon	CHR_HSCHR	30079474	30140140
ENSG0000010569	-2.589621345	0.004	0.01	LSR	protein_coding	lipolysis stin	19	35248330	35267964
ENSG0000021346	0.496941967	0.004	0.01	ERV3-1	protein_coding	endogenous	7	64990356	65006743
ENSG0000023582	-0.53170266	0.004	0.01	OLMALINC	lncRNA	oligodendrc	10	100373099	100454043
ENSG0000010335	-0.164616817	0.004	0.01	UBFD1	protein_coding	ubiquitin fai	16	23557721	23574389
ENSG0000017626	0.419895217	0.004	0.01	ZBTB8OS	protein_coding	zinc finger a	1	32600172	32650903
ENSG0000014822	0.842469716	0.004	0.01	WDR31	protein_coding	WD repeat (	9	113313222	113340298
ENSG0000025576	0.536140478	0.004	0.01	GOLGA2P1	transcribed_un	GOLGA2 ps	15	82472993	82513950
ENSG0000027260	-1.070565056	0.004	0.01	AC073073.2	lncRNA	novel transc	7	105571083	105573660
ENSG0000014539	-0.933741751	0.004	0.01	USP53	protein_coding	ubiquitin sp	4	119212587	119295517
ENSG0000014458	0.206181901	0.004	0.01	CNOT9	protein_coding	CCR4-NOT	2	218568580	218597080
ENSG0000010901	0.776105643	0.004	0.01	DHRS7B	protein_coding	dehydroger	17	21123364	21193265
ENSG0000025006	0.83669081	0.004	0.01	YJEFN3	protein_coding	YjeF N-term	19	19528861	19537581
ENSG0000018235	0.724725604	0.004	0.01	KBTBD3	protein_coding	kelch repeat	11	106051098	106077459
ENSG0000010429	0.199352514	0.004	0.01	FZD3	protein_coding	frizzled class	8	28494205	28574267
ENSG0000011539	0.322029579	0.004	0.01	FANCL	protein_coding	FA complen	2	58159243	58241372
ENSG0000014480	0.554627926	0.004	0.01	NFKBIZ	protein_coding	NFKB inhibi	3	101827991	101861022
ENSG0000017785	0.800973747	0.004	0.01	TMEM187	protein_coding	transmembı	X	153972754	153983194
ENSG0000023921	1.228918594	0.004	0.01	NCK1-DT	lncRNA	NCK1 diverç	3	136835345	136862618
ENSG0000018437	-1.625173828	0.004	0.01	ACTRT3	protein_coding	actin relatec	3	169766921	169769561
ENSG0000019665	0.32761059	0.004	0.01	ZKSCAN5	protein_coding	zinc finger v	7	99504662	99534700
ENSG0000027324	-3.926748602	0.004	0.01	AC013468.1	lncRNA	novel transc	2	189763859	189764456
ENSG0000008409	-0.400210773	0.004	0.01	REST	protein_coding	RE1 silencin	4	56907876	56966808
ENSG0000010786	-0.702285529	0.004	0.01	CPEB3	protein_coding	cytoplasmic	10	92046692	92291078
ENSG0000016094	0.375524646	0.004	0.01	TONSL	protein_coding	tonsoku like	8	144428775	144444440
ENSG0000027773	1.132533987	0.004	0.01	5_8S_rRNA	rRNA	5.8S ribosor	21	8256781	8256933
ENSG0000012531	0.670439051	0.004	0.01	HROB	protein_coding	homologou	17	44141906	44162476
ENSG0000007335	-1.463312062	0.004	0.01	LLGL2	protein_coding	LLGL scribbl	17	75525080	75575209
ENSG0000022512	1.861849162	0.004	0.01	RANP4	processed_pse	RAN pseudo	X	136807223	136807877
ENSG0000010578	1.530759663	0.004	0.01	RUNDC3B	protein_coding	RUN domai	7	87627548	87832296
ENSG0000017640	1.152609873	0.004	0.01	EID2B	protein_coding	EP300 intera	19	39530987	39532852
ENSG0000026545	2.517007237	0.004	0.01	AC132938.4	lncRNA	novel transc	17	82454140	82458521
ENSG0000016694	-0.32258935	0.004	0.01	CCNDBP1	protein_coding	cyclin D1 bi	15	43185118	43197177
ENSG0000009043	0.35204303	0.004	0.01	MUL1	protein_coding	mitochondr	1	20499448	20508151
ENSG0000003317	-0.263893776	0.004	0.01	FUT8	protein_coding	fucosyltrans	14	65410592	65744121
ENSG0000022827	0.953685732	0.004	0.01	AL021707.2	lncRNA	novel transc	22	38667585	38681847
ENSG0000024888	-5.979879237	0.004	0.01	AC010280.2	lncRNA	novel transc	5	68430427	68434481
ENSG0000010008	0.300096925	0.004	0.01	GGA1	protein_coding	golgi associ	22	37608725	37633564
ENSG0000017501	-4.360768243	0.004	0.01	TEX36	protein_coding	testis expres	10	125576522	125683163
ENSG0000016286	0.453840364	0.004	0.01	PPP1R21	protein_coding	protein pho	2	48440598	48515391
ENSG0000023809	1.786496999	0.004	0.01	LINC02037	lncRNA	long interge	3	194247638	194260720
ENSG0000010015	-0.206869788	0.004	0.01	TTC28	protein_coding	tetratricope	22	27978014	28679865
ENSG0000015355	0.970546826	0.004	0.01	FBXL2	protein_coding	F-box and l	3	33277025	33403662

ENSG0000014522	0.373118436	0.004	0.01	LYAR	protein_coding	Ly1 antibod	4	4267701	4290154
ENSG0000023560	0.600548236	0.004	0.01	AF127577.4	lncRNA	novel transc	21	14818843	15014430
ENSG0000018786	-1.010559637	0.004	0.01	CCDC157	protein_coding	coiled-coil c	22	30356635	30378658
ENSG0000014157	-1.706668717	0.004	0.01	SECTM1	protein_coding	secreted an	17	82321024	82334074
ENSG0000013381	-0.761944616	0.004	0.01	MICAL2	protein_coding	microtubule	11	12094008	12359144
ENSG0000018580	0.419761219	0.004	0.01	DMWD	protein_coding	DM1 locus,	19	45782947	45792845
ENSG0000022705	-0.519630806	0.004	0.01	WDR46	protein_coding	WD repeat c	6	33279108	33289247
ENSG0000004986	0.328897503	0.004	0.01	HEXB	protein_coding	hexosaminic	5	74640023	74722647
ENSG0000014388	-3.014595864	0.004	0.01	ATP6V1C2	protein_coding	ATPase H+	2	10721100	10785110
ENSG0000027347	1.287749804	0.004	0.01	AC096733.2	lncRNA	novel transc	4	140756528	140757921
ENSG0000026804	-0.329125512	0.004	0.01	NBPF12	protein_coding	NBPF memb	1	146938744	146996202
ENSG0000013787	-1.788064839	0.004	0.01	GCOM1	protein_coding	GRINL1A cc	15	57591908	57714745
ENSG0000020444	0.213964328	0.004	0.01	FAM155A	protein_coding	family with s	13	107163510	107867496
ENSG0000020564	-5.974129459	0.004	0.01	HTN3	protein_coding	histatin 3 [S	4	70028455	70036538
ENSG0000025467	3.086364497	0.004	0.01	NA	NA	NA	NA	NA	NA
ENSG0000021525	-0.300398661	0.004	0.01	GOLGA8B	protein_coding	golgin A8 fa	15	34525207	34588503
ENSG0000009994	0.291278951	0.004	0.01	SNAP29	protein_coding	synaptosom	22	20859007	20891214
ENSG0000026899	1.305337371	0.004	0.01	MAN1B1-D	lncRNA	MAN1B1 div	9	137084946	137086817
ENSG0000013692	-0.288585656	0.004	0.01	TSTD2	protein_coding	thiosulfate s	9	97600080	97633368
ENSG0000026762	-0.998483317	0.004	0.01	AC138430.1	lncRNA	novel transc	19	19254748	19273369
ENSG0000017723	0.24718138	0.004	0.01	MAN1B1	protein_coding	mannosidas	9	137086985	137109183
ENSG0000015303	-0.274163639	0.004	0.01	SRP19	protein_coding	signal recog	5	112861188	112898371
ENSG0000026029	-1.141034884	0.004	0.01	AC095057.3	lncRNA	novel transc	4	40166675	40167831
ENSG0000011437	-2.19979197	0.004	0.01	USP9Y	protein_coding	ubiquitin sp	Y	12537650	12860839
ENSG0000016712	-0.650873565	0.004	0.01	CERCAM	protein_coding	cerebral enc	9	128411751	128437351
ENSG0000010570	0.473500011	0.004	0.01	ZNF14	protein_coding	zinc finger p	19	19710472	19733112
ENSG0000011713	0.260389375	0.004	0.01	RPF1	protein_coding	ribosome pr	1	84479259	84498352
ENSG0000019871	-0.292651038	0.004	0.01	TOGARAM1	protein_coding	TOG array r	14	44962190	45074431
ENSG0000025471	2.29899675	0.004	0.01	GLYATL1P2	transcribed_un	glycine-N-a	11	58878302	58893460
ENSG0000012456	0.224972526	0.004	0.01	SNRPC	protein_coding	small nuclea	6	34757505	34773857
ENSG0000011187	0.24947941	0.004	0.01	ASF1A	protein_coding	anti-silencir	6	118894152	118909171
ENSG0000015411	-0.212238495	0.004	0.01	TBCEL	protein_coding	tubulin foldi	11	121024072	121090775
ENSG0000023152	-1.029760623	0.004	0.01	FAM225A	lncRNA	family with s	9	113113042	113119928
ENSG0000023479	-0.924832056	0.004	0.01	DXO	protein_coding	decapping e	CHR_HSCHR	31962464	31964946
ENSG0000026647	-0.199667047	0.004	0.01	MRPS21	protein_coding	mitochondr	1	150293861	150308979
ENSG0000023056	1.907220338	0.004	0.01	AL121757.1	lncRNA	novel transc	20	5445838	5475483
ENSG0000014849	-0.258832917	0.004	0.01	PARD3	protein_coding	par-3 family	10	34109560	34815325
ENSG0000016300	-0.349610358	0.004	0.01	CFAP36	protein_coding	cilia and flag	2	55519604	55545079
ENSG0000026798	1.712851896	0.004	0.01	AC007292.1	lncRNA	novel transc	19	4363789	4364640
ENSG0000017479	-0.308437801	0.004	0.01	CEP135	protein_coding	centrosoma	4	55948871	56033361
ENSG0000012858	0.45242928	0.004	0.01	IFT22	protein_coding	intraflagella	7	101310914	101321823
ENSG0000025622	0.569602451	0.004	0.01	ZNF10	protein_coding	zinc finger p	12	133130575	133159465
ENSG0000016401	0.60894855	0.004	0.01	ZNF691	protein_coding	zinc finger p	1	42846573	42852477
ENSG0000009991	0.403893201	0.004	0.01	KLHL22	protein_coding	kelch like fa	22	20441519	20495844
ENSG0000016540	-2.553238814	0.004	0.01	TSHR	protein_coding	thyroid stim	14	80954989	81146302
ENSG0000028006	1.747635454	0.004	0.01	AC012676.5	TEC	tec	16	4346694	4348648
ENSG0000014459	-0.289011599	0.004	0.01	EAF1	protein_coding	ELL associat	3	15427598	15450635
ENSG0000018347	-1.196659602	0.004	0.01	TREX2	protein_coding	three prime	X	153444720	153470587
ENSG0000017382	-0.224381587	0.004	0.01	RNF213	protein_coding	ring finger p	17	80260866	80398786
ENSG0000022817	1.813943549	0.004	0.01	GEMIN8P4	processed_pse	gem nuclea	1	89993593	89994321
ENSG0000010513	-0.833711585	0.004	0.01	SYDE1	protein_coding	synapse def	19	15107401	15114985
ENSG0000011594	0.307143	0.004	0.01	PNO1	protein_coding	partner of N	2	68157888	68176238
ENSG0000021359	0.192500445	0.004	0.01	TMX2	protein_coding	thioredoxin	11	57712593	57740973
ENSG0000018888	-2.062506986	0.004	0.01	KLRG2	protein_coding	killer cell lec	7	139452690	139483673
ENSG0000023322	1.138128747	0.004	0.01	SSBP3P1	processed_pse	SSBP3 pseu	7	39833571	39834718
ENSG0000007915	0.231306782	0.004	0.01	FKBP7	protein_coding	FKBP prolyl	2	178463664	178478600
ENSG0000019702	0.439230218	0.004	0.01	EOLA2	protein_coding	endotheliun	X	149929527	149938700
ENSG0000010882	-0.152193133	0.004	0.01	LRRC59	protein_coding	leucine rich	17	50375059	50397523

ENSG0000013311	-0.250965596	0.004	0.01	RFC3	protein_coding	replication f	13	33818069	33966558
ENSG0000016243	-0.265818365	0.004	0.01	RAVER2	protein_coding	ribonucleop	1	64745095	64833232
ENSG0000006493	-0.237768568	0.004	0.01	PMS1	protein_coding	PMS1 homo	2	189784085	189877629
ENSG0000011042	0.221732685	0.004	0.01	HIPK3	protein_coding	homeodom	11	33256672	33357023
ENSG0000011006	0.28383481	0.004	0.01	PUS3	protein_coding	pseudouridi	11	125893485	125903221
ENSG0000013075	-0.50206545	0.004	0.01	MAP3K10	protein_coding	mitogen-ac	19	40191426	40215575
ENSG0000016969	-0.935089796	0.004	0.01	AGPAT2	protein_coding	1-acylglycer	9	136673143	136687457
ENSG0000010028	0.271101348	0.004	0.01	HMGXB4	protein_coding	HMG-box c	22	35257452	35295807
ENSG0000018096	-0.275243635	0.004	0.01	TCEAL8	protein_coding	transcriptior	X	103252995	103255192
ENSG0000012313	-0.212311992	0.004	0.01	PRDX4	protein_coding	peroxiredox	X	23664262	23686397
ENSG0000027676	3.770056504	0.004	0.01	AC069499.2	processed_pse	ribosomal p	3	108574683	108575064
ENSG0000021530	-0.111146561	0.005	0.01	DDX3X	protein_coding	DEAD-box I	X	41333348	41364472
ENSG0000010316	0.346759102	0.005	0.01	TAF1C	protein_coding	TATA-box b	16	84177847	84187070
ENSG0000018652	0.933608946	0.005	0.01	FAM86B1	protein_coding	family with s	8	12182096	12194133
ENSG0000025487	-1.521419843	0.005	0.01	AL845464.1	protein_coding	novel transc	CHR_HSCHR	32111446	32129586
ENSG0000020407	-0.427739209	0.005	0.01	SYS1	protein_coding	SYS1 golgi t	20	45361937	45376798
ENSG0000016710	-1.101093228	0.005	0.01	SAMD14	protein_coding	sterile alpha	17	50110040	50130160
ENSG0000010194	-0.326166396	0.005	0.01	WDR13	protein_coding	WD repeat d	X	48590042	48608869
ENSG0000016103	0.401397611	0.005	0.01	LRWD1	protein_coding	leucine rich	7	102464956	102473168
ENSG0000025933	-1.46004597	0.005	0.01	ST20-MTHI	protein_coding	ST20-MTHF	15	79845150	79923754
ENSG0000016771	-2.557237777	0.005	0.01	SERPINF2	protein_coding	serpin family	17	1742836	1755265
ENSG0000026995	0.700767427	0.005	0.01	AL049840.6	lncRNA	novel transc	14	103696353	103697163
ENSG0000017054	-1.091520754	0.005	0.01	SMAGP	protein_coding	small cell ac	12	51244558	51270415
ENSG0000012195	0.192560072	0.005	0.01	GPSM2	protein_coding	G protein si	1	108875350	108934545
ENSG0000025801	1.468980779	0.005	0.01	AC011603.3	lncRNA	novel transc	12	49127782	49188484
ENSG0000020646	1.117865484	0.005	0.01	GABBR1	protein_coding	gamma-am	CHR_HSCHR	29555423	29632960
ENSG0000018777	-1.11297729	0.005	0.01	DNAH17	protein_coding	dynein axor	17	78423697	78577396
ENSG0000008101	0.287248973	0.005	0.01	AP4E1	protein_coding	adaptor rela	15	50908672	51005895
ENSG0000014909	0.281508798	0.005	0.01	DGKZ	protein_coding	diacylglycer	11	46332905	46380554
ENSG0000012695	0.505615967	0.005	0.01	TIMM8A	protein_coding	translocase	X	101345661	101348742
ENSG0000012094	0.395249539	0.005	0.01	UBIAD1	protein_coding	UbiA prenyl	1	11273198	11296049
ENSG0000009933	1.001526484	0.005	0.01	OCEL1	protein_coding	occludin/ELI	19	17226213	17229219
ENSG0000027160	0.206520714	0.005	0.01	LIX1L	protein_coding	limb and CN	1	145933423	145958017
ENSG0000014590	-0.211514558	0.005	0.01	ZNF300	protein_coding	zinc finger p	5	150894392	150904983
ENSG0000013254	-0.216432266	0.005	0.01	VPS13B	protein_coding	vacuolar pro	8	99013266	99877580
ENSG0000027625	-1.397309905	0.005	0.01	AC009118.3	lncRNA	novel transc	16	58522970	58523842
ENSG0000021727	3.466025289	0.005	0.01	AL031777.1	processed_pse	ribosomal p	6	26202156	26202654
ENSG0000013693	-0.53284655	0.005	0.01	TRMO	protein_coding	tRNA methy	9	97904489	97922516
ENSG0000022965	1.044835469	0.005	0.01	ZNRD1ASP	lncRNA	zinc ribbon	CHR_HSCHR	29990518	30051536
ENSG0000024713	-1.287142829	0.005	0.01	AC090204.1	lncRNA	novel transc	8	32927913	33045445
ENSG0000026644	1.689186369	0.005	0.01	MSH5-SAP	protein_coding	MSH5-SAPC	CHR_HSCHR	31816303	31841147
ENSG0000025100	-2.704248865	0.005	0.01	ZFPM2-AS1	lncRNA	ZFPM2 antis	8	105546089	106060524
ENSG0000019753	0.264368652	0.005	0.01	MYO5A	protein_coding	myosin VA [	15	52307283	52529050
ENSG0000016996	-0.181607785	0.005	0.01	MAP3K2	protein_coding	mitogen-ac	2	127298730	127388465
ENSG0000027407	-1.818396877	0.005	0.01	TSEN34	protein_coding	tRNA splicir	CHR_HSCHR	54190866	54195141
ENSG0000020412	0.161996509	0.005	0.01	GIGYF2	protein_coding	GRB10 inter	2	232697299	232860605
ENSG0000018509	0.35780302	0.005	0.01	MANEAL	protein_coding	mannosidas	1	37793802	37801137
ENSG0000024168	-1.344257484	0.005	0.01	ADAMTS9-	lncRNA	ADAMTS9 a	3	64684909	65053439
ENSG0000006524	0.252901901	0.005	0.01	PKN2	protein_coding	protein kina	1	88684222	88836255
ENSG0000014893	-2.419806705	0.005	0.01	GAS2	protein_coding	growth arre	11	22625509	22813055
ENSG0000017704	-0.744434686	0.005	0.01	SIX5	protein_coding	SIX homeob	19	45764785	45769252
ENSG0000020634	-1.122840913	0.005	0.01	DXO	protein_coding	decapping e	CHR_HSCHR	31960187	31962669
ENSG0000009978	0.120760499	0.005	0.01	HNRNPM	protein_coding	heterogene	19	8444767	8489114
ENSG0000018198	-1.760797369	0.005	0.01	CCDC149	protein_coding	coiled-coil c	4	24806117	24980204
ENSG0000015871	0.393981608	0.005	0.01	RNF166	protein_coding	ring finger p	16	88696499	88706408
ENSG0000020521	-0.411434666	0.005	0.01	LGR4	protein_coding	leucine rich	11	27365961	27472790
ENSG0000015542	2.088991656	0.005	0.01	TRIM74	protein_coding	tripartite mo	7	72959485	72969466
ENSG0000017230	0.539302299	0.005	0.01	COPRS	protein_coding	coordinator	17	31851871	31859291

ENSG0000008551	-1.611467638	0.005	0.01	PILRA	protein_coding	paired immu	7	100367530	100400096
ENSG0000017281	-2.209472188	0.005	0.01	CYP7B1	protein_coding	cytochrome	8	64587763	64798737
ENSG0000023595	0.447461801	0.005	0.01	TTC28-AS1	lncRNA	TTC28 antis	22	27919376	28008581
ENSG0000016707	-0.481566402	0.005	0.01	TEF	protein_coding	TEF transcrip	22	41367333	41399326
ENSG0000015829	-0.915887783	0.005	0.01	GPR153	protein_coding	G protein-c	1	6247353	6261098
ENSG0000008084	0.252048526	0.005	0.01	DLGAP4	protein_coding	DLG associa	20	36306336	36528637
ENSG0000026630	-2.921094147	0.005	0.01	AC098850.3	protein_coding	novel transc	17	16690261	16804453
ENSG0000016032	0.787946222	0.005	0.01	ZNF208	protein_coding	zinc finger p	19	21932958	22010949
ENSG0000023339	0.712898185	0.005	0.01	LINC01719	lncRNA	long interge	1	146052566	146061948
ENSG0000017113	-0.244976925	0.005	0.01	PRKCE	protein_coding	protein kina	2	45651345	46187990
ENSG0000018563	-1.401265191	0.005	0.01	NDUFA4L2	protein_coding	NDUFA4 mi	12	57234903	57240715
ENSG0000014552	-0.433662777	0.005	0.01	CDH18	protein_coding	cadherin 18	5	19471296	20575873
ENSG0000009982	0.498921146	0.005	0.01	HCN2	protein_coding	hyperpolariz	19	589881	617159
ENSG0000012947	-0.293199522	0.005	0.01	BCL2L2	protein_coding	BCL2 like 2	14	23298790	23311751
ENSG0000028019	-1.556131957	0.005	0.01	AC132219.2	TEC	TEC	8	81696368	81698694
ENSG0000017539	-0.392866796	0.005	0.01	ZNF25	protein_coding	zinc finger p	10	37949573	37976647
ENSG0000016515	-0.297164629	0.005	0.01	PGAP4	protein_coding	post-GPI at	9	101473170	101533537
ENSG0000023625	-0.763092376	0.005	0.01	AC009404.1	lncRNA	novel transc	2	117833937	117841658
ENSG0000013675	0.241702906	0.005	0.01	ABI1	protein_coding	abl interacto	10	26746593	26861087
ENSG0000002936	0.175719439	0.005	0.01	SLC39A9	protein_coding	solute carrie	14	69398015	69462390
ENSG0000016393	-1.943326866	0.005	0.01	PRKCD	protein_coding	protein kina	3	53156009	53192717
ENSG0000017032	0.236967132	0.005	0.01	NFRKB	protein_coding	nuclear facto	11	129863636	129895590
ENSG0000016126	-1.003322782	0.005	0.01	U2AF1L4	protein_coding	U2 small nu	19	35742464	35745445
ENSG0000012545	0.27604283	0.005	0.01	NUP85	protein_coding	nucleoporin	17	75205659	75235758
ENSG0000025730	-1.612227645	0.005	0.01	FAHD2P1	processed_pse	fumarylacet	12	70671918	70672856
ENSG0000014682	0.518822652	0.005	0.01	MAP11	protein_coding	microtubule	7	100154420	100158723
ENSG0000014839	0.35866571	0.005	0.01	DPH7	protein_coding	diphthamide	9	137554444	137578925
ENSG0000016643	0.512643677	0.005	0.01	XRRA1	protein_coding	X-ray radiat	11	74807739	74949200
ENSG0000027157	1.087309046	0.005	0.01	AL359504.1	lncRNA	novel transc	1	84076331	84077931
ENSG0000018666	0.64497925	0.005	0.01	C17orf58	protein_coding	chromosom	17	67991101	67996431
ENSG0000017160	0.149883805	0.005	0.01	CLSTN1	protein_coding	calsyntenin	1	9728926	9823984
ENSG0000006835	-0.405484268	0.005	0.01	TBC1D25	protein_coding	TBC1 doma	X	48539714	48562609
ENSG0000024626	0.893432161	0.005	0.01	UBR5-AS1	lncRNA	UBR5 antise	8	102239394	102253750
ENSG0000013361	0.552920516	0.005	0.01	KRBA1	protein_coding	KRAB-A dor	7	149714781	149734575
ENSG0000011984	-0.241036678	0.005	0.01	AFTPH	protein_coding	aftiphilin [So	2	64524305	64593005
ENSG0000027275	1.701200823	0.005	0.01	AC083798.2	lncRNA	novel transc	3	122416207	122443180
ENSG0000013425	0.26265282	0.005	0.01	CEPT1	protein_coding	choline/etha	1	111139627	111185104
ENSG0000016878	0.297961063	0.005	0.01	PPIP5K1	protein_coding	diphosphoir	15	43533462	43590253
ENSG0000013433	-0.318169569	0.005	0.01	LDHA	protein_coding	lactate dehy	11	18394560	18408425
ENSG0000006013	-1.304803929	0.005	0.01	YBX3	protein_coding	Y-box bindi	12	10699089	10723323
ENSG0000019855	1.672049512	0.005	0.01	ZNF789	protein_coding	zinc finger p	7	99472890	99503650
ENSG0000007523	-0.385273716	0.005	0.01	TTC38	protein_coding	tetratricope	22	46267961	46294008
ENSG0000026161	-1.075573111	0.005	0.01	AC036108.3	lncRNA	novel transc	15	99139317	99145370
ENSG0000027618	0.781400039	0.005	0.01	ERMARD	protein_coding	ER membra	CHR_HSCHR	169751622	169781584
ENSG0000019637	-0.362943614	0.005	0.01	ASB13	protein_coding	ankyrin repe	10	5638867	5666595
ENSG0000009491	-0.258163667	0.005	0.01	AAAS	protein_coding	aladin WD r	12	53307456	53324864
ENSG0000011307	0.789479329	0.005	0.01	HBEGF	protein_coding	heparin binc	5	140332843	140346603
ENSG0000004899	0.179114349	0.005	0.01	R3HDM1	protein_coding	R3H domair	2	135531455	135725270
ENSG0000020615	-4.228420831	0.005	0.01	GYG2P1	transcribed_un	glycogenin	Y	12088010	12421587
ENSG0000013690	0.324943188	0.005	0.01	DPM2	protein_coding	dolichyl-pho	9	127935099	127937854
ENSG0000017546	3.086236883	0.005	0.01	TBC1D10C	protein_coding	TBC1 doma	11	67403915	67410089
ENSG0000004764	-0.339620252	0.005	0.01	WWC3	protein_coding	WWC family	X	10015254	10144474
ENSG0000015589	0.243712224	0.005	0.01	PXYLP1	protein_coding	2-phospho>	3	141228726	141367753
ENSG0000010066	0.123224869	0.005	0.01	EIF5	protein_coding	eukaryotic t	14	103333544	103345025
ENSG0000000720	0.171953622	0.005	0.01	KIAA0100	protein_coding	KIAA0100 [S	17	28614446	28645454
ENSG0000023529	-0.415511236	0.005	0.01	PPP1R10	protein_coding	protein pho	CHR_HSCHR	30678854	30697067
ENSG0000001298	-0.1775308	0.005	0.01	MAP4K5	protein_coding	mitogen-ac	14	50418501	50561126
ENSG0000012674	0.335250702	0.005	0.01	EMG1	protein_coding	EMG1 N1-s	12	6970913	6997428

ENSG0000022376	0.790046503	0.005	0.01	LINC00205	lncRNA	long interge	21	45288050	45297806
ENSG0000017314	-0.492017247	0.005	0.01	MRPL57	protein_coding	mitochondr	13	21176658	21179084
ENSG0000014768	0.277848465	0.005	0.01	TATDN1	protein_coding	TatD DNase	8	124488485	124539458
ENSG0000026823	1.266132501	0.005	0.01	AC012313.1	lncRNA	novel transc	19	58346854	58362751
ENSG0000013445	0.198420511	0.005	0.01	RBM17	protein_coding	RNA binding	10	6089034	6117457
ENSG0000015184	0.284113578	0.005	0.01	CENPJ	protein_coding	centromere	13	24882279	24922889
ENSG0000023163	-0.791035307	0.005	0.01	NA	NA	NA	NA	NA	NA
ENSG0000011955	0.410331941	0.005	0.01	C19orf25	protein_coding	chromosom	19	1461143	1479219
ENSG0000012248	0.554130512	0.005	0.01	RWDD3	protein_coding	RWD domai	1	95234210	95247225
ENSG0000022664	2.62849383	0.005	0.01	PLCG1-AS1	lncRNA	PLCG1 antis	20	41098019	41138003
ENSG0000010975	2.205666116	0.005	0.01	HGFAC	protein_coding	HGF activat	4	3441968	3449486
ENSG0000020648	0.422033148	0.005	0.01	PPP1R18	protein_coding	protein pho	CHR_HSCHR	30665910	30677416
ENSG0000027381	-1.279014341	0.005	0.01	OCLN	protein_coding	occludin [Sc	CHR_HSCHR	69492007	69556424
ENSG0000000607	-1.361484503	0.005	0.01	ABCC8	protein_coding	ATP binding	11	17392498	17476879
ENSG0000027233	-0.592679936	0.005	0.01	AC093297.2	lncRNA	novel transc	5	44826076	44828592
ENSG0000016951	0.335616421	0.005	0.01	METTL15	protein_coding	methyltrans	11	28108248	28527041
ENSG0000018501	0.472961091	0.005	0.01	UBOX5	protein_coding	U-box dom	20	3107573	3160196
ENSG0000011467	-1.799463561	0.005	0.01	NEK11	protein_coding	NIMA relate	3	131026850	131350465
ENSG0000016690	0.321726506	0.005	0.01	MRPL16	protein_coding	mitochondr	11	59806140	59810778
ENSG0000019615	0.577203532	0.005	0.01	ZNF79	protein_coding	zinc finger p	9	127424374	127445372
ENSG0000013025	-0.182404138	0.005	0.01	SAFB2	protein_coding	scaffold atta	19	5586999	5624046
ENSG0000016849	-1.866716511	0.005	0.01	PHYHIP	protein_coding	phytanoyl-C	8	22219703	22232101
ENSG0000021482	1.039433494	0.005	0.01	MTCP1	protein_coding	mature T ce	X	155064034	155147937
ENSG0000010304	0.287765005	0.005	0.01	VAC14	protein_coding	VAC14 com	16	70687439	70801160
ENSG0000013846	0.46905487	0.005	0.01	SLC49A4	protein_coding	solute carrie	3	122795069	122881139
ENSG0000016937	-0.311789149	0.005	0.01	ARL13B	protein_coding	ADP ribosyl	3	93980139	94055678
ENSG0000006642	0.299474166	0.005	0.01	ATXN3	protein_coding	ataxin 3 [So	14	92044496	92106621
ENSG0000018876	-1.888816861	0.005	0.01	SPRED3	protein_coding	sprouty rela	19	38388421	38399587
ENSG0000012287	-0.282629642	0.005	0.01	CISD1	protein_coding	CDGSH iron	10	58269162	58289586
ENSG0000010552	0.394527364	0.005	0.01	PLPPR2	protein_coding	phospholipi	19	11355386	11365698
ENSG0000027063	2.362243169	0.005	0.01	AL023806.1	lncRNA	novel transc	6	145735570	145737218
ENSG0000025440	1.535441177	0.005	0.01	MSH5-SAP	protein_coding	MSH5-SAP(	CHR_HSCHR	31727419	31752228
ENSG0000010564	-1.56120525	0.005	0.01	KCNN1	protein_coding	potassium c	19	17951293	18000080
ENSG0000023124	0.430743808	0.005	0.01	PPP1R18	protein_coding	protein pho	CHR_HSCHR	30666252	30677758
ENSG0000018904	-1.790023809	0.005	0.01	ANKDD1B	protein_coding	ankyrin repe	5	75611182	75681773
ENSG0000018289	-0.365059857	0.005	0.01	RPL35A	protein_coding	ribosomal p	3	197950190	197956610
ENSG0000010292	-0.223348316	0.005	0.01	N4BP1	protein_coding	NEDD4 binc	16	48538726	48620148
ENSG0000010785	0.168605076	0.005	0.01	TNKS2	protein_coding	tankyrase 2	10	91798426	91865475
ENSG0000016199	0.273751284	0.005	0.01	JMJD8	protein_coding	jumonji don	16	681670	684528
ENSG0000011373	-0.563798705	0.005	0.01	BNIP1	protein_coding	BCL2 intera	5	173144442	173164387
ENSG0000005370	1.043059632	0.005	0.01	NRIP2	protein_coding	nuclear rece	12	2825348	2835544
ENSG0000015169	-0.182850227	0.005	0.01	ADAM17	protein_coding	ADAM meta	2	9488486	9556732
ENSG0000019874	-0.452877442	0.005	0.01	GPATCH3	protein_coding	G-patch do	1	26890488	26900467
ENSG0000010399	0.16632165	0.005	0.01	ZNF106	protein_coding	zinc finger p	15	42412823	42491141
ENSG0000010967	0.284319656	0.005	0.01	FBXW7	protein_coding	F-box and V	4	152320544	152536092
ENSG0000023676	-0.762345476	0.005	0.01	DXO	protein_coding	decapping e	CHR_HSCHR	31951992	31954474
ENSG0000020495	0.443346754	0.005	0.01	C12orf73	protein_coding	chromosom	12	103940763	103965708
ENSG0000016191	-0.79792874	0.005	0.01	ZNF653	protein_coding	zinc finger p	19	11483427	11505839
ENSG0000018077	0.274481441	0.005	0.01	ZDHHC20	protein_coding	zinc finger D	13	21372571	21459370
ENSG0000013875	0.254070627	0.005	0.01	NUP54	protein_coding	nucleoporin	4	76114659	76148444
ENSG0000013937	-0.53525274	0.005	0.01	SLC15A4	protein_coding	solute carrie	12	128793194	128823958
ENSG0000018290	-0.351563056	0.006	0.01	ZNF721	protein_coding	zinc finger p	4	425815	499156
ENSG0000028016	1.739938904	0.006	0.01	AC022413.1	TEC	TEC	5	180810401	180811384
ENSG0000019893	0.347300998	0.006	0.01	APRT	protein_coding	adenine phc	16	88809339	88811937
ENSG0000011893	0.440629445	0.006	0.01	UCHL3	protein_coding	ubiquitin C-	13	75549480	75606020
ENSG0000013260	0.349202805	0.006	0.01	PRMT7	protein_coding	protein argi	16	68310974	68358584
ENSG0000010579	0.29917248	0.006	0.01	GTPBP10	protein_coding	GTP binding	7	90335223	90391453
ENSG0000013281	0.458835641	0.006	0.01	RBM38	protein_coding	RNA binding	20	57391396	57409333

ENSG0000014894	0.235157823	0.006	0.01	LIN7C	protein_coding	lin-7 homol	11	27494418	27506769
ENSG0000014848	0.246876016	0.006	0.01	RSU1	protein_coding	Ras suppres	10	16590611	16817463
ENSG0000013401	0.236883379	0.006	0.01	ELP3	protein_coding	elongator a	8	28089673	28191156
ENSG0000027678	0.305999702	0.006	0.01	FAN1	protein_coding	FANCD2 an	CHR_HSCHR	31055318	31094547
ENSG0000027641	-2.433252626	0.006	0.01	AC036214.3	protein_coding	novel protei	8	79918860	80080775
ENSG0000023084	-2.589782731	0.006	0.01	OCLNP1	unprocessed_p	OCLN pseud	5	71074225	71093193
ENSG0000025114	0.859821881	0.006	0.01	AP002490.1	lncRNA	novel transc	11	72014291	72020910
ENSG0000028005	1.874465272	0.006	0.01	AL022069.2	TEC	TEC	6	166388136	166389920
ENSG0000016020	-1.534117612	0.006	0.01	HSF2BP	protein_coding	heat shock t	21	43529186	43659488
ENSG0000022826	2.137275021	0.006	0.01	NA	NA	NA	NA	NA	NA
ENSG0000017423	-0.145255021	0.006	0.01	PRPF8	protein_coding	pre-mRNA	17	1650629	1684867
ENSG0000016620	-0.155995705	0.006	0.02	COPS2	protein_coding	COP9 signa	15	49106068	49155661
ENSG0000011581	0.15435869	0.006	0.02	CEBPZ	protein_coding	CCAAT enha	2	37201612	37231596
ENSG0000016370	-0.925147814	0.006	0.02	IL17RC	protein_coding	interleukin 1	3	9917074	9933630
ENSG0000003704	0.427126491	0.006	0.02	TUBG2	protein_coding	tubulin gam	17	42659284	42667006
ENSG0000027008	0.405242645	0.006	0.02	NA	NA	NA	NA	NA	NA
ENSG0000018930	-0.241492093	0.006	0.02	LIN54	protein_coding	lin-54 DREA	4	82909973	83012926
ENSG0000020619	-0.241589115	0.006	0.02	DUXAP8	lncRNA	double hom	22	15784959	15829984
ENSG0000011989	-0.465513547	0.006	0.02	SLC17A5	protein_coding	solute carrie	6	73593379	73653992
ENSG0000006282	0.360136126	0.006	0.02	POLD1	protein_coding	DNA polym	19	50384204	50418018
ENSG0000011311	0.580536237	0.006	0.02	TMCO6	protein_coding	transmembr	5	140639435	140645408
ENSG0000014809	-0.491063562	0.006	0.02	AUH	protein_coding	AU RNA bin	9	91213815	91361918
ENSG0000024549	1.354428688	0.006	0.02	AP000866.1	lncRNA	novel transc	11	124800424	124834487
ENSG0000012460	0.364357565	0.006	0.02	AARS2	protein_coding	alanyl-tRNA	6	44298731	44313347
ENSG0000009995	-0.681663415	0.006	0.02	MMP11	protein_coding	matrix meta	22	23768226	23784316
ENSG0000021356	-0.37007213	0.006	0.02	C8orf82	protein_coding	chromosom	8	144525733	144529132
ENSG0000026063	0.866526723	0.006	0.02	SNAI3-AS1	lncRNA	SNAI3 antis	16	88663298	88687278
ENSG0000014471	-0.570961703	0.006	0.02	IQSEC1	protein_coding	IQ motif and	3	12897043	13283281
ENSG0000018565	-0.172683964	0.006	0.02	BRWD1	protein_coding	bromodoma	21	39184176	39321559
ENSG0000016354	0.233795512	0.006	0.02	SUCLG1	protein_coding	succinate-C	2	84423528	84460045
ENSG0000018291	-0.252078425	0.006	0.02	C11orf54	protein_coding	chromosom	11	93741591	93764749
ENSG0000018352	0.301949727	0.006	0.02	PSMG1	protein_coding	proteasome	21	39174769	39183488
ENSG0000019687	0.267213281	0.006	0.02	SCN8A	protein_coding	sodium volt	12	51590266	51812864
ENSG0000027394	-1.09853439	0.006	0.02	CNOT3	protein_coding	CCR4-NOT	CHR_HSCHR	54138182	54156191
ENSG0000025736	-0.425994966	0.006	0.02	FNTB	protein_coding	farnesyltran	14	64986895	65062652
ENSG0000027460	1.099252278	0.006	0.02	PCCA-DT	lncRNA	PCCA diverg	13	100053074	100088950
ENSG0000025867	1.251263683	0.006	0.02	AC011448.1	protein_coding	novel protei	19	19516227	19536076
ENSG0000026258	2.47540474	0.006	0.02	AC133552.2	transcribed_un	leucine carb	16	25031744	25058109
ENSG0000007848	-1.67032282	0.006	0.02	ZCWPW1	protein_coding	zinc finger C	7	100400826	100428992
ENSG0000014712	-0.243395208	0.006	0.02	NDUFB11	protein_coding	NADH:ubiqu	X	47142216	47145038
ENSG0000027067	2.666961369	0.006	0.02	YTHDF3-AS	lncRNA	YTHDF3 ant	8	63167725	63168442
ENSG0000007460	0.188085103	0.006	0.02	DPP8	protein_coding	dipeptidyl p	15	65442463	65517704
ENSG0000020462	1.146901334	0.006	0.02	ZNRD1ASP	transcribed_un	zinc ribbon	6	30001011	30061640
ENSG0000013109	1.421773226	0.006	0.02	C1QL1	protein_coding	complemen	17	44959693	44968303
ENSG0000026065	2.152121679	0.006	0.02	NA	NA	NA	NA	NA	NA
ENSG0000014591	-0.187374257	0.006	0.02	NHP2	protein_coding	NHP2 ribon	5	178149463	178153894
ENSG0000018538	0.679658868	0.006	0.02	MAPK11	protein_coding	mitogen-ac	22	50263713	50270767
ENSG0000027412	-1.091289528	0.006	0.02	TSEN34	protein_coding	tRNA splicir	CHR_HSCHR	54190535	54195132
ENSG0000013572	0.968279479	0.006	0.02	FBXL8	protein_coding	F-box and l	16	67159932	67164570
ENSG0000014318	0.164966206	0.006	0.02	TMCO1	protein_coding	transmembr	1	165724293	165827755
ENSG0000027549	0.560052438	0.006	0.02	CU633906.1	lncRNA	uncharacter	21	6228966	6267317
ENSG0000026094	-0.903077436	0.006	0.02	AL390195.2	lncRNA	novel transc	1	111431046	111433068
ENSG0000023107	-0.589471025	0.006	0.02	HCG18	lncRNA	HLA comple	6	30286690	30327382
ENSG0000027956	1.003246847	0.006	0.02	AL845472.1	TEC	uncharacter	9	41269916	41273997
ENSG0000009537	-0.671414478	0.006	0.02	SH2D3C	protein_coding	SH2 domair	9	127738317	127778710
ENSG0000012722	-0.655152932	0.006	0.02	ABHD8	protein_coding	abhydrolase	19	17292131	17310236
ENSG0000010203	-1.056537317	0.006	0.02	RENBP	protein_coding	renin bindin	X	153935269	153944687
ENSG0000026180	-1.901329995	0.006	0.02	LOXL1-AS1	lncRNA	LOXL1 antis	15	73908071	73928248

ENSG0000025468	-1.080808435	0.006	0.02	AP002387.1	lncRNA	novel transc		11	71448674	71452157
ENSG0000025809	-2.922590602	0.006	0.02	AC025031.2	lncRNA	novel transc		12	46371463	46373778
ENSG0000016623	0.162466834	0.006	0.02	ARIH1	protein_coding	ariadne RBR		15	72474330	72602987
ENSG0000013876	-0.209087059	0.006	0.02	CCNG2	protein_coding	cyclin G2 [S		4	77157207	77433388
ENSG0000009061	0.257699255	0.006	0.02	ZNF268	protein_coding	zinc finger p		12	133181409	133214832
ENSG0000013331	-0.373824282	0.006	0.02	MACROD1	protein_coding	mono-ADP		11	63998558	64166113
ENSG0000024927	-1.432644972	0.006	0.02	PDLIM1P4	processed_pse	PDZ and LIM		3	98782188	98783193
ENSG0000023983	-5.845625125	0.006	0.02	DEFA3	protein_coding	defensin alp		8	7015869	7018297
ENSG0000019776	0.436939901	0.006	0.02	TXNRD3	protein_coding	thioredoxin		3	126607059	126655124
ENSG0000010945	-0.230549483	0.006	0.02	GAB1	protein_coding	GRB2 associ		4	143336762	143474568
ENSG0000018631	0.681577075	0.006	0.02	PRELID2	protein_coding	PRELI doma		5	145471799	145835369
ENSG0000024968	-1.748656834	0.006	0.02	AC079921.2	lncRNA	novel transc		4	39133913	39135608
ENSG0000027471	0.888492655	0.006	0.02	AL136964.1	lncRNA	novel transc		13	107870383	107873372
ENSG0000013022	-0.219184205	0.006	0.02	LRCH2	protein_coding	leucine rich	X		115110616	115234096
ENSG0000014156	-0.404943349	0.006	0.02	TRIM65	protein_coding	tripartite mo		17	75880335	75896951
ENSG0000023392	-0.14987586	0.006	0.02	RPS28	protein_coding	ribosomal p		19	8321158	8323340
ENSG0000020459	-1.120941182	0.006	0.02	TRIM39	protein_coding	tripartite mo		6	30326479	30343729
ENSG0000022381	-1.820782341	0.006	0.02	AC073365.1	lncRNA	novel transc		3	191425493	191591097
ENSG0000027571	1.550819179	0.006	0.02	H2BC9	protein_coding	H2B clustere		6	26251651	26253710
ENSG0000009219	-0.093822184	0.006	0.02	HNRNPC	protein_coding	heterogene		14	21209136	21269494
ENSG0000010184	-0.262025157	0.006	0.02	TBL1X	protein_coding	transducin b	X		9463295	9741037
ENSG0000009026	0.304779387	0.006	0.02	MRPS33	protein_coding	mitochondr		7	141002610	141015228
ENSG0000016937	0.213240326	0.006	0.02	SIN3A	protein_coding	SIN3 transcr		15	75369379	75455842
ENSG0000025042	0.788078185	0.006	0.02	AACSP1	transcribed_un	acetoacetyl-		5	178764861	178818435
ENSG0000012032	0.504774753	0.006	0.02	PCDHB10	protein_coding	protocadhe		5	141192353	141195647
ENSG0000011582	-0.133517044	0.006	0.02	PRKD3	protein_coding	protein kina		2	37250502	37324833
ENSG0000013260	0.242497746	0.006	0.02	TERF2	protein_coding	telomeric re		16	69355567	69408571
ENSG0000022634	-3.230875686	0.006	0.02	USP8P2	processed_pse	USP8 pseud		2	148872253	148872797
ENSG0000017520	-3.268040288	0.006	0.02	NPPA	protein_coding	natriuretic p		1	11845709	11848345
ENSG0000017193	-2.234653682	0.006	0.02	FBXW10	protein_coding	F-box and W		17	18744026	18779349
ENSG0000015735	0.258402093	0.006	0.02	ST3GAL2	protein_coding	ST3 beta-ga		16	70375977	70439237
ENSG0000016682	-0.303403598	0.006	0.02	TMEM170A	protein_coding	transmembr		16	75443054	75465497
ENSG0000023496	0.649610804	0.006	0.02	FABP5P7	processed_pse	fatty acid bi		11	59781318	59781722
ENSG0000013656	0.312291943	0.006	0.02	TANK	protein_coding	TRAF family		2	161136908	161236230
ENSG0000019615	0.314229012	0.006	0.02	ZNF250	protein_coding	zinc finger p		8	144876497	144902168
ENSG0000026028	0.51386529	0.006	0.02	SLX1B-SUL	lncRNA	SLX1B-SULT		16	29455105	29464963
ENSG0000022675	1.382204485	0.006	0.02	AL606760.1	lncRNA	novel transc		1	53238550	53246482
ENSG0000015071	0.279501971	0.006	0.02	MTMR12	protein_coding	myotubulari		5	32226994	32312987
ENSG0000019610	1.552208461	0.006	0.02	ZNF676	protein_coding	zinc finger p		19	22179089	22215801
ENSG0000017710	-0.7304753	0.006	0.02	ZDHHC22	protein_coding	zinc finger D		14	77131270	77142734
ENSG0000015841	0.402399025	0.006	0.02	MITD1	protein_coding	microtubule		2	99161427	99181058
ENSG0000012565	-0.843755361	0.006	0.02	PSPN	protein_coding	persephin [S		19	6375148	6379058
ENSG0000010663	0.353055343	0.006	0.02	TBL2	protein_coding	transducin b		7	73567537	73578791
ENSG0000016634	-0.183447415	0.006	0.02	DCHS1	protein_coding	dachsous ca		11	6621330	6655809
ENSG0000014196	-0.201229617	0.006	0.02	FEM1A	protein_coding	fem-1 homo		19	4791734	4801273
ENSG0000025550	1.225026183	0.007	0.02	AP002990.1	protein_coding	novel protei		11	62559603	62591531
ENSG0000017200	1.201572074	0.007	0.02	ZNF556	protein_coding	zinc finger p		19	2867335	2883445
ENSG0000009934	-0.200202586	0.007	0.02	PSMD8	protein_coding	proteasome		19	38374536	38383824
ENSG0000021519	0.523191257	0.007	0.02	LINC00680	transcribed_un	long interge		6	57946074	57961501
ENSG0000010303	-0.379900837	0.007	0.02	SETD6	protein_coding	SET domain		16	58515479	58523842
ENSG0000017985	-1.098655622	0.007	0.02	RNF227	protein_coding	ring finger p		17	7913324	7916276
ENSG0000015574	1.387844621	0.007	0.02	FLACC1	protein_coding	flagellum as		2	201288271	201357398
ENSG0000015409	-1.550425262	0.007	0.02	DNAAF1	protein_coding	dynein axor		16	84145287	84178767
ENSG0000000510	-0.244079889	0.007	0.02	DHX33	protein_coding	DEAH-box h		17	5440917	5468982
ENSG0000005212	-0.289038138	0.007	0.02	PLEKHA5	protein_coding	pleckstrin ho		12	19129752	19376400
ENSG0000023342	-1.962875014	0.007	0.02	AL009181.1	lncRNA	novel transc		1	28870483	28877336
ENSG0000024092	1.085227384	0.007	0.02	NA	NA	NA	NA	NA	NA	
ENSG0000018718	-0.876826527	0.007	0.02	ZNF546	protein_coding	zinc finger p		19	39984134	40021038

ENSG0000025483	3.764860916	0.007	0.02	AP001893.3	lncRNA	novel transc	11	126160714	126176035
ENSG0000013066	-0.35415016	0.007	0.02	PAK4	protein_coding	p21 (RAC1)	19	39125770	39182816
ENSG0000026573	-1.195025545	0.007	0.02	RN7SL5P	misc_RNA	RNA, 7SL, cy	9	9442060	9442380
ENSG0000017990	0.747527339	0.007	0.02	ZNF154	protein_coding	zinc finger p	19	57697367	57709194
ENSG0000015687	-0.286614549	0.007	0.02	SASS6	protein_coding	SAS-6 centr	1	100083563	100132955
ENSG0000015596	-0.894971386	0.007	0.02	AFF2	protein_coding	AF4/FMR2 f	X	148500617	149000663
ENSG0000021805	-5.094877672	0.007	0.02	ADAMTS7P	transcribed_un	ADAMTS7 p	15	85255369	85330334
ENSG0000011438	-0.493600252	0.007	0.02	NPRL2	protein_coding	NPR2 like, G	3	50347330	50350826
ENSG0000021571	0.252745153	0.007	0.02	TMEM167B	protein_coding	transmembr	1	109090764	109096934
ENSG0000015597	0.290372312	0.007	0.02	MICU3	protein_coding	mitochondr	8	17027238	17122642
ENSG0000016120	0.243334661	0.007	0.02	ABCF3	protein_coding	ATP binding	3	184186023	184194012
ENSG0000022725	1.928198481	0.007	0.02	AC012063.1	lncRNA	novel transc	2	236910797	237085838
ENSG0000025483	0.754603168	0.007	0.02	AP001372.2	lncRNA	uncharacter	11	74493366	74498533
ENSG0000022905	-3.789201859	0.007	0.02	HECW2-AS	lncRNA	HECW2 anti	2	196260024	196264204
ENSG0000022631	1.201978028	0.007	0.02	ZNF192P1	transcribed_un	zinc finger p	6	28161769	28169594
ENSG0000017114	-0.325690105	0.007	0.02	TADA3	protein_coding	transcription	3	9779860	9793011
ENSG0000018644	0.321859536	0.007	0.02	ZNF197	protein_coding	zinc finger p	3	44584888	44648471
ENSG0000008905	-0.323264061	0.007	0.02	RBBP9	protein_coding	RB binding p	20	18486540	18497225
ENSG0000009114	0.176062654	0.007	0.02	DLD	protein_coding	dihydrolipoa	7	107891162	107931730
ENSG0000015603	-0.225807416	0.007	0.02	MIDEAS	protein_coding	mitotic deac	14	73715122	73790285
ENSG0000027570	4.171282231	0.007	0.02	AC090527.4	lncRNA	novel transc	15	45585757	45586304
ENSG0000001100	0.310828559	0.007	0.02	LYPLA2	protein_coding	lysophosphо	1	23791145	23795539
ENSG0000016607	-0.493007411	0.007	0.02	GPR176	protein_coding	G protein-c	15	39799008	39920266
ENSG0000017348	-0.38030815	0.007	0.02	FKBP2	protein_coding	FKBP prolyl	11	64241003	64244132
ENSG0000017705	-0.712141526	0.007	0.02	FBXO46	protein_coding	F-box prote	19	45710629	45730896
ENSG0000027017	1.961369563	0.007	0.02	AC104109.2	lncRNA	novel transc	5	134226410	134227827
ENSG0000011950	-0.371715432	0.007	0.02	INVS	protein_coding	inversin [Sou	9	100099243	100302175
ENSG0000016461	-0.432411135	0.007	0.02	RP9	protein_coding	RP9 pre-mR	7	33094797	33109404
ENSG0000013574	-1.152803865	0.007	0.02	SLC9A5	protein_coding	solute carrie	16	67237683	67272191
ENSG0000027294	-1.016499653	0.007	0.02	NA	NA	NA	NA	NA	NA
ENSG0000021496	-1.621275785	0.007	0.02	CRPPA	protein_coding	CDP-L-ribit	7	16087525	16502504
ENSG0000007218	-1.184221753	0.007	0.02	ASIC4	protein_coding	acid sensing	2	219514170	219538772
ENSG0000025466	-2.03415147	0.007	0.02	NA	NA	NA	NA	NA	NA
ENSG0000010660	0.271971813	0.007	0.02	COA1	protein_coding	cytochrome	7	43608456	43729717
ENSG0000026094	0.897033213	0.007	0.02	CAPN10-D	lncRNA	CAPN10 div	2	240582700	240586699
ENSG0000019777	0.165403854	0.007	0.02	MCMBP	protein_coding	minichromo	10	119829404	119892556
ENSG0000018347	-0.30542345	0.007	0.02	ASB7	protein_coding	ankyrin repe	15	100602589	100651701
ENSG0000026113	1.361544936	0.007	0.02	AC023908.3	lncRNA	novel transc	15	39782571	39785617
ENSG0000025654	-2.083129905	0.007	0.02	AC156455.1	lncRNA	novel transc	12	122063306	122068616
ENSG0000013339	0.338127989	0.007	0.02	CEP20	protein_coding	centrosoma	16	15865719	15888625
ENSG0000012695	-0.649565318	0.007	0.02	TMEM35A	protein_coding	transmembr	X	101078879	101096367
ENSG0000020641	-0.733306363	0.007	0.02	GNL1	protein_coding	G protein nu	CHR_HSCHR	30605288	30617469
ENSG0000017897	-1.550310201	0.007	0.02	LINC00324	lncRNA	long interge	17	8220642	8224043
ENSG0000014332	-0.120029054	0.007	0.02	XPR1	protein_coding	xenotropic a	1	180632022	180890279
ENSG0000019877	-1.725623266	0.007	0.02	RCSD1	protein_coding	RCSD doma	1	167630093	167708696
ENSG0000027747	-0.867908929	0.007	0.02	AC005332.5	lncRNA	novel transc	17	68133201	68135935
ENSG0000010010	-0.780161559	0.007	0.02	PIK3IP1	protein_coding	phosphoino	22	31281594	31292534
ENSG0000010778	0.256219401	0.007	0.02	MINPP1	protein_coding	multiple ino	10	87504875	87553461
ENSG0000022719	0.837428865	0.007	0.02	ST7-AS1	lncRNA	ST7 antisens	7	116952446	116954334
ENSG0000014144	-0.456848548	0.007	0.02	GAREM1	protein_coding	GRB2 associ	18	32124877	32470882
ENSG0000020553	0.661210829	0.007	0.02	NAP1L4	protein_coding	nucleosome	11	2944431	2992377
ENSG0000021363	-0.767053101	0.007	0.02	ADAT3	protein_coding	adenosine d	19	1905399	1913447
ENSG0000000497	0.280603295	0.007	0.02	DVL2	protein_coding	dishevelled	17	7225342	7234517
ENSG0000007360	0.793846822	0.007	0.02	GSDMB	protein_coding	gasdermin B	17	39904595	39919854
ENSG0000025383	1.34946771	0.007	0.02	AC090197.1	lncRNA	novel transc	8	23336171	23366125
ENSG0000000645	-0.196058583	0.007	0.02	RALA	protein_coding	RAS like pro	7	39623565	39708120
ENSG0000021368	1.124204617	0.007	0.02	TREX1	protein_coding	three prime	3	48465811	48467645
ENSG0000010810	0.214193329	0.007	0.02	CCNY	protein_coding	cyclin Y [Sou	10	35247025	35572669

ENSG0000020443	0.648466894	0.007	0.02	CSNK2B	protein_coding	casein kinas	6	31665236	31670343
ENSG0000010003	-0.657947283	0.007	0.02	SLC35E4	protein_coding	solute carrie	22	30635781	30669016
ENSG0000010013	-0.18566053	0.007	0.02	SNU13	protein_coding	small nuclea	22	41673933	41690504
ENSG0000020496	1.051978589	0.007	0.02	PCDHA9	protein_coding	protocadhe	5	140847772	141012347
ENSG0000013582	0.115010046	0.007	0.02	DHX9	protein_coding	DExH-box h	1	182839347	182887982
ENSG0000020492	0.55529628	0.007	0.02	UQCC3	protein_coding	ubiquinol-c	11	62670273	62673686
ENSG0000007665	-0.448871905	0.007	0.02	GPATCH1	protein_coding	G-patch do	19	33080899	33130542
ENSG0000010488	1.228446517	0.007	0.02	PEX11G	protein_coding	peroxisoma	19	7476875	7497449
ENSG0000016020	-0.188548517	0.007	0.02	RRP1B	protein_coding	ribosomal R	21	43659560	43696079
ENSG0000007896	-0.387889667	0.007	0.02	UBE2D4	protein_coding	ubiquitin co	7	43926436	43956136
ENSG0000026905	2.878893456	0.007	0.02	NA	NA	NA	NA	NA	NA
ENSG0000016577	0.235662493	0.007	0.02	FUNDC2	protein_coding	FUN14 dom	X	155025980	155060304
ENSG0000018325	-0.163900263	0.007	0.02	PTTG1IP	protein_coding	PTTG1 inter	21	44849585	44873903
ENSG0000021681	0.541884263	0.007	0.02	TUBB2BP1	unprocessed_p	tubulin beta	6	3177044	3179764
ENSG0000027289	-1.367293285	0.007	0.02	ATP6V1FNB	protein_coding	ATP6V1F ne	7	128866330	128872047
ENSG0000025371	1.285250549	0.007	0.02	MINCR	lncRNA	MYC-induce	8	143280161	143281690
ENSG0000027749	-2.229510313	0.007	0.02	AL357033.4	lncRNA	novel transc	20	62648961	62650767
ENSG0000017490	-0.163602115	0.007	0.02	RAB1B	protein_coding	RAB1B, mer	11	66268590	66277492
ENSG0000025903	0.804990285	0.007	0.02	ENSAP2	processed_pse	endosulfine	14	82692704	82693055
ENSG0000022726	1.05687485	0.007	0.02	KLLN	protein_coding	killin, p53 re	10	87859158	87863437
ENSG0000011216	0.438026761	0.007	0.02	SAYSD1	protein_coding	SAYSVFN m	6	39104063	39115186
ENSG0000016041	-0.468291039	0.007	0.02	SHKBP1	protein_coding	SH3KBP1 bi	19	40576853	40591399
ENSG0000022437	-2.529439627	0.007	0.02	AC017104.1	lncRNA	novel transc	2	231388976	231394991
ENSG0000013237	0.439688893	0.007	0.02	INPP5K	protein_coding	inositol poly	17	1494577	1516742
ENSG0000027499	5.7762207	0.007	0.02	H2AC12	protein_coding	H2A cluster	6	27147106	27147562
ENSG0000017558	-0.255324161	0.007	0.02	MRPL48	protein_coding	mitochondr	11	73787872	73865133
ENSG0000010592	0.49050139	0.008	0.02	GSDME	protein_coding	gasdermin E	7	24698355	24757940
ENSG0000027314	2.1464733	0.008	0.02	BX537318.1	lncRNA	novel transc	22	46013606	46015498
ENSG0000021019	0.479725626	0.008	0.02	MT-TP	Mt_tRNA	mitochondr	MT	15956	16023
ENSG0000027506	0.311677388	0.008	0.02	SYNRG	protein_coding	synergin ga	17	37514807	37609472
ENSG0000012427	-0.252318596	0.008	0.02	MTRR	protein_coding	5-methyltet	5	7851186	7906025
ENSG0000012314	0.164557581	0.008	0.02	TRIR	protein_coding	telomerase	19	12730640	12734684
ENSG0000001156	0.198260666	0.008	0.02	MAP4K3	protein_coding	mitogen-ac	2	39249266	39437301
ENSG0000005880	0.186407841	0.008	0.02	NDC1	protein_coding	NDC1 trans	1	53765478	53838463
ENSG0000019711	0.403464173	0.008	0.02	ZGPAT	protein_coding	zinc finger C	20	63707465	63736142
ENSG0000009006	0.119732226	0.008	0.02	PAPOLA	protein_coding	poly(A) poly	14	96501433	96567111
ENSG0000018028	-5.561925257	0.008	0.02	PLD5	protein_coding	phospholipa	1	242082986	242524697
ENSG0000010094	-0.129065179	0.008	0.02	PNN	protein_coding	pinin, desm	14	39175183	39183220
ENSG0000023252	-5.786699802	0.008	0.02	AL031773.1	lncRNA	novel transc	6	155380511	155381183
ENSG0000028035	-0.756093772	0.008	0.02	AL132656.4	TEC	novel transc	10	79681973	79684094
ENSG0000017794	0.295934815	0.008	0.02	CENPBD1	protein_coding	CENPB DNA	16	89969773	89972832
ENSG0000023323	-2.827269572	0.008	0.02	NPIPB7	protein_coding	nuclear pore	16	28456372	28471175
ENSG0000027646	-0.327112526	0.008	0.02	TCF20	protein_coding	transcription	CHR_HSCHR	42160013	42215442
ENSG0000026750	0.664346872	0.008	0.02	ZNF285	protein_coding	zinc finger p	19	44382298	44401608
ENSG0000018037	0.14072985	0.008	0.02	PAK2	protein_coding	p21 (RAC1)	3	196739857	196832647
ENSG0000019892	-0.250583342	0.008	0.02	ATG9A	protein_coding	autophagy r	2	219219380	219229717
ENSG0000011674	-0.150141955	0.008	0.02	RO60	protein_coding	Ro60, Y RNA	1	193059454	193091777
ENSG0000023503	-0.855591938	0.008	0.02	IER3	protein_coding	immediate e	CHR_HSCHR	30735460	30736815
ENSG0000011165	-0.285113652	0.008	0.02	ING4	protein_coding	inhibitor of	12	6650301	6663142
ENSG0000014150	-0.511741522	0.008	0.02	SAT2	protein_coding	spermidine/	17	7626234	7627876
ENSG0000017707	1.039820028	0.008	0.02	ACER2	protein_coding	alkaline cera	9	19409009	19452505
ENSG0000012934	0.254030659	0.008	0.02	KRI1	protein_coding	KRI1 homol	19	10553085	10566031
ENSG0000010416	0.176818748	0.008	0.02	BLOC1S6	protein_coding	biogenesis c	15	45587214	45615945
ENSG0000012440	-0.158435686	0.008	0.02	ATP8A1	protein_coding	ATPase pho	4	42408373	42657105
ENSG0000019827	0.585874006	0.008	0.02	TMEM116	protein_coding	transmemb	12	111931282	112013185
ENSG0000013631	0.347667249	0.008	0.02	TTC5	protein_coding	tetratricope	14	20256558	20305960
ENSG0000018502	0.295827404	0.008	0.02	BRF1	protein_coding	BRF1 RNA p	14	105209286	105315589
ENSG0000027740	-0.708951894	0.008	0.02	AC145212.1	protein_coding	MaFF-intera	GL000194.1	53590	115018

ENSG0000026041	-3.140424904	0.008	0.02	AL023284.4	lncRNA	novel transc	6	136335714	136336087
ENSG0000019639	-0.865901985	0.008	0.02	ZNF774	protein_coding	zinc finger p	15	90352284	90369146
ENSG0000016668	-0.181227583	0.008	0.02	BEX3	protein_coding	brain expres	X	103376395	103378164
ENSG0000017060	-0.398617599	0.008	0.02	IRF2BP1	protein_coding	interferon re	19	45883608	45886141
ENSG0000019746	-1.207844852	0.008	0.02	GYPE	protein_coding	glycophorin	4	143870864	143905563
ENSG0000011384	0.197340193	0.008	0.02	TIMMDC1	protein_coding	translocase	3	119498547	119525090
ENSG0000010565	0.448378459	0.008	0.02	ELL	protein_coding	elongation f	19	18442663	18522116
ENSG0000017638	-1.696016271	0.008	0.02	B3GNT4	protein_coding	UDP-GlcNA	12	122203681	122208952
ENSG0000027539	1.605998473	0.008	0.02	DHRS11	protein_coding	dehydroger	CHR_HSCHR	36592761	36601768
ENSG0000024424	-1.615744641	0.008	0.02	IFITM10	protein_coding	interferon ir	11	1732406	1750595
ENSG0000012052	0.217453077	0.008	0.02	NUDCD1	protein_coding	NudC doma	8	109240919	109334118
ENSG0000019802	-0.384719409	0.008	0.02	ZNF335	protein_coding	zinc finger p	20	45948660	45972203
ENSG0000013507	-0.527198174	0.008	0.02	ADAM19	protein_coding	ADAM meta	5	157395534	157575775
ENSG0000020389	0.976324722	0.008	0.02	LIME1	protein_coding	Lck interacti	20	63736283	63739103
ENSG0000016217	0.274847564	0.008	0.02	ASRGL1	protein_coding	asparaginas	11	62337448	62393412
ENSG0000028006	0.578375039	0.008	0.02	AC011504.	TEC	TEC	19	30553956	30558058
ENSG0000026149	-1.987237602	0.008	0.02	AC005674.2	lncRNA	novel transc	4	10068089	10073019
ENSG0000018863	0.192043397	0.008	0.02	RTL6	protein_coding	retrotranspo	22	44492583	44498233
ENSG0000013073	0.325384356	0.008	0.02	ATG4D	protein_coding	autophagy r	19	10543895	10553418
ENSG0000015471	0.430452742	0.008	0.02	MRPL39	protein_coding	mitochondr	21	25585656	25607517
ENSG0000011164	-0.102468369	0.008	0.02	CHD4	protein_coding	chromodom	12	6570082	6614524
ENSG0000023491	1.928486801	0.008	0.02	TEX21P	transcribed_un	testis expres	14	64341673	64387986
ENSG0000024797	1.980406069	0.008	0.02	AL160313.1	lncRNA	novel transc	14	99604556	99625740
ENSG0000018103	0.660087873	0.008	0.02	SLC25A42	protein_coding	solute carrie	19	19063994	19113030
ENSG0000014015	0.182765437	0.008	0.02	NIPA2	protein_coding	NIPA magn	15	22838644	22869362
ENSG0000015501	0.39125041	0.008	0.02	CYP2U1	protein_coding	cytochrome	4	107931549	107953461
ENSG0000016497	0.614992074	0.008	0.02	NUDT2	protein_coding	nudix hydro	9	34329506	34343711
ENSG0000016973	-0.441074411	0.008	0.02	RFNG	protein_coding	RFNG O-fuc	17	82047902	82051831
ENSG0000010673	0.999516414	0.008	0.02	NMRK1	protein_coding	nicotinamid	9	75060573	75088217
ENSG0000020591	-0.341691947	0.008	0.02	PDPK2P	transcribed_un	3-phosphoi	16	2616121	2643296
ENSG0000022986	-0.6948077	0.008	0.02	MRPS18B	protein_coding	mitochondr	CHR_HSCHR	30696164	30704850
ENSG0000011963	-0.71077491	0.008	0.02	PGF	protein_coding	placental gr	14	74941834	74955626
ENSG0000019636	0.147296066	0.008	0.02	ELAVL3	protein_coding	ELAV like RI	19	11451326	11481046
ENSG0000027672	-0.126225358	0.008	0.02	CEP170	protein_coding	centrosoma	CHR_HSCHR	243124428	243255321
ENSG0000022936	1.768071768	0.008	0.02	AC090587.2	lncRNA	novel transc	11	3854612	3855399
ENSG0000019630	-1.689062134	0.008	0.02	AC146944.	unprocessed_p	glucuronida	5	70516387	70555266
ENSG0000016921	0.184177906	0.008	0.02	CD2BP2	protein_coding	CD2 cytopla	16	30350773	30355308
ENSG0000022369	1.557193085	0.008	0.02	AF230666.1	lncRNA	novel transc	8	132838117	132844298
ENSG0000027986	2.365846614	0.008	0.02	AC006511.	TEC	TEC	12	8080381	8082163
ENSG0000017278	-0.718809656	0.008	0.02	HOXC5	protein_coding	homeobox C	12	54032853	54035358
ENSG0000022926	1.399237229	0.008	0.02	SNHG31	lncRNA	small nucleo	2	214810181	214963575
ENSG0000011187	-0.441428079	0.008	0.02	MCM9	protein_coding	minichromo	6	118813442	118935162
ENSG0000011229	-0.168164892	0.008	0.02	WASF1	protein_coding	WASP family	6	110099819	110180004
ENSG0000006623	1.168187184	0.008	0.02	SLC9A3	protein_coding	solute carrie	5	470456	524449
ENSG0000016007	0.335337828	0.008	0.02	ATAD3B	protein_coding	ATPase fam	1	1471765	1497848
ENSG0000013559	-0.224748297	0.008	0.02	REPS1	protein_coding	RALBP1 asso	6	138903493	138988261
ENSG0000010322	-0.251974015	0.008	0.02	NOMO3	protein_coding	NODAL mo	16	16232528	16294811
ENSG0000027345	1.650762207	0.008	0.02	AC064836.2	lncRNA	novel transc	2	202374932	202375604
ENSG0000015422	0.377215014	0.008	0.02	CC2D1B	protein_coding	coiled-coil a	1	52345723	52366193
ENSG0000010304	-0.3795248	0.008	0.02	SLC38A7	protein_coding	solute carrie	16	58665109	58684770
ENSG0000024948	-2.9436448	0.008	0.02	STK19B	unprocessed_p	serine/threo	CHR_HSCHR	31995452	31995969
ENSG0000002297	-0.570166612	0.008	0.02	ZNF839	protein_coding	zinc finger p	14	102317377	102342702
ENSG0000026935	1.177780121	0.008	0.02	PTOV1-AS2	lncRNA	PTOV1 antis	19	49856970	49859289
ENSG0000024344	-0.977454363	0.008	0.02	C4orf48	protein_coding	chromosom	4	2041993	2043970
ENSG0000006385	0.272476804	0.008	0.02	HAGH	protein_coding	hydroxyacyl	16	1795620	1827157
ENSG0000026211	1.918894532	0.008	0.02	MTCO1P28	processed_pse	MT-CO1 ps	16	3367719	3369246
ENSG0000017129	0.459890148	0.008	0.02	ZNF439	protein_coding	zinc finger p	19	11848726	11883750
ENSG0000013991	-0.712486911	0.008	0.02	NOVA1	protein_coding	NOVA alteri	14	26443090	26598033

ENSG0000019839	-0.26044326	0.008	0.02	ITSN2	protein_coding	intersectin 2	2	24202864	24360714
ENSG0000010007	0.355299769	0.009	0.02	SLC25A1	protein_coding	solute carrie	22	19175581	19178739
ENSG0000010429	0.281157023	0.009	0.02	INTS9	protein_coding	integrator c	8	28767661	28890242
ENSG0000006611	0.150593093	0.009	0.02	SMARCD1	protein_coding	SWI/SNF rel	12	50085200	50100707
ENSG0000020480	0.652932148	0.009	0.02	AL590399.1	lncRNA	novel transc	9	62374128	62376836
ENSG0000016703	1.100564458	0.009	0.02	NKX3-1	protein_coding	NK3 homeo	8	23678697	23682938
ENSG0000010165	-0.228765593	0.009	0.02	RNMT	protein_coding	RNA guanin	18	13726660	13764556
ENSG0000023591	1.291037719	0.009	0.02	ASH1L-AS1	lncRNA	ASH1L antis	1	155562042	155563944
ENSG0000018931	-0.252531362	0.009	0.02	FAM53B	protein_coding	family with s	10	124619292	124744378
ENSG0000016161	-0.545055953	0.009	0.02	ALDH16A1	protein_coding	aldehyde de	19	49453225	49471050
ENSG0000026042	-1.751923294	0.009	0.02	SCX	protein_coding	scleraxis bH	8	144266453	144268481
ENSG0000027034	0.983498364	0.009	0.02	POC1B-AS1	lncRNA	POC1B antis	12	89524594	89548005
ENSG0000027017	-0.463276861	0.009	0.02	NA	NA	NA	NA	NA	NA
ENSG0000010796	-1.524333596	0.009	0.02	MAP3K8	protein_coding	mitogen-ac	10	30434021	30461833
ENSG0000013236	-0.235959528	0.009	0.02	CLUH	protein_coding	clustered m	17	2689386	2712663
ENSG0000011203	-0.212301036	0.009	0.02	MTRF1L	protein_coding	mitochondr	6	152987362	153002709
ENSG0000015377	0.231800537	0.009	0.02	CFDP1	protein_coding	craniofacial	16	75293698	75433503
ENSG0000018232	-1.497947949	0.009	0.02	C1S	protein_coding	complemen	12	6988259	7071032
ENSG0000006733	0.199602673	0.009	0.02	DNTTIP2	protein_coding	deoxynucle	1	93866284	93879918
ENSG0000018900	0.432695558	0.009	0.02	ADAT2	protein_coding	adenosine c	6	143422832	143450695
ENSG0000015495	0.853540492	0.009	0.02	ZNF18	protein_coding	zinc finger p	17	11977439	11997475
ENSG0000010296	0.539878565	0.009	0.02	DHODH	protein_coding	dihydroorot	16	72008588	72027664
ENSG0000013972	0.160365104	0.009	0.02	DENR	protein_coding	density regu	12	122752824	122771064
ENSG0000013425	-3.098167458	0.009	0.02	NGF	protein_coding	nerve growt	1	115285917	115338256
ENSG0000023042	2.399708561	0.009	0.02	EMC1-AS1	lncRNA	EMC1 antise	1	19210501	19240704
ENSG0000004060	-0.830387593	0.009	0.02	RTN4R	protein_coding	reticulon 4 r	22	20241415	20283246
ENSG0000024202	0.455246959	0.009	0.02	HYPK	protein_coding	huntingtin ir	15	43796142	43804427
ENSG0000019650	0.477406077	0.009	0.02	TCEAL3	protein_coding	transcription	X	103607451	103629690
ENSG0000023775	1.201705461	0.009	0.02	AC079922.2	lncRNA	novel transc	2	112641832	112645720
ENSG0000010034	-1.127567406	0.009	0.02	PNPLA3	protein_coding	patatin like	22	43923792	43964488
ENSG0000016416	0.393287336	0.009	0.02	PRMT9	protein_coding	protein argi	4	147637785	147684163
ENSG0000023659	0.997471729	0.009	0.02	ZNRD1ASP	lncRNA	zinc ribbon	CHR_HSCHR	29993130	30054145
ENSG0000026122	-0.577876924	0.009	0.02	ZNF865	protein_coding	zinc finger p	19	55605405	55617269
ENSG0000023759	-1.234767477	0.009	0.02	TAP2	protein_coding	transporter	CHR_HSCHR	32855328	32872254
ENSG0000010100	-1.863078835	0.009	0.02	PROCR	protein_coding	protein C re	20	35172072	35216240
ENSG0000011186	0.339351513	0.009	0.02	CEP85L	protein_coding	centrosoma	6	118460772	118710075
ENSG0000014000	0.335499324	0.009	0.02	WDR89	protein_coding	WD repeat c	14	63597039	63641861
ENSG0000015809	-0.346518125	0.009	0.02	NCK1	protein_coding	NCK adapto	3	136862208	136951606
ENSG0000016415	0.142809111	0.009	0.02	ICE1	protein_coding	interactor o	5	5420664	5490220
ENSG0000025480	-1.438288226	0.009	0.02	SYS1-DBNI	protein_coding	SYS1-DBND	20	45363200	45410610
ENSG0000016903	0.252172587	0.009	0.02	MAP2K1	protein_coding	mitogen-ac	15	66386837	66491544
ENSG0000012677	-0.300116155	0.009	0.02	ATG14	protein_coding	autophagy r	14	55366391	55411830
ENSG0000024708	-1.317604532	0.009	0.02	BAALC-AS1	lncRNA	BAALC antis	8	103153394	103298772
ENSG0000017769	-2.299127969	0.009	0.02	DNAJC28	protein_coding	DnaJ heat sl	21	33485530	33491716
ENSG0000006193	-0.325447994	0.009	0.02	TNK2	protein_coding	tyrosine kina	3	195863364	195911945
ENSG0000006813	-0.279103742	0.009	0.02	PLEKHH3	protein_coding	pleckstrin ho	17	42667914	42676994
ENSG0000015605	0.129662317	0.009	0.02	GNAQ	protein_coding	G protein su	9	77716097	78031811
ENSG0000015509	0.122128664	0.009	0.02	AZIN1	protein_coding	antizyme inl	8	102826308	102893864
ENSG0000016688	-1.496836049	0.009	0.02	STAT6	protein_coding	signal transo	12	57095408	57132139
ENSG0000016924	-0.49171379	0.009	0.02	ZRSR2	protein_coding	zinc finger C	X	15790472	15823260
ENSG0000016755	0.77306466	0.009	0.02	RHEBL1	protein_coding	RHEB like 1	12	49064676	49070025
ENSG0000011438	0.622839105	0.009	0.02	TUSC2	protein_coding	tumor supp	3	50320027	50328251
ENSG0000010134	0.23751623	0.009	0.02	CRNKL1	protein_coding	crooked nec	20	20034368	20056046
ENSG0000022349	0.657085865	0.009	0.02	SKIV2L	protein_coding	Ski2 like RN	CHR_HSCHR	31941262	31951933
ENSG0000013200	0.536982603	0.009	0.02	FBXW9	protein_coding	F-box and W	19	12688053	12696631
ENSG0000026774	-1.348658906	0.009	0.02	SINHCAFP2	processed_pse	SINHCAF ps	18	60016778	60017441
ENSG0000006744	0.226084848	0.009	0.02	TRO	protein_coding	trophinin [S	X	54920462	54931431
ENSG0000010037	-0.298282188	0.009	0.02	FAM118A	protein_coding	family with s	22	45308968	45341955

ENSG0000011695	-0.269266572	0.009	0.02	RRAGC	protein_codinc	Ras related	1	38838198	38859772
ENSG0000019835	0.22214083	0.009	0.02	GET3	protein_codinc	guided entr	19	12737139	12748323
ENSG0000015141	0.236798755	0.009	0.02	NEK7	protein_codinc	NIMA relate	1	198156994	198322420
ENSG0000001282	0.241464882	0.009	0.02	CALCOCO1	protein_codinc	calcium binc	12	53708517	53727745
ENSG0000022674	-1.297025631	0.009	0.02	HSBP1L1	protein_codinc	heat shock f	18	79964582	79970822
ENSG0000024027	-5.704545213	0.009	0.02	RPL12P37	processed_pse	ribosomal p	17	80976454	80976948
ENSG0000008198	-1.263214236	0.009	0.02	IL12RB2	protein_codinc	interleukin 1	1	67307364	67398724
ENSG0000018882	-2.456678267	0.009	0.02	CALHM6	protein_codinc	calcium hon	6	116461370	116463771
ENSG0000000258	0.39159781	0.009	0.02	CD99	protein_codinc	CD99 molec	X	2691187	2741309
ENSG0000016943	-0.182068588	0.009	0.02	SDC2	protein_codinc	syndecan 2	8	96493813	96611790
ENSG0000018499	0.297874221	0.009	0.02	BRI3BP	protein_codinc	BRI3 bindinc	12	124993645	125031231
ENSG0000012200	-0.237392973	0.009	0.02	POLK	protein_codinc	DNA polym	5	75511756	75601144
ENSG0000001910	2.239473919	0.009	0.02	VSIG2	protein_codinc	V-set and ir	11	124747474	124752255
ENSG0000007380	0.312354856	0.009	0.02	MAP3K13	protein_codinc	mitogen-ac	3	185282941	185489094
ENSG0000019852	1.99179922	0.009	0.02	ARMH1	protein_codinc	armadillo lik	1	44674692	44725591
ENSG0000022477	-0.509980697	0.009	0.02	CSNK2B	protein_codinc	casein kinas	CHR_HSCHR	31655985	31664295
ENSG0000016409	0.300859401	0.009	0.02	C4orf3	protein_codinc	chromosom	4	119296419	119304445
ENSG0000023684	1.122145015	0.009	0.02	NRM	protein_codinc	nurim [Sour	CHR_HSCHR	30678308	30681681
ENSG0000027449	-2.105729962	0.009	0.02	AL121768.1	lncRNA	novel transc	14	88819608	88822151
ENSG0000016351	0.928695243	0.009	0.02	TGFBR2	protein_codinc	transforminc	3	30606601	30694142
ENSG0000013525	-0.22063654	0.009	0.02	SRPK2	protein_codinc	SRSF proteir	7	105110704	105399308
ENSG0000027686	-3.742183847	0.009	0.02	AC074050.4	lncRNA	novel transc	16	31704728	31705260
ENSG0000018795	1.174172691	0.009	0.02	DNER	protein_codinc	delta/notch	2	229357629	229714555
ENSG0000013705	-0.255300263	0.01	0.02	POLR1E	protein_codinc	RNA polyme	9	37485948	37503697
ENSG0000015530	-0.116810819	0.01	0.02	HSPA13	protein_codinc	heat shock p	21	14371115	14383484
ENSG0000019887	-1.571537133	0.01	0.02	STKLD1	protein_codinc	serine/threc	9	133376366	133406096
ENSG0000012742	0.604659863	0.01	0.02	AUNIP	protein_codinc	aurora kinas	1	25831913	25859458
ENSG0000025932	1.59066999	0.01	0.02	AL136295.2	lncRNA	novel transc	14	24139445	24140444
ENSG0000023480	1.244608402	0.01	0.02	NRM	protein_codinc	nurim [Sour	CHR_HSCHR	30766531	30769904
ENSG0000011730	0.501665762	0.01	0.02	GALE	protein_codinc	UDP-galact	1	23795599	23800781
ENSG0000012908	-0.126347667	0.01	0.02	COPB1	protein_codinc	COPI coat c	11	14443440	14500027
ENSG0000021396	0.55099996	0.01	0.02	ZNF726	protein_codinc	zinc finger p	19	23914876	23945159
ENSG0000027194	-1.628677959	0.01	0.02	AC017076.1	lncRNA	novel transc	2	6905724	6906301
ENSG0000014223	-0.898890893	0.01	0.02	LMTK3	protein_codinc	lemur tyrosi	19	48485271	48513935
ENSG0000015646	-0.128684957	0.01	0.02	UQCRB	protein_codinc	ubiquinol-c	8	96222947	96235546
ENSG0000007624	0.230452022	0.01	0.02	UNG	protein_codinc	uracil DNA (	12	109097597	109110992
ENSG0000026156	-2.203223039	0.01	0.02	NA	NA	NA	NA	NA	NA
ENSG0000025437	-1.273446187	0.01	0.02	MIR124-2H	lncRNA	MIR124-2 h	8	64373151	64383787
ENSG0000017345	-0.350579246	0.01	0.02	THAP2	protein_codinc	THAP doma	12	71664301	71680644
ENSG0000022439	-0.368612909	0.01	0.02	SLC39A7	protein_codinc	solute carrie	CHR_HSCHR	33370805	33374799
ENSG0000018328	-1.543387932	0.01	0.02	CCBE1	protein_codinc	collagen an	18	59430939	59697662
ENSG0000018936	0.437549716	0.01	0.02	NEMP2	protein_codinc	nuclear env	2	190504338	190534722
ENSG0000010155	-0.181463087	0.01	0.02	VAPA	protein_codinc	VAMP assoc	18	9914016	9960021
ENSG0000017718	-1.210628545	0.01	0.02	CLVS1	protein_codinc	clavesin 1 [S	8	61057158	61501645
ENSG0000024052	2.398048103	0.01	0.02	RPL7AP10	processed_pse	ribosomal p	19	21149648	21150438
ENSG0000018083	0.904119064	0.01	0.02	MAP6D1	protein_codinc	MAP6 doma	3	183815922	183825594
ENSG0000006799	0.528817325	0.01	0.02	PDK3	protein_codinc	pyruvate de	X	24465270	24550466
ENSG0000018718	1.235859447	0.01	0.02	AL162231.1	protein_codinc	uncharacter	9	34664163	34666112
ENSG0000010142	0.216818151	0.01	0.02	CHMP4B	protein_codinc	charged mu	20	33811348	33854366
ENSG0000013031	-0.346535085	0.01	0.02	PGLS	protein_codinc	6-phospho(	19	17511636	17521288
ENSG0000010611	-1.70595307	0.01	0.02	CRHR2	protein_codinc	corticotropi	7	30651942	30700129
ENSG0000011764	0.286498239	0.01	0.02	MTFR1L	protein_codinc	mitochondr	1	25818640	25832942
ENSG0000026999	0.676790165	0.01	0.02	NA	NA	NA	NA	NA	NA
ENSG0000019815	2.400090208	0.01	0.02	ZNF849P	unprocessed_p	zinc finger p	19	22685167	22686732
ENSG0000013322	0.151822911	0.01	0.02	SRRM1	protein_codinc	serine and a	1	24631716	24673281
ENSG0000019795	0.091751344	0.01	0.02	RPL12	protein_codinc	ribosomal p	9	127447674	127451406
ENSG0000021372	0.641037159	0.01	0.02	DDAH2	protein_codinc	dimethylarg	6	31727038	31730617
ENSG0000010717	0.283557317	0.01	0.02	CREB3	protein_codinc	cAMP respc	9	35732598	35736999

ENSG0000023952	0.463752051	0.01	0.02	CASTOR3	transcribed_un	CASTOR far	7	100200653	100272218
ENSG0000007095	0.299026645	0.01	0.02	RAD18	protein_coding	RAD18 E3 u	3	8775402	8963773
ENSG0000027215	-0.572290695	0.01	0.03	NBPF25P	transcribed_un	NBPF memb	1	145572345	145607858
ENSG0000022849	-1.191394622	0.01	0.03	RAB11FIP1I	processed_pse	RAB11 fami	X	74202834	74204595
ENSG0000016553	-0.155478217	0.01	0.03	TTC8	protein_coding	tetratricope	14	88824153	88881078
ENSG0000016342	0.143745678	0.01	0.03	LRRC58	protein_coding	leucine rich	3	120324509	120349354
ENSG0000021429	-0.534718439	0.01	0.03	APTR	lncRNA	Alu-mediate	7	77657659	77696267
ENSG0000027389	-0.459594426	0.01	0.03	NOL12	protein_coding	nucleolar pr	22	37681673	37693476
ENSG0000017478	-0.122837757	0.01	0.03	SRP72	protein_coding	signal recog	4	56467617	56503681
ENSG0000006068	0.228548152	0.01	0.03	SNRNP40	protein_coding	small nuclea	1	31259568	31296788
ENSG0000011214	-1.642464225	0.01	0.03	CD83	protein_coding	CD83 molec	6	14117256	14136918
ENSG0000022879	-2.23748482	0.01	0.03	NA	NA	NA	NA	NA	NA
ENSG0000023324	5.662448279	0.01	0.03	PHC2-AS1	lncRNA	PHC2 antise	1	33350352	33363245
ENSG0000021593	-3.304042727	0.01	0.03	NA	NA	NA	NA	NA	NA
ENSG0000014152	-1.472846551	0.01	0.03	CARD14	protein_coding	caspase rec	17	80169992	80209331
ENSG0000027265	-0.858313919	0.01	0.03	POLR2J4	transcribed_un	RNA polyme	7	44013562	44019170
ENSG0000019781	0.417570222	0.01	0.03	SLC9A8	protein_coding	solute carrie	20	49812713	49892242
ENSG0000016756	-0.671264252	0.01	0.03	SERTAD3	protein_coding	SERTA dom	19	40440844	40444335
ENSG0000023302	-0.523672582	0.01	0.03	NPIPA9	protein_coding	nuclear pore	16	18358086	18379331
ENSG0000010020	-0.314089965	0.01	0.03	TCF20	protein_coding	transcription	22	42160013	42343616
ENSG0000014286	0.44193639	0.01	0.03	BCL10	protein_coding	BCL10 immu	1	85265776	85276632
ENSG0000023539	1.109212732	0.01	0.03	ZNRD1ASP	lncRNA	zinc ribbon	CHR_HSCHR	29996148	30056801
ENSG0000027914	-1.34361041	0.01	0.03	AC011912.1	lncRNA	TEC	15	55171378	55176739
ENSG0000010412	-0.531206121	0.01	0.03	DNAJC17	protein_coding	DnaJ heat sl	15	40765161	40807478
ENSG0000012398	0.30170664	0.01	0.03	CHPF	protein_coding	chondroitin	2	219538948	219543809
ENSG0000016357	0.383515724	0.01	0.03	EIF5A2	protein_coding	eukaryotic t	3	170888418	170908644
ENSG0000020458	-0.736565603	0.01	0.03	DDR1	protein_coding	discoidin do	6	30876421	30900156
ENSG0000024400	0.389460838	0.01	0.03	NFS1	protein_coding	NFS1 cystei	20	35668052	35699355
ENSG0000008138	-0.256438888	0.01	0.03	ZNF510	protein_coding	zinc finger p	9	96755865	96778129
ENSG0000019675	0.55016509	0.01	0.03	ZNF700	protein_coding	zinc finger p	19	11925068	11950773
ENSG0000010406	0.205721715	0.01	0.03	GABPB1	protein_coding	GA binding	15	50275392	50355408
ENSG0000013034	0.394157776	0.01	0.03	MTRES1	protein_coding	mitochondr	6	107028199	107051586
ENSG0000006995	0.165148027	0.01	0.03	MAPK6	protein_coding	mitogen-ac	15	51952106	52067375
ENSG0000021407	-1.210490238	0.01	0.03	CPSF1P1	transcribed_pr	cleavage an	22	32269381	32273110
ENSG0000017555	-1.728523578	0.01	0.03	LONRF3	protein_coding	LON peptid	X	118974614	119018355
ENSG0000007879	2.775086147	0.01	0.03	PKD2L2	protein_coding	polycystin 2	5	137887968	137942747
ENSG0000013124	-0.364585976	0.01	0.03	RAB11FIP4	protein_coding	RAB11 fami	17	31391675	31538211
ENSG0000011172	-1.065617988	0.011	0.03	ST8SIA1	protein_coding	ST8 alpha-N	12	22063773	22437041
ENSG0000027670	-0.49928474	0.011	0.03	AC245049.1	protein_coding	Pyridoxal-d	CHR_HSCHR	15241230	15297417
ENSG0000022580	2.493389518	0.011	0.03	AL121917.1	lncRNA	novel transc	20	58863528	58888809
ENSG0000021375	-0.374767175	0.011	0.03	CENPBD1P	transcribed_pr	CENPB DNA	19	58573503	58605223
ENSG0000027297	0.66458849	0.011	0.03	AL008721.2	lncRNA	novel transc	22	25476218	25479971
ENSG0000017522	-0.186093575	0.011	0.03	ATG13	protein_coding	autophagy r	11	46617527	46674818
ENSG0000023299	0.958164834	0.011	0.03	RGS5	lncRNA	regulator of	1	163244505	163321894
ENSG0000016186	1.207924632	0.011	0.03	SYCE2	protein_coding	synaptonem	19	12898786	12919293
ENSG0000024473	-1.469427914	0.011	0.03	AL132656.2	lncRNA	novel transc	10	79660891	79677996
ENSG0000018556	-0.182650051	0.011	0.03	LSAMP	protein_coding	limbic syster	3	115802363	117139389
ENSG0000013082	-1.640531391	0.011	0.03	PNCK	protein_coding	pregnancy u	X	153669733	153689010
ENSG0000027940	-1.526417718	0.011	0.03	AL359183.1	TEC	TEC	10	71364243	71366374
ENSG0000025995	0.184879415	0.011	0.03	RBM15B	protein_coding	RNA binding	3	51391285	51397908
ENSG0000014711	-1.397421778	0.011	0.03	ZNF157	protein_coding	zinc finger p	X	47370578	47414498
ENSG0000022652	-0.975981771	0.011	0.03	RPS7P10	processed_pse	ribosomal p	13	21628413	21629015
ENSG0000009214	0.199124243	0.011	0.03	G2E3	protein_coding	G2/M-phas	14	30559158	30620064
ENSG0000013684	-0.195374938	0.011	0.03	DAB2IP	protein_coding	DAB2 intera	9	121567057	121785530
ENSG0000010443	-0.884620146	0.011	0.03	IL7	protein_coding	interleukin 7	8	78675743	78805523
ENSG0000020437	-0.443371356	0.011	0.03	EHMT2	protein_coding	euchromatic	6	31879759	31897687
ENSG0000021396	-0.454158025	0.011	0.03	NUDT19	protein_coding	nudix hydro	19	32691961	32713796
ENSG0000015382	-0.124908364	0.011	0.03	TRIP12	protein_coding	thyroid horr	2	229763837	229923239

ENSG0000018466	-4.327714983	0.011	0.03	OR7E14P	transcribed_un	olfactory re	11	17013998	17053024
ENSG0000016764	-2.513789219	0.011	0.03	PPP1R14A	protein_coding	protein pho	19	38251237	38256532
ENSG0000028000	-2.293235434	0.011	0.03	NA	NA	NA	NA	NA	NA
ENSG0000013146	-0.114892323	0.011	0.03	RPL27	protein_coding	ribosomal p	17	42998273	43002959
ENSG0000007542	-0.238543603	0.011	0.03	FOSL2	protein_coding	FOS like 2, /	2	28392448	28417317
ENSG0000018590	0.527229774	0.011	0.03	POMK	protein_coding	protein O-n	8	43093498	43131180
ENSG0000027521	1.020417667	0.011	0.03	RNA5-8SN	rRNA	RNA, 5.8S ri	21	8395607	8395759
ENSG0000018072	-2.057665137	0.011	0.03	CHRM4	protein_coding	cholinergic	11	46385098	46386608
ENSG0000014418	0.858546964	0.011	0.03	LIPT1	protein_coding	lipoyltransfe	2	99154955	99163157
ENSG0000026111	-1.049904479	0.011	0.03	AC012181.1	lncRNA	novel transc	16	56941028	56941726
ENSG0000025586	-1.248105855	0.011	0.03	DENND5B-	lncRNA	DENND5B a	12	31589923	31615666
ENSG0000016924	-0.352416311	0.011	0.03	SLC50A1	protein_coding	solute carrie	1	155135344	155138857
ENSG0000027294	-2.575400252	0.011	0.03	AL022324.3	lncRNA	novel transc	22	25434324	25435070
ENSG0000027389	-1.349046736	0.011	0.03	AL133520.1	lncRNA	novel transc	20	46681676	46682375
ENSG0000022717	3.36078505	0.011	0.03	NSA2P5	processed_pse	NSA2 pseuc	2	170077224	170078294
ENSG0000017991	-0.188984645	0.011	0.03	R3HDM2	protein_coding	R3H domair	12	57253762	57431005
ENSG0000022020	-0.326236259	0.011	0.03	VAMP2	protein_coding	vesicle asso	17	8159149	8163546
ENSG0000023067	0.258212455	0.011	0.03	BRD2	protein_coding	bromodoma	CHR_HSCHR	32955787	32959446
ENSG0000025920	-1.325242409	0.011	0.03	PRKXP1	processed_pse	PRKX pseud	15	100553529	100558954
ENSG0000027926	-0.958338246	0.011	0.03	AL078621.1	TEC	TEC	2	113605867	113607908
ENSG0000025079	1.758892672	0.011	0.03	AC127070.2	lncRNA	novel transc	12	132911470	132914732
ENSG0000017510	-0.294738918	0.011	0.03	TRAF6	protein_coding	TNF recepto	11	36483769	36510272
ENSG0000027267	1.77058401	0.011	0.03	AC124016.1	lncRNA	novel transc	4	82374301	82384027
ENSG0000027452	-0.300922775	0.011	0.03	RCC1L	protein_coding	RCC1 like [S	7	75027122	75074228
ENSG0000013862	1.136228897	0.011	0.03	SEMA7A	protein_coding	semaphorin	15	74409289	74433958
ENSG0000013432	-0.138584391	0.011	0.03	LPIN1	protein_coding	lipin 1 [Sour	2	11677595	11827409
ENSG0000014169	0.207224484	0.011	0.03	RETREG3	protein_coding	reticulophag	17	42579513	42610623
ENSG0000011823	-2.26368233	0.011	0.03	CRYGD	protein_coding	crystallin ga	2	208121607	208124524
ENSG0000022569	-0.978423594	0.011	0.03	HLA-C	protein_coding	major histo	CHR_HSCHR	31345885	31349256
ENSG0000025774	-0.558475447	0.011	0.03	NA	NA	NA	NA	NA	NA
ENSG0000016635	1.952900635	0.011	0.03	WDR88	protein_coding	WD repeat	19	33132090	33175799
ENSG0000010293	0.935059239	0.011	0.03	PLLP	protein_coding	plasmolipin	16	57248547	57284672
ENSG0000020452	-0.933749194	0.011	0.03	ZNF805	protein_coding	zinc finger p	19	57240632	57262728
ENSG0000018613	-0.226694219	0.011	0.03	ZBTB6	protein_coding	zinc finger a	9	122908056	122913323
ENSG0000015497	0.249818169	0.011	0.03	VOPP1	protein_coding	VOPP1 WW	7	55436056	55572988
ENSG0000013081	0.388139486	0.011	0.03	ZNF426	protein_coding	zinc finger p	19	9523223	9538645
ENSG0000002442	0.522623698	0.011	0.03	EHD2	protein_coding	EH domain	19	47713422	47743134
ENSG0000012693	-0.17760046	0.011	0.03	MAP2K2	protein_coding	mitogen-ac	19	4090321	4124122
ENSG0000015314	0.11155477	0.011	0.03	SMARCA5	protein_coding	SWI/SNF rel	4	143513702	143557486
ENSG0000018796	0.494816608	0.011	0.03	KLHL17	protein_coding	kelch like fa	1	960584	965719
ENSG0000013675	-0.111792525	0.011	0.03	YME1L1	protein_coding	YME1 like 1	10	27110111	27155266
ENSG0000010858	-0.134714781	0.011	0.03	CCDC47	protein_coding	coiled-coil	17	63745255	63776351
ENSG0000015572	0.411281062	0.011	0.03	KCTD18	protein_coding	potassium c	2	200488958	200519784
ENSG0000016566	0.241355058	0.011	0.03	ABRAXAS2	protein_coding	abraxas 2, B	10	124801819	124836667
ENSG0000028007	-0.413659871	0.011	0.03	GATD3B	protein_coding	glutamine a	21	5079294	5128413
ENSG0000014750	-0.963262368	0.011	0.03	RGS20	protein_coding	regulator of	8	53851795	53959303
ENSG0000019822	-0.920350681	0.011	0.03	FKBP1C	protein_coding	FKBP prolyl	6	63211446	63213024
ENSG0000026007	0.650810941	0.011	0.03	NSFP1	unprocessed_p	N-ethylmale	17	46372855	46487141
ENSG0000027504	0.454679373	0.011	0.03	GTF2H2	protein_coding	general trar	CHR_HSCHR	69559974	69592641
ENSG0000013591	0.207591958	0.011	0.03	USP37	protein_coding	ubiquitin sp	2	218450251	218568351
ENSG0000010047	0.481110778	0.011	0.03	POLE2	protein_coding	DNA polym	14	49643555	49688422
ENSG0000025837	1.546240992	0.012	0.03	AL139099.1	lncRNA	novel transc	14	49620815	49623480
ENSG0000014537	0.331224313	0.012	0.03	SPATA5	protein_coding	spermatoge	4	122923070	123319433
ENSG0000025541	1.591510743	0.012	0.03	MSH5-SAP	protein_coding	MSH5-SAP(	CHR_HSCHR	31748922	31755608
ENSG0000026125	2.411602944	0.012	0.03	Z97055.2	lncRNA	novel transc	22	43812456	43817394
ENSG0000014948	-1.352085047	0.012	0.03	ROM1	protein_coding	retinal outer	11	62611722	62615116
ENSG0000000819	-1.933096562	0.012	0.03	TFAP2D	protein_coding	transcriptior	6	50713526	50773033
ENSG0000027931	-1.763920239	0.012	0.03	AC002525.1	TEC	TEC	13	31960325	31961946

ENSG0000014514	0.847749889	0.012	0.03	SLIT2	protein_coding	slit guidance	4	20251905	20620561
ENSG0000023744	-0.602855079	0.012	0.03	RGL2	protein_coding	ral guanine	6	33291654	33298942
ENSG0000018161	0.224749773	0.012	0.03	MRPS23	protein_coding	mitochondr	17	57834781	57850056
ENSG0000008085	-0.22616549	0.012	0.03	IGSF9B	protein_coding	immunoglol	11	133896438	133956968
ENSG0000011259	-0.32047618	0.012	0.03	TBP	protein_coding	TATA-box b	6	170554302	170572870
ENSG0000017047	0.156731279	0.012	0.03	RALGAPB	protein_coding	Ral GTPase	20	38472816	38578859
ENSG0000008379	-0.261328015	0.012	0.03	CYLD	protein_coding	CYLD lysine	16	50742050	50801935
ENSG0000012359	-0.439240733	0.012	0.03	RAB9A	protein_coding	RAB9A, mer	X	13689128	13710504
ENSG0000012582	0.224973942	0.012	0.03	DTD1	protein_coding	D-aminoacy	20	18587942	18766644
ENSG0000007807	-0.335274351	0.012	0.03	MCCC1	protein_coding	methylcrotc	3	183015218	183116075
ENSG0000027827	-1.940854609	0.012	0.03	RPS9	protein_coding	ribosomal p	CHR_HSCHR	54201357	54249609
ENSG0000016368	-0.907971544	0.012	0.03	ABHD6	protein_coding	abhydrolase	3	58237532	58295693
ENSG0000014685	0.836767674	0.012	0.03	AGBL3	protein_coding	ATP/GTP bi	7	134986508	135147963
ENSG0000021544	0.569893345	0.012	0.03	NA	NA	NA	NA	NA	NA
ENSG0000013022	-0.718145945	0.012	0.03	GADD45G	protein_coding	growth arre	9	89605012	89606555
ENSG0000018240	0.451156571	0.012	0.03	PGBD4	protein_coding	piggyBac tr	15	34102083	34108686
ENSG0000017737	-1.208678344	0.012	0.03	HIC1	protein_coding	HIC ZBTB tr	17	2054154	2063241
ENSG0000011223	0.195133688	0.012	0.03	CCNC	protein_coding	cyclin C [Sou	6	99542387	99568825
ENSG0000022764	0.632518486	0.012	0.03	AGPAT1	protein_coding	1-acylglycer	CHR_HSCHR	32207600	32217484
ENSG0000023255	0.628752833	0.012	0.03	NA	NA	NA	NA	NA	NA
ENSG0000024767	1.864066378	0.012	0.03	LRP4-AS1	lncRNA	LRP4 antiser	11	46846412	46874421
ENSG0000026111	0.1764561	0.012	0.03	TMEM178B	protein_coding	transmembr	7	141074064	141480380
ENSG0000020576	-0.641701926	0.012	0.03	RP9P	transcribed_un	RP9 pseudo	7	32916815	32943176
ENSG0000028028	1.290359438	0.012	0.03	AC131212.1	TEC	novel transc	12	132550729	132554947
ENSG0000017038	-0.948073802	0.012	0.03	SEMA3E	protein_coding	semaphorin	7	83363238	83649139
ENSG0000018635	0.870779517	0.012	0.03	ANKRD37	protein_coding	ankyrin repe	4	185396021	185400628
ENSG0000010549	-2.0342128	0.012	0.03	PLA2G4C	protein_coding	phospholipa	19	48047843	48110817
ENSG0000015696	-0.793347189	0.012	0.03	MPV17L	protein_coding	MPV17 mitc	16	15395754	15413271
ENSG0000012099	-0.172015033	0.012	0.03	LYPLA1	protein_coding	lysophosphc	8	54046367	54102017
ENSG0000010499	-1.146651721	0.012	0.03	IL27RA	protein_coding	interleukin 2	19	14031762	14053218
ENSG0000023575	0.500464716	0.012	0.03	AGPAT1	protein_coding	1-acylglycer	CHR_HSCHR	32244640	32254524
ENSG0000027049	-3.27512086	0.012	0.03	AC137695.1	processed_pse	MAP/microt	3	129334586	129335074
ENSG0000015268	0.185750757	0.012	0.03	SLC30A6	protein_coding	solute carrie	2	32165841	32224379
ENSG0000016028	-0.238971948	0.012	0.03	LSS	protein_coding	lanosterol sy	21	46188141	46228824
ENSG0000013932	0.195910616	0.012	0.03	TMTC3	protein_coding	transmembr	12	88142296	88199887
ENSG0000016430	0.194997497	0.012	0.03	SERINC5	protein_coding	serine incor	5	80111651	80256048
ENSG0000028040	2.591956899	0.012	0.03	AC022532.1	TEC	TEC	10	70434829	70436556
ENSG0000027433	0.333260255	0.012	0.03	VASN	protein_coding	vasorin [Sou	CHR_HSCHR	4373807	4385487
ENSG0000020380	-0.83312897	0.012	0.03	BVES-AS1	lncRNA	BVES antise	6	105136308	105169952
ENSG0000012682	-0.356068823	0.012	0.03	PLEKHG3	protein_coding	pleckstrin h	14	64704102	64750249
ENSG0000027937	0.950836021	0.012	0.03	AC003973.2	TEC	TEC	19	21965708	21968529
ENSG0000016307	2.072815023	0.012	0.03	NOSTRIN	protein_coding	nitric oxide	2	168786539	168865514
ENSG0000013257	-0.452804192	0.012	0.03	PCBD2	protein_coding	pterin-4 alp	5	134905120	135007959
ENSG0000005827	0.157723785	0.012	0.03	PPP1R12A	protein_coding	protein pho	12	79773563	79935460
ENSG0000019707	0.517548859	0.012	0.03	ARRDC1	protein_coding	arrestin don	9	137605685	137615360
ENSG0000017090	4.914938167	0.012	0.03	NDUFA3	protein_coding	NADH:ubiqu	19	54102728	54109257
ENSG0000020588	-1.39249317	0.012	0.03	C1RL-AS1	lncRNA	C1RL antise	12	7108052	7122501
ENSG0000013150	0.13752737	0.012	0.03	NDFIP1	protein_coding	Nedd4 fami	5	142108779	142154440
ENSG0000011049	0.239412075	0.012	0.03	AMBRA1	protein_coding	autophagy a	11	46396414	46594125
ENSG0000018537	-0.401105306	0.012	0.03	RAD51D	protein_coding	RAD51 para	17	35092208	35121522
ENSG0000017990	3.348194945	0.012	0.03	C1orf194	protein_coding	chromosom	1	109105951	109113857
ENSG0000022377	0.544483063	0.012	0.03	MRPS18B	protein_coding	mitochondr	CHR_HSCHR	30609932	30618618
ENSG0000010483	-0.728197719	0.012	0.03	TUBB4A	protein_coding	tubulin beta	19	6494319	6502848
ENSG0000024029	-0.868289459	0.012	0.03	AL450384.2	lncRNA	novel transc	10	18513115	18545651
ENSG0000019623	-0.199932622	0.012	0.03	LCOR	protein_coding	ligand depe	10	96832254	96995956
ENSG0000008803	-0.462995399	0.013	0.03	CNOT3	protein_coding	CCR4-NOT	19	54137749	54155681
ENSG0000020496	1.435165767	0.013	0.03	PCDHA5	protein_coding	protocadhe	5	140821604	141012347
ENSG0000017046	-1.227907057	0.013	0.03	SPATA24	protein_coding	spermatoge	5	139396563	139404088

ENSG0000027894	-0.752349082	0.013	0.03	AL031587.5	TEC	TEC		22	37943050	37944898
ENSG0000014344	0.638293625	0.013	0.03	C1orf56	protein_coding	chromosom		1	151047751	151051986
ENSG0000016334	0.158312739	0.013	0.03	HIPK1	protein_coding	homeodom		1	113929324	113977869
ENSG0000013296	-0.256096069	0.013	0.03	CDK8	protein_coding	cyclin deper		13	26254104	26405238
ENSG0000010105	0.288266063	0.013	0.03	IFT52	protein_coding	intraflagella		20	43590937	43647299
ENSG0000013361	0.175809197	0.013	0.03	AGAP3	protein_coding	ArfGAP with		7	151085831	151144436
ENSG0000026859	1.065482657	0.013	0.03	RAET1E-AS	lncRNA	RAET1E anti		6	149863494	149919507
ENSG0000025095	-2.103182167	0.013	0.03	AC012055.1	lncRNA	novel transc		4	174135055	174154637
ENSG0000012545	0.448445865	0.013	0.03	MIF4GD	protein_coding	MIF4G dom		17	75266228	75271227
ENSG0000011448	-0.294035392	0.013	0.03	GBE1	protein_coding	1,4-alpha-g		3	81489703	81761645
ENSG0000008968	-0.361884731	0.013	0.03	RBM41	protein_coding	RNA binding	X		107064420	107118823
ENSG0000022540	1.500845398	0.013	0.03	RAB28P5	processed_pse	RAB28, mer	X		136847384	136848034
ENSG0000008969	0.15081186	0.013	0.03	MLF2	protein_coding	myeloid leu		12	6747996	6767475
ENSG0000017161	-0.249627558	0.013	0.03	SLC25A33	protein_coding	solute carrie		1	9539465	9585173
ENSG0000019723	1.371883319	0.013	0.03	H4C11	protein_coding	H4 clustered		6	27824092	27824480
ENSG0000027908	-0.837952472	0.013	0.03	AC005839.1	TEC	TEC		17	51183191	51186403
ENSG0000021392	-0.961221223	0.013	0.03	IRF9	protein_coding	interferon re		14	24161265	24166565
ENSG0000013971	-0.219820552	0.013	0.03	VPS33A	protein_coding	VPS33A cor		12	122229564	122266494
ENSG0000023748	1.555122115	0.013	0.03	C10orf143	protein_coding	chromosom		10	130020025	130110830
ENSG0000025522	1.166828009	0.013	0.03	AC109322.1	lncRNA	TEC		8	144078002	144079265
ENSG0000017902	-0.287271067	0.013	0.03	C3orf38	protein_coding	chromosom		3	88149959	88168729
ENSG0000016814	0.336599973	0.013	0.03	VASN	protein_coding	vasorin [Sou		16	4371848	4383538
ENSG0000014129	-2.070239171	0.013	0.03	LRRC46	protein_coding	leucine rich		17	47831634	47837719
ENSG0000027323	-0.867401837	0.013	0.03	NA	NA	NA	NA	NA	NA	
ENSG0000009047	0.275205716	0.013	0.03	PDCD7	protein_coding	programme		15	65117379	65133808
ENSG0000017031	0.139235176	0.013	0.03	UBB	protein_coding	ubiquitin B [		17	16380798	16382745
ENSG0000015816	-0.242458475	0.013	0.03	FANCC	protein_coding	FA complem		9	95099054	95426796
ENSG0000011575	-0.291821357	0.013	0.03	TAF1B	protein_coding	TATA-box b		2	9843443	9934416
ENSG0000026568	3.97987675	0.013	0.03	SYPL1P2	processed_pse	synaptophy		17	27351858	27352584
ENSG0000017301	-0.288252006	0.013	0.03	TADA2B	protein_coding	transcription		4	7041899	7057952
ENSG0000018219	0.495743065	0.013	0.03	LDOC1	protein_coding	LDOC1 regu	X		141111605	141177129
ENSG0000018420	0.399921146	0.013	0.03	SNRNP35	protein_coding	small nuclea		12	123458088	123473154
ENSG0000026929	0.821877836	0.013	0.03	ZSCAN16-A	lncRNA	ZSCAN16 an		6	28015122	28137293
ENSG0000015187	-0.878423355	0.013	0.03	FBXO4	protein_coding	F-box prote		5	41925254	41941743
ENSG0000022731	0.760038252	0.013	0.03	NEU1	protein_coding	neuraminida	CHR_HSCHR		31845195	31850443
ENSG0000018045	1.42849056	0.013	0.03	AC022148.1	lncRNA	novel transc		19	37545470	37549171
ENSG0000018613	0.527140484	0.013	0.03	C2orf76	protein_coding	chromosom		2	119302225	119366834
ENSG0000017083	-0.175645997	0.013	0.03	USP32	protein_coding	ubiquitin sp		17	60177327	60422470
ENSG0000017817	-0.213397575	0.013	0.03	LCORL	protein_coding	ligand depe		4	17841199	18021876
ENSG0000027917	-2.005506854	0.013	0.03	CTBP2P9	processed_pse	CTBP2 pseu		21	7626344	7627528
ENSG0000018873	-0.203122006	0.013	0.03	RBM34	protein_coding	RNA binding		1	235131183	235161283
ENSG0000014036	-1.437509384	0.013	0.03	PSTPIP1	protein_coding	proline-seri		15	76993359	77037475
ENSG0000027980	-0.367424865	0.013	0.03	BCLAF1P2	processed_pse	BCL2 associ		16	34266041	34268649
ENSG0000010278	-0.308225169	0.013	0.03	KATNAL1	protein_coding	katanin cata		13	30202630	30307551
ENSG0000027501	-0.318657706	0.013	0.03	AL121581.1	protein_coding	novel transc	CHR_HSCHR		64211718	64278487
ENSG0000016741	-3.101039703	0.013	0.03	GNG8	protein_coding	G protein su		19	46634076	46634685
ENSG0000017541	0.312281943	0.013	0.03	CLTB	protein_coding	clathrin ligh		5	176392455	176416569
ENSG0000018821	-0.332159066	0.013	0.03	DCUN1D3	protein_coding	defective in		16	20854925	20900358
ENSG0000022020	-1.3100309	0.013	0.03	ZGLP1	protein_coding	zinc finger (		19	10304803	10309880
ENSG0000022785	2.826369739	0.013	0.03	AL358075.2	lncRNA	novel transc		1	46134531	46139081
ENSG0000006317	-0.141303394	0.013	0.03	RPL18	protein_coding	ribosomal p		19	48615328	48619184
ENSG0000027729	0.174427505	0.013	0.03	PIP4K2B	protein_coding	phosphatidy	CHR_HSCHR		38566476	38600913
ENSG0000015906	-0.234779681	0.013	0.03	FBXW5	protein_coding	F-box and V		9	136940435	136944738
ENSG0000025874	1.783395307	0.014	0.03	AL359399.1	lncRNA	novel transc		14	104137150	104137898
ENSG0000027576	1.159450707	0.014	0.03	AC091982.3	lncRNA	novel transc		5	151770242	151771508
ENSG0000022921	0.619717885	0.014	0.03	AC044860.1	transcribed_un	golgin subfa		15	85180200	85234795
ENSG0000018328	0.164979958	0.014	0.03	DAZAP2	protein_coding	DAZ associa		12	51238724	51271362
ENSG0000027473	0.484445181	0.014	0.03	NA	NA	NA	NA	NA	NA	

ENSG0000013538	0.099084019	0.014	0.03	CAPRIN1	protein_coding	cell cycle as	11	34051731	34102610
ENSG0000027572	-3.396874692	0.014	0.03	NDUFA3	protein_coding	NADH:ubiqu	CHR_HSCHR	54102906	54107026
ENSG0000027915	1.522953507	0.014	0.03	AC003681.1	lncRNA	novel transc	22	29978950	30028236
ENSG0000018671	-0.229722607	0.014	0.03	BCR	protein_coding	BCR activato	22	23179704	23318037
ENSG0000025452	1.10947076	0.014	0.03	AP000757.1	lncRNA	novel transc	11	117833719	117838942
ENSG0000014934	0.401865323	0.014	0.03	SLX4IP	protein_coding	SLX4 interac	20	10435305	10636829
ENSG0000024774	1.237556366	0.014	0.03	USP51	protein_coding	ubiquitin sp	X	55484616	55489202
ENSG0000027322	-1.223691985	0.014	0.03	AL391834.2	lncRNA	novel transc	9	19375451	19375996
ENSG0000012739	-0.299692922	0.014	0.03	LRRC61	protein_coding	leucine rich	7	150323263	150338156
ENSG0000016545	-0.162168603	0.014	0.03	INPPL1	protein_coding	inositol poly	11	72223701	72239147
ENSG0000023522	0.958303226	0.014	0.03	MSH5	protein_coding	mutS homo	CHR_HSCHR	31727390	31750052
ENSG0000011752	0.10993694	0.014	0.03	PRRC2C	protein_coding	proline rich	1	171485551	171593511
ENSG0000004351	-0.338817712	0.014	0.03	TRIT1	protein_coding	tRNA isoper	1	39838110	39883511
ENSG0000018541	0.19882824	0.014	0.03	MRPL30	protein_coding	mitochondr	2	99181152	99199561
ENSG0000013843	-0.2834987	0.014	0.03	CIR1	protein_coding	corepressor	2	174348022	174395712
ENSG0000011183	-0.205840379	0.014	0.03	RWDD1	protein_coding	RWD domai	6	116571409	116597675
ENSG0000023735	0.776580765	0.014	0.03	BX088651.4	lncRNA	novel transc	9	42566679	42569353
ENSG0000008083	0.297735516	0.014	0.03	RBL1	protein_coding	RB transcrip	20	36996349	37095997
ENSG0000026165	-0.966863717	0.014	0.03	C15orf65	protein_coding	chromosom	15	55408495	55418798
ENSG0000018366	0.454126637	0.014	0.03	TRMT12	protein_coding	tRNA methy	8	124450820	124462150
ENSG0000026840	4.806286504	0.014	0.03	AC008763.2	protein_coding	novel transc	19	7629796	7643048
ENSG0000013568	-0.452332278	0.014	0.03	KLHL36	protein_coding	kelch like fa	16	84648511	84667686
ENSG0000017802	0.292906786	0.014	0.03	DMAP1	protein_coding	DNA methy	1	44213455	44220681
ENSG0000016901	0.391568723	0.014	0.03	COMMD8	protein_coding	COMM dom	4	47450787	47463702
ENSG0000021712	-0.202317511	0.014	0.03	FNIP1	protein_coding	folliculin int	5	131641714	131797063
ENSG0000015172	0.208949124	0.014	0.03	SLC25A4	protein_coding	solute carrie	4	185143266	185150382
ENSG0000022721	2.213138263	0.014	0.03	AC079145.1	lncRNA	novel transc	2	19990209	20004795
ENSG0000017528	-2.181971679	0.014	0.03	PHYHD1	protein_coding	phytanoyl-C	9	128920966	128942041
ENSG0000018470	-0.25347456	0.014	0.03	EIF4ENIF1	protein_coding	eukaryotic t	22	31436977	31496108
ENSG0000027799	0.545249809	0.014	0.03	FP236241.1	lncRNA	uncharacter	21	7669397	7681742
ENSG0000012080	-0.204601262	0.014	0.03	ARL1	protein_coding	ADP ribosyl	12	101393116	101407772
ENSG0000017063	0.716595563	0.014	0.03	ACYP2	protein_coding	acylphospha	2	53970838	54305300
ENSG0000018494	0.463323059	0.014	0.03	FAM227A	protein_coding	family with s	22	38578120	38656629
ENSG0000015090	-1.074936634	0.014	0.03	FOXO1	protein_coding	forkhead bo	13	40555667	40666641
ENSG0000015132	0.244800814	0.014	0.03	FAM177A1	protein_coding	family with s	14	35044907	35113130
ENSG0000011740	0.224769602	0.014	0.03	IPO13	protein_coding	importin 13	1	43946950	43968022
ENSG0000012312	0.28104268	0.014	0.03	WWP1	protein_coding	WW domair	8	86342547	86478420
ENSG0000022575	-1.546951249	0.014	0.03	DBH-AS1	lncRNA	DBH antiser	9	133654586	133657313
ENSG0000013698	-0.173525959	0.014	0.03	DERL1	protein_coding	derlin 1 [Sou	8	123013170	123042302
ENSG0000018434	-0.275654658	0.014	0.03	EFNA5	protein_coding	ephrin A5 [S	5	107376889	107670937
ENSG0000027281	1.245721083	0.014	0.03	AC004908.2	lncRNA	novel transc	8	233119	233692
ENSG0000016562	0.120818325	0.014	0.03	ATP5F1C	protein_coding	ATP synthas	10	7788147	7807815
ENSG0000025391	0.785986212	0.014	0.03	PCDHGB2	protein_coding	protocadhe	5	141360042	141512979
ENSG0000015944	-0.388307814	0.014	0.03	THEM4	protein_coding	thioesterase	1	151870866	151909637
ENSG0000011498	0.357429407	0.014	0.03	LMAN2L	protein_coding	lectin, manr	2	96705929	96740064
ENSG0000012853	0.263843821	0.014	0.03	LSM8	protein_coding	LSM8 homo	7	118184144	118204035
ENSG0000010800	0.250010796	0.014	0.03	EBF3	protein_coding	EBF transcri	10	129835233	129973053
ENSG0000010226	0.366445129	0.014	0.03	TIMP1	protein_coding	TIMP metall	X	47582408	47586789
ENSG0000011536	0.180080618	0.014	0.03	WDR75	protein_coding	WD repeat	2	189441446	189475552
ENSG0000011880	1.37959129	0.014	0.03	STBD1	protein_coding	starch bindi	4	76306026	76311599
ENSG0000017413	0.649016888	0.014	0.03	FAM174A	protein_coding	family with s	5	100535374	100586741
ENSG0000025898	2.886282262	0.014	0.03	AL133523.1	lncRNA	novel transc	14	100207407	100238555
ENSG0000016612	0.253411165	0.014	0.03	RAB8B	protein_coding	RAB8B, mer	15	63189560	63267776
ENSG0000021411	-2.246258688	0.014	0.03	LDHAP4	transcribed_pr	lactate dehy	9	14921337	14922334
ENSG0000015333	0.264985443	0.014	0.03	TRAPPC8	protein_coding	trafficking p	18	31829180	31953136
ENSG0000027945	-0.420202516	0.014	0.03	WASH9P	unprocessed_p	WAS protei	1	185217	195411
ENSG0000011452	0.208827472	0.014	0.03	SNX4	protein_coding	sorting nexi	3	125446650	125520202
ENSG0000017344	0.534043496	0.014	0.03	EHBP1L1	protein_coding	EH domain	11	65576046	65592650

ENSG0000027174	1.394634693	0.014	0.03	AF287957.1	lncRNA	novel transc		8	6615604	6617198
ENSG0000022980	-0.565757344	0.014	0.04	ZNF688	protein_coding	zinc finger p		16	30569346	30572734
ENSG0000010254	0.604526915	0.015	0.04	CDADC1	protein_coding	cytidine anc		13	49247925	49293485
ENSG0000024942	0.761767486	0.015	0.04	ADAMTS19	lncRNA	ADAMTS19		5	129424782	129461076
ENSG0000009579	-0.449450242	0.015	0.04	CREM	protein_coding	cAMP respo		10	35126791	35212958
ENSG0000028010	-1.87357862	0.015	0.04	NA	NA	NA	NA	NA	NA	
ENSG0000017718	-0.168868785	0.015	0.04	RPS6KA3	protein_coding	ribosomal p	X		20149911	20267519
ENSG0000023216	-0.355072433	0.015	0.04	ABCF1	protein_coding	ATP binding	CHR_HSCHR		30561610	30587416
ENSG0000010185	0.202864091	0.015	0.04	PGRMC1	protein_coding	progesteror	X		119236245	119244466
ENSG0000018586	-0.392739981	0.015	0.04	NPIPB4	protein_coding	nuclear pore		16	21834569	21880827
ENSG0000017026	-0.340772598	0.015	0.04	GLB1	protein_coding	galactosidas		3	32996609	33097202
ENSG0000027965	-2.303677718	0.015	0.04	AL132780.4	TEC	TEC		14	23023083	23024217
ENSG0000018461	-0.191070116	0.015	0.04	NELL2	protein_coding	neural EGFL		12	44508275	44921848
ENSG0000017022	-0.550566561	0.015	0.04	ADPRM	protein_coding	ADP-ribose		17	10697594	10711558
ENSG0000027397	-1.563036365	0.015	0.04	NA	NA	NA	NA	NA	NA	
ENSG0000009285	-2.90209862	0.015	0.04	TEKT2	protein_coding	tektin 2 [Sou		1	36084094	36088275
ENSG0000018261	-2.271658956	0.015	0.04	TSPAN10	protein_coding	tetraspanin		17	81637171	81648749
ENSG0000006562	1.609047365	0.015	0.04	GSTO2	protein_coding	glutathione		10	104268873	104304950
ENSG0000018878	-0.304210263	0.015	0.04	MTF1	protein_coding	metal regula		1	37809574	37859592
ENSG0000023125	-0.484574669	0.015	0.04	ANAPC1P2	unprocessed_p	ANAPC1 pse		2	87031815	87052992
ENSG0000015028	-2.192132751	0.015	0.04	CTF1	protein_coding	cardiotroph		16	30896614	30903547
ENSG0000012925	-0.502408591	0.015	0.04	KIF1C	protein_coding	kinesin fami		17	4997950	5028401
ENSG0000015130	0.332712407	0.015	0.04	SRFBP1	protein_coding	serum respo		5	121961975	122075570
ENSG0000010177	-0.229833941	0.015	0.04	RBBP8	protein_coding	RB binding		18	22798261	23026488
ENSG0000025822	-1.981982605	0.015	0.04	PRSS58	protein_coding	serine prote		7	142252143	142258058
ENSG0000027922	2.187394771	0.015	0.04	AC009303.4	TEC	novel transc		2	118014174	118015673
ENSG0000019626	-0.884298684	0.015	0.04	ZNF836	protein_coding	zinc finger p		19	52153864	52171643
ENSG0000001001	0.162427175	0.015	0.04	RANBP9	protein_coding	RAN binding		6	13621498	13711835
ENSG0000020545	1.506251396	0.015	0.04	TP53TG3C	protein_coding	TP53 target		16	33193659	33196858
ENSG0000018120	2.577923991	0.015	0.04	H2BU2P	unitary_pseudo	H2B.U histo		1	228464103	228464626
ENSG0000015613	0.240465241	0.015	0.04	DCK	protein_coding	deoxycytidir		4	70992538	71030914
ENSG0000015348	0.306037924	0.015	0.04	ING1	protein_coding	inhibitor of		13	110712736	110723339
ENSG0000010497	0.224140462	0.015	0.04	C19orf53	protein_coding	chromosom		19	13774456	13778773
ENSG0000018106	-0.244624756	0.015	0.04	HIGD1A	protein_coding	HIG1 hypox		3	42782908	42804490
ENSG0000010999	-1.926250543	0.015	0.04	P2RX3	protein_coding	purinergic r		11	57338352	57372396
ENSG0000010794	0.182450526	0.015	0.04	BCCIP	protein_coding	BRCA2 and		10	125823546	125853695
ENSG0000015581	-0.757334834	0.015	0.04	FMN2	protein_coding	formin 2 [Sc		1	240014348	240475187
ENSG0000019668	-1.489686967	0.015	0.04	TRPV1	protein_coding	transient rec		17	3565444	3609411
ENSG0000013693	0.277769339	0.015	0.04	GOLGA1	protein_coding	golgin A1 [S		9	124878275	124948492
ENSG0000026300	-0.493253004	0.015	0.04	ZNF234	protein_coding	zinc finger p		19	44141554	44160313
ENSG0000014254	0.320720353	0.016	0.04	NOSIP	protein_coding	nitric oxide		19	49555468	49590262
ENSG0000015721	0.230743864	0.016	0.04	PAXIP1	protein_coding	PAX interac		7	154943687	155003124
ENSG0000017618	-0.83864103	0.016	0.04	MYPOP	protein_coding	Myb relatec		19	45890023	45902613
ENSG0000024637	0.760705591	0.016	0.04	AC040168.1	lncRNA	novel transc		16	55984009	56191118
ENSG0000012748	-0.120660254	0.016	0.04	UBR4	protein_coding	ubiquitin pr		1	19074510	19210266
ENSG0000006554	0.153689081	0.016	0.04	ZC3H15	protein_coding	zinc finger C		2	186486253	186509361
ENSG0000019878	-0.262977315	0.016	0.04	ZNF830	protein_coding	zinc finger p		17	34961540	34963777
ENSG0000014405	-0.416382696	0.016	0.04	ST6GAL2	protein_coding	ST6 beta-ga		2	106801600	106887108
ENSG0000007361	0.141818373	0.016	0.04	KDM5A	protein_coding	lysine deme		12	280057	389320
ENSG0000017136	-0.185828954	0.016	0.04	CLCN5	protein_coding	chloride vol	X		49922596	50099235
ENSG0000016021	-0.301846114	0.016	0.04	CSTB	protein_coding	cystatin B [S		21	43772511	43776330
ENSG0000026207	3.156616199	0.016	0.04	SNORD3B-	snoRNA	small nucle		17	19063346	19064136
ENSG0000013983	-2.03652676	0.016	0.04	GRTP1	protein_coding	growth horr		13	113324163	113364148
ENSG0000012425	-1.988569397	0.016	0.04	NEURL2	protein_coding	neuralized E		20	45888625	45891287
ENSG0000017276	0.306910753	0.016	0.04	TMCC1	protein_coding	transmembr		3	129647792	129893606
ENSG0000005699	-0.969078471	0.016	0.04	GYG2	protein_coding	glycogenin	X		2828822	2882820
ENSG0000027055	-1.752322099	0.016	0.04	AC025449.1	processed_pse	chromobox		5	37286449	37286977
ENSG0000027127	-5.528647805	0.016	0.04	AC007326.1	processed_pse	POM121 tra		22	18997138	18997595

ENSG0000016798	0.526885015	0.016	0.04	ZNF597	protein_coding	zinc finger p		16	3432414	3443504
ENSG0000022653	0.872212943	0.016	0.04	C6orf47	protein_coding	chromosom	CHR_HSCHR		31734577	31737051
ENSG0000011603	0.130459826	0.016	0.04	SUMO1	protein_coding	small ubiqui		2	202206182	202238599
ENSG0000025022	2.644070564	0.016	0.04	AC008443.4	lncRNA	novel transc		5	181191875	181194429
ENSG0000026030	-1.307082612	0.016	0.04	AC092375.2	lncRNA	novel transc		16	21794095	21795759
ENSG0000013387	0.363135658	0.016	0.04	RNF122	protein_coding	ring finger p		8	33547754	33567128
ENSG0000018090	0.402799368	0.016	0.04	D2HGDH	protein_coding	D-2-hydrox		2	241734602	241768816
ENSG0000011942	0.230999122	0.016	0.04	NDUFA8	protein_coding	NADH:ubiqu		9	122144058	122159779
ENSG0000016299	-0.97651822	0.016	0.04	NEUROD1	protein_coding	neuronal dif		2	181673088	181680547
ENSG0000027571	5.499074682	0.016	0.04	H3C1	protein_coding	H3 clustered		6	26020451	26020958
ENSG0000017497	-0.796742507	0.016	0.04	AC026271.1	processed_pse	poly(A) binc		17	18650195	18651542
ENSG0000013682	0.491369062	0.016	0.04	RALGPS1	protein_coding	Ral GEF with		9	126914774	127223166
ENSG0000013290	0.616609233	0.016	0.04	CASP9	protein_coding	caspase 9 [S		1	15490832	15526534
ENSG0000014343	0.194232196	0.016	0.04	ARNT	protein_coding	aryl hydroca		1	150809713	150876708
ENSG0000013868	-0.175859023	0.016	0.04	KIAA1109	protein_coding	KIAA1109 [S		4	122152333	122362758
ENSG0000027657	1.375813677	0.016	0.04	AC010327.6	lncRNA	novel transc		19	55227219	55230279
ENSG0000022638	-0.95849534	0.016	0.04	GTF2H4	protein_coding	general trar	CHR_HSCHR		30898895	30904817
ENSG0000023375	-0.776311717	0.016	0.04	AC092835.1	protein_coding	novel C2H2		2	95207535	95259774
ENSG0000021361	-0.251679565	0.016	0.04	HEXA	protein_coding	hexosaminic		15	72340924	72376420
ENSG0000028025	1.681218179	0.016	0.04	AC233723.2	TEC	TEC		17	4787057	4789162
ENSG0000024568	-0.388454104	0.016	0.04	ZNF585B	protein_coding	zinc finger p		19	37181579	37218153
ENSG0000012319	0.582523598	0.016	0.04	ATP7B	protein_coding	ATPase cop		13	51930436	52012125
ENSG0000022196	-0.468546312	0.016	0.04	FADS3	protein_coding	fatty acid de		11	61873519	61892051
ENSG0000015393	-0.52784019	0.016	0.04	DGKE	protein_coding	diacylglycer		17	56834107	56869567
ENSG0000018170	-0.180476231	0.016	0.04	YIPF6	protein_coding	Yip1 domair	X		68498562	68537282
ENSG0000019619	-0.206460744	0.016	0.04	MPHOSPH8	protein_coding	M-phase ph		13	19633659	19673441
ENSG0000010222	-0.248877403	0.016	0.04	JADE3	protein_coding	jade family	X		46912276	47061242
ENSG0000024935	-3.550180786	0.016	0.04	LINC02198	lncRNA	long interge		5	68970692	69030165
ENSG0000026559	-3.305228212	0.016	0.04	CFAP298-T	protein_coding	CFAP298-TC		21	32402511	32612865
ENSG0000016569	0.772965724	0.016	0.04	SPACA9	protein_coding	sperm acros		9	132878027	132890201
ENSG0000022487	0.399599217	0.016	0.04	NDUFAF8	protein_coding	NADH:ubiqu		17	81239305	81241310
ENSG0000026097	-1.952980899	0.016	0.04	AC009021.2	lncRNA	novel transc		16	22610531	22612196
ENSG0000013220	-0.671290976	0.016	0.04	EMILIN2	protein_coding	elastin micr		18	2847006	2916003
ENSG0000015554	0.586119118	0.016	0.04	SETD9	protein_coding	SET domain		5	56909260	56925532
ENSG0000006830	0.153966447	0.016	0.04	MEF2A	protein_coding	myocyte en		15	99565417	99716466
ENSG0000023938	1.523206792	0.016	0.04	PCDHA13	protein_coding	protocadhe		5	140882124	141012347
ENSG0000010635	0.536873501	0.016	0.04	AGFG2	protein_coding	ArfGAP with		7	100539203	100568220
ENSG0000013575	-0.65376682	0.016	0.04	KCNK1	protein_coding	potassium t		1	233614106	233672514
ENSG0000026715	-1.34808442	0.016	0.04	AC093227.1	lncRNA	novel transc		19	37728586	37730643
ENSG0000011960	-1.48898478	0.016	0.04	PROX2	protein_coding	prospero hc		14	74852871	74876154
ENSG0000004757	-0.649181918	0.016	0.04	DTNBP1	protein_coding	dystrobrevir		6	15522807	15663058
ENSG0000014402	0.096953082	0.016	0.04	SNRNP200	protein_coding	small nuclea		2	96274338	96321271
ENSG0000008584	0.321859391	0.016	0.04	ORC1	protein_coding	origin recog		1	52372829	52404423
ENSG0000022447	0.194975866	0.016	0.04	ATXN1L	protein_coding	ataxin 1 like		16	71845976	71885268
ENSG0000018320	0.229048221	0.016	0.04	RUVBL2	protein_coding	RuvB like A/		19	48993562	49015970
ENSG0000011536	-0.822824999	0.016	0.04	EVA1A	protein_coding	eva-1 homc		2	75469302	75569722
ENSG0000023263	0.934697472	0.016	0.04	PRPS1P2	processed_pse	phosphoribc		9	125150653	125151589
ENSG0000021542	-0.527631655	0.017	0.04	MCM3AP-A	lncRNA	MCM3AP ar		21	46229196	46259390
ENSG0000027355	-0.40185218	0.017	0.04	CWC25	protein_coding	CWC25 spli		17	38800441	38825355
ENSG0000027441	-1.056903279	0.017	0.04	TBC1D3D	protein_coding	TBC1 doma		17	38003976	38014902
ENSG0000013225	-0.223167356	0.017	0.04	ARFIP2	protein_coding	ADP ribosyl		11	6474683	6481479
ENSG0000014717	-1.011320832	0.017	0.04	GCNA	protein_coding	germ cell nu	X		71578411	71613583
ENSG0000018426	-1.023727079	0.017	0.04	KCNK12	protein_coding	potassium t		2	47509290	47570985
ENSG0000000000	-0.293390628	0.017	0.04	TSPAN6	protein_coding	tetraspanin	X		100627108	100639991
ENSG0000015876	0.863278446	0.017	0.04	F11R	protein_coding	F11 recepto		1	160995211	161021343
ENSG0000012144	0.298434943	0.017	0.04	PDZRN3	protein_coding	PDZ domair		3	73382431	73624941
ENSG0000023630	5.480513607	0.017	0.04	EEF1E1P1	processed_pse	eukaryotic t		2	111887914	111888741
ENSG0000016739	0.213101959	0.017	0.04	ZNF646	protein_coding	zinc finger p		16	31074422	31084196

ENSG0000016889	-1.006854022	0.017	0.04	VAMP5	protein_codinç	vesicle asso	2	85584431	85593406
ENSG0000017261	0.372392497	0.017	0.04	RAD9A	protein_codinç	RAD9 check	11	67317871	67398410
ENSG0000016622	-0.160216356	0.017	0.04	SGPL1	protein_codinç	sphingosine	10	70815948	70881184
ENSG0000010029	-0.410154127	0.017	0.04	MCAT	protein_codinç	malonyl-Co	22	43132209	43143398
ENSG0000012718	-0.144227043	0.017	0.04	COX7C	protein_codinç	cytochrome	5	86617928	86620962
ENSG0000019795	0.4666062	0.017	0.04	ZNF71	protein_codinç	zinc finger p	19	56595300	56626481
ENSG0000017029	-0.171688243	0.017	0.04	GABARAP	protein_codinç	GABA type	17	7240008	7242449
ENSG0000023102	-0.480787902	0.017	0.04	NA	NA	NA	NA	NA	NA
ENSG0000023538	-4.678040215	0.017	0.04	AC113174.	unprocessed_p	pseudogene	1	246818076	246818180
ENSG0000019651	0.167299047	0.017	0.04	ANAPC7	protein_codinç	anaphase pr	12	110372900	110403730
ENSG0000023778	2.181074715	0.017	0.04	ADAMTSL4	lncRNA	ADAMTSL4	1	150548562	150557724
ENSG0000011357	0.122874311	0.017	0.04	PPP2CA	protein_codinç	protein pho	5	134194332	134226073
ENSG0000011004	-0.265426152	0.017	0.04	EHD1	protein_codinç	EH domain	11	64851642	64888296
ENSG0000020570	2.667921781	0.017	0.04	CYP2D7	polymorphic_p	cytochrome	22	42140203	42149455
ENSG0000027231	-0.607478752	0.017	0.04	AL021368.2	lncRNA	novel transc	6	57908560	57913911
ENSG0000022348	1.01299051	0.017	0.04	AC092198.	lncRNA	novel transc	X	40009276	40012182
ENSG0000023619	-0.864140974	0.017	0.04	NFKBIL1	protein_codinç	NFKB inhibi	CHR_HSCHR	31529043	31540993
ENSG0000018909	-0.117732367	0.017	0.04	SF3B3	protein_codinç	splicing fact	16	70523791	70577670
ENSG0000013714	-0.16233905	0.017	0.04	DENND4C	protein_codinç	DENN doma	9	19230435	19373545
ENSG0000019781	-0.966936129	0.017	0.04	CCDC180	protein_codinç	coiled-coil d	9	97307304	97378751
ENSG0000011584	-0.647912821	0.017	0.04	RMDN2	protein_codinç	regulator of	2	37923187	38067142
ENSG0000012676	0.227674708	0.017	0.04	ELK1	protein_codinç	ETS transcri	X	47635521	47650604
ENSG0000019633	-1.335830951	0.017	0.04	NLGN3	protein_codinç	neuroligin 3	X	71144831	71171201
ENSG0000026736	1.144718361	0.017	0.04	UPK3BL1	protein_codinç	uroplakin 3E	7	102637025	102642791
ENSG0000007268	0.476444869	0.017	0.04	P4HA2	protein_codinç	prolyl 4-hyc	5	132191838	132295315
ENSG0000023524	1.716021716	0.017	0.04	AL360181.2	lncRNA	novel transc	10	133295187	133295977
ENSG0000018551	-4.106527553	0.017	0.04	FAM131C	protein_codinç	family with s	1	16057769	16073651
ENSG0000002390	-0.187759528	0.017	0.04	PLEKHO1	protein_codinç	pleckstrin ho	1	150149183	150164720
ENSG0000014452	-0.192111748	0.017	0.04	COPS7B	protein_codinç	COP9 signa	2	231781671	231809254
ENSG0000025887	-2.875137854	0.017	0.04	AL135818.1	lncRNA	novel transc	14	91242759	91252211
ENSG0000027960	0.518428458	0.017	0.04	AC067930.5	TEC	TEC	8	143578358	143583304
ENSG0000006891	0.197301244	0.017	0.04	ERLEC1	protein_codinç	endoplasmi	2	53787044	53818819
ENSG0000026176	0.547230854	0.017	0.04	AC027228.2	lncRNA	novel transc	15	78589123	78591276
ENSG0000014421	-0.88801589	0.017	0.04	AFF3	protein_codinç	AF4/FMR2 f	2	99545419	100192428
ENSG0000026378	1.119086215	0.017	0.04	AC022211.	lncRNA	novel transc	17	75145261	75146546
ENSG0000018500	0.149125166	0.017	0.04	AP3M1	protein_codinç	adaptor rela	10	74120255	74151063
ENSG0000027884	1.442624681	0.017	0.04	TP53TG3F	protein_codinç	TP53 target	16	33459045	33462249
ENSG0000014624	0.115930049	0.017	0.04	PHIP	protein_codinç	pleckstrin ho	6	78934419	79078254
ENSG0000022794	0.934503609	0.017	0.04	AC007383.	lncRNA	novel transc	2	206084605	206086564
ENSG0000008838	-0.464617544	0.017	0.04	DOCK9	protein_codinç	dedicator of	13	98793429	99086625
ENSG0000017479	-1.252886075	0.017	0.04	RIN1	protein_codinç	Ras and Rab	11	66330241	66336840
ENSG0000007578	-0.121715227	0.017	0.04	RAB7A	protein_codinç	RAB7A, mer	3	128693669	128825942
ENSG0000022821	-1.34862352	0.017	0.04	ATF4P3	processed_pse	activating tr	17	76225751	76226806
ENSG0000025366	-1.333759195	0.017	0.04	ZFHX4-AS1	lncRNA	ZFHX4 antis	8	76491200	76683308
ENSG0000017168	-0.146623021	0.017	0.04	ATF7IP	protein_codinç	activating tr	12	14365632	14502935
ENSG0000021393	3.885171386	0.017	0.04	AC091153.	processed_pse	ribosomal p	17	4704816	4705217
ENSG0000023244	-3.015415379	0.017	0.04	AC010745.4	lncRNA	novel transc	2	16316324	16319566
ENSG0000017495	0.155340344	0.017	0.04	DHX36	protein_codinç	DEAH-box l	3	154272546	154324487
ENSG0000020456	0.531220764	0.017	0.04	DHX16	protein_codinç	DEAH-box l	6	30653119	30673006
ENSG0000005513	-0.166240491	0.018	0.04	CUL1	protein_codinç	cullin 1 [Sou	7	148697914	148801110
ENSG0000013385	0.168246364	0.018	0.04	ZFC3H1	protein_codinç	zinc finger C	12	71609599	71667725
ENSG0000018294	0.099895876	0.018	0.04	EWSR1	protein_codinç	EWS RNA b	22	29268009	29300525
ENSG0000020548	-0.723142023	0.018	0.04	AC004980.	transcribed_un	pseudogene	7	76549360	76627982
ENSG0000026095	-2.539102705	0.018	0.04	AC005100.	lncRNA	novel transc	7	25358235	25432202
ENSG0000015637	-0.381014663	0.018	0.04	PCGF6	protein_codinç	polycomb g	10	103302796	103351144
ENSG0000018724	-0.262994062	0.018	0.04	MAGED4B	protein_codinç	MAGE famil	X	52061827	52069248
ENSG0000026291	-2.155090234	0.018	0.04	DNAJC28	protein_codinç	DnaJ heat sl	CHR_HSCHR	33495087	33501278
ENSG0000011896	0.187024089	0.018	0.04	LDAH	protein_codinç	lipid droplet	2	20684014	20823130

ENSG0000015141	0.431162715	0.018	0.04	NUBPL	protein_coding	nucleotide b	14	31489956	31861224
ENSG0000003965	0.389252447	0.018	0.04	PNKP	protein_coding	polynucleot	19	49859882	49878351
ENSG0000013627	-0.198309767	0.018	0.04	DDX56	protein_coding	DEAD-box l	7	44565417	44575051
ENSG0000023694	-0.890240064	0.018	0.04	ZNRD1	protein_coding	zinc ribbon	CHR_HSCHR	30049228	30055232
ENSG0000016918	-1.22037151	0.018	0.04	GSG1L	protein_coding	GSG1 like [S	16	27787528	28063714
ENSG0000014338	0.12697277	0.018	0.04	MCL1	protein_coding	MCL1 apop	1	150574551	150579738
ENSG0000025770	1.373005089	0.018	0.04	INAFM1	protein_coding	InaF motif c	19	47274885	47275723
ENSG0000022521	-0.236349376	0.018	0.04	DUXAP9	transcribed_pr	double hom	14	19062316	19131167
ENSG0000016899	-0.415549545	0.018	0.04	CPLX1	protein_coding	complexin 1	4	784957	826129
ENSG0000013802	0.151897436	0.018	0.04	HADHB	protein_coding	hydroxyacyl	2	26243170	26290465
ENSG0000011691	0.162894755	0.018	0.04	TSNAX	protein_coding	translin asso	1	231528541	231566524
ENSG0000019889	-1.572151105	0.018	0.04	SHISA4	protein_coding	shisa family	1	201888680	201892587
ENSG0000010214	0.101382985	0.018	0.04	PGK1	protein_coding	phosphogly	X	77910739	78129295
ENSG0000017067	-0.240131439	0.018	0.04	SOCS6	protein_coding	suppressor	18	70289045	70330199
ENSG0000007754	-0.143038616	0.018	0.04	CAPZB	protein_coding	capping act	1	19338775	19485539
ENSG0000007072	-0.547240101	0.018	0.04	CNGB1	protein_coding	cyclic nucle	16	57882340	57971128
ENSG0000012833	-0.656703951	0.018	0.04	APOL2	protein_coding	apolipoprot	22	36226209	36239954
ENSG0000007880	-0.181162776	0.018	0.04	SDF4	protein_coding	stromal cell	1	1216908	1232067
ENSG0000016711	-0.240435364	0.018	0.04	URM1	protein_coding	ubiquitin rel	9	128371361	128392016
ENSG0000027210	5.551140679	0.018	0.04	AL513218.1	lncRNA	novel transc	1	52353487	52353877
ENSG0000027892	-0.590223978	0.018	0.04	AC002310.5	TEC	novel transc	16	30526918	30528294
ENSG0000013243	0.187564388	0.018	0.04	SEC61G	protein_coding	SEC61 trans	7	54752250	54759974
ENSG0000019772	-0.205442281	0.018	0.04	PHF2	protein_coding	PHD finger	9	93576584	93679587
ENSG0000024222	-3.515754043	0.018	0.04	TCP10L	protein_coding	t-complex 1	21	32497967	32587373
ENSG0000014495	0.731151833	0.018	0.04	NCEH1	protein_coding	neutral chol	3	172630249	172711218
ENSG0000020489	-0.217394996	0.018	0.04	MZT1	protein_coding	mitotic spin	13	72708367	72727629
ENSG0000017569	-1.402969465	0.018	0.04	GPR156	protein_coding	G protein-c	3	120164645	120285094
ENSG0000018580	-0.463135624	0.018	0.04	PIGP	protein_coding	phosphatidy	21	37059170	37073170
ENSG0000013274	0.281689136	0.018	0.04	IGHMBP2	protein_coding	immunoglol	11	68903863	68940602
ENSG0000010713	0.173961566	0.018	0.04	NCS1	protein_coding	neuronal ca	9	130172404	130237303
ENSG0000016593	0.147879145	0.018	0.04	CPSF2	protein_coding	cleavage an	14	92121969	92172145
ENSG0000006918	0.785003901	0.018	0.04	SDK2	protein_coding	sidekick cell	17	73334384	73644445
ENSG0000021402	0.273240691	0.018	0.04	ZNF891	protein_coding	zinc finger p	12	133106817	133130473
ENSG0000016420	0.164834332	0.018	0.04	SLC25A46	protein_coding	solute carrie	5	110738136	110765161
ENSG0000010354	0.153334756	0.018	0.04	CCP110	protein_coding	centriolar co	16	19523811	19553408
ENSG0000023515	2.804918954	0.018	0.04	AL121672.2	lncRNA	novel transc	22	46067356	46069891
ENSG0000023263	1.697724031	0.018	0.04	AL390294.1	lncRNA	novel transc	10	8051541	8053084
ENSG0000014753	-0.258221915	0.018	0.04	PLPP5	protein_coding	phospholipi	8	38263130	38269243
ENSG0000017617	0.186833457	0.018	0.04	BNIP3	protein_coding	BCL2 intera	10	131966455	131982013
ENSG0000027332	3.375856047	0.018	0.04	AC007032.1	lncRNA	novel transc	7	106285480	106286326
ENSG0000012561	0.488428748	0.018	0.04	CHCHD5	protein_coding	coiled-coil-	2	112584240	112589275
ENSG0000028018	1.288033724	0.018	0.04	AL023806.3	TEC	TEC	6	145789270	145791973
ENSG0000012840	1.103721611	0.018	0.04	RIBC2	protein_coding	RIB43A dom	22	45413693	45432509
ENSG0000014645	-0.152197248	0.018	0.04	WTAP	protein_coding	WT1 associa	6	159725585	159756319
ENSG0000019801	-1.099079166	0.019	0.04	DLGAP2	protein_coding	DLG associa	8	737628	1708476
ENSG0000014521	-0.195028557	0.019	0.04	FIP1L1	protein_coding	factor intera	4	53377641	53460862
ENSG0000015991	-0.678583831	0.019	0.04	ZNF235	protein_coding	zinc finger p	19	44228729	44305046
ENSG0000014929	-0.716307104	0.019	0.04	DRD2	protein_coding	dopamine re	11	113409605	113475691
ENSG0000010792	0.15236861	0.019	0.04	LARP4B	protein_coding	La ribonucle	10	806914	931705
ENSG0000015027	-1.76668387	0.019	0.04	PCDH15	protein_coding	protocadhe	10	53802771	55627942
ENSG0000017380	-0.205683497	0.019	0.04	JUP	protein_coding	junction pla	17	41754604	41786931
ENSG0000018836	-1.327854476	0.019	0.04	PRR19	protein_coding	proline rich	19	42302098	42310821
ENSG0000022891	2.072219501	0.019	0.04	OR7E128P	unprocessed_p	olfactory rec	11	71893410	71894433
ENSG0000013002	0.478436635	0.019	0.04	ERMARD	protein_coding	ER membra	6	169751622	169781600
ENSG0000011075	0.225488739	0.019	0.04	HPS5	protein_coding	HPS5 bioge	11	18278668	18322198
ENSG0000027060	0.751862816	0.019	0.04	AL353622.1	lncRNA	novel transc	1	28239509	28241453
ENSG0000016393	0.475565822	0.019	0.04	RFT1	protein_coding	RFT1 homol	3	53088483	53130453
ENSG0000026775	-1.609874213	0.019	0.04	AC009005.1	lncRNA	novel transc	19	567210	572228

ENSG0000026150	1.402052879	0.019	0.04	TP53TG3B	protein_coding	TP53 target		16	33360274	33363478
ENSG0000010009	0.234098888	0.019	0.04	HPS4	protein_coding	HPS4 bioge		22	26443423	26483837
ENSG0000017317	-1.092910477	0.019	0.04	ADCY5	protein_coding	adenylate cy		3	123282296	123449090
ENSG0000023499	2.884459172	0.019	0.04	AC244453.3	lncRNA	novel transc		1	121087528	121116676
ENSG0000013678	0.374823943	0.019	0.04	NIPSNAP3A	protein_coding	nipsnap hor		9	104747683	104760120
ENSG0000027818	1.12267649	0.019	0.04	RNA5-8SN	rRNA	RNA, 5.8S ri		21	8439823	8439975
ENSG0000011545	0.430493134	0.019	0.04	ELMOD3	protein_coding	ELMO doma		2	85354394	85391752
ENSG0000015212	-0.136241184	0.019	0.04	MGAT5	protein_coding	alpha-1,6-m		2	134119983	134454621
ENSG0000027110	2.917801946	0.019	0.04	SCML2P2	processed_pse	SCML2 pseu		16	25069570	25070109
ENSG0000015320	0.550332578	0.019	0.04	MERTK	protein_coding	MER proto-		2	111898607	112029561
ENSG0000020328	0.616303769	0.019	0.04	TDRKH-AS1	lncRNA	TDRKH antis		1	151790804	151794402
ENSG0000016249	-2.500678052	0.019	0.04	DHRS3	protein_coding	dehydroger		1	12567910	12618210
ENSG0000017638	-0.383456706	0.019	0.04	CDC26	protein_coding	cell division		9	113255835	113275572
ENSG0000013029	0.275312544	0.019	0.05	GTPBP3	protein_coding	GTP binding		19	17334920	17342731
ENSG0000013093	0.152994314	0.019	0.05	NOL11	protein_coding	nucleolar pr		17	67717931	67744531
ENSG0000003310	-0.182331163	0.019	0.05	CHPF2	protein_coding	chondroitin		7	151232489	151238827
ENSG0000016412	-1.716284603	0.019	0.05	HPGD	protein_coding	15-hydroxy		4	174490175	174523154
ENSG0000012317	-0.572494633	0.019	0.05	SPRYD7	protein_coding	SPRY domai		13	49912702	49936490
ENSG0000022820	-5.44241799	0.019	0.05	AC016717.1	processed_pse	similar to pa		2	225698254	225698539
ENSG0000016647	0.212166482	0.019	0.05	TMX3	protein_coding	thioredoxin		18	68673688	68715108
ENSG0000023009	2.578754751	0.019	0.05	AC108025.1	lncRNA	novel transc		2	5618327	5691118
ENSG0000018607	-0.36645661	0.019	0.05	C15orf41	protein_coding	chromosom		15	36579626	36810248
ENSG0000011941	0.167750203	0.019	0.05	PPP6C	protein_coding	protein pho		9	125146573	125189939
ENSG0000009557	-0.219282666	0.019	0.05	IKZF5	protein_coding	IKAROS fam		10	122990807	123008812
ENSG0000016896	-1.471047676	0.019	0.05	LGALS9	protein_coding	galectin 9 [S		17	27629798	27649560
ENSG0000024159	-2.827997563	0.019	0.05	AC099542.1	lncRNA	novel transc		3	81246579	81297345
ENSG0000024343	2.017938066	0.019	0.05	RPL5P30	processed_pse	ribosomal p		11	118560690	118561580
ENSG0000026795	-2.517204932	0.019	0.05	AC008878.1	protein_coding	novel transc		19	7507052	7519622
ENSG0000022801	1.493250973	0.019	0.05	AC073343.2	lncRNA	novel transc		7	6663974	6708901
ENSG0000011779	-1.441267589	0.019	0.05	MTARC2	protein_coding	mitochondr		1	220748225	220784815
ENSG0000018560	0.296932991	0.019	0.05	MRPL40	protein_coding	mitochondr		22	19431902	19436075
ENSG0000026821	-1.04582453	0.019	0.05	AC137932.3	lncRNA	novel transc		16	89268104	89273044
ENSG0000016076	0.414677889	0.019	0.05	GBAP1	transcribed_un	glucosylcera		1	155213821	155227422
ENSG0000023738	-0.774205263	0.019	0.05	HOXD-AS2	lncRNA	HOXD clust		2	176121611	176137098
ENSG0000017715	0.169171914	0.019	0.05	TALDO1	protein_coding	transaldolas		11	747415	765012
ENSG0000025889	-0.191060626	0.019	0.05	CEP95	protein_coding	centrosoma		17	64506865	64542461
ENSG0000022387	-0.919137196	0.02	0.05	NBEAP3	unprocessed_p	neurobeach		22	15852990	15893942
ENSG0000016171	0.416983785	0.02	0.05	PLCD3	protein_coding	phospholipa		17	45108959	45133354
ENSG0000017952	0.386830229	0.02	0.05	SHARPIN	protein_coding	SHANK asso		8	144098633	144108124
ENSG0000016262	-0.267695304	0.02	0.05	TYW3	protein_coding	tRNA-yW sy		1	74733152	74766678
ENSG0000022531	2.342538043	0.02	0.05	AL513327.1	lncRNA	novel transc		1	33307348	33349245
ENSG0000027862	-1.067459364	0.02	0.05	TSEN34	protein_coding	tRNA splicir	CHR_HSCHR		54190368	54194965
ENSG0000022647	-0.271183532	0.02	0.05	TMEM185B	protein_coding	transmembr		2	120217479	120223400
ENSG0000027014	-3.774649685	0.02	0.05	TERC	lncRNA	telomerase		3	169764520	169765060
ENSG0000017787	0.920260674	0.02	0.05	ZNF619	protein_coding	zinc finger p		3	40477113	40491053
ENSG0000013600	-0.957788336	0.02	0.05	ARHGEF4	protein_coding	Rho guanine		2	130836914	131047263
ENSG0000013401	-0.384339504	0.02	0.05	LOXL2	protein_coding	lysyl oxidase		8	23296897	23425328
ENSG0000009090	0.136247326	0.02	0.05	TNRC6A	protein_coding	trinucleotide		16	24610209	24827632
ENSG0000006332	0.260692461	0.02	0.05	MED29	protein_coding	mediator co		19	39391303	39400641
ENSG0000010060	-0.349179891	0.02	0.05	ALKBH1	protein_coding	alkB homolo		14	77672404	77708023
ENSG0000013733	-0.864765966	0.02	0.05	IER3	protein_coding	immediate e		6	30743199	30744548
ENSG0000014780	-0.682099385	0.02	0.05	SLC39A4	protein_coding	solute carrie		8	144409742	144416844
ENSG0000018600	0.20168297	0.02	0.05	LRCH3	protein_coding	leucine rich		3	197791226	197888436
ENSG0000020629	-0.932339092	0.02	0.05	TAP2	protein_coding	transporter	CHR_HSCHR		32750528	32767476
ENSG0000005112	-0.58805933	0.02	0.05	HOMER3	protein_coding	homer scaff		19	18929201	18941261
ENSG0000025498	0.248363515	0.02	0.05	DPP3	protein_coding	dipeptidyl p		11	66480013	66509657
ENSG0000027300	1.215951091	0.02	0.05	AC010864.1	lncRNA	novel transc		10	43136824	43138334
ENSG0000027649	1.353969089	0.02	0.05	APBA1	protein_coding	amyloid bet	CHR_HSCHR		69435727	69484339

ENSG0000023519	0.636677579	0.02	0.05	PPP1R3E	protein_coding	protein pho	14	23295652	23302859
ENSG0000024515	-0.984037769	0.02	0.05	AP001107.1	lncRNA	novel transc	11	66269832	66278525
ENSG0000027239	-0.190951527	0.02	0.05	POM121C	protein_coding	POM121 tra	7	75416786	75486299
ENSG0000012524	0.29881959	0.02	0.05	TMTC4	protein_coding	transmembr	13	100603927	100675093
ENSG0000010883	1.483933538	0.02	0.05	ALOX12	protein_coding	arachidonat	17	6996049	7010754
ENSG0000019786	-0.62934051	0.02	0.05	ZNF790	protein_coding	zinc finger p	19	36817428	36850787
ENSG0000024324	-0.577634044	0.02	0.05	STON1	protein_coding	stonin 1 [So	2	48529383	48598513
ENSG0000020533	-0.094854126	0.02	0.05	IPO7	protein_coding	importin 7 [	11	9384652	9448127
ENSG0000021309	-0.222516667	0.02	0.05	ZNF254	protein_coding	zinc finger p	19	24033405	24129968
ENSG0000016554	-0.451527799	0.02	0.05	TMEM63C	protein_coding	transmembr	14	77116568	77259495
ENSG0000017353	-0.822004495	0.02	0.05	MST1	protein_coding	macrophage	3	49683947	49689501
ENSG0000017511	0.234263605	0.02	0.05	MRPS22	protein_coding	mitochondr	3	139005806	139357223
ENSG0000019756	0.818425635	0.02	0.05	HHLA3	protein_coding	HERV-H LTI	1	70354805	70385339
ENSG0000023782	-0.565750848	0.02	0.05	RGL2	protein_coding	ral guanine	CHR_HSCHR	33269579	33277249
ENSG0000027644	-1.257526588	0.02	0.05	AC004076.2	lncRNA	novel transc	19	57449689	57453011
ENSG0000014069	-0.487931303	0.02	0.05	ARMC5	protein_coding	armadillo re	16	31458080	31467166
ENSG0000025128	2.031702431	0.02	0.05	ALG1L2	protein_coding	ALG1 chitob	3	130081831	130113227
ENSG0000014708	-4.194210004	0.02	0.05	CCNB3	protein_coding	cyclin B3 [Sc	X	50202713	50351914
ENSG0000014928	0.646003563	0.02	0.05	ZC3H12C	protein_coding	zinc finger C	11	110093392	110171841
ENSG0000019641	0.372465432	0.02	0.05	ZNF124	protein_coding	zinc finger p	1	247121975	247172016
ENSG0000013861	-0.212827147	0.02	0.05	INTS14	protein_coding	integrator c	15	65578753	65611289
ENSG0000017649	-0.269880425	0.02	0.05	DIRAS1	protein_coding	DIRAS famil	19	2714567	2721372
ENSG0000001110	-0.250907845	0.02	0.05	TSPAN9	protein_coding	tetraspanin	12	3077355	3286564
ENSG0000021512	0.429322745	0.02	0.05	CBWD6	protein_coding	COBW dom	9	41131306	41199261
ENSG0000016911	0.17357604	0.02	0.05	CSNK1G1	protein_coding	casein kinas	15	64165525	64356173
ENSG0000017589	-0.82979454	0.02	0.05	PLEKHF2	protein_coding	pleckstrin h	8	95133785	95156685
ENSG0000012102	0.173587395	0.02	0.05	COPS5	protein_coding	COP9 signa	8	67043079	67083783
ENSG0000016299	-0.569242217	0.02	0.05	CLHC1	protein_coding	clathrin hea	2	55172547	55232563
ENSG0000014378	-0.892225637	0.02	0.05	CNIH3	protein_coding	cornichon fa	1	224434660	224740554
ENSG0000010668	0.400137559	0.02	0.05	LIMK1	protein_coding	LIM domain	7	74082933	74122525
ENSG0000023380	0.373841788	0.02	0.05	NELFE	protein_coding	negative elc	CHR_HSCHR	31944741	31951746
ENSG0000023163	-1.986415522	0.02	0.05	PSMB8	protein_coding	proteasome	CHR_HSCHR	32931434	32935368
ENSG0000020435	0.533942268	0.021	0.05	SKIV2L	protein_coding	Ski2 like RN	6	31959117	31969751
ENSG0000021145	0.187482289	0.021	0.05	STK38L	protein_coding	serine/threc	12	27243968	27325959
ENSG0000025697	1.209915162	0.021	0.05	AC053513.2	lncRNA	novel transc	12	22460519	22463914
ENSG0000022488	1.956254124	0.021	0.05	EIPR1-IT1	lncRNA	EIPR1 intror	2	3298341	3301465
ENSG0000017709	-0.718522024	0.021	0.05	PHETA2	protein_coding	PH domain	22	42074248	42079438
ENSG0000018305	-0.439638726	0.021	0.05	RGPD6	protein_coding	RANBP2 like	2	110513812	110577185
ENSG0000023718	-1.276567135	0.021	0.05	NR2F1-AS1	lncRNA	NR2F1 antis	5	93360779	93585649
ENSG0000024933	-3.228085416	0.021	0.05	AC093523.1	lncRNA	novel transc	5	68792609	69007185
ENSG0000018330	0.24396291	0.021	0.05	ZNF623	protein_coding	zinc finger p	8	143636019	143656418
ENSG0000001337	0.177614956	0.021	0.05	NUB1	protein_coding	negative reg	7	151341699	151378449
ENSG0000023008	-2.466473134	0.021	0.05	PRRT3-AS1	lncRNA	PRRT3 antis	3	9947404	9954787
ENSG0000026730	-1.218534974	0.021	0.05	AC092295.2	lncRNA	novel transc	19	36489649	36491040
ENSG0000010255	-1.158053479	0.021	0.05	KLF5	protein_coding	Kruppel like	13	73054976	73077541
ENSG0000016879	0.921067716	0.021	0.05	ABHD15	protein_coding	abhydrolase	17	29560547	29567037
ENSG0000022348	0.30598429	0.021	0.05	NUTM2A-A	lncRNA	NUTM2A ar	10	87201647	87342612
ENSG0000023470	3.798869765	0.021	0.05	UPF3AP3	transcribed_pr	UPF3A pseu	9	99998301	99999069
ENSG0000019629	1.170725329	0.021	0.05	ATP2A1	protein_coding	ATPase sarc	16	28878405	28904466
ENSG0000013940	0.378555592	0.021	0.05	RITA1	protein_coding	RBPJ interac	12	113185526	113192368
ENSG0000017541	0.130447957	0.021	0.05	ARL10	protein_coding	ADP ribosyla	5	176365487	176401865
ENSG0000016872	0.216144228	0.021	0.05	DNAJC21	protein_coding	DnaJ heat sl	5	34929559	34958964
ENSG0000025620	4.742850552	0.021	0.05	AC018523.2	protein_coding	novel protei	11	14493783	14520344
ENSG0000000546	0.514322921	0.021	0.05	CROT	protein_coding	carnitine O-	7	87345664	87399794
ENSG0000012992	0.301310358	0.021	0.05	PGAP6	protein_coding	post-glycos	16	370788	387113
ENSG0000011535	0.50024042	0.021	0.05	POLE4	protein_coding	DNA polym	2	74958643	74970128
ENSG0000013801	-0.111385344	0.021	0.05	SELENOI	protein_coding	selenoprote	2	26308547	26395891
ENSG0000012514	0.273027746	0.021	0.05	C16orf70	protein_coding	chromosom	16	67109941	67148544

ENSG0000017718	-0.447262325	0.021	0.05	RIMKLA	protein_coding	ribosomal m	1	42380792	42424232
ENSG0000007371	-0.092149404	0.021	0.05	FERMT2	protein_coding	fermitin fam	14	52857268	52952435
ENSG0000022999	0.33658609	0.021	0.05	PPP1R18	protein_coding	protein pho	CHR_HSCHR	30721049	30732555
ENSG0000023810	-0.183092495	0.021	0.05	RPL9P7	processed_pse	ribosomal p	X	23836742	23837321
ENSG0000010298	-0.400467234	0.021	0.05	ZNF821	protein_coding	zinc finger p	16	71859680	71895336
ENSG0000010003	0.233271701	0.021	0.05	PPM1F	protein_coding	protein pho	22	21919425	21952848
ENSG0000010223	-0.289638614	0.021	0.05	PCYT1B	protein_coding	phosphate c	X	24558087	24672677
ENSG0000016493	-0.499264033	0.021	0.05	CTHRC1	protein_coding	collagen trip	8	103371538	103382989
ENSG0000027760	-0.931994044	0.021	0.05	CNOT3	protein_coding	CCR4-NOT	CHR_HSCHR	54138190	54156165
ENSG0000024248	0.228989346	0.021	0.05	MRPL20	protein_coding	mitochondr	1	1401909	1407293
ENSG0000018172	-1.52617917	0.021	0.05	ZBTB20	protein_coding	zinc finger a	3	114314500	115147288
ENSG0000010044	0.366890369	0.021	0.05	SDR39U1	protein_coding	short chain	14	24439766	24442905
ENSG0000027672	1.053676192	0.021	0.05	AC142472.1	lncRNA	novel transc	17	45146730	45148470
ENSG0000012999	-0.661793114	0.021	0.05	SYT5	protein_coding	synaptotagr	19	55171196	55180289
ENSG0000009830	-0.338901934	0.021	0.05	POMT2	protein_coding	protein O-m	14	77274956	77320883
ENSG0000003100	0.14429329	0.021	0.05	FAM13B	protein_coding	family with s	5	137937960	138051961
ENSG0000015113	0.63471477	0.021	0.05	C12orf45	protein_coding	chromosom	12	104986316	105074197
ENSG0000022593	-1.171923455	0.021	0.05	LINC02249	lncRNA	long interge	15	30195809	30217552
ENSG0000014473	0.261591731	0.021	0.05	SHQ1	protein_coding	SHQ1, H/AC	3	72749277	72861914
ENSG0000019615	-0.434254654	0.021	0.05	WDSUB1	protein_coding	WD repeat,	2	159235798	159286703
ENSG0000000453	0.181756982	0.021	0.05	RBM6	protein_coding	RNA binding	3	49940007	50100045
ENSG0000018555	-0.285011244	0.021	0.05	NR2F2	protein_coding	nuclear rece	15	96325938	96340263
ENSG0000018714	-0.228002839	0.022	0.05	RNF220	protein_coding	ring finger p	1	44405194	44651724
ENSG0000022513	0.556441135	0.022	0.05	DYNC1I2P1	processed_pse	dynein cyto	10	50264978	50266815
ENSG0000015214	0.305981987	0.022	0.05	GEMIN6	protein_coding	gem nuclea	2	38751534	38785002
ENSG0000000674	-1.421018375	0.022	0.05	ARHGAP44	protein_coding	Rho GTPase	17	12789498	12991643
ENSG0000008381	0.797851461	0.022	0.05	ZNF416	protein_coding	zinc finger p	19	57571566	57578911
ENSG0000018382	-1.164546548	0.022	0.05	NUDT14	protein_coding	nudix hydro	14	105172938	105181323
ENSG0000027895	-1.760248189	0.022	0.05	AP003068.4	TEC	TEC	11	65118310	65119111
ENSG0000008606	0.176448225	0.022	0.05	CHMP5	protein_coding	charged mu	9	33264879	33282070
ENSG0000016346	0.238530555	0.022	0.05	KRTCAP2	protein_coding	keratinocyte	1	155169408	155173475
ENSG0000018218	0.165703553	0.022	0.05	MRPS16	protein_coding	mitochondr	10	73248843	73252693
ENSG0000026080	-0.308473056	0.022	0.05	LINC01963	lncRNA	long interge	2	216217045	216220192
ENSG0000018329	0.165641198	0.022	0.05	SELENOF	protein_coding	selenoprote	1	86862445	86914424
ENSG0000012548	0.283712183	0.022	0.05	TTF1	protein_coding	transcription	9	132375548	132406851
ENSG0000010908	-0.185424766	0.022	0.05	TMEM97	protein_coding	transmembr	17	28319200	28328685
ENSG0000019890	-0.396268793	0.022	0.05	BHLHB9	protein_coding	basic helix-l	X	102720688	102753540
ENSG0000007715	-0.603195639	0.022	0.05	NFKB2	protein_coding	nuclear fact	10	102394110	102402524
ENSG0000027491	1.140371886	0.022	0.05	RNA5-8SN	rRNA	RNA, 5.8S ri	GL000220.1	112025	112177
ENSG0000015293	-2.110503393	0.022	0.05	LMNTD1	protein_coding	lamin tail do	12	25409307	25648579
ENSG0000026566	1.129626384	0.022	0.05	RARA-AS1	lncRNA	RARA antise	17	40340867	40343136
ENSG0000017253	-0.133431496	0.022	0.05	HCFC1	protein_coding	host cell fac	X	153947557	153971818
ENSG0000027547	-2.461968396	0.022	0.05	AC009318.4	lncRNA	novel transc	12	29277397	29277882
ENSG0000027097	1.415807247	0.022	0.05	AC015849.5	transcribed_pr	leucine rich	17	35893707	35911651
ENSG0000028040	1.584633372	0.022	0.05	AC093525.1	TEC	tec	16	2578395	2579963
ENSG0000014924	-1.104607022	0.022	0.05	KLHL35	protein_coding	kelch like fa	11	75422394	75430629
ENSG0000000518	1.921679341	0.022	0.05	ACSM3	protein_coding	acyl-CoA sy	16	20610243	20797581
ENSG0000027945	1.272474618	0.022	0.05	AC006277.1	TEC	TEC	19	2949035	2950644
ENSG0000012043	0.174093683	0.022	0.05	ACAT2	protein_coding	acetyl-CoA	6	159762045	159779112
ENSG0000016407	0.347589883	0.022	0.05	ABHD18	protein_coding	abhydrolase	4	127965306	128039927
ENSG0000019846	0.304565597	0.022	0.05	ZNF587	protein_coding	zinc finger p	19	57849859	57865117
ENSG0000014653	-0.774651528	0.022	0.05	VWDE	protein_coding	von Willebra	7	12330885	12403941
ENSG0000013165	1.608021831	0.022	0.05	KREMEN2	protein_coding	kringle cont	16	2964216	2968383
ENSG0000012065	-0.331323232	0.022	0.05	ENOX1	protein_coding	ecto-NOX c	13	43213518	43786908
ENSG0000017302	-0.183462779	0.022	0.05	GRK2	protein_coding	G protein-c	11	67266473	67286556
ENSG0000023570	0.826763301	0.022	0.05	DICER1-AS	lncRNA	DICER1 anti	14	95157645	95181475
ENSG0000026026	-2.464389302	0.022	0.05	AC105036.3	lncRNA	novel transc	15	75527150	75601205
ENSG0000010602	0.512007085	0.022	0.05	SSBP1	protein_coding	single strand	7	141738334	141787922

ENSG0000010507	-0.497028743	0.022	0.05	C19orf44	protein_coding	chromosom	19	16496394	16521352
ENSG0000018228	-0.169596382	0.022	0.05	AP1S2	protein_coding	adaptor rela	X	15825806	15854931
ENSG0000007096	-0.146489221	0.022	0.05	ATP2B1	protein_coding	ATPase plas	12	89588049	89709300
ENSG0000023046	-0.411642337	0.023	0.05	PROX1-AS1	lncRNA	PROX1 antis	1	213817751	213988508
ENSG0000012827	-1.045470141	0.023	0.05	ADORA2A	protein_coding	adenosine A	22	24417879	24442357
ENSG0000006006	0.445222715	0.023	0.05	CTDP1	protein_coding	CTD phosph	18	79679803	79754510
ENSG0000012381	-0.464914244	0.023	0.05	COQ8B	protein_coding	coenzyme Q	19	40691530	40718207
ENSG0000023215	-0.741916299	0.023	0.05	ST13P4	processed_pse	ST13, Hsp70	13	50172089	50173181
ENSG0000026033	-2.275148859	0.023	0.05	AC079148.1	lncRNA	novel transc	2	18547386	18548204
ENSG0000013967	-0.30785836	0.023	0.05	HNRNPA1L	protein_coding	heterogene	13	52642425	52643796
ENSG0000011613	0.27085442	0.023	0.05	DNAJC16	protein_coding	DnaJ heat sh	1	15526813	15592379
ENSG0000013064	-2.137142073	0.023	0.05	CYP2E1	protein_coding	cytochrome	10	133520406	133561220
ENSG0000024835	-5.372753998	0.023	0.05	AC010280.1	lncRNA	novel transc	5	68508223	68565515
ENSG0000014621	-1.794436944	0.023	0.05	CRIP3	protein_coding	cysteine rich	6	43299710	43308826
ENSG0000012243	0.242967204	0.023	0.05	TRMT13	protein_coding	tRNA methy	1	100133150	100150498
ENSG0000027482	2.055217018	0.023	0.05	AL023803.2	lncRNA	novel transc	20	38955910	38956547
ENSG0000007541	0.149998065	0.023	0.05	MARK3	protein_coding	microtubule	14	103385392	103503831
ENSG0000016845	0.132351748	0.023	0.05	HR	protein_coding	HR lysine de	8	22114419	22133384
ENSG0000021448	-0.136077199	0.023	0.05	RPL7P1	processed_pse	ribosomal p	5	150094302	150095048
ENSG0000021445	-1.014254794	0.023	0.05	RCN1P2	processed_pse	reticulocalbi	13	45390353	45391267
ENSG0000017637	0.266178875	0.023	0.05	ZSCAN2	protein_coding	zinc finger a	15	84600986	84627796
ENSG0000018325	1.168427211	0.023	0.05	LINC01547	lncRNA	long interge	21	44932814	44939937
ENSG0000014538	0.221788953	0.023	0.05	METTL14	protein_coding	methyltrans	4	118685392	118715433
ENSG0000015515	0.817671642	0.023	0.05	TTC39B	protein_coding	tetratricope	9	15163622	15307360
ENSG0000023400	0.351030862	0.023	0.05	PPP1R18	protein_coding	protein pho	CHR_HSCHR	30666650	30678156
ENSG0000027289	3.149991792	0.023	0.05	AL355987.3	protein_coding	novel protei	9	136791379	136800595
ENSG0000026142	1.523857718	0.023	0.05	AL022069.1	lncRNA	novel transc	6	166383189	166384824
ENSG0000022643	-0.703008237	0.023	0.05	TRIM39	protein_coding	tripartite mo	CHR_HSCHR	30371111	30388367
ENSG0000020508	-1.777854329	0.023	0.05	FAM71F2	protein_coding	family with s	7	128672288	128687872
ENSG0000019614	-0.291648188	0.023	0.05	SPATS2L	protein_coding	spermatoge	2	200305881	200482264
ENSG0000016708	-0.183269809	0.023	0.05	PBX3	protein_coding	PBX homeo	9	125747345	125967377
ENSG0000012800	0.33533926	0.023	0.05	ZNF780B	protein_coding	zinc finger p	19	40028260	40056231
ENSG0000011677	-1.006972752	0.023	0.05	OLFML3	protein_coding	olfactomedi	1	113979391	114035572
ENSG0000016497	0.770306519	0.023	0.05	MYORG	protein_coding	myogenesis	9	34366666	34376898
ENSG0000028001	0.953258551	0.023	0.05	CU634019.5	lncRNA	novel transc	21	6986450	6997765
ENSG0000014974	0.425061983	0.023	0.05	TRPT1	protein_coding	tRNA phosp	11	64223799	64226254
ENSG0000011864	1.821377622	0.023	0.05	VAMP8	protein_coding	vesicle asso	2	85561562	85582031
ENSG0000016261	-0.266859653	0.023	0.05	DNAJB4	protein_coding	DnaJ heat sh	1	77979175	78017964
ENSG0000019811	-1.299196882	0.023	0.05	TOR4A	protein_coding	torsin family	9	137277726	137282641
ENSG0000023234	-2.358355109	0.023	0.05	AC087163.2	processed_pse	ribosomal p	17	18010643	18011822
ENSG0000026541	0.494747086	0.023	0.05	AC099850.3	lncRNA	novel transc	17	59202677	59203829
ENSG0000027298	-1.832153936	0.023	0.05	LINC02012	lncRNA	long interge	3	197505262	197506986
ENSG0000018340	0.905399791	0.023	0.05	CCDC159	protein_coding	coiled-coil d	19	11344684	11354944
ENSG0000013431	0.853367881	0.024	0.05	GRHL1	protein_coding	grainyhead	2	9951693	10002277
ENSG0000009480	0.158229126	0.024	0.05	CDC6	protein_coding	cell division	17	40287879	40304657
ENSG0000010022	-0.151622547	0.024	0.05	RTCB	protein_coding	RNA 2',3'-cy	22	32387582	32412248
ENSG0000005595	2.638027644	0.024	0.05	ITIH4	protein_coding	inter-alpha-	3	52812962	52830688
ENSG0000016757	0.841633163	0.024	0.05	RAB4B	protein_coding	RAB4B, mer	19	40778216	40796942
ENSG0000016959	0.281469095	0.024	0.05	NFU1	protein_coding	NFU1 iron-s	2	69396113	69437628
ENSG0000018881	-0.349641318	0.024	0.05	NHLRC3	protein_coding	NHL repeat	13	39038306	39050109
ENSG0000025405	-1.860599103	0.024	0.05	AC087273.2	lncRNA	novel transc	8	17900484	17908011
ENSG0000020620	-0.333725517	0.024	0.05	DAXX	protein_coding	death doma	CHR_HSCHR	33296480	33307190
ENSG0000011167	0.25576707	0.024	0.05	GNPTAB	protein_coding	N-acetylglu	12	101745499	101830959
ENSG0000020396	0.409600054	0.024	0.05	EFCAB7	protein_coding	EF-hand cal	1	63523372	63572693
ENSG0000027209	1.721304445	0.024	0.05	AL024498.1	lncRNA	novel transc	6	10743324	10747663
ENSG0000016207	0.368202127	0.024	0.05	FLYWCH2	protein_coding	FLYWCH far	16	2883213	2899382
ENSG0000025398	0.853802999	0.024	0.05	AC100810.1	lncRNA	novel transc	8	1761054	1764508
ENSG0000007666	0.660267867	0.024	0.05	ICAM3	protein_coding	intercellular	19	10333776	10339661

ENSG0000010335	0.306456126	0.024	0.05	CLUAP1	protein_coding	clusterin ass	16	3500976	3539048
ENSG0000012321	0.143216253	0.024	0.05	NLN	protein_coding	neurolysin [	5	65722205	65871725
ENSG0000027432	-0.3258261	0.024	0.05	GOLGA6L9	protein_coding	golgin A6 fa	CHR_HSCHR	82509086	82518216
ENSG0000013788	-3.295463802	0.024	0.05	GCHFR	protein_coding	GTP cyclohy	15	40764068	40767708
ENSG0000021495	-3.015546731	0.024	0.05	AP000317.1	lncRNA	novel transc	21	34205055	34325034
ENSG0000016922	0.297697624	0.024	0.05	RAB24	protein_coding	RAB24, mer	5	177301198	177303744
ENSG0000026756	-0.755071742	0.024	0.05	AC011477.2	lncRNA	novel transc	19	19892433	19895847
ENSG0000019840	1.407917058	0.024	0.05	BZW1P2	processed_pse	basic leucin	3	116645902	116646727
ENSG0000013092	-0.244632493	0.024	0.05	C12orf65	protein_coding	chromosom	12	123233436	123257960
ENSG0000018588	0.441192477	0.024	0.05	ATP6V0C	protein_coding	ATPase H+	16	2513952	2520218
ENSG0000013325	0.830437136	0.024	0.05	PDE6B	protein_coding	phosphodie	4	625584	670782
ENSG0000008972	-1.215552747	0.024	0.05	OTUB2	protein_coding	OTU deubic	14	94026340	94048930
ENSG0000015235	0.280614587	0.024	0.05	POC5	protein_coding	POC5 centri	5	75674124	75717448
ENSG0000016918	0.263098296	0.024	0.05	NSMCE1	protein_coding	NSE1 homo	16	27224994	27268772
ENSG0000017891	0.602885821	0.024	0.05	ZNF852	protein_coding	zinc finger p	3	44494847	44510636
ENSG0000014384	-0.66498497	0.024	0.05	ETNK2	protein_coding	ethanolamin	1	204131062	204152044
ENSG0000000201	0.334211768	0.024	0.06	RAD52	protein_coding	RAD52 hom	12	911736	990053
ENSG0000023283	0.538720203	0.024	0.06	PET117	protein_coding	PET117 cytc	20	18137863	18143169
ENSG0000011017	-0.122972493	0.024	0.06	CHORDC1	protein_coding	cysteine and	11	90200429	90223077
ENSG0000018831	0.714531976	0.024	0.06	PLSCR1	protein_coding	phospholipi	3	146515180	146544856
ENSG0000020648	-0.350818649	0.024	0.06	PPP1R10	protein_coding	protein pho	CHR_HSCHR	30589896	30608107
ENSG0000011587	0.111311317	0.024	0.06	SRSF7	protein_coding	serine and a	2	38743599	38751494
ENSG0000023230	-1.627037431	0.024	0.06	FAM215B	lncRNA	family with s	17	46558830	46562795
ENSG0000014315	0.260991992	0.024	0.06	NME7	protein_coding	NME/NM23	1	169132531	169367948
ENSG0000017400	0.655493417	0.024	0.06	CEP19	protein_coding	centrosoma	3	196706277	196712250
ENSG0000025325	-0.615414604	0.024	0.06	SHLD3	protein_coding	shieldin con	5	65625027	65630928
ENSG0000024068	0.205800664	0.024	0.06	ISY1	protein_coding	ISY1 splicing	3	129127415	129161063
ENSG0000026998	0.839614063	0.024	0.06	AC078795.1	lncRNA	novel transc	3	169777192	169780334
ENSG0000022912	-1.619335481	0.025	0.06	KANSL1L-A	lncRNA	KANSL1L ar	2	210030572	210064356
ENSG0000013020	0.253324212	0.025	0.06	TOMM40	protein_coding	translocase	19	44890569	44903689
ENSG0000027899	1.171050937	0.025	0.06	AC002350.2	TEC	novel transc	12	110501614	110503441
ENSG0000020532	0.162040103	0.025	0.06	SARNP	protein_coding	SAP domain	12	55752463	55817724
ENSG0000010606	-0.242508483	0.025	0.06	CPVL	protein_coding	carboxypep	7	28995235	29195451
ENSG0000023288	-1.376652156	0.025	0.06	RPS11P5	processed_pse	ribosomal p	12	132825701	132826184
ENSG0000022927	0.596883921	0.025	0.06	BX664615.1	processed_pse	novel pseuc	9	39809562	39810019
ENSG0000017134	-1.393937059	0.025	0.06	KRT19	protein_coding	keratin 19 [	17	41523617	41528308
ENSG0000016019	0.231079531	0.025	0.06	PKNOX1	protein_coding	PBX/knottec	21	42974510	43033931
ENSG0000019715	2.461742642	0.025	0.06	H3C12	protein_coding	H3 clusterec	6	27890315	27893106
ENSG0000012593	-1.126773976	0.025	0.06	CITED1	protein_coding	Cbp/p300 ir	X	72301638	72307187
ENSG0000011259	1.659689584	0.025	0.06	GUCA1B	protein_coding	guanylate c	6	42183284	42194956
ENSG0000028035	-1.245953839	0.025	0.06	AC127496.7	TEC	TEC	17	81003335	81005132
ENSG0000023070	-1.804007619	0.025	0.06	HLA-DPB1	protein_coding	major histo	CHR_HSCHR	32997896	33009106
ENSG0000016016	1.299128524	0.025	0.06	CILP2	protein_coding	cartilage int	19	19538248	19546659
ENSG0000014166	0.729486343	0.025	0.06	ZCCHC2	protein_coding	zinc finger C	18	62523025	62587709
ENSG0000008446	0.128630057	0.025	0.06	WBP11	protein_coding	WW domain	12	14784582	14803486
ENSG0000025491	1.55153957	0.025	0.06	SCARNA9	lncRNA	small Cajal b	11	93721513	93721865
ENSG0000007610	-0.116259372	0.025	0.06	BAZ2A	protein_coding	bromodoma	12	56595596	56636816
ENSG0000027750	2.139179174	0.025	0.06	AP000351.2	unprocessed_p	D-dopachrc	CHR_HSCHR	24030925	24031599
ENSG0000007220	-1.117692244	0.025	0.06	LNX1	protein_coding	ligand of nu	4	53459301	53701405
ENSG0000014303	-4.16111383	0.025	0.06	SLC44A3	protein_coding	solute carrie	1	94820342	94895246
ENSG0000019947	5.314808932	0.025	0.06	Y_RNA	misc_RNA	Y RNA [Sou	1	202914880	202914980
ENSG0000016550	0.291455362	0.025	0.06	LRR1	protein_coding	leucine rich	14	49598761	49614672
ENSG0000024985	0.314028038	0.025	0.06	HS3ST5	protein_coding	heparan sul	6	114055586	114343045
ENSG0000017365	0.367350725	0.025	0.06	RCE1	protein_coding	Ras convert	11	66842835	66846552
ENSG0000027219	1.425051162	0.025	0.06	AL356512.1	lncRNA	novel transc	1	244969350	244971088
ENSG0000024837	3.134521608	0.025	0.06	AC104066.3	processed_pse	novel pseuc	4	52720081	52720831
ENSG0000028001	-1.398006883	0.025	0.06	AP001350.2	TEC	TEC	11	58627435	58628528
ENSG0000017237	-0.22950241	0.025	0.06	C2CD2L	protein_coding	C2CD2 like	11	119102198	119118544

ENSG0000017104	-0.9131334	0.025	0.06	XKR6	protein_coding	XK related 6	8	10896045	11201833
ENSG0000013839	-0.210930754	0.025	0.06	FASTKD1	protein_coding	FAST kinase	2	169528508	169573875
ENSG0000016386	-0.257385786	0.025	0.06	ZMYM6	protein_coding	zinc finger M	1	34986165	35031945
ENSG0000016200	-0.8768446	0.025	0.06	CCDC78	protein_coding	coiled-coil c	16	722582	726954
ENSG0000023261	0.489784202	0.025	0.06	SKIV2L	protein_coding	Ski2 like RN	CHR_HSCHR	32035523	32046194
ENSG0000025931	1.348659256	0.025	0.06	AF111167.2	lncRNA	novel transc	14	75423683	75427741
ENSG0000017144	-1.105565129	0.025	0.06	ZNF524	protein_coding	zinc finger p	19	55600022	55603138
ENSG0000027263	0.566094655	0.025	0.06	AC067750.1	lncRNA	novel transc	10	86749754	86756298
ENSG0000017875	-1.497675491	0.025	0.06	ERFE	protein_coding	erythroferro	2	238158970	238168900
ENSG0000027646	0.238324351	0.025	0.06	TAF9	protein_coding	TATA-box b	CHR_HSCHR	69364756	69370027
ENSG0000023926	-0.624651736	0.025	0.06	TXNDC5	protein_coding	thioredoxin	6	7881517	7910788
ENSG0000028043	0.854193906	0.025	0.06	NA	NA	NA	NA	NA	NA
ENSG0000020439	-0.261025546	0.025	0.06	VARS1	protein_coding	valyl-tRNA s	6	31777518	31795752
ENSG0000016867	1.217911644	0.025	0.06	SLC16A4	protein_coding	solute carrie	1	110362851	110391082
ENSG0000013695	0.240363715	0.025	0.06	ARPC5L	protein_coding	actin relatec	9	124862130	124877733
ENSG0000013780	-0.370957212	0.025	0.06	ITGA11	protein_coding	integrin sub	15	68296532	68432163
ENSG0000011020	0.290527865	0.025	0.06	ANAPC15	protein_coding	anaphase pr	11	72106378	72112780
ENSG0000012309	0.173272186	0.025	0.06	RNF11	protein_coding	ring finger p	1	51236273	51273447
ENSG0000016910	0.321729908	0.026	0.06	CHST14	protein_coding	carbohydrat	15	40470984	40473158
ENSG0000017638	-1.931315653	0.026	0.06	HSD11B2	protein_coding	hydroxyster	16	67430652	67437553
ENSG0000023159	-1.931315653	0.026	0.06	NA	NA	NA	NA	NA	NA
ENSG0000018832	-0.229789324	0.026	0.06	SBK1	protein_coding	SH3 domair	16	28259246	28323849
ENSG0000023810	-1.086332821	0.026	0.06	GOLGA2P5	transcribed_un	GOLGA2 ps	12	100156357	100173659
ENSG0000013052	0.195507245	0.026	0.06	JUND	protein_coding	JunD proto-	19	18279694	18281622
ENSG0000010882	-0.13995887	0.026	0.06	VAT1	protein_coding	vesicle amir	17	43014607	43025123
ENSG0000024355	0.843802557	0.026	0.06	AC004967.1	transcribed_un	CCZ1 homc	7	97969005	97972254
ENSG0000013719	0.184877254	0.026	0.06	PIM1	protein_coding	Pim-1 protc	6	37170152	37175428
ENSG0000010995	0.299410482	0.026	0.06	B3GAT1	protein_coding	beta-1,3-gl	11	134378504	134412242
ENSG0000027279	1.879893826	0.026	0.06	AC073389.3	lncRNA	novel transc	10	73630556	73631490
ENSG0000012483	0.158041606	0.026	0.06	LRRFIP1	protein_coding	LRR binding	2	237627587	237813682
ENSG0000013963	-0.307478408	0.026	0.06	MYG1	protein_coding	MYG1 exon	12	53299695	53307177
ENSG0000012900	-1.256669348	0.026	0.06	ISLR	protein_coding	immunoglol	15	74173710	74176872
ENSG0000026763	0.698561466	0.026	0.06	AC067852.3	lncRNA	novel transc	17	42509784	42511519
ENSG0000027964	-1.495230982	0.026	0.06	AC120057.3	TEC	TEC	17	7242200	7243814
ENSG0000013597	0.270194266	0.026	0.06	MRPS9	protein_coding	mitochondr	2	105038069	105099960
ENSG0000013595	1.123319982	0.026	0.06	TSGA10	protein_coding	testis specifi	2	98997261	99154964
ENSG0000010497	-0.379738539	0.026	0.06	SNAPC2	protein_coding	small nuclea	19	7920338	7923250
ENSG0000012075	-0.286794843	0.026	0.06	PLS1	protein_coding	plastin 1 [Sc	3	142596393	142713664
ENSG0000010801	0.154143899	0.026	0.06	GLRX3	protein_coding	glutaredoxir	10	130136391	130184521
ENSG0000019611	1.082960927	0.026	0.06	CCDC189	protein_coding	coiled-coil c	16	30757423	30762221
ENSG0000013171	0.070304524	0.026	0.06	MAP1B	protein_coding	microtubule	5	72107234	72209565
ENSG0000027808	-0.175980653	0.026	0.06	NOMO3	protein_coding	NODAL mo	CHR_HSCHR	16317633	16379962
ENSG0000012004	-1.112555777	0.026	0.06	KCNIP2	protein_coding	potassium v	10	101825974	101843920
ENSG0000027963	1.572238037	0.026	0.06	AL158211.5	TEC	TEC	10	22252072	22254589
ENSG0000027881	1.044771169	0.026	0.06	LINC00624	lncRNA	long interge	1	147258885	147517875
ENSG0000018122	0.291176096	0.026	0.06	ZNF746	protein_coding	zinc finger p	7	149472696	149497817
ENSG0000021346	-0.238266545	0.026	0.06	ARL2	protein_coding	ADP ribosyl	11	65014160	65022184
ENSG0000024132	-2.066564291	0.026	0.06	CDRT1	protein_coding	CMT1A dup	17	15565483	15619704
ENSG0000013671	-0.342818994	0.026	0.06	CCDC115	protein_coding	coiled-coil c	2	130337933	130342699
ENSG0000000978	0.419663604	0.026	0.06	FAM76A	protein_coding	family with s	1	27725961	27763116
ENSG0000027172	1.107803136	0.026	0.06	MROH7-TT	protein_coding	MROH7-TT	1	54641786	54742308
ENSG0000013542	1.073824338	0.026	0.06	ITGA7	protein_coding	integrin sub	12	55684568	55716043
ENSG0000007054	-0.581004201	0.026	0.06	WIPI1	protein_coding	WD repeat c	17	68420948	68457513
ENSG0000011239	0.60227593	0.026	0.06	SLC16A10	protein_coding	solute carrie	6	111087503	111231194
ENSG0000009982	-0.225087229	0.026	0.06	POLRMT	protein_coding	RNA polyme	19	617221	633537
ENSG0000014963	0.114232159	0.026	0.06	SOGA1	protein_coding	suppressor c	20	36777447	36863538
ENSG0000013030	-0.2764688	0.026	0.06	NSUN5	protein_coding	NOP2/Sun F	7	73302516	73308826
ENSG0000022610	0.785824068	0.026	0.06	C6orf47	protein_coding	chromosom	CHR_HSCHR	31649040	31651514

ENSG0000028023	-0.150792409	0.027	0.06	NA	NA	NA	NA	NA	NA
ENSG0000014992	0.216581179	0.027	0.06	PPP4C	protein_coding	protein pho	16	30075978	30085376
ENSG0000026393	2.424117046	0.027	0.06	SNORD3A	snoRNA	small nuclec	17	19188016	19188714
ENSG0000024528	1.232725743	0.027	0.06	AC124242.1	lncRNA	novel transc	8	18084386	18097644
ENSG0000027693	1.956743025	0.027	0.06	AC009041.3	lncRNA	novel transc	16	968375	969012
ENSG0000026106	0.737869423	0.027	0.06	AC092718.4	lncRNA	novel transc	16	81030770	81031485
ENSG0000018800	0.49382895	0.027	0.06	AC026412.1	transcribed_un	programme	5	1598920	1634005
ENSG0000022723	-0.387589717	0.027	0.06	WASH7P	unprocessed_p	WASP famil	1	14404	29570
ENSG0000014046	0.337513179	0.027	0.06	PML	protein_coding	promyelocy	15	73994673	74047827
ENSG0000024418	3.727144879	0.027	0.06	AC091153.3	lncRNA	novel transc	17	4704230	4705529
ENSG0000018726	-0.577226841	0.027	0.06	EPOR	protein_coding	erythropoie	19	11377207	11384342
ENSG0000020522	-0.884938043	0.027	0.06	PSMB10	protein_coding	proteasome	16	67934506	67936864
ENSG0000015221	-2.097111683	0.027	0.06	RIT2	protein_coding	Ras like with	18	42743227	43115691
ENSG0000014712	0.371590965	0.027	0.06	KRBOX4	protein_coding	KRAB box d	X	46447292	46497422
ENSG0000017733	-1.851255451	0.027	0.06	DLGAP1-AS	lncRNA	DLGAP1 ant	18	3593732	3598363
ENSG0000027685	-1.317536919	0.027	0.06	AC015922.3	lncRNA	novel transc	17	15789016	15789705
ENSG0000027200	-2.331958078	0.027	0.06	AC010904.2	lncRNA	novel transc	2	7260871	7261504
ENSG0000008620	0.195398124	0.027	0.06	IPO11	protein_coding	importin 11	5	62403972	62628582
ENSG0000016647	-0.319445516	0.027	0.06	ZNF143	protein_coding	zinc finger p	11	9460319	9528524
ENSG0000015491	-0.584270577	0.027	0.06	RAB6B	protein_coding	RAB6B, mer	3	133824235	133895882
ENSG0000013446	1.345986802	0.027	0.06	ECHDC3	protein_coding	enoyl-CoA	10	11742366	11764070
ENSG0000028016	-5.173759458	0.027	0.06	PCDH20	protein_coding	protocadhe	13	61409685	61415522
ENSG0000013918	1.415794235	0.027	0.06	KLRG1	protein_coding	killer cell lec	12	8950044	9010760
ENSG0000017672	1.260151056	0.027	0.06	ZNF843	protein_coding	zinc finger p	16	31432593	31443160
ENSG0000012587	0.20226412	0.027	0.06	ITPA	protein_coding	inosine triph	20	3208868	3223870
ENSG0000025447	1.026918725	0.027	0.06	AL354920.1	lncRNA	novel transc	9	83707594	83713378
ENSG0000013505	0.115723606	0.027	0.06	GOLM1	protein_coding	golgi memb	9	86026146	86100173
ENSG0000024859	-0.942187096	0.027	0.06	DSTNP2	transcribed_un	DSTN pseuc	12	6884682	6885786
ENSG0000010289	1.017981298	0.027	0.06	ELMO3	protein_coding	engulfment	16	67199111	67204029
ENSG0000014594	-0.57608323	0.027	0.06	FAM50B	protein_coding	family with s	6	3849373	3851320
ENSG0000021306	0.210690644	0.027	0.06	CEP43	protein_coding	centrosoma	6	166999317	167094789
ENSG0000027215	3.267064527	0.027	0.06	AC008280.2	lncRNA	novel transc	2	54082554	54085066
ENSG0000010324	-0.314393787	0.027	0.06	CIAO3	protein_coding	cytosolic iro	16	729760	741329
ENSG0000015792	0.4242402	0.027	0.06	RADIL	protein_coding	Rap associa	7	4797055	4883716
ENSG0000023405	-0.501854097	0.027	0.06	PPP1R11	protein_coding	protein pho	CHR_HSCHR	30056599	30060223
ENSG0000018166	-0.294417358	0.027	0.06	ZNF875	protein_coding	zinc finger p	19	37312837	37369365
ENSG0000017556	-1.155205819	0.028	0.06	UCP3	protein_coding	uncoupling	11	74000277	74009085
ENSG0000009590	0.231997231	0.028	0.06	NUBP2	protein_coding	nucleotide b	16	1782932	1789186
ENSG0000016497	-2.534946368	0.028	0.06	C9orf24	protein_coding	chromosom	9	34379019	34397810
ENSG0000015068	0.656802188	0.028	0.06	PRSS23	protein_coding	serine prote	11	86791059	86952910
ENSG0000017146	0.184715241	0.028	0.06	ZNF562	protein_coding	zinc finger p	19	9641807	9675100
ENSG0000013234	0.086312856	0.028	0.06	RAN	protein_coding	RAN, memb	12	130872037	130877678
ENSG0000011010	0.225557914	0.028	0.06	TMEM109	protein_coding	transmembr	11	60914158	60923443
ENSG0000016102	0.16599927	0.028	0.06	MAML1	protein_coding	mastermind	5	179732822	179777283
ENSG0000016528	-0.289524443	0.028	0.06	PIGO	protein_coding	phosphatidy	9	35088688	35096601
ENSG0000019673	0.155341252	0.028	0.06	DAPK1	protein_coding	death assoc	9	87497228	87708634
ENSG0000017535	-0.370865221	0.028	0.06	PTPN2	protein_coding	protein tyro	18	12785478	12929643
ENSG0000015811	-2.651617405	0.028	0.06	LRRC43	protein_coding	leucine rich	12	122167738	122203471
ENSG0000027727	-1.252726041	0.028	0.06	OTUB2	protein_coding	OTU deubic	CHR_HSCHR	94026329	94049624
ENSG0000025045	-1.672080792	0.028	0.06	HOXC-AS1	lncRNA	HOXC cluste	12	53999022	54000010
ENSG0000014336	-0.161317091	0.028	0.06	SF3B4	protein_coding	splicing fact	1	149923317	149927803
ENSG0000020545	0.932387576	0.028	0.06	TP53TG3D	protein_coding	TP53 target	16	32252719	32255922
ENSG0000013106	-0.859262713	0.028	0.06	ZNF341	protein_coding	zinc finger p	20	33731657	33792269
ENSG0000022401	-2.874237926	0.028	0.06	AC092681.1	unprocessed_p	pseudogene	7	149891191	149909704
ENSG0000025325	0.44212696	0.028	0.06	C8orf88	protein_coding	chromosom	8	90958471	90985238
ENSG0000018185	-0.172078086	0.028	0.06	RNF41	protein_coding	ring finger p	12	56202179	56221933
ENSG0000014655	-0.404747709	0.028	0.06	SDK1	protein_coding	sidekick cell	7	3301252	4269000
ENSG0000013574	-0.19187835	0.028	0.06	PCNX2	protein_coding	pecanex 2 [	1	232983435	233295725

ENSG0000016496	0.158700233	0.028	0.06	WASHC5	protein_coding	WASH comp	8	125024260	125091819
ENSG0000018373	-0.188053077	0.028	0.06	TBK1	protein_coding	TANK bindir	12	64452092	64502114
ENSG0000016463	0.28088864	0.028	0.06	SLC29A4	protein_coding	solute carrie	7	5274369	5306870
ENSG0000013266	-0.376680363	0.028	0.06	NXT1	protein_coding	nuclear tran	20	23350791	23354771
ENSG0000026106	0.820276546	0.028	0.06	AC109460.3	lncRNA	novel transc	16	28974804	28990775
ENSG0000009061	-0.13764061	0.028	0.06	GOLGA3	protein_coding	golgin A3 [S	12	132768914	132829078
ENSG0000017795	-0.390522276	0.028	0.06	RPS27	protein_coding	ribosomal p	1	153990762	153992155
ENSG0000024707	-0.212231018	0.028	0.06	PGAM5	protein_coding	PGAM famil	12	132710819	132722734
ENSG0000026331	-5.266944197	0.028	0.06	LINC01982	lncRNA	long interge	17	52390515	52535701
ENSG0000013258	0.325145451	0.028	0.06	SDF2	protein_coding	stromal cell	17	28648346	28662189
ENSG0000017423	-1.992593331	0.028	0.06	REP15	protein_coding	RAB15 effec	12	27696447	27697596
ENSG0000016603	-0.139242945	0.028	0.06	CEP57	protein_coding	centrosoma	11	95789965	95832693
ENSG0000012625	-0.273820687	0.029	0.06	RBM42	protein_coding	RNA binding	19	35629030	35637686
ENSG0000011477	0.138169146	0.029	0.06	ABCC5	protein_coding	ATP binding	3	183919934	184017939
ENSG0000011071	-1.177685852	0.029	0.06	TCIRG1	protein_coding	T cell immu	11	68039025	68050895
ENSG0000000156	-0.883592337	0.029	0.06	ENPP4	protein_coding	ectonucleot	6	46129989	46146688
ENSG0000027542	1.787267856	0.029	0.06	AC253576.2	lncRNA	novel transc	4	149738	150317
ENSG0000018786	0.253729666	0.029	0.06	FAM122A	protein_coding	family with s	9	68780065	68785566
ENSG0000021633	2.75661343	0.029	0.06	H1-12P	unprocessed_p	H1.12 linker	6	26195554	26196633
ENSG0000011898	-0.630210898	0.029	0.06	ELL2	protein_coding	elongation f	5	95885098	95961851
ENSG0000013112	0.325047861	0.029	0.06	ZNF141	protein_coding	zinc finger p	4	337814	384868
ENSG0000018801	0.484462676	0.029	0.06	MORN2	protein_coding	MORN repe	2	38875962	38929072
ENSG0000017916	-1.154886865	0.029	0.06	FUCA1	protein_coding	alpha-L-fuc	1	23845077	23868290
ENSG0000001163	-1.073466149	0.029	0.06	TMEM159	protein_coding	transmembr	16	21158377	21180616
ENSG0000022455	0.674174861	0.029	0.06	ABHD16A	protein_coding	abhydrolase	CHR_HSCHR	31726403	31742871
ENSG0000027591	-0.71842958	0.029	0.06	CHRFAM7A	protein_coding	CHRNA7 (ex	CHR_HSCHR	30511927	30544252
ENSG0000013432	-1.12261736	0.029	0.06	CMPK2	protein_coding	cytidine/uric	2	6840570	6866635
ENSG0000015987	-1.908954331	0.029	0.06	LYPD5	protein_coding	LY6/PLAUR	19	43785874	43827206
ENSG0000011922	0.999090406	0.029	0.06	PIGZ	protein_coding	phosphatidy	3	196946356	196969060
ENSG0000012360	-0.281571968	0.029	0.06	METTL8	protein_coding	methyltrans	2	171317405	171434802
ENSG0000010099	0.15691053	0.029	0.06	TRPC4AP	protein_coding	transient rec	20	35002404	35092807
ENSG0000012141	0.431103101	0.029	0.06	ZSCAN18	protein_coding	zinc finger a	19	58083838	58118427
ENSG0000013393	-0.308944134	0.029	0.06	ERG28	protein_coding	ergosterol b	14	75649791	75660876
ENSG0000014337	0.246585195	0.029	0.07	TARS2	protein_coding	threonyl-tR	1	150487364	150507609
ENSG0000011324	-0.574289442	0.029	0.07	PCDHB15	protein_coding	protocadhe	5	141245395	141249365
ENSG0000026461	1.347027949	0.029	0.07	MSH5-SAP	protein_coding	MSH5-SAP(	CHR_HSCHR	31825184	31831870
ENSG0000020479	-1.842217831	0.029	0.07	SMPD5	transcribed_un	sphingomye	8	144049079	144051522
ENSG0000023418	-3.642375731	0.029	0.07	AC068533.3	lncRNA	novel transc	7	66119603	66165011
ENSG0000023384	-0.402099652	0.029	0.07	HLA-C	protein_coding	major histo	CHR_HSCHR	31259794	31263175
ENSG0000021401	0.273918789	0.029	0.07	GANC	protein_coding	glucosidase	15	42273233	42356935
ENSG0000008909	-0.193048357	0.029	0.07	KDM2B	protein_coding	lysine deme	12	121429096	121581023
ENSG0000016153	0.203290113	0.029	0.07	ACOX1	protein_coding	acyl-CoA ox	17	75941507	75979177
ENSG0000016288	0.162434732	0.029	0.07	MAPKAPK2	protein_coding	MAPK activa	1	206684905	206734281
ENSG0000010197	0.147539998	0.03	0.07	ATP11C	protein_coding	ATPase pho	X	139726346	139945276
ENSG0000000828	-0.196193699	0.03	0.07	SYPL1	protein_coding	synaptophy	7	106090503	106112576
ENSG0000011285	0.147103442	0.03	0.07	ERBIN	protein_coding	erbb2 intera	5	65926475	66082549
ENSG0000026302	-3.576119758	0.03	0.07	AL662899.2	protein_coding	novel protei	6	31666102	31673546
ENSG0000026277	-1.397703869	0.03	0.07	LINC01977	lncRNA	long interge	17	79819083	79827704
ENSG0000014060	1.79753071	0.03	0.07	SH3GL3	protein_coding	SH3 domair	15	83447228	83618743
ENSG0000010121	-0.324640957	0.03	0.07	GMEB2	protein_coding	glucocortico	20	63587602	63627101
ENSG0000011664	-0.207346825	0.03	0.07	SRM	protein_coding	spermidine	1	11054584	11060020
ENSG0000016040	1.268202205	0.03	0.07	CFAP157	protein_coding	cilia and flag	9	127706988	127716002
ENSG0000007044	-0.272222247	0.03	0.07	MNT	protein_coding	MAX netwo	17	2384073	2401104
ENSG0000014867	-0.135346192	0.03	0.07	GLUD1	protein_coding	glutamate c	10	87050202	87094843
ENSG0000014273	1.048704764	0.03	0.07	MAP3K6	protein_coding	mitogen-ac	1	27354067	27366961
ENSG0000015659	0.15565189	0.03	0.07	ZDHHC5	protein_coding	zinc finger D	11	57667747	57701182
ENSG0000022947	-0.420163268	0.03	0.07	GNL1	protein_coding	G protein nu	CHR_HSCHR	30531607	30547405
ENSG0000020362	0.785161401	0.03	0.07	C6orf47	protein_coding	chromosom	CHR_HSCHR	31648523	31650997

ENSG0000016475	0.323354467	0.03	0.07	MED30	protein_coding	mediator cc	8	117520713	117540262
ENSG0000027365	-0.683541571	0.03	0.07	AC139494.1	transcribed_un	glucuronida	CHR_HSCHR	69755077	69848798
ENSG0000025320	0.539910711	0.03	0.07	AC037459.2	lncRNA	novel transc	8	22613908	22616657
ENSG0000012596	0.379684429	0.03	0.07	ARMCX5	protein_coding	armadillo re	X	102599168	102604159
ENSG0000027503	1.527506738	0.03	0.07	TP53TG3E	protein_coding	TP53 target	16	33303739	33306935
ENSG0000011253	-2.281163807	0.03	0.07	PACRG	protein_coding	parkin coreg	6	162727132	163315492
ENSG0000024233	1.459736221	0.03	0.07	BMS1P4	transcribed_un	BMS1 pseuc	10	73715843	73730469
ENSG0000017378	0.183079401	0.03	0.07	CNP	protein_coding	2',3'-cyclic r	17	41966763	41977740
ENSG0000018427	0.226933394	0.03	0.07	TM2D3	protein_coding	TM2 domair	15	101621444	101652391
ENSG0000023120	0.424017262	0.03	0.07	ZNF826P	transcribed_un	zinc finger p	19	20340269	20424969
ENSG0000017862	-1.183907528	0.03	0.07	GPR35	protein_coding	G protein-c	2	240605430	240631259
ENSG0000027291	1.003372072	0.03	0.07	AC022400.5	protein_coding	novel transc	10	73796514	73811651
ENSG0000012421	0.443765939	0.03	0.07	SNAI1	protein_coding	snail family	20	49982980	49988886
ENSG0000015076	-0.188087495	0.03	0.07	DOCK1	protein_coding	dedicator of	10	126905409	127452517
ENSG0000026202	-0.277459946	0.03	0.07	TCF20	protein_coding	transcriptior	CHR_HSCHR	42147859	42203293
ENSG0000027062	-0.46589249	0.03	0.07	NBPF14	protein_coding	NBPF memb	1	148531385	148679742
ENSG0000018190	0.132876723	0.03	0.07	C5orf24	protein_coding	chromosom	5	134845680	134859735
ENSG0000026939	0.941759547	0.03	0.07	AC008764.6	lncRNA	novel transc	19	16542746	16544814
ENSG0000016378	0.160465209	0.03	0.07	RYK	protein_coding	receptor like	3	134065303	134250859
ENSG0000018616	0.588281279	0.03	0.07	CIDECP1	transcribed_un	cell death ir	3	10014238	10026365
ENSG0000016403	0.848923592	0.031	0.07	SLC9B1	protein_coding	solute carrie	4	102885048	103019719
ENSG0000016101	0.077068919	0.031	0.07	RPL8	protein_coding	ribosomal p	8	144789765	144792587
ENSG0000020247	1.403808832	0.031	0.07	RNA5SP283	rRNA_pseudog	RNA, 5S ribo	9	62802320	62802438
ENSG0000022688	-2.467489057	0.031	0.07	AL359541.1	lncRNA	novel transc	1	161765325	161766227
ENSG0000025332	0.970588521	0.031	0.07	AZIN1-AS1	lncRNA	AZIN1 antis	8	102864271	103002424
ENSG0000006248	0.090306155	0.031	0.07	CS	protein_coding	citrate synth	12	56271699	56300391
ENSG0000021974	-2.953857669	0.031	0.07	AL133260.1	processed_pse	60S ribosom	6	151099529	151099933
ENSG0000013311	-0.314113186	0.031	0.07	RFXAP	protein_coding	regulatory fa	13	36819222	36829104
ENSG0000022885	0.925617365	0.031	0.07	NRM	protein_coding	nurim [Sour	CHR_HSCHR	30751961	30755334
ENSG0000016739	-0.350028054	0.031	0.07	ZNF668	protein_coding	zinc finger p	16	31060843	31074240
ENSG0000016382	-0.562357252	0.031	0.07	FYCO1	protein_coding	FYVE and cc	3	45917899	45995824
ENSG0000010326	-0.22495664	0.031	0.07	FBXO31	protein_coding	F-box prote	16	87326987	87392142
ENSG0000017627	0.366672025	0.031	0.07	SLC35G1	protein_coding	solute carrie	10	93893973	93956062
ENSG0000018563	1.187707128	0.031	0.07	SHC4	protein_coding	SHC adaptc	15	48823741	48963919
ENSG0000005976	-0.336278249	0.031	0.07	DNAJC25	protein_coding	DnaJ heat sl	9	111631334	111654351
ENSG0000011310	0.351805959	0.031	0.07	APBB3	protein_coding	amyloid bet	5	140558268	140564781
ENSG0000021362	-0.23467213	0.031	0.07	LBH	protein_coding	LBH regulat	2	30231534	30323730
ENSG0000020459	-1.396983195	0.031	0.07	HLA-E	protein_coding	major histoc	6	30489509	30494194
ENSG0000014136	0.082640107	0.031	0.07	CLTC	protein_coding	clathrin hea	17	59619689	59696956
ENSG0000026816	-1.653627244	0.031	0.07	NA	NA	NA	NA	NA	NA
ENSG0000022188	0.463809639	0.031	0.07	ZBED8	protein_coding	zinc finger B	5	160393148	160400054
ENSG0000012907	0.205709161	0.031	0.07	MBD4	protein_coding	methyl-CpG	3	129430944	129440179
ENSG0000017880	-1.020386226	0.031	0.07	ADORA2A-	lncRNA	ADORA2A a	22	24429206	24495074
ENSG0000024930	-1.959730559	0.031	0.07	LINC01088	lncRNA	long interge	4	78939485	79309673
ENSG0000021383	-0.468167626	0.031	0.07	TMX2P1	processed_pse	thioredoxin	9	37885683	37886390
ENSG0000024317	2.647330878	0.031	0.07	AC092944.1	lncRNA	novel transc	3	157174201	157381265
ENSG0000021369	0.22650707	0.031	0.07	SLC35F6	protein_coding	solute carrie	2	26764284	26781231
ENSG0000011134	-1.767907503	0.031	0.07	ARHGDIB	protein_coding	Rho GDP dis	12	14942031	14961728
ENSG0000020377	0.442377011	0.031	0.07	FAM229B	protein_coding	family with s	6	112087591	112102790
ENSG0000014901	0.358236918	0.031	0.07	TUT1	protein_coding	terminal uric	11	62575045	62591637
ENSG0000015617	-0.343312079	0.031	0.07	NDUFAF6	protein_coding	NADH:ubiqu	8	94895767	95116455
ENSG0000021588	0.434420206	0.031	0.07	CYB5RL	protein_coding	cytochrome	1	54169651	54200036
ENSG0000014696	0.35923129	0.031	0.07	DENND2A	protein_coding	DENN doma	7	140518420	140673993
ENSG0000001147	0.831103558	0.032	0.07	QPCTL	protein_coding	glutaminyl-	19	45692666	45703989
ENSG0000017068	-0.375171448	0.032	0.07	CAVIN4	protein_coding	caveolae as	9	100578079	100588389
ENSG0000017374	0.142314481	0.032	0.07	AGFG1	protein_coding	ArfGAP with	2	227472152	227561214
ENSG0000027361	1.228627273	0.032	0.07	NDUFA6-D	lncRNA	NDUFA6 div	CHR_HSCHR	42090956	42125351
ENSG0000013722	-0.233728157	0.032	0.07	TJAP1	protein_coding	tight junctio	6	43477523	43506556

ENSG0000022704	-0.319685174	0.032	0.07	DAXX	protein_coding	death doma	CHR_HSCHR	33240162	33250873
ENSG0000026155	0.559045345	0.032	0.07	AL137782.1	lncRNA	novel transc	13	75549773	75807120
ENSG0000024770	-0.78196018	0.032	0.07	STX18-AS1	lncRNA	STX18 antis	4	4542131	4787359
ENSG0000021549	1.930397405	0.032	0.07	AC007731.	processed_pse	kelch-like 1	22	20450122	20451824
ENSG0000012974	-1.432967656	0.032	0.07	CHRNA10	protein_coding	cholinergic	11	3665587	3671384
ENSG0000016908	-0.74770746	0.032	0.07	DHRSX	protein_coding	dehydroger	X	2219506	2502805
ENSG0000025953	-2.402031058	0.032	0.07	AC051619.5	lncRNA	novel transc	15	45152664	45167526
ENSG0000016564	-0.778903342	0.032	0.07	SLC18A2	protein_coding	solute carrie	10	117241093	117279430
ENSG0000007533	0.313173846	0.032	0.07	TIMM21	protein_coding	translocase	18	74148523	74160531
ENSG0000011522	-0.707344126	0.032	0.07	FNDC4	protein_coding	fibronectin t	2	27491883	27495200
ENSG0000016851	-0.713461471	0.032	0.07	HEXIM2	protein_coding	HEXIM P-TE	17	45160700	45170040
ENSG0000013469	-0.299371359	0.032	0.07	AGO4	protein_coding	argonaute R	1	35808016	35857890
ENSG0000017064	0.551319925	0.032	0.07	NA	NA	NA	NA	NA	NA
ENSG0000016703	0.447444254	0.032	0.07	SGSM1	protein_coding	small G prot	22	24806169	24927578
ENSG0000017516	-0.26214783	0.032	0.07	CADM2	protein_coding	cell adhesio	3	84958981	86074429
ENSG0000016567	0.32003562	0.032	0.07	ENOX2	protein_coding	ecto-NOX c	X	130623369	130903317
ENSG0000016586	1.09146462	0.032	0.07	C10orf82	protein_coding	chromosom	10	116663696	116670264
ENSG0000026266	2.146567853	0.032	0.07	AC087222.	lncRNA	novel transc	17	82918282	82918785
ENSG0000012948	0.233450972	0.032	0.07	PARP2	protein_coding	poly(ADP-ri	14	20343582	20357905
ENSG0000013784	-1.388160496	0.032	0.07	PAK6	protein_coding	p21 (RAC1)	15	40217428	40277487
ENSG0000026327	0.541393789	0.032	0.07	AC004148.	lncRNA	novel transc	17	5425139	5432876
ENSG0000013415	-0.234178471	0.032	0.07	EMC7	protein_coding	ER membra	15	34084017	34101862
ENSG0000027361	1.174138627	0.032	0.07	AL121832.2	lncRNA	novel transc	20	62386303	62386970
ENSG0000019611	-0.654534399	0.032	0.07	TDRD7	protein_coding	tudor doma	9	97412096	97496125
ENSG0000018356	0.886132561	0.032	0.07	SERHL2	protein_coding	serine hydro	22	42553617	42574382
ENSG0000022405	0.442351117	0.032	0.07	CPTP	protein_coding	ceramide-1	1	1324756	1328896
ENSG0000017219	1.065757688	0.032	0.07	MBOAT1	protein_coding	membrane	6	20099684	20212469
ENSG0000027881	1.0060908	0.032	0.07	AC007325.4	protein_coding	protein DG(	KI270734.1	131494	137392
ENSG0000013687	-0.245796315	0.032	0.07	ZNF189	protein_coding	zinc finger p	9	101398873	101410660
ENSG0000012861	-0.783787211	0.032	0.07	OPN1SW	protein_coding	opsin 1, sho	7	128772491	128775790
ENSG0000016975	-0.134987405	0.032	0.07	LIMS1	protein_coding	LIM zinc fing	2	108534355	108687246
ENSG0000014364	0.168441148	0.032	0.07	TTC13	protein_coding	tetratricope	1	230906243	230978875
ENSG0000027309	2.972006914	0.032	0.07	AP000255.	lncRNA	novel transc	21	31735732	31736407
ENSG0000021496	-0.422790233	0.032	0.07	NPIPA7	protein_coding	nuclear pore	16	16379055	16393954
ENSG0000001517	-0.149436257	0.032	0.07	ZMYND11	protein_coding	zinc finger M	10	134465	254637
ENSG0000012544	-0.447106872	0.032	0.07	ARMC7	protein_coding	armadillo re	17	75109952	75130272
ENSG0000020468	0.731641893	0.033	0.07	GABBR1	protein_coding	gamma-am	6	29555629	29633976
ENSG0000020908	0.144712706	0.033	0.07	MT-TL1	Mt_tRNA	mitochondr	MT	3230	3304
ENSG0000026074	-1.344634898	0.033	0.07	AC007823.	lncRNA	novel transc	3	179340322	179341887
ENSG0000024656	1.140537292	0.033	0.07	UBE2D3-AS	lncRNA	UBE2D3 ant	4	102828055	102844075
ENSG0000026052	1.234430916	0.033	0.07	AC109347.	lncRNA	novel transc	4	112229561	112231596
ENSG0000023571	-2.339048859	0.033	0.07	PSMB8	protein_coding	proteasome	CHR_HSCHR	32763034	32767021
ENSG0000013794	0.272671129	0.033	0.07	GTF2B	protein_coding	general trar	1	88852633	88891944
ENSG0000016806	-0.7711677	0.033	0.07	MAP4K2	protein_coding	mitogen-ac	11	64784918	64803241
ENSG0000026323	1.047068424	0.033	0.07	AC217785.	unprocessed_p	glucosidase	CHR_HSCHR	155229777	155234285
ENSG0000002157	-0.171360222	0.033	0.07	SPAST	protein_coding	spastin [Sou	2	32063556	32157637
ENSG0000021457	-1.400793715	0.033	0.07	CPEB1	protein_coding	cytoplasmic	15	82543201	82648861
ENSG0000023388	1.015740515	0.033	0.07	YEATS2-AS	lncRNA	YEATS2 anti	3	183806457	183810783
ENSG0000011230	-0.24550842	0.033	0.07	RPS12	protein_coding	ribosomal p	6	132814569	132817564
ENSG0000013540	0.108746135	0.033	0.07	PRPH	protein_coding	peripherin [	12	49293252	49298686
ENSG0000018850	1.089175253	0.033	0.07	LCTL	protein_coding	lactase like	15	66547179	66565979
ENSG0000013388	-0.157773769	0.033	0.07	DPF2	protein_coding	double PHD	11	65333852	65354262
ENSG0000013232	0.258420938	0.033	0.07	ILKAP	protein_coding	ILK associat	2	238170402	238203708
ENSG0000016079	-0.513240244	0.033	0.07	NBEAL2	protein_coding	neurobeach	3	46979666	47009704
ENSG0000014898	0.369731144	0.033	0.07	PGAP2	protein_coding	post-GPI at	11	3797724	3826371
ENSG0000016344	-0.145495091	0.033	0.07	TMEM183A	protein_coding	transmemb	1	203007374	203024848
ENSG0000020648	0.985235713	0.033	0.07	NRM	protein_coding	nurim [Sour	CHR_HSCHR	30677568	30680941
ENSG0000012772	-0.904137522	0.033	0.07	METTL25	protein_coding	methyltrans	12	82358528	82479239

ENSG0000027795	-0.992669704	0.033	0.07	AC012314.1	protein_coding	pre-mRNA	CHR_HSCHR	54115536	54131896
ENSG0000022459	0.239550032	0.033	0.07	SVIL-AS1	transcribed_un	SVIL antiser	10	29409402	29487745
ENSG0000022621	-0.578149444	0.033	0.07	WASH8P	unprocessed_p	WAS protei	12	14522	32015
ENSG0000010498	0.821769212	0.033	0.07	CCDC61	protein_coding	coiled-coil c	19	45995461	46021318
ENSG0000016552	-0.142030695	0.033	0.07	NEMF	protein_coding	nuclear exp	14	49782083	49852821
ENSG0000017639	0.195825092	0.033	0.07	RNPEP	protein_coding	arginyl amir	1	201982372	202006147
ENSG0000017325	-0.624159632	0.033	0.07	ZNF483	protein_coding	zinc finger p	9	111525159	111577844
ENSG0000000163	0.18320528	0.033	0.07	KRIT1	protein_coding	KRIT1 ankyr	7	92198969	92246166
ENSG0000010126	-0.707211122	0.033	0.07	RASSF2	protein_coding	Ras associat	20	4780023	4823608
ENSG0000025901	0.452279099	0.034	0.07	AL133163.3	lncRNA	novel transc	14	35447003	35447625
ENSG0000009184	0.290565666	0.034	0.07	RGS17	protein_coding	regulator of	6	153004459	153131282
ENSG0000006561	0.183995823	0.034	0.07	CYB5R4	protein_coding	cytochrome	6	83859656	83967423
ENSG0000023217	-0.392349999	0.034	0.07	AL161909.1	processed_pse	ribosomal p	9	19200335	19201046
ENSG0000023494	-1.708793032	0.034	0.07	GTF3C2-AS	lncRNA	GTF3C2 ant	2	27335520	27342599
ENSG0000023759	1.585038842	0.034	0.07	AP000251.1	lncRNA	uncharacter	21	31559245	31560487
ENSG0000000543	0.22325656	0.034	0.07	GCFC2	protein_coding	GC-rich seq	2	75652000	75710985
ENSG0000007480	-0.08387596	0.034	0.07	ENO1	protein_coding	enolase 1 [S	1	8861000	8879190
ENSG0000011609	-0.634876271	0.034	0.07	SPR	protein_coding	sepiapterin	2	72887382	72892158
ENSG0000007831	0.487185815	0.034	0.07	PMS2P1	transcribed_un	PMS1 homc	7	100319222	100336307
ENSG0000023743	-1.167007676	0.034	0.07	CECR7	transcribed_un	cat eye sync	22	17036570	17060825
ENSG0000021457	-3.580682292	0.034	0.07	HMGN2P15	processed_pse	high mobilit	17	42753530	42753799
ENSG0000006555	-0.234113784	0.034	0.07	MAP2K4	protein_coding	mitogen-ac	17	12020829	12143830
ENSG0000027240	-1.024010536	0.034	0.07	AL365181.3	lncRNA	novel transc	1	156641666	156644887
ENSG0000013270	2.089035158	0.034	0.07	HAPLN2	protein_coding	hyaluronan	1	156619331	156625725
ENSG0000027759	-0.368771727	0.034	0.07	DDX52	protein_coding	DExD-box h	CHR_HSCHR	37614320	37648026
ENSG0000025583	1.358035319	0.034	0.07	AC131206.1	processed_pse	M-phase ph	12	7438780	7439990
ENSG0000007435	0.203826088	0.034	0.07	NCBP3	protein_coding	nuclear cap	17	3802158	3846246
ENSG0000019885	-0.551508967	0.034	0.07	FICD	protein_coding	FIC domain	12	108515277	108525837
ENSG0000015812	-0.483349574	0.034	0.07	PRXL2C	protein_coding	peroxiredox	9	96639577	96655317
ENSG0000010367	0.253613281	0.034	0.07	TRIP4	protein_coding	thyroid horr	15	64387748	64455303
ENSG0000024305	-1.282794235	0.034	0.07	EIF4EBP3	protein_coding	eukaryotic t	5	140547662	140549576
ENSG0000015605	0.541456348	0.034	0.07	FAM161B	protein_coding	FAM161 cer	14	73931501	73950414
ENSG0000017484	-0.234892072	0.034	0.07	PDE12	protein_coding	phosphodie	3	57556274	57566848
ENSG0000019629	0.915907335	0.034	0.07	ZNRD1ASP	lncRNA	zinc ribbon	CHR_HSCHR	29990282	30052027
ENSG0000023034	0.333570948	0.034	0.08	PPP1R18	protein_coding	protein pho	CHR_HSCHR	30740303	30751809
ENSG0000025181	-2.560920007	0.034	0.08	RNU6-322P	snRNA	RNA, U6 sm	15	100551820	100551922
ENSG0000010564	-0.40004881	0.034	0.08	RAB3A	protein_coding	RAB3A, mer	19	18196784	18204042
ENSG0000015422	-0.107095896	0.034	0.08	PRKCA	protein_coding	protein kina	17	66302613	66810743
ENSG0000014375	-0.167703661	0.034	0.08	SDE2	protein_coding	SDE2 telom	1	225982702	225999343
ENSG0000025378	-0.860754493	0.035	0.08	AC008429.2	processed_pse	pyrophosph	5	172975511	172976374
ENSG0000027804	3.16963665	0.035	0.08	U2	snRNA	U2 spliceos	17	43251829	43252019
ENSG0000020750	5.242919507	0.035	0.08	RNVU1-14	snRNA	RNA, varian	1	145281116	145281279
ENSG0000027396	-3.670584405	0.035	0.08	NA	NA	NA	NA	NA	NA
ENSG0000013202	1.702364078	0.035	0.08	RTBDN	protein_coding	retbindin [S	19	12825478	12835428
ENSG0000027575	-3.68753963	0.035	0.08	AC026367.3	lncRNA	novel transc	12	118428281	118428870
ENSG0000017846	-0.372863536	0.035	0.08	RPL10P16	processed_pse	ribosomal p	19	12643275	12643919
ENSG0000027796	0.948779305	0.035	0.08	AC006449.6	lncRNA	novel transc	17	38702452	38704747
ENSG0000015120	-0.172813838	0.035	0.08	DLG5	protein_coding	discs large M	10	77790791	77926755
ENSG0000023556	0.638983203	0.035	0.08	MSH5	protein_coding	mutS homo	CHR_HSCHR	31730217	31751987
ENSG0000017610	-1.231423332	0.035	0.08	YES1	protein_coding	YES proto-c	18	721588	812546
ENSG0000015174	0.497885533	0.035	0.08	AMN1	protein_coding	antagonist c	12	31671142	31729121
ENSG0000025759	0.760744671	0.035	0.08	ZNF625	protein_coding	zinc finger p	19	12142090	12156734
ENSG0000013866	-0.205403507	0.035	0.08	AP1AR	protein_coding	adaptor rela	4	112231740	112273110
ENSG0000018914	-0.820825089	0.035	0.08	ZNF573	protein_coding	zinc finger p	19	37735833	37817300
ENSG0000027348	1.384034809	0.035	0.08	AC096992.2	lncRNA	novel transc	3	136837338	136839021
ENSG0000015735	-0.460714642	0.035	0.08	FCSK	protein_coding	fucose kinas	16	70454595	70480274
ENSG0000017782	-0.559289258	0.035	0.08	TENM3-AS	lncRNA	TENM3 anti	4	181874438	182145249
ENSG0000019736	1.485053817	0.035	0.08	ZNF98	protein_coding	zinc finger p	19	22391019	22532485

ENSG0000022365	1.286600536	0.035	0.08	C1GALT1C1	protein_coding	C1GALT1 sp	2	43675151	43676429
ENSG0000007504	-0.307769159	0.035	0.08	KCNQ2	protein_coding	potassium v	20	63400210	63472677
ENSG0000018329	-1.22840269	0.035	0.08	RPSAP19	processed_pse	ribosomal p	1	101786340	101787219
ENSG0000015782	-0.112729924	0.035	0.08	FMNL2	protein_coding	formin like 2	2	152335174	152649826
ENSG0000012422	-0.61962652	0.035	0.08	PPP4R1L	transcribed_un	protein pho	20	58228940	58309451
ENSG0000009962	-0.264773098	0.035	0.08	ATP5F1D	protein_coding	ATP synthas	19	1241746	1244825
ENSG0000012860	0.150736245	0.035	0.08	KLHDC10	protein_coding	kelch doma	7	130070534	130135705
ENSG0000023474	-0.874672316	0.035	0.08	AC144530.1	processed_pse	ribosomal p	3	197850400	197850954
ENSG0000010350	-0.282219899	0.035	0.08	BCKDK	protein_coding	branched ch	16	31106107	31112791
ENSG0000014605	-1.741925023	0.035	0.08	TRIM7	protein_coding	tripartite mo	5	181193924	181205293
ENSG0000016634	0.841957081	0.035	0.08	MSS51	protein_coding	MSS51 mito	10	73423579	73433561
ENSG0000027948	1.095382051	0.035	0.08	AC004623.1	TEC	TEC	19	1372238	1374364
ENSG0000017280	0.627948105	0.035	0.08	SNX32	protein_coding	sorting nexi	11	65833834	65856896
ENSG0000016710	-1.569202746	0.035	0.08	ACSF2	protein_coding	acyl-CoA sy	17	50426158	50474845
ENSG0000013284	-0.158862862	0.035	0.08	AP3B1	protein_coding	adaptor rela	5	78002326	78294698
ENSG0000012908	0.12871076	0.035	0.08	PSMA1	protein_coding	proteasome	11	14504874	14643635
ENSG0000026305	3.022390097	0.035	0.08	AC090617.5	lncRNA	novel transc	17	2043475	2044968
ENSG0000021365	-5.290607983	0.035	0.08	RPL31P44	processed_pse	ribosomal p	10	52389112	52389488
ENSG0000026889	1.79548492	0.035	0.08	A1BG-AS1	lncRNA	A1BG antise	19	58347718	58355455
ENSG0000021475	0.499880172	0.035	0.08	CSKMT	protein_coding	citrate synth	11	62665309	62668496
ENSG0000015742	0.659581908	0.036	0.08	ZNF19	protein_coding	zinc finger p	16	71464555	71565089
ENSG0000022533	2.147610189	0.036	0.08	AL354740.1	lncRNA	novel transc	6	34248535	34286768
ENSG0000027266	2.136448784	0.036	0.08	AL590560.3	lncRNA	uncharacter	1	159854870	159867685
ENSG0000010191	0.268416113	0.036	0.08	PRPS2	protein_coding	phosphoribo	X	12791355	12824222
ENSG0000023098	-1.43928181	0.036	0.08	DSTNP1	processed_pse	DSTN pseud	21	46653558	46654022
ENSG0000025454	-1.422120024	0.036	0.08	AC036111.1	unprocessed_p	Ankyrin repe	11	55867569	55874537
ENSG0000010851	-0.132358105	0.036	0.08	PFN1	protein_coding	profilin 1 [So	17	4945652	4949061
ENSG0000009981	0.137059917	0.036	0.08	MTAP	protein_coding	methylthioa	9	21802636	21937651
ENSG0000021494	-0.390831705	0.036	0.08	NPIPA8	protein_coding	nuclear pore	16	18317942	18336736
ENSG0000021542	-0.356656969	0.036	0.08	ZNF407	protein_coding	zinc finger p	18	74597870	75065671
ENSG0000008476	0.219425575	0.036	0.08	MAPRE3	protein_coding	microtubule	2	26970637	27027219
ENSG0000024584	-0.994251912	0.036	0.08	CEBPA	protein_coding	CCAAT enha	19	33299934	33302534
ENSG0000013551	1.174515841	0.036	0.08	KCNH3	protein_coding	potassium v	12	49539030	49558337
ENSG0000019754	0.359321434	0.036	0.08	ATG7	protein_coding	autophagy r	3	11272309	11557665
ENSG0000015245	0.41192532	0.036	0.08	DCLRE1C	protein_coding	DNA cross-	10	14897359	14954432
ENSG0000012342	-0.518215892	0.036	0.08	EEF1AKMT3	protein_coding	EEF1A lysine	12	57771492	57782541
ENSG0000018701	1.797731982	0.036	0.08	LINC02875	lncRNA	long interge	17	61411751	61413280
ENSG0000011232	-0.226799482	0.036	0.08	SOBP	protein_coding	sine oculis b	6	107490106	107661306
ENSG0000016252	0.306280659	0.036	0.08	KIAA1522	protein_coding	KIAA1522 [S	1	32741830	32774970
ENSG0000025366	1.612625285	0.036	0.08	GASAL1	lncRNA	growth arre	8	102805517	102810039
ENSG0000018755	-2.98686887	0.036	0.08	NANOS3	protein_coding	nanos C2HC	19	13862063	13880757
ENSG0000026315	-4.625521495	0.036	0.08	MYZAP	protein_coding	myocardial z	15	57591904	57685364
ENSG0000018753	0.324533224	0.036	0.08	SIRT7	protein_coding	sirtuin 7 [So	17	81911939	81921323
ENSG0000008915	-0.100305818	0.036	0.08	GCN1	protein_coding	GCN1 activa	12	120127202	120194715
ENSG0000026159	0.609686815	0.036	0.08	HERC2P8	transcribed_un	hect domair	16	33094204	33129090
ENSG0000014949	-0.276987413	0.036	0.08	EML3	protein_coding	EMAP like 3	11	62602218	62612775
ENSG0000027283	2.565339312	0.036	0.08	AC027644.3	lncRNA	novel transc	7	66739829	66740385
ENSG0000011700	-0.166665188	0.036	0.08	RLF	protein_coding	RLF zinc fing	1	40161387	40240921
ENSG0000022597	1.576288171	0.036	0.08	LINC01534	lncRNA	long interge	19	36682636	36687449
ENSG0000022884	-5.152771818	0.036	0.08	RPS20P13	processed_pse	ribosomal p	2	148870024	148870381
ENSG0000026716	-1.439570313	0.036	0.08	AC091152.2	lncRNA	novel transc	17	44673689	44676257
ENSG0000027264	1.989458666	0.036	0.08	AC005020.2	protein_coding	novel protei	7	99558695	99607810
ENSG0000000520	0.215356525	0.037	0.08	SPPL2B	protein_coding	signal pepti	19	2328615	2355095
ENSG0000027831	0.361218554	0.037	0.08	ZNF229	protein_coding	zinc finger p	19	44417519	44448578
ENSG0000013911	0.261071368	0.037	0.08	GABARAPL	protein_coding	GABA type A	12	10212458	10223128
ENSG0000013844	1.299907347	0.037	0.08	SLC40A1	protein_coding	solute carrie	2	189560590	189583758
ENSG0000020465	-3.273436155	0.037	0.08	CBY3	protein_coding	chibby fami	5	179678560	179681034
ENSG0000013559	0.175920931	0.037	0.08	MICAL1	protein_coding	microtubule	6	109444062	109465968

ENSG0000014535	-0.174057021	0.037	0.08	CISD2	protein_coding	CDGSH iron	4	102868974	102892807
ENSG0000027006	1.722742007	0.037	0.08	AL606834.2	lncRNA	novel transc	14	50723777	50724272
ENSG0000018328	-0.701753088	0.037	0.08	PLGLB1	protein_coding	plasminoge	2	87002559	87021852
ENSG0000013620	1.450703542	0.037	0.08	SPDYE1	protein_coding	speedy/RIN	7	43997897	44010122
ENSG0000019644	-0.234154373	0.037	0.08	YRDC	protein_coding	yrdC N6-thr	1	37802945	37808208
ENSG0000014910	-0.117786571	0.037	0.08	EIF3M	protein_coding	eukaryotic t	11	32583798	32606264
ENSG0000027224	3.212638182	0.037	0.08	AL138831.2	lncRNA	novel transc	6	4018713	4019202
ENSG0000008758	0.201922623	0.037	0.08	AURKA	protein_coding	aurora kinas	20	56369389	56392337
ENSG0000018592	-0.151321492	0.037	0.08	PTCH1	protein_coding	patched 1 [S	9	95442980	95517057
ENSG0000017909	0.174263041	0.037	0.08	CYC1	protein_coding	cytochrome	8	144095039	144097525
ENSG0000010435	0.165894725	0.037	0.08	POP1	protein_coding	POP1 homo	8	98117293	98159835
ENSG0000020417	0.465860342	0.037	0.08	AGAP9	protein_coding	ArfGAP with	10	47501854	47523638
ENSG0000011184	0.450506726	0.037	0.08	GCNT2	protein_coding	glucosaminy	6	10492223	10629368
ENSG0000012889	0.320914724	0.037	0.08	CCDC32	protein_coding	coiled-coil c	15	40528683	40565057
ENSG0000018726	-1.234122995	0.037	0.08	WDR86	protein_coding	WD repeat c	7	151375909	151410727
ENSG0000026072	-1.688074673	0.037	0.08	SLC7A5P1	transcribed_un	solute carrie	16	29613103	29613640
ENSG0000013694	0.589380803	0.037	0.08	CTSV	protein_coding	cathepsin V	9	97029677	97039643
ENSG0000018852	-0.132896864	0.037	0.08	SRSF10	protein_coding	serine and a	1	23964347	23980927
ENSG0000011132	0.441201891	0.037	0.08	OGFOD2	protein_coding	2-oxoglutar	12	122974580	122980043
ENSG0000017017	-1.144655882	0.037	0.08	CHRNB1	protein_coding	cholinergic r	17	7445061	7457710
ENSG0000017131	0.247358169	0.038	0.08	EXOSC1	protein_coding	exosome cc	10	97435909	97446017
ENSG0000017238	-0.15476296	0.038	0.08	GNG12	protein_coding	G protein su	1	67701475	67833467
ENSG0000023390	-1.344912948	0.038	0.08	HLA-E	protein_coding	major histoc	CHR_HSCHR	30479321	30484059
ENSG0000014363	0.435497153	0.038	0.08	HCN3	protein_coding	hyperpolariz	1	155277463	155289848
ENSG0000024193	-1.264063725	0.038	0.08	HOGA1	protein_coding	4-hydroxy-2	10	97584323	97612802
ENSG0000013140	1.485303101	0.038	0.08	NAPSA	protein_coding	napsin A asp	19	50358477	50365830
ENSG0000022512	-0.551395848	0.038	0.08	LINC00237	lncRNA	long interge	20	21085576	21106514
ENSG0000025999	-0.906011151	0.038	0.08	AL353796.1	lncRNA	novel transc	10	30302826	30306066
ENSG0000006685	-0.199592436	0.038	0.08	MTFR1	protein_coding	mitochondr	8	65644734	65771261
ENSG0000024622	0.926552319	0.038	0.08	LINC01550	lncRNA	long interge	14	97924637	97978124
ENSG0000018794	-1.555298679	0.038	0.08	C2orf66	protein_coding	chromosom	2	196804415	196810276
ENSG0000005284	-0.128824189	0.038	0.08	TTC17	protein_coding	tetratricope	11	43358920	43494933
ENSG0000016972	0.164936277	0.038	0.08	GPS1	protein_coding	G protein pa	17	82050691	82057470
ENSG0000018411	1.517863743	0.038	0.08	CLDN5	protein_coding	claudin 5 [S	22	19523024	19527545
ENSG0000027271	-1.120465444	0.038	0.08	AC112236.2	lncRNA	novel transc	4	139556799	139557643
ENSG0000016188	0.318803848	0.038	0.08	SPC24	protein_coding	SPC24 comp	19	11131520	11155808
ENSG0000006692	-0.250319025	0.038	0.08	FECH	protein_coding	ferrochelata	18	57544377	57586702
ENSG0000018545	-0.475156968	0.038	0.08	ZSWIM9	protein_coding	zinc finger S	19	48170680	48197620
ENSG0000023536	0.751648209	0.038	0.08	C6orf47	protein_coding	chromosom	CHR_HSCHR	31645706	31648178
ENSG0000010504	-1.860662161	0.038	0.08	TNNT1	protein_coding	troponin T1	19	55132698	55149206
ENSG0000014293	-0.071580358	0.038	0.08	RPS8	protein_coding	ribosomal p	1	44775251	44778779
ENSG0000021581	-0.645400315	0.038	0.08	ZC3H11B	protein_coding	zinc finger C	1	219608010	219613145
ENSG0000015641	0.206857479	0.038	0.08	ATP5MPL	protein_coding	ATP synthas	14	103912288	103928269
ENSG0000015482	-0.641504744	0.038	0.08	PLCL2	protein_coding	phospholipa	3	16802651	17090604
ENSG0000027272	3.229550014	0.038	0.08	AC131235.2	lncRNA	novel transc	3	184134019	184135238
ENSG0000015971	-1.059708593	0.038	0.08	ZDHHC1	protein_coding	zinc finger D	16	67394152	67416833
ENSG0000018917	-0.710435205	0.039	0.08	S100A13	protein_coding	S100 calciur	1	153618787	153631360
ENSG0000017844	-0.403719719	0.039	0.08	COX14	protein_coding	cytochrome	12	50112082	50120457
ENSG0000017116	-0.227425882	0.039	0.08	ZNF692	protein_coding	zinc finger p	1	248850006	248859144
ENSG0000013482	0.210310412	0.039	0.08	TMEM258	protein_coding	transmembr	11	61768501	61792802
ENSG0000022404	1.588983324	0.039	0.08	AC005076.1	lncRNA	novel transc	7	87151422	87152420
ENSG0000018520	-1.999198576	0.039	0.08	IFITM2	protein_coding	interferon ir	11	307631	315272
ENSG0000001556	-0.410613024	0.039	0.08	RGPD5	protein_coding	RANBP2 like	2	109792758	109857705
ENSG0000013485	-0.521876983	0.039	0.08	PDGFRA	protein_coding	platelet deri	4	54229280	54298245
ENSG0000013923	-0.562135292	0.039	0.08	RPL14P1	processed_pse	ribosomal p	12	62965325	62965969
ENSG0000017126	0.157450316	0.039	0.08	FAM98B	protein_coding	family with s	15	38454127	38487710
ENSG0000012269	-0.267253271	0.039	0.08	SLC25A51	protein_coding	solute carrie	9	37879400	37904353
ENSG0000016485	-0.646369524	0.039	0.08	GPER1	protein_coding	G protein-c	7	1082208	1093815

ENSG0000018199	0.214325899	0.039	0.08	MRPS11	protein_coding	mitochondr	15	88467453	88480776
ENSG0000027189	0.649322886	0.039	0.08	AL109811.2	lncRNA	novel transc	1	11029659	11030528
ENSG0000020564	0.66535818	0.039	0.08	CDPF1	protein_coding	cysteine rich	22	46244011	46250311
ENSG0000010175	-0.285250773	0.039	0.08	POLI	protein_coding	DNA polym	18	54269404	54321266
ENSG0000027860	0.573420607	0.039	0.08	AC015871.3	lncRNA	novel transc	15	79920195	79922455
ENSG0000011058	0.206890054	0.039	0.08	NAA40	protein_coding	N-alpha-ac	11	63938959	63957319
ENSG0000016114	0.563562282	0.039	0.08	TUBA3FP	lncRNA	tubulin alph	22	21008193	21014292
ENSG0000008115	-0.110038313	0.039	0.08	PCNP	protein_coding	PEST protec	3	101574180	101594465
ENSG0000017026	-0.357480253	0.039	0.08	FAM161A	protein_coding	FAM161 cer	2	61824848	61854143
ENSG0000027315	0.466527219	0.039	0.08	AL121845.3	protein_coding	novel protei	20	63708864	63739103
ENSG0000022600	1.0244054	0.039	0.08	BX005266.2	lncRNA	novel transc	9	62532337	62534724
ENSG0000014820	-0.984889676	0.039	0.08	NR6A1	protein_coding	nuclear rece	9	124517275	124771311
ENSG0000023601	-0.568365198	0.039	0.08	GPANK1	protein_coding	G-patch do	CHR_HSCHR	31648638	31653693
ENSG0000010761	0.357888749	0.039	0.08	TRDMT1	protein_coding	tRNA aspart	10	17137336	17202054
ENSG0000016773	-0.636851683	0.039	0.08	HSD11B1L	protein_coding	hydroxyster	19	5680604	5688523
ENSG0000027792	2.299274114	0.039	0.08	Telomerase	misc_RNA	Vertebrate t	3	169764610	169765047
ENSG0000020523	-0.738690786	0.039	0.08	AC105052.1	protein_coding	novel protei	7	102582523	102642869
ENSG0000014266	0.349538026	0.039	0.08	SH3BGRL3	protein_coding	SH3 domair	1	26280086	26281522
ENSG0000017301	1.841447641	0.039	0.08	CCDC96	protein_coding	coiled-coil c	4	7040849	7043001
ENSG0000016405	-0.476390707	0.039	0.08	CCDC51	protein_coding	coiled-coil c	3	48432164	48440456
ENSG0000017590	-0.393793791	0.04	0.08	ARL4D	protein_coding	ADP ribosyl	17	43398993	43401137
ENSG0000024816	2.036147367	0.04	0.08	AC098487.1	lncRNA	novel transc	4	102418602	102450010
ENSG0000022850	-3.524041084	0.04	0.08	LINC01760	lncRNA	long interge	1	95310928	95318263
ENSG0000017111	-2.0742001	0.04	0.08	NRTN	protein_coding	neurturin [S	19	5823802	5828324
ENSG0000001491	-0.872845872	0.04	0.09	MTMR11	protein_coding	myotubulari	1	149928651	149936879
ENSG0000023214	0.381750858	0.04	0.09	GNL1	protein_coding	G protein nu	CHR_HSCHR	30586013	30601810
ENSG0000014195	0.297989555	0.04	0.09	PFKL	protein_coding	phosphofru	21	44300051	44327376
ENSG0000016390	0.140781837	0.04	0.09	SENP2	protein_coding	SUMO spec	3	185582496	185633551
ENSG0000023805	1.659606243	0.04	0.09	AL355574.1	lncRNA	novel transc	9	135907812	135916126
ENSG0000011556	-0.156245388	0.04	0.09	CHMP3	protein_coding	charged mu	2	86503430	86563479
ENSG0000013604	-0.447891584	0.04	0.09	PLXNC1	protein_coding	plexin C1 [S	12	94148577	94307675
ENSG0000023056	0.93006694	0.04	0.09	FAM133DP	processed_pse	family with s	2	157379724	157380414
ENSG0000001130	-0.086284271	0.04	0.09	PTBP1	protein_coding	polypyrimid	19	797075	812327
ENSG0000023701	-1.170459873	0.04	0.09	C6orf136	protein_coding	chromosom	CHR_HSCHR	30637275	30643339
ENSG0000017809	0.924328131	0.04	0.09	TSSK6	protein_coding	testis specifi	19	19512418	19515548
ENSG0000015320	-0.111918546	0.04	0.09	AHCTF1	protein_coding	AT-hook co	1	246839098	246931948
ENSG0000008185	-2.412856588	0.04	0.09	PCDHGA2	protein_coding	protocadhe	5	141338760	141512975
ENSG0000018059	0.270322731	0.04	0.09	SKIDA1	protein_coding	SKI/DACH c	10	21513475	21526368
ENSG0000022402	-1.358583564	0.04	0.09	MIR181A2H	lncRNA	MIR181A2 h	9	124658467	124698631
ENSG0000024970	0.467987074	0.04	0.09	ZNF564	protein_coding	zinc finger p	19	12525373	12551542
ENSG0000016594	0.592378841	0.04	0.09	IFI27L1	protein_coding	interferon a	14	94081301	94103846
ENSG0000014138	0.182600101	0.04	0.09	SS18	protein_coding	SS18 subun	18	26016253	26091217
ENSG0000018828	-0.554659912	0.04	0.09	ZNF383	protein_coding	zinc finger p	19	37217926	37248738
ENSG0000023537	1.037288841	0.04	0.09	NA	NA	NA	NA	NA	NA
ENSG0000025398	2.406038583	0.04	0.09	ALG1L13P	transcribed_un	ALG1 like 13	8	8236003	8244667
ENSG0000015804	0.227328205	0.04	0.09	MRPL17	protein_coding	mitochondr	11	6680385	6683340
ENSG0000018224	-0.350542064	0.04	0.09	UBE2E2	protein_coding	ubiquitin co	3	23203020	23591794
ENSG0000022507	0.567562126	0.041	0.09	DDX39B	protein_coding	DExD-box h	CHR_HSCHR	31517671	31534027
ENSG0000014158	0.143017139	0.041	0.09	WDR45B	protein_coding	WD repeat c	17	82614562	82648553
ENSG0000000734	-0.279151058	0.041	0.09	ST7L	protein_coding	suppression	1	112523514	112620825
ENSG0000021515	-0.321751715	0.041	0.09	AC141586.1	transcribed_un	potassium c	16	2603350	2630494
ENSG0000022887	1.032641029	0.041	0.09	SEPTIN7-D	lncRNA	SEPTIN7 div	7	35751856	35800676
ENSG0000014464	-0.433367281	0.041	0.09	POMGNT2	protein_coding	protein O-li	3	43079232	43106079
ENSG0000022754	1.224730876	0.041	0.09	SPAG5-AS1	lncRNA	SPAG5 antis	17	28598790	28617377
ENSG0000010089	-0.314363451	0.041	0.09	PRORP	protein_coding	protein only	14	35121846	35277622
ENSG0000001025	0.172905268	0.041	0.09	UQCRC1	protein_coding	ubiquinol-cy	3	48599002	48610976
ENSG0000024850	0.971900331	0.041	0.09	AL356235.1	transcribed_pr	chromosom	X	77910741	77964890
ENSG0000017943	-0.340540091	0.041	0.09	FJX1	protein_coding	four-jointed	11	35618460	35620865

ENSG0000026607	0.264379682	0.041	0.09	BAHCC1	protein_coding	BAH domai	17	81395475	81466332
ENSG0000013727	0.266840192	0.041	0.09	RIPK1	protein_coding	receptor int	6	3063991	3115187
ENSG0000014099	-0.242594075	0.041	0.09	DEF8	protein_coding	differentially	16	89947925	89968060
ENSG0000025982	1.416237651	0.041	0.09	AC072061.1	lncRNA	novel transc	7	39947522	39949755
ENSG0000017697	0.374256047	0.041	0.09	SHMT1	protein_coding	serine hydro	17	18327860	18363563
ENSG0000027013	-2.527151587	0.041	0.09	MICOS10-N	protein_coding	MICOS10-N	1	19597067	19656927
ENSG0000024412	1.032152288	0.041	0.09	ATP1B3-AS	lncRNA	ATP1B3 ant	3	141918252	141919021
ENSG0000027618	0.507575603	0.041	0.09	H4C9	protein_coding	H4 clustered	6	27138588	27139881
ENSG0000011224	0.151365541	0.041	0.09	ASCC3	protein_coding	activating si	6	100508194	100881372
ENSG0000028033	-1.931058682	0.041	0.09	CTBP2P10	processed_pse	CTBP2 pseu	21	7129103	7130287
ENSG0000016505	0.174614216	0.041	0.09	METTL2B	protein_coding	methyltrans	7	128476729	128506602
ENSG0000002351	-0.131034382	0.041	0.09	AKAP11	protein_coding	A-kinase an	13	42272152	42323261
ENSG0000018882	1.028748182	0.041	0.09	LINC00910	lncRNA	long interge	17	43338741	43389199
ENSG0000000832	0.457396671	0.041	0.09	SS18L2	protein_coding	SS18 like 2		3	42581840	42596934
ENSG0000027570	-0.325318558	0.041	0.09	AATF	protein_coding	apoptosis ar	17	36948954	37056871
ENSG0000027495	-0.328062496	0.041	0.09	ITPK1	protein_coding	inositol-tetr	CHR_HSCHR	92936914	93116320
ENSG0000016275	0.892574995	0.041	0.09	KLHDC9	protein_coding	kelch doma	1	161098361	161100346
ENSG0000018210	0.378794995	0.041	0.09	DEXI	protein_coding	Dexi homol	16	10928891	10942468
ENSG0000016165	-0.217431003	0.041	0.09	LSM12	protein_coding	LSM12 hom	17	44034328	44067619
ENSG0000016465	-0.185645724	0.041	0.09	MIOS	protein_coding	meiosis regu	7	7566875	7608932
ENSG0000011981	0.153888008	0.041	0.09	FAM98A	protein_coding	family with s	2	33583660	33599382
ENSG0000016263	-1.307521642	0.041	0.09	HENMT1	protein_coding	HEN methyl	1	108648290	108661526
ENSG0000022963	-0.549081336	0.041	0.09	RPL4P4	processed_pse	ribosomal p	3	185417495	185418778
ENSG0000016650	-0.131112387	0.042	0.09	HDGFL3	protein_coding	HDGF like 3	15	83112738	83207823
ENSG0000027630	-2.577026881	0.042	0.09	AL021997.3	protein_coding	novel protei	6	28267121	28281580
ENSG0000011126	0.211766749	0.042	0.09	CREBL2	protein_coding	cAMP respo	12	12611827	12645108
ENSG0000027948	-0.504271416	0.042	0.09	AC090498.1	processed_pse	ribosomal p	7	137513859	137513933
ENSG0000020431	0.364282923	0.042	0.09	AGPAT1	protein_coding	1-acylglycer	6	32168212	32178096
ENSG0000026045	-0.95440798	0.042	0.09	NBAT1	lncRNA	neuroblasto	6	22133205	22147193
ENSG0000019783	1.145583971	0.042	0.09	H4-16	protein_coding	H4 histone 1	12	14767999	14771131
ENSG0000013201	-0.616264077	0.042	0.09	C19orf57	protein_coding	chromosom	19	13882348	13906452
ENSG0000027032	2.637110858	0.042	0.09	BNIP3P9	processed_pse	BCL2 intera	19	19856975	19857527
ENSG0000012471	-1.679877499	0.042	0.09	GNMT	protein_coding	glycine N-m	6	42960754	42963880
ENSG0000017764	0.760922537	0.042	0.09	CASC2	lncRNA	cancer susc	10	118046279	118210158
ENSG0000010495	0.244065478	0.042	0.09	CCDC130	protein_coding	coiled-coil c	19	13731760	13763296
ENSG0000017043	0.387978363	0.042	0.09	MGMT	protein_coding	O-6-methy	10	129467190	129770983
ENSG0000017233	-0.553758015	0.042	0.09	ALG14	protein_coding	ALG14 UDP	1	94974405	95072951
ENSG0000011247	2.174922047	0.042	0.09	HSD17B8	protein_coding	hydroxyster	CHR_HSCHR	33133453	33135646
ENSG0000017829	1.348146603	0.042	0.09	TMPRSS9	protein_coding	transmembr	19	2360238	2426239
ENSG0000002130	-1.158646232	0.042	0.09	PLEKHB1	protein_coding	pleckstrin h	11	73646178	73662819
ENSG0000018864	-1.333414619	0.042	0.09	S100A16	protein_coding	S100 calciur	1	153606886	153613145
ENSG0000013101	0.183080284	0.042	0.09	PPIL4	protein_coding	peptidylprol	6	149504495	149546043
ENSG0000020376	0.305115049	0.042	0.09	CENPW	protein_coding	centromere	6	126340115	126348875
ENSG0000000737	-0.294037219	0.042	0.09	RPUSD1	protein_coding	RNA pseudo	16	784974	788397
ENSG0000016360	0.11108338	0.042	0.09	RYBP	protein_coding	RING1 and Y	3	72371825	72446621
ENSG0000026045	0.777092805	0.042	0.09	C16orf95	protein_coding	chromosom	16	87083562	87317420
ENSG0000010122	0.240273862	0.042	0.09	C20orf27	protein_coding	chromosom	20	3753508	3767781
ENSG0000015566	-0.594150722	0.042	0.09	KDM8	protein_coding	lysine deme	16	27203508	27221768
ENSG0000016830	-0.261751211	0.043	0.09	MPLKIP	protein_coding	M-phase sp	7	40126027	40134622
ENSG0000026044	1.755652733	0.043	0.09	ATP2A1-AS	lncRNA	ATP2A1 ant	16	28878957	28879920
ENSG0000017784	-0.496532582	0.043	0.09	ZNF620	protein_coding	zinc finger p	3	40477131	40518736
ENSG0000025524	0.241441254	0.043	0.09	MIR100HG	lncRNA	mir-100-let	11	122028325	122556721
ENSG0000023113	0.358551314	0.043	0.09	MDC1	protein_coding	mediator of	CHR_HSCHR	30692058	30710140
ENSG0000023307	-1.335040139	0.043	0.09	LINC01271	lncRNA	long interge	20	50310711	50321342
ENSG0000016527	-0.378019071	0.043	0.09	TRMT10B	protein_coding	tRNA methy	9	37753803	37778972
ENSG0000022508	5.079027637	0.043	0.09	DAP3P1	unprocessed_p	death assoc	1	155586644	155602197
ENSG0000016151	0.71570073	0.043	0.09	FDXR	protein_coding	ferredoxin re	17	74862497	74873031
ENSG0000016616	0.12594466	0.043	0.09	BRD7	protein_coding	bromodoma	16	50313487	50368988

ENSG0000010806	0.14532273	0.043	0.09	TFAM	protein_coding	transcription	10	58385345	58399220
ENSG0000022606	0.49480175	0.043	0.09	TRIM26	protein_coding	tripartite mo	CHR_HSCHR	30174734	30203710
ENSG0000004854	-1.582320998	0.043	0.09	GUCA1A	protein_coding	guanylate cy	6	42155406	42180049
ENSG0000013512	0.87294044	0.043	0.09	BICDL1	protein_coding	BICD family	12	119989869	120094494
ENSG0000025672	-1.869154221	0.044	0.09	NA	NA	NA	NA	NA	NA
ENSG0000027017	1.288545864	0.044	0.09	AC023509.3	lncRNA	novel transc	12	53500162	53500936
ENSG0000014863	0.167586209	0.044	0.09	HERC4	protein_coding	HECT and R	10	67921899	68075348
ENSG0000020574	-0.234805602	0.044	0.09	AC126755.1	transcribed_un	polycystic ki	16	18334400	18352476
ENSG0000012805	0.126998416	0.044	0.09	PPAT	protein_coding	phosphoribo	4	56393362	56435615
ENSG0000009538	-0.871246155	0.044	0.09	TBC1D2	protein_coding	TBC1 doma	9	98199011	98255649
ENSG0000017034	-0.096870969	0.044	0.09	TMED10	protein_coding	transmembr	14	75131469	75176612
ENSG0000013803	-0.164956435	0.044	0.09	PNPT1	protein_coding	polyribonuc	2	55634061	55693863
ENSG0000014992	0.169158311	0.044	0.09	HIRIP3	protein_coding	HIRA interac	16	29992330	29996074
ENSG0000018590	-0.204720846	0.044	0.09	LINC00839	lncRNA	long interge	10	42475480	42495337
ENSG0000011220	0.249012947	0.044	0.09	BAG2	protein_coding	BAG cochap	6	57172326	57189833
ENSG0000020370	-0.747912724	0.044	0.09	SERTAD4-A	lncRNA	SERTAD4 ar	1	210231456	210234047
ENSG0000011552	-0.272091584	0.044	0.09	COQ10B	protein_coding	coenzyme Q	2	197453423	197475308
ENSG0000016097	0.310564245	0.044	0.09	PPP1R16A	protein_coding	protein pho	8	144477969	144502121
ENSG0000010141	0.193033337	0.044	0.09	RPRD1B	protein_coding	regulation c	20	38033725	38127780
ENSG0000000643	-0.324701889	0.044	0.09	MAP3K9	protein_coding	mitogen-ac	14	70722526	70809534
ENSG0000010522	0.1523375	0.044	0.09	PLD3	protein_coding	phospholipa	19	40348456	40380439
ENSG0000023148	0.820679809	0.044	0.09	ABHD16A	protein_coding	abhydrolase	CHR_HSCHR	31669120	31685587
ENSG0000027321	0.602690865	0.044	0.09	H3-2	protein_coding	H3.2 histone	1	143894544	143905966
ENSG0000015687	0.29978562	0.044	0.09	PHKG2	protein_coding	phosphoryla	16	30748293	30761176
ENSG0000015773	-0.646554928	0.044	0.09	SNX22	protein_coding	sorting nexi	15	64151715	64157481
ENSG0000013215	-0.179330345	0.044	0.09	DHX30	protein_coding	DExH-box h	3	47802909	47850195
ENSG0000010906	-0.235431814	0.045	0.09	NAT9	protein_coding	N-acetyltran	17	74770529	74776367
ENSG0000027080	-2.356724926	0.045	0.09	RPS10-NUD	protein_coding	RPS10-NUD	6	34284887	34426071
ENSG0000018580	-0.251385912	0.045	0.09	SLC52A2	protein_coding	solute carrie	8	144333957	144361286
ENSG0000012128	0.302344453	0.045	0.09	CEP89	protein_coding	centrosoma	19	32875925	32971991
ENSG0000017012	-1.559576801	0.045	0.09	FOXD4	protein_coding	forkhead bo	9	116231	118204
ENSG0000021015	0.567796137	0.045	0.09	MT-TD	Mt_tRNA	mitochondr	MT	7518	7585
ENSG0000025606	-1.806661379	0.045	0.09	AC063926.1	lncRNA	novel transc	12	130419535	130421019
ENSG0000013339	-0.229818255	0.045	0.09	MED10	protein_coding	mediator co	5	6371874	6378571
ENSG0000016956	-0.129702417	0.045	0.09	HINT1	protein_coding	histidine tria	5	131155383	131224468
ENSG0000014846	-0.190203804	0.045	0.09	FAM171A1	protein_coding	family with s	10	15211643	15371289
ENSG0000022463	-0.379083487	0.045	0.09	RPS27AP16	transcribed_pr	ribosomal p	16	61055399	61055964
ENSG0000026745	-0.718025639	0.045	0.09	LINC02073	lncRNA	long interge	17	51336646	51521401
ENSG0000022612	-1.557970089	0.045	0.09	FTCDNL1	protein_coding	formiminotr	2	199760544	199851173
ENSG0000013370	-0.172906347	0.045	0.1	IPO8	protein_coding	importin 8 [	12	30628988	30695869
ENSG0000010147	0.368941362	0.045	0.1	ACOT8	protein_coding	acyl-CoA th	20	45841721	45857405
ENSG0000024003	-0.852661384	0.045	0.1	AMY2B	protein_coding	amylase alp	1	103553815	103579534
ENSG0000023745	-3.210027607	0.045	0.1	LINC01351	lncRNA	long interge	1	190478551	190480735
ENSG0000016751	0.216208342	0.045	0.1	TRAPPC2L	protein_coding	trafficking p	16	88856220	88862686
ENSG0000017579	0.128297301	0.045	0.1	RUVBL1	protein_coding	RuvB like AA	3	128064778	128153914
ENSG0000016770	0.464163423	0.045	0.1	KIFC2	protein_coding	kinesin fami	8	144466043	144474202
ENSG0000020399	-1.099267653	0.045	0.1	LINC01270	lncRNA	long interge	20	50292709	50315488
ENSG0000019748	-0.69130624	0.045	0.1	ZNF628	protein_coding	zinc finger p	19	55476617	55484487
ENSG0000011851	0.191228382	0.045	0.1	RNF146	protein_coding	ring finger p	6	127266682	127288567
ENSG0000022847	-3.039867135	0.045	0.1	AL663070.1	processed_pse	ribosomal p	1	39962680	39963404
ENSG0000003952	-0.266388095	0.046	0.1	RIPOR1	protein_coding	RHO family	16	67518418	67546788
ENSG0000027337	0.57757304	0.046	0.1	AL355488.1	lncRNA	novel transc	1	110370154	110373003
ENSG0000019746	-1.441938148	0.046	0.1	COL13A1	protein_coding	collagen typ	10	69801867	69964275
ENSG0000018209	-0.143026388	0.046	0.1	TNRC18	protein_coding	trinucleotide	7	5306790	5425414
ENSG0000015221	0.201093769	0.046	0.1	ARL14EP	protein_coding	ADP ribosyla	11	30323104	30338223
ENSG0000025931	2.813465523	0.046	0.1	AL356801.1	processed_pse	high mobilit	14	55394940	55395233
ENSG0000012962	0.155912845	0.046	0.1	REEP5	protein_coding	receptor acc	5	112876385	112922289
ENSG0000017340	-0.271971594	0.046	0.1	ARV1	protein_coding	ARV1 homo	1	230978981	231000733

ENSG0000006602	0.19902272	0.046	0.1	PPP2R5A	protein_coding	protein pho		1	212285410	212361853
ENSG0000027990	1.257988706	0.046	0.1	AP001767.4	TEC	TEC		11	83083687	83084138
ENSG0000022780	0.300250995	0.046	0.1	PPP1R10	protein_coding	protein pho	CHR_HSCHR		30590637	30608849
ENSG0000027576	-1.373793741	0.046	0.1	ZNF516-DT	lncRNA	ZNF516 dive		18	76495521	76498088
ENSG0000027683	0.130879152	0.046	0.1	TAF15	protein_coding	TATA-box b	CHR_HSCHR		35809455	35864615
ENSG0000024904	-1.088022615	0.046	0.1	AC008771.1	lncRNA	novel transc		5	80411231	80488095
ENSG0000026985	-0.258658076	0.046	0.1	EGLN2	protein_coding	egl-9 family		19	40798996	40808434
ENSG0000004718	0.150176791	0.046	0.1	YTHDC2	protein_coding	YTH domair		5	113513694	113595285
ENSG0000014732	0.196104689	0.046	0.1	MFHAS1	protein_coding	malignant fi		8	8783354	8893630
ENSG0000027364	-7.159666801	0.046	0.1	NDUFA3	protein_coding	NADH:ubiqu	CHR_HSCHR		54102906	54107026
ENSG0000019651	1.308707937	0.046	0.1	TPK1	protein_coding	thiamin pyro		7	144451941	144836395
ENSG0000023711	0.679537321	0.046	0.1	GABBR1	protein_coding	gamma-am	CHR_HSCHR		29555562	29633117
ENSG0000021406	-0.390399753	0.046	0.1	TSPAN4	protein_coding	tetraspanin		11	842812	867116
ENSG0000020568	0.591995529	0.046	0.1	DPF3	protein_coding	double PHD		14	72619296	72894116
ENSG0000022673	-5.039836785	0.046	0.1	NA	NA	NA	NA		NA	NA
ENSG0000027920	0.242242987	0.046	0.1	AC015813.6	TEC	novel transc		17	58095672	58101479
ENSG0000022625	-0.772514234	0.046	0.1	STK19	protein_coding	serine/threo	CHR_HSCHR		31953357	31965001
ENSG0000012946	-0.202904447	0.046	0.1	NGDN	protein_coding	neuroguidir		14	23469689	23509862
ENSG0000027574	-1.199481356	0.046	0.1	AC091959.3	protein_coding	novel readth		5	146203550	146339251
ENSG0000027826	0.236084797	0.047	0.1	MOAP1	protein_coding	modulator c	CHR_HSCHR		93182196	93184928
ENSG0000011326	0.87563178	0.047	0.1	GRM6	protein_coding	glutamate n		5	178977587	178996206
ENSG0000027928	1.472590778	0.047	0.1	AC131009.4	TEC	novel transc		12	131896500	131898239
ENSG0000027906	1.655776857	0.047	0.1	HEXD-IT1	lncRNA	HEXD intror		17	82425498	82427310
ENSG0000015973	-0.774503165	0.047	0.1	ZFYVE28	protein_coding	zinc finger F		4	2269582	2418651
ENSG0000022668	1.372117436	0.047	0.1	LINC01535	lncRNA	long interge		19	37251885	37265535
ENSG0000013075	-1.631398745	0.047	0.1	NPAS1	protein_coding	neuronal PA		19	47019837	47045775
ENSG0000008650	-0.193011135	0.047	0.1	MRPL28	protein_coding	mitochondr		16	366969	371289
ENSG0000026965	-2.895775843	0.047	0.1	NOP53-AS	lncRNA	NOP53 antis		19	47757036	47768840
ENSG0000024190	4.231908039	0.047	0.1	RPS20P4	processed_pse	ribosomal p		5	150021567	150021921
ENSG0000014980	-0.385526148	0.047	0.1	TM7SF2	protein_coding	transmembr		11	65111845	65116408
ENSG0000010432	0.19253805	0.047	0.1	DECR1	protein_coding	2,4-dienoyl		8	90001405	90053633
ENSG0000013042	0.362286713	0.047	0.1	ARPC1B	protein_coding	actin relatec		7	99374249	99394816
ENSG0000012190	0.472187935	0.047	0.1	ZSCAN20	protein_coding	zinc finger a		1	33472645	33496507
ENSG0000027867	1.657120915	0.047	0.1	H2AC17	protein_coding	H2A cluster		6	27892699	27893185
ENSG0000026160	0.786835625	0.047	0.1	AC233266.2	lncRNA	novel transc		2	91580336	91580863
ENSG0000021144	-0.579304964	0.047	0.1	GPX3	protein_coding	glutathione		5	151020438	151028992
ENSG0000025440	-1.115295635	0.047	0.1	LRRC24	protein_coding	leucine rich		8	144522388	144527033
ENSG0000027553	0.479987345	0.047	0.1	NA	NA	NA	NA		NA	NA
ENSG0000016366	-1.60513749	0.047	0.1	HESX1	protein_coding	HESX home		3	57197838	57227606
ENSG0000013592	-1.049854221	0.047	0.1	TMBIM1	protein_coding	transmembr		2	218274197	218292586
ENSG0000012393	0.132189914	0.047	0.1	MXD4	protein_coding	MAX dimeri		4	2247432	2262109
ENSG0000012652	-0.240814828	0.047	0.1	ASL	protein_coding	argininosuc		7	66075800	66094697
ENSG0000023661	1.134497949	0.047	0.1	PITPNA-AS	lncRNA	PITPNA anti		17	1516931	1518096
ENSG0000023926	0.376856534	0.047	0.1	AC092691.1	lncRNA	novel transc		3	117672154	117997592
ENSG0000011658	0.280135622	0.048	0.1	LAMTOR2	protein_coding	late endoso		1	156054782	156058506
ENSG0000012574	-0.537962219	0.048	0.1	EML2	protein_coding	EMAP like 2		19	45606994	45645629
ENSG0000021145	-1.281250951	0.048	0.1	AKR7L	polymorphic_p	aldo-keto re		1	19265982	19274194
ENSG0000016070	0.414456676	0.048	0.1	NLRX1	protein_coding	NLR family r		11	119166568	119184016
ENSG0000019797	-0.273686156	0.048	0.1	GOLGA6L9	protein_coding	golgin A6 fa		15	82430018	82439153
ENSG0000016550	-1.177570938	0.048	0.1	DEPP1	protein_coding	DEPP1 auto		10	44970981	44978809
ENSG0000009986	0.528754544	0.048	0.1	GADD45B	protein_coding	growth arre		19	2476122	2478259
ENSG0000028014	0.603759149	0.048	0.1	AP000892.4	TEC	novel transc		11	117204967	117210292
ENSG0000016518	0.165053315	0.048	0.1	KIAA1958	protein_coding	KIAA1958 [S		9	112486827	112669397
ENSG0000027404	0.219751451	0.048	0.1	SYNRG	protein_coding	synergin ga	CHR_HSCHR		37519433	37614077
ENSG0000022771	0.914470956	0.048	0.1	AC006042.1	lncRNA	novel transc		7	8114025	8116561
ENSG0000016889	0.267224957	0.048	0.1	RNF181	protein_coding	ring finger p		2	85595725	85597708
ENSG0000018752	-0.161759767	0.048	0.1	HSPA14	protein_coding	heat shock p		10	14838306	14871741
ENSG0000008181	0.319402332	0.048	0.1	PCDHB4	protein_coding	protocadhe		5	141121818	141125623

ENSG0000013584	-0.493583343	0.048	0.1	NIBAN1	protein_coding	niban apopt	1	184790724	184974508
ENSG0000018573	0.371953773	0.048	0.1	ZNF696	protein_coding	zinc finger p	8	143289676	143299952
ENSG0000024627	0.576763215	0.048	0.1	SBF2-AS1	lncRNA	SBF2 antiser	11	9758268	9811335
ENSG0000021321	1.215895531	0.048	0.1	CCDC183	protein_coding	coiled-coil c	9	136796338	136807741
ENSG0000027256	2.12063695	0.048	0.1	AL512343.2	lncRNA	novel transc	1	226045561	226061898
ENSG0000013306	-0.237406412	0.048	0.1	SLC41A1	protein_coding	solute carrie	1	205789094	205813748
ENSG0000016019	0.238941703	0.048	0.1	NDUFV3	protein_coding	NADH:ubiqu	21	42879644	42913304
ENSG0000025509	-5.028772955	0.048	0.1	AC087442.1	lncRNA	novel transc	11	45651529	45652691
ENSG0000021414	-1.870812922	0.048	0.1	LINC00887	lncRNA	long interge	3	194296191	194322871
ENSG0000013843	0.162584293	0.048	0.1	ITPRID2	protein_coding	ITPR interac	2	181891730	181930738
ENSG0000018335	-0.18736507	0.048	0.1	KIAA2026	protein_coding	KIAA2026 [S	9	5881596	6008482
ENSG0000020580	0.460368865	0.048	0.1	PLPP6	protein_coding	phospholipi	9	4662294	4665258
ENSG0000023663	-1.194293442	0.048	0.1	HLA-E	protein_coding	major histoc	CHR_HSCHR	30534109	30538847
ENSG0000010091	-0.153863304	0.048	0.1	REC8	protein_coding	REC8 meiot	14	24171853	24180257
ENSG0000023090	0.964474125	0.048	0.1	ATP6V1G2	protein_coding	ATPase H+	CHR_HSCHR	31620808	31623190
ENSG0000024795	2.061369996	0.048	0.1	SEC24B-AS	lncRNA	SEC24B anti	4	109347475	109433817
ENSG0000007708	0.373703422	0.048	0.1	ACTL6B	protein_coding	actin like 6B	7	100643097	100656448
ENSG0000016962	0.405952707	0.048	0.1	APLF	protein_coding	aprataxin ar	2	68467572	68655862
ENSG0000022428	0.308126737	0.048	0.1	MSL3P1	transcribed_pr	MSL comple	2	233865437	233868444
ENSG0000022850	-0.788329435	0.048	0.1	EEF1A1P11	processed_pse	eukaryotic t	1	96446930	96448318
ENSG0000015987	0.454367111	0.049	0.1	CCDC117	protein_coding	coiled-coil c	22	28772674	28789301
ENSG0000010485	-1.836897082	0.049	0.1	RELB	protein_coding	RELB proto-	19	45001449	45038198
ENSG0000021390	1.640908308	0.049	0.1	LIPE-AS1	lncRNA	LIPE antisen	19	42397128	42652355
ENSG0000026353	-1.328486116	0.049	0.1	AK4P1	processed_pse	adenylate ki	17	31345519	31346190
ENSG0000027259	1.02606115	0.049	0.1	AC016394.1	lncRNA	novel transc	10	73124573	73125532
ENSG0000012433	0.268889556	0.049	0.1	VAMP7	protein_coding	vesicle asso	X	155881345	155943769
ENSG0000022193	0.674146766	0.049	0.1	DENND10P	transcribed_pr	DENND10 p	X	130494965	130496588
ENSG0000026068	-1.461290042	0.049	0.1	AC008669.1	lncRNA	novel transc	5	122832356	122834533
ENSG0000007236	-0.083279244	0.049	0.1	AFF4	protein_coding	AF4/FMR2 f	5	132875395	132963634
ENSG0000016976	-0.165766423	0.049	0.1	UGP2	protein_coding	UDP-glucos	2	63840940	63891562
ENSG0000016804	0.226254699	0.049	0.1	FADD	protein_coding	Fas associat	11	70203296	70207390
ENSG0000008475	-0.082610937	0.049	0.1	HADHA	protein_coding	hydroxyacyl	2	26190635	26244672
ENSG0000013500	0.227594271	0.049	0.1	RFK	protein_coding	riboflavin ki	9	76385526	76394517
ENSG0000019873	0.433162128	0.049	0.1	MSRB1	protein_coding	methionine	16	1938210	1943326
ENSG0000018363	1.71905095	0.049	0.1	TP53TG3	protein_coding	TP53 target	16	32673528	32676732
ENSG0000026728	-1.041601299	0.049	0.1	ATF7-NPFF	protein_coding	ATF7-NPFF	12	53506688	53625979
ENSG0000020328	2.348868382	0.049	0.1	KIAA1671-A	lncRNA	KIAA1671 a	22	25102433	25112692
ENSG0000023401	0.324286019	0.049	0.1	MDC1	protein_coding	mediator of	CHR_HSCHR	30763719	30781800
ENSG0000016843	0.157003906	0.049	0.1	CDC40	protein_coding	cell division	6	110180141	110254275
ENSG0000010169	-0.7365306	0.049	0.1	RNF125	protein_coding	ring finger p	18	32018825	32073219
ENSG0000013368	0.207752891	0.049	0.1	TMTC1	protein_coding	transmembr	12	29500840	29784759
ENSG0000025520	2.380151076	0.049	0.1	AC087623.1	lncRNA	novel transc	8	38421889	38426096
ENSG0000027916	-1.100268082	0.049	0.1	AC093503.2	TEC	TEC	19	46636810	46639161
ENSG0000018602	-0.629911553	0.049	0.1	ZNF284	protein_coding	zinc finger p	19	44072159	44089613
ENSG0000025920	1.716216976	0.049	0.1	AC004943.1	lncRNA	novel transc	16	72805998	72809872
ENSG0000017936	-0.306778833	0.049	0.1	ARID3B	protein_coding	AT-rich inte	15	74541177	74598131
ENSG0000018881	-0.60656741	0.05	0.1	ZDHHC11	protein_coding	zinc finger D	5	795606	850986
ENSG0000012270	-0.13475955	0.05	0.1	CLTA	protein_coding	clathrin ligh	9	36190856	36304781
ENSG0000019893	0.773032376	0.05	0.1	ZFP2	protein_coding	ZFP2 zinc fi	5	178895898	178933212
ENSG0000022520	-1.414461939	0.05	0.1	HLA-E	protein_coding	major histoc	CHR_HSCHR	30567930	30572668
ENSG0000024471	-0.655463777	0.05	0.1	BX679664.3	processed_pse	ribosomal p	1	108992282	108992836
ENSG0000019844	0.618470588	0.05	0.1	ZNF583	protein_coding	zinc finger p	19	56397966	56436035
ENSG0000027787	1.029570094	0.05	0.1	AL391988.1	lncRNA	novel transc	10	117267116	117268668
ENSG0000025627	-2.825209348	0.05	0.1	CACNA1C-	lncRNA	CACNA1C a	12	2668500	2672220
ENSG0000016405	0.374224645	0.05	0.1	ATRIP	protein_coding	ATR interact	3	48446710	48467645
ENSG0000026007	1.751551324	0.05	0.1	AC104794.3	lncRNA	novel transc	2	10039092	10040663
ENSG0000017040	-4.142400754	0.05	0.1	AC091390.1	unprocessed_p	pseudogene	7	102327256	102329530
ENSG0000010036	0.159497913	0.05	0.1	KIAA0930	protein_coding	KIAA0930 [S	22	45190338	45240769

ENSG0000014099	0.404201592	0.05	0.1	TIGD7	protein_coding	tigger trans	16	3298808	3305729
ENSG0000023454	0.637783385	0.05	0.1	ATAT1	protein_coding	alpha tubuli	CHR_HSCHR	30619065	30639047
ENSG0000027634	0.231850323	0.05	0.1	ZNF623	protein_coding	zinc finger p	CHR_HSCHR	143621803	143641845
ENSG0000027801	-0.582120736	0.05	0.1	TIGD5	protein_coding	tigger trans	CHR_HSCHR	143597835	143603224
ENSG0000015652	0.265400058	0.05	0.1	TYSND1	protein_coding	trypsin dom	10	70137981	70146700
ENSG0000027588	0.658865501	0.05	0.1	IKBKGP1	unprocessed_p	inhibitor of	X	154639978	154648275
ENSG0000022347	1.179315461	0.05	0.1	AL441992.1	lncRNA	novel transc	9	128724445	128733194
ENSG0000026724	0.917026716	0.05	0.1	AC025048.2	lncRNA	novel transc	17	60122642	60135743
ENSG0000014501	0.215375198	0.05	0.1	RUBCN	protein_coding	rubicon aut	3	197668867	197749727
ENSG0000010939	0.269339673	0.05	0.1	NDUFC1	protein_coding	NADH:ubiqu	4	139266880	139302551
ENSG0000018411	-0.100557522	0.05	0.1	EIF3C	protein_coding	eukaryotic t	16	28688558	28735727
ENSG0000016222	-0.290030275	0.05	0.1	TTC9C	protein_coding	tetratricope	11	62728069	62740293
ENSG0000026897	1.849288399	0.05	0.1	AC022150.2	lncRNA	novel transc	19	52597699	52598887
ENSG0000008201	-0.423456772	0.05	0.1	SMARCD3	protein_coding	SWI/SNF rel	7	151238764	151277896
ENSG0000013324	0.399894688	0.05	0.1	KMT5C	protein_coding	lysine methy	19	55339853	55348121
ENSG0000017080	0.175645328	0.05	0.1	FOXN2	protein_coding	forkhead bo	2	48314637	48379295
ENSG0000018301	0.298255212	0.05	0.1	NAA38	protein_coding	N-alpha-ac	17	7856685	7885238
ENSG0000009932	-0.36966392	0.05	0.1	MZF1	protein_coding	myeloid zin	19	58561931	58573575
ENSG0000016454	0.19106701	0.051	0.1	STK17A	protein_coding	serine/threo	7	43582758	43650713
ENSG0000027531	-1.26395736	0.051	0.1	AL136981.2	unprocessed_p	novel zinc fi	9	92840955	92841688
ENSG0000019714	0.170274242	0.051	0.1	LRRC8B	protein_coding	leucine rich	1	89524829	89597864
ENSG0000022376	-0.483181809	0.051	0.11	PRR3	protein_coding	proline rich	CHR_HSCHR	30601522	30608359
ENSG0000022694	-1.533557507	0.051	0.11	RNF207-AS	lncRNA	RNF207 ant	1	6204840	6205780
ENSG0000013642	-0.518097201	0.051	0.11	CIB2	protein_coding	calcium and	15	78104606	78131535
ENSG0000025921	-1.629229898	0.051	0.11	AC084855.2	lncRNA	novel transc	15	99976481	99980774
ENSG0000012712	-0.364467914	0.051	0.11	PPCS	protein_coding	phosphopar	1	42456117	42473385
ENSG0000020440	-0.200546322	0.051	0.11	MBD5	protein_coding	methyl-CpG	2	148021011	148516971
ENSG0000026467	-2.757273929	0.051	0.11	SEPTIN4-A	lncRNA	SEPTIN4 ant	17	58519837	58557799
ENSG0000005134	0.220222052	0.051	0.11	POLQ	protein_coding	DNA polym	3	121431427	121546641
ENSG0000010680	0.154226262	0.051	0.11	SEC61B	protein_coding	SEC61 trans	9	99222064	99230615
ENSG0000008708	-1.535100399	0.051	0.11	ACHE	protein_coding	acetylcholin	7	100889994	100896974
ENSG0000027373	1.162004434	0.051	0.11	5_8S_rRNA	rRNA	5.8S ribosor	GL000220.1	155997	156149
ENSG0000019679	-0.170973525	0.051	0.11	STRN3	protein_coding	striatin 3 [So	14	30893799	31026401
ENSG0000024154	-1.795212674	0.051	0.11	ACTG1P20	processed_pse	actin gamm	1	27325329	27325796
ENSG0000023465	-0.554199354	0.051	0.11	BAG6	protein_coding	BAG cochap	CHR_HSCHR	31715314	31728984
ENSG0000027246	1.918748259	0.051	0.11	AL021807.1	lncRNA	novel transc	6	27122657	27123221
ENSG0000010421	-0.229313057	0.051	0.11	CSPP1	protein_coding	centrosome	8	67062417	67196778
ENSG0000026725	-0.74114049	0.052	0.11	ZNF790-AS	lncRNA	ZNF790 ant	19	36797502	36831596
ENSG0000010324	0.331406761	0.052	0.11	MTHFSD	protein_coding	methenyltet	16	86530178	86555235
ENSG0000027869	0.839710733	0.052	0.11	GSTT2B	protein_coding	glutathione	CHR_HSCHR	23957418	23961206
ENSG0000015810	-1.0979353	0.052	0.11	RHPN1	protein_coding	rhophilin Rh	8	143368876	143384221
ENSG0000017047	-0.267463444	0.052	0.11	PYM1	protein_coding	PYM homol	12	55901413	55932618
ENSG0000026749	0.784320001	0.052	0.11	CIRBP-AS1	lncRNA	CIRBP antise	19	1267471	1270260
ENSG0000002883	-0.155917169	0.052	0.11	TBPL1	protein_coding	TATA-box b	6	133952170	133990432
ENSG0000027969	-1.0036709	0.052	0.11	AC110285.6	TEC	TEC	17	81395609	81397144
ENSG0000027301	-1.140737657	0.052	0.11	AC018645.3	lncRNA	novel transc	7	32758882	32759353
ENSG0000016550	-0.210582216	0.052	0.11	DNAAF2	protein_coding	dynein axor	14	49625174	49635244
ENSG0000016882	0.149230561	0.052	0.11	GFM1	protein_coding	G elongatio	3	158644527	158695581
ENSG0000007539	0.449269908	0.052	0.11	VPS9D1	protein_coding	VPS9 doma	16	89707134	89720898
ENSG0000007723	-0.112681942	0.052	0.11	DNAJC10	protein_coding	DnaJ heat sl	2	182716041	182794464
ENSG0000010918	0.124232816	0.052	0.11	DCUN1D4	protein_coding	defective in	4	51843000	51916837
ENSG0000027934	-0.348768097	0.052	0.11	AC012513.3	TEC	TEC	2	216211404	216213519
ENSG0000010119	0.360113933	0.052	0.11	TCFL5	protein_coding	transcription	20	62841005	62861822
ENSG0000024887	0.800860213	0.052	0.11	C5orf17	lncRNA	chromosom	5	23951348	24178263
ENSG0000024163	1.041625438	0.052	0.11	AC069499.1	processed_pse	ribosomal p	3	108543367	108543875
ENSG0000012089	0.528959287	0.052	0.11	PTK2B	protein_coding	protein tyro	8	27311482	27459391
ENSG0000012131	-0.816666506	0.052	0.11	PLBD1	protein_coding	phospholipa	12	14503661	14567883
ENSG0000010034	-0.196081818	0.052	0.11	TXN2	protein_coding	thioredoxin	22	36467046	36481640

ENSG0000019674	1.346169159	0.052	0.11	H2AC13	protein_coding	H2A cluster		6	27808199	27808701
ENSG0000015528	0.347645554	0.052	0.11	SLC25A28	protein_coding	solute carrie		10	99610522	99620609
ENSG0000016719	-1.13008583	0.052	0.11	GPRC5B	protein_coding	G protein-c		16	19856691	19886167
ENSG0000017876	-0.23118651	0.052	0.11	FAM219B	protein_coding	family with s		15	74899992	74906883
ENSG0000016410	0.177099029	0.052	0.11	SAP30	protein_coding	Sin3A assoc		4	173369969	173377532
ENSG0000026061	1.78303681	0.052	0.11	NA	NA	NA	NA		NA	NA
ENSG0000017345	-0.153833855	0.052	0.11	PPP1R14B	protein_coding	protein pho		11	64244479	64246943
ENSG0000023475	-2.754788583	0.052	0.11	FOXP4-AS1	lncRNA	FOXP4 antis		6	41494853	41548621
ENSG0000027746	0.360474543	0.052	0.11	ZNF670	protein_coding	zinc finger p		1	247034637	247078811
ENSG0000021404	0.160669002	0.052	0.11	SMIM7	protein_coding	small integr		19	16630751	16660442
ENSG0000013370	-0.114462359	0.052	0.11	KRAS	protein_coding	KRAS proto		12	25205246	25250936
ENSG0000013797	-0.146357298	0.052	0.11	RPL7P9	processed_pse	ribosomal p		1	96678874	96679620
ENSG0000016739	-0.183967202	0.053	0.11	VKORC1	protein_coding	vitamin K ep		16	31090842	31095980
ENSG0000010239	0.294761415	0.053	0.11	PBDC1	protein_coding	polysacchar	X		76173040	76178314
ENSG0000023035	-1.475526317	0.053	0.11	RPL35AP3	processed_pse	ribosomal p		6	136973930	136974217
ENSG0000016888	-0.138704471	0.053	0.11	USP39	protein_coding	ubiquitin sp		2	85602856	85649283
ENSG0000008709	0.294848321	0.053	0.11	NLK	protein_coding	nemo like ki		17	28041737	28196381
ENSG0000015102	-0.454472578	0.053	0.11	GPR158	protein_coding	G protein-c		10	25174802	25602229
ENSG0000016246	1.456252345	0.053	0.11	SLC25A34	protein_coding	solute carrie		1	15736258	15741392
ENSG0000016060	1.15658858	0.053	0.11	TLCD1	protein_coding	TLC domain		17	28724348	28727935
ENSG0000028032	-2.190673289	0.053	0.11	AC074183.2	TEC	novel transc		7	84939335	84940256
ENSG0000027444	2.102228904	0.053	0.11	AC078909.2	lncRNA	novel transc		15	37109587	37109984
ENSG0000027944	-0.896092012	0.053	0.11	AL513497.1	TEC	TEC		1	28544460	28546542
ENSG0000026298	1.576615356	0.053	0.11	SMIM10L1	processed_pse	small integr	CHR_HSCHR		11203312	11203513
ENSG0000026757	0.455876097	0.053	0.11	AC006504.5	lncRNA	novel transc		19	27793431	27984984
ENSG0000010022	0.567364331	0.053	0.11	RAB36	protein_coding	RAB36, mer		22	23145326	23164350
ENSG0000026712	-1.478767267	0.053	0.11	AC090360.1	protein_coding	novel protei		18	80034346	80097088
ENSG0000010449	0.266421463	0.053	0.11	NCALD	protein_coding	neurocalcin		8	101686542	102124907
ENSG0000010726	0.1566101	0.053	0.11	RAPGEF1	protein_coding	Rap guanine		9	131576770	131740074
ENSG0000024960	1.081533688	0.053	0.11	AL589765.4	lncRNA	novel transc		1	151763384	151769501
ENSG0000010607	-0.398473562	0.053	0.11	GRB10	protein_coding	growth facto		7	50590063	50793462
ENSG0000017381	0.392687432	0.053	0.11	ENDOV	protein_coding	endonuclea		17	80415165	80438086
ENSG0000018418	-0.209745782	0.054	0.11	UBE2F	protein_coding	ubiquitin co		2	237966827	238042782
ENSG0000024411	0.52353103	0.054	0.11	DNAJC25-(	protein_coding	DNAJC25-G		9	111631386	111670229
ENSG0000013563	0.303338869	0.054	0.11	CCDC142	protein_coding	coiled-coil (		2	74471986	74483408
ENSG0000011548	0.079094444	0.054	0.11	CCT4	protein_coding	chaperonin		2	61868085	61888671
ENSG0000025387	1.961151959	0.054	0.11	AC087752.3	lncRNA	novel transc		8	94950037	94951396
ENSG0000013943	-0.228467109	0.054	0.11	TCHP	protein_coding	trichoplein k		12	109900264	109983841
ENSG0000027389	1.013253696	0.054	0.11	AC211476.3	unprocessed_p	PMS2 postn		7	73037371	73049246
ENSG0000022487	0.328150762	0.054	0.11	MRPL20-AS	lncRNA	MRPL20 ant		1	1399520	1402046
ENSG0000022636	1.864945754	0.054	0.11	HAGLROS	lncRNA	HAGLR opp		2	176177717	176179008
ENSG0000011454	-0.25805824	0.054	0.11	SLC41A3	protein_coding	solute carrie		3	126006355	126101561
ENSG0000012956	0.305618201	0.054	0.11	TEP1	protein_coding	telomerase		14	20365667	20413501
ENSG0000016821	-0.225820934	0.054	0.11	LMBRD1	protein_coding	LMBR1 dom		6	69672757	69867236
ENSG0000005496	0.102548411	0.054	0.11	FAM168A	protein_coding	family with s		11	73400487	73598189
ENSG0000023537	0.977126332	0.054	0.11	SSR4P1	transcribed_pr	signal seque		21	45070952	45074165
ENSG0000000752	0.287957693	0.054	0.11	TSR3	protein_coding	TSR3 riboso		16	1349240	1351878
ENSG0000007632	0.178631748	0.054	0.11	KLHL20	protein_coding	kelch like fa		1	173714941	173786692
ENSG0000015945	0.145352209	0.054	0.11	UBR1	protein_coding	ubiquitin pr		15	42942897	43106113
ENSG0000014917	-0.252469992	0.054	0.11	C11orf49	protein_coding	chromosom		11	46936689	47164385
ENSG0000003274	0.375906879	0.054	0.11	IFT88	protein_coding	intraflagella		13	20567069	20691437
ENSG0000014490	-1.291204619	0.054	0.11	ALDH1L1	protein_coding	aldehyde de		3	126103562	126197994
ENSG0000016864	-0.615732381	0.054	0.11	AXIN2	protein_coding	axin 2 [Sour		17	65528563	65561648
ENSG0000023644	-1.768846661	0.054	0.11	PSMB8	protein_coding	proteasome	CHR_HSCHR		32994477	32998463
ENSG0000016550	0.150090735	0.055	0.11	RPL36AL	protein_coding	ribosomal p		14	49618530	49620626
ENSG0000013714	0.219558264	0.055	0.11	IGFBPL1	protein_coding	insulin like g		9	38406528	38424454
ENSG0000008375	-0.29477779	0.055	0.11	RRAGB	protein_coding	Ras related	X		55717749	55758774
ENSG0000010790	-0.897846095	0.055	0.11	LHPP	protein_coding	phospholysi		10	124461823	124617888

ENSG0000005504	-0.110956755	0.055	0.11	NOP58	protein_coding	NOP58 ribo	2	202265736	202303661
ENSG0000018515	-0.427932673	0.055	0.11	LRRC37B	protein_coding	leucine rich	17	32007872	32053504
ENSG0000009556	-0.112571119	0.055	0.11	BTAF1	protein_coding	B-TFIID TAT	10	91923770	92030325
ENSG0000026290	2.316395096	0.055	0.11	AC027796.4	lncRNA	novel transc	17	3655621	3658092
ENSG0000027334	1.104205025	0.055	0.11	AC104109.4	lncRNA	novel transc	5	134205614	134371044
ENSG0000016512	-0.602686194	0.055	0.11	AL353743.1	transcribed_un	kinesin fami	9	85805359	85849542
ENSG0000013504	0.16915253	0.055	0.11	NAA35	protein_coding	N-alpha-ac	9	85941146	86025462
ENSG0000017474	-0.070083673	0.055	0.11	RPL15	protein_coding	ribosomal p	3	23916591	23924374
ENSG0000016203	-1.561022247	0.055	0.11	MEIOB	protein_coding	meiosis spe	16	1833983	1884294
ENSG0000023466	0.866642974	0.055	0.11	ATP6V1G2	protein_coding	ATPase H+	CHR_HSCHR	31526639	31529021
ENSG0000023618	1.67436104	0.055	0.11	TCEA1P4	processed_pse	transcription	9	32979560	32980403
ENSG0000011904	0.138401176	0.055	0.11	GTF3C3	protein_coding	general tran	2	196763035	196799725
ENSG0000011169	0.348732309	0.055	0.11	NT5DC3	protein_coding	5'-nucleotic	12	103770453	103841234
ENSG0000018410	0.624006226	0.055	0.11	BRD7P2	processed_pse	bromodoma	3	160100850	160102793
ENSG0000022918	0.664165206	0.055	0.11	AC006001.3	transcribed_un	RAB guanin	7	66526088	66592397
ENSG0000010958	0.281639945	0.055	0.11	GALNT7	protein_coding	polypeptide	4	173168811	173323967
ENSG0000017416	0.436242873	0.055	0.11	ZDHHC24	protein_coding	zinc finger D	11	66520637	66546235
ENSG0000027957	1.001627152	0.055	0.11	AL162426.1	lncRNA	novel transc	9	127690098	127690840
ENSG0000010699	-0.30164747	0.055	0.11	AK1	protein_coding	adenylate ki	9	127866486	127877675
ENSG0000018146	0.492349628	0.055	0.11	RAP2B	protein_coding	RAP2B, mer	3	153162226	153170627
ENSG0000022405	-2.807818173	0.055	0.11	NA	NA	NA	NA	NA	NA
ENSG0000013070	-0.194059757	0.055	0.11	OSBPL2	protein_coding	oxysterol bi	20	62231922	62296213
ENSG0000027670	1.148948185	0.055	0.11	RNA5-8SN4	rRNA	RNA, 5.8S ri	KI270733.1	128877	129029
ENSG0000011168	0.271007569	0.055	0.11	LPCAT3	protein_coding	lysophospha	12	6976185	7018477
ENSG0000023264	1.283838008	0.056	0.11	AL354892.2	lncRNA	novel transc	6	169725091	169725854
ENSG0000020770	2.964207808	0.056	0.11	MIR548AA1	miRNA	microRNA 5	8	123348034	123348130
ENSG0000013953	-0.787549724	0.056	0.11	CCDC65	protein_coding	coiled-coil c	12	48904110	48931840
ENSG0000022969	0.481435174	0.056	0.11	SOS1-IT1	lncRNA	SOS1 intron	2	38992279	38993857
ENSG0000023705	0.83915039	0.056	0.11	GABBR1	protein_coding	gamma-am	CHR_HSCHR	29555178	29632716
ENSG0000012589	-0.475085718	0.056	0.11	FAM110A	protein_coding	family with s	20	833715	857463
ENSG0000023232	0.656759627	0.056	0.11	MXRA7P1	processed_pse	MXRA7 pse	2	161340816	161341326
ENSG0000022641	1.645915825	0.056	0.11	MRPL23-AS	lncRNA	MRPL23 ant	11	1983237	1989920
ENSG0000013605	0.595500486	0.056	0.11	SLC41A2	protein_coding	solute carrie	12	104802553	104958744
ENSG0000017833	-0.292508657	0.056	0.11	ZNF354B	protein_coding	zinc finger p	5	178859953	178888122
ENSG0000018397	0.283727154	0.056	0.11	COA3	protein_coding	cytochrome	17	42795147	42798704
ENSG0000027315	-0.406746919	0.056	0.11	AC073957.3	lncRNA	novel transc	7	879790	886547
ENSG0000002373	0.097329251	0.056	0.11	STRAP	protein_coding	serine/threo	12	15882387	15903478
ENSG0000025013	0.534059042	0.056	0.11	AC004803.1	lncRNA	novel transc	12	974133	991190
ENSG0000017179	1.042863508	0.056	0.11	SLFNL1	protein_coding	schlafen like	1	41015597	41023237
ENSG0000027826	1.133667187	0.056	0.11	AC079949.2	lncRNA	novel transc	12	127147149	127150081
ENSG0000014805	1.066277132	0.056	0.11	IDNK	protein_coding	IDNK glucor	9	83623049	83644130
ENSG0000011748	-0.993126258	0.056	0.12	FAAH	protein_coding	fatty acid ar	1	46394317	46413848
ENSG0000017822	0.596082804	0.056	0.12	ZNF543	protein_coding	zinc finger p	19	57320472	57330770
ENSG0000011185	0.423186501	0.056	0.12	SMIM8	protein_coding	small integr	6	87322583	87399749
ENSG0000016335	1.334419667	0.056	0.12	DCST1	protein_coding	DC-STAMP	1	155033824	155050930
ENSG0000000233	-0.334066367	0.057	0.12	BAD	protein_coding	BCL2 associ	11	64269830	64284704
ENSG0000012859	-0.368551691	0.057	0.12	LRRC4	protein_coding	leucine rich	7	128027071	128032107
ENSG0000013238	0.136353096	0.057	0.12	RPA1	protein_coding	replication p	17	1829702	1900082
ENSG0000014741	-0.100882804	0.057	0.12	ATP6V1B2	protein_coding	ATPase H+	8	20197381	20226819
ENSG0000020572	0.187005103	0.057	0.12	ITSN1	protein_coding	intersectin 1	21	33642400	33899861
ENSG0000021388	-2.117702353	0.057	0.12	RPL13AP7	transcribed_pr	ribosomal p	21	25361821	25381756
ENSG0000012624	-0.491975957	0.057	0.12	LRFN3	protein_coding	leucine rich	19	35935358	35946624
ENSG0000023573	1.385803593	0.057	0.12	HMGN1P36	processed_pse	high mobilit	2	97827248	97827545
ENSG0000013771	0.294530864	0.057	0.12	FDX1	protein_coding	ferredoxin 1	11	110429948	110464884
ENSG0000022832	-0.558459693	0.057	0.12	AL669831.1	transcribed_un	general tran	1	725885	778626
ENSG0000011913	0.149755117	0.057	0.12	TJP2	protein_coding	tight junctio	9	69121264	69274615
ENSG0000015222	0.26549543	0.057	0.12	EPG5	protein_coding	ectopic P-g	18	45847609	45967329
ENSG0000013486	-0.976020061	0.057	0.12	GGACT	protein_coding	gamma-glu	13	100530164	100589528

ENSG0000027018	-0.534590556	0.057	0.12	NA	NA	NA	NA	NA	NA
ENSG0000020650	-0.182713202	0.057	0.12	HLA-A	protein_coding	major histoc	6	29941260	29945884
ENSG0000018095	0.089565644	0.057	0.12	PITPNB	protein_coding	phosphatidy	22	27851669	27920134
ENSG0000023812	-2.377997154	0.057	0.12	LINC00426	lncRNA	long interge	13	30340267	30377145
ENSG0000012969	0.257763756	0.057	0.12	TTI2	protein_coding	TELO2 inter	8	33473386	33513185
ENSG0000025006	4.944864424	0.057	0.12	AL445187.1	unprocessed_p	vacuolar pro	9	112581779	112582590
ENSG0000027424	-1.652125249	0.057	0.12	AJ011932.1	lncRNA	novel transc	21	45974489	45974953
ENSG0000013280	-0.489595624	0.057	0.12	ZSWIM3	protein_coding	zinc finger S	20	45857614	45879122
ENSG0000023626	1.478506463	0.057	0.12	RPL26P30	transcribed_pr	ribosomal p	11	2335135	2347685
ENSG0000024555	0.841416557	0.057	0.12	AP000787.1	lncRNA	novel transc	11	95150539	95234391
ENSG0000023132	0.983744249	0.057	0.12	LINC01816	lncRNA	long interge	2	70124036	70125317
ENSG0000000653	-0.535499614	0.057	0.12	ALDH3B1	protein_coding	aldehyde de	11	68008578	68029282
ENSG0000026208	2.695527114	0.057	0.12	AC040977.1	lncRNA	novel transc	17	6994642	6995189
ENSG0000011507	0.192143966	0.058	0.12	ACTR1B	protein_coding	actin relatec	2	97655939	97664044
ENSG0000020660	-4.067690276	0.058	0.12	RNU6-431P	snRNA	RNA, U6 sm	4	108652150	108652256
ENSG0000016777	-1.549152278	0.058	0.12	IGFBP6	protein_coding	insulin like g	12	53097436	53102345
ENSG0000015059	0.563862117	0.058	0.12	ADRA2A	protein_coding	adrenocept	10	111077029	111080907
ENSG0000008384	-0.275706146	0.058	0.12	ZNF264	protein_coding	zinc finger p	19	57191500	57222846
ENSG0000021563	-0.71600081	0.058	0.12	GUSBP9	unprocessed_p	GUSB pseuc	5	71197646	71208130
ENSG0000022760	-0.507930231	0.058	0.12	PPT2	protein_coding	palmitoyl-p	CHR_HSCHR	32160569	32179650
ENSG0000018258	1.108192265	0.058	0.12	ACTL10	protein_coding	actin like 10	20	33666498	33668525
ENSG0000007746	0.295427475	0.058	0.12	SIRT6	protein_coding	sirtuin 6 [So	19	4174109	4182566
ENSG0000019649	-1.050476367	0.058	0.12	IPO4	protein_coding	importin 4 [	14	24180219	24188869
ENSG0000015484	-0.174002121	0.058	0.12	PPP4R1	protein_coding	protein pho	18	9546791	9615240
ENSG0000016223	0.198199812	0.058	0.12	STX5	protein_coding	syntaxin 5 [S	11	62806860	62832051
ENSG0000004988	0.378662699	0.058	0.12	PTCD2	protein_coding	pentatricop	5	72320367	72368395
ENSG0000027588	1.208167889	0.058	0.12	AC132872.1	lncRNA	novel transc	17	82244770	82245591
ENSG0000016295	-0.175333148	0.058	0.12	MEMO1	protein_coding	mediator of	2	31865060	32011230
ENSG0000016995	-0.261066181	0.058	0.12	ZNF764	protein_coding	zinc finger p	16	30553764	30558374
ENSG0000000588	-0.594280141	0.058	0.12	ITGA3	protein_coding	integrin sub	17	50055968	50090481
ENSG0000024176	1.177966125	0.058	0.12	AC002467.1	lncRNA	novel transc	7	107739999	107744581
ENSG0000026864	2.097335077	0.058	0.12	AC006486.1	protein_coding	novel protei	19	42234583	42255132
ENSG0000012203	-1.521724339	0.058	0.12	RASL11A	protein_coding	RAS like fan	13	27270830	27275192
ENSG0000023988	0.423335355	0.059	0.12	C1orf226	protein_coding	chromosom	1	162378841	162386812
ENSG0000023033	-1.315670884	0.059	0.12	AC004160.1	lncRNA	novel transc	7	11180902	11520175
ENSG0000027992	1.239271921	0.059	0.12	AL138831.3	TEC	TEC	6	3982673	3984130
ENSG0000027461	-0.614050167	0.059	0.12	AC233968.1	unprocessed_p	aminopeptic	17	38078262	38124770
ENSG0000027172	-2.388211572	0.059	0.12	NA	NA	NA	NA	NA	NA
ENSG0000025904	2.851863624	0.059	0.12	BLOC1S5-T	protein_coding	BLOC1S5-T	6	7881522	8064364
ENSG0000027019	-1.338501442	0.059	0.12	AC097359.2	lncRNA	novel transc	3	37241789	37244177
ENSG0000016388	-0.717310098	0.059	0.12	CAMK2N2	protein_coding	calcium/calr	3	184259213	184261553
ENSG0000017817	0.845637977	0.059	0.12	AMER3	protein_coding	APC membr	2	130755540	130768134
ENSG0000022248	4.912325932	0.059	0.12	NA	NA	NA	NA	NA	NA
ENSG0000027253	4.912325932	0.059	0.12	SNORA28	snoRNA	small nucleo	14	103337849	103337974
ENSG0000016351	0.820348291	0.059	0.12	HDAC11	protein_coding	histone dea	3	13479724	13506424
ENSG0000021145	1.340023358	0.059	0.12	GNRHR2	transcribed_un	gonadotrop	1	145919013	145925341
ENSG0000027169	1.680298033	0.059	0.12	AC233992.2	protein_coding	novel transc	8	144353228	144355609
ENSG0000027186	-1.132200482	0.059	0.12	AC104118.1	lncRNA	novel transc	5	83049376	83050964
ENSG0000023075	-1.511565169	0.059	0.12	RHOQP3	processed_pse	ras homolog	2	130212870	130213490
ENSG0000010162	-0.378541098	0.059	0.12	CEP76	protein_coding	centrosoma	18	12661833	12702777
ENSG0000011685	-0.194678418	0.059	0.12	TMEM9	protein_coding	transmembr	1	201134772	201171574
ENSG0000013721	0.385396758	0.059	0.12	FRS3	protein_coding	fibroblast gr	6	41770176	41786542
ENSG0000011528	0.234800912	0.059	0.12	TTC31	protein_coding	tetratricope	2	74483073	74494559
ENSG0000017684	0.420985904	0.059	0.12	IRX5	protein_coding	iroquois hor	16	54930865	54934485
ENSG0000022665	-0.356788339	0.059	0.12	CLIC1	protein_coding	chloride intr	CHR_HSCHR	31770035	31779217
ENSG0000017787	-0.884486168	0.059	0.12	CCDC184	protein_coding	coiled-coil c	12	48183644	48185926
ENSG0000013813	0.109282128	0.059	0.12	ATAD1	protein_coding	ATPase fam	10	87751512	87841343
ENSG0000019764	0.928237774	0.06	0.12	ZNF433	protein_coding	zinc finger p	19	12014732	12035741

ENSG0000013983	-1.726391235	0.06	0.12	RAB20	protein_coding	RAB20, mer	13	110523066	110561722
ENSG0000013971	0.973362847	0.06	0.12	MORN3	protein_coding	MORN repe	12	121648742	121672631
ENSG0000022843	0.759642344	0.06	0.12	C6orf47	protein_coding	chromosom	CHR_HSCHR	31640476	31642950
ENSG0000022817	0.668929236	0.06	0.12	C6orf47	protein_coding	chromosom	CHR_HSCHR	31697751	31700226
ENSG0000024347	0.928359379	0.06	0.12	NAA80	protein_coding	N-alpha-ac	3	50296402	50299416
ENSG0000028010	1.205996473	0.06	0.12	AL022393.1	TEC	TEC	6	28170845	28172521
ENSG0000006760	1.255036457	0.06	0.12	PMS2P4	transcribed_un	PMS1 homo	7	67287541	67302419
ENSG0000016506	-0.49947723	0.06	0.12	FXN	protein_coding	frataxin [Sou	9	69035751	69079076
ENSG0000014764	-0.251838586	0.06	0.12	SYBU	protein_coding	syntabulin [	8	109573978	109691791
ENSG0000016081	0.384896609	0.06	0.12	PPP1R35	protein_coding	protein pho	7	100435282	100436497
ENSG0000016058	-0.153382289	0.06	0.12	SIK3	protein_coding	SIK family ki	11	116843402	117098437
ENSG0000027887	1.460571555	0.06	0.12	CU638689.	lncRNA	novel transc	21	6667304	6670667
ENSG0000015405	0.20332648	0.06	0.12	IMPACT	protein_coding	impact RWD	18	24426634	24453531
ENSG0000023362	1.004427039	0.06	0.12	LINC01137	lncRNA	long interge	1	37350934	37474411
ENSG0000014610	-0.212981787	0.06	0.12	ABT1	protein_coding	activator of	6	26596953	26600739
ENSG0000019777	0.241530568	0.06	0.12	EME2	protein_coding	essential me	16	1772810	1781708
ENSG0000027602	-0.301068357	0.06	0.12	DUSP14	protein_coding	dual specific	17	37489891	37513501
ENSG0000015320	-0.083659768	0.06	0.12	RANBP2	protein_coding	RAN binding	2	108719482	108785809
ENSG0000019722	-0.24240965	0.06	0.12	C1D	protein_coding	C1D nuclear	2	68041130	68110948
ENSG0000010080	-0.260070291	0.06	0.12	C14orf93	protein_coding	chromosom	14	22985894	23010166
ENSG0000011998	-0.716677379	0.06	0.12	AVPI1	protein_coding	arginine vas	10	97677424	97687241
ENSG0000020451	-0.414671357	0.06	0.12	ZNF814	protein_coding	zinc finger p	19	57848731	57889074
ENSG0000025934	-2.303508091	0.06	0.12	TMC3-AS1	lncRNA	TMC3 antise	15	81324338	81518200
ENSG0000026704	-1.167866763	0.061	0.12	AC027097.	lncRNA	novel transc	18	57630302	57669296
ENSG0000017902	0.805015133	0.061	0.12	TMEM107	protein_coding	transmembr	17	8172457	8176399
ENSG0000022450	3.041736324	0.061	0.12	AC138150.	lncRNA	novel transc	17	45150400	45161510
ENSG0000017953	0.35287702	0.061	0.12	DNHD1	protein_coding	dynein heav	11	6497260	6593758
ENSG0000027238	-1.306567814	0.061	0.12	AC015802.	lncRNA	novel transc	17	76551352	76551750
ENSG0000013414	0.390404854	0.061	0.12	DPH6	protein_coding	diphthamine	15	35217345	35546193
ENSG0000027087	1.931315609	0.061	0.12	AL136038.6	processed_pse	glycine C-a	14	63594031	63595291
ENSG0000024245	1.892326727	0.061	0.12	RBBP4P2	processed_pse	RBBP4 pseu	3	94075912	94077175
ENSG0000017785	-0.182949124	0.061	0.12	ZNF518A	protein_coding	zinc finger p	10	96129715	96205288
ENSG0000027593	-0.274525316	0.061	0.12	DUSP14	protein_coding	dual specific	CHR_HSCHR	37494470	37518136
ENSG0000017156	-0.12583487	0.061	0.12	PLRG1	protein_coding	pleiotropic r	4	154535005	154550400
ENSG0000020653	-0.425426578	0.061	0.12	LNP1	protein_coding	leukemia NU	3	100401532	100456319
ENSG0000025923	2.508620489	0.061	0.12	AC066612.	lncRNA	novel transc	15	48187121	48191691
ENSG0000013278	-0.34553011	0.061	0.12	MUTYH	protein_coding	mutY DNA g	1	45329163	45340893
ENSG0000024381	-1.475475188	0.061	0.12	APOBEC3D	protein_coding	apolipoprot	22	39021113	39033277
ENSG0000027332	0.563062031	0.061	0.12	AC078846.	lncRNA	novel transc	7	129604548	129611630
ENSG0000007128	-0.745579298	0.062	0.12	LMCD1	protein_coding	LIM and cys	3	8501807	8574668
ENSG0000016645	0.767397011	0.062	0.12	C16orf46	protein_coding	chromosom	16	81053497	81077267
ENSG0000011371	0.0862157	0.062	0.12	CSNK1A1	protein_coding	casein kinas	5	149492982	149551471
ENSG0000012948	0.229401165	0.062	0.12	DTD2	protein_coding	D-aminoacy	14	31446036	31457506
ENSG0000017612	1.071744625	0.062	0.12	UFSP1	protein_coding	UFM1 speci	7	100888721	100889715
ENSG0000027155	-3.418882573	0.062	0.12	AC113139.	lncRNA	novel transc	8	52722903	52723141
ENSG0000016421	0.224999102	0.062	0.12	PGGT1B	protein_coding	protein gera	5	115204012	115262877
ENSG0000023585	0.83503096	0.062	0.12	AC006978.	processed_pse	proteasome	7	30371508	30372741
ENSG0000022194	0.491530395	0.062	0.12	TIGD1	protein_coding	tigger trans	2	232543883	232550557
ENSG0000016407	-0.208185208	0.062	0.12	HSPA4L	protein_coding	heat shock p	4	127781821	127840733
ENSG0000027899	0.766335958	0.062	0.12	AC090181.	TEC	TEC	15	77063397	77064910
ENSG0000016179	0.19279016	0.062	0.12	FMNL3	protein_coding	formin like 3	12	49636499	49708165
ENSG0000010493	0.275381964	0.062	0.12	DMPK	protein_coding	DM1 protein	19	45769709	45782552
ENSG0000026198	-1.681135817	0.062	0.12	TAS2R14	protein_coding	taste 2 rece	CHR_HSCHR	10937406	11203521
ENSG0000015885	-0.537450475	0.062	0.12	DMTN	protein_coding	dematin act	8	22048995	22082527
ENSG0000013099	0.769466817	0.062	0.13	POLN	protein_coding	DNA polym	4	2071918	2242121
ENSG0000022758	1.180414027	0.062	0.13	ATP6V1G2	protein_coding	ATPase H+	CHR_HSCHR	31531911	31534293
ENSG0000017602	0.881075192	0.062	0.13	AMIGO3	protein_coding	adhesion m	3	49716829	49719684
ENSG0000026427	1.657346527	0.062	0.13	ZNF236-DT	lncRNA	ZNF236 dive	18	76794732	76822295

ENSG0000027472	-0.218315941	0.062	0.13	ARHGEF10	protein_coding	Rho guanin	CHR_HSCHR		1823976	1958641
ENSG0000012247	0.723479732	0.063	0.13	LRRC39	protein_coding	leucine rich		1	100148448	100178273
ENSG0000026812	2.78398451	0.063	0.13	AC026304.1	lncRNA	novel transc		3	143000907	143001467
ENSG0000016923	0.141990484	0.063	0.13	PRELID1	protein_coding	PRELI doma		5	177303799	177306949
ENSG0000015356	0.14233049	0.063	0.13	UBP1	protein_coding	upstream bi		3	33388336	33441371
ENSG0000015034	-0.177645351	0.063	0.13	ARID5B	protein_coding	AT-rich inte		10	61901684	62096944
ENSG0000007588	-1.426240268	0.063	0.13	TUBA3D	protein_coding	tubulin alph		2	131476119	131482934
ENSG0000011862	0.305908984	0.063	0.13	ZNF430	protein_coding	zinc finger p		19	21020634	21060050
ENSG0000015543	-0.149200826	0.063	0.13	NIFK	protein_coding	nucleolar pr		2	121726945	121736911
ENSG0000021433	-0.44173172	0.063	0.13	AC009053.1	transcribed_un	pyruvate de		16	74332402	74368240
ENSG0000018000	0.106783963	0.063	0.13	SOCS4	protein_coding	suppressor c		14	55027230	55049489
ENSG0000023629	0.759367924	0.063	0.13	GUSBP5	transcribed_pr	GUSB pseuc		4	143559472	143561460
ENSG0000025666	1.901350328	0.063	0.13	AC025423.2	processed_pse	ribosomal L		12	68841946	68842384
ENSG0000010756	-0.770105139	0.063	0.13	CXCL12	protein_coding	C-X-C moti		10	44370165	44386493
ENSG0000001016	0.179429495	0.063	0.13	EEF1AKNM	protein_coding	eEF1A lysine		1	171781660	171814023
ENSG0000010535	-0.262324272	0.063	0.13	PLIN3	protein_coding	perilipin 3 [S		19	4838341	4867694
ENSG0000017642	-0.34493668	0.063	0.13	SPRYD4	protein_coding	SPRY domai		12	56468578	56479708
ENSG0000011619	-0.172200958	0.063	0.13	CEP104	protein_coding	centrosoma		1	3812086	3857396
ENSG0000026929	-2.373491915	0.063	0.13	NA	NA	NA	NA	NA		NA
ENSG0000016594	0.217239319	0.064	0.13	MOAP1	protein_coding	modulator c		14	93182199	93184923
ENSG0000016626	0.562061292	0.064	0.13	FAM227B	protein_coding	family with s		15	49326962	49620929
ENSG0000018178	0.107151885	0.064	0.13	COPG1	protein_coding	COPI coat c		3	129249606	129277773
ENSG0000026269	-1.395007494	0.064	0.13	AC040160.1	lncRNA	novel transc		16	67261108	67263784
ENSG0000005673	-0.781539418	0.064	0.13	IL17RB	protein_coding	interleukin 1		3	53846568	53865794
ENSG0000027742	0.330547625	0.064	0.13	HMOX2	protein_coding	heme oxyge	CHR_HSCHR		4476649	4512306
ENSG0000012288	-0.180818221	0.064	0.13	ECD	protein_coding	ecdysoneles		10	73130155	73169055
ENSG0000022335	-1.644650086	0.064	0.13	AL590666.1	lncRNA	novel transc		1	156712212	156713174
ENSG0000012677	-0.109674762	0.064	0.13	PCNX4	protein_coding	pecanex 4 [S		14	60091911	60169133
ENSG0000001840	0.31081239	0.065	0.13	WWTR1	protein_coding	WW domair		3	149517235	149736714
ENSG0000018122	1.864085791	0.065	0.13	DLSTP1	processed_pse	dihydrolipoa		1	75743423	75744776
ENSG0000015206	0.202125596	0.065	0.13	RABGAP1L	protein_coding	RAB GTPase		1	174159410	174995308
ENSG0000017211	-0.456640303	0.065	0.13	NME6	protein_coding	NME/NM23		3	48290722	48301685
ENSG0000027006	1.365676318	0.065	0.13	AC090589.3	lncRNA	novel transc		11	47168281	47169563
ENSG0000011178	-0.131442984	0.065	0.13	SRSF9	protein_coding	serine and a		12	120461672	120469748
ENSG0000024963	1.251858585	0.065	0.13	AC008438.1	lncRNA	novel transc		5	140370891	140401460
ENSG0000012681	0.182590058	0.065	0.13	TRMT5	protein_coding	tRNA methy		14	60971441	60981170
ENSG0000022484	-0.461268956	0.065	0.13	RGL2	protein_coding	ral guanine	CHR_HSCHR		33213258	33220928
ENSG0000023743	-1.229242205	0.065	0.13	ASS1P12	processed_pse	argininosuc		9	32945996	32947222
ENSG0000017712	-0.167995152	0.065	0.13	ZBTB34	protein_coding	zinc finger a		9	126860665	126885878
ENSG0000017456	-0.971770676	0.065	0.13	IL20RB	protein_coding	interleukin 2		3	136946230	137011085
ENSG0000021528	2.211369177	0.065	0.13	HMGB3P24	processed_pse	high mobilit		9	36303499	36304924
ENSG0000026314	0.743344614	0.065	0.13	PAXBP1	protein_coding	PAX3 and P	CHR_HSCHR		32769814	32771858
ENSG0000027724	4.044019935	0.065	0.13	AL157762.1	lncRNA	novel transc		13	108267320	108267734
ENSG0000027797	-0.410844968	0.065	0.13	CISD3	protein_coding	CDGSH iron		17	38730341	38735605
ENSG0000022760	1.040029698	0.065	0.13	NA	NA	NA	NA	NA		NA
ENSG0000015353	0.804730494	0.065	0.13	ADPRHL1	protein_coding	ADP-ribosyl		13	113399610	113453524
ENSG0000026990	-3.044877893	0.065	0.13	NA	NA	NA	NA	NA		NA
ENSG0000017320	0.106065157	0.065	0.13	CKS1B	protein_coding	CDC28 prot		1	154974653	154979251
ENSG0000016876	-0.122954776	0.065	0.13	TET2	protein_coding	tet methylcy		4	105145875	105279816
ENSG0000024128	1.915514068	0.065	0.13	LINC02614	lncRNA	long interge		3	125827238	125916384
ENSG0000019883	-0.835510355	0.065	0.13	GJC2	protein_coding	gap junctior		1	228149930	228159826
ENSG0000022993	0.876211361	0.065	0.13	YWHAZP3	processed_pse	tyrosine 3-r		10	23136924	23137661
ENSG0000010532	-0.426221284	0.065	0.13	CCDC9	protein_coding	coiled-coil c		19	47255980	47273701
ENSG0000015844	0.276996666	0.065	0.13	KCNB1	protein_coding	potassium v		20	49293394	49484297
ENSG0000016582	-0.211558982	0.065	0.13	SALL2	protein_coding	spalt like tra		14	21521080	21537216
ENSG0000025377	3.351363674	0.065	0.13	C8orf37-AS	lncRNA	C8orf37 ant		8	95204456	95811254
ENSG0000012478	-0.414134699	0.065	0.13	RPP40	protein_coding	ribonuclease		6	4994717	5004063
ENSG0000017401	-0.180317448	0.065	0.13	KLHL15	protein_coding	kelch like fa	X		23983720	24027186

ENSG0000010894	0.091669584	0.065	0.13	PRKAR1A	protein_coding	protein kina	17	68511780	68551319
ENSG0000017117	1.054240862	0.065	0.13	RBKS	protein_coding	ribokinase [	2	27781379	27890681
ENSG0000023620	1.041735245	0.066	0.13	LINC01376	lncRNA	long interge	2	18986451	19348067
ENSG0000013583	-0.505504754	0.066	0.13	NPL	protein_coding	N-acetylneu	1	182789293	182830384
ENSG0000024188	2.781318057	0.066	0.13	AC112496.1	lncRNA	novel transc	X	30698207	30721932
ENSG0000023432	-1.533707628	0.066	0.13	AL604028.2	processed_pse	ribosomal p	1	45651039	45651826
ENSG0000018095	-0.663271492	0.066	0.13	ST20	protein_coding	suppressor	15	79898840	79923702
ENSG0000026134	-0.729721818	0.066	0.13	AC006538.1	lncRNA	novel transc	19	2727743	2729327
ENSG0000012544	0.172224655	0.066	0.13	GGA3	protein_coding	golgi associ	17	75236599	75262363
ENSG0000019781	1.761150139	0.066	0.13	AC011450.1	lncRNA	novel transc	19	49331525	49340303
ENSG0000010663	1.673182285	0.066	0.13	GCK	protein_coding	glucokinase	7	44143213	44198170
ENSG0000026291	0.380769307	0.066	0.13	CCNQ	protein_coding	cyclin Q [So	X	153587925	153600045
ENSG0000009068	0.130179402	0.066	0.13	USP48	protein_coding	ubiquitin sp	1	21678298	21783606
ENSG0000014195	-0.313773246	0.066	0.13	PRDM15	protein_coding	PR/SET dom	21	41798225	41879482
ENSG0000020354	1.100650469	0.066	0.13	AL139353.1	protein_coding	novel protei	14	31334312	31457441
ENSG0000013362	0.231012559	0.066	0.13	ACTR3B	protein_coding	actin relatec	7	152759749	152855378
ENSG0000013288	-0.697001879	0.066	0.13	CPLANE2	protein_coding	ciliogenesis	1	16231692	16237183
ENSG0000010061	0.133223801	0.066	0.13	PPM1A	protein_coding	protein pho	14	60245752	60299087
ENSG0000027905	1.328609408	0.066	0.13	AC141586.4	TEC	tec	16	2620222	2621794
ENSG0000022620	-1.860981364	0.066	0.13	PSMB8	protein_coding	proteasome	CHR_HSCHR	32874193	32878179
ENSG0000023161	-0.284278437	0.066	0.13	DAXX	protein_coding	death doma	CHR_HSCHR	33488900	33499609
ENSG0000023971	-1.064678816	0.067	0.13	APOBEC3G	protein_coding	apolipoprot	22	39077067	39087743
ENSG0000012584	0.155808537	0.067	0.13	RRBP1	protein_coding	ribosome bi	20	17613678	17682295
ENSG0000013734	0.686078314	0.067	0.13	ATAT1	protein_coding	alpha tubuli	6	30626842	30646823
ENSG0000023791	2.311543865	0.067	0.13	RPL37P12	processed_pse	ribosomal p	2	109552536	109552846
ENSG0000016697	0.157372417	0.067	0.13	MAPRE2	protein_coding	microtubule	18	34976928	35143470
ENSG0000022486	0.328741753	0.067	0.13	YBX1P1	processed_pse	Y-box bindi	14	66012830	66013789
ENSG0000011991	-0.134092545	0.067	0.13	IDE	protein_coding	insulin degr	10	92451684	92574093
ENSG0000012873	-0.11727577	0.067	0.13	HERC2	protein_coding	HECT and R	15	28111040	28322179
ENSG0000016800	-0.431741954	0.067	0.13	BSCL2	protein_coding	BSCL2 lipid	11	62690275	62709845
ENSG0000027002	1.133174884	0.067	0.13	BMS1P7	transcribed_un	BMS1 pseuc	10	48050282	48060016
ENSG0000024455	-1.912530899	0.067	0.13	KCNK15-AS	lncRNA	KCNK15 anc	20	44694892	44746021
ENSG0000022994	0.336428395	0.067	0.13	EIF4EP2	processed_pse	eukaryotic t	17	49424269	49424922
ENSG0000014325	-1.275328466	0.067	0.13	NR1I3	protein_coding	nuclear rece	1	161229666	161238244
ENSG0000025528	1.212354237	0.067	0.13	AP006621.3	lncRNA	novel transc	11	777578	784297
ENSG0000011071	-0.188936709	0.067	0.13	NDUFS8	protein_coding	NADH:ubiqu	11	68030617	68036644
ENSG0000025888	2.219412601	0.067	0.13	LINC02321	lncRNA	long interge	14	90820064	90828199
ENSG0000017198	-0.751033776	0.067	0.13	SHLD1	protein_coding	shieldin con	20	5750393	5864395
ENSG0000023045	1.220601773	0.067	0.13	U73166.1	lncRNA	novel transc	3	50260303	50263358
ENSG0000006561	0.128194142	0.067	0.13	SLK	protein_coding	STE20 like k	10	103967140	104029233
ENSG0000023254	1.71205657	0.067	0.13	AC253536.3	lncRNA	novel transc	22	24101689	24103354
ENSG0000009989	-0.223296774	0.067	0.13	TRMT2A	protein_coding	tRNA methy	22	20111875	20117392
ENSG0000027237	0.798466363	0.067	0.13	Z97832.2	lncRNA	novel transc	6	35220370	35224630
ENSG0000010188	0.45327469	0.067	0.13	NXT2	protein_coding	nuclear tran	X	109535781	109544690
ENSG0000010525	-0.335942652	0.067	0.13	POLR2I	protein_coding	RNA polyme	19	36113709	36115213
ENSG0000013915	-0.159797696	0.067	0.13	AEBP2	protein_coding	AE binding	12	19404045	19720801
ENSG0000011621	-0.250601883	0.067	0.13	WRAP73	protein_coding	WD repeat	1	3630767	3652761
ENSG0000014411	0.302153535	0.068	0.13	THNSL2	protein_coding	threonine sy	2	88170274	88186636
ENSG0000001007	-0.190120314	0.068	0.13	SPRTN	protein_coding	SprT-like N	1	231337104	231355023
ENSG0000018461	0.587536522	0.068	0.13	KRBA2	protein_coding	KRAB-A dor	17	8356902	8376704
ENSG0000021155	1.804171245	0.068	0.13	NA	NA	NA	NA	NA	NA
ENSG0000028023	-0.673315837	0.068	0.13	AC011498.7	TEC	TEC	19	4448810	4450836
ENSG0000020443	0.639575117	0.068	0.13	C6orf47	protein_coding	chromosom	6	31658298	31660778
ENSG0000018523	0.162945283	0.068	0.13	PRMT3	protein_coding	protein argi	11	20387558	20509338
ENSG0000027606	-0.358116895	0.068	0.13	NAIP	protein_coding	NLR family	CHR_HSCHR	70312214	70368830
ENSG0000023904	2.221966819	0.068	0.13	Y_RNA	misc_RNA	Y RNA [Sou	4	55412636	55412738
ENSG0000014739	-1.083260507	0.068	0.13	ZNF185	protein_coding	zinc finger p	X	152914442	152973480
ENSG0000010053	0.286217794	0.068	0.13	CGRRF1	protein_coding	cell growth	14	54509812	54539292

ENSG0000016337	-0.58339108	0.068	0.13	TAFA4	protein_coding	TAFA chem	3	68731766	68953297
ENSG0000027923	0.574967119	0.068	0.13	AC122688.3	TEC	novel transc	12	125138245	125141711
ENSG0000026547	-1.164678341	0.068	0.14	DTX2P1-UP	lncRNA	DTX2P1-UP	7	76959835	77043775
ENSG0000021313	0.601267903	0.068	0.14	CRYGS	protein_coding	crystallin ga	3	186538441	186546702
ENSG0000010636	-0.158672127	0.068	0.14	AP1S1	protein_coding	adaptor rela	7	101154456	101161596
ENSG0000027729	1.120276146	0.068	0.14	AC136475.1	processed_pse	signal recog	11	243099	243483
ENSG0000014479	0.359477305	0.068	0.14	ZNF660	protein_coding	zinc finger p	3	44578223	44599694
ENSG0000007927	0.31393167	0.068	0.14	MKNK1	protein_coding	MAPK intera	1	46557407	46616843
ENSG0000010113	0.183610932	0.068	0.14	CSTF1	protein_coding	cleavage sti	20	56392371	56406362
ENSG0000014069	0.262152645	0.068	0.14	PARN	protein_coding	poly(A)-spe	16	14435700	14632728
ENSG0000027452	1.617360664	0.068	0.14	AC090970.2	lncRNA	novel transc	15	52017167	52018032
ENSG0000011689	-0.192903159	0.068	0.14	MRPS15	protein_coding	mitochondr	1	36455718	36464384
ENSG0000010326	0.526554159	0.068	0.14	METRN	protein_coding	meteorin, gl	16	715118	719655
ENSG0000017462	-0.58617855	0.069	0.14	IQCK	protein_coding	IQ motif cor	16	19716456	19858467
ENSG0000019759	1.89708474	0.069	0.14	CCDC154	protein_coding	coiled-coil c	16	1434383	1444556
ENSG0000025499	0.168220986	0.069	0.14	BRK1	protein_coding	BRICK1 subu	3	10115675	10127190
ENSG0000019638	1.270948725	0.069	0.14	INCA1	protein_coding	inhibitor of	17	4988130	4997610
ENSG0000016381	0.19620187	0.069	0.14	ZDHHC3	protein_coding	zinc finger D	3	44915257	44976185
ENSG0000004048	-0.372432918	0.069	0.14	SLC66A1	protein_coding	solute carrie	1	19312326	19329300
ENSG0000013949	-0.126730773	0.069	0.14	NUP58	protein_coding	nucleoporin	13	25301556	25349800
ENSG0000017597	0.120136827	0.069	0.14	UNC119B	protein_coding	unc-119 lipi	12	120710458	120723640
ENSG0000010885	-0.367258042	0.069	0.14	MPP2	protein_coding	membrane p	17	43875357	43909711
ENSG0000026982	0.797852659	0.069	0.14	AC022150.4	lncRNA	novel transc	19	52650437	52653284
ENSG0000017233	0.250596708	0.069	0.14	POP7	protein_coding	POP7 homo	7	100706121	100707486
ENSG0000019691	0.358109992	0.069	0.14	ANKRD36B	protein_coding	ankyrin repe	2	97492663	97589965
ENSG0000010081	-0.131666245	0.069	0.14	TRIP11	protein_coding	thyroid horr	14	91965991	92040896
ENSG0000015871	-0.689829464	0.069	0.14	SLC45A3	protein_coding	solute carrie	1	205657851	205680509
ENSG0000017522	-0.236791388	0.069	0.14	MED16	protein_coding	mediator co	19	867630	893218
ENSG0000014541	0.436799922	0.069	0.14	MARCHF1	protein_coding	membrane a	4	163524298	164384050
ENSG0000026933	0.375789895	0.069	0.14	IKBKG	protein_coding	inhibitor of	X	154541199	154565046
ENSG0000013782	0.154657892	0.069	0.14	RMDN3	protein_coding	regulator of	15	40735884	40755851
ENSG0000014296	-0.878083945	0.069	0.14	MOB3C	protein_coding	MOB kinase	1	46607715	46616891
ENSG0000017880	0.762278964	0.069	0.14	MPI	protein_coding	mannose ph	15	74890005	74902219
ENSG0000027196	2.003211751	0.069	0.14	AC021321.1	lncRNA	novel transc	8	67343975	67345087
ENSG0000012846	0.133008247	0.07	0.14	EMC4	protein_coding	ER membra	15	34225013	34230156
ENSG0000016803	1.005322909	0.07	0.14	ULK4	protein_coding	unc-51 like	3	41246599	41962130
ENSG0000018270	-0.24526653	0.07	0.14	TSKU	protein_coding	tsukushi, sm	11	76782251	76798153
ENSG0000015922	0.230746929	0.07	0.14	CBR1	protein_coding	carbonyl red	21	36069941	36073166
ENSG0000023235	1.944753321	0.07	0.14	HSD17B8	protein_coding	hydroxyster	CHR_HSCHR	33375002	33377191
ENSG0000005655	-0.428078812	0.07	0.14	TRAF1	protein_coding	TNF recepto	9	120902393	120929173
ENSG0000024924	-1.085897436	0.07	0.14	TMEM150C	protein_coding	transmembr	4	82483170	82562357
ENSG0000020380	1.245652872	0.07	0.14	ADAMTSL4	lncRNA	ADAMTSL4	1	150560202	150574552
ENSG0000011465	0.189769451	0.07	0.14	SCAP	protein_coding	SREBF chap	3	47413681	47477126
ENSG0000019734	0.12477468	0.07	0.14	ZNF655	protein_coding	zinc finger p	7	99558406	99576453
ENSG0000013943	0.318670082	0.07	0.14	GLTP	protein_coding	glycolipid tr	12	109850945	109880541
ENSG0000021501	1.251360193	0.07	0.14	AL645728.1	lncRNA	novel transc	1	1574102	1577075
ENSG0000012851	0.212072535	0.07	0.14	POT1	protein_coding	protection c	7	124822386	124929983
ENSG0000012356	0.086366053	0.07	0.14	MORF4L2	protein_coding	mortality fac	X	103675496	103688158
ENSG0000027153	-0.3802452	0.071	0.14	Z83843.1	lncRNA	novel transc	X	74209976	74213660
ENSG0000015383	0.634187343	0.071	0.14	FBXO36	protein_coding	F-box prote	2	229922302	230013119
ENSG0000026133	1.466130841	0.071	0.14	AC005837.1	lncRNA	novel transc	17	76671942	76673658
ENSG0000010259	0.174929739	0.071	0.14	UGGT2	protein_coding	UDP-glucos	13	95801580	96053482
ENSG0000018534	0.157664049	0.071	0.14	ATP6V0A2	protein_coding	ATPase H+	12	123712353	123761755
ENSG0000024934	0.598940536	0.071	0.14	UGDH-AS1	lncRNA	UGDH antis	4	39528019	39594707
ENSG0000009491	0.069612319	0.071	0.14	CBX5	protein_coding	chromobox	12	54230942	54280133
ENSG0000017029	-0.216791564	0.071	0.14	ELP5	protein_coding	elongator a	17	7251416	7259940
ENSG0000027674	-1.141898103	0.071	0.14	AL445649.1	lncRNA	novel transc	13	107465984	107466674
ENSG0000019859	-0.47611725	0.071	0.14	MMP17	protein_coding	matrix meta	12	131828393	131851783

ENSG0000018376	0.256671527	0.071	0.14	CHEK2	protein_codinc	checkpoint I	22	28687743	28742422
ENSG0000010809	-0.134592084	0.071	0.14	CCDC6	protein_codinc	coiled-coil c	10	59788747	59906556
ENSG0000027515	1.576970563	0.071	0.14	AC027348.:	lncRNA	novel transc	16	49847018	49847632
ENSG0000014131	-0.474588567	0.071	0.14	RHBDL3	protein_codinc	rhomboid li	17	32265832	32324659
ENSG0000000515	-0.128762663	0.071	0.14	LIG3	protein_codinc	DNA ligase	17	34980512	35009743
ENSG0000013582	0.138203805	0.071	0.14	STX6	protein_codinc	syntaxin 6 [S	1	180972712	181023121
ENSG0000018870	-0.181242828	0.071	0.14	ZDHHC9	protein_codinc	zinc finger D	X	129803288	129843909
ENSG0000020497	0.925114595	0.071	0.14	PCDHA1	protein_codinc	protocadhe	5	140786136	141012347
ENSG0000018365	0.351010133	0.071	0.14	KLHL25	protein_codinc	kelch like fa	15	85759326	85794925
ENSG0000017880	1.028996135	0.072	0.14	TRIM73	protein_codinc	tripartite mc	7	75395063	75410996
ENSG0000009055	-2.417830842	0.072	0.14	FLT3LG	protein_codinc	fms related	19	49474207	49486231
ENSG0000010388	-0.513016524	0.072	0.14	CEMIP	protein_codinc	cell migratic	15	80779343	80951776
ENSG0000025056	1.95535018	0.072	0.14	NTAN1P2	processed_pse	N-terminal	8	86481754	86483002
ENSG0000023390	2.965230946	0.072	0.14	NA	NA	NA	NA	NA	NA
ENSG0000024848	-0.757725937	0.072	0.14	ABHD14A	protein_codinc	abhydrolase	3	51971426	51981199
ENSG0000023450	0.185636032	0.072	0.14	BRD2	protein_codinc	bromodoma	CHR_HSCHR	32890988	32903839
ENSG0000021376	0.858426766	0.072	0.14	ATP6V1G2	protein_codinc	ATPase H+	6	31544462	31548427
ENSG0000025855	0.798700845	0.072	0.14	AC005519.:	lncRNA	novel transc	14	74289127	74294425
ENSG0000017846	-1.063536487	0.072	0.14	MCMDC2	protein_codinc	minichromc	8	66870749	66922048
ENSG0000014422	-0.09346585	0.072	0.14	UBXN4	protein_codinc	UBX domair	2	135741734	135785056
ENSG0000013065	-0.604951684	0.072	0.14	PNPLA7	protein_codinc	patatin like	9	137459952	137550402
ENSG0000023238	-1.160803216	0.072	0.14	AC015712.:	lncRNA	novel transc	15	100849561	100861756
ENSG0000026626	-3.015871895	0.073	0.14	AC005324.!	lncRNA	novel transc	17	15651590	15654489
ENSG0000024965	3.325011056	0.073	0.14	AC008434.:	lncRNA	novel transc	5	80630313	80631590
ENSG0000011845	0.206174526	0.073	0.14	ANKRD13C	protein_codinc	ankyrin repe	1	70258999	70354734
ENSG0000027133	0.941500492	0.073	0.14	AL117336.2	lncRNA	novel transc	10	35314552	35336401
ENSG0000020628	-0.460571708	0.073	0.14	RGL2	protein_codinc	ral guanine	CHR_HSCHR	33220485	33228152
ENSG0000016799	-0.766334319	0.073	0.14	VWCE	protein_codinc	von Willebra	11	61258286	61295316
ENSG0000006637	-1.038276374	0.073	0.14	ZNRD1	protein_codinc	zinc ribbon	6	30058899	30064909
ENSG0000010823	-0.333690504	0.073	0.14	TBC1D12	protein_codinc	TBC1 doma	10	94402541	94536332
ENSG0000021525	0.282669232	0.073	0.14	DHRS4-AS:	lncRNA	DHRS4 antis	14	23938219	24052555
ENSG0000026646	-0.236732246	0.073	0.14	AC005288.:	lncRNA	novel transc	17	39401793	39406233
ENSG0000007306	-0.153749225	0.073	0.14	SCARB1	protein_codinc	scavenger re	12	124776856	124882668
ENSG0000016829	0.346308485	0.073	0.14	PXK	protein_codinc	PX domain c	3	58332880	58426127
ENSG0000013034	0.361252815	0.073	0.14	RTN4IP1	protein_codinc	reticulon 4 i	6	106570771	106629498
ENSG0000016531	-0.533211015	0.073	0.14	OTUD1	protein_codinc	OTU deubic	10	23439458	23442390
ENSG0000011509	0.100672977	0.073	0.14	ACTR3	protein_codinc	actin relatec	2	113890063	113962596
ENSG0000011331	0.225865544	0.073	0.14	MSH3	protein_codinc	mutS homo	5	80654652	80876815
ENSG0000011170	0.122458583	0.073	0.14	SUDS3	protein_codinc	SDS3 homo	12	118376555	118418033
ENSG0000025487	0.817130984	0.073	0.14	SUGT1P4-S	lncRNA	SUGT1P4-S	9	97238449	97297314
ENSG0000025542	0.739903691	0.073	0.14	EBLN2	protein_codinc	endogenous	3	73061659	73063337
ENSG0000026155	-0.273872228	0.073	0.14	SMG1P7	transcribed_un	SMG1 pseuc	16	70219574	70246612
ENSG0000022838	-1.346660724	0.074	0.14	NA	NA	NA	NA	NA	NA
ENSG0000015957	0.124250063	0.074	0.14	RSPRY1	protein_codinc	ring finger a	16	57186137	57240469
ENSG0000016622	0.146468274	0.074	0.14	FRS2	protein_codinc	fibroblast gr	12	69470349	69579789
ENSG0000020474	-0.456116385	0.074	0.14	AC083899.:	unprocessed_p	anaphase pr	2	87125198	87196629
ENSG0000014217	-0.190771254	0.074	0.14	COL6A2	protein_codinc	collagen typ	21	46098112	46132848
ENSG0000018404	0.130872311	0.074	0.14	DIABLO	protein_codinc	diablo IAP-I	12	122207663	122227456
ENSG0000011381	0.219822085	0.074	0.14	ACTR8	protein_codinc	actin relatec	3	53867066	53882152
ENSG0000023287	4.791349016	0.074	0.14	AL591806.1	processed_pse	glutaredoxir	1	161034834	161035006
ENSG0000008969	0.872999602	0.074	0.14	LAG3	protein_codinc	lymphocyte	12	6772512	6778455
ENSG0000025496	-2.606017005	0.074	0.15	NA	NA	NA	NA	NA	NA
ENSG0000017172	1.020640919	0.074	0.15	SPATA46	protein_codinc	spermatoge	1	162373203	162376854
ENSG0000018706	0.733742525	0.074	0.15	TMEM262	protein_codinc	transmembr	11	65084979	65089375
ENSG0000011454	-0.117983361	0.074	0.15	FRMD4B	protein_codinc	FERM doma	3	69168782	69542583
ENSG0000015750	0.164989638	0.074	0.15	APPL1	protein_codinc	adaptor pro	3	57227726	57278105
ENSG0000012470	-0.12415643	0.074	0.15	KLHDC3	protein_codinc	kelch doma	6	43014103	43021298
ENSG0000017316	0.357244649	0.074	0.15	RAPH1	protein_codinc	Ras associat	2	203394345	203535335

ENSG0000025533	-0.578465811	0.074	0.15	AL133352.1	protein_coding	NADH dehy		10	100505628	100529881
ENSG0000016575	0.367799604	0.074	0.15	STK32C	protein_coding	serine/threo		10	132207492	132331847
ENSG0000027306	1.514106517	0.074	0.15	AC017083.1	lncRNA	novel transc		2	68252870	68253848
ENSG0000027704	0.32556515	0.075	0.15	NA	NA	NA	NA		NA	NA
ENSG0000017933	0.170179728	0.075	0.15	RAB39A	protein_coding	RAB39A, me		11	107928448	107963482
ENSG0000018215	0.158824046	0.075	0.15	ERCC6L2	protein_coding	ERCC excisio		9	95871264	96121154
ENSG0000011943	0.522528182	0.075	0.15	HDHD3	protein_coding	haloacid de		9	113373419	113376999
ENSG0000023689	1.07176088	0.075	0.15	GTF2H4	protein_coding	general tran	CHR_HSCHR		30898049	30903971
ENSG0000025893	0.953774594	0.075	0.15	AL162311.3	lncRNA	novel transc		14	35819224	35826765
ENSG0000011207	-0.088178855	0.075	0.15	KCTD20	protein_coding	potassium c		6	36442767	36491143
ENSG0000021525	-0.205854617	0.075	0.15	FASTKD5	protein_coding	FAST kinase		20	3146519	3159865
ENSG0000013229	-0.112868176	0.075	0.15	EFR3A	protein_coding	EFR3 homol		8	131904093	132013642
ENSG0000026080	-1.228300955	0.075	0.15	AC092803.1	lncRNA	novel transc		1	212557833	212559731
ENSG0000017058	0.179384324	0.075	0.15	NUDCD2	protein_coding	NudC doma		5	163446526	163460102
ENSG0000023323	1.182343465	0.075	0.15	AC079807.1	lncRNA	novel transc		2	47905678	47907810
ENSG0000016772	-0.154380831	0.075	0.15	TSR1	protein_coding	TSR1 riboso		17	2322396	2336657
ENSG0000026147	1.130266971	0.075	0.15	AC026471.4	lncRNA	novel transc		16	31449535	31453493
ENSG0000009990	-0.201540809	0.075	0.15	ZDHHC8	protein_coding	zinc finger D		22	20129456	20148007
ENSG0000022583	-0.199611824	0.075	0.15	ERCC6	protein_coding	ERCC excisio		10	49454470	49539538
ENSG0000019684	0.651044393	0.075	0.15	ARID5A	protein_coding	AT-rich inte		2	96536743	96552634
ENSG0000025385	0.510428523	0.075	0.15	AC010834.3	lncRNA	novel transc		8	93719574	93721167
ENSG0000012900	0.101207564	0.075	0.15	VPS13C	protein_coding	vacuolar pro		15	61852389	62060473
ENSG0000017115	0.303112319	0.075	0.15	C1GALT1C1	protein_coding	C1GALT1 sp	X		120625674	120630054
ENSG0000027574	-1.052680451	0.075	0.15	ARL17B	protein_coding	ADP ribosyla	CHR_HSCHR		45699087	45820912
ENSG0000016411	0.073134501	0.076	0.15	CEP44	protein_coding	centrosoma		4	174283730	174333380
ENSG0000024893	0.738641183	0.076	0.15	AC046134.2	lncRNA	novel transc		3	139389761	139782699
ENSG0000009530	-0.875614439	0.076	0.15	PTGS1	protein_coding	prostagland		9	122370530	122395703
ENSG0000026046	0.741688341	0.076	0.15	AL133355.1	lncRNA	novel transc		10	103877374	103879761
ENSG0000025817	-1.115837083	0.076	0.15	LINC02412	lncRNA	long interge		12	93173470	93182299
ENSG0000013052	1.082541113	0.076	0.15	TRPM4	protein_coding	transient rec		19	49157741	49211836
ENSG0000022445	-0.425510783	0.076	0.15	VPS52	protein_coding	VPS52 subu	CHR_HSCHR		33420661	33442416
ENSG0000010884	0.081474617	0.076	0.15	LUC7L3	protein_coding	LUC7 like 3		17	50719565	50756219
ENSG0000017083	0.93053261	0.076	0.15	CEL	protein_coding	carboxyl est		9	133061978	133071863
ENSG0000026312	1.003774755	0.076	0.15	AC040162.3	lncRNA	novel transc		16	67882461	67886367
ENSG0000026313	2.879746102	0.076	0.15	NA	NA	NA	NA		NA	NA
ENSG0000011335	-0.482782318	0.076	0.15	POLR3G	protein_coding	RNA polyme		5	90471748	90514557
ENSG0000015274	0.245500736	0.076	0.15	GPR180	protein_coding	G protein-c		13	94601857	94634661
ENSG0000026776	-2.665442466	0.077	0.15	AC100793.3	lncRNA	novel transc		17	42683187	42699466
ENSG0000016214	1.10247715	0.077	0.15	PPP1R32	protein_coding	protein pho		11	61481120	61490931
ENSG0000023013	-2.006610308	0.077	0.15	LINC02812	lncRNA	long interge		1	53366656	53368245
ENSG0000013360	-0.123191749	0.077	0.15	MKRN1	protein_coding	makorin ring		7	140453033	140479536
ENSG0000027742	-0.358153844	0.077	0.15	SERF1B	protein_coding	small EDRK-	CHR_HSCHR		70045915	70063796
ENSG0000022892	-0.81410407	0.077	0.15	RPS13P2	processed_pse	ribosomal p		1	52772194	52772648
ENSG0000012032	0.354693374	0.077	0.15	PCDHB14	protein_coding	protocadhe		5	141222932	141227759
ENSG0000022380	-2.753573826	0.077	0.15	AC244669.1	transcribed_un	Poly [ADP-r		1	120267334	120341871
ENSG0000024021	-1.338175928	0.077	0.15	AL512306.2	lncRNA	novel transc		1	204626775	204629712
ENSG0000013782	-0.143780185	0.077	0.15	TUBGCP4	protein_coding	tubulin gam		15	43369221	43409771
ENSG0000026991	0.765351146	0.077	0.15	AL049840.4	lncRNA	novel transc		14	103694516	103695050
ENSG0000022561	0.679473947	0.077	0.15	ZNRD1ASP	lncRNA	zinc ribbon	CHR_HSCHR		29990956	30051969
ENSG0000023062	0.161579485	0.077	0.15	DDX39B	protein_coding	DExD-box h	CHR_HSCHR		31512399	31528755
ENSG0000022676	-1.189854269	0.077	0.15	SRRM5	protein_coding	serine/argin		19	43596617	43614498
ENSG0000018344	0.546696131	0.077	0.15	OR7E38P	unprocessed_p	olfactory rec		7	97966090	97967074
ENSG0000014869	0.197782001	0.077	0.15	FRA10AC1	protein_coding	FRA10A ass		10	93667883	93702592
ENSG0000018239	0.98336285	0.077	0.15	DNM1P46	transcribed_un	dynamin 1 p		15	99790156	99806927
ENSG0000014155	0.10517019	0.078	0.15	CSNK1D	protein_coding	casein kinas		17	82239023	82273731
ENSG0000010208	0.136729385	0.078	0.15	FMR1	protein_coding	FMRP transl	X		147911919	147951125
ENSG0000014654	0.211755903	0.078	0.15	C7orf50	protein_coding	chromosom		7	996986	1138260
ENSG0000017205	-0.148620666	0.078	0.15	QARS1	protein_coding	glutaminyl-		3	49095932	49105130

ENSG0000021398	0.258794304	0.078	0.15	ZNF90	protein_coding	zinc finger p	19	20077994	20127076
ENSG0000022592	1.108049228	0.078	0.15	RIMKLBP2	processed_pse	ribosomal m	1	219199914	219200567
ENSG0000027900	-2.215411958	0.078	0.15	NA	NA	NA	NA	NA	NA
ENSG0000009310	0.788715576	0.078	0.15	AC016026.1	lncRNA	novel transc	22	17787652	17811497
ENSG0000003821	-0.110839651	0.078	0.15	BOD1L1	protein_coding	biorientation	4	13568738	13627725
ENSG0000023060	-0.330531759	0.078	0.15	AC092683.1	lncRNA	novel transc	2	97416165	97433527
ENSG0000022393	-0.499657545	0.078	0.15	GPANK1	protein_coding	G-patch do	CHR_HSCHR	31700683	31705737
ENSG0000015255	0.085580964	0.078	0.15	TMEM123	protein_coding	transmembr	11	102396332	102470384
ENSG0000026091	2.922164516	0.078	0.15	AC100835.1	lncRNA	novel transc	15	74613194	74615596
ENSG0000012543	-0.610737398	0.078	0.15	HS3ST3B1	protein_coding	heparan sul	17	14301081	14349404
ENSG0000020205	1.653803526	0.078	0.15	RN7SKP80	misc_RNA	RN7SK pseu	22	42565048	42565330
ENSG0000012204	-0.235780969	0.078	0.15	UBL3	protein_coding	ubiquitin lik	13	29764371	29850617
ENSG0000012239	-0.203433416	0.078	0.15	NAA60	protein_coding	N-alpha-ac	16	3443649	3486953
ENSG0000027402	-3.966198082	0.078	0.15	AC024909.1	lncRNA	novel transc	12	89351015	89353271
ENSG0000016181	0.111117877	0.078	0.15	LARP4	protein_coding	La ribonucle	12	50392383	50480004
ENSG0000013602	-0.116045899	0.078	0.15	CKAP4	protein_coding	cytoskeleton	12	106237881	106304279
ENSG0000015671	-0.627035778	0.078	0.15	MAPK13	protein_coding	mitogen-ac	6	36127809	36144524
ENSG0000016331	0.210263253	0.078	0.15	MRPS18C	protein_coding	mitochondr	4	83455932	83469735
ENSG0000019815	-1.461680184	0.078	0.15	NPIPB6	protein_coding	nuclear pore	16	28342555	28363508
ENSG0000014625	1.048740568	0.079	0.15	PRSS35	protein_coding	serine prote	6	83512534	83525704
ENSG0000016629	0.126761559	0.079	0.15	ANAPC16	protein_coding	anaphase p	10	72216000	72235860
ENSG0000021966	-0.579190981	0.079	0.15	ZNF433-AS	lncRNA	ZNF433 anc	19	11987617	12046275
ENSG0000024620	-0.822823195	0.079	0.15	AL353807.3	unprocessed_p	novel pseuc	1	155614726	155660245
ENSG0000010561	-1.228330686	0.079	0.15	TFPT	protein_coding	TCF3 fusion	19	54107013	54115675
ENSG0000027651	1.016715131	0.079	0.15	AL133243.3	lncRNA	novel transc	2	32526504	32529507
ENSG0000017602	-0.728084412	0.079	0.15	ZNF613	protein_coding	zinc finger p	19	51927147	51948759
ENSG0000027418	0.590218095	0.079	0.15	NATD1	protein_coding	N-acetyltrar	17	21238870	21253410
ENSG0000012438	0.150205973	0.079	0.15	MPHOSPH1	protein_coding	M-phase ph	2	71130632	71150101
ENSG0000011940	-0.315433858	0.079	0.15	TRIM32	protein_coding	tripartite mo	9	116687302	116701300
ENSG0000011345	0.178392695	0.079	0.15	RAD1	protein_coding	RAD1 check	5	34905260	34918989
ENSG0000017905	0.076597322	0.079	0.15	RCC2	protein_coding	regulator of	1	17406760	17439677
ENSG0000020598	0.211584252	0.079	0.15	DNAJC19	protein_coding	DnaJ heat sl	3	180983709	180989774
ENSG0000017838	-0.323956586	0.08	0.15	PLEKHM3	protein_coding	pleckstrin ho	2	207821288	208025527
ENSG0000023793	2.226822313	0.079	0.15	AL450998.2	lncRNA	novel transc	1	15720312	15736896
ENSG0000025359	-1.07542125	0.08	0.15	SLC10A5	protein_coding	solute carrie	8	81693631	81695058
ENSG0000013173	1.683246586	0.08	0.15	CKMT2	protein_coding	creatine kin	5	81233320	81266398
ENSG0000027429	-0.470384528	0.08	0.15	AC084018.2	lncRNA	novel transc	12	121800797	121803403
ENSG0000015898	0.135177341	0.08	0.15	RAPGEF6	protein_coding	Rap guanine	5	131423921	131635231
ENSG0000022798	-1.05351566	0.08	0.15	TRIM60P18	unprocessed_p	tripartite mo	7	64355078	64356199
ENSG0000016631	-0.162340892	0.08	0.15	APBB1	protein_coding	amyloid bet	11	6395124	6419414
ENSG0000011741	-0.196171143	0.08	0.15	ERI3	protein_coding	ERI1 exoribo	1	44221070	44355260
ENSG0000026460	2.425551664	0.08	0.15	NA	NA	NA	NA	NA	NA
ENSG0000016266	0.548697892	0.08	0.15	HFM1	protein_coding	helicase for	1	91260766	91404856
ENSG0000024456	-0.85207973	0.08	0.16	AC004890.2	transcribed_un	zinc finger p	7	149285281	149297312
ENSG0000008749	-0.240461426	0.08	0.16	PHACTR3	protein_coding	phosphatase	20	59577509	59847711
ENSG0000008730	-0.110751229	0.08	0.16	RTRAF	protein_coding	RNA transcr	14	51989514	52010694
ENSG0000020546	0.453706498	0.08	0.16	ATP6AP1L	protein_coding	ATPase H+	5	82279462	82386977
ENSG0000011406	0.089132201	0.08	0.16	UBE3A	protein_coding	ubiquitin pr	15	25333728	25439051
ENSG0000000883	0.128967455	0.08	0.16	MED24	protein_coding	mediator co	17	40019097	40061215
ENSG0000025979	-1.100134788	0.081	0.16	NA	NA	NA	NA	NA	NA
ENSG0000011944	-0.156021502	0.081	0.16	RBM18	protein_coding	RNA binding	9	122237622	122264840
ENSG0000017450	0.320295047	0.081	0.16	ANKRD36C	protein_coding	ankyrin repe	2	95836919	95991831
ENSG0000012583	0.19205608	0.081	0.16	STK35	protein_coding	serine/threo	20	2101827	2177038
ENSG0000017347	-0.090465256	0.081	0.16	SMARCC1	protein_coding	SWI/SNF rel	3	47585269	47782106
ENSG0000016424	-0.115795028	0.081	0.16	PRRC1	protein_coding	proline rich	5	127517640	127555085
ENSG0000025370	1.539254135	0.081	0.16	AC023632.2	lncRNA	novel transc	8	94553668	94570648
ENSG0000023620	1.086845837	0.081	0.16	KDM4A-AS	lncRNA	KDM4A anti	1	43685123	43708138
ENSG0000012251	0.237338158	0.081	0.16	PMS2	protein_coding	PMS1 homo	7	5970925	6009106

ENSG0000006219	0.102152417	0.081	0.16	GPBP1	protein_coding	GC-rich pro	5	57173948	57264679
ENSG0000016568	0.159642198	0.081	0.16	PMPCA	protein_coding	peptidase, r	9	136410641	136423761
ENSG0000027446	1.102131887	0.081	0.16	AC092119.2	lncRNA	novel transc	16	21950218	21951708
ENSG0000024038	1.22408317	0.081	0.16	EGFL8	protein_coding	EGF like dor	CHR_HSCHR	32231591	32235290
ENSG0000025519	1.5024759	0.082	0.16	SNHG9	lncRNA	small nucleo	16	1964959	1965509
ENSG0000016830	-0.151690632	0.082	0.16	PCMTD1	protein_coding	protein-L-is	8	51817575	51899186
ENSG0000015348	0.471161286	0.082	0.16	TMEM251	protein_coding	transmembr	14	93184951	93188463
ENSG0000026376	1.771970112	0.082	0.16	AC025682.1	lncRNA	novel transc	17	47603860	47649420
ENSG0000024351	-0.812117647	0.082	0.16	AC024940.2	processed_pse	ribosomal p	12	31251992	31252597
ENSG0000027876	-0.550356607	0.082	0.16	BACE1-AS	lncRNA	BACE1 antis	11	117288453	117293571
ENSG0000009001	0.925669699	0.082	0.16	BLVRB	protein_coding	biliverdin re	19	40447765	40465764
ENSG0000024487	-0.110178254	0.082	0.16	GABPB1-AS	lncRNA	GABPB1 ant	15	50354959	50372202
ENSG0000018076	-0.628049892	0.082	0.16	PIPSL	transcribed_pr	PIP5K1A and	10	93958191	93961540
ENSG0000014348	-0.190287314	0.082	0.16	EIF2D	protein_coding	eukaryotic t	1	206571292	206612465
ENSG0000019732	-0.102794331	0.082	0.16	PELI1	protein_coding	pellino E3 u	2	64092652	64144420
ENSG0000008214	0.263400122	0.082	0.16	STRADB	protein_coding	STE20 relate	2	201387858	201480846
ENSG0000012419	-0.352363846	0.082	0.16	TOX2	protein_coding	TOX high m	20	43914852	44069616
ENSG0000023125	0.519139937	0.082	0.16	ATAT1	protein_coding	alpha tubuli	CHR_HSCHR	30705297	30725280
ENSG0000025515	1.177356433	0.082	0.16	MSH5-SAP	protein_coding	MSH5-SAP(	6	31740020	31764851
ENSG0000026568	-0.636000957	0.082	0.16	MAFG-DT	lncRNA	MAFG diver	17	81927829	81930753
ENSG0000017203	0.26979372	0.082	0.16	LAMB2	protein_coding	laminin subu	3	49121114	49133118
ENSG0000027248	-1.225912439	0.082	0.16	NA	NA	NA	NA	NA	NA
ENSG0000018709	-0.430561361	0.083	0.16	MITF	protein_coding	melanocyte	3	69739464	69968336
ENSG0000010655	0.118583056	0.083	0.16	CHCHD3	protein_coding	coiled-coil-	7	132784870	133082090
ENSG0000022418	0.984404016	0.083	0.16	SDHDP6	processed_pse	succinate de	1	25294164	25294643
ENSG0000015411	-1.071908566	0.083	0.16	JPH3	protein_coding	junctophilin	16	87601835	87698156
ENSG0000024113	1.036242338	0.083	0.16	LY6G5B	protein_coding	lymphocyte	CHR_HSCHR	31745397	31749825
ENSG0000016292	0.360140874	0.083	0.16	PUS10	protein_coding	pseudouridi	2	60940222	61018259
ENSG0000026011	0.594943099	0.083	0.16	NA	NA	NA	NA	NA	NA
ENSG0000013787	-0.141217151	0.083	0.16	ZNF280D	protein_coding	zinc finger p	15	56630181	56734086
ENSG0000017116	-0.221213472	0.083	0.16	MORN4	protein_coding	MORN repe	10	97614553	97633500
ENSG0000018548	0.171187841	0.083	0.16	PARPBP	protein_coding	PARP1 bind	12	102120185	102197520
ENSG0000026399	2.382475265	0.083	0.16	AC004253.1	lncRNA	novel transc	17	31873926	31886666
ENSG0000022802	-1.269503366	0.083	0.16	AL158835.1	lncRNA	novel transc	10	125683229	125709677
ENSG0000022248	1.94797335	0.083	0.16	SNORA79B	snoRNA	small nucleo	14	20323179	20323326
ENSG0000017930	0.258773163	0.083	0.16	FAM156B	protein_coding	family with s	X	52891306	52908560
ENSG0000025477	3.883765955	0.083	0.16	AP001893.2	processed_pse	EA domain	11	126179497	126179698
ENSG0000015920	-0.296326481	0.083	0.16	RCAN1	protein_coding	regulator of	21	34513142	34615113
ENSG0000023345	-1.285295922	0.083	0.16	STXBP5-AS	lncRNA	STXBP5 anti	6	146824539	147204614
ENSG0000015417	0.093750043	0.083	0.16	TOMM70	protein_coding	translocase	3	100363431	100401089
ENSG0000024280	0.229705566	0.083	0.16	AP5Z1	protein_coding	adaptor rela	7	4775615	4794397
ENSG0000023351	0.834123828	0.083	0.16	LINC01518	lncRNA	long interge	10	42644445	42691723
ENSG0000013054	0.274373483	0.084	0.16	ZNF557	protein_coding	zinc finger p	19	7069703	7087968
ENSG0000014534	0.154603968	0.084	0.16	TBCK	protein_coding	TBC1 doma	4	106041599	106316683
ENSG0000016523	0.381261452	0.084	0.16	CARD19	protein_coding	caspase recr	9	93096217	93113283
ENSG0000025952	-0.381568838	0.084	0.16	AL136295.5	protein_coding	novel transc	14	24147548	24166452
ENSG0000019883	0.229209715	0.084	0.16	ZNF277	protein_coding	zinc finger p	7	112206695	112343934
ENSG0000012624	-0.551519497	0.084	0.16	PDCD2L	protein_coding	programme	19	34404399	34426168
ENSG0000027456	1.417894008	0.084	0.16	AC080038.1	lncRNA	novel transc	17	62626437	62627590
ENSG0000015168	-0.747822468	0.084	0.16	ANKAR	protein_coding	ankyrin and	2	189674290	189761193
ENSG0000024233	1.992745256	0.084	0.16	INHCAP	transcribed_un	inhibitor of	3	133661998	133754576
ENSG0000013726	-0.531027214	0.084	0.16	LRRC1	protein_coding	leucine rich	6	53794497	53924125
ENSG0000026108	-0.541343526	0.084	0.16	ZNNT1	lncRNA	ZNF706 nei	8	101166805	101169629
ENSG0000024943	-0.432467401	0.084	0.16	NAIP	protein_coding	NLR family a	5	70968166	71025339
ENSG0000000439	-0.16045075	0.084	0.16	PLXND1	protein_coding	plexin D1 [S	3	129555214	129606676
ENSG0000018248	-0.544204717	0.084	0.16	WASH6P	transcribed_un	WASP family	X	156020826	156025710
ENSG0000017984	0.257033763	0.084	0.16	AKAP5	protein_coding	A-kinase an	14	64465499	64474503
ENSG0000022850	0.554601774	0.084	0.16	AL513550.1	lncRNA	novel transc	6	99424922	99432323

ENSG0000010134	0.695723889	0.084	0.16	PAK5	protein_coding	p21 (RAC1)	20	9537389	9839041
ENSG0000012563	-0.155902234	0.084	0.16	CCDC93	protein_coding	coiled-coil c	2	117915478	118014133
ENSG0000014292	1.209416042	0.084	0.16	AZIN2	protein_coding	antizyme inl	1	33081104	33123492
ENSG0000023411	0.496384298	0.084	0.16	CCHCR1	protein_coding	coiled-coil a	CHR_HSCHR	31134989	31150760
ENSG0000013000	-0.269461112	0.084	0.16	GAMT	protein_coding	guanidinoac	19	1397026	1401570
ENSG0000023111	-0.812794194	0.084	0.16	VARS1	protein_coding	valyl-tRNA s	CHR_HSCHR	31844536	31862971
ENSG0000014507	-0.386630846	0.084	0.16	CCDC39	lncRNA	coiled-coil c	3	180614008	180870933
ENSG0000025208	-2.552656672	0.084	0.16	RNU6-277P	snRNA	RNA, U6 sm	11	105974826	105974931
ENSG0000013913	0.338625241	0.084	0.16	ALG10	protein_coding	ALG10 alph	12	34022468	34029694
ENSG0000016924	-0.259003317	0.084	0.16	NPIPB3	protein_coding	nuclear pore	16	21402237	21448567
ENSG0000023434	-0.454250882	0.084	0.16	NEU1	protein_coding	neuraminida	CHR_HSCHR	31839844	31845091
ENSG0000017144	0.230095014	0.084	0.16	ZBTB26	protein_coding	zinc finger a	9	122915566	122931512
ENSG0000002404	0.138995562	0.085	0.16	UBR2	protein_coding	ubiquitin pr	6	42564062	42693504
ENSG0000023067	2.492717771	0.085	0.16	ENO1-AS1	lncRNA	ENO1 antise	1	8878835	8879894
ENSG0000009318	0.183762388	0.085	0.16	SEC22C	protein_coding	SEC22 hom	3	42547969	42601080
ENSG0000027072	0.808547325	0.085	0.16	AL035413.1	processed_pse	synaptonem	1	19297080	19297903
ENSG0000015751	-0.685269817	0.085	0.16	AFAP1L1	protein_coding	actin filamer	5	149271859	149343637
ENSG0000025030	1.265554454	0.085	0.16	LINC02762	lncRNA	long interge	11	112270749	112362534
ENSG0000018536	0.320263043	0.085	0.16	TNFAIP8L1	protein_coding	TNF alpha ir	19	4639516	4655568
ENSG0000008462	0.114998895	0.085	0.16	EIF3I	protein_coding	eukaryotic t	1	32221928	32231604
ENSG0000010417	0.082000816	0.085	0.16	MYEF2	protein_coding	myelin expr	15	48134632	48178353
ENSG0000026114	-1.173315728	0.085	0.16	ADAMTS7P	transcribed_un	ADAMTS7 p	15	77976042	77993057
ENSG0000013810	0.213540766	0.085	0.16	DTNB	protein_coding	dystrobrevir	2	25377198	25673647
ENSG0000018644	0.386815149	0.085	0.16	ZNF501	protein_coding	zinc finger p	3	44729596	44737083
ENSG0000023719	0.212136584	0.085	0.16	CDKN2AIPN	protein_coding	CDKN2A int	5	134402065	134411881
ENSG0000027625	0.188360001	0.085	0.16	AC011043.1	protein_coding	uncharacter	GL000195.1	42939	49164
ENSG0000020525	0.178990413	0.085	0.16	E2F4	protein_coding	E2F transcrip	16	67192155	67198918
ENSG0000012494	0.066764967	0.086	0.16	AHNAK	protein_coding	AHNAK nuc	11	62433542	62556235
ENSG0000026994	2.127537102	0.086	0.16	AC135178.1	lncRNA	novel transc	17	8277763	8278436
ENSG0000011972	0.162445522	0.086	0.16	ZNF410	protein_coding	zinc finger p	14	73886617	73932521
ENSG0000022863	-0.847353058	0.086	0.16	FCF1P2	transcribed_pr	FCF1 pseud	3	48290793	48291375
ENSG0000022855	1.68508812	0.086	0.16	AC004837.1	lncRNA	novel transc	7	39700341	39703296
ENSG0000013673	0.152818961	0.086	0.16	STAM	protein_coding	signal transc	10	17644151	17716824
ENSG0000010332	-0.205318182	0.086	0.16	CAPN15	protein_coding	calpain 15 [	16	527712	554636
ENSG0000016962	0.331142933	0.086	0.16	BOLA2B	protein_coding	bolA family	16	30192932	30194306
ENSG0000025852	-1.671440834	0.086	0.16	AL157871.2	lncRNA	novel transc	14	100339832	100340554
ENSG0000018236	-0.424659063	0.086	0.16	YBEY	protein_coding	ybeY metall	21	46286342	46297751
ENSG0000016355	0.130418365	0.086	0.16	PRKCI	protein_coding	protein kina	3	170222424	170305977
ENSG0000020628	-0.265834258	0.086	0.16	SLC39A7	protein_coding	solute carrie	CHR_HSCHR	33129256	33133250
ENSG0000022942	1.174518422	0.086	0.16	AL512625.2	lncRNA	novel transc	9	62837139	62838302
ENSG0000017576	-0.407506492	0.086	0.17	TTLL11	protein_coding	tubulin tyros	9	121821928	122093606
ENSG0000026822	1.530084531	0.087	0.17	NA	NA	NA	NA	NA	NA
ENSG0000025637	-2.141582219	0.087	0.17	AC009509.2	lncRNA	novel transc	12	27696388	27710803
ENSG0000018438	0.324404317	0.087	0.17	PLA2G6	protein_coding	phospholipa	22	38111495	38214778
ENSG0000008309	-0.219135573	0.087	0.17	PALB2	protein_coding	partner and	16	23603160	23641310
ENSG0000024050	-0.919096208	0.087	0.17	RPL34P18	processed_pse	ribosomal p	8	93957338	93957691
ENSG0000015157	0.173403802	0.087	0.17	QTRT2	protein_coding	queuine tRN	3	114005833	114088422
ENSG0000023797	2.647540155	0.087	0.17	DNAJB12P1	processed_pse	DNAJB12 ps	2	65500993	65502138
ENSG0000022722	0.675245378	0.087	0.17	DHX16	protein_coding	DEAH-box I	CHR_HSCHR	30723385	30736950
ENSG0000010049	-1.127175619	0.087	0.17	CDKL1	protein_coding	cyclin deper	14	50329404	50416461
ENSG0000025821	1.195753494	0.087	0.17	AC144548.1	lncRNA	novel transc	12	110387463	110445548
ENSG0000026984	-1.386558078	0.087	0.17	AC008537.2	lncRNA	novel transc	19	40831221	40837210
ENSG0000018388	-0.675037565	0.087	0.17	AC138969.1	protein_coding	novel memb	16	16317444	16350590
ENSG0000027421	4.698048497	0.087	0.17	RNVU1-27	snRNA	RNA, varian	1	148522601	148522765
ENSG0000027338	1.16859471	0.087	0.17	AL356488.3	lncRNA	novel transc	1	109087971	109090858
ENSG0000013597	0.146049336	0.088	0.17	C2orf49	protein_coding	chromosom	2	105337532	105349211
ENSG0000016477	0.906356631	0.088	0.17	PHKG1	protein_coding	phosphoryla	7	56080283	56092996
ENSG0000009140	-0.412887318	0.088	0.17	ITGA6	protein_coding	integrin sub	2	172427354	172506459

ENSG0000014458	-0.2127391	0.088	0.17	MARCHF4	protein_coding	membrane a	2	216257865	216372483
ENSG0000027589	-0.408475239	0.088	0.17	U2AF1L5	protein_coding	U2 small nu	21	6484623	6499261
ENSG0000016912	0.474754876	0.088	0.17	FAM110B	protein_coding	family with s	8	57994509	58204279
ENSG0000013247	-0.146261457	0.088	0.17	WBP2	protein_coding	WW domair	17	75845699	75856507
ENSG0000015706	0.093059206	0.088	0.17	NMNAT2	protein_coding	nicotinamid	1	183248237	183418380
ENSG0000019786	0.149512075	0.088	0.17	SGTB	protein_coding	small glutan	5	65665928	65723035
ENSG0000013389	0.157862958	0.088	0.17	MEN1	protein_coding	menin 1 [So	11	64803510	64811294
ENSG0000019631	-0.132546539	0.088	0.17	POM121	protein_coding	POM121 tra	7	72879365	72951440
ENSG0000023391	-2.910773697	0.088	0.17	AC026202.2	lncRNA	novel transc	3	5156905	5187329
ENSG0000006460	0.141452586	0.088	0.17	CTSA	protein_coding	cathepsin A	20	45890144	45898820
ENSG0000008034	-0.087914116	0.089	0.17	RIF1	protein_coding	replication t	2	151409883	151508013
ENSG0000017440	0.201735513	0.089	0.17	LIG4	protein_coding	DNA ligase	13	108207439	108218368
ENSG0000014379	0.225019705	0.089	0.17	C1orf35	protein_coding	chromosom	1	228100726	228105411
ENSG0000018448	-0.37193151	0.089	0.17	PTP4A3	protein_coding	protein tyro	8	141391995	141432454
ENSG0000018362	0.703003945	0.089	0.17	DGCR6	protein_coding	DiGeorge sy	22	18906028	18914238
ENSG0000023297	-1.623077151	0.089	0.17	CYP1B1-AS	lncRNA	CYP1B1 ant	2	38073447	38231651
ENSG0000002360	-0.213456252	0.089	0.17	SNAPC1	protein_coding	small nuclea	14	61762420	61796428
ENSG0000015280	-0.646758681	0.089	0.17	HHEX	protein_coding	hematopoie	10	92689955	92695647
ENSG0000016901	0.099763844	0.089	0.17	FEM1B	protein_coding	fem-1 hom	15	68277745	68295862
ENSG0000014963	-1.040948945	0.089	0.17	SPATA25	protein_coding	spermatoge	20	45886489	45887635
ENSG0000027924	0.224869944	0.089	0.17	AK6	protein_coding	adenylate ki	CHR_HSCHR	69350995	69370027
ENSG0000017356	0.770784658	0.089	0.17	ADGRF3	protein_coding	adhesion G	2	26308173	26346817
ENSG0000011658	-0.111691123	0.089	0.17	GON4L	protein_coding	gon-4 like [	1	155749662	155859400
ENSG0000013544	-0.351188203	0.089	0.17	BLOC1S1	protein_coding	biogenesis c	12	55716037	55720087
ENSG0000019868	0.327531775	0.089	0.17	TUSC1	protein_coding	tumor supp	9	25676389	25678440
ENSG0000016154	-0.158008237	0.089	0.17	PRPSAP1	protein_coding	phosphoribo	17	76309478	76384521
ENSG0000017388	-0.140277733	0.089	0.17	PHC3	protein_coding	polyhomeot	3	170086732	170181749
ENSG0000026124	3.180384118	0.089	0.17	AC009065.0	lncRNA	novel transc	16	2112335	2113342
ENSG0000016892	-0.163800287	0.089	0.17	LETM1	protein_coding	leucine zipp	4	1811479	1856156
ENSG0000027184	2.363543475	0.089	0.17	AC073389.2	lncRNA	novel transc	10	73653980	73675450
ENSG0000011954	0.155334337	0.089	0.17	VPS4B	protein_coding	vacuolar pro	18	63389190	63422483
ENSG0000025966	0.768634463	0.09	0.17	AC066613.1	lncRNA	novel transc	15	51455268	51460582
ENSG0000016715	-0.364357398	0.09	0.17	PRRX2	protein_coding	paired relat	9	129665647	129722674
ENSG0000025429	-0.855317154	0.09	0.17	AC026688.2	lncRNA	novel transc	5	154483917	154486150
ENSG0000017518	0.245701841	0.09	0.17	FAM131A	protein_coding	family with s	3	184335926	184348421
ENSG0000012752	0.148466918	0.09	0.17	EPS15L1	protein_coding	epidermal g	19	16355239	16472085
ENSG0000020371	1.324944516	0.09	0.17	C6orf99	lncRNA	chromosom	6	158869848	158919105
ENSG0000027629	-1.401957396	0.09	0.17	TFPT	protein_coding	TCF3 fusion	CHR_HSCHR	54107066	54115801
ENSG0000022866	1.473371364	0.09	0.17	AC090587.1	lncRNA	novel transc	11	3854318	3855509
ENSG0000027181	0.552551615	0.09	0.17	BMS1P4	lncRNA	BMS1 pseu	10	73699151	73730487
ENSG0000010521	-1.776152054	0.09	0.17	CCNP	protein_coding	cyclin P [Sou	19	40222208	40226697
ENSG0000020628	0.469022457	0.09	0.17	VPS52	protein_coding	VPS52 subu	CHR_HSCHR	33179079	33200871
ENSG0000020756	-3.101280226	0.091	0.17	MIR23B	miRNA	microRNA 2	9	95085208	95085304
ENSG0000027594	0.457288128	0.091	0.17	TMEM251	protein_coding	transmembr	CHR_HSCHR	93184951	93187089
ENSG0000012320	0.106486767	0.091	0.17	ZC3H13	protein_coding	zinc finger C	13	45954465	46052759
ENSG0000020535	0.217286735	0.091	0.17	TECPR1	protein_coding	tectonin bet	7	98214624	98252232
ENSG0000022889	-0.413975104	0.091	0.17	HCG18	lncRNA	HLA comple	CHR_HSCHR	30280607	30316946
ENSG0000026602	0.858496716	0.091	0.17	MSH5-SAP	protein_coding	MSH5-SAP(	CHR_HSCHR	31722193	31747032
ENSG0000022579	1.624722438	0.091	0.17	TMEM30A-	lncRNA	TMEM30A c	6	75285014	75317525
ENSG0000012512	1.428871815	0.091	0.17	LRRC29	protein_coding	leucine rich	16	67207139	67227048
ENSG0000021317	0.388589312	0.091	0.17	LINGO4	protein_coding	leucine rich	1	151800264	151805419
ENSG0000011183	-1.095828912	0.091	0.17	RSPH4A	protein_coding	radial spoke	6	116616479	116632985
ENSG0000018532	0.211276627	0.091	0.17	CDK10	protein_coding	cyclin deper	16	89680737	89696354
ENSG0000016581	-0.174491482	0.091	0.17	CCDC186	protein_coding	coiled-coil c	10	114120862	114174232
ENSG0000024853	-1.560418515	0.091	0.17	AC022784.1	lncRNA	novel transc	8	9151695	9425524
ENSG0000012250	-0.340844811	0.092	0.17	BBS9	protein_coding	Bardet-Biec	7	33109557	33877180
ENSG0000016626	0.098459063	0.092	0.17	CUL5	protein_coding	cullin 5 [Sou	11	108008898	108107761
ENSG0000015161	-0.187087923	0.092	0.17	ZNF827	protein_coding	zinc finger p	4	145757627	145938823

ENSG0000017200	0.141756178	0.092	0.17	THOP1	protein_coding	thimet oligo	19	2785503	2815807
ENSG0000014626	0.173983081	0.092	0.17	MMS22L	protein_coding	MMS22 like	6	97142161	97283217
ENSG0000011119	0.206165909	0.092	0.17	MAGOHB	protein_coding	mago homo	12	10604193	10613609
ENSG0000027477	-0.116707499	0.092	0.17	NOMO1	protein_coding	NODAL mo	CHR_HSCHR	15317897	15380303
ENSG0000011069	-1.011305327	0.092	0.17	SOX6	protein_coding	SRY-box tra	11	15966449	16739591
ENSG0000009107	-0.656630441	0.092	0.17	DTX2	protein_coding	deltex E3 ub	7	76461676	76505995
ENSG0000017559	0.194318515	0.092	0.17	ERCC4	protein_coding	ERCC excisio	16	13920154	13952348
ENSG0000018873	0.189793228	0.092	0.17	TMEM120B	protein_coding	transmembr	12	121712752	121783001
ENSG0000011412	0.122605816	0.092	0.17	XRN1	protein_coding	5'-3' exoribo	3	142306607	142448062
ENSG0000014203	-0.219285534	0.092	0.17	CCDC97	protein_coding	coiled-coil c	19	41310172	41324873
ENSG0000018362	0.171966658	0.092	0.17	HMCES	protein_coding	5-hydroxym	3	129278828	129306186
ENSG0000022514	-1.45355185	0.093	0.18	AL358216.1	lncRNA	novel transc	10	628638	631255
ENSG0000017639	0.299016574	0.093	0.18	EID2	protein_coding	EP300 intera	19	39538707	39540161
ENSG0000023681	0.993867838	0.093	0.18	ELOA-AS1	lncRNA	ELOA antise	1	23706901	23778296
ENSG0000024389	1.200409435	0.093	0.18	EGFL8	protein_coding	EGF like dor	CHR_HSCHR	32203996	32207669
ENSG0000019818	-0.298520129	0.093	0.18	ZNF607	protein_coding	zinc finger p	19	37696371	37719761
ENSG0000015799	1.462233618	0.093	0.18	KRTCAP3	protein_coding	keratinocyte	2	27442366	27446481
ENSG0000011514	0.177507991	0.093	0.18	STAM2	protein_coding	signal transc	2	152116801	152175763
ENSG0000011715	-1.157640383	0.093	0.18	IGSF21	protein_coding	immunoglob	1	18107798	18378483
ENSG0000014375	0.111045774	0.093	0.18	DEGS1	protein_coding	delta 4-desa	1	224175756	224193441
ENSG0000016502	-0.969149132	0.093	0.18	NIPSNAP3B	protein_coding	nipsnap hor	9	104764129	104777764
ENSG0000026536	4.659315142	0.094	0.18	GLUD1P2	transcribed_un	glutamate c	10	46759019	46786770
ENSG0000026835	0.703714278	0.094	0.18	VN1R81P	unprocessed_p	vomeronasa	19	21123817	21124188
ENSG0000027575	1.080393713	0.094	0.18	5_8S_rRNA	rRNA	5.8S ribosor	KI270733.1	173956	174108
ENSG0000016682	-0.577640628	0.094	0.18	PEX11A	protein_coding	peroxisoma	15	89677764	89690783
ENSG0000015943	0.172455875	0.094	0.18	STARD9	protein_coding	StAR relatec	15	42575606	42720998
ENSG0000022953	1.838574357	0.094	0.18	PANK2-AS1	lncRNA	PANK2 antis	20	3888239	3888868
ENSG0000021503	0.182237795	0.094	0.18	RPL13P12	processed_pse	ribosomal p	17	17383377	17384012
ENSG0000025589	0.8187599	0.094	0.18	AP000786.1	lncRNA	novel transc	11	94472908	94473570
ENSG0000021427	-1.238458634	0.094	0.18	ANG	protein_coding	angiogenin	14	20684177	20698971
ENSG0000010847	0.475546304	0.094	0.18	PIGL	protein_coding	phosphatidy	17	16217191	16351797
ENSG0000011285	0.846695714	0.094	0.18	PCDHB2	protein_coding	protocadhe	5	141094606	141098703
ENSG0000021322	0.832084172	0.094	0.18	TOMM40P4	processed_pse	TOMM40 ps	2	131723253	131724279
ENSG0000023494	-0.709087311	0.094	0.18	STK19	protein_coding	serine/threo	CHR_HSCHR	31958721	31968992
ENSG0000010005	-0.214841354	0.094	0.18	ESS2	protein_coding	ess-2 splicir	22	19130279	19144684
ENSG0000015304	-0.27443474	0.094	0.18	CDYL	protein_coding	chromodom	6	4706159	4955551
ENSG0000019808	0.261710956	0.094	0.18	ZBTB14	protein_coding	zinc finger a	18	5289019	5297053
ENSG0000011285	0.167066103	0.094	0.18	HARS2	protein_coding	histidyl-tRN	5	140691430	140699305
ENSG0000007431	0.126416687	0.094	0.18	TSG101	protein_coding	tumor susce	11	18468336	18526951
ENSG0000011970	-0.765233406	0.095	0.18	ZC2HC1C	protein_coding	zinc finger C	14	75064109	75119502
ENSG0000023941	-1.122773741	0.095	0.18	AP001469.3	lncRNA	novel transc	21	46251549	46254133
ENSG0000016711	-0.20562492	0.095	0.18	TRUB2	protein_coding	TruB pseudo	9	128305159	128322447
ENSG0000010351	-0.167682347	0.095	0.18	KAT8	protein_coding	lysine acetyl	16	31114489	31131393
ENSG0000009202	-0.179394261	0.095	0.18	PPP2R3C	protein_coding	protein pho	14	35085467	35122517
ENSG0000015508	-0.364044346	0.095	0.18	AK9	protein_coding	adenylate ki	6	109492856	109691217
ENSG0000011289	0.185229281	0.095	0.18	MAN2A1	protein_coding	mannosidas	5	109689366	109869625
ENSG0000023590	1.288603021	0.095	0.18	CPB2-AS1	lncRNA	CPB2 antise	13	46052497	46161379
ENSG0000011006	0.268479534	0.095	0.18	DCPS	protein_coding	decapping e	11	126304060	126350005
ENSG0000014440	-0.507366675	0.095	0.18	PTH2R	protein_coding	parathyroid	2	208359714	208854503
ENSG0000023283	-0.611298606	0.095	0.18	TRIM39	protein_coding	tripartite mc	CHR_HSCHR	30316726	30333982
ENSG0000026163	-1.986446744	0.095	0.18	AC092378.1	lncRNA	novel transc	16	58847715	58880351
ENSG0000027138	0.260369106	0.095	0.18	NBPF19	protein_coding	NBPF memb	1	149475338	149556361
ENSG0000015765	-1.123061775	0.095	0.18	C9orf43	protein_coding	chromosom	9	113410054	113429690
ENSG0000017225	0.974225729	0.095	0.18	SERHL	transcribed_un	serine hydro	22	42500568	42512560
ENSG0000017085	0.224834892	0.095	0.18	TRIAP1	protein_coding	TP53 regula	12	120443964	120446384
ENSG0000001779	0.154452694	0.095	0.18	RALBP1	protein_coding	ralA binding	18	9475009	9538114
ENSG0000024762	0.677792464	0.095	0.18	MTND4P12	processed_pse	MT-ND4 ps	5	134926660	134928036
ENSG0000017317	-0.219494208	0.095	0.18	MTX1	protein_coding	metaxin 1 [S	1	155208699	155213824

ENSG0000014716	0.078293025	0.096	0.18	OGT	protein_codinç	O-linked N-	X	71533104	71575892
ENSG0000025835	-2.115824402	0.096	0.18	PCNPP1	processed_pse	PEST contai	12	111669852	111670362
ENSG0000013728	0.181673943	0.096	0.18	UQCC2	protein_codinç	ubiquinol-c	6	33694293	33711727
ENSG0000023464	-0.927679911	0.096	0.18	AL162151.2	processed_pse	pseudogene	14	98973314	98973471
ENSG0000010436	0.411528135	0.096	0.18	JPH1	protein_codinç	junctophilin	8	74234700	74321540
ENSG0000013663	-0.097322986	0.096	0.18	KCTD3	protein_codinç	potassium c	1	215567304	215621807
ENSG0000020416	-0.241363725	0.096	0.18	ZDHHC18	protein_codinç	zinc finger D	1	26826688	26857604
ENSG0000027187	-1.768131701	0.096	0.18	AC005740.4	lncRNA	novel transc	5	141952419	141953375
ENSG0000016828	0.101610358	0.097	0.18	KIF5C	protein_codinç	kinesin fami	2	148875227	149026759
ENSG0000027469	1.527026039	0.097	0.18	AC099521.2	lncRNA	novel transc	16	68450283	68452318
ENSG0000016922	-0.685678332	0.097	0.18	RGS14	protein_codinç	regulator of	5	177357924	177372596
ENSG0000017393	0.091050814	0.097	0.18	RBM4	protein_codinç	RNA bindinç	11	66638667	66668374
ENSG0000026193	-1.070594181	0.097	0.18	PCDHGA9	protein_codinç	protocadhe	5	141402932	141512979
ENSG0000016950	0.078766577	0.097	0.18	CLIC4	protein_codinç	chloride intr	1	24745382	24844321
ENSG0000014453	-0.210973571	0.097	0.18	DIS3L2	protein_codinç	DIS3 like 3'-	2	231961245	232344350
ENSG0000023342	-0.314250482	0.097	0.18	EIF3FP3	processed_pse	eukaryotic t	2	58251440	58252525
ENSG0000011087	0.175386927	0.097	0.18	COQ5	protein_codinç	coenzyme C	12	120503279	120534434
ENSG0000014635	-0.31457384	0.097	0.18	TBC1D32	protein_codinç	TBC1 doma	6	121079494	121334745
ENSG0000027448	-0.506447061	0.097	0.18	NPEPPSP1	transcribed_un	NPEPPS pse	17	38195703	38257192
ENSG0000024571	1.68373473	0.097	0.18	NADK2-AS	lncRNA	NADK2 anti	5	36221055	36221902
ENSG0000017820	-0.963186346	0.097	0.18	VN1R1	protein_codinç	vomeronasa	19	57454790	57457142
ENSG0000020648	-0.479318972	0.097	0.18	DHX16	protein_codinç	DEAH-box I	CHR_HSCHR	30642608	30662557
ENSG0000011750	-0.136718805	0.097	0.18	TMED5	protein_codinç	transmembr	1	93149742	93180516
ENSG0000018800	-1.166365894	0.097	0.18	TPRG1	protein_codinç	tumor prote	3	188947214	189325304
ENSG0000017938	0.552923613	0.098	0.18	EGR3	protein_codinç	early growtl	8	22687659	22693480
ENSG0000023045	-0.374415991	0.098	0.18	DDR1	protein_codinç	discoidin dc	CHR_HSCHR	30871677	30890873
ENSG0000011251	-0.299830015	0.098	0.18	PHF1	protein_codinç	PHD finger	6	33410399	33416453
ENSG0000021586	-0.728194662	0.098	0.18	LINC01356	lncRNA	long interge	1	112820170	112850643
ENSG0000015995	1.734826752	0.098	0.18	TNFRSF13C	protein_codinç	TNF recepto	22	41922032	41926806
ENSG0000016998	-1.283101582	0.098	0.18	TIGD4	protein_codinç	tigger trans	4	152769354	152779730
ENSG0000013184	0.314596978	0.098	0.18	ZNF304	protein_codinç	zinc finger p	19	57351271	57359898
ENSG0000018372	0.51697761	0.098	0.18	LHFPL6	protein_codinç	LHFPL tetra	13	39209116	39603528
ENSG0000020483	-1.142371865	0.098	0.18	FGF7P3	transcribed_un	fibroblast gı	9	39816542	40106661
ENSG0000013576	-0.138427245	0.098	0.18	EGLN1	protein_codinç	egl-9 family	1	231363751	231422287
ENSG0000023146	-0.682243497	0.098	0.18	AL022324.2	processed_pse	PHD finger	22	25349543	25350322
ENSG0000025873	-0.769843344	0.098	0.18	AC025884.1	unprocessed_p	arginine-glu	15	22278971	22282872
ENSG0000017034	0.15809156	0.098	0.18	B3GNT2	protein_codinç	UDP-GlcNA	2	62196115	62224731
ENSG0000026860	1.401323919	0.098	0.18	AC053503.4	lncRNA	novel transc	2	219497611	219498246
ENSG0000024131	-1.468752415	0.098	0.18	SUCLG2-AS	lncRNA	SUCLG2 ant	3	67654669	67947713
ENSG0000018277	0.317489689	0.098	0.18	GRID1	protein_codinç	glutamate io	10	85599552	86366795
ENSG0000027653	-2.637883753	0.098	0.18	AC092666.1	unprocessed_p	dipeptidyl-p	7	150047609	150047854
ENSG0000013719	-0.822660183	0.099	0.18	GMPR	protein_codinç	guanosine r	6	16238587	16295549
ENSG0000026720	-2.271454606	0.099	0.18	LINC01775	lncRNA	long interge	19	2458935	2462185
ENSG0000026931	-1.278808655	0.099	0.18	MAGIX	protein_codinç	MAGI family	X	49162564	49168483
ENSG0000023244	0.845505588	0.099	0.18	MHENCR	lncRNA	melanoma I	20	63627227	63628824
ENSG0000024265	-3.184422262	0.099	0.18	AC108693.2	lncRNA	novel transc	3	113746872	113747408
ENSG0000015988	-0.486089267	0.099	0.18	ZNF230	protein_codinç	zinc finger p	19	44002957	44013924
ENSG0000025438	1.338419628	0.099	0.18	RHPN1-AS	lncRNA	RHPN1 anti	8	143366631	143368548
ENSG0000023026	-1.170812675	0.099	0.18	LINC02525	lncRNA	long interge	6	3182626	3195784
ENSG0000025518	0.475280425	0.099	0.18	AC084125.2	lncRNA	novel transc	8	144495458	144505444
ENSG0000014150	0.194646848	0.099	0.18	MINK1	protein_codinç	misshapen I	17	4833340	4898061
ENSG0000012865	-0.348965018	0.099	0.18	HOXD3	protein_codinç	homeobox I	2	176136612	176173102
ENSG0000027343	1.557226689	0.099	0.18	AC004951.4	lncRNA	novel transc	7	43951910	44019151
ENSG0000014112	0.171979694	0.099	0.19	PRPSAP2	protein_codinç	phosphoribo	17	18840085	18931287
ENSG0000025728	2.803057072	0.099	0.19	AL132780.1	lncRNA	novel transc	14	22929607	22956374
ENSG0000015687	0.127100712	0.099	0.19	MFSD14A	protein_codinç	major facilit	1	100038095	100083377
ENSG0000014677	-0.305531358	0.099	0.19	ATXN7L1	protein_codinç	ataxin 7 like	7	105605067	105876604
ENSG0000015990	-0.620259324	0.099	0.19	ZNF221	protein_codinç	zinc finger p	19	43951223	43967709

ENSG0000010911	-0.107721478	0.1	0.19	SUPT6H	protein_coding	SPT6 homol	17	28662198	28702679
ENSG0000011724	0.227516993	0.099	0.19	PINK1-AS	lncRNA	PINK1 antis	1	20642657	20652193
ENSG0000023477	0.862870938	0.099	0.19	AC012618.3	transcribed_un	novel zinc fi	19	12195015	12237767
ENSG0000027431	-1.4984783	0.1	0.19	AC009318.3	lncRNA	novel transc	12	29331434	29331936
ENSG0000016678	0.200572383	0.1	0.19	BMERB1	protein_coding	bMERB dom	16	15434475	15625028
ENSG0000015896	0.152781786	0.1	0.19	CACHD1	protein_coding	cache doma	1	64470129	64693058
ENSG0000013319	-0.164603398	0.1	0.19	FAM104A	protein_coding	family with s	17	73207353	73236753
ENSG0000025481	2.436016687	0.1	0.19	AC067930.2	lncRNA	novel transc	8	143632071	143633756
ENSG0000010104	1.348477038	0.1	0.19	SGK2	protein_coding	serum/gluco	20	43558968	43588237
ENSG0000000621	-0.253216208	0.1	0.19	CX3CL1	protein_coding	C-X3-C mo	16	57372477	57385044
ENSG0000025997	-0.40115027	0.1	0.19	AC009120.2	lncRNA	novel transc	16	74305127	74335346
ENSG0000012652	-0.130778868	0.1	0.19	SBDS	protein_coding	SBDS ribosc	7	66987680	66995587
ENSG0000025949	0.259985245	0.1	0.19	AC016705.2	lncRNA	novel transc	15	80344853	80404214
ENSG0000012799	0.219238683	0.1	0.19	RBM48	protein_coding	RNA binding	7	92528773	92540481
ENSG0000014608	0.160386792	0.1	0.19	MMUT	protein_coding	methylmalo	6	49430360	49463253
ENSG0000027570	1.341110613	0.101	0.19	MIR3648-1	miRNA	microRNA 3	21	8208473	8208652
ENSG0000013282	-0.798769515	0.101	0.19	VSTM2L	protein_coding	V-set and tr	20	37903111	37945350
ENSG0000010528	0.283700568	0.101	0.19	PRKD2	protein_coding	protein kina	19	46674275	46717127
ENSG0000021502	0.717875494	0.101	0.19	AL008729.1	lncRNA	novel transc	6	13264861	13295586
ENSG0000017181	0.639804535	0.101	0.19	ZNF540	protein_coding	zinc finger p	19	37551406	37614179
ENSG0000017326	-1.346839767	0.101	0.19	PLAC8L1	protein_coding	PLAC8 like 1	5	146084313	146105577
ENSG0000025551	0.909329143	0.101	0.19	AP002748.4	lncRNA	novel transc	11	66473490	66480233
ENSG0000027484	0.548916299	0.101	0.19	MAFIP	protein_coding	MAFF intera	GL000194.1	53594	115055
ENSG0000024712	0.74820267	0.101	0.19	AC009126.1	lncRNA	novel transc	5	96814028	96935809
ENSG0000012731	-0.474623123	0.101	0.19	HELB	protein_coding	DNA helicas	12	66302493	66343643
ENSG0000013302	-0.179914806	0.101	0.19	SCO1	protein_coding	synthesis of	17	10672474	10698375
ENSG0000017087	0.150960264	0.101	0.19	TMEM43	protein_coding	transmembr	3	14125015	14143680
ENSG0000016902	0.136921018	0.101	0.19	UQCRFS1	protein_coding	ubiquinol-cy	19	29205320	29213151
ENSG0000010585	-0.321042789	0.101	0.19	PON2	protein_coding	paraoxonas	7	95404862	95435329
ENSG0000016859	-0.177404652	0.101	0.19	TMUB2	protein_coding	transmembr	17	44186970	44191929
ENSG0000022933	1.092084447	0.102	0.19	AC046143.1	lncRNA	novel transc	3	194632923	194645401
ENSG0000017477	-0.386481288	0.102	0.19	HRAS	protein_coding	HRas proto-	11	532242	537287
ENSG0000018033	-0.787809416	0.102	0.19	MEIOC	protein_coding	meiosis spe	17	44656404	44690308
ENSG0000018406	0.4175198	0.102	0.19	SREBF2-AS	lncRNA	SREBF2 anti	22	41831215	41834665
ENSG0000016763	-0.391046192	0.102	0.19	ZNF283	protein_coding	zinc finger p	19	43827321	43852017
ENSG0000006301	0.461888531	0.102	0.19	SEZ6	protein_coding	seizure relat	17	28954901	29006440
ENSG0000022747	-0.868758002	0.102	0.19	TSSK5P	unitary_pseudo	testis specifi	8	144141214	144143664
ENSG0000010859	-0.258188766	0.102	0.19	DRG2	protein_coding	developmer	17	18087892	18107970
ENSG0000015454	-0.185936681	0.102	0.19	MAGED4	protein_coding	MAGE famil	X	52184823	52192268
ENSG0000013740	0.737409846	0.102	0.19	NRM	protein_coding	nurim [Sour	6	30688047	30691420
ENSG0000018737	-0.601609521	0.102	0.19	PCDHB13	protein_coding	protocadhe	5	141213919	141218979
ENSG0000010048	0.13036229	0.102	0.19	SOS2	protein_coding	SOS Ras/Rh	14	50117130	50231578
ENSG0000027574	-0.448833857	0.102	0.19	SLC2A11	protein_coding	solute carrie	CHR_HSCHR	23856703	23886309
ENSG0000022852	-3.75493743	0.102	0.19	AC016396.1	lncRNA	novel transc	10	58304553	58305621
ENSG0000010060	-0.108706172	0.102	0.19	SNW1	protein_coding	SNW domai	14	77717599	77761207
ENSG0000012888	-1.090232592	0.102	0.19	ELL3	protein_coding	elongation f	15	43772605	43777315
ENSG0000008381	0.581970058	0.102	0.19	ZNF671	protein_coding	zinc finger p	19	57719751	57727624
ENSG0000021514	0.308302258	0.102	0.19	BX322639.1	transcribed_un	zinc finger p	10	42331866	42367974
ENSG0000002543	0.469700999	0.102	0.19	NR1H3	protein_coding	nuclear rece	11	47248300	47269033
ENSG0000027517	-0.113480533	0.102	0.19	ACACA	protein_coding	acetyl-CoA	CHR_HSCHR	37086456	37411442
ENSG0000016679	0.834598252	0.103	0.19	YPEL4	protein_coding	yippee like 4	11	57645087	57649944
ENSG0000025947	-0.405112322	0.103	0.19	SORD2P	transcribed_un	sorbitol deh	15	44825747	44884694
ENSG0000027883	-0.378663051	0.103	0.19	SERF1B	protein_coding	small EDRK-	CHR_HSCHR	69709074	69726961
ENSG0000013919	-0.560622407	0.103	0.19	TAPBPL	protein_coding	TAP binding	12	6451690	6466517
ENSG0000012454	0.606627129	0.103	0.19	BTN2A3P	transcribed_un	butyrophilin	6	26421391	26432383
ENSG0000022484	-2.379493573	0.103	0.19	AL589843.1	lncRNA	novel transc	9	96687050	96774318
ENSG0000025994	-1.101818294	0.103	0.19	AC109449.1	lncRNA	novel transc	16	27213308	27214993
ENSG0000016167	0.435033441	0.103	0.19	JOSD2	protein_coding	Josephin do	19	50505998	50511220

ENSG0000022680	-1.085891169	0.103	0.19	ZNF451-AS	lncRNA	ZNF451 reg	6	57114894	57174236
ENSG0000017998	-0.420622316	0.103	0.19	PSTK	protein_coding	phosphoser	10	122954381	122997513
ENSG0000011690	0.114823639	0.103	0.19	GNPAT	protein_coding	glyceroneph	1	231241207	231277973
ENSG0000024087	2.690798216	0.104	0.19	RN7SL521P	misc_RNA	RNA, 7SL, cy	7	149125690	149125986
ENSG0000016238	0.171018284	0.104	0.19	MAGOH	protein_coding	mago homo	1	53226900	53238518
ENSG0000019663	-0.256242367	0.104	0.19	WNK3	protein_coding	WNK lysine	X	54192823	54358642
ENSG0000027924	1.184472783	0.104	0.19	AP003108.3	TEC	novel transc	11	61352244	61354477
ENSG0000025056	0.257882699	0.104	0.19	ATP6V1E2	protein_coding	ATPase H+	2	46490750	46542577
ENSG0000027216	0.367458658	0.104	0.19	AF106564.1	lncRNA	novel transc	8	24912165	24914717
ENSG0000018296	-0.104273411	0.104	0.19	GJC1	protein_coding	gap junction	17	44798448	44830816
ENSG0000021741	1.040221031	0.104	0.19	ISCA1P1	processed_pse	iron-sulfur c	5	62776877	62777263
ENSG0000026076	0.420665596	0.104	0.19	NA	NA	NA	NA	NA	NA
ENSG0000020718	-1.671465318	0.104	0.19	SNORA14B	snoRNA	small nucleo	1	235127803	235127937
ENSG0000013459	-0.199687308	0.104	0.19	RBMX2	protein_coding	RNA binding	X	130401987	130413656
ENSG0000024617	0.521609359	0.104	0.19	KCTD21-AS	lncRNA	KCTD21 ant	11	78139771	78175323
ENSG0000027212	0.959740628	0.104	0.19	AL359715.3	lncRNA	novel transc	6	80355424	80356859
ENSG0000017271	0.202029191	0.104	0.19	SLFN11	protein_coding	schlafen fan	17	35350305	35373701
ENSG0000027434	0.431603261	0.104	0.19	ZNF658	protein_coding	zinc finger p	9	66856426	66932141
ENSG0000023816	-2.295577682	0.105	0.19	AC022748.1	transcribed_un	TBC1 doma	15	78752107	78753373
ENSG0000012145	0.414254002	0.105	0.19	LHX4	protein_coding	LIM homeol	1	180230264	180278984
ENSG0000011692	-0.152147938	0.105	0.19	C1orf109	protein_coding	chromosom	1	37681570	37692249
ENSG0000011504	-1.239081749	0.105	0.19	KCNIP3	protein_coding	potassium v	2	95297327	95386077
ENSG0000016454	-0.25701104	0.105	0.19	KIAA0895	protein_coding	KIAA0895 [S	7	36324152	36390125
ENSG0000017814	1.295226989	0.105	0.19	AL672207.1	processed_pse	glycerol kin	X	101778969	101780538
ENSG0000013616	-0.4429356	0.105	0.19	LCP1	protein_coding	lymphocyte	13	46125920	46211871
ENSG0000027217	1.598131084	0.105	0.19	AC138696.2	lncRNA	novel transc	8	143290399	143290621
ENSG0000025972	-0.876684988	0.105	0.19	CSPG4P11	transcribed_un	chondroitin	15	84186752	84197384
ENSG0000022463	-0.808713412	0.105	0.19	AL391095.1	lncRNA	novel transc	20	38404893	38416797
ENSG0000024959	-0.496670776	0.105	0.19	AC139887.2	lncRNA	novel transc	4	760202	781859
ENSG0000027914	-1.145344803	0.105	0.19	AC063926.2	TEC	novel transc	12	130465008	130466315
ENSG0000014992	-1.079733494	0.105	0.19	TBX6	protein_coding	T-box trans	16	30085793	30091924
ENSG0000027319	-0.896456308	0.105	0.19	AL671710.1	lncRNA	novel transc	22	49902228	49904576
ENSG0000025862	-3.74544375	0.106	0.2	COX7A2P1	processed_pse	cytochrome	14	67652300	67652614
ENSG0000014685	-0.247715455	0.106	0.2	TMEM140	protein_coding	transmembr	7	135148072	135166215
ENSG0000010976	-0.160610056	0.106	0.2	SNX25	protein_coding	sorting nexi	4	185204237	185370185
ENSG0000008875	-0.246560146	0.106	0.2	ARHGAP28	protein_coding	Rho GTPase	18	6729716	6915716
ENSG0000018868	-0.458280616	0.106	0.2	TEKT4P2	transcribed_un	tektin 4 pse	21	9068361	9129752
ENSG0000026793	-3.011684767	0.106	0.2	EIF1P6	unprocessed_p	eukaryotic t	19	4347780	4349061
ENSG0000026136	1.322481861	0.106	0.2	MANEA-DT	lncRNA	MANEA dive	6	95575183	95577450
ENSG0000026204	0.360854478	0.106	0.2	AC139530.1	lncRNA	novel transc	17	81701324	81703300
ENSG0000019758	-0.200247192	0.106	0.2	ENTPD6	protein_coding	ectonucleos	20	25195693	25228075
ENSG0000023678	-2.330149155	0.107	0.2	AL391650.1	protein_coding	novel protei	1	26169516	26171831
ENSG0000016826	0.134500757	0.107	0.2	NT5DC2	protein_coding	5'-nucleotic	3	52524385	52535054
ENSG0000019695	0.870815533	0.107	0.2	SCOC-AS1	lncRNA	SCOC antise	4	140283724	140373403
ENSG0000010608	-0.153497531	0.107	0.2	FKBP14	protein_coding	FKBP prolyl	7	30010587	30026702
ENSG0000026840	0.566398409	0.107	0.2	AC132192.2	lncRNA	novel transc	11	9459556	9460702
ENSG0000011105	-0.559248018	0.107	0.2	KRT18	protein_coding	keratin 18 [S	12	52948871	52952906
ENSG0000027848	0.771467174	0.107	0.2	NAPRT	protein_coding	nicotinate p	CHR_HSCHR	143574785	143578649
ENSG0000023148	-1.946333476	0.107	0.2	NA	NA	NA	NA	NA	NA
ENSG0000018332	-0.310292303	0.107	0.2	CCDC125	protein_coding	coiled-coil c	5	69280175	69332809
ENSG0000012646	-0.243260135	0.107	0.2	PRR12	protein_coding	proline rich	19	49591182	49626439
ENSG0000021482	1.153921584	0.107	0.2	MPRIPP1	processed_pse	myosin pho	3	44579938	44581026
ENSG0000017708	-0.20653498	0.107	0.2	WDR73	protein_coding	WD repeat c	15	84639285	84654343
ENSG0000028020	-3.694809134	0.107	0.2	NA	NA	NA	NA	NA	NA
ENSG0000006324	-0.087773622	0.107	0.2	U2AF2	protein_coding	U2 small nu	19	55654146	55674716
ENSG0000012410	0.384465692	0.107	0.2	SNX21	protein_coding	sorting nexi	20	45833799	45843276
ENSG0000018109	0.120894853	0.107	0.2	EHMT1	protein_coding	euchromatic	9	137618992	137870016
ENSG0000017683	0.234353236	0.107	0.2	VSIG10	protein_coding	V-set and ir	12	118063593	118136026

ENSG0000026031	0.843059917	0.107	0.2	AC009812.	lncRNA	novel transc	8	80541300	80543104
ENSG0000016963	0.288680081	0.107	0.2	HIC2	protein_codin	HIC ZBTB tr	22	21417371	21451463
ENSG0000017904	-0.158345363	0.108	0.2	RRS1	protein_codin	ribosome bi	8	66429014	66430733
ENSG0000023200	1.093610121	0.108	0.2	CAP1P2	processed_pse	CAP1 pseuc	10	43604843	43606251
ENSG0000000398	0.33360614	0.108	0.2	MTMR7	protein_codin	myotubulari	8	17296794	17413528
ENSG0000025544	-2.251279484	0.108	0.2	AP003064.	lncRNA	novel transc	11	62421845	62426724
ENSG0000026994	0.547013734	0.108	0.2	AL049840.	lncRNA	novel transc	14	103694560	103695170
ENSG0000022182	0.202152256	0.108	0.2	FANCG	protein_codin	FA complen	9	35073835	35079942
ENSG0000027877	2.657789724	0.108	0.2	U2	snRNA	U2 spliceos	17	43273101	43273292
ENSG0000011464	-0.209134576	0.108	0.2	KLHL18	protein_codin	kelch like fa	3	47282917	47346816
ENSG0000027007	0.965310619	0.108	0.2	AC087203.	lncRNA	novel transc	8	12412827	12414373
ENSG0000027115	-1.726904978	0.108	0.2	RPL23AP88	processed_pse	ribosomal p	2	242175181	242175634
ENSG0000021358	0.079112998	0.109	0.2	VDAC1	protein_codin	voltage dep	5	133971871	134004975
ENSG0000027482	0.258336227	0.109	0.2	PARN	protein_codin	poly(A)-spe	CHR_HSCHR	14435701	14632727
ENSG0000027965	1.434510991	0.109	0.2	AL451064.	TEC	TEC	6	79573877	79575530
ENSG0000000414	0.146226272	0.109	0.2	POLDIP2	protein_codin	DNA polym	17	28346633	28357527
ENSG0000010593	0.126191502	0.109	0.2	ZC3HAV1	protein_codin	zinc finger (	7	139043515	139109720
ENSG0000023150	0.527457292	0.109	0.2	LSM2	protein_codin	LSM2 homc	CHR_HSCHR	31779577	31789166
ENSG0000010867	0.135015913	0.109	0.2	PSMD11	protein_codin	proteasome	17	32444379	32483319
ENSG0000010658	0.109373117	0.109	0.2	PSMA2	protein_codin	proteasome	7	42916861	42932185
ENSG0000004664	-0.271970973	0.109	0.2	GEMIN8	protein_codin	gem nuclea	X	14008279	14029893
ENSG0000013478	-0.306671687	0.109	0.2	DAGLA	protein_codin	diacylglycer	11	61680391	61747001
ENSG0000017085	-0.113116335	0.109	0.2	KBTBD2	protein_codin	kelch repea	7	32868172	32894131
ENSG0000023268	-1.50168965	0.109	0.2	AL592430.	lncRNA	novel transc	10	60050668	60060743
ENSG0000027433	-0.281022968	0.109	0.2	CDIP1	protein_codin	cell death ir	CHR_HSCHR	4512634	4526261
ENSG0000014174	0.228601327	0.109	0.2	MIEN1	protein_codin	migration a	17	39728496	39730532
ENSG0000011002	0.198899948	0.109	0.2	SNX15	protein_codin	sorting nexi	11	65027439	65040572
ENSG0000012225	-0.097510419	0.11	0.2	RBBP6	protein_codin	RB binding	16	24537693	24572863
ENSG0000000500	-0.096230498	0.11	0.2	UPF1	protein_codin	UPF1 RNA h	19	18831959	18868230
ENSG0000016366	0.095105746	0.11	0.2	CCNL1	protein_codin	cyclin L1 [Sc	3	157146508	157160760
ENSG0000020435	-1.187520135	0.11	0.2	C9orf129	transcribed_un	chromosom	9	93318199	93346414
ENSG0000013611	-1.012792794	0.11	0.2	THSD1	protein_codin	thrombospc	13	52377167	52416373
ENSG0000019772	0.091267765	0.11	0.2	RPS26	protein_codin	ribosomal p	12	56041351	56044697
ENSG0000016603	-0.263360406	0.11	0.2	HTRA1	protein_codin	HtrA serine	10	122458551	122514907
ENSG0000027019	1.496343365	0.11	0.2	AC016773.	lncRNA	novel transc	4	1712821	1715945
ENSG0000021411	-0.172397376	0.11	0.2	LYRM4	protein_codin	LYR motif c	6	5103629	5260950
ENSG0000008309	0.128897469	0.11	0.2	DOP1A	protein_codin	DOP1 leucir	6	83067666	83171350
ENSG0000010878	-0.303047517	0.11	0.2	HSD17B1	protein_codin	hydroxyster	17	42552922	42555214
ENSG0000023228	-0.745568921	0.11	0.2	FLOT1	protein_codin	flotillin 1 [Sc	CHR_HSCHR	30717565	30732592
ENSG0000004975	0.191317328	0.11	0.2	NEDD4L	protein_codin	NEDD4 like	18	58044226	58401540
ENSG0000020635	0.358544954	0.11	0.2	SKIV2L	protein_codin	Ski2 like RN	CHR_HSCHR	31949457	31960128
ENSG0000027306	2.009580077	0.11	0.2	CDC37L1-D	lncRNA	CDC37L1 di	9	4676600	4679502
ENSG0000017202	0.123879141	0.111	0.2	GAP43	protein_codin	growth assc	3	115623510	115721490
ENSG0000025824	1.538608919	0.111	0.2	AC002351.	lncRNA	novel transc	12	110951683	110957820
ENSG0000027718	1.217668457	0.111	0.2	AC006449.	lncRNA	novel transc	17	38749360	38751457
ENSG0000010991	-0.153483789	0.111	0.2	ZPR1	protein_codin	ZPR1 zinc fi	11	116773799	116788039
ENSG0000018820	-0.115873035	0.111	0.2	NA	NA	NA	NA	NA	NA
ENSG0000014474	-0.143544859	0.111	0.2	ARL6IP5	protein_codin	ADP ribosyl	3	69084937	69106092
ENSG0000019868	-0.65305516	0.111	0.2	PAPSS2	protein_codin	3'-phospho	10	87659613	87747705
ENSG0000010647	-0.176156255	0.111	0.2	CEP41	protein_codin	centrosoma	7	130393771	130442433
ENSG0000008544	-0.128862847	0.111	0.2	WDFY1	protein_codin	WD repeat	2	223855716	223945357
ENSG0000015444	0.173096194	0.111	0.2	SH3RF1	protein_codin	SH3 domair	4	169094259	169270956
ENSG0000027830	1.928305505	0.111	0.2	TAS2R19	protein_codin	taste 2 rece	CHR_HSCHR	11021607	11022608
ENSG0000012898	0.074476289	0.111	0.2	ARPP19	protein_codin	cAMP regul	15	52547045	52569883
ENSG0000014233	0.237251512	0.111	0.2	CAPN10	protein_codin	calpain 10 [	2	240586734	240617705
ENSG0000019646	0.485121891	0.111	0.2	ZNF799	protein_codin	zinc finger p	19	12390016	12401271
ENSG0000026069	2.155276073	0.111	0.2	AC026150.	lncRNA	novel transc	15	30540093	30545969
ENSG0000015462	-1.561597269	0.111	0.2	TMSB4Y	protein_codin	thymosin be	Y	13703899	13706024

ENSG0000027756	0.072612988	0.112	0.2	RBFOX2	protein_coding	RNA binding	CHR_HSCHR	35744967	35945128
ENSG0000010034	-0.142143529	0.112	0.2	SAMM50	protein_coding	SAMM50 sc	22	43955442	44010531
ENSG0000017392	-0.895112927	0.112	0.2	MARCHF3	protein_coding	membrane	5	126867714	127030558
ENSG0000023210	1.448378134	0.112	0.2	RFX3-AS1	lncRNA	RFX3 antise	9	3526723	3691814
ENSG0000027638	1.841285405	0.112	0.2	NA	NA	NA	NA	NA	NA
ENSG0000018523	0.154811552	0.112	0.2	RAB11B	protein_coding	RAB11B, me	19	8389981	8404434
ENSG0000018534	-0.607691865	0.112	0.2	GAS2L1	protein_coding	growth arre	22	29306582	29312785
ENSG0000027442	0.470015735	0.112	0.2	AC245060.	lncRNA	novel transc	22	22283928	22287220
ENSG0000023517	-0.950497924	0.112	0.21	ZNRD1	protein_coding	zinc ribbon	CHR_HSCHR	30054063	30060065
ENSG0000020651	0.641051858	0.112	0.21	GABBR1	protein_coding	gamma-am	CHR_HSCHR	29555518	29633080
ENSG0000024271	-0.406730293	0.112	0.21	CCDC169	protein_coding	coiled-coil	13	36222008	36297840
ENSG0000025015	-0.640686682	0.112	0.21	AC106791.	lncRNA	novel transc	5	137809780	137889394
ENSG0000016262	0.690551955	0.112	0.21	LRRIQ3	protein_coding	leucine rich	1	74026015	74198187
ENSG0000024112	-0.251425307	0.112	0.21	YAE1	protein_coding	YAE1 matur	7	39566385	39610320
ENSG0000025477	-0.924707877	0.112	0.21	AL662828.1	protein_coding	palmitoyl-p	CHR_HSCHR	32102303	32120449
ENSG0000011626	0.154838004	0.113	0.21	STXBP3	protein_coding	syntaxin bin	1	108746674	108809523
ENSG0000012708	-0.356047624	0.113	0.21	ZNF484	protein_coding	zinc finger p	9	92844182	92878038
ENSG0000026847	0.398234347	0.113	0.21	MIR4453H(	lncRNA	MIR4453 hc	4	152536264	152539263
ENSG0000026800	-1.383049209	0.113	0.21	CARD8-AS	lncRNA	CARD8 antis	19	48255675	48258199
ENSG0000010279	-0.437018111	0.113	0.21	DHRS12	protein_coding	dehydroger	13	51767993	51804162
ENSG0000011265	-0.222032911	0.113	0.21	MRPL2	protein_coding	mitochondr	6	43054029	43059438
ENSG0000019681	0.206962665	0.113	0.21	CTBP1-DT	lncRNA	CTBP1 diver	4	1249300	1288291
ENSG0000022569	1.932674573	0.113	0.21	LAGE3P1	processed_pse	L antigen fa	9	33019682	33020165
ENSG0000025402	2.249438215	0.113	0.21	AC083843.	lncRNA	novel transc	8	134832747	134834482
ENSG0000027953	-2.465237593	0.113	0.21	AC093458.	TEC	tec	7	149881477	149882105
ENSG0000013511	0.290759752	0.113	0.21	RNFT2	protein_coding	ring finger p	12	116738178	116853631
ENSG0000000645	-1.416591575	0.113	0.21	BAIAP2L1	protein_coding	BAR/IMD dc	7	98291650	98401090
ENSG0000018450	-0.498778518	0.113	0.21	HDDC3	protein_coding	HD domain	15	90929968	90935196
ENSG0000027462	0.806091131	0.113	0.21	RPS9	protein_coding	ribosomal p	CHR_HSCHR	54201473	54208260
ENSG0000016321	-0.125404908	0.113	0.21	DHX57	protein_coding	DExH-box h	2	38797729	38875934
ENSG0000025653	0.323596326	0.114	0.21	SMIM10L1	protein_coding	small integr	12	11171194	11176016
ENSG0000016329	-1.059571663	0.114	0.21	NIPAL1	protein_coding	NIPA like dc	4	47914142	48040173
ENSG0000013489	-0.132788459	0.114	0.21	BIVM	protein_coding	basic, immu	13	102799119	102841533
ENSG0000025416	0.85817633	0.114	0.21	AC009812.	lncRNA	novel transc	8	80535006	80539135
ENSG0000016718	-0.20474757	0.114	0.21	SP2	protein_coding	Sp2 transcri	17	47896150	47928957
ENSG0000015630	-0.116025601	0.114	0.21	SCAF4	protein_coding	SR-related (	21	31671000	31732118
ENSG0000016224	-0.440150479	0.114	0.21	SLC25A45	protein_coding	solute carrie	11	65375192	65383701
ENSG0000010472	-0.194720464	0.114	0.21	ARHGEF10	protein_coding	Rho guanine	8	1823926	1958641
ENSG0000012190	1.531818334	0.114	0.21	TMEM54	protein_coding	transmembr	1	32894594	32901438
ENSG0000014399	-1.288602226	0.114	0.21	ABHD1	protein_coding	abhydrolase	2	27123789	27130812
ENSG0000027770	-0.326390406	0.114	0.21	AC159540.	lncRNA	novel transc	2	97281356	97291849
ENSG0000014959	0.435115976	0.114	0.21	TAGLN	protein_coding	transgelin [S	11	117199370	117207464
ENSG0000013187	-0.171507709	0.114	0.21	SELENOS	protein_coding	selenoprote	15	101270817	101277500
ENSG0000017772	-0.144846173	0.115	0.21	TMEM94	protein_coding	transmembr	17	75441159	75500452
ENSG0000027188	1.988355394	0.115	0.21	AP001330.	lncRNA	novel transc	8	101208148	101208558
ENSG0000027111	-0.820193511	0.115	0.21	AC026412.	lncRNA	novel transc	5	1594626	1611467
ENSG0000026538	1.822828061	0.115	0.21	RN7SL219P	misc_RNA	RNA, 7SL, c	16	2013181	2013476
ENSG0000016393	-0.118601994	0.115	0.21	PBRM1	protein_coding	polybromo	3	52545352	52685917
ENSG0000023261	1.354191157	0.115	0.21	AC026412.	processed_pse	protein pho	5	1614836	1616334
ENSG0000010734	-0.100455775	0.115	0.21	UBE2R2	protein_coding	ubiquitin co	9	33817160	33920399
ENSG0000022781	-0.516384351	0.115	0.21	INKA2-AS1	lncRNA	INKA2 antis	1	111739579	111747798
ENSG0000015940	0.123672244	0.115	0.21	CELF3	protein_coding	CUGBP Elav	1	151700058	151716803
ENSG0000025605	-0.20466146	0.115	0.21	COA8	protein_coding	cytochrome	14	103562960	103607523
ENSG0000019613	-0.218721465	0.115	0.21	MYT1	protein_coding	myelin trans	20	64102394	64242253
ENSG0000024892	1.676760851	0.115	0.21	MTND5P11	processed_pse	MT-ND5 ps	5	134924648	134926459
ENSG0000012164	0.102648411	0.115	0.21	DESI2	protein_coding	desumoylat	1	244653103	244709033
ENSG0000016362	-0.829253619	0.115	0.21	NKX6-1	protein_coding	NK6 homeo	4	84491987	84498450
ENSG0000024825	1.541806947	0.116	0.21	OCIAD1-AS	lncRNA	OCIAD1 ant	4	48852008	48860203

ENSG0000010199	-0.307826592	0.116	0.21	CCDC22	protein_coding	coiled-coil c	X	49235470	49250520
ENSG0000016801	0.267436757	0.116	0.21	ATG16L2	protein_coding	autophagy r	11	72814406	72843674
ENSG0000005914	0.200734195	0.116	0.21	UNKL	protein_coding	unk like zinc	16	1363205	1414751
ENSG0000025743	-0.72457967	0.116	0.21	AC004241.1	lncRNA	novel transc	12	47706083	47742294
ENSG0000018237	-0.873791963	0.116	0.21	NXPH4	protein_coding	neurexophil	12	57216794	57226449
ENSG0000001327	-0.134236563	0.116	0.21	PSMC4	protein_coding	proteasome	19	39971165	39981764
ENSG0000018533	-0.713757419	0.116	0.21	TCN2	protein_coding	transcobalar	22	30607003	30627271
ENSG0000017346	-0.232109912	0.116	0.21	ZNRD2	protein_coding	zinc ribbon	11	65570460	65573942
ENSG0000011741	0.113121102	0.116	0.21	ATP6V0B	protein_coding	ATPase H+	1	43974487	43978295
ENSG0000014254	-0.562552637	0.116	0.21	CTU1	protein_coding	cytosolic thi	19	51097606	51108409
ENSG0000015806	0.356690352	0.116	0.21	UBXN11	protein_coding	UBX domain	1	26281328	26318363
ENSG0000028043	-0.263510586	0.116	0.21	FP565260.6	protein_coding	novel protei	21	5155499	5165472
ENSG0000012953	-1.04898785	0.116	0.21	NRL	protein_coding	neural retina	14	24078662	24115010
ENSG0000021318	-0.495813606	0.116	0.21	FAM24B	protein_coding	family with s	10	122849078	122879641
ENSG0000026281	-0.132354918	0.116	0.21	MRPL12	protein_coding	mitochondr	17	81703367	81707517
ENSG0000015264	-0.185151999	0.116	0.21	GPD1L	protein_coding	glycerol-3-p	3	32105689	32168709
ENSG0000024314	0.172007219	0.117	0.21	MRPL33	protein_coding	mitochondr	2	27771717	27988087
ENSG0000009687	0.213024173	0.117	0.21	IFT74	protein_coding	intraflagella	9	26947039	27062930
ENSG0000010115	0.110169416	0.117	0.21	DNAJC5	protein_coding	DnaJ heat sh	20	63895126	63936031
ENSG0000025015	1.028081518	0.117	0.21	ARPC4-TTL	protein_coding	ARPC4-TTLL	3	9793082	9835401
ENSG0000016686	-1.045902891	0.117	0.21	CACNG2	protein_coding	calcium volt	22	36563921	36703558
ENSG0000011857	-0.095529506	0.117	0.21	MED28	protein_coding	mediator co	4	17614641	17634105
ENSG0000021524	-1.613290078	0.117	0.21	LINC02449	lncRNA	long interge	12	8235415	8242564
ENSG0000017008	0.33095263	0.117	0.21	AC106795.1	transcribed_un	THO comple	5	177809407	177950732
ENSG0000027794	2.611813658	0.117	0.21	AC007435.1	misc_RNA		2	175092401	175092587
ENSG0000011629	0.480403675	0.117	0.21	ELAPOR1	protein_coding	endosome-	1	109113679	109206781
ENSG0000007420	0.095828898	0.118	0.21	CLNS1A	protein_coding	chloride nuc	11	77514936	77637794
ENSG0000017293	-0.297288911	0.118	0.21	MYD88	protein_coding	MYD88 inna	3	38138478	38143022
ENSG0000019609	0.178598249	0.118	0.21	PAX5	protein_coding	paired box 5	9	36833269	37034268
ENSG0000013567	0.107517149	0.118	0.22	GNS	protein_coding	glucosamine	12	64713445	64759431
ENSG0000014200	-0.135543601	0.118	0.22	DPP9	protein_coding	dipeptidyl p	19	4675224	4724673
ENSG0000015331	-0.075703664	0.118	0.22	ASAP1	protein_coding	ArfGAP with	8	130052104	130443674
ENSG0000027634	-0.85821271	0.118	0.22	TBC1D3L	protein_coding	TBC1 doma	CHR_HSCHR	37982250	37993199
ENSG0000010801	0.10670806	0.119	0.22	SORCS1	protein_coding	sortilin relat	10	106573663	107164534
ENSG0000016815	0.299434408	0.119	0.22	THAP9	protein_coding	THAP doma	4	82900684	82919969
ENSG0000021388	-1.414969641	0.119	0.22	PPM1N	protein_coding	protein pho	19	45488777	45502510
ENSG0000025001	2.639051082	0.119	0.22	AC079848.1	lncRNA	novel transc	3	126083659	126095349
ENSG0000025973	1.471893469	0.119	0.22	CRTC3-AS1	lncRNA	CRTC3 antis	15	90620007	90717141
ENSG0000027456	-0.906162484	0.119	0.22	AC005332.3	lncRNA	novel transc	17	68131462	68131907
ENSG0000017162	0.520771236	0.119	0.22	SPSB1	protein_coding	splA/ryanoc	1	9292894	9369532
ENSG0000014785	0.115197533	0.119	0.22	UHRF2	protein_coding	ubiquitin lik	9	6413151	6507054
ENSG0000015290	-0.127685181	0.119	0.22	GGPS1	protein_coding	geranylgera	1	235327350	235344532
ENSG0000025836	-1.257089684	0.119	0.22	AC073655.2	lncRNA	novel transc	12	94277758	94282844
ENSG0000021954	1.508397769	0.119	0.22	UMAD1	protein_coding	UBAP1-MVI	7	7640711	7968020
ENSG0000027558	-0.339377588	0.119	0.22	SERF1A	protein_coding	small EDRK-	CHR_HSCHR	70244409	70262299
ENSG0000017447	-0.910012069	0.119	0.22	GALNTL6	protein_coding	polypeptide	4	171812254	173041559
ENSG0000027268	0.451802163	0.119	0.22	WASL-DT	lncRNA	WASL diverg	7	123749068	123751166
ENSG0000023115	-1.053548871	0.119	0.22	MORF4L2-A	lncRNA	MORF4L2 a	X	103687284	103691772
ENSG0000016770	0.731566466	0.119	0.22	GPT	protein_coding	glutamic--p	8	144502973	144507174
ENSG0000016433	0.237514074	0.119	0.22	ANKRA2	protein_coding	ankyrin repe	5	73552190	73565667
ENSG0000002042	-0.222135928	0.12	0.22	MNAT1	protein_coding	MNAT1 com	14	60734742	60969965
ENSG0000027116	-1.972176912	0.12	0.22	BOLA2P2	transcribed_pr	bolA family	3	47499841	47500407
ENSG0000023393	-1.611199211	0.12	0.22	KRTAP5-AS	lncRNA	KRTAP5-1/K	11	1571353	1599184
ENSG0000026142	-1.229190967	0.12	0.22	TMEM202-	lncRNA	TMEM202 a	15	72407778	72475168
ENSG0000023530	3.039625005	0.12	0.22	AL445991.1	processed_pse	novel pseuc	1	88923370	88923844
ENSG0000022778	-2.39084852	0.12	0.22	MTCO3P43	processed_pse	MT-CO3 ps	2	120211727	120212862
ENSG0000002304	-0.144237829	0.12	0.22	ZDHHC6	protein_coding	zinc finger D	10	112424428	112446917
ENSG0000019705	1.08071865	0.12	0.22	ZNF763	protein_coding	zinc finger p	19	11965037	11980617

ENSG0000024856	0.812959314	0.12	0.22	AC026410.3	processed_pse	mediator cc	5	80351021	80351956
ENSG0000018686	-0.741989138	0.12	0.22	PDZD7	protein_coding	PDZ domair	10	101007679	101032295
ENSG0000018548	-0.945100738	0.12	0.22	STAC3	protein_coding	SH3 and cys	12	57243453	57251188
ENSG0000018525	-0.14966605	0.12	0.22	ZNF74	protein_coding	zinc finger p	22	20394115	20408461
ENSG0000022508	1.602953387	0.12	0.22	PFN1P4	processed_pse	profilin 1 ps	1	148129497	148129898
ENSG0000023636	1.034640499	0.12	0.22	AL358115.1	lncRNA	novel transc	1	165889725	165900995
ENSG0000017057	-0.8180744	0.12	0.22	SIX2	protein_coding	SIX homeob	2	45005182	45009452
ENSG0000013839	0.099403629	0.12	0.22	PPIG	protein_coding	peptidylprol	2	169584344	169641406
ENSG0000023601	0.703269971	0.12	0.22	AC011290.2	processed_pse	thioredoxin	7	39569376	39570198
ENSG0000015180	-0.141283556	0.12	0.22	GUF1	protein_coding	GUF1 homo	4	44678420	44700928
ENSG0000017308	0.293283142	0.12	0.22	COQ2	protein_coding	coenzyme C	4	83261536	83284914
ENSG0000007814	-0.087113823	0.12	0.22	UBE2K	protein_coding	ubiquitin co	4	39698109	39782792
ENSG0000026924	-0.371348992	0.12	0.22	AC010422.3	protein_coding	novel transc	19	12643831	12648397
ENSG0000018813	0.217778383	0.121	0.22	MAPK12	protein_coding	mitogen-ac	22	50245450	50261716
ENSG0000010661	0.341646351	0.121	0.22	STAG3L4	transcribed_un	stromal anti	7	67302621	67362950
ENSG0000025602	0.307659851	0.121	0.22	AC026362.1	lncRNA	novel transc	12	122975320	122982907
ENSG0000021010	-0.579176145	0.121	0.22	MT-TQ	Mt_tRNA	mitochondr	MT	4329	4400
ENSG0000025070	-1.107117258	0.121	0.22	LINC02269	lncRNA	long interge	4	173699082	174001488
ENSG0000023206	2.704901901	0.121	0.22	LINC01063	lncRNA	long interge	3	196631498	196632587
ENSG0000007933	0.250771088	0.121	0.22	CDC14A	protein_coding	cell division	1	100345001	100520277
ENSG0000016684	1.033236002	0.121	0.22	GLYATL1	protein_coding	glycine-N-a	11	58905398	59043527
ENSG0000024531	0.739056205	0.122	0.22	AC008393.1	lncRNA	novel transc	5	179859013	179861283
ENSG0000014459	-0.639959831	0.122	0.22	GRIP2	protein_coding	glutamate r	3	14489107	14556075
ENSG0000016750	-0.169667134	0.122	0.22	MVD	protein_coding	mevalonate	16	88651935	88663161
ENSG0000023577	0.672157487	0.122	0.22	NRM	protein_coding	nurim [Sour	CHR_HSCHR	30677910	30681283
ENSG0000026724	-1.455906246	0.122	0.22	AP005482.2	lncRNA	novel transc	18	12670426	12671145
ENSG0000022658	-0.191490243	0.122	0.22	VARS1	protein_coding	valyl-tRNA s	CHR_HSCHR	31764879	31783312
ENSG0000026804	1.116240851	0.122	0.22	AC012313.2	lncRNA	novel transc	19	58357999	58359603
ENSG0000025366	-1.108791344	0.122	0.22	AC100821.1	processed_pse	ESF1, nucle	8	53971231	53974210
ENSG0000017989	0.345312511	0.122	0.22	PHC1P1	processed_pse	polyhomeot	12	55411727	55414787
ENSG0000027607	-0.213701099	0.122	0.22	AATF	protein_coding	apoptosis a	CHR_HSCHR	36950708	37058704
ENSG0000006074	0.085092743	0.122	0.22	QSER1	protein_coding	glutamine a	11	32892811	32993316
ENSG0000025394	1.160485506	0.122	0.22	VPS13B-DT	lncRNA	VPS13B dive	8	98958277	99013743
ENSG0000013669	0.104120792	0.123	0.22	SMPD4	protein_coding	sphingomye	2	130151392	130182750
ENSG0000023461	0.130886649	0.123	0.22	JRK	protein_coding	Jrk helix-tur	8	142657460	142681968
ENSG0000013343	0.361634732	0.123	0.22	GSTT2B	protein_coding	glutathione	22	23957414	23961195
ENSG0000013408	0.136385415	0.123	0.22	VHL	protein_coding	von Hippel-	3	10141778	10153667
ENSG0000018044	-0.31440408	0.123	0.22	GAS1	protein_coding	growth arre	9	86944362	86947506
ENSG0000000606	-0.316729567	0.123	0.22	MAP3K14	protein_coding	mitogen-ac	17	45263119	45317029
ENSG0000016566	0.172958973	0.123	0.22	FAM204A	protein_coding	family with s	10	118297925	118342328
ENSG0000017386	1.656102097	0.123	0.22	AC013489.1	protein_coding	novel transc	15	88459501	88546585
ENSG0000010895	1.270328164	0.123	0.22	AC130689.1	processed_pse	succinate de	17	1858039	1858446
ENSG0000025954	-1.328266588	0.123	0.22	AC027020.1	lncRNA	novel transc	15	100558677	100559798
ENSG0000027004	1.915579501	0.123	0.22	AC068790.4	lncRNA	novel transc	12	123962555	123962817
ENSG0000027876	-1.386739705	0.123	0.22	FAM27B	processed_pse	family with s	9	67725690	67725902
ENSG0000019744	-0.2691995	0.123	0.22	MAP3K5	protein_coding	mitogen-ac	6	136557046	136792477
ENSG0000016647	-0.119870061	0.124	0.22	LEO1	protein_coding	LEO1 homo	15	51938025	51971778
ENSG0000027335	1.829102908	0.124	0.22	NA	NA	NA	NA	NA	NA
ENSG0000022695	-2.091504127	0.124	0.22	NCKAP5-A	lncRNA	NCKAP5 ant	2	133264968	133285445
ENSG0000022204	-2.184465637	0.124	0.22	AC079305.1	lncRNA	novel transc	2	177264359	177265515
ENSG0000010482	0.076118943	0.124	0.22	HNRNPL	protein_coding	heterogene	19	38836388	38852347
ENSG0000011701	0.430806345	0.124	0.22	ZNF684	protein_coding	zinc finger p	1	40531573	40548167
ENSG0000003956	-0.652185403	0.124	0.22	RAI14	protein_coding	retinoic acic	5	34656328	34832612
ENSG0000020648	-0.799326207	0.124	0.22	C6orf136	protein_coding	chromosom	CHR_HSCHR	30636534	30642598
ENSG0000010006	-0.266136612	0.124	0.22	LRP5L	protein_coding	LDL recepto	22	25351418	25405377
ENSG0000011738	-0.166641528	0.124	0.22	P3H1	protein_coding	prolyl 3-hyc	1	42746335	42767084
ENSG0000012425	-1.126329613	0.125	0.22	TP53TG5	protein_coding	TP53 target	20	45372557	45407889
ENSG0000011178	0.290940619	0.125	0.22	AC009533.1	unprocessed_p	DEAD/H (As	12	9277235	9313241

ENSG0000010548	0.227424923	0.125	0.23	LIG1	protein_coding	DNA ligase	19	48115445	48170603
ENSG0000006470	0.133415886	0.125	0.23	DDX20	protein_coding	DEAD-box l	1	111755245	111768000
ENSG0000027636	2.573648818	0.125	0.23	H2AC14	protein_coding	H2A cluster	6	27814302	27814777
ENSG0000016642	-0.358091544	0.125	0.23	CRABP1	protein_coding	cellular retir	15	78340353	78348225
ENSG0000026770	-1.724684066	0.125	0.23	NA	NA	NA	NA	NA	NA
ENSG0000017845	0.470709565	0.125	0.23	H3P16	processed_pse	H3 histone	4	139698144	139698554
ENSG0000018699	-1.26894628	0.125	0.23	EMID1	protein_coding	EMI domain	22	29205896	29259597
ENSG0000026094	-0.962232776	0.125	0.23	AP006545.1	lncRNA	novel transc	8	38062881	38063791
ENSG0000016791	-0.444925794	0.126	0.23	AC090152.1	lncRNA	novel transc	8	59119040	59123478
ENSG0000023545	0.809753039	0.126	0.23	SMIM27	protein_coding	small integr	9	32551144	32568621
ENSG0000010652	-0.899304058	0.126	0.23	ACTR3C	protein_coding	actin relatec	7	150243916	150323725
ENSG0000019720	1.070111188	0.126	0.23	SLC22A4	protein_coding	solute carrie	5	132294394	132344190
ENSG0000016894	-0.138925858	0.126	0.23	CEP120	protein_coding	centrosoma	5	123344890	123423592
ENSG0000024084	0.186689243	0.126	0.23	TMEM189	protein_coding	transmembr	20	50118254	50153734
ENSG0000022531	1.935892082	0.126	0.23	HSD17B8	protein_coding	hydroxyster	CHR_HSCHR	33182548	33184737
ENSG0000016599	-0.327097549	0.126	0.23	HACD1	protein_coding	3-hydroxya	10	17589032	17617374
ENSG0000007758	-0.456809149	0.126	0.23	GPR137B	protein_coding	G protein-c	1	236142505	236221865
ENSG0000012072	1.57788475	0.126	0.23	MYOT	protein_coding	myotilin [So	5	137867858	137887851
ENSG0000014395	0.321638644	0.126	0.23	WDPCP	protein_coding	WD repeat	2	63119559	63827843
ENSG0000027540	-1.362889487	0.126	0.23	AL391095.3	lncRNA	novel transc	20	38418483	38419202
ENSG0000027762	0.547205432	0.126	0.23	WNT3	protein_coding	Wnt family	CHR_HSCHR	47057885	47113984
ENSG0000016030	1.197261847	0.126	0.23	S100B	protein_coding	S100 calciur	21	46598604	46605208
ENSG0000017986	1.019510832	0.126	0.23	CITED4	protein_coding	Cbp/p300 ir	1	40861054	40862363
ENSG0000027005	0.382475367	0.127	0.23	AC127502.2	lncRNA	novel transc	15	30487963	30490313
ENSG0000026835	0.222492567	0.127	0.23	FAM156A	protein_coding	family with	X	52926402	52995472
ENSG0000011651	-0.306393178	0.127	0.23	RNF19B	protein_coding	ring finger p	1	32936445	32964685
ENSG0000000637	0.367508404	0.127	0.23	DLX6	protein_coding	distal-less h	7	97005553	97011040
ENSG0000027688	0.584254322	0.127	0.23	IFI27L1	protein_coding	interferon a	CHR_HSCHR	94081282	94103846
ENSG0000007038	1.718502656	0.127	0.23	FGF22	protein_coding	fibroblast gi	19	639879	644371
ENSG0000027230	0.762146133	0.127	0.23	NA	NA	NA	NA	NA	NA
ENSG0000018420	0.171542421	0.127	0.23	C22orf46	transcribed_un	chromosom	22	41688877	41698136
ENSG0000012951	0.182597536	0.127	0.23	EAPP	protein_coding	E2F associat	14	34515938	34539711
ENSG0000014336	-0.185494344	0.127	0.23	PRUNE1	protein_coding	prune exop	1	151008420	151035713
ENSG0000018597	-0.283603262	0.127	0.23	TMLHE	protein_coding	trimethyllysi	X	155489011	155719098
ENSG0000028021	-0.646510206	0.127	0.23	UCKL1-AS1	lncRNA	UCKL1 antis	20	63953384	63956985
ENSG0000022604	1.242154384	0.127	0.23	AC005740.1	processed_pse	glycine clea	5	141896168	141896689
ENSG0000027181	2.215522793	0.128	0.23	U3	snoRNA	Small nucle	4	158700691	158700909
ENSG0000023390	2.195652294	0.128	0.23	LINC01503	lncRNA	long interge	9	129332300	129359541
ENSG0000010670	-0.192903819	0.128	0.23	FSD1L	protein_coding	fibronectin t	9	105447796	105552433
ENSG0000016473	0.083048637	0.128	0.23	CTSB	protein_coding	cathepsin B	8	11842524	11869448
ENSG0000026580	0.848707377	0.128	0.23	AC022211.1	lncRNA	novel transc	17	75138416	75141350
ENSG0000020632	-0.576123846	0.128	0.23	PPT2	protein_coding	palmitoyl-p	CHR_HSCHR	32111042	32121288
ENSG0000014038	0.209463555	0.128	0.23	SCAPER	protein_coding	S-phase cyc	15	76347904	76905444
ENSG0000018922	-0.390909445	0.128	0.23	C15orf61	protein_coding	chromosom	15	67521131	67530146
ENSG0000023238	-0.559961639	0.128	0.23	AL583856.1	processed_pse	spermine sy	6	70608234	70609334
ENSG0000024105	0.203277515	0.128	0.23	NSUN6	protein_coding	NOP2/Sun	10	18545561	18659285
ENSG0000019674	0.581591141	0.129	0.23	LINC01560	lncRNA	long interge	X	47483571	47484823
ENSG0000011229	-0.644558461	0.129	0.23	GPLD1	protein_coding	glycosylpho	6	24424565	24495205
ENSG0000009987	0.134388104	0.129	0.23	MKNK2	protein_coding	MAPK intera	19	2037465	2051244
ENSG0000026706	-0.752427463	0.129	0.23	PTGES3L	protein_coding	prostagland	17	42968088	42980433
ENSG0000011680	0.193515216	0.129	0.23	ZBTB17	protein_coding	zinc finger a	1	15941869	15976132
ENSG0000022707	-2.331689018	0.129	0.23	AL158166.1	lncRNA	novel transc	10	127934698	127936167
ENSG0000026809	-2.331689018	0.129	0.23	AC022154.1	lncRNA	novel transc	19	48619272	48624132
ENSG0000018912	-2.562045369	0.129	0.23	PLAC9	protein_coding	placenta ass	10	80131682	80145359
ENSG0000024295	-1.02137222	0.129	0.23	ERVW-1	protein_coding	endogenous	7	92468380	92477986
ENSG0000014263	0.376247955	0.129	0.23	EFHD2	protein_coding	EF-hand do	1	15409888	15430339
ENSG0000019771	0.123608482	0.129	0.23	RPE	protein_coding	ribulose-5-	2	210002565	210022260
ENSG0000020605	-0.175151384	0.129	0.23	DOK6	protein_coding	docking prc	18	69400888	69849087

ENSG0000011278	-0.165426065	0.129	0.23	FBRSL1	protein_coding	fibrosin like	12	132489551	132585188
ENSG0000018511	0.263405082	0.129	0.23	NSMCE3	protein_coding	NSE3 homo	15	29264989	29269822
ENSG0000006502	-0.190017716	0.129	0.23	ZNF76	protein_coding	zinc finger p	6	35258909	35295985
ENSG0000012538	0.331179213	0.13	0.23	GRK4	protein_coding	G protein-c	4	2963571	3040760
ENSG0000022711	2.956918933	0.13	0.23	AC073210.1	unprocessed_p	pseudogene	7	65075023	65078780
ENSG0000023818	2.088005805	0.13	0.23	AL603839.2	lncRNA	novel transc	1	40515754	40517174
ENSG0000023211	0.159834944	0.13	0.23	MCTS1	protein_coding	MCTS1 re-ii	X	120594010	120621159
ENSG0000006499	-0.148927573	0.13	0.23	ANKS1A	protein_coding	ankyrin repe	6	34889255	35091406
ENSG0000006551	0.109656882	0.13	0.23	NDUFB4	protein_coding	NADH:ubiqu	3	120596328	120602507
ENSG0000017871	-0.151715271	0.13	0.23	GRINA	protein_coding	glutamate io	8	143990056	143993415
ENSG0000024194	-0.284077608	0.13	0.23	PWP2	protein_coding	PWP2 small	21	44107373	44131181
ENSG0000017273	0.157262301	0.13	0.23	MUS81	protein_coding	MUS81 stru	11	65857126	65867653
ENSG0000010548	-0.254760488	0.13	0.23	CARD8	protein_coding	caspase recr	19	48180770	48255946
ENSG0000016659	-1.571197045	0.13	0.23	RRAD	protein_coding	RRAD, Ras r	16	66921679	66925536
ENSG0000027057	1.397232565	0.13	0.23	AC010680.2	lncRNA	novel transc	2	178578790	178580906
ENSG0000013614	-1.006167404	0.13	0.23	RPL13AP25	processed_pse	ribosomal p	13	54440704	54441315
ENSG0000011108	-1.013092306	0.13	0.23	GLI1	protein_coding	GLI family zi	12	57459785	57472268
ENSG0000012005	-1.222669833	0.13	0.23	C10orf95	protein_coding	chromosom	10	102449837	102451543
ENSG0000023327	-0.541420083	0.13	0.23	SNRPEP4	processed_pse	SNRPE pseu	19	5576660	5576938
ENSG0000026398	2.977822805	0.13	0.23	AC009716.1	lncRNA	novel transc	18	76372717	76378275
ENSG0000023492	0.693558276	0.13	0.23	ATP6V1G2	protein_coding	ATPase H+	CHR_HSCHR	31535211	31537597
ENSG0000027846	-0.753305785	0.131	0.23	MRPS36	protein_coding	mitochondr	CHR_HSCHR	69217760	69230127
ENSG0000015355	-0.443319545	0.131	0.23	CMTM7	protein_coding	CKLF like M.	3	32391698	32483067
ENSG0000027760	-0.668198962	0.131	0.23	GUSBP1	transcribed_un	GUSB pseuc	CHR_HSCHR	69856054	69898246
ENSG0000025386	2.321522997	0.131	0.23	AC131025.1	lncRNA	novel transc	5	149372174	149375116
ENSG0000027383	-1.287812784	0.131	0.23	TFPT	protein_coding	TCF3 fusion	CHR_HSCHR	54107066	54115801
ENSG0000014838	0.284429038	0.131	0.23	INPP5E	protein_coding	inositol poly	9	136428619	136439845
ENSG0000022404	0.882859624	0.131	0.23	CCNT2-AS1	lncRNA	CCNT2 antis	2	134735464	134918710
ENSG0000024037	-0.728361897	0.131	0.23	RPL13P5	transcribed_pr	ribosomal p	12	6873389	6884741
ENSG0000010950	-0.203777707	0.131	0.23	WFS1	protein_coding	wolframin E	4	6269849	6303265
ENSG0000010968	0.290113888	0.131	0.23	TBC1D19	protein_coding	TBC1 doma	4	26576437	26756223
ENSG0000020639	1.098012334	0.131	0.23	DDAH2	protein_coding	dimethylarg	CHR_HSCHR	31717256	31720835
ENSG0000017727	-1.314067011	0.131	0.23	KCNA3	protein_coding	potassium v	1	110672465	110674940
ENSG0000026727	-0.965189763	0.131	0.23	MAP3K14-A	lncRNA	MAP3K14 a	17	45247916	45269824
ENSG0000012864	-0.930687258	0.131	0.23	HOXD1	protein_coding	homeobox I	2	176188668	176190907
ENSG0000014787	0.10163333	0.132	0.23	HAUS6	protein_coding	HAUS augm	9	19053141	19102904
ENSG0000014144	0.620244724	0.132	0.23	GREB1L	protein_coding	GREB1 like r	18	21242242	21525417
ENSG0000014860	-0.116335178	0.132	0.23	POLR3A	protein_coding	RNA polyme	10	77969251	78029515
ENSG0000015800	0.265677296	0.132	0.24	PAFAH2	protein_coding	platelet acti	1	25959767	25998117
ENSG0000027919	-0.629735473	0.132	0.24	AC008894.3	TEC	TEC	19	16074293	16077395
ENSG0000025368	0.698107513	0.132	0.24	AC027309.2	processed_pse	coiled-coil c	5	172656522	172656713
ENSG0000009497	-0.093962038	0.132	0.24	SUCO	protein_coding	SUN domain	1	172532349	172611833
ENSG0000023491	-0.292499796	0.132	0.24	SNHG20	lncRNA	small nucleo	17	77086716	77099902
ENSG0000015919	0.164251435	0.132	0.24	ATP5MC1	protein_coding	ATP synthas	17	48892765	48895871
ENSG0000010795	-0.088544348	0.132	0.24	SH3PXD2A	protein_coding	SH3 and PX	10	103594027	103855543
ENSG0000009921	-0.430101119	0.132	0.24	ERMP1	protein_coding	endoplasmi	9	5765076	5833117
ENSG0000007015	-0.196182205	0.132	0.24	PTPN3	protein_coding	protein tyro	9	109375466	109498313
ENSG0000022416	-1.262568951	0.132	0.24	LINC01357	lncRNA	long interge	1	112849821	112877871
ENSG0000026569	-0.68022172	0.132	0.24	AC074143.1	protein_coding	novel protei	16	67163385	67165815
ENSG0000019737	0.228296964	0.132	0.24	ZNF675	protein_coding	zinc finger p	19	23525631	23687220
ENSG0000028030	1.162386598	0.133	0.24	AC117503.4	TEC	novel transc	12	123754246	123754794
ENSG0000012015	-0.19555363	0.133	0.24	RCL1	protein_coding	RNA termin	9	4792944	4885917
ENSG0000016770	-0.287336586	0.133	0.24	MFSD3	protein_coding	major facilit	8	144509070	144511213
ENSG0000024871	-0.915683639	0.133	0.24	AC021127.1	lncRNA	putative nov	4	80182637	80197410
ENSG0000027979	-1.158714753	0.133	0.24	AC015909.5	TEC	TEC	17	50362967	50365232
ENSG0000009110	0.927726877	0.133	0.24	NLRC4	protein_coding	NLR family C	2	32224453	32265732
ENSG0000016923	0.192378439	0.133	0.24	THBS3	protein_coding	thrombospo	1	155195588	155209051
ENSG0000026852	0.977440371	0.133	0.24	VN1R83P	unprocessed_p	vomeronasa	19	21289554	21289998

ENSG0000016022	0.577004708	0.133	0.24	ZNF66	protein_coding	zinc finger p	19	20776304	20809995
ENSG0000027905	-0.286606519	0.133	0.24	AC007485.2	TEC	TEC	17	54956760	54958426
ENSG0000010908	-0.309970888	0.133	0.24	IFT20	protein_coding	intraflagella	17	28328325	28335489
ENSG0000017180	0.353468388	0.133	0.24	METTL18	protein_coding	methyltrans	1	169792529	169794963
ENSG0000011769	0.137130434	0.133	0.24	NSL1	protein_coding	NSL1 compo	1	212726153	212791782
ENSG0000016206	0.182655007	0.133	0.24	TBC1D24	protein_coding	TBC1 doma	16	2475051	2509560
ENSG0000025406	-0.913976368	0.133	0.24	AC105206.2	lncRNA	novel transc	8	22254576	22275162
ENSG0000027266	-1.267951962	0.133	0.24	AL021707.6	lncRNA	novel transc	22	38742625	38743115
ENSG0000011565	0.463598622	0.134	0.24	ABCB6	protein_coding	ATP binding	2	219209772	219218994
ENSG0000027325	1.199629671	0.134	0.24	AL022328.4	lncRNA	novel transc	22	50199090	50200837
ENSG0000009275	-0.684453958	0.134	0.24	COL9A3	protein_coding	collagen typ	20	62816244	62841159
ENSG0000013074	0.383539058	0.134	0.24	TMEM160	protein_coding	transmembr	19	47045909	47048624
ENSG0000017153	0.09457972	0.134	0.24	TBCA	protein_coding	tubulin foldi	5	77691166	77868780
ENSG0000023358	1.117858327	0.134	0.24	AL138789.1	lncRNA	novel transc	1	68479129	68483539
ENSG0000021430	0.797408922	0.134	0.24	SPDYE3	protein_coding	speedy/RIN	7	100307702	100322196
ENSG0000027257	-1.602653365	0.134	0.24	AC027271.1	lncRNA	novel transc	4	51918772	51919381
ENSG0000027616	-0.718257661	0.134	0.24	RN7SL1	misc_RNA	RNA compo	14	49586580	49586878
ENSG0000024274	-2.04925363	0.134	0.24	RPL23AP81	transcribed_pr	ribosomal p	20	41196691	41197157
ENSG0000026025	2.937455822	0.134	0.24	AC009087.1	lncRNA	novel transc	16	72084857	72087443
ENSG0000018572	-0.115161935	0.134	0.24	DRG1	protein_coding	developmer	22	31399604	31530634
ENSG0000014908	-0.121646969	0.134	0.24	HSD17B12	protein_coding	hydroxyster	11	43680680	43856617
ENSG0000027287	0.384948751	0.135	0.24	AP000525.1	lncRNA	novel transc	22	15823197	15823890
ENSG0000027787	-1.428559398	0.135	0.24	NA	NA	NA	NA	NA	NA
ENSG0000017160	0.305192534	0.135	0.24	ZNF274	protein_coding	zinc finger p	19	58183029	58213562
ENSG0000018099	-0.167373408	0.135	0.24	MRPL14	protein_coding	mitochondr	6	44113451	44127452
ENSG0000017227	0.151402308	0.135	0.24	HINFP	protein_coding	histone H4 t	11	119121580	119136059
ENSG0000018287	-1.044570993	0.135	0.24	PRKCZ-AS1	lncRNA	PRKCZ antis	1	2181794	2184389
ENSG0000012912	0.083543327	0.135	0.24	SPCS3	protein_coding	signal pepti	4	176319966	176332245
ENSG0000022617	0.409864984	0.135	0.24	DHX16	protein_coding	DEAH-box l	CHR_HSCHR	30645335	30665285
ENSG0000011809	0.18075207	0.135	0.24	IFT46	protein_coding	intraflagella	11	118544528	118572970
ENSG0000016721	-0.741296673	0.135	0.24	KATNAL2	protein_coding	katanin cata	18	46917492	47102243
ENSG0000011124	0.271173356	0.136	0.24	CUX2	protein_coding	cut like hom	12	111034165	111350554
ENSG0000025847	0.997812957	0.136	0.24	AC005726.1	protein_coding	novel protei	17	28455752	28614185
ENSG0000022917	1.952633777	0.136	0.24	SAPCD1	protein_coding	suppressor /	CHR_HSCHR	31750175	31752228
ENSG0000011262	-0.14558116	0.136	0.24	BICRAL	protein_coding	BRD4 intera	6	42746958	42868560
ENSG0000020431	0.578806894	0.136	0.24	PJVK	protein_coding	pejvakin [Sc	2	178451346	178462102
ENSG0000022467	-1.431754195	0.136	0.24	PDIA3P2	unprocessed_p	protein disu	15	43649123	43649280
ENSG0000014680	-0.171936919	0.136	0.24	TMEM168	protein_coding	transmembr	7	112762377	112790423
ENSG0000011968	0.172176701	0.136	0.24	ABCD4	protein_coding	ATP binding	14	74285269	74303055
ENSG0000013652	0.137296267	0.136	0.24	MRPL47	protein_coding	mitochondr	3	179588285	179604649
ENSG0000021264	-1.448864604	0.136	0.24	ZRSR2P1	transcribed_pr	ZRSR2 pseu	5	112891610	112893079
ENSG0000013610	0.215430873	0.136	0.24	RNASEH2B	protein_coding	ribonuclease	13	50909747	51024120
ENSG0000021145	0.152604111	0.136	0.24	SELENOH	protein_coding	selenoprote	11	57741250	57743554
ENSG0000027829	-0.867591542	0.136	0.24	TBC1D3C	protein_coding	TBC1 doma	17	38057693	38068592
ENSG0000025470	-0.500611177	0.136	0.24	AC138866.2	unprocessed_p	glucuronida	5	70197255	70207745
ENSG0000026111	1.460367211	0.136	0.24	AC092123.1	lncRNA	novel transc	16	89492017	89504460
ENSG0000027069	-0.299959637	0.136	0.24	AC005034.3	lncRNA	novel transc	2	75660462	75662208
ENSG0000024089	0.819298404	0.137	0.24	PLCXD2	protein_coding	phosphatidy	3	111674676	111846447
ENSG0000020635	0.365985524	0.137	0.24	NELFE	protein_coding	negative elo	CHR_HSCHR	31942469	31949469
ENSG0000026115	0.806942776	0.137	0.24	AC112484.3	lncRNA	novel transc	3	128859716	128860526
ENSG0000018311	-0.539148481	0.137	0.24	ARHGEF37	protein_coding	Rho guanine	5	149551947	149634968
ENSG0000026942	1.132171098	0.137	0.24	AC104521.1	lncRNA	novel transc	19	4339577	4343491
ENSG0000010032	0.179658858	0.137	0.24	TAB1	protein_coding	TGF-beta ac	22	39399778	39437060
ENSG0000026704	-1.718990551	0.137	0.24	AC092143.3	lncRNA	novel transc	16	89906157	89918233
ENSG0000023711	-1.557018411	0.137	0.24	CYP2F2P	transcribed_pr	cytochrome	19	40818414	40826772
ENSG0000027126	-1.654850855	0.137	0.24	AL355297.2	lncRNA	novel transc	6	156774217	156774662
ENSG0000013111	-0.192438084	0.137	0.24	ZNF227	protein_coding	zinc finger p	19	44207547	44237268
ENSG0000026061	0.796727565	0.137	0.24	AC025917.1	lncRNA	novel transc	15	52577842	52598709

ENSG0000015270	-1.369986083	0.137	0.24	CATSPER3	protein_coding	cation chan	5	134967907	135011696
ENSG0000010303	-0.111805562	0.137	0.24	NDRG4	protein_coding	NDRG famil	16	58462846	58513628
ENSG0000013041	0.094283933	0.137	0.24	NDUFA10	protein_coding	NADH:ubiqu	2	239892450	240025345
ENSG0000016257	-0.557182985	0.138	0.24	MXRA8	protein_coding	matrix remo	1	1352689	1361777
ENSG0000016920	-0.263642779	0.138	0.24	NPIPB12	protein_coding	nuclear pore	16	29483642	29505999
ENSG0000020734	1.846205103	0.138	0.24	AL133238.1	misc_RNA		14	88840165	88840263
ENSG0000023380	-1.63614911	0.138	0.24	LINC01237	lncRNA	long interge	2	241881363	242078722
ENSG0000023282	-0.308768606	0.138	0.24	AC003986.2	lncRNA	novel transc	7	19112474	19114271
ENSG0000026551	-2.050768325	0.138	0.24	AC020558.2	lncRNA	novel transc	17	17507351	17508308
ENSG0000006076	0.157055716	0.138	0.24	MPC1	protein_coding	mitochondr	6	166364919	166383013
ENSG0000017908	0.37557786	0.138	0.24	DPM3	protein_coding	dolichyl-pho	1	155139891	155140595
ENSG0000025677	-0.146454625	0.138	0.24	ZNF253	protein_coding	zinc finger p	19	19865882	19894674
ENSG0000011996	-0.207232065	0.138	0.24	C10orf88	protein_coding	chromosom	10	122930901	122954311
ENSG0000014185	0.142553075	0.138	0.24	SAMD1	protein_coding	sterile alpha	19	14087851	14091036
ENSG0000023066	-0.290557238	0.138	0.24	HCG18	lncRNA	HLA comple	CHR_HSCHR	30335445	30371782
ENSG0000018526	0.623375525	0.138	0.24	CDNF	protein_coding	cerebral do	10	14819245	14838575
ENSG0000017615	0.172679246	0.139	0.25	CCDC57	protein_coding	coiled-coil c	17	82101460	82212830
ENSG0000016297	-0.251691622	0.139	0.25	MAIP1	protein_coding	matrix AAA	2	199955317	200008540
ENSG0000022408	-1.820325397	0.139	0.25	UBE2FP1	processed_pse	UBE2F pseu	3	37143512	37143958
ENSG0000012069	0.197343722	0.139	0.25	ELF1	protein_coding	E74 like ETS	13	40931924	41061440
ENSG0000014074	0.175349913	0.139	0.25	CDR2	protein_coding	cerebellar d	16	22345936	22437165
ENSG0000019701	0.259908253	0.139	0.25	ZNF429	protein_coding	zinc finger p	19	21496682	21556270
ENSG0000013592	-0.725750573	0.139	0.25	CYP27A1	protein_coding	cytochrome	2	218781749	218815293
ENSG0000006897	0.877011905	0.139	0.25	PYGM	protein_coding	glycogen ph	11	64746389	64759974
ENSG0000017444	0.243080883	0.139	0.25	SNAPC5	protein_coding	small nuclea	15	66490135	66497780
ENSG0000017031	0.408527009	0.139	0.25	STX8	protein_coding	syntaxin 8 [S	17	9250471	9576591
ENSG0000019879	-0.148645782	0.139	0.25	LRIG2	protein_coding	leucine rich	1	113073198	113132260
ENSG0000018819	0.520560196	0.14	0.25	NUTM2B	protein_coding	NUT family	10	79703227	79714681
ENSG0000027856	-0.375573923	0.14	0.25	AC243922.1	transcribed_un	glucuronida	CHR_HSCHR	70543654	70549533
ENSG0000016632	-0.501800243	0.14	0.25	NUDT13	protein_coding	nudix hydro	10	73110375	73131828
ENSG0000010423	-0.137995437	0.14	0.25	ZFAND1	protein_coding	zinc finger A	8	81701334	81732903
ENSG0000013919	-2.140693488	0.14	0.25	RBP5	protein_coding	retinol bindi	12	7115736	7128889
ENSG0000017087	0.123877172	0.14	0.25	MTSS1	protein_coding	MTSS I-BAR	8	124550784	124728473
ENSG0000014002	-0.350831557	0.141	0.25	STON2	protein_coding	stonin 2 [So	14	81260656	81436465
ENSG0000023277	0.460005357	0.141	0.25	BMS1P22	transcribed_un	BMS1 pseud	22	15805263	15820884
ENSG0000010387	0.616860875	0.141	0.25	FAH	protein_coding	fumarylacet	15	80152490	80186946
ENSG0000020547	0.135909264	0.141	0.25	CCDC85C	protein_coding	coiled-coil c	14	99500190	99604207
ENSG0000017122	-0.410304274	0.141	0.25	FAM241B	protein_coding	family with s	10	69630247	69633596
ENSG0000025463	-0.464235457	0.141	0.25	SMG1P6	unprocessed_p	SMG1 pseud	16	29425800	29447026
ENSG0000026332	0.29552199	0.141	0.25	HCN3	protein_coding	hyperpolariz	CHR_HSCHR	155292987	155305252
ENSG0000020626	0.980524794	0.141	0.25	NELFE	protein_coding	negative elo	CHR_HSCHR	31934274	31941274
ENSG0000018795	0.196767439	0.141	0.25	AC091057.1	lncRNA	OTU deubic	15	30658717	30772993
ENSG0000016155	0.351324287	0.141	0.25	ZNF577	protein_coding	zinc finger p	19	51804816	51890950
ENSG0000007405	0.076801735	0.141	0.25	CLASP1	protein_coding	cytoplasmic	2	121337776	121649476
ENSG0000013880	-0.122367762	0.142	0.25	SEC24B	protein_coding	SEC24 hom	4	109433772	109540896
ENSG0000027515	-0.961069159	0.142	0.25	TBC1D3K	protein_coding	TBC1 doma	CHR_HSCHR	37928493	37939442
ENSG0000010878	0.620585945	0.142	0.25	HSD17B1P1	unprocessed_p	hydroxyster	17	42546764	42548706
ENSG0000015857	1.258188138	0.142	0.25	PFKFB1	protein_coding	6-phosphof	X	54932961	54998534
ENSG0000022846	-0.582678262	0.142	0.25	AP006222.1	transcribed_pr	ribosomal p	1	257864	359681
ENSG0000013606	0.110843767	0.142	0.25	FLNB	protein_coding	filamin B [So	3	58008400	58172251
ENSG0000027461	-0.697435881	0.142	0.25	TBC1D3	protein_coding	TBC1 doma	17	38181659	38192541
ENSG0000015077	-0.100598766	0.142	0.25	NKAPD1	protein_coding	NKAP doma	11	112074086	112085150
ENSG0000011154	0.093812706	0.142	0.25	RAB5B	protein_coding	RAB5B, mer	12	55973913	55996683
ENSG0000024336	-0.695695314	0.142	0.25	EFNA4	protein_coding	ephrin A4 [S	1	155063737	155069553
ENSG0000026192	-3.326973811	0.142	0.25	AC127496.1	lncRNA	novel transc	17	80966239	80971213
ENSG0000018505	-0.432145124	0.142	0.25	SGCZ	protein_coding	sarcoglycan	8	14084845	15238431
ENSG0000022537	-0.532693324	0.142	0.25	NRSN2-AS	lncRNA	NRSN2 antis	20	316860	348490
ENSG0000023788	-0.713864823	0.142	0.25	DGUOK-AS	lncRNA	DGUOK ant	2	73947322	73981441

ENSG0000017041	-3.122064441	0.143	0.25	GPRC5C	protein_coding	G protein-c		17	74424851	74451653
ENSG0000025061	-0.790132888	0.143	0.25	AC012645.1	lncRNA	novel transc		16	30096430	30104116
ENSG0000026806	-1.722745744	0.143	0.25	AC004466.1	lncRNA	novel transc		12	47784923	47786002
ENSG0000010012	0.182008949	0.143	0.25	ANKRD54	protein_coding	ankyrin repe		22	37830855	37849327
ENSG0000010570	-0.128171921	0.143	0.25	KXD1	protein_coding	KxDL motif		19	18557762	18569387
ENSG0000022773	0.837542632	0.143	0.25	AC239809.3	lncRNA	novel transc		1	148159213	148255012
ENSG0000028004	-0.880356474	0.143	0.25	AC091825.1	TEC	TEC		5	142165767	142168387
ENSG0000010525	0.175032115	0.143	0.25	TBCB	protein_coding	tubulin foldi		19	36114289	36125947
ENSG0000011688	1.141329861	0.143	0.25	NA	NA	NA	NA	NA	NA	
ENSG0000022617	0.755840362	0.144	0.25	TEX22	protein_coding	testis expres		14	105398538	105450106
ENSG0000010215	0.09804981	0.144	0.25	MAGT1	protein_coding	magnesium	X		77825747	77895593
ENSG0000017294	-0.145680916	0.144	0.25	PHF8	protein_coding	PHD finger	X		53936676	54048958
ENSG0000023278	-2.454804632	0.144	0.25	NA	NA	NA	NA	NA	NA	
ENSG0000015133	0.197598631	0.144	0.25	MBIP	protein_coding	MAP3K12 b		14	36298564	36320637
ENSG0000019681	-0.281210765	0.144	0.25	ZSCAN16	protein_coding	zinc finger a		6	28124609	28130082
ENSG0000023685	0.287596745	0.144	0.25	NA	NA	NA	NA	NA	NA	
ENSG0000023810	-0.214960765	0.144	0.25	PPP1R10	protein_coding	protein pho	CHR_HSCHR		30592623	30610835
ENSG0000009112	0.124667745	0.144	0.25	PUS7	protein_coding	pseudouridi		7	105439661	105522271
ENSG0000023273	1.574888602	0.144	0.25	AC097717.1	lncRNA	novel transc		2	199867396	199911159
ENSG0000008790	0.23944657	0.144	0.25	RFX2	protein_coding	regulatory f		19	5993164	6199572
ENSG0000016658	-0.12794518	0.144	0.25	CENPV	protein_coding	centromere		17	16342534	16353656
ENSG0000024901	2.892559841	0.144	0.25	HMGN2P4	processed_pse	high mobilit		5	76242024	76242404
ENSG0000016777	-0.117208304	0.144	0.25	OTUB1	protein_coding	OTU deubic		11	63985853	64001811
ENSG0000015802	-0.744381236	0.144	0.25	CFAP251	protein_coding	cilia and flag		12	121918592	122003927
ENSG0000021015	-0.419246374	0.144	0.25	MT-TK	Mt_tRNA	mitochondr	MT		8295	8364
ENSG0000008363	-0.264229135	0.145	0.25	NUFIP1	protein_coding	nuclear FMF		13	44939249	44989471
ENSG0000027876	-1.348783055	0.145	0.25	AC004477.2	lncRNA	novel transc		17	48066704	48067293
ENSG0000011265	-0.174353368	0.145	0.25	CUL9	protein_coding	cullin 9 [Sou		6	43182184	43224587
ENSG0000012919	-1.125149129	0.145	0.25	SOX15	protein_coding	SRY-box tra		17	7588178	7590094
ENSG0000010537	-0.914243551	0.145	0.25	ICAM5	protein_coding	intercellular		19	10289952	10296778
ENSG0000014895	0.326594842	0.145	0.25	IMMP1L	protein_coding	inner mitocl		11	31432401	31509645
ENSG0000010021	0.098851965	0.145	0.25	TOMM22	protein_coding	translocase		22	38681957	38685421
ENSG0000010342	-0.189893625	0.145	0.25	DNAJA3	protein_coding	DnaJ heat sl		16	4425805	4456775
ENSG0000025502	-1.665796302	0.145	0.26	AP000842.2	lncRNA	novel transc		11	125903247	125938916
ENSG0000012565	-1.406444163	0.145	0.26	TNFSF9	protein_coding	TNF superfa		19	6531026	6535924
ENSG0000023317	1.064049531	0.146	0.26	AC138356.2	processed_pse	chromosom		8	37747338	37748004
ENSG0000020786	-1.457921612	0.146	0.26	MIR27B	miRNA	microRNA 2		9	95085445	95085541
ENSG0000021473	1.058410103	0.146	0.26	TOMM6	protein_coding	translocase		6	41787662	41789898
ENSG0000018222	-0.442899603	0.146	0.26	ZAR1	protein_coding	zygote arres		4	48490252	48494389
ENSG0000016800	0.12994134	0.146	0.26	SPINDOC	protein_coding	spindlin inte		11	63813456	63827716
ENSG0000016770	-0.671435608	0.146	0.26	SLC43A2	protein_coding	solute carrie		17	1569268	1628886
ENSG0000015695	0.255303487	0.146	0.26	LHFPL4	protein_coding	LHFPL tetra:		3	9498361	9553822
ENSG0000014658	-0.136270535	0.146	0.26	RBAK	protein_coding	RB associate		7	5045821	5069487
ENSG0000026341	0.632771384	0.146	0.26	NFE2L1-DT	lncRNA	NFE2L1 dive		17	48045141	48048073
ENSG0000023463	1.552314068	0.146	0.26	AC245060.2	lncRNA	novel transc		22	22293733	22294794
ENSG0000019718	0.292126332	0.146	0.26	MIRLET7BH	lncRNA	MIRLET7B h		22	46053869	46113928
ENSG0000011740	-1.340487317	0.146	0.26	ARTN	protein_coding	artemin [So		1	43933320	43937240
ENSG0000025029	-0.458905645	0.147	0.26	MRPS31P4	transcribed_un	mitochondr		13	52600119	52637446
ENSG0000013589	-0.701980557	0.147	0.26	SP110	protein_coding	SP110 nucle		2	230165186	230225729
ENSG0000013971	-0.141614243	0.147	0.26	SETD1B	protein_coding	SET domain		12	121804180	121832584
ENSG0000018689	0.248232716	0.147	0.26	C1QL4	protein_coding	complemen		12	49332409	49337188
ENSG0000024340	-0.286032115	0.147	0.26	MRPS31P5	transcribed_un	mitochondr		13	52167709	52194465
ENSG0000013961	0.100873012	0.147	0.26	SMARCC2	protein_coding	SWI/SNF rel		12	56162359	56189567
ENSG0000027953	-0.670965478	0.147	0.26	AC092881.1	TEC	TEC		12	70321542	70324274
ENSG0000014381	0.140417032	0.147	0.26	PYCR2	protein_coding	pyrroline-5-		1	225919877	225924340
ENSG0000020557	-0.407809342	0.147	0.26	POM121B	unprocessed_p	POM121 tra		7	73293497	73301161
ENSG0000026106	1.387642026	0.147	0.26	LINC02256	lncRNA	long interge		15	32536047	32587613
ENSG0000016971	1.698001306	0.147	0.26	MT1E	protein_coding	metallothior		16	56625475	56627112

ENSG0000018362	-0.366532943	0.147	0.26	ZNF438	protein_coding	zinc finger p	10	30820207	31031937
ENSG0000007570	0.22055192	0.147	0.26	WDR62	protein_coding	WD repeat d	19	36054881	36105108
ENSG0000011775	0.129986376	0.148	0.26	PPP1R8	protein_coding	protein pho	1	27830782	27851676
ENSG0000015788	0.315940783	0.148	0.26	PANK4	protein_coding	pantothena	1	2508537	2526597
ENSG0000022669	1.723422277	0.148	0.26	LENG8-AS1	lncRNA	LENG8 antis	19	54444807	54449045
ENSG0000024462	-2.473363347	0.148	0.26	AC246787.2	lncRNA	novel transc	14	105890084	105896577
ENSG0000023018	-1.310058326	0.148	0.26	CNOT6LP1	processed_pse	CNOT6L pse	15	56005715	56007176
ENSG0000012255	0.143120459	0.148	0.26	KLHL7	protein_coding	kelch like fa	7	23105758	23177914
ENSG0000027204	1.781559526	0.148	0.26	AC091965.4	lncRNA	novel transc	5	8444949	8445535
ENSG0000024793	0.811841761	0.148	0.26	AC022364.1	lncRNA	novel transc	12	28163298	28190738
ENSG0000014005	0.908894609	0.148	0.26	AK7	protein_coding	adenylate ki	14	96392128	96489427
ENSG0000017142	0.240508335	0.148	0.26	MRPL36	protein_coding	mitochondr	5	1798385	1801366
ENSG0000011474	0.224714845	0.148	0.26	GORASP1	protein_coding	golgi reasse	3	39096659	39108363
ENSG0000016752	-0.064747956	0.148	0.26	RPL13	protein_coding	ribosomal p	16	89560677	89564542
ENSG0000019800	-0.961983881	0.148	0.26	CCDC151	protein_coding	coiled-coil d	19	11420604	11435782
ENSG0000010838	0.862692541	0.148	0.26	SEPTIN4	protein_coding	septin 4 [So	17	58520250	58544368
ENSG0000014967	0.304218386	0.148	0.26	CABLES2	protein_coding	Cdk5 and A	20	62388632	62407285
ENSG0000026077	0.672152034	0.148	0.26	AC021087.3	lncRNA	novel transc	5	213898	217279
ENSG0000026774	1.313009409	0.148	0.26	AC104985.1	lncRNA	novel transc	18	34222965	34224761
ENSG0000026347	-1.081294149	0.149	0.26	NA	NA	NA	NA	NA	NA
ENSG0000011884	-0.927404052	0.149	0.26	RARRES1	protein_coding	retinoic acid	3	158696892	158732489
ENSG0000027533	2.251351886	0.149	0.26	Z99129.1	processed_pse	mitogen-ac	6	122454358	122454612
ENSG0000027790	2.478874713	0.149	0.26	U2	snRNA	U2 spliceoso	17	43271369	43271559
ENSG0000024879	-1.59482377	0.149	0.26	AC010627.1	processed_pse	pleckstrin ho	5	14620020	14621313
ENSG0000013024	-0.468405789	0.149	0.26	FAM98C	protein_coding	family with s	19	38403135	38409088
ENSG0000013614	-0.150061542	0.15	0.26	MED4	protein_coding	mediator co	13	48053323	48095131
ENSG0000027368	-2.55232987	0.15	0.26	AC004223.4	lncRNA	novel transc	17	35018660	35018991
ENSG0000019767	0.957581818	0.15	0.26	AL157838.1	lncRNA	novel transc	20	53552770	53575863
ENSG0000024208	2.118920655	0.15	0.26	RPS20P33	processed_pse	ribosomal p	14	105374618	105374971
ENSG0000026300	-0.759822898	0.15	0.26	ROCK1P1	transcribed_un	Rho associa	18	109065	122219
ENSG0000022971	-0.880355771	0.15	0.26	EEF1DP3	transcribed_un	eukaryotic t	13	31952580	31953361
ENSG0000016544	-0.384340541	0.15	0.26	SLC16A9	protein_coding	solute carrie	10	59650761	59736002
ENSG0000018710	0.842120921	0.15	0.26	HEATR4	protein_coding	HEAT repea	14	73478484	73558947
ENSG0000017147	0.390467828	0.15	0.26	NA	NA	NA	NA	NA	NA
ENSG0000019890	0.069357302	0.15	0.26	TOP1	protein_coding	DNA topois	20	41028822	41124487
ENSG0000016962	-0.285414211	0.151	0.26	RGPD8	protein_coding	RANBP2 like	2	112368369	112434488
ENSG0000010974	-0.976620228	0.151	0.26	BST1	protein_coding	bone marro	4	15703065	15738313
ENSG0000010230	-0.182604686	0.151	0.26	FGD1	protein_coding	FYVE, RhoG	X	54445454	54496234
ENSG0000013593	0.095116662	0.151	0.26	CAB39	protein_coding	calcium bind	2	230712842	230821075
ENSG0000006401	-0.628677962	0.151	0.26	CASP8	protein_coding	caspase 8 [S	2	201233443	201287711
ENSG0000025942	-1.896325174	0.151	0.26	IRAIN	lncRNA	IGF1R antise	15	98646951	98647371
ENSG0000016650	0.284757749	0.151	0.26	NDST2	protein_coding	N-deacetyla	10	73801911	73811798
ENSG0000013874	0.342116305	0.151	0.26	NAAA	protein_coding	N-acylethar	4	75913660	75941013
ENSG0000021530	0.573129881	0.151	0.26	AC127502.1	transcribed_un	WAS protein	15	30470779	30507623
ENSG0000013072	-0.178090299	0.151	0.26	CHMP2A	protein_coding	charged mu	19	58551566	58555105
ENSG0000013296	0.124721906	0.151	0.26	POMP	protein_coding	proteasome	13	28659104	28678959
ENSG0000023560	1.446768048	0.151	0.26	POU5F1P3	processed_pse	POU class 5	12	8133772	8134849
ENSG0000008987	-0.363850017	0.151	0.26	DHX32	protein_coding	DEAH-box h	10	125836337	125896436
ENSG0000010174	0.248506752	0.151	0.26	NOL4	protein_coding	nucleolar pr	18	33851100	34224952
ENSG0000013445	0.106164502	0.151	0.26	FBH1	protein_coding	F-box DNA	10	5890203	5937594
ENSG0000014002	0.374898344	0.151	0.26	EFCAB11	protein_coding	EF-hand cal	14	89794669	89954777
ENSG0000013733	-0.455319969	0.151	0.26	DDR1	protein_coding	discoidin do	CHR_HSCHR	30870863	30890046
ENSG0000019712	-0.075000894	0.152	0.26	PGAP1	protein_coding	post-GPI att	2	196833004	196927796
ENSG0000021308	-1.13834929	0.152	0.26	CFAP45	protein_coding	cilia and flag	1	159872364	159900165
ENSG0000016277	-0.137307248	0.152	0.26	RBM15	protein_coding	RNA binding	1	110338506	110346681
ENSG0000015830	1.614044167	0.152	0.26	GPRASP2	protein_coding	G protein-c	X	102712176	102717733
ENSG0000009696	0.451393748	0.152	0.26	JAK2	protein_coding	Janus kinase	9	4984390	5129948
ENSG0000010580	0.330733385	0.152	0.27	RASA4	protein_coding	RAS p21 pro	7	102573807	102616756

ENSG0000013731	-0.715043976	0.152	0.27	FLOT1	protein_coding	flotillin 1 [Sc	6	30727709	30742732
ENSG0000016752	0.360280685	0.152	0.27	SPATA33	protein_coding	spermatoge	16	89657802	89671272
ENSG0000021351	-0.191640679	0.152	0.27	RBMXL1	protein_coding	RBMX like 1	1	88979456	88992960
ENSG0000015623	-0.261481754	0.153	0.27	WHAMM	protein_coding	WASP homo	15	82809628	82836108
ENSG0000013250	0.071318557	0.153	0.27	EIF5A	protein_coding	eukaryotic t	17	7306999	7312463
ENSG0000014125	0.163943875	0.153	0.27	VPS53	protein_coding	VPS53 subu	17	508668	721717
ENSG0000013059	-0.84565931	0.153	0.27	LSP1	protein_coding	lymphocyte	11	1850904	1892267
ENSG0000017748	-0.103491121	0.153	0.27	ZBTB33	protein_coding	zinc finger a	X	120250752	120258398
ENSG0000013326	-0.241957587	0.153	0.27	HSPBP1	protein_coding	HSPA (Hsp7	19	55262223	55280381
ENSG0000016404	0.195549147	0.153	0.27	CDC25A	protein_coding	cell division	3	48157146	48188417
ENSG0000027720	1.384694329	0.153	0.27	RPPH1	ribozyme	ribonuclease	14	20343075	20343407
ENSG0000015760	0.164509459	0.153	0.27	TMEM164	protein_coding	transmembr	X	110002631	110182734
ENSG0000014139	-0.547532703	0.153	0.27	PRELID3A	protein_coding	PRELI doma	18	12407896	12432238
ENSG0000024749	-0.522540827	0.153	0.27	GPRC5D-A	lncRNA	GPRC5D an	12	12927726	12984645
ENSG0000000046	-0.175469861	0.153	0.27	C1orf112	protein_coding	chromosom	1	169662007	169854080
ENSG0000025552	1.438687766	0.154	0.27	NEDD8-MD	protein_coding	NEDD8-MD	14	24213955	24232352
ENSG0000022761	-0.45490009	0.154	0.27	AP001324.	processed_pse	ribosomal p	11	74745716	74746114
ENSG0000018437	-0.349528859	0.154	0.27	CSF1	protein_coding	colony stim	1	109910242	109930992
ENSG0000023481	-1.027062894	0.154	0.27	SVIL2P	transcribed_un	supervillin fa	10	30655489	30717266
ENSG0000012852	0.128144725	0.154	0.27	ATP6V1F	protein_coding	ATPase H+	7	128862856	128865847
ENSG0000019740	1.29110573	0.154	0.27	H3C4	protein_coding	H3 clustered	6	26196784	26197286
ENSG0000027648	0.366101815	0.154	0.27	NA	NA	NA	NA	NA	NA
ENSG0000012685	-0.162826389	0.154	0.27	RHOT1	protein_coding	ras homolog	17	32142454	32253374
ENSG0000018061	-0.237323621	0.154	0.27	MB21D2	protein_coding	Mab-21 do	3	192796815	192917856
ENSG0000026164	0.927514726	0.154	0.27	AC093849.	lncRNA	novel transc	4	173467833	173470041
ENSG0000013769	-0.676234214	0.154	0.27	YAP1	protein_coding	Yes1 associa	11	102110447	102233424
ENSG0000010714	0.279872816	0.154	0.27	TESK1	protein_coding	testis associ	9	35605262	35610041
ENSG0000026871	-0.838112177	0.154	0.27	AC005261.	lncRNA	novel transc	19	57261354	57262738
ENSG0000018496	0.215383459	0.154	0.27	NOC4L	protein_coding	nucleolar co	12	132144457	132152473
ENSG0000010273	-0.206320596	0.154	0.27	MRPS31	protein_coding	mitochondr	13	40729128	40771190
ENSG0000013919	1.366512002	0.154	0.27	CD27	protein_coding	CD27 molec	12	6444867	6451718
ENSG0000026449	-1.87044471	0.155	0.27	AC005544.	lncRNA	novel transc	17	67019934	67021743
ENSG0000023997	0.962987584	0.155	0.27	EGFL8	protein_coding	EGF like dor	CHR_HSCHR	32172254	32175953
ENSG0000027256	0.324810427	0.155	0.27	NA	NA	NA	NA	NA	NA
ENSG0000003827	-0.110999198	0.155	0.27	MAT2B	protein_coding	methionine	5	163503114	163519354
ENSG0000023988	-0.234183802	0.155	0.27	PARGP1	transcribed_un	poly(ADP-ri	10	45854093	45972154
ENSG0000012196	-0.539433732	0.155	0.27	CXCR4	protein_coding	C-X-C moti	2	136114349	136118149
ENSG0000018251	0.258457546	0.155	0.27	FAM104B	protein_coding	family with s	X	55143102	55161310
ENSG0000010540	-0.088115637	0.155	0.27	ATP1A3	protein_coding	ATPase Na+	19	41966582	41997497
ENSG0000023361	-2.911658833	0.155	0.27	AC006328.	processed_pse	novel pseuc	Y	25487284	25487662
ENSG0000011364	-0.075031867	0.155	0.27	TCERG1	protein_coding	transcription	5	146447311	146511961
ENSG0000015296	-0.283536105	0.155	0.27	JAKMIP1	protein_coding	janus kinase	4	6026199	6200591
ENSG0000009961	-0.940456108	0.155	0.27	EFNA2	protein_coding	ephrin A2 [S	19	1285873	1301431
ENSG0000022627	0.689125351	0.155	0.27	PSPHP1	unprocessed_p	phosphoser	7	55764797	55773288
ENSG0000027866	0.355568071	0.156	0.27	AC106795.	transcribed_un	THO comple	CHR_HSCHR	177875522	177950732
ENSG0000023622	-0.24705589	0.156	0.27	WDR46	protein_coding	WD repeat c	CHR_HSCHR	33449481	33459870
ENSG0000026993	-0.234201857	0.156	0.27	AC093525.	lncRNA	novel transc	16	2561471	2565096
ENSG0000023068	1.006835117	0.156	0.27	CLIC1	protein_coding	chloride intr	CHR_HSCHR	31717988	31727162
ENSG0000022706	-0.453526284	0.156	0.27	RPL41P1	processed_pse	ribosomal p	20	21755270	21755350
ENSG0000025490	1.000390364	0.156	0.27	AP003392.	processed_pse	potassium c	11	119005727	119005934
ENSG0000026540	1.103794117	0.156	0.27	AC093484.	lncRNA	novel transc	17	16382152	16382669
ENSG0000027988	-1.162957896	0.156	0.27	AC134407.	TEC	TEC	17	67973934	67976072
ENSG0000026528	-1.85516094	0.156	0.27	NA	NA	NA	NA	NA	NA
ENSG0000025105	-1.651415767	0.156	0.27	RBX1P2	processed_pse	RBX1 pseud	5	80019609	80019920
ENSG0000011180	0.116187465	0.157	0.27	TDP2	protein_coding	tyrosyl-DNA	6	24649979	24666930
ENSG0000025073	1.627348757	0.157	0.27	TPM3P6	processed_pse	tropomyosin	19	53479350	53480091
ENSG0000026056	0.621457908	0.157	0.27	AC127459.	lncRNA	novel transc	16	23061406	23064173
ENSG0000017670	0.255417351	0.157	0.27	SCAND2P	transcribed_un	SCAN doma	15	84631451	84647478

ENSG0000022828	0.781699482	0.157	0.27	KATNBL1P6	transcribed_pr	katanin regu	6	146802359	146803824
ENSG0000016348	0.216962889	0.157	0.27	RNF25	protein_coding	ring finger p	2	218663892	218672002
ENSG0000013227	-0.192223573	0.158	0.27	RRP8	protein_coding	ribosomal R	11	6595072	6603616
ENSG0000008933	-0.140850516	0.158	0.27	ZNF302	protein_coding	zinc finger p	19	34677639	34686397
ENSG0000025461	0.340400527	0.158	0.27	DNAJB6P1	processed_pse	DNAJB6 pse	11	127940913	127941654
ENSG0000016823	-0.759335458	0.158	0.27	GLYCTK	protein_coding	glycerate ki	3	52287089	52295257
ENSG0000027744	-0.977469912	0.158	0.27	CPEB1	protein_coding	cytoplasmic	CHR_HSCHR	82622289	82720771
ENSG0000019705	-0.246520834	0.158	0.27	ZNF420	protein_coding	zinc finger p	19	37007857	37130368
ENSG0000011747	-0.143661353	0.158	0.27	BLZF1	protein_coding	basic leucin	1	169367970	169396540
ENSG0000019874	0.167506522	0.158	0.27	SMURF1	protein_coding	SMAD speci	7	99027438	99144100
ENSG0000018854	0.355147697	0.158	0.27	DUSP28	protein_coding	dual specific	2	240560054	240565256
ENSG0000017197	0.472289025	0.158	0.27	ZNF57	protein_coding	zinc finger p	19	2900898	2918473
ENSG0000017711	0.072531846	0.158	0.27	ANO6	protein_coding	anoctamin 6	12	45215987	45440404
ENSG0000020497	-0.138188899	0.159	0.27	TRIM13	protein_coding	tripartite mo	13	49995888	50020481
ENSG0000023188	3.448494746	0.159	0.27	NDUFB1P1	processed_pse	NADH:ubiqu	3	48181547	48181726
ENSG0000008372	-0.1228848	0.159	0.27	OXCT1	protein_coding	3-oxoacid C	5	41730065	41870425
ENSG0000026620	-0.584380517	0.159	0.27	AC080112.1	lncRNA	novel transc	17	40360655	40364693
ENSG0000017798	0.169449015	0.159	0.27	ASB8	protein_coding	ankyrin repe	12	48147789	48181213
ENSG0000021258	1.470997697	0.159	0.27	SNORA26	snoRNA	small nucleo	4	52713249	52713370
ENSG0000001145	0.096361339	0.159	0.27	RABGAP1	protein_coding	RAB GTPase	9	122940833	123104866
ENSG0000017605	-0.17512451	0.159	0.27	MBLAC2	protein_coding	metallo-bet	5	90458209	90474771
ENSG0000012752	-0.1121703	0.159	0.27	SLC35E1	protein_coding	solute carrie	19	16549831	16572415
ENSG0000009995	-0.636399897	0.159	0.27	P2RX6	protein_coding	purinergic r	22	21009808	21028830
ENSG0000016480	0.154790786	0.159	0.27	SPIDR	protein_coding	scaffold pro	8	47260878	47736306
ENSG0000025160	-2.455342512	0.159	0.28	NA	NA	NA	NA	NA	NA
ENSG0000023479	-0.41253207	0.159	0.28	RPS3AP6	processed_pse	RPS3A pseu	15	59768352	59769146
ENSG0000013518	-0.2423568	0.159	0.28	TMEM243	protein_coding	transmembr	7	87196160	87220587
ENSG0000015575	-0.108293964	0.159	0.28	TMEM237	protein_coding	transmembr	2	201620184	201643570
ENSG0000027764	0.490194441	0.159	0.28	WNT3	protein_coding	Wnt family r	CHR_HSCHR	46591415	46662074
ENSG0000023272	0.625379121	0.16	0.28	YWHAEP1	processed_pse	tyrosine 3-r	7	64433830	64434592
ENSG0000025155	-1.863785235	0.16	0.28	AC091887.1	lncRNA	novel transc	5	146099406	146120412
ENSG0000010291	0.102332989	0.16	0.28	LONP2	protein_coding	lon peptidas	16	48244300	48363122
ENSG0000012860	-0.261780456	0.16	0.28	SMO	protein_coding	smoothened	7	129188633	129213545
ENSG0000000466	-0.306772869	0.16	0.28	CAMKK1	protein_coding	calcium/calr	17	3860315	3894891
ENSG0000013068	-0.205248571	0.16	0.28	ZNF337	protein_coding	zinc finger p	20	25673195	25696853
ENSG0000023228	-1.568743441	0.16	0.28	GNG12-AS	lncRNA	GNG12, DIR	1	67832303	68202987
ENSG0000018522	-0.124213873	0.16	0.28	TCEAL9	protein_coding	transcription	X	103356489	103358462
ENSG0000022222	3.430572946	0.16	0.28	RNU2-17P	snRNA	RNA, U2 sm	1	150236967	150237156
ENSG0000023815	3.430572946	0.16	0.28	MLLT10P1	processed_pse	MLLT10 pse	20	30403123	30403384
ENSG0000010029	0.104556495	0.16	0.28	MCM5	protein_coding	minichromo	22	35400134	35425431
ENSG0000016498	-0.288607617	0.16	0.28	CCDC171	protein_coding	coiled-coil c	9	15553043	16061663
ENSG0000022485	-1.043418748	0.161	0.28	ZNRD1	protein_coding	zinc ribbon	CHR_HSCHR	30048795	30054799
ENSG0000019686	0.932842729	0.161	0.28	H2AC7	protein_coding	H2A cluster	6	26198784	26199293
ENSG0000012368	-1.303315897	0.161	0.28	BATF3	protein_coding	basic leucin	1	212686417	212699840
ENSG0000022418	0.606440499	0.161	0.28	CCHCR1	protein_coding	coiled-coil a	CHR_HSCHR	31220974	31236769
ENSG0000014255	0.209503937	0.161	0.28	ZNF614	protein_coding	zinc finger p	19	52012765	52030240
ENSG0000010600	0.1574043	0.161	0.28	BRAT1	protein_coding	BRCA1 asso	7	2537810	2555694
ENSG0000012005	0.105153106	0.161	0.28	GOT1	protein_coding	glutamic-ox	10	99396870	99430624
ENSG0000017362	-0.209779671	0.161	0.28	LRFN4	protein_coding	leucine rich	11	66856647	66860475
ENSG0000019886	-0.199074193	0.161	0.28	RUNDC1	protein_coding	RUN domai	17	42980565	42993690
ENSG0000016030	-0.133080081	0.161	0.28	DIP2A	protein_coding	disco intera	21	46458891	46570015
ENSG0000025968	-1.645882192	0.161	0.28	AC243562.2	unprocessed_p	golgin A6 fa	15	84389729	84395903
ENSG0000020492	-0.368035364	0.161	0.28	ZNF155	protein_coding	zinc finger p	19	43967862	43998326
ENSG0000026064	0.716456534	0.162	0.28	AC114811.2	lncRNA	novel transc	4	98658904	98664550
ENSG0000001217	0.78954093	0.162	0.28	SEMA3B	protein_coding	semaphorin	3	50267558	50277546
ENSG0000011603	0.408138287	0.162	0.28	ATP6V1B1	protein_coding	ATPase H+	2	70935900	70965431
ENSG0000019756	-0.280407861	0.162	0.28	PIGN	protein_coding	phosphatidy	18	61905255	62187118
ENSG0000016206	0.203978346	0.162	0.28	AMDHD2	protein_coding	amidohydro	16	2520357	2531422

ENSG0000027387	-0.128259508	0.162	0.28	BDP1	protein_coding	B double pr	CHR_HSCHR		70670918	70783123
ENSG0000022509	-2.482562971	0.162	0.28	AL445250.1	lncRNA	novel transc		6	57961438	58438364
ENSG0000018075	-0.683787606	0.162	0.28	GPR157	protein_coding	G protein-c		1	9100305	9129102
ENSG0000026112	-0.593577298	0.162	0.28	RBFADN	lncRNA	RBFA downs		18	80046900	80095482
ENSG0000019627	-0.621309368	0.162	0.28	GRM7	protein_coding	glutamate m		3	6770001	7741533
ENSG0000027359	1.300084487	0.162	0.28	AC211486.3	transcribed_un	postmeiotic		7	75316048	75327765
ENSG0000007620	0.204781634	0.163	0.28	PTPN23	protein_coding	protein tyro		3	47381011	47413435
ENSG0000024099	1.625685834	0.163	0.28	AL157400.4	lncRNA	novel transc		10	89694295	89697928
ENSG0000008536	0.085819563	0.163	0.28	SCAMP1	protein_coding	secretory ca		5	78360611	78480739
ENSG0000008888	0.136274878	0.163	0.28	CPXM1	protein_coding	carboxypep		20	2794074	2800627
ENSG0000023828	1.444309761	0.163	0.28	AL603839.3	lncRNA	novel transc		1	40493157	40508661
ENSG0000018760	-1.292534423	0.163	0.28	ISG15	protein_coding	ISG15 ubiqu		1	1001138	1014540
ENSG0000023030	-0.544880906	0.163	0.28	TRIM39	protein_coding	tripartite mo	CHR_HSCHR		30390376	30407632
ENSG0000027532	1.126799503	0.163	0.28	AL138781.1	lncRNA	novel transc		9	136122521	136124363
ENSG0000011071	0.072845014	0.163	0.28	NUP98	protein_coding	nucleoporin		11	3671083	3797792
ENSG0000016692	-0.164209344	0.163	0.28	TSC22D4	protein_coding	TSC22 dom		7	100463359	100479232
ENSG0000017871	-0.346460556	0.163	0.28	RPP25	protein_coding	ribonucleas		15	74954418	74956772
ENSG0000007146	0.12281714	0.163	0.28	BUD23	protein_coding	BUD23 rRNA		7	73683025	73705161
ENSG0000025929	0.340519552	0.164	0.28	CSPG4P12	transcribed_un	chondroitin		15	85191438	85213905
ENSG0000019638	-0.634833933	0.164	0.28	ZNF781	protein_coding	zinc finger p		19	37667751	37692315
ENSG0000025874	0.875358569	0.164	0.28	H2AZ2P1	transcribed_pr	H2AZ2 pseu		15	92713477	92734205
ENSG0000010041	-0.14074373	0.164	0.28	TRMU	protein_coding	tRNA 5-met		22	46330875	46357340
ENSG0000016981	0.334469939	0.164	0.28	BTD	protein_coding	biotinidase		3	15601341	15722311
ENSG0000026306	0.903536772	0.164	0.28	RNF213-AS	lncRNA	RNF213 ant		17	80351828	80415168
ENSG0000027362	1.883740399	0.164	0.28	NA	NA	NA	NA		NA	NA
ENSG0000020512	-1.612816235	0.165	0.28	C4orf47	protein_coding	chromosom		4	185426249	185449826
ENSG0000012981	0.235251344	0.165	0.28	SGO1	protein_coding	shugoshin 1		3	20160593	20186206
ENSG0000016233	-0.110351907	0.165	0.28	LRP5	protein_coding	LDL recepto		11	68312591	68449275
ENSG0000023098	-0.079868338	0.165	0.28	HSBP1	protein_coding	heat shock f		16	83807978	83819737
ENSG0000026764	-0.776939466	0.165	0.28	AC016582.3	transcribed_un	novel zinc fi		19	37817359	37832160
ENSG0000017680	-0.308544448	0.165	0.28	LRRC37A3	protein_coding	leucine rich		17	64854312	64919480
ENSG0000020682	3.420544319	0.165	0.28	RNVU1-30	snRNA	RNA, varian		1	149636766	149636929
ENSG0000012000	0.093587145	0.165	0.28	WDR11	protein_coding	WD repeat d		10	120851305	120909524
ENSG0000016645	0.098036105	0.165	0.28	ATMIN	protein_coding	ATM interac		16	81035842	81047350
ENSG0000024892	1.314747397	0.165	0.28	AC021087.1	lncRNA	novel transc		5	269858	271516
ENSG0000010143	-0.196002766	0.165	0.28	CST3	protein_coding	cystatin C [S		20	23626706	23638473
ENSG0000025136	-0.456052354	0.165	0.28	AC107884.1	lncRNA	novel transc		11	7418826	7513644
ENSG0000000716	-0.073245918	0.165	0.28	PAFAH1B1	protein_coding	platelet acti		17	2593210	2685615
ENSG0000018061	-0.186427961	0.166	0.28	SSTR2	protein_coding	somatostati		17	73165010	73176633
ENSG0000010779	-0.105923024	0.166	0.28	LIPA	protein_coding	lipase A, lys		10	89213569	89414557
ENSG0000024974	-0.791780136	0.166	0.28	AC093890.1	processed_pse	salvador ho		4	143911514	143912053
ENSG0000007804	0.211123082	0.166	0.28	PIAS2	protein_coding	protein inhil		18	46803218	46920160
ENSG0000023986	-0.411999665	0.166	0.28	RPP21	protein_coding	ribonucleas	CHR_HSCHR		30373944	30391525
ENSG0000026269	1.614916222	0.166	0.28	AC116914.2	lncRNA	novel transc		17	3721628	3722488
ENSG0000026435	-1.437617522	0.166	0.28	NEK4P2	processed_pse	NIMA-relate		17	16666107	16667118
ENSG0000026774	0.551915042	0.166	0.28	AC024592.3	protein_coding	novel protei		19	5866171	5903787
ENSG0000027720	-0.284385564	0.166	0.28	AC005696.4	lncRNA	novel transc		17	2720801	2723947
ENSG0000026767	-0.289516017	0.166	0.28	FDX2	protein_coding	ferredoxin 2		19	10310045	10316015
ENSG0000020419	0.308788059	0.166	0.29	RPL12P16	processed_pse	ribosomal p		2	203190780	203191277
ENSG0000026732	-0.384604278	0.166	0.29	SNHG30	lncRNA	small nucleo		17	35568076	35574900
ENSG0000000808	0.179143223	0.167	0.29	CDKL5	protein_coding	cyclin deper	X		18425583	18653629
ENSG0000026693	1.226279763	0.167	0.29	AC005746.1	lncRNA	novel transc		17	61382785	61384680
ENSG0000023970	-2.306935919	0.167	0.29	CDRT4	protein_coding	CMT1A dup		17	15436015	15503608
ENSG0000010660	0.175260485	0.167	0.29	BLVRA	protein_coding	biliverdin re		7	43758680	43807342
ENSG0000022201	0.408961079	0.167	0.29	FAM185A	protein_coding	family with s		7	102748971	102809225
ENSG0000016891	-0.448731115	0.167	0.29	ENHO	protein_coding	energy hom		9	34521043	34522990
ENSG0000024008	-0.919167456	0.167	0.29	RPSAP12	processed_pse	ribosomal p		12	68552995	68553882
ENSG0000012177	-0.219616638	0.168	0.29	TMEM39B	protein_coding	transmembr		1	32072031	32102866

ENSG0000027714	-0.261666392	0.168	0.29	TYW1B	protein_coding	tRNA-yW sy	7	72558744	72828200
ENSG0000010781	-0.166955903	0.168	0.29	SFXN3	protein_coding	sideroflexin	10	101031234	101041244
ENSG0000015704	-0.320412249	0.168	0.29	NTAN1	protein_coding	N-terminal	16	15037854	15056079
ENSG0000021433	0.937310327	0.168	0.29	SOGA3	protein_coding	SOGA family	6	127472794	127519191
ENSG0000025459	1.432905155	0.168	0.29	OR7E126P	unprocessed_p	olfactory rec	11	71903194	71904081
ENSG0000021418	1.196974917	0.168	0.29	GCC2-AS1	lncRNA	GCC2 antise	2	108507515	108534196
ENSG0000017868	-1.159461431	0.168	0.29	PARP10	protein_coding	poly(ADP-ri	8	143977153	144012772
ENSG0000023946	1.594347422	0.168	0.29	AC007405.3	lncRNA	glutamate r	2	170766878	170778729
ENSG0000013803	0.150554193	0.168	0.29	ADCY3	protein_coding	adenylate cy	2	24819169	24919839
ENSG0000016399	-0.431829784	0.168	0.29	ABLIM2	protein_coding	actin bindin	4	7965310	8158832
ENSG0000027250	1.567263742	0.168	0.29	AC087752.4	lncRNA	novel transc	8	94884609	94885070
ENSG0000017517	0.081550107	0.168	0.29	PPM1E	protein_coding	protein pho	17	58755854	58985179
ENSG0000011221	-0.385424145	0.168	0.29	GPR63	protein_coding	G protein-c	6	96794125	96837477
ENSG0000011473	-0.525205908	0.168	0.29	CISH	protein_coding	cytokine inc	3	50606489	50611774
ENSG0000016387	0.207806235	0.168	0.29	TPRA1	protein_coding	transmembr	3	127571232	127598267
ENSG0000026700	-0.436277191	0.169	0.29	AC060780.1	lncRNA	novel transc	17	43148368	43171037
ENSG0000008473	-0.071591508	0.169	0.29	KIF3C	protein_coding	kinesin fami	2	25926598	25982749
ENSG0000012959	0.810647976	0.169	0.29	CDO1	protein_coding	cysteine dio	5	115804733	115816659
ENSG0000023312	0.907440438	0.169	0.29	CTAGE7P	processed_pse	CTAGE fami	10	130106046	130108481
ENSG0000025844	0.159613195	0.169	0.29	LINC00641	lncRNA	long interge	14	21200079	21206900
ENSG0000010588	-1.390394235	0.169	0.29	STEAP1B	protein_coding	STEAP famil	7	22419444	22727613
ENSG0000026017	-0.438974007	0.169	0.29	AC141586.2	lncRNA	novel transc	16	2644084	2645214
ENSG0000018636	-2.965197061	0.169	0.29	MINAR2	protein_coding	membrane i	5	129748094	129766732
ENSG0000008904	-0.119361008	0.169	0.29	ESF1	protein_coding	ESF1 nuclec	20	13714322	13784886
ENSG0000025696	3.198525241	0.169	0.29	AL513165.2	protein_coding	novel transc	9	37512547	37592469
ENSG0000026671	0.594536658	0.169	0.29	MYO15B	protein_coding	myosin XVB	17	75588058	75626849
ENSG0000025612	0.526483908	0.169	0.29	LINC01152	lncRNA	long interge	17	72030291	72041310
ENSG0000027884	1.682288113	0.169	0.29	AC009878.1	lncRNA	novel transc	15	26557598	26557937
ENSG0000020593	0.902882519	0.169	0.29	C21orf62-A	lncRNA	C21orf62 ar	21	32772100	32955437
ENSG0000027251	-2.179324371	0.169	0.29	AL158211.4	lncRNA	novel transc	10	22332404	22332987
ENSG0000007026	0.187446435	0.169	0.29	TMEM260	protein_coding	transmembr	14	56488354	56650606
ENSG0000021239	2.399319704	0.169	0.29	RNA5SP323	rRNA_pseudog	RNA, 5S ribo	10	93510548	93510680
ENSG0000025908	-1.21831493	0.169	0.29	AF111169.3	lncRNA	novel transc	14	76774284	76781518
ENSG0000011992	-0.215402417	0.169	0.29	CUTC	protein_coding	cutC copper	10	99702558	99756134
ENSG0000025125	-2.360965532	0.17	0.29	AC004069.1	lncRNA	novel transc	4	105137280	105140619
ENSG0000013184	0.440137047	0.17	0.29	ZNF132	protein_coding	zinc finger p	19	58432814	58440153
ENSG0000024692	0.391632482	0.17	0.29	UBAP1L	protein_coding	ubiquitin as	15	65092770	65115197
ENSG0000016867	0.246431612	0.17	0.29	LDLRAD4	protein_coding	low density	18	13217498	13652755
ENSG0000015192	-0.070747041	0.17	0.29	TIAL1	protein_coding	TIA1 cytotox	10	119571802	119597029
ENSG0000026393	-0.968372936	0.17	0.29	NA	NA	NA	NA	NA	NA
ENSG0000024037	-0.198247671	0.17	0.29	AC010343.1	processed_pse	ribosomal p	5	33162179	33162807
ENSG0000014572	0.093739811	0.17	0.29	PPIP5K2	protein_coding	diphosphoir	5	103120149	103212799
ENSG0000016339	-0.059747223	0.17	0.29	ATP1A1	protein_coding	ATPase Na+	1	116372668	116410261
ENSG0000024203	0.762335159	0.17	0.29	EGFL8	protein_coding	EGF like dor	CHR_HSCHR	32113054	32116752
ENSG0000027576	-0.974072416	0.17	0.29	TBC1D3G	protein_coding	TBC1 doma	CHR_HSCHR	36324256	36335131
ENSG0000011427	0.604581775	0.17	0.29	COL7A1	protein_coding	collagen typ	3	48564073	48595267
ENSG0000012457	0.070684479	0.17	0.29	XPO5	protein_coding	exportin 5 [S	6	43522334	43576038
ENSG0000018248	-0.898545628	0.17	0.29	XKRX	protein_coding	XK related X	X	100913445	100929433
ENSG0000011121	-0.771646782	0.17	0.29	PRR4	protein_coding	proline rich	12	10845849	10849475
ENSG0000010555	-0.337604855	0.171	0.29	PLEKHA4	protein_coding	pleckstrin ho	19	48837097	48868617
ENSG0000015246	-0.248676551	0.171	0.29	RPP38	protein_coding	ribonucleas	10	15097180	15139818
ENSG0000012066	-0.341040047	0.171	0.29	MTRF1	protein_coding	mitochondr	13	41216369	41263577
ENSG0000023683	1.599021125	0.171	0.29	CBR3-AS1	lncRNA	CBR3 antise	21	36131767	36175815
ENSG0000018845	-0.733269464	0.171	0.29	CERKL	protein_coding	ceramide ki	2	181536672	181680665
ENSG0000018709	-0.773106871	0.171	0.29	PLCD1	protein_coding	phospholipa	3	38007496	38029642
ENSG0000023204	-0.915097002	0.171	0.29	EHMT2	protein_coding	euchromatic	CHR_HSCHR	31946781	31964716
ENSG0000010542	1.580352695	0.171	0.29	CNFN	protein_coding	cornifelin [S	19	42387019	42390297
ENSG0000011476	-0.190598013	0.171	0.29	RRP9	protein_coding	ribosomal R	3	51933429	51941904

ENSG0000014685	-0.254420663	0.171	0.29	ZC3HAV1L	protein_coding	zinc finger C	7	139025706	139036042
ENSG0000020406	-0.463032917	0.171	0.29	FOXO6	protein_coding	forkhead bo	1	41361922	41383590
ENSG0000020771	2.707600953	0.171	0.29	NA	NA	NA	NA	NA	NA
ENSG0000025575	2.158718916	0.171	0.29	AC009533.2	processed_pse	WAS protein	12	9314402	9316173
ENSG0000021422	-1.109807918	0.171	0.29	C17orf67	protein_coding	chromosom	17	56791913	56838773
ENSG0000022454	0.903393933	0.171	0.29	SNRPGP15	processed_pse	small nuclea	19	14489388	14489609
ENSG0000018628	0.468764186	0.172	0.29	KDM4D	protein_coding	lysine deme	11	94973709	94999519
ENSG0000020795	-2.708853526	0.172	0.29	MIR656	miRNA	microRNA 6	14	101066724	101066801
ENSG0000017208	0.149426344	0.172	0.29	KRCC1	protein_coding	lysine rich c	2	88027205	88064252
ENSG0000027768	0.708278644	0.172	0.29	AL139407.1	lncRNA	novel transc	10	118692361	118693535
ENSG0000011122	-0.085714583	0.172	0.29	ARPC3	protein_coding	actin relatec	12	110434823	110450422
ENSG0000016293	0.757922183	0.172	0.29	TRIM17	protein_coding	tripartite mo	1	228407935	228416861
ENSG0000021355	-0.103241377	0.172	0.29	RPLP0P6	processed_pse	ribosomal p	2	38481851	38482804
ENSG0000021349	-1.002434034	0.172	0.29	NT5C3AP1	transcribed_pr	NT5C3A pse	4	117574512	117576174
ENSG0000018309	-0.158531078	0.172	0.29	GPC6	protein_coding	glypican 6 [	13	93226807	94408020
ENSG0000017895	-0.082629902	0.172	0.29	TUFM	protein_coding	Tu translatic	16	28842411	28846348
ENSG0000022834	-0.46659482	0.172	0.29	AC115618.2	lncRNA	novel transc	X	48579774	48581157
ENSG0000012033	0.169824967	0.172	0.29	CENPL	protein_coding	centromere	1	173799550	173824720
ENSG0000027178	1.370300961	0.172	0.29	AL080317.1	lncRNA	novel transc	6	111297126	111298510
ENSG0000025046	-1.193778099	0.172	0.29	PHOX2B-A	lncRNA	PHOX2B an	4	41748293	41824119
ENSG0000012082	-0.736055869	0.172	0.29	GLT8D2	protein_coding	glycosyltran	12	103988984	104064183
ENSG0000013504	0.330750572	0.172	0.29	C9orf40	protein_coding	chromosom	9	74946583	74952912
ENSG0000027172	0.603717762	0.172	0.29	NA	NA	NA	NA	NA	NA
ENSG0000016532	0.29553777	0.173	0.29	FAT3	protein_coding	FAT atypica	11	92352096	92896470
ENSG0000016786	-0.198009094	0.173	0.29	MRPL58	protein_coding	mitochondr	17	75012670	75021261
ENSG0000027113	-1.933344184	0.173	0.29	IGLVIVOR2	IG_V_pseudog	immunoglol	22	25437306	25437823
ENSG0000027291	-1.598956432	0.173	0.29	AC090425.3	lncRNA	novel transc	3	179583262	179583762
ENSG0000010626	-0.058411257	0.173	0.29	EIF3B	protein_coding	eukaryotic t	7	2354086	2380745
ENSG0000028016	1.519786085	0.174	0.29	CU638689.5	lncRNA	novel transc	21	6721812	6725209
ENSG0000011178	0.149015151	0.174	0.29	RIC8B	protein_coding	RIC8 guanin	12	106774621	106889316
ENSG0000018880	0.342791514	0.174	0.29	ZNF322P1	processed_pse	zinc finger p	9	97198303	97199511
ENSG0000010062	0.66721217	0.174	0.29	GALNT16	protein_coding	polypeptide	14	69259277	69357033
ENSG0000018048	1.078030056	0.174	0.29	GLIPR1L2	protein_coding	GLIPR1 like	12	75391089	75432688
ENSG0000018842	0.175058879	0.174	0.3	BLOC1S5	protein_coding	biogenesis c	6	8013567	8064396
ENSG0000010322	0.097767068	0.174	0.3	ABCC1	protein_coding	ATP binding	16	15949577	16143074
ENSG0000017783	0.194395267	0.174	0.3	PCDHB9	protein_coding	protocadhe	5	141187127	141191541
ENSG0000016506	-0.72277823	0.174	0.3	ZMAT4	protein_coding	zinc finger r	8	40530590	40897833
ENSG0000024804	-0.295231458	0.174	0.3	UBA6-AS1	lncRNA	UBA6 antise	4	67701209	68080952
ENSG0000011475	-0.153226832	0.174	0.3	PEX5L	protein_coding	peroxisoma	3	179794958	180037053
ENSG0000013381	0.145279367	0.174	0.3	RRAS2	protein_coding	RAS related	11	14277922	14364506
ENSG0000013721	-0.141296385	0.174	0.3	TMEM63B	protein_coding	transmembı	6	44126914	44155519
ENSG0000027263	0.831238029	0.174	0.3	AL731563.3	lncRNA	novel transc	10	73098044	73101297
ENSG0000020427	0.166466317	0.175	0.3	NBDY	protein_coding	negative reg	X	56729241	56819179
ENSG0000027348	-1.970794374	0.175	0.3	AL354760.1	lncRNA	novel transc	1	112517799	112518441
ENSG0000021440	1.639770856	0.175	0.3	KANSL1-AS	lncRNA	KANSL1 ant	17	46193576	46196723
ENSG0000017278	2.252038614	0.175	0.3	RAB43	protein_coding	RAB43, mer	3	129087569	129122801
ENSG0000013869	0.104700097	0.175	0.3	RAP1GDS1	protein_coding	Rap1 GTPas	4	98261384	98443858
ENSG0000019896	0.066455646	0.175	0.3	PJA2	protein_coding	praja ring fi	5	109334713	109409974
ENSG0000025609	-0.792558934	0.176	0.3	SBNO1-AS	lncRNA	SBNO1 anti	12	123363868	123366113
ENSG0000013182	0.110916836	0.176	0.3	PDHA1	protein_coding	pyruvate de	X	19343893	19361718
ENSG0000010101	-0.12319929	0.176	0.3	UQCC1	protein_coding	ubiquinol-c	20	35302566	35412031
ENSG0000022602	0.786662168	0.176	0.3	LINC01772	lncRNA	long interge	1	16460948	16468481
ENSG0000021576	-0.410545539	0.176	0.3	ARHGAP27	lncRNA	ARHGAP27I	17	64749663	64781707
ENSG0000023180	0.255515535	0.176	0.3	PCAT7	lncRNA	prostate car	9	94555054	94603990
ENSG0000028037	0.885237056	0.176	0.3	AC019080.5	lncRNA	novel transc	2	177317715	177318471
ENSG0000027191	1.121832553	0.176	0.3	AL035530.2	lncRNA	novel transc	6	158988178	159088114
ENSG0000013769	0.114527908	0.176	0.3	DCUN1D5	protein_coding	defective in	11	103050686	103092194
ENSG0000025148	-1.202758473	0.176	0.3	NRBF2P6	processed_pse	NRBF2 pseu	2	232343116	232343903

ENSG0000027243	1.896472166	0.176	0.3	AC104117.!	lncRNA	novel transc	5	178938677	178939223
ENSG0000017057	-0.189208036	0.176	0.3	EMB	protein_coding	embigin [Sc	5	50396192	50443248
ENSG0000020752	2.684649087	0.176	0.3	Y_RNA	misc_RNA	Y RNA [Sou	16	74463888	74463988
ENSG0000023015	1.363812876	0.176	0.3	AIDAP1	processed_pse	AIDA pseud	2	11308025	11308941
ENSG0000020774	1.519632051	0.176	0.3	MIR590	miRNA	microRNA 5	7	74191198	74191294
ENSG0000016440	-0.839112468	0.177	0.3	SHROOM1	protein_coding	shroom fam	5	132822141	132830898
ENSG0000013076	0.106509181	0.177	0.3	LRRC47	protein_coding	leucine rich	1	3778559	3796498
ENSG0000014080	0.483111816	0.177	0.3	NKD1	protein_coding	NKD inhibit	16	50548396	50649249
ENSG0000024097	0.765334419	0.177	0.3	RPL23AP64	processed_pse	ribosomal p	11	119003012	119003446
ENSG0000023018	0.975943101	0.177	0.3	C9orf147	lncRNA	chromosom	9	112399512	112487204
ENSG0000014883	-0.83448226	0.177	0.3	PAOX	protein_coding	polyamine c	10	133379261	133391694
ENSG0000027485	0.634178691	0.177	0.3	MRM1	protein_coding	mitochondr	CHR_HSCHR	36602534	36609940
ENSG0000017120	0.177216271	0.177	0.3	TMEM126A	protein_coding	transmembr	11	85647967	85656547
ENSG0000016997	-0.332018705	0.177	0.3	PUSL1	protein_coding	pseudouridi	1	1308597	1311677
ENSG0000022576	-1.545311992	0.177	0.3	DHRS4L1	transcribed_un	dehydroger	14	24036453	24051028
ENSG0000014436	0.314074515	0.177	0.3	PHOSPHO2	protein_coding	phosphatas	2	169694454	169701708
ENSG0000023176	-0.904420467	0.177	0.3	RPS27AP5	protein_coding	ribosomal p	1	192716132	192716653
ENSG0000024366	1.106980795	0.177	0.3	ZNF487	protein_coding	zinc finger p	10	43436841	43483181
ENSG0000027114	1.645436869	0.178	0.3	AC010680.4	lncRNA	novel transc	2	178616581	178617123
ENSG0000016416	0.107241052	0.178	0.3	TMEM184C	protein_coding	transmembr	4	147617386	147672044
ENSG0000022647	-1.519178118	0.178	0.3	Z93930.2	lncRNA	novel transc	22	28800683	28848559
ENSG0000024444	1.030973895	0.178	0.3	EGFL8	protein_coding	EGF like dor	CHR_HSCHR	32122191	32125889
ENSG0000011826	0.080333307	0.178	0.3	CREB1	protein_coding	cAMP respo	2	207529737	207605988
ENSG0000022454	-0.942845831	0.178	0.3	EIF4BP3	processed_pse	eukaryotic t	9	96146007	96147856
ENSG0000014055	0.115261035	0.178	0.3	UNC45A	protein_coding	unc-45 myo	15	90930180	90954093
ENSG0000022472	1.190882602	0.178	0.3	CEP57L1P1	processed_pse	centrosoma	10	70389426	70390626
ENSG0000027411	-0.347358947	0.178	0.3	SMDT1	protein_coding	single-pass	CHR_HSCHR	42079691	42084284
ENSG0000022763	-2.090592817	0.178	0.3	AC018804.1	unprocessed_p	pseudogene	2	130202312	130212628
ENSG0000013064	0.116552297	0.179	0.3	TUBGCP2	protein_coding	tubulin gam	10	133278630	133312337
ENSG0000027290	-0.342812187	0.179	0.3	SMDT1	protein_coding	single-pass	CHR_HSCHR	42079691	42124100
ENSG0000017122	0.137872645	0.179	0.3	SCAND1	protein_coding	SCAN doma	20	35953617	35959472
ENSG0000013644	0.081754997	0.179	0.3	NMT1	protein_coding	N-myristoyl	17	45051610	45109016
ENSG0000018913	-0.72683916	0.179	0.3	UBE2Q2P1	transcribed_un	ubiquitin co	15	84526781	84571216
ENSG0000015650	0.333250541	0.179	0.3	FBXO43	protein_coding	F-box prote	8	100133351	100145817
ENSG0000027475	0.559854692	0.179	0.3	H3C6	protein_coding	H3 clustere	6	26224199	26227473
ENSG0000008191	-0.516814114	0.18	0.3	PHLPP1	protein_coding	PH domain	18	62715541	62980433
ENSG0000025870	0.56418082	0.18	0.3	LINC00638	lncRNA	long interge	14	104821201	104823718
ENSG0000027000	2.280378571	0.18	0.3	AC010531.6	lncRNA	novel transc	16	87317509	87318043
ENSG0000023731	0.653704583	0.18	0.3	GS1-124K5	lncRNA	uncharacter	7	66493607	66495758
ENSG0000014249	-0.86629671	0.18	0.3	SLC47A1	protein_coding	solute carrie	17	19495385	19579034
ENSG0000025680	-0.530826391	0.18	0.3	C17orf100	protein_coding	chromosom	17	6651762	6693202
ENSG0000012624	-0.096335844	0.18	0.3	CAPNS1	protein_coding	calpain sma	19	36139953	36150353
ENSG0000026362	-1.20329868	0.181	0.3	AC055811.1	lncRNA	novel transc	17	17167946	17185554
ENSG0000027841	0.878150416	0.181	0.3	PMS2P2	translated_unp	PMS1 homo	7	75344015	75359550
ENSG0000023570	-1.643628959	0.181	0.31	PCBP2P1	processed_pse	poly(rC) bin	21	39171130	39172106
ENSG0000026777	1.874921772	0.181	0.31	NA	NA	NA	NA	NA	NA
ENSG0000021318	-0.407466679	0.181	0.31	BTF3L4P2	processed_pse	basic transc	2	159003975	159004320
ENSG0000016628	-1.271161978	0.181	0.31	PLEKHF1	protein_coding	pleckstrin h	19	29665459	29675477
ENSG0000017648	-0.714876297	0.181	0.31	PLAAT3	protein_coding	phospholipa	11	63573195	63616883
ENSG0000027745	-1.446970092	0.182	0.31	AC245078.1	protein_coding	PRH1-PRR4	CHR_HSCHR	10845849	11167832
ENSG0000014738	-0.181426747	0.182	0.31	NSDHL	protein_coding	NAD(P) dep	X	152830967	152869729
ENSG0000015686	0.577577922	0.182	0.31	FRRS1	protein_coding	ferric chelat	1	99703970	99766635
ENSG0000014638	-0.224270421	0.182	0.31	ABRACL	protein_coding	ABRA C-ter	6	139028745	139043302
ENSG0000011527	0.142126313	0.182	0.31	MOGS	protein_coding	mannosyl-c	2	74461057	74465410
ENSG0000025485	1.337868141	0.182	0.31	NPIPA2	protein_coding	nuclear pore	16	14748066	14765413
ENSG0000013114	0.157514979	0.182	0.31	EMC8	protein_coding	ER membra	16	85771758	85799608
ENSG0000013838	0.072904047	0.182	0.31	SSB	protein_coding	small RNA b	2	169791933	169812064
ENSG0000023517	-0.289203323	0.182	0.31	RPL39P3	processed_pse	ribosomal p	6	73373108	73373263

ENSG0000023413	1.693817326	0.182	0.31	AL158835.2	lncRNA	novel transc		10	125718771	125719365
ENSG0000023675	-0.582517093	0.182	0.31	EHMT2	protein_coding	euchromatic	CHR_HSCHR		31867303	31885231
ENSG0000025618	-2.368768026	0.182	0.31	AC055720.2	lncRNA	novel transc		12	26335864	26336950
ENSG0000027406	2.13215518	0.183	0.31	U2	snRNA	U2 spliceosc		17	43284139	43284329
ENSG0000016411	-0.117577779	0.183	0.31	MAP9	protein_coding	microtubule		4	155342658	155376970
ENSG0000022728	-0.797341722	0.183	0.31	NA	NA	NA	NA	NA	NA	
ENSG0000022902	1.409145641	0.183	0.31	AKR7A2P1	processed_pse	aldo-keto re		1	112923423	112924337
ENSG0000027742	0.627512647	0.183	0.31	NA	NA	NA	NA	NA	NA	
ENSG0000010098	-0.144711505	0.183	0.31	PCIF1	protein_coding	PDX1 C-tern		20	45934683	45948023
ENSG0000023625	-0.443343445	0.183	0.31	STK19	protein_coding	serine/threo	CHR_HSCHR		31963829	31974100
ENSG0000001416	0.241121512	0.183	0.31	ZC3H3	protein_coding	zinc finger C		8	143437659	143541447
ENSG0000027618	-2.230837317	0.183	0.31	AC069234.4	lncRNA	novel transc		12	120709112	120709523
ENSG0000026066	0.478943896	0.183	0.31	AC004158.1	lncRNA	novel transc		16	72425948	72533892
ENSG0000026995	0.807373182	0.183	0.31	AC090181.2	lncRNA	novel transc		15	77067654	77068325
ENSG0000016619	0.061253516	0.183	0.31	NOLC1	protein_coding	nucleolar an		10	102152176	102163871
ENSG0000013469	0.091778134	0.183	0.31	GNL2	protein_coding	G protein nu		1	37566816	37595937
ENSG0000024185	-0.54349873	0.183	0.31	C8orf58	protein_coding	chromosom		8	22599599	22604150
ENSG0000021941	-1.524824022	0.183	0.31	AC125494.1	lncRNA	novel transc		12	6663260	6672069
ENSG0000027207	1.244249162	0.183	0.31	AC004233.2	lncRNA	novel transc		16	3006120	3007388
ENSG0000027175	1.394384948	0.184	0.31	NA	NA	NA	NA	NA	NA	
ENSG0000027754	-2.302885058	0.184	0.31	AC018926.3	lncRNA	novel transc		15	55346347	55346752
ENSG0000014403	-1.294782077	0.184	0.31	ANKRD53	protein_coding	ankyrin repe		2	70978380	70985499
ENSG0000020628	-0.345501144	0.184	0.31	RXRB	protein_coding	retinoid X re	CHR_HSCHR		33122398	33129499
ENSG0000016011	-0.754408646	0.184	0.31	CPAMD8	protein_coding	C3 and PZP		19	16892951	17026815
ENSG0000014152	0.49104594	0.184	0.31	SLC16A3	protein_coding	solute carrie		17	82228397	82261129
ENSG0000027583	2.458022063	0.184	0.31	AC073107.2	unprocessed_p	sulfatase mo		7	65764535	65768306
ENSG0000020456	-0.196796864	0.184	0.31	PPP1R10	protein_coding	protein pho		6	30600413	30618612
ENSG0000010866	-0.175989158	0.184	0.31	C17orf75	protein_coding	chromosom		17	32324565	32350023
ENSG0000014440	0.151291729	0.185	0.31	UNC80	protein_coding	unc-80 hom		2	209771832	209999300
ENSG0000023383	0.33903242	0.185	0.31	EIF4HP1	processed_pse	eukaryotic t		7	27458163	27458849
ENSG0000027435	-2.306888422	0.185	0.31	AL355987.5	unprocessed_p	ral guanine		9	136728953	136729855
ENSG0000025048	-0.952792259	0.185	0.31	PPM1AP1	processed_pse	protein pho		8	15806149	15807283
ENSG0000027426	-1.409550221	0.185	0.31	AC245297.3	lncRNA	novel transc		1	149175748	149259987
ENSG0000000895	-0.07602077	0.185	0.31	SEC62	protein_coding	SEC62 hom		3	169966635	169998373
ENSG0000025134	0.997845503	0.185	0.31	HSPD1P11	processed_pse	heat shock p		5	95768999	95770700
ENSG0000009608	-0.18961342	0.185	0.31	MRPS18A	protein_coding	mitochondr		6	43671303	43687791
ENSG0000015135	-0.126511479	0.185	0.31	TMEM18	protein_coding	transmembr		2	663877	677406
ENSG0000022799	-2.131721733	0.186	0.31	AC108463.1	processed_pse	pseudogene		2	111203964	111206215
ENSG0000022466	-0.174846787	0.186	0.31	SH3BP5-AS	lncRNA	SH3BP5 ant		3	15254184	15264515
ENSG0000021506	0.556638575	0.186	0.31	ALOX12-AS	lncRNA	ALOX12 ant		17	6875232	7012530
ENSG0000023451	0.478638726	0.186	0.31	PTGES3P1	processed_pse	prostagland		1	89104285	89104767
ENSG0000022624	-0.575432526	0.186	0.31	RPL37AP1	processed_pse	ribosomal p		20	44466564	44466842
ENSG0000027941	0.909285169	0.186	0.31	AC020763.3	TEC	novel transc		16	70713987	70716890
ENSG0000017121	1.24916663	0.186	0.31	CLDN20	protein_coding	claudin 20 [		6	155264013	155276548
ENSG0000022829	-0.849077253	0.187	0.31	HLA-C	protein_coding	major histoc	CHR_HSCHR		31260956	31264327
ENSG0000027025	3.306265744	0.187	0.31	AC096720.1	processed_pse	small EDRK-		4	67638177	67638359
ENSG0000005912	0.164736041	0.187	0.31	FLYWCH1	protein_coding	FLYWCH-ty		16	2911931	2951208
ENSG0000022633	1.00403607	0.187	0.31	AL354836.1	lncRNA	novel transc		20	62305432	62306325
ENSG0000027466	1.90003878	0.187	0.31	AC090517.2	lncRNA	novel transc		15	56729932	56730611
ENSG0000017687	-0.106733497	0.187	0.31	WSB2	protein_coding	WD repeat a		12	118032687	118062430
ENSG0000010843	0.2228617	0.187	0.31	PNPO	protein_coding	pyridoxamir		17	47941506	47949308
ENSG0000027145	-0.816783972	0.187	0.31	AC005034.5	lncRNA	novel transc		2	75669989	75670454
ENSG0000012324	-0.179006322	0.187	0.31	OPTN	protein_coding	optineurin [		10	13099449	13138308
ENSG0000015415	-0.686466963	0.187	0.31	RETREG1	protein_coding	reticulophag		5	16473038	16617058
ENSG0000027744	0.895159107	0.187	0.31	CEBPB-AS1	lncRNA	CEBPB antis		20	50184598	50191498
ENSG0000027937	0.607198566	0.187	0.31	AC244517.1	lncRNA	novel transc		5	141191599	141194088
ENSG0000011426	-0.593019557	0.188	0.31	PFKFB4	protein_coding	6-phosphof		3	48517684	48562015
ENSG0000013260	-0.124195172	0.188	0.31	NIP7	protein_coding	nucleolar pr		16	69337996	69343106

ENSG0000013094	0.44207726	0.188	0.31	PKDREJ	protein_coding	polycystin fa	22	46255663	46263343
ENSG0000009484	0.267961838	0.188	0.31	UPRT	protein_coding	uracil phosp	X	75156388	75304885
ENSG0000024374	0.677666467	0.188	0.31	TMEM35B	protein_coding	transmembr	1	34981535	34985353
ENSG0000011431	0.299080798	0.188	0.31	HES1	protein_coding	hes family b	3	194136148	194138732
ENSG0000025247	1.848249468	0.188	0.31	AC007684.1	snoRNA		2	39283657	39283791
ENSG0000027479	0.67530264	0.188	0.31	F8A2	protein_coding	coagulation	X	155382115	155383230
ENSG0000017491	-0.227869212	0.188	0.32	MICOS13	protein_coding	mitochondr	19	5678421	5680516
ENSG0000027193	-0.423280217	0.188	0.32	AC012073.1	lncRNA	novel transc	2	24825610	24826717
ENSG0000018489	-0.352216446	0.188	0.32	RBM43	protein_coding	RNA binding	2	151247940	151261863
ENSG0000018420	-0.614314599	0.188	0.32	GOLGA6L4	protein_coding	golgin A6 fa	15	84235773	84245368
ENSG0000011948	0.106344407	0.189	0.32	MAPKAP1	protein_coding	MAPK assoc	9	125437393	125707234
ENSG0000010240	-0.078643374	0.189	0.32	ARMCX3	protein_coding	armadillo re	X	101622797	101627843
ENSG0000016436	-0.125465887	0.189	0.32	CCDC127	protein_coding	coiled-coil c	5	196868	218153
ENSG0000027327	-0.693410651	0.189	0.32	AP000254.2	lncRNA	novel transc	21	31666728	31667247
ENSG0000006449	-0.191370913	0.189	0.32	RFXANK	protein_coding	regulatory fa	19	19192229	19201869
ENSG0000010039	0.158893402	0.189	0.32	L3MBTL2	protein_coding	L3MBTL hist	22	41205282	41231271
ENSG0000025531	1.691895806	0.189	0.32	NA	NA	NA	NA	NA	NA
ENSG0000027308	1.158283295	0.189	0.32	AC009309.1	lncRNA	novel transc	2	86195590	86196049
ENSG0000025186	0.813265415	0.189	0.32	AC009812.1	lncRNA	novel transc	8	80484561	80486699
ENSG0000011747	-0.492943054	0.189	0.32	CCDC181	protein_coding	coiled-coil c	1	169394870	169460669
ENSG0000000502	-0.701515679	0.189	0.32	SKAP2	protein_coding	src kinase as	7	26667068	26995239
ENSG0000024456	-0.345832102	0.189	0.32	NA	NA	NA	NA	NA	NA
ENSG0000018911	-0.361900107	0.19	0.32	BLOC1S3	protein_coding	biogenesis c	19	45178784	45216933
ENSG0000023162	0.760831836	0.19	0.32	AL133406.2	lncRNA	novel transc	6	105279016	105281755
ENSG0000015969	-0.079630112	0.19	0.32	CTBP1	protein_coding	C-terminal I	4	1211445	1249953
ENSG0000014881	-0.2616864	0.19	0.32	LRRC27	protein_coding	leucine rich	10	132332154	132381508
ENSG0000027836	3.070203189	0.19	0.32	NDUFA3	protein_coding	NADH:ubiqu	CHR_HSCHR	54102906	54107026
ENSG0000011668	0.086984532	0.19	0.32	MFN2	protein_coding	mitofusin 2	1	11980181	12015211
ENSG0000014343	-0.107984172	0.19	0.32	MRPL9	protein_coding	mitochondr	1	151759647	151763496
ENSG0000014628	-0.103955832	0.19	0.32	PM20D2	protein_coding	peptidase M	6	89146055	89165565
ENSG0000026270	1.206260655	0.19	0.32	AC009121.3	lncRNA	novel transc	16	11348143	11349321
ENSG0000014471	-0.438734071	0.191	0.32	CAND2	protein_coding	cullin associ	3	12796472	12871916
ENSG0000014204	0.730163665	0.191	0.32	TMEM91	protein_coding	transmembr	19	41350911	41384083
ENSG0000014140	0.461374002	0.191	0.32	IMPA2	protein_coding	inositol mon	18	11981025	12030877
ENSG0000017205	-0.53470731	0.191	0.32	SERF1A	protein_coding	small EDRK-	5	70900665	70918530
ENSG0000023029	-0.47467223	0.191	0.32	AC078817.1	processed_pse	ribosomal p	12	80102899	80103333
ENSG0000027532	1.014487118	0.191	0.32	NOSTRIN	protein_coding	nitric oxide	CHR_HSCHR	168831472	168865516
ENSG0000017760	0.263601296	0.191	0.32	HASPIN	protein_coding	histone H3 a	17	3723903	3726699
ENSG0000010480	-0.135947479	0.191	0.32	NUCB1	protein_coding	nucleobindi	19	48900312	48923372
ENSG0000023104	1.242479039	0.191	0.32	NELFE	protein_coding	negative elc	CHR_HSCHR	31939633	31946638
ENSG0000026623	1.456032043	0.191	0.32	NARF-IT1	lncRNA	NARF intror	17	82482098	82483388
ENSG0000013194	-0.183373873	0.191	0.32	C19orf12	protein_coding	chromosom	19	29698886	29715789
ENSG0000015546	-0.090278975	0.192	0.32	OXA1L	protein_coding	OXA1L mitc	14	22766522	22773042
ENSG0000008916	-0.479634454	0.192	0.32	SIRT4	protein_coding	sirtuin 4 [So	12	120302316	120313249
ENSG0000026776	-1.271298064	0.192	0.32	AC011498.0	lncRNA	novel transc	19	4454014	4455286
ENSG0000008280	-0.090770563	0.192	0.32	ERC1	protein_coding	ELKS/RAB6-	12	990509	1495933
ENSG0000014428	0.103365275	0.192	0.32	PKP4	protein_coding	plakophilin 4	2	158456952	158682879
ENSG0000025160	-0.495127125	0.193	0.32	AL928654.1	lncRNA	novel transc	14	105416884	105419739
ENSG0000013431	0.084842192	0.193	0.32	ROCK2	protein_coding	Rho associa	2	11179759	11348330
ENSG0000012329	-0.162209944	0.193	0.32	TSFM	protein_coding	Ts translatio	12	57782761	57808071
ENSG0000014892	0.140910531	0.193	0.32	BTBD10	protein_coding	BTB domain	11	13388008	13463297
ENSG0000004409	-0.124970953	0.193	0.32	CUL7	protein_coding	cullin 7 [Sou	6	43037617	43053945
ENSG0000024044	-1.959120517	0.193	0.32	AC007622.1	processed_pse	ribosomal p	12	107719599	107720133
ENSG0000018431	0.250674977	0.193	0.32	RPL23AP82	transcribed_un	ribosomal p	22	50756948	50801309
ENSG0000023811	0.350389696	0.193	0.32	LINC01410	lncRNA	long interge	9	62801461	62813486
ENSG0000026807	-2.005843978	0.193	0.32	AC006539.1	processed_pse	BCL2/adenc	19	20033444	20034332
ENSG0000026094	0.256946116	0.193	0.32	AL356489.2	lncRNA	novel transc	9	33697459	33700986
ENSG0000013597	0.201201434	0.193	0.32	ANKRD36	protein_coding	ankyrin repe	2	97113153	97264521

ENSG0000024493	0.918886745	0.194	0.32	AL449212.1	unprocessed_p	pseudogene	3	129381298	129394149
ENSG0000009247	0.130029203	0.194	0.32	WDR76	protein_coding	WD repeat	15	43826980	43868412
ENSG0000027987	0.890690899	0.194	0.32	LINC01126	lncRNA	long interge	2	43227210	43228855
ENSG0000020507	1.221613265	0.194	0.32	SYCE1L	protein_coding	synaptonem	16	77199408	77213215
ENSG0000007073	-0.491685757	0.194	0.32	ST6GALNA	protein_coding	ST6 N-acety	17	76565377	76586956
ENSG0000023049	-1.965854876	0.194	0.32	AL035409.1	lncRNA	novel transc	1	77081984	77086402
ENSG0000014592	-0.075098011	0.194	0.32	CPLX2	protein_coding	complexin 2	5	175796310	175884021
ENSG0000014884	0.100761638	0.194	0.32	CNNM2	protein_coding	cyclin and C	10	102918294	103090222
ENSG0000024437	1.369555462	0.194	0.32	PFN1P8	processed_pse	profilin 1 ps	1	146957117	146957659
ENSG0000018343	0.079372015	0.194	0.32	SF3A3	protein_coding	splicing fact	1	37956975	37990075
ENSG0000022890	1.085944674	0.194	0.32	RNF5	protein_coding	ring finger p	CHR_HSCHR	32217742	32220181
ENSG0000025838	0.564036379	0.194	0.32	PPT2-EGFL	protein_coding	PPT2-EGFL8	6	32153845	32171978
ENSG0000026200	-1.020211028	0.194	0.32	DLGAP1-AS	lncRNA	DLGAP1 ant	18	3603000	3608336
ENSG0000024186	-0.387394051	0.194	0.32	RPP21	protein_coding	ribonuclease	CHR_HSCHR	30319108	30336684
ENSG0000023333	-0.902674507	0.195	0.32	UBE2FP3	processed_pse	UBE2F pseu	1	111437514	111438037
ENSG0000026434	0.904581224	0.194	0.32	NOTCH2NL	protein_coding	notch 2 N-t	1	146146203	146229026
ENSG0000026058	0.856187952	0.195	0.32	AC027702.1	lncRNA	novel transc	8	41828165	41829934
ENSG0000014556	0.155019487	0.195	0.32	OTULINL	protein_coding	OTU deubic	5	14581792	14616180
ENSG0000015146	0.086569278	0.195	0.32	CDC123	protein_coding	cell division	10	12195965	12250589
ENSG0000026108	2.905834295	0.195	0.32	NA	NA	NA	NA	NA	NA
ENSG0000021538	-0.345727557	0.195	0.32	MIR99AHG	lncRNA	mir-99a-let	21	15928296	16645467
ENSG0000022478	0.278179909	0.195	0.32	PFDN6	protein_coding	prefoldin su	CHR_HSCHR	33267227	33276326
ENSG0000016774	-0.137582038	0.195	0.32	C19orf48	transcribed_un	chromosom	19	50797704	50804929
ENSG0000013316	-0.095766204	0.195	0.32	BEX1	protein_coding	brain expres	X	103062651	103064171
ENSG0000027481	1.837279631	0.195	0.32	AC126915.2	unprocessed_p	immunoglol	5	178825382	178825571
ENSG0000018426	1.064316551	0.195	0.32	H2AC20	protein_coding	H2A cluster	1	149886918	149887411
ENSG0000012073	-0.0796	0.195	0.32	KDM3B	protein_coding	lysine deme	5	138352685	138437028
ENSG0000025751	-0.517132242	0.195	0.32	AC084824.1	processed_pse	nucleosome	12	32726383	32727387
ENSG0000006830	0.113997568	0.195	0.32	OTUD5	protein_coding	OTU deubic	X	48922028	48958386
ENSG0000024800	-0.423401314	0.195	0.32	NRAV	lncRNA	negative reg	12	120488079	120495946
ENSG0000005879	-0.231585441	0.196	0.33	YIPF1	protein_coding	Yip1 domair	1	53851719	53889798
ENSG0000016613	0.120389762	0.196	0.33	NDUFB8	protein_coding	NADH:ubiqu	10	100523740	100530000
ENSG0000018232	1.493913029	0.196	0.33	GLTPD2	protein_coding	glycolipid tr	17	4788964	4790589
ENSG0000007165	-0.086395984	0.196	0.33	MBD3	protein_coding	methyl-CpG	19	1573596	1592865
ENSG0000016634	-0.738751679	0.196	0.33	RAG1	protein_coding	recombinati	11	36510709	36593156
ENSG0000013818	-0.126190769	0.196	0.33	ENTPD1	protein_coding	ectonucleos	10	95711779	95877266
ENSG0000026133	-0.768494429	0.197	0.33	AC021016.1	lncRNA	novel transc	2	218255319	218257366
ENSG0000022889	0.339564198	0.197	0.33	SKIV2L	protein_coding	Ski2 like RN	CHR_HSCHR	31951734	31962405
ENSG0000016370	0.520677856	0.197	0.33	PRRT3	protein_coding	proline rich	3	9945542	9952408
ENSG0000008883	0.135295128	0.197	0.33	NSFL1C	protein_coding	NSFL1 cofac	20	1442162	1473842
ENSG0000026660	2.13733449	0.197	0.33	AC018521.0	lncRNA	novel transc	17	47929682	47933106
ENSG0000010864	0.454009905	0.197	0.33	B9D1	protein_coding	B9 domain	17	19334308	19378193
ENSG0000019753	-2.795018497	0.197	0.33	IRF1-AS1	lncRNA	IRF1 antisen	5	132410636	132488702
ENSG0000027816	-1.183000181	0.197	0.33	TFPT	protein_coding	TCF3 fusion	CHR_HSCHR	54107066	54115801
ENSG0000023023	0.342779193	0.197	0.33	TRIM26	protein_coding	tripartite mo	CHR_HSCHR	30174354	30203317
ENSG0000024569	0.150643507	0.197	0.33	CRNDE	lncRNA	colorectal n	16	54845189	54929189
ENSG0000010188	0.178579575	0.197	0.33	NKAP	protein_coding	NFKB activa	X	119920672	119943751
ENSG0000011062	0.816060147	0.198	0.33	SLC22A18	protein_coding	solute carrie	11	2899721	2925246
ENSG0000027155	-0.852241644	0.198	0.33	AL355297.3	lncRNA	novel transc	6	156776360	156778422
ENSG0000011848	-0.085667048	0.198	0.33	PHF3	protein_coding	PHD finger	6	63635802	63779336
ENSG0000017008	-0.152829653	0.198	0.33	TMEM192	protein_coding	transmembr	4	165070608	165208549
ENSG0000018549	-1.154375556	0.198	0.33	MUC1	protein_coding	mucin 1, cel	1	155185824	155192916
ENSG0000015402	-0.18301768	0.198	0.33	AK5	protein_coding	adenylate ki	1	77282019	77559966
ENSG0000024261	0.58367837	0.198	0.33	AC022415.1	transcribed_pr	ribosomal p	19	12141806	12143322
ENSG0000010135	0.689613778	0.198	0.33	MROH8	protein_coding	maestro hea	20	37101226	37179588
ENSG0000021584	0.650506183	0.198	0.33	TSTD1	protein_coding	thiosulfate s	1	161037631	161038977
ENSG0000000738	-0.7600863	0.199	0.33	RHBDF1	protein_coding	rhomboid 5	16	58059	76355
ENSG0000007006	0.083900338	0.199	0.33	ELP1	protein_coding	elongator co	9	108866898	108934328

ENSG0000025696	-1.396997162	0.199	0.33	AC018653.3	lncRNA	novel transc	12	7129079	7131198
ENSG0000008872	-0.526487117	0.199	0.33	KIF9	protein_coding	kinesin fami	3	47228026	47283451
ENSG0000023643	-0.513862374	0.199	0.33	AC099336.2	processed_pse	ribosomal p	1	202471864	202472117
ENSG0000026699	-0.578171657	0.199	0.33	AL050343.2	lncRNA	novel transc	1	51793934	51799154
ENSG0000027264	-0.811567494	0.199	0.33	NA	NA	NA	NA	NA	NA
ENSG0000022497	0.746651395	0.199	0.33	INE1	lncRNA	inactivation	X	47204921	47205865
ENSG0000026001	0.945991367	0.199	0.33	AC040169.1	lncRNA	novel transc	16	84117051	84117571
ENSG0000027490	1.714463789	0.199	0.33	AC004241.1	lncRNA	novel transc	12	47731908	47732351
ENSG0000027863	2.074264239	0.199	0.33	AC141557.2	unprocessed_p	WAS protei	12	9415641	9416718
ENSG0000024456	-2.6008679	0.2	0.33	RPS26P19	processed_pse	ribosomal p	2	127846736	127847084
ENSG0000025036	0.875041343	0.2	0.33	AL139353.2	lncRNA	novel transc	14	31420286	31452883
ENSG0000025598	-1.065983267	0.2	0.33	AC007848.1	lncRNA	novel transc	12	5388589	5406651
ENSG0000027925	0.49664401	0.2	0.33	AL121753.2	TEC	novel transc	20	35262727	35264187
ENSG0000016939	0.06879299	0.2	0.33	PTK2	protein_coding	protein tyro	8	140657900	141002216
ENSG0000019762	0.285355313	0.2	0.33	EOLA1	protein_coding	endotheliun	X	149540355	149550510
ENSG0000017088	-0.104206695	0.2	0.33	RNF139	protein_coding	ring finger p	8	124474880	124488618
ENSG0000018352	-0.111155231	0.2	0.33	UTP11	protein_coding	UTP11 smal	1	38009258	38024820
ENSG0000024877	-1.43355262	0.2	0.33	AC097534.1	lncRNA	novel transc	4	173322206	173329694
ENSG0000027919	0.209771087	0.2	0.33	PWAR5	TEC	Prader Willi	15	24985053	24988232
ENSG0000027577	-0.290715007	0.2	0.33	NTAN1	protein_coding	N-terminal	CHR_HSCHR	15040743	15058977
ENSG0000026843	-0.48158758	0.2	0.33	NA	NA	NA	NA	NA	NA
ENSG0000016802	0.731955855	0.201	0.33	TTC21A	protein_coding	tetratricope	3	39107704	39138903
ENSG0000013126	-0.156376344	0.201	0.33	ABCB7	protein_coding	ATP binding	X	75051048	75156732
ENSG0000027200	0.461981757	0.201	0.33	AL139274.2	lncRNA	novel transc	6	87151159	87155285
ENSG0000025022	1.460869651	0.201	0.33	AC053527.2	lncRNA	novel transc	4	73259209	73317953
ENSG0000024891	-2.927865059	0.201	0.33	ATP5MF-PT	protein_coding	ATP5MF-PT	7	99419749	99466197
ENSG0000011485	0.360798125	0.201	0.33	CLCN2	protein_coding	chloride vol	3	184346185	184361650
ENSG0000012445	-0.212596104	0.201	0.33	ZNF45	protein_coding	zinc finger p	19	43912624	43935282
ENSG0000011932	0.214024738	0.202	0.33	ABITRAM	protein_coding	actin bindin	9	108934400	108950744
ENSG0000016195	-0.548858855	0.202	0.33	SENP3	protein_coding	SUMO spec	17	7561919	7571969
ENSG0000027549	0.315630393	0.202	0.33	AC114936.1	transcribed_un	THO compl	CHR_HSCHR	177875522	177955274
ENSG0000014829	0.229889552	0.202	0.33	SURF2	protein_coding	surfeit 2 [So	9	133356550	133361158
ENSG0000016364	-0.22843355	0.202	0.33	PPM1K	protein_coding	protein pho	4	88257620	88284769
ENSG0000018382	-0.180102542	0.202	0.33	BTBD9	protein_coding	BTB domain	6	38168451	38640148
ENSG0000016796	-0.968242954	0.202	0.33	RAB26	protein_coding	RAB26, mer	16	2140803	2154165
ENSG0000023543	-0.250871495	0.202	0.33	LINC01278	lncRNA	long interge	X	63222993	63561095
ENSG0000014647	-0.136062115	0.202	0.33	ARMT1	protein_coding	acidic residu	6	151452258	151470101
ENSG0000014406	-0.537496455	0.202	0.33	NPHP1	protein_coding	nephrocysti	2	110122311	110205066
ENSG0000026751	0.956719572	0.202	0.33	AC011451.1	lncRNA	novel transc	19	9291515	9294482
ENSG0000025776	-1.535866279	0.202	0.33	AC078860.2	lncRNA	novel transc	12	71587642	71594404
ENSG0000012221	-0.060630248	0.203	0.33	COPA	protein_coding	COPI coat c	1	160288594	160343273
ENSG0000026923	1.321396394	0.203	0.33	ZNF350-AS	lncRNA	ZNF350 ant	19	51949134	51981367
ENSG0000027540	0.385356031	0.203	0.33	AC006001.4	processed_pse	translocase	7	66553805	66554199
ENSG0000027715	0.625830012	0.203	0.33	F8A3	protein_coding	coagulation	X	155456914	155458620
ENSG0000022941	0.360960181	0.203	0.33	RALGAPA1I	processed_pse	RALGAPA1	9	105520128	105526359
ENSG0000013069	0.207679341	0.203	0.34	TAF4	protein_coding	TATA-box b	20	61953469	62065810
ENSG0000020565	-0.158910354	0.203	0.34	LIN52	protein_coding	lin-52 DREA	14	74084796	74201235
ENSG0000014670	0.074205155	0.203	0.34	MDH2	protein_coding	malate dehy	7	76048051	76067508
ENSG0000024871	2.432863628	0.203	0.34	AC091180.1	lncRNA	novel transc	17	49361150	49369998
ENSG0000019879	0.069447326	0.203	0.34	CNOT7	protein_coding	CCR4-NOT	8	17224966	17246878
ENSG0000011477	0.29332482	0.203	0.34	ABHD14B	protein_coding	abhydrolase	3	51968510	51983409
ENSG0000015109	0.357221569	0.203	0.34	OXSM	protein_coding	3-oxoacyl-A	3	25782917	25794534
ENSG0000015988	-0.409514327	0.203	0.34	CCDC107	protein_coding	coiled-coil c	9	35658290	35661511
ENSG0000024295	-0.869524355	0.203	0.34	AC007182.2	processed_pse	ribosomal p	14	75595805	75596206
ENSG0000021319	-0.055601172	0.203	0.34	MLLT11	protein_coding	MLLT11 trar	1	151060397	151069544
ENSG0000016748	-0.297847674	0.204	0.34	KLHL26	protein_coding	kelch like fa	19	18637025	18671721
ENSG0000025754	2.430703545	0.204	0.34	AC063948.1	lncRNA	novel transc	12	101408372	101409060
ENSG0000026137	-0.318338392	0.204	0.34	VPS9D1-AS	lncRNA	VPS9D1 ant	16	89711856	89718165

ENSG0000022840	0.143797536	0.204	0.34	RNF5	protein_coding	ring finger p	CHR_HSCHR	32126831	32129264
ENSG0000027349	0.289037119	0.204	0.34	PANK4	protein_coding	pantothena	CHR_HSCHR	2508533	2526600
ENSG0000022353	0.567038149	0.204	0.34	CCHCR1	protein_coding	coiled-coil a	CHR_HSCHR	31187361	31203155
ENSG0000012594	0.055135861	0.204	0.34	HNRNPR	protein_coding	heterogene	1	23303771	23344336
ENSG0000017149	0.103783044	0.204	0.34	PPID	protein_coding	peptidylprol	4	158709127	158723396
ENSG0000012733	0.148519405	0.205	0.34	YEATS4	protein_coding	YEATS dom	12	69359710	69390870
ENSG0000018281	0.231290589	0.205	0.34	DDX28	protein_coding	DEAD-box l	16	68020916	68023232
ENSG0000023172	1.300841191	0.205	0.34	TMSB15B-A	lncRNA	TMSB15B ar	X	103845151	103919548
ENSG0000009520	0.214926942	0.205	0.34	TMEM38B	protein_coding	transmembr	9	105694541	105776629
ENSG0000025556	1.435415894	0.205	0.34	BRWD1-AS	lncRNA	BRWD1 anti	21	39313935	39314962
ENSG0000025940	-1.493214793	0.205	0.34	AC010809.2	lncRNA	novel transc	15	33851785	33856809
ENSG0000009979	-0.144595517	0.205	0.34	NDUFB7	protein_coding	NADH:ubiqu	19	14566078	14572066
ENSG0000027975	0.441288857	0.205	0.34	AC118344.2	TEC	TEC	19	40355842	40360606
ENSG0000015165	0.910359568	0.205	0.34	ADAM8	protein_coding	ADAM meta	10	133262420	133276868
ENSG0000022860	-0.323348599	0.205	0.34	GPANK1	protein_coding	G-patch do	CHR_HSCHR	31651971	31657032
ENSG0000010916	-0.957475325	0.205	0.34	GNRHR	protein_coding	gonadotrop	4	67737118	67754388
ENSG0000008578	0.082223839	0.206	0.34	DDHD2	protein_coding	DDHD dom	8	38225218	38275558
ENSG0000008473	0.067040155	0.206	0.34	RAB10	protein_coding	RAB10, mer	2	26034084	26137454
ENSG0000017936	-1.590850003	0.206	0.34	TMEM31	protein_coding	transmembr	X	103710909	103714032
ENSG0000023602	1.716139526	0.206	0.34	AL591043.2	lncRNA	novel transc	1	176207648	176447919
ENSG0000026891	-0.518855257	0.206	0.34	AC012313.5	lncRNA	novel transc	19	58428632	58431148
ENSG0000015923	0.475017795	0.206	0.34	CBR3	protein_coding	carbonyl rec	21	36135079	36146562
ENSG0000019824	0.248140586	0.206	0.34	SLC29A3	protein_coding	solute carrie	10	71319259	71381423
ENSG0000021552	-0.382165769	0.206	0.34	DDR1	protein_coding	discoidin dc	CHR_HSCHR	30870550	30889733
ENSG0000018511	-0.172199262	0.206	0.34	FAM43A	protein_coding	family with s	3	194685883	194689037
ENSG0000022989	-0.785093975	0.206	0.34	LINC01315	lncRNA	long interge	22	42364390	42369284
ENSG0000015631	0.258882234	0.206	0.34	RPGR	protein_coding	retinitis pigr	X	38269163	38327544
ENSG0000018217	-0.707680423	0.206	0.34	UBA7	protein_coding	ubiquitin like	3	49805209	49813953
ENSG0000006642	-0.088966475	0.206	0.34	ZBTB11	protein_coding	zinc finger a	3	101648889	101677132
ENSG0000015730	0.444314415	0.206	0.34	ZFHX2-AS1	lncRNA	ZFHX2 antis	14	23511760	23560778
ENSG0000027238	-1.070797372	0.207	0.34	NA	NA	NA	NA	NA	NA
ENSG0000004724	-0.119510615	0.207	0.34	ATP6V1H	protein_coding	ATPase H+	8	53715543	53843558
ENSG0000024461	-0.948193124	0.207	0.34	ASPRV1	protein_coding	aspartic pep	2	69960089	69962265
ENSG0000011932	0.11739206	0.207	0.34	FKBP15	protein_coding	FKBP prolyl	9	113161006	113221318
ENSG0000027850	0.355604431	0.207	0.34	TIMM22	protein_coding	translocase	CHR_HSCHR	997117	1003671
ENSG0000015389	-0.298579226	0.207	0.34	ZNF599	protein_coding	zinc finger p	19	34758073	34773229
ENSG0000023531	-1.150236222	0.207	0.34	HM13-IT1	lncRNA	HM13 intror	20	31563166	31564076
ENSG0000013543	1.169787325	0.207	0.34	RDH5	protein_coding	retinol dehy	12	55720367	55724705
ENSG0000001547	-0.045343441	0.208	0.34	MATR3	protein_coding	matrin 3 [Sc	5	139293674	139331677
ENSG0000027809	-0.477787775	0.208	0.34	NA	NA	NA	NA	NA	NA
ENSG0000014713	-0.119770697	0.208	0.34	ZMYM3	protein_coding	zinc finger M	X	71239624	71255146
ENSG0000026014	1.464856148	0.208	0.34	AC145350.2	lncRNA	novel transc	16	33043609	33095431
ENSG0000023513	0.952301829	0.208	0.34	AL445931.1	lncRNA	novel transc	9	134054290	134058805
ENSG0000020423	-0.224905824	0.209	0.34	RXRB	protein_coding	retinoid X re	6	33193588	33200665
ENSG0000008538	0.129907172	0.209	0.34	HACE1	protein_coding	HECT doma	6	104728094	104859919
ENSG0000007118	0.097969448	0.209	0.34	SNX13	protein_coding	sorting nexi	7	17790761	17940501
ENSG0000010138	0.495674664	0.209	0.34	JAG1	protein_coding	jagged canc	20	10637684	10673999
ENSG0000010486	0.557378823	0.209	0.34	LIN7B	protein_coding	lin-7 homol	19	49114324	49118460
ENSG0000012066	-1.926691213	0.209	0.34	SPART-AS1	lncRNA	SPART antis	13	36346431	36369601
ENSG0000027893	1.071375832	0.209	0.34	CR381653.1	lncRNA	novel transc	21	9325013	9368775
ENSG0000011993	0.34084063	0.209	0.34	PPP1R3C	protein_coding	protein pho	10	91628442	91633071
ENSG0000025420	-0.396040086	0.209	0.34	NPIPB11	protein_coding	nuclear pore	16	29381354	29404029
ENSG0000026105	0.653339358	0.209	0.34	SULT1A3	protein_coding	sulfotransfe	16	30199228	30204310
ENSG0000022906	0.452855302	0.209	0.34	ATAT1	protein_coding	alpha tubuli	CHR_HSCHR	30617079	30637059
ENSG0000022585	-0.317716561	0.21	0.34	RUSC1-AS1	lncRNA	RUSC1 antis	1	155316863	155324176
ENSG0000011824	-0.134876928	0.21	0.34	FASTKD2	protein_coding	FAST kinase	2	206765357	206796189
ENSG0000011474	0.098854036	0.21	0.34	COMMD2	protein_coding	COMM dom	3	149738472	149752495
ENSG0000022779	1.338014079	0.21	0.34	CDPF1P1	processed_pse	CDPF1 pseu	2	55224280	55225908

ENSG0000016032	-0.239879525	0.21	0.34	SLC2A6	protein_coding	solute carrie	9	133471094	133479127
ENSG0000013637	-0.283613197	0.21	0.34	MTHFS	protein_coding	methenyltet	15	79833585	79897379
ENSG0000027328	1.439513185	0.21	0.34	AP001033.2	lncRNA	novel transc	18	9259388	9260390
ENSG0000026691	1.210230298	0.211	0.35	NA	NA	NA	NA	NA	NA
ENSG0000005426	0.078495614	0.211	0.35	ARID4B	protein_coding	AT-rich inte	1	235131634	235328219
ENSG0000022687	1.928573986	0.211	0.35	AC005154.1	processed_pse	pseudogene	7	30544053	30544431
ENSG0000018350	0.248387353	0.211	0.35	PI4KAP2	transcribed_un	phosphatidy	22	21473000	21517533
ENSG0000019643	-0.213443392	0.211	0.35	ZNF569	protein_coding	zinc finger p	19	37411155	37469275
ENSG0000026323	-0.877020634	0.211	0.35	AC006111.2	lncRNA	novel transc	16	3650636	3651703
ENSG0000024948	-0.259130316	0.211	0.35	RBBP4P1	processed_pse	RBBP4 pseu	5	14797125	14798400
ENSG0000010803	0.107427979	0.212	0.35	XPNPEP1	protein_coding	X-prolyl am	10	109864766	109923553
ENSG0000021497	-0.721730467	0.212	0.35	CHCHD3P3	processed_pse	coiled-coil-	1	27200834	27201473
ENSG0000012066	-0.575520192	0.212	0.35	SOHLH2	protein_coding	spermatoge	13	36168217	36214588
ENSG0000006782	0.119955752	0.212	0.35	IDH3G	protein_coding	isocitrate de	X	153785766	153794523
ENSG0000025019	2.038762686	0.212	0.35	HMGN1P15	processed_pse	high mobilit	5	115289036	115289330
ENSG0000018697	0.752111988	0.212	0.35	EFCAB6	protein_coding	EF-hand cal	22	43528744	43812337
ENSG0000015329	0.677223258	0.212	0.35	SLC25A27	protein_coding	solute carrie	6	46652915	46678190
ENSG0000027553	-1.899996196	0.212	0.35	AC006449.2	lncRNA	novel transc	17	38703480	38706261
ENSG0000024300	-1.377798263	0.213	0.35	RPP21	protein_coding	ribonuclease	CHR_HSCHR	30319559	30337137
ENSG0000020655	-0.458484514	0.213	0.35	TRIM71	protein_coding	tripartite mo	3	32817997	32897824
ENSG0000017186	0.060348051	0.213	0.35	PTEN	protein_coding	phosphatase	10	87863625	87971930
ENSG0000027284	-0.682643117	0.213	0.35	AL391834.1	lncRNA	novel transc	9	19371386	19371945
ENSG0000020514	1.187551616	0.213	0.35	ARID3C	protein_coding	AT-rich inte	9	34621379	34628086
ENSG0000015942	0.168723113	0.213	0.35	ALDH4A1	protein_coding	aldehyde de	1	18871430	18902724
ENSG0000017110	0.137596309	0.213	0.35	TRMT61B	protein_coding	tRNA methy	2	28849821	28870309
ENSG0000010793	0.109001811	0.213	0.35	EDRF1	protein_coding	erythroid di	10	125719515	125764143
ENSG0000023507	-1.343475041	0.213	0.35	ARNILA	lncRNA	androgen re	2	25369136	25375845
ENSG0000011478	1.434569838	0.214	0.35	ABHD14A-	protein_coding	ABHD14A-A	3	51974706	51989183
ENSG0000027626	-1.746397923	0.214	0.35	AC009509.5	lncRNA	novel transc	12	27700066	27700574
ENSG0000019758	0.740846556	0.214	0.35	BCO2	protein_coding	beta-carote	11	112175510	112224699
ENSG0000026894	-1.529437118	0.214	0.35	AC010422.2	lncRNA	novel transc	19	12682693	12687279
ENSG0000027214	-0.666928746	0.214	0.35	LYRM4-AS1	lncRNA	LYRM4 antis	6	5003774	5045666
ENSG0000010659	0.122557018	0.214	0.35	MRPL32	protein_coding	mitochondr	7	42932376	42948958
ENSG0000025371	0.217878861	0.214	0.35	ALG11	protein_coding	ALG11 alph	13	52012398	52033600
ENSG0000023392	-3.234473944	0.214	0.35	MT1XP1	processed_pse	metallothior	1	16241213	16241398
ENSG0000015786	0.176839724	0.214	0.35	RAB28	protein_coding	RAB28, mer	4	13361354	13484365
ENSG0000027347	0.887993703	0.214	0.35	NA	NA	NA	NA	NA	NA
ENSG0000027937	2.068409851	0.214	0.35	AC137723.1	TEC	TEC	17	82035403	82035800
ENSG0000023709	0.595561098	0.214	0.35	AL732372.2	transcribed_un	pseudogene	1	365389	522928
ENSG0000027904	1.59740789	0.214	0.35	AL353898.2	pseudogene		1	54099968	54100224
ENSG0000022811	1.528748939	0.214	0.35	AC003991.1	lncRNA	novel transc	7	88219359	88304367
ENSG0000027675	0.792701772	0.214	0.35	RN7SL192P	misc_RNA	RNA, 7SL, cy	19	11011708	11012006
ENSG0000010486	0.195963436	0.214	0.35	PPP1R37	protein_coding	protein pho	19	45091396	45148077
ENSG0000012715	-0.245011428	0.214	0.35	BCL11B	protein_coding	BAF chroma	14	99169287	99272197
ENSG0000015886	-1.900644007	0.214	0.35	FCER1G	protein_coding	Fc fragment	1	161215234	161220699
ENSG0000025836	0.491434653	0.215	0.35	RTEL1	protein_coding	regulator of	20	63657810	63696253
ENSG0000015260	-0.079371404	0.215	0.35	MBNL1	protein_coding	muscleblind	3	152243828	152465780
ENSG0000025512	1.080992185	0.215	0.35	AP000880.1	lncRNA	novel transc	11	113278250	113314482
ENSG0000027008	0.732933165	0.215	0.35	GAS5-AS1	lncRNA	GAS5 antise	1	173862473	173863941
ENSG0000023677	0.571189807	0.215	0.35	INTS6-AS1	lncRNA	INTS6 antise	13	51452364	51554678
ENSG0000027226	-0.878324306	0.216	0.35	AL138724.1	lncRNA	novel transc	6	17706257	17707344
ENSG0000022606	-1.113175433	0.216	0.35	LINC00623	lncRNA	long interge	1	120913184	121009291
ENSG0000014374	0.130113282	0.216	0.35	SNAP47	protein_coding	synaptosom	1	227728539	227781231
ENSG0000019697	0.31621055	0.216	0.35	LAGE3	protein_coding	L antigen fa	X	154477769	154479257
ENSG0000026005	0.910164438	0.216	0.35	AL031600.1	lncRNA	novel transc	16	1451760	1452653
ENSG0000018211	-0.099314875	0.216	0.35	NOP10	protein_coding	NOP10 ribo	15	34341719	34343136
ENSG0000013803	-0.343072738	0.216	0.35	KHK	protein_coding	ketohexokir	2	27086747	27100762
ENSG0000017113	-0.116001572	0.216	0.35	ATP6V0E2	protein_coding	ATPase H+	7	149872968	149891204

ENSG0000011829	-0.944694522	0.216	0.35	C1orf54	protein_coding	chromosom		1	150268200	150280916
ENSG0000008868	0.132116748	0.217	0.35	COQ9	protein_coding	coenzyme C		16	57447425	57461270
ENSG0000023300	-0.989522062	0.217	0.35	MIR3936HC	lncRNA	MIR3936 hc		5	132311285	132370170
ENSG0000027902	1.323806967	0.217	0.35	AL359715.4	TEC	TEC		6	80440730	80441172
ENSG0000010311	-0.112131317	0.217	0.35	MON1B	protein_coding	MON1 hom		16	77190835	77202398
ENSG0000027283	-0.319435167	0.217	0.35	SMDT1	protein_coding	single-pass	CHR_HSCHR		42079691	42084284
ENSG0000018529	-1.001065715	0.217	0.35	IL3RA	protein_coding	interleukin 3	X		1336616	1382689
ENSG0000023160	-0.65816968	0.217	0.35	AC007098.1	lncRNA	novel transc		2	62957326	63048640
ENSG0000025833	0.25714587	0.217	0.35	AC125611.4	lncRNA	novel transc		12	49292631	49324576
ENSG0000012545	-0.215698389	0.218	0.35	NT5C	protein_coding	5', 3'-nucle		17	75130225	75131757
ENSG0000026150	0.972482558	0.218	0.35	LINC01686	lncRNA	long interge		1	182615254	182616629
ENSG0000017493	0.142732048	0.218	0.35	ASPHD1	protein_coding	aspartate be		16	29900375	29919864
ENSG0000024849	-0.441475867	0.218	0.35	ZBED9	protein_coding	zinc finger E	CHR_HSCHR		28573507	28587330
ENSG0000024177	-1.335925454	0.218	0.35	RPP21	protein_coding	ribonucleas	CHR_HSCHR		30393209	30410787
ENSG0000025854	-2.111120962	0.218	0.35	CR354443.1	protein_coding	novel protei	CHR_HSCHR		31705334	31711904
ENSG0000027235	0.745850645	0.218	0.35	AL080317.2	lncRNA	novel transc		6	111309203	111313517
ENSG0000020628	0.255845713	0.218	0.35	PFDN6	protein_coding	prefoldin su	CHR_HSCHR		33218133	33227229
ENSG0000025437	-1.520050697	0.218	0.35	AC025871.2	lncRNA	novel transc		8	28415524	28420055
ENSG0000018205	1.697245466	0.218	0.35	OGFRP1	lncRNA	opioid grow		22	42269703	42279534
ENSG0000014095	0.25903736	0.218	0.35	MEAK7	protein_coding	MTOR assoc		16	84476355	84554033
ENSG0000025457	1.819786867	0.218	0.35	AC087276.1	lncRNA	novel transc		11	43390283	43395495
ENSG0000018753	0.65893957	0.218	0.36	PRR13P5	processed_pse	proline rich		19	39943239	39943680
ENSG0000014501	-0.275658519	0.218	0.36	TMEM44	protein_coding	transmembr		3	194587673	194633689
ENSG0000017186	-0.211936383	0.219	0.36	MRM3	protein_coding	mitochondr		17	782353	792509
ENSG0000026948	-1.292980889	0.219	0.36	AC010319.4	lncRNA	novel transc		19	17414257	17422324
ENSG0000027537	1.888354891	0.219	0.36	AC113385.3	unprocessed_p	glucuronida		5	100375700	100381398
ENSG0000025724	-1.883097389	0.219	0.36	AC020612.1	processed_pse	Protein DJ-1		12	49595148	49595688
ENSG0000022843	0.636575445	0.219	0.36	NA	NA	NA	NA	NA	NA	NA
ENSG0000025326	1.903388087	0.219	0.36	AP003354.1	lncRNA	novel transc		8	102891876	102893608
ENSG0000027895	0.787905845	0.219	0.36	AC138907.8	TEC	novel transc		16	32781883	32784138
ENSG0000027986	-0.619042591	0.219	0.36	AC069547.1	TEC	TEC		10	50334538	50336123
ENSG0000008932	-0.80500362	0.219	0.36	FXYD5	protein_coding	FXYD doma		19	35154730	35169881
ENSG0000025499	-0.325771665	0.219	0.36	ANKHD1-E	protein_coding	ANKHD1-EI		5	140401908	140549569
ENSG0000027669	-0.890510334	0.219	0.36	AL136295.6	lncRNA	novel transc		14	24198433	24199090
ENSG0000017802	0.342952379	0.219	0.36	LRRC75B	protein_coding	leucine rich		22	24585620	24593208
ENSG0000027486	2.225595863	0.219	0.36	U2	snRNA	U2 spliceos		17	43277978	43278168
ENSG0000026139	-1.375208167	0.219	0.36	MAFTRR	lncRNA	MAF transcr		16	79715220	79770651
ENSG0000011554	-0.091703076	0.219	0.36	MOB4	protein_coding	MOB family		2	197515571	197553699
ENSG0000022188	0.920331187	0.219	0.36	ARIH2OS	lncRNA	ARIH2 oppo		3	48917782	48918823
ENSG0000022336	1.411405272	0.219	0.36	FTH1P10	transcribed_pr	ferritin heav		5	17353695	17354624
ENSG0000027953	1.887304715	0.22	0.36	AC002094.5	TEC	novel transc		17	28373673	28374301
ENSG0000008245	0.298223933	0.22	0.36	DLG3	protein_coding	discs large M	X		70444835	70505490
ENSG0000014033	0.127554805	0.22	0.36	TLE3	protein_coding	TLE family n		15	70047790	70098176
ENSG0000012444	-0.253991608	0.22	0.36	ZNF576	protein_coding	zinc finger p		19	43596392	43601157
ENSG0000023108	1.283770899	0.22	0.36	RPL22P24	processed_pse	ribosomal p		1	185171335	185171710
ENSG0000026793	2.30424226	0.22	0.36	AC008946.1	lncRNA	novel transc		19	8008729	8016025
ENSG0000025336	-1.726837857	0.22	0.36	AC069120.1	lncRNA	novel transc		8	38543276	38560877
ENSG0000010014	0.339007295	0.22	0.36	CCDC134	protein_coding	coiled-coil c		22	41800679	41826299
ENSG0000011171	-0.107476492	0.22	0.36	GOLT1B	protein_coding	golgi transp		12	21501781	21518408
ENSG0000019781	0.727400475	0.22	0.36	AC122129.1	lncRNA	novel transc		17	17858227	17860041
ENSG0000021378	-0.111612228	0.22	0.36	DDX47	protein_coding	DEAD-box I		12	12813316	12829981
ENSG0000025009	0.334481594	0.22	0.36	DNAH10OS	lncRNA	dynein axon		12	123925461	123934984
ENSG0000023648	2.020618974	0.221	0.36	AC133473.1	processed_pse	ribosomal p		3	185606575	185606873
ENSG0000016390	0.16991189	0.221	0.36	TMEM41A	protein_coding	transmembr		3	185476496	185499057
ENSG0000006165	0.734499156	0.221	0.36	SPAG4	protein_coding	sperm assoc		20	35615829	35621094
ENSG0000018009	0.682942315	0.221	0.36	SEPTIN1	protein_coding	septin 1 [So		16	30378133	30395991
ENSG0000027556	0.517796018	0.221	0.36	AC008115.3	lncRNA	novel transc		12	12718973	12719521
ENSG0000013540	-0.404741965	0.221	0.36	AVIL	protein_coding	advillin [Sou		12	57797376	57818704

ENSG0000025694	1.290218697	0.221	0.36	PPP1R14B-	lncRNA	PPP1R14B a		11	64245961	64248218
ENSG0000019747	0.240027168	0.221	0.36	ZNF695	protein_codinç	zinc finger p		1	246945547	247008093
ENSG0000022488	-0.80739745	0.221	0.36	AC138028.2	lncRNA	novel transc		16	88731180	88741425
ENSG0000026000	0.937372212	0.221	0.36	AL133338.1	lncRNA	novel transc		6	100881471	100882987
ENSG0000027831	-0.12323386	0.221	0.36	GGNBP2	protein_codinç	gametogen		17	36544912	36589848
ENSG0000028005	0.757500939	0.221	0.36	AC004241.5	TEC	TEC		12	47728151	47730598
ENSG0000017786	-0.299460086	0.221	0.36	SVBP	protein_codinç	small vasoh		1	42807052	42817397
ENSG0000023719	-1.682669913	0.221	0.36	SNAI1P1	processed_pse	snail family		2	209808804	209809580
ENSG0000027460	0.242564297	0.222	0.36	PI4KAP1	transcribed_un	phosphatidy		22	18533646	18577968
ENSG0000014311	0.875361781	0.222	0.36	C1orf162	protein_codinç	chromosom		1	111473792	111478512
ENSG0000019829	-0.382607025	0.222	0.36	ZNF485	protein_codinç	zinc finger p		10	43606419	43617904
ENSG0000007694	0.940717969	0.222	0.36	STXBP2	protein_codinç	syntaxin bin		19	7636881	7647873
ENSG0000027640	1.782477345	0.222	0.36	AC025287.2	lncRNA	novel transc		16	75556392	75557059
ENSG0000020237	2.210390619	0.222	0.36	SNORA70	snoRNA	Small nucle		3	108574565	108574698
ENSG0000023400	-0.359385169	0.222	0.36	RPL5P34	processed_pse	ribosomal p		22	42776406	42777296
ENSG0000013071	0.147543514	0.222	0.36	UCK1	protein_codinç	uridine-cyti		9	131523801	131531264
ENSG0000025460	0.899383565	0.222	0.36	AP000662.1	lncRNA	novel transc		11	57638024	57652790
ENSG0000022573	-0.110165185	0.222	0.36	FGD5-AS1	lncRNA	FGD5 antise		3	14920347	14948424
ENSG0000027597	-0.426117148	0.222	0.36	CNOT3	protein_codinç	CCR4-NOT	CHR_HSCHR		54138182	54156191
ENSG0000021212	-0.969943087	0.222	0.36	TAS2R14	protein_codinç	taste 2 rece		12	10937408	11171573
ENSG0000018526	0.465176113	0.222	0.36	KIAA0825	protein_codinç	KIAA0825 [S		5	94152966	94618597
ENSG0000015093	0.446441885	0.223	0.36	CRIM1	protein_codinç	cysteine rich		2	36355778	36551135
ENSG0000010268	-1.182965844	0.223	0.36	SGCG	protein_codinç	sarcoglycan		13	23180979	23325162
ENSG0000017626	-0.98825554	0.223	0.36	CYCSP34	processed_pse	CYCS pseuc		13	40863599	40863902
ENSG0000024023	0.160347831	0.223	0.36	COX19	protein_codinç	cytochrome		7	898778	975549
ENSG0000000350	-0.147056346	0.223	0.36	NDUFAF7	protein_codinç	NADH:ubiqu		2	37231631	37253403
ENSG0000007874	0.087114343	0.223	0.36	ITCH	protein_codinç	itchy E3 ubi		20	34363241	34540748
ENSG0000025882	1.029190892	0.223	0.36	AL122035.1	lncRNA	novel transc		14	64422935	64448557
ENSG0000023579	1.755537354	0.224	0.36	AC093157.2	lncRNA	novel transc		1	100995473	100996260
ENSG0000024032	1.755537354	0.224	0.36	RN7SL481P	misc_RNA	RNA, 7SL, c		7	143298516	143298814
ENSG0000021019	0.308326229	0.224	0.36	MT-TT	Mt_tRNA	mitochondr	MT		15888	15953
ENSG0000023074	-0.666816164	0.224	0.36	AC021188.1	lncRNA	novel transc		2	96307263	96321731
ENSG0000019809	0.244504437	0.224	0.36	ZNF649	protein_codinç	zinc finger p		19	51889235	51905040
ENSG0000025294	3.156385061	0.224	0.36	SCARNA1	scaRNA	small Cajal b		1	27834401	27834566
ENSG0000020380	-0.874719656	0.224	0.36	PLPP4	protein_codinç	phospholipi		10	120457227	120592065
ENSG0000023140	2.106958273	0.224	0.36	GORAB-AS	lncRNA	GORAB anti		1	170460453	170532647
ENSG0000010003	-0.167855391	0.224	0.36	TOP3B	protein_codinç	DNA topois		22	21957025	21982813
ENSG0000022833	-0.208565086	0.224	0.36	RXRB	protein_codinç	retinoid X re	CHR_HSCHR		33171494	33178594
ENSG0000027727	-0.221178073	0.224	0.36	CDK7	protein_codinç	cyclin deper	CHR_HSCHR		69234793	69277413
ENSG0000013806	-0.268825644	0.224	0.36	CYP1B1	protein_codinç	cytochrome		2	38066973	38109902
ENSG0000027669	0.608255127	0.224	0.36	NA	NA	NA	NA	NA		NA
ENSG0000026324	-0.685352858	0.225	0.36	PRR4	protein_codinç	proline rich	CHR_HSCHR		10824960	11203561
ENSG0000017575	0.114008061	0.225	0.36	AURKAIP1	protein_codinç	aurora kinas		1	1373730	1375495
ENSG0000027315	1.443980818	0.225	0.36	AC092587.1	protein_codinç	novel LIPT1		2	99154998	99195298
ENSG0000027882	0.154372599	0.225	0.36	BMERB1	protein_codinç	bMERB don	CHR_HSCHR		15519415	15710184
ENSG0000021663	-1.643333493	0.225	0.36	AL133406.1	processed_pse	ribosomal p		6	105298149	105298895
ENSG0000022469	0.858198266	0.225	0.36	LAMTOR5-	lncRNA	LAMTOR5 a		1	110347116	110443817
ENSG0000023025	-0.890248831	0.225	0.36	HLA-E	protein_codinç	major histo	CHR_HSCHR		30481701	30486439
ENSG0000023733	0.258640913	0.225	0.36	PFDN6	protein_codinç	prefoldin su	CHR_HSCHR		33210906	33220005
ENSG0000027961	1.027872024	0.225	0.36	AC012313.1	TEC	novel transc		19	58350795	58351720
ENSG0000017691	-0.92373398	0.225	0.36	TYMSOS	lncRNA	TYMS oppo		18	630886	658340
ENSG0000017391	0.133825235	0.225	0.36	RBM4B	protein_codinç	RNA bindinç		11	66664998	66677887
ENSG0000022644	-1.131314609	0.225	0.36	BX322234.1	lncRNA	novel transc		6	169213254	169245773
ENSG0000010783	-0.155059086	0.225	0.36	NPM3	protein_codinç	nucleophos		10	101781325	101783413
ENSG0000023693	2.204259415	0.225	0.36	PTGES3P4	processed_pse	prostagland		10	102845595	102845950
ENSG0000018216	0.44823879	0.226	0.36	TP53TG1	lncRNA	TP53 target		7	87322943	87345528
ENSG0000023512	0.982368351	0.226	0.36	AL645504.1	lncRNA	novel transc		1	201723294	201737506
ENSG0000017074	0.15051552	0.226	0.36	SYT9	protein_codinç	synaptotagr		11	7238778	7469043

ENSG0000019635	-0.457992516	0.226	0.36	ZNF565	protein_coding	zinc finger p	19	36182276	36246257
ENSG0000021246	3.136591657	0.226	0.36	SNORA12	snoRNA	small nucleo	10	100237156	100237302
ENSG0000025475	1.3591883	0.226	0.36	NAP1L1P1	processed_pse	nucleosome	11	126067539	126068601
ENSG0000027142	-0.394808981	0.226	0.36	NBPF10	protein_coding	NBPF memb	1	146064711	146229000
ENSG0000023391	-0.279944956	0.226	0.36	RPL10P9	processed_pse	ribosomal p	5	168616352	168616996
ENSG0000014103	-0.10189796	0.226	0.36	COPS3	protein_coding	COP9 signa	17	17246616	17281273
ENSG0000014765	-0.171253018	0.226	0.36	EBAG9	protein_coding	estrogen rec	8	109539711	109565996
ENSG0000017205	0.143042156	0.226	0.36	ORMDL3	protein_coding	ORMDL sph	17	39921041	39927601
ENSG0000027956	0.659644667	0.227	0.37	NA	NA	NA	NA	NA	NA
ENSG0000013881	0.074022759	0.227	0.37	PPP3CA	protein_coding	protein pho	4	101023409	101348278
ENSG0000013005	-0.271455353	0.227	0.37	FAM155B	protein_coding	family with s	X	69504326	69532508
ENSG0000015471	0.11991117	0.227	0.37	RABGEF1	protein_coding	RAB guanin	7	66682164	66811464
ENSG0000010436	-0.148774956	0.227	0.37	IKBKB	protein_coding	inhibitor of	8	42271302	42332653
ENSG0000018359	1.169376641	0.227	0.37	H3C13	protein_coding	H3 clustered	1	149813225	149813693
ENSG0000021724	0.281843764	0.227	0.37	CBX3P9	processed_pse	chromobox	6	116453014	116453565
ENSG0000027764	0.345970557	0.227	0.37	TIMM22	protein_coding	translocase	CHR_HSCHR	997117	1003671
ENSG0000011698	-0.480619927	0.227	0.37	BMP8B	protein_coding	bone morph	1	39757182	39788865
ENSG0000016968	0.224547526	0.228	0.37	LRRC45	protein_coding	leucine rich	17	82023305	82031151
ENSG0000012873	0.079415828	0.228	0.37	SNRPN	protein_coding	small nuclea	15	24823637	24978723
ENSG0000014435	0.827190505	0.228	0.37	DLX1	protein_coding	distal-less h	2	172084740	172089677
ENSG0000023718	0.419156581	0.228	0.37	AC242426.2	lncRNA	novel transc	1	147172755	147295734
ENSG0000025024	-1.637475242	0.228	0.37	AC008840.1	lncRNA	novel transc	5	95701249	95732295
ENSG0000022721	-1.156415555	0.228	0.37	AL157935.1	lncRNA	novel transc	9	127934503	127940952
ENSG0000014389	-0.347266097	0.228	0.37	GALM	protein_coding	galactose m	2	38666081	38741237
ENSG0000022685	1.116227956	0.229	0.37	AC010894.1	lncRNA	novel transc	2	174487380	174488386
ENSG0000024802	1.031543744	0.229	0.37	ARHGAP42	lncRNA	ARHGAP42	11	100684162	100687955
ENSG0000017205	0.34823939	0.229	0.37	KLF11	protein_coding	Kruppel like	2	10042849	10054836
ENSG0000018510	0.867519855	0.229	0.37	MYADML2	protein_coding	myeloid ass	17	81939645	81947233
ENSG0000023819	1.022881447	0.229	0.37	LRIG2-DT	lncRNA	LRIG2 diverg	1	113011687	113073113
ENSG0000024199	-0.72063487	0.229	0.37	PRR34-AS1	lncRNA	PRR34 antis	22	46053705	46057210
ENSG0000007745	0.0890796	0.229	0.37	FAM76B	protein_coding	family with s	11	95768953	95790409
ENSG0000010640	0.121382966	0.229	0.37	ZNHIT1	protein_coding	zinc finger H	7	101218165	101224190
ENSG0000018152	-0.373428674	0.229	0.37	RPL24P4	processed_pse	ribosomal p	6	42956345	42956765
ENSG0000010278	-0.14909602	0.229	0.37	DGKH	protein_coding	diacylglycer	13	42040036	42256578
ENSG0000008708	-0.068330758	0.23	0.37	SRRT	protein_coding	serrate, RNA	7	100875103	100888664
ENSG0000025530	1.380998811	0.23	0.37	AC004923.4	lncRNA	novel transc	11	68024809	68030461
ENSG0000027611	1.197345525	0.23	0.37	FUT8-AS1	lncRNA	FUT8 antise	14	65411170	65412690
ENSG0000015223	-0.05811631	0.23	0.37	ATP5F1A	protein_coding	ATP synthas	18	46080248	46104334
ENSG0000027653	0.316664792	0.23	0.37	HRAS	protein_coding	HRas proto-	CHR_HSCHR	532242	537287
ENSG0000011681	-0.322917501	0.23	0.37	CD58	protein_coding	CD58 molec	1	116514534	116571039
ENSG0000011166	0.150135901	0.23	0.37	CHPT1	protein_coding	choline pho	12	101696947	101744140
ENSG0000017614	-0.1239469	0.23	0.37	TCP11L1	protein_coding	t-complex 1	11	33039417	33105943
ENSG0000017825	-0.084780249	0.23	0.37	WDR6	protein_coding	WD repeat d	3	49007062	49015953
ENSG0000022608	-0.462018969	0.23	0.37	AC113935.1	processed_pse	ribosomal p	1	77129114	77129668
ENSG0000026008	1.157277961	0.23	0.37	DDX59-AS1	lncRNA	DDX59 antis	1	200669507	200694250
ENSG0000017372	-1.54483095	0.231	0.37	C1orf100	protein_coding	chromosom	1	244352635	244389663
ENSG0000018183	-0.230338713	0.231	0.37	SLC35C1	protein_coding	solute carrie	11	45804072	45813016
ENSG0000025924	0.850877132	0.231	0.37	USP3-AS1	lncRNA	USP3 antise	15	63544247	63601589
ENSG0000016060	-0.516663804	0.231	0.37	NEK8	protein_coding	NIMA relate	17	28725897	28743455
ENSG0000023769	1.494361245	0.231	0.37	IRGM	protein_coding	immunity re	5	150846523	150900736
ENSG0000023976	-0.592787801	0.231	0.37	AC009120.1	processed_pse	ribosomal p	16	74312609	74313390
ENSG0000024195	-2.056868186	0.232	0.37	AL021937.4	lncRNA	novel transc	22	32383786	32385631
ENSG0000026188	-0.412670453	0.232	0.37	AC040162.1	protein_coding	novel transc	16	67929614	67936017
ENSG0000010016	0.218455073	0.232	0.37	CENPM	protein_coding	centromere	22	41938737	41947152
ENSG0000012535	0.550316301	0.232	0.37	TMEM255A	protein_coding	transmembr	X	120258650	120311556
ENSG0000024370	1.657808093	0.232	0.37	PLA2G4B	protein_coding	phospholipa	15	41837775	41848147
ENSG0000018317	-0.306045073	0.232	0.37	SMDT1	protein_coding	single-pass	22	42079691	42084284
ENSG0000009009	-0.126034482	0.232	0.37	PCBP4	protein_coding	poly(rC) bin	3	51957454	51974016

ENSG0000023634	-0.189289394	0.232	0.37	ABCF1	protein_coding	ATP binding	CHR_HSCHR		30649829	30675633
ENSG0000025419	0.452033261	0.232	0.37	AC113191.1	processed_pse	Family with		8	43125995	43126397
ENSG0000016251	0.130285629	0.232	0.37	SDC3	protein_coding	syndecan 3		1	30869466	30908758
ENSG0000023061	1.049069724	0.232	0.37	AL139220.2	lncRNA	novel transc		1	44030437	44115913
ENSG0000027521	1.831875194	0.232	0.37	U2	snRNA	U2 spliceos		17	43296425	43296615
ENSG0000016823	-0.199342365	0.232	0.37	TTC39C	protein_coding	tetratricope		18	23992773	24135610
ENSG0000016685	-0.09699232	0.233	0.37	CLPX	protein_coding	caseinolytic		15	65148219	65185342
ENSG0000016459	0.096853079	0.233	0.37	COG5	protein_coding	component		7	107201555	107564514
ENSG0000000792	-0.078938893	0.233	0.37	DNAJC11	protein_coding	DnaJ heat sl		1	6634168	6701924
ENSG0000026276	-0.744650085	0.233	0.37	AC135050.5	lncRNA	novel transc		16	31118078	31118747
ENSG0000011953	0.169356289	0.233	0.37	KDSR	protein_coding	3-ketodihyd		18	63327726	63367228
ENSG0000007212	0.124429722	0.233	0.37	ZFYVE26	protein_coding	zinc finger F		14	67727374	67816590
ENSG0000025439	1.428095258	0.233	0.37	AC055876.2	unprocessed_p	hect domair		15	28679405	28682252
ENSG0000004275	0.148675785	0.233	0.37	AP2S1	protein_coding	adaptor rela		19	46838136	46850846
ENSG0000022925	-0.715008497	0.233	0.37	HLA-E	protein_coding	major histoc	CHR_HSCHR		30479706	30484444
ENSG0000023124	1.144972277	0.233	0.37	ITPR1-DT	lncRNA	ITPR1 diverg		3	4490891	4493163
ENSG0000024431	-0.213970894	0.234	0.37	AC024293.1	processed_pse	ribosomal p		11	46428653	46429150
ENSG0000022685	-1.488920856	0.234	0.37	AC004112.1	lncRNA	novel transc		7	112328189	112409623
ENSG0000025830	-0.466228272	0.234	0.37	VASH1-AS1	lncRNA	VASH1 antis		14	76781733	76786724
ENSG0000018803	-0.275150391	0.234	0.37	ZNF490	protein_coding	zinc finger p		19	12576100	12640098
ENSG0000016737	0.211230483	0.234	0.37	ZNF23	protein_coding	zinc finger p		16	71447597	71463095
ENSG0000016362	-0.154811124	0.234	0.37	COX18	protein_coding	cytochrome		4	73052362	73069755
ENSG0000017004	-0.362230613	0.234	0.37	KCNAB3	protein_coding	potassium v		17	7921859	7929803
ENSG0000024844	0.583260064	0.234	0.37	SEMA6A-A	lncRNA	SEMA6A an		5	116447547	116508276
ENSG0000023164	0.346109371	0.234	0.37	TRIM26	protein_coding	tripartite mc	CHR_HSCHR		30262891	30291774
ENSG0000016363	0.108409548	0.234	0.37	THOC7	protein_coding	THO comple		3	63833870	63863868
ENSG0000025188	2.468510285	0.235	0.38	RNU7-75P	snRNA	RNA, U7 sm		5	37327135	37327196
ENSG0000026623	2.468510285	0.235	0.38	NA	NA	NA	NA	NA		NA
ENSG0000017640	-1.568925576	0.235	0.38	GJC3	protein_coding	gap junctior		7	99923266	99929620
ENSG0000014555	0.374040883	0.235	0.38	MYO10	protein_coding	myosin X [S		5	16661907	16936288
ENSG0000024306	1.858563629	0.235	0.38	AL359853.2	lncRNA	novel transc		1	179730191	179742697
ENSG0000012917	0.251927717	0.235	0.38	E2F8	protein_coding	E2F transcri		11	19224063	19241620
ENSG0000014572	-0.243169524	0.235	0.38	GIN1	protein_coding	gypsy retrot		5	103086000	103120138
ENSG0000023961	-1.513645227	0.235	0.38	AC073610.1	processed_pse	ribosomal p		12	48903418	48903813
ENSG0000023975	-1.028961717	0.235	0.38	PHF1	lncRNA	PHD finger	CHR_HSCHR		33339192	33340037
ENSG0000016203	0.267543014	0.235	0.38	SPSB3	protein_coding	splA/ryanoc		16	1776712	1793700
ENSG0000015841	0.058593605	0.236	0.38	EIF5B	protein_coding	eukaryotic t		2	99337371	99401326
ENSG0000026027	0.748903455	0.236	0.38	AC137932.1	lncRNA	novel transc		16	89296128	89298317
ENSG0000016457	-0.102989888	0.236	0.38	SAP30L	protein_coding	SAP30 like [		5	154445997	154461053
ENSG0000027615	-2.466796544	0.236	0.38	AL031591.1	misc_RNA			22	27922022	27922178
ENSG0000010334	0.233801068	0.236	0.38	ZNF174	protein_coding	zinc finger p		16	3401215	3409364
ENSG0000015905	0.145436449	0.236	0.38	MIS18A	protein_coding	MIS18 kinet		21	32268228	32279049
ENSG0000000825	0.105840195	0.237	0.38	CYTH3	protein_coding	cytohesin 3		7	6161776	6272644
ENSG0000016087	0.08853447	0.237	0.38	NACC1	protein_coding	nucleus acc		19	13116862	13141147
ENSG0000013263	-0.086147127	0.237	0.38	SNAP25	protein_coding	synaptosom		20	10218830	10307418
ENSG0000026321	0.679093909	0.237	0.38	NA	NA	NA	NA	NA		NA
ENSG0000011885	0.130235713	0.237	0.38	MFSD1	protein_coding	major facilit		3	158732198	158829719
ENSG0000023281	0.372817816	0.237	0.38	DUX4L50	unprocessed_p	double hom		9	63817748	63818462
ENSG0000028033	0.671974218	0.237	0.38	AC020917.4	TEC	TEC		19	16356329	16358327
ENSG0000010533	0.297283755	0.237	0.38	DENND3	protein_coding	DENN doma		8	141117278	141195808
ENSG0000010844	0.094542366	0.237	0.38	RPS6KB1	protein_coding	ribosomal p		17	59893046	59950574
ENSG0000015211	0.133275704	0.237	0.38	SMPD4BP	transcribed_pr	sphingomye		2	131492813	131521573
ENSG0000026230	0.501611692	0.237	0.38	AC027796.3	protein_coding	novel readtl		17	3585149	3636249
ENSG0000025534	1.845081418	0.238	0.38	AP001775.2	lncRNA	novel transc		11	134178824	134186166
ENSG0000001036	-0.25913528	0.238	0.38	FUZ	protein_coding	fuzzy planar		19	49806866	49817376
ENSG0000012297	-0.463070254	0.238	0.38	ACADS	protein_coding	acyl-CoA de		12	120725774	120740008
ENSG0000011001	0.219736285	0.238	0.38	DNAJC4	protein_coding	DnaJ heat sl		11	64230278	64234286
ENSG0000012273	0.998012228	0.238	0.38	DNAI1	protein_coding	dynein axor		9	34457414	34520988

ENSG0000019860	0.05918347	0.238	0.38	BAZ1A	protein_coding	bromodoma	14	34752731	34875647
ENSG0000022999	-0.71018706	0.238	0.38	RPL5P4	processed_pse	ribosomal p	1	35350722	35351607
ENSG0000016578	0.12738163	0.238	0.38	PIP4P1	protein_coding	phosphatidy	14	20457681	20461465
ENSG0000017054	0.72896043	0.238	0.38	SERPINB9	protein_coding	serpin famil	6	2887270	2903309
ENSG0000025231	1.958058365	0.238	0.38	RNU1-103I	snRNA	RNA, U1 sm	16	85781775	85781899
ENSG0000015453	-0.946598214	0.238	0.38	NA	NA	NA	NA	NA	NA
ENSG0000020495	-0.780557551	0.238	0.38	PCDHGA1	protein_coding	protocadhe	5	141330571	141512981
ENSG0000023571	-0.236027211	0.238	0.38	RXRB	protein_coding	retinoid X re	CHR_HSCHR	33363951	33371048
ENSG0000017940	0.315064243	0.238	0.38	LINC00174	lncRNA	long interge	7	66376044	66493566
ENSG0000010612	-0.329449385	0.238	0.38	MINDY4	protein_coding	MINDY lysir	7	30771417	30892387
ENSG0000001914	-0.090618351	0.239	0.38	PHLDB1	protein_coding	pleckstrin ho	11	118606440	118658031
ENSG0000013973	-0.133730635	0.239	0.38	DIAPH3	protein_coding	diaphanous	13	59665583	60163928
ENSG0000026260	0.495463146	0.239	0.38	NA	NA	NA	NA	NA	NA
ENSG0000027808	-0.907795551	0.239	0.38	RPS9	protein_coding	ribosomal p	CHR_HSCHR	54201473	54208260
ENSG0000008715	0.148526937	0.239	0.38	PGS1	protein_coding	phosphatidy	17	78378649	78425114
ENSG0000016877	0.33549158	0.239	0.38	TCTN2	protein_coding	tectonic fam	12	123671113	123708399
ENSG0000025872	0.297634108	0.239	0.38	AL135999.1	lncRNA	novel transc	14	23561097	23568073
ENSG0000026013	-1.193450992	0.239	0.38	AC008915.3	lncRNA	novel transc	16	23452758	23457606
ENSG0000025163	-0.399568625	0.239	0.38	NAIPP4	unprocessed_p	NAIP pseud	5	71102898	71128753
ENSG0000024740	-0.387973312	0.239	0.38	DNAJC3-D	lncRNA	DNAJC3 div	13	95648733	95676955
ENSG0000027215	-1.25387023	0.24	0.38	AC055822.1	lncRNA	novel transc	8	65714334	65714778
ENSG0000027635	0.254638172	0.24	0.38	PLEKHM1	protein_coding	pleckstrin ho	CHR_HSCHR	45435902	45490401
ENSG0000016368	0.13389274	0.24	0.38	RPP14	protein_coding	ribonuclease	3	58306247	58324695
ENSG0000027370	0.466053894	0.24	0.38	CDKN1C	protein_coding	cyclin deper	CHR_HSCHR	2883205	2885904
ENSG0000000517	0.092323826	0.24	0.38	RPAP3	protein_coding	RNA polyme	12	47661249	47706030
ENSG0000025864	-1.179992055	0.24	0.38	AL049780.1	lncRNA	novel transc	14	75004719	75008481
ENSG0000017003	0.098524717	0.24	0.38	UBE2E3	protein_coding	ubiquitin co	2	180967248	181076585
ENSG0000015480	-0.162596717	0.24	0.38	FLCN	protein_coding	folliculin [Sc	17	17212212	17237188
ENSG0000027244	1.368623942	0.241	0.38	AL118558.4	lncRNA	novel transc	14	101952416	101953063
ENSG0000027835	1.486241464	0.241	0.38	AC005911.1	lncRNA	novel transc	12	2885819	2886329
ENSG0000026012	-1.185996799	0.241	0.38	ULK4P2	transcribed_un	ULK4 pseud	15	30572738	30600647
ENSG0000007462	0.191828395	0.241	0.38	SLC24A1	protein_coding	solute carrie	15	65611366	65660995
ENSG0000026737	1.258626455	0.241	0.38	AC008752.4	unprocessed_p	zinc finger p	19	9756152	9756863
ENSG0000014250	0.121851866	0.242	0.38	PSMB6	protein_coding	proteasome	17	4796144	4798502
ENSG0000017691	-0.072496637	0.242	0.38	ANKLE2	protein_coding	ankyrin repe	12	132725503	132761832
ENSG0000027501	-2.209844487	0.242	0.38	AL353748.2	processed_pse	developmer	9	88391362	88391832
ENSG0000027370	1.97010588	0.242	0.38	U2	snRNA	U2 spliceoso	17	43241180	43241370
ENSG0000008541	0.111181717	0.242	0.39	SEH1L	protein_coding	SEH1 like nu	18	12947133	12987536
ENSG0000024300	-1.081049853	0.242	0.39	AL359538.1	lncRNA	novel transc	13	24566873	24597676
ENSG0000009100	-0.081595831	0.243	0.39	RBM27	protein_coding	RNA binding	5	146203605	146289223
ENSG0000021755	0.18753153	0.243	0.39	CKLF	protein_coding	chemokine l	16	66552563	66566251
ENSG0000009925	-0.612896705	0.243	0.39	HSD17B7P2	transcribed_un	hydroxyster	10	38356380	38378505
ENSG0000018681	0.129301496	0.243	0.39	TPCN1	protein_coding	two pore se	12	113221050	113298585
ENSG0000018434	-0.700862728	0.243	0.39	SRPK3	protein_coding	SRSF protei	X	153776412	153785732
ENSG0000011698	0.069976321	0.243	0.39	MTR	protein_coding	5-methyltet	1	236795292	236921278
ENSG0000011321	0.290887642	0.243	0.39	PCDHB6	protein_coding	protocadhe	5	141150022	141153287
ENSG0000020431	0.111627246	0.243	0.39	MRPL38	protein_coding	mitochondr	17	75898644	75905093
ENSG0000023112	1.109727957	0.243	0.39	AF129075.1	lncRNA	novel transc	21	29058073	29060095
ENSG0000025008	-1.377954939	0.243	0.39	AC025176.1	lncRNA	novel transc	5	65486444	65487048
ENSG0000002786	-0.414367761	0.243	0.39	SH2D2A	protein_coding	SH2 domair	1	156806243	156816848
ENSG0000017814	0.327982455	0.243	0.39	DALRD3	protein_coding	DALR antico	3	49015488	49022293
ENSG0000022363	0.47830231	0.243	0.39	CLIC1	protein_coding	chloride intr	CHR_HSCHR	31721342	31730525
ENSG0000011172	0.963678191	0.244	0.39	CLEC4A	protein_coding	C-type lecti	12	8123632	8138607
ENSG0000012538	-0.105507434	0.244	0.39	FAM193A	protein_coding	family with s	4	2536647	2732573
ENSG0000015734	-0.127778079	0.244	0.39	DDX19B	protein_coding	DEAD-box l	16	70289663	70335305
ENSG0000026593	1.609399342	0.244	0.39	UBE2CP2	processed_pse	ubiquitin co	18	22900486	22900995
ENSG0000016481	0.162442808	0.244	0.39	ORC5	protein_coding	origin recog	7	104126341	104208047
ENSG0000006997	-0.199096641	0.244	0.39	RAB27A	protein_coding	RAB27A, me	15	55202966	55319113

ENSG0000028007	0.356344213	0.244	0.39	AC011447.7	TEC	TEC	19	20220597	20222186
ENSG0000027185	0.758912754	0.244	0.39	AC073195.1	lncRNA	novel transc	2	9555899	9556775
ENSG0000018008	0.962820023	0.244	0.39	TMEM86B	protein_codinç	transmembı	19	55226638	55228784
ENSG0000027983	1.626435772	0.245	0.39	AC112694.2	TEC	novel transc	11	18601882	18602649
ENSG0000024659	0.699187728	0.245	0.39	AC139795.1	transcribed_un	SUMO inter	5	177619059	177672209
ENSG0000018639	0.192616384	0.245	0.39	KRT10	protein_codinç	keratin 10 [S	17	40818117	40822614
ENSG0000027949	0.888293205	0.245	0.39	AP003733.3	TEC	TEC	11	61967794	61969490
ENSG0000016769	-0.203588399	0.245	0.39	NXN	protein_codinç	nucleoredox	17	799310	979776
ENSG0000012407	-0.235960529	0.245	0.39	ENKD1	protein_codinç	enkurin don	16	67662945	67667265
ENSG0000023443	-0.476292555	0.245	0.39	AC092171.3	lncRNA	uncharacter	7	5426277	5428927
ENSG0000027022	-0.592160393	0.245	0.39	NA	NA	NA	NA	NA	NA
ENSG0000027784	1.089765906	0.245	0.39	SNORD30	snoRNA	small nuclec	11	62853663	62853732
ENSG0000018601	-0.221990631	0.245	0.39	NDUFA13	protein_codinç	NADH:ubiqu	19	19515736	19529054
ENSG0000020377	-0.32374718	0.246	0.39	SPRN	protein_codinç	shadow of p	10	133420666	133424572
ENSG0000015132	-0.090913327	0.246	0.39	AKAP6	protein_codinç	A-kinase an	14	32329298	32837684
ENSG0000014529	0.093213822	0.246	0.39	ENOPH1	protein_codinç	enolase-pho	4	82430590	82461177
ENSG0000010587	0.098316552	0.246	0.39	CBLL1	protein_codinç	Cbl proto-o	7	107743949	107761667
ENSG0000015777	0.190072396	0.246	0.39	PSMG3	protein_codinç	proteasome	7	1567332	1571005
ENSG0000018859	-0.26695981	0.246	0.39	NPIPP1	transcribed_un	nuclear pore	16	15104312	15123498
ENSG0000022901	0.544281752	0.246	0.39	PMS2P7	unprocessed_p	PMS1 homo	7	73005541	73021103
ENSG0000023933	-0.932812718	0.246	0.39	LINC01119	lncRNA	long interge	2	46816697	46859007
ENSG0000014465	0.260789159	0.246	0.39	CSRNP1	protein_codinç	cysteine anc	3	39141855	39154562
ENSG0000027257	-0.284653982	0.246	0.39	NA	NA	NA	NA	NA	NA
ENSG0000018760	-0.331238766	0.246	0.39	EXD3	protein_codinç	exonuclease	9	137306896	137423211
ENSG0000011678	-0.094672601	0.246	0.39	PLEKHM2	protein_codinç	pleckstrin ho	1	15684320	15734769
ENSG0000023712	-0.878901533	0.246	0.39	AC073254.1	lncRNA	novel transc	2	232580948	232611971
ENSG0000027131	1.808929047	0.247	0.39	AL161729.2	lncRNA	novel transc	9	95494924	95495379
ENSG0000017929	0.061706217	0.247	0.39	PTPN11	protein_codinç	protein tyro	12	112418351	112509918
ENSG0000027829	0.562594735	0.247	0.39	AL161772.1	lncRNA	novel transc	13	20699307	20703718
ENSG0000017507	-0.091958052	0.247	0.39	VCPIP1	protein_codinç	valosin cont	8	66628487	66667231
ENSG0000022749	1.350703679	0.247	0.39	AL138921.1	lncRNA	novel transc	10	100229629	100234398
ENSG0000009220	0.157918943	0.247	0.39	GEMIN2	protein_codinç	gem nuclea	14	39114223	39136973
ENSG0000012573	-0.146600874	0.247	0.39	GPR108	protein_codinç	G protein-c	19	6729914	6737603
ENSG0000013997	0.095104846	0.248	0.39	NAA30	protein_codinç	N-alpha-ac	14	57390544	57415906
ENSG0000025373	1.454664965	0.248	0.39	AC015909.2	lncRNA	novel transc	17	50158333	50161276
ENSG0000026719	0.466622687	0.248	0.39	NA	NA	NA	NA	NA	NA
ENSG0000022951	-1.9704849	0.248	0.39	AC068580.1	lncRNA	novel transc	11	1763009	1763749
ENSG0000021324	-0.10141924	0.248	0.39	SUPT4H1	protein_codinç	SPT4 homol	17	58345175	58353093
ENSG0000016511	-0.130446819	0.248	0.39	C9orf64	protein_codinç	chromosom	9	83938311	83956986
ENSG0000013256	0.590678402	0.249	0.39	MATN2	protein_codinç	matrilin 2 [S	8	97868840	98036724
ENSG0000021443	1.292665466	0.249	0.39	VPS33B-DT	lncRNA	VPS33B dive	15	91022619	91036611
ENSG0000026702	-0.256469472	0.249	0.39	LRRC37A16	transcribed_un	leucine rich	17	68125777	68152468
ENSG0000016613	0.130154018	0.249	0.39	IKBIP	protein_codinç	IKBKB intera	12	98613405	98645113
ENSG0000011359	0.117095521	0.249	0.39	TRAPPC13	protein_codinç	trafficking p	5	65625004	65666233
ENSG0000018711	0.325387761	0.249	0.39	CMC1	protein_codinç	C-X9-C mo	3	28241584	28325142
ENSG0000021269	-0.294084205	0.249	0.39	LINC01089	lncRNA	long interge	12	121795267	121803906
ENSG0000023035	1.207164872	0.249	0.39	STK19B	unprocessed_p	serine/threc	CHR_HSCHR	32089713	32090230
ENSG0000011544	-0.129400806	0.249	0.39	UNC50	protein_codinç	unc-50 inne	2	98608579	98618515
ENSG0000010645	0.144859506	0.249	0.39	NRF1	protein_codinç	nuclear resp	7	129611720	129757082
ENSG0000003796	-0.204708192	0.249	0.39	HOXC8	protein_codinç	homeobox (	12	54008985	54012769
ENSG0000023630	0.529053166	0.249	0.39	AL138921.2	lncRNA	novel transc	10	100190036	100190747
ENSG0000017157	-0.321801019	0.249	0.39	ZNF584	protein_codinç	zinc finger p	19	58401504	58418327
ENSG0000015460	-0.220478333	0.25	0.39	CEP170P1	transcribed_un	centrosoma	4	118467590	118554100
ENSG0000027806	-0.556972159	0.25	0.39	AC023491.1	pseudogene		KI270731.1	26533	27138
ENSG0000011203	0.174693444	0.25	0.39	FANCE	protein_codinç	FA complen	6	35452338	35467102
ENSG0000023689	-1.497942191	0.25	0.39	AL354726.1	lncRNA	novel transc	9	97986551	97987656
ENSG0000019707	-0.558597428	0.25	0.39	KIAA1671	protein_codinç	KIAA1671 [S	22	24952730	25197448
ENSG0000016547	0.472946882	0.25	0.39	CRYL1	protein_codinç	crystallin lar	13	20403666	20525873

ENSG0000021280	-0.166835568	0.25	0.39	RPL15P3	processed_pse	ribosomal p	6	12514110	12514724
ENSG0000007485	-0.148157392	0.25	0.4	ANO8	protein_coding	anoctamin 8	19	17323223	17334855
ENSG0000018395	-0.089288609	0.251	0.4	KMT5A	protein_coding	lysine methy	12	123383773	123409353
ENSG0000008219	-0.460518427	0.251	0.4	C1QTNF3	protein_coding	C1q and TN	5	34019448	34043832
ENSG0000011330	0.066402724	0.251	0.4	CNOT6	protein_coding	CCR4-NOT	5	180494379	180578358
ENSG0000014830	-0.117118939	0.251	0.4	REXO4	protein_coding	REX4 homo	9	133406059	133418096
ENSG0000013807	0.084086153	0.251	0.4	PREB	protein_coding	prolactin re	2	27130756	27134666
ENSG0000015855	-0.194523579	0.251	0.4	ZFAND2B	protein_coding	zinc finger A	2	219195237	219209651
ENSG0000015919	-1.352713061	0.251	0.4	KCNE2	protein_coding	potassium v	21	34364024	34371389
ENSG0000026874	1.576966586	0.251	0.4	AC008737.1	lncRNA	novel transc	19	16844025	16846473
ENSG0000018490	0.274420308	0.251	0.4	IMMP2L	protein_coding	inner mitoch	7	110662644	111562517
ENSG0000025895	-0.979320399	0.252	0.4	AL118558.1	transcribed_un	novel pseuc	14	102036315	102066228
ENSG0000024820	0.927386947	0.252	0.4	AC074194.1	lncRNA	novel transc	4	183987669	183996680
ENSG0000012587	-0.095696902	0.252	0.4	MGME1	protein_coding	mitochondr	20	17969018	17991122
ENSG0000023134	-0.98935702	0.252	0.4	ACTG1P10	processed_pse	actin gamm	X	53142832	53143913
ENSG0000007714	-0.055975437	0.252	0.4	TM9SF3	protein_coding	transmembr	10	96518110	96587452
ENSG0000008923	0.112245486	0.252	0.4	BRAP	protein_coding	BRCA1 asso	12	111642146	111685956
ENSG0000027382	1.60383921	0.252	0.4	AL096828.3	lncRNA	novel transc	20	63218041	63218502
ENSG0000013194	0.515748492	0.252	0.4	FAAP24	protein_coding	FA core con	19	32972209	32978229
ENSG0000023007	0.920406592	0.252	0.4	RPL4P6	processed_pse	ribosomal p	22	40586700	40588003
ENSG0000023436	-1.922011392	0.252	0.4	PFN1P3	processed_pse	profilin 1 ps	1	145407772	145408173
ENSG0000027313	0.570512477	0.252	0.4	AL022328.2	lncRNA	novel transc	22	50208461	50209542
ENSG0000025629	-0.271329423	0.252	0.4	ZNF225	protein_coding	zinc finger p	19	44112181	44134822
ENSG0000017805	-0.212200405	0.253	0.4	MLF1	protein_coding	myeloid leu	3	158571163	158607252
ENSG0000015276	0.444451901	0.253	0.4	WDR78	protein_coding	WD repeat c	1	66812885	66924856
ENSG0000025874	1.80827014	0.253	0.4	AL132800.1	lncRNA	novel transc	14	24443016	24508688
ENSG0000023182	0.634548433	0.253	0.4	SMC3P1	processed_pse	SMC3 pseuc	2	99102018	99102752
ENSG0000021385	0.519251367	0.253	0.4	KCTD11	protein_coding	potassium c	17	7351889	7354944
ENSG0000015846	0.172533849	0.253	0.4	AHCYL2	protein_coding	adenosylhor	7	129225023	129430211
ENSG0000017242	1.140389231	0.253	0.4	TTC36	protein_coding	tetratricope	11	118527472	118531197
ENSG0000026428	-0.703956022	0.253	0.4	AC016596.2	processed_pse	ribosomal p	5	55944656	55944733
ENSG0000026605	1.034965323	0.254	0.4	NDUFV2-A	lncRNA	NDUFV2 an	18	9121265	9136645
ENSG0000027290	0.483230832	0.254	0.4	AL122035.2	lncRNA	novel transc	14	64440369	64442238
ENSG0000019714	-0.661805819	0.254	0.4	ADAM32	protein_coding	ADAM meta	8	39106990	39284917
ENSG0000017547	-0.118188075	0.254	0.4	PPP2R2D	protein_coding	protein pho	10	131901008	131959834
ENSG0000020336	0.6375043	0.254	0.4	POLH-AS1	lncRNA	POLH antise	6	43588230	43591362
ENSG0000010647	0.128055012	0.254	0.4	ZNF862	protein_coding	zinc finger p	7	149838375	149867479
ENSG0000019631	0.301624931	0.254	0.4	MFSD14C	transcribed_un	major facilit	9	96887373	97013708
ENSG0000017452	0.336744273	0.254	0.4	TMEM81	protein_coding	transmembr	1	205083129	205084460
ENSG0000027860	-0.799587814	0.254	0.4	TSEN34	protein_coding	tRNA splicir	CHR_HSCHR	54190536	54194332
ENSG0000023599	-1.074824089	0.254	0.4	RPL23AP20	processed_pse	ribosomal p	1	241916123	241916543
ENSG0000016683	0.268517204	0.254	0.4	ANKDD1A	protein_coding	ankyrin repe	15	64911902	64958691
ENSG0000013537	0.100317276	0.255	0.4	NAT10	protein_coding	N-acetyltra	11	34105617	34147670
ENSG0000011499	0.221886104	0.255	0.4	RTKN	protein_coding	rhotekin [Sc	2	74425835	74442422
ENSG0000023936	2.491525858	0.255	0.4	RN7SL477P	misc_RNA	RNA, 7SL, c	19	1570575	1570860
ENSG0000008948	-0.181112224	0.255	0.4	CDIP1	protein_coding	cell death ir	16	4510669	4538828
ENSG0000024737	0.43749439	0.255	0.4	TMED2-DT	lncRNA	TMED2 dive	12	123575891	123585115
ENSG0000022890	1.46484234	0.255	0.4	AL353804.1	lncRNA	novel transc	X	73948973	73949558
ENSG0000014781	0.42106131	0.255	0.4	NAPRT	protein_coding	nicotinate p	8	143574785	143578649
ENSG0000026999	-1.453674957	0.256	0.4	NA	NA	NA	NA	NA	NA
ENSG0000023548	1.584436649	0.256	0.4	UBE2R2-AS	lncRNA	UBE2R2 ant	9	33775183	33818795
ENSG0000010315	0.218415476	0.256	0.4	MPG	protein_coding	N-methylpu	16	77007	85851
ENSG0000026906	-0.530298724	0.256	0.4	AC007842.1	processed_pse	protein asso	19	40023384	40025502
ENSG0000011003	0.359048567	0.256	0.4	LPXN	protein_coding	leupaxin [Sc	11	58526871	58578220
ENSG0000018659	-2.953463752	0.256	0.4	MIR22HG	lncRNA	MIR22 host	17	1711493	1717174
ENSG0000021010	-0.375811382	0.256	0.4	MT-TI	Mt_tRNA	mitochondr	MT	4263	4331
ENSG0000022432	0.122804452	0.256	0.4	HLA-A	protein_coding	major histo	CHR_HSCHR	29879141	29999464
ENSG0000027203	-1.051648406	0.256	0.4	AL162258.2	lncRNA	novel transc	1	153631438	153634397

ENSG0000027205	0.319173466	0.256	0.4	AC007390.1	lncRNA	novel transc		2	37208875	37212677
ENSG0000027357	-0.673757095	0.256	0.4	AC009283.1	lncRNA	novel transc		17	39566915	39567559
ENSG0000018646	-0.061211609	0.256	0.4	GNG2	protein_coding	G protein su		14	51826195	51979342
ENSG0000025925	1.764663898	0.256	0.4	AC022405.1	lncRNA	novel transc		15	40642933	40646857
ENSG0000022682	-1.008285128	0.257	0.4	HLA-DPB1	protein_coding	major histoc	CHR_HSCHR		33216374	33227649
ENSG0000027967	0.682330607	0.257	0.4	AP006621.5	TEC	TEC		11	779617	780755
ENSG0000023026	0.32284179	0.257	0.4	HERC2P4	transcribed_un	hect domair		16	32103245	32188107
ENSG0000028025	-2.095908452	0.257	0.4	NA	NA	NA	NA	NA	NA	
ENSG0000017737	0.31042014	0.257	0.4	TIMM22	protein_coding	translocase		17	997129	1003671
ENSG0000018618	0.107905676	0.257	0.4	ZNRF1	protein_coding	zinc and rin		16	74999024	75110994
ENSG0000000972	0.354707136	0.257	0.4	MASP2	protein_coding	mannan bin		1	11026523	11047239
ENSG0000027475	-1.398777591	0.257	0.4	AC243732.1	processed_pse	ribosomal p		17	36574462	36575325
ENSG0000001686	-0.144249715	0.258	0.4	GLT8D1	protein_coding	glycosyltran		3	52694488	52706032
ENSG0000023699	0.953700052	0.258	0.4	EDRF1-AS1	lncRNA	EDRF1 antis		10	125725634	125752110
ENSG0000000291	0.180457188	0.258	0.4	SNX11	protein_coding	sorting nexi		17	48103357	48123601
ENSG0000011965	-0.301754054	0.258	0.4	NPC2	protein_coding	NPC intrace		14	74476192	74494177
ENSG0000016270	0.208603271	0.258	0.4	CADM3	protein_coding	cell adhesio		1	159171609	159203313
ENSG0000023555	-0.583060229	0.258	0.4	AC146949.1	unprocessed_p	glucuronida		5	71220356	71259238
ENSG0000027954	-0.92495567	0.258	0.4	AC005261.5	TEC	TEC		19	57293083	57294469
ENSG0000026021	0.662638328	0.258	0.4	CD2BP2-DT	lncRNA	CD2BP2 div		16	30355441	30357104
ENSG0000021426	-1.218780695	0.259	0.4	AC124312.1	protein_coding	novel protei		15	24955034	25000276
ENSG0000010697	0.099268578	0.259	0.4	DNM1	protein_coding	dynamin 1 [		9	128191655	128255248
ENSG0000017818	-0.105326121	0.259	0.4	SH2B1	protein_coding	SH2B adapt		16	28846600	28874212
ENSG0000022767	0.147037775	0.259	0.41	AL390728.4	transcribed_un	zinc finger p		1	247189851	247210856
ENSG0000010660	-0.075199244	0.259	0.41	TMEM248	protein_coding	transmembr		7	66921225	66958551
ENSG0000014884	-0.490699761	0.259	0.41	ITPRIP	protein_coding	inositol 1,4,5		10	104309698	104338465
ENSG0000013623	-0.076073727	0.259	0.41	IGF2BP3	protein_coding	insulin like g		7	23310209	23470491
ENSG0000016713	-0.126301109	0.259	0.41	DOLPP1	protein_coding	dolichyldiph		9	129081111	129090438
ENSG0000017807	0.46635532	0.259	0.41	STAP2	protein_coding	signal transc		19	4324043	4342786
ENSG0000012006	-0.065423229	0.259	0.41	GNA13	protein_coding	G protein su		17	65009289	65056740
ENSG0000010069	0.0711358	0.26	0.41	DICER1	protein_coding	dicer 1, ribo		14	95086228	95158010
ENSG0000016380	-0.097550887	0.26	0.41	KIAA1143	protein_coding	KIAA1143 [S		3	44737661	44761619
ENSG0000023132	0.286351744	0.26	0.41	RXRB	protein_coding	retinoid X re	CHR_HSCHR		33347482	33354568
ENSG0000014279	-0.199127713	0.26	0.41	HSPG2	protein_coding	heparan sulf		1	21822244	21937310
ENSG0000022435	0.71491762	0.26	0.41	TMCO1-AS	lncRNA	TMCO1 anti		1	165768929	165775176
ENSG0000014932	-0.19063132	0.26	0.41	GLB1L2	protein_coding	galactosidas		11	134331874	134378341
ENSG0000016368	0.247273414	0.26	0.41	CFAP20DC	protein_coding	CFAP20 dor		3	58717365	59050084
ENSG0000018397	1.010182843	0.26	0.41	NPW	protein_coding	neuropeptic		16	2009926	2020755
ENSG0000027569	-1.904966277	0.26	0.41	UBE2Q2P6	transcribed_un	ubiquitin co		15	82445719	82454837
ENSG0000021419	-0.826970946	0.26	0.41	TTC41P	transcribed_un	tetratricope		12	103843749	103930211
ENSG0000027675	-1.56505004	0.26	0.41	MIR6821	miRNA	microRNA 6		22	49962866	49962939
ENSG0000019709	0.542825586	0.261	0.41	GAL3ST4	protein_coding	galactose-3		7	100159244	100168617
ENSG0000019757	-0.112703386	0.261	0.41	TOPORS	protein_coding	TOP1 bindir		9	32540544	32552586
ENSG0000020628	0.791471744	0.261	0.41	RING1	protein_coding	ring finger p	CHR_HSCHR		33137310	33141537
ENSG0000025115	-1.089910923	0.261	0.41	SEPTIN14P4	processed_pse	septin 14 ps		4	118640673	118640858
ENSG0000010634	-0.115437465	0.261	0.41	USP42	protein_coding	ubiquitin sp		7	6104884	6161564
ENSG0000023673	-0.623306883	0.261	0.41	CLIC4P1	processed_pse	chloride intr		9	22747700	22748234
ENSG0000011136	0.130620125	0.261	0.41	DDX55	protein_coding	DEAD-box l		12	123602077	123620943
ENSG0000019770	0.076872747	0.261	0.41	PARVA	protein_coding	parvin alpha		11	12377563	12535356
ENSG0000016548	0.141024432	0.261	0.41	SKA3	protein_coding	spindle and		13	21153595	21176552
ENSG0000024149	-0.186788628	0.261	0.41	AL355032.1	processed_pse	ribosomal p		14	101677943	101678380
ENSG0000017089	0.182008192	0.262	0.41	CYTL1	protein_coding	cytokine like		4	5014586	5019458
ENSG0000025674	-0.750036221	0.262	0.41	AP002784.2	processed_pse	actin relatec		11	94188449	94188997
ENSG0000027236	1.121012418	0.262	0.41	NA	NA	NA	NA	NA	NA	
ENSG0000017682	0.609622442	0.262	0.41	FKBP9P1	transcribed_un	FKBP prolyl		7	55681074	55713252
ENSG0000013449	0.235588201	0.263	0.41	TMEM241	protein_coding	transmembr		18	23197144	23437961
ENSG0000024061	0.848821976	0.263	0.41	RPS6P25	processed_pse	ribosomal p		19	12894133	12894880
ENSG0000012381	-0.455452417	0.263	0.41	B9D2	protein_coding	B9 domain c		19	41354417	41364165

ENSG0000026088	-0.660959299	0.263	0.41	AC009120.3	lncRNA	novel transc		16	74313337	74315634
ENSG0000027134	-1.228678022	0.263	0.41	AC018638.6	lncRNA	novel transc		7	128690451	128691717
ENSG0000026118	0.483610321	0.263	0.41	Z95115.1	lncRNA	novel transc		22	26512537	26514568
ENSG0000027109	0.692480196	0.263	0.41	TLCD4-RW	protein_coding	TLCD4-RWI		1	95117923	95247225
ENSG0000012437	-0.284259632	0.264	0.41	MCEE	protein_coding	methylmalo		2	71109684	71130239
ENSG0000026018	-1.058582853	0.264	0.41	LINC02137	lncRNA	long interge		16	58421326	58462470
ENSG0000016960	0.066069886	0.264	0.41	ANTXR1	protein_coding	ANTXR cell		2	69013178	69249327
ENSG0000022732	-0.954639115	0.264	0.41	AL139396.1	processed_pse	pseudogene	X		53164391	53164557
ENSG0000014791	-0.127078949	0.264	0.41	FBXO10	protein_coding	F-box prote		9	37510892	37576380
ENSG0000026491	1.179950831	0.264	0.41	NA	NA	NA	NA		NA	NA
ENSG0000021585	0.733797105	0.264	0.41	PDZK1P1	transcribed_un	PDZ domair		1	147993862	148014956
ENSG0000027296	-0.878811751	0.264	0.41	NA	NA	NA	NA		NA	NA
ENSG0000010432	-0.092578144	0.264	0.41	NBN	protein_coding	nibrin [Sour		8	89933331	90003228
ENSG0000014265	-0.055765844	0.264	0.41	PGD	protein_coding	phosphoglu		1	10398592	10420511
ENSG0000018044	-0.363642695	0.264	0.41	ARHGAP45	protein_coding	Rho GTPase		19	1065923	1086628
ENSG0000011297	-0.071275256	0.264	0.41	DAP	protein_coding	death assoc		5	10679230	10761234
ENSG0000027699	-1.373694584	0.264	0.41	AL513314.2	lncRNA	novel transc		1	222477252	222504622
ENSG0000027652	-0.547880165	0.265	0.41	AP001505.1	lncRNA	novel transc		21	44978832	44979274
ENSG0000026013	2.00238279	0.265	0.41	AL032819.1	lncRNA	novel transc		16	1408834	1412248
ENSG0000010551	-0.13985631	0.265	0.41	TMEM205	protein_coding	transmembi		19	11342776	11346518
ENSG0000025641	-0.480300112	0.265	0.41	AC006206.2	lncRNA	novel transc		12	5290480	5383653
ENSG0000009994	-0.107035781	0.265	0.41	LZTR1	protein_coding	leucine zipp		22	20982269	20999032
ENSG0000023188	-1.581330466	0.265	0.41	PRH1	protein_coding	proline rich		12	10824960	11171608
ENSG0000003238	0.119011985	0.265	0.41	EIPR1	protein_coding	EARP comp		2	3188925	3377830
ENSG0000019715	-0.051797218	0.265	0.41	SND1	protein_coding	staphylococ		7	127652194	128092609
ENSG0000020037	-2.950269146	0.265	0.41	RNU5E-10P	snRNA	RNA, U5E sr		11	47576471	47576588
ENSG0000027824	0.235643891	0.265	0.41	TSTA3	protein_coding	tissue specif	CHR_HSCHR		143612618	143618048
ENSG0000010882	0.136082171	0.265	0.41	MRPL27	protein_coding	mitochondr		17	50367857	50373207
ENSG0000016416	-0.05496584	0.265	0.41	ABCE1	protein_coding	ATP binding		4	145098288	145129524
ENSG0000016299	0.263500401	0.266	0.41	DUSP19	protein_coding	dual specific		2	183078559	183100008
ENSG0000024772	1.031184126	0.266	0.41	AC091057.3	lncRNA	novel transc		15	30616998	30625773
ENSG0000024272	-1.227594302	0.266	0.41	RPP21	protein_coding	ribonucleas	CHR_HSCHR		30318794	30336373
ENSG0000025943	0.194855613	0.266	0.41	THTPA	protein_coding	thiamine tri		14	23555988	23560271
ENSG0000012582	-0.146530428	0.266	0.41	RBCK1	protein_coding	RANBP2-typ		20	407498	432139
ENSG0000016433	0.120720508	0.266	0.41	UTP15	protein_coding	UTP15 smal		5	73565443	73583380
ENSG0000023064	1.468044754	0.266	0.41	AL138831.1	lncRNA	novel transc		6	4018843	4021215
ENSG0000002629	-0.364933449	0.266	0.41	RNASET2	protein_coding	ribonucleas		6	166922113	166957191
ENSG0000024652	0.462206684	0.266	0.41	AC079089.1	lncRNA	novel transc		8	69834111	69854971
ENSG0000016911	-0.447256337	0.267	0.41	PARM1	protein_coding	prostate and		4	74933095	75050115
ENSG0000019792	0.502989622	0.267	0.41	C2orf27A	transcribed_un	chromosom		2	131722417	131751669
ENSG0000021338	-0.92245666	0.267	0.41	AC104297.1	processed_pse	pyrophosph		3	17871729	17872598
ENSG0000012665	-0.133088726	0.267	0.41	NSRP1	protein_coding	nuclear spe		17	30115521	30186475
ENSG0000021476	0.189938396	0.267	0.41	SEPTIN7P2	transcribed_un	septin 7 pse		7	45723780	45768985
ENSG0000022936	0.187258087	0.267	0.41	NELFE	protein_coding	negative elc	CHR_HSCHR		32028530	32035535
ENSG0000024397	0.574366938	0.267	0.41	PPIEL	transcribed_un	peptidylprol		1	39522280	39559671
ENSG0000007635	-0.300451761	0.267	0.41	SLC46A1	protein_coding	solute carrie		17	28394642	28407197
ENSG0000016012	-0.162670176	0.267	0.41	CCDC58	protein_coding	coiled-coil c		3	122359591	122383231
ENSG0000020647	-0.202470831	0.267	0.41	VARS2	protein_coding	valyl-tRNA s	CHR_HSCHR		30903778	30916043
ENSG0000014693	-0.175557552	0.267	0.41	NLGN4X	protein_coding	neuroligin 4	X		5840637	6228867
ENSG0000023253	1.007692439	0.267	0.41	AC093673.1	lncRNA	novel transc		7	143379692	143380495
ENSG0000022870	1.016425354	0.267	0.42	TNKS2-AS1	lncRNA	TNKS2 antis		10	91782835	91798304
ENSG0000027822	1.872534943	0.267	0.42	PRICKLE4	protein_coding	prickle plan		6	41780782	41787452
ENSG0000021196	1.257530467	0.267	0.42	IGHV1-46	IG_V_gene	immunoglol		14	106511117	106511856
ENSG0000026713	2.068627351	0.267	0.42	AC005954.1	lncRNA	novel transc		19	3754620	3756659
ENSG0000023404	0.298598413	0.267	0.42	TRIM26	protein_coding	tripartite mc	CHR_HSCHR		30174807	30203783
ENSG0000025622	-0.134450165	0.267	0.42	ZNF486	protein_coding	zinc finger p		19	20167214	20200488
ENSG0000010195	-0.482160742	0.268	0.42	SRPX	protein_coding	sushi repeat	X		38149336	38220924
ENSG0000018191	-0.087021146	0.268	0.42	ADO	protein_coding	2-aminoeth		10	62804720	62808479

ENSG0000024229	-0.262857868	0.268	0.42	AC073861.1	processed_pse	ribosomal p	3	101576489	101576947
ENSG0000022776	-0.35132199	0.268	0.42	BAG6	protein_coding	BAG cochap	CHR_HSCHR	31621198	31634883
ENSG0000018283	0.067846093	0.268	0.42	C16orf72	protein_coding	chromosom	16	9091644	9121635
ENSG0000017893	0.380545808	0.268	0.42	ZNF552	protein_coding	zinc finger p	19	57803841	57814913
ENSG0000006692	0.243194774	0.268	0.42	STAG3	protein_coding	stromal anti	7	100177563	100221488
ENSG0000013837	-0.539201052	0.268	0.42	STAT4	protein_coding	signal transc	2	191029576	191151596
ENSG0000023264	1.178693716	0.268	0.42	AC107214.1	lncRNA	novel transc	4	183494884	183504494
ENSG0000018231	-0.281995663	0.269	0.42	ZSCAN22	protein_coding	zinc finger a	19	58326994	58342332
ENSG0000019885	-0.144565635	0.269	0.42	RUSC2	protein_coding	RUN and SH	9	35490111	35561898
ENSG0000011804	-0.103531959	0.269	0.42	STK11	protein_coding	serine/threo	19	1177558	1228431
ENSG0000021307	-0.438778028	0.269	0.42	NA	NA	NA	NA	NA	NA
ENSG0000027292	1.43768634	0.269	0.42	AC107464.2	lncRNA	novel transc	4	661209	661945
ENSG0000017260	0.860973792	0.269	0.42	RND1	protein_coding	Rho family (	12	48857145	48865870
ENSG0000011253	-0.060516612	0.269	0.42	QKI	protein_coding	QKI, KH dor	6	163414000	163578592
ENSG0000027187	-0.7510584	0.269	0.42	AC024060.2	lncRNA	novel transc	3	3152942	3153435
ENSG0000022774	0.759457883	0.269	0.42	AL121987.2	lncRNA	novel transc	1	160202199	160208869
ENSG0000021317	-0.112664998	0.269	0.42	RPL22P1	processed_pse	ribosomal p	3	169483671	169484080
ENSG0000003667	-0.329744914	0.27	0.42	USP2	protein_coding	ubiquitin sp	11	119355215	119381711
ENSG0000012459	0.143456314	0.27	0.42	OARD1	protein_coding	O-acyl-ADP	6	41033627	41097787
ENSG0000013047	-0.347011074	0.27	0.42	FCHO1	protein_coding	FCH and mu	19	17747718	17788568
ENSG0000018525	-0.543115801	0.27	0.42	PPIL6	protein_coding	peptidylprol	6	109390215	109441171
ENSG0000023456	2.418905471	0.27	0.42	AL024497.2	lncRNA	novel transc	6	133452857	133456605
ENSG0000026768	-0.960605706	0.27	0.42	AC135721.1	transcribed_pr	high mobilit	17	43144956	43145255
ENSG0000001337	-0.07797487	0.27	0.42	PGM3	protein_coding	phosphoglu	6	83161150	83193936
ENSG0000005969	-0.14735362	0.27	0.42	GATB	protein_coding	glutamyl-tR	4	151670504	151761007
ENSG0000018392	-1.086156793	0.27	0.42	DUSP5P1	processed_pse	dual specific	1	228650241	228651379
ENSG0000027777	1.753624516	0.27	0.42	H3C7	protein_coding	H3 clustered	6	26250142	26250635
ENSG0000013997	-0.278452334	0.27	0.42	SLC38A6	protein_coding	solute carrie	14	60981114	61083733
ENSG0000027220	1.363346633	0.27	0.42	AL451050.2	lncRNA	novel transc	1	167219831	167220512
ENSG0000023508	1.619103773	0.27	0.42	SUMO1P3	processed_pse	SUMO1 pse	1	160317403	160317706
ENSG0000010421	-0.425593984	0.27	0.42	PDGFRL	protein_coding	platelet deri	8	17576433	17644071
ENSG0000015832	-0.073805458	0.27	0.42	AUTS2	protein_coding	activator of	7	69598296	70793506
ENSG0000027346	-0.709869008	0.27	0.42	AC012510.1	lncRNA	novel transc	2	218633256	218634014
ENSG0000022639	-1.20252275	0.271	0.42	AL031727.1	processed_pse	ribosomal p	1	19608114	19608568
ENSG0000019878	0.421684759	0.271	0.42	GRIN3A	protein_coding	glutamate io	9	101569352	101738647
ENSG0000016551	0.091576126	0.271	0.42	ZNF22	protein_coding	zinc finger p	10	45000923	45005326
ENSG0000024137	-0.330212814	0.271	0.42	RPP21	protein_coding	ribonuclease	6	30345131	30346884
ENSG0000015157	0.277170912	0.271	0.42	TEX9	protein_coding	testis expres	15	56244009	56445997
ENSG0000025166	-1.208389927	0.271	0.42	BRCC3P1	processed_pse	BRCA1/BRC	5	176308063	176309013
ENSG0000025347	0.519007646	0.271	0.42	AC012213.1	lncRNA	novel transc	8	103483398	103501676
ENSG0000016419	0.062149332	0.271	0.42	NIPBL	protein_coding	NIPBL cohes	5	36876769	37066413
ENSG0000018762	0.618862699	0.271	0.42	C17orf97	protein_coding	chromosom	17	410325	431062
ENSG0000026625	-1.290390859	0.271	0.42	LINC01909	lncRNA	long interge	18	70335439	70352459
ENSG0000008587	0.077911933	0.271	0.42	CHERP	protein_coding	calcium hor	19	16517894	16542437
ENSG0000016484	-0.686125666	0.271	0.42	TMEM74	protein_coding	transmembr	8	108606850	108787594
ENSG0000027532	1.882837819	0.271	0.42	RPS9	protein_coding	ribosomal p	CHR_HSCHR	54201473	54208260
ENSG0000020235	2.346884482	0.271	0.42	RNU6-652P	snRNA	RNA, U6 sm	4	55885595	55885701
ENSG0000022804	-0.752414536	0.271	0.42	POLR2J2	protein_coding	RNA polyme	7	102665368	102671629
ENSG0000023265	-1.189856778	0.271	0.42	IDI2-AS1	lncRNA	IDI2 antisen	10	1022630	1045425
ENSG0000014074	-0.061789846	0.272	0.42	UQCRC2	protein_coding	ubiquinol-cy	16	21953288	21983660
ENSG0000015488	0.288296805	0.272	0.42	MPPE1	protein_coding	metallophos	18	11882622	11908366
ENSG0000027422	-0.774660723	0.272	0.42	AC073575.2	lncRNA	novel transc	12	112018804	112019430
ENSG0000024088	-0.845519866	0.272	0.42	NDUFB2-A	lncRNA	NDUFB2 an	7	140695336	140697077
ENSG0000023560	-1.189577772	0.272	0.42	AL355472.1	processed_pse	ribosomal p	1	234356704	234357141
ENSG0000009999	0.306444741	0.272	0.42	RNF215	protein_coding	ring finger p	22	30368811	30421771
ENSG0000015880	-0.122930113	0.272	0.42	ZNF276	protein_coding	zinc finger p	16	89720400	89740925
ENSG0000027723	1.066459795	0.272	0.42	GTSE1-DT	lncRNA	GTSE1 diver	22	46295143	46296660
ENSG0000027934	0.744596535	0.272	0.42	AP000866.6	lncRNA	novel transc	11	124789240	124792818

ENSG0000023714	-0.616214427	0.272	0.42	ZNF503-AS	lncRNA	ZNF503 ant	10	75401519	75408982
ENSG0000010992	0.313287603	0.272	0.42	TECTA	protein_coding	tectorin alpl	11	121101173	121191493
ENSG0000013448	0.130448711	0.272	0.42	CCNH	protein_coding	cyclin H [So	5	87318416	87412930
ENSG0000022934	-1.365816029	0.273	0.42	HYI-AS1	lncRNA	HYI antisens	1	43453927	43456995
ENSG0000024325	-0.679336474	0.273	0.42	NA	NA	NA	NA	NA	NA
ENSG0000025865	-1.019165286	0.273	0.42	AC005520.	protein_coding	novel protei	14	73851971	73932278
ENSG0000012027	-0.752005425	0.273	0.42	MYCT1	protein_coding	MYC target	6	152697895	152724567
ENSG0000022623	1.17172313	0.273	0.42	NPIPB14P	transcribed_un	nuclear pore	16	69976388	69996188
ENSG0000025842	0.882537129	0.273	0.42	AL512791.1	lncRNA	novel transc	14	90402523	90405235
ENSG0000021530	0.141598594	0.273	0.42	VPS16	protein_coding	VPS16 core	20	2840703	2866732
ENSG0000016652	-0.085076027	0.273	0.42	ZNF3	protein_coding	zinc finger p	7	100064033	100082548
ENSG0000025946	-2.027060606	0.273	0.42	AC078909.	lncRNA	novel transc	15	37099339	37100173
ENSG0000027300	0.500684701	0.273	0.42	AL078644.1	lncRNA	novel transc	1	185317779	185318530
ENSG0000027520	0.851355127	0.273	0.42	AL161421.1	lncRNA	novel transc	13	48974967	48976867
ENSG0000025936	-1.232587013	0.273	0.42	AC090825.	lncRNA	novel transc	15	99805793	99916818
ENSG0000013918	-0.086310918	0.274	0.42	NDUFA9	protein_coding	NADH:ubiqu	12	4649095	4694317
ENSG0000016403	-0.081864955	0.274	0.42	DNAJB14	protein_coding	DnaJ heat sl	4	99896248	99946618
ENSG0000016754	-0.389301024	0.274	0.42	CORO6	protein_coding	coronin 6 [S	17	29614756	29622907
ENSG0000022171	-2.932465848	0.274	0.42	SNORA11	snoRNA	small nucle	X	54814370	54814497
ENSG0000027201	1.139201823	0.274	0.42	AC100814.	lncRNA	novel transc	8	65591850	65592472
ENSG0000026164	0.730398334	0.274	0.42	NA	NA	NA	NA	NA	NA
ENSG0000017265	0.358764271	0.274	0.42	AGAP5	protein_coding	ArfGAP with	10	73674285	73698159
ENSG0000010198	0.216689925	0.275	0.42	ABCD1	protein_coding	ATP binding	X	153724856	153744755
ENSG0000010837	0.392168438	0.275	0.42	WNT3	protein_coding	Wnt family r	17	46762506	46833154
ENSG0000012190	-1.034354354	0.275	0.42	HPCA	protein_coding	hippocalcin	1	32885994	32898441
ENSG0000016730	0.183814948	0.275	0.42	TEPSIN	protein_coding	TEPSIN ada	17	81228277	81239091
ENSG0000018533	0.817707068	0.275	0.42	SOCS1	protein_coding	suppressor	16	11254408	11256204
ENSG0000027951	-0.397983237	0.275	0.42	AC007382.	TEC	TEC	2	36839922	36842539
ENSG0000012732	0.324184505	0.275	0.42	RAB3IP	protein_coding	RAB3A inter	12	69738860	69823204
ENSG0000023783	-0.841678679	0.275	0.42	PHKA2-AS	lncRNA	PHKA2 antis	X	18890296	18894974
ENSG0000023055	0.64220809	0.275	0.42	AL450326.1	lncRNA	novel transc	10	43420738	43422100
ENSG0000024932	-0.798638957	0.275	0.42	AC036214.	lncRNA	novel transc	8	79769372	79871759
ENSG0000010304	-0.1899662	0.276	0.42	TANGO6	protein_coding	transport ar	16	68843531	69085182
ENSG0000014253	-0.906690511	0.276	0.42	FAM71E1	protein_coding	family with	19	50466785	50476848
ENSG0000014458	-0.18098104	0.276	0.42	STK11IP	protein_coding	serine/threc	2	219597857	219616451
ENSG0000027173	1.441343976	0.276	0.42	AL390208.1	lncRNA	novel transc	6	108998482	108999125
ENSG0000027864	-0.142470563	0.276	0.42	PCGF2	protein_coding	polycomb g	CHR_HSCHR	38534684	38550604
ENSG0000017195	0.198360916	0.276	0.42	ATPAF2	protein_coding	ATP synthas	17	17977409	18039209
ENSG0000010319	0.205070597	0.276	0.42	ZNF500	protein_coding	zinc finger p	16	4748239	4767624
ENSG0000018297	-0.136757635	0.276	0.43	CNOT10	protein_coding	CCR4-NOT	3	32685145	32773875
ENSG0000016852	-0.072640801	0.276	0.43	FNTA	protein_coding	farnesyltran	8	43034194	43085788
ENSG0000016260	0.086916432	0.277	0.43	MYSM1	protein_coding	Myb like, SW	1	58643440	58700077
ENSG0000018527	0.851876276	0.277	0.43	CD24P4	processed_pse	CD24 molec	Y	18992467	18992709
ENSG0000027899	1.085812452	0.277	0.43	AL662907.1	TEC	TEC	1	33141871	33143230
ENSG0000022537	-0.215680481	0.277	0.43	NA	NA	NA	NA	NA	NA
ENSG0000017171	0.22205614	0.277	0.43	ANO5	protein_coding	anoctamin 5	11	21799934	22283357
ENSG0000026693	-1.338135129	0.277	0.43	AC005775.	lncRNA	novel transc	19	490046	507833
ENSG0000013773	-0.671036051	0.277	0.43	FXYD2	protein_coding	FXYD doma	11	117800844	117828698
ENSG0000023799	0.467135538	0.278	0.43	RPL35P1	processed_pse	ribosomal p	1	236981339	236981708
ENSG0000016464	-0.071810489	0.278	0.43	CDCA7L	protein_coding	cell division	7	21900899	21945903
ENSG0000022459	1.636416568	0.278	0.43	RPL29P19	processed_pse	ribosomal p	8	48384590	48385049
ENSG0000001861	0.133108368	0.278	0.43	CXorf56	protein_coding	chromosom	X	119538149	119565409
ENSG0000023184	1.652425008	0.278	0.43	AC073342.	lncRNA	novel transc	7	143255264	143287997
ENSG0000020415	0.143948178	0.278	0.43	TIMM23B	protein_coding	translocase	10	49942049	49974850
ENSG0000013313	0.268731731	0.278	0.43	MORC4	protein_coding	MORC fami	X	106813871	107000212
ENSG0000023407	-0.290441807	0.278	0.43	DDR1	protein_coding	discoidin dc	CHR_HSCHR	30925624	30944801
ENSG0000013860	0.116404038	0.278	0.43	SPPL2A	protein_coding	signal pepti	15	50702266	50765709
ENSG0000027765	0.353924549	0.279	0.43	GSTT1	protein_coding	glutathione	CHR_HSCHR	24033952	24042493

ENSG0000011526	-0.090753358	0.279	0.43	APC2	protein_coding	APC regulat	19	1446302	1473244
ENSG0000024404	-0.790498785	0.279	0.43	LINC01011	lncRNA	long interge	6	2987967	2991173
ENSG0000025124	-1.376681232	0.279	0.43	AL691442.2	protein_coding	novel ephrir	1	155063748	155086807
ENSG0000015611	0.091995016	0.279	0.43	ADK	protein_coding	adenosine k	10	74151202	74709963
ENSG0000018347	0.207904368	0.279	0.43	GTF2H2C	protein_coding	GTF2H2 fam	5	69560208	69594723
ENSG0000014829	-0.103172584	0.279	0.43	SURF6	protein_coding	surfeit 6 [So	9	133328776	133336188
ENSG0000022047	-0.177187024	0.279	0.43	AL139095.2	processed_pse	ribosomal p	6	7338839	7339568
ENSG0000024908	0.30447186	0.279	0.43	ZNF436-AS	lncRNA	ZNF436 ant	1	23368997	23371839
ENSG0000010145	0.136970594	0.279	0.43	DNTTIP1	protein_coding	deoxynucle	20	45791954	45811427
ENSG0000018695	0.636688936	0.28	0.43	TMEM232	protein_coding	transmembr	5	110289233	110738956
ENSG0000022869	0.328185781	0.28	0.43	NEU1	protein_coding	neuraminida	CHR_HSCHR	31924682	31929929
ENSG0000016206	-0.651991089	0.28	0.43	NTN3	protein_coding	netrin 3 [So	16	2471297	2474145
ENSG0000020405	-0.612384134	0.28	0.43	LRRC73	protein_coding	leucine rich	6	43506969	43510686
ENSG0000023501	0.590602612	0.28	0.43	SEMA3F-AS	lncRNA	SEMA3F ant	3	50116022	50156085
ENSG0000011957	-0.280703811	0.281	0.43	ZBTB45	protein_coding	zinc finger a	19	58513530	58538911
ENSG0000014145	0.098889611	0.281	0.43	PELP1	protein_coding	proline, glut	17	4669774	4704337
ENSG0000016708	0.06759934	0.281	0.43	PHB	protein_coding	prohibitin [S	17	49404049	49414905
ENSG0000026340	-0.949722948	0.281	0.43	TMEM220-	lncRNA	TMEM220 a	17	10729777	10815164
ENSG0000013693	0.164575371	0.281	0.43	XPA	protein_coding	XPA, DNA d	9	97674909	97697340
ENSG0000012925	-0.141704442	0.281	0.43	MPDU1	protein_coding	mannose-P	17	7583529	7592789
ENSG0000018009	-0.185550837	0.281	0.43	TRNAU1AP	protein_coding	tRNA selenc	1	28553085	28578545
ENSG0000019804	0.191301682	0.281	0.43	ZNF667	protein_coding	zinc finger p	19	56439325	56478065
ENSG0000017810	0.082535159	0.281	0.43	DDX10	protein_coding	DEAD-box l	11	108665069	108940927
ENSG0000018899	0.148725718	0.281	0.43	KCTD21	protein_coding	potassium c	11	78171249	78188626
ENSG0000027288	1.393976017	0.281	0.43	AC092574.1	lncRNA	novel transc	4	416118	416537
ENSG0000027467	-0.829256388	0.281	0.43	TSEN34	protein_coding	tRNA splicir	CHR_HSCHR	54190866	54195141
ENSG0000012935	-0.037191005	0.282	0.43	ILF3	protein_coding	interleukin e	19	10654261	10692417
ENSG0000014655	0.258776986	0.282	0.43	WASH2P	transcribed_un	WASP family	2	113588550	113599043
ENSG0000009154	-0.089478716	0.282	0.43	ALKBH5	protein_coding	alkB homolo	17	18183078	18209954
ENSG0000022409	0.86269181	0.282	0.43	AC097468.1	lncRNA	novel transc	2	219002215	219015721
ENSG0000025362	-2.447494156	0.282	0.43	AC144568.1	processed_pse	family with s	8	64091	64320
ENSG0000014354	-0.106901544	0.282	0.43	RAB13	protein_coding	RAB13, mer	1	153981617	153986358
ENSG0000025917	0.730869545	0.282	0.43	AC023024.1	lncRNA	novel transc	15	101295419	101305737
ENSG0000027586	0.879117726	0.282	0.43	AC136612.1	protein_coding	TP53-target	KI270728.1	933862	936467
ENSG0000017402	0.104065136	0.283	0.43	GNG5	protein_coding	G protein su	1	84498325	84506581
ENSG0000025851	0.468219761	0.283	0.43	AL355075.2	lncRNA	novel transc	14	20451305	20451918
ENSG0000027087	0.814481211	0.283	0.43	AC015849.3	lncRNA	novel transc	17	35816717	35830293
ENSG0000017592	-0.27433822	0.283	0.43	LRRN1	protein_coding	leucine rich	3	3799431	3849834
ENSG0000020562	0.138466686	0.283	0.43	LCMT1	protein_coding	leucine carb	16	25111731	25178231
ENSG0000015136	1.11637545	0.283	0.43	KCTD14	protein_coding	potassium c	11	78015715	78046191
ENSG0000025477	-0.619046606	0.283	0.43	NA	NA	NA	NA	NA	NA
ENSG0000006950	-0.194797722	0.283	0.43	FUNDC1	protein_coding	FUN14 dom	X	44523639	44542859
ENSG0000027481	2.890813871	0.283	0.43	NPHP3-AC	protein_coding	NPHP3-ACA	3	132558142	132722459
ENSG0000027950	-1.701702386	0.284	0.43	AC112484.4	TEC	TEC	3	128914833	128915060
ENSG0000017610	0.128093684	0.284	0.43	SSNA1	protein_coding	SS nuclear a	9	137188660	137190370
ENSG0000027708	0.312792531	0.284	0.43	MTMR10	protein_coding	myotubulari	CHR_HSCHR	31092912	31142971
ENSG0000019932	2.027886897	0.284	0.43	RNU4-39P	snRNA	RNA, U4 sm	11	66614964	66615090
ENSG0000008463	-0.563619414	0.284	0.43	COL16A1	protein_coding	collagen typ	1	31652263	31704319
ENSG0000019717	0.074621358	0.284	0.43	PSMD12	protein_coding	proteasome	17	67337916	67366605
ENSG0000016334	0.128146585	0.284	0.44	PBXIP1	protein_coding	PBX homeo	1	154944076	154956123
ENSG0000012905	0.100893724	0.285	0.44	ANAPC13	protein_coding	anaphase pr	3	134477706	134486716
ENSG0000021583	0.338318385	0.285	0.44	AL596087.1	processed_pse	Ewing sarco	1	166275629	166277597
ENSG0000024208	-1.188643618	0.285	0.44	SLC5A4-AS	lncRNA	SLC5A4 ant	22	32205115	32278382
ENSG0000017408	-0.127133306	0.285	0.44	CTSF	protein_coding	cathepsin F	11	66563464	66568841
ENSG0000020649	-0.720653354	0.285	0.44	HLA-E	protein_coding	major histoc	CHR_HSCHR	30478970	30483708
ENSG0000021590	-0.160913855	0.285	0.44	CROCCP2	transcribed_un	CROCC pse	1	16618253	16657232
ENSG0000017997	-0.394269324	0.286	0.44	NAIPP2	unprocessed_p	NAIP pseud	5	70094659	70128434
ENSG0000016276	0.094396969	0.286	0.44	FLVCR1	protein_coding	FLVCR hem	1	212858275	212899363

ENSG0000015315	-0.757169442	0.286	0.44	SYCP2L	protein_coding	synaptonem	6	10886831	10979320
ENSG0000011850	-0.827314218	0.286	0.44	AKAP7	protein_coding	A-kinase an	6	131135467	131283535
ENSG0000027691	2.098373943	0.286	0.44	AP001107.1	misc_RNA		11	66374138	66374435
ENSG0000017659	-0.307246629	0.286	0.44	AC008969.1	lncRNA	novel transc	19	58002061	58011232
ENSG0000023682	-0.854808625	0.286	0.44	Z97634.1	transcribed_pr	ribosomal p	16	382097	392960
ENSG0000022520	-0.475417466	0.286	0.44	AC246787.1	processed_pse	ribosomal p	14	105978520	105979146
ENSG0000026670	0.686933155	0.286	0.44	AC005224.1	lncRNA	novel transc	17	14303854	14305505
ENSG0000016414	0.097639491	0.286	0.44	ARFIP1	protein_coding	ADP ribosyl	4	152779937	152918463
ENSG0000017217	0.241656953	0.286	0.44	TEFM	protein_coding	transcriptior	17	30897336	30906238
ENSG0000018824	0.163545957	0.286	0.44	COMMD6	protein_coding	COMM don	13	75525219	75549439
ENSG0000014602	0.243680342	0.286	0.44	KLHL3	protein_coding	kelch like fa	5	137617500	137736089
ENSG0000021223	0.850287573	0.286	0.44	SNORD17	snoRNA	small nucleo	20	17962710	17962946
ENSG0000022819	0.682768809	0.286	0.44	AL512353.1	lncRNA	novel transc	1	42832522	42846422
ENSG0000012412	0.185251862	0.287	0.44	PREX1	protein_coding	phosphatidy	20	48624252	48827999
ENSG0000018291	0.174901468	0.287	0.44	TCEAL7	protein_coding	transcriptior	X	103330229	103332326
ENSG0000020648	0.37173085	0.287	0.44	ATAT1	protein_coding	alpha tubuli	CHR_HSCHR	30616337	30636318
ENSG0000025291	1.831676023	0.287	0.44	Y_RNA	misc_RNA	Y RNA [Sou	21	39344537	39344628
ENSG0000010476	0.069677954	0.287	0.44	ASAH1	protein_coding	N-acylsphin	8	18055992	18084998
ENSG0000023422	0.727151742	0.287	0.44	LIX1L-AS1	lncRNA	LIX1L antise	1	145926590	145959179
ENSG0000028014	-2.035672621	0.288	0.44	NA	NA	NA	NA	NA	NA
ENSG0000025453	0.387685346	0.288	0.44	FLJ20021	lncRNA	uncharacter	4	101347780	101348883
ENSG0000025830	0.929984316	0.288	0.44	AC025034.1	lncRNA	novel transc	12	89561129	89594878
ENSG0000017886	-0.272499563	0.288	0.44	MSC	protein_coding	musculin [Sc	8	71841549	71844468
ENSG0000015294	-0.118328616	0.288	0.44	RAD17	protein_coding	RAD17 chec	5	69369293	69414801
ENSG0000017368	-0.213755745	0.289	0.44	BCLAF3	protein_coding	BCLAF1 anc	X	19912860	19970298
ENSG0000021497	-0.45345449	0.289	0.44	PPIAP29	processed_pse	peptidylprol	6	24976419	24976982
ENSG0000025546	1.498804967	0.289	0.44	ZDHHC20P	processed_pse	zinc finger D	11	75228322	75228657
ENSG0000023393	0.334647172	0.289	0.44	CTC-338M	lncRNA	uncharacter	5	181246507	181272167
ENSG0000016291	0.709879761	0.289	0.44	OBSCN-AS	lncRNA	OBSCN anti	1	228203503	228213664
ENSG0000022461	-0.258477954	0.289	0.44	HCG18	lncRNA	HLA comple	CHR_HSCHR	30280729	30317069
ENSG0000010489	0.442704619	0.289	0.44	CD37	protein_coding	CD37 molec	19	49335171	49343335
ENSG0000023795	-1.242952994	0.289	0.44	AL357079.1	lncRNA	novel transc	1	43937918	43940781
ENSG0000027220	-0.301587254	0.289	0.44	NA	NA	NA	NA	NA	NA
ENSG0000022563	0.322060165	0.289	0.44	DDAH2	protein_coding	dimethylarg	CHR_HSCHR	31803320	31806899
ENSG0000018838	1.357648105	0.289	0.44	GPAT2P2	unprocessed_p	glycerol-3-p	2	97081098	97083249
ENSG0000017958	0.172575364	0.29	0.44	ZFPM1	protein_coding	zinc finger p	16	88453280	88537031
ENSG0000014714	-0.037232647	0.29	0.44	NONO	protein_coding	non-POU d	X	71283192	71301168
ENSG0000005350	-0.247183356	0.29	0.44	USE1	protein_coding	unconventio	19	17215346	17219829
ENSG0000021503	0.850562488	0.29	0.44	GNL3LP1	processed_pse	G protein nu	5	60891935	60893577
ENSG0000012091	0.188602355	0.29	0.44	EPHX2	protein_coding	epoxide hyc	8	27490781	27545564
ENSG0000013840	-0.639625375	0.29	0.44	MDH1B	protein_coding	malate dehy	2	206737763	206765328
ENSG0000025991	-0.399083066	0.29	0.44	HNRNPLP2	unprocessed_p	heterogene	15	34489002	34490571
ENSG0000010699	0.131252887	0.29	0.44	CDC37L1	protein_coding	cell division	9	4679559	4708399
ENSG0000022624	-0.81902663	0.29	0.44	ZNF32-AS1	lncRNA	ZNF32 antis	10	43643872	43645047
ENSG0000023787	-1.179870978	0.29	0.44	LINC01473	lncRNA	long interge	2	186032884	186422432
ENSG0000018762	0.215288889	0.291	0.44	ZKSCAN4	protein_coding	zinc finger v	6	28241697	28252269
ENSG0000027443	2.003412096	0.291	0.44	U2	snRNA	U2 spliceoso	17	43245674	43245864
ENSG0000022806	1.690734707	0.291	0.44	PABPC4-AS	lncRNA	PABPC4 ant	1	39565052	39573860
ENSG0000023245	-0.662642988	0.291	0.44	AL133517.1	transcribed_pr	ribosomal p	1	113698884	113699631
ENSG0000016579	0.106522536	0.291	0.44	METTL17	protein_coding	methyltrans	14	20989973	20997035
ENSG0000017610	-0.207799319	0.291	0.44	CHMP6	protein_coding	charged mu	17	80991598	81009517
ENSG0000025872	-0.618953727	0.291	0.44	PRC1-AS1	lncRNA	PRC1 antise	15	90966340	90988625
ENSG0000024915	0.161206633	0.292	0.44	PCDHA11	protein_coding	protocadhe	5	140868183	141012347
ENSG0000020435	-0.216947846	0.292	0.44	NELFE	protein_coding	negative elc	6	31952087	31959038
ENSG0000027934	0.537038823	0.292	0.44	Z98885.3	lncRNA	novel transc	22	49805452	49807208
ENSG0000022641	0.198909795	0.292	0.44	CLIC1	protein_coding	chloride intr	CHR_HSCHR	31806863	31816046
ENSG0000016192	0.358628822	0.292	0.44	MED11	protein_coding	mediator co	17	4731428	4733608
ENSG0000016446	0.547389291	0.292	0.44	DCBLD1	protein_coding	discoidin, CU	6	117453817	117569858

ENSG0000022616	0.593322199	0.292	0.44	AP4B1-AS1	lncRNA	AP4B1 antis	1	113856635	113901237
ENSG0000010572	0.089896176	0.292	0.44	ATP13A1	protein_coding	ATPase 13A	19	19645198	19663676
ENSG0000014355	0.416272067	0.292	0.44	SLC27A3	protein_coding	solute carrie	1	153774354	153780157
ENSG0000017096	-0.422132796	0.292	0.44	HAS2	protein_coding	hyaluronan	8	121612116	121641440
ENSG0000018816	-0.651165722	0.292	0.44	TMPPE	protein_coding	transmembr	3	33090421	33097146
ENSG0000011142	-0.657402934	0.293	0.44	VDR	protein_coding	vitamin D re	12	47841537	47943048
ENSG0000013707	0.09249043	0.293	0.44	RNF38	protein_coding	ring finger p	9	36336396	36487548
ENSG0000006033	0.051098845	0.293	0.44	CCAR1	protein_coding	cell division	10	68721012	68792377
ENSG0000016779	0.609112172	0.293	0.44	NUDT8	protein_coding	nudix hydro	11	67627938	67629937
ENSG0000020492	-0.808495036	0.293	0.45	AC007389.1	lncRNA	novel transc	2	65436711	66200373
ENSG0000023100	0.268896742	0.293	0.45	TRIM26	protein_coding	tripartite mo	CHR_HSCHR	30229518	30258487
ENSG0000016959	-0.102383166	0.293	0.45	INO80E	protein_coding	INO80 comp	16	29995715	30005793
ENSG0000026854	-1.343337107	0.294	0.45	AC012313.4	TEC	novel transc	19	58418560	58419310
ENSG0000014102	1.202642736	0.294	0.45	CDRT15P1	unprocessed_p	CMT1A dup	17	14024514	14025488
ENSG0000022876	-0.460412282	0.294	0.45	BAG6	protein_coding	BAG cochap	CHR_HSCHR	31678475	31692158
ENSG0000028023	-1.085684051	0.294	0.45	AL031719.2	TEC	tec	16	1553655	1554130
ENSG0000025759	1.019783786	0.294	0.45	SCAT2	lncRNA	S-phase car	12	54262615	54279063
ENSG0000002700	-0.252695471	0.294	0.45	MIPEP	protein_coding	mitochondr	13	23730189	23889400
ENSG0000011584	-0.143936496	0.294	0.45	SLC25A12	protein_coding	solute carrie	2	171783405	171999859
ENSG0000018598	0.173284794	0.294	0.45	RASA3	protein_coding	RAS p21 pro	13	113977783	114132623
ENSG0000025928	-1.59457553	0.294	0.45	AC010809.1	lncRNA	novel transc	15	33858602	33864825
ENSG0000026161	0.303370937	0.294	0.45	AC010547.4	protein_coding	novel transc	16	71447600	71489311
ENSG0000010059	0.067211815	0.294	0.45	AHSA1	protein_coding	activator of	14	77457870	77469472
ENSG0000026151	0.741416666	0.294	0.45	AC092368.3	lncRNA	novel transc	16	46622861	46624451
ENSG0000016633	0.091036467	0.295	0.45	ILK	protein_coding	integrin link	11	6603708	6610874
ENSG0000011329	-0.597145526	0.295	0.45	THBS4	protein_coding	thrombospo	5	79991311	80083287
ENSG0000024192	-0.634829917	0.295	0.45	RPL14P3	processed_pse	ribosomal p	4	140366240	140366861
ENSG0000022470	-0.274633611	0.295	0.45	HCG18	lncRNA	HLA comple	CHR_HSCHR	30281062	30317397
ENSG0000014316	-0.09377137	0.296	0.45	DCAF6	protein_coding	DDB1 and C	1	167935783	168075843
ENSG0000025485	0.541739474	0.296	0.45	AP003390.1	lncRNA	novel transc	11	119729583	119739623
ENSG0000027318	-0.986757407	0.296	0.45	AC093726.2	lncRNA	novel transc	7	154956429	154957107
ENSG0000017176	0.15555643	0.296	0.45	SPATA5L1	protein_coding	spermatoge	15	45402336	45421415
ENSG0000027374	0.074479166	0.296	0.45	CYFIP1	protein_coding	cytoplasmic	15	22867052	22981063
ENSG0000016484	-0.523133892	0.296	0.45	GPR146	protein_coding	G protein-c	7	1044546	1059261
ENSG0000026457	0.423663408	0.297	0.45	AC010761.1	lncRNA	novel transc	17	28721487	28722877
ENSG0000011679	0.12946901	0.297	0.45	PHTF1	protein_coding	putative hor	1	113696831	113759489
ENSG0000010547	0.634367892	0.297	0.45	CLEC11A	protein_coding	C-type lecti	19	50723364	50725718
ENSG0000013803	0.208335466	0.297	0.45	DYNC2LI1	protein_coding	dynein cyto	2	43774039	43810010
ENSG0000008329	-0.175952564	0.297	0.45	ULK2	protein_coding	unc-51 like	17	19770829	19867936
ENSG0000026083	-0.380409401	0.297	0.45	NA	NA	NA	NA	NA	NA
ENSG0000022412	1.555334941	0.297	0.45	LINC01248	lncRNA	long interge	2	5602505	5691488
ENSG0000009997	-1.087650635	0.298	0.45	DDTL	protein_coding	D-dopachro	22	23966888	23972556
ENSG0000021962	0.239600696	0.298	0.45	FAM228B	protein_coding	family with s	2	24076526	24169640
ENSG0000025543	1.096369505	0.298	0.45	AP001922.6	lncRNA	novel transc	11	75596144	75597270
ENSG0000016511	0.186177973	0.298	0.45	KIF27	protein_coding	kinesin fami	9	83834099	83921465
ENSG0000012574	0.212039762	0.298	0.45	RTN2	protein_coding	reticulon 2 [	19	45485294	45497055
ENSG0000026077	1.332631781	0.298	0.45	AC055855.1	lncRNA	novel transc	15	66314914	66331703
ENSG0000027806	-0.175723502	0.298	0.45	ZNF251	protein_coding	zinc finger p	CHR_HSCHR	144724139	144759649
ENSG0000016606	-0.089994206	0.298	0.45	SPRED1	protein_coding	sprouty rela	15	38252836	38357249
ENSG0000014615	0.309616902	0.299	0.45	HMGCLL1	protein_coding	3-hydroxym	6	55434373	55579197
ENSG0000027722	0.20919894	0.299	0.45	LRRC37A2	protein_coding	leucine rich	CHR_HSCHR	46339497	46384852
ENSG0000019788	1.257083234	0.299	0.45	MEIG1	protein_coding	meiosis/spe	10	14959388	14988050
ENSG0000027702	-0.770711739	0.299	0.45	MBOAT7	protein_coding	membrane	CHR_HSCHR	54173854	54190480
ENSG0000027859	1.978603135	0.299	0.45	U2	snRNA	U2 spliceos	17	43300041	43300231
ENSG0000010855	0.510820493	0.299	0.45	RASD1	protein_coding	ras related c	17	17494437	17496395
ENSG0000023130	0.475407738	0.299	0.45	AC112484.1	lncRNA	novel transc	3	128860620	128871540
ENSG0000023737	-0.847088071	0.299	0.45	AC010907.2	lncRNA	novel transc	2	3603397	3604242
ENSG0000017008	0.167246072	0.3	0.45	SIMC1	protein_coding	SUMO inter	5	176238367	176345991

ENSG0000014581	-0.094596308	0.3	0.45	ARHGAP26	protein_coding	Rho GTPase	5	142770377	143229011
ENSG0000015732	0.192471212	0.3	0.45	DHRS4	protein_coding	dehydroger	14	23953734	23969279
ENSG0000022909	1.555871974	0.3	0.45	IGHV3-47	IG_V_pseudog	immunoglol	14	106518582	106519027
ENSG0000028021	-1.944008986	0.3	0.45	AL022326.2	TEC	TEC	22	39379610	39380015
ENSG0000010489	0.264072636	0.3	0.45	AMH	protein_coding	anti-Mulleri	19	2249309	2252073
ENSG0000020678	2.256982421	0.3	0.45	SNORA15B	snoRNA	small nucleo	7	65760052	65760186
ENSG0000020695	1.676690524	0.3	0.45	SNORA50A	snoRNA	small nucleo	16	58559796	58559929
ENSG0000025998	-1.005322659	0.3	0.45	AC017100.1	lncRNA	novel transc	18	31685655	31686823
ENSG0000016614	0.078057339	0.3	0.45	FBN1	protein_coding	fibrillin 1 [So	15	48408313	48645721
ENSG0000008922	-0.05758006	0.3	0.45	PEBP1	protein_coding	phosphatidy	12	118136124	118145584
ENSG0000001021	-0.288510102	0.301	0.45	DYRK4	protein_coding	dual specific	12	4562204	4615302
ENSG0000026529	-0.642405735	0.301	0.45	AC132812.1	unprocessed_p	small nuclea	17	64750420	64751311
ENSG0000021374	0.22210977	0.301	0.45	SERBP1P1	processed_pse	SERPINE1 m	X	68783472	68785066
ENSG0000016424	-0.247021605	0.301	0.45	C5orf63	protein_coding	chromosom	5	127042558	127073492
ENSG0000022197	0.460049745	0.301	0.45	GTF2H4	protein_coding	general tran	CHR_HSCHR	30897757	30903679
ENSG0000018841	0.097151522	0.301	0.45	CHM	protein_coding	CHM Rab es	X	85861180	86047561
ENSG0000027720	1.280971522	0.301	0.45	NA	NA	NA	NA	NA	NA
ENSG0000016306	0.060546198	0.301	0.45	SGCB	protein_coding	sarcoglycan	4	52020706	52038299
ENSG0000023129	2.077115606	0.302	0.45	AL050341.1	processed_pse	pseudogene	1	40262672	40262984
ENSG0000024309	1.898175846	0.302	0.45	AC079203.1	processed_pse	ribosomal p	15	34085911	34086314
ENSG0000019819	0.102586598	0.302	0.46	SZT2	protein_coding	SZT2 subun	1	43389882	43454247
ENSG0000027179	-0.957681328	0.302	0.46	AL589666.1	protein_coding	novel protei	6	85557978	85615234
ENSG0000013536	0.873977335	0.302	0.46	LMO2	protein_coding	LIM domain	11	33858576	33892076
ENSG0000022588	0.747307598	0.302	0.46	LINC00115	lncRNA	long interge	1	826206	827522
ENSG0000024116	-0.446171654	0.302	0.46	LINC00877	lncRNA	long interge	3	71943592	72279503
ENSG0000025510	1.153996353	0.302	0.46	AP003119.2	lncRNA	novel transc	11	76782581	76783062
ENSG0000027837	0.870077665	0.302	0.46	AP004609.3	lncRNA	novel transc	11	118791254	118793137
ENSG0000018455	-0.733215601	0.302	0.46	SOCS3	protein_coding	suppressor o	17	78356778	78360077
ENSG0000027212	0.673870582	0.302	0.46	AC006058.3	lncRNA	novel transc	3	43998081	43999149
ENSG0000027585	-0.465891437	0.302	0.46	AC084824.4	lncRNA	novel transc	12	32736930	32737660
ENSG0000011173	0.074909634	0.303	0.46	C2CD5	protein_coding	C2 calcium	12	22448583	22544546
ENSG0000017146	-0.070130466	0.303	0.46	ZNF318	protein_coding	zinc finger p	6	43307134	43369647
ENSG0000027684	0.988047372	0.303	0.46	PMS2P10	transcribed_un	PMS1 homo	7	75288120	75299795
ENSG0000011959	0.151463286	0.303	0.46	DCAF4	protein_coding	DDB1 and C	14	72926377	72959703
ENSG0000020361	2.453160202	0.303	0.46	GP1BB	protein_coding	glycoprotein	22	19723539	19724771
ENSG0000027178	0.889571702	0.303	0.46	AC026740.1	lncRNA	novel transc	5	675826	676616
ENSG0000009164	-0.12786029	0.303	0.46	SPAG7	protein_coding	sperm assoc	17	4959226	4967817
ENSG0000018559	-0.221169223	0.303	0.46	WASH3P	transcribed_un	WASP family	15	101961603	101976543
ENSG0000019815	-0.736838577	0.303	0.46	HMGN5	protein_coding	high mobilit	X	81113699	81201913
ENSG0000027691	-0.17591904	0.303	0.46	CEP20	protein_coding	centrosoma	CHR_HSCHR	15950862	15973769
ENSG0000022759	1.275809814	0.304	0.46	Z94721.1	lncRNA	novel transc	6	166969626	166999065
ENSG0000026779	-1.200322413	0.304	0.46	AC009977.1	transcribed_pr	ADAM meta	Y	19598188	19598757
ENSG0000010485	-0.136803129	0.304	0.46	CLASRP	protein_coding	CLK4 associ	19	45039045	45070956
ENSG0000026722	-1.278202229	0.304	0.46	C17orf113	protein_coding	chromosom	17	42038232	42050601
ENSG0000023970	1.603183826	0.304	0.46	AL354710.2	lncRNA	novel transc	9	125241663	125257071
ENSG0000025342	-1.373744012	0.304	0.46	HSPA8P13	unprocessed_p	heat shock p	8	46549089	46550802
ENSG0000023292	-1.344636519	0.304	0.46	AC000078.1	processed_pse	ribosomal p	22	19887289	19887970
ENSG0000023326	1.016243272	0.304	0.46	AC006042.2	processed_pse	peptidylprol	7	7980312	7982228
ENSG0000010558	-0.08069624	0.305	0.46	WDR83OS	protein_coding	WD repeat c	19	12668073	12669415
ENSG0000022631	0.669851522	0.305	0.46	RPS3AP38	processed_pse	RPS3A pseu	10	67960702	67961464
ENSG0000023047	0.367661079	0.305	0.46	ABHD16A	protein_coding	abhydrolase	CHR_HSCHR	31677699	31694178
ENSG0000011431	0.113122401	0.305	0.46	USP4	protein_coding	ubiquitin sp	3	49277144	49340712
ENSG0000016568	0.14410119	0.305	0.46	SNAPC4	protein_coding	small nuclea	9	136375577	136400168
ENSG0000015124	0.090237997	0.306	0.46	DIP2C	protein_coding	disco intera	10	274190	689668
ENSG0000023570	1.658248149	0.306	0.46	CYCSP52	processed_pse	CYCS pseuc	1	157128362	157128671
ENSG0000024597	-0.214498288	0.306	0.46	AP003352.1	lncRNA	novel transc	8	98041726	98044121
ENSG0000027714	-0.088679157	0.306	0.46	MARF1	protein_coding	meiosis regu	CHR_HSCHR	15679520	15728320
ENSG0000023106	0.529071834	0.306	0.46	AC234582.1	lncRNA	novel transc	1	155195004	155205495

ENSG0000020485	-0.116166601	0.306	0.46	FAM216A	protein_coding	family with s		12	110468415	110490385
ENSG0000026480	-1.043376147	0.306	0.46	ERVFRD-3	transcribed_pr	endogenous		9	21929457	21931073
ENSG0000027308	0.284853444	0.306	0.46	NA	NA	NA	NA	NA	NA	
ENSG0000023782	-0.256059199	0.306	0.46	PPP1R11	protein_coding	protein pho	CHR_HSCHR		30057093	30060717
ENSG0000004053	-0.206394946	0.306	0.46	CTNS	protein_coding	cystinosin, ly		17	3636459	3663103
ENSG0000022793	0.892962974	0.307	0.46	AL732414.1	processed_pse	ring finger p		1	231021611	231022183
ENSG0000023288	-0.460597209	0.307	0.46	PHKA1P1	processed_pse	phosphoryla		1	90892992	90893612
ENSG0000011230	0.050116296	0.307	0.46	C6orf62	protein_coding	chromosom		6	24704861	24719998
ENSG0000023221	0.961099954	0.307	0.46	IGHV3-43	IG_V_gene	immunoglol		14	106470264	106470800
ENSG0000014455	0.139777842	0.308	0.46	FANCD2	protein_coding	FA complen		3	10026414	10101932
ENSG0000022558	0.462657895	0.308	0.46	MDC1	protein_coding	mediator of	CHR_HSCHR		30690066	30708147
ENSG0000028012	-0.444391994	0.308	0.46	AL132780.5	TEC	TEC		14	23005499	23007123
ENSG0000024125	1.631142681	0.308	0.46	AL136126.1	processed_pse	ribosomal p		11	33237008	33237409
ENSG0000016798	-0.148360565	0.308	0.46	VPS37C	protein_coding	VPS37C sub		11	61130257	61161615
ENSG0000026875	-1.193975954	0.308	0.46	AC010522.1	protein_coding	novel protei		19	57819719	57858941
ENSG0000026149	-0.57050284	0.308	0.46	AC233699.1	unprocessed_p	aminopeptic		17	36274191	36320547
ENSG0000021307	-0.257626568	0.308	0.46	AL353625.1	transcribed_pr	calcium binc		6	160093082	160096212
ENSG0000008003	-0.369375733	0.309	0.46	PTPRH	protein_coding	protein tyro		19	55181247	55209506
ENSG0000018138	-0.302336714	0.309	0.46	DDX60L	protein_coding	DExD/H-bo		4	168356735	168537786
ENSG0000023784	-0.748745216	0.309	0.46	SMU1P1	processed_pse	SMU1 pseuc		1	157059232	157060762
ENSG0000019846	0.585626099	0.309	0.46	FLVCR1-DT	lncRNA	FLVCR1 dive		1	212852105	212858126
ENSG0000022579	0.247989923	0.309	0.46	TRAM2-AS	lncRNA	TRAM2 anti		6	52576787	52643058
ENSG0000022056	0.547945763	0.309	0.46	PKMP3	transcribed_pr	pyruvate kir		6	85659892	85660606
ENSG0000027467	-0.328226746	0.309	0.46	SPATC1L	protein_coding	spermatoge	CHR_HSCHR		46161526	46186787
ENSG0000017308	1.072236774	0.31	0.46	NA	NA	NA	NA	NA	NA	
ENSG0000017993	-0.85402772	0.31	0.46	LINC00652	lncRNA	long interge		20	18786065	18794579
ENSG0000021455	0.871271432	0.31	0.46	COPS8P2	processed_pse	COP9 signa		3	68145004	68145630
ENSG0000018818	0.153676529	0.31	0.46	LAMTOR4	protein_coding	late endoso		7	100148907	100155944
ENSG0000026106	-0.36862238	0.31	0.46	AC124312.3	lncRNA	novel transc		15	25087661	25088896
ENSG0000013056	-0.111756872	0.31	0.46	UBAC1	protein_coding	UBA domair		9	135932969	135961373
ENSG0000021428	0.837853104	0.31	0.46	AC046134.1	processed_pse	DTW domai		3	139582928	139583593
ENSG0000011486	-0.036655896	0.31	0.46	EIF4G1	protein_coding	eukaryotic t		3	184314495	184335358
ENSG0000018922	-0.150011552	0.31	0.46	PAX8-AS1	lncRNA	PAX8 antise		2	113211421	113276581
ENSG0000027505	1.347775417	0.31	0.46	AC011468.5	lncRNA	novel transc		19	52049007	52049754
ENSG0000011402	-0.143068013	0.31	0.46	NIT2	protein_coding	nitrilase fam		3	100334739	100361635
ENSG0000023812	0.502315001	0.311	0.47	MID1IP1-A	lncRNA	MID1IP1 an	X		38801568	38803883
ENSG0000027712	0.664139466	0.311	0.47	AC211476.5	unprocessed_p	PMS2 postn		7	73065529	73077353
ENSG0000011928	0.080853594	0.311	0.47	C1orf198	protein_coding	chromosom		1	230837119	230869589
ENSG0000017622	0.415910428	0.311	0.47	ZNF404	protein_coding	zinc finger p		19	43872365	43901385
ENSG0000013194	0.226328629	0.311	0.47	RHPN2	protein_coding	rhophilin Rh		19	32978592	33064888
ENSG0000016710	0.099681086	0.311	0.47	FAM102A	protein_coding	family with s		9	127940582	127980989
ENSG0000027226	-1.171274601	0.312	0.47	AC034198.2	lncRNA	novel transc		3	12832219	12832728
ENSG0000018630	-0.657340937	0.312	0.47	MST1P2	unprocessed_p	macrophage		1	16645622	16650289
ENSG0000017604	0.099898372	0.312	0.47	JAKMIP2	protein_coding	janus kinase		5	147585438	147782775
ENSG0000025606	-0.562139191	0.312	0.47	DNAAF4	protein_coding	dynein axor		15	55410525	55508234
ENSG0000017889	0.159891868	0.312	0.47	EXOSC4	protein_coding	exosome cc		8	144078648	144080648
ENSG0000022877	0.524364931	0.312	0.47	WEE2-AS1	lncRNA	WEE2 antise		7	141704003	141738346
ENSG0000017273	-1.091241243	0.313	0.47	TMEM217	protein_coding	transmembr		6	37212178	37258155
ENSG0000014992	0.217734064	0.313	0.47	DOC2A	protein_coding	double C2 c		16	30005514	30023270
ENSG0000024581	0.752390033	0.313	0.47	LINC02202	lncRNA	long interge		5	159100483	159117478
ENSG0000018427	1.77248658	0.313	0.47	H2AC21	protein_coding	H2A cluster		1	149887469	149887965
ENSG0000021828	1.937370406	0.313	0.47	H2AC9P	unprocessed_p	H2A cluster		6	26233122	26233255
ENSG0000021693	-0.400000772	0.313	0.47	CCDC7	protein_coding	coiled-coil c		10	32446140	32882874
ENSG0000010030	0.403116366	0.313	0.47	CBX7	protein_coding	chromobox		22	39120167	39152680
ENSG0000022737	-1.685371547	0.313	0.47	AC254562.1	processed_pse	chromosom		22	42132543	42132998
ENSG0000013584	-0.141146977	0.314	0.47	PIGC	protein_coding	phosphatidy		1	172370189	172444086
ENSG0000027010	-1.668247853	0.314	0.47	NA	NA	NA	NA	NA	NA	
ENSG0000022268	1.846440627	0.314	0.47	RN7SKP119	misc_RNA	RN7SK pseu		2	47359505	47359777

ENSG0000022552	1.521778514	0.314	0.47	AL450384.1	lncRNA	novel transc	10	18531849	18533336
ENSG0000007273	0.07666817	0.314	0.47	NFATC3	protein_coding	nuclear fact	16	68084751	68229259
ENSG0000013545	0.122592217	0.314	0.47	B4GALNT1	protein_coding	beta-1,4-N	12	57623409	57633239
ENSG0000027596	1.205006207	0.315	0.47	NA	NA	NA	NA	NA	NA
ENSG0000017438	0.683172054	0.315	0.47	PMS2P6	unprocessed_p	PMS1 homo	7	73093657	73105389
ENSG0000023290	0.594779709	0.315	0.47	AL157823.2	lncRNA	novel transc	6	35733867	35736947
ENSG0000022913	0.172200611	0.315	0.47	EIF4A1P10	processed_pse	eukaryotic t	X	92113246	92114461
ENSG0000027671	-1.360280771	0.315	0.47	AC005840.4	lncRNA	novel transc	12	6466537	6467135
ENSG0000025902	0.627045238	0.315	0.47	DNAJC8P1	processed_pse	DnaJ heat sl	14	35430260	35430631
ENSG0000013433	0.129039119	0.315	0.47	IAH1	protein_coding	isoamyl ace	2	9473658	9496543
ENSG0000015464	0.120401159	0.315	0.47	BTG3	protein_coding	BTG anti-pr	21	17593653	17612945
ENSG0000013563	-0.141346289	0.315	0.47	SMYD5	protein_coding	SMYD family	2	73214222	73227221
ENSG0000020453	0.407072321	0.315	0.47	CCHCR1	protein_coding	coiled-coil a	6	31142439	31158238
ENSG0000022074	-0.993249222	0.315	0.47	RPL21P28	processed_pse	ribosomal p	1	212051524	212052006
ENSG0000023188	0.223184528	0.315	0.47	TRAF3IP2-A	lncRNA	TRAF3IP2 ar	6	111483511	111598302
ENSG0000007704	0.076757653	0.316	0.47	DGKD	protein_coding	diacylglycer	2	233354494	233472104
ENSG0000025487	1.506413714	0.316	0.47	AP001267.1	lncRNA	novel transc	11	118397095	118401895
ENSG0000022959	0.878464673	0.316	0.47	PRDX3P1	processed_pse	peroxiredox	22	38722743	38723505
ENSG0000023379	0.497978193	0.316	0.47	ZNRD1	protein_coding	zinc ribbon	CHR_HSCHR	30049286	30055293
ENSG0000025717	1.575974757	0.316	0.47	HMGN2P6	processed_pse	high mobilit	14	25267064	25267336
ENSG0000010287	0.210652759	0.316	0.47	HSF4	protein_coding	heat shock t	16	67164681	67169945
ENSG0000026396	1.095113827	0.316	0.47	RN7SL381P	misc_RNA	RNA, 7SL, cy	16	85659378	85659675
ENSG0000026975	0.30594357	0.316	0.47	NA	NA	NA	NA	NA	NA
ENSG0000024392	-1.061854389	0.316	0.47	TIPARP-AS	lncRNA	TIPARP antis	3	156671862	156674446
ENSG0000010898	0.242019843	0.317	0.47	MAP2K6	protein_coding	mitogen-ac	17	69414697	69553865
ENSG0000026992	0.354119974	0.317	0.47	MIRLET7A1	lncRNA	miRlet-7a-1	9	94166258	94200902
ENSG0000023039	1.788939969	0.317	0.47	SPTLC1P1	transcribed_pr	serine palm	10	31360955	31361215
ENSG0000027009	1.295036945	0.317	0.47	AC068790.0	lncRNA	novel transc	12	123971457	123971714
ENSG0000010577	0.078753005	0.317	0.47	AVL9	protein_coding	AVL9 cell m	7	32495426	32588726
ENSG0000007752	-0.399595982	0.318	0.47	ACTN2	protein_coding	actinin alpha	1	236664141	236764631
ENSG0000015128	0.125572907	0.318	0.47	TEX30	protein_coding	testis expres	13	102765888	102773811
ENSG0000022337	-0.891604322	0.318	0.47	AC005104.1	lncRNA	novel transc	2	241351340	241353104
ENSG0000023207	-0.907105587	0.318	0.47	TMEM253	protein_coding	transmembr	14	21098811	21103724
ENSG0000026257	0.440389659	0.318	0.47	PCDHGA4	protein_coding	protocadhe	5	141355021	141512975
ENSG0000012287	-0.109996583	0.318	0.47	BICC1	protein_coding	BicC family	10	58512872	58831435
ENSG0000013746	-0.776884849	0.318	0.47	MGARP	protein_coding	mitochondr	4	139266165	139280225
ENSG0000017885	-0.356646075	0.318	0.47	EFCAB13	protein_coding	EF-hand cal	17	47323290	47441312
ENSG0000026241	-1.154867891	0.318	0.47	AL160291.1	lncRNA	novel transc	10	27243130	27250804
ENSG0000025098	0.452398158	0.319	0.47	SNHG21	lncRNA	small nucleo	15	82750564	82757206
ENSG0000010325	0.222455914	0.319	0.47	ANTKMT	protein_coding	adenine nuc	16	720581	722590
ENSG0000012888	0.07155762	0.319	0.48	TTBK2	protein_coding	tau tubulin k	15	42738730	42920809
ENSG0000016827	0.392030662	0.319	0.48	SMIM4	protein_coding	small integr	3	52534013	52579237
ENSG0000016490	0.085008625	0.32	0.48	PHAX	protein_coding	phosphoryla	5	126600925	126627252
ENSG0000017586	-0.741904681	0.32	0.48	CALCB	protein_coding	calcitonin re	11	14904997	15082342
ENSG0000019841	1.023188095	0.32	0.48	MT1F	protein_coding	metallothion	16	56657731	56660698
ENSG0000026631	1.850701894	0.32	0.48	AC026254.2	lncRNA	novel transc	17	27333241	27352828
ENSG0000013670	0.06034073	0.32	0.48	WDR33	protein_coding	WD repeat d	2	127701027	127811187
ENSG0000016214	-0.110085384	0.32	0.48	CYB561A3	protein_coding	cytochrome	11	61348754	61362283
ENSG0000022526	-0.555405459	0.321	0.48	ZNRF2P2	transcribed_pr	zinc and rin	7	29598795	29685255
ENSG0000010505	0.069950533	0.321	0.48	FAM32A	protein_coding	family with s	19	16185380	16192046
ENSG0000017238	-0.415636725	0.321	0.48	PRSS27	protein_coding	serine prote	16	2712419	2720551
ENSG0000026187	0.661592107	0.321	0.48	AC087500.1	lncRNA	novel transc	17	5192027	5248182
ENSG0000026545	-1.61062733	0.321	0.48	MIR3682	miRNA	microRNA 3	2	53849122	53849205
ENSG0000027331	0.508860534	0.321	0.48	DGCR11	lncRNA	DiGeorge sy	22	19046162	19048375
ENSG0000013831	0.392164807	0.322	0.48	ZNF365	protein_coding	zinc finger p	10	62374192	62480288
ENSG0000018503	-0.168214432	0.322	0.48	SEMA4B	protein_coding	semaphorin	15	90160604	90229679
ENSG0000018662	0.121896004	0.322	0.48	KATNA1	protein_coding	katanin cata	6	149594873	149648972
ENSG0000019821	-0.089414675	0.322	0.48	QRICH1	protein_coding	glutamine ri	3	49029707	49094363

ENSG0000023641	-1.519082579	0.321	0.48	NDUFAF4P	processed_pse	NADH:ubiqu	3	37789921	37790445
ENSG0000025970	1.403394412	0.322	0.48	AC105339.4	processed_pse	serine/argin	15	82691723	82692358
ENSG0000026453	1.345664449	0.322	0.48	MIR548AR	miRNA	microRNA 5	13	114244505	114244561
ENSG0000012414	0.176313207	0.322	0.48	SDC4	protein_coding	syndecan 4	20	45325288	45348424
ENSG0000023219	0.877376194	0.322	0.48	MTRNR2L4	protein_coding	MT-RNR2 li	16	3370979	3372668
ENSG0000026023	-1.292067342	0.322	0.48	PMF1-BGLA	protein_coding	PMF1-BGLA	1	156212982	156243332
ENSG0000023746	0.493033842	0.322	0.48	TRIM27	protein_coding	tripartite mo	CHR_HSCHR	28914742	28923955
ENSG0000018512	0.080582291	0.322	0.48	HSF1	protein_coding	heat shock t	8	144291591	144314720
ENSG0000024162	-0.415831946	0.322	0.48	UBQLN4P1	processed_pse	ubiquilin 4 p	3	148985868	148987668
ENSG0000017830	0.651821182	0.322	0.48	AQP11	protein_coding	aquaporin 1	11	77589391	77610356
ENSG0000015779	-0.079621817	0.322	0.48	WDR19	protein_coding	WD repeat d	4	39182504	39285810
ENSG0000020112	-2.183034392	0.322	0.48	SNORA58B	snoRNA	small nucleo	1	154259727	154259862
ENSG0000020688	-2.183034392	0.322	0.48	RNU6-48P	snRNA	RNA, U6 sm	1	27325219	27325325
ENSG0000022978	-1.794585157	0.322	0.48	UBE2Q1-AS	lncRNA	UBE2Q1 ant	1	154553609	154555017
ENSG0000025170	-2.183034392	0.322	0.48	AC142283.1	snoRNA		2	16199203	16199295
ENSG0000020515	-0.180787318	0.323	0.48	PSENEN	protein_coding	presenilin er	19	35745600	35747519
ENSG0000014670	0.377196917	0.323	0.48	POMZP3	protein_coding	POM121 an	7	76609986	76627261
ENSG0000023199	-1.220569595	0.323	0.48	AL590399.4	processed_pse	pseudogene	9	62266319	62266919
ENSG0000022700	-0.692681281	0.323	0.48	NBPF2P	unprocessed_p	NBPF memb	1	21424625	21427967
ENSG0000001350	-0.107149655	0.323	0.48	POLR3B	protein_coding	RNA polyme	12	106357748	106510198
ENSG0000013531	0.044857225	0.323	0.48	SYNCRIP	protein_coding	synaptotagn	6	85607785	85643792
ENSG0000026174	1.192903477	0.323	0.48	AC116552.1	lncRNA	novel transc	16	88512960	88531053
ENSG0000027812	-0.907648106	0.323	0.48	AC139768.1	lncRNA	novel transc	12	51201684	51202581
ENSG0000003362	0.070289222	0.324	0.48	ATP6V0A1	protein_coding	ATPase H+	17	42458844	42522582
ENSG0000013048	-0.220031598	0.324	0.48	NA	NA	NA	NA	NA	NA
ENSG0000020769	1.917785231	0.324	0.48	MIR659	miRNA	microRNA 6	22	37847678	37847774
ENSG0000027417	1.584143098	0.324	0.48	CNOT3	protein_coding	CCR4-NOT	CHR_HSCHR	54138182	54156191
ENSG0000021308	-0.447253806	0.324	0.48	AL354714.2	processed_pse	spermine sy	1	160894980	160896076
ENSG0000024899	-1.238302135	0.324	0.48	AC145098.1	lncRNA	novel transc	5	177494995	177503647
ENSG0000007945	-0.050883373	0.325	0.48	FDFT1	protein_coding	farnesyl-dip	8	11795573	11839304
ENSG0000014788	-0.224159946	0.325	0.48	CDKN2A	protein_coding	cyclin deper	9	21967753	21995301
ENSG0000018185	-0.659176018	0.325	0.48	SLC2A4	protein_coding	solute carrie	17	7281718	7288257
ENSG0000021410	0.256626212	0.325	0.48	PAXIP1-AS	lncRNA	PAXIP1 antis	7	154928460	154952188
ENSG0000023096	1.126220849	0.325	0.48	MSH5	protein_coding	mutS homo	CHR_HSCHR	31779445	31786880
ENSG0000027271	0.243231737	0.325	0.48	AC019069.1	lncRNA	novel transc	2	74832655	74833987
ENSG0000018567	0.285187902	0.325	0.48	ZBTB3	protein_coding	zinc finger a	11	62748319	62754184
ENSG0000022519	0.205677072	0.325	0.48	PLEKHM1	protein_coding	pleckstrin ho	17	45435900	45490749
ENSG0000010855	0.089445354	0.325	0.48	NUP88	protein_coding	nucleoporin	17	5360963	5419676
ENSG0000021405	-0.63025707	0.325	0.48	FBXO16	protein_coding	F-box prote	8	28348287	28490278
ENSG0000013627	-0.076812003	0.325	0.48	TBRG4	protein_coding	transforming	7	45100100	45112047
ENSG0000023477	-1.579788698	0.325	0.48	C11orf94	protein_coding	chromosom	11	45906513	45907272
ENSG0000026999	0.744633853	0.325	0.48	AC068790.1	lncRNA	novel transc	12	123966077	123966629
ENSG0000024494	0.758860953	0.325	0.48	RUFY1-AS1	lncRNA	RUFY1 antis	5	179595904	179603741
ENSG0000027344	0.508933087	0.325	0.48	AC006480.2	lncRNA	novel transc	7	67333047	67334383
ENSG0000027554	-0.658127913	0.326	0.48	MPV17L	protein_coding	MPV17 mito	CHR_HSCHR	15480860	15498390
ENSG0000024396	-1.102919966	0.326	0.48	AL390195.1	lncRNA	novel transc	1	111438638	111441364
ENSG0000017760	0.050809167	0.326	0.48	RPLP2	protein_coding	ribosomal p	11	809965	812880
ENSG0000013033	-0.068627465	0.326	0.48	TULP4	protein_coding	TUB like pro	6	158232236	158511828
ENSG0000016139	0.247711798	0.326	0.48	PGAP3	protein_coding	post-GPI at	17	39671122	39696797
ENSG0000011896	-0.074798462	0.326	0.48	WDR35	protein_coding	WD repeat d	2	19910260	19990131
ENSG0000023406	-0.691944713	0.326	0.48	AL390879.1	transcribed_un	transmembr	X	136909369	137022143
ENSG0000017113	-0.153479338	0.326	0.48	JAGN1	protein_coding	jagunal hom	3	9890574	9894349
ENSG0000020364	0.435380291	0.326	0.48	AC083799.1	lncRNA	novel transc	3	129847048	129847957
ENSG0000018126	-0.763003878	0.327	0.48	MTHFD2P7	processed_pse	methylenete	3	179464346	179465328
ENSG0000022526	-0.590642715	0.327	0.48	TAF1A-AS1	lncRNA	TAF1A antis	1	222589825	222593843
ENSG0000023152	-0.911591022	0.327	0.48	FAM27C	lncRNA	family with s	9	61854148	62096920
ENSG0000027300	-0.721586903	0.327	0.48	AC021205.3	lncRNA	novel transc	4	122879778	122885805
ENSG0000012315	0.223707898	0.328	0.48	WDR83	protein_coding	WD repeat d	19	12666802	12675832

ENSG0000013800	-0.094320687	0.328	0.48	IFT172	protein_coding	intraflagella		2	27444377	27489805
ENSG0000021325	-0.964019466	0.328	0.48	RBMS2P1	processed_pse	RNA binding		12	94423744	94424969
ENSG0000010452	-0.181374019	0.328	0.48	TSTA3	protein_coding	tissue specif		8	143612618	143618048
ENSG0000016600	-0.196241598	0.328	0.49	SMCO4	protein_coding	single-pass		11	93478472	93543391
ENSG0000013504	-0.833630756	0.328	0.49	ANXA1	protein_coding	annexin A1		9	73151865	73170393
ENSG0000013545	-0.113718038	0.329	0.49	TSPAN31	protein_coding	tetraspanin		12	57738013	57750219
ENSG0000022972	0.212094473	0.329	0.49	NA	NA	NA	NA	NA	NA	
ENSG0000014659	0.089273387	0.329	0.49	CREB5	protein_coding	cAMP respo		7	28299321	28825894
ENSG0000026316	0.397999571	0.329	0.49	SLC27A3	protein_coding	solute carrie	CHR_HSCHR	153791585	153797388	
ENSG0000028019	-1.274372267	0.329	0.49	AC087163.3	TEC	novel transc		17	18026072	18026771
ENSG0000025426	0.862888563	0.329	0.49	PKIA-AS1	lncRNA	PKIA antiser		8	78268637	78558503
ENSG0000026737	0.853948218	0.329	0.49	AC005330.1	lncRNA	novel transc		19	1321225	1322846
ENSG0000026263	0.198472664	0.329	0.49	SKA1	protein_coding	spindle and	CHR_HSCHR	50374995	50394173	
ENSG0000012795	-0.265303142	0.329	0.49	PMS2P3	transcribed_un	PMS1 homo		7	75507747	75528148
ENSG0000026754	-1.72085941	0.329	0.49	AC007229.1	processed_pse	chromosom		19	10841839	10842250
ENSG0000024817	-0.319800444	0.33	0.49	NA	NA	NA	NA	NA	NA	
ENSG0000011747	-0.130612606	0.33	0.49	SLC19A2	protein_coding	solute carrie		1	169463909	169485944
ENSG0000013031	0.087674438	0.33	0.49	DDA1	protein_coding	DET1 and D		19	17309518	17323298
ENSG0000024251	-0.226115761	0.33	0.49	LINC00960	lncRNA	long interge		3	75672300	75742048
ENSG0000022404	1.471711958	0.33	0.49	HMGN1P4	processed_pse	high mobilit		1	182942115	182942404
ENSG0000023661	1.471711958	0.33	0.49	AC127070.1	lncRNA	novel transc		12	132887842	132888583
ENSG0000022664	0.974234216	0.33	0.49	AL365356.1	lncRNA	novel transc		10	5712174	5744067
ENSG0000013858	0.233393516	0.33	0.49	MNS1	protein_coding	meiosis spe		15	56421544	56465137
ENSG0000018277	-0.171154312	0.33	0.49	RPS17	protein_coding	ribosomal p		15	82536750	82540459
ENSG0000018574	0.585218234	0.33	0.49	IFIT1	protein_coding	interferon in		10	89392546	89406487
ENSG0000027740	-1.25062186	0.331	0.49	SEC22B4P	transcribed_un	SEC22 hom		1	146321214	146401647
ENSG0000017312	-0.05149545	0.331	0.49	KDM2A	protein_coding	lysine deme		11	67119263	67258082
ENSG0000020424	-0.357593979	0.331	0.49	LINC02731	lncRNA	long interge		11	134032216	134067936
ENSG0000022507	-0.883637205	0.331	0.49	LINC00337	lncRNA	long interge		1	6234692	6239444
ENSG0000023281	0.497523245	0.331	0.49	RPS2P32	processed_pse	ribosomal p		7	23490473	23491364
ENSG0000023295	0.493320422	0.331	0.49	AL512844.1	processed_pse	matrin 3 (M		1	105891739	105893517
ENSG0000006770	-0.059177002	0.331	0.49	IARS2	protein_coding	isoleucyl-tR		1	220094132	220148041
ENSG0000018626	-0.076576162	0.331	0.49	MRTFB	protein_coding	myocardin r		16	14071319	14266773
ENSG0000023295	-0.117772896	0.331	0.49	SNHG15	lncRNA	small nucle		7	44983023	44986961
ENSG0000010421	-0.078136379	0.332	0.49	ZDHHC2	protein_coding	zinc finger D		8	17156482	17224799
ENSG0000016678	-0.083698959	0.332	0.49	MARF1	protein_coding	meiosis regu		16	15594387	15643154
ENSG0000020556	0.281447552	0.332	0.49	CPT1B	protein_coding	carnitine pa		22	50568861	50578465
ENSG0000027376	2.14892342	0.332	0.49	RNVU1-29	snRNA	RNA, varian		1	146376807	146376970
ENSG0000027378	0.348991252	0.332	0.49	AL137058.2	lncRNA	novel transc		13	52600042	52642542
ENSG0000013187	-0.069062532	0.332	0.49	CHSY1	protein_coding	chondroitin		15	101175727	101252048
ENSG0000012584	-0.197996278	0.332	0.49	AP5S1	protein_coding	adaptor rela		20	3820524	3828838
ENSG0000021343	-0.226323514	0.332	0.49	HSPD1P1	processed_pse	heat shock p		5	21882585	21884310
ENSG0000023316	-0.524741068	0.332	0.49	C6orf136	protein_coding	chromosom	CHR_HSCHR	30691675	30697740	
ENSG0000027611	-0.180786218	0.332	0.49	SDCCAG8	protein_coding	SHH signali	CHR_HSCHR	243256056	243500091	
ENSG0000010582	-0.141911601	0.333	0.49	BET1	protein_coding	Bet1 golgi v		7	93962762	94004382
ENSG0000014093	-0.11429613	0.333	0.49	CMTM3	protein_coding	CKLF like M		16	66603874	66613892
ENSG0000023827	0.812147401	0.333	0.49	ALG1L6P	unprocessed_p	ALG1 like 6,		3	75415070	75422143
ENSG0000025828	-0.093606334	0.333	0.49	CHURC1	protein_coding	churchill do		14	64914361	64944591
ENSG0000026453	-0.244154588	0.333	0.49	SUZ12P1	transcribed_un	SUZ12 pseu		17	30709299	30790908
ENSG0000013646	0.12840911	0.334	0.49	TACO1	protein_coding	translationa		17	63600882	63608365
ENSG0000018505	-0.753588337	0.334	0.49	EFCAB10	protein_coding	EF-hand cal		7	105565120	105600875
ENSG0000018861	-0.188699441	0.334	0.49	ASAH2	protein_coding	N-acylsphin		10	50182778	50279720
ENSG0000026996	1.414697465	0.334	0.49	AC010359.2	lncRNA	novel transc		5	65924629	65925135
ENSG0000013731	0.222285899	0.334	0.49	TRIM26	protein_coding	tripartite mo	CHR_HSCHR	30173958	30202936	
ENSG0000008708	-0.12383687	0.334	0.49	BAX	protein_coding	BCL2 associ		19	48954815	48961798
ENSG0000021338	1.045259151	0.334	0.49	AC105052.2	processed_pse	ribosomal p		7	102755146	102755939
ENSG0000022406	1.262462282	0.334	0.49	AL049795.1	lncRNA	novel transc		1	32204769	32206814
ENSG0000022998	0.409835579	0.335	0.49	TOB1-AS1	lncRNA	TOB1 antise		17	50866679	50910774

ENSG0000013750	-0.07493168	0.335	0.49	PRCP	protein_coding	prolylcarbox	11	82822936	82970584
ENSG0000024827	0.327431118	0.335	0.49	TRIM52-AS	lncRNA	TRIM52 anti	5	181261169	181272307
ENSG0000014745	0.076386097	0.335	0.49	CHMP7	protein_coding	charged mu	8	23243637	23262000
ENSG0000020504	-0.573305111	0.335	0.49	AC118344.1	lncRNA	novel transc	19	40273489	40275479
ENSG0000018591	-0.137709865	0.336	0.49	SETD4	protein_coding	SET domain	21	36034541	36079389
ENSG0000020524	-0.292719606	0.336	0.49	NA	NA	NA	NA	NA	NA
ENSG0000022599	0.255168658	0.336	0.49	LSM2	protein_coding	LSM2 homo	CHR_HSCHR	31873692	31883281
ENSG0000027909	0.930371389	0.336	0.49	LINC01670	lncRNA	long interge	21	5499151	5502542
ENSG0000021219	2.238851827	0.336	0.49	U3	snoRNA	Small nucle	17	58631641	58631836
ENSG0000027090	0.78104496	0.336	0.49	HNRNPA3P	processed_pse	heterogene	11	32591793	32592771
ENSG0000027290	0.863862492	0.336	0.49	AL353708.3	lncRNA	novel transc	1	179881607	179882595
ENSG0000019892	-0.161118233	0.336	0.49	KIAA0753	protein_coding	KIAA0753 [S	17	6578147	6640711
ENSG0000020027	-1.751561718	0.337	0.49	SNORD114	snoRNA	small nucle	14	100967052	100967123
ENSG0000027017	0.844325912	0.337	0.49	AP002840.2	lncRNA	novel transc	11	113368478	113369117
ENSG0000011851	-0.237662877	0.337	0.49	MYB	protein_coding	MYB proto-	6	135181308	135219173
ENSG0000026998	-0.457635521	0.337	0.49	AC018809.2	lncRNA	novel transc	3	9958717	9962539
ENSG0000020091	1.282622748	0.337	0.49	SNORD46	snoRNA	small nucle	1	44776490	44776593
ENSG0000015238	-0.468730812	0.337	0.49	FAM151B	protein_coding	family with s	5	80487969	80542563
ENSG0000017448	-0.08094179	0.337	0.49	DENND4A	protein_coding	DENN doma	15	65658046	65792293
ENSG0000026247	0.153688848	0.337	0.49	GART	protein_coding	phosphoribo	CHR_HSCHR	33508081	33521016
ENSG0000018333	0.234390306	0.337	0.49	BOLA2	protein_coding	bolA family	16	29443056	29454964
ENSG0000010415	-0.225936502	0.338	0.5	SLC30A4	protein_coding	solute carrie	15	45479606	45522755
ENSG0000026152	-0.238594937	0.338	0.5	AC012615.1	lncRNA	novel transc	19	1874871	1876169
ENSG0000022354	-0.841625984	0.338	0.5	MTND5P28	processed_pse	MT-ND5 ps	2	120215181	120217279
ENSG0000013843	-0.053917096	0.339	0.5	OLA1	protein_coding	Obg like AT	2	174072447	174248599
ENSG0000017348	0.184125985	0.339	0.5	ZNF417	protein_coding	zinc finger p	19	57900296	57916610
ENSG0000012736	0.571203264	0.339	0.5	TAS2R4	protein_coding	taste 2 rece	7	141776674	141781691
ENSG0000015795	0.076171547	0.339	0.5	WIPI2	protein_coding	WD repeat d	7	5190196	5233840
ENSG0000015242	0.1744882	0.339	0.5	XRCC4	protein_coding	X-ray repair	5	83077498	83353787
ENSG0000024253	1.777119414	0.339	0.5	AC083798.1	unprocessed_p	pseudogene	3	122416882	122417012
ENSG0000027599	0.294599355	0.339	0.5	SIK1B	protein_coding	salt inducibl	21	6111131	6123778
ENSG0000019744	-0.811162101	0.339	0.5	NA	NA	NA	NA	NA	NA
ENSG0000016269	-0.090514886	0.34	0.5	SLC30A7	protein_coding	solute carrie	1	100896076	100981757
ENSG0000023594	0.558865659	0.34	0.5	H2BW3P	processed_pse	H2B.W histo	X	104062542	104063037
ENSG0000026551	-0.946833367	0.34	0.5	AC015922.2	lncRNA	novel transc	17	15787787	15788205
ENSG0000010288	-0.858570574	0.34	0.5	GDPD3	protein_coding	glycerophos	16	30104810	30113537
ENSG0000017996	0.212502797	0.34	0.5	ZNF771	protein_coding	zinc finger p	16	30407414	30431108
ENSG0000020442	1.173003922	0.34	0.5	ABHD16A	protein_coding	abhydrolase	6	31686955	31703356
ENSG0000027973	-1.544338341	0.34	0.5	AC124312.4	TEC	TEC	15	25091960	25092377
ENSG0000018214	0.05757333	0.34	0.5	IST1	protein_coding	IST1 factor a	16	71845996	71931199
ENSG0000018035	0.107973738	0.34	0.5	MTURN	protein_coding	maturin, neu	7	30134986	30162762
ENSG0000023484	0.26353594	0.34	0.5	NEU1	protein_coding	neuraminida	CHR_HSCHR	31850351	31855624
ENSG0000025838	0.735505652	0.34	0.5	RCCD1-AS1	lncRNA	RCCD1 and	15	90952239	90955225
ENSG0000027760	-1.854199411	0.34	0.5	AC005363.2	lncRNA	novel transc	16	1971655	1971896
ENSG0000010322	-0.235333638	0.341	0.5	LMF1	protein_coding	lipase matu	16	853634	981318
ENSG0000013816	-0.188391579	0.341	0.5	DUSP5	protein_coding	dual specific	10	110497907	110511533
ENSG0000018319	-0.20031397	0.341	0.5	HSP90AB3P	processed_pse	heat shock p	4	87891843	87894015
ENSG0000023690	-1.665010619	0.341	0.5	AC104333.3	processed_pse	ribosomal p	1	212824027	212824505
ENSG0000008381	0.178074515	0.341	0.5	ZNF324	protein_coding	zinc finger p	19	58467045	58475436
ENSG0000018022	0.215073422	0.341	0.5	HERC2P3	transcribed_un	hect domain	15	20379495	20506180
ENSG0000010960	-0.039914978	0.342	0.5	DHX15	protein_coding	DEAH-box h	4	24517441	24584554
ENSG0000014637	-0.469942029	0.342	0.5	ARHGAP18	protein_coding	Rho GTPase	6	129576132	129710177
ENSG0000017259	-0.107602632	0.342	0.5	MRPL52	protein_coding	mitochondr	14	22829879	22835037
ENSG0000026153	-0.596745112	0.342	0.5	NA	NA	NA	NA	NA	NA
ENSG0000016371	0.135200949	0.342	0.5	MTMR14	protein_coding	myotubulari	3	9649433	9702393
ENSG0000024225	-1.028538343	0.342	0.5	BGLAP	protein_coding	bone gamm	1	156242184	156243317
ENSG0000026750	0.498428984	0.342	0.5	ZNF887P	transcribed_un	zinc finger p	19	11648364	11652877
ENSG0000027932	-0.533027364	0.343	0.5	AC020910.6	TEC	novel transc	19	34675717	34677581

ENSG0000016460	-0.15510974	0.343	0.5	BMT2	protein_coding	base methy		7	112819147	112939875
ENSG0000022384	-1.428341435	0.343	0.5	AL354893.1	processed_pse	ribosomal p		9	95123521	95123868
ENSG0000017897	-0.143918103	0.343	0.5	CTC1	protein_coding	CST telomer		17	8224815	8248056
ENSG0000019793	0.23373021	0.343	0.5	ZNF823	protein_coding	zinc finger p		19	11721265	11739009
ENSG0000025108	-1.827038194	0.343	0.5	NA	NA	NA	NA	NA	NA	
ENSG0000027103	-1.711564523	0.343	0.5	AC020907.4	lncRNA	novel transc		19	35014961	35025335
ENSG0000023488	-0.672796934	0.343	0.5	EIF3EP1	processed_pse	eukaryotic t		6	73291962	73293277
ENSG0000018304	0.197902392	0.344	0.5	SLC25A10	protein_coding	solute carrie		17	81712236	81721016
ENSG0000020465	0.458193162	0.344	0.5	ASPDH	protein_coding	aspartate de		19	50511600	50514690
ENSG0000027236	-0.696101909	0.344	0.5	NA	NA	NA	NA	NA	NA	
ENSG0000027275	0.125658653	0.344	0.5	STAG3L5P-	lncRNA	STAG3L5P-I		7	100336104	100367831
ENSG0000003763	-0.081273032	0.344	0.5	FBXO42	protein_coding	F-box prote		1	16246840	16352480
ENSG0000012072	-0.133344798	0.344	0.5	SIL1	protein_coding	SIL1 nucleot		5	138946724	139293557
ENSG0000005648	-0.086359615	0.344	0.5	PHF21B	protein_coding	PHD finger		22	44881162	45010005
ENSG0000023797	-0.558212655	0.345	0.5	RFX5-AS1	lncRNA	RFX5 antiser		1	151346967	151348027
ENSG0000025136	-0.164820268	0.345	0.5	ZNF550	protein_coding	zinc finger p		19	57535257	57559863
ENSG0000013238	-0.079734544	0.345	0.5	UBE2G1	protein_coding	ubiquitin co		17	4269259	4366628
ENSG0000023588	0.958728929	0.345	0.5	LINC00941	lncRNA	long interge		12	30755167	30802602
ENSG0000016806	0.044204095	0.345	0.5	SF1	protein_coding	splicing fact		11	64764606	64778786
ENSG0000022365	0.663595833	0.345	0.5	FLOT1	protein_coding	flotillin 1 [Sc	CHR_HSCHR		30791619	30794413
ENSG0000025645	-0.80930064	0.345	0.5	DND1	protein_coding	DND microR		5	140670794	140673576
ENSG0000016449	0.132633951	0.346	0.5	PDSS2	protein_coding	decaprenyl		6	107152562	107459564
ENSG0000022272	1.445271461	0.346	0.5	RNU2-7P	snRNA	RNA, U2 sm		13	20612161	20612338
ENSG0000023965	-0.415122357	0.346	0.5	PSMD6-AS	lncRNA	PSMD6 anti		3	64004022	64012148
ENSG0000023735	0.528501796	0.346	0.5	CDC42P6	processed_pse	cell division		4	22727375	22727950
ENSG0000014230	0.436047762	0.346	0.5	ADAMTS10	protein_coding	ADAM meta		19	8580240	8610735
ENSG0000021213	1.294307619	0.346	0.5	SNORD67	snoRNA	small nucleo		11	46762389	46762499
ENSG0000000601	-0.427419922	0.347	0.51	CRLF1	protein_coding	cytokine rec		19	18572220	18607741
ENSG0000025224	1.671882235	0.347	0.51	NA	NA	NA	NA	NA	NA	
ENSG0000021418	0.165909405	0.347	0.51	PTMAP5	transcribed_pr	prothymosin		13	81689911	81691072
ENSG0000027786	-1.141634916	0.347	0.51	AC138649.4	processed_pse	dexamethas		15	22714939	22715225
ENSG0000022419	-0.748064406	0.347	0.51	AC022400.1	lncRNA	novel transc		10	73813518	73814737
ENSG0000017124	0.099686813	0.348	0.51	SHCBP1	protein_coding	SHC binding		16	46578591	46621379
ENSG0000027715	-0.780155564	0.348	0.51	AC012314.1	protein_coding	pre-mRNA	CHR_HSCHR		54115536	54131896
ENSG0000016682	-0.893279775	0.348	0.51	MESP1	protein_coding	mesoderm p		15	89748661	89751310
ENSG0000020366	0.079097236	0.348	0.51	CHML	protein_coding	CHM like Ra		1	241628853	241640254
ENSG0000026775	-0.675715669	0.348	0.51	AC006557.4	unprocessed_p	vomeronasa		18	14074912	14075741
ENSG0000027528	1.224957042	0.348	0.51	CFL1P5	processed_pse	cofilin 1 pse	CHR_HSCHR		69313353	69313854
ENSG0000023356	-0.22260847	0.348	0.51	PRR3	protein_coding	proline rich	CHR_HSCHR		30635339	30642176
ENSG0000020156	1.369264226	0.349	0.51	Y_RNA	misc_RNA	Y RNA [Sou		12	32705403	32705506
ENSG0000022585	-0.090301252	0.349	0.51	DDX39B	protein_coding	DExD-box h	CHR_HSCHR		31569699	31586038
ENSG0000023921	0.680172282	0.349	0.51	RPS20P22	transcribed_pr	ribosomal p		8	38434347	38435664
ENSG0000016919	-0.212434629	0.349	0.51	CCDC126	protein_coding	coiled-coil c		7	23597382	23644708
ENSG0000015888	0.155637232	0.349	0.51	TOMM40L	protein_coding	translocase		1	161225939	161230746
ENSG0000027046	1.535666279	0.349	0.51	AC005034.2	lncRNA	novel transc		2	75697583	75697996
ENSG0000014880	0.339017289	0.349	0.51	FUOM	protein_coding	fucose muta		10	133355158	133358025
ENSG0000020121	1.227511666	0.349	0.51	Y_RNA	misc_RNA	Y RNA [Sou		3	152449272	152449384
ENSG0000022888	0.215656677	0.349	0.51	TRIM26	protein_coding	tripartite mc	CHR_HSCHR		30176913	30205889
ENSG0000027210	0.306974528	0.349	0.51	AL691432.2	lncRNA	artifact		1	1613758	1615795
ENSG0000023879	1.235275001	0.35	0.51	SNORD124	snoRNA	small nucleo		17	40027542	40027645
ENSG0000022462	-1.121044118	0.35	0.51	AC004975.1	processed_pse	ribosomal p		7	157263022	157263229
ENSG0000017962	-0.382753509	0.35	0.51	ZBTB42	protein_coding	zinc finger a		14	104800596	104804712
ENSG0000020526	-0.097236335	0.35	0.51	TMEM170B	protein_coding	transmembr		6	11537749	11583524
ENSG0000022506	0.15378864	0.35	0.51	PPP1R18	protein_coding	protein pho	CHR_HSCHR		30754873	30766379
ENSG0000022548	-0.132756206	0.35	0.51	NUTM2B-A	lncRNA	NUTM2B an		10	79661394	79826594
ENSG0000024402	-0.826419168	0.35	0.51	AC093591.1	processed_pse	ribosomal p		4	127812720	127813200
ENSG0000021384	-0.793961293	0.35	0.51	NA	NA	NA	NA	NA	NA	
ENSG0000011620	0.065240429	0.351	0.51	TMEM59	protein_coding	transmembr		1	54026681	54053504

ENSG0000017532	0.092416372	0.351	0.51	LSM1	protein_coding	LSM1 homo	8	38163335	38176730
ENSG0000023031	-0.314798373	0.351	0.51	TOMM20P	processed_pse	TOMM20 p	X	73223124	73223558
ENSG0000019890	-0.048354449	0.351	0.51	PRC1	protein_coding	protein regu	15	90966040	90995629
ENSG0000006736	0.156284373	0.351	0.51	METTL22	protein_coding	methyltrans	16	8621683	8649654
ENSG0000027344	0.558115115	0.351	0.51	AC093788.	lncRNA	novel transc	4	163529771	163530697
ENSG0000006448	1.289313188	0.351	0.51	BORCS8-M	protein_coding	BORCS8-M	19	19145569	19192158
ENSG0000009210	-0.075910155	0.351	0.51	SCFD1	protein_coding	sec1 family	14	30622311	30735850
ENSG0000010252	0.727410205	0.351	0.51	TNFSF13B	protein_coding	TNF superfa	13	108251240	108308484
ENSG0000016880	0.151138975	0.352	0.51	ADAL	protein_coding	adenosine d	15	43330672	43354569
ENSG0000026994	-1.33106862	0.352	0.51	AC069307.	lncRNA	novel transc	4	56960927	56961373
ENSG0000015106	-0.190097969	0.352	0.51	DCP1B	protein_coding	decapping m	12	1946053	2004535
ENSG0000024224	-0.088670546	0.352	0.51	ARFGAP3	protein_coding	ADP ribosyl	22	42796502	42858106
ENSG0000025471	1.483158016	0.352	0.51	AC080023.	processed_pse	guanine nuc	11	10272052	10272259
ENSG0000013230	0.058219618	0.352	0.51	IMMT	protein_coding	inner memb	2	86143932	86195770
ENSG0000020409	-0.946672432	0.353	0.51	NEU4	protein_coding	neuraminida	2	241808825	241817413
ENSG0000014244	0.171108802	0.353	0.51	TIMM29	protein_coding	translocase	19	10928811	10933535
ENSG0000005845	-0.150912402	0.353	0.51	CROCC	protein_coding	ciliary rootle	1	16740273	16972964
ENSG0000024035	-0.215648253	0.353	0.51	RPL23AP7	transcribed_pr	ribosomal p	2	113610502	113627090
ENSG0000013419	-0.274326393	0.353	0.51	TSPAN2	protein_coding	tetraspanin	1	115048011	115089503
ENSG0000027438	1.11944743	0.353	0.51	TMEM269	protein_coding	transmembr	1	42785007	42816619
ENSG0000014502	0.288658243	0.353	0.51	NICN1	protein_coding	nicolin 1 [Sc	3	49422333	49429326
ENSG0000025787	-1.295066479	0.353	0.51	NA	NA	NA	NA	NA	NA
ENSG0000018090	-0.095485674	0.354	0.51	KCTD2	protein_coding	potassium c	17	75032575	75065889
ENSG0000022983	-1.358361173	0.354	0.51	AL360091.1	lncRNA	novel transc	1	212357418	212358353
ENSG0000027250	1.821015192	0.354	0.51	RNU6-88P	snRNA	RNA, U6 sm	10	12116280	12116379
ENSG0000026159	0.115161425	0.354	0.51	TPBGL	protein_coding	trophoblast	11	75240774	75243704
ENSG0000026539	-1.027626814	0.354	0.51	AC084125.	lncRNA	novel transc	8	144512567	144513672
ENSG0000014450	-0.239001052	0.355	0.51	ANKMY1	protein_coding	ankyrin repe	2	240479422	240569209
ENSG0000025727	-0.63595445	0.355	0.51	AL928654.2	lncRNA	novel transc	14	105467793	105470617
ENSG0000028031	-1.025398102	0.355	0.51	AC092437.	TEC	tec	4	4820405	4821452
ENSG0000013604	0.090069891	0.355	0.51	APPL2	protein_coding	adaptor pro	12	105173297	105236203
ENSG0000023066	-0.823503604	0.355	0.52	SETSIP	protein_coding	SET like pro	1	92074533	92075441
ENSG0000012758	0.158422937	0.355	0.52	WDR24	protein_coding	WD repeat	16	684622	690444
ENSG0000022201	-1.12113974	0.356	0.52	FAM243A	protein_coding	family with	21	34400112	34401072
ENSG0000028008	0.29821017	0.356	0.52	AC126474.	TEC	TEC	12	100032325	100034074
ENSG0000007427	0.377457967	0.356	0.52	CDHR2	protein_coding	cadherin rel	5	176542511	176595974
ENSG0000008820	-0.048167759	0.356	0.52	DDX18	protein_coding	DEAD-box I	2	117814691	117832377
ENSG0000012452	-0.164676165	0.356	0.52	SIRT5	protein_coding	sirtuin 5 [So	6	13574529	13615158
ENSG0000027245	0.270309234	0.356	0.52	MRPL20-D	lncRNA	MRPL20 div	1	1409096	1410618
ENSG0000013095	-0.60004336	0.356	0.52	FBP2	protein_coding	fructose-bis	9	94558720	94593824
ENSG0000016822	0.152985263	0.356	0.52	ZCCHC4	protein_coding	zinc finger C	4	25312774	25370383
ENSG0000016408	0.087280787	0.356	0.52	RAD54L2	protein_coding	RAD54 like	3	51541144	51668667
ENSG0000000669	-0.169251381	0.356	0.52	COX10	protein_coding	cytochrome	17	14069490	14231736
ENSG0000012725	-0.847895566	0.356	0.52	PLAAT1	protein_coding	phospholipa	3	193241128	193277738
ENSG0000017653	-0.482156965	0.357	0.52	PHLDB3	protein_coding	pleckstrin h	19	43474954	43504935
ENSG0000027659	1.410292981	0.357	0.52	U2	snRNA	U2 spliceos	17	43266633	43266823
ENSG0000024040	1.076698446	0.357	0.52	AC012358.	lncRNA	novel transc	2	55282350	55346049
ENSG0000023715	0.971457779	0.357	0.52	CNTFR-AS	lncRNA	CNTFR antis	9	34568012	34583072
ENSG0000012562	-0.107196095	0.357	0.52	INSIG2	protein_coding	insulin induc	2	118088452	118110997
ENSG0000011353	-0.351022495	0.357	0.52	ST8SIA4	protein_coding	ST8 alpha-N	5	100806933	100903282
ENSG0000024425	0.129018565	0.357	0.52	PKD1P1	unprocessed_p	polycystin 1	16	16310341	16334190
ENSG0000025095	0.310537425	0.357	0.52	GLUD1P3	transcribed_un	glutamate c	10	73730562	73737311
ENSG0000016515	-0.055453823	0.358	0.52	ZHX1	protein_coding	zinc fingers	8	123248451	123275541
ENSG0000019622	-0.214197223	0.358	0.52	SRGAP3	protein_coding	SLIT-ROBO	3	8980591	9363053
ENSG0000020457	-0.222773065	0.358	0.52	PRR3	protein_coding	proline rich	6	30557280	30563723
ENSG0000026957	-0.733306699	0.358	0.52	AP001350.	lncRNA	novel transc	11	58611119	58612642
ENSG0000022379	0.779420243	0.358	0.52	ENTPD3-A	lncRNA	ENTPD3 ant	3	40313802	40453329
ENSG0000016644	0.133844432	0.358	0.52	DENND2B	protein_coding	DENN doma	11	8693351	8910951

ENSG0000019830	0.076363457	0.358	0.52	SDAD1	protein_coding	SDA1 doma	4	75940950	75990962
ENSG0000022124	2.215942876	0.358	0.52	NA	NA	NA	NA	NA	NA
ENSG0000013663	-0.071655165	0.358	0.52	VPS45	protein_coding	vacuolar pro	1	150067279	150145329
ENSG0000016393	-0.351123094	0.358	0.52	SFMBT1	protein_coding	Scm like wit	3	52903572	53046750
ENSG0000024467	0.451884576	0.358	0.52	LY6G5B	protein_coding	lymphocyte	CHR_HSCHR	31652558	31655955
ENSG0000007660	0.093152298	0.359	0.52	TRAF4	protein_coding	TNF recepto	17	28744005	28750956
ENSG0000017487	0.212510108	0.359	0.52	CNIH2	protein_coding	cornichon fa	11	66278175	66285301
ENSG0000014673	0.037615276	0.359	0.52	CCT6A	protein_coding	chaperonin	7	56051685	56063989
ENSG0000016071	0.07081368	0.359	0.52	CHRNB2	protein_coding	cholinergic	1	154567778	154580013
ENSG0000022468	-0.897612416	0.359	0.52	E2F6P2	processed_pse	E2F transcri	22	21141624	21142371
ENSG0000013718	-0.132840662	0.359	0.52	ZSCAN9	protein_coding	zinc finger a	6	28224886	28233487
ENSG0000016856	-0.095192707	0.359	0.52	SNRNP48	protein_coding	small nuclea	6	7590198	7611967
ENSG0000015525	0.070930407	0.359	0.52	MARVELD1	protein_coding	MARVEL do	10	97713173	97718150
ENSG0000027822	-0.167577655	0.359	0.52	RPS17	protein_coding	ribosomal p	CHR_HSCHR	82615842	82619653
ENSG0000000839	-1.036621604	0.36	0.52	MGST1	protein_coding	microsomal	12	16347142	16609259
ENSG0000025870	-0.677726807	0.36	0.52	SRP54-AS1	transcribed_un	SRP54 antis	14	34920858	34982532
ENSG0000014038	-0.072042099	0.36	0.52	HMG20A	protein_coding	high mobilit	15	77420412	77485607
ENSG0000015765	-1.818748138	0.36	0.52	PALM2AKA	protein_coding	PALM2 and	9	109498325	110172512
ENSG0000026770	0.918862298	0.36	0.52	AC015961.	lncRNA	novel transc	18	37243776	37247506
ENSG0000025198	2.08083215	0.36	0.52	RNU4ATAC	snRNA	RNA, U4atac	6	159720415	159720540
ENSG0000014263	-0.264746954	0.36	0.52	ARHGEF19	protein_coding	Rho guanine	1	16197854	16212652
ENSG0000027266	-1.513038435	0.36	0.52	AC073352.	lncRNA	novel transc	3	119497678	119498181
ENSG0000020203	1.166778382	0.36	0.52	SNORD38A	snoRNA	small nucleo	1	44777843	44777912
ENSG0000011223	0.109302242	0.36	0.52	FBXL4	protein_coding	F-box and l	6	98868535	98948006
ENSG0000018623	-0.218263479	0.36	0.52	ZNF749	protein_coding	zinc finger p	19	57435325	57447101
ENSG0000010239	0.136659045	0.361	0.52	GLA	protein_coding	galactosidas	X	101393273	101408012
ENSG0000013624	-0.091646518	0.361	0.52	NUP42	protein_coding	nucleoporin	7	23181841	23201011
ENSG0000016691	-0.160712117	0.361	0.52	MTMR10	protein_coding	myotubulari	15	30938941	30991628
ENSG0000023207	1.534906814	0.361	0.52	MRPL35P2	processed_pse	mitochondr	10	63634317	63634827
ENSG0000025248	1.002343587	0.361	0.52	SCARNA13	scaRNA	small Cajal	14	95533355	95533629
ENSG0000027577	-0.907436363	0.361	0.52	AC018630.	protein_coding	PRH1-PRR4	12	10845849	11171600
ENSG0000014216	0.079660524	0.361	0.52	IFNAR1	protein_coding	interferon a	21	33324429	33359864
ENSG0000027889	0.423654686	0.362	0.52	AC020951.	TEC	TEC	19	11856032	11858302
ENSG0000024342	-1.009140083	0.362	0.52	RPL23AP49	transcribed_pr	ribosomal p	3	75624275	75670156
ENSG0000010349	0.158659984	0.362	0.52	STX4	protein_coding	syntaxin 4 [S	16	31032889	31042975
ENSG0000013460	0.103666815	0.362	0.52	STK26	protein_coding	serine/threo	X	132023302	132075943
ENSG0000024827	0.463056818	0.362	0.52	SUMO2P17	transcribed_pr	SUMO2 pse	17	48874860	48908983
ENSG0000027252	0.909506823	0.362	0.52	AC099522.	lncRNA	novel transc	5	73497550	73498293
ENSG0000023321	-0.306128312	0.363	0.52	GPANK1	protein_coding	G-patch do	CHR_HSCHR	31643407	31648462
ENSG0000021011	0.506648745	0.363	0.52	MT-TW	Mt_tRNA	mitochondr	MT	5512	5579
ENSG0000022742	-0.506154129	0.363	0.52	MRPS18B	protein_coding	mitochondr	CHR_HSCHR	30607552	30616238
ENSG0000027629	-1.038906793	0.363	0.52	AC131159.	lncRNA	novel transc	12	118037869	118038081
ENSG0000012287	0.376344288	0.363	0.52	EGR2	protein_coding	early growth	10	62811996	62919900
ENSG0000027711	0.251355062	0.363	0.52	PLEKHM1	protein_coding	pleckstrin h	CHR_HSCHR	45435832	45490687
ENSG0000011617	-0.091685438	0.363	0.52	SCP2	protein_coding	sterol carrie	1	52927276	53051698
ENSG0000021495	-0.646868387	0.364	0.52	LRRC69	protein_coding	leucine rich	8	91101832	91219236
ENSG0000026012	-0.569580029	0.364	0.52	AC138028.	lncRNA	novel transc	16	88718615	88720459
ENSG0000027302	1.344191312	0.364	0.52	AL844908.	lncRNA	novel transc	21	44929653	44930112
ENSG0000006802	0.082206976	0.364	0.52	HDAC4	protein_coding	histone dea	2	239048168	239401654
ENSG0000010518	-0.125789545	0.364	0.52	ANKRD27	protein_coding	ankyrin repe	19	32597006	32676597
ENSG0000013531	0.335981961	0.364	0.53	KHDC1	protein_coding	KH domain	6	73241314	73310365
ENSG0000012015	-0.081533905	0.365	0.53	CAAP1	protein_coding	caspase acti	9	26840685	26892803
ENSG0000025725	1.531882997	0.365	0.53	AC124947.	lncRNA	novel transc	12	93316722	93377753
ENSG0000023671	0.593837035	0.365	0.53	SMAD9-IT1	lncRNA	SMAD9 intr	13	36849366	36850046
ENSG0000026696	-0.782303714	0.365	0.53	AP002449.	lncRNA	novel transc	18	12984694	12991173
ENSG0000023585	-1.015737844	0.365	0.53	AC005540.	lncRNA	novel transc	2	190880797	190882059
ENSG0000019892	-0.095937191	0.365	0.53	DCLRE1A	protein_coding	DNA cross-	10	113834725	113854383
ENSG0000026109	0.255907308	0.365	0.53	AP000766.	lncRNA	novel transc	11	107312132	107316271

ENSG0000011609	0.139272394	0.365	0.53	PLEKHA3	protein_codinç	pleckstrin hc		2	178480457	178516463
ENSG0000026266	-0.415020735	0.365	0.53	OVCA2	protein_codinç	OVCA2 serii		17	2042022	2043425
ENSG0000027995	-2.885318903	0.365	0.53	NA	NA	NA	NA	NA	NA	
ENSG0000027530	0.285683173	0.365	0.53	MCCC2	protein_codinç	methylcrotc	CHR_HSCHR	70802600	70819771	
ENSG0000020410	0.14357217	0.365	0.53	TRAF3IP1	protein_codinç	TRAF3 intera		2	238320441	238400897
ENSG0000026050	1.400666344	0.365	0.53	AC010336.2	lncRNA	novel transc		19	7918652	7919157
ENSG0000026029	0.896328674	0.365	0.53	AC092115.1	processed_pse	non-POU d		16	69756536	69757937
ENSG0000021544	0.277860635	0.366	0.53	NPEPL1	protein_codinç	aminopeptic		20	58689131	58719238
ENSG0000020557	-0.643113698	0.366	0.53	SERF1B	protein_codinç	small EDRK-		5	70025247	70043113
ENSG0000021853	-0.34611855	0.366	0.53	MIF-AS1	lncRNA	MIF antisens		22	23894426	23898930
ENSG0000027935	-0.514394062	0.366	0.53	NA	NA	NA	NA	NA	NA	
ENSG0000025921	-1.144686666	0.366	0.53	AC084757.1	processed_pse	oxidation re		15	48725338	48725827
ENSG0000021770	0.827339937	0.366	0.53	AC073263.1	lncRNA	novel transc		2	74123965	74135640
ENSG0000026456	0.834691198	0.366	0.53	DCXR-DT	lncRNA	DCXR diverç		17	82037905	82039380
ENSG0000000813	0.073536447	0.366	0.53	NADK	protein_codinç	NAD kinase		1	1751232	1780457
ENSG0000027356	-0.57122443	0.366	0.53	AC131009.3	lncRNA	novel transc		12	131934642	131934928
ENSG0000013073	-0.16715566	0.366	0.53	METTL26	protein_codinç	methyltrans		16	634427	636366
ENSG0000009999	-0.057760787	0.367	0.53	SF3A1	protein_codinç	splicing fact		22	30331988	30356919
ENSG0000022603	-0.335699851	0.367	0.53	STK19	protein_codinç	serine/threc	CHR_HSCHR	32047618	32057890	
ENSG0000013138	0.072185272	0.367	0.53	SLC6A6	protein_codinç	solute carrie		3	14402576	14489349
ENSG0000016028	-0.288757856	0.367	0.53	SPATC1L	protein_codinç	spermatoge		21	46161148	46184476
ENSG0000027285	0.749489853	0.367	0.53	AC069544.1	lncRNA	novel transc		10	14877688	14878686
ENSG0000012306	0.051258899	0.367	0.53	MED13L	protein_codinç	mediator cc		12	115957905	116277693
ENSG0000020802	-0.882065871	0.367	0.53	MIR616	miRNA	microRNA 6		12	57519163	57519259
ENSG0000021260	0.922563025	0.367	0.53	SNORA3B	snoRNA	small nucleo		11	8685439	8685569
ENSG0000023301	1.022939115	0.367	0.53	HDAC1P2	transcribed_pr	histone dea		1	220625740	220628056
ENSG0000023544	1.793042774	0.367	0.53	AC098934.2	processed_pse	cytochrome		1	202767229	202767418
ENSG0000025666	0.324310496	0.367	0.53	AC112777.1	processed_pse	ubiquitin-lik		12	20551590	20553012
ENSG0000027310	-0.913740341	0.367	0.53	AL121929.2	lncRNA	novel transc		10	103608619	103610050
ENSG0000027969	-0.563160684	0.367	0.53	AC099521.3	TEC	novel transc		16	68367325	68370262
ENSG0000024451	1.485881037	0.368	0.53	AC008267.5	transcribed_un	general tran		7	66480394	66490108
ENSG0000026114	-0.531245133	0.368	0.53	AC093525.4	lncRNA	novel transc		16	2569043	2571936
ENSG0000027602	-0.683897277	0.368	0.53	TFPT	protein_codinç	TCF3 fusion	CHR_HSCHR	54107066	54115801	
ENSG0000015165	-0.133300443	0.368	0.53	KIN	protein_codinç	Kin17 DNA		10	7750962	7787993
ENSG0000018018	-1.107812317	0.368	0.53	HMGB1P14	processed_pse	high mobilit		14	58284773	58285363
ENSG0000027623	-0.141526382	0.368	0.53	RNH1	protein_codinç	ribonucleas	CHR_HSCHR	494512	507300	
ENSG0000023813	0.184183039	0.368	0.53	EHMT2	protein_codinç	euchromati	CHR_HSCHR	31956231	31974166	
ENSG0000012110	-0.218886245	0.368	0.53	FAM117A	protein_codinç	family with		17	49710332	49789180
ENSG0000019744	0.113999172	0.369	0.53	GSTK1	protein_codinç	glutathione		7	143244093	143270854
ENSG0000017480	0.098973113	0.369	0.53	CD248	protein_codinç	CD248 mole		11	66314494	66317044
ENSG0000025962	-0.114017685	0.369	0.53	AC125257.1	lncRNA	novel transc		17	41848518	41851447
ENSG0000014338	0.258060488	0.369	0.53	CTSK	protein_codinç	cathepsin K		1	150796208	150808260
ENSG0000022732	-0.150819829	0.37	0.53	RXRB	protein_codinç	retinoid X re	CHR_HSCHR	33115214	33122477	
ENSG0000022791	1.068415338	0.37	0.53	AC092634.3	transcribed_pr	TP53 target		7	63924787	63926306
ENSG0000024198	1.14045694	0.37	0.53	RPL7AP2	processed_pse	ribosomal p		14	39156742	39157852
ENSG0000026142	-0.335882913	0.37	0.53	NA	NA	NA	NA	NA	NA	
ENSG0000026213	1.235420823	0.371	0.53	AC092115.3	lncRNA	novel transc		16	69727013	69742563
ENSG0000026110	-0.500962197	0.371	0.53	AC234775.3	lncRNA	novel transc	X	101627868	101628523	
ENSG0000019755	0.131746971	0.371	0.53	TTC30A	protein_codinç	tetratricope		2	177612999	177618742
ENSG0000024275	-0.722506375	0.371	0.53	LINC00882	lncRNA	long interge		3	106449775	107240671
ENSG0000023675	-0.433469013	0.371	0.53	MKLN1-AS	lncRNA	MKLN1 anti		7	131309469	131328312
ENSG0000027786	-0.208976697	0.372	0.53	CCDC125	protein_codinç	coiled-coil	CHR_HSCHR	69281282	69332741	
ENSG0000023240	-2.065327414	0.372	0.53	RPL27P5	processed_pse	ribosomal p		2	63108118	63108429
ENSG0000022682	0.736959379	0.372	0.53	AC006001.2	lncRNA	novel transc		7	66654538	66671523
ENSG0000021948	-0.085297372	0.372	0.53	NBPF1	protein_codinç	NBPF memb		1	16562319	16613562
ENSG0000020069	-2.085609209	0.373	0.53	AL136038.1	snoRNA			14	63651296	63651499
ENSG0000023771	0.904847676	0.373	0.53	Z95152.1	processed_pse	nudix (nucle		6	36091991	36092646
ENSG0000013838	-0.061858036	0.373	0.53	ASNSD1	protein_codinç	asparagine		2	189661385	189670831

ENSG0000021453	-0.581155205	0.373	0.53	ZNF705E	protein_coding	zinc finger p		11	71814045	71821548
ENSG0000026184	0.748413493	0.373	0.53	AC093249.6	lncRNA	uncharacter		16	30697707	30699058
ENSG0000024279	-1.628323793	0.373	0.53	AC117395.1	lncRNA	novel transc		3	149976755	149979355
ENSG0000016634	-0.08202671	0.374	0.53	TPP1	protein_coding	tripeptidyl p		11	6612768	6619448
ENSG0000025460	0.626912482	0.374	0.53	AP002336.2	lncRNA	novel transc		11	70282367	70363368
ENSG0000026854	-1.796931565	0.374	0.53	VN1R107P	unprocessed_p	vomeronasa		19	57459912	57460120
ENSG0000027286	-1.400971751	0.374	0.53	NA	NA	NA	NA	NA	NA	
ENSG0000023701	-0.474751164	0.374	0.54	AL031186.1	lncRNA	novel transc		22	29260889	29262037
ENSG0000016402	0.073931785	0.375	0.54	AIMP1	protein_coding	aminoacyl t		4	106315544	106349456
ENSG0000019804	-0.081245804	0.375	0.54	MAK16	protein_coding	MAK16 hom		8	33485182	33501262
ENSG0000014519	-0.071794649	0.375	0.54	EIF2B5	protein_coding	eukaryotic t		3	184135038	184146127
ENSG0000020493	-0.228486284	0.375	0.54	ATP6V0E2-	lncRNA	ATP6V0E2 a		7	149867697	149880610
ENSG0000010977	-0.103896721	0.375	0.54	UFSP2	protein_coding	UFM1 speci		4	185399537	185425979
ENSG0000021343	0.931693112	0.375	0.54	RPLP1P6	transcribed_pr	ribosomal p		5	151765859	151766378
ENSG0000016877	0.089590689	0.375	0.54	CXXC4	protein_coding	CXXC finger		4	104468308	104494901
ENSG0000012708	-0.179680281	0.376	0.54	IPPK	protein_coding	inositol-per		9	92613183	92670131
ENSG0000016998	-0.177596817	0.376	0.54	ZNF35	protein_coding	zinc finger p		3	44648732	44660791
ENSG0000019842	0.256086318	0.376	0.54	ZNF69	protein_coding	zinc finger p		19	11887782	11914329
ENSG0000010496	-0.056224192	0.376	0.54	SGTA	protein_coding	small glutan		19	2754715	2783282
ENSG0000027317	0.343504301	0.376	0.54	SNURF	protein_coding	SNRPN upst		15	24954986	24977850
ENSG0000012621	-0.670032289	0.376	0.54	F10	protein_coding	coagulation		13	113122799	113149529
ENSG0000016594	-0.501721924	0.376	0.54	IFI27	protein_coding	interferon a		14	94104836	94116698
ENSG0000027003	1.464827814	0.376	0.54	AL020997.3	lncRNA	novel transc		1	27819983	27820341
ENSG0000025613	0.745761502	0.376	0.54	AC007637.1	lncRNA	novel transc		12	109111218	109125594
ENSG0000010209	-0.095782585	0.377	0.54	SCML2	protein_coding	Scm polyco	X		18239313	18354688
ENSG0000019718	-0.447655207	0.377	0.54	CH17-340M	lncRNA	uncharacter	X		154424380	154428479
ENSG0000026603	-1.587838908	0.376	0.54	NA	NA	NA	NA	NA	NA	
ENSG0000027300	-1.471294629	0.377	0.54	NA	NA	NA	NA	NA	NA	
ENSG0000020170	-1.777901652	0.377	0.54	SNORD113	snoRNA	small nucleo		14	100929919	100929990
ENSG0000007066	-0.037969518	0.377	0.54	ASNS	protein_coding	asparagine s		7	97851677	97872542
ENSG0000025367	0.961405733	0.377	0.54	AC027117.1	lncRNA	novel transc		8	17808361	17822183
ENSG0000027890	0.542470956	0.377	0.54	CU633906.2	lncRNA	novel transc		21	6318434	6360415
ENSG0000026677	-0.585866567	0.377	0.54	SH3GL1P1	transcribed_pr	SH3 domain		17	32039974	32042828
ENSG0000009173	-0.112433388	0.377	0.54	ZC3HC1	protein_coding	zinc finger C		7	130018287	130051451
ENSG0000022601	-0.423490487	0.377	0.54	CCT8P1	processed_pse	chaperonin		1	147203276	147204932
ENSG0000025367	-1.222092771	0.377	0.54	TAGLN2P1	processed_pse	transgelin 2		8	106697427	106698013
ENSG0000015667	0.069610003	0.378	0.54	SAMD8	protein_coding	sterile alpha		10	75099586	75182123
ENSG0000022304	-2.017077482	0.378	0.54	RNU6-130P	snRNA	RNA, U6 sm		6	82210338	82210440
ENSG0000010471	0.115651933	0.378	0.54	ERICH1	protein_coding	glutamate r		8	614746	738106
ENSG0000021367	0.16155204	0.378	0.54	NCKIPSD	protein_coding	NCK interac		3	48673844	48686364
ENSG0000022712	0.257337887	0.378	0.54	NEU1	protein_coding	neuraminida	CHR_HSCHR		31934136	31939383
ENSG0000022335	-0.91512246	0.378	0.54	HLA-U	unprocessed_p	major histo	CHR_HSCHR		29923817	29924002
ENSG0000023469	-0.333386056	0.379	0.54	EPCAM-DT	lncRNA	EPCAM dive		2	47192405	47345074
ENSG0000027861	-0.278398268	0.379	0.54	BEND3P3	processed_pse	BEN domain		10	79682997	79685436
ENSG0000023554	-0.826508606	0.379	0.54	AC103923.1	lncRNA	novel transc		1	62688482	62710694
ENSG0000027677	-2.009207239	0.379	0.54	ARMC4P1	unprocessed_p	armadillo re	CHR_HSCHR		27288669	27288857
ENSG0000017940	0.135791432	0.379	0.54	GEMIN4	protein_coding	gem nuclea		17	744421	753999
ENSG0000017399	-0.507238491	0.379	0.54	TCAP	protein_coding	titin-cap [So		17	39665349	39666554
ENSG0000011968	-0.078805021	0.379	0.54	MLH3	protein_coding	mutL homo		14	75013769	75051532
ENSG0000023332	-1.12768449	0.379	0.54	PFN1P1	processed_pse	profilin 1 ps		1	171670517	171670939
ENSG0000027368	-2.02487963	0.38	0.54	AC109583.3	processed_pse	biliverdin re		3	46918807	46918935
ENSG0000011167	-0.731571472	0.38	0.54	PTPN6	protein_coding	protein tyro		12	6946468	6961316
ENSG0000021541	0.533489151	0.38	0.54	PSMA6P1	processed_pse	proteasome	Y		13286638	13287378
ENSG0000019780	-0.22592458	0.38	0.54	ZNF461	protein_coding	zinc finger p		19	36636618	36666853
ENSG0000010497	-0.139973896	0.381	0.54	MED25	protein_coding	mediator co		19	49818282	49840383
ENSG0000013094	-0.162004844	0.381	0.54	CASZ1	protein_coding	castor zinc f		1	10636604	10796650
ENSG0000027511	-0.444725032	0.381	0.54	MBOAT7	protein_coding	membrane	CHR_HSCHR		54173853	54190413
ENSG0000022772	0.90268258	0.381	0.54	GCOM2	processed_pse	GRINL1B co		4	68038544	68039647

ENSG0000027071	-1.582486624	0.381	0.54	AL162431.3	processed_pse	cell division		1	180970837	180971247
ENSG0000025514	1.280084498	0.381	0.54	AP006621.2	lncRNA	novel transc		11	781645	782105
ENSG0000017730	0.095331068	0.381	0.54	TOP3A	protein_coding	DNA topois		17	18271428	18315007
ENSG0000021320	0.536552371	0.381	0.54	AL049697.1	protein_coding	novel transc		6	87408012	87511634
ENSG0000026601	0.581765064	0.382	0.54	MIR4477B	miRNA	microRNA 4		9	63819574	63819654
ENSG0000027930	1.136169642	0.382	0.54	AC008761.3	TEC	TEC		19	17622447	17623338
ENSG0000017757	0.106078548	0.382	0.54	C18orf32	protein_coding	chromosom		18	49477243	49487252
ENSG0000027307	-1.771678621	0.382	0.54	AC110609.1	lncRNA	novel transc		4	128552590	128553416
ENSG0000027411	-1.771678621	0.382	0.54	NA	NA	NA	NA	NA	NA	
ENSG0000010767	0.085080276	0.382	0.54	NSMCE4A	protein_coding	NSE4 homo		10	121957091	121975217
ENSG0000006376	-0.244786589	0.382	0.54	ADCK1	protein_coding	aarF domair		14	77800109	77935014
ENSG0000020729	1.995415526	0.382	0.54	SNORD7	snoRNA	small nucleo		17	35573657	35573753
ENSG0000023476	-0.373229037	0.382	0.54	E2F6P1	processed_pse	E2F transcri		22	18833694	18834438
ENSG0000010209	-0.175994676	0.383	0.54	PIM2	protein_coding	Pim-2 proto	X		48913182	48919024
ENSG0000018864	0.049636046	0.383	0.54	PTAR1	protein_coding	protein prer		9	69709522	69760011
ENSG0000010472	0.030996802	0.383	0.54	NEFM	protein_coding	neurofilame		8	24913758	24919098
ENSG0000020050	-2.133371079	0.383	0.54	Y_RNA	misc_RNA	Y RNA [Sou		9	37160137	37160237
ENSG0000027329	-2.133371079	0.383	0.54	AC093458.1	lncRNA	novel transc		7	149881359	149881580
ENSG0000008654	-0.224637267	0.383	0.54	ITPKC	protein_coding	inositol-tris		19	40717112	40740860
ENSG0000025543	1.063371599	0.383	0.54	AP001267.3	lncRNA	novel transc		11	118510273	118531094
ENSG0000017316	0.209384558	0.383	0.54	COMMD1	protein_coding	copper met		2	61888724	62147247
ENSG0000022843	0.650768594	0.383	0.54	AL139260.1	lncRNA	novel transc		1	38859912	38965038
ENSG0000023262	-1.412278871	0.383	0.54	AC021876.1	processed_pse	ribosomal p		7	23365618	23365976
ENSG0000014778	0.101978868	0.383	0.54	ZNF7	protein_coding	zinc finger p		8	144827464	144847509
ENSG0000028017	0.425041731	0.384	0.54	AC104447.1	TEC	TEC		3	47346950	47349073
ENSG0000008618	0.076257381	0.384	0.54	DIMT1	protein_coding	DIMT1 rRNA		5	62347284	62403939
ENSG0000018870	-0.182765773	0.384	0.54	ZBED6CL	protein_coding	ZBED6 C-te		7	150322639	150332721
ENSG0000011657	-0.081068791	0.384	0.54	RHOU	protein_coding	ras homolog		1	228735479	228746664
ENSG0000027549	0.67308355	0.384	0.54	AC133552.5	lncRNA	novel transc		16	25106569	25107102
ENSG0000016824	-0.059044302	0.384	0.55	UBTD2	protein_coding	ubiquitin dc		5	172209646	172283764
ENSG0000016728	-0.611004057	0.384	0.55	RBFOX3	protein_coding	RNA binding		17	79089345	79516148
ENSG0000009296	-0.036546743	0.385	0.55	DPYSL2	protein_coding	dihydropyri		8	26514031	26658178
ENSG0000013951	-0.166966522	0.385	0.55	LNX2	protein_coding	ligand of nu		13	27545913	27620529
ENSG0000020739	0.997212804	0.385	0.55	SNORA20	snoRNA	small nucleo		6	159780250	159780381
ENSG0000026727	-0.84994862	0.385	0.55	PARD6G-A	lncRNA	PARD6G an		18	80147924	80179839
ENSG0000024630	0.640029988	0.385	0.55	AC116535.1	lncRNA	novel transc		11	10809204	10822931
ENSG0000008599	0.103549739	0.385	0.55	POMGNT1	protein_coding	protein O-li		1	46188682	46220305
ENSG0000027897	-0.278470474	0.385	0.55	AC093909.6	TEC	tec		4	188740507	188741281
ENSG0000016587	-0.360777465	0.385	0.55	SHLD2P1	transcribed_un	shieldin con		10	46610474	46652025
ENSG0000016493	0.057774559	0.386	0.55	DCAF13	protein_coding	DDB1 and C		8	103414714	103443453
ENSG0000018615	-0.209698661	0.386	0.55	WWOX	protein_coding	WW domair		16	78099430	79212667
ENSG0000026728	1.127335262	0.386	0.55	AC005306.1	lncRNA	novel transc		19	1989401	1990370
ENSG0000021356	-0.660962459	0.386	0.55	AL390039.1	processed_pse	general trar	X		107131659	107132269
ENSG0000026446	-0.842879122	0.386	0.55	NA	NA	NA	NA	NA	NA	
ENSG0000011596	-0.075808159	0.386	0.55	RND3	protein_coding	Rho family C		2	150468195	150539011
ENSG0000021628	0.249218244	0.386	0.55	AC078819.1	processed_pse	phosphogly		12	104030779	104031543
ENSG0000023144	0.943944901	0.386	0.55	AC124944.1	processed_pse	protein pho		3	195937243	195938739
ENSG0000026027	1.322281972	0.387	0.55	AC093525.2	protein_coding	novel protei		16	2496032	2520218
ENSG0000027416	-0.460657223	0.387	0.55	GOLGA6L1	protein_coding	golgin A6 fa	CHR_HSCHR		82418974	82428498
ENSG0000025084	0.899404335	0.387	0.55	AC021087.2	processed_pse	protein pho		5	288833	290321
ENSG0000014623	0.254584208	0.387	0.55	NFKBIE	protein_coding	NFKB inhibi		6	44258166	44265788
ENSG0000023656	-0.2559164	0.387	0.55	PPP1R11	protein_coding	protein pho	CHR_HSCHR		30061865	30065490
ENSG0000026906	0.614623756	0.387	0.55	ZNF728	protein_coding	zinc finger p		19	22974883	23003176
ENSG0000017430	-0.078706984	0.387	0.55	ZHX3	protein_coding	zinc fingers		20	41178448	41317672
ENSG0000025046	-0.103889498	0.387	0.55	LRRC37BP1	transcribed_un	leucine rich		17	30629680	30637466
ENSG0000017690	-0.616736208	0.388	0.55	MAMSTR	protein_coding	MEF2 activa		19	48712725	48719725
ENSG0000025930	0.72351025	0.388	0.55	ZHX1-C8or	protein_coding	ZHX1-C8or		8	123226189	123274284
ENSG0000027458	1.555650332	0.388	0.55	RNU2-1	snRNA	RNA, U2 sm		17	43233787	43233977

ENSG0000012419	-0.034518254	0.388	0.55	SRSF6	protein_coding	serine and a	20	43457893	43466046
ENSG0000015595	-0.126832456	0.388	0.55	TMBIM4	protein_coding	transmembr	12	66135846	66170072
ENSG0000023041	0.889750178	0.388	0.55	LINC01786	lncRNA	long interge	1	1275223	1280420
ENSG0000020434	0.320779268	0.388	0.55	STK19	protein_coding	serine/threo	6	31971091	31982821
ENSG0000024566	1.262900262	0.388	0.55	AC006064.1	lncRNA	novel transc	12	6543504	6544931
ENSG0000025350	-1.238012355	0.388	0.55	AC104393.1	lncRNA	novel transc	8	41275115	41277003
ENSG0000026158	1.146692437	0.389	0.55	AL121753.1	protein_coding	novel transc	20	35267885	35280043
ENSG0000016241	-0.522904354	0.389	0.55	ZSWIM5	protein_coding	zinc finger S	1	45016399	45306209
ENSG0000022852	-0.199115854	0.389	0.55	RING1	protein_coding	ring finger p	CHR_HSCHR	33186401	33190628
ENSG0000026146	-0.791303607	0.389	0.55	UBE2MP1	processed_pse	ubiquitin co	16	35169692	35170241
ENSG0000013189	0.116175834	0.389	0.55	LLGL1	protein_coding	LLGL scribbl	17	18225635	18244875
ENSG0000022650	0.711677614	0.389	0.55	LINC01918	lncRNA	long interge	2	105144113	105145424
ENSG0000026064	0.827144611	0.389	0.55	AC092718.3	protein_coding	novel protei	16	81053587	81096296
ENSG0000015959	-0.110499946	0.389	0.55	TMEM69	protein_coding	transmembr	1	45688181	45694436
ENSG0000027538	1.278255044	0.389	0.55	AC019206.1	processed_pse	Zinc finger p	2	69844509	69844933
ENSG0000027348	-0.8477198	0.389	0.55	NA	NA	NA	NA	NA	NA
ENSG0000022387	-1.028483663	0.389	0.55	PPIAP53	processed_pse	peptidylprol	17	15506866	15507354
ENSG0000012676	0.103594468	0.389	0.55	TIMM17B	protein_coding	translocase	X	48893447	48898143
ENSG0000013509	-0.111336541	0.39	0.55	USP30	protein_coding	ubiquitin sp	12	109023089	109088023
ENSG0000020736	-1.99310728	0.39	0.55	RNU6-178P	snRNA	RNA, U6 sm	8	28416448	28416551
ENSG0000022589	0.364201265	0.39	0.55	NA	NA	NA	NA	NA	NA
ENSG0000026699	-3.951480997	0.39	0.55	DHX40P1	transcribed_un	DEAH-box I	17	59976009	60002384
ENSG0000027165	0.513324086	0.39	0.55	AL161729.4	lncRNA	novel transc	9	95514045	95514520
ENSG0000027472	1.272503935	0.39	0.55	AL137028.1	processed_pse	IQ motif and	20	64327418	64327972
ENSG0000024335	1.132965203	0.39	0.55	NA	NA	NA	NA	NA	NA
ENSG0000014845	0.219318651	0.39	0.55	MSRB2	protein_coding	methionine	10	23095579	23122013
ENSG0000026023	0.567592308	0.39	0.55	AC099778.1	lncRNA	novel transc	3	47379089	47380999
ENSG0000025674	-0.795060579	0.391	0.55	KDM2B-DT	lncRNA	KDM2B dive	12	121580792	121593504
ENSG0000025450	0.12213445	0.391	0.55	CHMP4A	protein_coding	charged mu	14	24209615	24213830
ENSG0000025168	0.64080332	0.391	0.55	AC079140.5	processed_pse	novel zinc fi	4	269866	270175
ENSG0000013305	0.075447277	0.391	0.55	DSTYK	protein_coding	dual serine/	1	205142505	205211702
ENSG0000021330	1.113596161	0.391	0.55	AC008481.2	processed_pse	ribosomal p	19	11521968	11522498
ENSG0000011149	-0.197641875	0.392	0.55	TBC1D30	protein_coding	TBC1 doma	12	64759484	64881033
ENSG0000027563	-0.508244189	0.392	0.55	AL035461.2	lncRNA	novel transc	20	6000418	6000941
ENSG0000011498	-0.062418403	0.392	0.55	KANSL3	protein_coding	KAT8 regula	2	96593170	96642787
ENSG0000013916	0.050585768	0.392	0.55	ETNK1	protein_coding	ethanolamir	12	22625075	22690665
ENSG0000022453	0.088717695	0.392	0.55	SMIM13	protein_coding	small integr	6	11093834	11138733
ENSG0000002357	0.164345662	0.392	0.55	GLRX2	protein_coding	glutaredoxir	1	193090866	193106114
ENSG0000016315	0.09843638	0.392	0.55	SCNM1	protein_coding	sodium char	1	151156664	151170296
ENSG0000018878	0.186888005	0.392	0.55	ZNF548	protein_coding	zinc finger p	19	57389850	57402992
ENSG0000024867	0.668449762	0.392	0.55	ALG1L9P	lncRNA	ALG1 like 9,	11	71673885	71818238
ENSG0000027952	-0.35700907	0.392	0.55	AC115618.1	TEC	TEC	X	48580741	48581165
ENSG0000021386	0.140855045	0.392	0.55	YBX1P10	processed_pse	Y-box bindi	9	35971344	35972318
ENSG0000002827	-0.302510039	0.392	0.55	POU2F2	protein_coding	POU class 2	19	42086110	42196585
ENSG0000023096	1.150559527	0.393	0.55	AC233266.1	processed_pse	pseudogene	2	91578478	91578573
ENSG0000025611	0.936941452	0.393	0.55	AP001453.2	lncRNA	novel transc	11	64229214	64234352
ENSG0000016626	0.065140898	0.393	0.55	COX11	protein_coding	cytochrome	17	54951902	54968703
ENSG0000015075	-0.034508111	0.393	0.55	CCT5	protein_coding	chaperonin	5	10249929	10266389
ENSG0000020468	-0.233148653	0.393	0.55	STARD7-AS	lncRNA	STARD7 ant	2	96208403	96243353
ENSG0000011532	0.206364939	0.393	0.55	DOK1	protein_coding	docking pro	2	74549026	74557554
ENSG0000023343	1.097871338	0.393	0.55	NA	NA	NA	NA	NA	NA
ENSG0000022789	0.419733151	0.393	0.55	AL731569.1	lncRNA	novel transc	10	86521945	86525101
ENSG0000019800	-0.063028969	0.393	0.55	NOL8	protein_coding	nucleolar pr	9	92297358	92325636
ENSG0000018886	0.506838345	0.393	0.55	ZNF563	protein_coding	zinc finger p	19	12317477	12333720
ENSG0000024423	-0.616240581	0.394	0.55	AC007009.1	lncRNA	novel transc	7	8262233	8262821
ENSG0000007692	-0.064274188	0.394	0.55	XAB2	protein_coding	XPA binding	19	7619525	7629545
ENSG0000026420	-0.420217387	0.394	0.55	AGAP7P	unprocessed_p	ArfGAP with	10	46109621	46131358
ENSG0000024404	0.127793797	0.394	0.55	TMEM199	protein_coding	transmembr	17	28357642	28363683

ENSG0000018551	0.219501037	0.394	0.55	L3MBTL1	protein_coding	L3MBTL hist	20	43489442	43550954
ENSG0000027209	0.632372165	0.394	0.55	AC087623.2	lncRNA	novel transc	8	38382364	38383461
ENSG0000027491	-0.601271747	0.395	0.56	NA	NA	NA	NA	NA	NA
ENSG0000024336	1.144870235	0.395	0.56	MCCC1-AS	lncRNA	MCCC1 anti	3	183016255	183017808
ENSG0000020042	1.876122322	0.395	0.56	Y_RNA	misc_RNA	Y RNA [Sou	12	50743568	50743684
ENSG0000021386	-0.294109904	0.395	0.56	AC044787.1	processed_pse	ribosomal p	15	47730144	47730935
ENSG0000008184	0.442580794	0.395	0.56	PCDHA6	protein_coding	protocadhe	5	140827958	141012347
ENSG0000025469	0.49116559	0.395	0.56	AP001893.1	lncRNA	novel transc	11	126208611	126209027
ENSG0000015805	0.766075212	0.395	0.56	DUSP2	protein_coding	dual specific	2	96143169	96145440
ENSG0000026112	0.717349215	0.395	0.56	AC009065.5	lncRNA	novel transc	16	2094830	2097026
ENSG0000023018	0.315873771	0.395	0.56	AC008267.2	transcribed_un	RAB guanin	7	66427949	66453634
ENSG0000027078	1.958174992	0.396	0.56	AC135983.6	processed_pse	novel pseuc	15	32461666	32461809
ENSG0000011728	-0.618924851	0.396	0.56	CD160	protein_coding	CD160 mole	1	145719471	145739288
ENSG0000026137	-0.414129889	0.396	0.56	NA	NA	NA	NA	NA	NA
ENSG0000021368	0.561088692	0.396	0.56	AC002056.1	processed_pse	small nuclea	22	50754675	50755434
ENSG0000022848	0.353466812	0.396	0.56	C2orf92	protein_coding	chromosom	2	97664217	97703064
ENSG0000015829	0.060801385	0.396	0.56	CUL4B	protein_coding	cullin 4B [Sc	X	120523858	120575794
ENSG0000027811	-0.507578488	0.396	0.56	MUC20	protein_coding	mucin 20, c	CHR_HSCHR	195720882	195741123
ENSG0000011556	-0.089802146	0.396	0.56	ZNF142	protein_coding	zinc finger p	2	218637916	218659655
ENSG0000028012	0.198819117	0.397	0.56	AC073857.1	TEC	novel transc	12	123152324	123153377
ENSG0000024368	1.434520865	0.397	0.56	RPLP1P11	processed_pse	ribosomal p	17	16044621	16044961
ENSG0000009314	-0.067079336	0.397	0.56	ECHDC1	protein_coding	ethylmalony	6	127288712	127343609
ENSG0000014767	0.08484869	0.397	0.56	UTP23	protein_coding	UTP23 smal	8	116766505	116849463
ENSG0000015224	-0.133345205	0.397	0.56	HAUS1	protein_coding	HAUS augm	18	46104378	46128333
ENSG0000011323	-0.26525734	0.397	0.56	PDE8B	protein_coding	phosphodie	5	77210449	77428256
ENSG0000022857	-1.012101255	0.398	0.56	AC010731.1	lncRNA	novel transc	2	206606497	206609812
ENSG0000010033	-0.340487188	0.398	0.56	MTMR3	protein_coding	myotubulari	22	29883169	30030868
ENSG0000019849	-0.175610093	0.398	0.56	NBR2	lncRNA	neighbor of	17	43125551	43153671
ENSG0000020414	-0.140938055	0.398	0.56	ASAH2B	protein_coding	N-acylsphin	10	50739318	50816495
ENSG0000025347	-0.862207856	0.398	0.56	AC091185.1	lncRNA	novel transc	8	25425521	25426580
ENSG0000014445	0.155445518	0.398	0.56	SPAG16	protein_coding	sperm assoc	2	213284379	214410501
ENSG0000027155	1.013797672	0.398	0.56	BNIP3P11	processed_pse	BCL2 interac	7	64678954	64687393
ENSG0000004208	0.11349038	0.398	0.56	TDP1	protein_coding	tyrosyl-DNA	14	89954939	90044764
ENSG0000027284	0.321276356	0.399	0.56	AL139393.3	lncRNA	novel transc	6	160990318	160992342
ENSG0000007418	-0.17125682	0.399	0.56	NOTCH3	protein_coding	notch recep	19	15159038	15200995
ENSG0000027857	-0.36190436	0.399	0.56	AC007325.3	pseudogene		KI270734.1	59711	60316
ENSG0000018571	-0.108655666	0.399	0.56	MOSMO	protein_coding	modulator c	16	22007638	22087534
ENSG0000027381	-1.458917765	0.399	0.56	AC123788.2	processed_pse	mastermind	11	3335125	3335407
ENSG0000027280	0.673948423	0.399	0.56	AC021851.1	lncRNA	novel transc	2	183178806	183215414
ENSG0000027255	-0.830393233	0.399	0.56	GTF2IP13	transcribed_un	general trar	7	45769105	45815263
ENSG0000018637	-0.098663007	0.399	0.56	ZNF75D	protein_coding	zinc finger p	X	135248920	135344109
ENSG0000015991	0.359359458	0.399	0.56	ZNF233	protein_coding	zinc finger p	19	44259880	44275317
ENSG0000016086	-0.386664984	0.399	0.56	FGFR4	protein_coding	fibroblast gr	5	177086905	177098144
ENSG0000018826	0.503103858	0.399	0.56	HYKK	protein_coding	hydroxylysir	15	78507564	78537372
ENSG0000010917	0.05903683	0.4	0.56	SLAIN2	protein_coding	SLAIN motif	4	48341529	48426201
ENSG0000019867	-0.045166204	0.4	0.56	TTC37	protein_coding	tetratricope	5	95461755	95554977
ENSG0000023801	0.44534188	0.4	0.56	AC093110.1	lncRNA	novel transc	2	54661011	54680045
ENSG0000027489	0.832185236	0.4	0.56	AC012314.6	protein_coding	pre-mRNA	CHR_HSCHR	54115536	54131896
ENSG0000001886	-0.359939164	0.401	0.56	ZNF582	protein_coding	zinc finger p	19	56375846	56393545
ENSG0000017853	-0.13989222	0.401	0.56	CTXN1	protein_coding	cortexin 1 [S	19	7924491	7926135
ENSG0000023412	-0.524829893	0.401	0.56	AC073529.1	lncRNA	novel transc	X	10847578	11111220
ENSG0000024034	-0.042552879	0.401	0.56	RPS2P5	processed_pse	ribosomal p	12	118246084	118246962
ENSG0000027217	-0.365990045	0.401	0.56	U47924.2	lncRNA	novel transc	12	6943508	6944604
ENSG0000023267	0.659893351	0.402	0.56	ZNF687-AS	lncRNA	ZNF687 ant	1	151279678	151281950
ENSG0000017142	-0.494555788	0.402	0.56	NAT1	protein_coding	N-acetyltrar	8	18170477	18223689
ENSG0000015483	0.835238137	0.402	0.56	SKA1	protein_coding	spindle and	18	50375040	50394168
ENSG0000027983	-0.578889715	0.402	0.56	AL356273.3	TEC	TEC	1	185292384	185294372
ENSG0000026317	0.895688601	0.402	0.56	MTND1P8	processed_pse	MT-ND1 ps	16	3369957	3370906

ENSG0000015855	-0.227991287	0.402	0.56	GDPD5	protein_codinç	glycerophos	11	75434640	75525941
ENSG0000027359	-0.317275038	0.402	0.56	MBOAT7	protein_codinç	membrane	CHR_HSCHR	54173824	54190479
ENSG0000012555	-0.363652489	0.402	0.56	PLGLB2	protein_codinç	plasminoge	2	87748087	87759476
ENSG0000007333	-0.514335234	0.402	0.56	ALPK1	protein_codinç	alpha kinase	4	112285509	112442621
ENSG0000027015	-0.462845301	0.402	0.56	AC004918.3	lncRNA	novel transc	7	141662922	141663846
ENSG0000024759	-0.913127016	0.402	0.56	SPTY2D1OS	protein_codinç	SPTY2D1 op	11	18588781	18610255
ENSG0000016573	0.255724222	0.402	0.56	STOX1	protein_codinç	storkhead b	10	68827531	68895432
ENSG0000024040	1.75133347	0.402	0.56	MTATP8P1	unprocessed_p	MT-ATP8 p	1	633535	633741
ENSG0000018901	-0.419563595	0.402	0.56	SHLD2P3	unprocessed_p	shieldin con	10	47689707	47730436
ENSG0000027982	0.296857802	0.403	0.56	AC145098.2	TEC	TEC	5	177476631	177479656
ENSG0000013155	0.04817732	0.403	0.56	EXOC4	protein_codinç	exocyst com	7	133253073	134066589
ENSG0000027003	-0.714907758	0.403	0.56	AC025165.5	lncRNA	novel transc	12	57803838	57804415
ENSG0000000544	-0.11395856	0.403	0.56	WDR54	protein_codinç	WD repeat	2	74421678	74425755
ENSG0000024585	-0.675577833	0.403	0.56	GS1-24F4.2	lncRNA	uncharacter	8	6835535	6885276
ENSG0000019756	0.259514926	0.403	0.56	ZNF624	protein_codinç	zinc finger p	17	16620734	16653856
ENSG0000020673	2.008233771	0.404	0.56	AC004461.1	misc_RNA		22	19045256	19045367
ENSG0000024176	0.432987053	0.404	0.56	LINC00893	lncRNA	long interge	X	149527591	149540959
ENSG0000027496	0.261546863	0.404	0.56	AC026356.1	lncRNA	novel transc	12	32339368	32340724
ENSG0000025094	-1.408341964	0.404	0.56	AC091180.4	lncRNA	novel transc	17	49375380	49380094
ENSG0000011666	-0.181964936	0.404	0.56	SWT1	protein_codinç	SWT1 RNA	1	185157080	185291781
ENSG0000018245	-0.299530063	0.405	0.57	TEX19	protein_codinç	testis expres	17	82359247	82363775
ENSG0000025053	0.710037066	0.405	0.57	KRT8P33	processed_pse	keratin 8 ps	5	123400922	123402344
ENSG0000021910	-0.729252346	0.405	0.57	HNRNPA3P	processed_pse	heterogene	1	53974969	53976031
ENSG0000016915	0.082418815	0.405	0.57	ZBTB43	protein_codinç	zinc finger a	9	126805006	126838210
ENSG0000012243	0.578798267	0.405	0.57	SPATA1	protein_codinç	spermatoge	1	84506300	84566194
ENSG0000018649	-0.706307415	0.405	0.57	ZNF396	protein_codinç	zinc finger p	18	35366694	35377337
ENSG0000027511	1.08901927	0.405	0.57	AC012314.8	protein_codinç	pre-mRNA	CHR_HSCHR	54115536	54131896
ENSG0000026171	-1.117044838	0.405	0.57	AC009163.5	protein_codinç	novel TMEM	16	75412684	75464706
ENSG0000022030	-0.830125727	0.405	0.57	HNRNPH1P	processed_pse	heterogene	6	159712801	159713985
ENSG0000015835	0.314755765	0.406	0.57	SHROOM4	protein_codinç	shroom fam	X	50591647	50814302
ENSG0000014833	0.083718287	0.406	0.57	SLC25A25	protein_codinç	solute carrie	9	128068201	128109245
ENSG0000022597	0.242496401	0.406	0.57	PIGBOS1	protein_codinç	PIGB oppos	15	55317184	55319161
ENSG0000025330	0.480689349	0.406	0.57	PCDHGB6	protein_codinç	protocadhe	5	141408021	141512979
ENSG0000007469	-0.055182141	0.406	0.57	LMAN1	protein_codinç	lectin, manr	18	59327823	59359265
ENSG0000023617	0.247440232	0.406	0.57	VARS2	protein_codinç	valyl-tRNA	CHR_HSCHR	30904070	30916176
ENSG0000027024	-0.426198235	0.406	0.57	NA	NA	NA	NA	NA	NA
ENSG0000014347	0.129227521	0.406	0.57	DYRK3	protein_codinç	dual specific	1	206635536	206684419
ENSG0000017510	0.089156733	0.406	0.57	ZNF654	protein_codinç	zinc finger p	3	88059255	88144664
ENSG0000025608	-0.190401498	0.406	0.57	ZNF432	protein_codinç	zinc finger p	19	52031378	52095738
ENSG0000027705	1.224772905	0.406	0.57	AL122125.1	lncRNA	novel transc	14	51637348	51637947
ENSG0000025636	1.034827988	0.406	0.57	NA	NA	NA	NA	NA	NA
ENSG0000010326	0.088464935	0.407	0.57	STUB1	protein_codinç	STIP1 homo	16	680224	682870
ENSG0000025644	1.627441245	0.407	0.57	AP000763.3	lncRNA	novel transc	11	73405297	73410682
ENSG0000016790	0.087269619	0.407	0.57	TMEM68	protein_codinç	transmembr	8	55696424	55773407
ENSG0000012595	1.531619225	0.407	0.57	CHURC1-FI	protein_codinç	CHURC1-FN	14	64914485	65061803
ENSG0000027968	-0.184325655	0.407	0.57	AC022400.7	TEC	TEC	10	73769264	73772862
ENSG0000011299	-0.050584007	0.407	0.57	NNT	protein_codinç	nicotinamid	5	43602692	43707405
ENSG0000025498	-1.293068238	0.407	0.57	NA	NA	NA	NA	NA	NA
ENSG0000026049	-1.266619108	0.407	0.57	MYL12BP1	processed_pse	myosin light	15	42571927	42572433
ENSG0000026836	-1.389029712	0.407	0.57	AC010271.1	lncRNA	novel transc	19	40443436	40444087
ENSG0000023437	-0.976635381	0.407	0.57	SNX18P7	processed_pse	sorting nexi	9	33576380	33577014
ENSG0000021579	-0.176477746	0.407	0.57	SLC35E2A	protein_codinç	solute carrie	1	1724838	1745999
ENSG0000023955	-0.606364529	0.408	0.57	AC092045.1	processed_pse	protein pho	3	52373652	52374882
ENSG0000018295	0.314036358	0.408	0.57	SPATA13	protein_codinç	spermatoge	13	23979805	24307074
ENSG0000027202	1.066963099	0.408	0.57	AC064807.4	lncRNA	novel transc	8	51950284	51950690
ENSG0000016639	-0.5202619	0.408	0.57	CYB5R2	protein_codinç	cytochrome	11	7665100	7677222
ENSG0000025708	-0.452144963	0.408	0.57	AP001453.3	lncRNA	novel transc	11	64246939	64249494
ENSG0000013794	0.067502347	0.408	0.57	TTLL7	protein_codinç	tubulin tyro	1	83865024	83999150

ENSG0000024521	0.698376471	0.408	0.57	AC105285.1	lncRNA	novel transc	4	173094868	173169652
ENSG0000027461	-0.251754339	0.408	0.57	CNOT3	protein_coding	CCR4-NOT	CHR_HSCHR	54138182	54156191
ENSG0000016331	0.118727428	0.409	0.57	HELQ	protein_coding	helicase, PC	4	83407343	83455855
ENSG0000018763	0.190926581	0.409	0.57	DHRS4L2	protein_coding	dehydroger	14	23969874	24006408
ENSG0000021483	0.679439363	0.409	0.57	LINC01347	transcribed_un	long interge	1	243056307	243101744
ENSG0000025313	1.300526952	0.41	0.57	AC009630.1	lncRNA	novel transc	8	41509593	41578421
ENSG0000014331	0.062112043	0.41	0.57	ISG20L2	protein_coding	interferon st	1	156721891	156728766
ENSG0000027801	-0.174720137	0.41	0.57	AC245690.1	unprocessed_p	glucuronida	CHR_HSCHR	69910279	69916203
ENSG0000016532	0.090400029	0.41	0.57	ARHGAP12	protein_coding	Rho GTPase	10	31805398	31928876
ENSG0000025459	-0.422698298	0.41	0.57	AC084337.1	processed_pse	voltage-dep	11	6488186	6489377
ENSG0000012975	0.327899372	0.41	0.57	CDKN1C	protein_coding	cyclin deper	11	2883213	2885775
ENSG0000017934	-0.041830462	0.41	0.57	GATA2	protein_coding	GATA bindi	3	128479427	128493201
ENSG0000022409	-1.352232445	0.411	0.57	BCAR3-AS1	lncRNA	BCAR3 antis	1	93592199	93605573
ENSG0000027521	-0.691584355	0.411	0.57	CYP2D6	protein_coding	cytochrome	CHR_HSCHR	42126495	42130902
ENSG0000026016	0.748872097	0.411	0.57	AC093249.2	lncRNA	novel transc	16	30585907	30608593
ENSG0000024475	1.169203678	0.411	0.57	AL137024.1	lncRNA	novel transc	9	116288618	116318689
ENSG0000027126	1.022009709	0.411	0.57	AL353807.4	processed_pse	ATP synthas	1	155566050	155566306
ENSG0000023150	0.576480308	0.411	0.57	PTMAP4	processed_pse	prothymosi	12	9239986	9240331
ENSG0000012313	-0.101689636	0.411	0.57	ACOT9	protein_coding	acyl-CoA th	X	23701055	23766475
ENSG0000020420	-0.129776742	0.412	0.57	DAXX	protein_coding	death doma	6	33318558	33323016
ENSG0000027228	0.597183796	0.412	0.57	AL451165.2	lncRNA	novel transc	6	34696317	34697470
ENSG0000027457	1.296531774	0.412	0.57	AC006249.1	lncRNA	novel transc	18	28146233	28146703
ENSG0000014682	0.110469087	0.413	0.57	SLC12A9	protein_coding	solute carrie	7	100826820	100867010
ENSG0000025646	-0.843174738	0.413	0.57	YWHABP2	processed_pse	tyrosine 3-r	11	18490243	18490955
ENSG0000027532	-0.396087901	0.413	0.57	PDCD6IPP1	transcribed_un	PDCD6IP ps	15	22727104	22744918
ENSG0000014642	0.09334892	0.413	0.57	TIAM2	protein_coding	TIAM Rac1 a	6	154832697	155257723
ENSG0000022823	0.269074255	0.413	0.57	GAPDHP1	processed_pse	glyceraldeh	X	39787132	39788136
ENSG0000018140	-0.196752592	0.413	0.57	WASHC1	protein_coding	WASH comp	9	14475	73865
ENSG0000026021	0.877868281	0.414	0.57	AC092718.2	lncRNA	novel transc	16	81016792	81035759
ENSG0000027480	0.57713134	0.414	0.57	NA	NA	NA	NA	NA	NA
ENSG0000025795	0.284513219	0.414	0.57	P2RX5-TAX	protein_coding	P2RX5-TAX	17	3663063	3696194
ENSG0000027293	-0.664989595	0.414	0.57	AC099568.2	lncRNA	novel transc	1	89820174	89820868
ENSG0000012417	0.050392219	0.414	0.57	CHD6	protein_coding	chromodom	20	41402083	41618384
ENSG0000026742	-0.710825748	0.414	0.57	AC020934.1	lncRNA	novel transc	19	12825711	12832983
ENSG0000027280	-0.574897284	0.414	0.58	AC007038.1	lncRNA	novel transc	2	210028417	210029156
ENSG0000027797	-0.405854171	0.414	0.58	TBC1D3H	protein_coding	TBC1 doma	CHR_HSCHR	36377903	36388795
ENSG0000022439	-0.993815327	0.414	0.58	CSNK2B	protein_coding	casein kinas	CHR_HSCHR	31741515	31749825
ENSG0000010567	-0.072370365	0.415	0.58	DDX49	protein_coding	DEAD-box I	19	18919705	18929189
ENSG0000022368	-0.466961539	0.415	0.58	DDR1	protein_coding	discoidin dc	CHR_HSCHR	30959531	30970646
ENSG0000011687	0.056740554	0.415	0.58	MAP7D1	protein_coding	MAP7 doma	1	36155579	36180849
ENSG0000023444	-0.349422856	0.415	0.58	NA	NA	NA	NA	NA	NA
ENSG0000024698	0.600865901	0.415	0.58	Z84485.1	lncRNA	novel transc	6	36146698	36197205
ENSG0000027055	1.02184059	0.415	0.58	AC013731.1	lncRNA	novel transc	2	157877683	157878565
ENSG0000012741	-0.652978175	0.415	0.58	IDUA	protein_coding	alpha-L-idu	4	986997	1004564
ENSG0000011786	0.072991423	0.416	0.58	TXNDC12	protein_coding	thioredoxin	1	52020131	52055191
ENSG0000010793	-0.049985499	0.416	0.58	GTPBP4	protein_coding	GTP binding	10	988434	1019932
ENSG0000011859	-0.84901769	0.416	0.58	SLC16A7	protein_coding	solute carrie	12	59596029	59789855
ENSG0000011478	0.08595577	0.416	0.58	EIF1B	protein_coding	eukaryotic t	3	40309707	40312424
ENSG0000013876	-0.049090613	0.416	0.58	USO1	protein_coding	USO1 vesicl	4	75724577	75814286
ENSG0000012584	-0.146142048	0.416	0.58	ZNF133	protein_coding	zinc finger p	20	18288283	18316996
ENSG0000010802	0.040612927	0.417	0.58	TASOR2	protein_coding	transcriptior	10	5684838	5763740
ENSG0000018513	1.044609176	0.417	0.58	H2BC13	protein_coding	H2B cluster	6	27807444	27807931
ENSG0000022383	-1.04743031	0.417	0.58	AL645941.1	lncRNA	novel transc	6	32970232	32970886
ENSG0000010189	-0.215077745	0.418	0.58	MCTS2P	protein_coding	MCTS family	20	31547412	31548081
ENSG0000027818	-0.079584557	0.418	0.58	ABCC1	protein_coding	ATP binding	CHR_HSCHR	16034690	16228109
ENSG0000020096	1.132824744	0.418	0.58	RN7SKP87	misc_RNA	RN7SK pseu	9	100581079	100581379
ENSG0000023749	-0.294949722	0.418	0.58	AC034102.1	processed_pse	RAB13 mem	12	55980432	55981035
ENSG0000014421	-0.473491698	0.418	0.58	LYG1	protein_coding	lysozyme g1	2	99284238	99304742

ENSG0000016467	0.18821214	0.418	0.58	SYTL3	protein_coding	synaptotagr	6	158650014	158764876
ENSG0000013451	-0.457487885	0.418	0.58	DOCK2	protein_coding	dedicator of	5	169637268	170083382
ENSG0000018119	-0.059507483	0.418	0.58	DHTKD1	protein_coding	dehydroger	10	12068954	12123221
ENSG0000010144	-0.152363629	0.418	0.58	ACTR5	protein_coding	actin relatec	20	38748460	38772520
ENSG0000020475	0.868865161	0.418	0.58	AC008429.1	lncRNA	novel transc	5	172954786	172959392
ENSG0000020595	0.611337284	0.419	0.58	AC105345.1	lncRNA	novel transc	4	8482270	8516759
ENSG0000024879	-1.373672715	0.419	0.58	AC026436.1	processed_pse	ribosomal p	5	81242330	81242599
ENSG0000028020	0.310367512	0.419	0.58	AC026401.3	lncRNA	novel transc	16	15701237	15702118
ENSG0000024382	-0.508530566	0.419	0.58	AC011495.1	processed_pse	ribosomal p	19	49719498	49720081
ENSG0000020628	-0.274102314	0.419	0.58	ZBTB22	protein_coding	zinc finger a	CHR_HSCHR	33243252	33246776
ENSG0000027881	-1.359431945	0.419	0.58	C8orf31	lncRNA	chromosom	CHR_HSCHR	143039209	143059942
ENSG0000022612	-0.121983709	0.419	0.58	AHCTF1P1	processed_pse	AT-hook co	2	161500885	161507705
ENSG0000025626	0.090048907	0.419	0.58	HMBS	protein_coding	hydroxymet	11	119084866	119093549
ENSG0000027926	0.627576764	0.419	0.58	AC000123.2	TEC	TEC	7	127349833	127350293
ENSG0000016493	-0.066281568	0.42	0.58	SLC25A32	protein_coding	solute carrie	8	103398635	103415189
ENSG0000012461	-0.037364903	0.42	0.58	RPS10	protein_coding	ribosomal p	6	34417454	34426069
ENSG0000025443	0.616597352	0.42	0.58	AP001001.1	lncRNA	novel transc	11	106085990	106101975
ENSG0000017062	-0.158087329	0.42	0.58	DPY19L2P2	transcribed_un	DPY19L2 ps	7	103175133	103280410
ENSG0000022790	0.376341972	0.42	0.58	SNAP25-AS	lncRNA	SNAP25 ant	20	10006381	10368776
ENSG0000011069	-0.04285582	0.42	0.58	C11orf58	protein_coding	chromosom	11	16613132	16758340
ENSG0000023489	-0.193570021	0.421	0.58	HCG18	lncRNA	HLA comple	CHR_HSCHR	30280294	30316632
ENSG0000028021	0.392218472	0.421	0.58	AC106886.5	TEC	novel transc	16	30773532	30776033
ENSG0000012645	0.414734188	0.421	0.58	RRAS	protein_coding	RAS related	19	49635292	49640143
ENSG0000028029	0.36721529	0.421	0.58	NA	NA	NA	NA	NA	NA
ENSG0000008080	-0.073386702	0.421	0.58	CNOT4	protein_coding	CCR4-NOT	7	135361795	135510127
ENSG0000020627	-0.126678215	0.422	0.58	DAXX	protein_coding	death doma	CHR_HSCHR	33247394	33258105
ENSG0000023503	0.798949826	0.422	0.58	AL035456.1	processed_pse	pseudogene	20	10612861	10614229
ENSG0000015719	-0.081804017	0.422	0.58	LRP8	protein_coding	LDL recepto	1	53242364	53328469
ENSG0000025942	0.719806209	0.423	0.58	AC011767.1	transcribed_un	Golgin subfa	15	22770523	22776199
ENSG0000009149	-0.094237383	0.423	0.59	SEL1L3	protein_coding	SEL1L family	4	25747433	25863760
ENSG0000026917	0.400837672	0.423	0.59	AP001160.3	lncRNA	novel transc	11	62786023	62786785
ENSG0000020219	-0.361137772	0.424	0.59	AL162581.1	misc_RNA		6	52995620	52995950
ENSG0000013741	0.233677156	0.424	0.59	VARS2	protein_coding	valyl-tRNA s	6	30914205	30926459
ENSG0000013621	0.13778767	0.424	0.59	CHST12	protein_coding	carbohydrat	7	2403588	2448484
ENSG0000013574	-0.478133264	0.424	0.59	ZNF670-ZN	protein_coding	ZNF670-ZN	1	246945547	247078811
ENSG0000015976	-0.383453184	0.424	0.59	C16orf86	protein_coding	chromosom	16	67666814	67668757
ENSG0000011679	-0.109777576	0.424	0.59	CRYZ	protein_coding	crystallin ze	1	74705482	74733408
ENSG0000014217	0.332502888	0.425	0.59	SIK1	protein_coding	salt inducibl	21	43414483	43427131
ENSG0000016380	-0.44676948	0.425	0.59	SPDYA	protein_coding	speedy/RIN	2	28782517	28850611
ENSG0000007585	-0.0537352	0.425	0.59	SART3	protein_coding	spliceosome	12	108522214	108561400
ENSG0000013699	0.503715261	0.425	0.59	CCN3	protein_coding	cellular com	8	119416446	119424434
ENSG0000027113	-0.55768503	0.425	0.59	AC004130.2	lncRNA	novel transc	7	20328299	20331747
ENSG0000022628	0.757812695	0.426	0.59	ARPC3P1	processed_pse	actin relatec	20	49134480	49135012
ENSG0000023941	0.859361171	0.425	0.59	RN7SL535P	misc_RNA	RNA, 7SL, cy	7	143290615	143290910
ENSG0000025376	0.935953784	0.426	0.59	AC019257.1	lncRNA	novel transc	8	1971153	1974637
ENSG0000025584	0.817246338	0.426	0.59	AP000593.3	lncRNA	novel transc	11	72302139	72302965
ENSG0000026148	-0.936007733	0.426	0.59	AC135048.1	lncRNA	novel transc	16	30948386	30956511
ENSG0000023338	-0.484048762	0.426	0.59	NKAPP1	transcribed_pr	NFKB activa	X	120120953	120245267
ENSG0000008906	0.351076855	0.426	0.59	SLC8B1	protein_coding	solute carrie	12	113298759	113359493
ENSG0000011599	-0.141099151	0.426	0.59	C2orf42	protein_coding	chromosom	2	70149880	70248615
ENSG0000013432	0.512734645	0.426	0.59	RSAD2	protein_coding	radical S-ac	2	6865806	6898239
ENSG0000027264	0.213126038	0.426	0.59	GTF2IP20	transcribed_un	general trar	1	223951394	223992594
ENSG0000025667	-0.681995984	0.426	0.59	AC141557.1	unprocessed_p	ovostatin (C	12	9398355	9414851
ENSG0000011051	-0.053163743	0.426	0.59	MADD	protein_coding	MAP kinase	11	47269161	47330031
ENSG0000014315	-0.087089071	0.426	0.59	MPC2	protein_coding	mitochondr	1	167916675	167937072
ENSG0000026273	0.474092398	0.426	0.59	AC217785.2	unprocessed_p	glucosidase	CHR_HSCHR	155211179	155213671
ENSG0000026995	-0.655937445	0.427	0.59	AC022239.1	lncRNA	novel transc	8	11552488	11564694
ENSG0000026214	-1.072612058	0.427	0.59	AC124283.1	lncRNA	novel transc	17	82587313	82588411

ENSG0000027302	0.700383236	0.427	0.59	AL358472.3	lncRNA	novel transc		1	153966516	153966930
ENSG0000011681	0.570048805	0.427	0.59	TFAP2E	protein_coding	transcription		1	35573314	35595328
ENSG0000013214	-0.297447297	0.427	0.59	CCT6B	protein_coding	chaperonin		17	34927859	34981078
ENSG0000027372	-0.396352304	0.427	0.59	AC007686.3	lncRNA	novel transc		14	77028086	77031572
ENSG0000024884	1.146026573	0.427	0.59	AL645949.1	transcribed_pr	X antigen fa		4	3312512	3313058
ENSG0000016407	0.119684982	0.427	0.59	MFSD8	protein_coding	major facilit		4	127917799	127966034
ENSG0000021267	0.431426639	0.427	0.59	NA	NA	NA	NA	NA	NA	
ENSG0000027764	0.373019312	0.427	0.59	NA	NA	NA	NA	NA	NA	
ENSG0000021586	0.24609027	0.428	0.59	AC245297.1	transcribed_un	phosphodie		1	149197992	149321732
ENSG0000023991	1.933155729	0.428	0.59	RPL39P36	processed_pse	ribosomal p		19	53413190	53413345
ENSG0000019686	0.057769386	0.428	0.59	NHLRC2	protein_coding	NHL repeat		10	113854661	113917194
ENSG0000021266	-1.193583062	0.428	0.59	AC064799.1	processed_pse	ribosomal p		15	71341158	71341712
ENSG0000025442	0.388922463	0.428	0.59	AP003392.1	lncRNA	novel transc		11	119003742	119004893
ENSG0000013179	-0.066011411	0.428	0.59	PRKAB2	protein_coding	protein kina		1	147155106	147172550
ENSG0000013808	-0.146374798	0.428	0.59	SIX3	protein_coding	SIX homeob		2	44941702	44946071
ENSG0000026222	0.503795687	0.428	0.59	NA	NA	NA	NA	NA	NA	
ENSG0000017842	-0.944762845	0.429	0.59	RPS3AP5	processed_pse	RPS3A pseu		10	84560443	84561263
ENSG0000016738	0.139153171	0.429	0.59	ZNF226	protein_coding	zinc finger p		19	44165073	44178381
ENSG0000019975	0.63262393	0.429	0.59	SNORD104	snoRNA	small nucleo		17	64146083	64146152
ENSG0000016792	-0.168867645	0.429	0.59	TMEM99	lncRNA	transmembr		17	40819106	40836270
ENSG0000025908	-0.687105103	0.429	0.59	KIAA1143P	processed_pse	KIAA1143 p		14	32271857	32272301
ENSG0000023591	0.698127586	0.429	0.59	AL031729.1	processed_pse	novel pseud		1	27649419	27649610
ENSG0000010561	0.644505476	0.429	0.59	PRPF31	protein_coding	pre-mRNA		19	54115410	54131719
ENSG0000011220	0.053115065	0.429	0.59	ZNF451	protein_coding	zinc finger p		6	57086844	57170305
ENSG0000014835	-0.110203113	0.429	0.59	LRSAM1	protein_coding	leucine rich		9	127451486	127503501
ENSG0000019710	-0.386824316	0.429	0.59	SLC6A17	protein_coding	solute carrie		1	110150494	110202202
ENSG0000025497	0.815722495	0.43	0.59	ALG1L9P	transcribed_un	ALG1 like 9,		11	71800541	71804640
ENSG0000026726	-0.988216687	0.43	0.59	AC006504.3	processed_pse	vomeronasa		19	27803538	27804060
ENSG0000026716	0.60960187	0.43	0.59	NA	NA	NA	NA	NA	NA	
ENSG0000007913	0.085496484	0.431	0.59	THOC1	protein_coding	THO comple		18	214520	268050
ENSG0000016429	0.111826213	0.431	0.59	ARSK	protein_coding	arylsulfatase		5	95555101	95605102
ENSG0000027365	0.663210078	0.431	0.59	AC020904.2	transcribed_pr	citrate synth		19	18022403	18032099
ENSG0000022911	-0.169942332	0.431	0.59	AC026403.1	processed_pse	60S acidic ri		5	166382305	166382599
ENSG0000017983	-0.212671949	0.431	0.59	MROH1	protein_coding	maestro hea		8	144148016	144261940
ENSG0000021253	1.620036552	0.431	0.59	SNORA75	snoRNA	Small nucleo		12	31076867	31077016
ENSG0000023362	-1.135845832	0.431	0.59	AC092814.2	processed_pse	protein (pep		1	211936249	211936634
ENSG0000013058	0.35031831	0.431	0.59	ZBTB46	protein_coding	zinc finger a		20	63743668	63832038
ENSG0000023112	-0.338438122	0.432	0.59	ABCF1	protein_coding	ATP binding	CHR_HSCHR		30616012	30641816
ENSG0000010756	0.067506683	0.432	0.59	ERLIN1	protein_coding	ER lipid raft		10	100150094	100186033
ENSG0000014711	0.592060287	0.432	0.59	CHST7	protein_coding	carbohydrat	X		46573765	46598496
ENSG0000021390	-0.358514535	0.432	0.59	SLC23A3	protein_coding	solute carrie		2	219161465	219170095
ENSG0000013393	-0.397466042	0.432	0.59	GSC	protein_coding	goosecoid h		14	94768223	94770113
ENSG0000026903	-1.049947772	0.432	0.59	AP001462.1	lncRNA	novel transc		11	64778954	64779405
ENSG0000016708	0.069011558	0.432	0.59	SNRPD1	protein_coding	small nuclea		18	21612314	21633524
ENSG0000020009	0.615315733	0.432	0.59	Y_RNA	misc_RNA	Y RNA [Sou		11	47726894	47726992
ENSG0000009067	-0.094210442	0.433	0.59	MCOLN1	protein_coding	mucolipin 1		19	7522624	7534009
ENSG0000012675	-0.103522482	0.433	0.59	UXT	protein_coding	ubiquitously	X		47651796	47659180
ENSG0000017026	-0.107096058	0.433	0.59	ZNF212	protein_coding	zinc finger p		7	149239651	149255606
ENSG0000017402	-0.370960393	0.433	0.59	FAM3C2	processed_pse	family with s	X		23075758	23076767
ENSG0000008746	-0.027859242	0.433	0.59	GNAS	protein_coding	GNAS comp		20	58839718	58911192
ENSG0000025402	0.7232178	0.434	0.59	AC009902.2	lncRNA	novel transc		8	81279871	81281446
ENSG0000013512	-0.273487538	0.434	0.59	P2RX4	protein_coding	purinergic re		12	121209861	121234106
ENSG0000014442	0.105817783	0.434	0.59	NBEAL1	protein_coding	neurobeach		2	203014879	203226378
ENSG0000027915	0.820806474	0.434	0.59	AC233300.1	TEC	TEC	X		48939992	48940808
ENSG0000011346	0.064058302	0.434	0.59	BRIX1	protein_coding	biogenesis c		5	34915677	34925996
ENSG0000017063	0.062913014	0.434	0.59	RNF34	protein_coding	ring finger p		12	121400041	121430623
ENSG0000018779	0.156365279	0.434	0.59	ZNF70	protein_coding	zinc finger p		22	23738682	23751112
ENSG0000023352	0.498435912	0.434	0.59	ZNF529-AS	lncRNA	ZNF529 ant		19	36573070	36594708

ENSG0000022753	0.518629946	0.434	0.59	SLC2A1-AS	lncRNA	SLC2A1 ant	1	42959049	42996814
ENSG0000024085	0.800627407	0.434	0.59	PSMC2P1	processed_pse	PSMC2 pseu	3	132175402	132176711
ENSG0000025735	-0.202926082	0.434	0.59	AC048341.1	lncRNA	novel transc	12	62602752	62622213
ENSG0000026995	-3.589419047	0.434	0.59	FMC1-LUC	protein_coding	FMC1-LUC7	7	139341360	139422599
ENSG0000027889	0.785754723	0.434	0.59	AL358852.1	lncRNA	novel transc	6	149717621	149718888
ENSG0000010946	-0.125102849	0.434	0.59	KLHL2	protein_coding	kelch like fa	4	165207561	165323156
ENSG0000027906	0.459967601	0.435	0.6	AC244517.0	lncRNA	novel transc	5	141182409	141188582
ENSG0000025448	1.222965219	0.435	0.6	AC026191.1	lncRNA	novel transc	3	9292588	9363303
ENSG0000010118	0.087899466	0.435	0.6	MTG2	protein_coding	mitochondr	20	62183029	62203568
ENSG0000027298	0.947876808	0.435	0.6	AC009570.1	lncRNA	novel transc	4	70703747	70704491
ENSG0000027188	-1.021671529	0.435	0.6	AC016747.2	lncRNA	novel transc	2	61151433	61162105
ENSG0000012687	0.093560506	0.435	0.6	WDR60	protein_coding	WD repeat	7	158856558	158956747
ENSG0000026768	0.945900634	0.435	0.6	AC016590.2	lncRNA	novel transc	19	37337236	37337743
ENSG0000027768	1.176273241	0.435	0.6	MIR6739	miRNA	microRNA 6	1	201863373	201863447
ENSG0000019738	0.130686047	0.435	0.6	DACT3	protein_coding	dishevelled	19	46647551	46661182
ENSG0000002811	-0.2004123	0.435	0.6	VRK2	protein_coding	VRK serine/t	2	57907629	58159920
ENSG0000023458	-0.193685947	0.436	0.6	CCT6P3	transcribed_un	chaperonin	7	65038354	65074713
ENSG0000027226	-0.750842768	0.436	0.6	AC021242.3	lncRNA	novel transc	8	9555144	9556520
ENSG0000015576	0.078489445	0.436	0.6	FZD7	protein_coding	frizzled clas	2	202034587	202038445
ENSG0000026275	0.151058105	0.436	0.6	NA	NA	NA	NA	NA	NA
ENSG0000011758	0.265486821	0.436	0.6	TNFSF4	protein_coding	TNF superfa	1	173183731	173207331
ENSG0000015539	0.089740653	0.436	0.6	HEATR3	protein_coding	HEAT repea	16	50065970	50107272
ENSG0000023290	0.668595277	0.436	0.6	AC092685.1	processed_pse	novel pseuc	7	65355934	65357176
ENSG0000026067	1.560565129	0.436	0.6	AC093249.3	transcribed_pr	zinc finger p	16	30615256	30615679
ENSG0000023061	-0.781502694	0.437	0.6	HM13-AS1	lncRNA	HM13 antis	20	31567707	31573263
ENSG0000018467	0.06564058	0.437	0.6	ZBTB40	protein_coding	zinc finger a	1	22428838	22531157
ENSG0000026177	0.456890922	0.437	0.6	AC012184.3	lncRNA	novel transc	16	70315442	70346806
ENSG0000008707	-0.440407392	0.438	0.6	TRIP6	protein_coding	thyroid horr	7	100867387	100873454
ENSG0000011319	0.051818833	0.438	0.6	HAND1	protein_coding	heart and n	5	154474972	154478227
ENSG0000012078	-0.103399859	0.437	0.6	ZFP30	protein_coding	ZFP30 zinc f	19	37613749	37692337
ENSG0000012798	0.143574375	0.438	0.6	MTERF1	protein_coding	mitochondr	7	91692008	91880702
ENSG0000027956	0.616040363	0.438	0.6	AC093525.9	TEC	tec	16	2575628	2577373
ENSG0000013490	0.068358876	0.438	0.6	POGLUT2	protein_coding	protein O-g	13	102784281	102798976
ENSG0000021244	-1.234253213	0.438	0.6	SNORA53	snoRNA	small nuclec	12	98599635	98599883
ENSG0000026724	-0.609902858	0.438	0.6	NA	NA	NA	NA	NA	NA
ENSG0000018688	-0.218076609	0.438	0.6	TMEM17	protein_coding	transmembr	2	62500218	62511894
ENSG0000026380	1.96420599	0.438	0.6	AC135178.3	protein_coding	novel protei	17	8368638	8383187
ENSG0000010988	-0.116822693	0.438	0.6	CCDC34	protein_coding	coiled-coil	11	27330827	27363234
ENSG0000022712	-0.213627581	0.439	0.6	ZNF717	protein_coding	zinc finger p	3	75678660	75785583
ENSG0000001356	-0.068777767	0.439	0.6	RNF14	protein_coding	ring finger p	5	141958328	141990292
ENSG0000027291	0.412672886	0.439	0.6	AC005070.3	lncRNA	novel transc	7	105102838	105105483
ENSG0000024319	-0.174559475	0.439	0.6	AC115223.1	processed_pse	ribosomal p	4	65573459	65574328
ENSG0000018602	-0.103549999	0.439	0.6	ZNF529	protein_coding	zinc finger p	19	36534774	36605276
ENSG0000019888	-0.141876194	0.44	0.6	ITPRIPL1	protein_coding	ITPRIP like 1	2	96325331	96330517
ENSG0000013772	-0.119754672	0.44	0.6	C11orf1	protein_coding	chromosom	11	111878935	111885975
ENSG0000012357	-0.069925211	0.44	0.6	FAM199X	protein_coding	family with s	X	104166453	104195902
ENSG0000018109	-0.682073938	0.44	0.6	LINC02878	lncRNA	long interge	8	143696154	143698413
ENSG0000012414	0.601812972	0.44	0.6	SLC12A5	protein_coding	solute carrie	20	46021690	46060150
ENSG0000019670	0.070301841	0.44	0.6	ZNF431	protein_coding	zinc finger p	19	21142024	21196053
ENSG0000025881	0.347945591	0.44	0.6	LINC02820	lncRNA	long interge	12	85318060	85342912
ENSG0000022979	1.588032893	0.44	0.6	RPS21P1	transcribed_pr	ribosomal p	1	235432985	235433231
ENSG0000024798	-0.493929525	0.44	0.6	LINC00926	lncRNA	long interge	15	57300365	57307769
ENSG0000022993	0.810388495	0.441	0.6	ATXN1-AS	lncRNA	ATXN1 antis	6	16761138	16762652
ENSG0000026324	-1.301000711	0.441	0.6	AC087190.3	lncRNA	novel transc	16	9104848	9113181
ENSG0000027134	0.446591672	0.441	0.6	NA	NA	NA	NA	NA	NA
ENSG0000021367	-0.127555393	0.441	0.6	ATF6B	protein_coding	activating tr	6	32115264	32128253
ENSG0000014187	0.086060035	0.441	0.6	SLC39A3	protein_coding	solute carrie	19	2732204	2740028
ENSG0000014818	0.199188949	0.441	0.6	GSN	protein_coding	gelsolin [So	9	121207794	121332843

ENSG0000027666	0.397739514	0.441	0.6	NA	NA	NA	NA	NA	NA	
ENSG0000022845	0.394073966	0.441	0.6	AC098484.1	lncRNA	novel transc		1	42775813	42776790
ENSG0000017960	0.186623941	0.441	0.6	GRM8	protein_coding	glutamate m		7	126438598	127253093
ENSG0000022883	1.321753243	0.441	0.6	AL445189.2	processed_pse	ATP synthas		6	108907615	108907873
ENSG0000019987	1.823472365	0.441	0.6	Y_RNA	misc_RNA	Y RNA [Sou		11	93719603	93719715
ENSG0000023500	0.561509137	0.441	0.6	EIF4A1P2	processed_pse	eukaryotic t	Y		5337745	5338964
ENSG0000025274	1.823472365	0.441	0.6	RNU6-850P	snRNA	RNA, U6 sm		6	31756951	31757053
ENSG0000026455	1.823472365	0.441	0.6	U6	snRNA	U6 spliceos	CHR_HSCHR		31747180	31747282
ENSG0000026458	0.943306981	0.441	0.6	MAPT-AS1	lncRNA	MAPT antise		17	45799390	45895680
ENSG0000022638	0.370801071	0.442	0.6	LINC01876	lncRNA	long interge		2	156011530	156254950
ENSG0000026176	0.997368044	0.442	0.6	AC133550.3	lncRNA	novel transc		16	28862166	28863340
ENSG0000014337	-0.081996128	0.442	0.6	ZNF687	protein_coding	zinc finger p		1	151281618	151292176
ENSG0000006468	0.157464623	0.442	0.6	ABCA7	protein_coding	ATP binding		19	1039997	1065572
ENSG0000022509	1.101292837	0.442	0.6	SNORA71A	snoRNA	small nucleo		20	38427309	38427442
ENSG0000010571	-0.511249083	0.442	0.6	PBX4	protein_coding	PBX homeo		19	19561707	19618693
ENSG0000019617	0.105447444	0.442	0.6	ACADSB	protein_coding	acyl-CoA de		10	123008979	123058290
ENSG0000023329	-0.524103924	0.442	0.6	RASA4DP	unprocessed_p	RAS p21 pro		7	102681836	102690469
ENSG0000017273	-0.152807061	0.442	0.6	PURG	protein_coding	purine rich e		8	30995802	31033715
ENSG0000018037	-0.136060305	0.443	0.6	CCDC66	protein_coding	coiled-coil c		3	56557161	56621837
ENSG0000025864	-1.090327806	0.443	0.6	BCL2L2-PA	protein_coding	BCL2L2-PAI		14	23306835	23325369
ENSG0000008799	0.089594537	0.443	0.6	METTL2A	protein_coding	methyltrans		17	62423875	62453385
ENSG0000008882	0.23264264	0.443	0.6	SMOX	protein_coding	spermine ox		20	4120980	4187747
ENSG0000026178	-0.973867164	0.443	0.6	NA	NA	NA	NA	NA	NA	
ENSG0000025227	-1.820639398	0.443	0.6	AC080037.1	scaRNA			17	72818836	72818972
ENSG0000027838	0.900993316	0.443	0.6	AL031673.1	lncRNA	novel transc		20	25680781	25681246
ENSG0000016009	-0.397161966	0.443	0.6	FNDC5	protein_coding	fibronectin t		1	32862268	32872482
ENSG0000018032	-0.077758991	0.444	0.6	CCDC43	protein_coding	coiled-coil c		17	44673069	44689779
ENSG0000025961	1.091082721	0.443	0.6	NA	NA	NA	NA	NA	NA	
ENSG0000023086	-0.378019772	0.444	0.6	AGAP10P	unprocessed_p	ArfGAP with		10	45678692	45700532
ENSG0000021378	-0.407643382	0.444	0.6	GTF2H4	protein_coding	general tran		6	30908207	30914106
ENSG0000016798	0.094095089	0.444	0.6	SDHAF2	protein_coding	succinate de		11	61430042	61446733
ENSG0000018535	0.055482268	0.444	0.6	HGS	protein_coding	hepatocyte		17	81683326	81703138
ENSG0000020542	-0.275033511	0.444	0.6	KRT81	protein_coding	keratin 81 [S		12	52285913	52291534
ENSG0000017653	0.288088166	0.444	0.6	GNG7	protein_coding	G protein su		19	2511219	2702694
ENSG0000023361	1.226530759	0.444	0.6	DCUN1D2-	lncRNA	DCUN1D2 a		13	113468901	113476135
ENSG0000011172	-0.090011454	0.444	0.6	HCFC2	protein_coding	host cell fac		12	104064531	104106524
ENSG0000013311	-0.111726049	0.444	0.6	GPALPP1	protein_coding	GPALPP mo		13	44989529	45037669
ENSG0000014278	-0.084048985	0.444	0.6	WDTC1	protein_coding	WD and tetr		1	27234632	27308636
ENSG0000022754	-0.4845756	0.444	0.6	AC092614.1	lncRNA	novel transc		2	191229165	191246172
ENSG0000022925	-0.254237914	0.444	0.6	ZBTB22	protein_coding	zinc finger a	CHR_HSCHR		33236020	33239546
ENSG0000025381	-0.275790974	0.444	0.6	AC138866.1	unprocessed_p	glucuronida		5	70219918	70258930
ENSG0000022670	-0.614132532	0.445	0.6	AL161452.1	lncRNA	novel transc		9	135503273	135506447
ENSG0000026836	-0.54732685	0.445	0.6	SMC5-AS1	lncRNA	SMC5 antise		9	70193997	70258866
ENSG0000014345	0.229838729	0.445	0.6	OAZ3	protein_coding	ornithine de		1	151762899	151771334
ENSG0000005138	0.104038323	0.445	0.6	PIK3CB	protein_coding	phosphatidy		3	138652698	138834928
ENSG0000020645	0.275999897	0.445	0.6	CCHCR1	protein_coding	coiled-coil a	CHR_HSCHR		31134861	31150652
ENSG0000015096	0.174160856	0.446	0.61	ABCB9	protein_coding	ATP binding		12	122920951	122981649
ENSG0000024237	0.341030382	0.446	0.61	AL590705.3	lncRNA	novel transc		9	97195351	97197687
ENSG0000025098	-1.236929674	0.446	0.61	MARCHF11	lncRNA	MARCHF11		5	16129178	16141602
ENSG0000026857	0.128565788	0.446	0.61	AL031282.2	lncRNA	novel transc		1	1702736	1737688
ENSG0000016640	-0.276206087	0.446	0.61	SERPINB8	protein_coding	serpin family		18	63970029	64019779
ENSG0000013689	0.086154887	0.446	0.61	MRPL50	protein_coding	mitochondr		9	101387633	101398618
ENSG0000014705	-0.086175674	0.446	0.61	KDM6A	protein_coding	lysine deme	X		44873188	45112779
ENSG0000010517	-0.065456308	0.447	0.61	URI1	protein_coding	URI1 prefolc		19	29923644	30016612
ENSG0000008383	-0.216504918	0.447	0.61	ZNF446	protein_coding	zinc finger p		19	58474017	58481230
ENSG0000010212	-0.114770553	0.447	0.61	TAZ	protein_coding	tafazzin [So	X		154411524	154421726
ENSG0000012825	0.822914486	0.447	0.61	C22orf24	lncRNA	chromosom		22	31925519	31945518
ENSG0000027487	0.260552697	0.447	0.61	NA	NA	NA	NA	NA	NA	

ENSG0000019817	0.057774017	0.447	0.61	TFDP1	protein_coding	transcription		13	113584721	113641473
ENSG0000013137	0.078712825	0.447	0.61	CAPN7	protein_coding	calpain 7 [S		3	15206152	15252916
ENSG0000022580	0.898089867	0.447	0.61	DNAJC19P5	processed_pse	DnaJ heat sh		2	177229191	177229506
ENSG0000022805	-0.503511151	0.447	0.61	TOP3BP1	processed_pse	DNA topois		22	22223187	22224566
ENSG0000010938	0.069477555	0.448	0.61	ELF2	protein_coding	E74 like ETS		4	139028112	139177218
ENSG0000020366	0.12927348	0.448	0.61	EFCAB2	protein_coding	EF-hand cal		1	244969705	245127164
ENSG0000022820	-0.407813785	0.448	0.61	AC131235.1	processed_pse	ribosomal p		3	184149704	184150435
ENSG0000026331	-1.376329867	0.448	0.61	AC009127.2	unprocessed_p	coiled-coil c		16	72004239	72004803
ENSG0000027938	1.572352007	0.448	0.61	CR392039.2	processed_pse	ankyrin repe		21	9088188	9088391
ENSG0000022339	-0.825027209	0.448	0.61	AL118511.1	lncRNA	novel transc		1	230868259	230879141
ENSG0000027287	0.259104225	0.448	0.61	SAP30-DT	lncRNA	SAP30 diver		4	173349543	173370721
ENSG0000021939	0.759030889	0.448	0.61	HSPA8P15	processed_pse	heat shock p		6	151411259	151413945
ENSG0000026359	-1.557534246	0.449	0.61	MIR3936	miRNA	microRNA 3		5	132365490	132365599
ENSG0000018510	0.057542758	0.449	0.61	FAF1	protein_coding	Fas associat		1	50437028	50960267
ENSG0000024828	0.713434423	0.449	0.61	NA	NA	NA	NA	NA	NA	
ENSG0000013550	0.351613114	0.449	0.61	SLC26A10	protein_coding	solute carrie		12	57619527	57626151
ENSG0000011966	0.052538238	0.449	0.61	IRF2BPL	protein_coding	interferon re		14	77024543	77028708
ENSG0000014150	-0.781115931	0.449	0.61	ASGR1	protein_coding	asialoglycop		17	7173431	7179564
ENSG0000025774	-0.702266241	0.449	0.61	AC073896.3	lncRNA	novel transc		12	56308868	56309449
ENSG0000011816	-0.167604262	0.45	0.61	KPTN	protein_coding	kaptin, actin		19	47475150	47484265
ENSG0000016309	-0.186056878	0.45	0.61	BBS5	protein_coding	Bardet-Bied		2	169479480	169506655
ENSG0000025515	0.547031805	0.45	0.61	TOLLIP-AS1	lncRNA	TOLLIP antis		11	1309769	1310707
ENSG0000007213	-0.073058721	0.45	0.61	EPN2	protein_coding	epsin 2 [Sou		17	19215615	19336715
ENSG0000017639	0.118979992	0.45	0.61	CRLF3	protein_coding	cytokine rec		17	30769388	30824692
ENSG0000019638	-0.097483076	0.45	0.61	ZNF140	protein_coding	zinc finger p		12	133079838	133107544
ENSG0000026889	0.998582419	0.451	0.61	AC009955.3	lncRNA	novel transc		2	219547211	219547658
ENSG0000022778	-0.252981426	0.451	0.61	ZBTB22	protein_coding	zinc finger a	CHR_HSCHR		33292340	33295864
ENSG0000023124	-0.293037176	0.451	0.61	C1DP1	processed_pse	C1D nuclear		10	32511336	32511737
ENSG0000026150	0.505478388	0.451	0.61	AL031714.1	lncRNA	novel transc		16	1317891	1322845
ENSG0000023529	0.308894433	0.451	0.61	AL354733.3	lncRNA	novel transc		9	83972233	83975777
ENSG0000027142	1.173754024	0.451	0.61	AL358072.1	lncRNA	novel transc		1	117364899	117365473
ENSG0000009615	-0.104227972	0.452	0.61	BAG6	protein_coding	BAG cochap	CHR_HSCHR		31629248	31642931
ENSG0000026333	-0.758900341	0.452	0.61	AF001548.2	lncRNA	novel transc		16	15726674	15732993
ENSG0000008623	-0.039554749	0.452	0.61	EIF2AK1	protein_coding	eukaryotic t		7	6022247	6059175
ENSG0000023775	0.980503141	0.452	0.61	LINC01143	lncRNA	long interge		2	70887871	70889959
ENSG0000025091	-0.886389083	0.452	0.61	AC097467.3	lncRNA	novel transc		4	155173716	155381694
ENSG0000027452	0.980503141	0.452	0.61	AC008443.6	unprocessed_p	general tran		5	181291673	181295572
ENSG0000027972	0.858359344	0.452	0.61	SOWAHCP2	processed_pse	SOWAHC p		21	9082601	9083101
ENSG0000016676	0.273143505	0.453	0.61	CATSPER2	protein_coding	cation chann		15	43628503	43668118
ENSG0000016798	0.032942529	0.453	0.61	DDB1	protein_coding	damage spe		11	61299451	61342596
ENSG0000026034	-0.7083265	0.453	0.61	AC138811.2	protein_coding	novel protei		16	18788063	18801519
ENSG0000027271	0.632904815	0.453	0.61	AL121658.1	lncRNA	novel transc		2	32165046	32165757
ENSG0000027618	-0.370701675	0.453	0.61	NA	NA	NA	NA	NA	NA	
ENSG0000025813	0.699023882	0.453	0.61	AC007622.2	lncRNA	novel transc		12	107736555	107759968
ENSG0000017293	-0.101168563	0.453	0.61	ANKRD13D	protein_coding	ankyrin repe		11	67289300	67302485
ENSG0000019884	-0.139100989	0.454	0.61	KTI12	protein_coding	KTI12 chron		1	52032103	52033810
ENSG0000025515	0.441393831	0.454	0.61	HTD2	protein_coding	hydroxyacyl		3	58306245	58320193
ENSG0000026777	0.945486525	0.454	0.61	AC004221.1	lncRNA	novel transc		19	1238179	1239522
ENSG0000023364	-0.440544871	0.454	0.61	C6orf136	protein_coding	chromosom	CHR_HSCHR		30636880	30642942
ENSG0000027023	-1.155673562	0.454	0.61	NA	NA	NA	NA	NA	NA	
ENSG0000025574	-0.831898548	0.455	0.61	AP000808.1	lncRNA	novel transc		11	68941503	68942852
ENSG0000019893	0.211464737	0.455	0.61	GPRASP1	protein_coding	G protein-c	X		102651092	102659083
ENSG0000022351	0.751528117	0.455	0.61	KIF28P	transcribed_un	kinesin fami		1	246771837	246839611
ENSG0000021398	0.117195461	0.455	0.61	AP1G2	protein_coding	adaptor rela		14	23559565	23568070
ENSG0000023563	-0.449235567	0.455	0.61	NUS1P1	processed_pse	NUS1 pseud	X		47512602	47513483
ENSG0000015045	0.220054981	0.456	0.61	EEF1AKMT1	protein_coding	EEF1A lysine		13	20728731	20773961
ENSG0000013605	-0.276231775	0.456	0.61	VILL	protein_coding	villin like [So		3	37988059	38007188
ENSG0000027334	0.406586237	0.456	0.61	AC007663.4	lncRNA	novel transc		22	20318119	20318749

ENSG0000011412	-0.114679029	0.456	0.61	TFDP2	protein_coding	transcriptior	3	141944428	142149544
ENSG0000012679	0.14415004	0.456	0.61	L3HYPDH	protein_coding	trans-L-3-h	14	59460363	59484408
ENSG0000020530	-0.262137249	0.456	0.61	NT5M	protein_coding	5',3'-nucleo	17	17303335	17347663
ENSG0000018260	-0.522436778	0.456	0.61	SNORC	protein_coding	secondary c	2	232857270	232878708
ENSG0000027780	-0.52755934	0.457	0.62	AC006213.4	lncRNA	novel transc	19	43976815	43977448
ENSG0000027782	-1.071854735	0.457	0.62	PRH1	protein_coding	proline rich	CHR_HSCHR	10824960	11167778
ENSG0000013346	-0.216913343	0.457	0.62	SLC2A11	protein_coding	solute carrie	22	23856703	23886309
ENSG0000027004	-0.644298304	0.457	0.62	AC009061.2	lncRNA	novel transc	16	67481314	67505977
ENSG0000012032	0.362163248	0.457	0.62	PCDHB12	protein_coding	protocadhe	5	141208697	141212571
ENSG0000000523	-0.17242883	0.458	0.62	FAM214B	protein_coding	family with s	9	35104112	35116341
ENSG0000015219	-0.092335444	0.457	0.62	OBI1	protein_coding	ORC ubiquit	13	78614289	78659155
ENSG0000021499	-0.542890213	0.457	0.62	AC129492.1	lncRNA	novel transc	17	8079482	8081565
ENSG0000023118	0.762077092	0.457	0.62	AL954705.1	processed_pse	ribosomal p	1	9576427	9576985
ENSG0000024140	0.531901083	0.458	0.62	EGFL8	protein_coding	EGF like dor	6	32164595	32168281
ENSG0000022509	0.736262422	0.458	0.62	ATP6V1E1P	processed_pse	ATPase H+	1	42903232	42903912
ENSG0000027761	-0.329286202	0.458	0.62	CNOT3	protein_coding	CCR4-NOT	CHR_HSCHR	54138182	54156191
ENSG0000020712	-1.135279167	0.458	0.62	RNA5SP187	rRNA_pseudog	RNA, 5S ribo	5	88274258	88274376
ENSG0000027328	-0.429348725	0.459	0.62	NA	NA	NA	NA	NA	NA
ENSG0000027796	-1.151620441	0.459	0.62	AC091825.2	misc_RNA		5	142317830	142317920
ENSG0000025869	1.071992967	0.459	0.62	AC005225.3	lncRNA	novel transc	14	73522878	73530610
ENSG0000016587	-0.321546553	0.46	0.62	FRAT1	protein_coding	FRAT regula	10	97319271	97321915
ENSG0000024955	-0.326708212	0.46	0.62	LINC01234	lncRNA	long interge	12	113583886	113773726
ENSG0000025251	1.287388456	0.46	0.62	RNA5SP82	rRNA_pseudog	RNA, 5S ribo	1	247204570	247204661
ENSG0000027494	0.588962256	0.46	0.62	AC079684.1	lncRNA	novel transc	12	42361267	42361703
ENSG0000026210	-0.191826346	0.46	0.62	DSEL	protein_coding	dermatan su	CHR_HSCHR	67576031	67586429
ENSG0000022932	0.614436705	0.46	0.62	ACAP2-IT1	lncRNA	ACAP2 intro	3	195280723	195282741
ENSG0000022803	1.063569147	0.46	0.62	AC078842.1	lncRNA	novel transc	7	137344930	137354483
ENSG0000024855	1.150115166	0.46	0.62	AC024558.1	processed_pse	methyltrans	3	126624792	126626061
ENSG0000027001	-0.220292162	0.46	0.62	NA	NA	NA	NA	NA	NA
ENSG0000022639	-0.90585688	0.46	0.62	IFNA20P	unprocessed_p	interferon a	9	21321300	21321827
ENSG0000022698	0.779154424	0.46	0.62	PRELID1P5	processed_pse	PRELID1 pse	1	211207239	211207897
ENSG0000026021	-0.432696483	0.46	0.62	AC139426.1	processed_pse	hyperpolariz	15	32313126	32315654
ENSG0000027978	0.236527194	0.461	0.62	AC120114.3	TEC	novel transc	16	29913144	29915337
ENSG0000026670	-0.903032635	0.461	0.62	AP001793.1	lncRNA	novel transc	18	8635179	8636347
ENSG0000023396	0.57657793	0.461	0.62	AL359715.1	lncRNA	novel transc	6	80441295	80466674
ENSG0000023453	0.129066376	0.461	0.62	ATF6B	protein_coding	activating tr	CHR_HSCHR	32063716	32076703
ENSG0000025934	0.778011846	0.461	0.62	AC011921.1	lncRNA	novel transc	17	60526293	60550798
ENSG0000012269	0.052279292	0.462	0.62	SMU1	protein_coding	SMU1 DNA	9	33041765	33076674
ENSG0000011550	-0.049647446	0.462	0.62	EHBP1	protein_coding	EH domain	2	62673851	63046487
ENSG0000022806	0.282164445	0.462	0.62	LINC01515	lncRNA	long interge	10	65570338	65880289
ENSG0000023315	-0.732223638	0.462	0.62	HMGA1P8	processed_pse	high mobilit	2	74418122	74418442
ENSG0000023884	1.539154832	0.462	0.62	U8	snoRNA	U8 small nu	10	4962436	4962568
ENSG0000027561	1.539154832	0.462	0.62	U2	snRNA	U2 spliceoso	17	43239921	43240111
ENSG0000017234	0.363170545	0.462	0.62	STARD5	protein_coding	StAR relatec	15	81309053	81324183
ENSG0000025856	0.373815871	0.462	0.62	RHOQP1	processed_pse	ras homolog	14	49599994	49600572
ENSG0000014145	0.130176675	0.462	0.62	RMC1	protein_coding	regulator of	18	23503470	23531822
ENSG0000016248	-0.483416874	0.462	0.62	AKR7A3	protein_coding	aldo-keto re	1	19282573	19288770
ENSG0000025752	0.253369711	0.463	0.62	RPL36A-HN	protein_coding	RPL36A-HN	X	101391011	101412297
ENSG0000027827	1.468298779	0.463	0.62	NA	NA	NA	NA	NA	NA
ENSG0000020137	1.840526814	0.463	0.62	SNORA70	snoRNA	Small nucleo	14	51244090	51244224
ENSG0000023741	1.840526814	0.463	0.62	AC069257.2	processed_pse	ribosomal p	3	196298061	196298215
ENSG0000003644	-0.286570758	0.463	0.62	MYOM2	protein_coding	myomesin 2	8	2045046	2165552
ENSG0000004665	-0.092386899	0.463	0.62	OFD1	protein_coding	OFD1 centri	X	13734745	13769357
ENSG0000027516	-0.516665481	0.463	0.62	TSEN34	protein_coding	tRNA splicir	CHR_HSCHR	54190866	54195141
ENSG0000020349	-0.247848327	0.463	0.62	PDCD4-AS	lncRNA	PDCD4 antis	10	110869868	110872233
ENSG0000021438	-0.149730611	0.464	0.62	RPS3AP26	processed_pse	RPS3A pseu	7	98385801	98386584
ENSG0000027821	0.509624833	0.464	0.62	TRIM69	protein_coding	tripartite mo	CHR_HSCHR	44729020	44767863
ENSG0000012761	0.033728022	0.464	0.62	SMARCA4	protein_coding	SWI/SNF rel	19	10960932	11079426

ENSG0000013150	0.348372853	0.464	0.62	ANKHD1	protein_coding	ankyrin repe	5	140401814	140539856
ENSG0000016394	0.104088716	0.464	0.62	UVSSA	protein_coding	UV stimulat	4	1347208	1395989
ENSG0000013136	0.088881269	0.464	0.62	MRPS25	protein_coding	mitochondr	3	15042460	15065317
ENSG0000021301	1.070513067	0.465	0.62	AL590762.1	processed_pse	pseudogene	X	71420083	71420514
ENSG0000022388	-0.15147167	0.465	0.62	PRR3	protein_coding	proline rich	CHR_HSCHR	30547117	30553954
ENSG0000027727	-0.923493762	0.465	0.62	FAM243B	protein_coding	family with s	21	7768884	7770591
ENSG0000013399	-0.078535707	0.465	0.62	MED6	protein_coding	mediator cc	14	70581257	70600690
ENSG0000011214	0.053141124	0.465	0.62	CILK1	protein_coding	ciliogenesis	6	53001279	53061824
ENSG0000025926	-0.559575354	0.466	0.62	NA	NA	NA	NA	NA	NA
ENSG0000011416	-0.210891445	0.466	0.62	KAT2B	protein_coding	lysine acetyl	3	20040446	20154404
ENSG0000017120	-0.036799296	0.466	0.62	NETO2	protein_coding	neuropilin a	16	47077703	47143945
ENSG0000023327	-0.070318787	0.466	0.62	GPX1	polymorphic_p	glutathione	3	49357176	49358358
ENSG0000021371	0.126907459	0.466	0.62	CLIC1	protein_coding	chloride intr	6	31730581	31739763
ENSG0000025496	-0.603246766	0.466	0.62	AC103974.1	lncRNA	novel transc	11	18706537	18740568
ENSG0000027735	0.558401265	0.466	0.62	RPS9	protein_coding	ribosomal p	CHR_HSCHR	54201473	54208260
ENSG0000017048	0.604698329	0.466	0.62	SLC23A1	protein_coding	solute carrie	5	139367196	139384553
ENSG0000008070	-0.516492443	0.466	0.62	KCNN2	protein_coding	potassium c	5	114055926	114496500
ENSG0000023365	0.998114007	0.467	0.62	AC108047.1	lncRNA	novel transc	2	190534855	190569776
ENSG0000025159	0.170034051	0.467	0.62	ABCA11P	transcribed_pr	ATP binding	4	425435	474129
ENSG0000007077	-0.050525872	0.467	0.62	CSNK2A2	protein_coding	casein kinas	16	58157907	58198106
ENSG0000027257	0.860989049	0.467	0.62	AL596325.2	lncRNA	novel transc	1	162593103	162593754
ENSG0000026287	0.575343029	0.467	0.62	AC110285.2	lncRNA	novel transc	17	81388126	81390319
ENSG0000010823	0.441094457	0.467	0.62	LGI1	protein_coding	leucine rich	10	93757840	93806272
ENSG0000026665	0.720293353	0.467	0.62	AC093484.4	lncRNA	novel transc	17	16440479	16440952
ENSG0000026064	0.225720206	0.467	0.62	HERC2P5	transcribed_un	hect domair	16	32741314	32776196
ENSG0000020425	-0.172002506	0.467	0.62	HNRNPCP2	processed_pse	heterogene	2	189923336	189924216
ENSG0000015494	-0.050862324	0.467	0.63	ANKRD40	protein_coding	ankyrin repe	17	50693198	50707914
ENSG0000019661	-0.390759422	0.467	0.63	MMP1	protein_coding	matrix meta	11	102789919	102798160
ENSG0000020948	1.252888286	0.467	0.63	SNORD83A	snoRNA	small nuclec	22	39315213	39315307
ENSG0000022507	-0.404782338	0.467	0.63	AC004552.1	processed_pse	ribosomal p	X	24429573	24429920
ENSG0000022441	-1.114442783	0.467	0.63	AC007683.1	processed_pse	ribosomal p	7	103141349	103141939
ENSG0000018747	-0.095929932	0.468	0.63	C11orf96	protein_coding	chromosom	11	43925342	43943878
ENSG0000027671	1.374505808	0.468	0.63	MIR7111	miRNA	microRNA 7	6	35470508	35470579
ENSG0000028016	-0.587870486	0.468	0.63	AC135050.7	TEC	novel transc	16	31093727	31095944
ENSG0000023146	0.812537686	0.468	0.63	PRDX3P2	processed_pse	peroxiredox	1	28526318	28527227
ENSG0000026421	0.672828364	0.468	0.63	NA	NA	NA	NA	NA	NA
ENSG0000024254	0.512584934	0.469	0.63	AC010729.2	lncRNA	novel transc	2	5696220	5719670
ENSG0000000477	0.583459509	0.469	0.63	HSPB6	protein_coding	heat shock p	19	35754566	35758079
ENSG0000023840	1.001996043	0.469	0.63	RNA5SP311	rRNA_pseudog	RNA, 5S ribo	10	46807458	46807573
ENSG0000025831	-0.28558841	0.469	0.63	AC009779.3	protein_coding	novel protei	12	55716036	55724703
ENSG0000026301	0.394534414	0.469	0.63	NA	NA	NA	NA	NA	NA
ENSG0000019637	-0.258826132	0.469	0.63	FUT4	protein_coding	fucosyltrans	11	94543840	94549898
ENSG0000027855	-0.392513603	0.47	0.63	SLC43A2	protein_coding	solute carrie	CHR_HSCHR	1569973	1628886
ENSG0000022380	0.295949229	0.47	0.63	RPS20P14	processed_pse	ribosomal p	3	186900198	186900557
ENSG0000027509	-0.075737886	0.47	0.63	GGNBP2	protein_coding	gametogen	CHR_HSCHR	36545260	36590811
ENSG0000012037	0.11199784	0.47	0.63	GORAB	protein_coding	golgin, RAB	1	170532131	170553834
ENSG0000021534	0.760830645	0.47	0.63	SLC25A5P1	transcribed_pr	solute carrie	22	42000446	42001991
ENSG0000018679	-0.305111369	0.47	0.63	HYAL3	protein_coding	hyaluronida	3	50292831	50299405
ENSG0000027081	0.141251563	0.47	0.63	LINC00221	lncRNA	long interge	14	106482435	106521073
ENSG0000011542	-0.079572226	0.47	0.63	PAPOLG	protein_coding	poly(A) poly	2	60756253	60802086
ENSG0000023724	0.306640692	0.471	0.63	LINC00987	lncRNA	long interge	12	9240003	9257960
ENSG0000027197	-0.423478086	0.471	0.63	AL359643.2	lncRNA	novel transc	6	5031756	5054423
ENSG0000027527	-0.063480995	0.471	0.63	YTHDC1	protein_coding	YTH domair	CHR_HSCHR	68310387	68350089
ENSG0000010850	0.071651856	0.471	0.63	INTS2	protein_coding	integrator c	17	61865367	61928016
ENSG0000013266	0.090966021	0.471	0.63	POLR3F	protein_coding	RNA polyme	20	18467389	18484646
ENSG0000022409	-0.498091247	0.471	0.63	AC021148.1	transcribed_pr	WD repeat	4	39480255	39481905
ENSG0000027722	0.067650453	0.471	0.63	BTBD7	protein_coding	BTB domair	CHR_HSCHR	93237550	93333092
ENSG0000023199	-0.410211981	0.471	0.63	LRRC8C-DT	lncRNA	LRRC8C div	1	89581291	89632916

ENSG0000025383	1.266477085	0.471	0.63	AC022868.1	processed_pse	destrin (acti	8	73984493	73984883
ENSG0000008465	0.045370422	0.471	0.63	TXLNA	protein_coding	taxilin alpha	1	32179675	32198285
ENSG0000017045	0.070188202	0.471	0.63	DENND5B	protein_coding	DENN doma	12	31382226	31591136
ENSG0000027859	0.359722655	0.471	0.63	NA	NA	NA	NA	NA	NA
ENSG0000016299	-0.313117291	0.471	0.63	PRORSD1P	transcribed_un	prolyl-tRNA	2	55282319	55284522
ENSG0000023084	1.166349898	0.471	0.63	RPL23AP15	processed_pse	ribosomal p	1	228449163	228449621
ENSG0000025628	-0.838679495	0.472	0.63	AC112694.1	transcribed_pr	mitochondr	11	18595459	18599683
ENSG0000027464	0.402237444	0.472	0.63	AC244669.2	unprocessed_p	phosphodie	1	120197085	120319680
ENSG0000010977	-0.231074391	0.472	0.63	LRP2BP	protein_coding	LRP2 bindin	4	185363879	185395899
ENSG0000015744	0.178048127	0.472	0.63	CACNA2D3	protein_coding	calcium volt	3	54122547	55074557
ENSG0000026092	-0.623465289	0.472	0.63	LINC02193	lncRNA	long interge	16	90173217	90222851
ENSG0000023819	-0.519895565	0.472	0.63	NOTCH4	protein_coding	notch recep	CHR_HSCHR	32261849	32271406
ENSG0000023700	-0.658165118	0.473	0.63	ZNRF2P1	transcribed_pr	zinc and rin	7	32728569	32753744
ENSG0000017798	0.652673684	0.473	0.63	ODF3B	protein_coding	outer dense	22	50529710	50532580
ENSG0000018670	-0.646944891	0.473	0.63	DTX2P1	transcribed_un	DTX2 pseuc	7	76978617	77004308
ENSG0000027498	0.746699619	0.473	0.63	AC092794.1	lncRNA	novel transc	12	25210652	25211233
ENSG0000010194	0.119101852	0.473	0.63	SUV39H1	protein_coding	suppressor (	X	48695554	48709016
ENSG0000023111	0.15146925	0.473	0.63	RING1	protein_coding	ring finger p	CHR_HSCHR	33378855	33383082
ENSG0000026691	0.368892776	0.473	0.63	ZNF793-AS	lncRNA	ZNF793 ant	19	37497159	37507122
ENSG0000016260	0.157251546	0.474	0.63	OMA1	protein_coding	OMA1 zinc	1	58415384	58546802
ENSG0000018306	0.539386536	0.474	0.63	WBP2NL	protein_coding	WBP2 N-ter	22	41998725	42058456
ENSG0000024812	0.651344916	0.474	0.63	AC026774.1	lncRNA	novel transc	5	76081946	76084186
ENSG0000027258	0.36911172	0.474	0.63	AC139887.4	lncRNA	novel transc	4	757022	757740
ENSG0000015244	0.097806696	0.474	0.63	ZNF776	protein_coding	zinc finger p	19	57746815	57758148
ENSG0000026778	-0.424449758	0.474	0.63	AC027097.2	lncRNA	novel transc	18	57639455	57738603
ENSG0000026594	0.848771529	0.474	0.63	AC090912.1	lncRNA	novel transc	18	22699481	22933764
ENSG0000017089	-1.043175541	0.475	0.63	PLA2G1B	protein_coding	phospholipa	12	120322115	120327779
ENSG0000024239	1.239074916	0.475	0.63	AC099677.3	lncRNA	novel transc	1	52881216	52881730
ENSG0000027625	-1.20718895	0.475	0.63	AC243790.1	protein_coding		CHR_HSCHR	30361040	30361438
ENSG0000019851	-0.310359511	0.475	0.63	MAFK	protein_coding	MAF bZIP tr	7	1530702	1543043
ENSG0000027634	-0.431801433	0.475	0.63	AC004556.3	protein_coding	39S ribosom	KI270721.1	2585	11802
ENSG0000013446	-0.151286383	0.475	0.63	ANKRD16	protein_coding	ankyrin repe	10	5861616	5889906
ENSG0000018777	-0.067806179	0.475	0.63	MCRS1	protein_coding	microspheru	12	49556544	49568145
ENSG0000020671	-0.974177052	0.475	0.63	RNU6-26P	snRNA	RNA, U6 sm	3	98804978	98805084
ENSG0000018772	0.682712271	0.475	0.63	DNAJB13	protein_coding	DnaJ heat sl	11	73951026	73970366
ENSG0000020387	0.351508576	0.475	0.63	C6orf163	protein_coding	chromosom	6	87344813	87365463
ENSG0000026036	0.560334301	0.475	0.63	AC109460.1	lncRNA	novel transc	16	28973962	28978824
ENSG0000011160	-0.036370513	0.475	0.63	CPSF6	protein_coding	cleavage an	12	69239569	69274358
ENSG0000027260	-0.634572214	0.476	0.63	AC010655.3	transcribed_un	EF-hand cal	7	128466563	128469171
ENSG0000012792	0.266169773	0.476	0.63	GNG11	protein_coding	G protein su	7	93921735	93928610
ENSG0000016534	0.564578435	0.476	0.63	SLC7A3	protein_coding	solute carrie	X	70925579	70931125
ENSG0000021441	-0.087941735	0.476	0.63	BBIP1	protein_coding	BBSome inte	10	110898730	110919201
ENSG0000001027	-0.090156993	0.476	0.63	CD9	protein_coding	CD9 molecu	12	6199715	6238271
ENSG0000016794	0.776872514	0.476	0.63	NA	NA	NA	NA	NA	NA
ENSG0000025485	1.09269224	0.476	0.63	AP001107.8	lncRNA	novel transc	11	66264777	66265666
ENSG0000022993	-0.345521242	0.477	0.63	AL589880.1	processed_pse	novel pseuc	X	111618808	111619094
ENSG0000027410	-0.648306661	0.477	0.63	AC084824.3	lncRNA	novel transc	12	32728169	32729024
ENSG0000021413	-0.095163046	0.477	0.63	AC132008.2	transcribed_un	succinate de	3	197578213	197627906
ENSG0000027920	0.872060685	0.477	0.63	AC005785.2	TEC	TEC	19	15383202	15383832
ENSG0000008444	0.119728192	0.477	0.63	FAM234B	protein_coding	family with s	12	13044381	13142521
ENSG0000022747	-0.564509055	0.477	0.63	RPL39P16	processed_pse	ribosomal p	2	108878308	108878460
ENSG0000025310	-0.21295846	0.477	0.63	AC090198.1	lncRNA	novel transc	8	124488510	124491643
ENSG0000026314	0.502332778	0.477	0.63	C21orf62-A	lncRNA	C21orf62 ar	CHR_HSCHR	32772100	32798969
ENSG0000024151	-1.177392273	0.477	0.63	RPS15AP24	processed_pse	ribosomal p	8	30117384	30117779
ENSG0000020656	0.356411382	0.477	0.63	COLQ	protein_coding	collagen like	3	15450133	15521751
ENSG0000027745	-0.611846424	0.478	0.63	LENG1	protein_coding	leukocyte re	CHR_HSCHR	54156127	54160192
ENSG0000017326	0.105225777	0.478	0.63	GPR137	protein_coding	G protein-c	11	64270062	64289500
ENSG0000018607	0.136526154	0.478	0.63	AC012085.1	processed_pse	phosphogly	12	93640822	93641586

ENSG0000027265	-0.29627348	0.478	0.63	AL358472.2	lncRNA	novel transc	1	153977743	153979160
ENSG0000025951	-0.774552804	0.478	0.63	AL117209.1	lncRNA	novel transc	14	102933574	102937177
ENSG0000026426	1.162749939	0.478	0.63	MIR4767	miRNA	microRNA 4	X	7147860	7147937
ENSG0000011669	-0.128028951	0.478	0.63	MIIP	protein_coding	migration ar	1	12019466	12032045
ENSG0000019635	-0.27368469	0.478	0.63	NTNG2	protein_coding	netrin G2 [S	9	132161676	132244526
ENSG0000027252	-0.486101759	0.478	0.63	LINC01023	lncRNA	long interge	5	108727825	108728260
ENSG0000025717	-0.525987781	0.479	0.64	CR383656.1	processed_pse	ubiquitin sp	14	18634955	18637208
ENSG0000001206	-0.084554934	0.479	0.64	ERCC1	protein_coding	ERCC excisio	19	45407334	45478828
ENSG0000019645	0.097723243	0.479	0.64	TRAPPC2	protein_coding	trafficking p	X	13712244	13734635
ENSG0000026159	0.833556666	0.479	0.64	AC005632.3	lncRNA	novel transc	16	21626742	21627569
ENSG0000024584	-0.199880993	0.479	0.64	RAD51-AS1	lncRNA	RAD51 antis	15	40686183	40695107
ENSG0000026064	0.364457411	0.479	0.64	AL359715.2	lncRNA	novel transc	6	80466958	80469080
ENSG0000024897	0.686012904	0.479	0.64	KRT8P46	processed_pse	keratin 8 ps	4	102728746	102730171
ENSG0000027006	0.686012904	0.479	0.64	AC068790.9	lncRNA	novel transc	12	123969990	123970344
ENSG0000013757	-0.058261813	0.479	0.64	TGS1	protein_coding	trimethylgua	8	55773446	55826445
ENSG0000026701	0.625778862	0.479	0.64	AC011498.1	lncRNA	novel transc	19	4457962	4471493
ENSG0000014667	0.813227328	0.479	0.64	AC004453.1	processed_pse	ribosomal p	7	44467897	44468304
ENSG0000020727	1.505060219	0.479	0.64	RNA5SP284	rRNA_pseudog	RNA, 5S ribo	9	63813190	63813307
ENSG0000022523	-0.573821403	0.48	0.64	AC008937.1	lncRNA	novel transc	5	56900041	56910714
ENSG0000026760	0.604392064	0.48	0.64	AC016590.1	lncRNA	novel transc	19	37235507	37304395
ENSG0000027496	1.242200363	0.48	0.64	RN7SL600P	misc_RNA	RNA, 7SL, cy	1	150568971	150569269
ENSG0000014765	0.064086051	0.48	0.64	LRP12	protein_coding	LDL recepto	8	104489231	104589024
ENSG0000027193	0.370216716	0.481	0.64	AL603756.1	lncRNA	novel transc	10	84138420	84140582
ENSG0000022598	0.1096557	0.481	0.64	ABCF1	protein_coding	ATP binding	CHR_HSCHR	30563598	30589402
ENSG0000011675	-0.048393181	0.481	0.64	UCHL5	protein_coding	ubiquitin C-	1	193012250	193060080
ENSG0000021359	0.170968793	0.481	0.64	SLX1A-SUL	lncRNA	SLX1A-SULT	16	30193892	30204310
ENSG0000027389	0.569528787	0.481	0.64	TSEN34	protein_coding	tRNA splicin	CHR_HSCHR	54190866	54195141
ENSG0000015099	0.105748641	0.481	0.64	UBC	protein_coding	ubiquitin C	12	124911604	124917368
ENSG0000011053	0.089797746	0.481	0.64	PTPMT1	protein_coding	protein tyro	11	47565430	47573461
ENSG0000003305	-0.049106587	0.481	0.64	ABCF2	protein_coding	ATP binding	7	151211484	151227205
ENSG0000019816	-0.113270293	0.481	0.64	ZNF251	protein_coding	zinc finger p	8	144720907	144756417
ENSG0000018683	-0.061477488	0.481	0.64	HEXIM1	protein_coding	HEXIM P-TE	17	45148475	45152099
ENSG0000022361	-0.409780875	0.481	0.64	VPS52	protein_coding	VPS52 subu	CHR_HSCHR	33404140	33405839
ENSG0000017703	0.056795994	0.481	0.64	MTX3	protein_coding	metaxin 3 [S	5	79976716	79991262
ENSG0000014441	-0.63735777	0.482	0.64	CPO	protein_coding	carboxypep	2	206939518	206969474
ENSG0000026404	0.900673733	0.482	0.64	MIR4737	miRNA	microRNA 4	17	60043025	60043105
ENSG0000027215	0.388188496	0.482	0.64	AL365330.1	lncRNA	novel transc	1	3785008	3785538
ENSG0000008912	-0.18599517	0.482	0.64	TASP1	protein_coding	taspase 1 [S	20	13389392	13638932
ENSG0000010290	-0.488731411	0.483	0.64	TSNAXIP1	protein_coding	translin asso	16	67806765	67832148
ENSG0000016601	0.100975518	0.483	0.64	ABTB2	protein_coding	ankyrin repe	11	34150987	34358010
ENSG0000014341	-0.239042739	0.483	0.64	SELENBP1	protein_coding	selenium bi	1	151364304	151372707
ENSG0000020628	-0.230877909	0.483	0.64	TAPBP	protein_coding	TAP binding	CHR_HSCHR	33228522	33243221
ENSG0000013636	0.122101824	0.483	0.64	ZFHX2	protein_coding	zinc finger h	14	23520857	23556192
ENSG0000026928	-1.155279053	0.483	0.64	AC092070.3	lncRNA	novel transc	19	53240675	53241913
ENSG0000023435	0.666284829	0.483	0.64	AP000346.2	transcribed_un	immunoglob	22	23638492	23640762
ENSG0000024043	0.362755499	0.484	0.64	LY6G5B	protein_coding	lymphocyte	CHR_HSCHR	31709833	31713000
ENSG0000027476	0.564556541	0.484	0.64	AC016747.4	lncRNA	novel transc	2	61115787	61164825
ENSG0000013321	-0.108969243	0.484	0.64	EPHB2	protein_coding	EPH recepto	1	22710839	22921500
ENSG0000026301	0.96966457	0.484	0.64	AC108134.4	lncRNA	novel transc	16	3106764	3109576
ENSG0000012856	-0.108392507	0.484	0.64	PODXL	protein_coding	podocalyxin	7	131500262	131558217
ENSG0000011542	-0.13495344	0.485	0.64	PECR	protein_coding	peroxisoma	2	215996329	216082955
ENSG0000012347	-0.073244101	0.485	0.64	ATPAF1	protein_coding	ATP synthas	1	46632737	46673867
ENSG0000010044	0.051070453	0.485	0.64	KHNYN	protein_coding	KH and NYN	14	24429286	24441843
ENSG0000023009	0.712216579	0.485	0.64	AL669831.4	transcribed_un	F-box only p	1	800879	817712
ENSG0000018162	0.268629792	0.485	0.64	SLX1B	protein_coding	SLX1 homol	16	29454501	29458219
ENSG0000027294	-0.455073357	0.485	0.64	AC083862.1	lncRNA	novel transc	7	135168403	135169547
ENSG0000026757	0.706930779	0.485	0.64	AC104532.2	lncRNA	novel transc	19	5911578	5913899
ENSG0000015810	0.094559167	0.485	0.64	TPRG1L	protein_coding	tumor prote	1	3625015	3630127

ENSG0000006865	0.041156794	0.485	0.64	POLR1A	protein_coding	RNA polyme		2	86020216	86106155
ENSG0000025410	-0.920501899	0.486	0.64	NA	NA	NA	NA	NA	NA	
ENSG0000024285	-0.165767897	0.486	0.64	ZNF709	protein_coding	zinc finger p		19	12461184	12513854
ENSG0000016856	-0.071216711	0.486	0.64	CDKN2AIP	protein_coding	CDKN2A int		4	183444635	183449064
ENSG0000027199	-0.276764754	0.486	0.64	NA	NA	NA	NA	NA	NA	
ENSG0000027175	-0.805656899	0.487	0.64	AP003392.4	lncRNA	novel transc		11	119065263	119065677
ENSG0000013070	0.080413604	0.487	0.64	LAMA5	protein_coding	laminin subu		20	62307955	62367312
ENSG0000025489	-3.177160648	0.487	0.64	BX248516.1	protein_coding	ATPase H+	CHR_HSCHR		31520968	31537331
ENSG0000020182	1.21423111	0.488	0.64	Y_RNA	misc_RNA	Y RNA [Sou		14	51253933	51254023
ENSG0000025842	-0.195819924	0.488	0.64	PDF	protein_coding	peptide defo		16	69326913	69330588
ENSG0000022811	-0.824181532	0.488	0.64	PPT2	protein_coding	palmitoyl-p	CHR_HSCHR		32129213	32134104
ENSG0000018769	0.415189134	0.488	0.64	C2orf88	protein_coding	chromosom		2	189879609	190203484
ENSG0000027335	0.851623462	0.488	0.64	AL008718.3	lncRNA	novel transc		22	45178990	45179310
ENSG0000000898	-0.034997634	0.488	0.64	RPS20	protein_coding	ribosomal p		8	56067295	56074581
ENSG0000027468	1.014528935	0.488	0.64	SUDS3P1	processed_pse	SDS3 homo	CHR_HSCHR		177974393	177975358
ENSG0000021388	-0.888726279	0.488	0.64	NPM1P6	processed_pse	nucleophos		8	61202350	61203220
ENSG0000023097	-0.325389415	0.488	0.64	AC079250.1	processed_pse	ribosomal p		2	47690716	47691246
ENSG0000018739	-0.725676046	0.488	0.64	MAGI2	protein_coding	membrane a		7	78017055	79453667
ENSG0000021302	1.232131535	0.488	0.64	COX20P1	processed_pse	COX20 pseu		10	68632371	68632727
ENSG0000023166	0.479212227	0.488	0.64	COA6-AS1	lncRNA	COA6 antise		1	234372807	234373593
ENSG0000027773	-0.53866883	0.488	0.64	MBOAT7	protein_coding	membrane I	CHR_HSCHR		54173854	54190480
ENSG0000017838	0.248988753	0.489	0.64	ZNF223	protein_coding	zinc finger p		19	44051367	44067999
ENSG0000012016	-0.167501754	0.489	0.64	MOB3B	protein_coding	MOB kinase		9	27325209	27529814
ENSG0000017016	-0.787138943	0.489	0.64	CCDC144A	protein_coding	coiled-coil c		17	16689537	16777881
ENSG0000017116	-0.118537726	0.489	0.64	ZNF672	protein_coding	zinc finger p		1	248838210	248849517
ENSG0000013481	-0.106321103	0.489	0.64	DHX34	protein_coding	DExH-box h		19	47349315	47382704
ENSG0000023656	0.248708308	0.489	0.64	HNRNPA3P	processed_pse	heterogene		13	65787932	65788764
ENSG0000026434	-0.743262037	0.489	0.64	MIR4258	miRNA	microRNA 4		1	154975693	154975783
ENSG0000015725	-0.065639565	0.489	0.65	GATAD1	protein_coding	GATA zinc f		7	92447482	92460075
ENSG0000020698	-0.884214643	0.489	0.65	SNORD63	snoRNA	small nucleo		5	138561043	138561110
ENSG0000028030	0.797661735	0.49	0.65	ERICD	lncRNA	E2F1-regula		8	140636281	140638283
ENSG0000016008	-0.068358077	0.49	0.65	UBE2J2	protein_coding	ubiquitin co		1	1253909	1273864
ENSG0000011711	0.06758864	0.49	0.65	SDHB	protein_coding	succinate de		1	17018722	17054032
ENSG0000013077	0.164460335	0.49	0.65	MED18	protein_coding	mediator co		1	28329002	28335965
ENSG0000026946	-1.193497142	0.49	0.65	AP001160.4	lncRNA	novel transc		11	62807682	62808063
ENSG0000026989	-0.832852889	0.49	0.65	AC018809.1	lncRNA	novel transc		3	9935706	9936258
ENSG0000023567	0.27006025	0.49	0.65	ABHD16A	protein_coding	abhydrolase	CHR_HSCHR		31674358	31690824
ENSG0000017684	0.32181479	0.49	0.65	METRNL	protein_coding	meteorin lik		17	83079609	83095122
ENSG0000018422	-0.056569621	0.491	0.65	CMSS1	protein_coding	cms1 ribosc		3	99817837	100181732
ENSG0000016652	-0.115708998	0.491	0.65	ZSCAN21	protein_coding	zinc finger a		7	100049774	100065040
ENSG0000025725	-0.895854949	0.491	0.65	AC012150.1	lncRNA	novel transc		12	29142969	29158537
ENSG0000007594	0.07241203	0.491	0.65	KIFAP3	protein_coding	kinesin asso		1	169921326	170085208
ENSG0000015791	0.115919458	0.492	0.65	PEX10	protein_coding	peroxisoma		1	2403964	2413797
ENSG0000022753	-0.584161113	0.492	0.65	SOCS5P4	processed_pse	suppressor o	X		71043214	71045470
ENSG0000022567	1.04636151	0.493	0.65	LINC01771	lncRNA	long interge		1	53328233	53336509
ENSG0000025050	0.608440001	0.493	0.65	CDK3	protein_coding	cyclin deper		17	76000906	76005999
ENSG0000012670	0.229710356	0.494	0.65	IFI6	protein_coding	interferon a		1	27666064	27672198
ENSG0000015701	0.075265202	0.493	0.65	TATDN2	protein_coding	TatD DNase		3	10248023	10281218
ENSG0000026476	-0.831618235	0.494	0.65	AC145207.8	lncRNA	novel transc		17	81922899	81924511
ENSG0000010557	-0.038930372	0.494	0.65	TNPO2	protein_coding	transportin 2		19	12699194	12724011
ENSG0000012460	0.390510679	0.494	0.65	UNC5CL	protein_coding	unc-5 family		6	41026895	41039221
ENSG0000017830	-0.116777976	0.494	0.65	TMEM11	protein_coding	transmembr		17	21197954	21214176
ENSG0000019788	-0.134790727	0.494	0.65	NKIRAS1	protein_coding	NFKB inhibi		3	23889951	23946591
ENSG0000025856	0.953243461	0.494	0.65	AL359232.1	lncRNA	novel transc		14	66212810	66509394
ENSG0000027341	0.907842754	0.494	0.65	AL732292.2	lncRNA	novel transc		1	235104180	235104609
ENSG0000014121	0.057238579	0.494	0.65	C17orf80	protein_coding	chromosom		17	73232233	73248947
ENSG0000020456	-0.54000161	0.494	0.65	MRPS18B	protein_coding	mitochondr		6	30617840	30626395
ENSG0000027568	0.798547054	0.494	0.65	NA	NA	NA	NA	NA	NA	

ENSG0000003912	-0.036061988	0.495	0.65	MTREX	protein_coding	Mtr4 exosor		5	55307989	55425579
ENSG0000022482	-0.351880888	0.495	0.65	RORB-AS1	lncRNA	RORB antise		9	74485551	74499127
ENSG0000023008	0.846933159	0.495	0.65	AC006059.1	lncRNA	novel transc		3	42601963	42654388
ENSG0000023615	-1.270901515	0.494	0.65	MRPS36P1	processed_pse	mitochondr		3	6773037	6773343
ENSG0000027510	1.454923746	0.495	0.65	MIR6766	miRNA	microRNA 6		15	89326739	89326810
ENSG0000018350	-0.273591419	0.495	0.65	TENT5C	protein_coding	terminal nu		1	117606048	117628389
ENSG0000025428	0.920524245	0.495	0.65	NA	NA	NA	NA	NA	NA	
ENSG0000012396	0.679126342	0.495	0.65	PMS2P5	unprocessed_p	PMS1 homo		7	74894116	74897835
ENSG0000018339	1.900043415	0.495	0.65	C19orf71	protein_coding	chromosom		19	3539154	3544030
ENSG0000023807	-0.595648813	0.495	0.65	LINC01352	lncRNA	long interge		1	220829255	220832429
ENSG0000015191	-0.102425088	0.495	0.65	BEND6	protein_coding	BEN domair		6	56955107	57027346
ENSG0000023249	-0.360138556	0.496	0.65	AL391058.1	processed_pse	heterogene		1	113449700	113450728
ENSG0000023043	1.303950765	0.496	0.65	AC114803.1	lncRNA	novel transc		2	219299002	219304130
ENSG0000023204	-0.308482776	0.496	0.65	SILC1	lncRNA	sciatic injury		2	5932543	6003510
ENSG0000026734	-0.529453614	0.496	0.65	GEMIN7-AS	lncRNA	GEMIN7 ant		19	45076510	45092635
ENSG0000027942	0.43000473	0.496	0.65	AC092279.2	TEC	TEC		19	24078481	24080669
ENSG0000025379	0.057686399	0.496	0.65	UTP14C	protein_coding	UTP14C sma		13	52024691	52033600
ENSG0000017371	-0.126629005	0.496	0.65	C11orf80	protein_coding	chromosom		11	66744451	66843516
ENSG0000011339	0.071435253	0.497	0.65	FAM172A	protein_coding	family with s		5	93617725	94111699
ENSG0000027252	0.788603623	0.497	0.65	NA	NA	NA	NA	NA	NA	
ENSG0000023555	-0.768847418	0.497	0.65	AC005822.1	processed_pse	splicing fact		17	16616848	16617881
ENSG0000024003	-0.284172835	0.497	0.65	AC104563.1	processed_pse	novel pseuc		11	27581680	27582055
ENSG0000006133	-0.072820521	0.497	0.65	LZTS1	protein_coding	leucine zipp		8	20246165	20303963
ENSG0000018232	0.330355615	0.497	0.65	KCNJ14	protein_coding	potassium ir		19	48455574	48466980
ENSG0000027549	0.786458551	0.498	0.65	LINC01730	lncRNA	long interge		20	3808357	3812434
ENSG0000027068	0.312803655	0.498	0.65	AC095055.1	lncRNA	novel transc		4	151139991	151141103
ENSG0000023038	-0.214749163	0.498	0.65	AC009245.1	processed_pse	ribosomal p		7	137721985	137722846
ENSG0000012947	-0.068910306	0.498	0.65	RAB2B	protein_coding	RAB2B, mer		14	21459020	21476960
ENSG0000017015	-1.455646674	0.498	0.65	AL391987.1	protein_coding			9	61861848	61862567
ENSG0000018480	0.591898344	0.498	0.65	B3GALT5-A	lncRNA	B3GALT5 ar		21	39597147	39612910
ENSG0000025219	-1.455646674	0.498	0.65	AC127002.1	snoRNA			12	122492113	122492241
ENSG0000027639	0.89802639	0.498	0.65	AC004241.3	lncRNA	novel transc		12	47699401	47699917
ENSG0000021390	-0.146134567	0.498	0.65	LTB4R	protein_coding	leukotriene		14	24311450	24318036
ENSG0000017808	0.206034206	0.498	0.65	TWF1P1	processed_pse	twinfilin 1 p		17	29203426	29204474
ENSG0000020650	0.224839543	0.499	0.65	PPP1R11	protein_coding	protein pho	CHR_HSCHR		30056276	30059900
ENSG0000027777	0.088588779	0.499	0.65	SMN2	protein_coding	survival of n	CHR_HSCHR		69673667	69702549
ENSG0000010625	0.522775234	0.499	0.65	CYP3A5	protein_coding	cytochrome		7	99648194	99679998
ENSG0000012902	-0.123172381	0.499	0.65	THAP10	protein_coding	THAP doma		15	70881342	70892433
ENSG0000025544	-0.794421352	0.499	0.65	AP002812.5	lncRNA	novel transc		11	77866412	77870091
ENSG0000026570	1.437705313	0.499	0.65	SNORD53B	snoRNA	small nucle		2	28927983	28928060
ENSG0000022810	-0.379223966	0.5	0.65	NA	NA	NA	NA	NA	NA	
ENSG0000023572	-0.180023698	0.5	0.65	HCG18	lncRNA	HLA comple	CHR_HSCHR		30354705	30391047
ENSG0000001142	0.36048348	0.5	0.65	PLAUR	protein_coding	plasminoge		19	43646095	43670547
ENSG0000016362	-0.040310102	0.5	0.65	PTPN13	protein_coding	protein tyro		4	86594315	86815171
ENSG0000025043	-0.850926377	0.5	0.65	FAM242C	lncRNA	family with s		12	54085132	54125992
ENSG0000023256	0.224340277	0.5	0.65	GABBR1	protein_coding	gamma-am	CHR_HSCHR		29555714	29633284
ENSG0000015971	-0.382880807	0.501	0.66	TPPP3	protein_coding	tubulin poly		16	67389809	67393518
ENSG0000027438	0.730981077	0.501	0.66	AC103691.1	lncRNA	novel transc		15	64950916	64951435
ENSG0000020607	0.142746048	0.501	0.66	ZDHHC11B	protein_coding	zinc finger D		5	710355	784729
ENSG0000027004	0.403997135	0.501	0.66	NA	NA	NA	NA	NA	NA	
ENSG0000023437	-0.339508606	0.501	0.66	GTF2H4	protein_coding	general trar	CHR_HSCHR		30986738	30992660
ENSG0000011533	0.458338702	0.501	0.66	GALNT3	protein_coding	polypeptide		2	165747588	165794659
ENSG0000023308	0.594685051	0.501	0.66	RAB6D	protein_coding	RAB6D, mer		2	131361111	131364142
ENSG0000027861	0.801329659	0.501	0.66	ZNF426-DT	lncRNA	ZNF426 dive		19	9538693	9539734
ENSG0000023651	0.611180045	0.501	0.66	AL135791.1	lncRNA	novel transc		10	37857740	37859110
ENSG0000013054	-0.095311102	0.502	0.66	SULT4A1	protein_coding	sulfotransfe		22	43824509	43862513
ENSG0000021454	-0.307144877	0.502	0.66	GTF2IRD2P	transcribed_un	GTF2I repea		7	73242751	73280119
ENSG0000025715	0.100047339	0.502	0.66	NA	NA	NA	NA	NA	NA	

ENSG0000009526	0.060068426	0.502	0.66	PSMD5	protein_coding	proteasome		9	120815496	120842951
ENSG0000013029	0.033900966	0.502	0.66	KIF1A	protein_coding	kinesin fami		2	240713761	240824293
ENSG0000019816	-0.058768049	0.502	0.66	MIER1	protein_coding	MIER1 trans		1	66924895	66988619
ENSG0000025674	1.07713951	0.502	0.66	MADD-AS1	lncRNA	MADD antis		11	47270657	47272110
ENSG0000012792	0.062813263	0.502	0.66	SEM1	protein_coding	SEM1 26S p		7	96481626	96709891
ENSG0000027011	-0.540686483	0.503	0.66	AL353593.2	lncRNA	novel transc		1	228295549	228303002
ENSG0000014549	0.071106044	0.503	0.66	NDUFS6	protein_coding	NADH:ubiqu		5	1801407	1816048
ENSG0000018478	-0.042185655	0.503	0.66	UBE2G2	protein_coding	ubiquitin co		21	44768580	44801826
ENSG0000024405	-0.510496178	0.503	0.66	AC007566.1	lncRNA	novel transc		7	92457564	92495601
ENSG0000027465	0.635633457	0.503	0.66	AC106782.5	lncRNA	novel transc		16	30359825	30360336
ENSG0000018181	0.116690075	0.503	0.66	LSM10	protein_coding	LSM10, U7 s		1	36391238	36397908
ENSG0000019794	-0.15791782	0.504	0.66	FCHSD1	protein_coding	FCH and do		5	141639302	141651418
ENSG0000014979	0.059533546	0.504	0.66	MRPL49	protein_coding	mitochondr		11	65122183	65127371
ENSG0000017176	-0.39712537	0.504	0.66	GATM	protein_coding	glycine amic		15	45361124	45402327
ENSG0000027098	-0.760863285	0.504	0.66	AL589674.1	processed_pse	ubiquitin-lik		6	142062717	142063053
ENSG0000020430	-0.637540115	0.504	0.66	AGER	protein_coding	advanced g		6	32180968	32184322
ENSG0000013650	0.046291451	0.504	0.66	KAT7	protein_coding	lysine acetyl		17	49788648	49835026
ENSG0000013693	0.038473076	0.504	0.66	PSMB7	protein_coding	proteasome		9	124353465	124415444
ENSG0000022843	0.562119111	0.504	0.66	AC004951.1	lncRNA	novel transc		7	44004046	44007866
ENSG0000016637	0.110783837	0.505	0.66	ATP9B	protein_coding	ATPase pho		18	79069285	79378287
ENSG0000027275	-0.321593154	0.505	0.66	AL592148.3	lncRNA	novel transc		1	222658867	222661512
ENSG0000022622	-0.246328253	0.505	0.66	RPL26P19	processed_pse	ribosomal p		5	56504635	56505072
ENSG0000027922	-0.296747058	0.505	0.66	GPR1-AS	lncRNA	GPR1 antise		2	206203543	206265326
ENSG0000012931	-0.091529136	0.505	0.66	PUS7L	protein_coding	pseudouridi		12	43718993	43758817
ENSG0000019607	0.072369156	0.505	0.66	BLOC1S2	protein_coding	biogenesis c		10	100273280	100286680
ENSG0000021494	-0.148434233	0.505	0.66	ZSWIM7	protein_coding	zinc finger S		17	15976560	15999717
ENSG0000027359	-0.161449442	0.505	0.66	AL731571.1	lncRNA	novel transc		10	124996064	125001491
ENSG0000027520	0.308404828	0.506	0.66	NA	NA	NA	NA	NA	NA	
ENSG0000017129	0.102026169	0.506	0.66	ZNF440	protein_coding	zinc finger p		19	11814273	11835216
ENSG0000027787	-0.364066689	0.506	0.66	NA	NA	NA	NA	NA	NA	
ENSG0000023173	0.523747824	0.506	0.66	AC010976.1	lncRNA	novel transc		2	127455394	127514623
ENSG0000020427	-0.129407485	0.506	0.66	SPIN3	protein_coding	spindlin fam	X		56818298	56995827
ENSG0000017976	-0.5408747	0.506	0.66	PIPOX	protein_coding	pipecolic ac		17	28950513	29057216
ENSG0000011213	0.078436762	0.506	0.66	RNF8	protein_coding	ring finger p		6	37353979	37394734
ENSG0000018189	0.167523651	0.506	0.66	ZNF329	protein_coding	zinc finger p		19	58126248	58155110
ENSG0000026943	0.424633689	0.506	0.66	AC010618.3	lncRNA	novel transc		19	17488990	17511889
ENSG0000027632	1.418854524	0.506	0.66	NA	NA	NA	NA	NA	NA	
ENSG0000016240	0.068220097	0.507	0.66	NOL9	protein_coding	nucleolar pr		1	6521347	6554513
ENSG0000010555	0.12335955	0.507	0.66	BCAT2	protein_coding	branched ch		19	48795062	48811029
ENSG0000025505	0.709531082	0.507	0.66	FAM66D	lncRNA	family with s		8	12115767	12177550
ENSG0000016797	-0.0236154	0.507	0.66	SRRM2	protein_coding	serine/argin		16	2752626	2772538
ENSG0000011331	0.056289631	0.507	0.66	TTC1	protein_coding	tetratricope		5	160009113	160065543
ENSG0000010020	0.021829748	0.507	0.66	DDX17	protein_coding	DEAD-box h		22	38483438	38507660
ENSG0000010422	-0.112446117	0.508	0.66	BRF2	protein_coding	BRF2 RNA p		8	37843268	37849861
ENSG0000026023	0.292030477	0.508	0.66	ZNRD2-AS	lncRNA	ZNRD2 antis		11	65568482	65570423
ENSG0000022058	1.274421057	0.508	0.66	RPL35P2	processed_pse	ribosomal p		6	34263311	34263673
ENSG0000023822	-0.633911481	0.508	0.66	OR7E7P	unprocessed_p	olfactory rec		7	97946987	97947998
ENSG0000026968	-0.458584241	0.508	0.66	AC008982.2	lncRNA	novel transc		19	38844729	38845499
ENSG0000027330	0.305116147	0.508	0.66	AC018690.1	lncRNA	novel transc		2	99405218	99405843
ENSG0000027661	-0.085525835	0.508	0.66	RAD17	protein_coding	RAD17 chec	CHR_HSCHR		69369307	69415042
ENSG0000016697	0.115346721	0.508	0.66	AKTIP	protein_coding	AKT interact		16	53491040	53504411
ENSG0000019744	-0.073277146	0.509	0.66	OGDHL	protein_coding	oxoglutarate		10	49734641	49762379
ENSG0000012300	-0.525111263	0.509	0.66	NME2P1	processed_pse	NME2 pseu		12	120282303	120282716
ENSG0000017145	-0.172731357	0.509	0.66	DSEL	protein_coding	dermatan su		18	67506587	67516720
ENSG0000019808	-0.374927932	0.509	0.66	NUP62CL	protein_coding	nucleoporin	X		107123427	107206433
ENSG0000019902	-1.262007974	0.509	0.66	MIR103A2	miRNA	microRNA 1		20	3917494	3917571
ENSG0000027505	0.415377506	0.509	0.66	ZNF66	protein_coding	zinc finger p	CHR_HSCHR		20776304	20807420
ENSG0000027642	0.854530751	0.509	0.66	AC012314.1	protein_coding	pre-mRNA	CHR_HSCHR		54115536	54131896

ENSG0000013543	-0.465190975	0.51	0.66	FAM186B	protein_coding	family with s	12	49582885	49605639
ENSG0000020649	-0.275707562	0.51	0.66	TRIM39	protein_coding	tripartite mo	CHR_HSCHR	30315961	30333218
ENSG0000020508	-0.140974325	0.51	0.66	TMEM231	protein_coding	transmembr	16	75536741	75556268
ENSG0000022812	-0.885839633	0.51	0.66	FALEC	lncRNA	focally ampl	1	150515757	150518032
ENSG0000024117	-0.385822404	0.51	0.66	AP001992.1	transcribed_pr	ribosomal p	11	74876286	74920022
ENSG0000024005	0.371345115	0.51	0.66	LY6G5B	protein_coding	lymphocyte	6	31670167	31673776
ENSG0000022592	-0.056053839	0.511	0.66	NOL7	protein_coding	nucleolar pr	6	13615335	13632739
ENSG0000024367	-0.189603795	0.511	0.66	AC018638.5	processed_pse	novel pseuc	7	128653969	128654722
ENSG0000018490	-0.690630572	0.511	0.66	TCEAL2	protein_coding	transcription	X	102125679	102140426
ENSG0000023720	-0.900882451	0.511	0.66	ZBTB40-IT1	lncRNA	ZBTB40 intr	1	22517474	22519708
ENSG0000024016	1.036906765	0.511	0.66	AC087385.1	processed_pse	ribosomal p	15	60390371	60390682
ENSG0000010943	-0.060608366	0.511	0.66	TBC1D9	protein_coding	TBC1 doma	4	140620782	140756385
ENSG0000019806	-0.136141294	0.511	0.66	NPIPB13	protein_coding	nuclear pore	16	30222937	30254510
ENSG0000024514	0.194641869	0.512	0.66	RNF139-AS	lncRNA	RNF139 ant	8	124462485	124474582
ENSG0000024433	0.370939766	0.512	0.66	AL138759.1	lncRNA	novel transc	10	94577439	94611238
ENSG0000022551	0.88544144	0.512	0.66	LINC01703	lncRNA	long interge	1	226083590	226090465
ENSG0000014287	-0.227209034	0.512	0.66	CCN1	protein_coding	cellular com	1	85580761	85584589
ENSG0000014225	0.143100882	0.512	0.66	GEMIN7	protein_coding	gem nuclea	19	45079195	45091518
ENSG0000027469	-0.594259147	0.512	0.67	NA	NA	NA	NA	NA	NA
ENSG0000015045	-0.164385993	0.513	0.67	TIRAP	protein_coding	TIR domain	11	126283065	126298845
ENSG0000024949	-0.547255162	0.513	0.67	AC114956.2	lncRNA	novel transc	5	43483913	43509356
ENSG0000013969	-0.03533138	0.513	0.67	SBNO1	protein_coding	strawberry r	12	123289109	123364847
ENSG0000026122	-0.514664919	0.513	0.67	AC103706.1	lncRNA	novel transc	8	133573183	133573861
ENSG0000027660	0.764520355	0.513	0.67	AL109614.1	lncRNA	novel transc	20	37526642	37527060
ENSG0000021503	-0.099744022	0.513	0.67	CD27-AS1	lncRNA	CD27 antise	12	6439001	6451567
ENSG0000018899	0.476985998	0.513	0.67	LIPI	protein_coding	lipase I [Sou	21	14108813	14210891
ENSG0000027389	0.337944738	0.513	0.67	AL731566.2	lncRNA	novel transc	10	121965764	121967700
ENSG0000020797	1.257724934	0.513	0.67	MIR589	miRNA	microRNA 5	7	5495819	5495917
ENSG0000018038	0.443622856	0.513	0.67	EMC3-AS1	transcribed_un	EMC3 antise	3	9986893	10006990
ENSG0000019832	-0.116721808	0.514	0.67	PHETA1	protein_coding	PH domain	12	111360651	111369121
ENSG0000022678	0.55210131	0.514	0.67	PGAM4	protein_coding	phosphogly	X	77967949	77969638
ENSG0000026384	-1.190128077	0.514	0.67	NA	NA	NA	NA	NA	NA
ENSG0000023017	0.355973576	0.514	0.67	NA	NA	NA	NA	NA	NA
ENSG0000023750	-0.399784113	0.514	0.67	RPSAP15	processed_pse	ribosomal p	X	87703343	87704203
ENSG0000027887	-1.016468875	0.515	0.67	AC145207.9	TEC	TEC	17	81862778	81863102
ENSG0000014490	0.06829261	0.515	0.67	OSBPL11	protein_coding	oxysterol bi	3	125528858	125595497
ENSG0000024943	-0.375092308	0.515	0.67	NA	NA	NA	NA	NA	NA
ENSG0000011566	0.089153295	0.515	0.67	STK16	protein_coding	serine/threo	2	219245455	219250337
ENSG0000014149	-0.105923357	0.515	0.67	WRAP53	protein_coding	WD repeat c	17	7686071	7703502
ENSG0000023326	1.020498419	0.515	0.67	HMGB1P31	processed_pse	high mobilit	2	54051334	54051760
ENSG0000025355	-0.718792887	0.515	0.67	OSGEPL1-A	lncRNA	OSGEPL1 ar	2	189762704	189765556
ENSG0000005469	-0.149395882	0.515	0.67	PLEKHH1	protein_coding	pleckstrin ho	14	67533290	67589612
ENSG0000010910	0.201463745	0.516	0.67	UNC119	protein_coding	unc-119 lipi	17	28546708	28552631
ENSG0000025914	-0.73345462	0.516	0.67	AC005476.2	lncRNA	novel transc	14	71292729	71321882
ENSG0000022426	0.092464019	0.516	0.67	VARS1	protein_coding	valyl-tRNA s	CHR_HSCHR	31853814	31872249
ENSG0000025099	-0.836794476	0.516	0.67	AC136604.2	lncRNA	novel transc	5	179657762	179665136
ENSG0000011166	-0.204131699	0.517	0.67	GNB3	protein_coding	G protein su	12	6839954	6847393
ENSG0000018132	-0.44775244	0.517	0.67	NME9	protein_coding	NME/NM23	3	138261437	138329886
ENSG0000027115	0.611119009	0.517	0.67	AC016737.1	lncRNA	novel transc	2	173968351	173969418
ENSG0000001119	0.140165781	0.517	0.67	ABHD5	protein_coding	abhydrolase	3	43690108	43734371
ENSG0000010922	-0.098581488	0.517	0.67	CHIC2	protein_coding	cysteine rich	4	54009789	54064605
ENSG0000013184	-0.053330858	0.517	0.67	MCCC2	protein_coding	methylcrotc	5	71587288	71658706
ENSG0000027345	0.836313568	0.517	0.67	AC112503.2	lncRNA	novel transc	3	123277353	123277904
ENSG0000010536	-0.07208203	0.517	0.67	MRPL4	protein_coding	mitochondr	19	10251901	10260055
ENSG0000027842	0.682242615	0.517	0.67	LENG1	protein_coding	leukocyte re	CHR_HSCHR	54156127	54160192
ENSG0000027294	0.951493224	0.517	0.67	U62317.2	lncRNA	novel transc	22	50597152	50597599
ENSG0000025158	-0.513217203	0.517	0.67	LINC02482	lncRNA	long interge	4	6648695	6673965
ENSG0000014137	0.095869776	0.517	0.67	BCAS3	protein_coding	BCAS3 micr	17	60677453	61392838

ENSG0000027101	1.807464895	0.517	0.67	AC013244.1	processed_pse	mitochondr	12	50756892	50757433
ENSG0000027823	0.531127911	0.517	0.67	AL359513.1	lncRNA	novel transc	13	52454775	52455331
ENSG0000023178	-0.684732529	0.518	0.67	PIK3CD-AS	lncRNA	PIK3CD anti	1	9672426	9687555
ENSG0000022195	-0.246179012	0.518	0.67	SLC12A8	protein_coding	solute carrie	3	125082636	125212864
ENSG0000016272	0.157222643	0.518	0.67	IGSF8	protein_coding	immunoglol	1	160091340	160098943
ENSG0000026754	1.140345007	0.518	0.67	MTCO2P2	processed_pse	MT-CO2 ps	18	47853233	47853457
ENSG0000024112	0.998951879	0.518	0.67	HMGN1P8	processed_pse	high mobilit	3	167694355	167694642
ENSG0000017815	-0.310743424	0.518	0.67	ZNF114	protein_coding	zinc finger p	19	48270081	48287608
ENSG0000025450	0.732004218	0.518	0.67	AP000866.3	processed_pse	endothelial	11	124791228	124791660
ENSG0000023254	-0.939792877	0.519	0.67	AC027644.1	unprocessed_p	pseudogene	7	66848496	66858136
ENSG0000024832	-0.595007303	0.519	0.67	LUCAT1	lncRNA	lung cancer	5	91054834	91314547
ENSG0000026693	0.425229114	0.519	0.67	AC011442.1	lncRNA	novel transc	19	11010917	11016011
ENSG0000022921	-0.057532733	0.519	0.67	HLA-A	protein_coding	major histoc	CHR_HSCHR	29881755	30002076
ENSG0000000628	-0.096013648	0.519	0.67	SPATA20	protein_coding	spermatoge	17	50543058	50555852
ENSG0000014335	-0.106032697	0.519	0.67	LYPLAL1	protein_coding	lysophosph	1	219173869	219212865
ENSG0000025265	-1.374979274	0.52	0.67	Y_RNA	misc_RNA	Y RNA [Sou	11	43331261	43331356
ENSG0000027692	0.507393092	0.519	0.67	NA	NA	NA	NA	NA	NA
ENSG0000022516	0.77570442	0.52	0.67	LINC00618	lncRNA	long interge	14	96931367	96945394
ENSG0000023626	0.851248652	0.52	0.67	NA	NA	NA	NA	NA	NA
ENSG0000021301	-0.117820166	0.52	0.67	ZNF580	protein_coding	zinc finger p	19	55635016	55643470
ENSG0000013904	0.294193084	0.52	0.67	B4GALNT3	protein_coding	beta-1,4-N	12	459939	563509
ENSG0000023062	0.715710467	0.52	0.67	AC011005.1	protein_coding	novel protei	7	129126518	129130793
ENSG0000014445	0.135083539	0.52	0.67	SUMF1	protein_coding	sulfatase mo	3	3700814	4467273
ENSG0000020632	0.149453317	0.52	0.67	AGPAT1	protein_coding	1-acylglycer	CHR_HSCHR	32125820	32135704
ENSG0000015774	0.039962355	0.521	0.67	UBN2	protein_coding	ubinuclein 2	7	139230356	139308236
ENSG0000022883	0.713610588	0.521	0.67	PIK3IP1-DT	lncRNA	PIK3IP1 dive	22	31292499	31338021
ENSG0000026948	-0.412085338	0.521	0.67	AL589765.6	lncRNA	novel transc	1	151798054	151798602
ENSG0000025435	0.645416511	0.521	0.67	AC100854.1	processed_pse	chromosom	8	78723796	78724136
ENSG0000008750	0.057643636	0.521	0.67	ERGIC2	protein_coding	ERGIC and g	12	29337352	29381189
ENSG0000023643	0.325341666	0.521	0.67	MFF-DT	lncRNA	MFF diverge	2	227221052	227325711
ENSG0000027661	-0.993197431	0.521	0.67	NA	NA	NA	NA	NA	NA
ENSG0000018641	-0.073871475	0.521	0.67	NKRF	protein_coding	NFKB repres	X	119588337	119606443
ENSG0000020655	-0.587600265	0.521	0.67	ZCWPW2	protein_coding	zinc finger C	3	28348721	28538122
ENSG0000022495	0.402095466	0.521	0.67	NA	NA	NA	NA	NA	NA
ENSG0000025949	-0.084005213	0.521	0.67	MRPL46	protein_coding	mitochondr	15	88459477	88467390
ENSG0000021444	-1.576820105	0.521	0.67	FAM187A	protein_coding	family with s	17	44899712	44905390
ENSG0000026373	0.369905452	0.521	0.67	AC145207.5	lncRNA	novel transc	17	81878425	81881106
ENSG0000010385	-0.057172893	0.521	0.67	CD276	protein_coding	CD276 mole	15	73683966	73714514
ENSG0000018290	-0.450684062	0.522	0.67	SLC25A18	protein_coding	solute carrie	22	17563450	17590995
ENSG0000009998	0.361892999	0.522	0.67	GSTT2	polymorphic_p	glutathione	22	23980058	23983915
ENSG0000016902	0.097673559	0.522	0.67	ATP5ME	protein_coding	ATP synthas	4	672436	674330
ENSG0000019658	0.234684583	0.522	0.67	AJAP1	protein_coding	adherens ju	1	4654609	4792534
ENSG0000015532	-0.089192854	0.522	0.67	ZCCHC10	protein_coding	zinc finger C	5	132996985	133026604
ENSG0000020648	0.099538846	0.523	0.67	MDC1	protein_coding	mediator of	CHR_HSCHR	30689327	30707408
ENSG0000025733	0.286520714	0.523	0.67	AC068888.1	lncRNA	novel transc	12	53012884	53054450
ENSG0000020576	-0.040113373	0.523	0.67	C5orf51	protein_coding	chromosom	5	41904188	41921636
ENSG0000023159	-0.746557507	0.523	0.67	NA	NA	NA	NA	NA	NA
ENSG0000027492	-0.23316549	0.523	0.67	ZKSCAN2-I	lncRNA	ZKSCAN2 d	16	25257952	25261066
ENSG0000023490	-1.068786585	0.523	0.67	AC007879.3	lncRNA	novel transc	2	207186717	207236066
ENSG0000022862	-0.11615416	0.524	0.67	ATF6B	protein_coding	activating tr	CHR_HSCHR	32191691	32204680
ENSG0000027862	0.710382096	0.524	0.68	AC005962.1	lncRNA	novel transc	17	58202352	58203003
ENSG0000023073	0.429787498	0.524	0.68	AC016949.1	lncRNA	novel transc	3	196912646	196914579
ENSG0000023331	-0.158492196	0.524	0.68	PPP1R11	protein_coding	protein pho	CHR_HSCHR	30059208	30062832
ENSG0000025032	0.552981479	0.524	0.68	AC104596.1	lncRNA	novel transc	4	142933195	143184861
ENSG0000015755	0.051593201	0.524	0.68	ETS2	protein_coding	ETS proto-c	21	38805183	38824955
ENSG0000023202	-0.470570059	0.524	0.68	LSM12P1	processed_pse	LSM12 pseu	8	35525176	35525763
ENSG0000007814	0.075199733	0.525	0.68	PIK3C3	protein_coding	phosphatidy	18	41955234	42087830
ENSG0000023583	0.908813835	0.525	0.68	AC073333.1	lncRNA	novel transc	7	16695871	16719898

ENSG0000023462	0.609510844	0.525	0.68	WDR82P1	processed_pse	WD repeat		3	94937086	94938024
ENSG0000024411	0.460388544	0.525	0.68	PDCL3P4	processed_pse	PDCL3 pseu		3	101712472	101713191
ENSG0000027414	0.885881814	0.525	0.68	AC012314.3	protein_coding	pre-mRNA	CHR_HSCHR		54115536	54131896
ENSG0000016313	-0.334979235	0.526	0.68	MSX1	protein_coding	msh homeo		4	4859665	4863936
ENSG0000027282	-0.309340296	0.526	0.68	U62317.1	lncRNA	novel transc		22	50523926	50524780
ENSG0000027691	0.716418351	0.526	0.68	AL442125.2	lncRNA	novel transc		13	113511747	113514473
ENSG0000016846	0.108537882	0.526	0.68	ATF6B	protein_coding	activating tr	CHR_HSCHR		32091037	32104024
ENSG0000024949	0.51569734	0.526	0.68	ANKRD20A	unprocessed_p	ankyrin repe		21	14064325	14069087
ENSG0000027648	-0.670156998	0.526	0.68	AC008735.4	lncRNA	novel transc		19	55612490	55613097
ENSG0000027952	0.323921522	0.526	0.68	AC008764.8	lncRNA	novel transc		19	16572624	16575340
ENSG0000017842	0.076020658	0.526	0.68	NT5DC1	protein_coding	5'-nucleotic		6	116100851	116249497
ENSG0000023468	0.190749962	0.527	0.68	SDCBP2-AS	lncRNA	SDCBP2 ant		20	1325405	1378735
ENSG0000017061	-0.098546352	0.527	0.68	COMMD5	protein_coding	COMM dom		8	144841042	144853736
ENSG0000010139	0.076561713	0.527	0.68	CDK5RAP1	protein_coding	CDK5 regul		20	33358839	33401561
ENSG0000013144	-0.06607706	0.527	0.68	MGAT1	protein_coding	alpha-1,3-m		5	180784780	180815652
ENSG0000025639	-0.523131822	0.527	0.68	RPL41P5	processed_pse	ribosomal p		12	93083598	93083675
ENSG0000020585	-0.318655141	0.527	0.68	RFPL3S	lncRNA	RFPL3 antis		22	32359886	32382106
ENSG0000016387	-0.220816349	0.528	0.68	ZC3H12A	protein_coding	zinc finger C		1	37474580	37484377
ENSG0000018583	-1.050448768	0.527	0.68	HDHD5-AS	lncRNA	HDHD5 anti		22	17159384	17165445
ENSG0000025535	-0.377752394	0.528	0.68	NA	NA	NA	NA	NA	NA	
ENSG0000017746	0.051306633	0.528	0.68	NR2C2	protein_coding	nuclear rece		3	14947584	15053600
ENSG0000018858	0.29042779	0.528	0.68	PAQR9	protein_coding	progestin ar		3	142949164	142963674
ENSG0000023264	0.661151867	0.528	0.68	LINC01431	lncRNA	long interge		20	23356594	23358116
ENSG0000027888	0.883756919	0.528	0.68	NA	NA	NA	NA	NA	NA	
ENSG0000013405	-0.087399596	0.528	0.68	MRPS36	protein_coding	mitochondr		5	69217760	69230158
ENSG0000017384	0.155013833	0.528	0.68	PLK3	protein_coding	polo like kin		1	44800377	44805990
ENSG0000023144	-0.940146379	0.528	0.68	DST-AS1	lncRNA	DST antisen		6	56843928	56864078
ENSG0000014993	-0.109431845	0.528	0.68	TMEM219	protein_coding	transmembr		16	29940885	29973050
ENSG0000018320	0.274319303	0.528	0.68	GDPGP1	protein_coding	GDP-D-glu		15	90233808	90245811
ENSG0000014947	-0.066692488	0.529	0.68	KAT14	protein_coding	lysine acetyl		20	18138118	18188387
ENSG0000025093	-0.817389924	0.529	0.68	AC073475.1	lncRNA	novel transc		4	120066958	120564789
ENSG0000026132	-0.135751612	0.529	0.68	LINC01355	lncRNA	long interge		1	23281307	23287488
ENSG0000020456	-0.33534909	0.529	0.68	C6orf136	protein_coding	chromosom		6	30647039	30653210
ENSG0000014342	0.128552791	0.53	0.68	LSP1P4	transcribed_un	LSP1 pseud		2	91617160	91659972
ENSG0000022795	-0.440618377	0.53	0.68	TARID	lncRNA	TCF21 antis		6	133502252	133892802
ENSG0000013501	0.030771647	0.53	0.68	UBQLN1	protein_coding	ubiquilin 1 [		9	83659968	83707958
ENSG0000025562	0.225185464	0.53	0.68	C10orf88B	transcribed_pr	C10orf88B (		10	122879681	122898615
ENSG0000012677	0.025500733	0.53	0.68	KTN1	protein_coding	kinectin 1 [S		14	55559072	55701526
ENSG0000027191	0.581633403	0.53	0.68	AL139286.1	lncRNA	novel transc		1	35929720	35930115
ENSG0000010146	0.148198926	0.531	0.68	MAP1LC3A	protein_coding	microtubule		20	34546854	34560345
ENSG0000012869	0.049665807	0.53	0.68	ORMDL1	protein_coding	ORMDL sph		2	189770267	189784364
ENSG0000023793	0.434786627	0.53	0.68	CLIC4P3	processed_pse	chloride intr	X		41076999	41077750
ENSG0000025972	-0.511299655	0.531	0.68	AC090877.2	lncRNA	novel transc		15	32717270	32719007
ENSG0000026003	1.135789563	0.531	0.68	AC009090.1	lncRNA	novel transc		16	57247350	57248492
ENSG0000027817	0.240515974	0.531	0.68	GLIDR	lncRNA	glioblastom		9	39748514	39810097
ENSG0000027080	-0.740285612	0.531	0.68	AC022400.3	processed_pse	ataxin 1-like		10	73740538	73740823
ENSG0000027964	-0.26073981	0.531	0.68	AC020978.9	TEC	novel transc		16	68290087	68292790
ENSG0000023004	0.810989861	0.531	0.68	AK3P3	processed_pse	adenylate ki		7	23129178	23129841
ENSG0000025971	0.618315255	0.531	0.68	NA	NA	NA	NA	NA	NA	
ENSG0000023531	0.294441262	0.531	0.68	DUSP8P5	processed_pse	dual specific		10	73731824	73733638
ENSG0000016624	0.416381927	0.531	0.68	C16orf71	protein_coding	chromosom		16	4734344	4749396
ENSG0000022625	0.292477781	0.531	0.68	GTF2H2B	transcribed_un	general trar		5	70415352	70448015
ENSG0000026650	0.505178976	0.531	0.68	AC025198.1	processed_pse	ATG12 auto		17	73067870	73068288
ENSG0000023788	0.092721879	0.532	0.68	DDX39B	protein_coding	DExD-box h	CHR_HSCHR		31597175	31606140
ENSG0000018148	-0.43408753	0.532	0.68	RNF135	protein_coding	ring finger p		17	30970984	30999911
ENSG0000025394	-0.811968184	0.532	0.68	AC027117.2	lncRNA	novel transc		8	17801345	17861069
ENSG0000027825	0.573955647	0.532	0.68	LENG1	protein_coding	leukocyte re	CHR_HSCHR		54156127	54160192
ENSG0000025018	-0.412451944	0.532	0.68	EEF1A1P13	processed_pse	eukaryotic t		5	14651941	14653329

ENSG0000025932	0.639736969	0.533	0.68	AC090607.1	lncRNA	novel transc	15	78293286	78296049
ENSG0000027973	0.450934755	0.533	0.68	KMT5AP1	processed_pse	KMT5A pseu	13	18297554	18298733
ENSG0000018058	0.292904557	0.533	0.68	SRP9P1	processed_pse	signal recog	10	91807179	91807439
ENSG0000023101	-0.567301998	0.533	0.68	AL121672.1	lncRNA	novel transc	22	46055740	46058160
ENSG0000026563	1.420437533	0.533	0.68	BX547991.2	unprocessed_p	glutamate c	10	47563604	47569697
ENSG0000025246	1.212688749	0.533	0.68	RN7SKP70	misc_RNA	RN7SK pseu	5	178619728	178619998
ENSG0000027422	0.327031165	0.533	0.68	NA	NA	NA	NA	NA	NA
ENSG0000020477	0.733911467	0.533	0.68	CBWD4P	unprocessed_p	COBW dom	9	65287914	65323015
ENSG0000016591	-0.074457312	0.534	0.68	SLC39A13	protein_coding	solute carrie	11	47407132	47416496
ENSG0000026037	-0.79429441	0.534	0.68	AC026464.3	protein_coding	novel protei	16	69335091	69356306
ENSG0000026145	-1.476739566	0.534	0.68	AC002310.4	protein_coding	novel transc	16	30525923	30534852
ENSG0000026241	0.808641162	0.534	0.68	AC024361.1	lncRNA	novel transc	17	82745068	82745709
ENSG0000017850	-0.096855813	0.534	0.68	KLHL11	protein_coding	kelch like fa	17	41848518	41865423
ENSG0000016560	-0.047643977	0.534	0.68	NUDT5	protein_coding	nudix hydro	10	12165330	12196144
ENSG0000012079	0.066349628	0.534	0.68	NR2C1	protein_coding	nuclear rece	12	95020229	95073628
ENSG0000026806	-0.601559635	0.534	0.68	NAPA-AS1	lncRNA	NAPA antise	19	47484282	47501597
ENSG0000027608	-1.049682524	0.534	0.68	AL137060.6	misc_RNA		13	50082295	50082484
ENSG0000016349	-0.729987554	0.534	0.68	CRYBA2	protein_coding	crystallin be	2	218990189	218993422
ENSG0000025046	-0.260662864	0.535	0.68	AC122718.1	processed_pse	family with s	5	61375112	61375855
ENSG0000018389	-0.130539902	0.535	0.68	TTC32	protein_coding	tetratricope	2	19896631	19901983
ENSG0000020518	-0.040595499	0.535	0.69	ZBTB10	protein_coding	zinc finger a	8	80485619	80526265
ENSG0000013808	-0.041085397	0.535	0.69	ATRAID	protein_coding	all-trans ret	2	27212027	27217178
ENSG0000013916	0.270786673	0.536	0.69	ETFBKMT	protein_coding	electron tra	12	31647160	31673114
ENSG0000023346	-0.35429293	0.536	0.69	AL445524.1	lncRNA	novel transc	1	231520729	231528618
ENSG0000011228	0.05032921	0.536	0.69	MED23	protein_coding	mediator cc	6	131573966	131628242
ENSG0000025924	-0.868438524	0.536	0.69	AC048382.2	lncRNA	novel transc	15	84513241	84526949
ENSG0000014466	0.121667301	0.536	0.69	ITGA9	protein_coding	integrin sub	3	37452115	37823507
ENSG0000026233	0.474668018	0.537	0.69	HNRNPA1F	processed_pse	heterogene	17	2306761	2307715
ENSG0000027316	0.395791886	0.537	0.69	AL133215.2	lncRNA	novel transc	10	101060029	101061005
ENSG0000027861	0.25497369	0.537	0.69	MRM1	protein_coding	mitochondr	17	36601583	36608964
ENSG0000007931	-0.054505773	0.537	0.69	REXO1	protein_coding	RNA exonuc	19	1815248	1848483
ENSG0000026990	0.901130944	0.537	0.69	AC010531.5	lncRNA	novel transc	16	87362536	87367476
ENSG0000016429	-0.027961752	0.537	0.69	RHOBTB3	protein_coding	Rho related	5	95713522	95824383
ENSG0000016296	-0.055673184	0.537	0.69	DPY30	protein_coding	dpy-30 hist	2	31867809	32039805
ENSG0000017710	0.097689304	0.538	0.69	RHOG	protein_coding	ras homolog	11	3826978	3840959
ENSG0000027089	-0.71786669	0.538	0.69	NA	NA	NA	NA	NA	NA
ENSG0000018524	0.408110647	0.538	0.69	GP1BA	protein_coding	glycoprotei	17	4932277	4935023
ENSG0000025991	0.380617436	0.538	0.69	NDUFA5P1	processed_pse	NADH:ubiqu	16	47598654	47598996
ENSG0000018756	0.606802121	0.538	0.69	NHLRC1	protein_coding	NHL repeat	6	18120440	18122677
ENSG0000018822	-0.930872575	0.538	0.69	AD000671.1	protein_coding	novel protei	19	35745678	35754519
ENSG0000023478	-0.711923685	0.538	0.69	AL590369.1	lncRNA	novel transc	9	129640476	129641282
ENSG0000011479	0.21353275	0.539	0.69	ARHGEF26	protein_coding	Rho guanin	3	154121003	154257827
ENSG0000018443	-0.037397428	0.539	0.69	COPB2	protein_coding	COPI coat c	3	139355600	139389680
ENSG0000010352	-0.266731054	0.539	0.69	SYT17	protein_coding	synaptotagr	16	19167971	19268332
ENSG0000010598	-0.195336827	0.539	0.69	RNF32	protein_coding	ring finger p	7	156640281	156677130
ENSG0000010639	-0.077501468	0.539	0.69	C1GALT1	protein_coding	core 1 synth	7	7156934	7248616
ENSG0000011160	0.045082431	0.539	0.69	TIMELESS	protein_coding	timeless circ	12	56416363	56449426
ENSG0000020234	1.344232591	0.539	0.69	AL078581.1	snoRNA		6	149594625	149594759
ENSG0000020373	-0.32298022	0.539	0.69	AL645568.1	lncRNA	novel transc	1	173417793	173489407
ENSG0000023535	1.344232591	0.539	0.69	RPS29P16	processed_pse	ribosomal p	7	103348601	103348771
ENSG0000025438	1.028205798	0.539	0.69	DUTP2	processed_pse	deoxyuridin	8	123352181	123352643
ENSG0000027759	1.344232591	0.539	0.69	MIR7845	miRNA	microRNA 7	2	207166400	207166498
ENSG0000017089	0.256983894	0.539	0.69	TSEN34	protein_coding	tRNA splicir	19	54189938	54194536
ENSG0000018405	-0.517144462	0.539	0.69	TBX1	protein_coding	T-box trans	22	19756703	19783593
ENSG0000020523	0.266587438	0.54	0.69	SPDYE2	protein_coding	speedy/RIN	7	102551232	102562308
ENSG0000009287	0.092780148	0.54	0.69	RFFL	protein_coding	ring finger a	17	35005990	35089319
ENSG0000026567	0.442822833	0.54	0.69	AC129510.1	lncRNA	novel transc	17	82153430	82154815
ENSG0000027104	0.962380386	0.54	0.69	AL390955.2	lncRNA	novel transc	6	157323964	157324477

ENSG0000027306	-0.252361321	0.541	0.69	AL355987.4	lncRNA	novel transc	9	136799223	136810042
ENSG0000023449	0.251316927	0.541	0.69	SP2-AS1	lncRNA	SP2 antisens	17	47897330	47941410
ENSG0000010451	-0.581927832	0.541	0.69	GSDMD	protein_coding	gasdermin D	8	143553207	143563062
ENSG0000016649	0.267260537	0.541	0.69	FAM86GP	unprocessed_p	family with s	11	3410352	3422496
ENSG0000014025	0.545036308	0.541	0.69	DUOXA1	protein_coding	dual oxidase	15	45117367	45129938
ENSG0000027580	0.336527431	0.541	0.69	AC145285.6	lncRNA	novel transc	16	28822431	28823969
ENSG0000008345	0.124934498	0.542	0.69	ITGAE	protein_coding	integrin sub	17	3714628	3801188
ENSG0000010089	-0.057008503	0.542	0.69	DCAF11	protein_coding	DDB1 and C	14	24114195	24125242
ENSG0000021295	0.904573546	0.542	0.69	BX005266.1	unprocessed_p	novel pseuc	9	62532397	62532853
ENSG0000024196	0.417311838	0.542	0.69	AC079447.1	protein_coding	novel protei	2	99141485	99322741
ENSG0000024228	0.431821965	0.542	0.69	BMS1P4-AG	lncRNA	BMS1P4-AC	10	73674295	73730466
ENSG0000027857	-0.081375889	0.542	0.69	ZNHIT3	protein_coding	zinc finger H	CHR_HSCHR	36487001	36499682
ENSG0000021399	-0.426021377	0.542	0.69	TM6SF2	protein_coding	transmembr	19	19264364	19273391
ENSG0000017361	0.0798788	0.542	0.69	SCAI	protein_coding	suppressor c	9	124942608	125143528
ENSG0000024877	1.087127803	0.542	0.69	AC108727.1	lncRNA	novel transc	3	140972744	140973255
ENSG0000017382	0.391979843	0.542	0.69	TIGD3	protein_coding	tigger trans	11	65354751	65357613
ENSG0000025739	-1.117184195	0.543	0.69	AC023055.1	protein_coding	novel protei	12	55757275	55827546
ENSG0000013395	0.061791184	0.543	0.69	UNC79	protein_coding	unc-79 hom	14	93333219	93707876
ENSG0000012544	0.052813627	0.543	0.69	MRPS7	protein_coding	mitochondr	17	75261674	75266376
ENSG0000012459	-0.360153056	0.543	0.69	AL365205.1	protein_coding	novel protei	6	41780349	41790141
ENSG0000023653	-0.680546705	0.543	0.69	AC003986.1	lncRNA	novel transc	7	19119933	19121916
ENSG0000027092	0.771895822	0.543	0.69	TAS2R6P	processed_pse	taste 2 rece	7	141787815	141788640
ENSG0000022700	0.683829579	0.543	0.69	AL136988.2	lncRNA	novel transc	1	230258694	230272100
ENSG0000026540	1.180996357	0.544	0.69	U6	snRNA	U6 spliceos	CHR_HSCHR	31744327	31744429
ENSG0000027573	0.636564674	0.544	0.69	AC010538.1	lncRNA	novel transc	16	89671568	89685304
ENSG0000024354	-1.187647194	0.544	0.69	RN7SL104P	misc_RNA	RNA, 7SL, cy	2	15950689	15950981
ENSG0000022671	0.282908212	0.544	0.69	FAM66C	lncRNA	family with s	12	8180209	8216151
ENSG0000027823	1.326735234	0.544	0.69	AC005076.2	misc_RNA		7	87184849	87185020
ENSG0000015816	0.166392882	0.544	0.69	DZIP1L	protein_coding	DAZ interac	3	138061990	138115818
ENSG0000025321	0.870337949	0.544	0.69	AC040970.1	lncRNA	novel transc	8	141124717	141130064
ENSG0000024424	-1.007059929	0.544	0.69	AC133134.1	processed_pse	ribosomal p	5	108593609	108593967
ENSG0000017036	0.088989938	0.544	0.69	SETMAR	protein_coding	SET domain	3	4303304	4317567
ENSG0000027576	0.830632881	0.544	0.69	AC068792.1	lncRNA	novel transc	12	31443792	31444208
ENSG0000011594	-0.035648303	0.544	0.69	COX7A2L	protein_coding	cytochrome	2	42333546	42425088
ENSG0000022588	-0.45496153	0.545	0.69	AC012368.1	lncRNA	novel transc	2	64143239	64252859
ENSG0000007211	-0.039469807	0.545	0.69	ACTN1	protein_coding	actinin alpha	14	68874128	68979440
ENSG0000014430	0.066829759	0.545	0.69	SCRN3	protein_coding	secernin 3 [S	2	174395730	174429575
ENSG0000020608	0.449360033	0.545	0.69	NA	NA	NA	NA	NA	NA
ENSG0000027541	0.975262113	0.545	0.69	AC002553.2	lncRNA	novel transc	17	16023323	16023653
ENSG0000011010	0.036560974	0.545	0.69	PRPF19	protein_coding	pre-mRNA	11	60890547	60906585
ENSG0000023238	-1.022439118	0.546	0.69	SKA2P1	processed_pse	spindle and	9	122761844	122762233
ENSG0000022536	-0.331573408	0.546	0.69	PPP1R26-A	lncRNA	PPP1R26 an	9	135462727	135480777
ENSG0000027374	0.147112613	0.546	0.69	AL592183.1	protein_coding		GL000219.1	54224	83311
ENSG0000015278	-0.077292525	0.546	0.69	PANK1	protein_coding	pantothena	10	89579497	89645572
ENSG0000022766	-1.00842589	0.546	0.69	CYCSP24	processed_pse	CYCS pseuc	9	94038030	94038345
ENSG0000024037	0.637654775	0.546	0.69	SEC62-AS1	lncRNA	SEC62 antis	3	169978536	169985715
ENSG0000001329	0.382492265	0.547	0.69	CLDN11	protein_coding	claudin 11 [	3	170418868	170454733
ENSG0000027904	-0.843721435	0.547	0.69	AC007787.2	TEC	TEC	19	13081966	13082564
ENSG0000028015	0.363419803	0.547	0.69	AC133065.1	TEC	TEC	16	10933903	10936280
ENSG0000016828	-0.043454466	0.547	0.69	BMI1	protein_coding	BMI1 proto-	10	22321099	22331484
ENSG0000014712	0.67876358	0.547	0.69	RAB41	protein_coding	RAB41, mer	X	70282093	70285002
ENSG0000018375	-0.070488938	0.547	0.7	TBL3	protein_coding	transducin b	16	1972053	1982929
ENSG0000016236	0.043598642	0.547	0.7	CMPK1	protein_coding	cytidine/uric	1	47333797	47378839
ENSG0000026818	-0.561477621	0.547	0.7	AC005785.1	lncRNA	novel transc	19	15379076	15381194
ENSG0000016767	-0.041818433	0.547	0.7	HDGFL2	protein_coding	HDGF like 2	19	4472297	4502208
ENSG0000015463	-0.085957573	0.548	0.7	CXADR	protein_coding	CXADR Ig-li	21	17513043	17593579
ENSG0000020430	0.140730885	0.548	0.7	RNF5	protein_coding	ring finger p	6	32178405	32180793
ENSG0000014670	-0.447000215	0.548	0.7	SSC4D	protein_coding	scavenger re	7	76389334	76409697

ENSG0000024962	0.543241051	0.548	0.7	AC024560.2	processed_pse	protein pho	3	197634315	197635811
ENSG0000023187	0.69170795	0.548	0.7	IPO9-AS1	lncRNA	IPO9 antiser	1	201688259	201829559
ENSG0000022662	-0.47754486	0.548	0.7	SHANK2-A	lncRNA	SHANK2 an	11	70626441	70635658
ENSG0000025965	-0.65905922	0.548	0.7	PIGHP1	processed_pse	phosphatidy	15	59676757	59677321
ENSG0000026598	0.790710214	0.548	0.7	AC103810.3	unprocessed_p	Rho GTPase	17	64778842	64778937
ENSG0000020530	0.052647683	0.548	0.7	SNX2	protein_coding	sorting nexi	5	122775079	122834543
ENSG0000020675	0.783558777	0.549	0.7	SNORD101	snoRNA	small nucleo	6	132815307	132815379
ENSG0000027594	0.46697876	0.549	0.7	AC139677.1	protein_coding	ADP-ribosyl	CHR_HSCHR	46812294	46875243
ENSG0000022656	-0.628028828	0.549	0.7	CYP4F26P	lncRNA	cytochrome	9	33580695	33607874
ENSG0000015481	-0.099794698	0.55	0.7	DPH3	protein_coding	diphthamide	3	16257061	16264969
ENSG0000015988	0.179254947	0.55	0.7	ZNF222	protein_coding	zinc finger p	19	44025342	44033110
ENSG0000023274	-0.606786562	0.55	0.7	RHOQP2	processed_pse	ras homolog	2	131460999	131461577
ENSG0000025762	-0.054310138	0.55	0.7	PSMA3-AS	lncRNA	PSMA3 anti	14	58265365	58298134
ENSG0000026513	0.712941756	0.55	0.7	AC124283.4	processed_pse	ADP-ribosyl	17	82576616	82577391
ENSG0000023603	-0.665738583	0.55	0.7	NA	NA	NA	NA	NA	NA
ENSG0000014047	-0.070322173	0.55	0.7	LINS1	protein_coding	lines homol	15	100559369	100603230
ENSG0000007404	0.104423593	0.55	0.7	GLI2	protein_coding	GLI family z	2	120735623	120992653
ENSG0000013093	-0.038872688	0.55	0.7	UBE4B	protein_coding	ubiquitinatio	1	10032832	10181239
ENSG0000014415	0.173232345	0.55	0.7	SNRPA1P1	processed_pse	SNRPA1 pse	2	113657908	113658675
ENSG0000010532	-0.058671298	0.55	0.7	FZR1	protein_coding	fizzy and ce	19	3506273	3538330
ENSG0000013078	0.083292142	0.55	0.7	HIP1R	protein_coding	huntingtin in	12	122834453	122862961
ENSG0000027058	0.563065113	0.55	0.7	AC046185.2	processed_pse	family with s	17	63610772	63611184
ENSG0000026997	-0.333956126	0.55	0.7	NA	NA	NA	NA	NA	NA
ENSG0000011686	0.097372801	0.551	0.7	ADPRS	protein_coding	ADP-ribosyl	1	36088892	36093932
ENSG0000017709	-0.314690996	0.551	0.7	SCN4B	protein_coding	sodium volt	11	118133377	118152888
ENSG0000021483	0.348616239	0.551	0.7	UPF3AP2	transcribed_pr	UPF3A pseu	17	20375369	20376840
ENSG0000012399	-0.119224621	0.551	0.7	DNPEP	protein_coding	aspartyl ami	2	219373527	219400022
ENSG0000023073	-0.320862085	0.551	0.7	AL021937.1	lncRNA	novel transc	22	32376664	32384343
ENSG0000022860	-0.447018859	0.552	0.7	AL139011.1	lncRNA	novel transc	1	160261741	160263328
ENSG0000012309	-0.057220599	0.552	0.7	RASSF8	protein_coding	Ras associat	12	25959029	26079892
ENSG0000027001	0.239783939	0.552	0.7	AC232271.1	lncRNA	novel transc	X	49273054	49275768
ENSG0000025864	-0.276818715	0.552	0.7	HSPE1P2	processed_pse	heat shock p	14	63601509	63601814
ENSG0000025057	0.312936086	0.553	0.7	AL732372.3	unprocessed_p	novel pseuc	1	491225	493241
ENSG0000027010	-0.536730602	0.553	0.7	NA	NA	NA	NA	NA	NA
ENSG0000026894	-0.272211523	0.553	0.7	CKS1BP3	processed_pse	CDC28 prot	5	62512246	62512482
ENSG0000023231	-0.164578108	0.554	0.7	GPANK1	protein_coding	G-patch do	CHR_HSCHR	31737508	31742562
ENSG0000015474	-0.120421699	0.554	0.7	TSEN2	protein_coding	tRNA splicin	3	12484432	12539623
ENSG0000013055	-0.032761824	0.554	0.7	CAMSAP1	protein_coding	calmodulin	9	135808487	135907546
ENSG0000008537	0.050879825	0.554	0.7	PREP	protein_coding	prolyl endop	6	105273218	105454062
ENSG0000021018	-0.120217693	0.554	0.7	MT-TS2	Mt_tRNA	mitochondr	MT	12207	12265
ENSG0000028004	-0.126654989	0.554	0.7	AC104581.4	TEC	TEC	17	7858943	7866083
ENSG0000015638	0.11593167	0.554	0.7	ANKRD9	protein_coding	ankyrin repe	14	102501767	102509799
ENSG0000017659	0.089971551	0.554	0.7	B3GNT5	protein_coding	UDP-GlcNA	3	183253253	183298504
ENSG0000025249	0.938950839	0.555	0.7	RNU6-101	snRNA	RNA, U6 sm	6	75644084	75644177
ENSG0000022647	0.692253726	0.555	0.7	AC008013.1	transcribed_un	tetraspanin	12	30978308	31006010
ENSG0000010238	-0.127586068	0.555	0.7	ZDHHC15	protein_coding	zinc finger D	X	75368427	75523502
ENSG0000023649	0.318258126	0.555	0.7	LINC00896	lncRNA	long interge	22	20206397	20208524
ENSG0000016738	-0.132126522	0.556	0.7	ZNF180	protein_coding	zinc finger p	19	44474428	44500524
ENSG0000018222	0.138714802	0.556	0.7	CYB5D1	protein_coding	cytochrome	17	7857746	7862282
ENSG0000020641	-0.268753059	0.556	0.7	H1-10-AS1	lncRNA	H1-10 antis	3	129315392	129326225
ENSG0000016028	0.648219541	0.556	0.7	FTCD	protein_coding	formimidoyl	21	46136262	46155579
ENSG0000027207	-0.394413811	0.556	0.7	AC004492.1	lncRNA	novel transc	7	107192559	107193300
ENSG0000023550	-0.262264525	0.556	0.7	AC105942.1	lncRNA	novel transc	1	94927361	94963616
ENSG0000020145	0.768165047	0.557	0.7	SNORA55	snoRNA	small nucleo	1	39567374	39567508
ENSG0000007107	-0.220110648	0.557	0.7	MGAT4A	protein_coding	alpha-1,3-m	2	98619106	98731132
ENSG0000027887	0.424887289	0.557	0.7	AP000560.1	TEC	TEC	11	74830574	74832510
ENSG0000010787	-0.050235149	0.557	0.7	CUEDC2	protein_coding	CUE domain	10	102423245	102432584
ENSG0000016762	-0.122001635	0.557	0.7	ZNF526	protein_coding	zinc finger p	19	42220312	42228201

ENSG0000026016	0.80817306	0.557	0.7	AC011468.1	lncRNA	novel transc		19	52058490	52063703
ENSG0000010091	0.064227492	0.558	0.7	BRMS1L	protein_coding	BRMS1 like		14	35826338	35932325
ENSG0000015902	-0.036058056	0.558	0.7	EPB41	protein_coding	erythrocyte		1	28887091	29120046
ENSG0000006265	0.036163161	0.558	0.7	WAPL	protein_coding	WAPL cohe:		10	86435256	86521792
ENSG0000023682	0.113065486	0.558	0.7	BCYRN1	scRNA	brain cytopl		2	47335315	47335514
ENSG0000000282	0.075752715	0.558	0.7	MAD1L1	protein_coding	mitotic arre:		7	1815793	2233243
ENSG0000026087	-0.72071987	0.558	0.7	AC106820.5	lncRNA	novel transc		16	2456252	2459979
ENSG0000017491	-0.0866937	0.558	0.71	PTDSS2	protein_coding	phosphatidy		11	448268	491399
ENSG0000023610	-0.190041103	0.559	0.71	ZBTB22	protein_coding	zinc finger a		6	33314406	33317942
ENSG0000025504	0.634799619	0.558	0.71	AC109635.3	transcribed_un	septin 7 (SE		11	50279828	50298452
ENSG0000001062	0.226805191	0.559	0.71	LRRC23	protein_coding	leucine rich		12	6873569	6914241
ENSG0000009986	-0.062868925	0.559	0.71	PALM	protein_coding	paralemmin		19	708935	748329
ENSG0000017451	0.173025344	0.559	0.71	PELI3	protein_coding	pellino E3 u		11	66466327	66477337
ENSG0000026006	-0.354841544	0.559	0.71	NA	NA	NA	NA	NA	NA	NA
ENSG0000026265	-0.767895553	0.559	0.71	AC124283.3	lncRNA	novel transc		17	82578023	82578616
ENSG0000027227	0.70437953	0.559	0.71	AL031963.3	lncRNA	novel transc		6	3068045	3068894
ENSG0000027903	0.828125264	0.559	0.71	AC004232.2	TEC	TEC		16	3292879	3293403
ENSG0000028013	-0.506502337	0.56	0.71	AC240565.1	lncRNA	novel transc		17	88641	118578
ENSG0000010000	-0.224168463	0.56	0.71	SEC14L2	protein_coding	SEC14 like li		22	30396941	30425303
ENSG0000020181	1.309939423	0.56	0.71	RNU6-915P	snRNA	RNA, U6 sm		2	195670372	195670474
ENSG0000020669	1.309939423	0.56	0.71	RNU1-73P	snRNA	RNA, U1 sm		5	97175950	97176113
ENSG0000026057	-0.350326716	0.56	0.71	AC069224.1	lncRNA	novel transc		3	160753428	160755142
ENSG0000027617	0.83370794	0.56	0.71	AC244153.1	lncRNA	novel transc		17	38450394	38452444
ENSG0000001140	-0.032124847	0.56	0.71	PIK3C2A	protein_coding	phosphatidy		11	17077730	17207983
ENSG0000016719	-0.920865769	0.56	0.71	C16orf92	protein_coding	chromosom		16	30023334	30027736
ENSG0000027336	0.308558662	0.56	0.71	AC096586.2	lncRNA	novel transc		4	44693946	44694386
ENSG0000027454	-0.397489425	0.56	0.71	NA	NA	NA	NA	NA	NA	NA
ENSG0000024082	0.979926137	0.56	0.71	RN7SL23P	misc_RNA	RNA, 7SL, cy		11	61444149	61444423
ENSG0000027058	-0.273808281	0.561	0.71	AL158163.1	lncRNA	novel transc		10	110910596	110912244
ENSG0000020749	0.970755859	0.561	0.71	SNORA46	snoRNA	small nucleo		16	58548499	58548633
ENSG0000020499	-0.10127174	0.561	0.71	SPIRE2	protein_coding	spire type a		16	89818179	89871319
ENSG0000025280	1.28745005	0.561	0.71	NA	NA	NA	NA	NA	NA	NA
ENSG0000027321	-0.822344114	0.561	0.71	AC000068.2	lncRNA	novel transc		22	19456503	19456962
ENSG0000027670	1.28745005	0.561	0.71	NA	NA	NA	NA	NA	NA	NA
ENSG0000026739	0.615664649	0.562	0.71	AC036176.1	lncRNA	novel transc		18	63367328	63381629
ENSG0000017879	-0.368199878	0.562	0.71	RIIAD1	protein_coding	regulatory s		1	151710433	151729805
ENSG0000027303	-1.04309836	0.562	0.71	NA	NA	NA	NA	NA	NA	NA
ENSG0000009027	0.039434679	0.562	0.71	NUDC	protein_coding	nuclear distr		1	26900238	26946871
ENSG0000027815	-0.331164715	0.562	0.71	TSC22D1-A	lncRNA	TSC22D1 ar		13	44575893	44580432
ENSG0000023776	-0.080417201	0.562	0.71	FAM200B	protein_coding	family with :		4	15681662	15690447
ENSG0000027939	0.351943071	0.563	0.71	AL158801.5	processed_pse	ubiquitin-co		14	55229217	55229681
ENSG0000011652	0.06750607	0.563	0.71	SCAMP3	protein_coding	secretory ca		1	155255979	155262430
ENSG0000027006	0.796043901	0.563	0.71	AL356488.2	lncRNA	small Cajal I		1	109100193	109100619
ENSG0000014098	-0.019967985	0.563	0.71	RPS2	protein_coding	ribosomal p		16	1962058	1964841
ENSG0000013838	0.083373153	0.563	0.71	CARF	protein_coding	calcium resp		2	202912214	202988263
ENSG0000025889	-0.964633455	0.563	0.71	SCOCP1	processed_pse	short coiled		14	60158976	60159324
ENSG0000026382	0.229610021	0.563	0.71	AC112907.3	lncRNA	novel transc		3	186781780	186784179
ENSG0000022350	-1.079343022	0.564	0.71	AC114402.1	processed_pse	ribosomal p		1	203835585	203835912
ENSG0000020570	-0.174941011	0.564	0.71	LINC00634	protein_coding	long interge		22	41952174	41958933
ENSG0000027522	0.450548599	0.564	0.71	AL121906.2	lncRNA	novel transc		20	33655701	33656423
ENSG0000027861	-0.150095968	0.564	0.71	NAIP	protein_coding	NLR family a	CHR_HSCHR		69602550	69659159
ENSG0000018640	-0.367691477	0.564	0.71	CCDC30	protein_coding	coiled-coil c		1	42463221	42657190
ENSG0000016363	0.289347623	0.564	0.71	C4orf36	protein_coding	chromosom		4	86876205	86892422
ENSG0000011235	-0.112145605	0.564	0.71	PEX7	protein_coding	peroxisoma		6	136822564	136913934
ENSG0000025921	-0.541961613	0.564	0.71	AC103739.1	lncRNA	novel transc		15	90595840	90596447
ENSG0000003625	-0.033981392	0.565	0.71	CUL3	protein_coding	cullin 3 [Sou		2	224470150	224585397
ENSG0000016440	-0.048328843	0.565	0.71	SEPTIN8	protein_coding	septin 8 [So		5	132750819	132807241
ENSG0000024048	0.276779569	0.565	0.71	SETP14	processed_pse	SET pseudo		3	155987304	155988176

ENSG0000013495	0.039157256	0.565	0.71	ETS1	protein_coding	ETS proto-c	11	128458761	128587558
ENSG0000019886	0.91952749	0.565	0.71	MTND4LP3	processed_pse	MT-ND4L p	5	134928030	134928326
ENSG0000023319	0.517494382	0.565	0.71	RNF224	protein_coding	ring finger p	9	137227502	137229640
ENSG0000019778	-0.054613379	0.566	0.71	ATAD3A	protein_coding	ATPase fam	1	1512162	1534685
ENSG0000025513	-0.431591944	0.566	0.71	AP001972.3	lncRNA	novel transc	11	75206048	75241173
ENSG0000027755	-1.284856405	0.566	0.71	NA	NA	NA	NA	NA	NA
ENSG0000015205	0.169440745	0.566	0.71	AP1S3	protein_coding	adaptor rela	2	223751686	223838027
ENSG0000006924	-0.037412442	0.566	0.71	NUP133	protein_coding	nucleoporin	1	229440259	229508341
ENSG0000011963	-0.325774232	0.566	0.71	BBOF1	protein_coding	basal body	14	74019349	74082863
ENSG0000012878	0.073740055	0.566	0.71	PSMG2	protein_coding	proteasome	18	12658043	12725740
ENSG0000028002	-0.72157859	0.566	0.71	AC244517.1	lncRNA	novel transc	5	141136683	141245380
ENSG0000022685	0.276089589	0.567	0.71	ATP6V1G2	protein_coding	ATPase H+	CHR_HSCHR	31583922	31586304
ENSG0000024186	0.414615968	0.567	0.71	AL627309.5	lncRNA	novel transc	1	141474	173862
ENSG0000016678	0.06158486	0.567	0.71	SAAL1	protein_coding	serum amyl	11	18069935	18106087
ENSG0000025348	0.317137181	0.568	0.71	PCDHGA5	protein_coding	protocadhe	5	141364232	141512979
ENSG0000007475	0.047602871	0.568	0.71	ZZEF1	protein_coding	zinc finger Z	17	4004445	4143030
ENSG0000021202	-1.125375457	0.568	0.71	MIR374B	miRNA	microRNA 3	X	74218547	74218618
ENSG0000023705	-0.184163943	0.568	0.71	ZBTB22	protein_coding	zinc finger a	CHR_HSCHR	33484759	33488283
ENSG0000025392	0.779625608	0.568	0.71	AC091982.1	lncRNA	novel transc	5	151753992	151767247
ENSG0000020413	0.071239866	0.569	0.71	RUFY2	protein_coding	RUN and FY	10	68341107	68407294
ENSG0000010549	-0.11299451	0.569	0.71	ZNF175	protein_coding	zinc finger p	19	51571283	51592510
ENSG0000013544	0.029973208	0.569	0.71	CDK4	protein_coding	cyclin deper	12	57747727	57756013
ENSG0000015673	0.055515646	0.569	0.71	BAG4	protein_coding	BAG cochap	8	38176533	38213301
ENSG0000025809	0.870455533	0.569	0.71	ATXN2-AS	lncRNA	ATXN2 antis	12	111599498	111600256
ENSG0000026997	-0.504222847	0.57	0.71	AL359881.1	lncRNA	novel transc	1	7700704	7700970
ENSG0000027821	0.55173506	0.57	0.71	NA	NA	NA	NA	NA	NA
ENSG0000025353	-0.956403721	0.57	0.71	PCDHGA7	protein_coding	protocadhe	5	141382739	141512975
ENSG0000013806	-0.035158176	0.57	0.71	RAB1A	protein_coding	RAB1A, mer	2	65070696	65130331
ENSG0000024013	-0.463329067	0.57	0.71	ERICH6-AS	lncRNA	ERICH6 anti	3	150703564	150723005
ENSG0000026306	-1.032691893	0.57	0.71	NA	NA	NA	NA	NA	NA
ENSG0000006343	0.072161234	0.57	0.72	AHRR	protein_coding	aryl-hydroc	5	321759	435110
ENSG0000013305	0.068975059	0.57	0.72	PIK3C2B	protein_coding	phosphatidy	1	204422628	204494724
ENSG0000015949	0.246704166	0.571	0.72	RGL4	protein_coding	ral guanine	22	23688136	23699176
ENSG0000020431	-0.544427369	0.571	0.72	PRRT1	protein_coding	proline rich	6	32148359	32153083
ENSG0000027303	0.130938881	0.571	0.72	LINC02035	lncRNA	long interge	3	122886941	122892416
ENSG0000016078	0.095997936	0.571	0.72	PAQR6	protein_coding	progestin ar	1	156243320	156248117
ENSG0000027560	0.480026577	0.571	0.72	AC011330.2	lncRNA	novel transc	15	43642389	43643023
ENSG0000024812	-0.114941355	0.572	0.72	RRN3P1	transcribed_un	RRN3 pseuc	16	21796106	21820410
ENSG0000027742	-0.907658622	0.572	0.72	AC069234.5	lncRNA	novel transc	12	120703867	120704282
ENSG0000011359	0.053523101	0.572	0.72	PPWD1	protein_coding	peptidylprol	5	65563236	65587549
ENSG0000023134	-0.114051148	0.572	0.72	BEND3P1	transcribed_pr	BEN domair	10	50655967	50660472
ENSG0000013816	-0.178285457	0.572	0.72	CUZD1	protein_coding	CUB and zo	10	122832158	122846175
ENSG0000027850	-1.047640778	0.572	0.72	NA	NA	NA	NA	NA	NA
ENSG0000026161	1.042447649	0.572	0.72	SUB1P3	processed_pse	SUB1 pseud	16	4562814	4563128
ENSG0000013498	-0.037573445	0.573	0.72	WDR36	protein_coding	WD repeat	5	111091716	111130502
ENSG0000026938	-0.702927817	0.573	0.72	AC018755.3	transcribed_pr	putative ATI	19	51594561	51606150
ENSG0000016923	0.123170673	0.573	0.72	CA5B	protein_coding	carbonic an	X	15688830	15788411
ENSG0000025902	-0.861588727	0.573	0.72	TVP23C-CD	protein_coding	TVP23C-CD	17	15436021	15563561
ENSG0000022555	-0.10062125	0.573	0.72	PHF1	protein_coding	PHD finger	CHR_HSCHR	33550562	33556643
ENSG0000010767	-0.047250073	0.573	0.72	PLEKHA1	protein_coding	pleckstrin h	10	122374696	122442602
ENSG0000023309	0.297577361	0.573	0.72	CCDC144N	lncRNA	CCDC144NI	17	20868433	21002276
ENSG0000022525	0.471037016	0.573	0.72	ST13P6	processed_pse	ST13, Hsp70	8	133408006	133409107
ENSG0000023982	0.49836837	0.573	0.72	SUGT1P3	transcribed_un	SUGT1 pseu	13	40882577	40921774
ENSG0000013861	0.103945369	0.574	0.72	PARP16	protein_coding	poly(ADP-ri	15	65234460	65300618
ENSG0000019629	-0.064810665	0.574	0.72	NIF3L1	protein_coding	NGG1 intera	2	200889327	200903938
ENSG0000025982	-0.089920747	0.574	0.72	AC083843.2	lncRNA	novel transc	8	134792020	134798272
ENSG0000018501	0.443513427	0.574	0.72	CA13	protein_coding	carbonic an	8	85220587	85284073
ENSG0000017794	0.199996714	0.574	0.72	MAMDC4	protein_coding	MAM doma	9	136850943	136860799

ENSG0000024129	0.555868266	0.574	0.72	PPATP1	processed_pse	phosphoribo	3	87051192	87052738
ENSG0000024998	1.018198148	0.574	0.72	YWHAQP6	processed_pse	YWHAQ pse	3	141600276	141600579
ENSG0000026417	-0.578092183	0.574	0.72	MAGOH2P	transcribed_pr	mago homo	17	10716002	10716511
ENSG0000027483	1.018198148	0.574	0.72	AC110285.5	lncRNA	novel transc	17	81387415	81387766
ENSG0000023253	-0.231081637	0.575	0.72	KMT5AP2	processed_pse	KMT5A pseu	2	91747940	91748986
ENSG0000021398	0.395814523	0.575	0.72	AC078899.1	processed_pse	ARP2 actin-	19	20257720	20259418
ENSG0000026870	0.759657597	0.575	0.72	BNIP3P26	processed_pse	BCL2 intera	19	21521343	21521896
ENSG0000022736	0.789003531	0.575	0.72	HLA-U	unprocessed_p	major histo	CHR_HSCHR	29926431	29926616
ENSG0000025925	-0.398582658	0.576	0.72	AC018904.1	lncRNA	novel transc	15	58587507	58591676
ENSG0000025702	0.240416345	0.576	0.72	AC010186.3	lncRNA	novel transc	12	9658567	9662085
ENSG0000015550	0.037362154	0.576	0.72	CNOT8	protein_coding	CCR4-NOT	5	154857553	154876793
ENSG0000019711	0.021976789	0.576	0.72	PCBP2	protein_coding	poly(rC) bin	12	53452102	53481162
ENSG0000027218	-0.474701811	0.576	0.72	AC005041.3	lncRNA	novel transc	2	74501717	74502365
ENSG0000026080	0.333108243	0.576	0.72	AL163051.1	lncRNA	novel transc	14	96500810	96502321
ENSG0000023111	-0.400714542	0.577	0.72	AL035587.1	lncRNA	novel transc	6	42940364	42948360
ENSG0000024887	-0.584260499	0.577	0.72	SERBP1P6	processed_pse	SERPINE1 m	5	42465400	42468868
ENSG0000006604	-0.031646798	0.577	0.72	ELAVL1	protein_coding	ELAV like RN	19	7958573	8005659
ENSG0000018861	0.025943338	0.577	0.72	SUMO2	protein_coding	small ubiqui	17	75165586	75182959
ENSG0000022857	0.206125252	0.577	0.72	NUTM2E	protein_coding	NUT family	10	79841358	79850878
ENSG0000020765	0.397829947	0.577	0.72	MIR621	miRNA	microRNA 6	13	40810766	40810861
ENSG0000023636	-0.418478047	0.577	0.72	RRAS2P1	processed_pse	RRAS2 pseu	1	52993201	52993702
ENSG0000013217	-0.316880367	0.577	0.72	PPARG	protein_coding	peroxisome	3	12287368	12434356
ENSG0000022815	0.659948282	0.578	0.72	AL357140.2	lncRNA	novel transc	1	9942923	9949974
ENSG0000025262	1.229234501	0.578	0.72	RNU6ATAC	snRNA	RNA, U6ata	3	195523087	195523221
ENSG0000026347	1.229234501	0.578	0.72	NA	NA	NA	NA	NA	NA
ENSG0000019816	-0.106007435	0.578	0.72	SVIP	protein_coding	small VCP in	11	22813799	22830299
ENSG0000011269	0.137277518	0.578	0.72	GMDS	protein_coding	GDP-manno	6	1623806	2245605
ENSG0000015787	0.136264567	0.578	0.72	PRXL2B	protein_coding	peroxiredox	1	2586491	2591469
ENSG0000017060	0.026623123	0.578	0.72	HSPA4	protein_coding	heat shock p	5	133052013	133106449
ENSG0000020769	-0.842553335	0.578	0.72	MIR573	miRNA	microRNA 5	4	24520192	24520290
ENSG0000025139	-0.559692881	0.579	0.72	AC005280.1	lncRNA	novel transc	14	73462362	73477175
ENSG0000015907	-0.064660319	0.579	0.72	CFAP298	protein_coding	cilia and flag	21	32592079	32612603
ENSG0000018867	-0.094531108	0.579	0.72	PARVB	protein_coding	parvin beta	22	43999211	44172939
ENSG0000000496	0.083047149	0.579	0.72	HCCS	protein_coding	holocytochr	X	11111301	11123086
ENSG0000022483	0.132605833	0.579	0.72	TMEM183B	processed_pse	transmembr	3	149982181	149983308
ENSG0000023752	0.405652035	0.579	0.72	NONOP2	processed_pse	non-POU d	2	60936819	60938049
ENSG0000027237	-0.608765349	0.579	0.72	AC092354.2	lncRNA	novel transc	5	65020038	65020551
ENSG0000025114	-0.632230452	0.58	0.72	MRPS30-D	lncRNA	MRPS30 div	5	44742420	44808777
ENSG0000026934	-0.124108506	0.58	0.72	ZNF587B	protein_coding	zinc finger p	19	57819721	57846238
ENSG0000024005	-0.466431946	0.58	0.72	NA	NA	NA	NA	NA	NA
ENSG0000026769	0.757615384	0.58	0.72	AC002116.2	lncRNA	uncharacter	19	36014508	36045972
ENSG0000027693	0.797957298	0.58	0.72	NA	NA	NA	NA	NA	NA
ENSG0000010927	-0.049428172	0.58	0.72	LAMTOR3	protein_coding	late endoso	4	99878336	99894428
ENSG0000010274	0.103084912	0.581	0.72	SLC25A15	protein_coding	solute carrie	13	40789412	40812460
ENSG0000016666	-0.088920437	0.581	0.72	ATF7IP2	protein_coding	activating tr	16	10326434	10483638
ENSG0000023089	-0.427511716	0.581	0.72	RPS18P12	processed_pse	ribosomal p	17	14705076	14705534
ENSG0000023474	-0.220712134	0.581	0.72	EIF5AP4	processed_pse	eukaryotic t	10	80247219	80247683
ENSG0000027805	-0.049113906	0.581	0.72	DDX52	protein_coding	DExD-box h	17	37609739	37643446
ENSG0000023944	0.385768555	0.581	0.72	ST3GAL6-A	lncRNA	ST3GAL6 ar	3	98706236	98732757
ENSG0000025832	0.756110871	0.581	0.72	ITFG2-AS1	lncRNA	ITFG2 antise	12	2796877	2812902
ENSG0000026993	0.453494465	0.581	0.72	AC068790.2	lncRNA	novel transc	12	123968023	123968579
ENSG0000028014	0.261530549	0.581	0.72	CU638689.4	lncRNA	novel transc	21	6630182	6670695
ENSG0000027898	0.44986288	0.582	0.72	AC091060.1	TEC	TEC	18	35972151	35973916
ENSG0000019665	0.129725095	0.582	0.72	ZNF502	protein_coding	zinc finger p	3	44712643	44723831
ENSG0000022830	-0.102771514	0.582	0.72	FAM174C	protein_coding	family with s	19	1275530	1279244
ENSG0000024970	0.431646065	0.582	0.72	SRD5A3-AS	lncRNA	SRD5A3 ant	4	55363971	55395847
ENSG0000013475	0.063652609	0.582	0.72	RNF138	protein_coding	ring finger p	18	32091874	32131561
ENSG0000018865	-0.288655141	0.582	0.72	SAXO2	protein_coding	stabilizer of	15	82262810	82284930

ENSG0000026641	-0.028030764	0.582	0.72	NCOA4	protein_coding	nuclear rece	10	46005088	46030714
ENSG0000027217	0.710315086	0.583	0.72	AL355385.1	lncRNA	novel transc	6	43074331	43074739
ENSG0000023558	-0.838160394	0.583	0.72	AC011247.1	lncRNA	novel transc	2	38601598	38602178
ENSG0000025588	0.785481062	0.583	0.72	FUNDC2P1	processed_pse	FUN14 dom	5	73508685	73509254
ENSG0000023529	0.832869342	0.583	0.72	FAUP1	processed_pse	FAU pseudo	18	74389884	74390297
ENSG0000022559	-0.138155302	0.583	0.72	VPS52	protein_coding	VPS52 subu	CHR_HSCHR	33171888	33193647
ENSG0000024959	0.477459565	0.583	0.73	BMPR1B-D	lncRNA	BMPR1B div	4	94743668	94757681
ENSG0000006410	-0.048963208	0.583	0.73	INTS13	protein_coding	integrator c	12	26905181	26938326
ENSG0000027184	-0.585531316	0.583	0.73	AC012557.1	lncRNA	novel transc	3	64008082	64008692
ENSG0000018295	-0.05452048	0.584	0.73	HMGN4	protein_coding	high mobilit	6	26538366	26546933
ENSG0000027152	0.331746767	0.584	0.73	BNIP3P17	processed_pse	BCL2 intera	19	20221869	20222463
ENSG0000015716	-0.128359004	0.584	0.73	NRG1	protein_coding	neuregulin 1	8	31639222	32855666
ENSG0000017517	0.393165635	0.584	0.73	FAM182B	lncRNA	family with s	20	25762384	25868225
ENSG0000016839	0.062394442	0.584	0.73	ING5	protein_coding	inhibitor of	2	241687085	241729478
ENSG0000014484	0.076089938	0.584	0.73	RABL3	protein_coding	RAB, memb	3	120684938	120742993
ENSG0000023771	0.846750424	0.584	0.73	AC009095.1	lncRNA	novel transc	16	67614381	67616146
ENSG0000015564	-0.091548395	0.585	0.73	NA	NA	NA	NA	NA	NA
ENSG0000013475	-0.04331616	0.585	0.73	ELP2	protein_coding	elongator ac	18	36129444	36180557
ENSG0000025956	0.537137708	0.585	0.73	AC025430.1	lncRNA	novel transc	15	44516650	44517483
ENSG0000025152	-0.869755555	0.585	0.73	AL162431.2	unprocessed_p	pseudogene	1	180964511	180965909
ENSG0000022869	0.20380747	0.585	0.73	ARL17B	protein_coding	ADP ribosyl	17	46274784	46361797
ENSG0000027942	-0.116214023	0.585	0.73	AL445363.3	TEC	TEC	14	34541044	34541714
ENSG0000021453	-0.722187807	0.585	0.73	RPS15AP1	processed_pse	ribosomal p	20	21166206	21166596
ENSG0000024931	-1.421236231	0.585	0.73	AC068533.4	protein_coding	novel protei	7	66087761	66152277
ENSG0000023158	0.609776536	0.586	0.73	SNORD62B	snoRNA	small nucleo	9	131490486	131490571
ENSG0000027781	0.78686743	0.586	0.73	NA	NA	NA	NA	NA	NA
ENSG0000023334	-0.073475519	0.586	0.73	BAG6	protein_coding	BAG cochap	CHR_HSCHR	31629769	31643448
ENSG0000023416	-0.223252407	0.586	0.73	AL513165.1	lncRNA	novel transc	9	37509150	37510299
ENSG0000016402	0.04013198	0.586	0.73	METAP1	protein_coding	methionyl a	4	98995659	99062809
ENSG0000011621	-0.088142218	0.586	0.73	LRRC42	protein_coding	leucine rich	1	53946085	53968168
ENSG0000013824	0.030851256	0.586	0.73	DNAJC13	protein_coding	DnaJ heat sl	3	132417502	132539032
ENSG0000026337	1.153923548	0.586	0.73	AC104564.1	lncRNA	novel transc	17	29639627	29640825
ENSG0000013591	0.065725072	0.586	0.73	TTLL4	protein_coding	tubulin tyro	2	218710835	218755416
ENSG0000027304	-1.490618643	0.586	0.73	AC012531.3	protein_coding	novel protei	12	53985845	54034888
ENSG0000024527	0.479714911	0.586	0.73	SAP30L-AS	lncRNA	SAP30L anti	5	154325568	154445850
ENSG0000013657	-0.288923511	0.587	0.73	BLK	protein_coding	BLK proto-c	8	11486894	11564599
ENSG0000008251	-0.064057241	0.587	0.73	MRPL22	protein_coding	mitochondr	5	154941073	154969411
ENSG0000010090	0.096378399	0.587	0.73	EMC9	protein_coding	ER membra	14	24138959	24141591
ENSG0000012427	-0.085807248	0.587	0.73	FASTKD3	protein_coding	FAST kinase	5	7859159	7869037
ENSG0000016067	-0.276810392	0.587	0.73	S100A1	protein_coding	S100 calciur	1	153627926	153632039
ENSG0000016817	-0.037244751	0.587	0.73	MAPK1IP1L	protein_coding	mitogen-ac	14	55051647	55070194
ENSG0000025403	0.402460821	0.587	0.73	AC104117.3	lncRNA	novel transc	5	178969390	178990116
ENSG0000025908	0.663934609	0.587	0.73	AL132639.3	lncRNA	novel transc	14	39174885	39175880
ENSG0000011820	0.026722069	0.588	0.73	CAMSAP2	protein_coding	calmodulin	1	200739558	200860704
ENSG0000015845	0.244367138	0.588	0.73	TSPAN33	protein_coding	tetraspanin	7	129144707	129169699
ENSG0000027448	0.297681865	0.588	0.73	NA	NA	NA	NA	NA	NA
ENSG0000027695	-0.486951479	0.588	0.73	AL121772.3	lncRNA	novel transc	20	25284915	25285588
ENSG0000011403	0.037744735	0.588	0.73	KPNA1	protein_coding	karyopherin	3	122421902	122514945
ENSG0000027080	-0.296437063	0.588	0.73	AC010326.4	transcribed_pr	ubiquitin-co	19	57867885	57868834
ENSG0000020643	-0.435575156	0.588	0.73	HLA-C	protein_coding	major histoc	CHR_HSCHR	31262172	31265564
ENSG0000017746	-0.144432008	0.588	0.73	CAVIN1	protein_coding	caveolae ass	17	42402449	42423256
ENSG0000026751	0.116182691	0.588	0.73	AC020916.1	lncRNA	novel transc	19	13823880	13842928
ENSG0000014857	0.067569342	0.589	0.73	NRBF2	protein_coding	nuclear rece	10	63133247	63155031
ENSG0000023743	0.865001997	0.589	0.73	CAMTA1-D	lncRNA	CAMTA1 div	1	6783892	6784843
ENSG0000027351	0.279816713	0.589	0.73	TBC1D3K	protein_coding	TBC1 doma	17	37924415	37935365
ENSG0000027780	0.388133289	0.589	0.73	LENG1	protein_coding	leukocyte re	CHR_HSCHR	54156104	54160226
ENSG0000012586	-0.029844265	0.589	0.73	DSTN	protein_coding	destrin, acti	20	17570075	17609919
ENSG0000017605	-0.608081585	0.589	0.73	RPL23P2	processed_pse	ribosomal p	21	28997613	28998033

ENSG0000021017	-0.106902056	0.589	0.73	MT-TH	Mt_tRNA	mitochondr	MT	12138	12206
ENSG0000018583	0.316606034	0.589	0.73	AL035411.1	processed_pse	adenylate ki	1	58630841	58631530
ENSG0000017322	-0.082500419	0.589	0.73	IQCB1	protein_coding	IQ motif cor	3	121769761	121835079
ENSG0000022153	0.930035235	0.589	0.73	SNORD99	snoRNA	small nuclec	1	28578749	28578822
ENSG0000026008	-0.231802315	0.589	0.73	MIR762HG	lncRNA	MIR762 hos	16	30875222	30895220
ENSG0000028019	0.18124588	0.589	0.73	AC245140.2	lncRNA	novel transc	X	154396878	154398816
ENSG0000013246	-0.03301945	0.59	0.73	GRSF1	protein_coding	G-rich RNA	4	70815783	70839897
ENSG0000014965	-0.035525779	0.59	0.73	YTHDF1	protein_coding	YTH N6-me	20	63195429	63216139
ENSG0000014345	-0.095708719	0.59	0.73	GABPB2	protein_coding	GA binding	1	151070578	151125542
ENSG0000011229	-0.091582128	0.59	0.73	ALDH5A1	protein_coding	aldehyde de	6	24494867	24537207
ENSG0000011107	-0.287913097	0.59	0.73	TNS2	protein_coding	tensin 2 [So	12	53046969	53064379
ENSG0000024451	0.227259602	0.59	0.73	AC109587.1	lncRNA	novel transc	3	69013941	69056622
ENSG0000022890	-0.266442082	0.591	0.73	RASA4CP	transcribed_un	RAS p21 prc	7	44026951	44041892
ENSG0000026384	0.211297928	0.591	0.73	AC022211.2	lncRNA	novel transc	17	75271369	75273895
ENSG0000026997	0.32911146	0.591	0.73	NA	NA	NA	NA	NA	NA
ENSG0000025041	-0.550482221	0.591	0.73	AC112722.1	lncRNA	novel transc	4	185370725	185390928
ENSG0000023572	-0.233241709	0.591	0.73	GABPAP	processed_pse	GABPA pseu	7	63893286	63894641
ENSG0000027614	-0.680917338	0.591	0.73	AC084824.5	lncRNA	novel transc	12	32725248	32725660
ENSG0000027011	-0.671615665	0.591	0.73	AL513327.2	lncRNA	novel transc	1	33261212	33261680
ENSG0000021402	0.189342532	0.591	0.73	MRPL23	protein_coding	mitochondr	11	1947278	1984522
ENSG0000027017	-0.25983885	0.591	0.73	NA	NA	NA	NA	NA	NA
ENSG0000024553	0.457831494	0.592	0.73	RORA-AS1	lncRNA	RORA antise	15	60479152	60630637
ENSG0000027575	-0.406079861	0.592	0.73	DDTL	protein_coding	D-dopachrc	CHR_HSCHR	23966902	23972534
ENSG0000021019	-0.106361772	0.592	0.73	MT-TL2	Mt_tRNA	mitochondr	MT	12266	12336
ENSG0000027640	0.277547836	0.592	0.73	NA	NA	NA	NA	NA	NA
ENSG0000016880	0.041797677	0.592	0.73	SNTB2	protein_coding	syntrophin b	16	69187129	69309052
ENSG0000015879	-0.169991162	0.592	0.73	SPATA2L	protein_coding	spermatoge	16	89696357	89701705
ENSG0000016613	0.098819758	0.592	0.73	RPUSD2	protein_coding	RNA pseudc	15	40569299	40574949
ENSG0000002226	0.02742641	0.592	0.73	FHL1	protein_coding	four and a h	X	136146702	136211359
ENSG0000007227	-0.025841026	0.593	0.73	TFRC	protein_coding	transferrin r	3	196027183	196082096
ENSG0000011257	-0.05198343	0.593	0.73	BYSL	protein_coding	bystin like [S	6	41921499	41933046
ENSG0000024928	-0.590185675	0.593	0.73	AMD1P3	processed_pse	adenosylme	5	43586918	43588223
ENSG0000023768	-0.451536015	0.593	0.73	AL109615.3	lncRNA	novel transc	6	43995723	44074652
ENSG0000026844	-0.596476517	0.593	0.73	AC073534.1	processed_pse	hepatitis A v	19	24162370	24163425
ENSG0000020149	0.917685569	0.593	0.73	RNU6-312P	snRNA	RNA, U6 sm	2	200881715	200881821
ENSG0000025494	0.917685569	0.593	0.73	AP000866.4	processed_pse	ring finger p	11	124807822	124808269
ENSG0000025952	0.54673794	0.593	0.73	AL136295.4	protein_coding	novel protei	14	24180395	24190416
ENSG0000010918	0.066404467	0.593	0.73	USP46	protein_coding	ubiquitin sp	4	52590960	52659301
ENSG0000027361	-0.071284836	0.593	0.73	ZNHIT3	protein_coding	zinc finger H	17	36486629	36499310
ENSG0000016563	-0.031077632	0.594	0.73	VDAC2	protein_coding	voltage dep	10	75210154	75231448
ENSG0000012624	0.44969195	0.594	0.73	IGFLR1	protein_coding	IGF like fam	19	35738801	35742453
ENSG0000024021	-0.945170779	0.594	0.73	PPP1R35-A	lncRNA	PPP1R35 an	7	100436204	100438504
ENSG0000025254	0.80226289	0.594	0.73	SNORD36C	snoRNA	small nuclec	9	133350847	133350912
ENSG0000027693	-0.209366346	0.594	0.73	MBOAT7	protein_coding	membrane	CHR_HSCHR	54173818	54190312
ENSG0000013426	0.073404195	0.594	0.73	NAPG	protein_coding	NSF attachr	18	10525905	10552764
ENSG0000024925	-0.448132253	0.594	0.73	AC079193.2	lncRNA	novel transc	8	17131181	17149789
ENSG0000019926	0.908269654	0.594	0.73	SNORA60	snoRNA	small nuclec	20	38449370	38449503
ENSG0000009038	-0.568166668	0.594	0.73	LYZ	protein_coding	lysozyme [S	12	69348381	69354234
ENSG0000027839	0.654397921	0.594	0.73	AL122023.1	lncRNA	novel transc	14	93334528	93335057
ENSG0000010496	0.100886586	0.595	0.73	NOVA2	protein_coding	NOVA alterı	19	45933734	45974044
ENSG0000024258	-0.098628196	0.595	0.73	AC108010.1	lncRNA	novel transc	7	128574751	128626473
ENSG0000022499	-0.199504753	0.595	0.73	TRIM39	protein_coding	tripartite mc	CHR_HSCHR	30316275	30333529
ENSG0000011889	-0.259666061	0.595	0.73	PPL	protein_coding	periplakin [S	16	4882507	4960741
ENSG0000017358	0.067272377	0.595	0.73	CEP83	protein_coding	centrosoma	12	94306449	94459988
ENSG0000025955	0.417983245	0.595	0.73	AC067863.1	lncRNA	novel transc	15	78625895	78628193
ENSG0000013604	0.051470349	0.595	0.73	PWP1	protein_coding	PWP1 homc	12	107685799	107713162
ENSG0000024953	-0.683541909	0.595	0.73	MIR302CH(	lncRNA	miR-302/36	4	112646476	112650051
ENSG0000013457	-0.081805987	0.596	0.73	ACP2	protein_coding	acid phosph	11	47239302	47248906

ENSG0000017998	0.067010518	0.596	0.73	TSHZ1	protein_coding	teashirt zinc	18	75210755	75289950
ENSG0000018848	0.121460227	0.596	0.73	IER5L	protein_coding	immediate e	9	129175552	129178261
ENSG0000023474	-0.50899445	0.596	0.73	AL162386.2	lncRNA	novel transc	9	13274510	13279187
ENSG0000007358	-0.027805649	0.596	0.73	SMARCE1	protein_coding	SWI/SNF rel	17	40624962	40648654
ENSG0000014269	-0.178983886	0.596	0.73	EVA1B	protein_coding	eva-1 homo	1	36322031	36324154
ENSG0000016961	0.064332909	0.596	0.73	RAMAC	protein_coding	RNA guanin	15	82986210	82991057
ENSG0000020656	-0.060858623	0.596	0.73	ANKRD28	protein_coding	ankyrin repe	3	15667236	15859771
ENSG0000027400	-1.229078417	0.597	0.74	RPS9	protein_coding	ribosomal p	CHR_HSCHR	54201473	54208260
ENSG0000022782	-0.162154369	0.597	0.74	AC138969.2	unprocessed_p	polycystic ki	16	16356224	16377507
ENSG0000013101	-0.361559526	0.597	0.74	ULBP3	protein_coding	UL16 bindin	6	150061053	150069121
ENSG0000020634	-0.660184452	0.597	0.74	STK19	protein_coding	serine/threo	CHR_HSCHR	31961552	31971824
ENSG0000023495	0.81008447	0.598	0.74	SAPCD1	protein_coding	suppressor	CHR_HSCHR	31839095	31841147
ENSG0000027140	-0.598914163	0.598	0.74	AC010680.5	lncRNA	novel transc	2	178644717	178645179
ENSG0000024568	-0.610111755	0.598	0.74	FRG1-DT	lncRNA	FRG1 diverg	4	189764391	189940733
ENSG0000017588	-0.254745559	0.598	0.74	RPL7AP66	processed_pse	ribosomal p	18	39334872	39335672
ENSG0000018936	0.67849901	0.598	0.74	ALG1L	protein_coding	ALG1 chitob	3	125929275	125937039
ENSG0000004249	-0.551949277	0.599	0.74	CAPG	protein_coding	capping act	2	85394753	85418432
ENSG0000022995	0.560611218	0.599	0.74	ZRANB2-A	lncRNA	ZRANB2 ant	1	71081324	71489976
ENSG0000008597	0.054789881	0.599	0.74	ATG16L1	protein_coding	autophagy r	2	233210051	233295674
ENSG0000028006	-0.303318237	0.599	0.74	AC023818.1	TEC	novel transc	16	48343755	48346058
ENSG0000026591	0.470493003	0.599	0.74	MIR3685	miRNA	microRNA 3	12	95309923	95309984
ENSG0000010326	-0.457824096	0.599	0.74	RHBDL1	protein_coding	rhomboid li	16	675666	678268
ENSG0000023536	-1.087642872	0.6	0.74	RPL36AP15	processed_pse	ribosomal p	2	47797826	47798091
ENSG0000022608	-0.654504575	0.6	0.74	EIF3LP3	processed_pse	eukaryotic t	10	38080056	38080877
ENSG0000013866	-0.020465118	0.601	0.74	HNRNPD	protein_coding	heterogene	4	82352498	82374503
ENSG0000017578	-0.198879359	0.601	0.74	ZNF169	protein_coding	zinc finger p	9	94259298	94301829
ENSG0000019672	-0.188265197	0.601	0.74	ZNF418	protein_coding	zinc finger p	19	57921882	57935402
ENSG0000003182	-0.041573666	0.602	0.74	RANBP3	protein_coding	RAN binding	19	5916139	5978142
ENSG0000024197	0.438744257	0.602	0.74	ELOCP19	processed_pse	elongin C p	1	88829102	88829419
ENSG0000027419	0.392500686	0.602	0.74	MBOAT7	protein_coding	membrane	CHR_HSCHR	54173854	54190480
ENSG0000022713	-0.640355247	0.602	0.74	NA	NA	NA	NA	NA	NA
ENSG0000017451	-0.404064136	0.602	0.74	MFSD4A	protein_coding	major facilit	1	205568885	205602918
ENSG0000015685	-0.069587221	0.603	0.74	PRR14	protein_coding	proline rich	16	30650717	30656413
ENSG0000012736	0.361196321	0.603	0.74	TAS2R5	protein_coding	taste 2 rece	7	141790217	141791367
ENSG0000010032	0.023608686	0.603	0.74	RBFOX2	protein_coding	RNA binding	22	35738736	36028824
ENSG0000023376	0.151903852	0.603	0.74	RPS15P4	processed_pse	ribosomal p	2	171517270	171517716
ENSG0000027641	-0.061021488	0.604	0.74	UNC79	protein_coding	unc-79 hom	CHR_HSCHR	93333219	93707876
ENSG0000025124	-0.15921554	0.604	0.74	ZNF345	protein_coding	zinc finger p	19	36850361	36913029
ENSG0000016425	0.054982253	0.604	0.74	NDUFS4	protein_coding	NADH:ubiqu	5	53560633	53683338
ENSG0000021471	0.130558539	0.604	0.74	AC005562.1	lncRNA	novel transc	17	30576464	30672789
ENSG0000023304	0.123794283	0.604	0.74	DHX16	protein_coding	DEAH-box I	CHR_HSCHR	30697750	30717697
ENSG0000010341	-0.119665836	0.605	0.74	HMOX2	protein_coding	heme oxyge	16	4474690	4510347
ENSG0000025546	0.781603534	0.605	0.74	SEPTIN14P8	unprocessed_p	septin 14 ps	8	200385	200562
ENSG0000026138	0.644684313	0.605	0.74	AC027682.4	lncRNA	novel transc	16	67549214	67563958
ENSG0000024985	0.267858407	0.605	0.74	EEF1A1P19	processed_pse	eukaryotic t	5	43495073	43496454
ENSG0000026511	0.640316972	0.605	0.74	MIR3153	miRNA	microRNA 3	9	89312225	89312306
ENSG0000026747	0.154711155	0.605	0.74	ZNF571-AS	lncRNA	ZNF571 ant	19	37548914	37587348
ENSG0000023565	-0.105982712	0.605	0.74	HLA-A	protein_coding	major histo	CHR_HSCHR	29879362	29999797
ENSG0000026501	0.855925326	0.605	0.74	AC087301.1	lncRNA	novel transc	17	73243093	73244706
ENSG0000014716	0.044267765	0.605	0.74	SNX12	protein_coding	sorting nexi	X	71056332	71073426
ENSG0000017645	-0.092062361	0.606	0.74	LPCAT4	protein_coding	lysophospha	15	34358633	34367196
ENSG0000024751	0.106078542	0.606	0.74	MIR4458HG	lncRNA	MIR4458 ho	5	8450701	8486930
ENSG0000016388	-0.046504741	0.606	0.74	POLR2H	protein_coding	RNA polyme	3	184361718	184368596
ENSG0000025079	-0.878529628	0.606	0.74	AC112484.2	processed_pse	cell death-i	3	128869624	128870026
ENSG0000024002	-0.091326	0.606	0.74	LINC00888	transcribed_un	long interge	3	183447608	183456013
ENSG0000027938	0.499514329	0.606	0.74	NA	NA	NA	NA	NA	NA
ENSG0000020660	-1.160135652	0.606	0.74	SNORA22B	snoRNA	small nucleo	7	56055365	56055502
ENSG0000014720	-0.158139384	0.607	0.74	DIAPH2	protein_coding	diaphanous	X	96684663	97604997

ENSG0000020496	0.367606641	0.607	0.74	PCDHA7	protein_coding	protocadhe		5	140834248	141012347
ENSG0000018937	-0.070478734	0.607	0.74	C8orf76	protein_coding	chromosom		8	123219967	123241377
ENSG0000022319	1.162998867	0.607	0.74	RNU2-22P	snRNA	RNA, U2 sm		2	231501990	231502201
ENSG0000027632	-0.399292268	0.607	0.74	TFPT	protein_coding	TCF3 fusion	CHR_HSCHR		54107066	54115801
ENSG0000006358	0.059706525	0.607	0.74	ZNF275	protein_coding	zinc finger p	X		153334147	153360110
ENSG0000014833	0.043341959	0.607	0.74	ASB6	protein_coding	ankyrin repe		9	129634604	129642169
ENSG0000026424	0.111516666	0.607	0.74	LINC00909	lncRNA	long interge		18	74590317	74598885
ENSG0000026580	-0.635080687	0.607	0.74	MIR4292	miRNA	microRNA 4		9	136830957	136831023
ENSG0000027199	-0.777073062	0.607	0.74	AC013400.1	lncRNA	novel transc		2	19902025	19902569
ENSG0000004870	0.040649827	0.608	0.74	VPS13D	protein_coding	vacuolar pro		1	12230030	12512047
ENSG0000021217	-0.84123078	0.608	0.74	AC015982.1	snoRNA			2	55565703	55565850
ENSG0000026764	-0.385903354	0.608	0.74	AC010327.5	lncRNA	novel transc		19	55216660	55221616
ENSG0000027839	0.408105922	0.608	0.74	AL354696.2	lncRNA	novel transc		13	41132939	41236686
ENSG0000020008	-0.482484775	0.608	0.74	SNORA73B	snoRNA	small nucleo		1	28508559	28508762
ENSG0000027522	1.03059887	0.608	0.74	AC093416.2	misc_RNA			3	25618132	25618312
ENSG0000016300	-0.058947606	0.608	0.74	CCDC138	protein_coding	coiled-coil d		2	108786757	108885477
ENSG0000022418	-0.284005705	0.608	0.74	C5orf66	lncRNA	chromosom		5	135033280	135358219
ENSG0000018349	-0.04116543	0.608	0.74	MEX3B	protein_coding	mex-3 RNA		15	82041778	82046119
ENSG0000012021	0.341888513	0.609	0.74	MLANA	protein_coding	melan-A [So		9	5890889	5910606
ENSG0000025047	0.503798713	0.609	0.74	WBP1LP2	processed_pse	WBP1L pseu		7	107628553	107629550
ENSG0000026401	1.01893303	0.609	0.74	AC015908.3	lncRNA	novel transc		17	10741257	10769089
ENSG0000027714	0.329066837	0.609	0.74	LINC00235	lncRNA	long interge		16	525155	527407
ENSG0000010508	0.079656248	0.61	0.75	MED26	protein_coding	mediator co		19	16574907	16629062
ENSG0000023919	1.224375907	0.61	0.75	AC090985.1	snoRNA			15	92417547	92417689
ENSG0000024915	0.617839542	0.61	0.75	AC091965.1	lncRNA	novel transc		5	8387638	8457564
ENSG0000025532	0.553780472	0.61	0.75	AP001922.5	lncRNA	novel transc		11	75583196	75594665
ENSG0000027898	0.381473825	0.61	0.75	AL031009.1	TEC	TEC		16	1688355	1690536
ENSG0000021293	-0.32958932	0.611	0.75	RPL23AP4	processed_pse	ribosomal p		21	46690764	46691226
ENSG0000005766	-0.051294054	0.611	0.75	ATG5	protein_coding	autophagy r		6	106045423	106325791
ENSG0000021665	0.604449286	0.611	0.75	GLRX3P2	processed_pse	glutaredoxin		6	3978062	3979099
ENSG0000013726	-0.177330419	0.611	0.75	SLC22A23	protein_coding	solute carrie		6	3268962	3457022
ENSG0000013137	-0.118954151	0.611	0.75	HACL1	protein_coding	2-hydroxya		3	15560699	15601852
ENSG0000018152	-0.067103639	0.611	0.75	SGSH	protein_coding	N-sulfogluc		17	80206716	80220923
ENSG0000025879	-0.694610213	0.611	0.75	AL121594.1	protein_coding	novel transc		14	35122549	35317474
ENSG0000018548	-0.09192636	0.612	0.75	SDHAP1	transcribed_un	succinate de		3	195959748	195990318
ENSG0000018524	0.036353204	0.612	0.75	PRPF39	protein_coding	pre-mRNA		14	45084107	45116282
ENSG0000025890	-0.238942758	0.612	0.75	HNRNPCP1	processed_pse	heterogene		14	58521223	58521988
ENSG0000027385	0.14212325	0.612	0.75	PPP2R3B	protein_coding	protein pho	CHR_HSCHR		333240	385935
ENSG0000017367	0.234071035	0.612	0.75	SPDYE2B	protein_coding	speedy/RIN		7	102650325	102661398
ENSG0000025908	-0.879449884	0.613	0.75	AC074091.2	lncRNA	novel transc		2	27583046	27635174
ENSG0000027276	0.906699008	0.613	0.75	AL596094.1	lncRNA	novel transc		10	5813985	5814441
ENSG0000027933	-0.220557667	0.613	0.75	RBM12B-A	TEC	RBM12B ant		8	93740121	93740773
ENSG0000016383	0.077323917	0.613	0.75	ELP6	protein_coding	elongator ac		3	47495640	47513712
ENSG0000021322	-0.241294948	0.614	0.75	DNLZ	protein_coding	DNL-type z		9	136359480	136363744
ENSG0000012758	0.105681205	0.614	0.75	TUBBP1	transcribed_pr	tubulin beta		8	30351873	30353518
ENSG0000027131	-0.585109636	0.614	0.75	AC174048.1	processed_pse	polymerase		2	16149251	16149394
ENSG0000023247	-0.381319835	0.614	0.75	AL136368.1	lncRNA	novel transc		10	110869743	110871594
ENSG0000009214	-0.024534794	0.614	0.75	HECTD1	protein_coding	HECT doma		14	31100112	31207804
ENSG0000025039	0.285914164	0.614	0.75	AP006623.1	lncRNA	novel transc		11	856880	859795
ENSG0000027797	0.456579159	0.615	0.75	AC010542.5	lncRNA	novel transc		16	66565620	66566001
ENSG0000006890	0.061585437	0.615	0.75	SIRT2	protein_coding	sirtuin 2 [So		19	38878555	38899862
ENSG0000026922	-0.340800701	0.615	0.75	NA	NA	NA	NA		NA	NA
ENSG0000018686	-0.071199824	0.615	0.75	POFUT2	protein_coding	protein O-fu		21	45263928	45287898
ENSG0000023355	-0.623739786	0.615	0.75	AL050331.1	processed_pse	RPS5 (40S R		6	116258493	116259115
ENSG0000007251	0.038478452	0.615	0.75	MARK2	protein_coding	microtubule		11	63838928	63911019
ENSG0000028044	-1.205026374	0.615	0.75	NA	NA	NA	NA		NA	NA
ENSG0000023325	0.856266834	0.616	0.75	AC233728.1	lncRNA	novel transc	X		53432722	53433032
ENSG0000014306	0.044826977	0.616	0.75	ZNF697	protein_coding	zinc finger p		1	119619377	119648266

ENSG0000026596	-0.404582527	0.616	0.75	GACAT2	lncRNA	gastric canc	18	8695856	8707621
ENSG0000012606	0.033174433	0.617	0.75	PSMB2	protein_coding	proteasome	1	35599541	35641526
ENSG0000020744	0.653109951	0.617	0.75	SNORD15B	snoRNA	small nucle	11	75404421	75404566
ENSG0000025095	0.574061996	0.616	0.75	AC093752.	lncRNA	novel transc	4	119456350	119457277
ENSG0000027859	-1.007154124	0.617	0.75	MIR6775	miRNA	microRNA 6	16	87834592	87834660
ENSG0000025517	-0.425059609	0.617	0.75	AP000941.	lncRNA	novel transc	11	118636620	118638097
ENSG0000027914	-0.536765321	0.617	0.75	AL590326.1	TEC	novel transc	14	104815449	104815753
ENSG0000027990	0.414686554	0.617	0.75	AC073539.	lncRNA	novel transc	CHR_HSCHR	22251461	22253554
ENSG0000027673	0.043074896	0.617	0.75	KIF5C	protein_coding	kinesin fami	CHR_HSCHR	148937284	149030199
ENSG0000010729	-0.027189989	0.618	0.75	SETX	protein_coding	senataxin [S	9	132261356	132354986
ENSG0000011670	0.075399771	0.618	0.75	SLC35D1	protein_coding	solute carrie	1	66999350	67054148
ENSG0000018102	-0.08976991	0.618	0.75	TRAPPC5	protein_coding	trafficking p	19	7680833	7687703
ENSG0000027401	-0.105013128	0.618	0.75	RN7SL2	misc_RNA	RNA compo	14	49862550	49862849
ENSG0000027437	-0.603158453	0.618	0.75	AC005702.	unprocessed_p	double C2-	17	60025859	60030746
ENSG0000027600	-0.301501608	0.618	0.75	NA	NA	NA	NA	NA	NA
ENSG0000007715	0.046068017	0.618	0.75	PPP1R12B	protein_coding	protein pho	1	202348699	202592706
ENSG0000025892	0.278906894	0.618	0.75	FOXN3-AS	lncRNA	FOXN3 anti	14	89417354	89419793
ENSG0000020408	-0.068384376	0.619	0.75	INPP5B	protein_coding	inositol poly	1	37860697	37947057
ENSG0000026992	0.548756837	0.619	0.75	AC068620.	lncRNA	novel transc	4	56387625	56388153
ENSG0000017341	-0.04951044	0.619	0.75	NAA20	protein_coding	N-alpha-ac	20	20017310	20033655
ENSG0000019839	-0.066885325	0.619	0.75	ZNF26	protein_coding	zinc finger p	12	132986365	133032952
ENSG0000012465	0.053049139	0.62	0.75	TBCC	protein_coding	tubulin foldi	6	42744498	42746103
ENSG0000009194	-0.069108118	0.62	0.75	TMEM101	protein_coding	transmembr	17	44011188	44023946
ENSG0000012635	-0.040423701	0.62	0.75	THRA	protein_coding	thyroid horr	17	40058290	40093867
ENSG0000019891	-0.074953736	0.62	0.75	C1orf174	protein_coding	chromosom	1	3889125	3900293
ENSG0000024941	0.875165188	0.62	0.75	AC117383.	lncRNA	novel transc	3	141267353	141367137
ENSG0000027600	-0.390028451	0.62	0.75	AC079414.	lncRNA	novel transc	16	78123243	78124332
ENSG0000022597	-0.438837673	0.62	0.75	CKS1BP2	processed_pse	CDC28 prot	10	29697935	29698174
ENSG0000016765	0.049780835	0.62	0.75	DAPK3	protein_coding	death assoc	19	3958453	3971123
ENSG0000012545	0.098312169	0.621	0.75	SLC25A19	protein_coding	solute carrie	17	75272981	75289510
ENSG0000014696	0.025595049	0.621	0.75	LUC7L2	protein_coding	LUC7 like 2,	7	139340359	139423457
ENSG0000020463	-0.055273932	0.621	0.75	TBC1D8	protein_coding	TBC1 doma	2	101007617	101252866
ENSG0000020662	-0.999370263	0.621	0.75	SNORD116	snoRNA	small nucle	15	25080142	25080233
ENSG0000026685	0.994942707	0.621	0.75	MIR4482	miRNA	microRNA 4	10	104268336	104268405
ENSG0000026779	0.168715954	0.621	0.75	LIN37	protein_coding	lin-37 DREA	19	35748361	35754519
ENSG0000027526	-0.677150905	0.621	0.75	AC135048.	lncRNA	novel transc	16	30956872	30957199
ENSG0000013150	-0.032582547	0.622	0.75	UBE2D2	protein_coding	ubiquitin co	5	139526431	139628434
ENSG0000016155	-0.17150111	0.622	0.75	TMEM143	protein_coding	transmembr	19	48332356	48364059
ENSG0000021764	-0.485673887	0.622	0.75	AL136116.	processed_pse	pseudogene	6	143342246	143343383
ENSG0000024039	-0.33174677	0.622	0.75	RPL5P23	processed_pse	ribosomal p	8	42341767	42342634
ENSG0000025361	-0.916110758	0.622	0.75	GRPEL2-AS	lncRNA	GRPEL2 anti	5	149348116	149357642
ENSG0000014315	0.032066172	0.622	0.75	TIPRL	protein_coding	TOR signali	1	168178962	168202109
ENSG0000025334	-0.440036101	0.622	0.75	AC115837.	processed_pse	poly(rC) bin	8	74603244	74604244
ENSG0000027965	-0.797906285	0.622	0.76	NA	NA	NA	NA	NA	NA
ENSG0000021228	0.771561137	0.622	0.76	SNORD89	snoRNA	small nucle	2	101272936	101273049
ENSG0000023702	-0.294091441	0.622	0.76	NA	NA	NA	NA	NA	NA
ENSG0000009031	0.036786752	0.623	0.76	MAEA	protein_coding	macrophage	4	1289887	1340147
ENSG0000011087	0.381438032	0.623	0.76	SELPLG	protein_coding	selectin P lig	12	108621895	108633894
ENSG0000026332	0.574294928	0.623	0.76	AC133552.	unprocessed_p	thyroid horr	16	25071490	25072727
ENSG0000013927	-0.921304499	0.623	0.76	GLIPR1	protein_coding	GLI pathoge	12	75480753	75503863
ENSG0000018755	0.026267898	0.623	0.76	USP7	protein_coding	ubiquitin sp	16	8892097	8975328
ENSG0000026992	0.45264373	0.623	0.76	Z98884.2	lncRNA	novel transc	1	7776383	7776775
ENSG0000006316	-0.092835692	0.623	0.76	BICRA	protein_coding	BRD4 intera	19	47608196	47703277
ENSG0000018270	0.215787666	0.623	0.76	IGIP	protein_coding	IgA inducing	5	140125937	140129392
ENSG0000023503	0.333957058	0.623	0.76	C19orf81	protein_coding	chromosom	19	50649445	50659310
ENSG0000006759	0.033947771	0.623	0.76	DHX8	protein_coding	DEAH-box l	17	43483865	43610338
ENSG0000023229	-0.805160462	0.624	0.76	AL589935.1	lncRNA	novel transc	6	71221457	71328228
ENSG0000027315	-0.666876663	0.624	0.76	AC124016.	lncRNA	novel transc	4	82344876	82346842

ENSG0000027407	-0.413680825	0.624	0.76	TFPT	protein_coding	TCF3 fusion	CHR_HSCHR	54107066	54115801
ENSG0000022856	-0.338308686	0.624	0.76	AC013287.1	lncRNA	novel transc	10	63664664	64693446
ENSG0000025915	-0.541487336	0.624	0.76	AC004816.1	lncRNA	novel transc	14	70809841	70815994
ENSG0000020649	0.093154923	0.625	0.76	PRR3	protein_coding	proline rich	CHR_HSCHR	30546378	30553215
ENSG0000027044	0.375619976	0.625	0.76	AC135506.1	unprocessed_p	laminin, bet	3	49140086	49160851
ENSG0000027783	0.586573727	0.625	0.76	AL138960.1	lncRNA	novel transc	13	44369365	44369877
ENSG0000020659	-0.937892044	0.625	0.76	RNU1-27P	snRNA	RNA, U1 sm	14	34546714	34546877
ENSG0000015308	0.452304534	0.625	0.76	ACMSD	protein_coding	aminocarbo	2	134838616	134902034
ENSG0000020404	0.376845085	0.625	0.76	SLC12A5-A	lncRNA	SLC12A5 an	20	46013500	46022073
ENSG0000025916	0.103961205	0.625	0.76	GNRHR2P1	unprocessed_p	GNRHR2 ps	14	60398934	60400013
ENSG0000027024	-0.861104864	0.625	0.76	AC011468.4	processed_pse	transmembr	19	52110318	52110726
ENSG0000020683	0.539041606	0.625	0.76	SNORA5A	snoRNA	small nucleo	7	45104349	45104482
ENSG0000011525	0.317934269	0.626	0.76	PCSK4	protein_coding	proprotein c	19	1481428	1490752
ENSG0000022895	-0.633140133	0.626	0.76	AL591623.2	processed_pse	ribosomal p	1	246632251	246632478
ENSG0000022481	-0.510056502	0.626	0.76	AC010789.1	lncRNA	novel transc	10	101701994	101730037
ENSG0000023507	-0.425262493	0.626	0.76	AC231981.1	lncRNA	novel transc	2	3519275	3523197
ENSG0000010765	0.039958511	0.626	0.76	SEC23IP	protein_coding	SEC23 intera	10	119892730	119944657
ENSG0000022386	-0.437970971	0.627	0.76	HLA-DPB1	protein_coding	major histoc	6	33075990	33089696
ENSG0000000683	-0.143675634	0.627	0.76	CDKL3	protein_coding	cyclin deper	5	134286350	134371047
ENSG0000012567	-0.025717011	0.627	0.76	THOC2	protein_coding	THO comple	X	123600561	123733056
ENSG0000018356	-0.481590791	0.627	0.76	AC131971.1	lncRNA	novel transc	11	2989863	2991344
ENSG0000016764	-1.231012101	0.627	0.76	SPINT2	protein_coding	serine pepti	19	38244035	38292615
ENSG0000027216	-0.05566143	0.628	0.76	CASC15	lncRNA	cancer susce	6	21664185	22654455
ENSG0000013540	-0.030221558	0.628	0.76	CD63	protein_coding	CD63 molec	12	55725323	55729707
ENSG0000014524	0.026422933	0.628	0.76	SLC10A4	protein_coding	solute carrie	4	48483343	48489526
ENSG0000011380	-0.143480589	0.628	0.76	CNTN3	protein_coding	contactin 3	3	74262568	74521140
ENSG0000008825	0.031395296	0.628	0.76	GNA11	protein_coding	G protein su	19	3094362	3123999
ENSG0000023274	-0.247590364	0.628	0.76	AC135050.1	lncRNA	novel transc	16	31056460	31062803
ENSG0000012951	-0.033311518	0.628	0.76	SNX6	protein_coding	sorting nexi	14	34561093	34630160
ENSG0000022678	0.368918318	0.628	0.76	TBCAP1	processed_pse	tubulin foldi	X	33041395	33041721
ENSG0000011896	-0.056729826	0.628	0.76	HS1BP3	protein_coding	HCLS1 bind	2	20560448	20651130
ENSG0000008606	0.062459514	0.629	0.76	B4GALT1	protein_coding	beta-1,4-ga	9	33104082	33167356
ENSG0000022483	0.206515662	0.629	0.76	GCSHP5	processed_pse	glycine clea	1	168055901	168056422
ENSG0000018177	-0.211034581	0.629	0.76	GPR3	protein_coding	G protein-c	1	27392622	27395814
ENSG0000023386	0.457774048	0.629	0.76	LLPHP3	processed_pse	LLPH pseud	2	221944223	221944612
ENSG0000010610	0.108113423	0.629	0.76	NOD1	protein_coding	nucleotide b	7	30424527	30478784
ENSG0000009991	0.046188406	0.629	0.76	MED15	protein_coding	mediator co	22	20495913	20587632
ENSG0000016852	-0.165803589	0.629	0.76	SERINC2	protein_coding	serine incor	1	31409565	31434680
ENSG0000017472	0.038025551	0.629	0.76	LARP7	protein_coding	La ribonucle	4	112637107	112657592
ENSG0000026973	0.219461869	0.629	0.76	AL691432.1	transcribed_pr	ribosomal p	1	1671990	1674397
ENSG0000011995	0.042191447	0.629	0.76	SMNDC1	protein_coding	survival mot	10	110290730	110304938
ENSG0000027480	0.060190752	0.629	0.76	BDP1	protein_coding	B double pr	CHR_HSCHR	70780935	70893131
ENSG0000018765	0.219592655	0.629	0.76	TMSB4XP8	processed_pse	TMSB4X pse	4	90838903	90839037
ENSG0000027534	-0.098971115	0.63	0.76	SMN1	protein_coding	survival of n	CHR_HSCHR	70268827	70296783
ENSG0000024787	0.368102359	0.63	0.76	AC021086.1	lncRNA	novel transc	5	100428124	100535262
ENSG0000018210	0.167681109	0.63	0.76	NA	NA	NA	NA	NA	NA
ENSG0000012774	0.547191208	0.63	0.76	IL17B	protein_coding	interleukin 1	5	149371324	149404202
ENSG0000024415	-0.269351266	0.63	0.76	AC010973.2	lncRNA	novel transc	7	151074742	151076530
ENSG0000017668	-0.20786764	0.631	0.76	LRRC37A	protein_coding	leucine rich	17	46292733	46337794
ENSG0000022455	0.784377749	0.631	0.76	AC114491.1	processed_pse	NADH dehy	1	107505203	107505448
ENSG0000023426	-0.574960829	0.631	0.76	DEPDC1-AS	lncRNA	DEPDC1 ant	1	68496676	68538627
ENSG0000027257	-0.085706319	0.631	0.76	AP000347.1	transcribed_un	glucuronida	22	23658094	23717356
ENSG0000021245	0.784656532	0.631	0.76	SNORD69	snoRNA	small nucleo	3	52692736	52692812
ENSG0000023510	0.130479341	0.631	0.76	RING1	protein_coding	ring finger p	CHR_HSCHR	33362379	33366606
ENSG0000027353	0.667258993	0.631	0.76	NA	NA	NA	NA	NA	NA
ENSG0000014384	-0.2518297	0.631	0.76	PPFIA4	protein_coding	PTPRF intera	1	203026498	203078740
ENSG0000001100	-0.035652836	0.632	0.76	ELOA	protein_coding	elongin A [S	1	23743155	23762059
ENSG0000013247	-0.049297822	0.632	0.76	UNK	protein_coding	unk zinc fing	17	75784806	75825799

ENSG0000027903	-0.328382707	0.632	0.76	AC022211.4	TEC	TEC		17	75130883	75133043
ENSG0000013533	-0.038363782	0.632	0.76	AKIRIN2	protein_coding	akirin 2 [Sou		6	87674860	87702233
ENSG0000025446	0.307807487	0.632	0.76	AP001107.4	lncRNA	novel transc		11	66259567	66261834
ENSG0000020720	0.895766178	0.632	0.76	RNVU1-15	snRNA	RNA, varian		1	144412576	144412740
ENSG0000027970	0.454726024	0.632	0.76	AC131212.2	TEC	novel transc		12	132565071	132566425
ENSG0000020557	0.101022271	0.632	0.76	SMN2	protein_coding	survival of n		5	70049638	70078522
ENSG0000008824	-0.026341026	0.632	0.76	KHSRP	protein_coding	KH-type spl		19	6413348	6424794
ENSG0000016039	-0.101797265	0.632	0.76	C19orf47	protein_coding	chromosom		19	40319536	40348527
ENSG0000010817	-0.043395016	0.633	0.76	PPIF	protein_coding	peptidylprol		10	79347469	79355334
ENSG0000019632	0.032733021	0.633	0.76	ZBTB44	protein_coding	zinc finger a		11	130226677	130314917
ENSG0000027380	0.495011807	0.633	0.76	AC244489.1	protein_coding	novel memb	CHR_HSCHR		16418201	16436927
ENSG0000016346	-0.407007928	0.633	0.76	TSACC	protein_coding	TSSK6 activa		1	156337314	156346995
ENSG0000026095	-0.770806023	0.633	0.76	AC100821.2	lncRNA	novel transc		8	54042989	54045629
ENSG0000016643	-0.037915249	0.633	0.76	RNF169	protein_coding	ring finger p		11	74748849	74842413
ENSG0000027464	0.435035945	0.633	0.76	H2BC17	protein_coding	H2B clustere		6	27893425	27893891
ENSG0000006807	-0.481430309	0.633	0.76	IFI35	protein_coding	interferon ir		17	43006740	43014456
ENSG0000025853	-0.240991501	0.634	0.76	AC068896.1	protein_coding	novel transc		10	124617080	124791727
ENSG0000025852	-0.521994463	0.634	0.76	AL049830.3	lncRNA	novel transc		14	30876179	30889808
ENSG0000026765	0.6414517	0.634	0.76	AC125437.1	lncRNA	novel transc		18	79117207	79117920
ENSG0000024863	0.182041916	0.634	0.76	NA	NA	NA	NA		NA	NA
ENSG0000021548	0.768486581	0.634	0.76	CALM2P3	processed_pse	calmodulin		13	41170700	41171148
ENSG0000027546	0.089024163	0.634	0.76	FP565260.1	protein_coding	periodic tryp		21	5130871	5154658
ENSG0000027060	-0.172557704	0.634	0.76	AC009549.1	lncRNA	novel transc		11	19710934	19712619
ENSG0000023710	-0.30506028	0.635	0.76	C6orf136	protein_coding	chromosom	CHR_HSCHR		30639263	30645325
ENSG0000017996	-0.154419334	0.635	0.76	PPP1R14BP	processed_pse	protein pho		4	139114930	139115374
ENSG0000025883	-0.170312849	0.635	0.76	NA	NA	NA	NA		NA	NA
ENSG0000025985	0.589636091	0.635	0.76	RAB43P1	processed_pse	RAB43 pseu		16	46626404	46626992
ENSG0000023691	0.554957258	0.635	0.76	LINC01852	lncRNA	long interge		15	38068812	38072990
ENSG0000027974	0.274084286	0.635	0.76	AC008764.9	TEC	TEC		19	16586905	16589409
ENSG0000024354	0.125635161	0.636	0.77	HNRNPKP4	processed_pse	heterogene		3	96349554	96350939
ENSG0000027500	0.204123301	0.636	0.77	ZNF280B	protein_coding	zinc finger p		22	22484421	22508742
ENSG0000010334	0.024271102	0.636	0.77	GSPT1	protein_coding	G1 to S pha		16	11868128	11916082
ENSG0000020327	0.728091042	0.636	0.77	AL590705.1	lncRNA	novel transc		9	97200475	97238745
ENSG0000021390	-0.126437243	0.636	0.77	LTB4R2	protein_coding	leukotriene		14	24305734	24312053
ENSG0000013591	-0.039118712	0.636	0.77	SERPINE2	protein_coding	serpin family		2	223975045	224039318
ENSG0000024136	-0.891148941	0.636	0.77	RPL36AP43	processed_pse	ribosomal p		15	89687937	89688243
ENSG0000011287	0.095159184	0.637	0.77	CEP72	protein_coding	centrosoma		5	612340	667168
ENSG0000027462	0.102532499	0.637	0.77	NA	NA	NA	NA		NA	NA
ENSG0000027854	-0.031225261	0.637	0.77	ACACA	protein_coding	acetyl-CoA		17	37084992	37406836
ENSG0000023883	0.637458679	0.637	0.77	AC004839.1	snoRNA			7	107603363	107603507
ENSG0000026864	0.457600351	0.637	0.77	AL132655.2	lncRNA	novel transc		20	58817132	58817725
ENSG0000024282	0.963996454	0.637	0.77	RPS26P21	processed_pse	ribosomal p		3	119298665	119299012
ENSG0000009062	-0.024055157	0.637	0.77	PABPC4	protein_coding	poly(A) binc		1	39560816	39576790
ENSG0000026065	-0.530378664	0.637	0.77	AF213884.3	lncRNA	novel transc		4	102500841	102501319
ENSG0000025561	-0.384469637	0.638	0.77	LINC02440	lncRNA	long interge		12	118773031	118775137
ENSG0000011094	0.557500301	0.638	0.77	IL23A	protein_coding	interleukin 2		12	56334174	56340410
ENSG0000010544	-0.056920778	0.638	0.77	GRWD1	protein_coding	glutamate r		19	48445841	48457022
ENSG0000022693	-0.296841931	0.638	0.77	CEP164P1	transcribed_pr	centrosoma		10	45002222	45076066
ENSG0000011789	0.039595456	0.639	0.77	MESD	protein_coding	mesoderm c		15	80946289	80989828
ENSG0000027933	-0.582661362	0.639	0.77	AC090772.4	TEC	TEC		18	24135468	24135829
ENSG0000007047	0.053172642	0.639	0.77	ZXDC	protein_coding	ZXD family z		3	126437601	126475891
ENSG0000027513	0.062888824	0.639	0.77	AC241952.1	transcribed_un	phosphodie		1	120489860	120701289
ENSG0000017063	0.070882905	0.64	0.77	ARMC10	protein_coding	armadillo re		7	103074881	103099759
ENSG0000027774	-0.598220023	0.64	0.77	AC011462.4	lncRNA	novel transc		19	41373971	41374419
ENSG0000013973	-0.083265545	0.64	0.77	SLAIN1	protein_coding	SLAIN motif		13	77697687	77764242
ENSG0000022244	0.696150212	0.64	0.77	RN7SKP150	misc_RNA	RN7SK pseu		5	179845832	179846152
ENSG0000023559	-0.243646139	0.64	0.77	GNAS-AS1	lncRNA	GNAS antise		20	58818919	58850903
ENSG0000021399	0.415537596	0.64	0.77	MEF2B	protein_coding	myocyte en		19	19145567	19192131

ENSG0000016034	-0.300270843	0.64	0.77	C9orf116	protein_coding	chromosom		9	135495181	135501734
ENSG0000027282	0.714736127	0.64	0.77	AL445423.1	lncRNA	novel transc		1	220828676	220829211
ENSG0000022636	-0.169889739	0.641	0.77	RPL10AP6	processed_pse	ribosomal p		3	61742455	61743106
ENSG0000026800	-0.121100453	0.641	0.77	PTOV1-AS1	lncRNA	PTOV1 antis		19	49838639	49851676
ENSG0000010118	0.157685169	0.641	0.77	HRH3	protein_coding	histamine re		20	62214960	62220278
ENSG0000027707	-0.157785213	0.641	0.77	RPS9	protein_coding	ribosomal p	CHR_HSCHR		54201473	54208260
ENSG0000026287	0.088139133	0.641	0.77	AC005670.1	lncRNA	novel transc		17	46983287	47100323
ENSG0000018617	0.039172455	0.641	0.77	BCL9L	protein_coding	BCL9 like [S		11	118893875	118925608
ENSG0000014502	0.164976942	0.641	0.77	AMT	protein_coding	aminomethy		3	49416778	49422685
ENSG0000025513	0.604041196	0.641	0.77	AP000442.1	lncRNA	novel transc		11	59565923	59639861
ENSG0000025316	0.936828265	0.642	0.77	AC131281.1	unprocessed_p	family with s		8	202660	202897
ENSG0000026135	-0.260743322	0.642	0.77	AC116913.1	lncRNA	novel transc		15	66488658	66492109
ENSG0000023056	-0.223143922	0.642	0.77	ZNF32-AS2	lncRNA	ZNF32 antis		10	43645942	43648019
ENSG0000015379	0.148386893	0.642	0.77	C7orf31	protein_coding	chromosom		7	25134692	25180356
ENSG0000026693	-0.493190447	0.642	0.77	NA	NA	NA	NA	NA	NA	
ENSG0000027230	-0.503694308	0.642	0.77	NA	NA	NA	NA	NA	NA	
ENSG0000027510	0.374373396	0.642	0.77	NA	NA	NA	NA	NA	NA	
ENSG0000022236	0.75241153	0.643	0.77	SNORD12B	snoRNA	small nucleo		20	49280319	49280409
ENSG0000023797	-0.674977864	0.643	0.77	EIF4HP2	processed_pse	eukaryotic t		22	30902219	30902781
ENSG0000027758	-0.025106019	0.643	0.77	NEFL	protein_coding	neurofilame		8	24950955	24956721
ENSG0000011462	0.106152315	0.643	0.77	ABTB1	protein_coding	ankyrin repe		3	127672935	127680926
ENSG0000018485	0.095079154	0.643	0.77	TMEM186	protein_coding	transmembr		16	8780384	8797642
ENSG0000021425	0.041818555	0.643	0.77	FIS1	protein_coding	fission, mito		7	101239458	101252316
ENSG0000027008	0.600398817	0.643	0.77	AL021878.2	lncRNA	novel transc		22	42089630	42090028
ENSG0000026085	0.188382987	0.643	0.77	AC109460.2	lncRNA	novel transc		16	28952816	28966883
ENSG0000023935	0.885827291	0.643	0.77	NA	NA	NA	NA	NA	NA	
ENSG0000017846	0.164533438	0.644	0.77	P4HTM	protein_coding	prolyl 4-hyd		3	48989889	49007153
ENSG0000020438	0.137496337	0.644	0.77	NEU1	protein_coding	neuraminida		6	31857659	31862822
ENSG0000023741	0.66937161	0.644	0.77	NRXN2-AS	lncRNA	NRXN2 anti		11	64646399	64659681
ENSG0000024020	-0.342863717	0.644	0.77	AC080013.1	lncRNA	novel transc		3	158732263	158784070
ENSG0000014598	0.09392591	0.644	0.77	FARS2	protein_coding	phenylalany		6	5261044	5829192
ENSG0000027578	0.496447859	0.644	0.77	NA	NA	NA	NA	NA	NA	
ENSG0000002486	0.077608058	0.644	0.77	CCDC28A	protein_coding	coiled-coil c		6	138773509	138793319
ENSG0000016828	0.041253272	0.644	0.77	MGAT2	protein_coding	alpha-1,6-m		14	49620799	49623481
ENSG0000026168	0.300975432	0.644	0.77	NA	NA	NA	NA	NA	NA	
ENSG0000025047	-0.439270821	0.644	0.77	GMPSP1	processed_pse	guanine mo		4	8174421	8174999
ENSG0000027191	0.231631115	0.644	0.77	AC034236.2	lncRNA	novel transc		5	116083807	116085416
ENSG0000017532	-0.090400126	0.645	0.77	ZNF519	protein_coding	zinc finger p		18	14057457	14132490
ENSG0000025911	-0.383130013	0.645	0.77	AL049869.3	lncRNA	novel transc		14	64513952	64540368
ENSG0000026735	-0.430222352	0.645	0.77	RPL9P29	processed_pse	ribosomal p		17	78845953	78846868
ENSG0000026545	-0.687855097	0.645	0.77	AC012447.1	lncRNA	novel transc		2	121530422	121532705
ENSG0000025965	0.402768797	0.645	0.77	AC027559.1	transcribed_un	putative ubi		15	101732201	101746870
ENSG0000020579	0.275550128	0.645	0.77	LOH12CR2	lncRNA	loss of heter		12	12355406	12357067
ENSG0000017391	-0.038278956	0.646	0.77	ATP5MD	protein_coding	ATP synthas		10	103389041	103396492
ENSG0000023774	-0.936076906	0.646	0.77	AL034379.1	processed_pse	ribosomal p		1	37556247	37556499
ENSG0000010041	0.048027945	0.646	0.77	PHF5A	protein_coding	PHD finger		22	41459717	41468692
ENSG0000020233	0.744393325	0.646	0.77	RNU6-8	snRNA	RNA, U6 sm		14	32203163	32203269
ENSG0000021935	0.824509759	0.646	0.77	RPL31P52	processed_pse	ribosomal p		12	120450437	120450803
ENSG0000025706	0.446936305	0.646	0.77	AL049844.1	protein_coding	novel protei		6	143807092	143844617
ENSG0000027563	0.744393325	0.646	0.77	AC021755.3	lncRNA	novel transc		15	40078892	40079347
ENSG0000027851	0.343664398	0.646	0.77	LENG1	protein_coding	leukocyte re	CHR_HSCHR		54156107	54160227
ENSG0000023502	-0.60120928	0.646	0.77	AC097468.2	lncRNA	novel transc		2	218944629	218961903
ENSG0000028014	0.384428418	0.646	0.77	AC004877.2	TEC	TEC		7	149851572	149852170
ENSG0000011447	-0.149104741	0.646	0.77	IQCG	protein_coding	IQ motif cor		3	197889077	197960142
ENSG0000023002	-0.56271923	0.647	0.77	AL669831.3	transcribed_pr	septin 14 (S		1	586071	827796
ENSG0000023006	-0.381815488	0.647	0.77	LRRC37A15	processed_pse	leucine rich		4	102727274	102730721
ENSG0000021713	-0.370041156	0.647	0.77	AL139100.1	processed_pse	ribosomal p		6	34744176	34744673
ENSG0000022479	-0.512870702	0.647	0.77	AL139397.1	lncRNA	novel transc	X		56973510	56974796

ENSG0000015585	0.034810516	0.647	0.77	SLC26A2	protein_coding	solute carrie	5	149960758	149993455
ENSG0000027547	0.853225185	0.647	0.77	AC087741.1	lncRNA	novel transc	17	80149627	80149798
ENSG0000022632	0.071434313	0.647	0.77	NUP50-DT	lncRNA	NUP50 dive	22	45133020	45163781
ENSG0000024856	0.771457939	0.648	0.77	AC079140.1	processed_pse	novel pseuc	4	338097	338583
ENSG0000014743	-0.194303567	0.648	0.77	GNRH1	protein_coding	gonadotrop	8	25419258	25424654
ENSG0000017112	0.407724064	0.648	0.77	FUT3	protein_coding	fucosyltrans	19	5842888	5851474
ENSG0000021383	0.648088995	0.648	0.77	CFL1P5	processed_pse	cofilin 1 pse	5	69313371	69313872
ENSG0000024039	-0.506235285	0.648	0.77	AC004801.1	transcribed_pr	ribosomal p	12	48054813	48055591
ENSG0000027354	-0.697631914	0.648	0.77	AL137060.1	misc_RNA		13	50104833	50105174
ENSG0000017742	0.195953278	0.648	0.77	MIEF2	protein_coding	mitochondr	17	18260597	18266552
ENSG0000011954	-0.045048728	0.648	0.77	ONECUT2	protein_coding	one cut hon	18	57435374	57491298
ENSG0000027383	-0.328181079	0.648	0.77	LRRC56	protein_coding	leucine rich	CHR_HSCHR	537527	554915
ENSG0000017217	-0.09816363	0.648	0.77	MALT1	protein_coding	MALT1 para	18	58671465	58754477
ENSG0000013258	-0.050122301	0.648	0.78	FLOT2	protein_coding	flotillin 2 [Sc	17	28879335	28897733
ENSG0000027673	0.636701775	0.649	0.78	NA	NA	NA	NA	NA	NA
ENSG0000023619	-0.540232138	0.649	0.78	AL359076.1	lncRNA	novel transc	9	2041900	2046023
ENSG0000009092	0.049995076	0.649	0.78	PLEKHG2	protein_coding	pleckstrin ho	19	39412669	39428415
ENSG0000011855	0.301264399	0.649	0.78	PMFBP1	protein_coding	polyamine r	16	72112157	72176878
ENSG0000022375	0.18610263	0.649	0.78	ATAT1	protein_coding	alpha tubuli	CHR_HSCHR	30616685	30636664
ENSG0000022474	-0.534808171	0.649	0.78	AC063965.1	lncRNA	novel transc	10	87878692	87880427
ENSG0000025171	1.051283464	0.649	0.78	RNU7-20P	snRNA	RNA, U7 sm	7	148821012	148821073
ENSG0000026989	-0.38012934	0.649	0.78	COMMD3-	protein_coding	COMMD3-E	10	22316388	22329542
ENSG0000016230	0.077589133	0.649	0.78	ZFPL1	protein_coding	zinc finger p	11	65084210	65088398
ENSG0000011513	0.071814815	0.65	0.78	DNAJC27	protein_coding	DnaJ heat sl	2	24943636	24972094
ENSG0000020513	-0.122825323	0.65	0.78	SDHAF1	protein_coding	succinate de	19	35995188	35996312
ENSG0000010160	-0.048034434	0.651	0.78	MYL12A	protein_coding	myosin light	18	3247481	3256236
ENSG0000017247	-0.198134449	0.651	0.78	RAB40A	protein_coding	RAB40A, me	X	103499130	103519489
ENSG0000023511	-2.062117708	0.651	0.78	AL138895.1	lncRNA	novel transc	9	114396724	114398503
ENSG0000023784	0.664328053	0.651	0.78	FAM21FP	transcribed_un	family with s	10	45706431	45727231
ENSG0000024275	0.793057973	0.651	0.78	RHOT1P3	processed_pse	ras homolog	13	18837768	18838098
ENSG0000024171	0.240229223	0.651	0.78	LY6G5B	protein_coding	lymphocyte	CHR_HSCHR	31656528	31660956
ENSG0000023045	0.164643005	0.651	0.78	ANKRD18B	protein_coding	ankyrin repe	9	33524394	33573009
ENSG0000024987	0.584546645	0.652	0.78	AC116616.1	lncRNA	novel transc	4	174354854	174376445
ENSG0000011184	0.046447599	0.652	0.78	PAK1IP1	protein_coding	PAK1 intera	6	10694972	10709782
ENSG0000012157	-0.049708052	0.652	0.78	B4GALT4	protein_coding	beta-1,4-ga	3	119211732	119240946
ENSG0000023545	0.375248684	0.652	0.78	HAUS6P3	processed_pse	HAUS augm	7	53862233	53863339
ENSG0000027765	0.247147967	0.653	0.78	NA	NA	NA	NA	NA	NA
ENSG0000020577	-0.25705373	0.653	0.78	CATSPER2P	transcribed_un	cation chan	15	43726918	43747094
ENSG0000026154	-0.277610263	0.653	0.78	AC011978.1	lncRNA	novel transc	8	63215981	63218034
ENSG0000011818	0.019236691	0.653	0.78	RPS25	protein_coding	ribosomal p	11	119015712	119018691
ENSG0000013819	-0.11822062	0.653	0.78	PLCE1	protein_coding	phospholipa	10	93993931	94332823
ENSG0000015500	-0.050097531	0.653	0.78	APOOL	protein_coding	apolipoprot	X	85003877	85093316
ENSG0000015877	-0.039804421	0.653	0.78	USF1	protein_coding	upstream tr	1	161039251	161045977
ENSG0000010859	0.037935624	0.653	0.78	FTSJ3	protein_coding	FtsJ RNA 2'-	17	63819433	63830012
ENSG0000027835	0.736293312	0.653	0.78	NA	NA	NA	NA	NA	NA
ENSG0000018091	0.235366852	0.653	0.78	OXTR	protein_coding	oxytocin rec	3	8750408	8769628
ENSG0000017427	0.108515251	0.654	0.78	ZNHIT2	protein_coding	zinc finger H	11	65116403	65117701
ENSG0000022118	1.033222217	0.654	0.78	NA	NA	NA	NA	NA	NA
ENSG0000027401	1.033222217	0.654	0.78	NA	NA	NA	NA	NA	NA
ENSG0000022542	0.240535747	0.654	0.78	RBMS1P1	processed_pse	RNA binding	12	66234079	66235234
ENSG0000021386	0.139356795	0.654	0.78	NA	NA	NA	NA	NA	NA
ENSG0000021370	0.201610353	0.654	0.78	HMGB1P10	processed_pse	high mobilit	22	26560526	26561088
ENSG0000023536	0.418001503	0.654	0.78	SNRPGP10	processed_pse	small nuclea	1	205351247	205351471
ENSG0000027816	0.605213714	0.654	0.78	NA	NA	NA	NA	NA	NA
ENSG0000027950	-0.327925323	0.654	0.78	AD001527.1	TEC	TEC	19	36114362	36115146
ENSG0000019738	-0.062328183	0.655	0.78	ADARB1	protein_coding	adenosine c	21	45073853	45226560
ENSG0000021842	-0.148226994	0.655	0.78	AL590867.2	processed_pse	60S ribosom	6	153282287	153282733
ENSG0000022731	0.516118445	0.655	0.78	DDAH2	protein_coding	dimethylarg	CHR_HSCHR	31709206	31712785

ENSG0000023940	0.289754108	0.655	0.78	TMED10P2	processed_pse	transmembr	3	128538020	128538631
ENSG0000015816	-0.039940231	0.655	0.78	EYA3	protein_coding	EYA transcri	1	27970344	28088637
ENSG0000021364	-0.435719222	0.656	0.78	EEF1DP4	processed_pse	eukaryotic t	7	64862999	64864370
ENSG0000027330	0.65573432	0.656	0.78	AC024560.3	lncRNA	novel transc	3	197645669	197646017
ENSG0000011959	0.023983739	0.656	0.78	YLPM1	protein_coding	YLP motif co	14	74763316	74859435
ENSG0000013080	0.040133978	0.656	0.78	ZNF317	protein_coding	zinc finger p	19	9140397	9163424
ENSG0000023161	-0.369276408	0.656	0.78	NA	NA	NA	NA	NA	NA
ENSG0000026710	-0.11260706	0.656	0.78	ZNF561-AS	lncRNA	ZNF561 ant	19	9621249	9647203
ENSG0000012514	0.858424173	0.656	0.78	MT1G	protein_coding	metallothion	16	56666730	56668065
ENSG0000027998	0.448257244	0.656	0.78	AC011815.3	TEC	TEC	18	35257040	35257512
ENSG0000012031	0.046442584	0.656	0.78	WDR55	protein_coding	WD repeat d	5	140664676	140674124
ENSG0000013117	-0.033272821	0.656	0.78	COX7B	protein_coding	cytochrome	X	77899440	77907376
ENSG0000018836	0.381622859	0.656	0.78	AC092171.1	lncRNA	novel transc	7	5419846	5423122
ENSG0000022688	-0.413953409	0.656	0.78	E2F6P3	processed_pse	E2F transcrip	22	21268161	21268903
ENSG0000016260	-0.069660511	0.657	0.78	TM2D1	protein_coding	TM2 domain	1	61681046	61725423
ENSG0000024854	0.452544912	0.657	0.78	ANP32C	processed_pse	acidic nucle	4	164197007	164197711
ENSG0000027413	0.175240885	0.657	0.78	MYOM2	protein_coding	myomesin 2	CHR_HSCHR	2044989	2165303
ENSG0000021477	0.310962544	0.657	0.78	AL161756.1	lncRNA	novel transc	14	64329431	64338639
ENSG0000027070	-0.550504887	0.657	0.78	NA	NA	NA	NA	NA	NA
ENSG0000027596	-0.580940467	0.657	0.78	AC008115.4	lncRNA	novel transc	12	12648939	12649713
ENSG0000022889	0.2851147	0.657	0.78	AGPAT1	protein_coding	1-acylglycer	CHR_HSCHR	32175884	32185768
ENSG0000026981	0.568783834	0.657	0.78	AC010463.3	lncRNA	novel transc	19	17296196	17296695
ENSG0000026745	0.418877196	0.658	0.78	ZNF582-AS	lncRNA	ZNF582 ant	19	56393656	56399172
ENSG0000027518	0.193401442	0.658	0.78	AC048341.2	lncRNA	novel transc	12	62603909	62604399
ENSG0000026039	-0.383331482	0.658	0.78	NA	NA	NA	NA	NA	NA
ENSG0000027742	0.195838552	0.658	0.78	ZNF280B	protein_coding	zinc finger p	CHR_HSCHR	22493526	22518259
ENSG0000025659	-0.130039943	0.658	0.78	AC010186.2	transcribed_un	C-type lecti	12	9648047	9658412
ENSG0000027041	-0.342223422	0.659	0.78	CAHM	lncRNA	colon adeno	6	163413065	163413960
ENSG0000027680	-0.081752959	0.659	0.78	HERC2	protein_coding	HECT and R	CHR_HSCHR	28110217	28435080
ENSG0000025339	0.38238575	0.659	0.78	AC104561.1	lncRNA	novel transc	8	23458601	23484971
ENSG0000027629	0.779121087	0.659	0.78	AC234782.3	unprocessed_p	HYDIN, axon	X	103909126	103909449
ENSG0000011405	-0.050643546	0.659	0.78	PCCB	protein_coding	propionyl-C	3	136250340	136337896
ENSG0000012584	-0.055712517	0.659	0.78	NRSN2	protein_coding	neurensin 2	20	346782	359660
ENSG0000021312	-0.121863078	0.66	0.78	TCTEX1D2	protein_coding	Tctex1 dom	3	196291219	196318299
ENSG0000022118	-0.416486463	0.66	0.78	AL513534.1	miRNA		10	68759318	68759414
ENSG0000026070	-0.174713339	0.66	0.78	AL118516.1	lncRNA	novel transc	22	46761894	46762563
ENSG0000027301	-0.162835548	0.66	0.78	FAM106A	lncRNA	family with s	17	18511221	18551705
ENSG0000023764	0.555646332	0.66	0.78	RPL28P2	processed_pse	ribosomal p	2	231789481	231789887
ENSG0000024855	0.504917144	0.66	0.78	C5orf34-AS	lncRNA	C5orf34 ant	5	43511058	43521811
ENSG0000027033	0.555646332	0.66	0.78	SMC2-AS1	lncRNA	SMC2 antise	9	104080024	104093073
ENSG0000024924	0.409938437	0.66	0.78	AC010226.1	lncRNA	novel transc	5	115602057	115623897
ENSG0000012177	-0.020926035	0.66	0.78	KHDRBS1	protein_coding	KH RNA bin	1	32013868	32060850
ENSG0000023590	-0.438687599	0.66	0.78	RBMS3-AS3	lncRNA	RBMS3 antis	3	29054570	29290726
ENSG0000016856	0.093579003	0.66	0.78	TMEM223	protein_coding	transmembr	11	62771629	62792006
ENSG0000023937	-0.624328513	0.66	0.78	AC073111.1	lncRNA	novel transc	7	150379854	150383885
ENSG0000020738	-0.628470673	0.66	0.78	Y_RNA	misc_RNA	Y RNA [Sou	2	112579484	112579584
ENSG0000024421	-0.676562054	0.66	0.78	RN7SL81P	misc_RNA	RNA, 7SL, cy	7	128761338	128761598
ENSG0000013428	-0.03029418	0.661	0.78	PPHLN1	protein_coding	periphilin 1	12	42238447	42459715
ENSG0000023092	-0.759450664	0.661	0.78	AC099513.1	processed_pse	ribosomal p	5	126628019	126628319
ENSG0000026075	0.222764795	0.661	0.78	AC008870.2	lncRNA	novel transc	16	23568673	23569696
ENSG0000027508	-0.38853543	0.661	0.78	TFPT	protein_coding	TCF3 fusion	CHR_HSCHR	54107066	54115801
ENSG0000017519	0.04523117	0.661	0.78	PARL	protein_coding	presenilin as	3	183829271	183884933
ENSG0000017692	0.228737542	0.662	0.79	EFCAB5	protein_coding	EF-hand cal	17	29929200	30108452
ENSG0000026009	0.474375065	0.662	0.79	AC106820.3	lncRNA	novel transc	16	2452581	2452977
ENSG0000014474	-0.03456314	0.662	0.79	TMF1	protein_coding	TATA eleme	3	69019827	69052339
ENSG0000022377	-0.180048639	0.663	0.79	SUMO2P1	processed_pse	SUMO2 pse	CHR_HSCHR	29632655	29632938
ENSG0000018478	0.288658518	0.663	0.79	SMIM10	protein_coding	small integr	X	134990991	134992473
ENSG0000021950	-0.479902123	0.663	0.79	FTH1P8	processed_pse	ferritin heav	X	148052233	148052746

ENSG0000024179	0.442101331	0.663	0.79	ENO1P4	processed_pse	enolase 1 ps	2	201621646	201623430
ENSG0000027820	-0.251898018	0.663	0.79	AC243919.2	lncRNA	novel transc	15	82418651	82434235
ENSG0000023072	0.251513332	0.663	0.79	LINC01001	lncRNA	long interge	11	127204	139612
ENSG0000010346	-0.169162414	0.664	0.79	TOX3	protein_coding	TOX high m	16	52436417	52547802
ENSG0000014575	0.364046739	0.664	0.79	SPATA9	protein_coding	spermatoge	5	95652181	95698711
ENSG0000016634	-0.087951623	0.664	0.79	CYB5A	protein_coding	cytochrome	18	74250846	74291973
ENSG0000021016	-0.092985578	0.664	0.79	MT-TG	Mt_tRNA	mitochondr	MT	9991	10058
ENSG0000007431	-0.361261658	0.664	0.79	SNCB	protein_coding	synuclein be	5	176620082	176630556
ENSG0000023084	0.152561367	0.664	0.79	ZNF674-AS	lncRNA	ZNF674 ant	X	46545438	46548841
ENSG0000022668	0.410784405	0.664	0.79	ENTPD1-AS	lncRNA	ENTPD1 ant	10	95732976	96090250
ENSG0000027203	0.361268441	0.664	0.79	AP002907.1	lncRNA	novel transc	8	102256392	102257821
ENSG0000022688	0.089078449	0.665	0.79	GNL1	protein_coding	G protein nu	CHR_HSCHR	30533598	30549394
ENSG0000022610	0.254776828	0.665	0.79	SEPTIN7P3	unprocessed_p	septin 7 pse	7	35946294	35972587
ENSG0000005461	-0.073654468	0.665	0.79	TBC1D22A	protein_coding	TBC1 doma	22	46762617	47175699
ENSG0000010210	0.065481101	0.665	0.79	SLC35A2	protein_coding	solute carrie	X	48903180	48911958
ENSG0000027377	0.060318621	0.665	0.79	SMN2	protein_coding	survival of n	CHR_HSCHR	70070287	70099184
ENSG0000022734	0.037250094	0.665	0.79	PARG	protein_coding	poly(ADP-ri	10	49818279	49970203
ENSG0000021947	-0.514406359	0.665	0.79	AL355802.1	processed_pse	40S ribosom	6	43538822	43539703
ENSG0000012032	-0.289990073	0.666	0.79	PCDHB8	protein_coding	protocadhe	5	141177790	141180539
ENSG0000010953	0.066054373	0.666	0.79	FRG1	protein_coding	FSHD regior	4	189940870	189963202
ENSG0000027010	0.224631368	0.666	0.79	AL049840.7	lncRNA	novel transc	14	103687576	103688127
ENSG0000011474	-0.045699427	0.666	0.79	WDR48	protein_coding	WD repeat c	3	39052013	39096671
ENSG0000003006	0.026134419	0.666	0.79	NUP160	protein_coding	nucleoporin	11	47778087	47848555
ENSG0000011490	0.089844504	0.667	0.79	NEK4	protein_coding	NIMA relate	3	52708444	52770946
ENSG0000023649	0.559030267	0.667	0.79	AC107081.1	lncRNA	novel transc	2	61868432	61886082
ENSG0000023079	0.208379882	0.667	0.79	YY2	protein_coding	YY2 transcri	X	21855987	21858740
ENSG0000008511	-0.256300209	0.667	0.79	CD82	protein_coding	CD82 molec	11	44564427	44620358
ENSG0000023110	-0.403912741	0.667	0.79	AC022395.1	lncRNA	novel transc	10	118017487	118045810
ENSG0000025858	-0.472529109	0.668	0.79	CR753842.1	protein_coding	novel protei	CHR_HSCHR	31648058	31654629
ENSG0000012400	-0.493877924	0.668	0.79	MOGAT1	protein_coding	monoacylgly	2	222671658	222709930
ENSG0000018758	-0.5875927	0.668	0.79	COX8C	protein_coding	cytochrome	14	93347182	93348356
ENSG0000023461	-0.5875927	0.668	0.79	C2CD4D-A	lncRNA	C2CD4D an	1	151841877	151850385
ENSG0000025348	0.536940446	0.668	0.79	AC104561.2	processed_pse	DnaJ (Hsp40	8	23483016	23483534
ENSG0000025945	0.22936667	0.668	0.79	ADNP-AS1	lncRNA	ADNP antise	20	50930984	50945148
ENSG0000027695	-0.718962183	0.668	0.79	AC109309.2	processed_pse	mastermind	11	3289311	3289642
ENSG0000011969	0.224934779	0.668	0.79	TGFB3	protein_coding	transforming	14	75958097	75983011
ENSG0000015169	-0.025350338	0.668	0.79	RNF144A	protein_coding	ring finger p	2	6917412	7068286
ENSG0000026035	0.676124458	0.669	0.79	AC012173.1	lncRNA	novel transc	16	8847650	8848724
ENSG0000027800	0.661903827	0.669	0.79	AL139099.3	unprocessed_p	phosphatidy	14	49601011	49601124
ENSG0000020566	-0.231852036	0.669	0.79	NA	NA	NA	NA	NA	NA
ENSG0000017172	-0.049209015	0.669	0.79	VAT1L	protein_coding	vesicle amir	16	77788564	77980107
ENSG0000027039	-0.208586365	0.669	0.79	PFN1P2	transcribed_pr	profilin 1 ps	1	120432204	120434052
ENSG0000027629	-0.089577613	0.669	0.79	FRG1HP	transcribed_un	FSHD regior	9	40992249	41218410
ENSG0000022771	-0.103876906	0.669	0.79	HLA-A	protein_coding	major histoc	CHR_HSCHR	30021043	30024386
ENSG0000026444	0.985904079	0.67	0.79	NA	NA	NA	NA	NA	NA
ENSG0000026532	0.985904079	0.67	0.79	MIR4263	miRNA	microRNA 4	2	27996367	27996449
ENSG0000027444	-0.023755677	0.67	0.79	PRPF8	protein_coding	pre-mRNA	CHR_HSCHR	1650629	1684882
ENSG0000025956	0.254979741	0.67	0.79	AC090607.2	transcribed_pr	zinc finger C	15	78290527	78291221
ENSG0000018690	0.030845803	0.67	0.79	ZDHHC17	protein_coding	zinc finger D	12	76763588	76853696
ENSG0000027183	0.31851241	0.67	0.79	AL445222.2	lncRNA	novel transc	9	127366770	127367397
ENSG0000023016	-0.338872282	0.67	0.79	AL122010.1	lncRNA	novel transc	1	34850694	34851555
ENSG0000021017	-0.077382096	0.67	0.79	MT-TR	Mt_tRNA	mitochondr	MT	10405	10469
ENSG0000025625	0.565447254	0.67	0.79	AC073912.1	lncRNA	novel transc	12	130810606	130812438
ENSG0000026640	0.294633437	0.67	0.79	SNHG25	lncRNA	small nuclec	17	64145970	64146476
ENSG0000027882	-0.743650412	0.67	0.79	NA	NA	NA	NA	NA	NA
ENSG0000027896	0.325215756	0.67	0.79	AC026310.1	TEC	novel transc	12	24902309	24904681
ENSG0000016583	0.577138819	0.67	0.79	ERICH6B	protein_coding	glutamate r	13	45534522	45615739
ENSG0000019917	-0.811354703	0.671	0.79	MIR331	miRNA	microRNA 3	12	95308420	95308513

ENSG0000027945	-0.17273763	0.671	0.79	Z99129.4	TEC	TEC	6	122436789	122439223
ENSG0000027820	0.602460309	0.671	0.79	NA	NA	NA	NA	NA	NA
ENSG0000026026	0.106168911	0.671	0.79	SNHG19	lncRNA	small nucleo	16	2154797	2155358
ENSG0000027687	-0.061543746	0.671	0.79	MYT1	protein_coding	myelin trans	CHR_HSCHR	64155437	64245898
ENSG0000023879	-0.338879338	0.671	0.79	SCARNA12	snoRNA	small Cajal b	12	6967337	6967606
ENSG0000022779	0.283064608	0.672	0.79	RPS18	protein_coding	ribosomal p	CHR_HSCHR	33412156	33415676
ENSG0000017922	0.021688174	0.672	0.79	MAGED1	protein_coding	MAGE famil	X	51803007	51902354
ENSG0000011359	-0.04257282	0.672	0.79	TRIM23	protein_coding	tripartite mo	5	65589690	65625975
ENSG0000023989	-0.769465831	0.672	0.79	RN7SL674P	misc_RNA	RNA, 7SL, cy	2	11584773	11585047
ENSG0000022413	0.28786539	0.672	0.79	AC000123.1	lncRNA	novel transc	7	127350128	127351523
ENSG0000023040	0.170229125	0.672	0.79	TCEA1P2	processed_pse	transcription	3	37275693	37276598
ENSG0000010487	-0.052228493	0.672	0.79	PIH1D1	protein_coding	PIH1 domai	19	49446298	49453497
ENSG0000020151	0.799887074	0.673	0.79	SNORA71C	snoRNA	small nucleo	20	38429670	38429803
ENSG0000023996	0.403954866	0.673	0.79	AC091390.4	transcribed_un	uncharacter	7	102364162	102380633
ENSG0000027232	0.150824579	0.673	0.79	NA	NA	NA	NA	NA	NA
ENSG0000027657	-0.382127595	0.673	0.79	AC002550.2	lncRNA	novel transc	16	19694035	19694428
ENSG0000025478	0.184523663	0.673	0.79	CKLF-CMTI	protein_coding	CKLF-CMTM	16	66552587	66579135
ENSG0000027242	-0.238383824	0.673	0.79	AC009902.3	lncRNA	novel transc	8	81275399	81277570
ENSG0000025994	-0.268792967	0.673	0.79	BX005019.1	lncRNA	novel transc	1	97967005	97968814
ENSG0000016263	-0.230932245	0.673	0.79	B3GALT2	protein_coding	beta-1,3-ga	1	193178730	193186613
ENSG0000019971	0.525597032	0.674	0.79	RN7SKP74	misc_RNA	RN7SK pseu	20	18295143	18295451
ENSG0000027014	-0.42227798	0.674	0.79	AC068620.2	lncRNA	novel transc	4	56396312	56396871
ENSG0000026028	0.508071022	0.674	0.79	ITFG1-AS1	lncRNA	ITFG1 antise	16	47144323	47162749
ENSG0000024315	-0.304167576	0.674	0.79	AL162431.1	lncRNA	novel transc	1	180944042	180976482
ENSG0000026040	-0.073572474	0.674	0.79	AC110079.1	transcribed_un	general tran	4	118591773	118633729
ENSG0000027218	0.521948631	0.675	0.79	AP003392.5	lncRNA	novel transc	11	119067374	119067698
ENSG0000023839	0.600890041	0.675	0.79	AP000254.1	snoRNA		21	31664306	31664482
ENSG0000022853	-0.14630635	0.675	0.79	AC005000.1	processed_pse	small ubiqui	X	115718942	115719229
ENSG0000023940	-0.174665723	0.675	0.79	Z68871.1	lncRNA	novel transc	X	102884414	102906158
ENSG0000000489	-0.0233313	0.675	0.8	CDC27	protein_coding	cell division	17	47117703	47189422
ENSG0000026371	-0.965437254	0.675	0.8	MIR4639	miRNA	microRNA 4	6	16141556	16141624
ENSG0000014275	-0.050034178	0.675	0.8	GPN2	protein_coding	GPN-loop G	1	26876132	26890283
ENSG0000005042	-0.036438287	0.675	0.8	LETMD1	protein_coding	LETM1 dom	12	51047962	51060424
ENSG0000024046	0.694798717	0.676	0.8	RPS19P3	processed_pse	ribosomal p	14	34568689	34569129
ENSG0000023221	-0.793621977	0.676	0.8	AL021937.2	unprocessed_p	Ral-GDS rel	22	32386668	32386868
ENSG0000011819	0.027433821	0.676	0.8	KIF14	protein_coding	kinesin fami	1	200551497	200620751
ENSG0000027433	0.131255746	0.676	0.8	LRRC37A2	protein_coding	leucine rich	CHR_HSCHR	45505209	45550562
ENSG0000026692	0.300781488	0.676	0.8	AC006213.1	lncRNA	novel transc	19	43996896	44003298
ENSG0000023686	0.398407824	0.676	0.8	BET1-AS1	lncRNA	BET1 antise	7	93969442	94011113
ENSG0000026990	0.398407824	0.676	0.8	AL606834.1	lncRNA	novel transc	14	50662511	50663178
ENSG0000022104	-0.893488232	0.677	0.8	U3	snoRNA	Small nucleo	17	44025020	44025223
ENSG0000027776	0.286696634	0.677	0.8	NA	NA	NA	NA	NA	NA
ENSG0000023803	-0.524637787	0.677	0.8	AC244197.2	lncRNA	novel transc	X	149511509	149526264
ENSG0000007529	0.021160167	0.678	0.8	ZNF638	protein_coding	zinc finger p	2	71276561	71435069
ENSG0000010324	-0.032366251	0.678	0.8	CLCN7	protein_coding	chloride vol	16	1444934	1475084
ENSG0000011240	0.049648368	0.678	0.8	HECA	protein_coding	hdc homolo	6	139135080	139180802
ENSG0000027378	0.570488501	0.678	0.8	AC020658.4	lncRNA	novel transc	15	40226386	40227069
ENSG0000022721	0.272164602	0.679	0.8	PFN1P6	processed_pse	profilin 1 ps	1	144442606	144443004
ENSG0000022981	0.296063753	0.679	0.8	DDX50P1	processed_pse	DEAD-box I	2	32201600	32203999
ENSG0000012422	0.049502704	0.679	0.8	PMEPA1	protein_coding	prostate tra	20	57648392	57711536
ENSG0000020130	0.463193257	0.679	0.8	SNORA65	snoRNA	small nucleo	9	127448501	127448630
ENSG0000023694	-0.562911741	0.679	0.8	NA	NA	NA	NA	NA	NA
ENSG0000025819	0.219742928	0.679	0.8	AC073896.4	lncRNA	novel transc	12	56162359	56190284
ENSG0000026027	-0.731189453	0.679	0.8	AC009019.1	lncRNA	novel transc	16	22083256	22092485
ENSG0000018069	0.042960719	0.679	0.8	TMEM64	protein_coding	transmembr	8	90621995	90791632
ENSG0000021427	-0.220311374	0.68	0.8	SCART1	protein_coding	scavenger re	10	133453928	133523558
ENSG0000025868	0.324484492	0.68	0.8	AL132989.1	lncRNA	novel transc	14	58264662	58269681
ENSG0000012543	0.155175089	0.68	0.8	SLC25A35	protein_coding	solute carrie	17	8287763	8295400

ENSG0000024382	-0.374494018	0.68	0.8	RPL12P6	processed_pse	ribosomal p		14	34740336	34740833
ENSG0000025442	0.385180829	0.68	0.8	LINC02696	lncRNA	long interge		11	45355371	45367490
ENSG0000019820	0.063661009	0.68	0.8	RPS6KL1	protein_coding	ribosomal p		14	74903951	74923302
ENSG0000025132	0.357832018	0.68	0.8	LINC02728	lncRNA	long interge		11	78423982	78429836
ENSG0000020509	0.251669954	0.68	0.8	TMEM240	protein_coding	transmembr		1	1534778	1540624
ENSG0000025007	-0.414451955	0.68	0.8	AP000866.2	lncRNA	novel transc		11	124759129	124766119
ENSG0000010601	-0.313837897	0.68	0.8	ANKRD7	protein_coding	ankyrin repe		7	118214669	118496171
ENSG0000025890	-0.433317607	0.681	0.8	AL157871.5	lncRNA	novel transc		14	100263781	100265405
ENSG0000023013	-0.518006702	0.682	0.8	LINC02641	lncRNA	long interge		10	123356450	123537767
ENSG0000009984	0.317423957	0.682	0.8	RASSF7	protein_coding	Ras associat		11	560404	564025
ENSG0000027473	-0.077879934	0.682	0.8	ARHGAP11	protein_coding	Rho GTPase	CHR_HSCHR		30776860	30837039
ENSG0000002333	-0.052230163	0.682	0.8	ALAS1	protein_coding	5'-aminolev		3	52198086	52214327
ENSG0000018417	0.052427553	0.682	0.8	SCFD2	protein_coding	sec1 family		4	52872982	53366066
ENSG0000011759	0.022861508	0.682	0.8	PRDX6	protein_coding	peroxiredox		1	173477330	173488815
ENSG0000017454	0.036363956	0.682	0.8	MRPL11	protein_coding	mitochondr		11	66435075	66466738
ENSG0000022688	0.205852666	0.682	0.8	ERVMER34	protein_coding	endogenous		4	52722618	52751586
ENSG0000020177	0.63918479	0.682	0.8	SNORA5C	snoRNA	small nucleo		7	45104906	45105042
ENSG0000018566	0.28794373	0.683	0.8	SNAI3	protein_coding	snail family		16	88677688	88686507
ENSG0000025540	0.616429505	0.683	0.8	RSF1-IT1	lncRNA	RSF1 introni		11	77738680	77739568
ENSG0000025532	-0.325909828	0.683	0.8	AP000759.1	lncRNA	novel transc		11	66244840	66246239
ENSG0000018694	0.501123283	0.683	0.8	CHCHD2P9	processed_pse	coiled-coil-		9	79391304	79391759
ENSG0000016259	-0.036708334	0.683	0.8	NFIA	protein_coding	nuclear fact		1	60865259	61462788
ENSG0000025416	0.336711686	0.684	0.8	AC090739.1	lncRNA	novel transc		8	42537529	42538304
ENSG0000027943	0.251201235	0.684	0.8	AL049776.1	TEC	TEC		14	35086035	35088059
ENSG0000013663	-0.12816953	0.684	0.8	HLX	protein_coding	H2.0 like ho		1	220879431	220885059
ENSG0000012577	-0.212321285	0.684	0.8	SDCBP2	protein_coding	syndecan bi		20	1309909	1329139
ENSG0000017280	-0.06227895	0.684	0.8	RPL38	protein_coding	ribosomal p		17	74203582	74210655
ENSG0000027274	-0.545200346	0.684	0.8	AC004948.1	lncRNA	novel transc		7	7619872	7620543
ENSG0000026759	-0.654263184	0.684	0.8	AC060780.1	unprocessed_p	breast cance		17	43168170	43168249
ENSG0000015248	0.041738532	0.684	0.8	USP12	protein_coding	ubiquitin sp		13	27066156	27171811
ENSG0000027247	-0.230735458	0.685	0.8	AL024507.2	lncRNA	novel transc		6	107957413	107959986
ENSG0000017354	-0.058023705	0.685	0.8	GMPPB	protein_coding	GDP-manno		3	49716844	49723951
ENSG0000020462	-0.358215292	0.685	0.8	AC115618.1	lncRNA	novel transc	X		48568014	48574860
ENSG0000026165	-0.142312853	0.685	0.8	Z92544.1	lncRNA	novel transc		16	689001	692554
ENSG0000017975	0.261764614	0.685	0.8	APOBEC3B	protein_coding	apolipoprot		22	38982347	38992804
ENSG0000013845	-0.033767913	0.685	0.8	SLC35A5	protein_coding	solute carrie		3	112561709	112585579
ENSG0000020752	0.550475776	0.686	0.8	SNORA66	snoRNA	small nucleo		1	92840719	92840851
ENSG0000023396	0.314300491	0.686	0.8	UBE2SP1	processed_pse	ubiquitin co		17	15704232	15704900
ENSG0000020640	-0.140418176	0.686	0.8	ABHD16A	protein_coding	abhydrolase	CHR_HSCHR		31677180	31693655
ENSG0000023699	0.276503176	0.687	0.8	RPL12P12	processed_pse	ribosomal p		20	19823443	19823943
ENSG0000013750	-0.042967581	0.687	0.8	RAB30	protein_coding	RAB30, mer		11	82973133	83071923
ENSG0000011597	-0.030951151	0.687	0.8	THADA	protein_coding	THADA arm		2	43230836	43596046
ENSG0000015339	-0.09153682	0.687	0.8	INO80C	protein_coding	INO80 comp		18	35452230	35497979
ENSG0000011764	0.14852009	0.687	0.8	MAN1C1	protein_coding	mannosidas		1	25617468	25786207
ENSG0000026332	-0.136855509	0.687	0.8	TAPT1-AS1	lncRNA	TAPT1 antis		4	16226685	16320140
ENSG0000012860	0.322011597	0.688	0.8	IRF5	protein_coding	interferon re		7	128937457	128950038
ENSG0000017182	0.028983588	0.688	0.8	EXOSC10	protein_coding	exosome co		1	11066618	11099869
ENSG0000022839	0.234669455	0.688	0.8	LINC01004	lncRNA	long interge		7	104950315	105013044
ENSG0000023938	0.118388816	0.688	0.81	ALKBH6	protein_coding	alkB homolo		19	36009120	36014239
ENSG0000023135	0.273363281	0.688	0.81	WBP1P1	processed_pse	WBP1 pseu		2	86930250	86930754
ENSG0000026066	-0.311844013	0.689	0.81	AC093536.1	processed_pse	emopamil b		16	64260570	64261239
ENSG0000017587	0.129881631	0.689	0.81	NA	NA	NA	NA		NA	NA
ENSG0000028003	-0.625348212	0.689	0.81	NA	NA	NA	NA		NA	NA
ENSG0000016569	-0.028531223	0.689	0.81	TSC1	protein_coding	TSC comple		9	132891348	132946874
ENSG0000020111	0.702938853	0.689	0.81	Y_RNA	misc_RNA	Y RNA [Sou		9	19089593	19089688
ENSG0000025845	0.532092824	0.689	0.81	AL139099.2	lncRNA	novel transc		14	49629291	49629890
ENSG0000026190	0.153500591	0.689	0.81	AC217785.1	unprocessed_p	metaxin 1 (M	CHR_HSCHR		155225758	155229107
ENSG0000011376	0.053802657	0.689	0.81	ZNF346	protein_coding	zinc finger p		5	177022696	177081189

ENSG0000026446	0.456228383	0.69	0.81	MIR3648-2	miRNA	microRNA 3	21	8986999	8987178
ENSG0000020699	-0.943764694	0.69	0.81	RNU6-574I	snRNA	RNA, U6 sm	12	51987657	51987763
ENSG0000022112	-0.943764694	0.69	0.81	MIR1224	miRNA	microRNA 1	3	184241405	184241489
ENSG0000027204	-0.263256312	0.69	0.81	AC010245.2	lncRNA	novel transc	5	75608817	75609983
ENSG0000026836	-0.136801711	0.69	0.81	AC092279.1	lncRNA	novel transc	19	24033401	24077377
ENSG0000016476	-0.240683278	0.691	0.81	SBSPON	protein_coding	somatomed	8	73064543	73124088
ENSG0000023397	-0.263482713	0.691	0.81	AC138951.1	processed_pse	POM121 me	5	21481327	21484011
ENSG0000027158	0.461470969	0.691	0.81	AL583832.1	processed_pse	glycogenin	1	205091163	205091946
ENSG0000022778	-0.360072995	0.691	0.81	AC002553.1	lncRNA	novel transc	17	16040472	16041273
ENSG0000023419	0.519819342	0.691	0.81	ETV5-AS1	lncRNA	ETV5 antise	3	186079170	186080947
ENSG0000022604	-0.160596156	0.691	0.81	TLK2P1	processed_pse	tousled like	17	34036681	34039872
ENSG0000010586	0.081132851	0.692	0.81	DUS4L	protein_coding	dihydrourid	7	107563484	107578523
ENSG0000019933	0.836578535	0.692	0.81	AL354797.1	misc_RNA		9	109042386	109042487
ENSG0000021308	0.25619632	0.692	0.81	ACKR1	protein_coding	atypical che	1	159203307	159206500
ENSG0000022723	0.398366975	0.692	0.81	AL606534.1	lncRNA	novel transc	1	243135898	243140588
ENSG0000027440	0.360113725	0.692	0.81	AC090510.2	lncRNA	novel transc	15	42726583	42727211
ENSG0000027871	-0.273969175	0.692	0.81	TSEN34	protein_coding	tRNA splicir	CHR_HSCHR	54190866	54195141
ENSG0000027897	-0.070775073	0.692	0.81	HEIH	lncRNA	hepatocellu	5	180826871	180831605
ENSG0000016634	0.054618772	0.692	0.81	USP54	protein_coding	ubiquitin sp	10	73497538	73625953
ENSG0000027979	0.395515393	0.692	0.81	AC138907.1	TEC	novel transc	16	32787962	32788944
ENSG0000013941	0.209831123	0.693	0.81	SDSL	protein_coding	serine dehy	12	113422380	113438276
ENSG0000023281	-0.221353467	0.693	0.81	AL360270.1	lncRNA	novel transc	1	110943467	110952848
ENSG0000026493	0.783864739	0.693	0.81	AC100830.2	lncRNA	novel transc	15	64696622	64696861
ENSG0000017293	-0.036772554	0.693	0.81	OXSR1	protein_coding	oxidative str	3	38165089	38255484
ENSG0000016009	-0.041377528	0.693	0.81	ZNF362	protein_coding	zinc finger p	1	33256492	33300719
ENSG0000016099	0.071001442	0.693	0.81	SH2B2	protein_coding	SH2B adapt	7	102285091	102321711
ENSG0000025965	0.319577895	0.693	0.81	AC009996.1	lncRNA	novel transc	15	44557829	44559188
ENSG0000023642	0.13644607	0.693	0.81	PPP1R18	protein_coding	protein pho	CHR_HSCHR	30668637	30671637
ENSG0000025492	0.16988842	0.693	0.81	AL591684.2	lncRNA	novel transc	10	47517816	47553514
ENSG0000019800	-0.081393672	0.694	0.81	IRAK4	protein_coding	interleukin 1	12	43758944	43789543
ENSG0000027867	-0.469042363	0.694	0.81	NA	NA	NA	NA	NA	NA
ENSG0000019881	0.049124087	0.694	0.81	SFT2D1	protein_coding	SFT2 domai	6	166319728	166342576
ENSG0000025544	0.328358438	0.694	0.81	AP003072.2	processed_pse	aminoacyl t	11	93152075	93152750
ENSG0000016713	-0.276258475	0.694	0.81	CCDC103	protein_coding	coiled-coil c	17	44899142	44905390
ENSG0000017263	-0.105620927	0.694	0.81	EFEMP2	protein_coding	EGF contain	11	65866441	65873592
ENSG0000023569	0.408880818	0.695	0.81	PA2G4P2	processed_pse	proliferatior	20	12380056	12381255
ENSG0000011695	-0.035538722	0.695	0.81	NA	NA	NA	NA	NA	NA
ENSG0000017240	-0.059827841	0.695	0.81	CLP1	protein_coding	cleavage fac	11	57648992	57661865
ENSG0000025883	0.064278751	0.695	0.81	MC1R	protein_coding	melanocorti	16	89912119	89920973
ENSG0000027266	-0.818600138	0.695	0.81	AC090425.2	lncRNA	novel transc	3	179584271	179584410
ENSG0000011213	0.083247975	0.695	0.81	MDGA1	protein_coding	MAM doma	6	37630679	37699306
ENSG0000019895	0.019565758	0.695	0.81	SMG5	protein_coding	SMG5 nons	1	156249224	156282825
ENSG0000023965	0.283563803	0.695	0.81	GUSBP4	transcribed_un	GUSB pseuc	6	57919784	57930291
ENSG0000004913	0.049013545	0.695	0.81	KITLG	protein_coding	KIT ligand [S	12	88492793	88580851
ENSG0000008221	-0.041458442	0.695	0.81	ME2	protein_coding	malic enzym	18	50879080	50954257
ENSG0000024255	-0.410853093	0.696	0.81	AP001432.1	lncRNA	novel transc	21	37221419	37237744
ENSG0000026272	0.507171535	0.696	0.81	AC123768.2	lncRNA	novel transc	15	32585524	32615158
ENSG0000027305	-0.166664251	0.696	0.81	AC005046.1	lncRNA	novel transc	7	107942116	107942740
ENSG0000022727	0.106742595	0.696	0.81	RNF5	protein_coding	ring finger p	CHR_HSCHR	32186026	32188465
ENSG0000006887	-0.02184918	0.696	0.81	PSME4	protein_coding	proteasome	2	53864069	53970993
ENSG0000016580	0.320292897	0.696	0.81	PPP1R36	protein_coding	protein pho	14	64549920	64589381
ENSG0000016095	-0.393819378	0.697	0.81	PTGER1	protein_coding	prostagland	19	14472466	14475354
ENSG0000011084	-0.096602453	0.697	0.81	PRPF40B	protein_coding	pre-mRNA	12	49568218	49644666
ENSG0000023088	-0.15867872	0.697	0.81	SUMO2P1	processed_pse	SUMO2 pse	CHR_HSCHR	29635855	29636138
ENSG0000015062	-0.03918661	0.697	0.81	WDR17	protein_coding	WD repeat c	4	176065834	176182818
ENSG0000016081	-0.035605825	0.697	0.81	GPATCH4	protein_coding	G-patch do	1	156594301	156601496
ENSG0000024368	0.388334056	0.697	0.81	RPL37P23	processed_pse	ribosomal p	19	52143043	52143336
ENSG0000017702	-0.482106092	0.697	0.81	C19orf18	protein_coding	chromosom	19	57958437	57974534

ENSG0000022733	-0.064654784	0.697	0.81	EHMT2	protein_coding	euchromatic	CHR_HSCHR	31861940	31879868
ENSG0000011308	-0.155525302	0.698	0.81	LOX	protein_coding	lysyl oxidase	5	122063195	122078360
ENSG0000020328	0.550951865	0.698	0.81	Metazoa_SI	misc_RNA	Metazoan s	9	128745857	128746138
ENSG0000021419	0.141577795	0.698	0.81	UBE2V1P2	processed_pse	ubiquitin co	3	175718231	175718670
ENSG0000022472	0.366509052	0.697	0.81	IMPDH1P8	processed_pse	inosine mor	3	15878047	15879571
ENSG0000025515	0.221554623	0.698	0.81	EID3	protein_coding	EP300 intera	12	104303739	104305205
ENSG0000027560	0.5775008	0.698	0.81	NDUFA3	protein_coding	NADH:ubiqu	CHR_HSCHR	54102906	54107026
ENSG0000023807	-0.137550107	0.698	0.81	AC009244.1	processed_pse	cell division	7	129410113	129410370
ENSG0000008950	-0.238022171	0.698	0.81	CMTM1	protein_coding	CKLF like M	16	66566393	66579137
ENSG0000015076	0.026835597	0.698	0.81	DLAT	protein_coding	dihydrolipoa	11	112025408	112064404
ENSG0000025236	-0.809831875	0.698	0.81	RNU6-118P	snRNA	RNA, U6 sm	11	62815966	62816067
ENSG0000026158	0.72688828	0.698	0.81	AC093249.4	processed_pse	zinc finger p	16	30587662	30587920
ENSG0000022820	0.439092256	0.699	0.81	AL022341.1	lncRNA	novel transc	16	648473	649200
ENSG0000023606	0.183581886	0.699	0.81	ABHD16A	protein_coding	abhydrolase	CHR_HSCHR	31763228	31779696
ENSG0000020729	-0.765500477	0.699	0.81	RNU6-30P	snRNA	RNA, U6 sm	X	101634006	101634112
ENSG0000025334	-0.50696648	0.699	0.81	AC040934.1	lncRNA	novel transc	17	47409322	47423526
ENSG0000027797	0.568323101	0.699	0.81	AC010332.1	unprocessed_p	novel zinc fi	19	52392659	52392755
ENSG0000023949	0.193413103	0.699	0.81	LY6G5B	protein_coding	lymphocyte	CHR_HSCHR	31659867	31664295
ENSG0000024338	-0.675698006	0.7	0.81	AC012442.2	lncRNA	novel transc	2	112589040	112614431
ENSG0000021370	-0.598925304	0.7	0.81	RPL17P50	processed_pse	ribosomal p	10	73005833	73006595
ENSG0000025993	0.452157903	0.7	0.81	AC009065.2	lncRNA	novel transc	16	2091436	2095433
ENSG0000027185	-0.308676394	0.7	0.81	AL162258.1	lncRNA	novel transc	1	153626332	153634340
ENSG0000022572	0.379595126	0.7	0.81	AL592166.1	lncRNA	novel transc	1	44759037	44775810
ENSG0000010518	-0.041727255	0.7	0.81	PDCD5	protein_coding	programme	19	32581190	32587453
ENSG0000024042	-0.125931581	0.701	0.81	LRRFIP1P1	processed_pse	LRR binding	3	179259028	179261273
ENSG0000023569	-0.087232116	0.701	0.81	PFDN6	protein_coding	prefoldin su	CHR_HSCHR	33459645	33468744
ENSG0000026109	-0.246047433	0.701	0.81	AC007066.2	lncRNA	novel transc	9	122937623	122940333
ENSG0000016462	-0.151906208	0.701	0.81	KCNK5	protein_coding	potassium t	6	39188971	39229475
ENSG0000023949	-0.450304667	0.701	0.81	RN7SL333P	misc_RNA	RNA, 7SL, cy	1	169859756	169860052
ENSG0000016394	-0.03165092	0.701	0.81	TASOR	protein_coding	transcription	3	56620133	56683237
ENSG0000024903	0.51652613	0.701	0.81	SUMO2P6	processed_pse	SUMO2 pse	5	174561776	174562063
ENSG0000027820	0.229189435	0.702	0.81	AL031320.2	lncRNA	novel transc	6	143484979	143507327
ENSG0000027002	0.53691972	0.702	0.81	Z93241.1	lncRNA	RNA, U12 sr	22	42615244	42615907
ENSG0000010088	0.023765704	0.702	0.81	CHD8	protein_coding	chromodom	14	21385194	21456126
ENSG0000011852	0.194958721	0.702	0.81	CCN2	protein_coding	cellular com	6	131948176	131951372
ENSG0000027094	-0.531948556	0.702	0.81	MTDHP1	processed_pse	metadherin	7	64942143	64942926
ENSG0000012189	-0.057841501	0.702	0.81	LIAS	protein_coding	lipoic acid s	4	39459004	39485109
ENSG0000022797	0.394159414	0.703	0.82	PIN4P1	processed_pse	peptidylprol	15	43875849	43876244
ENSG0000024343	0.421707014	0.703	0.82	AC010973.1	lncRNA	novel transc	7	151028781	151029754
ENSG0000026120	0.409416924	0.703	0.82	AL031717.1	lncRNA	novel transc	16	1751559	1752262
ENSG0000022490	0.10532478	0.703	0.82	AP001347.1	lncRNA	novel transc	21	14027419	14144468
ENSG0000017991	0.086832581	0.703	0.82	NRXN1	protein_coding	neurexin 1 [	2	49918503	51225575
ENSG0000018474	-0.033445525	0.703	0.82	ATL3	protein_coding	atlastin GTP	11	63624087	63671921
ENSG0000025281	0.629790397	0.704	0.82	NA	NA	NA	NA	NA	NA
ENSG0000013212	-0.097816367	0.704	0.82	SPATA6	protein_coding	spermatoge	1	48295373	48472208
ENSG0000012839	0.1840939	0.704	0.82	APOBEC3F	protein_coding	apolipoprot	22	39040604	39055972
ENSG0000013210	0.149301087	0.704	0.82	TRIM21	protein_coding	tripartite mo	11	4384897	4393702
ENSG0000017715	-0.071014846	0.704	0.82	FAM210A	protein_coding	family with s	18	13663347	13726663
ENSG0000027611	0.213908352	0.704	0.82	AC026356.2	lncRNA	novel transc	12	32352349	32354144
ENSG0000011523	0.022979673	0.704	0.82	SNX17	protein_coding	sorting nexi	2	27370496	27377535
ENSG0000025881	0.426317042	0.704	0.82	AL442663.3	lncRNA	novel transc	14	73057925	73059415
ENSG0000015177	0.190973905	0.704	0.82	CCDC122	protein_coding	coiled-coil c	13	43823909	43879740
ENSG0000024100	-0.316923764	0.705	0.82	SEPTIN7P6	processed_pse	septin 7 pse	7	152367519	152368661
ENSG0000020706	0.673479672	0.705	0.82	SNORA15B	snoRNA	small nucleo	7	65070538	65070672
ENSG0000020649	0.282131263	0.705	0.82	ABCF1	protein_coding	ATP binding	CHR_HSCHR	30560870	30586674
ENSG0000027616	-0.54880576	0.705	0.82	AC092118.2	lncRNA	novel transc	16	57759358	57760024
ENSG0000027613	0.356399708	0.705	0.82	SLC22A18	protein_coding	solute carrie	CHR_HSCHR	2899745	2925175
ENSG0000019884	0.029316689	0.706	0.82	SELENOT	protein_coding	selenoprote	3	150602875	150630445

ENSG0000021424	-0.499659186	0.706	0.82	AC004980.2	processed_pse	pseudogene		7	76650401	76650897
ENSG0000025845	0.495058451	0.706	0.82	AL160314.2	lncRNA	novel transc		14	22701476	22766562
ENSG0000026042	-0.559787803	0.706	0.82	AL031709.1	lncRNA	novel transc		16	1358900	1361405
ENSG0000027986	-0.237092564	0.706	0.82	AC073548.1	TEC	TEC		19	47487638	47489519
ENSG0000022366	0.203513876	0.706	0.82	FKBPL	protein_coding	FKBP prolyl	CHR_HSCHR		32205134	32206718
ENSG0000022550	-0.177216074	0.706	0.82	AC069282.1	processed_pse	pseudogene		7	47956793	47957318
ENSG0000018534	0.105432473	0.707	0.82	PRKN	protein_coding	parkin RBR l		6	161347417	162727775
ENSG0000027138	0.230734238	0.707	0.82	AL451085.2	lncRNA	uncharacter		1	154961825	154962623
ENSG0000022920	0.249370742	0.707	0.82	PTGES3P3	processed_pse	prostagland		4	169791221	169791702
ENSG0000027175	-0.280242494	0.707	0.82	AL355802.3	lncRNA	novel transc		6	43519180	43519724
ENSG0000020661	0.477743572	0.707	0.82	SNORA2A	snoRNA	small nucleo		12	48656648	48656782
ENSG0000022347	-0.804348316	0.707	0.82	VN1R42P	processed_pse	vomeronasa		7	64933273	64933616
ENSG0000018781	-0.099573777	0.708	0.82	ZFP69	protein_coding	ZFP69 zinc f		1	40477290	40496343
ENSG0000013259	-0.043091736	0.708	0.82	ERAL1	protein_coding	Era like 12S		17	28855010	28861061
ENSG0000026199	0.182385383	0.708	0.82	WEE2-AS1	lncRNA	WEE2 antise	CHR_HSCHR		141710346	141744354
ENSG0000027610	0.781635243	0.709	0.82	NA	NA	NA	NA	NA		NA
ENSG0000027421	-0.586269102	0.709	0.82	AC015912.3	lncRNA	novel transc		17	56914186	56914533
ENSG0000017504	0.078476182	0.71	0.82	ZDHHC14	protein_coding	zinc finger D		6	157381133	157678157
ENSG0000027403	0.586879455	0.71	0.82	NA	NA	NA	NA	NA		NA
ENSG0000016237	-0.102465833	0.71	0.82	BEND5	protein_coding	BEN domain		1	48727519	48776969
ENSG0000027843	0.333408612	0.71	0.82	NA	NA	NA	NA	NA		NA
ENSG0000022980	-0.056771739	0.71	0.82	SLC39A7	protein_coding	solute carrie	CHR_HSCHR		33178351	33182345
ENSG0000024197	-0.674361632	0.71	0.82	NA	NA	NA	NA	NA		NA
ENSG0000025182	0.621664246	0.71	0.82	RNU6-583P	snRNA	RNA, U6 sm		4	127891991	127892082
ENSG0000027016	-0.437042324	0.71	0.82	AC010530.1	lncRNA	novel transc		16	67738588	67739922
ENSG0000014624	0.060519925	0.711	0.82	IRAK1BP1	protein_coding	interleukin 1		6	78867551	78946440
ENSG0000027623	-0.718747689	0.711	0.82	NA	NA	NA	NA	NA		NA
ENSG0000016075	-0.046530738	0.711	0.82	RUSC1	protein_coding	RUN and SH		1	155320894	155331114
ENSG0000025752	0.347066162	0.711	0.82	AL157935.2	protein_coding	novel protei		9	127867850	127906022
ENSG0000017246	-0.039986289	0.711	0.82	MANEA	protein_coding	mannosidas		6	95577535	95609452
ENSG0000022471	-0.214207007	0.711	0.82	AC025165.1	lncRNA	novel transc		12	57612118	57619638
ENSG0000023118	0.25983191	0.712	0.82	SPRY4-AS1	lncRNA	SPRY4 antis		5	142325293	142672001
ENSG0000027608	-0.130636836	0.712	0.82	CNOT3	protein_coding	CCR4-NOT	CHR_HSCHR		54138182	54156191
ENSG0000022074	-0.386908399	0.712	0.82	RPL5P18	processed_pse	ribosomal p		6	127362513	127363403
ENSG0000027295	-0.38200845	0.713	0.82	AC092171.4	lncRNA	novel transc		7	5425770	5426401
ENSG0000017387	-0.032249797	0.713	0.82	ZNF791	protein_coding	zinc finger p		19	12610918	12633840
ENSG0000010933	-0.01499539	0.713	0.82	UBE2D3	protein_coding	ubiquitin co		4	102794383	102868896
ENSG0000024154	0.214073225	0.713	0.82	GUSBP2	transcribed_un	GUSB pseud		6	26871484	26956554
ENSG0000018648	-0.033758989	0.713	0.82	INSIG1	protein_coding	insulin induc		7	155297776	155310235
ENSG0000027407	0.113853954	0.714	0.82	CASTOR2	protein_coding	cytosolic arg		7	74964705	75031528
ENSG0000015455	0.063574786	0.714	0.82	PDLIM3	protein_coding	PDZ and LIM		4	185500660	185535507
ENSG0000028007	0.112978887	0.714	0.82	AL353763.2	TEC	TEC		9	41012231	41014133
ENSG0000017392	0.191702297	0.714	0.82	SWSAP1	protein_coding	SWIM-type		19	11374666	11376951
ENSG0000025002	0.372393967	0.714	0.82	ARPIN-AP3	protein_coding	ARPIN-AP3		15	89834308	89912882
ENSG0000026769	0.742653499	0.714	0.82	NA	NA	NA	NA	NA		NA
ENSG0000019623	-0.024963689	0.715	0.82	SUPT5H	protein_coding	SPT5 homol		19	39436156	39476670
ENSG0000027905	0.254617324	0.715	0.82	AGAP14P	unprocessed_p	ArfGAP with		10	46337224	46358714
ENSG0000021561	0.066110342	0.715	0.82	HMX1	protein_coding	H6 family ho		4	8846076	8871839
ENSG0000022446	-0.426169765	0.715	0.82	NA	NA	NA	NA	NA		NA
ENSG0000026608	-0.502626397	0.715	0.82	AC015813.2	protein_coding	novel transc		17	57989038	58007246
ENSG0000015155	0.032635271	0.715	0.82	FAM160B1	protein_coding	family with s		10	114821744	114899832
ENSG0000010859	0.049798141	0.716	0.83	AKAP10	protein_coding	A-kinase an		17	19904302	19978343
ENSG0000024115	0.280086197	0.716	0.83	ARHGAP31	lncRNA	ARHGAP31		3	119314293	119322760
ENSG0000025648	-0.691264532	0.716	0.83	AC023796.2	lncRNA	novel transc		12	24830421	24836558
ENSG0000022626	0.417216758	0.717	0.83	AC009961.1	lncRNA	novel transc		2	159670708	159712435
ENSG0000013077	0.041812924	0.717	0.83	ATP5IF1	protein_coding	ATP synthas		1	28236109	28246906
ENSG0000015270	-0.02946772	0.717	0.83	SAR1B	protein_coding	secretion as		5	134601149	134649271
ENSG0000024449	-0.21669847	0.717	0.83	RWDD4P1	processed_pse	RWD domai		7	105301522	105302086

ENSG0000026814	0.250712015	0.717	0.83	PCGF7P	processed_pse	polycomb g		19	22137931	22138981
ENSG0000020431	-0.165146767	0.717	0.83	FKBPL	protein_coding	FKBP prolyl		6	32128707	32130288
ENSG0000026701	-0.278162795	0.717	0.83	NA	NA	NA	NA	NA	NA	
ENSG0000027405	-0.703443265	0.717	0.83	AC005837.3	misc_RNA			17	76721216	76721396
ENSG0000016079	0.054737678	0.717	0.83	CCDC12	protein_coding	coiled-coil c		3	46921726	46982010
ENSG0000017773	-0.17550587	0.717	0.83	AC025171.1	lncRNA	novel transc		5	43013060	43067452
ENSG0000018087	-0.023584378	0.718	0.83	SSR4	protein_coding	signal seque	X		153793516	153798499
ENSG0000026049	0.446901694	0.717	0.83	AC002310.3	unprocessed_p	ribosomal p		16	30537202	30538231
ENSG0000012921	-0.098430963	0.718	0.83	PLD2	protein_coding	phospholipa		17	4807152	4823434
ENSG0000022554	-0.439294977	0.718	0.83	AC245452.1	processed_pse	pseudogene		22	21885282	21885673
ENSG0000010304	-0.244606467	0.718	0.83	HAS3	protein_coding	hyaluronan		16	69105653	69118719
ENSG0000018135	-0.191943215	0.718	0.83	LRRC75A	protein_coding	leucine rich		17	16441577	16492193
ENSG0000020746	0.551126867	0.719	0.83	SNORA19	snoRNA	small nucleo		10	119060011	119060138
ENSG0000023822	-0.044994182	0.718	0.83	TMEM250	protein_coding	transmembr		9	136114581	136118875
ENSG0000023990	-0.536476731	0.719	0.83	RN7SL75P	misc_RNA	RNA, 7SL, cy		9	100102559	100102839
ENSG0000019686	-0.518725766	0.719	0.83	TOMM20L	protein_coding	translocase		14	58395928	58408702
ENSG0000027710	0.249899538	0.719	0.83	NA	NA	NA	NA	NA	NA	
ENSG0000027799	0.539341595	0.719	0.83	AC107075.1	unprocessed_p	eukaryotic t		2	164573741	164574054
ENSG0000020344	0.379454013	0.719	0.83	LINC00449	lncRNA	long interge		13	99499727	99501052
ENSG0000026501	0.187103283	0.719	0.83	AGAP12P	transcribed_un	ArfGAP with		10	48009873	48031640
ENSG0000016616	0.035855943	0.72	0.83	BTRC	protein_coding	beta-transd		10	101354033	101557321
ENSG0000013616	-0.073101103	0.72	0.83	SETDB2	protein_coding	SET domain		13	49444374	49495003
ENSG0000014600	0.086792864	0.72	0.83	LRRTM2	protein_coding	leucine rich		5	138868921	138875368
ENSG0000022573	0.086331027	0.72	0.83	SKIV2L	protein_coding	Ski2 like RN	CHR_HSCHR		31946626	31957297
ENSG0000013403	-0.033403594	0.72	0.83	CTIF	protein_coding	cap binding		18	48539031	48863217
ENSG0000022457	-0.507375864	0.72	0.83	LINC01117	lncRNA	long interge		2	176495255	176819180
ENSG0000023173	0.047335193	0.72	0.83	PPP1R10	protein_coding	protein pho	CHR_HSCHR		30645036	30663248
ENSG0000021295	0.362834202	0.72	0.83	BX088651.1	processed_pse	novel pseuc		9	42568865	42569315
ENSG0000000254	0.031553976	0.72	0.83	LAP3	protein_coding	leucine amir		4	17577192	17607972
ENSG0000019789	-0.038081298	0.72	0.83	KIF13B	protein_coding	kinesin fami		8	29067278	29263124
ENSG0000025391	0.200855372	0.72	0.83	THAP12P7	processed_pse	THAP doma		8	78760142	78762431
ENSG0000027260	0.429971634	0.72	0.83	AC015982.2	lncRNA	novel transc		2	55617909	55618373
ENSG0000006879	0.025388274	0.721	0.83	KIF2A	protein_coding	kinesin fami		5	62306162	62391025
ENSG0000016007	0.025571189	0.721	0.83	SSU72	protein_coding	SSU72 hom		1	1541673	1574863
ENSG0000015451	-0.130000475	0.721	0.83	DIPK1A	protein_coding	divergent pr		1	92832737	92961522
ENSG0000011571	-0.327505757	0.721	0.83	PROC	protein_coding	protein C, ir		2	127418427	127429242
ENSG0000018174	0.120772959	0.721	0.83	DIPK2A	protein_coding	divergent pr		3	143971823	144048719
ENSG0000023102	-0.233858005	0.722	0.83	LINC00271	lncRNA	long interge		6	135497422	135813990
ENSG0000027284	-0.131720871	0.722	0.83	AC084018.1	lncRNA	novel transc		12	121797511	121801972
ENSG0000023403	-0.514370457	0.722	0.83	TXNP6	processed_pse	thioredoxin		4	76958860	76959169
ENSG0000011439	-0.118707287	0.722	0.83	CYB561D2	protein_coding	cytochrome		3	50350845	50358460
ENSG0000022485	0.532971804	0.722	0.83	RPL29P11	processed_pse	ribosomal p		3	37016523	37017014
ENSG0000023740	-0.089849195	0.722	0.83	PPP1R11	protein_coding	protein pho	CHR_HSCHR		30145206	30148830
ENSG0000012083	0.028908269	0.723	0.83	NFYB	protein_coding	nuclear tran		12	104117086	104138241
ENSG0000017992	-0.152163392	0.722	0.83	ZNF784	protein_coding	zinc finger p		19	55620741	55624566
ENSG0000023777	0.401306391	0.723	0.83	AC126615.1	processed_pse	ribosomal p		12	95125760	95126389
ENSG0000020332	-0.303610484	0.723	0.83	AC012358.1	lncRNA	novel transc		2	55214387	55216126
ENSG0000023057	-0.34883844	0.723	0.83	AC234781.5	transcribed_un	trimethyllysi	X		155348752	155349799
ENSG0000017019	-0.048737429	0.723	0.83	NANP	protein_coding	N-acetylneu		20	25612935	25624014
ENSG0000026752	-0.149084597	0.723	0.83	AC010733.1	lncRNA	novel transc		2	60925909	60931610
ENSG0000020711	-0.646405065	0.723	0.83	RNVU1-32	snRNA	RNA, varian		8	127999131	127999294
ENSG0000023387	0.450543749	0.723	0.83	DLG5-AS1	lncRNA	DLG5 antise		10	77927372	77929824
ENSG0000010116	-0.274738163	0.724	0.83	CTSZ	protein_coding	cathepsin Z		20	58995185	59007254
ENSG0000022833	-0.28206734	0.724	0.83	AC073063.1	processed_pse	cytokine inc		7	99442890	99443496
ENSG0000016328	0.033099148	0.724	0.83	GNPDA2	protein_coding	glucosamine		4	44682200	44726588
ENSG0000023928	-0.982319322	0.724	0.83	LY6G5B	protein_coding	lymphocyte	CHR_HSCHR		31659349	31663777
ENSG0000010473	-0.037102636	0.724	0.83	KLHDC4	protein_coding	kelch doma		16	87696485	87765992
ENSG0000023955	-0.302473111	0.724	0.83	RPL37P2	processed_pse	ribosomal p		11	67682772	67683058

ENSG0000028040	-0.166577554	0.724	0.83	AC132872.4	TEC	TEC		17	82249067	82251844
ENSG0000014833	-0.043996976	0.724	0.83	NTMT1	protein_coding	N-terminal		9	129608884	129636135
ENSG0000020663	-0.500736039	0.724	0.83	SNORA22	snoRNA	small nucle		7	65755526	65755659
ENSG0000022238	0.810696026	0.724	0.83	RNA5SP203	rRNA_pseudog	RNA, 5S ribo		6	10752791	10752926
ENSG0000026323	0.125049269	0.725	0.83	CTSO	protein_coding	cathepsin O	CHR_HSCHR		155924118	155953911
ENSG0000023134	-0.719108995	0.725	0.83	AL020997.1	processed_pse	novel pseuc		1	27739091	27739439
ENSG0000002603	0.148253388	0.725	0.83	RTEL1-TNF	protein_coding	RTEL1-TNFI		20	63659300	63698684
ENSG0000013006	0.067684647	0.725	0.83	SAT1	protein_coding	spermidine/	X		23783173	23786226
ENSG0000017704	-0.068337082	0.725	0.83	TMEM80	protein_coding	transmembr		11	695428	705028
ENSG0000019777	-0.0441202	0.726	0.83	ZNF81	protein_coding	zinc finger p	X		47836902	48002561
ENSG0000025362	-0.093078032	0.726	0.83	EIF5AL1	protein_coding	eukaryotic t		10	79512601	79516440
ENSG0000027002	0.253565212	0.726	0.83	NA	NA	NA	NA		NA	NA
ENSG0000014252	0.0562464	0.726	0.83	ZNF473	protein_coding	zinc finger p		19	50025714	50053414
ENSG0000023289	0.190554535	0.727	0.83	RGS5-AS1	lncRNA	RGS5 antise		1	163161675	163213023
ENSG0000026366	0.75357003	0.727	0.83	NA	NA	NA	NA		NA	NA
ENSG0000015509	0.061029484	0.727	0.83	PTPRN2	protein_coding	protein tyro		7	157539056	158587823
ENSG0000014683	0.024921199	0.727	0.83	GIGYF1	protein_coding	GRB10 inter		7	100679507	100694037
ENSG0000023928	0.264533997	0.727	0.83	CASTOR1	protein_coding	cytosolic arg		22	30285117	30289622
ENSG0000023383	-0.364791208	0.727	0.83	EIF3LP2	processed_pse	eukaryotic t		10	42566677	42567483
ENSG0000026732	0.309046652	0.727	0.83	SNHG22	lncRNA	small nucle		18	49814023	49851059
ENSG0000021374	-0.090908422	0.728	0.83	RPS29	protein_coding	ribosomal p		14	49570984	49599164
ENSG0000025467	-0.269448653	0.728	0.83	AP000873.4	lncRNA	novel transc		11	83180144	83184520
ENSG0000022835	0.626561162	0.728	0.83	LINC02585	lncRNA	long interge		3	48663776	48669174
ENSG0000018678	0.093014434	0.728	0.83	SPIN2B	protein_coding	spindlin fam	X		57118551	57121546
ENSG0000018101	0.12491938	0.728	0.83	LSMEM1	protein_coding	leucine rich		7	112480853	112491062
ENSG0000026222	-0.44158393	0.728	0.83	AC004771.2	lncRNA	novel transc		17	4987706	4988446
ENSG0000019761	0.083855473	0.729	0.83	ZNF615	protein_coding	zinc finger p		19	51991332	52008230
ENSG0000020207	-0.736351817	0.729	0.83	AC104335.1	misc_RNA			1	247294835	247294941
ENSG0000024134	-0.017826778	0.729	0.83	RPL36A	protein_coding	ribosomal p	X		101390824	101396155
ENSG0000018564	0.057369042	0.729	0.83	AC034236.1	processed_pse	ribosomal p		5	116051917	116052289
ENSG0000027212	-0.451331485	0.729	0.83	AP006545.2	lncRNA	novel transc		8	38099471	38099931
ENSG0000022879	0.46333042	0.729	0.83	FAM207BP	processed_pse	family with s		13	18697003	18697507
ENSG0000023324	0.145342891	0.729	0.83	AC002524.1	processed_pse	ubiquitin-cc	X		14244382	14244928
ENSG0000025214	0.667208538	0.729	0.83	RNA5SP315	rRNA_pseudog	RNA, 5S ribo		10	48040462	48040577
ENSG0000025287	0.667208538	0.729	0.83	RNA5SP312	rRNA_pseudog	RNA, 5S ribo		10	47532352	47532467
ENSG0000004198	-0.068191576	0.729	0.83	THAP3	protein_coding	THAP doma		1	6624866	6635586
ENSG0000019665	0.049089221	0.729	0.83	TTC30B	protein_coding	tetratricope		2	177548998	177552796
ENSG0000018451	-0.405622588	0.73	0.83	BEX5	protein_coding	brain expres	X		102153708	102155977
ENSG0000027570	0.161265324	0.73	0.83	NA	NA	NA	NA		NA	NA
ENSG0000027785	-0.673631805	0.73	0.83	AC233755.2	protein_coding		KI270726.1		26241	26534
ENSG0000023445	0.362226647	0.73	0.83	AC005534.1	processed_pse	ribosomal p		7	156633355	156633787
ENSG0000016759	-0.074474897	0.73	0.84	PROSER3	protein_coding	proline and		19	35758143	35771028
ENSG0000015668	-0.190607414	0.73	0.84	GLYATL2	protein_coding	glycine-N-a		11	58834065	58904215
ENSG0000012108	-0.496040168	0.73	0.84	NACA3P	processed_pse	NACA famil		4	164943290	164943937
ENSG0000027329	0.149665119	0.73	0.84	AC009275.1	lncRNA	novel transc		7	134368737	134432534
ENSG0000016361	-0.177457138	0.731	0.84	CCDC191	protein_coding	coiled-coil c		3	113964137	114056594
ENSG0000023985	0.039567508	0.731	0.84	GET4	protein_coding	guided entr		7	876554	896436
ENSG0000001111	0.030818115	0.731	0.84	BTBD7	protein_coding	BTB domain		14	93237550	93333092
ENSG0000024470	-0.268915655	0.731	0.84	AC004918.1	lncRNA	novel transc		7	141652381	141656810
ENSG0000025744	0.276446739	0.731	0.84	ZNF878	protein_coding	zinc finger p		19	12043805	12052961
ENSG0000026728	-0.178282498	0.731	0.84	AC068473.3	lncRNA	novel transc		18	79576460	79589010
ENSG0000017738	0.030440488	0.731	0.84	MAGEF1	protein_coding	MAGE famil		3	184710364	184712064
ENSG0000018151	-0.134144755	0.731	0.84	ACBD4	protein_coding	acyl-CoA bi		17	45132600	45144181
ENSG0000023956	0.206975621	0.732	0.84	KMT2E-AS1	lncRNA	KMT2E antis		7	105013425	105014321
ENSG0000028011	0.209499162	0.732	0.84	NA	NA	NA	NA		NA	NA
ENSG0000014747	0.037095071	0.732	0.84	PLPBP	protein_coding	pyridoxal ph		8	37762595	37779768
ENSG0000016230	0.086448717	0.732	0.84	RPS6KA4	protein_coding	ribosomal p		11	64359148	64372215
ENSG0000023686	0.359311723	0.732	0.84	Z98885.1	processed_pse	suppressor		22	49817248	49817712

ENSG0000023508	0.430409532	0.733	0.84	CHCHD2P6	processed_pse	coiled-coil-		1	15604597	15605043
ENSG0000010636	-0.079500151	0.733	0.84	SERPINE1	protein_coding	serpin family		7	101127104	101139247
ENSG0000017766	-0.052016988	0.733	0.84	IL17RA	protein_coding	interleukin 1		22	17084954	17115694
ENSG0000019854	0.059423126	0.733	0.84	ZNF511	protein_coding	zinc finger p		10	133308914	133313162
ENSG0000027372	-0.165925091	0.734	0.84	SUGT1-DT	lncRNA	SUGT1 dive		13	52651305	52652279
ENSG0000023808	0.109941358	0.734	0.84	LRRC37A2	protein_coding	leucine rich		17	46511511	46555650
ENSG0000026902	0.220671438	0.735	0.84	MTRNR2L1	protein_coding	MT-RNR2 li		3	96617188	96618236
ENSG0000010404	-0.041190452	0.735	0.84	DTWD1	protein_coding	DTW domai		15	49621037	49656232
ENSG0000014772	-0.14291456	0.735	0.84	FAM135B	protein_coding	family with s		8	138130023	138497261
ENSG0000020422	-0.07730095	0.735	0.84	RING1	protein_coding	ring finger p		6	33208500	33212722
ENSG0000022347	-0.16230647	0.735	0.84	NA	NA	NA	NA		NA	NA
ENSG0000022506	-0.591860267	0.735	0.84	RPL23AP2	processed_pse	ribosomal p		19	15611657	15612122
ENSG0000012919	0.030737919	0.736	0.84	RPAIN	protein_coding	RPA interact		17	5419641	5432876
ENSG0000022858	-0.074272474	0.736	0.84	SPCS2P4	processed_pse	signal pepti		1	28095742	28096422
ENSG0000023626	-0.703755739	0.736	0.84	SHANK2-A	lncRNA	SHANK2 an		11	70646165	70647562
ENSG0000025275	0.72548529	0.736	0.84	Y_RNA	misc_RNA	Y RNA [Sou		2	200863152	200863247
ENSG0000027234	-0.15480033	0.736	0.84	AL137003.1	lncRNA	novel transc		6	16764346	16766883
ENSG0000019637	0.083824394	0.736	0.84	ZNF34	protein_coding	zinc finger p		8	144772224	144787345
ENSG0000021990	0.538628421	0.736	0.84	RPL35P3	processed_pse	ribosomal p		6	105302453	105302823
ENSG0000027272	-0.265012025	0.736	0.84	AC009336.1	lncRNA	novel transc		2	176164164	176165716
ENSG0000010703	-0.030118692	0.736	0.84	RIC1	protein_coding	RIC1 homol		9	5629025	5776557
ENSG0000011829	-0.224796218	0.736	0.84	CA14	protein_coding	carbonic an		1	150257251	150265078
ENSG0000014711	-0.050739942	0.736	0.84	ZNF182	protein_coding	zinc finger p	X		47974851	48003989
ENSG0000016728	0.044936981	0.736	0.84	ENGASE	protein_coding	endo-beta-		17	79074824	79088599
ENSG0000026153	-0.092586992	0.736	0.84	AL596244.1	lncRNA	novel transc		9	121815674	121819452
ENSG0000014597	-0.359098342	0.736	0.84	FAM217A	protein_coding	family with s		6	4049434	4087344
ENSG0000020084	-0.650864814	0.736	0.84	Y_RNA	misc_RNA	Y RNA [Sou		7	23491792	23491893
ENSG0000022349	-0.090552745	0.736	0.84	VARS2	protein_coding	valyl-tRNA s	CHR_HSCHR		30958848	30971104
ENSG0000025453	0.352833076	0.736	0.84	AC239804.1	lncRNA	novel transc		1	149048576	149051273
ENSG0000010078	0.046297528	0.737	0.84	RPS6KA5	protein_coding	ribosomal p		14	90847861	91060641
ENSG0000023463	0.161975245	0.737	0.84	MED14OS	lncRNA	MED14 opp	X		40735400	40738701
ENSG0000027474	0.493092268	0.738	0.84	AC138827.1	unprocessed_p	occludin (O	CHR_HSCHR		70221290	70240276
ENSG0000012315	-0.034378969	0.738	0.84	GIPC1	protein_coding	GIPC PDZ d		19	14477760	14496149
ENSG0000014125	-0.041853129	0.738	0.84	SGSM2	protein_coding	small G prot		17	2337498	2381058
ENSG0000026102	0.480115931	0.738	0.84	GS1-279B7	transcribed_pr	microtubule		1	185321157	185335039
ENSG0000019644	0.02985281	0.738	0.84	ARMCX4	protein_coding	armadillo re	X		101418287	101533459
ENSG0000025697	-0.529147443	0.739	0.84	LIMS3	protein_coding	LIM zinc fing		2	109898428	109924868
ENSG0000025196	0.504691321	0.739	0.84	NA	NA	NA	NA		NA	NA
ENSG0000015246	-0.037781644	0.739	0.84	NMT2	protein_coding	N-myristoyl		10	15102584	15168693
ENSG0000019856	0.105354879	0.739	0.84	DDX39B	protein_coding	DExD-box h		6	31530219	31542448
ENSG0000012552	-0.07209899	0.739	0.84	SLC2A4RG	protein_coding	SLC2A4 reg		20	63739776	63744050
ENSG0000013219	-0.059318845	0.739	0.84	HSD17B7	protein_coding	hydroxyster		1	162790702	162812817
ENSG0000016127	-0.117543304	0.739	0.84	THAP8	protein_coding	THAP doma		19	36034984	36054739
ENSG0000016312	0.192402424	0.739	0.84	ANKRD23	protein_coding	ankyrin repe		2	96824526	96857934
ENSG0000023543	0.041685747	0.739	0.84	DDX39B	protein_coding	DExD-box h	CHR_HSCHR		31520968	31533197
ENSG0000026012	-0.382882753	0.739	0.84	AC013565.1	lncRNA	novel transc		15	89041223	89082819
ENSG0000026789	0.300349844	0.739	0.84	NA	NA	NA	NA		NA	NA
ENSG0000010626	-0.062197206	0.74	0.84	SNX8	protein_coding	sorting nexi		7	2251770	2354318
ENSG0000024834	-1.527696433	0.74	0.84	NA	NA	NA	NA		NA	NA
ENSG0000020380	0.370014363	0.74	0.84	LIN28B-AS	lncRNA	LIN28B anti		6	104864464	104941447
ENSG0000018942	-0.443851092	0.74	0.84	USP32P3	transcribed_un	ubiquitin sp		17	20408311	20434286
ENSG0000026727	-0.428426199	0.74	0.84	AC008770.1	lncRNA	novel transc		19	11939959	11953381
ENSG0000016422	0.035929676	0.74	0.84	CCDC112	protein_coding	coiled-coil c		5	115267190	115296693
ENSG0000023091	0.426265261	0.74	0.84	AL021707.4	lncRNA	novel transc		22	38666508	38668750
ENSG0000025920	-0.125923304	0.74	0.84	ITGB3	protein_coding	integrin sub		17	47253827	47313743
ENSG0000027186	0.322320643	0.74	0.84	AC026979.1	lncRNA	novel transc		8	30155830	30156232
ENSG0000027209	0.078163664	0.74	0.84	NA	NA	NA	NA		NA	NA
ENSG0000010906	0.174869341	0.74	0.84	MYH3	protein_coding	myosin heav		17	10628526	10657309

ENSG0000016379	0.223544882	0.741	0.84	UCN	protein_coding	urocortin [S	2	27307400	27308445
ENSG0000027860	0.64246751	0.741	0.84	AC245033.3	unprocessed_p	dynamin 1 (	15	82472203	82472426
ENSG0000017311	0.022712516	0.741	0.84	TRMT112	protein_coding	tRNA methy	11	64316460	64317559
ENSG0000026704	-0.359957314	0.742	0.84	AC002398.1	lncRNA	novel transc	19	35769144	35771028
ENSG0000017112	-0.111939022	0.742	0.84	KCNMB3	protein_coding	potassium c	3	179236691	179267002
ENSG0000010146	0.108325562	0.742	0.84	SYNDIG1	protein_coding	synapse diff	20	24469629	24666616
ENSG0000016845	-0.102019716	0.742	0.84	PPT2	protein_coding	palmitoyl-p	CHR_HSCHR	32192849	32211366
ENSG0000020091	0.752685458	0.742	0.84	RNU6-553P	snRNA	RNA, U6 sm	4	105406997	105407092
ENSG0000020656	-0.061263677	0.742	0.84	METTL6	protein_coding	methyltrans	3	15381275	15440566
ENSG0000020745	0.653839561	0.742	0.84	RNU6-476P	snRNA	RNA, U6 sm	22	41679711	41679817
ENSG0000022442	0.280093192	0.742	0.84	ADM5	protein_coding	adrenomed	19	49688664	49690575
ENSG0000023680	-0.174483352	0.742	0.84	ZNRD1	protein_coding	zinc ribbon	CHR_HSCHR	30137399	30143406
ENSG0000026267	-0.245306876	0.742	0.84	AC004918.2	lncRNA	novel transc	CHR_HSCHR	141652381	141656810
ENSG0000022231	0.454209712	0.742	0.84	RNA5SP118	rRNA_pseudog	RNA, 5S ribo	2	209721913	209722021
ENSG0000016561	-0.209815072	0.742	0.84	DACT1	protein_coding	dishevelled	14	58633967	58648321
ENSG0000026205	-0.366250888	0.743	0.84	AC005696.1	lncRNA	novel transc	17	2712309	2712833
ENSG0000012794	-0.022207968	0.743	0.84	PTPN12	protein_coding	protein tyro	7	77537295	77640069
ENSG0000019774	-0.123769839	0.744	0.85	PTMAP2	processed_pse	prothymosi	5	118973796	118974122
ENSG0000023785	-0.110608829	0.744	0.85	LINC00674	transcribed_un	long interge	17	68101908	68115518
ENSG0000015906	-0.032312764	0.744	0.85	ALG8	protein_coding	ALG8 alpha	11	78100936	78139653
ENSG0000018345	-0.039786901	0.744	0.85	AC138932.1	transcribed_un	polycystic ki	16	14911551	14935708
ENSG0000022980	-0.324293986	0.744	0.85	AL391825.1	transcribed_pr	proteasome	1	161890833	161892196
ENSG0000026156	-0.814931434	0.744	0.85	NA	NA	NA	NA	NA	NA
ENSG0000023121	0.470762237	0.744	0.85	AL590399.3	lncRNA	novel transc	9	62349941	62351536
ENSG0000023510	0.333928666	0.744	0.85	AL356968.2	processed_pse	amyotrophi	1	48435967	48437223
ENSG0000013474	-0.021696936	0.745	0.85	TUT4	protein_coding	terminal uri	1	52408282	52553487
ENSG0000027481	-0.420662732	0.745	0.85	AC004825.2	lncRNA	novel transc	14	70906657	70907111
ENSG0000007591	-0.052702844	0.745	0.85	EXOSC7	protein_coding	exosome co	3	44975241	45036066
ENSG0000012157	-0.016597208	0.745	0.85	NAA50	protein_coding	N-alpha-ac	3	113716458	113746300
ENSG0000014059	0.032549103	0.745	0.85	EFL1	protein_coding	elongation f	15	82130233	82262734
ENSG0000023914	0.631182051	0.745	0.85	SNORA59A	snoRNA	small nucleo	1	12507246	12507397
ENSG0000027382	-0.085930232	0.745	0.85	USP27X	protein_coding	ubiquitin sp	X	49879484	49882558
ENSG0000022646	-0.669417035	0.746	0.85	AL390198.1	lncRNA	novel transc	20	25697003	25752776
ENSG0000023681	0.105580101	0.746	0.85	AC046176.1	processed_pse	proteasome	8	56050038	56051207
ENSG0000019640	0.405771128	0.746	0.85	THEM5	protein_coding	thioesterase	1	151847101	151853712
ENSG0000016648	-0.046118918	0.746	0.85	MAPK7	protein_coding	mitogen-ac	17	19377721	19383544
ENSG0000023686	-0.403274488	0.747	0.85	ZKSCAN7-A	lncRNA	ZKSCAN7 Z	3	44557357	44685653
ENSG0000000885	0.027935526	0.747	0.85	RHOBTB2	protein_coding	Rho related	8	22987417	23020199
ENSG0000024472	-0.367262125	0.747	0.85	ASLP1	unprocessed_p	argininosuc	22	23649213	23653334
ENSG0000026117	0.442390203	0.747	0.85	AC018845.3	lncRNA	novel transc	16	46973989	46978983
ENSG0000024104	0.151795188	0.747	0.85	NA	NA	NA	NA	NA	NA
ENSG0000010830	-0.03080104	0.747	0.85	FBXL20	protein_coding	F-box and l	17	39252663	39402523
ENSG0000022361	-0.251485927	0.747	0.85	AC241585.1	processed_pse	EGFR-coam	1	145233001	145233519
ENSG0000023667	0.33214462	0.747	0.85	KRT18P5	processed_pse	keratin 18 p	22	20482815	20484105
ENSG0000024923	-0.346923532	0.747	0.85	CDH12P2	unprocessed_p	cadherin 12	5	69985204	69985501
ENSG0000023538	0.084322473	0.748	0.85	AL596202.1	lncRNA	novel transc	6	155253139	155256724
ENSG0000019871	-0.156426936	0.748	0.85	SSBP3-AS1	lncRNA	SSBP3 antis	1	54236440	54239063
ENSG0000024945	-0.132341854	0.748	0.85	AL731577.2	lncRNA	novel transc	10	124917143	124942881
ENSG0000020406	0.104506793	0.748	0.85	TCEAL5	protein_coding	transcriptior	X	103273691	103276750
ENSG0000018443	0.048562815	0.748	0.85	THAP7	protein_coding	THAP doma	22	20999104	21002196
ENSG0000023487	-0.253131558	0.749	0.85	NOTCH4	protein_coding	notch recep	CHR_HSCHR	32202513	32231740
ENSG0000024370	0.256691352	0.748	0.85	LEFTY1	protein_coding	left-right de	1	225886282	225911382
ENSG0000026337	0.549998486	0.748	0.85	NA	NA	NA	NA	NA	NA
ENSG0000017258	-0.043498549	0.749	0.85	CHCHD1	protein_coding	coiled-coil-	10	73782047	73783652
ENSG0000020747	0.709898347	0.749	0.85	SNORA80E	snoRNA	small nucleo	1	155919909	155920045
ENSG0000017410	-0.027985528	0.749	0.85	LEMD3	protein_coding	LEM domair	12	65169583	65248355
ENSG0000014641	0.050570225	0.749	0.85	AIG1	protein_coding	androgen ir	6	143060496	143341058
ENSG0000011715	0.066172167	0.75	0.85	CTBS	protein_coding	chitobiase [S	1	84549611	84574480

ENSG0000016882	0.08206403	0.75	0.85	ZBTB49	protein_coding	zinc finger a	4	4290251	4321786
ENSG0000022641	0.094098848	0.75	0.85	TPI1P1	processed_pse	triosephosp	1	76699789	76700538
ENSG0000022704	0.329356036	0.75	0.85	AL589765.1	lncRNA	novel transc	1	151701026	151708386
ENSG0000026092	0.186699405	0.75	0.85	LINC01311	lncRNA	long interge	22	19171395	19172839
ENSG0000027203	0.309538307	0.75	0.85	SNORD14A	snoRNA	small nucle	11	17074654	17074744
ENSG0000012468	-0.041627216	0.75	0.85	MAD2L1BP	protein_coding	MAD2L1 bir	6	43629540	43640941
ENSG0000015731	-0.257441701	0.75	0.85	TMED6	protein_coding	transmembr	16	69343250	69351786
ENSG0000027679	0.162147709	0.75	0.85	AC108108.1	transcribed_un	glucuronida	CHR_HSCHR	70064043	70134190
ENSG0000025552	0.024396798	0.75	0.85	POLR2M	protein_coding	RNA polyme	15	57706695	57782762
ENSG0000001127	0.026673784	0.75	0.85	RNF216	protein_coding	ring finger p	7	5620047	5781696
ENSG0000015617	-0.085605728	0.75	0.85	C8orf37	protein_coding	chromosom	8	95244913	95269201
ENSG0000021920	-0.039137888	0.751	0.85	RNASEK	protein_coding	ribonuclease	17	7012417	7014532
ENSG0000008382	0.080930362	0.751	0.85	ZNF586	protein_coding	zinc finger p	19	57769655	57819939
ENSG0000024864	0.548590008	0.751	0.85	AC113410.1	processed_pse	NADH dehy	5	133003119	133003365
ENSG0000014779	-0.055163961	0.751	0.85	ARHGAP39	protein_coding	Rho GTPase	8	144529179	144605816
ENSG0000025599	-0.415741019	0.751	0.85	AC131009.1	lncRNA	novel transc	12	131924736	131929351
ENSG0000027511	-0.068013947	0.751	0.85	ZNF2	protein_coding	zinc finger p	2	95165432	95184317
ENSG0000010786	0.01868598	0.751	0.85	ARHGAP21	protein_coding	Rho GTPase	10	24583609	24723887
ENSG0000023271	0.393597003	0.751	0.85	RPS12P3	processed_pse	ribosomal p	2	60938204	60938604
ENSG0000023432	0.20440399	0.751	0.85	AC012146.1	lncRNA	novel transc	17	5111468	5115004
ENSG0000025323	-0.106041885	0.751	0.85	MIR124-1H	lncRNA	MIR124-1 h	8	9899871	9906678
ENSG0000011134	0.045319666	0.752	0.85	MGP	protein_coding	matrix Gla p	12	14880864	14885857
ENSG0000027593	-0.33612673	0.752	0.85	LENG1	protein_coding	leukocyte re	CHR_HSCHR	54156127	54160192
ENSG0000017940	-0.204574638	0.752	0.85	VWA1	protein_coding	von Willebr	1	1434861	1442882
ENSG0000022412	-0.208855084	0.752	0.85	C6orf136	protein_coding	chromosom	CHR_HSCHR	30725496	30731566
ENSG0000010009	0.173283644	0.752	0.85	SH3BP1	protein_coding	SH3 domain	22	37634654	37656117
ENSG0000010289	0.044730011	0.752	0.85	LYRM1	protein_coding	LYR motif co	16	20899868	20925006
ENSG0000016928	-0.033815785	0.752	0.85	MRPL1	protein_coding	mitochondr	4	77862830	77952785
ENSG0000026028	0.113990678	0.752	0.85	AL133367.1	lncRNA	novel transc	14	103525010	103529072
ENSG0000023090	-0.143244052	0.753	0.85	FKBPL	protein_coding	FKBP prolyl	CHR_HSCHR	32077157	32078741
ENSG0000022706	-0.224879352	0.753	0.85	LINC00629	lncRNA	long interge	X	134549770	134560399
ENSG0000027279	-0.134923573	0.753	0.85	NA	NA	NA	NA	NA	NA
ENSG0000013016	0.033063482	0.753	0.85	ELOF1	protein_coding	elongation f	19	11551147	11559236
ENSG0000027197	0.099099084	0.753	0.85	AC012467.2	lncRNA	novel transc	3	53858994	53861576
ENSG0000027847	-0.48024252	0.753	0.85	AC009268.2	lncRNA	novel transc	15	33972059	33972515
ENSG0000026087	-0.222617712	0.754	0.85	NA	NA	NA	NA	NA	NA
ENSG0000022817	0.191511359	0.754	0.85	AL020996.1	lncRNA	novel transc	1	25816749	25820797
ENSG0000021835	-0.256819551	0.754	0.85	LYPLA1P3	processed_pse	LYPLA1 pse	6	71165076	71165770
ENSG0000023243	0.112484531	0.755	0.85	AJM1	protein_coding	apical juncti	9	136844415	136848801
ENSG0000023245	-0.574130212	0.755	0.85	AL138752.1	processed_pse	mitochondr	9	37836044	37836394
ENSG0000027650	-0.366729811	0.755	0.85	AP000892.3	lncRNA	novel transc	11	117297005	117297328
ENSG0000012622	-0.028381331	0.755	0.85	PCID2	protein_coding	PCI domain	13	113177536	113208715
ENSG0000025499	-0.143148885	0.755	0.85	STX16-NPE	protein_coding	STX16-NPE	20	58651434	58715410
ENSG0000009037	0.028083393	0.755	0.85	STRN4	protein_coding	striatin 4 [So	19	46719511	46746994
ENSG0000012821	-0.365965756	0.756	0.85	VPREB3	protein_coding	V-set pre-B	22	23752743	23754425
ENSG0000020017	-0.50231712	0.756	0.85	Y_RNA	misc_RNA	Y RNA [Sou	10	72220752	72220852
ENSG0000024709	-0.090755167	0.756	0.85	SNHG10	lncRNA	small nucle	14	95521943	95534889
ENSG0000011717	-0.030759475	0.757	0.85	ZNHIT6	protein_coding	zinc finger H	1	85649417	85708433
ENSG0000008572	0.032094615	0.757	0.85	RRN3	protein_coding	RRN3 homo	16	15060033	15094311
ENSG0000016440	0.188807494	0.757	0.86	GDF9	protein_coding	growth diffe	5	132861181	132866884
ENSG0000022681	-0.361342473	0.757	0.86	MEIS1-AS3	lncRNA	MEIS1 antis	2	66426735	66433470
ENSG0000024762	-0.041222872	0.758	0.86	MARS2	protein_coding	methionyl-t	2	197705369	197708395
ENSG0000005428	0.047693088	0.758	0.86	SDCCAG8	protein_coding	SHH signali	1	243256034	243500091
ENSG0000000519	0.022908955	0.758	0.86	CIAPIN1	protein_coding	cytokine inc	16	57428187	57447420
ENSG0000022641	-0.081367774	0.758	0.86	SLC16A1-A	lncRNA	SLC16A1 an	1	112956415	113047055
ENSG0000022944	0.41497047	0.758	0.86	AC114495.2	processed_pse	general tran	1	31263245	31263681
ENSG0000016650	-0.01526284	0.758	0.86	MCM7	protein_coding	minichromo	7	100092728	100101940
ENSG0000006996	0.056599461	0.758	0.86	GNB5	protein_coding	G protein su	15	52115105	52191369

ENSG0000008105	-0.075826016	0.758	0.86	TCF7	protein_coding	transcriptior	5	134114681	134151865
ENSG0000022700	-0.306269566	0.758	0.86	AL009174.1	processed_pse	ribosomal p	X	133670971	133671386
ENSG0000026477	0.086817353	0.758	0.86	AC016876.3	lncRNA	novel transc	17	7572826	7582024
ENSG0000013471	-0.205093617	0.759	0.86	CYP2J2	protein_coding	cytochrome	1	59893308	59926773
ENSG0000013694	0.031459631	0.759	0.86	PDCL	protein_coding	phosducin li	9	122798389	122828588
ENSG0000016270	0.023195406	0.759	0.86	ZNF281	protein_coding	zinc finger p	1	200404940	200410056
ENSG0000017297	0.03773371	0.759	0.86	VDAC2P5	processed_pse	VDAC2 pseu	2	65205108	65205988
ENSG0000024135	-0.367883242	0.759	0.86	AC007688.2	processed_pse	ribosomal p	12	12850982	12851740
ENSG0000026219	0.282094083	0.759	0.86	NA	NA	NA	NA	NA	NA
ENSG0000017866	-0.023110471	0.759	0.86	CSRNP3	protein_coding	cysteine anc	2	165469647	165689407
ENSG0000022501	0.396767644	0.76	0.86	AC092647.1	processed_pse	pseudogene	7	55876738	55877024
ENSG0000023387	0.709362973	0.76	0.86	AC007881.1	unprocessed_p	pseudogene	2	71178587	71178773
ENSG0000022610	0.616693427	0.76	0.86	AC004383.1	processed_pse	ribosomal p	X	134522702	134522990
ENSG0000025523	-0.360088764	0.76	0.86	AP002992.1	lncRNA	novel transc	11	68121624	68130518
ENSG0000007040	-0.09938127	0.76	0.86	FSTL3	protein_coding	follistatin lik	19	676392	683392
ENSG0000027027	0.511692125	0.76	0.86	BNIP3P10	processed_pse	BCL2 intera	19	19870723	19871299
ENSG0000013082	-0.02867234	0.76	0.86	SLC6A8	protein_coding	solute carrie	X	153687926	153696588
ENSG0000009962	-0.047869128	0.76	0.86	CBARP	protein_coding	CACN subu	19	1228287	1238027
ENSG0000016991	0.104735984	0.761	0.86	OTUD7A	protein_coding	OTU deubic	15	31475398	31870789
ENSG0000022394	0.371270811	0.761	0.86	AC244033.1	processed_pse	ribosomal p	1	150053864	150055034
ENSG0000022617	0.521675866	0.761	0.86	LINC00685	lncRNA	long interge	X	320990	321851
ENSG0000023531	-0.135773036	0.761	0.86	LINC00957	lncRNA	long interge	7	44039171	44042306
ENSG0000025722	0.381965989	0.761	0.86	AC079907.1	lncRNA	novel transc	12	102063355	102074820
ENSG0000026912	0.521675866	0.761	0.86	AL137002.1	lncRNA	novel transc	13	113165002	113165183
ENSG0000027080	0.521675866	0.761	0.86	AC005776.1	processed_pse	ribosomal p	19	15338948	15339237
ENSG0000016616	-0.048543739	0.761	0.86	POLL	protein_coding	DNA polym	10	101578882	101588270
ENSG0000018379	0.06255782	0.762	0.86	NPIPA5	protein_coding	nuclear pore	16	15363628	15381047
ENSG0000020053	0.298602812	0.762	0.86	SNORA33	snoRNA	small nucleo	6	132817219	132817348
ENSG0000011860	-0.041869415	0.762	0.86	RXYLT1	protein_coding	ribitol xylosy	12	63779842	63809562
ENSG0000022758	0.404875717	0.762	0.86	AL121877.1	processed_pse	staufen RNA	X	90039213	90039834
ENSG0000025548	0.439010597	0.762	0.86	AC087362.2	lncRNA	novel transc	8	38275888	38276680
ENSG0000016288	-0.033861332	0.762	0.86	B3GALNT2	protein_coding	beta-1,3-N	1	235447190	235504452
ENSG0000027324	0.300607998	0.762	0.86	Z82243.1	lncRNA	novel transc	22	45155539	45156011
ENSG0000019913	0.345796887	0.763	0.86	MIRLET7D	miRNA	microRNA le	9	94178834	94178920
ENSG0000025682	-0.466753738	0.763	0.86	AC068790.1	processed_pse	spindle and	12	124005871	124006225
ENSG0000017915	0.037163511	0.763	0.86	TCAIM	protein_coding	T cell activa	3	44338119	44409451
ENSG0000018595	0.023706162	0.763	0.86	IRS2	protein_coding	insulin recep	13	109752695	109786583
ENSG0000022414	-0.073090063	0.763	0.86	EHMT2	protein_coding	euchromatic	CHR_HSCHR	31872446	31882289
ENSG0000016471	0.024897854	0.763	0.86	LMTK2	protein_coding	lemur tyrosi	7	98106862	98209638
ENSG0000025143	-0.141679592	0.763	0.86	LINC02615	lncRNA	long interge	4	128292751	128519398
ENSG0000022129	-0.668944137	0.763	0.86	NA	NA	NA	NA	NA	NA
ENSG0000027233	-0.289800884	0.764	0.86	AC067838.1	lncRNA	novel transc	8	33360839	33361415
ENSG0000027208	0.21026454	0.764	0.86	AC080013.4	lncRNA	novel transc	3	158693120	158693768
ENSG0000027058	0.33562847	0.764	0.86	AL445435.1	processed_pse	sorting nexi	1	220207618	220208282
ENSG0000025955	0.342361241	0.764	0.86	AC090971.4	unprocessed_p	Leo1, Paf1/I	15	51919073	51930627
ENSG0000026741	-0.279923976	0.764	0.86	ZNF56	transcribed_un	zinc finger p	19	19776602	19836073
ENSG0000026768	-0.046267597	0.764	0.86	ZNF224	protein_coding	zinc finger p	19	44094339	44109886
ENSG0000027234	-0.208528279	0.764	0.86	AL031775.1	lncRNA	novel transc	6	24700907	24701793
ENSG0000011711	0.017665112	0.764	0.86	ADGRL2	protein_coding	adhesion G	1	81306147	81992436
ENSG0000023649	-0.093721414	0.764	0.86	TAPBP	protein_coding	TAP binding	CHR_HSCHR	33277619	33292309
ENSG0000023433	0.313337096	0.765	0.86	BCAS2P2	processed_pse	BCAS2 pseu	1	100393033	100394026
ENSG0000006465	0.073250922	0.765	0.86	SNX24	protein_coding	sorting nexi	5	122843439	123029354
ENSG0000012421	0.048047286	0.765	0.86	MOCS3	protein_coding	molybdenur	20	50958818	50963929
ENSG0000011188	-0.014999894	0.766	0.86	MAN1A1	protein_coding	mannosidas	6	119177205	119349761
ENSG0000027509	-0.377967327	0.766	0.86	AC024940.5	lncRNA	novel transc	12	31280422	31280895
ENSG0000027209	0.07633874	0.766	0.86	NA	NA	NA	NA	NA	NA
ENSG0000020904	-0.658668735	0.766	0.86	SNORD12C	snoRNA	small nucleo	20	49278940	49279028
ENSG0000023918	-0.722895192	0.766	0.86	SNORA84	snoRNA	small nucleo	9	92292461	92292593

ENSG0000026225	-0.216672571	0.766	0.86	NA	NA	NA	NA	NA	NA	
ENSG0000017997	-0.057644983	0.766	0.86	NA	NA	NA	NA	NA	NA	
ENSG0000016689	-0.221598598	0.766	0.86	ATP23	protein_coding	ATP23 meta	12	57906039	57959148	
ENSG0000026707	0.338924332	0.766	0.86	CCDC58P3	processed_pse	coiled-coil c	18	12211378	12211807	
ENSG0000024322	-0.38534773	0.767	0.86	AC006252.1	lncRNA	novel transc	3	52239258	52241097	
ENSG0000018891	-0.060049291	0.767	0.86	TRMT2B	protein_coding	tRNA methy	X	101009346	101052116	
ENSG0000010467	0.039471559	0.767	0.86	R3HCC1	protein_coding	R3H domain	8	23270120	23296279	
ENSG0000021326	0.360635996	0.767	0.86	EEF1B2P6	processed_pse	eukaryotic t	7	131661952	131662624	
ENSG0000012581	0.02819968	0.767	0.86	PSMF1	protein_coding	proteasome	20	1113240	1189415	
ENSG0000025704	-0.33192479	0.767	0.86	SRSF3P1	processed_pse	SRSF3 pseu	11	18665050	18665548	
ENSG0000010653	0.179843402	0.767	0.86	RARRES2	protein_coding	retinoic acic	7	150338317	150341662	
ENSG0000017882	0.352522096	0.767	0.86	TMEM52	protein_coding	transmembr	1	1917590	1919279	
ENSG0000025439	0.410875063	0.767	0.86	AC132192.1	lncRNA	novel transc	11	9430356	9433486	
ENSG0000027360	0.093841334	0.767	0.86	CHCHD10	protein_coding	coiled-coil-	CHR_HSCHR	23765834	23768443	
ENSG0000024930	-0.351144465	0.768	0.86	LINC01411	lncRNA	long interge	5	174336295	174532457	
ENSG0000007061	0.022636816	0.768	0.86	GBA2	protein_coding	glucosylcera	9	35736866	35749228	
ENSG0000023933	-0.243812892	0.768	0.86	LLPH-DT	lncRNA	LLPH diverg	12	66130751	66134449	
ENSG0000008389	-0.022660923	0.768	0.86	YTHDC1	protein_coding	YTH domain	4	68310387	68350090	
ENSG0000010397	0.02469043	0.768	0.86	TMEM87A	protein_coding	transmembr	15	42210447	42273584	
ENSG0000016298	0.025511626	0.768	0.86	ARL5A	protein_coding	ADP ribosyl	2	151788984	151828492	
ENSG0000023155	-0.315386821	0.768	0.86	AC245100.4	transcribed_un	phosphodie	1	148402516	148432545	
ENSG0000025373	0.127035792	0.768	0.86	PCDHGA6	protein_coding	protocadhe	5	141373891	141512975	
ENSG0000013710	0.03216078	0.768	0.86	GRHPR	protein_coding	glyoxylate a	9	37422666	37436990	
ENSG0000022682	-0.249220328	0.768	0.86	SUGT1P1	unprocessed_p	SUGT1 pseu	9	33503655	33510711	
ENSG0000018218	0.084060599	0.769	0.86	RAD51B	protein_coding	RAD51 para	14	67819779	68730218	
ENSG0000024969	-0.173050477	0.769	0.86	AC110813.1	lncRNA	novel transc	4	150579089	150581545	
ENSG0000022479	-0.352147041	0.769	0.86	AP000704.2	lncRNA	novel transc	21	36966461	36975164	
ENSG0000010839	0.022149335	0.769	0.86	TRIM37	protein_coding	tripartite mo	17	58982638	59106921	
ENSG0000011564	-0.035515709	0.77	0.86	CNPPD1	protein_coding	cyclin Pas1/	2	219171897	219178106	
ENSG0000013961	0.02874707	0.77	0.86	BRCA2	protein_coding	BRCA2 DNA	13	32315086	32400268	
ENSG0000023293	0.056125108	0.77	0.86	LINC00342	lncRNA	long interge	2	95807052	95835003	
ENSG0000026577	0.160806402	0.77	0.86	AC018413.1	lncRNA	novel transc	18	76491629	76493918	
ENSG0000025011	0.168784051	0.77	0.86	AC107982.1	unprocessed_p	coiled-coil c	17	18835707	18842364	
ENSG0000017206	0.048924145	0.77	0.86	SMN1	protein_coding	survival of n	5	70925030	70953942	
ENSG0000021237	-0.456679858	0.77	0.86	AF111168.1	snoRNA		14	77466284	77466436	
ENSG0000013614	-0.026620913	0.77	0.86	RCBTB1	protein_coding	RCC1 and B	13	49531946	49585558	
ENSG0000015799	-0.210730321	0.77	0.86	ANKRD61	protein_coding	ankyrin repe	7	6031376	6036552	
ENSG0000023666	-0.495791121	0.77	0.86	AL133215.1	lncRNA	novel transc	10	100980507	100985614	
ENSG0000025047	0.095335054	0.77	0.86	CHCHD10	protein_coding	coiled-coil-	22	23765834	23767972	
ENSG0000023403	0.062037287	0.771	0.86	VARS2	protein_coding	valyl-tRNA s	CHR_HSCHR	30992759	31005022	
ENSG0000024008	0.157701549	0.771	0.86	AC092969.1	lncRNA	novel transc	3	135138469	135439888	
ENSG0000026540	0.640738004	0.771	0.86	MIR4324	miRNA	microRNA 4	19	49308797	49308868	
ENSG0000020074	-0.648619534	0.771	0.86	Y_RNA	misc_RNA	Y RNA [Sou	14	55651534	55651634	
ENSG0000025011	-0.292739487	0.771	0.86	RHOQ-AS1	lncRNA	RHOQ antis	2	46568256	46580238	
ENSG0000025119	-0.397697902	0.771	0.86	AC106760.1	unprocessed_p	novel pseuc	5	10652211	10655925	
ENSG0000014085	-0.032081329	0.772	0.86	KATNB1	protein_coding	katanin regu	16	57735739	57757244	
ENSG0000015668	0.213093668	0.772	0.86	UNC5D	protein_coding	unc-5 netrir	8	35235475	35796550	
ENSG0000016090	-0.047388527	0.772	0.86	ZNF394	protein_coding	zinc finger p	7	99473877	99504857	
ENSG0000027936	-0.069052045	0.772	0.86	AC046185.3	TEC	TEC	17	63700847	63702670	
ENSG0000015617	-0.032338699	0.772	0.86	DRAM2	protein_coding	DNA damag	1	111117333	111140216	
ENSG0000023504	-0.341436254	0.772	0.86	TECRP1	processed_pse	trans-2,3-er	4	86949669	86950113	
ENSG0000024229	0.754673685	0.772	0.86	DEFB109A	unprocessed_p	defensin be	8	12393209	12400366	
ENSG0000014051	0.111555477	0.772	0.86	HAPLN3	protein_coding	hyaluronan	15	88877294	88895597	
ENSG0000027330	-0.244709276	0.772	0.86	AC016747.3	lncRNA	novel transc	2	61199979	61200769	
ENSG0000008909	-0.084468291	0.773	0.86	DZANK1	protein_coding	double zinc	20	18383367	18467281	
ENSG0000000108	0.021096726	0.773	0.86	GCLC	protein_coding	glutamate-c	6	53497341	53616970	
ENSG0000023883	0.626219299	0.773	0.86	SCARNA18	snoRNA	small Cajal b	5	83064204	83064337	
ENSG0000026220	-0.176153295	0.773	0.87	PCDHGB3	protein_coding	protocadhe	5	141370242	141512975	

ENSG0000027242	-0.44132985	0.774	0.87	AL450270.1	lncRNA	novel transc	6	133540784	133541174
ENSG0000015243	0.11268669	0.774	0.87	ZNF547	protein_coding	zinc finger p	19	57363477	57379565
ENSG0000014541	0.036788796	0.774	0.87	NAF1	protein_coding	nuclear asse	4	163110073	163166890
ENSG0000015975	0.053526083	0.774	0.87	CARMIL2	protein_coding	capping pro	16	67644988	67657569
ENSG0000023491	0.305095651	0.774	0.87	AC098484.2	lncRNA	novel transc	1	42678735	42681659
ENSG0000027369	-0.310606841	0.774	0.87	C2orf27AP1	processed_pse	chromosom	Y	11085476	11086061
ENSG0000018010	0.031827103	0.774	0.87	EXOC3	protein_coding	exocyst com	5	443175	471937
ENSG0000015734	0.11113538	0.775	0.87	ARMC12	protein_coding	armadillo re	6	35737032	35749079
ENSG0000012534	-0.114606461	0.775	0.87	IRF1	protein_coding	interferon re	5	132481609	132490777
ENSG0000027266	-0.512214805	0.775	0.87	AC093635.1	lncRNA	novel transc	2	48440043	48440597
ENSG0000025983	0.133473385	0.776	0.87	ELOCP2	unprocessed_p	elongin C ps	15	41557027	41557435
ENSG0000027531	0.225983577	0.776	0.87	AC139494.3	lncRNA	novel transc	CHR_HSCHR	69758658	69759641
ENSG0000002769	0.026705044	0.777	0.87	IFNGR1	protein_coding	interferon g	6	137197485	137219449
ENSG0000013231	-0.026343188	0.777	0.87	MRPL35	protein_coding	mitochondr	2	86199355	86213790
ENSG0000017995	-0.026785332	0.777	0.87	PUF60	protein_coding	poly(U) binc	8	143816344	143829352
ENSG0000026693	-0.229668873	0.777	0.87	NA	NA	NA	NA	NA	NA
ENSG0000018181	-0.272065289	0.777	0.87	KCTD9P2	processed_pse	potassium c	X	105405647	105407039
ENSG0000021371	0.049700587	0.777	0.87	PIGCP1	processed_pse	phosphatidy	11	33075566	33076460
ENSG0000025777	-0.295368952	0.778	0.87	ST13P3	processed_pse	ST13, Hsp70	12	106015897	106017006
ENSG0000016460	-0.02211653	0.778	0.87	SLU7	protein_coding	SLU7 homo	5	160401641	160421711
ENSG0000007806	-0.034836919	0.778	0.87	ARAF	protein_coding	A-Raf protc	X	47561100	47571920
ENSG0000025206	-0.578266989	0.778	0.87	RNU6-415P	snRNA	RNA, U6 sm	15	78898840	78898936
ENSG0000022839	-0.21964804	0.778	0.87	AL356481.1	lncRNA	novel transc	9	128528657	128552410
ENSG0000010884	0.092881601	0.778	0.87	ABCC3	protein_coding	ATP binding	17	50634777	50692253
ENSG0000013511	-0.05071414	0.779	0.87	HRK	protein_coding	harakiri, BCl	12	116856144	116881441
ENSG0000016359	0.097723773	0.779	0.87	ICA1L	protein_coding	islet cell aut	2	202773150	202871985
ENSG0000018217	0.043248642	0.779	0.87	TSEN54	protein_coding	tRNA splicir	17	75516060	75524735
ENSG0000027676	-0.784155835	0.779	0.87	NDUFA3	protein_coding	NADH:ubiqu	CHR_HSCHR	54102906	54107026
ENSG0000017306	-0.053996398	0.779	0.87	FAM222B	protein_coding	family with s	17	28755978	28855232
ENSG0000025986	0.082019856	0.779	0.87	AL390728.6	lncRNA	novel transc	1	247187281	247188526
ENSG0000023606	-0.404652237	0.779	0.87	NA	NA	NA	NA	NA	NA
ENSG0000023682	0.068360785	0.779	0.87	LSM2	protein_coding	LSM2 homo	CHR_HSCHR	31784754	31794345
ENSG0000023098	0.059833892	0.78	0.87	VARS2	protein_coding	valyl-tRNA s	CHR_HSCHR	30904916	30917183
ENSG0000022445	0.440766416	0.78	0.87	RSL24D1P6	processed_pse	ribosomal L	20	32170390	32170790
ENSG0000017647	-0.255520337	0.78	0.87	ZNF575	protein_coding	zinc finger p	19	43525497	43536130
ENSG0000025239	0.612325907	0.78	0.87	RNU6-638P	snRNA	RNA, U6 sm	17	78696694	78696797
ENSG0000022865	0.237907771	0.78	0.87	HNRNPCP7	processed_pse	heterogene	7	64500825	64501729
ENSG0000018119	0.025734587	0.78	0.87	PJA1	protein_coding	praja ring fi	X	69160851	69165793
ENSG0000011321	-0.165241326	0.781	0.87	PCDHB7	protein_coding	protocadhe	5	141172644	141176383
ENSG0000018914	0.129139154	0.781	0.87	CLDN4	protein_coding	claudin 4 [S	7	73799542	73832693
ENSG0000023029	-0.26961331	0.781	0.87	GTF2IP23	unprocessed_p	general trar	7	66880708	66882981
ENSG0000024561	0.129552786	0.781	0.87	DDX11-AS1	lncRNA	DDX11 antis	12	31019434	31073847
ENSG0000013809	0.011080087	0.782	0.87	LRPPRC	protein_coding	leucine rich	2	43886224	43995989
ENSG0000027017	-0.257101291	0.782	0.87	AL359881.3	lncRNA	novel transc	1	7693124	7694844
ENSG0000011551	0.027896959	0.782	0.87	TXNDC9	protein_coding	thioredoxin	2	99318982	99340702
ENSG0000010581	0.024378154	0.782	0.87	PMPCB	protein_coding	peptidase, r	7	103297435	103329511
ENSG0000027701	-0.391403698	0.782	0.87	COX8C	protein_coding	cytochrome	CHR_HSCHR	93347191	93348356
ENSG0000023414	-0.317455594	0.782	0.87	AC006042.3	lncRNA	novel transc	7	7949853	7950709
ENSG0000023665	-0.475973079	0.782	0.87	AC079780.1	processed_pse	chromosom	7	23480753	23480959
ENSG0000020723	-0.601617103	0.783	0.87	RNU6-125P	snRNA	RNA, U6 sm	1	88816779	88816885
ENSG0000018396	0.036509296	0.783	0.87	KCNH8	protein_coding	potassium v	3	19148510	19535642
ENSG0000013841	0.016392621	0.783	0.87	IDH1	protein_coding	isocitrate de	2	208236229	208266074
ENSG0000024657	-0.324903844	0.783	0.87	AC093162.1	unprocessed_p	retinol satur	2	85315041	85316529
ENSG0000017615	0.33938451	0.783	0.87	GPX2	protein_coding	glutathione	14	64939152	64942905
ENSG0000023739	0.138575196	0.783	0.87	PITRM1-AS	lncRNA	PITRM1 anti	10	3141632	3167972
ENSG0000027173	-0.284783272	0.783	0.87	AC008608.2	lncRNA	novel transc	5	134005147	134005562
ENSG0000023697	-0.114601649	0.784	0.87	SUMO2P1	processed_pse	SUMO2 pse	CHR_HSCHR	29636180	29636463
ENSG0000023433	0.368993218	0.784	0.87	AC026462.1	processed_pse	tropomyosir	16	52655307	52656045

ENSG0000027849	0.463392522	0.784	0.87	AC006213.5	lncRNA	novel transc	19	43978376	43978663
ENSG0000027199	-0.366629292	0.784	0.87	AL354872.1	lncRNA	novel transc	1	70445071	70445536
ENSG0000022992	-0.109240111	0.785	0.87	TRIM39	protein_coding	tripartite mc	CHR_HSCHR	30316398	30333651
ENSG0000024993	0.179747208	0.785	0.87	RAC1P2	processed_pse	Rac family s	4	46723830	46724408
ENSG0000025486	0.545556242	0.785	0.87	DEFB109D	processed_pse	defensin be	8	12150895	12151134
ENSG0000027419	-0.389363371	0.785	0.87	AC026333.4	lncRNA	novel transc	12	122634130	122634673
ENSG0000008551	-0.028425336	0.786	0.87	MAP3K4	protein_coding	mitogen-ac	6	160991727	161117385
ENSG0000012550	0.134656617	0.786	0.87	MBOAT7	protein_coding	membrane	19	54173412	54189882
ENSG0000022813	-0.116813264	0.786	0.87	SUMO2P1	processed_pse	SUMO2 pse	CHR_HSCHR	29635589	29635872
ENSG0000027611	-0.464085777	0.786	0.87	AC242852.1	unprocessed_p	transmembr	1	143343180	143343275
ENSG0000026780	-0.529591199	0.786	0.87	AC012254.4	processed_pse	SUMO2 SM	18	47137018	47137290
ENSG0000026828	0.236902729	0.786	0.87	AL589765.5	lncRNA	novel transc	1	151766486	151767000
ENSG0000027907	-0.500001341	0.786	0.87	AC091965.5	TEC	TEC	5	8458842	8459183
ENSG0000022595	0.426113973	0.786	0.88	ODF2-AS1	lncRNA	ODF2 antise	9	128468957	128473012
ENSG0000027952	-0.159283359	0.786	0.88	AC093525.8	TEC	tec	16	2516658	2517999
ENSG0000022420	-0.103199889	0.787	0.88	FKBPL	protein_coding	FKBP prolyl	CHR_HSCHR	32104478	32106062
ENSG0000022288	0.429568647	0.787	0.88	Y_RNA	misc_RNA	Y RNA [Sou	17	40391232	40391338
ENSG0000024435	0.517283069	0.787	0.88	RN7SL145P	misc_RNA	RNA, 7SL, cy	3	45742675	45742970
ENSG0000027908	0.065106588	0.787	0.88	AC022400.6	TEC	TEC	10	73742995	73744230
ENSG0000021344	0.103048745	0.787	0.88	SIPA1	protein_coding	signal-induc	11	65638101	65650918
ENSG0000017284	0.01482618	0.787	0.88	SP3	protein_coding	Sp3 transcri	2	173880850	173965702
ENSG0000027179	0.593897981	0.787	0.88	SNORA51	snoRNA	small nucle	20	2655067	2655198
ENSG0000022932	-0.108640431	0.788	0.88	KRT8P12	transcribed_pr	keratin 8 ps	3	160565447	160569248
ENSG0000025757	-0.205256008	0.788	0.88	HSPD1P4	processed_pse	heat shock	12	56511002	56512703
ENSG0000021299	0.102565817	0.788	0.88	RPS26P6	processed_pse	ribosomal p	8	100895771	100896118
ENSG0000014723	0.160785575	0.788	0.88	RADX	protein_coding	RPA1 relate	X	106611930	106679439
ENSG0000023178	-0.400203899	0.788	0.88	RPL31P50	processed_pse	ribosomal p	12	31597114	31597409
ENSG0000022489	0.211555435	0.789	0.88	RPS4XP16	transcribed_pr	ribosomal p	13	51454164	51461315
ENSG0000027139	-0.273146216	0.789	0.88	AC091167.4	processed_pse	MKI67 (FHA	15	90284376	90285580
ENSG0000027269	-0.306424911	0.789	0.88	AL359091.3	lncRNA	novel transc	9	128316337	128316909
ENSG0000021945	0.307907762	0.789	0.88	RPL23P8	processed_pse	ribosomal p	7	20827326	20827753
ENSG0000025603	-0.3099719	0.789	0.88	NA	NA	NA	NA	NA	NA
ENSG0000025261	0.579588493	0.79	0.88	RNU6-807P	snRNA	RNA, U6 sm	15	101648206	101648312
ENSG0000025951	-0.188793021	0.79	0.88	ANP32AP1	transcribed_pr	acidic nucle	15	35181799	35238197
ENSG0000021361	0.023423229	0.79	0.88	NDUFS3	protein_coding	NADH:ubiqu	11	47565336	47584562
ENSG0000024990	-0.273091835	0.79	0.88	BRD9P2	transcribed_un	bromodoma	5	767382	768930
ENSG0000025929	0.209998611	0.79	0.88	NA	NA	NA	NA	NA	NA
ENSG0000027940	-0.272399686	0.79	0.88	NA	NA	NA	NA	NA	NA
ENSG0000026303	0.107121553	0.79	0.88	MTX1P1	unprocessed_p	metaxin 1 p	CHR_HSCHR	155246379	155249729
ENSG0000021302	-0.022258989	0.791	0.88	NUP62	protein_coding	nucleoporin	19	49906825	49929763
ENSG0000019652	-0.014758916	0.791	0.88	AFAP1	protein_coding	actin filame	4	7758714	7939926
ENSG0000026721	-0.306009015	0.791	0.88	AC007773.1	lncRNA	novel transc	19	32390050	32405560
ENSG0000025764	0.171937201	0.791	0.88	AC124312.2	lncRNA	novel transc	15	25027736	25032047
ENSG0000018916	-0.060051158	0.791	0.88	ZNF527	protein_coding	zinc finger p	19	37371061	37393066
ENSG0000014502	-0.044798897	0.791	0.88	TCTA	protein_coding	T cell leuker	3	49412206	49416475
ENSG0000019849	0.024598549	0.792	0.88	TMA16	protein_coding	translation r	4	163494442	163520539
ENSG0000008060	0.021182821	0.792	0.88	PUM3	protein_coding	pumilio RNA	9	2720469	2844095
ENSG0000025585	0.122646893	0.792	0.88	PXN-AS1	lncRNA	PXN antiser	12	120201291	120213231
ENSG0000016885	0.269694132	0.793	0.88	TPTE2P5	transcribed_un	transmembr	13	40812309	40921749
ENSG0000026334	0.272746884	0.793	0.88	AC006435.2	lncRNA	novel transc	17	2375061	2379306
ENSG0000019682	-0.017978908	0.793	0.88	ILRUN	protein_coding	inflammatio	6	34587288	34696859
ENSG0000023395	-0.066226564	0.793	0.88	UQCRHL	protein_coding	ubiquinol-c	1	15807169	15809348
ENSG0000014175	0.024904631	0.793	0.88	TXNL4A	protein_coding	thioredoxin	18	79970813	80033949
ENSG0000022181	-0.280195707	0.794	0.88	GAS8-AS1	lncRNA	GAS8 antise	16	90028908	90029901
ENSG0000022425	-0.39546866	0.794	0.88	AL391427.1	lncRNA	novel transc	10	4995488	4997380
ENSG0000027254	0.304597166	0.794	0.88	AL662797.1	lncRNA	novel transc	6	30723105	30723877
ENSG0000027595	-0.522947315	0.794	0.88	NA	NA	NA	NA	NA	NA
ENSG0000007242	-0.03558346	0.794	0.88	RHOBTB1	protein_coding	Rho related	10	60869438	61001440

ENSG0000017717	0.279334646	0.795	0.88	NAP1L4P1	processed_pse	nucleosome	1	116532936	116534092
ENSG0000022471	0.274837069	0.795	0.88	NPIPA3	protein_coding	nuclear pore	16	14708944	14726338
ENSG0000022557	-0.252348683	0.795	0.88	NCBP2-AS	lncRNA	NCBP2 antis	3	196939877	196942534
ENSG0000023452	-0.113813086	0.795	0.88	SUMO2P1	processed_pse	SUMO2 pse	CHR_HSCHR	29635969	29636252
ENSG0000027214	-0.206850027	0.795	0.88	AC013403.	lncRNA	novel transc	2	27062428	27062907
ENSG0000013732	0.271151384	0.795	0.88	RANP1	processed_pse	RAN pseudo	CHR_HSCHR	30478177	30478827
ENSG0000020649	-0.051110115	0.795	0.88	GNL1	protein_coding	G protein nu	CHR_HSCHR	30530868	30546666
ENSG0000023260	0.447302672	0.795	0.88	TONSL-AS	lncRNA	TONSL antis	8	144437675	144439971
ENSG0000015247	0.163550727	0.795	0.88	ZNF837	protein_coding	zinc finger p	19	58367618	58381030
ENSG0000023332	0.300043851	0.795	0.88	MIPEPP3	transcribed_un	mitochondr	13	21298139	21306373
ENSG0000022392	-0.258684414	0.795	0.88	ASS1P2	processed_pse	argininosuc	2	38810432	38811654
ENSG0000025564	-0.194993352	0.796	0.88	PABPC1P4	processed_pse	poly(A) binc	12	63822021	63823895
ENSG0000027442	-0.198526795	0.796	0.88	SEC22B2P	unprocessed_p	SEC22 hom	1	149345661	149357612
ENSG0000026780	0.221740169	0.796	0.88	NDUFV2P1	processed_pse	NADH:ubiqu	19	53223973	53224879
ENSG0000013615	-0.033260346	0.796	0.88	NUDT15	protein_coding	nudix hydro	13	48037726	48047221
ENSG0000014402	-0.041402207	0.796	0.88	ZNF514	protein_coding	zinc finger p	2	95145000	95165413
ENSG0000014500	0.162417856	0.796	0.88	FAM86B2	protein_coding	family with s	8	12425614	12436343
ENSG0000010979	-0.151548747	0.796	0.88	FAM149A	protein_coding	family with s	4	186104419	186172667
ENSG0000015340	0.072954086	0.796	0.88	NMRAL1	protein_coding	NmrA like re	16	4461680	4495763
ENSG0000005560	-0.013529137	0.797	0.88	KMT2C	protein_coding	lysine methy	7	152134922	152436003
ENSG0000027343	-0.330955676	0.797	0.88	AC108673.	lncRNA	novel transc	3	129163606	129163940
ENSG0000010635	0.023515738	0.797	0.88	LSM5	protein_coding	LSM5 homo	7	32485338	32495283
ENSG0000020693	0.378192999	0.797	0.88	RNU4-52P	snRNA	RNA, U4 sm	X	49082028	49082165
ENSG0000022768	-0.053339724	0.797	0.88	VARS1	protein_coding	valyl-tRNA s	CHR_HSCHR	31781844	31786187
ENSG0000026384	-0.395984796	0.798	0.88	AP005899.	lncRNA	novel transc	18	9112404	9115877
ENSG0000022770	0.294429277	0.798	0.88	RANP1	processed_pse	RAN pseudo	CHR_HSCHR	30475795	30476445
ENSG0000019861	0.03185864	0.798	0.88	PPIAP22	processed_pse	peptidylprol	21	18857779	18858276
ENSG0000022830	-0.182689246	0.798	0.88	AL512770.1	lncRNA	novel transc	10	12244751	12247845
ENSG0000024228	0.122617762	0.798	0.88	AC108488.	lncRNA	novel transc	2	3531813	3536873
ENSG0000027336	0.336899696	0.798	0.88	AL355472.3	lncRNA	novel transc	1	234372186	234372811
ENSG0000017224	-0.047098355	0.799	0.88	C5orf34	protein_coding	chromosom	5	43486709	43515148
ENSG0000015099	-0.015385721	0.799	0.88	ITPR1	protein_coding	inositol 1,4,5	3	4493345	4847840
ENSG0000026075	0.238479342	0.799	0.88	AC092720.	lncRNA	novel transc	16	87595131	87600337
ENSG0000023570	0.160408071	0.799	0.88	LINC00894	lncRNA	long interge	X	149938613	150224580
ENSG0000026405	0.66541638	0.799	0.88	AC073508.	protein_coding	novel transc	17	40627356	40665141
ENSG0000027927	0.072738451	0.799	0.88	AC245060.(	lncRNA	novel transc	22	22264601	22273020
ENSG0000007861	-0.01394679	0.799	0.88	NRDC	protein_coding	nardilysin co	1	51789191	51878937
ENSG0000026495	0.505815218	0.8	0.88	U6	snRNA	U6 spliceos	CHR_HSCHR	31833247	31833349
ENSG0000021537	0.229198586	0.8	0.88	FAM66B	lncRNA	family with s	8	7298744	7355359
ENSG0000022862	-0.086305426	0.8	0.88	HDAC2-AS	lncRNA	HDAC2 and	6	113969701	114471705
ENSG0000023198	0.38913427	0.8	0.88	AC112907.	unprocessed_p	MCF.2 cell li	3	186826549	186827337
ENSG0000026437	-0.474203997	0.801	0.88	MIR3125	miRNA	microRNA 3	2	12737367	12737444
ENSG0000017747	-0.020807211	0.801	0.88	ARIH2	protein_coding	ariadne RBR	3	48918821	48986382
ENSG0000027512	-0.064566004	0.801	0.88	PRXL2B	protein_coding	peroxiredox	CHR_HSCHR	2586491	2591469
ENSG0000025661	-0.164818765	0.801	0.88	AP002414.	transcribed_pr	G protein-c	18	12073232	12076654
ENSG0000006454	-0.041380193	0.801	0.88	TMEM161A	protein_coding	transmembr	19	19119169	19138513
ENSG0000027650	-0.199577408	0.801	0.88	TFPT	protein_coding	TCF3 fusion	CHR_HSCHR	54107066	54115801
ENSG0000013015	0.03242955	0.801	0.88	MOSPD2	protein_coding	motile sperr	X	14873421	14922327
ENSG0000021352	0.032019907	0.802	0.88	SRA1	protein_coding	steroid rece	5	140537340	140558252
ENSG0000017571	-0.097656471	0.802	0.89	B3GNTL1	protein_coding	UDP-GlcNA	17	82942155	83051810
ENSG0000023476	-0.079264337	0.802	0.89	WASH4P	unprocessed_p	WASP family	16	14381	18068
ENSG0000023209	0.188906321	0.803	0.89	DCST1-AS1	lncRNA	DCST1 antis	1	155045191	155046118
ENSG0000026734	0.222297985	0.803	0.89	AC060780.	processed_pse	ribosomal p	17	43169880	43170077
ENSG0000017691	0.346515607	0.803	0.89	C8G	protein_coding	complemen	9	136945185	136946975
ENSG0000024836	0.382700451	0.803	0.89	AC008610.	lncRNA	novel transc	5	180293245	180295253
[truncated: 185,045 more chars]
