# Supplementary material for: The BRD4 Inhibitor dBET57 Exerts Anticancer Effects by Targeting Superenhancer-Related Genes in Neuroblastoma
Source: J Immunol Res. 2022 Nov 16;2022:7945884. doi: 10.1155/2022/7945884 (PMC9691391; doi:10.1155/2022/7945884)
Supplement: Supplementary 4 — Table S4: information for the CCLE of ZMYND8 in this study. [file 7945884.f4.pdf]

Table S4. Expression of ZMYND8 in different tumors

DepMap I	ZMYND8 I	Cell Line N	Primary Di	Lineage	all Primary Disease
ACH-0011	4.514753	LC1SQSF	Lung Canc	Lung	Lung Cancer
ACH-0012	6.4099	COGAR35	Rhabdoid	Soft Tissue	Rhabdoid
ACH-0013	4.035624	COLO794	Skin Cance	Skin	Skin Cancer
ACH-0015	5.754086	KKU213	Bile Duct (	Bile Duct	Bile Duct Cancer
ACH-0002	4.663345	RT4	Bladder Ca	Urinary Tra	Bladder Cancer
ACH-0007	4.235727	SNU283	Colon/Col	Colorectal	Colon/Colorectal Cancer
ACH-0003	3.272023	NCIH1395	Lung Canc	Lung	Lung Cancer
ACH-0002	3.922198	DEL	Lymphom	Lymphocy	Lymphoma
ACH-0004	4.191405	SNU1196	Bile Duct (	Bile Duct	Bile Duct Cancer
ACH-0007	5.350851	LC1F	Lung Canc	Lung	Lung Cancer
ACH-0017	3.762349	93T449	Liposarcor	Soft Tissue	Liposarcoma
ACH-0020	3.577731	TGBC18TK	Colon/Col	Colorectal	Colon/Colorectal Cancer
ACH-0005	3.767655	ABC1	Lung Canc	Lung	Lung Cancer
ACH-0016	4.260778	SKN	Endometri	Uterus	Endometrial/Uterine Cancer
ACH-0001	3.810443	KE97	Myeloma	Plasma Ce	Myeloma
ACH-0007	3.726831	BFTC909	Kidney Ca	Kidney	Kidney Cancer
ACH-0010	3.037382	KCIMOH1	Pancreatic	Pancreas	Pancreatic Cancer
ACH-0005	5.001802	YKG1	Brain Canc	Central Ne	Brain Cancer
ACH-0003	4.31034	MKN1	Gastric Ca	Gastric	Gastric Cancer
ACH-0007	4.037382	LK2	Lung Canc	Lung	Lung Cancer
ACH-0001	4.329841	HS888T	Non-Canc	Fibroblast	Non-Cancerous
ACH-0008	4.395063	ZR7530	Breast Car	Breast	Breast Cancer
ACH-0004	4.405992	SW837	Colon/Col	Colorectal	Colon/Colorectal Cancer
ACH-0007	6.463851	WM983B	Skin Cance	Skin	Skin Cancer
ACH-0013	2.908813	PACADD1	Pancreatic	Pancreas	Pancreatic Cancer
ACH-0000	4.89724	A101D	Skin Cance	Skin	Skin Cancer
ACH-0018	3.911692	OS252	Bone Canc	Bone	Bone Cancer
ACH-0008	4.967169	NCIH446	Lung Canc	Lung	Lung Cancer
ACH-0006	3.432959	PECAPJ34	Head and	Upper Aer	Head and Neck Cancer
ACH-0003	4.269781	TUHR14TK	Kidney Ca	Kidney	Kidney Cancer
ACH-0001	6.19928	COV434	Ovarian Ca	Ovary	Ovarian Cancer
ACH-0007	3.996389	YD10B	Head and	Upper Aer	Head and Neck Cancer
ACH-0020	5.269033	HSSCH2	Sarcoma	Central Ne	Sarcoma
ACH-0015	2.364572	KKU100	Bile Duct (	Bile Duct	Bile Duct Cancer
ACH-0003	4.157852	KMRC3	Kidney Ca	Kidney	Kidney Cancer
ACH-0020	2.70044	HOKUG	Cervical Ca	Cervix	Cervical Cancer
ACH-0007	5.675534	KASUMI2	Leukemia	Blood	Leukemia
ACH-0007	4.316146	NCIH2882	Lung Canc	Lung	Lung Cancer
ACH-0008	3.607626	KYSE140	Esophagea	Esophagus	Esophageal Cancer
ACH-0006	4.192194	MKN7	Gastric Ca	Gastric	Gastric Cancer
ACH-0000	4.285402	MONOMA	Leukemia	Blood	Leukemia
ACH-0020	3.462052	SAS	Head and	Upper Aer	Head and Neck Cancer
ACH-0013	2.615887	PACADD1	Pancreatic	Pancreas	Pancreatic Cancer
ACH-0007	2.124328	L428	Lymphom	Lymphocy	Lymphoma
ACH-0004	5.063934	SKMEL1	Skin Cance	Skin	Skin Cancer
ACH-0001	3.812498	CFPAC1	Pancreatic	Pancreas	Pancreatic Cancer
ACH-0018	4.36247	YSCCC	Bile Duct (	Bile Duct	Bile Duct Cancer
ACH-0003	4.702658	OE33	Esophagea	Esophagus	Esophageal Cancer
ACH-0015	3.15056	LO68	Lung Canc	Lung	Lung Cancer
ACH-0009	4.15056	TE4	Esophagea	Esophagus	Esophageal Cancer
ACH-0024	2.596935	PSS008	Bone Canc	Bone	Bone Cancer
ACH-0009	3.950468	HEC6	Endometri	Uterus	Endometrial/Uterine Cancer
ACH-0014	4.828835	UMUC16	Bladder Ca	Urinary Tra	Bladder Cancer
ACH-0017	3.955127	WAOSEL	Leukemia	Blood	Leukemia
ACH-0001	4.446256	EFM192A	Breast Car	Breast	Breast Cancer
ACH-0009	3.628774	SNU1040	Colon/Col	Colorectal	Colon/Colorectal Cancer

ACH-0006	5.663914	FU97	Gastric Ca	Gastric	Gastric Cancer
ACH-0005	3.460743	59M	Ovarian C	Ovary	Ovarian Cancer
ACH-0001	3.719183	LOUNH91	Lung Canc	Lung	Lung Cancer
ACH-000(	4.234961	SKBR3	Breast Car	Breast	Breast Cancer
ACH-000(	5.194166	MHHCALL	Leukemia	Blood	Leukemia
ACH-0013	5.472163	RPMI2650	Head and	Upper Aer	Head and Neck Cancer
ACH-0005	4.434295	NCIH1975	Lung Canc	Lung	Lung Cancer
ACH-0007	3.917432	NCIH2126	Lung Canc	Lung	Lung Cancer
ACH-0005	3.790772	UACC893	Breast Car	Breast	Breast Cancer
ACH-0013	3.989139	SW156	Kidney Ca	Kidney	Kidney Cancer
ACH-0019	3.938286	NZOV9	Ovarian C	Ovary	Ovarian Cancer
ACH-0013	3.232661	BICR78	Head and	Upper Aer	Head and Neck Cancer
ACH-0005	4.32337	EPLC272H	Lung Canc	Lung	Lung Cancer
ACH-0005	5.238405	IGR39	Skin Cance	Skin	Skin Cancer
ACH-0006	3.82985	LCLC103H	Lung Canc	Lung	Lung Cancer
ACH-0007	3.798051	OAW42	Ovarian C	Ovary	Ovarian Cancer
ACH-0002	3.519793	HCC38	Breast Car	Breast	Breast Cancer
ACH-000(	5.419539	U87MG	Brain Canc	Central Ne	Brain Cancer
ACH-0008	4.978196	BT483	Breast Car	Breast	Breast Cancer
ACH-0016	5.008541	OCILY18	Lymphom	Lymphocy	Lymphoma
ACH-0006	5.379898	HEP3B217	Liver Canc	Liver	Liver Cancer
ACH-0009	4.428946	HEC1B	Endometri	Uterus	Endometrial/Uterine Cancer
ACH-0003	3.072106	CALU3	Lung Canc	Lung	Lung Cancer
ACH-0002	3.673556	AM38	Brain Canc	Central Ne	Brain Cancer
ACH-0016	3.813525	TASK1	Brain Canc	Peripheral	Brain Cancer
ACH-0008	5.116032	CAL51	Breast Car	Breast	Breast Cancer
ACH-0026	4.05398	WM3772F	Eye Cance	Eye	Eye Cancer
ACH-0011	2.017922	MAC2A	Lymphom	Lymphocy	Lymphoma
ACH-0003	3.577731	SF295	Brain Canc	Central Ne	Brain Cancer
ACH-0006	3.9241	L33	Pancreatic	Pancreas	Pancreatic Cancer
ACH-0007	5.100978	NCIH1963	Lung Canc	Lung	Lung Cancer
ACH-001(	3.01078	CHLA9	Bone Canc	Bone	Bone Cancer
ACH-0012	3.538538	TC106	Bone Canc	Bone	Bone Cancer
ACH-0008	4.441616	AGS	Gastric Ca	Gastric	Gastric Cancer
ACH-000(	3.493135	NCIH1650	Lung Canc	Lung	Lung Cancer
ACH-0012	5.078951	STM9101	Rhabdoid	Soft Tissue	Rhabdoid
ACH-0001	3.229588	HS934T	Non-Canc	Fibroblast	Non-Cancerous
ACH-0003	5.16792	HT144	Skin Cance	Skin	Skin Cancer
ACH-0005	5.119771	HT29	Colon/Col	Colorectal	Colon/Colorectal Cancer
ACH-0025	5.90135	WM3211	Skin Cance	Skin	Skin Cancer
ACH-0002	5.042644	ASPC1	Pancreatic	Pancreas	Pancreatic Cancer
ACH-0002	5.194954	SKNAS	Neuroblas	Peripheral	Neuroblastoma
ACH-0016	3.044394	SKGII	Endometri	Uterus	Endometrial/Uterine Cancer
ACH-0006	3.491853	COV644	Ovarian C	Ovary	Ovarian Cancer
ACH-0005	4.118526	LS123	Colon/Col	Colorectal	Colon/Colorectal Cancer
ACH-0006	4.018812	HUT102	Lymphom	Lymphocy	Lymphoma
ACH-0011	3.849999	SCMCRM2	Sarcoma	Soft Tissue	Sarcoma
ACH-0016	3.70044	UMRC7	Kidney Ca	Kidney	Kidney Cancer
ACH-0018	3.438293	ICC12	Bile Duct (	Bile Duct	Bile Duct Cancer
ACH-0006	5.181103	NCIH441	Lung Canc	Lung	Lung Cancer
ACH-0018	4.844486	ICC6	Bile Duct (	Bile Duct	Bile Duct Cancer
ACH-0007	4.413459	CL40	Colon/Col	Colorectal	Colon/Colorectal Cancer
ACH-0013	5.635174	TT	Thyroid Ca	Thyroid	Thyroid Cancer
ACH-0008	3.218781	NCIH1869	Lung Canc	Lung	Lung Cancer
ACH-0015	5.102658	MERO82	Lung Canc	Lung	Lung Cancer
ACH-0003	3.95977	SNU668	Gastric Ca	Gastric	Gastric Cancer
ACH-0002	4.993221	HCC1937	Breast Car	Breast	Breast Cancer
ACH-000(	4.798569	PANC0213	Pancreatic	Pancreas	Pancreatic Cancer

ACH-000(	3.66562	PLB985	Leukemia	Blood	Leukemia
ACH-0018	3.473787	TGBC1TKE	Gallbladde	Bile Duct	Gallbladder Cancer
ACH-0007	4.530445	TE441T	Sarcoma	Soft Tissue	Sarcoma
ACH-0009	3.693766	HEC265	Endometri	Uterus	Endometrial/Uterine Cancer
ACH-0005	4.017031	LCLC97TM	Lung Canc	Lung	Lung Cancer
ACH-0006	4.381283	HS944T	Skin Cance	Skin	Skin Cancer
ACH-0009	4.397118	EN	Endometri	Uterus	Endometrial/Uterine Cancer
ACH-0003	4.110196	YAPC	Pancreatic	Pancreas	Pancreatic Cancer
ACH-0018	3.253989	ICC15	Bile Duct (	Bile Duct	Bile Duct Cancer
ACH-0001	5.043957	NCIH1618	Lung Canc	Lung	Lung Cancer
ACH-0008	3.058316	SF172	Brain Canc	Central Ne	Brain Cancer
ACH-0003	3.8166	SNU1105	Brain Canc	Central Ne	Brain Cancer
ACH-0002	3.307429	HS600T	Non-Canc	Fibroblast	Non-Cancerous
ACH-0005	4.814038	HCC1171	Lung Canc	Lung	Lung Cancer
ACH-0016	4.714795	NP8	Brain Canc	Central Ne	Brain Cancer
ACH-0001	3.223423	HS940T	Non-Canc	Fibroblast	Non-Cancerous
ACH-0004	3.853996	U937	Leukemia	Blood	Leukemia
ACH-0015	2.867896	KMS28PE	Myeloma	Plasma Ce	Myeloma
ACH-0009	2.805292	CW2	Colon/Col	Colorectal	Colon/Colorectal Cancer
ACH-0004	5.224581	SH4	Skin Cance	Skin	Skin Cancer
ACH-0009	5.793636	REH	Leukemia	Blood	Leukemia
ACH-000(	4.508429	GAMG	Brain Canc	Central Ne	Brain Cancer
ACH-0016	3.109361	SKGI	Endometri	Uterus	Endometrial/Uterine Cancer
ACH-0014	2.582556	C125PM	Colon/Col	Colorectal	Colon/Colorectal Cancer
ACH-0013	3.023255	CASKI	Cervical C	Cervix	Cervical Cancer
ACH-0002	3.495695	HS840T	Non-Canc	Fibroblast	Non-Cancerous
ACH-0009	3.719183	HCT116	Colon/Col	Colorectal	Colon/Colorectal Cancer
ACH-0014	4.136684	C10	Colon/Col	Colorectal	Colon/Colorectal Cancer
ACH-0009	4.144046	HT55	Colon/Col	Colorectal	Colon/Colorectal Cancer
ACH-000(	4.976364	NCIH1819	Lung Canc	Lung	Lung Cancer
ACH-0005	3.795975	KMS27	Myeloma	Plasma Ce	Myeloma
ACH-000(	4.854494	MCF7	Breast Car	Breast	Breast Cancer
ACH-0015	4.442943	MUTZ8	Leukemia	Blood	Leukemia
ACH-0003	4.732812	EFM19	Breast Car	Breast	Breast Cancer
ACH-0008	5.08151	JHH7	Liver Canc	Liver	Liver Cancer
ACH-0004	4.327687	NCIH2170	Lung Canc	Lung	Lung Cancer
ACH-0015	3.357552	HEC1	Endometri	Uterus	Endometrial/Uterine Cancer
ACH-0003	3.363171	SR786	Lymphom	Lymphocy	Lymphoma
ACH-0019	4.438293	CCLP1	Bile Duct (	Bile Duct	Bile Duct Cancer
ACH-0014	4.802193	UMUC6	Bladder Ca	Urinary Tra	Bladder Cancer
ACH-0008	5.510646	NCIH510	Lung Canc	Lung	Lung Cancer
ACH-0014	4.296457	TO14	Ovarian C	Ovary	Ovarian Cancer
ACH-0008	4.81455	COLO679	Skin Cance	Skin	Skin Cancer
ACH-0011	4.279471	NHAHTDD	Non-Canc	Central Ne	Non-Cancerous
ACH-0015	4.448901	MEL202	Eye Cance	Eye	Eye Cancer
ACH-0008	3.754888	HGC27	Gastric Ca	Gastric	Gastric Cancer
ACH-0012	4.126808	TIG3TD	Non-Canc	Fibroblast	Non-Cancerous
ACH-0008	4.541019	CAL27	Head and	Upper Aer	Head and Neck Cancer
ACH-0004	5.334139	UO31	Kidney Ca	Kidney	Kidney Cancer
ACH-0013	3.952334	PACADD1	Pancreatic	Pancreas	Pancreatic Cancer
ACH-0013	3.929791	COGN305	Neuroblas	Peripheral	Neuroblastoma
ACH-0006	3.843984	HCC2814	Lung Canc	Lung	Lung Cancer
ACH-0011	1.963474	MYLA	Lymphom	Lymphocy	Lymphoma
ACH-0009	4.268285	HT	Lymphom	Lymphocy	Lymphoma
ACH-0008	3.626439	KNS62	Lung Canc	Lung	Lung Cancer
ACH-0014	4.478972	WERIRB1	Eye Cance	Eye	Eye Cancer
ACH-0005	2.825786	OE21	Esophage	Esophagu	Esophageal Cancer
ACH-0007	4.865424	HCC202	Breast Car	Breast	Breast Cancer

ACH-0005	3.976364	SW1710	Bladder Ca	Urinary Tra	Bladder Cancer
ACH-0007	3.544733	LNZ308	Brain Canc	Central Ne	Brain Cancer
ACH-0017	4.05398	RHJT	Sarcoma	Soft Tissue	Sarcoma
ACH-0007	3.382667	LXF289	Lung Canc	Lung	Lung Cancer
ACH-0007	3.811471	DMS273	Lung Canc	Lung	Lung Cancer
ACH-0013	3.182692	A431	Skin Cance	Skin	Skin Cancer
ACH-0010	2.467279	FEPD	Lymphom	Lymphocy	Lymphoma
ACH-0003	4.190615	NCIH69	Lung Canc	Lung	Lung Cancer
ACH-0016	3.667892	SKGT4	Esophagea	Esophagus	Esophageal Cancer
ACH-0015	3.385431	HKA1	Skin Cance	Skin	Skin Cancer
ACH-0002	4.661065	SNU1079	Bile Duct (	Bile Duct	Bile Duct Cancer
ACH-0015	4.458119	HG3	Leukemia	Blood	Leukemia
ACH-0002	4.811471	HUPT4	Pancreatic	Pancreas	Pancreatic Cancer
ACH-0016	3.769772	NP5	Brain Canc	Central Ne	Brain Cancer
ACH-0014	4.984589	UMUC14	Bladder Ca	Urinary Tra	Bladder Cancer
ACH-0009	4.482848	SNU81	Colon/Col	Colorectal	Colon/Colorectal Cancer
ACH-0014	4.171527	UMUC10	Bladder Ca	Urinary Tra	Bladder Cancer
ACH-0003	3.773996	ISTMES2	Lung Canc	Lung	Lung Cancer
ACH-0014	3.392317	UMUC13	Bladder Ca	Urinary Tra	Bladder Cancer
ACH-0005	5.549669	C32	Skin Cance	Skin	Skin Cancer
ACH-0005	5.537296	DMS153	Lung Canc	Lung	Lung Cancer
ACH-0006	2.916477	KMRC1	Kidney Ca	Kidney	Kidney Cancer
ACH-0007	3.109361	DM3	Non-Canc	Fibroblast	Non-Cancerous
ACH-0008	3.68594	HCC1195	Lung Canc	Lung	Lung Cancer
ACH-0007	2.754888	PECAPJ41	Head and	Upper Aer	Head and Neck Cancer
ACH-0004	3.139142	A704	Kidney Ca	Kidney	Kidney Cancer
ACH-0003	3.412782	IMR32	Neuroblas	Peripheral	Neuroblastoma
ACH-0024	4.412782	HAP1	Leukemia	Blood	Leukemia
ACH-0006	4.0268	HDQP1	Breast Car	Breast	Breast Cancer
ACH-0002	5.067811	CAKI2	Kidney Ca	Kidney	Kidney Cancer
ACH-0014	3.523562	FARAGE	Lymphom	Lymphocy	Lymphoma
ACH-0008	4.040016	647V	Bladder Ca	Urinary Tra	Bladder Cancer
ACH-0002	4.36807	NH6	Neuroblas	Peripheral	Neuroblastoma
ACH-0016	4.68594	NOZ	Gallbladde	Bile Duct	Gallbladder Cancer
ACH-0014	3.297191	921	Eye Cance	Eye	Eye Cancer
ACH-0001	4.106851	SIGM5	Leukemia	Blood	Leukemia
ACH-0001	4.152995	NCIH2444	Lung Canc	Lung	Lung Cancer
ACH-0006	4.491212	SW1573	Lung Canc	Lung	Lung Cancer
ACH-0000	4.040016	D283MED	Brain Canc	Central Ne	Brain Cancer
ACH-0004	4.300124	GSU	Gastric Ca	Gastric	Gastric Cancer
ACH-0006	3.746313	IALM	Lung Canc	Lung	Lung Cancer
ACH-0002	4.857981	NUDUL1	Lymphom	Lymphocy	Lymphoma
ACH-0007	3.344828	KYSE70	Esophagea	Esophagus	Esophageal Cancer
ACH-0005	5.6401	DMS114	Lung Canc	Lung	Lung Cancer
ACH-0020	5.596637	ECC4	Colon/Col	Colorectal	Colon/Colorectal Cancer
ACH-0003	4.045268	KMS12BM	Myeloma	Plasma Ce	Myeloma
ACH-0017	3.291309	CTV1DM	Leukemia	Blood	Leukemia
ACH-0014	3.98823	SW954	Cervical C	Cervix	Cervical Cancer
ACH-0010	3.651913	D458	Brain Canc	Central Ne	Brain Cancer
ACH-0000	5.864929	C2BBE1	Colon/Col	Colorectal	Colon/Colorectal Cancer
ACH-0004	5.809157	NCIH716	Colon/Col	Colorectal	Colon/Colorectal Cancer
ACH-0017	4.300856	VAL	Lymphom	Lymphocy	Lymphoma
ACH-0000	3.439623	HL60	Leukemia	Blood	Leukemia
ACH-0015	3.347666	MM386	Skin Cance	Skin	Skin Cancer
ACH-0009	3.825786	JHUEM7	Endometri	Uterus	Endometrial/Uterine Cancer
ACH-0026	3.66562	170MGBA	Brain Canc	Central Ne	Brain Cancer
ACH-0008	4.461398	EB1	Lymphom	Lymphocy	Lymphoma
ACH-0007	5.196528	JIMT1	Breast Car	Breast	Breast Cancer

ACH-0015	3.264536	HCS2	Cervical C	Cervix	Cervical Cancer
ACH-0028	4.053111	YUHOIN0	Unknown	Unknown	Unknown
ACH-0007	3.936402	L1236	Lymphom	Lymphocy	Lymphoma
ACH-0010	4.486071	BT16	Rhabdoid	Soft Tissue	Rhabdoid
ACH-0006	3.595742	SLR26	Kidney Ca	Kidney	Kidney Cancer
ACH-0003	4.183487	CAPAN1	Pancreatic	Pancreas	Pancreatic Cancer
ACH-0000	4.446918	HCC827	Lung Canc	Lung	Lung Cancer
ACH-0006	5.289466	RH18	Sarcoma	Soft Tissue	Sarcoma
ACH-0004	3.341986	TUHR10TK	Kidney Ca	Kidney	Kidney Cancer
ACH-0014	3.214125	UMUC11	Bladder C	Urinary Tr	Bladder Cancer
ACH-0006	4.573496	SNU878	Liver Canc	Liver	Liver Cancer
ACH-0003	5.123087	CL14	Colon/Col	Colorectal	Colon/Colorectal Cancer
ACH-0002	3.849999	U178	Brain Canc	Central Ne	Brain Cancer
ACH-0003	4.051372	NCIH508	Colon/Col	Colorectal	Colon/Colorectal Cancer
ACH-0002	4.84147	CL11	Colon/Col	Colorectal	Colon/Colorectal Cancer
ACH-0013	7.247453	OACM51	Esophagea	Esophagus	Esophageal Cancer
ACH-0002	4.187451	KALS1	Brain Canc	Central Ne	Brain Cancer
ACH-0009	4.719731	NCIH650	Lung Canc	Lung	Lung Cancer
ACH-0006	5.253989	CORL23	Lung Canc	Lung	Lung Cancer
ACH-0024	5.260402	HT144SKII	Skin Cance	Skin	Skin Cancer
ACH-0013	4.408032	PACADD1	Pancreatic	Pancreas	Pancreatic Cancer
ACH-0010	3.674687	CHLA10	Bone Canc	Bone	Bone Cancer
ACH-0002	4.533563	AU565	Breast Car	Breast	Breast Cancer
ACH-0009	2.639232	RKO	Colon/Col	Colorectal	Colon/Colorectal Cancer
ACH-0007	5.267161	OCIM1	Leukemia	Blood	Leukemia
ACH-0016	2.485427	UPCISCC1	Head and	Upper Aer	Head and Neck Cancer
ACH-0010	3.030336	BECKER	Brain Canc	Central Ne	Brain Cancer
ACH-0002	5.148121	NCIH1755	Lung Canc	Lung	Lung Cancer
ACH-0002	3.775051	RERFLCAI	Lung Canc	Lung	Lung Cancer
ACH-0001	3.903038	PANC1	Pancreatic	Pancreas	Pancreatic Cancer
ACH-0017	3.012569	LPS27	Liposarcor	Soft Tissue	Liposarcoma
ACH-0002	4.502076	UOK101	Kidney Ca	Kidney	Kidney Cancer
ACH-0001	3.795975	OCILY3	Lymphom	Lymphocy	Lymphoma
ACH-0007	3.480265	KIJK	Lymphom	Lymphocy	Lymphoma
ACH-0007	4.9241	SNUC1	Colon/Col	Colorectal	Colon/Colorectal Cancer
ACH-0008	3.458119	CAL851	Breast Car	Breast	Breast Cancer
ACH-0011	4.027685	MONOMA	Leukemia	Blood	Leukemia
ACH-0016	4.19456	OCUG1	Gallbladde	Bile Duct	Gallbladder Cancer
ACH-0001	3.615887	HS343T	Non-Canc	Fibroblast	Non-Cancerous
ACH-0003	2.904966	CCFSTTG1	Brain Canc	Central Ne	Brain Cancer
ACH-0015	4.365972	MM415	Skin Cance	Skin	Skin Cancer
ACH-0008	5.14037	HS939T	Skin Cance	Skin	Skin Cancer
ACH-0017	3.538538	UPCISCC2	Head and	Upper Aer	Head and Neck Cancer
ACH-0001	5.307793	TALL1	Leukemia	Blood	Leukemia
ACH-0005	4.181898	VMCUB1	Bladder C	Urinary Tr	Bladder Cancer
ACH-0009	3.794936	TGBC11TK	Gastric Ca	Gastric	Gastric Cancer
ACH-0001	4.791293	OCILY19	Lymphom	Lymphocy	Lymphoma
ACH-0005	4.755422	ECC10	Gastric Ca	Gastric	Gastric Cancer
ACH-0008	4.68538	FTC238	Thyroid C	Thyroid	Thyroid Cancer
ACH-0016	3.179511	OSC20	Head and	Upper Aer	Head and Neck Cancer
ACH-0006	4.274262	PECAPJ15	Head and	Upper Aer	Head and Neck Cancer
ACH-0019	3.602884	MM485	Skin Cance	Skin	Skin Cancer
ACH-0013	4.012569	COV413A	Ovarian C	Ovary	Ovarian Cancer
ACH-0005	4.690417	HSC4	Head and	Upper Aer	Head and Neck Cancer
ACH-0017	3.673556	WSUNHL	Lymphom	Lymphocy	Lymphoma
ACH-0020	3.574102	OMM25	Eye Cance	Eye	Eye Cancer
ACH-0001	5.509062	NCIH2052	Lung Canc	Lung	Lung Cancer
ACH-0005	2.998196	HS821T	Non-Canc	Fibroblast	Non-Cancerous

ACH-0001	4.635754	OAW28	Ovarian C	Ovary	Ovarian Cancer
ACH-0008	2.750607	KMH2	Lymphom	Lymphocy	Lymphoma
ACH-0012	4.094236	127399	Sarcoma	Soft Tissue	Sarcoma
ACH-0003	2.582556	JHOC5	Ovarian C	Ovary	Ovarian Cancer
ACH-0015	3.258519	MERO84	Lung Canc	Lung	Lung Cancer
ACH-0008	3.664483	SW480	Colon/Col	Colorectal	Colon/Colorectal Cancer
ACH-0008	5.897724	SNU719	Gastric Ca	Gastric	Gastric Cancer
ACH-0005	4.420887	SNU410	Pancreatic	Pancreas	Pancreatic Cancer
ACH-0014	4.697663	UMUC7	Bladder Ca	Urinary Tr	Bladder Cancer
ACH-0006	3.190615	A549	Lung Canc	Lung	Lung Cancer
ACH-0013	4.718088	HA1E	Non-Canc	Kidney	Non-Cancerous
ACH-0000	5.010332	697	Leukemia	Blood	Leukemia
ACH-0004	5.132988	VMRCRCV	Kidney Ca	Kidney	Kidney Cancer
ACH-0001	5.704319	KP3	Pancreatic	Pancreas	Pancreatic Cancer
ACH-0008	4.161081	HUH28	Bile Duct (	Bile Duct	Bile Duct Cancer
ACH-0006	4.980482	SUDHL8	Lymphom	Lymphocy	Lymphoma
ACH-0001	4.465974	KASUMI6	Leukemia	Blood	Leukemia
ACH-0009	3.684819	HCT15	Colon/Col	Colorectal	Colon/Colorectal Cancer
ACH-0008	4.408032	NCIH1048	Lung Canc	Lung	Lung Cancer
ACH-0008	4.843984	LN18	Brain Canc	Central Ne	Brain Cancer
ACH-0002	2.879706	SUPM2	Lymphom	Lymphocy	Lymphoma
ACH-0000	5.186263	SKES1	Bone Canc	Bone	Bone Cancer
ACH-0003	3.049631	HS870T	Non-Canc	Fibroblast	Non-Cancerous
ACH-0020	5.009885	SKMEL19	Skin Canc	Skin	Skin Cancer
ACH-0007	3.837943	MKN74	Gastric Ca	Gastric	Gastric Cancer
ACH-0001	2.430285	HCC1599	Breast Car	Breast	Breast Cancer
ACH-0002	4.873321	DU4475	Breast Car	Breast	Breast Cancer
ACH-0001	4.726286	SW579	Thyroid Ca	Thyroid	Thyroid Cancer
ACH-0000	5.945561	NCO2	Leukemia	Blood	Leukemia
ACH-0005	4.247168	HUT78	Lymphom	Lymphocy	Lymphoma
ACH-0011	5.584662	SKPNDW	Bone Canc	Bone	Bone Cancer
ACH-0005	4.563158	T98G	Brain Canc	Central Ne	Brain Cancer
ACH-0003	4.324811	MHHES1	Bone Canc	Bone	Bone Cancer
ACH-0002	4.012569	KLE	Endometri	Uterus	Endometrial/Uterine Cancer
ACH-0005	3.295723	SNU61	Colon/Col	Colorectal	Colon/Colorectal Cancer
ACH-0005	4.192194	JHOS4	Ovarian C	Ovary	Ovarian Cancer
ACH-0016	3.121015	UPCISCC0	Head and	Upper Aer	Head and Neck Cancer
ACH-0005	4.830357	NCIH1876	Lung Canc	Lung	Lung Cancer
ACH-0009	4.541639	NAMALW	Lymphom	Lymphocy	Lymphoma
ACH-0005	4.687061	TDOTT	Esophage	Esophagus	Esophageal Cancer
ACH-0016	3.570463	SHMAC4	Prostate C	Prostate	Prostate Cancer
ACH-0003	4.751142	NCIH2081	Lung Canc	Lung	Lung Cancer
ACH-0009	4.536053	HEC1A	Endometri	Uterus	Endometrial/Uterine Cancer
ACH-0002	3.070389	LP1	Myeloma	Plasma Ce	Myeloma
ACH-0001	4.313246	BT12	Rhabdoid	Soft Tissue	Rhabdoid
ACH-0009	2.811471	SNU175	Colon/Col	Colorectal	Colon/Colorectal Cancer
ACH-0013	5.164706	H157	Head and	Upper Aer	Head and Neck Cancer
ACH-0006	3.674687	HCC2157	Breast Car	Breast	Breast Cancer
ACH-0002	3.844988	SNU245	Bile Duct (	Bile Duct	Bile Duct Cancer
ACH-0001	3.235727	HS863T	Non-Canc	Fibroblast	Non-Cancerous
ACH-0003	5.334854	NCIH1781	Lung Canc	Lung	Lung Cancer
ACH-0013	4.280214	DOTC2451	Cervical C	Cervix	Cervical Cancer
ACH-0007	3.288359	KMS11	Myeloma	Plasma Ce	Myeloma
ACH-0009	3.430285	PF382	Leukemia	Blood	Leukemia
ACH-0007	3.982765	MM1S	Myeloma	Plasma Ce	Myeloma
ACH-0028	4.171527	PSS131R	Bone Canc	Bone	Bone Cancer
ACH-0016	3.803227	TFK1	Bile Duct (	Bile Duct	Bile Duct Cancer
ACH-0003	5.94228	JURLMK1	Leukemia	Blood	Leukemia

ACH-0003	5.049195	SLR25	Kidney Ca	Kidney	Kidney Cancer
ACH-0009	4.686501	HCC1359	Lung Canc	Lung	Lung Cancer
ACH-0008	4.760753	NCIH524	Lung Canc	Lung	Lung Cancer
ACH-0006	6.243745	CMK	Leukemia	Blood	Leukemia
ACH-0005	4.900625	CALU1	Lung Canc	Lung	Lung Cancer
ACH-0005	3.669027	MC116	Lymphom	Lymphocy	Lymphoma
ACH-0008	4.336283	YD15	Head and	Upper Aer	Head and Neck Cancer
ACH-0009	3.844988	HCC2450	Lung Canc	Lung	Lung Cancer
ACH-0001	5.5157	NCIH3255	Lung Canc	Lung	Lung Cancer
ACH-0004	4.603478	HCC56	Colon/Col	Colorectal	Colon/Colorectal Cancer
ACH-0007	5.745506	HCC1833	Lung Canc	Lung	Lung Cancer
ACH-0006	4.93121	HUH6	Liver Canc	Liver	Liver Cancer
ACH-0005	4.266787	NCIH1184	Lung Canc	Lung	Lung Cancer
ACH-0008	4.420887	COLO684	Endometri	Uterus	Endometrial/Uterine Cancer
ACH-0007	3.171527	TE14	Esophage	Esophagus	Esophageal Cancer
ACH-0001	4.484783	CHP126	Neuroblas	Peripheral	Neuroblastoma
ACH-0020	3.496974	NOS1	Bone Canc	Bone	Bone Cancer
ACH-0004	4.627607	CA46	Lymphom	Lymphocy	Lymphoma
ACH-0014	2.85997	C99	Colon/Col	Colorectal	Colon/Colorectal Cancer
ACH-0008	5.864186	SW1271	Lung Canc	Lung	Lung Cancer
ACH-0016	1.992768	UPCISCC0	Head and	Upper Aer	Head and Neck Cancer
ACH-0000	4.547203	GOS3	Brain Canc	Central Ne	Brain Cancer
ACH-0005	3.444932	HCC78	Lung Canc	Lung	Lung Cancer
ACH-0007	4.038261	SJSA1	Bone Canc	Bone	Bone Cancer
ACH-0016	3.731183	TANOUE	Leukemia	Blood	Leukemia
ACH-0008	4.62293	UMUC1	Bladder Ca	Urinary Tr	Bladder Cancer
ACH-0005	5.74443	RCM1	Colon/Col	Colorectal	Colon/Colorectal Cancer
ACH-0004	5.350851	MESSA	Sarcoma	Soft Tissue	Sarcoma
ACH-0003	5.013462	NCIH2196	Lung Canc	Lung	Lung Cancer
ACH-0020	4.941576	TN2	Neuroblas	Peripheral	Neuroblastoma
ACH-0007	3.795975	P31FUJ	Leukemia	Blood	Leukemia
ACH-0007	6.365798	NCIH854	Lung Canc	Lung	Lung Cancer
ACH-0005	4.488644	KE39	Gastric Ca	Gastric	Gastric Cancer
ACH-0000	3.742006	HCC4006	Lung Canc	Lung	Lung Cancer
ACH-0000	4.352617	PATU8988	Pancreatic	Pancreas	Pancreatic Cancer
ACH-0016	5.38405	SUSA	Teratoma	Embryo	Teratoma
ACH-0003	4.522307	DB	Lymphom	Lymphocy	Lymphoma
ACH-0003	4.877744	HCC1143	Breast Car	Breast	Breast Cancer
ACH-0005	3.576522	PEER	Leukemia	Blood	Leukemia
ACH-0009	4.925525	SNU520	Gastric Ca	Gastric	Gastric Cancer
ACH-0020	5.136273	A375SKIN	Skin Canc	Skin	Skin Cancer
ACH-0005	3.681449	FUOV1	Ovarian C	Ovary	Ovarian Cancer
ACH-0001	2.887525	HS578T	Breast Car	Breast	Breast Cancer
ACH-0024	4.939227	RVH421SK	Skin Canc	Skin	Skin Cancer
ACH-0006	4.401221	OVMANA	Ovarian C	Ovary	Ovarian Cancer
ACH-0003	5.544114	HCC2108	Lung Canc	Lung	Lung Cancer
ACH-0000	4.007196	U118MG	Brain Canc	Central Ne	Brain Cancer
ACH-0010	3.877744	DERL2	Lymphom	Lymphocy	Lymphoma
ACH-0004	5.343408	TYKNU	Ovarian C	Ovary	Ovarian Cancer
ACH-0010	3.599318	CHL1DM	Skin Canc	Skin	Skin Cancer
ACH-0019	4.690976	NO36	Lung Canc	Lung	Lung Cancer
ACH-0003	4.151372	SNU626	Brain Canc	Central Ne	Brain Cancer
ACH-0016	2.166715	PEO1	Ovarian C	Ovary	Ovarian Cancer
ACH-0004	4.674687	SW1088	Brain Canc	Central Ne	Brain Cancer
ACH-0004	4.8885	KO52	Leukemia	Blood	Leukemia
ACH-0013	5.27277	SUM185PI	Breast Car	Breast	Breast Cancer
ACH-0005	2.778209	BICR16	Head and	Upper Aer	Head and Neck Cancer
ACH-0003	4.890447	LN215	Brain Canc	Central Ne	Brain Cancer

ACH-0004	5.484138	NCIH747	Colon/Col	Colorectal	Colon/Colorectal Cancer
ACH-0004	4.060912	SKCO1	Colon/Col	Colorectal	Colon/Colorectal Cancer
ACH-0002	3.033863	HS819T	Non-Canc	Fibroblast	Non-Cancerous
ACH-0003	4.022368	TE10	Esophagea	Esophagus	Esophageal Cancer
ACH-0014	3.950468	TC138	Bone Canc	Bone	Bone Cancer
ACH-0009	3.243364	JHUEM1	Endometri	Uterus	Endometrial/Uterine Cancer
ACH-0008	4.166715	NCIH1651	Lung Canc	Lung	Lung Cancer
ACH-0008	3.891419	KMM1	Myeloma	Plasma Ce	Myeloma
ACH-0007	5.260402	HS695T	Skin Cance	Skin	Skin Cancer
ACH-0016	4.32553	ONDA8	Brain Canc	Central Ne	Brain Cancer
ACH-0007	5.002703	SNU601	Gastric Ca	Gastric	Gastric Cancer
ACH-0008	4.375039	MDAMB41	Breast Car	Breast	Breast Cancer
ACH-0012	4.203201	UW228	Brain Canc	Central Ne	Brain Cancer
ACH-0016	5.124328	OCIM2	Leukemia	Blood	Leukemia
ACH-0002	4.248687	PANC0403	Pancreatic	Pancreas	Pancreatic Cancer
ACH-0003	3.879706	T84	Colon/Col	Colorectal	Colon/Colorectal Cancer
ACH-0019	5.055716	NALM16	Leukemia	Blood	Leukemia
ACH-0013	4.193772	COGN278	Neuroblas	Peripheral	Neuroblastoma
ACH-0008	3.255501	OCIMY5	Myeloma	Plasma Ce	Myeloma
ACH-0001	6.041112	CORL311	Lung Canc	Lung	Lung Cancer
ACH-0005	4.337711	SNU489	Brain Canc	Central Ne	Brain Cancer
ACH-0005	4.814038	ISTMES1	Lung Canc	Lung	Lung Cancer
ACH-0006	4.781884	HCC70	Breast Car	Breast	Breast Cancer
ACH-0001	3.659925	HS274T	Non-Canc	Fibroblast	Non-Cancerous
ACH-0009	4.209453	NCIH2172	Lung Canc	Lung	Lung Cancer
ACH-0014	4.569856	BLUE1	Lymphom	Lymphocy	Lymphoma
ACH-0002	4.971314	OUMS23	Colon/Col	Colorectal	Colon/Colorectal Cancer
ACH-0007	3.477677	CAOV3	Ovarian Ca	Ovary	Ovarian Cancer
ACH-0013	3.420887	MS751	Cervical Ca	Cervix	Cervical Cancer
ACH-0002	4.395063	SF539	Brain Canc	Central Ne	Brain Cancer
ACH-0017	4.263034	CAL72	Bone Canc	Bone	Bone Cancer
ACH-0016	3.941106	OCILY7	Lymphom	Lymphocy	Lymphoma
ACH-0006	4.675816	NCIH211	Lung Canc	Lung	Lung Cancer
ACH-0005	3.349082	NCIH2073	Lung Canc	Lung	Lung Cancer
ACH-0005	3.852998	SQ1	Lung Canc	Lung	Lung Cancer
ACH-0000	5.45253	MUTZ3	Leukemia	Blood	Leukemia
ACH-0000	5.240696	HPAFII	Pancreatic	Pancreas	Pancreatic Cancer
ACH-0003	4.755422	SKHEP1	Liver Canc	Liver	Liver Cancer
ACH-0007	3.726831	RMGI	Ovarian Ca	Ovary	Ovarian Cancer
ACH-0013	5.296457	SUM52PE	Breast Car	Breast	Breast Cancer
ACH-0018	4.636915	ICC8	Bile Duct (	Bile Duct	Bile Duct Cancer
ACH-0003	4.280214	HCC461	Lung Canc	Lung	Lung Cancer
ACH-0020	3.624101	RMSYM	Sarcoma	Soft Tissue	Sarcoma
ACH-0002	3.701549	PL21	Leukemia	Blood	Leukemia
ACH-0007	4.102658	NCIH810	Lung Canc	Lung	Lung Cancer
ACH-0004	3.252476	HS739T	Non-Canc	Fibroblast	Non-Cancerous
ACH-0001	3.633431	TO175T	Non-Canc	Fibroblast	Non-Cancerous
ACH-0001	3.316146	HS839T	Non-Canc	Fibroblast	Non-Cancerous
ACH-0002	4.121844	LN382	Brain Canc	Central Ne	Brain Cancer
ACH-0010	3.370164	143B	Bone Canc	Bone	Bone Cancer
ACH-0004	4.293518	SNU685	Endometri	Uterus	Endometrial/Uterine Cancer
ACH-0020	3.595742	HOUAI	Endometri	Uterus	Endometrial/Uterine Cancer
ACH-0011	3.047887	MTA	Lymphom	Lymphocy	Lymphoma
ACH-0012	3.168321	BIN67	Ovarian Ca	Ovary	Ovarian Cancer
ACH-0000	5.647602	MHHCALL	Leukemia	Blood	Leukemia
ACH-0014	4.53294	VP229	Breast Car	Breast	Breast Cancer
ACH-0004	3.725741	OELE	Non-Canc	Ovary	Non-Cancerous
ACH-0008	3.24184	KYSE450	Esophagea	Esophagus	Esophageal Cancer

ACH-0004	5.307064	ME1	Leukemia	Blood	Leukemia
ACH-0015	4.408032	HSC1	Skin Cance	Skin	Skin Cancer
ACH-0000	3.998196	OPM2	Myeloma	Plasma Ce	Myeloma
ACH-0006	5.116864	NUGC4	Gastric Ca	Gastric	Gastric Cancer
ACH-0006	5.566511	SBC5	Lung Canc	Lung	Lung Cancer
ACH-0005	4.32625	PATU8902	Pancreatic	Pancreas	Pancreatic Cancer
ACH-0002	4.510962	KASUMI1	Leukemia	Blood	Leukemia
ACH-0003	4.386811	SNU5	Gastric Ca	Gastric	Gastric Cancer
ACH-0015	3.658783	MCC142	Skin Cance	Skin	Skin Cancer
ACH-0007	3.896272	KATOIII	Gastric Ca	Gastric	Gastric Cancer
ACH-0025	4.32625	M140325	Skin Cance	Skin	Skin Cancer
ACH-0002	4.454834	SNU1033	Colon/Col	Colorectal	Colon/Colorectal Cancer
ACH-0002	3.667892	KMRC20	Kidney Ca	Kidney	Kidney Cancer
ACH-0002	4.314697	HNT34	Leukemia	Blood	Leukemia
ACH-0001	3.119356	PANC0327	Pancreatic	Pancreas	Pancreatic Cancer
ACH-0017	4.93546	SEMK2	Leukemia	Blood	Leukemia
ACH-0013	3.346248	SUM1315	Breast Car	Breast	Breast Cancer
ACH-0011	4.883621	RT11284	Bladder Ca	Urinary Tra	Bladder Cancer
ACH-0005	5.508112	K562	Leukemia	Blood	Leukemia
ACH-0011	4.974529	CCLFPEDS	Kidney Ca	Kidney	Kidney Cancer
ACH-0000	4.590961	S117	Sarcoma	Soft Tissue	Sarcoma
ACH-0007	2.500802	NCIH1623	Lung Canc	Lung	Lung Cancer
ACH-0004	4.210233	SNU449	Liver Canc	Liver	Liver Cancer
ACH-0003	3.650765	OCIAML3	Leukemia	Blood	Leukemia
ACH-0004	5.27165	KU1919	Bladder Ca	Urinary Tra	Bladder Cancer
ACH-0006	4.258519	OE19	Esophagea	Esophagus	Esophageal Cancer
ACH-0015	4.1127	MERO41	Lung Canc	Lung	Lung Cancer
ACH-0002	4.453518	OCUM1	Gastric Ca	Gastric	Gastric Cancer
ACH-0009	5.463034	OVK18	Ovarian Ca	Ovary	Ovarian Cancer
ACH-0008	5.794156	SNU738	Brain Canc	Central Ne	Brain Cancer
ACH-0004	5.004951	JHH4	Liver Canc	Liver	Liver Cancer
ACH-0005	4.877253	NCIH2171	Lung Canc	Lung	Lung Cancer
ACH-0011	4.604664	OCILY132	Lymphom	Lymphocy	Lymphoma
ACH-0008	3.927896	NCIH661	Lung Canc	Lung	Lung Cancer
ACH-0000	3.389567	HS281T	Non-Canc	Fibroblast	Non-Cancerous
ACH-0004	3.943921	KNS81	Brain Canc	Central Ne	Brain Cancer
ACH-0002	2.493135	HS742T	Non-Canc	Fibroblast	Non-Cancerous
ACH-0016	1.9855	SNU638	Gastric Ca	Gastric	Gastric Cancer
ACH-0009	4.065228	SNU407	Colon/Col	Colorectal	Colon/Colorectal Cancer
ACH-0000	3.926948	HEKTE	Non-Canc	Kidney	Non-Cancerous
ACH-0006	3.933573	NCIH1734	Lung Canc	Lung	Lung Cancer
ACH-0001	2.998196	HS255T	Non-Canc	Fibroblast	Non-Cancerous
ACH-0001	3.462052	KARPAS62	Myeloma	Plasma Ce	Myeloma
ACH-0005	4.603478	HUTU80	Gastric Ca	Gastric	Gastric Cancer
ACH-0016	3.102658	SHI1	Leukemia	Blood	Leukemia
ACH-0016	4.562548	SHMAC5	Prostate C	Prostate	Prostate Cancer
ACH-0001	4.035624	SW1990	Pancreatic	Pancreas	Pancreatic Cancer
ACH-0002	3.411426	HPAC	Pancreatic	Pancreas	Pancreatic Cancer
ACH-0006	4.458119	SNU503	Colon/Col	Colorectal	Colon/Colorectal Cancer
ACH-0020	3.475085	OMM1	Eye Cance	Eye	Eye Cancer
ACH-0006	3.697107	SF268	Brain Canc	Central Ne	Brain Cancer
ACH-0001	5.335926	SKLMS1	Sarcoma	Soft Tissue	Sarcoma
ACH-0014	3.589763	EMTOKA	Endometri	Uterus	Endometrial/Uterine Cancer
ACH-0001	2.65306	SCC25	Head and	Upper Aer	Head and Neck Cancer
ACH-0000	4.554589	BDCM	Leukemia	Blood	Leukemia
ACH-0001	3.558268	THP1	Leukemia	Blood	Leukemia
ACH-0004	3.438293	BICR6	Head and	Upper Aer	Head and Neck Cancer
ACH-0015	2.709291	HEC116	Endometri	Uterus	Endometrial/Uterine Cancer

ACH-0008	3.344828	HCC1438	Lung Canc	Lung	Lung Cancer
ACH-0016	3.039138	OSC19	Head and	Upper Aer	Head and Neck Cancer
ACH-0008	3.991862	CL34	Colon/Col	Colorectal	Colon/Colorectal Cancer
ACH-0008	4.77663	MORCPR	Lung Canc	Lung	Lung Cancer
ACH-0008	3.933573	KYSE510	Esophagea	Esophagus	Esophageal Cancer
ACH-0005	4.786596	NCIH146	Lung Canc	Lung	Lung Cancer
ACH-0005	3.360364	HS606T	Non-Canc	Fibroblast	Non-Cancerous
ACH-0000	3.06695	HH	Lymphom	Lymphocy	Lymphoma
ACH-0001	3.226509	HS737T	Non-Canc	Fibroblast	Non-Cancerous
ACH-0005	3.547203	UACC812	Breast Car	Breast	Breast Cancer
ACH-0007	4.835419	P3HR1	Lymphom	Lymphocy	Lymphoma
ACH-0006	5.086189	BEN	Lung Canc	Lung	Lung Cancer
ACH-0009	4.566815	KCL22	Leukemia	Blood	Leukemia
ACH-0005	4.152183	KMS34	Myeloma	Plasma Ce	Myeloma
ACH-0006	4.581351	SW948	Colon/Col	Colorectal	Colon/Colorectal Cancer
ACH-0024	5.376082	HT144SKIN	Skin Cance	Skin	Skin Cancer
ACH-0001	4.954196	HCC1187	Breast Car	Breast	Breast Cancer
ACH-0024	3.749534	RPE1SS77	Non-Canc	Eye	Non-Cancerous
ACH-0003	6.124535	MOLM16	Leukemia	Blood	Leukemia
ACH-0006	3.751678	SNU1041	Head and	Upper Aer	Head and Neck Cancer
ACH-0004	4.250204	SW1463	Colon/Col	Colorectal	Colon/Colorectal Cancer
ACH-0020	5.134221	PK8	Pancreatic	Pancreas	Pancreatic Cancer
ACH-0001	3.467279	HUPT3	Pancreatic	Pancreas	Pancreatic Cancer
ACH-0006	5.965323	RVH421	Skin Cance	Skin	Skin Cancer
ACH-0013	4.227279	CHP134	Neuroblas	Peripheral	Neuroblastoma
ACH-0004	4.240314	BV173	Leukemia	Blood	Leukemia
ACH-0005	2.827819	SIHA	Cervical C	Cervix	Cervical Cancer
ACH-0018	2.748461	LPS510	Liposarcor	Soft Tissue	Liposarcoma
ACH-0013	5.060912	SCLC22H	Lung Canc	Lung	Lung Cancer
ACH-0010	3.121015	DL40	Lymphom	Lymphocy	Lymphoma
ACH-0015	2.893362	HT3	Cervical C	Cervix	Cervical Cancer
ACH-0015	3.491853	KML1	Lymphom	Lymphocy	Lymphoma
ACH-0000	5.479619	HS294T	Skin Cance	Skin	Skin Cancer
ACH-0027	3.014355	NCCLMS1	Unknown	Soft Tissue	Unknown
ACH-0006	4.283922	KYSE520	Esophagea	Esophagus	Esophageal Cancer
ACH-0026	5.75462	S462	Sarcoma	Peripheral	Sarcoma
ACH-0004	3.805292	LN428	Brain Canc	Central Ne	Brain Cancer
ACH-0003	4.028569	H4	Brain Canc	Central Ne	Brain Cancer
ACH-0027	5.891662	MPNST724	Sarcoma	Peripheral	Sarcoma
ACH-0001	5.448571	NCIH1341	Lung Canc	Lung	Lung Cancer
ACH-0004	2.295723	SF767	Cervical C	Cervix	Cervical Cancer
ACH-0004	3.619413	NCIH1915	Lung Canc	Lung	Lung Cancer
ACH-0010	2.467279	HELA	Cervical C	Cervix	Cervical Cancer
ACH-0020	4.560104	T3M5	Thyroid Ca	Thyroid	Thyroid Cancer
ACH-0005	5.521679	TTC709	Rhabdoid	Soft Tissue	Rhabdoid
ACH-0007	5.464342	GI1	Brain Canc	Central Ne	Brain Cancer
ACH-0003	4.27277	J82	Bladder Ca	Urinary Tra	Bladder Cancer
ACH-0000	5.018812	NCIH684	Colon/Col	Colorectal	Colon/Colorectal Cancer
ACH-0003	4.288359	RL	Lymphom	Lymphocy	Lymphoma
ACH-0002	5.618239	KP4	Pancreatic	Pancreas	Pancreatic Cancer
ACH-0001	3.269033	JHOS2	Ovarian C	Ovary	Ovarian Cancer
ACH-0002	4.521051	JHESOAD1	Esophagea	Esophagus	Esophageal Cancer
ACH-0006	3.743084	HOS	Bone Canc	Bone	Bone Cancer
ACH-0008	4.01614	KYSE150	Esophagea	Esophagus	Esophageal Cancer
ACH-0009	4.966707	ES2	Ovarian C	Ovary	Ovarian Cancer
ACH-0003	5.1918	RPMI7951	Skin Cance	Skin	Skin Cancer
ACH-0011	2.70044	KHYG	Lymphom	Lymphocy	Lymphoma
ACH-0002	4.925525	SCC4	Head and	Upper Aer	Head and Neck Cancer

ACH-0002	4.294988	JHOM1	Ovarian C	Ovary	Ovarian Cancer
ACH-0003	5.250583	HLF	Liver Canc	Liver	Liver Cancer
ACH-0006	4.029453	SNU201	Brain Canc	Central Ne	Brain Cancer
ACH-0003	3.847997	MG63	Bone Canc	Bone	Bone Cancer
ACH-0010	3.50462	CHLA06AT	Rhabdoid	Soft Tissue	Rhabdoid
ACH-0006	3.571677	MDAMB15	Breast Car	Breast	Breast Cancer
ACH-0016	3.261531	SAT	Head and	Upper Aer	Head and Neck Cancer
ACH-0000	3.868884	DOHH2	Lymphom	Lymphocy	Lymphoma
ACH-0004	4.275007	SNU182	Liver Canc	Liver	Liver Cancer
ACH-0005	5.095924	NCIH1092	Lung Canc	Lung	Lung Cancer
ACH-0000	3.997292	TE617T	Sarcoma	Soft Tissue	Sarcoma
ACH-0000	2.523562	HS895T	Non-Canc	Fibroblast	Non-Cancerous
ACH-0009	5.247548	EFO27	Ovarian C	Ovary	Ovarian Cancer
ACH-0007	3.760221	TT2609C0	Thyroid Ca	Thyroid	Thyroid Cancer
ACH-0009	2.443607	MFE319	Endometri	Uterus	Endometrial/Uterine Cancer
ACH-0009	5.164304	MDAPCA2	Prostate C	Prostate	Prostate Cancer
ACH-0000	3.375735	OCIAML5	Leukemia	Blood	Leukemia
ACH-0006	4.559492	SF126	Brain Canc	Central Ne	Brain Cancer
ACH-0014	4.178715	UMUC5	Bladder Ca	Urinary Tra	Bladder Cancer
ACH-0005	4.202418	SNB75	Brain Canc	Central Ne	Brain Cancer
ACH-0009	3.800123	22RV1	Prostate C	Prostate	Prostate Cancer
ACH-0017	3.273516	HS860T	Bone Canc	Bone	Bone Cancer
ACH-0006	4.331992	SUIT2	Pancreatic	Pancreas	Pancreatic Cancer
ACH-0004	2.244887	NCIH1944	Lung Canc	Lung	Lung Cancer
ACH-0004	3.588565	HSC2	Head and	Upper Aer	Head and Neck Cancer
ACH-0000	3.472488	LS513	Colon/Col	Colorectal	Colon/Colorectal Cancer
ACH-0004	4.851499	KPNSI9S	Neuroblas	Peripheral	Neuroblastoma
ACH-0003	5.365972	KG1	Leukemia	Blood	Leukemia
ACH-0018	4.281698	G415	Gallbladde	Bile Duct	Gallbladder Cancer
ACH-0007	3.252476	HCC2218	Breast Car	Breast	Breast Cancer
ACH-0000	5.155425	KPL1	Breast Car	Breast	Breast Cancer
ACH-0006	3.448901	SKMES1	Lung Canc	Lung	Lung Cancer
ACH-0000	3.130931	MHHNB11	Neuroblas	Peripheral	Neuroblastoma
ACH-0020	4.719183	HTMMT	Endometri	Uterus	Endometrial/Uterine Cancer
ACH-0003	5.350497	TEN	Endometri	Uterus	Endometrial/Uterine Cancer
ACH-0002	3.443607	HS822T	Non-Canc	Fibroblast	Non-Cancerous
ACH-0005	4.078951	CAL33	Head and	Upper Aer	Head and Neck Cancer
ACH-0015	3.743084	MM370	Skin Canc	Skin	Skin Cancer
ACH-0002	5.132988	LS1034	Colon/Col	Colorectal	Colon/Colorectal Cancer
ACH-0005	4.790251	G361	Skin Canc	Skin	Skin Cancer
ACH-0016	2.615887	NO10	Brain Canc	Central Ne	Brain Cancer
ACH-0002	6.051155	HCC1419	Breast Car	Breast	Breast Cancer
ACH-0012	5.648178	WM2664	Skin Canc	Skin	Skin Cancer
ACH-0007	4.20085	DAUDI	Lymphom	Lymphocy	Lymphoma
ACH-0008	5.613532	COLO668	Lung Canc	Lung	Lung Cancer
ACH-0019	5.128871	MM253	Skin Canc	Skin	Skin Cancer
ACH-0020	4.432959	HSKTC	Ovarian C	Ovary	Ovarian Cancer
ACH-0005	3.866908	SNU761	Liver Canc	Liver	Liver Cancer
ACH-0004	4.307429	KMS28BM	Myeloma	Plasma Ce	Myeloma
ACH-0007	4.853996	HT1376	Bladder Ca	Urinary Tra	Bladder Cancer
ACH-0005	4.087463	UMUC3	Bladder Ca	Urinary Tra	Bladder Cancer
ACH-0015	4.456806	LU135	Lung Canc	Lung	Lung Cancer
ACH-0007	2.277985	EKVX	Lung Canc	Lung	Lung Cancer
ACH-0007	3.104337	NCIH1385	Lung Canc	Lung	Lung Cancer
ACH-0005	4.929318	BHY	Head and	Upper Aer	Head and Neck Cancer
ACH-0018	3.615887	ICC137	Bile Duct (	Bile Duct	Bile Duct Cancer
ACH-0017	3.321928	LPS141	Liposarcor	Soft Tissue	Liposarcoma
ACH-0015	5.097189	MOLM1	Leukemia	Blood	Leukemia

ACH-0004	5.399171	UACC62	Skin Cance	Skin	Skin Cancer
ACH-0003	5.183089	G402	Kidney Ca	Kidney	Kidney Cancer
ACH-0007	4.919817	JMSU1	Bladder Ca	Urinary Tra	Bladder Cancer
ACH-0002	3.405992	HS834T	Non-Canc	Fibroblast	Non-Cancerous
ACH-0007	4.8212	PECAPJ49	Head and	Upper Aer	Head and Neck Cancer
ACH-0006	3.95977	RAJI	Lymphom	Lymphocy	Lymphoma
ACH-0010	5.734168	F5	Brain Canc	Central Ne	Brain Cancer
ACH-0001	5.139961	PRECLH	Non-Canc	Prostate	Non-Cancerous
ACH-0013	3.69933	SUM229PI	Breast Car	Breast	Breast Cancer
ACH-0004	4.857981	K029AX	Skin Cance	Skin	Skin Cancer
ACH-0008	4.519793	IGR1	Skin Cance	Skin	Skin Cancer
ACH-0015	3.522307	H357	Head and	Upper Aer	Head and Neck Cancer
ACH-0002	3.916477	CADOES1	Bone Canc	Bone	Bone Cancer
ACH-0002	3.872829	SCC15	Head and	Upper Aer	Head and Neck Cancer
ACH-0006	4.927422	KYO1	Leukemia	Blood	Leukemia
ACH-0000	2.754888	PC3	Prostate C	Prostate	Prostate Cancer
ACH-0001	6.293885	SET2	Leukemia	Blood	Leukemia
ACH-0017	2.946731	LPS6	Liposarcor	Soft Tissue	Liposarcoma
ACH-0016	3.109361	SISO	Cervical Ca	Cervix	Cervical Cancer
ACH-0006	3.75809	TE6	Esophagea	Esophagus	Esophageal Cancer
ACH-0004	5.006298	CJM	Skin Cance	Skin	Skin Cancer
ACH-0020	5.084915	MMAC	Skin Cance	Skin	Skin Cancer
ACH-0000	5.07596	HEL	Leukemia	Blood	Leukemia
ACH-0002	4.858976	NCIH2887	Lung Canc	Lung	Lung Cancer
ACH-0002	4.744161	JK1	Leukemia	Blood	Leukemia
ACH-0011	4.389567	SKRC31	Kidney Ca	Kidney	Kidney Cancer
ACH-0020	3.370164	TL1	Lymphom	Lymphocy	Lymphoma
ACH-0014	5.068241	ASH3	Thyroid Ca	Thyroid	Thyroid Cancer
ACH-0006	3.619413	OV7	Ovarian Ca	Ovary	Ovarian Cancer
ACH-0004	4.636915	MEC1	Leukemia	Blood	Leukemia
ACH-0008	4.346248	EJM	Myeloma	Plasma Ce	Myeloma
ACH-0016	5.137094	U2904	Lymphom	Lymphocy	Lymphoma
ACH-0016	5.032982	P4E6	Prostate C	Prostate	Prostate Cancer
ACH-0009	3.849999	NCIH2342	Lung Canc	Lung	Lung Cancer
ACH-0010	5.710393	CMK115	Leukemia	Blood	Leukemia
ACH-0005	3.947666	HT1197	Bladder Ca	Urinary Tra	Bladder Cancer
ACH-0008	2.969012	CAL12T	Lung Canc	Lung	Lung Cancer
ACH-0011	2.163499	OCILY12	Lymphom	Lymphocy	Lymphoma
ACH-0001	4.270529	GA10	Lymphom	Lymphocy	Lymphoma
ACH-0017	5.22265	RH36	Sarcoma	Soft Tissue	Sarcoma
ACH-0000	4.635754	T24	Bladder Ca	Urinary Tra	Bladder Cancer
ACH-0007	5.723286	HMC18	Breast Car	Breast	Breast Cancer
ACH-0000	5.180705	CACO2	Colon/Col	Colorectal	Colon/Colorectal Cancer
ACH-0000	6.046578	NCIH2452	Lung Canc	Lung	Lung Cancer
ACH-0006	4.177121	SW900	Lung Canc	Lung	Lung Cancer
ACH-0010	5.811728	KD	Rhabdoid	Soft Tissue	Rhabdoid
ACH-0006	3.815575	NCIH28	Lung Canc	Lung	Lung Cancer
ACH-0009	3.65306	LOVO	Colon/Col	Colorectal	Colon/Colorectal Cancer
ACH-0000	3.849999	NCIH929	Myeloma	Plasma Ce	Myeloma
ACH-0011	4.846493	KOPN8	Leukemia	Blood	Leukemia
ACH-0003	4.892391	NCIH226	Lung Canc	Lung	Lung Cancer
ACH-0005	3.719183	NCIH1437	Lung Canc	Lung	Lung Cancer
ACH-0005	2.408712	KHM1B	Myeloma	Plasma Ce	Myeloma
ACH-0015	4.327687	HMY1	Skin Cance	Skin	Skin Cancer
ACH-0008	5.833902	HEC50B	Endometri	Uterus	Endometrial/Uterine Cancer
ACH-0004	4.759156	TC71	Bone Canc	Bone	Bone Cancer
ACH-0004	5.137913	OVSAHO	Ovarian Ca	Ovary	Ovarian Cancer
ACH-0006	4.586164	HS746T	Gastric Ca	Gastric	Gastric Cancer

ACH-0006	4.899176	NCIH596	Lung Canc	Lung	Lung Cancer
ACH-0005	4.19456	INA6	Myeloma	Plasma Ce	Myeloma
ACH-0002	3.898208	U251MG	Brain Canc	Central Ne	Brain Cancer
ACH-0008	4.975447	SW403	Colon/Col	Colorectal	Colon/Colorectal Cancer
ACH-0012	4.197708	Y79	Eye Cance	Eye	Eye Cancer
ACH-0011	2.485427	CCLFPEDS	Sarcoma	Soft Tissue	Sarcoma
ACH-0008	3.200065	L540	Lymphom	Lymphocy	Lymphoma
ACH-0006	4.809929	JL1	Lung Canc	Lung	Lung Cancer
ACH-0000	4.398487	SIMA	Neuroblas	Peripheral	Neuroblastoma
ACH-0009	4.066089	CAL148	Breast Car	Breast	Breast Cancer
ACH-0006	4.604071	786O	Kidney Ca	Kidney	Kidney Cancer
ACH-0000	3.537296	ACCMESC	Lung Canc	Lung	Lung Cancer
ACH-0001	4.303781	SKNSH	Neuroblas	Peripheral	Neuroblastoma
ACH-0001	3.604071	CAL29	Bladder Ca	Urinary Tra	Bladder Cancer
ACH-0002	4.44427	KPNYN	Neuroblas	Peripheral	Neuroblastoma
ACH-0006	4.098453	A3KAW	Lymphom	Lymphocy	Lymphoma
ACH-0009	3.491853	HPBALL	Leukemia	Blood	Leukemia
ACH-0002	5.336997	CALU6	Lung Canc	Lung	Lung Cancer
ACH-0008	4.563158	KYSE410	Esophagea	Esophagus	Esophageal Cancer
ACH-0001	4.397118	RH41	Sarcoma	Soft Tissue	Sarcoma
ACH-0000	4.739308	PANC1005	Pancreatic	Pancreas	Pancreatic Cancer
ACH-0000	3.806324	NIHOVCA	Ovarian Ca	Ovary	Ovarian Cancer
ACH-0009	4.019702	LS180	Colon/Col	Colorectal	Colon/Colorectal Cancer
ACH-0006	4.970394	IGR37	Skin Canc	Skin	Skin Cancer
ACH-0010	3.314697	JR	Sarcoma	Soft Tissue	Sarcoma
ACH-0002	4.880686	HUG1N	Gastric Ca	Gastric	Gastric Cancer
ACH-0007	4.136684	YD38	Head and	Upper Aer	Head and Neck Cancer
ACH-0009	4.950935	RCHACV	Leukemia	Blood	Leukemia
ACH-0008	4.787641	HCC1954	Breast Car	Breast	Breast Cancer
ACH-0009	5.14037	HEC108	Endometri	Uterus	Endometrial/Uterine Cancer
ACH-0004	4.214125	SAOS2	Bone Canc	Bone	Bone Cancer
ACH-0009	6.122466	BT474	Breast Car	Breast	Breast Cancer
ACH-0018	3.50462	KMCH1	Bile Duct (	Bile Duct	Bile Duct Cancer
ACH-0006	3.89724	TE9	Esophagea	Esophagus	Esophageal Cancer
ACH-0009	3.542258	MDST8	Colon/Col	Colorectal	Colon/Colorectal Cancer
ACH-0009	2.498251	MOLT16	Leukemia	Blood	Leukemia
ACH-0007	3.987321	NUGC2	Gastric Ca	Gastric	Gastric Cancer
ACH-0015	4.245648	MM127	Skin Canc	Skin	Skin Cancer
ACH-0009	4.205549	SNGM	Endometri	Uterus	Endometrial/Uterine Cancer
ACH-0002	4.337711	NCIH889	Lung Canc	Lung	Lung Cancer
ACH-0017	3.500802	OCIC4P	Ovarian Ca	Ovary	Ovarian Cancer
ACH-0002	4.849999	DANG	Pancreatic	Pancreas	Pancreatic Cancer
ACH-0014	4.148121	UWB1289	Ovarian Ca	Ovary	Ovarian Cancer
ACH-0003	3.527321	MOLM13	Leukemia	Blood	Leukemia
ACH-0008	2.835924	HARA	Lung Canc	Lung	Lung Cancer
ACH-0008	3.485427	HCC366	Lung Canc	Lung	Lung Cancer
ACH-0018	4.712596	HKGZCC	Bile Duct (	Bile Duct	Bile Duct Cancer
ACH-0015	3.635754	IHH4	Thyroid Ca	Thyroid	Thyroid Cancer
ACH-0016	4.69933	SKNO1	Leukemia	Blood	Leukemia
ACH-0000	5.739578	SLR21	Kidney Ca	Kidney	Kidney Cancer
ACH-0001	3.452859	HS751T	Non-Canc	Fibroblast	Non-Cancerous
ACH-0014	4.294253	C84	Colon/Col	Colorectal	Colon/Colorectal Cancer
ACH-0005	3.549669	TM31	Brain Canc	Central Ne	Brain Cancer
ACH-0011	4.250962	SKNEP1	Bone Canc	Bone	Bone Cancer
ACH-0000	3.557042	MV411	Leukemia	Blood	Leukemia
ACH-0007	3.432959	UBLC1	Bladder Ca	Urinary Tra	Bladder Cancer
ACH-0009	4.264536	NCIH2286	Lung Canc	Lung	Lung Cancer
ACH-0005	3.072106	T173	Non-Canc	Fibroblast	Non-Cancerous

ACH-0005	5.773733	CORL88	Lung Canc	Lung	Lung Cancer
ACH-0005	4.456806	JHH2	Liver Canc	Liver	Liver Cancer
ACH-0007	4.542877	MDAMB23	Breast Car	Breast	Breast Cancer
ACH-0006	4.505891	RPMI8402	Leukemia	Blood	Leukemia
ACH-0006	2.889474	HCC44	Lung Canc	Lung	Lung Cancer
ACH-0009	3.578939	5637	Bladder Ca	Urinary Tra	Bladder Cancer
ACH-0014	3.327687	EGI1	Bile Duct (	Bile Duct	Bile Duct Cancer
ACH-0003	5.594847	TF1	Leukemia	Blood	Leukemia
ACH-0025	4.550285	M040416	Skin Cance	Skin	Skin Cancer
ACH-0013	2.682573	NB1643	Neuroblas	Peripheral	Neuroblastoma
ACH-0000	5.243745	253JBV	Bladder Ca	Urinary Tra	Bladder Cancer
ACH-0008	3.436961	AMO1	Myeloma	Plasma Ce	Myeloma
ACH-0011	3.833902	U251MGD	Brain Canc	Central Ne	Brain Cancer
ACH-0008	5.423578	SKMEL24	Skin Cance	Skin	Skin Cancer
ACH-0005	2.671293	RS5	Non-Canc	Fibroblast	Non-Cancerous
ACH-0003	3.790772	HCC1500	Breast Car	Breast	Breast Cancer
ACH-0005	4.025029	OVISE	Ovarian C	Ovary	Ovarian Cancer
ACH-0002	4.679199	EWS502	Bone Canc	Bone	Bone Cancer
ACH-0005	3.890447	CAL78	Bone Canc	Bone	Bone Cancer
ACH-0008	6.105175	COLO783	Skin Cance	Skin	Skin Cancer
ACH-0013	4.517276	CHLA15	Neuroblas	Peripheral	Neuroblastoma
ACH-0005	5.719457	KURAMO(	Ovarian C	Ovary	Ovarian Cancer
ACH-0006	4.221104	SUDHL6	Lymphom	Lymphocy	Lymphoma
ACH-0004	4.44427	769P	Kidney Ca	Kidney	Kidney Cancer
ACH-0007	4.885086	NCIH2291	Lung Canc	Lung	Lung Cancer
ACH-0009	4.192983	FTC133	Thyroid Ca	Thyroid	Thyroid Cancer
ACH-0020	5.715619	T3M3	Endometri	Uterus	Endometrial/Uterine Cancer
ACH-0004	3.478972	CAKI1	Kidney Ca	Kidney	Kidney Cancer
ACH-0004	4.907852	EW8	Bone Canc	Bone	Bone Cancer
ACH-0002	3.313246	COV362	Ovarian C	Ovary	Ovarian Cancer
ACH-0002	4.467932	COLO320	Colon/Col	Colorectal	Colon/Colorectal Cancer
ACH-0017	4.506526	HB1119	Leukemia	Blood	Leukemia
ACH-0013	4.532317	PACADD1	Pancreatic	Pancreas	Pancreatic Cancer
ACH-0001	4.283181	LN319	Brain Canc	Central Ne	Brain Cancer
ACH-0006	4.275752	OVCAR8	Ovarian C	Ovary	Ovarian Cancer
ACH-0000	5.156235	RDES	Bone Canc	Bone	Bone Cancer
ACH-0002	3.132577	HS675T	Non-Canc	Fibroblast	Non-Cancerous
ACH-0007	3.923149	NCIH1703	Lung Canc	Lung	Lung Cancer
ACH-0013	5.07382	8505C	Thyroid Ca	Thyroid	Thyroid Cancer
ACH-0003	4.409391	SKNFI	Neuroblas	Peripheral	Neuroblastoma
ACH-0017	5.048759	RH28	Sarcoma	Soft Tissue	Sarcoma
ACH-0004	4.113534	PANC0813	Pancreatic	Pancreas	Pancreatic Cancer
ACH-0013	4.596339	SUM190PT	Breast Car	Breast	Breast Cancer
ACH-0001	4.206331	CHP212	Neuroblas	Peripheral	Neuroblastoma
ACH-0016	4.627607	UPCISCC0	Head and	Upper Aer	Head and Neck Cancer
ACH-0015	4.087463	KON	Head and	Upper Aer	Head and Neck Cancer
ACH-0025	5.381975	WM4235	Skin Cance	Skin	Skin Cancer
ACH-0004	4.401903	LU65	Lung Canc	Lung	Lung Cancer
ACH-0013	4.495056	NGP	Neuroblas	Peripheral	Neuroblastoma
ACH-0006	4.106013	JJN3	Myeloma	Plasma Ce	Myeloma
ACH-0008	2.776104	SCABER	Bladder Ca	Urinary Tra	Bladder Cancer
ACH-0006	4.381283	LN443	Brain Canc	Central Ne	Brain Cancer
ACH-0015	5.292414	HSC5	Skin Cance	Skin	Skin Cancer
ACH-0000	4.456806	HS611T	Lymphom	Lymphocy	Lymphoma
ACH-0003	4.294988	42MGBA	Brain Canc	Central Ne	Brain Cancer
ACH-0000	3.626439	NCIH1693	Lung Canc	Lung	Lung Cancer
ACH-0018	3.390943	TKKK	Bile Duct (	Bile Duct	Bile Duct Cancer
ACH-0009	4.370164	NCIH1155	Lung Canc	Lung	Lung Cancer

ACH-0001	4.082362	JVM2	Lymphom	Lymphocy	Lymphoma
ACH-0007	4.289097	JHH5	Liver Canc	Liver	Liver Cancer
ACH-0008	3.509696	NCIH1373	Lung Canc	Lung	Lung Cancer
ACH-0019	5.087038	NZM3	Skin Cance	Skin	Skin Cancer
ACH-0007	4.138323	SKMEL5	Skin Cance	Skin	Skin Cancer
ACH-0013	3.430285	C4I	Cervical Ca	Cervix	Cervical Cancer
ACH-0006	4.621759	DMS53	Lung Canc	Lung	Lung Cancer
ACH-0008	4.571677	BFTC905	Bladder Ca	Urinary Tr	Bladder Cancer
ACH-0018	3.255501	ICC108	Bile Duct (	Bile Duct	Bile Duct Cancer
ACH-0005	3.837943	MDAMB43	Breast Car	Breast	Breast Cancer
ACH-0016	2.715893	UPCISCC1	Head and	Upper Aer	Head and Neck Cancer
ACH-0009	3.553361	CCK81	Colon/Col	Colorectal	Colon/Colorectal Cancer
ACH-0015	5.155425	MM383	Skin Cance	Skin	Skin Cancer
ACH-0016	3.085765	SKGT2	Gastric Ca	Gastric	Gastric Cancer
ACH-0008	3.987321	NCIH1435	Lung Canc	Lung	Lung Cancer
ACH-0018	5.347311	TGBC52TK	Bile Duct (	Bile Duct	Bile Duct Cancer
ACH-0006	5.154211	WM1799	Skin Cance	Skin	Skin Cancer
ACH-0015	3.799087	FLO1	Esophagea	Esophagus	Esophageal Cancer
ACH-0011	4.644433	SKMEL2	Skin Cance	Skin	Skin Cancer
ACH-0024	3.488001	RPE1SS51	Non-Canc	Eye	Non-Cancerous
ACH-0003	4.181103	U2OS	Bone Canc	Bone	Bone Cancer
ACH-0004	4.689299	NCIH1666	Lung Canc	Lung	Lung Cancer
ACH-0009	5.818902	SNU1	Gastric Ca	Gastric	Gastric Cancer
ACH-0007	4.643856	CAMA1	Breast Car	Breast	Breast Cancer
ACH-0020	4.786074	HSQ89	Head and	Upper Aer	Head and Neck Cancer
ACH-0003	4.858976	LAMA84	Leukemia	Blood	Leukemia
ACH-0024	4.853497	HT144SKIN	Skin Cance	Skin	Skin Cancer
ACH-0009	3.028569	HUCCT1	Bile Duct (	Bile Duct	Bile Duct Cancer
ACH-0015	3.593354	HCSC1	Cervical Ca	Cervix	Cervical Cancer
ACH-0013	3.730096	LAN2	Neuroblas	Peripheral	Neuroblastoma
ACH-0016	4.216455	SEKI	Skin Cance	Skin	Skin Cancer
ACH-0008	3.444932	CHAGOK1	Lung Canc	Lung	Lung Cancer
ACH-0003	6.395063	WM115	Skin Cance	Skin	Skin Cancer
ACH-0020	4.757023	HSOS1	Bone Canc	Bone	Bone Cancer
ACH-0009	4.063503	HMCB	Skin Cance	Skin	Skin Cancer
ACH-0013	5.713146	PA1	Ovarian Ca	Ovary	Ovarian Cancer
ACH-0003	3.82985	SNU1077	Endometri	Uterus	Endometrial/Uterine Cancer
ACH-0003	3.589763	ECGI10	Esophagea	Esophagus	Esophageal Cancer
ACH-0000	5.709842	MEG01	Leukemia	Blood	Leukemia
ACH-0007	5.299758	NCIH1648	Lung Canc	Lung	Lung Cancer
ACH-0005	5.193772	NCIH2004	Rhabdoid	Soft Tissue	Rhabdoid
ACH-0003	4.871351	NCIH522	Lung Canc	Lung	Lung Cancer
ACH-0001	4.195348	VMRCRCZ	Kidney Ca	Kidney	Kidney Cancer
ACH-0001	4.677508	JHUEM3	Endometri	Uterus	Endometrial/Uterine Cancer
ACH-0005	4.291309	BXPC3	Pancreatic	Pancreas	Pancreatic Cancer
ACH-0007	3.505891	RERFLCAD	Lung Canc	Lung	Lung Cancer
ACH-0001	5.569248	HS766T	Pancreatic	Pancreas	Pancreatic Cancer
ACH-0013	4.950468	8305C	Thyroid Ca	Thyroid	Thyroid Cancer
ACH-0009	4.398487	HEC59	Endometri	Uterus	Endometrial/Uterine Cancer
ACH-0011	3.211012	SHSY5Y	Neuroblas	Peripheral	Neuroblastoma
ACH-0003	4.15866	P12ICHIKA	Leukemia	Blood	Leukemia
ACH-0008	4.350497	NB1	Neuroblas	Peripheral	Neuroblastoma
ACH-0000	5.045268	253J	Bladder Ca	Urinary Tr	Bladder Cancer
ACH-0007	3.363171	NCIH1838	Lung Canc	Lung	Lung Cancer
ACH-0016	2.289834	NH12	Neuroblas	Peripheral	Neuroblastoma
ACH-0008	3.753818	HUNS1	Myeloma	Plasma Ce	Myeloma
ACH-0001	4.70044	SNU308	Gallbladde	Bile Duct	Gallbladder Cancer
ACH-0008	4.595146	SKMEL30	Skin Cance	Skin	Skin Cancer

ACH-0011	4.872336	OC316	Ovarian C	Ovary	Ovarian Cancer
ACH-0007	4.288359	COLO680I	Esophagea	Esophagus	Esophageal Cancer
ACH-0007	4.214125	GB1	Brain Canc	Central Ne	Brain Cancer
ACH-0018	3.754888	ICC3	Bile Duct (	Bile Duct	Bile Duct Cancer
ACH-0026	5.483493	JVE253	Colon/Col	Colorectal	Colon/Colorectal Cancer
ACH-0007	5.405652	NCIH1105	Lung Canc	Lung	Lung Cancer
ACH-0015	4.751678	LS	Neuroblas	Peripheral	Neuroblastoma
ACH-0007	5.476382	MDAMB17	Breast Car	Breast	Breast Cancer
ACH-0015	4.181898	MERO14	Lung Canc	Lung	Lung Cancer
ACH-0013	2.759156	OCIC5X	Ovarian C	Ovary	Ovarian Cancer
ACH-0009	3.536053	KM12	Colon/Col	Colorectal	Colon/Colorectal Cancer
ACH-0008	2.648465	KYSE270	Esophagea	Esophagus	Esophageal Cancer
ACH-0012	4.098453	SYO1	Sarcoma	Soft Tissue	Sarcoma
ACH-0009	3.436961	NCIH157D	Lung Canc	Lung	Lung Cancer
ACH-0013	4.308885	OV17R	Ovarian C	Ovary	Ovarian Cancer
ACH-0028	3.531069	NCCMPNS	Unknown	Peripheral	Unknown
ACH-0010	5.032542	CBAGPN	Bone Canc	Bone	Bone Cancer
ACH-0000	4.319762	G292CLON	Bone Canc	Bone	Bone Cancer
ACH-0004	4.643279	TE11	Esophagea	Esophagus	Esophageal Cancer
ACH-0007	4.700994	SHP77	Lung Canc	Lung	Lung Cancer
ACH-0001	5.836934	TM87	Rhabdoid	Soft Tissue	Rhabdoid
ACH-0006	4.595742	TE1	Esophagea	Esophagus	Esophageal Cancer
ACH-0006	6.073606	M07E	Leukemia	Blood	Leukemia
ACH-0001	5.946965	MHHCALL	Leukemia	Blood	Leukemia
ACH-0020	5.166715	A375SKIN	Skin Cance	Skin	Skin Cancer
ACH-0003	4.865424	KARPAS42	Lymphom	Lymphocy	Lymphoma
ACH-0024	3.346248	CCLFUPGI	Gastric Ca	Gastric	Gastric Cancer
ACH-0002	5.624393	A204	Rhabdoid	Soft Tissue	Rhabdoid
ACH-0004	3.568032	EFE184	Endometri	Uterus	Endometrial/Uterine Cancer
ACH-0009	3.295723	RL952	Endometri	Uterus	Endometrial/Uterine Cancer
ACH-0015	3.78031	MCC13	Skin Cance	Skin	Skin Cancer
ACH-0020	3.785551	HHUA	Endometri	Uterus	Endometrial/Uterine Cancer
ACH-0008	3.588565	SW1783	Brain Canc	Central Ne	Brain Cancer
ACH-0004	3.707083	RERFLCKJ	Lung Canc	Lung	Lung Cancer
ACH-0004	5.205158	MELHO	Skin Cance	Skin	Skin Cancer
ACH-0026	4.714246	MAPACHS	Pancreatic	Pancreas	Pancreatic Cancer
ACH-0008	5.078097	GCT	Sarcoma	Soft Tissue	Sarcoma
ACH-0018	4.424922	LPS853	Liposarco	Soft Tissue	Liposarcoma
ACH-0006	4.098453	IOMMLEE	Brain Canc	Central Ne	Brain Cancer
ACH-0007	3.297191	SNU1214	Head and	Upper Aer	Head and Neck Cancer
ACH-0008	4.381975	NCIH1930	Lung Canc	Lung	Lung Cancer
ACH-0008	4.482203	RS411	Leukemia	Blood	Leukemia
ACH-0009	4.840967	MDAMB45	Breast Car	Breast	Breast Cancer
ACH-0007	4.01078	PC9	Lung Canc	Lung	Lung Cancer
ACH-0001	4.214125	PFEIFFER	Lymphom	Lymphocy	Lymphoma
ACH-0011	4.011675	SMSCTR	Sarcoma	Soft Tissue	Sarcoma
ACH-0014	3.765535	CI	Leukemia	Blood	Leukemia
ACH-0006	3.473787	KYSE180	Esophagea	Esophagus	Esophageal Cancer
ACH-0008	4.574707	SKOV3	Ovarian C	Ovary	Ovarian Cancer
ACH-0001	3.607626	KE37	Leukemia	Blood	Leukemia
ACH-0004	6.032321	SKMEL3	Skin Cance	Skin	Skin Cancer
ACH-0002	4.63285	NCIH209	Lung Canc	Lung	Lung Cancer
ACH-0004	5.239933	MUTZ5	Leukemia	Blood	Leukemia
ACH-0015	5.465974	HEMCSS	Bone Canc	Bone	Bone Cancer
ACH-0001	4.913608	NCCSTCK1	Gastric Ca	Gastric	Gastric Cancer
ACH-0014	3.965323	ESO51	Esophagea	Esophagus	Esophageal Cancer
ACH-0001	4.334139	GMS10	Brain Canc	Central Ne	Brain Cancer
ACH-0008	4.192194	RPMI8226	Myeloma	Plasma Ce	Myeloma

ACH-0015	4.995032	MERO25	Lung Canc	Lung	Lung Cancer
ACH-0009	2.763412	MOLT3	Leukemia	Blood	Leukemia
ACH-0003	3.976364	NCIH647	Lung Canc	Lung	Lung Cancer
ACH-0009	3.936402	ESS1	Endometri	Uterus	Endometrial/Uterine Cancer
ACH-0005	4.238787	HCC364	Lung Canc	Lung	Lung Cancer
ACH-0006	5.368419	LN340	Brain Canc	Central Ne	Brain Cancer
ACH-0013	5.28244	CME1	Sarcoma	Soft Tissue	Sarcoma
ACH-0007	4.848998	NCIH526	Lung Canc	Lung	Lung Cancer
ACH-0016	3.419539	SCC3	Lymphom	Lymphocy	Lymphoma
ACH-0015	2.983678	MCC26	Skin Cance	Skin	Skin Cancer
ACH-0004	4.836429	NCIH2085	Lung Canc	Lung	Lung Cancer
ACH-0008	4.731726	MFE296	Endometri	Uterus	Endometrial/Uterine Cancer
ACH-0018	4.0268	ICC2	Bile Duct (	Bile Duct	Bile Duct Cancer
ACH-0010	3.818646	CHLA32	Bone Canc	Bone	Bone Cancer
ACH-0009	4.076388	GP2D	Colon/Col	Colorectal	Colon/Colorectal Cancer
ACH-0016	3.049631	RAMOS	Lymphom	Lymphocy	Lymphoma
ACH-0001	4.365273	RCC10RGI	Kidney Ca	Kidney	Kidney Cancer
ACH-0004	4.252476	HCC95	Lung Canc	Lung	Lung Cancer
ACH-0001	3.32625	HS229T	Non-Canc	Fibroblast	Non-Cancerous
ACH-0006	4.161888	SUDHL5	Lymphom	Lymphocy	Lymphoma
ACH-0011	2.885574	SMZ1	Lymphom	Lymphocy	Lymphoma
ACH-0009	4.557042	IPC298	Skin Cance	Skin	Skin Cancer
ACH-0004	4.300856	HUH7	Liver Canc	Liver	Liver Cancer
ACH-0007	4.705978	MOLP8	Myeloma	Plasma Ce	Myeloma
ACH-0001	3.195348	CORL105	Lung Canc	Lung	Lung Cancer
ACH-0003	5.100978	NCIH520	Lung Canc	Lung	Lung Cancer
ACH-0014	3.89724	C80	Colon/Col	Colorectal	Colon/Colorectal Cancer
ACH-0009	5.080231	NCIH1573	Lung Canc	Lung	Lung Cancer
ACH-0016	2.799087	NP3	Brain Canc	Central Ne	Brain Cancer
ACH-0019	2.538538	CCSW1	Bile Duct (	Bile Duct	Bile Duct Cancer
ACH-0018	4.041769	RBE	Bile Duct (	Bile Duct	Bile Duct Cancer
ACH-0002	3.957915	EM2	Leukemia	Blood	Leukemia
ACH-0014	3.329124	CII	Leukemia	Blood	Leukemia
ACH-0002	3.815575	NCIH841	Lung Canc	Lung	Lung Cancer
ACH-0001	4.36807	8MGBA	Brain Canc	Central Ne	Brain Cancer
ACH-0003	5.119356	EFO21	Ovarian C	Ovary	Ovarian Cancer
ACH-0004	4.187451	KNS60	Brain Canc	Central Ne	Brain Cancer
ACH-0012	2.9855	UPCISCC1	Head and	Upper Aer	Head and Neck Cancer
ACH-0001	4.511595	RERFGC1E	Gastric Ca	Gastric	Gastric Cancer
ACH-0008	3.927896	NCIH322	Lung Canc	Lung	Lung Cancer
ACH-0000	4.512859	TOV112D	Ovarian C	Ovary	Ovarian Cancer
ACH-0004	3.427606	NCIH460	Lung Canc	Lung	Lung Cancer
ACH-0008	4.62	MELJUSO	Skin Cance	Skin	Skin Cancer
ACH-0003	4.285402	NUDHL1	Lymphom	Lymphocy	Lymphoma
ACH-0019	4.587965	ONE58	Lung Canc	Lung	Lung Cancer
ACH-0001	3.956986	T47D	Breast Car	Breast	Breast Cancer
ACH-0009	5.733625	HCC1569	Breast Car	Breast	Breast Cancer
ACH-0008	5.308157	WM88	Skin Cance	Skin	Skin Cancer
ACH-0003	5.83289	SKNDZ	Neuroblas	Peripheral	Neuroblastoma
ACH-0015	2.127633	HUO9	Bone Canc	Bone	Bone Cancer
ACH-0000	3.759156	SALE	Non-Canc	Lung	Non-Cancerous
ACH-0013	3.747387	ANGMCSS	Brain Canc	Central Ne	Brain Cancer
ACH-0004	4.876271	SW1353	Bone Canc	Bone	Bone Cancer
ACH-0012	2.056584	SCS214	Sarcoma	Soft Tissue	Sarcoma
ACH-0008	4.145677	HOP62	Lung Canc	Lung	Lung Cancer
ACH-0006	5.038261	SNU899	Head and	Upper Aer	Head and Neck Cancer
ACH-0001	4.392317	CAPAN2	Pancreatic	Pancreas	Pancreatic Cancer
ACH-0001	3.917432	HS729	Unknown	Soft Tissue	Unknown

ACH-0003	3.317594	HS688AT	Non-Canc	Fibroblast	Non-Cancerous
ACH-0015	4.549669	JEG3	Endometri	Uterus	Endometrial/Uterine Cancer
ACH-0006	5.202026	SW620	Colon/Col	Colorectal	Colon/Colorectal Cancer
ACH-0005	5.035184	EBC1	Lung Canc	Lung	Lung Cancer
ACH-0000	5.277613	ML1	Thyroid Ca	Thyroid	Thyroid Cancer
ACH-0003	5.369117	KPNRTBM	Neuroblas	Peripheral	Neuroblastoma
ACH-0003	4.499527	SNU886	Liver Canc	Liver	Liver Cancer
ACH-0008	4.784504	MDAMB43	Skin Cance	Skin	Skin Cancer
ACH-0002	4.414136	SNU466	Brain Canc	Central Ne	Brain Cancer
ACH-0014	4.50462	BOKU	Cervical Ca	Cervix	Cervical Cancer
ACH-0003	4.631686	NCIH82	Lung Canc	Lung	Lung Cancer
ACH-0029	4.446256	UPMD1	Unknown	Unknown	Unknown
ACH-0009	4.723012	OC314	Ovarian Ca	Ovary	Ovarian Cancer
ACH-0004	5.339137	RT112	Bladder Ca	Urinary Tra	Bladder Cancer
ACH-0001	3.956057	MFE280	Endometri	Uterus	Endometrial/Uterine Cancer
ACH-0016	3.62293	ONDA9	Brain Canc	Central Ne	Brain Cancer
ACH-0018	3.902074	LPS067	Liposarcor	Soft Tissue	Liposarcoma
ACH-0024	3.454176	RPE1SS11	Non-Canc	Eye	Non-Cancerous
ACH-0013	5.661921	GIMEN	Neuroblas	Peripheral	Neuroblastoma
ACH-0007	2.95977	A427	Lung Canc	Lung	Lung Cancer
ACH-0000	4.922674	HT1080	Sarcoma	Soft Tissue	Sarcoma
ACH-0013	4.307429	PLCPRF5	Liver Canc	Liver	Liver Cancer
ACH-0006	4.535431	MIAPACA2	Pancreatic	Pancreas	Pancreatic Cancer
ACH-0006	3.471187	SNU119	Ovarian Ca	Ovary	Ovarian Cancer
ACH-0018	4.418865	SG231	Bile Duct (	Bile Duct	Bile Duct Cancer
ACH-0009	3.962549	IGROV1	Ovarian Ca	Ovary	Ovarian Cancer
ACH-0006	5.059182	YD8	Head and	Upper Aer	Head and Neck Cancer
ACH-0002	5.208283	ECC12	Gastric Ca	Gastric	Gastric Cancer
ACH-0006	5.78398	A2780	Ovarian Ca	Ovary	Ovarian Cancer
ACH-0006	3.647315	HCC1806	Breast Car	Breast	Breast Cancer
ACH-0006	4.216455	HCC1395	Breast Car	Breast	Breast Cancer
ACH-0009	4.361066	BCP1	Lymphom	Lymphocy	Lymphoma
ACH-0006	3.956986	CI1	Lymphom	Lymphocy	Lymphoma
ACH-0000	5.516015	MDAMB13	Breast Car	Breast	Breast Cancer
ACH-0007	5.077243	A253	Head and	Upper Aer	Head and Neck Cancer
ACH-0004	3.578939	NCIH2228	Lung Canc	Lung	Lung Cancer
ACH-0014	4.266787	UMUC9	Bladder Ca	Urinary Tra	Bladder Cancer
ACH-0003	3.765535	MPP89	Lung Canc	Lung	Lung Cancer
ACH-0001	3.464668	EOL1	Leukemia	Blood	Leukemia
ACH-0002	4.077243	SNU840	Ovarian Ca	Ovary	Ovarian Cancer
ACH-0001	5.667041	LOUCY	Leukemia	Blood	Leukemia
ACH-0018	3.732269	ICC106	Bile Duct (	Bile Duct	Bile Duct Cancer
ACH-0008	3.792855	NCIH1563	Lung Canc	Lung	Lung Cancer
ACH-0004	5.273889	SNU423	Liver Canc	Liver	Liver Cancer
ACH-0000	4.289097	PANC0203	Pancreatic	Pancreas	Pancreatic Cancer
ACH-0020	3.161888	HO1U1	Head and	Upper Aer	Head and Neck Cancer
ACH-0002	4.233428	SNU398	Liver Canc	Liver	Liver Cancer
ACH-0000	4.19456	PANC0504	Pancreatic	Pancreas	Pancreatic Cancer
ACH-0004	5.72792	MALME3M	Skin Cance	Skin	Skin Cancer
ACH-0007	3.040892	GSS	Gastric Ca	Gastric	Gastric Cancer
ACH-0003	5.048759	MKN45	Gastric Ca	Gastric	Gastric Cancer
ACH-0004	5.025029	CAL54	Kidney Ca	Kidney	Kidney Cancer
ACH-0009	5.041769	NCIH2106	Lung Canc	Lung	Lung Cancer
ACH-0002	3.496974	DKMG	Brain Canc	Central Ne	Brain Cancer
ACH-0006	4.832384	NCIH2227	Lung Canc	Lung	Lung Cancer
ACH-0000	5.513175	GDM1	Leukemia	Blood	Leukemia
ACH-0002	3.289834	LMSU	Gastric Ca	Gastric	Gastric Cancer
ACH-0010	4.678635	COGE352	Bone Canc	Bone	Bone Cancer

ACH-0003	3.571677	HCC1428	Breast Car	Breast	Breast Cancer
ACH-0020	4.054848	MEL290	Eye Cance	Eye	Eye Cancer
ACH-0004	3.370164	NCIH838	Lung Canc	Lung	Lung Cancer
ACH-0007	3.896272	NCIH196	Lung Canc	Lung	Lung Cancer
ACH-0008	4.324811	KMBC2	Bladder Ca	Urinary Tr	Bladder Cancer
ACH-0002	5.308157	SW1417	Colon/Col	Colorectal	Colon/Colorectal Cancer
ACH-0004	4.588565	COLO800	Skin Cance	Skin	Skin Cancer
ACH-0007	4.478972	HCC2279	Lung Canc	Lung	Lung Cancer
ACH-0008	3.554589	NCIH2347	Lung Canc	Lung	Lung Cancer
ACH-0006	4.214902	U266B1	Myeloma	Plasma Ce	Myeloma
ACH-0005	3.65306	BC3C	Bladder Ca	Urinary Tr	Bladder Cancer
ACH-0001	4.898692	SNU869	Bile Duct (	Bile Duct	Bile Duct Cancer
ACH-0004	4.678072	RERFLCSQ	Lung Canc	Lung	Lung Cancer
ACH-0001	5.354382	OSRC2	Kidney Ca	Kidney	Kidney Cancer
ACH-0009	3.505891	SNU349	Kidney Ca	Kidney	Kidney Cancer
ACH-0001	4.38405	BHT101	Thyroid Ca	Thyroid	Thyroid Cancer
ACH-0002	3.928844	PK59	Pancreatic	Pancreas	Pancreatic Cancer
ACH-0001	4.436961	CAOV4	Ovarian C	Ovary	Ovarian Cancer
ACH-0007	5.239551	HEPG2	Liver Canc	Liver	Liver Cancer
ACH-0015	4.139142	MERO48A	Lung Canc	Lung	Lung Cancer
ACH-0002	4.488644	C8166	Lymphom	Lymphocy	Lymphoma
ACH-0008	4.804776	NCIH2087	Lung Canc	Lung	Lung Cancer
ACH-0006	4.92505	SKMEL28	Skin Cance	Skin	Skin Cancer
ACH-0016	2.867896	OCIAML4	Leukemia	Blood	Leukemia
ACH-0016	2.707083	NP2	Brain Canc	Central Ne	Brain Cancer
ACH-0008	5.135863	HS936T	Skin Cance	Skin	Skin Cancer
ACH-0005	3.190615	HS618T	Non-Canc	Fibroblast	Non-Cancerous
ACH-0004	3.85997	CAS1	Brain Canc	Central Ne	Brain Cancer
ACH-0006	4.250204	KNS42	Brain Canc	Central Ne	Brain Cancer
ACH-0004	5.104337	TE8	Esophagea	Esophagus	Esophageal Cancer
ACH-0013	3.089159	SUM149PT	Breast Car	Breast	Breast Cancer
ACH-0014	4.209453	SW13	Adrenal C	Adrenal C	Adrenal Cancer
ACH-0003	3.870858	SUDHL4	Lymphom	Lymphocy	Lymphoma
ACH-0014	2.615887	TC205	Bone Canc	Bone	Bone Cancer
ACH-0004	3.435629	HUH1	Liver Canc	Liver	Liver Cancer
ACH-0008	4.525443	NCIH358	Lung Canc	Lung	Lung Cancer
ACH-0000	5.129283	KU812	Leukemia	Blood	Leukemia
ACH-0003	3.476382	SNU478	Bile Duct (	Bile Duct	Bile Duct Cancer
ACH-0015	3.447579	MERO95	Lung Canc	Lung	Lung Cancer
ACH-0002	4.580748	KELLY	Neuroblas	Peripheral	Neuroblastoma
ACH-0013	4.237258	SUM102PT	Breast Car	Breast	Breast Cancer
ACH-0014	3.269033	UMUC4	Bladder Ca	Urinary Tr	Bladder Cancer
ACH-0020	3.565597	TT1TKB	Colon/Col	Colorectal	Colon/Colorectal Cancer
ACH-0007	5.201634	TCCSUP	Bladder Ca	Urinary Tr	Bladder Cancer
ACH-0000	3.065228	D341	Brain Canc	Central Ne	Brain Cancer
ACH-0013	3.168321	GP5D	Colon/Col	Colorectal	Colon/Colorectal Cancer
ACH-0005	4.36807	A172	Brain Canc	Central Ne	Brain Cancer
ACH-0006	3.18428	SUDHL1	Lymphom	Lymphocy	Lymphoma
ACH-0001	4.568032	NCIH660	Prostate C	Prostate	Prostate Cancer
ACH-0003	4.767655	MSTO211	Lung Canc	Lung	Lung Cancer
ACH-0009	4.801676	AN3CA	Endometri	Uterus	Endometrial/Uterine Cancer
ACH-0006	4.584963	KS1	Brain Canc	Central Ne	Brain Cancer
ACH-0002	3.661065	DETROIT5	Head and	Upper Aer	Head and Neck Cancer
ACH-0007	4.745775	SH10TC	Gastric Ca	Gastric	Gastric Cancer
ACH-0009	3.058316	JURKAT	Leukemia	Blood	Leukemia
ACH-0003	3.568032	SKM1	Leukemia	Blood	Leukemia
ACH-0009	4.484138	SUPT1	Leukemia	Blood	Leukemia
ACH-0007	3.498251	KMRC2	Kidney Ca	Kidney	Kidney Cancer

ACH-0008	4.82069	FADU	Head and	Upper Aer	Head and Neck Cancer
ACH-0017	4.840967	VAESBJ	Sarcoma	Soft Tissue	Sarcoma
ACH-0003	5.876762	JHOM2B	Ovarian C	Ovary	Ovarian Cancer
ACH-0020	4.57289	P30OHK	Leukemia	Blood	Leukemia
ACH-0009	4.078951	NCIH2110	Lung Canc	Lung	Lung Cancer
ACH-0013	4.036503	SUM44PE	Breast Car	Breast	Breast Cancer
ACH-0009	3.866908	2313287	Gastric Ca	Gastric	Gastric Cancer
ACH-0020	5.516646	MEL285	Eye Cance	Eye	Eye Cancer
ACH-0009	3.747387	DND41	Leukemia	Blood	Leukemia
ACH-0006	3.039138	OVCAR4	Ovarian C	Ovary	Ovarian Cancer
ACH-0003	3.72792	NCIH2122	Lung Canc	Lung	Lung Cancer
ACH-0012	6.124328	TTC1240	Rhabdoid	Soft Tissue	Rhabdoid
ACH-0018	3.68594	ICC10	Bile Duct (	Bile Duct	Bile Duct Cancer
ACH-0003	3.941106	LUDLU1	Lung Canc	Lung	Lung Cancer
ACH-0004	4.658783	PK45H	Pancreatic	Pancreas	Pancreatic Cancer
ACH-0010	3.401903	DLD1	Colon/Col	Colorectal	Colon/Colorectal Cancer
ACH-0000	3.646163	MJ	Lymphom	Lymphocy	Lymphoma
ACH-0004	4.539159	BCPAP	Thyroid Ca	Thyroid	Thyroid Cancer
ACH-0004	4.470537	NCIH1792	Lung Canc	Lung	Lung Cancer
ACH-0025	4.324091	MM16011	Skin Cance	Skin	Skin Cancer
ACH-0000	3.794936	RERFLCMS	Lung Canc	Lung	Lung Cancer
ACH-0024	4.078951	RPE1SS6	Non-Canc	Eye	Non-Cancerous
ACH-0000	2.895303	HS172T	Non-Canc	Fibroblast	Non-Cancerous
ACH-0005	5.983678	BT20	Breast Car	Breast	Breast Cancer
ACH-0000	3.526069	HS706T	Non-Canc	Fibroblast	Non-Cancerous
ACH-0020	5.136273	UACC62SI	Skin Cance	Skin	Skin Cancer
ACH-0008	3.726831	HCC15	Lung Canc	Lung	Lung Cancer
ACH-0013	4.914565	PACADD1	Pancreatic	Pancreas	Pancreatic Cancer
ACH-0020	5.045268	P2URK562	Leukemia	Blood	Leukemia
ACH-0003	3.488001	SKRC20	Kidney Ca	Kidney	Kidney Cancer
ACH-0004	4.422233	SNU1197	Colon/Col	Colorectal	Colon/Colorectal Cancer
ACH-0004	4.820179	NCIN87	Gastric Ca	Gastric	Gastric Cancer
ACH-0009	4.028569	NALM6	Leukemia	Blood	Leukemia
ACH-0004	5.205158	NALM1	Leukemia	Blood	Leukemia
ACH-0009	4.662775	SKUT1	Sarcoma	Soft Tissue	Sarcoma
ACH-0003	4.615887	SNU620	Gastric Ca	Gastric	Gastric Cancer
ACH-0005	4.764474	HCC33	Lung Canc	Lung	Lung Cancer
ACH-0010	5.457463	CHLA57	Unknown	Central Ne	Unknown
ACH-0009	4.060912	IM95	Gastric Ca	Gastric	Gastric Cancer
ACH-0008	4.659925	DBTRG05M	Brain Canc	Central Ne	Brain Cancer
ACH-0006	5.132577	HMEL	Non-Canc	Breast	Non-Cancerous
ACH-0005	3.954196	SNU16	Gastric Ca	Gastric	Gastric Cancer
ACH-0010	3.519793	CW9019	Sarcoma	Soft Tissue	Sarcoma
ACH-0018	2.998196	C396	Bone Canc	Bone	Bone Cancer
ACH-0013	2.952334	RO82W1	Thyroid Ca	Thyroid	Thyroid Cancer
ACH-0007	2.9241	HLFA	Non-Canc	Fibroblast	Non-Cancerous
ACH-0002	4.792855	MINO	Lymphom	Lymphocy	Lymphoma
ACH-0015	4.147307	KARPAS17	Lymphom	Lymphocy	Lymphoma
ACH-0008	4.082362	WM793	Skin Cance	Skin	Skin Cancer
ACH-0009	4.014355	ISHIKAWA	Endometri	Uterus	Endometrial/Uterine Cancer
ACH-0007	3.733354	HSC3	Head and	Upper Aer	Head and Neck Cancer
ACH-0008	4.373648	HOP92	Lung Canc	Lung	Lung Cancer
ACH-0007	4.486714	CORL95	Lung Canc	Lung	Lung Cancer
ACH-0001	5.159871	HDMYZ	Unknown	Unknown	Unknown
ACH-0018	4.946263	MFM223	Breast Car	Breast	Breast Cancer
ACH-0001	4.530445	A4FUK	Lymphom	Lymphocy	Lymphoma
ACH-0017	2.650765	SNU1544	Colon/Col	Colorectal	Colon/Colorectal Cancer
ACH-0002	5.838195	COLO201	Colon/Col	Colorectal	Colon/Colorectal Cancer

ACH-0013	4.50716	PACADD1	Pancreatic	Pancreas	Pancreatic Cancer
ACH-0009	3.863938	SW48	Colon/Col	Colorectal	Colon/Colorectal Cancer
ACH-0002	4.874797	BICR31	Head and	Upper Aer	Head and Neck Cancer
ACH-0002	4.478972	SNU213	Pancreatic	Pancreas	Pancreatic Cancer
ACH-0017	3.927896	TTC442	Sarcoma	Soft Tissue	Sarcoma
ACH-0001	4.799605	CAL62	Thyroid Ca	Thyroid	Thyroid Cancer
ACH-0002	3.793896	BL41	Lymphom	Lymphocy	Lymphoma
ACH-0012	4.874305	SCCOHT1	Ovarian Ca	Ovary	Ovarian Cancer
ACH-0006	5.310704	JHH1	Liver Canc	Liver	Liver Cancer
ACH-0008	3.177918	T3M10	Lung Canc	Lung	Lung Cancer
ACH-0015	2.403268	HCA1	Cervical C	Cervix	Cervical Cancer
ACH-0001	3.638074	NOMO1	Leukemia	Blood	Leukemia
ACH-0002	5.466953	CORL279	Lung Canc	Lung	Lung Cancer
ACH-0002	4.568032	DAOY	Brain Canc	Central Ne	Brain Cancer
ACH-0002	5.418865	A1207	Brain Canc	Central Ne	Brain Cancer
ACH-0020	3.720278	HOTHC	Thyroid Ca	Thyroid	Thyroid Cancer
ACH-0009	5.481234	MEWO	Skin Cance	Skin	Skin Cancer
ACH-0006	4.612352	SNU1066	Head and	Upper Aer	Head and Neck Cancer
ACH-0000	4.585563	GRANTA5	Lymphom	Lymphocy	Lymphoma
ACH-0002	5.468909	CAL120	Breast Car	Breast	Breast Cancer
ACH-0000	5.555816	G401	Rhabdoid	Kidney	Rhabdoid
ACH-0014	4.274262	CCLFPEDS	Sarcoma	Soft Tissue	Sarcoma
ACH-0000	3.266037	HS616T	Non-Canc	Fibroblast	Non-Cancerous
ACH-0019	3.976364	9505BIK	Pancreatic	Pancreas	Pancreatic Cancer
ACH-0007	4.592756	ONS76	Brain Canc	Central Ne	Brain Cancer
ACH-0005	4.314697	NCIH2030	Lung Canc	Lung	Lung Cancer
ACH-0000	4.512859	CH157MN	Brain Canc	Central Ne	Brain Cancer
ACH-0005	4.72792	LN235	Brain Canc	Central Ne	Brain Cancer
ACH-0017	5.303415	GOTO	Neuroblas	Peripheral	Neuroblastoma
ACH-0008	2.763412	RH30	Sarcoma	Soft Tissue	Sarcoma
ACH-0016	3.980939	PEA1	Ovarian Ca	Ovary	Ovarian Cancer
ACH-0003	4.384741	RI1	Lymphom	Lymphocy	Lymphoma
ACH-0002	4.881175	HS852T	Skin Cance	Skin	Skin Cancer
ACH-0013	4.545351	OCIP5X	Ovarian Ca	Ovary	Ovarian Cancer
ACH-0000	5.001352	HCC827GI	Lung Canc	Lung	Lung Cancer
ACH-0018	2.448901	ICC9	Bile Duct (	Bile Duct	Bile Duct Cancer
ACH-0006	5.149341	COLO829	Skin Cance	Skin	Skin Cancer
ACH-0014	4.183487	WPE1NA2	Prostate C	Prostate	Prostate Cancer
ACH-0005	3.66562	KMS26	Myeloma	Plasma Ce	Myeloma
ACH-0008	4.521051	MDAMB4(	Breast Car	Breast	Breast Cancer
ACH-0002	5.835924	A375	Skin Cance	Skin	Skin Cancer
ACH-0001	4.907852	HCC2935	Lung Canc	Lung	Lung Cancer
ACH-0018	4.231893	ICC5	Bile Duct (	Bile Duct	Bile Duct Cancer
ACH-0003	5.154211	SW780	Bladder Ca	Urinary Tr	Bladder Cancer
ACH-0010	2.841973	D425	Brain Canc	Central Ne	Brain Cancer
ACH-0012	4.602291	SW982	Sarcoma	Soft Tissue	Sarcoma
ACH-0000	4.847496	OV56	Ovarian Ca	Ovary	Ovarian Cancer
ACH-0020	4.104337	MEL270	Eye Cance	Eye	Eye Cancer
ACH-0016	4.284662	PGA1	Leukemia	Blood	Leukemia
ACH-0004	4.459432	TUHR4TKE	Kidney Ca	Kidney	Kidney Cancer
ACH-0009	5.152995	NUGC3	Gastric Ca	Gastric	Gastric Cancer
ACH-0000	3.464668	ONCODG	Ovarian Ca	Ovary	Ovarian Cancer
ACH-0007	5.346957	KYSE30	Esophagea	Esophagus	Esophageal Cancer
ACH-0017	4.830357	RH4	Sarcoma	Soft Tissue	Sarcoma
ACH-0003	5.3067	SKNBE2	Neuroblas	Peripheral	Neuroblastoma
ACH-0000	4.505256	T3M4	Pancreatic	Pancreas	Pancreatic Cancer
ACH-0004	3.69265	OCIMY7	Myeloma	Plasma Ce	Myeloma
ACH-0008	3.468583	NCIH1568	Lung Canc	Lung	Lung Cancer

ACH-0002	3.155425	HDLM2	Lymphom	Lymphocy	Lymphoma
ACH-0004	3.523562	SW1116	Colon/Col	Colorectal	Colon/Colorectal Cancer
ACH-0004	5.270529	NCIH1694	Lung Canc	Lung	Lung Cancer
ACH-0001	3.289834	M059K	Brain Canc	Central Ne	Brain Cancer
ACH-0009	5.395748	JHUEM2	Endometri	Uterus	Endometrial/Uterine Cancer
ACH-0005	4.137504	WSUDLCL	Lymphom	Lymphocy	Lymphoma
ACH-0005	4.843984	COLO741	Skin Cance	Skin	Skin Cancer
ACH-0004	4.531693	SNU8	Ovarian C	Ovary	Ovarian Cancer
ACH-0004	3.377124	OVKATE	Ovarian C	Ovary	Ovarian Cancer
ACH-0015	3.620586	MM426	Skin Cance	Skin	Skin Cancer
ACH-0009	3.039138	HT115	Colon/Col	Colorectal	Colon/Colorectal Cancer
ACH-0013	3.793896	C4II	Cervical C	Cervix	Cervical Cancer
ACH-0000	4.728465	PATU8988	Pancreatic	Pancreas	Pancreatic Cancer
ACH-0020	4.713146	SLVL	Lymphom	Lymphocy	Lymphoma
ACH-0024	4.161081	RPE1SS48	Non-Canc	Eye	Non-Cancerous
ACH-0001	4.703211	JM1	Leukemia	Blood	Leukemia
ACH-0012	3.611172	YAMATO	Sarcoma	Soft Tissue	Sarcoma
ACH-0006	6.218587	KYM1	Rhabdoid	Soft Tissue	Rhabdoid
ACH-0026	3.593354	CCC5	Gastric Ca	Gastric	Gastric Cancer
ACH-0007	4.739308	DMS79	Lung Canc	Lung	Lung Cancer
ACH-0012	6.002252	TTC549	Rhabdoid	Soft Tissue	Rhabdoid
ACH-0015	4.422906	H413	Head and	Upper Aer	Head and Neck Cancer
ACH-0001	3.722466	OCIAML2	Leukemia	Blood	Leukemia
ACH-0003	3.593354	NCIH3122	Lung Canc	Lung	Lung Cancer
ACH-0005	3.320485	KMS21BM	Myeloma	Plasma Ce	Myeloma
ACH-0000	4.204767	OPM1	Myeloma	Plasma Ce	Myeloma
ACH-0002	4.004501	KP2	Pancreatic	Pancreas	Pancreatic Cancer
ACH-0003	3.460743	MOLM6	Leukemia	Blood	Leukemia
ACH-0009	4.141596	LS411N	Colon/Col	Colorectal	Colon/Colorectal Cancer
ACH-0004	5.396776	LU99	Lung Canc	Lung	Lung Cancer
ACH-0003	5.270903	QGP1	Pancreatic	Pancreas	Pancreatic Cancer
ACH-0013	4.786074	SW626	Colon/Col	Colorectal	Colon/Colorectal Cancer
ACH-0010	3.070389	KARPAS38	Lymphom	Lymphocy	Lymphoma
ACH-0001	5.233811	SLR20	Bladder Ca	Urinary Tr	Bladder Cancer
ACH-0019	4.598127	GB2	Gallbladde	Bile Duct	Gallbladder Cancer
ACH-0017	2.440952	95T1000	Liposarcor	Soft Tissue	Liposarcoma
ACH-0003	4.515384	PK1	Pancreatic	Pancreas	Pancreatic Cancer
ACH-0010	3.377124	CHLA218	Bone Canc	Bone	Bone Cancer
ACH-0005	4.540399	LN229	Brain Canc	Central Ne	Brain Cancer
ACH-0015	3.723559	KMLS1	Liposarcor	Soft Tissue	Liposarcoma
ACH-0002	5.788947	NCIH2029	Lung Canc	Lung	Lung Cancer
ACH-0007	3.748461	BICR56	Head and	Upper Aer	Head and Neck Cancer
ACH-0005	4.881175	SNU46	Head and	Upper Aer	Head and Neck Cancer
ACH-0003	4.982309	TE15	Esophagea	Esophagus	Esophageal Cancer
ACH-0003	4.299391	SKMM2	Myeloma	Plasma Ce	Myeloma
ACH-0003	4.903038	COLO678	Colon/Col	Colorectal	Colon/Colorectal Cancer
ACH-0000	3.789729	KARPAS29	Lymphom	Lymphocy	Lymphoma
ACH-0015	3.578939	KOSC2	Head and	Upper Aer	Head and Neck Cancer
ACH-0002	5.223809	SLR23	Kidney Ca	Kidney	Kidney Cancer
ACH-0006	3.649615	OVTOKO	Ovarian C	Ovary	Ovarian Cancer
ACH-0003	4.105175	JVM3	Leukemia	Blood	Leukemia
ACH-0009	4.498251	SNUC5	Colon/Col	Colorectal	Colon/Colorectal Cancer
ACH-0013	5.001802	C33A	Cervical C	Cervix	Cervical Cancer
ACH-0007	3.835924	BICR22	Head and	Upper Aer	Head and Neck Cancer
ACH-0000	3.674687	SKNMC	Bone Canc	Bone	Bone Cancer
ACH-0004	3.671293	LI7	Liver Canc	Liver	Liver Cancer
ACH-0008	3.552131	HS698T	Non-Canc	Fibroblast	Non-Cancerous
ACH-0002	4.034744	OV90	Ovarian C	Ovary	Ovarian Cancer

ACH-0014	3.978196	BPH1	Prostate C	Prostate	Prostate Cancer
ACH-0014	3.341986	C75	Colon/Col	Colorectal	Colon/Colorectal Cancer
ACH-0008	4.360364	NCIH2009	Lung Canc	Lung	Lung Cancer
ACH-0008	4.269781	NCIH1793	Lung Canc	Lung	Lung Cancer
ACH-0006	5.169925	SCLC21H	Lung Canc	Lung	Lung Cancer
ACH-0000	4.794936	PC14	Lung Canc	Lung	Lung Cancer
ACH-0012	3.731183	SUMB002	Brain Canc	Central Ne	Brain Cancer
ACH-0002	4.044394	SUDHL10	Lymphom	Lymphocy	Lymphoma
ACH-0005	4.646163	SNU1076	Head and	Upper Aer	Head and Neck Cancer
ACH-0005	4.352617	A498	Kidney Ca	Kidney	Kidney Cancer
ACH-0004	4.648465	SNU387	Liver Canc	Liver	Liver Cancer
ACH-0007	5.255123	NCIH727	Lung Canc	Lung	Lung Cancer
ACH-0000	3.942984	EHEB	Lymphom	Lymphocy	Lymphoma
ACH-0011	5.377471	KPMRTRY	Rhabdoid	Soft Tissue	Rhabdoid
ACH-0014	4.248687	A388	Skin Cance	Epidermoi	Skin Cancer
ACH-0005	5.874059	NCIH1836	Lung Canc	Lung	Lung Cancer
ACH-0005	4.765535	NCIH1299	Lung Canc	Lung	Lung Cancer
ACH-0020	4.718088	HSPSS	Sarcoma	Central Ne	Sarcoma
ACH-0006	5.912889	LN464	Brain Canc	Central Ne	Brain Cancer
ACH-0026	4.304511	JVE127	Colon/Col	Colorectal	Colon/Colorectal Cancer
ACH-0008	4.253233	DMS454	Lung Canc	Lung	Lung Cancer
ACH-0009	3.670161	DV90	Lung Canc	Lung	Lung Cancer
ACH-0024	5.353676	RPE1SS11	Non-Canc	Eye	Non-Cancerous
ACH-0002	3.791814	SLR24	Kidney Ca	Kidney	Kidney Cancer
ACH-0010	3.267536	CHLA99	Bone Canc	Bone	Bone Cancer
ACH-0005	3.218781	SNU1272	Kidney Ca	Kidney	Kidney Cancer
ACH-0010	6.01859	DL	Rhabdoid	Soft Tissue	Rhabdoid
ACH-0009	3.785551	SNUC4	Colon/Col	Colorectal	Colon/Colorectal Cancer
ACH-0001	4.634012	RD	Sarcoma	Soft Tissue	Sarcoma
ACH-0014	4.460087	SW756	Cervical C	Cervix	Cervical Cancer
ACH-0007	3.648465	MCAS	Ovarian C	Ovary	Ovarian Cancer
ACH-0001	5.944624	HCC2429	Lung Canc	Lung	Lung Cancer
ACH-0000	3.904002	HS683	Brain Canc	Central Ne	Brain Cancer
ACH-0006	5.32553	SKMEL31	Skin Cance	Skin	Skin Cancer
ACH-0016	4.581954	TGW	Neuroblas	Peripheral	Neuroblastoma
ACH-0016	2.02148	UPCISCC1	Head and	Upper Aer	Head and Neck Cancer
ACH-0020	4.514753	A375SKIN	Skin Cance	Skin	Skin Cancer
ACH-0020	5.229588	MP46	Eye Cance	Eye	Eye Cancer
ACH-0009	3.427606	SNUC2A	Colon/Col	Colorectal	Colon/Colorectal Cancer
ACH-0004	3.786596	YH13	Brain Canc	Central Ne	Brain Cancer
ACH-0013	3.736605	JOPACA1	Pancreatic	Pancreas	Pancreatic Cancer
ACH-0015	3.69933	MOLM14	Leukemia	Blood	Leukemia
ACH-0000	4.145677	ZR751	Breast Car	Breast	Breast Cancer
ACH-0002	3.740928	COV318	Ovarian C	Ovary	Ovarian Cancer
ACH-0009	4.851999	LNCAPCL(	Prostate C	Prostate	Prostate Cancer
ACH-0009	3.777157	HEC151	Endometri	Uterus	Endometrial/Uterine Cancer
ACH-0013	5.570463	NMB	Neuroblas	Peripheral	Neuroblastoma
ACH-0003	5.642413	CORL24	Lung Canc	Lung	Lung Cancer
ACH-0005	4.704872	UACC257	Skin Cance	Skin	Skin Cancer
ACH-0015	5.063071	JMURTK2	Rhabdoid	Kidney	Rhabdoid
ACH-0006	4.736605	CORL47	Lung Canc	Lung	Lung Cancer
ACH-0017	3.982765	94T778	Liposarcoi	Soft Tissue	Liposarcoma
ACH-0015	3.203201	H376	Head and	Upper Aer	Head and Neck Cancer
ACH-0005	5.433961	TCCPAN2	Pancreatic	Pancreas	Pancreatic Cancer
ACH-0001	5.033423	L363	Myeloma	Plasma Ce	Myeloma
ACH-0004	3.738768	TE5	Esophage	Esophagu	Esophageal Cancer
ACH-0014	4.209453	ESO26	Esophage	Esophagu	Esophageal Cancer
ACH-0007	4.334139	A2058	Skin Cance	Skin	Skin Cancer

ACH-0016	4.465974	ONDA7	Brain Canc	Central Ne	Brain Cancer
ACH-0004	5.419876	SNU216	Gastric Ca	Gastric	Gastric Cancer
ACH-0015	4.08151	MERO83	Lung Canc	Lung	Lung Cancer
ACH-0000	5.493135	HEL9217	Leukemia	Blood	Leukemia
ACH-0016	4.413459	UMRC3	Kidney Ca	Kidney	Kidney Cancer
ACH-0011	4.811471	OVCAR5	Ovarian C	Ovary	Ovarian Cancer
ACH-0007	3.907852	RMUGS	Ovarian C	Ovary	Ovarian Cancer
ACH-0005	5.096346	HEYA8	Ovarian C	Ovary	Ovarian Cancer
ACH-0001	6.31687	KG1C	Brain Canc	Central Ne	Brain Cancer
ACH-0016	3.31034	UHO1	Lymphom	Lymphocy	Lymphoma
ACH-0009	3.566815	SNU324	Pancreatic	Pancreas	Pancreatic Cancer
ACH-0000	3.806324	REC1	Lymphom	Lymphocy	Lymphoma
ACH-0000	4.071248	NCIH1581	Lung Canc	Lung	Lung Cancer
ACH-0011	2.797013	L82	Lymphom	Lymphocy	Lymphoma
ACH-0004	4.909773	F36P	Leukemia	Blood	Leukemia
ACH-0001	4.6094	SUPT11	Leukemia	Blood	Leukemia
ACH-0002	4.176323	BT549	Breast Car	Breast	Breast Cancer
ACH-0018	3.619413	COLO824	Breast Car	Breast	Breast Cancer
ACH-0007	5.609105	SEM	Leukemia	Blood	Leukemia
ACH-0016	3.228049	PEO4	Ovarian C	Ovary	Ovarian Cancer
ACH-0003	5.188638	JEKO1	Lymphom	Lymphocy	Lymphoma
ACH-0000	3.028569	TE125T	Non-Canc	Fibroblast	Non-Cancerous
ACH-0010	4.819668	CHLA266	Rhabdoid	Soft Tissue	Rhabdoid
ACH-0010	5.173927	COV504	Ovarian C	Ovary	Ovarian Cancer
ACH-0004	3.435629	MOLP2	Myeloma	Plasma Ce	Myeloma
ACH-0007	5.550285	RERFLCAD	Lung Canc	Lung	Lung Cancer
ACH-0005	3.952334	ST486	Lymphom	Lymphocy	Lymphoma
ACH-0013	4.709291	SUM159PT	Breast Car	Breast	Breast Cancer
ACH-0010	3.040892	NCIH292	Lung Canc	Lung	Lung Cancer
ACH-0001	4.550285	ALLSIL	Leukemia	Blood	Leukemia
ACH-0005	3.604071	AML193	Leukemia	Blood	Leukemia
ACH-0001	3.31904	TE159T	Non-Canc	Fibroblast	Non-Cancerous
ACH-0013	3.305971	MB1	Thyroid Ca	Thyroid	Thyroid Cancer
ACH-0018	5.31143	ICC4	Bile Duct (	Bile Duct	Bile Duct Cancer
ACH-0013	4.454834	H103	Head and	Upper Aer	Head and Neck Cancer
ACH-0008	3.850999	TOV21G	Ovarian C	Ovary	Ovarian Cancer
ACH-0009	3.811471	NCIH23	Lung Canc	Lung	Lung Cancer
ACH-0006	2.636915	NCIH1355	Lung Canc	Lung	Lung Cancer
ACH-0002	4.329124	JHH6	Liver Canc	Liver	Liver Cancer
ACH-0008	3.0268	HCC515	Lung Canc	Lung	Lung Cancer
ACH-0016	3.077243	RCK8	Lymphom	Lymphocy	Lymphoma
ACH-0012	2.214125	UPCISCC1	Head and	Upper Aer	Head and Neck Cancer
ACH-0001	4.100978	SCC9	Head and	Upper Aer	Head and Neck Cancer
ACH-0001	6.141188	VCAP	Prostate C	Prostate	Prostate Cancer
ACH-0001	4.960697	NALM19	Leukemia	Blood	Leukemia
ACH-0001	4.622345	NCIH2405	Lung Canc	Lung	Lung Cancer
ACH-0017	3.641546	PFSK1	Brain Canc	Central Ne	Brain Cancer
ACH-0009	5.856986	MDAMB36	Breast Car	Breast	Breast Cancer
ACH-0009	4.938286	HEC251	Endometri	Uterus	Endometrial/Uterine Cancer
ACH-0015	4.542258	JAR	Endometri	Uterus	Endometrial/Uterine Cancer
ACH-0017	4.679199	RC2	Sarcoma	Soft Tissue	Sarcoma
ACH-0003	4.862947	PSN1	Pancreatic	Pancreas	Pancreatic Cancer
ACH-0005	5.887525	RKN	Sarcoma	Soft Tissue	Sarcoma
ACH-0007	3.107688	NCIH2023	Lung Canc	Lung	Lung Cancer
ACH-0000	5.435962	SUPB15	Leukemia	Blood	Leukemia
ACH-0009	4.827311	CMLT1	Leukemia	Blood	Leukemia
ACH-0002	4.621759	NB4	Leukemia	Blood	Leukemia
ACH-0004	4.358256	SNU475	Liver Canc	Liver	Liver Cancer

ACH-0002	3.916477	NMCG1	Brain Canc	Central Ne	Brain Cancer
ACH-000(	4.545968	ACHN	Kidney Ca	Kidney	Kidney Cancer
ACH-0009	3.776104	COLO792	Skin Cance	Skin	Skin Cancer
ACH-0008	4.635174	NCIH1436	Lung Canc	Lung	Lung Cancer
ACH-0001	4.636915	SU8686	Pancreatic	Pancreas	Pancreatic Cancer
ACH-0004	3.991862	BL70	Lymphom	Lymphocy	Lymphoma
ACH-0015	6.079378	NCCIT	Embryona	Embryo	Embryonal Cancer
ACH-000(	4.380591	U343	Brain Canc	Central Ne	Brain Cancer
ACH-0009	3.447579	639V	Bladder Ca	Urinary Tr	Bladder Cancer
ACH-0011	5.989366	MON	Rhabdoid	Soft Tissue	Rhabdoid
ACH-0007	4.419539	LOXIMVI	Skin Cance	Skin	Skin Cancer
ACH-0002	3.930737	TOLEDO	Lymphom	Lymphocy	Lymphoma
ACH-0026	3.272023	KP363T	Colon/Col	Colorectal	Colon/Colorectal Cancer
ACH-0018	4.652486	SSP25	Bile Duct (	Bile Duct	Bile Duct Cancer
ACH-0019	3.958843	ECC2	Bile Duct (	Bile Duct	Bile Duct Cancer
ACH-000(	3.478972	A673	Bone Canc	Bone	Bone Cancer
