# Supplementary material for: The BRD4 Inhibitor dBET57 Exerts Anticancer Effects by Targeting Superenhancer-Related Genes in Neuroblastoma
Source: J Immunol Res. 2022 Nov 16;2022:7945884. doi: 10.1155/2022/7945884 (PMC9691391; doi:10.1155/2022/7945884)
Supplement: Supplementary 5 — Table S5: information for the CCLE of TBX3 in this study. [file 7945884.f5.pdf]

Table S5. Expression of TBX3 in different tumors

DepMap I	TBX3 log2	Cell Line N	Primary Di	Lineage	all Primary Disease
ACH-0011	3.541019	LC1SQSF	Lung Canc	Lung	Lung Cancer
ACH-0012	0.978196	COGAR35	Rhabdoid	Soft Tissue	Rhabdoid
ACH-0013	4.486714	COLO794	Skin Cance	Skin	Skin Cancer
ACH-0015	1.201634	KKU213	Bile Duct (	Bile Duct	Bile Duct Cancer
ACH-0002	6.973496	RT4	Bladder Ca	Urinary Tra	Bladder Cancer
ACH-0007	3.639232	SNU283	Colon/Col	Colorectal	Colon/Colorectal Cancer
ACH-0003	3.095924	NCIH1395	Lung Canc	Lung	Lung Cancer
ACH-0002	0.084064	DEL	Lymphom	Lymphocy	Lymphoma
ACH-0004	3.382667	SNU1196	Bile Duct (	Bile Duct	Bile Duct Cancer
ACH-0007	3.862947	LC1F	Lung Canc	Lung	Lung Cancer
ACH-0017	5.092123	93T449	Liposarcor	Soft Tissue	Liposarcoma
ACH-0020	1.636915	TGBC18TK	Colon/Col	Colorectal	Colon/Colorectal Cancer
ACH-0005	0.163499	ABC1	Lung Canc	Lung	Lung Cancer
ACH-0016	3.070389	SKN	Endometri	Uterus	Endometrial/Uterine Cancer
ACH-0001	0.124328	KE97	Myeloma	Plasma Ce	Myeloma
ACH-0007	4.690417	BFTC909	Kidney Ca	Kidney	Kidney Cancer
ACH-0010	0	KCIMOH1	Pancreatic	Pancreas	Pancreatic Cancer
ACH-0005	3.612352	YKG1	Brain Canc	Central Ne	Brain Cancer
ACH-0003	3.720278	MKN1	Gastric Ca	Gastric	Gastric Cancer
ACH-0007	5.66306	LK2	Lung Canc	Lung	Lung Cancer
ACH-0001	6.551055	HS888T	Non-Canc	Fibroblast	Non-Cancerous
ACH-0008	6.956405	ZR7530	Breast Car	Breast	Breast Cancer
ACH-0004	5.843733	SW837	Colon/Col	Colorectal	Colon/Colorectal Cancer
ACH-0007	5.300856	WM983B	Skin Cance	Skin	Skin Cancer
ACH-0013	2.606442	PACADD1	Pancreatic	Pancreas	Pancreatic Cancer
ACH-0000	4.221877	A101D	Skin Cance	Skin	Skin Cancer
ACH-0018	4.976364	OS252	Bone Canc	Bone	Bone Cancer
ACH-0008	0.070389	NCIH446	Lung Canc	Lung	Lung Cancer
ACH-0006	1.664483	PECAPJ34	Head and	Upper Aer	Head and Neck Cancer
ACH-0003	2.166715	TUHR14TK	Kidney Ca	Kidney	Kidney Cancer
ACH-0001	0.731183	COV434	Ovarian Ca	Ovary	Ovarian Cancer
ACH-0007	2.548437	YD10B	Head and	Upper Aer	Head and Neck Cancer
ACH-0020	4.878235	HSSCH2	Sarcoma	Central Ne	Sarcoma
ACH-0015	1.280956	KKU100	Bile Duct (	Bile Duct	Bile Duct Cancer
ACH-0003	3.746313	KMRC3	Kidney Ca	Kidney	Kidney Cancer
ACH-0020	2.24184	HOKUG	Cervical Ca	Cervix	Cervical Cancer
ACH-0007	0.214125	KASUMI2	Leukemia	Blood	Leukemia
ACH-0007	3.430285	NCIH2882	Lung Canc	Lung	Lung Cancer
ACH-0008	4.343408	KYSE140	Esophagea	Esophagus	Esophageal Cancer
ACH-0006	2.763412	MKN7	Gastric Ca	Gastric	Gastric Cancer
ACH-0000	0	MONOMA	Leukemia	Blood	Leukemia
ACH-0020	1.163499	SAS	Head and	Upper Aer	Head and Neck Cancer
ACH-0013	2.757023	PACADD1	Pancreatic	Pancreas	Pancreatic Cancer
ACH-0007	0.765535	L428	Lymphom	Lymphocy	Lymphoma
ACH-0004	2.304511	SKMEL1	Skin Cance	Skin	Skin Cancer
ACH-0001	1.735522	CFPAC1	Pancreatic	Pancreas	Pancreatic Cancer
ACH-0018	1.333424	YSCCC	Bile Duct (	Bile Duct	Bile Duct Cancer
ACH-0003	2.114367	OE33	Esophagea	Esophagus	Esophageal Cancer
ACH-0015	2.324811	LO68	Lung Canc	Lung	Lung Cancer
ACH-0009	0.15056	TE4	Esophagea	Esophagus	Esophageal Cancer
ACH-0024	2.063503	PSS008	Bone Canc	Bone	Bone Cancer
ACH-0009	3.166715	HEC6	Endometri	Uterus	Endometrial/Uterine Cancer
ACH-0014	3.22033	UMUC16	Bladder Ca	Urinary Tra	Bladder Cancer
ACH-0017	0.014355	WAOSEL	Leukemia	Blood	Leukemia
ACH-0001	3.114367	EFM192A	Breast Car	Breast	Breast Cancer
ACH-0009	4.77663	SNU1040	Colon/Col	Colorectal	Colon/Colorectal Cancer

ACH-0006	3.532317	FU97	Gastric Ca	Gastric	Gastric Cancer
ACH-0005	4.001802	59M	Ovarian C	Ovary	Ovarian Cancer
ACH-0001	3.032101	LOUNH91	Lung Canc	Lung	Lung Cancer
ACH-0000	2.358959	SKBR3	Breast Car	Breast	Breast Cancer
ACH-0000	0.014355	MHHCALL	Leukemia	Blood	Leukemia
ACH-0013	0	RPMI2650	Head and	Upper Aer	Head and Neck Cancer
ACH-0005	0.042644	NCIH1975	Lung Canc	Lung	Lung Cancer
ACH-0007	2.100978	NCIH2126	Lung Canc	Lung	Lung Cancer
ACH-0005	3.196922	UACC893	Breast Car	Breast	Breast Cancer
ACH-0013	0.765535	SW156	Kidney Ca	Kidney	Kidney Cancer
ACH-0019	0.847997	NZOV9	Ovarian C	Ovary	Ovarian Cancer
ACH-0013	0.782409	BICR78	Head and	Upper Aer	Head and Neck Cancer
ACH-0005	3.991862	EPLC272H	Lung Canc	Lung	Lung Cancer
ACH-0005	5.249825	IGR39	Skin Canc	Skin	Skin Cancer
ACH-0006	3.581351	LCLC103H	Lung Canc	Lung	Lung Cancer
ACH-0007	0.070389	OAW42	Ovarian C	Ovary	Ovarian Cancer
ACH-0002	4.102658	HCC38	Breast Car	Breast	Breast Cancer
ACH-0000	4.836429	U87MG	Brain Canc	Central Ne	Brain Cancer
ACH-0008	4.023255	BT483	Breast Car	Breast	Breast Cancer
ACH-0016	0	OCILY18	Lymphom	Lymphocy	Lymphoma
ACH-0006	3.749534	HEP3B217	Liver Canc	Liver	Liver Cancer
ACH-0009	0.042644	HEC1B	Endometri	Uterus	Endometrial/Uterine Cancer
ACH-0003	0.807355	CALU3	Lung Canc	Lung	Lung Cancer
ACH-0002	5.181898	AM38	Brain Canc	Central Ne	Brain Cancer
ACH-0016	1.063503	TASK1	Brain Canc	Peripheral	Brain Cancer
ACH-0008	2.179511	CAL51	Breast Car	Breast	Breast Cancer
ACH-0026	1.879706	WM3772F	Eye Cance	Eye	Eye Cancer
ACH-0011	0	MAC2A	Lymphom	Lymphocy	Lymphoma
ACH-0003	5.293885	SF295	Brain Canc	Central Ne	Brain Cancer
ACH-0006	1.427606	L33	Pancreatic	Pancreas	Pancreatic Cancer
ACH-0007	3.642702	NCIH1963	Lung Canc	Lung	Lung Cancer
ACH-0010	3.375735	CHLA9	Bone Canc	Bone	Bone Cancer
ACH-0012	2.341986	TC106	Bone Canc	Bone	Bone Cancer
ACH-0008	0.124328	AGS	Gastric Ca	Gastric	Gastric Cancer
ACH-0000	1.773996	NCIH1650	Lung Canc	Lung	Lung Cancer
ACH-0012	0.056584	STM9101	Rhabdoid	Soft Tissue	Rhabdoid
ACH-0001	4.568032	HS934T	Non-Canc	Fibroblast	Non-Cancerous
ACH-0003	4.392317	HT144	Skin Canc	Skin	Skin Cancer
ACH-0005	3.452859	HT29	Colon/Col	Colorectal	Colon/Colorectal Cancer
ACH-0025	5.224195	WM3211	Skin Canc	Skin	Skin Cancer
ACH-0002	1.077243	ASPC1	Pancreatic	Pancreas	Pancreatic Cancer
ACH-0002	6.693208	SKNAS	Neuroblas	Peripheral	Neuroblastoma
ACH-0016	2.883621	SKGII	Endometri	Uterus	Endometrial/Uterine Cancer
ACH-0006	4.715344	COV644	Ovarian C	Ovary	Ovarian Cancer
ACH-0005	3.647315	LS123	Colon/Col	Colorectal	Colon/Colorectal Cancer
ACH-0006	0.042644	HUT102	Lymphom	Lymphocy	Lymphoma
ACH-0011	0	SCMCRM2	Sarcoma	Soft Tissue	Sarcoma
ACH-0016	1.124328	UMRC7	Kidney Ca	Kidney	Kidney Cancer
ACH-0018	0.356144	ICC12	Bile Duct (	Bile Duct	Bile Duct Cancer
ACH-0006	0.042644	NCIH441	Lung Canc	Lung	Lung Cancer
ACH-0018	1.695994	ICC6	Bile Duct (	Bile Duct	Bile Duct Cancer
ACH-0007	4.319762	CL40	Colon/Col	Colorectal	Colon/Colorectal Cancer
ACH-0013	6.054848	TT	Thyroid Ca	Thyroid	Thyroid Cancer
ACH-0008	0.799087	NCIH1869	Lung Canc	Lung	Lung Cancer
ACH-0015	2.799087	MERO82	Lung Canc	Lung	Lung Cancer
ACH-0003	2.849999	SNU668	Gastric Ca	Gastric	Gastric Cancer
ACH-0002	1.941106	HCC1937	Breast Car	Breast	Breast Cancer
ACH-0000	0.163499	PANC0213	Pancreatic	Pancreas	Pancreatic Cancer

ACH-000(	0.014355	PLB985	Leukemia	Blood	Leukemia
ACH-001ε	1.899176	TGBC1TKE	Gallbladde	Bile Duct	Gallbladder Cancer
ACH-0007	5.174726	TE441T	Sarcoma	Soft Tissue	Sarcoma
ACH-000§	1.85599	HEC265	Endometri	Uterus	Endometrial/Uterine Cancer
ACH-000ξ	0.970854	LCLC97TM	Lung Canc	Lung	Lung Cancer
ACH-000ϵ	5.130519	HS944T	Skin Canc	Skin	Skin Cancer
ACH-000§	4.954196	EN	Endometri	Uterus	Endometrial/Uterine Cancer
ACH-000З	0.238787	YAPC	Pancreatic	Pancreas	Pancreatic Cancer
ACH-001ε	1.182692	ICC15	Bile Duct (	Bile Duct	Bile Duct Cancer
ACH-0001	8.064096	NCIH1618	Lung Canc	Lung	Lung Cancer
ACH-000ε	4.547203	SF172	Brain Canc	Central Ne	Brain Cancer
ACH-000З	3.738768	SNU1105	Brain Canc	Central Ne	Brain Cancer
ACH-000Ź	3.28244	HS600T	Non-Canc	Fibroblast	Non-Cancerous
ACH-000ξ	0.344828	HCC1171	Lung Canc	Lung	Lung Cancer
ACH-001ϵ	2.350497	NP8	Brain Canc	Central Ne	Brain Cancer
ACH-0001	3.570463	HS940T	Non-Canc	Fibroblast	Non-Cancerous
ACH-000⁄	0.014355	U937	Leukemia	Blood	Leukemia
ACH-001ξ	1.427606	KMS28PE	Myeloma	Plasma Ce	Myeloma
ACH-000§	3.922198	CW2	Colon/Col	Colorectal	Colon/Colorectal Cancer
ACH-000⁄	4.046142	SH4	Skin Canc	Skin	Skin Cancer
ACH-000§	0.084064	REH	Leukemia	Blood	Leukemia
ACH-000(	4.093391	GAMG	Brain Canc	Central Ne	Brain Cancer
ACH-001ϵ	3.238787	SKGI	Endometri	Uterus	Endometrial/Uterine Cancer
ACH-001⁄	4.513491	C125PM	Colon/Col	Colorectal	Colon/Colorectal Cancer
ACH-001З	2.094236	CASKI	Cervical C	Cervix	Cervical Cancer
ACH-000Ź	5.285032	HS840T	Non-Canc	Fibroblast	Non-Cancerous
ACH-000§	2.275007	HCT116	Colon/Col	Colorectal	Colon/Colorectal Cancer
ACH-001⁄	0.042644	C10	Colon/Col	Colorectal	Colon/Colorectal Cancer
ACH-000§	3.344828	HT55	Colon/Col	Colorectal	Colon/Colorectal Cancer
ACH-000(	3.090853	NCIH1819	Lung Canc	Lung	Lung Cancer
ACH-000ξ	0.028569	KMS27	Myeloma	Plasma Ce	Myeloma
ACH-000(	1.718088	MCF7	Breast Car	Breast	Breast Cancer
ACH-001ξ	0	MUTZ8	Leukemia	Blood	Leukemia
ACH-000З	5.644433	EFM19	Breast Car	Breast	Breast Cancer
ACH-000ε	4.572283	JHH7	Liver Canc	Liver	Liver Cancer
ACH-000⁄	1.117695	NCIH2170	Lung Canc	Lung	Lung Cancer
ACH-001ξ	0	HEC1	Endometri	Uterus	Endometrial/Uterine Cancer
ACH-000З	3.185867	SR786	Lymphom	Lymphocy	Lymphoma
ACH-001§	0.042644	CCLP1	Bile Duct (	Bile Duct	Bile Duct Cancer
ACH-001⁄	4.301588	UMUC6	Bladder C	Urinary Tr	Bladder Cancer
ACH-000ε	4.814038	NCIH510	Lung Canc	Lung	Lung Cancer
ACH-001⁄	4.056584	TO14	Ovarian C	Ovary	Ovarian Cancer
ACH-000ε	3.835924	COLO679	Skin Canc	Skin	Skin Cancer
ACH-0011	4.521679	NHAHTDD	Non-Canc	Central Ne	Non-Cancerous
ACH-001ξ	1.636915	MEL202	Eye Cance	Eye	Eye Cancer
ACH-000ε	4.060047	HGC27	Gastric Ca	Gastric	Gastric Cancer
ACH-001Ź	5.731726	TIG3TD	Non-Canc	Fibroblast	Non-Cancerous
ACH-000ε	0.367371	CAL27	Head and	Upper Aer	Head and Neck Cancer
ACH-000⁄	4.163499	UO31	Kidney Ca	Kidney	Kidney Cancer
ACH-001З	0.014355	PACADD1	Pancreatic	Pancreas	Pancreatic Cancer
ACH-001З	6.192983	COGN305	Neuroblas	Peripheral	Neuroblastoma
ACH-000ϵ	3.030336	HCC2814	Lung Canc	Lung	Lung Cancer
ACH-0011	0	MYLA	Lymphom	Lymphocy	Lymphoma
ACH-000§	0.028569	HT	Lymphom	Lymphocy	Lymphoma
ACH-000ε	1.117695	KNS62	Lung Canc	Lung	Lung Cancer
ACH-001⁄	0.286881	WERIRB1	Eye Cance	Eye	Eye Cancer
ACH-000ξ	1.604071	OE21	Esophage	Esophagu	Esophageal Cancer
ACH-0007	3.014355	HCC202	Breast Car	Breast	Breast Cancer

ACH-0005	0.286881	SW1710	Bladder Ca	Urinary Tra	Bladder Cancer
ACH-0007	2.969012	LNZ308	Brain Canc	Central Ne	Brain Cancer
ACH-0017	0.163499	RHJT	Sarcoma	Soft Tissue	Sarcoma
ACH-0007	4.373648	LXF289	Lung Canc	Lung	Lung Cancer
ACH-0007	1.560715	DMS273	Lung Canc	Lung	Lung Cancer
ACH-0013	0.903038	A431	Skin Cance	Skin	Skin Cancer
ACH-0010	0	FEPD	Lymphom	Lymphocy	Lymphoma
ACH-0003	3.344828	NCIH69	Lung Canc	Lung	Lung Cancer
ACH-0016	0.238787	SKGT4	Esophagea	Esophagus	Esophageal Cancer
ACH-0015	2.207893	HKA1	Skin Cance	Skin	Skin Cancer
ACH-0002	2.321928	SNU1079	Bile Duct (	Bile Duct	Bile Duct Cancer
ACH-0015	0	HG3	Leukemia	Blood	Leukemia
ACH-0002	1.765535	HUPT4	Pancreatic	Pancreas	Pancreatic Cancer
ACH-0016	4.218006	NP5	Brain Canc	Central Ne	Brain Cancer
ACH-0014	6.618973	UMUC14	Bladder Ca	Urinary Tra	Bladder Cancer
ACH-0009	8.051807	SNU81	Colon/Col	Colorectal	Colon/Colorectal Cancer
ACH-0014	4.169925	UMUC10	Bladder Ca	Urinary Tra	Bladder Cancer
ACH-0003	3.600508	ISTMES2	Lung Canc	Lung	Lung Cancer
ACH-0014	0.014355	UMUC13	Bladder Ca	Urinary Tra	Bladder Cancer
ACH-0005	2.790772	C32	Skin Cance	Skin	Skin Cancer
ACH-0005	5.839708	DMS153	Lung Canc	Lung	Lung Cancer
ACH-0006	2.24184	KMRC1	Kidney Ca	Kidney	Kidney Cancer
ACH-0007	4.454834	DM3	Non-Canc	Fibroblast	Non-Cancerous
ACH-0008	0.084064	HCC1195	Lung Canc	Lung	Lung Cancer
ACH-0007	1.189034	PECAPJ41	Head and	Upper Aer	Head and Neck Cancer
ACH-0004	0.411426	A704	Kidney Ca	Kidney	Kidney Cancer
ACH-0003	2.469886	IMR32	Neuroblas	Peripheral	Neuroblastoma
ACH-0024	2.495695	HAP1	Leukemia	Blood	Leukemia
ACH-0006	0.286881	HDQP1	Breast Car	Breast	Breast Cancer
ACH-0002	1.316146	CAKI2	Kidney Ca	Kidney	Kidney Cancer
ACH-0014	0	FARAGE	Lymphom	Lymphocy	Lymphoma
ACH-0008	0.028569	647V	Bladder Ca	Urinary Tra	Bladder Cancer
ACH-0002	7.673203	NH6	Neuroblas	Peripheral	Neuroblastoma
ACH-0016	1.831877	NOZ	Gallbladde	Bile Duct	Gallbladder Cancer
ACH-0014	1.815575	921	Eye Cance	Eye	Eye Cancer
ACH-0001	2.776104	SIGM5	Leukemia	Blood	Leukemia
ACH-0001	2.438293	NCIH2444	Lung Canc	Lung	Lung Cancer
ACH-0006	1.831877	SW1573	Lung Canc	Lung	Lung Cancer
ACH-0000	3.828835	D283MED	Brain Canc	Central Ne	Brain Cancer
ACH-0004	5.90665	GSU	Gastric Ca	Gastric	Gastric Cancer
ACH-0006	0.042644	IALM	Lung Canc	Lung	Lung Cancer
ACH-0002	0.014355	NUDUL1	Lymphom	Lymphocy	Lymphoma
ACH-0007	2.192194	KYSE70	Esophagea	Esophagus	Esophageal Cancer
ACH-0005	3.327687	DMS114	Lung Canc	Lung	Lung Cancer
ACH-0020	2.643856	ECC4	Colon/Col	Colorectal	Colon/Colorectal Cancer
ACH-0003	0.275007	KMS12BM	Myeloma	Plasma Ce	Myeloma
ACH-0017	0	CTV1DM	Leukemia	Blood	Leukemia
ACH-0014	0.378512	SW954	Cervical C	Cervix	Cervical Cancer
ACH-0010	1.895303	D458	Brain Canc	Central Ne	Brain Cancer
ACH-0000	3.377124	C2BBE1	Colon/Col	Colorectal	Colon/Colorectal Cancer
ACH-0004	3.099295	NCIH716	Colon/Col	Colorectal	Colon/Colorectal Cancer
ACH-0017	0	VAL	Lymphom	Lymphocy	Lymphoma
ACH-0000	0.15056	HL60	Leukemia	Blood	Leukemia
ACH-0015	1.182692	MM386	Skin Cance	Skin	Skin Cancer
ACH-0009	1.752749	JHUEM7	Endometri	Uterus	Endometrial/Uterine Cancer
ACH-0026	0.765535	170MGBA	Brain Canc	Central Ne	Brain Cancer
ACH-0008	0.189034	EB1	Lymphom	Lymphocy	Lymphoma
ACH-0007	1.794936	JIMT1	Breast Car	Breast	Breast Cancer

ACH-0015	1.691534	HCS2	Cervical C	Cervix	Cervical Cancer
ACH-0028	5.096768	YUHOIN0(	Unknown	Unknown	Unknown
ACH-0007	2.035624	L1236	Lymphom	Lymphocy	Lymphoma
ACH-001(	0.014355	BT16	Rhabdoid	Soft Tissue	Rhabdoid
ACH-0006	1.981853	SLR26	Kidney Ca	Kidney	Kidney Cancer
ACH-0003	0.042644	CAPAN1	Pancreatic	Pancreas	Pancreatic Cancer
ACH-000(	1.709291	HCC827	Lung Canc	Lung	Lung Cancer
ACH-0006	3.844988	RH18	Sarcoma	Soft Tissue	Sarcoma
ACH-0004	0.516015	TUHR10TK	Kidney Ca	Kidney	Kidney Cancer
ACH-0014	4.336283	UMUC11	Bladder Ca	Urinary Tr	Bladder Cancer
ACH-0006	3.638074	SNU878	Liver Canc	Liver	Liver Cancer
ACH-0003	4.456149	CL14	Colon/Col	Colorectal	Colon/Colorectal Cancer
ACH-0002	5.334854	U178	Brain Canc	Central Ne	Brain Cancer
ACH-0003	2.914565	NCIH508	Colon/Col	Colorectal	Colon/Colorectal Cancer
ACH-0002	6.818007	CL11	Colon/Col	Colorectal	Colon/Colorectal Cancer
ACH-0013	1.372952	OACM51	Esophagea	Esophagus	Esophageal Cancer
ACH-0002	0.097611	KALS1	Brain Canc	Central Ne	Brain Cancer
ACH-0009	0.238787	NCIH650	Lung Canc	Lung	Lung Cancer
ACH-0006	1.545968	CORL23	Lung Canc	Lung	Lung Cancer
ACH-0024	4.797532	HT144SKII	Skin Cance	Skin	Skin Cancer
ACH-0013	0	PACADD1	Pancreatic	Pancreas	Pancreatic Cancer
ACH-001(	0.344828	CHLA10	Bone Canc	Bone	Bone Cancer
ACH-0002	2.592158	AU565	Breast Car	Breast	Breast Cancer
ACH-0009	2.364572	RKO	Colon/Col	Colorectal	Colon/Colorectal Cancer
ACH-0007	0.014355	OCIM1	Leukemia	Blood	Leukemia
ACH-0016	0.739848	UPCISCC1	Head and	Upper Aer	Head and Neck Cancer
ACH-001(	1.682573	BECKER	Brain Canc	Central Ne	Brain Cancer
ACH-0002	3.145677	NCIH1755	Lung Canc	Lung	Lung Cancer
ACH-0002	0.163499	RERFLCAI	Lung Canc	Lung	Lung Cancer
ACH-0001	1.85599	PANC1	Pancreatic	Pancreas	Pancreatic Cancer
ACH-0017	3.678072	LPS27	Liposarcor	Soft Tissue	Liposarcoma
ACH-0002	3.675816	UOK101	Kidney Ca	Kidney	Kidney Cancer
ACH-0001	0.070389	OCILY3	Lymphom	Lymphocy	Lymphoma
ACH-0007	0.137504	KIJK	Lymphom	Lymphocy	Lymphoma
ACH-0007	3.372952	SNUC1	Colon/Col	Colorectal	Colon/Colorectal Cancer
ACH-0008	2.952334	CAL851	Breast Car	Breast	Breast Cancer
ACH-0011	0.028569	MONOMA	Leukemia	Blood	Leukemia
ACH-0016	0.124328	OCUG1	Gallbladde	Bile Duct	Gallbladder Cancer
ACH-0001	4.901591	HS343T	Non-Canc	Fibroblast	Non-Cancerous
ACH-0003	5.504938	CCFSTTG1	Brain Canc	Central Ne	Brain Cancer
ACH-0015	0.028569	MM415	Skin Cance	Skin	Skin Cancer
ACH-0008	2.513491	HS939T	Skin Cance	Skin	Skin Cancer
ACH-0017	1.757023	UPCISCC2	Head and	Upper Aer	Head and Neck Cancer
ACH-0001	0.15056	TALL1	Leukemia	Blood	Leukemia
ACH-0005	0.584963	VMCUB1	Bladder Ca	Urinary Tr	Bladder Cancer
ACH-0009	7.115824	TGBC11TK	Gastric Ca	Gastric	Gastric Cancer
ACH-0001	0.084064	OCILY19	Lymphom	Lymphocy	Lymphoma
ACH-0005	7.086826	ECC10	Gastric Ca	Gastric	Gastric Cancer
ACH-0008	3.896272	FTC238	Thyroid Ca	Thyroid	Thyroid Cancer
ACH-0016	0.807355	OSC20	Head and	Upper Aer	Head and Neck Cancer
ACH-0006	2.438293	PECAPJ15	Head and	Upper Aer	Head and Neck Cancer
ACH-0019	1.350497	MM485	Skin Cance	Skin	Skin Cancer
ACH-0013	2.641546	COV413A	Ovarian C	Ovary	Ovarian Cancer
ACH-0005	2.881665	HSC4	Head and	Upper Aer	Head and Neck Cancer
ACH-0017	0	WSUNHL	Lymphom	Lymphocy	Lymphoma
ACH-002(	0.014355	OMM25	Eye Cance	Eye	Eye Cancer
ACH-0001	4.678072	NCIH2052	Lung Canc	Lung	Lung Cancer
ACH-0005	1.176323	HS821T	Non-Canc	Fibroblast	Non-Cancerous

ACH-0001	0.042644	OAW28	Ovarian C	Ovary	Ovarian Cancer
ACH-0008	6.157044	KMH2	Lymphom	Lymphocy	Lymphoma
ACH-0012	3.412782	127399	Sarcoma	Soft Tissue	Sarcoma
ACH-0003	0.056584	JHOC5	Ovarian C	Ovary	Ovarian Cancer
ACH-0015	2.304511	MERO84	Lung Canc	Lung	Lung Cancer
ACH-0008	3.337711	SW480	Colon/Col	Colorectal	Colon/Colorectal Cancer
ACH-0008	4.669594	SNU719	Gastric Ca	Gastric	Gastric Cancer
ACH-0005	0.014355	SNU410	Pancreatic	Pancreas	Pancreatic Cancer
ACH-0014	6.272023	UMUC7	Bladder Ca	Urinary Tr	Bladder Cancer
ACH-0006	3.072106	A549	Lung Canc	Lung	Lung Cancer
ACH-0013	5.094236	HA1E	Non-Canc	Kidney	Non-Cancerous
ACH-0000	0.014355	697	Leukemia	Blood	Leukemia
ACH-0004	1.432959	VMRCRCW	Kidney Ca	Kidney	Kidney Cancer
ACH-0001	0.978196	KP3	Pancreatic	Pancreas	Pancreatic Cancer
ACH-0008	3.80426	HUH28	Bile Duct (	Bile Duct	Bile Duct Cancer
ACH-0006	0.042644	SUDHL8	Lymphom	Lymphocy	Lymphoma
ACH-0001	0.042644	KASUMI6	Leukemia	Blood	Leukemia
ACH-0009	4.540399	HCT15	Colon/Col	Colorectal	Colon/Colorectal Cancer
ACH-0008	1.85997	NCIH1048	Lung Canc	Lung	Lung Cancer
ACH-0008	5.264161	LN18	Brain Canc	Central Ne	Brain Cancer
ACH-0002	2.735522	SUPM2	Lymphom	Lymphocy	Lymphoma
ACH-0000	0.333424	SKES1	Bone Canc	Bone	Bone Cancer
ACH-0003	3.813525	HS870T	Non-Canc	Fibroblast	Non-Cancerous
ACH-0020	5.151372	SKMEL19	Skin Canc	Skin	Skin Cancer
ACH-0007	2.920293	MKN74	Gastric Ca	Gastric	Gastric Cancer
ACH-0001	4.876271	HCC1599	Breast Car	Breast	Breast Cancer
ACH-0002	5.803227	DU4475	Breast Car	Breast	Breast Cancer
ACH-0001	5.128046	SW579	Thyroid Ca	Thyroid	Thyroid Cancer
ACH-0000	0.028569	NCO2	Leukemia	Blood	Leukemia
ACH-0005	0.097611	HUT78	Lymphom	Lymphocy	Lymphoma
ACH-0011	1.400538	SKPNDW	Bone Canc	Bone	Bone Cancer
ACH-0005	6.187847	T98G	Brain Canc	Central Ne	Brain Cancer
ACH-0003	4.063503	MHHES1	Bone Canc	Bone	Bone Cancer
ACH-0002	3.467279	KLE	Endometri	Uterus	Endometrial/Uterine Cancer
ACH-0005	4.3067	SNU61	Colon/Col	Colorectal	Colon/Colorectal Cancer
ACH-0005	0.31034	JHOS4	Ovarian C	Ovary	Ovarian Cancer
ACH-0016	0.545968	UPCISCC0	Head and	Upper Aer	Head and Neck Cancer
ACH-0005	6.355263	NCIH1876	Lung Canc	Lung	Lung Cancer
ACH-0009	0	NAMALW.	Lymphom	Lymphocy	Lymphoma
ACH-0005	3.148934	TDOTT	Esophage	Esophagu	Esophageal Cancer
ACH-0016	1.695994	SHMAC4	Prostate C	Prostate	Prostate Cancer
ACH-0003	0.201634	NCIH2081	Lung Canc	Lung	Lung Cancer
ACH-0009	0.31034	HEC1A	Endometri	Uterus	Endometrial/Uterine Cancer
ACH-0002	2.127633	LP1	Myeloma	Plasma Ce	Myeloma
ACH-0001	0.014355	BT12	Rhabdoid	Soft Tissue	Rhabdoid
ACH-0009	2.788686	SNU175	Colon/Col	Colorectal	Colon/Colorectal Cancer
ACH-0013	1.899176	H157	Head and	Upper Aer	Head and Neck Cancer
ACH-0006	5.565902	HCC2157	Breast Car	Breast	Breast Cancer
ACH-0002	4.739308	SNU245	Bile Duct (	Bile Duct	Bile Duct Cancer
ACH-0001	2.555816	HS863T	Non-Canc	Fibroblast	Non-Cancerous
ACH-0003	0.014355	NCIH1781	Lung Canc	Lung	Lung Cancer
ACH-0013	1.887525	DOTC2451	Cervical C	Cervix	Cervical Cancer
ACH-0007	1.70044	KMS11	Myeloma	Plasma Ce	Myeloma
ACH-0009	0.097611	PF382	Leukemia	Blood	Leukemia
ACH-0007	0.163499	MM1S	Myeloma	Plasma Ce	Myeloma
ACH-0028	4.260026	PSS131R	Bone Canc	Bone	Bone Cancer
ACH-0016	2.157044	TFK1	Bile Duct (	Bile Duct	Bile Duct Cancer
ACH-0003	1.144046	JURLMK1	Leukemia	Blood	Leukemia

ACH-0003	2.160275	SLR25	Kidney Ca	Kidney	Kidney Cancer
ACH-0009	2.698218	HCC1359	Lung Canc	Lung	Lung Cancer
ACH-0008	0.176323	NCIH524	Lung Canc	Lung	Lung Cancer
ACH-0006	0.070389	CMK	Leukemia	Blood	Leukemia
ACH-0005	0.056584	CALU1	Lung Canc	Lung	Lung Cancer
ACH-0005	0.014355	MC116	Lymphom	Lymphocy	Lymphoma
ACH-0008	0.163499	YD15	Head and	Upper Aer	Head and Neck Cancer
ACH-0009	0.028569	HCC2450	Lung Canc	Lung	Lung Cancer
ACH-0001	0.014355	NCIH3255	Lung Canc	Lung	Lung Cancer
ACH-0004	4.205549	HCC56	Colon/Col	Colorectal	Colon/Colorectal Cancer
ACH-0007	2.49057	HCC1833	Lung Canc	Lung	Lung Cancer
ACH-0006	6.871474	HUH6	Liver Canc	Liver	Liver Cancer
ACH-0005	8.146696	NCIH1184	Lung Canc	Lung	Lung Cancer
ACH-0008	0.15056	COLO684	Endometri	Uterus	Endometrial/Uterine Cancer
ACH-0007	1.847997	TE14	Esophagea	Esophagus	Esophageal Cancer
ACH-0001	6.49857	CHP126	Neuroblas	Peripheral	Neuroblastoma
ACH-0020	3.017922	NOS1	Bone Canc	Bone	Bone Cancer
ACH-0004	0.084064	CA46	Lymphom	Lymphocy	Lymphoma
ACH-0014	4.093391	C99	Colon/Col	Colorectal	Colon/Colorectal Cancer
ACH-0008	2.341986	SW1271	Lung Canc	Lung	Lung Cancer
ACH-0016	1.613532	UPCISCC0	Head and	Upper Aer	Head and Neck Cancer
ACH-0000	0.722466	GOS3	Brain Canc	Central Ne	Brain Cancer
ACH-0005	0.070389	HCC78	Lung Canc	Lung	Lung Cancer
ACH-0007	5.088311	SJSA1	Bone Canc	Bone	Bone Cancer
ACH-0016	0	TANOUE	Leukemia	Blood	Leukemia
ACH-0008	4.305971	UMUC1	Bladder Ca	Urinary Tra	Bladder Cancer
ACH-0005	4.200065	RCM1	Colon/Col	Colorectal	Colon/Colorectal Cancer
ACH-0004	2.675816	MESSA	Sarcoma	Soft Tissue	Sarcoma
ACH-0003	6.929909	NCIH2196	Lung Canc	Lung	Lung Cancer
ACH-0020	4.273516	TN2	Neuroblas	Peripheral	Neuroblastoma
ACH-0007	0.014355	P31FUJ	Leukemia	Blood	Leukemia
ACH-0007	2.280956	NCIH854	Lung Canc	Lung	Lung Cancer
ACH-0005	6.048323	KE39	Gastric Ca	Gastric	Gastric Cancer
ACH-0000	3.243364	HCC4006	Lung Canc	Lung	Lung Cancer
ACH-0000	2.950468	PATU8988	Pancreatic	Pancreas	Pancreatic Cancer
ACH-0016	0.731183	SUSA	Teratoma	Embryo	Teratoma
ACH-0003	0.084064	DB	Lymphom	Lymphocy	Lymphoma
ACH-0003	4.163499	HCC1143	Breast Car	Breast	Breast Cancer
ACH-0005	0.124328	PEER	Leukemia	Blood	Leukemia
ACH-0009	2.235727	SNU520	Gastric Ca	Gastric	Gastric Cancer
ACH-0020	3.547203	A375SKIN	Skin Cance	Skin	Skin Cancer
ACH-0005	1.673556	FUOV1	Ovarian Ca	Ovary	Ovarian Cancer
ACH-0001	4.742545	HS578T	Breast Car	Breast	Breast Cancer
ACH-0024	5.953032	RVH421SK	Skin Cance	Skin	Skin Cancer
ACH-0006	0.111031	OVMANA	Ovarian Ca	Ovary	Ovarian Cancer
ACH-0003	1.565597	HCC2108	Lung Canc	Lung	Lung Cancer
ACH-0000	4.589763	U118MG	Brain Canc	Central Ne	Brain Cancer
ACH-0010	0.056584	DERL2	Lymphom	Lymphocy	Lymphoma
ACH-0004	0.176323	TYKNU	Ovarian Ca	Ovary	Ovarian Cancer
ACH-0010	2.920293	CHL1DM	Skin Cance	Skin	Skin Cancer
ACH-0019	1.480265	NO36	Lung Canc	Lung	Lung Cancer
ACH-0003	0.056584	SNU626	Brain Canc	Central Ne	Brain Cancer
ACH-0016	1.244887	PEO1	Ovarian Ca	Ovary	Ovarian Cancer
ACH-0004	5.102238	SW1088	Brain Canc	Central Ne	Brain Cancer
ACH-0004	0.056584	KO52	Leukemia	Blood	Leukemia
ACH-0013	2.744161	SUM185PI	Breast Car	Breast	Breast Cancer
ACH-0005	3.218781	BICR16	Head and	Upper Aer	Head and Neck Cancer
ACH-0003	2.392317	LN215	Brain Canc	Central Ne	Brain Cancer

ACH-0004	5.066089	NCIH747	Colon/Col	Colorectal	Colon/Colorectal Cancer
ACH-0004	5.703211	SKCO1	Colon/Col	Colorectal	Colon/Colorectal Cancer
ACH-0002	4.473137	HS819T	Non-Canc	Fibroblast	Non-Cancerous
ACH-0003	3.374344	TE10	Esophagea	Esophagus	Esophageal Cancer
ACH-0014	2.232661	TC138	Bone Canc	Bone	Bone Cancer
ACH-0009	1.627607	JHUEM1	Endometri	Uterus	Endometrial/Uterine Cancer
ACH-0008	0.084064	NCIH1651	Lung Canc	Lung	Lung Cancer
ACH-0008	0.176323	KMM1	Myeloma	Plasma Ce	Myeloma
ACH-0007	4.712045	HS695T	Skin Cance	Skin	Skin Cancer
ACH-0016	4.286881	ONDA8	Brain Canc	Central Ne	Brain Cancer
ACH-0007	4.731726	SNU601	Gastric Ca	Gastric	Gastric Cancer
ACH-0008	6.486714	MDAMB41	Breast Car	Breast	Breast Cancer
ACH-0012	3.582556	UW228	Brain Canc	Central Ne	Brain Cancer
ACH-0016	0	OCIM2	Leukemia	Blood	Leukemia
ACH-0002	0.014355	PANC0403	Pancreatic	Pancreas	Pancreatic Cancer
ACH-0003	4.294988	T84	Colon/Col	Colorectal	Colon/Colorectal Cancer
ACH-0019	0	NALM16	Leukemia	Blood	Leukemia
ACH-0013	6.238405	COGN278	Neuroblas	Peripheral	Neuroblastoma
ACH-0008	0.014355	OCIMY5	Myeloma	Plasma Ce	Myeloma
ACH-0001	4.554589	CORL311	Lung Canc	Lung	Lung Cancer
ACH-0005	4.505256	SNU489	Brain Canc	Central Ne	Brain Cancer
ACH-0005	3.155425	ISTMES1	Lung Canc	Lung	Lung Cancer
ACH-0006	0.275007	HCC70	Breast Car	Breast	Breast Cancer
ACH-0001	1.695994	HS274T	Non-Canc	Fibroblast	Non-Cancerous
ACH-0009	3.713696	NCIH2172	Lung Canc	Lung	Lung Cancer
ACH-0014	0	BLUE1	Lymphom	Lymphocy	Lymphoma
ACH-0002	6.564988	OUMS23	Colon/Col	Colorectal	Colon/Colorectal Cancer
ACH-0007	1.124328	CAOV3	Ovarian C	Ovary	Ovarian Cancer
ACH-0013	2.918386	MS751	Cervical C	Cervix	Cervical Cancer
ACH-0002	3.952334	SF539	Brain Canc	Central Ne	Brain Cancer
ACH-0017	4.05398	CAL72	Bone Canc	Bone	Bone Cancer
ACH-0016	0	OCILY7	Lymphom	Lymphocy	Lymphoma
ACH-0006	8.199329	NCIH211	Lung Canc	Lung	Lung Cancer
ACH-0005	1.521051	NCIH2073	Lung Canc	Lung	Lung Cancer
ACH-0005	0.084064	SQ1	Lung Canc	Lung	Lung Cancer
ACH-0000	0.042644	MUTZ3	Leukemia	Blood	Leukemia
ACH-0000	1.275007	HPAFII	Pancreatic	Pancreas	Pancreatic Cancer
ACH-0003	2.389567	SKHEP1	Liver Canc	Liver	Liver Cancer
ACH-0007	0.042644	RMGI	Ovarian C	Ovary	Ovarian Cancer
ACH-0013	4.736064	SUM52PE	Breast Car	Breast	Breast Cancer
ACH-0018	2.017922	ICC8	Bile Duct (	Bile Duct	Bile Duct Cancer
ACH-0003	0.042644	HCC461	Lung Canc	Lung	Lung Cancer
ACH-0020	2.327687	RMSYM	Sarcoma	Soft Tissue	Sarcoma
ACH-0002	2.715893	PL21	Leukemia	Blood	Leukemia
ACH-0007	4.101818	NCIH810	Lung Canc	Lung	Lung Cancer
ACH-0004	4.581351	HS739T	Non-Canc	Fibroblast	Non-Cancerous
ACH-0001	2.608809	TO175T	Non-Canc	Fibroblast	Non-Cancerous
ACH-0001	4.891419	HS839T	Non-Canc	Fibroblast	Non-Cancerous
ACH-0002	3.405992	LN382	Brain Canc	Central Ne	Brain Cancer
ACH-0010	2.179511	143B	Bone Canc	Bone	Bone Cancer
ACH-0004	3.609991	SNU685	Endometri	Uterus	Endometrial/Uterine Cancer
ACH-0020	0	HOUAI	Endometri	Uterus	Endometrial/Uterine Cancer
ACH-0011	0	MTA	Lymphom	Lymphocy	Lymphoma
ACH-0012	1.327687	BIN67	Ovarian C	Ovary	Ovarian Cancer
ACH-0000	0.028569	MHHCALL	Leukemia	Blood	Leukemia
ACH-0014	1.448901	VP229	Breast Car	Breast	Breast Cancer
ACH-0004	2.650765	OELE	Non-Canc	Ovary	Non-Cancerous
ACH-0008	1.555816	KYSE450	Esophagea	Esophagus	Esophageal Cancer

ACH-0004	0.028569	ME1	Leukemia	Blood	Leukemia
ACH-0015	0.948601	HSC1	Skin Cance	Skin	Skin Cancer
ACH-0000	1.035624	OPM2	Myeloma	Plasma Ce	Myeloma
ACH-0006	0.124328	NUGC4	Gastric Ca	Gastric	Gastric Cancer
ACH-0006	2.587365	SBC5	Lung Canc	Lung	Lung Cancer
ACH-0005	0.070389	PATU8902	Pancreatic	Pancreas	Pancreatic Cancer
ACH-0002	0.632268	KASUMI1	Leukemia	Blood	Leukemia
ACH-0003	2.618239	SNU5	Gastric Ca	Gastric	Gastric Cancer
ACH-0015	3.340562	MCC142	Skin Cance	Skin	Skin Cancer
ACH-0007	3.092546	KATOIII	Gastric Ca	Gastric	Gastric Cancer
ACH-0025	3.452859	M140325	Skin Cance	Skin	Skin Cancer
ACH-0002	3.798051	SNU1033	Colon/Col	Colorectal	Colon/Colorectal Cancer
ACH-0002	0.097611	KMRC20	Kidney Ca	Kidney	Kidney Cancer
ACH-0002	0.042644	HNT34	Leukemia	Blood	Leukemia
ACH-0001	0.028569	PANC0327	Pancreatic	Pancreas	Pancreatic Cancer
ACH-0017	0	SEMK2	Leukemia	Blood	Leukemia
ACH-0013	1.432959	SUM1315I	Breast Car	Breast	Breast Cancer
ACH-0011	3.408712	RT11284	Bladder Ca	Urinary Tr	Bladder Cancer
ACH-0005	0.367371	K562	Leukemia	Blood	Leukemia
ACH-0011	0.495695	CCLFPEDS	Kidney Ca	Kidney	Kidney Cancer
ACH-0000	4.505891	S117	Sarcoma	Soft Tissue	Sarcoma
ACH-0007	3.41819	NCIH1623	Lung Canc	Lung	Lung Cancer
ACH-0004	1.819668	SNU449	Liver Canc	Liver	Liver Cancer
ACH-0003	1.691534	OCIAML3	Leukemia	Blood	Leukemia
ACH-0004	0.970854	KU1919	Bladder Ca	Urinary Tr	Bladder Cancer
ACH-0006	5.236875	OE19	Esophagea	Esophagus	Esophageal Cancer
ACH-0015	2.456806	MERO41	Lung Canc	Lung	Lung Cancer
ACH-0002	4.68818	OCUM1	Gastric Ca	Gastric	Gastric Cancer
ACH-0009	0.028569	OVK18	Ovarian Ca	Ovary	Ovarian Cancer
ACH-0008	0.124328	SNU738	Brain Canc	Central Ne	Brain Cancer
ACH-0004	0.014355	JHH4	Liver Canc	Liver	Liver Cancer
ACH-0005	0.176323	NCIH2171	Lung Canc	Lung	Lung Cancer
ACH-0011	2.286881	OCILY132	Lymphom	Lymphocy	Lymphoma
ACH-0008	2.523562	NCIH661	Lung Canc	Lung	Lung Cancer
ACH-0000	4.087463	HS281T	Non-Canc	Fibroblast	Non-Cancerous
ACH-0004	3.431623	KNS81	Brain Canc	Central Ne	Brain Cancer
ACH-0002	4.549669	HS742T	Non-Canc	Fibroblast	Non-Cancerous
ACH-0016	2.803227	SNU638	Gastric Ca	Gastric	Gastric Cancer
ACH-0009	5.492815	SNU407	Colon/Col	Colorectal	Colon/Colorectal Cancer
ACH-0000	3.295723	HEKTE	Non-Canc	Kidney	Non-Cancerous
ACH-0006	0.042644	NCIH1734	Lung Canc	Lung	Lung Cancer
ACH-0001	3.378512	HS255T	Non-Canc	Fibroblast	Non-Cancerous
ACH-0001	1.604071	KARPAS62	Myeloma	Plasma Ce	Myeloma
ACH-0005	0.739848	HUTU80	Gastric Ca	Gastric	Gastric Cancer
ACH-0016	0	SHI1	Leukemia	Blood	Leukemia
ACH-0016	2.85599	SHMAC5	Prostate C	Prostate	Prostate Cancer
ACH-0001	0.042644	SW1990	Pancreatic	Pancreas	Pancreatic Cancer
ACH-0002	0.042644	HPAC	Pancreatic	Pancreas	Pancreatic Cancer
ACH-0006	2.214125	SNU503	Colon/Col	Colorectal	Colon/Colorectal Cancer
ACH-0020	1.169925	OMM1	Eye Cance	Eye	Eye Cancer
ACH-0006	2.15056	SF268	Brain Canc	Central Ne	Brain Cancer
ACH-0001	6.85437	SKLMS1	Sarcoma	Soft Tissue	Sarcoma
ACH-0014	0.238787	EMTOKA	Endometri	Uterus	Endometrial/Uterine Cancer
ACH-0001	1.536053	SCC25	Head and	Upper Aer	Head and Neck Cancer
ACH-0000	0.028569	BDCM	Leukemia	Blood	Leukemia
ACH-0001	2.117695	THP1	Leukemia	Blood	Leukemia
ACH-0004	0.650765	BICR6	Head and	Upper Aer	Head and Neck Cancer
ACH-0015	1.594549	HEC116	Endometri	Uterus	Endometrial/Uterine Cancer

ACH-0008	1.608809	HCC1438	Lung Canc	Lung	Lung Cancer
ACH-0016	1.169925	OSC19	Head and	Upper Aer	Head and Neck Cancer
ACH-0008	4.331275	CL34	Colon/Col	Colorectal	Colon/Colorectal Cancer
ACH-0008	1.765535	MORCPR	Lung Canc	Lung	Lung Cancer
ACH-0008	0.042644	KYSE510	Esophagea	Esophagus	Esophageal Cancer
ACH-0005	0.124328	NCIH146	Lung Canc	Lung	Lung Cancer
ACH-0005	5.103078	HS606T	Non-Canc	Fibroblast	Non-Cancerous
ACH-0000	0.042644	HH	Lymphom	Lymphocy	Lymphoma
ACH-0001	3.91934	HS737T	Non-Canc	Fibroblast	Non-Cancerous
ACH-0005	5.714795	UACC812	Breast Car	Breast	Breast Cancer
ACH-0007	0.084064	P3HR1	Lymphom	Lymphocy	Lymphoma
ACH-0006	2.38405	BEN	Lung Canc	Lung	Lung Cancer
ACH-0009	0.475085	KCL22	Leukemia	Blood	Leukemia
ACH-0005	0.286881	KMS34	Myeloma	Plasma Ce	Myeloma
ACH-0006	5.385431	SW948	Colon/Col	Colorectal	Colon/Colorectal Cancer
ACH-0024	4.592158	HT144SKIN	Skin Cance	Skin	Skin Cancer
ACH-0001	0.111031	HCC1187	Breast Car	Breast	Breast Cancer
ACH-0024	0	RPE1SS77	Non-Canc	Eye	Non-Cancerous
ACH-0003	0.201634	MOLM16	Leukemia	Blood	Leukemia
ACH-0006	0.124328	SNU1041	Head and	Upper Aer	Head and Neck Cancer
ACH-0004	4.557042	SW1463	Colon/Col	Colorectal	Colon/Colorectal Cancer
ACH-0020	2.201634	PK8	Pancreatic	Pancreas	Pancreatic Cancer
ACH-0001	0.056584	HUPT3	Pancreatic	Pancreas	Pancreatic Cancer
ACH-0006	4.3067	RVH421	Skin Cance	Skin	Skin Cancer
ACH-0013	2.286881	CHP134	Neuroblas	Peripheral	Neuroblastoma
ACH-0004	0.056584	BV173	Leukemia	Blood	Leukemia
ACH-0005	3.869871	SIHA	Cervical C	Cervix	Cervical Cancer
ACH-0018	4.629357	LPS510	Liposarcor	Soft Tissue	Liposarcoma
ACH-0013	0.669027	SCLC22H	Lung Canc	Lung	Lung Cancer
ACH-0010	0.014355	DL40	Lymphom	Lymphocy	Lymphoma
ACH-0015	1.427606	HT3	Cervical C	Cervix	Cervical Cancer
ACH-0015	0	KML1	Lymphom	Lymphocy	Lymphoma
ACH-0000	4.514122	HS294T	Skin Cance	Skin	Skin Cancer
ACH-0027	4.470537	NCCLMS1	Unknown	Soft Tissue	Unknown
ACH-0006	2.543496	KYSE520	Esophagea	Esophagus	Esophageal Cancer
ACH-0026	2.513491	S462	Sarcoma	Peripheral	Sarcoma
ACH-0004	0.028569	LN428	Brain Canc	Central Ne	Brain Cancer
ACH-0003	5.417177	H4	Brain Canc	Central Ne	Brain Cancer
ACH-0027	4.422233	MPNST724	Sarcoma	Peripheral	Sarcoma
ACH-0001	3.690417	NCIH1341	Lung Canc	Lung	Lung Cancer
ACH-0004	2.513491	SF767	Cervical C	Cervix	Cervical Cancer
ACH-0004	0.176323	NCIH1915	Lung Canc	Lung	Lung Cancer
ACH-0010	2.689299	HELA	Cervical C	Cervix	Cervical Cancer
ACH-0020	3.911692	T3M5	Thyroid Ca	Thyroid	Thyroid Cancer
ACH-0005	0.014355	TTC709	Rhabdoid	Soft Tissue	Rhabdoid
ACH-0007	4.429616	GI1	Brain Canc	Central Ne	Brain Cancer
ACH-0003	5.51822	J82	Bladder Ca	Urinary Tr	Bladder Cancer
ACH-0000	5.577429	NCIH684	Colon/Col	Colorectal	Colon/Colorectal Cancer
ACH-0003	0	RL	Lymphom	Lymphocy	Lymphoma
ACH-0002	1.516015	KP4	Pancreatic	Pancreas	Pancreatic Cancer
ACH-0001	1.871844	JHOS2	Ovarian C	Ovary	Ovarian Cancer
ACH-0002	2.443607	JHESOAD1	Esophagea	Esophagus	Esophageal Cancer
ACH-0006	3.266037	HOS	Bone Canc	Bone	Bone Cancer
ACH-0008	0.014355	KYSE150	Esophagea	Esophagus	Esophageal Cancer
ACH-0009	3.349082	ES2	Ovarian C	Ovary	Ovarian Cancer
ACH-0003	6.119564	RPMI7951	Skin Cance	Skin	Skin Cancer
ACH-0011	0	KHYG	Lymphom	Lymphocy	Lymphoma
ACH-0002	1.584963	SCC4	Head and	Upper Aer	Head and Neck Cancer

ACH-0002	3.15056	JHOM1	Ovarian C	Ovary	Ovarian Cancer
ACH-0003	1.15056	HLF	Liver Canc	Liver	Liver Cancer
ACH-0006	5.260778	SNU201	Brain Canc	Central Ne	Brain Cancer
ACH-0003	3.060047	MG63	Bone Canc	Bone	Bone Cancer
ACH-0010	0.042644	CHLA06AT	Rhabdoid	Soft Tissue	Rhabdoid
ACH-0006	4.983678	MDAMB15	Breast Car	Breast	Breast Cancer
ACH-0016	0.555816	SAT	Head and	Upper Aer	Head and Neck Cancer
ACH-0000	0.214125	DOHH2	Lymphom	Lymphocy	Lymphoma
ACH-0004	4.208673	SNU182	Liver Canc	Liver	Liver Cancer
ACH-0005	6.136273	NCIH1092	Lung Canc	Lung	Lung Cancer
ACH-0000	0.070389	TE617T	Sarcoma	Soft Tissue	Sarcoma
ACH-0000	4.677508	HS895T	Non-Canc	Fibroblast	Non-Cancerous
ACH-0009	3.134221	EFO27	Ovarian C	Ovary	Ovarian Cancer
ACH-0007	3.993674	TT2609C0	Thyroid Ca	Thyroid	Thyroid Cancer
ACH-0009	2.192194	MFE319	Endometri	Uterus	Endometrial/Uterine Cancer
ACH-0009	7.403097	MDAPCA2	Prostate C	Prostate	Prostate Cancer
ACH-0000	0.201634	OCIAML5	Leukemia	Blood	Leukemia
ACH-0006	4.06264	SF126	Brain Canc	Central Ne	Brain Cancer
ACH-0014	3.921246	UMUC5	Bladder Ca	Urinary Tra	Bladder Cancer
ACH-0005	2.790772	SNB75	Brain Canc	Central Ne	Brain Cancer
ACH-0009	7.061668	22RV1	Prostate C	Prostate	Prostate Cancer
ACH-0017	4.378512	HS860T	Bone Canc	Bone	Bone Cancer
ACH-0006	0.356144	SUIT2	Pancreatic	Pancreas	Pancreatic Cancer
ACH-0004	0.333424	NCIH1944	Lung Canc	Lung	Lung Cancer
ACH-0004	0.028569	HSC2	Head and	Upper Aer	Head and Neck Cancer
ACH-0000	4.792335	LS513	Colon/Col	Colorectal	Colon/Colorectal Cancer
ACH-0004	3.666757	KPNSI9S	Neuroblas	Peripheral	Neuroblastoma
ACH-0003	0.014355	KG1	Leukemia	Blood	Leukemia
ACH-0018	0.956057	G415	Gallbladde	Bile Duct	Gallbladder Cancer
ACH-0007	5.521993	HCC2218	Breast Car	Breast	Breast Cancer
ACH-0000	2.260026	KPL1	Breast Car	Breast	Breast Cancer
ACH-0006	0.226509	SKMES1	Lung Canc	Lung	Lung Cancer
ACH-0000	5.100978	MHHNB11	Neuroblas	Peripheral	Neuroblastoma
ACH-0020	4.321207	HTMMT	Endometri	Uterus	Endometrial/Uterine Cancer
ACH-0003	0.042644	TEN	Endometri	Uterus	Endometrial/Uterine Cancer
ACH-0002	3.422233	HS822T	Non-Canc	Fibroblast	Non-Cancerous
ACH-0005	0.432959	CAL33	Head and	Upper Aer	Head and Neck Cancer
ACH-0015	2.475085	MM370	Skin Cance	Skin	Skin Cancer
ACH-0002	5.540709	LS1034	Colon/Col	Colorectal	Colon/Colorectal Cancer
ACH-0005	3.077243	G361	Skin Cance	Skin	Skin Cancer
ACH-0016	0	NO10	Brain Canc	Central Ne	Brain Cancer
ACH-0002	4.969933	HCC1419	Breast Car	Breast	Breast Cancer
ACH-0012	0.275007	WM2664	Skin Cance	Skin	Skin Cancer
ACH-0007	0.042644	DAUDI	Lymphom	Lymphocy	Lymphoma
ACH-0008	0.378512	COLO668	Lung Canc	Lung	Lung Cancer
ACH-0019	1.70044	MM253	Skin Cance	Skin	Skin Cancer
ACH-0020	5.787641	HSKTC	Ovarian C	Ovary	Ovarian Cancer
ACH-0005	3.588565	SNU761	Liver Canc	Liver	Liver Cancer
ACH-0004	1.627607	KMS28BM	Myeloma	Plasma Ce	Myeloma
ACH-0007	2.769772	HT1376	Bladder Ca	Urinary Tra	Bladder Cancer
ACH-0005	2.95977	UMUC3	Bladder Ca	Urinary Tra	Bladder Cancer
ACH-0015	1.891419	LU135	Lung Canc	Lung	Lung Cancer
ACH-0007	2.217231	EKVX	Lung Canc	Lung	Lung Cancer
ACH-0007	3.456806	NCIH1385	Lung Canc	Lung	Lung Cancer
ACH-0005	1.664483	BHY	Head and	Upper Aer	Head and Neck Cancer
ACH-0018	0.189034	ICC137	Bile Duct (	Bile Duct	Bile Duct Cancer
ACH-0017	4.196135	LPS141	Liposarcor	Soft Tissue	Liposarcoma
ACH-0015	0	MOLM1	Leukemia	Blood	Leukemia

ACH-0004	3.221877	UACC62	Skin Cance	Skin	Skin Cancer
ACH-0003	0.475085	G402	Kidney Ca	Kidney	Kidney Cancer
ACH-0007	0.124328	JMSU1	Bladder Ca	Urinary Tra	Bladder Cancer
ACH-0002	2.776104	HS834T	Non-Canc	Fibroblast	Non-Cancerous
ACH-0007	1.269033	PECAPJ49	Head and	Upper Aer	Head and Neck Cancer
ACH-0006	0.028569	RAJI	Lymphom	Lymphocy	Lymphoma
ACH-0010	0.367371	F5	Brain Canc	Central Ne	Brain Cancer
ACH-0001	5.513175	PRECLH	Non-Canc	Prostate	Non-Cancerous
ACH-0013	1.207893	SUM229PE	Breast Car	Breast	Breast Cancer
ACH-0004	3.270529	K029AX	Skin Cance	Skin	Skin Cancer
ACH-0008	5.350851	IGR1	Skin Cance	Skin	Skin Cancer
ACH-0015	1.333424	H357	Head and	Upper Aer	Head and Neck Cancer
ACH-0002	4.990501	CADOES1	Bone Canc	Bone	Bone Cancer
ACH-0002	3.001802	SCC15	Head and	Upper Aer	Head and Neck Cancer
ACH-0006	0.014355	KYO1	Leukemia	Blood	Leukemia
ACH-0000	5.223809	PC3	Prostate C	Prostate	Prostate Cancer
ACH-0001	0.722466	SET2	Leukemia	Blood	Leukemia
ACH-0017	3.590961	LPS6	Liposarcor	Soft Tissue	Liposarcoma
ACH-0016	0.014355	SISO	Cervical Ca	Cervix	Cervical Cancer
ACH-0006	2.333424	TE6	Esophagea	Esophagus	Esophageal Cancer
ACH-0004	1.765535	CJM	Skin Cance	Skin	Skin Cancer
ACH-0020	2.250962	MMAC	Skin Cance	Skin	Skin Cancer
ACH-0000	0.028569	HEL	Leukemia	Blood	Leukemia
ACH-0002	1.669027	NCIH2887	Lung Canc	Lung	Lung Cancer
ACH-0002	0.042644	JK1	Leukemia	Blood	Leukemia
ACH-0011	2.397803	SKRC31	Kidney Ca	Kidney	Kidney Cancer
ACH-0020	0	TL1	Lymphom	Lymphocy	Lymphoma
ACH-0014	2.521051	ASH3	Thyroid Ca	Thyroid	Thyroid Cancer
ACH-0006	4.850499	OV7	Ovarian Ca	Ovary	Ovarian Cancer
ACH-0004	0.056584	MEC1	Leukemia	Blood	Leukemia
ACH-0008	0.263034	EJM	Myeloma	Plasma Ce	Myeloma
ACH-0016	0	U2904	Lymphom	Lymphocy	Lymphoma
ACH-0016	3.22033	P4E6	Prostate C	Prostate	Prostate Cancer
ACH-0009	0.042644	NCIH2342	Lung Canc	Lung	Lung Cancer
ACH-0010	0	CMK115	Leukemia	Blood	Leukemia
ACH-0005	3.261531	HT1197	Bladder Ca	Urinary Tra	Bladder Cancer
ACH-0008	1.85997	CAL12T	Lung Canc	Lung	Lung Cancer
ACH-0011	0	OCILY12	Lymphom	Lymphocy	Lymphoma
ACH-0001	0.111031	GA10	Lymphom	Lymphocy	Lymphoma
ACH-0017	2.485427	RH36	Sarcoma	Soft Tissue	Sarcoma
ACH-0000	4.579542	T24	Bladder Ca	Urinary Tra	Bladder Cancer
ACH-0007	0.713696	HMC18	Breast Car	Breast	Breast Cancer
ACH-0000	4.27277	CACO2	Colon/Col	Colorectal	Colon/Colorectal Cancer
ACH-0000	2.427606	NCIH2452	Lung Canc	Lung	Lung Cancer
ACH-0006	3.372952	SW900	Lung Canc	Lung	Lung Cancer
ACH-0010	0	KD	Rhabdoid	Soft Tissue	Rhabdoid
ACH-0006	0.925999	NCIH28	Lung Canc	Lung	Lung Cancer
ACH-0009	4.499527	LOVO	Colon/Col	Colorectal	Colon/Colorectal Cancer
ACH-0000	0.097611	NCIH929	Myeloma	Plasma Ce	Myeloma
ACH-0011	0.084064	KOPN8	Leukemia	Blood	Leukemia
ACH-0003	1.933573	NCIH226	Lung Canc	Lung	Lung Cancer
ACH-0005	0.286881	NCIH1437	Lung Canc	Lung	Lung Cancer
ACH-0005	0.028569	KHM1B	Myeloma	Plasma Ce	Myeloma
ACH-0015	2.691534	HMY1	Skin Cance	Skin	Skin Cancer
ACH-0008	1.871844	HEC50B	Endometri	Uterus	Endometrial/Uterine Cancer
ACH-0004	2.899176	TC71	Bone Canc	Bone	Bone Cancer
ACH-0004	0.042644	OVSAHO	Ovarian Ca	Ovary	Ovarian Cancer
ACH-0006	4.043519	HS746T	Gastric Ca	Gastric	Gastric Cancer

ACH-0006	1.85599	NCIH596	Lung Canc	Lung	Lung Cancer
ACH-0005	0	INA6	Myeloma	Plasma Ce	Myeloma
ACH-0002	3.997292	U251MG	Brain Canc	Central Ne	Brain Cancer
ACH-0008	4.747387	SW403	Colon/Col	Colorectal	Colon/Colorectal Cancer
ACH-0012	0.014355	Y79	Eye Cance	Eye	Eye Cancer
ACH-0011	3.760221	CCLFPEDS	Sarcoma	Soft Tissue	Sarcoma
ACH-0008	0.042644	L540	Lymphom	Lymphocy	Lymphoma
ACH-0006	2.720278	JL1	Lung Canc	Lung	Lung Cancer
ACH-0000	5.942749	SIMA	Neuroblas	Peripheral	Neuroblastoma
ACH-0009	3.419539	CAL148	Breast Car	Breast	Breast Cancer
ACH-0006	2.666757	786O	Kidney Ca	Kidney	Kidney Cancer
ACH-0000	2.169925	ACCMESC	Lung Canc	Lung	Lung Cancer
ACH-0001	5.890933	SKNSH	Neuroblas	Peripheral	Neuroblastoma
ACH-0001	4.473137	CAL29	Bladder Ca	Urinary Tra	Bladder Cancer
ACH-0002	4.971773	KPNYN	Neuroblas	Peripheral	Neuroblastoma
ACH-0006	0.056584	A3KAW	Lymphom	Lymphocy	Lymphoma
ACH-0009	0.042644	HPBALL	Leukemia	Blood	Leukemia
ACH-0002	3.966246	CALU6	Lung Canc	Lung	Lung Cancer
ACH-0008	2.292782	KYSE410	Esophagea	Esophagus	Esophageal Cancer
ACH-0001	0.275007	RH41	Sarcoma	Soft Tissue	Sarcoma
ACH-0000	0.613532	PANC1005	Pancreatic	Pancreas	Pancreatic Cancer
ACH-0000	0.056584	NIHOVCA	Ovarian Ca	Ovary	Ovarian Cancer
ACH-0009	5.400196	LS180	Colon/Col	Colorectal	Colon/Colorectal Cancer
ACH-0006	1.280956	IGR37	Skin Canc	Skin	Skin Cancer
ACH-0010	1.195348	JR	Sarcoma	Soft Tissue	Sarcoma
ACH-0002	3.80426	HUG1N	Gastric Ca	Gastric	Gastric Cancer
ACH-0007	1.321928	YD38	Head and	Upper Aer	Head and Neck Cancer
ACH-0009	0.31034	RCHACV	Leukemia	Blood	Leukemia
ACH-0008	3.336283	HCC1954	Breast Car	Breast	Breast Cancer
ACH-0009	3.586164	HEC108	Endometri	Uterus	Endometrial/Uterine Cancer
ACH-0004	4.790772	SAOS2	Bone Canc	Bone	Bone Cancer
ACH-0009	6.516961	BT474	Breast Car	Breast	Breast Cancer
ACH-0018	0	KMCH1	Bile Duct (	Bile Duct	Bile Duct Cancer
ACH-0006	2.961623	TE9	Esophagea	Esophagus	Esophageal Cancer
ACH-0009	5.140779	MDST8	Colon/Col	Colorectal	Colon/Colorectal Cancer
ACH-0009	0.028569	MOLT16	Leukemia	Blood	Leukemia
ACH-0007	3.541019	NUGC2	Gastric Ca	Gastric	Gastric Cancer
ACH-0015	0.604071	MM127	Skin Canc	Skin	Skin Cancer
ACH-0009	0.084064	SNGM	Endometri	Uterus	Endometrial/Uterine Cancer
ACH-0002	0.084064	NCIH889	Lung Canc	Lung	Lung Cancer
ACH-0017	0	OCIC4P	Ovarian Ca	Ovary	Ovarian Cancer
ACH-0002	0.505891	DANG	Pancreatic	Pancreas	Pancreatic Cancer
ACH-0014	0.970854	UWB1289	Ovarian Ca	Ovary	Ovarian Cancer
ACH-0003	0.097611	MOLM13	Leukemia	Blood	Leukemia
ACH-0008	2.295723	HARA	Lung Canc	Lung	Lung Cancer
ACH-0008	0.084064	HCC366	Lung Canc	Lung	Lung Cancer
ACH-0018	1.903038	HKGZCC	Bile Duct (	Bile Duct	Bile Duct Cancer
ACH-0015	3.82171	IHH4	Thyroid Ca	Thyroid	Thyroid Cancer
ACH-0016	0.070389	SKNO1	Leukemia	Blood	Leukemia
ACH-0000	2.253989	SLR21	Kidney Ca	Kidney	Kidney Cancer
ACH-0001	3.235727	HS751T	Non-Canc	Fibroblast	Non-Cancerous
ACH-0014	3.687061	C84	Colon/Col	Colorectal	Colon/Colorectal Cancer
ACH-0005	5.333424	TM31	Brain Canc	Central Ne	Brain Cancer
ACH-0011	4.290572	SKNEP1	Bone Canc	Bone	Bone Cancer
ACH-0000	2.41684	MV411	Leukemia	Blood	Leukemia
ACH-0007	0.475085	UBLC1	Bladder Ca	Urinary Tra	Bladder Cancer
ACH-0009	1.769772	NCIH2286	Lung Canc	Lung	Lung Cancer
ACH-0005	4.281698	T173	Non-Canc	Fibroblast	Non-Cancerous

ACH-0005	3.214125	CORL88	Lung Canc	Lung	Lung Cancer
ACH-0005	3.812498	JHH2	Liver Canc	Liver	Liver Cancer
ACH-0007	0.042644	MDAMB23	Breast Car	Breast	Breast Cancer
ACH-0006	0.963474	RPMI8402	Leukemia	Blood	Leukemia
ACH-0006	0.028569	HCC44	Lung Canc	Lung	Lung Cancer
ACH-0009	5.050066	5637	Bladder Ca	Urinary Tra	Bladder Cancer
ACH-0014	2.970854	EGI1	Bile Duct (	Bile Duct	Bile Duct Cancer
ACH-0003	3.476382	TF1	Leukemia	Blood	Leukemia
ACH-0025	2.097611	M040416	Skin Cance	Skin	Skin Cancer
ACH-0013	4.888987	NB1643	Neuroblas	Peripheral	Neuroblastoma
ACH-0000	1.757023	253JBV	Bladder Ca	Urinary Tra	Bladder Cancer
ACH-0008	0.070389	AMO1	Myeloma	Plasma Ce	Myeloma
ACH-0011	3.899176	U251MGD	Brain Canc	Central Ne	Brain Cancer
ACH-0008	4.253233	SKMEL24	Skin Cance	Skin	Skin Cancer
ACH-0005	4.794416	RS5	Non-Canc	Fibroblast	Non-Cancerous
ACH-0003	4.566815	HCC1500	Breast Car	Breast	Breast Cancer
ACH-0005	0.111031	OVISE	Ovarian Ca	Ovary	Ovarian Cancer
ACH-0002	5.317594	EWS502	Bone Canc	Bone	Bone Cancer
ACH-0005	5.325171	CAL78	Bone Canc	Bone	Bone Cancer
ACH-0008	1.570463	COLO783	Skin Cance	Skin	Skin Cancer
ACH-0013	4.982309	CHLA15	Neuroblas	Peripheral	Neuroblastoma
ACH-0005	0.042644	KURAMOC	Ovarian Ca	Ovary	Ovarian Cancer
ACH-0006	0.124328	SUDHL6	Lymphom	Lymphocy	Lymphoma
ACH-0004	1.226509	769P	Kidney Ca	Kidney	Kidney Cancer
ACH-0007	0.056584	NCIH2291	Lung Canc	Lung	Lung Cancer
ACH-0009	2.976364	FTC133	Thyroid Ca	Thyroid	Thyroid Cancer
ACH-0020	3.060047	T3M3	Endometri	Uterus	Endometrial/Uterine Cancer
ACH-0004	0.014355	CAKI1	Kidney Ca	Kidney	Kidney Cancer
ACH-0004	2.908813	EW8	Bone Canc	Bone	Bone Cancer
ACH-0002	0.201634	COV362	Ovarian Ca	Ovary	Ovarian Cancer
ACH-0002	4.730096	COLO320	Colon/Col	Colorectal	Colon/Colorectal Cancer
ACH-0017	0	HB1119	Leukemia	Blood	Leukemia
ACH-0013	0.15056	PACADD1	Pancreatic	Pancreas	Pancreatic Cancer
ACH-0001	4.737687	LN319	Brain Canc	Central Ne	Brain Cancer
ACH-0006	0.85599	OVCAR8	Ovarian Ca	Ovary	Ovarian Cancer
ACH-0000	7.313337	RDES	Bone Canc	Bone	Bone Cancer
ACH-0002	1.655352	HS675T	Non-Canc	Fibroblast	Non-Cancerous
ACH-0007	2.042644	NCIH1703	Lung Canc	Lung	Lung Cancer
ACH-0013	2.553361	8505C	Thyroid Ca	Thyroid	Thyroid Cancer
ACH-0003	8.634158	SKNFI	Neuroblas	Peripheral	Neuroblastoma
ACH-0017	0	RH28	Sarcoma	Soft Tissue	Sarcoma
ACH-0004	0.85599	PANC0813	Pancreatic	Pancreas	Pancreatic Cancer
ACH-0013	2.845992	SUM190PT	Breast Car	Breast	Breast Cancer
ACH-0001	6.354382	CHP212	Neuroblas	Peripheral	Neuroblastoma
ACH-0016	2.176323	UPCISCC0	Head and	Upper Aer	Head and Neck Cancer
ACH-0015	1.432959	KON	Head and	Upper Aer	Head and Neck Cancer
ACH-0025	2.827819	WM4235	Skin Cance	Skin	Skin Cancer
ACH-0004	0.042644	LU65	Lung Canc	Lung	Lung Cancer
ACH-0013	2.130931	NGP	Neuroblas	Peripheral	Neuroblastoma
ACH-0006	3.500802	JJN3	Myeloma	Plasma Ce	Myeloma
ACH-0008	1.967169	SCABER	Bladder Ca	Urinary Tra	Bladder Cancer
ACH-0006	5.554589	LN443	Brain Canc	Central Ne	Brain Cancer
ACH-0015	3.941106	HSC5	Skin Cance	Skin	Skin Cancer
ACH-0000	0.790772	HS611T	Lymphom	Lymphocy	Lymphoma
ACH-0003	3.730096	42MGBA	Brain Canc	Central Ne	Brain Cancer
ACH-0000	0.356144	NCIH1693	Lung Canc	Lung	Lung Cancer
ACH-0018	0.298658	TKKK	Bile Duct (	Bile Duct	Bile Duct Cancer
ACH-0009	1.687061	NCIH1155	Lung Canc	Lung	Lung Cancer

ACH-0001	0.070389	JVM2	Lymphom	Lymphocy	Lymphoma
ACH-0007	3.772941	JHH5	Liver Canc	Liver	Liver Cancer
ACH-0008	1.15056	NCIH1373	Lung Canc	Lung	Lung Cancer
ACH-0019	0.028569	NZM3	Skin Cance	Skin	Skin Cancer
ACH-0007	2.89724	SKMEL5	Skin Cance	Skin	Skin Cancer
ACH-0013	1.049631	C4I	Cervical C	Cervix	Cervical Cancer
ACH-0006	0.042644	DMS53	Lung Canc	Lung	Lung Cancer
ACH-0008	1.815575	BFTC905	Bladder Ca	Urinary Tr	Bladder Cancer
ACH-0018	2.62293	ICC108	Bile Duct (	Bile Duct	Bile Duct Cancer
ACH-0005	2.722466	MDAMB43	Breast Car	Breast	Breast Cancer
ACH-0016	1.137504	UPCISCC1	Head and	Upper Aer	Head and Neck Cancer
ACH-0009	4.707083	CCK81	Colon/Col	Colorectal	Colon/Colorectal Cancer
ACH-0015	4.968091	MM383	Skin Cance	Skin	Skin Cancer
ACH-0016	1.438293	SKGT2	Gastric Ca	Gastric	Gastric Cancer
ACH-0008	2.570463	NCIH1435	Lung Canc	Lung	Lung Cancer
ACH-0018	0	TGBC52TK	Bile Duct (	Bile Duct	Bile Duct Cancer
ACH-0006	5.564683	WM1799	Skin Cance	Skin	Skin Cancer
ACH-0015	2.408712	FLO1	Esophage	Esophagus	Esophageal Cancer
ACH-0011	3.109361	SKMEL2	Skin Cance	Skin	Skin Cancer
ACH-0024	0.014355	RPE1SS51	Non-Canc	Eye	Non-Cancerous
ACH-0003	3.517276	U2OS	Bone Canc	Bone	Bone Cancer
ACH-0004	1.130931	NCIH1666	Lung Canc	Lung	Lung Cancer
ACH-0009	3.263034	SNU1	Gastric Ca	Gastric	Gastric Cancer
ACH-0007	6.812883	CAMA1	Breast Car	Breast	Breast Cancer
ACH-0020	4.72792	HSQ89	Head and	Upper Aer	Head and Neck Cancer
ACH-0003	0	LAMA84	Leukemia	Blood	Leukemia
ACH-0024	5.964168	HT144SKII	Skin Cance	Skin	Skin Cancer
ACH-0009	1.570463	HUCCT1	Bile Duct (	Bile Duct	Bile Duct Cancer
ACH-0015	3.884598	HCSC1	Cervical C	Cervix	Cervical Cancer
ACH-0013	6.314153	LAN2	Neuroblas	Peripheral	Neuroblastoma
ACH-0016	5.056584	SEKI	Skin Cance	Skin	Skin Cancer
ACH-0008	0.807355	CHAGOK1	Lung Canc	Lung	Lung Cancer
ACH-0003	0.084064	WM115	Skin Cance	Skin	Skin Cancer
ACH-0020	3.496974	HSOS1	Bone Canc	Bone	Bone Cancer
ACH-0009	5.148527	HMCB	Skin Cance	Skin	Skin Cancer
ACH-0013	0	PA1	Ovarian C	Ovary	Ovarian Cancer
ACH-0003	0.097611	SNU1077	Endometri	Uterus	Endometrial/Uterine Cancer
ACH-0003	2.028569	ECGI10	Esophage	Esophagus	Esophageal Cancer
ACH-0000	0.042644	MEG01	Leukemia	Blood	Leukemia
ACH-0007	0.333424	NCIH1648	Lung Canc	Lung	Lung Cancer
ACH-0005	2.028569	NCIH2004	Rhabdoid	Soft Tissue	Rhabdoid
ACH-0003	0.163499	NCIH522	Lung Canc	Lung	Lung Cancer
ACH-0001	0.62293	VMRCRCZ	Kidney Ca	Kidney	Kidney Cancer
ACH-0001	4.071248	JHUEM3	Endometri	Uterus	Endometrial/Uterine Cancer
ACH-0005	0.042644	BXPC3	Pancreatic	Pancreas	Pancreatic Cancer
ACH-0007	0.536053	RERFLCAD	Lung Canc	Lung	Lung Cancer
ACH-0001	0.137504	HS766T	Pancreatic	Pancreas	Pancreatic Cancer
ACH-0013	4.034744	8305C	Thyroid Ca	Thyroid	Thyroid Cancer
ACH-0009	0.056584	HEC59	Endometri	Uterus	Endometrial/Uterine Cancer
ACH-0011	6.170526	SHSY5Y	Neuroblas	Peripheral	Neuroblastoma
ACH-0003	0.097611	P12ICHIKA	Leukemia	Blood	Leukemia
ACH-0008	6.26791	NB1	Neuroblas	Peripheral	Neuroblastoma
ACH-0000	1.521051	253J	Bladder Ca	Urinary Tr	Bladder Cancer
ACH-0007	0.042644	NCIH1838	Lung Canc	Lung	Lung Cancer
ACH-0016	5.391287	NH12	Neuroblas	Peripheral	Neuroblastoma
ACH-0008	0.056584	HUNS1	Myeloma	Plasma Ce	Myeloma
ACH-0001	0.084064	SNU308	Gallbladde	Bile Duct	Gallbladder Cancer
ACH-0008	2.611172	SKMEL30	Skin Cance	Skin	Skin Cancer

ACH-0011	2.989139	OC316	Ovarian C	Ovary	Ovarian Cancer
ACH-0007	3.221877	COLO680I	Esophagea	Esophagus	Esophageal Cancer
ACH-0007	0.014355	GB1	Brain Canc	Central Ne	Brain Cancer
ACH-0018	2.017922	ICC3	Bile Duct (	Bile Duct	Bile Duct Cancer
ACH-0026	2.523562	JVE253	Colon/Col	Colorectal	Colon/Colorectal Cancer
ACH-0007	7.627169	NCIH1105	Lung Canc	Lung	Lung Cancer
ACH-0015	3.377124	LS	Neuroblas	Peripheral	Neuroblastoma
ACH-0007	5.463034	MDAMB1	Breast Car	Breast	Breast Cancer
ACH-0015	2.353323	MERO14	Lung Canc	Lung	Lung Cancer
ACH-0013	0.815575	OCIC5X	Ovarian C	Ovary	Ovarian Cancer
ACH-0009	3.878725	KM12	Colon/Col	Colorectal	Colon/Colorectal Cancer
ACH-0008	2.422233	KYSE270	Esophagea	Esophagus	Esophageal Cancer
ACH-0012	5.384395	SYO1	Sarcoma	Soft Tissue	Sarcoma
ACH-0009	3.752749	NCIH157D	Lung Canc	Lung	Lung Cancer
ACH-0013	3.581351	OV17R	Ovarian C	Ovary	Ovarian Cancer
ACH-0028	4.564378	NCCMPNS	Unknown	Peripheral	Unknown
ACH-0010	2.655352	CBAGPN	Bone Canc	Bone	Bone Cancer
ACH-0000	6.380764	G292CLON	Bone Canc	Bone	Bone Cancer
ACH-0004	2.280956	TE11	Esophagea	Esophagus	Esophageal Cancer
ACH-0007	4.05398	SHP77	Lung Canc	Lung	Lung Cancer
ACH-0001	0.137504	TM87	Rhabdoid	Soft Tissue	Rhabdoid
ACH-0006	0.042644	TE1	Esophagea	Esophagus	Esophageal Cancer
ACH-0006	0.042644	M07E	Leukemia	Blood	Leukemia
ACH-0001	0.028569	MHHCALL	Leukemia	Blood	Leukemia
ACH-0020	3.628774	A375SKIN	Skin Cance	Skin	Skin Cancer
ACH-0003	0.189034	KARPAS42	Lymphom	Lymphocy	Lymphoma
ACH-0024	0.757023	CCLFUPGI	Gastric Ca	Gastric	Gastric Cancer
ACH-0002	0.124328	A204	Rhabdoid	Soft Tissue	Rhabdoid
ACH-0004	0.028569	EFE184	Endometri	Uterus	Endometrial/Uterine Cancer
ACH-0009	2.899176	RL952	Endometri	Uterus	Endometrial/Uterine Cancer
ACH-0015	0.137504	MCC13	Skin Cance	Skin	Skin Cancer
ACH-0020	0	HHUA	Endometri	Uterus	Endometrial/Uterine Cancer
ACH-0008	4.313971	SW1783	Brain Canc	Central Ne	Brain Cancer
ACH-0004	0.111031	RERFLCKJ	Lung Canc	Lung	Lung Cancer
ACH-0004	3.574102	MELHO	Skin Cance	Skin	Skin Cancer
ACH-0026	0	MAPACHS	Pancreatic	Pancreas	Pancreatic Cancer
ACH-0008	5.91576	GCT	Sarcoma	Soft Tissue	Sarcoma
ACH-0018	2.440952	LPS853	Liposarcor	Soft Tissue	Liposarcoma
ACH-0006	3.997292	IOMMLEE	Brain Canc	Central Ne	Brain Cancer
ACH-0007	1.464668	SNU1214	Head and	Upper Aer	Head and Neck Cancer
ACH-0008	4.831371	NCIH1930	Lung Canc	Lung	Lung Cancer
ACH-0008	0.028569	RS411	Leukemia	Blood	Leukemia
ACH-0009	5.349082	MDAMB45	Breast Car	Breast	Breast Cancer
ACH-0007	0.028569	PC9	Lung Canc	Lung	Lung Cancer
ACH-0001	0.084064	PFEIFFER	Lymphom	Lymphocy	Lymphoma
ACH-0011	2.014355	SMSCTR	Sarcoma	Soft Tissue	Sarcoma
ACH-0014	0	CI	Leukemia	Blood	Leukemia
ACH-0006	1.070389	KYSE180	Esophagea	Esophagus	Esophageal Cancer
ACH-0008	0.084064	SKOV3	Ovarian C	Ovary	Ovarian Cancer
ACH-0001	0.097611	KE37	Leukemia	Blood	Leukemia
ACH-0004	2.31034	SKMEL3	Skin Cance	Skin	Skin Cancer
ACH-0002	4.255501	NCIH209	Lung Canc	Lung	Lung Cancer
ACH-0004	0.014355	MUTZ5	Leukemia	Blood	Leukemia
ACH-0015	1.594549	HEMCSS	Bone Canc	Bone	Bone Cancer
ACH-0001	2.863938	NCCSTCK	Gastric Ca	Gastric	Gastric Cancer
ACH-0014	3.518535	ESO51	Esophagea	Esophagus	Esophageal Cancer
ACH-0001	4.417515	GMS10	Brain Canc	Central Ne	Brain Cancer
ACH-0008	0.863938	RPMI8226	Myeloma	Plasma Ce	Myeloma

ACH-0015	1.604071	MERO25	Lung Canc	Lung	Lung Cancer
ACH-0009	0.028569	MOLT3	Leukemia	Blood	Leukemia
ACH-0003	0.028569	NCIH647	Lung Canc	Lung	Lung Cancer
ACH-0009	0.321928	ESS1	Endometri	Uterus	Endometrial/Uterine Cancer
ACH-0005	2.78031	HCC364	Lung Canc	Lung	Lung Cancer
ACH-0006	4.860466	LN340	Brain Canc	Central Ne	Brain Cancer
ACH-0013	0.650765	CME1	Sarcoma	Soft Tissue	Sarcoma
ACH-0007	6.854744	NCIH526	Lung Canc	Lung	Lung Cancer
ACH-0016	0	SCC3	Lymphom	Lymphocy	Lymphoma
ACH-0015	2.784504	MCC26	Skin Cance	Skin	Skin Cancer
ACH-0004	0.594549	NCIH2085	Lung Canc	Lung	Lung Cancer
ACH-0008	3.57289	MFE296	Endometri	Uterus	Endometrial/Uterine Cancer
ACH-0018	1.485427	ICC2	Bile Duct (	Bile Duct	Bile Duct Cancer
ACH-0010	1.956057	CHLA32	Bone Canc	Bone	Bone Cancer
ACH-0009	4.62	GP2D	Colon/Col	Colorectal	Colon/Colorectal Cancer
ACH-0016	0	RAMOS	Lymphom	Lymphocy	Lymphoma
ACH-0001	2.752749	RCC10RGI	Kidney Ca	Kidney	Kidney Cancer
ACH-0004	1.752749	HCC95	Lung Canc	Lung	Lung Cancer
ACH-0001	4.942045	HS229T	Non-Canc	Fibroblast	Non-Cancerous
ACH-0006	0.367371	SUDHL5	Lymphom	Lymphocy	Lymphoma
ACH-0011	0	SMZ1	Lymphom	Lymphocy	Lymphoma
ACH-0009	0.056584	IPC298	Skin Cance	Skin	Skin Cancer
ACH-0004	4.606442	HUH7	Liver Canc	Liver	Liver Cancer
ACH-0007	0.137504	MOLP8	Myeloma	Plasma Ce	Myeloma
ACH-0001	0.042644	CORL105	Lung Canc	Lung	Lung Cancer
ACH-0003	6.579693	NCIH520	Lung Canc	Lung	Lung Cancer
ACH-0014	3.040892	C80	Colon/Col	Colorectal	Colon/Colorectal Cancer
ACH-0009	3.431623	NCIH1573	Lung Canc	Lung	Lung Cancer
ACH-0016	4.088311	NP3	Brain Canc	Central Ne	Brain Cancer
ACH-0019	0	CCSW1	Bile Duct (	Bile Duct	Bile Duct Cancer
ACH-0018	1.367371	RBE	Bile Duct (	Bile Duct	Bile Duct Cancer
ACH-0002	0.070389	EM2	Leukemia	Blood	Leukemia
ACH-0014	0	CII	Leukemia	Blood	Leukemia
ACH-0002	2.160275	NCIH841	Lung Canc	Lung	Lung Cancer
ACH-0001	3.62293	8MGBA	Brain Canc	Central Ne	Brain Cancer
ACH-0003	0.422233	EFO21	Ovarian C	Ovary	Ovarian Cancer
ACH-0004	0.070389	KNS60	Brain Canc	Central Ne	Brain Cancer
ACH-0012	0.925999	UPCISCC1	Head and	Upper Aer	Head and Neck Cancer
ACH-0001	3.9855	RERFGC1E	Gastric Ca	Gastric	Gastric Cancer
ACH-0008	0.056584	NCIH322	Lung Canc	Lung	Lung Cancer
ACH-0000	1.608809	TOV112D	Ovarian C	Ovary	Ovarian Cancer
ACH-0004	2.238787	NCIH460	Lung Canc	Lung	Lung Cancer
ACH-0008	4.408032	MELJUSO	Skin Cance	Skin	Skin Cancer
ACH-0003	0.028569	NUDHL1	Lymphom	Lymphocy	Lymphoma
ACH-0019	3.877744	ONE58	Lung Canc	Lung	Lung Cancer
ACH-0001	2.682573	T47D	Breast Car	Breast	Breast Cancer
ACH-0009	3.181103	HCC1569	Breast Car	Breast	Breast Cancer
ACH-0008	5.527321	WM88	Skin Cance	Skin	Skin Cancer
ACH-0003	5.924337	SKNDZ	Neuroblas	Peripheral	Neuroblastoma
ACH-0015	3.942984	HUO9	Bone Canc	Bone	Bone Cancer
ACH-0000	0.422233	SALE	Non-Canc	Lung	Non-Cancerous
ACH-0013	0.042644	ANGMCSS	Brain Canc	Central Ne	Brain Cancer
ACH-0004	5.315784	SW1353	Bone Canc	Bone	Bone Cancer
ACH-0012	5.848998	SCS214	Sarcoma	Soft Tissue	Sarcoma
ACH-0008	4.573496	HOP62	Lung Canc	Lung	Lung Cancer
ACH-0006	1.014355	SNU899	Head and	Upper Aer	Head and Neck Cancer
ACH-0001	2.408712	CAPAN2	Pancreatic	Pancreas	Pancreatic Cancer
ACH-0001	5.517906	HS729	Unknown	Soft Tissue	Unknown

ACH-0003	4.997744	HS688AT	Non-Canc	Fibroblast	Non-Cancerous
ACH-0015	3.012569	JEG3	Endometri	Uterus	Endometrial/Uterine Cancer
ACH-0006	1.646163	SW620	Colon/Col	Colorectal	Colon/Colorectal Cancer
ACH-0005	0.555816	EBC1	Lung Canc	Lung	Lung Cancer
ACH-0000	5.036064	ML1	Thyroid Ca	Thyroid	Thyroid Cancer
ACH-0003	8.196873	KPNRTBM	Neuroblas	Peripheral	Neuroblastoma
ACH-0003	4.625855	SNU886	Liver Canc	Liver	Liver Cancer
ACH-0008	3.531069	MDAMB43	Skin Canc	Skin	Skin Cancer
ACH-0002	0.238787	SNU466	Brain Canc	Central Ne	Brain Cancer
ACH-0014	0.85599	BOKU	Cervical Ca	Cervix	Cervical Cancer
ACH-0003	4.330558	NCIH82	Lung Canc	Lung	Lung Cancer
ACH-0029	0.432959	UPMD1	Unknown	Unknown	Unknown
ACH-0009	2.350497	OC314	Ovarian Ca	Ovary	Ovarian Cancer
ACH-0004	4.109361	RT112	Bladder Ca	Urinary Tra	Bladder Cancer
ACH-0001	4.128458	MFE280	Endometri	Uterus	Endometrial/Uterine Cancer
ACH-0016	0	ONDA9	Brain Canc	Central Ne	Brain Cancer
ACH-0018	2.722466	LPS067	Liposarcor	Soft Tissue	Liposarcoma
ACH-0024	0	RPE1SS11	Non-Canc	Eye	Non-Cancerous
ACH-0013	2.845992	GIMEN	Neuroblas	Peripheral	Neuroblastoma
ACH-0007	1.584963	A427	Lung Canc	Lung	Lung Cancer
ACH-0000	3.571677	HT1080	Sarcoma	Soft Tissue	Sarcoma
ACH-0013	3.107688	PLCPRF5	Liver Canc	Liver	Liver Cancer
ACH-0006	0.070389	MIAPACA2	Pancreatic	Pancreas	Pancreatic Cancer
ACH-0006	0.669027	SNU119	Ovarian Ca	Ovary	Ovarian Cancer
ACH-0018	0	SG231	Bile Duct (	Bile Duct	Bile Duct Cancer
ACH-0009	0.056584	IGROV1	Ovarian Ca	Ovary	Ovarian Cancer
ACH-0006	4.915043	YD8	Head and	Upper Aer	Head and Neck Cancer
ACH-0002	0.111031	ECC12	Gastric Ca	Gastric	Gastric Cancer
ACH-0006	0.042644	A2780	Ovarian Ca	Ovary	Ovarian Cancer
ACH-0006	2.176323	HCC1806	Breast Car	Breast	Breast Cancer
ACH-0006	5.047451	HCC1395	Breast Car	Breast	Breast Cancer
ACH-0009	0.028569	BCP1	Lymphom	Lymphocy	Lymphoma
ACH-0006	0.163499	CI1	Lymphom	Lymphocy	Lymphoma
ACH-0000	7.394034	MDAMB13	Breast Car	Breast	Breast Cancer
ACH-0007	1.937344	A253	Head and	Upper Aer	Head and Neck Cancer
ACH-0004	1.627607	NCIH2228	Lung Canc	Lung	Lung Cancer
ACH-0014	3.255501	UMUC9	Bladder Ca	Urinary Tra	Bladder Cancer
ACH-0003	0.042644	MPP89	Lung Canc	Lung	Lung Cancer
ACH-0001	1.02148	EOL1	Leukemia	Blood	Leukemia
ACH-0002	3.090853	SNU840	Ovarian Ca	Ovary	Ovarian Cancer
ACH-0001	0.014355	LOUCY	Leukemia	Blood	Leukemia
ACH-0018	2.056584	ICC106	Bile Duct (	Bile Duct	Bile Duct Cancer
ACH-0008	1.599318	NCIH1563	Lung Canc	Lung	Lung Cancer
ACH-0004	5.616475	SNU423	Liver Canc	Liver	Liver Cancer
ACH-0000	0.028569	PANC0203	Pancreatic	Pancreas	Pancreatic Cancer
ACH-0020	0.641546	HO1U1	Head and	Upper Aer	Head and Neck Cancer
ACH-0002	3.157044	SNU398	Liver Canc	Liver	Liver Cancer
ACH-0000	1.443607	PANC0504	Pancreatic	Pancreas	Pancreatic Cancer
ACH-0004	0.056584	MALME3M	Skin Canc	Skin	Skin Cancer
ACH-0007	3.163499	GSS	Gastric Ca	Gastric	Gastric Cancer
ACH-0003	3.007196	MKN45	Gastric Ca	Gastric	Gastric Cancer
ACH-0004	1.757023	CAL54	Kidney Ca	Kidney	Kidney Cancer
ACH-0009	0.137504	NCIH2106	Lung Canc	Lung	Lung Cancer
ACH-0002	2.592158	DKMG	Brain Canc	Central Ne	Brain Cancer
ACH-0006	0.176323	NCIH2227	Lung Canc	Lung	Lung Cancer
ACH-0000	0.189034	GDM1	Leukemia	Blood	Leukemia
ACH-0002	5.095503	LMSU	Gastric Ca	Gastric	Gastric Cancer
ACH-0010	2.137504	COGE352	Bone Canc	Bone	Bone Cancer

ACH-0003	1.550901	HCC1428	Breast Car	Breast	Breast Cancer
ACH-0020	0.903038	MEL290	Eye Cance	Eye	Eye Cancer
ACH-0004	0.275007	NCIH838	Lung Canc	Lung	Lung Cancer
ACH-0007	0.124328	NCIH196	Lung Canc	Lung	Lung Cancer
ACH-0008	6.029674	KMBC2	Bladder Ca	Urinary Tra	Bladder Cancer
ACH-0002	6.210623	SW1417	Colon/Col	Colorectal	Colon/Colorectal Cancer
ACH-0004	4.32553	COLO800	Skin Cance	Skin	Skin Cancer
ACH-0007	0.815575	HCC2279	Lung Canc	Lung	Lung Cancer
ACH-0008	0.042644	NCIH2347	Lung Canc	Lung	Lung Cancer
ACH-0006	0.604071	U266B1	Myeloma	Plasma Ce	Myeloma
ACH-0005	1.922198	BC3C	Bladder Ca	Urinary Tra	Bladder Cancer
ACH-0001	2.582556	SNU869	Bile Duct (	Bile Duct	Bile Duct Cancer
ACH-0004	5.380245	RERFLCSQ	Lung Canc	Lung	Lung Cancer
ACH-0001	0.056584	OSRC2	Kidney Ca	Kidney	Kidney Cancer
ACH-0009	2.763412	SNU349	Kidney Ca	Kidney	Kidney Cancer
ACH-0001	1.650765	BHT101	Thyroid Ca	Thyroid	Thyroid Cancer
ACH-0002	0.084064	PK59	Pancreatic	Pancreas	Pancreatic Cancer
ACH-0001	4.540399	CAOV4	Ovarian Ca	Ovary	Ovarian Cancer
ACH-0007	4.817623	HEPG2	Liver Canc	Liver	Liver Cancer
ACH-0015	3.192194	MERO48A	Lung Canc	Lung	Lung Cancer
ACH-0002	3.269033	C8166	Lymphom	Lymphocy	Lymphoma
ACH-0008	2.035624	NCIH2087	Lung Canc	Lung	Lung Cancer
ACH-0006	2.596935	SKMEL28	Skin Cance	Skin	Skin Cancer
ACH-0016	0	OCIAML4	Leukemia	Blood	Leukemia
ACH-0016	2.521051	NP2	Brain Canc	Central Ne	Brain Cancer
ACH-0008	1.823749	HS936T	Skin Cance	Skin	Skin Cancer
ACH-0005	5.438958	HS618T	Non-Canc	Fibroblast	Non-Cancerous
ACH-0004	4.370164	CAS1	Brain Canc	Central Ne	Brain Cancer
ACH-0006	3.649615	KNS42	Brain Canc	Central Ne	Brain Cancer
ACH-0004	3.494416	TE8	Esophagea	Esophagus	Esophageal Cancer
ACH-0013	2.238787	SUM149PT	Breast Car	Breast	Breast Cancer
ACH-0014	1.794936	SW13	Adrenal Ca	Adrenal C	Adrenal Cancer
ACH-0003	0.070389	SUDHL4	Lymphom	Lymphocy	Lymphoma
ACH-0014	1.937344	TC205	Bone Canc	Bone	Bone Cancer
ACH-0004	4.269033	HUH1	Liver Canc	Liver	Liver Cancer
ACH-0008	0.028569	NCIH358	Lung Canc	Lung	Lung Cancer
ACH-0000	0.070389	KU812	Leukemia	Blood	Leukemia
ACH-0003	3.02148	SNU478	Bile Duct (	Bile Duct	Bile Duct Cancer
ACH-0015	3.50716	MERO95	Lung Canc	Lung	Lung Cancer
ACH-0002	6.428444	KELLY	Neuroblas	Peripheral	Neuroblastoma
ACH-0013	0.641546	SUM102PT	Breast Car	Breast	Breast Cancer
ACH-0014	3.438293	UMUC4	Bladder Ca	Urinary Tra	Bladder Cancer
ACH-0020	2.414136	TT1TKB	Colon/Col	Colorectal	Colon/Colorectal Cancer
ACH-0007	0.378512	TCCSUP	Bladder Ca	Urinary Tra	Bladder Cancer
ACH-0000	1.944858	D341	Brain Canc	Central Ne	Brain Cancer
ACH-0013	2.879706	GP5D	Colon/Col	Colorectal	Colon/Colorectal Cancer
ACH-0005	2.025029	A172	Brain Canc	Central Ne	Brain Cancer
ACH-0006	1.794936	SUDHL1	Lymphom	Lymphocy	Lymphoma
ACH-0001	5.042644	NCIH660	Prostate C	Prostate	Prostate Cancer
ACH-0003	3.473787	MSTO211	Lung Canc	Lung	Lung Cancer
ACH-0009	0.62293	AN3CA	Endometri	Uterus	Endometrial/Uterine Cancer
ACH-0006	0.042644	KS1	Brain Canc	Central Ne	Brain Cancer
ACH-0002	1.056584	DETROIT5	Head and	Upper Aer	Head and Neck Cancer
ACH-0007	4.047887	SH10TC	Gastric Ca	Gastric	Gastric Cancer
ACH-0009	0.028569	JURKAT	Leukemia	Blood	Leukemia
ACH-0003	2.266037	SKM1	Leukemia	Blood	Leukemia
ACH-0009	0.124328	SUPT1	Leukemia	Blood	Leukemia
ACH-0007	3.157044	KMRC2	Kidney Ca	Kidney	Kidney Cancer

ACH-0008	2.313246	FADU	Head and	Upper Aer	Head and Neck Cancer
ACH-0017	0.111031	VAESBJ	Sarcoma	Soft Tissue	Sarcoma
ACH-0003	4.476382	JHOM2B	Ovarian C	Ovary	Ovarian Cancer
ACH-0020	0	P30OHK	Leukemia	Blood	Leukemia
ACH-0009	0.028569	NCIH2110	Lung Canc	Lung	Lung Cancer
ACH-0013	2.277985	SUM44PE	Breast Car	Breast	Breast Cancer
ACH-0009	2.495695	2313287	Gastric Ca	Gastric	Gastric Cancer
ACH-0020	0.782409	MEL285	Eye Cance	Eye	Eye Cancer
ACH-0009	0.536053	DND41	Leukemia	Blood	Leukemia
ACH-0006	0.516015	OVCAR4	Ovarian C	Ovary	Ovarian Cancer
ACH-0003	1.454176	NCIH2122	Lung Canc	Lung	Lung Cancer
ACH-0012	0.226509	TTC1240	Rhabdoid	Soft Tissue	Rhabdoid
ACH-0018	2.353323	ICC10	Bile Duct (	Bile Duct	Bile Duct Cancer
ACH-0003	1.327687	LUDLU1	Lung Canc	Lung	Lung Cancer
ACH-0004	1.275007	PK45H	Pancreatic	Pancreas	Pancreatic Cancer
ACH-0010	5.209063	DLD1	Colon/Col	Colorectal	Colon/Colorectal Cancer
ACH-0000	0.056584	MJ	Lymphom	Lymphocy	Lymphoma
ACH-0004	5.631686	BCPAP	Thyroid Ca	Thyroid	Thyroid Cancer
ACH-0004	1.803227	NCIH1792	Lung Canc	Lung	Lung Cancer
ACH-0025	1.286881	MM16011	Skin Cance	Skin	Skin Cancer
ACH-0000	3.894333	RERFLCMS	Lung Canc	Lung	Lung Cancer
ACH-0024	0	RPE1SS6	Non-Canc	Eye	Non-Cancerous
ACH-0000	5.730096	HS172T	Non-Canc	Fibroblast	Non-Cancerous
ACH-0005	3.142413	BT20	Breast Car	Breast	Breast Cancer
ACH-0000	0.042644	HS706T	Non-Canc	Fibroblast	Non-Cancerous
ACH-0020	0.815575	UACC62SI	Skin Cance	Skin	Skin Cancer
ACH-0008	6.324451	HCC15	Lung Canc	Lung	Lung Cancer
ACH-0013	0.084064	PACADD1	Pancreatic	Pancreas	Pancreatic Cancer
ACH-0020	0	P2URK562	Leukemia	Blood	Leukemia
ACH-0003	1.739848	SKRC20	Kidney Ca	Kidney	Kidney Cancer
ACH-0004	3.643856	SNU1197	Colon/Col	Colorectal	Colon/Colorectal Cancer
ACH-0004	4.1152	NCIN87	Gastric Ca	Gastric	Gastric Cancer
ACH-0009	0.056584	NALM6	Leukemia	Blood	Leukemia
ACH-0004	0.014355	NALM1	Leukemia	Blood	Leukemia
ACH-0009	3.273516	SKUT1	Sarcoma	Soft Tissue	Sarcoma
ACH-0003	6.224966	SNU620	Gastric Ca	Gastric	Gastric Cancer
ACH-0005	3.144046	HCC33	Lung Canc	Lung	Lung Cancer
ACH-0010	0.014355	CHLA57	Unknown	Central Ne	Unknown
ACH-0009	4.140779	IM95	Gastric Ca	Gastric	Gastric Cancer
ACH-0008	5.083639	DBTRG05M	Brain Canc	Central Ne	Brain Cancer
ACH-0006	4.277241	HMEL	Non-Canc	Breast	Non-Cancerous
ACH-0005	3.420887	SNU16	Gastric Ca	Gastric	Gastric Cancer
ACH-0010	3.28244	CW9019	Sarcoma	Soft Tissue	Sarcoma
ACH-0018	3.543496	C396	Bone Canc	Bone	Bone Cancer
ACH-0013	4.088311	RO82W1	Thyroid Ca	Thyroid	Thyroid Cancer
ACH-0007	4.779785	HLFA	Non-Canc	Fibroblast	Non-Cancerous
ACH-0002	0.056584	MINO	Lymphom	Lymphocy	Lymphoma
ACH-0015	0	KARPAS17	Lymphom	Lymphocy	Lymphoma
ACH-0008	5.236493	WM793	Skin Cance	Skin	Skin Cancer
ACH-0009	0.042644	ISHIKAWA	Endometri	Uterus	Endometrial/Uterine Cancer
ACH-0007	1.956057	HSC3	Head and	Upper Aer	Head and Neck Cancer
ACH-0008	0.028569	HOP92	Lung Canc	Lung	Lung Cancer
ACH-0007	2.280956	CORL95	Lung Canc	Lung	Lung Cancer
ACH-0001	3.729009	HDMYZ	Unknown	Unknown	Unknown
ACH-0018	5.557349	MFM223	Breast Car	Breast	Breast Cancer
ACH-0001	0.124328	A4FUK	Lymphom	Lymphocy	Lymphoma
ACH-0017	3.353323	SNU1544	Colon/Col	Colorectal	Colon/Colorectal Cancer
ACH-0002	3.670161	COLO201	Colon/Col	Colorectal	Colon/Colorectal Cancer

ACH-0013	2.825786	PACADD1	Pancreatic	Pancreas	Pancreatic Cancer
ACH-0009	2.704872	SW48	Colon/Col	Colorectal	Colon/Colorectal Cancer
ACH-0002	1.201634	BICR31	Head and	Upper Aer	Head and Neck Cancer
ACH-0002	0.097611	SNU213	Pancreatic	Pancreas	Pancreatic Cancer
ACH-0017	3.49057	TTC442	Sarcoma	Soft Tissue	Sarcoma
ACH-0001	2.78031	CAL62	Thyroid Ca	Thyroid	Thyroid Cancer
ACH-0002	0.028569	BL41	Lymphom	Lymphocy	Lymphoma
ACH-0012	0	SCCOHT1	Ovarian C	Ovary	Ovarian Cancer
ACH-0006	0.056584	JHH1	Liver Canc	Liver	Liver Cancer
ACH-0008	0.226509	T3M10	Lung Canc	Lung	Lung Cancer
ACH-0015	1.560715	HCA1	Cervical C	Cervix	Cervical Cancer
ACH-0001	0.575312	NOMO1	Leukemia	Blood	Leukemia
ACH-0002	1.286881	CORL279	Lung Canc	Lung	Lung Cancer
ACH-0002	4.258519	DAOY	Brain Canc	Central Ne	Brain Cancer
ACH-0002	4.913608	A1207	Brain Canc	Central Ne	Brain Cancer
ACH-0020	3.269033	HOTHC	Thyroid Ca	Thyroid	Thyroid Cancer
ACH-0009	1.867896	MEWO	Skin Cance	Skin	Skin Cancer
ACH-0006	1.811471	SNU1066	Head and	Upper Aer	Head and Neck Cancer
ACH-0000	0.070389	GRANTA5	Lymphom	Lymphocy	Lymphoma
ACH-0002	0.641546	CAL120	Breast Car	Breast	Breast Cancer
ACH-0000	0.056584	G401	Rhabdoid	Kidney	Rhabdoid
ACH-0014	0.056584	CCLFPEDS	Sarcoma	Soft Tissue	Sarcoma
ACH-0000	3.049631	HS616T	Non-Canc	Fibroblast	Non-Cancerous
ACH-0019	0	9505BIK	Pancreatic	Pancreas	Pancreatic Cancer
ACH-0007	0.815575	ONS76	Brain Canc	Central Ne	Brain Cancer
ACH-0005	3.275007	NCIH2030	Lung Canc	Lung	Lung Cancer
ACH-0000	3.553361	CH157MN	Brain Canc	Central Ne	Brain Cancer
ACH-0005	1.735522	LN235	Brain Canc	Central Ne	Brain Cancer
ACH-0017	1.996389	GOTO	Neuroblas	Peripheral	Neuroblastoma
ACH-0008	0.028569	RH30	Sarcoma	Soft Tissue	Sarcoma
ACH-0016	0	PEA1	Ovarian C	Ovary	Ovarian Cancer
ACH-0003	0.070389	RI1	Lymphom	Lymphocy	Lymphoma
ACH-0002	4.925525	HS852T	Skin Cance	Skin	Skin Cancer
ACH-0013	1.827819	OCIP5X	Ovarian C	Ovary	Ovarian Cancer
ACH-0000	1.560715	HCC827GI	Lung Canc	Lung	Lung Cancer
ACH-0018	0.62293	ICC9	Bile Duct (	Bile Duct	Bile Duct Cancer
ACH-0006	7.114888	COLO829	Skin Cance	Skin	Skin Cancer
ACH-0014	0.918386	WPE1NA2	Prostate C	Prostate	Prostate Cancer
ACH-0005	1.280956	KMS26	Myeloma	Plasma Ce	Myeloma
ACH-0008	0.111031	MDAMB4(	Breast Car	Breast	Breast Cancer
ACH-0002	5.056584	A375	Skin Cance	Skin	Skin Cancer
ACH-0001	2.163499	HCC2935	Lung Canc	Lung	Lung Cancer
ACH-0018	0	ICC5	Bile Duct (	Bile Duct	Bile Duct Cancer
ACH-0003	6.204962	SW780	Bladder Ca	Urinary Tra	Bladder Cancer
ACH-0010	1.084064	D425	Brain Canc	Central Ne	Brain Cancer
ACH-0012	4.157044	SW982	Sarcoma	Soft Tissue	Sarcoma
ACH-0000	2.748461	OV56	Ovarian C	Ovary	Ovarian Cancer
ACH-0020	0.042644	MEL270	Eye Cance	Eye	Eye Cancer
ACH-0016	0.028569	PGA1	Leukemia	Blood	Leukemia
ACH-0004	0.163499	TUHR4TKI	Kidney Ca	Kidney	Kidney Cancer
ACH-0009	2.990955	NUGC3	Gastric Ca	Gastric	Gastric Cancer
ACH-0000	0.028569	ONCODG	Ovarian C	Ovary	Ovarian Cancer
ACH-0007	0.028569	KYSE30	Esophagea	Esophagus	Esophageal Cancer
ACH-0017	0.963474	RH4	Sarcoma	Soft Tissue	Sarcoma
ACH-0003	6.918386	SKNBE2	Neuroblas	Peripheral	Neuroblastoma
ACH-0000	4.936873	T3M4	Pancreatic	Pancreas	Pancreatic Cancer
ACH-0004	0.111031	OCIMY7	Myeloma	Plasma Ce	Myeloma
ACH-0008	3.571677	NCIH1568	Lung Canc	Lung	Lung Cancer

ACH-0002	0.163499	HDLM2	Lymphom	Lymphocy	Lymphoma
ACH-0004	3.605257	SW1116	Colon/Col	Colorectal	Colon/Colorectal Cancer
ACH-0004	0.15056	NCIH1694	Lung Canc	Lung	Lung Cancer
ACH-0001	0.263034	M059K	Brain Canc	Central Ne	Brain Cancer
ACH-0009	2.435629	JHUEM2	Endometri	Uterus	Endometrial/Uterine Cancer
ACH-0005	0.575312	WSUDLCL	Lymphom	Lymphocy	Lymphoma
ACH-0005	2.869871	COLO741	Skin Cance	Skin	Skin Cancer
ACH-0004	0.042644	SNU8	Ovarian C	Ovary	Ovarian Cancer
ACH-0004	0.042644	OVKATE	Ovarian C	Ovary	Ovarian Cancer
ACH-0015	1.851999	MM426	Skin Cance	Skin	Skin Cancer
ACH-0009	4.271276	HT115	Colon/Col	Colorectal	Colon/Colorectal Cancer
ACH-0013	1.956057	C4II	Cervical C	Cervix	Cervical Cancer
ACH-0000	1.565597	PATU8988	Pancreatic	Pancreas	Pancreatic Cancer
ACH-0020	0	SLVL	Lymphom	Lymphocy	Lymphoma
ACH-0024	0	RPE1SS48	Non-Canc	Eye	Non-Cancerous
ACH-0001	0.014355	JM1	Leukemia	Blood	Leukemia
ACH-0012	3.938286	YAMATO	Sarcoma	Soft Tissue	Sarcoma
ACH-0006	1.176323	KYM1	Rhabdoid	Soft Tissue	Rhabdoid
ACH-0026	0.695994	CCC5	Gastric Ca	Gastric	Gastric Cancer
ACH-0007	8.499965	DMS79	Lung Canc	Lung	Lung Cancer
ACH-0012	0.659925	TTC549	Rhabdoid	Soft Tissue	Rhabdoid
ACH-0015	2.263034	H413	Head and	Upper Aer	Head and Neck Cancer
ACH-0001	3.087463	OCIAML2	Leukemia	Blood	Leukemia
ACH-0003	0.903038	NCIH3122	Lung Canc	Lung	Lung Cancer
ACH-0005	0.042644	KMS21BM	Myeloma	Plasma Ce	Myeloma
ACH-0000	1.367371	OPM1	Myeloma	Plasma Ce	Myeloma
ACH-0002	1.933573	KP2	Pancreatic	Pancreas	Pancreatic Cancer
ACH-0003	0.014355	MOLM6	Leukemia	Blood	Leukemia
ACH-0009	3.971773	LS411N	Colon/Col	Colorectal	Colon/Colorectal Cancer
ACH-0004	2.693766	LU99	Lung Canc	Lung	Lung Cancer
ACH-0003	2.124328	QGP1	Pancreatic	Pancreas	Pancreatic Cancer
ACH-0013	4.478325	SW626	Colon/Col	Colorectal	Colon/Colorectal Cancer
ACH-0010	0	KARPAS38	Lymphom	Lymphocy	Lymphoma
ACH-0001	3.435629	SLR20	Bladder Ca	Urinary Tr	Bladder Cancer
ACH-0019	1.090853	GB2	Gallbladde	Bile Duct	Gallbladder Cancer
ACH-0017	1.599318	95T1000	Liposarcor	Soft Tissue	Liposarcoma
ACH-0003	0.042644	PK1	Pancreatic	Pancreas	Pancreatic Cancer
ACH-0010	0.757023	CHLA218	Bone Canc	Bone	Bone Cancer
ACH-0005	4.563158	LN229	Brain Canc	Central Ne	Brain Cancer
ACH-0015	3.995485	KMLS1	Liposarcor	Soft Tissue	Liposarcoma
ACH-0002	4.618826	NCIH2029	Lung Canc	Lung	Lung Cancer
ACH-0007	0.956057	BICR56	Head and	Upper Aer	Head and Neck Cancer
ACH-0005	3.414136	SNU46	Head and	Upper Aer	Head and Neck Cancer
ACH-0003	1.104337	TE15	Esophagea	Esophagus	Esophageal Cancer
ACH-0003	0.042644	SKMM2	Myeloma	Plasma Ce	Myeloma
ACH-0003	5.75007	COLO678	Colon/Col	Colorectal	Colon/Colorectal Cancer
ACH-0000	0.344828	KARPAS29	Lymphom	Lymphocy	Lymphoma
ACH-0015	0.176323	KOSC2	Head and	Upper Aer	Head and Neck Cancer
ACH-0002	3.044394	SLR23	Kidney Ca	Kidney	Kidney Cancer
ACH-0006	0.014355	OVTOKO	Ovarian C	Ovary	Ovarian Cancer
ACH-0003	0.042644	JVM3	Leukemia	Blood	Leukemia
ACH-0009	3.31904	SNUC5	Colon/Col	Colorectal	Colon/Colorectal Cancer
ACH-0013	0.124328	C33A	Cervical C	Cervix	Cervical Cancer
ACH-0007	0.731183	BICR22	Head and	Upper Aer	Head and Neck Cancer
ACH-0000	5.655638	SKNMC	Bone Canc	Bone	Bone Cancer
ACH-0004	1.15056	LI7	Liver Canc	Liver	Liver Cancer
ACH-0008	3.764474	HS698T	Non-Canc	Fibroblast	Non-Cancerous
ACH-0002	0.042644	OV90	Ovarian C	Ovary	Ovarian Cancer

ACH-0014	2.400538	BPH1	Prostate C	Prostate	Prostate Cancer
ACH-0014	2.313246	C75	Colon/Col	Colorectal	Colon/Colorectal Cancer
ACH-0008	0.014355	NCIH2009	Lung Canc	Lung	Lung Cancer
ACH-0008	1.226509	NCIH1793	Lung Canc	Lung	Lung Cancer
ACH-0006	1.084064	SCLC21H	Lung Canc	Lung	Lung Cancer
ACH-0000	0.042644	PC14	Lung Canc	Lung	Lung Cancer
ACH-0012	1.157044	SUMB002	Brain Canc	Central Ne	Brain Cancer
ACH-0002	0.056584	SUDHL10	Lymphom	Lymphocy	Lymphoma
ACH-0005	1.899176	SNU1076	Head and	Upper Aer	Head and Neck Cancer
ACH-0005	2.238787	A498	Kidney Ca	Kidney	Kidney Cancer
ACH-0004	3.082362	SNU387	Liver Canc	Liver	Liver Cancer
ACH-0007	4.31034	NCIH727	Lung Canc	Lung	Lung Cancer
ACH-0000	0.056584	EHEB	Lymphom	Lymphocy	Lymphoma
ACH-0011	1.405992	KPMRTRY	Rhabdoid	Soft Tissue	Rhabdoid
ACH-0014	1.333424	A388	Skin Cance	Epidermoi	Skin Cancer
ACH-0005	3.080658	NCIH1836	Lung Canc	Lung	Lung Cancer
ACH-0005	1.176323	NCIH1299	Lung Canc	Lung	Lung Cancer
ACH-0020	2.553361	HSPSS	Sarcoma	Central Ne	Sarcoma
ACH-0006	4.777157	LN464	Brain Canc	Central Ne	Brain Cancer
ACH-0026	2.720278	JVE127	Colon/Col	Colorectal	Colon/Colorectal Cancer
ACH-0008	1.238787	DMS454	Lung Canc	Lung	Lung Cancer
ACH-0009	4.384741	DV90	Lung Canc	Lung	Lung Cancer
ACH-0024	0	RPE1SS11	Non-Canc	Eye	Non-Cancerous
ACH-0002	1.495695	SLR24	Kidney Ca	Kidney	Kidney Cancer
ACH-0010	2.560715	CHLA99	Bone Canc	Bone	Bone Cancer
ACH-0005	0.214125	SNU1272	Kidney Ca	Kidney	Kidney Cancer
ACH-0010	0	DL	Rhabdoid	Soft Tissue	Rhabdoid
ACH-0009	5.624101	SNUC4	Colon/Col	Colorectal	Colon/Colorectal Cancer
ACH-0001	2.032101	RD	Sarcoma	Soft Tissue	Sarcoma
ACH-0014	2.596935	SW756	Cervical C	Cervix	Cervical Cancer
ACH-0007	0.070389	MCAS	Ovarian C	Ovary	Ovarian Cancer
ACH-0001	0.028569	HCC2429	Lung Canc	Lung	Lung Cancer
ACH-0000	6.142618	HS683	Brain Canc	Central Ne	Brain Cancer
ACH-0006	5.267536	SKMEL31	Skin Cance	Skin	Skin Cancer
ACH-0016	6.613974	TGW	Neuroblas	Peripheral	Neuroblastoma
ACH-0016	0.298658	UPCISCC1	Head and	Upper Aer	Head and Neck Cancer
ACH-0020	3.958843	A375SKIN	Skin Cance	Skin	Skin Cancer
ACH-0020	2.990955	MP46	Eye Cance	Eye	Eye Cancer
ACH-0009	3.915521	SNUC2A	Colon/Col	Colorectal	Colon/Colorectal Cancer
ACH-0004	4.566206	YH13	Brain Canc	Central Ne	Brain Cancer
ACH-0013	0.799087	JOPACA1	Pancreatic	Pancreas	Pancreatic Cancer
ACH-0015	0.014355	MOLM14	Leukemia	Blood	Leukemia
ACH-0000	2.748461	ZR751	Breast Car	Breast	Breast Cancer
ACH-0002	0.042644	COV318	Ovarian C	Ovary	Ovarian Cancer
ACH-0009	7.136581	LNCAPCL(	Prostate C	Prostate	Prostate Cancer
ACH-0009	0.042644	HEC151	Endometri	Uterus	Endometrial/Uterine Cancer
ACH-0013	4.457463	NMB	Neuroblas	Peripheral	Neuroblastoma
ACH-0003	3.333424	CORL24	Lung Canc	Lung	Lung Cancer
ACH-0005	2.871844	UACC257	Skin Cance	Skin	Skin Cancer
ACH-0015	0.014355	JMURTK2	Rhabdoid	Kidney	Rhabdoid
ACH-0006	0.163499	CORL47	Lung Canc	Lung	Lung Cancer
ACH-0017	4.07382	94T778	Liposarcor	Soft Tissue	Liposarcoma
ACH-0015	3.203201	H376	Head and	Upper Aer	Head and Neck Cancer
ACH-0005	0.15056	TCCPAN2	Pancreatic	Pancreas	Pancreatic Cancer
ACH-0001	0.042644	L363	Myeloma	Plasma Ce	Myeloma
ACH-0004	3.294253	TE5	Esophagea	Esophagus	Esophageal Cancer
ACH-0014	3.084064	ESO26	Esophagea	Esophagus	Esophageal Cancer
ACH-0007	4.871844	A2058	Skin Cance	Skin	Skin Cancer

ACH-0016	4.372952	ONDA7	Brain Canc	Central Ne	Brain Cancer
ACH-0004	0.400538	SNU216	Gastric Ca	Gastric	Gastric Cancer
ACH-0015	3.215679	MERO83	Lung Canc	Lung	Lung Cancer
ACH-0000	0.176323	HEL9217	Leukemia	Blood	Leukemia
ACH-0016	2.201634	UMRC3	Kidney Ca	Kidney	Kidney Cancer
ACH-0011	0	OVCAR5	Ovarian Ca	Ovary	Ovarian Cancer
ACH-0007	0.028569	RMUGS	Ovarian Ca	Ovary	Ovarian Cancer
ACH-0005	3.389567	HEYA8	Ovarian Ca	Ovary	Ovarian Cancer
ACH-0001	5.19928	KG1C	Brain Canc	Central Ne	Brain Cancer
ACH-0016	3.408712	UHO1	Lymphom	Lymphocy	Lymphoma
ACH-0009	0.056584	SNU324	Pancreatic	Pancreas	Pancreatic Cancer
ACH-0000	0.070389	REC1	Lymphom	Lymphocy	Lymphoma
ACH-0000	0.367371	NCIH1581	Lung Canc	Lung	Lung Cancer
ACH-0011	2.02148	L82	Lymphom	Lymphocy	Lymphoma
ACH-0004	0.042644	F36P	Leukemia	Blood	Leukemia
ACH-0001	0.111031	SUPT11	Leukemia	Blood	Leukemia
ACH-0002	5.209453	BT549	Breast Car	Breast	Breast Cancer
ACH-0018	2.722466	COLO824	Breast Car	Breast	Breast Cancer
ACH-0007	0.070389	SEM	Leukemia	Blood	Leukemia
ACH-0016	0.028569	PEO4	Ovarian Ca	Ovary	Ovarian Cancer
ACH-0003	0.137504	JEKO1	Lymphom	Lymphocy	Lymphoma
ACH-0000	3.372952	TE125T	Non-Canc	Fibroblast	Non-Cancerous
ACH-0010	0.014355	CHLA266	Rhabdoid	Soft Tissue	Rhabdoid
ACH-0010	3.317594	COV504	Ovarian Ca	Ovary	Ovarian Cancer
ACH-0004	0.028569	MOLP2	Myeloma	Plasma Ce	Myeloma
ACH-0007	0.111031	RERFLCAD	Lung Canc	Lung	Lung Cancer
ACH-0005	0.464668	ST486	Lymphom	Lymphocy	Lymphoma
ACH-0013	2.395063	SUM159PT	Breast Car	Breast	Breast Cancer
ACH-0010	1.948601	NCIH292	Lung Canc	Lung	Lung Cancer
ACH-0001	0.028569	ALLSIL	Leukemia	Blood	Leukemia
ACH-0005	0.137504	AML193	Leukemia	Blood	Leukemia
ACH-0001	4.349082	TE159T	Non-Canc	Fibroblast	Non-Cancerous
ACH-0013	5.488322	MB1	Thyroid Ca	Thyroid	Thyroid Cancer
ACH-0018	1.918386	ICC4	Bile Duct C	Bile Duct	Bile Duct Cancer
ACH-0013	1.731183	H103	Head and	Upper Aer	Head and Neck Cancer
ACH-0008	1.867896	TOV21G	Ovarian Ca	Ovary	Ovarian Cancer
ACH-0009	2.169925	NCIH23	Lung Canc	Lung	Lung Cancer
ACH-0006	2.981853	NCIH1355	Lung Canc	Lung	Lung Cancer
ACH-0002	4.757023	JHH6	Liver Canc	Liver	Liver Cancer
ACH-0008	0.084064	HCC515	Lung Canc	Lung	Lung Cancer
ACH-0016	0	RCK8	Lymphom	Lymphocy	Lymphoma
ACH-0012	3.984589	UPCISCC1	Head and	Upper Aer	Head and Neck Cancer
ACH-0001	3.904002	SCC9	Head and	Upper Aer	Head and Neck Cancer
ACH-0001	6.606738	VCAP	Prostate C	Prostate	Prostate Cancer
ACH-0001	0.056584	NALM19	Leukemia	Blood	Leukemia
ACH-0001	0.111031	NCIH2405	Lung Canc	Lung	Lung Cancer
ACH-0017	3.307429	PFSK1	Brain Canc	Central Ne	Brain Cancer
ACH-0009	5.525443	MDAMB30	Breast Car	Breast	Breast Cancer
ACH-0009	0.014355	HEC251	Endometri	Uterus	Endometrial/Uterine Cancer
ACH-0015	1.863938	JAR	Endometri	Uterus	Endometrial/Uterine Cancer
ACH-0017	0.176323	RC2	Sarcoma	Soft Tissue	Sarcoma
ACH-0003	1.516015	PSN1	Pancreatic	Pancreas	Pancreatic Cancer
ACH-0005	4.408032	RKN	Sarcoma	Soft Tissue	Sarcoma
ACH-0007	1.226509	NCIH2023	Lung Canc	Lung	Lung Cancer
ACH-0000	0.137504	SUPB15	Leukemia	Blood	Leukemia
ACH-0009	0.201634	CMLT1	Leukemia	Blood	Leukemia
ACH-0002	0.641546	NB4	Leukemia	Blood	Leukemia
ACH-0004	4.129283	SNU475	Liver Canc	Liver	Liver Cancer

ACH-0002	1.62293	NMCG1	Brain Canc	Central Ne	Brain Cancer
ACH-0000	0.731183	ACHN	Kidney Ca	Kidney	Kidney Cancer
ACH-0009	3.223423	COLO792	Skin Cance	Skin	Skin Cancer
ACH-0008	5.539469	NCIH1436	Lung Canc	Lung	Lung Cancer
ACH-0001	0.85599	SU8686	Pancreatic	Pancreas	Pancreatic Cancer
ACH-0004	0.042644	BL70	Lymphom	Lymphocy	Lymphoma
ACH-0015	0.367371	NCCIT	Embryona	Embryo	Embryonal Cancer
ACH-0000	4.040892	U343	Brain Canc	Central Ne	Brain Cancer
ACH-0009	3.168321	639V	Bladder Ca	Urinary Tr	Bladder Cancer
ACH-0011	0.028569	MON	Rhabdoid	Soft Tissue	Rhabdoid
ACH-0007	1.655352	LOXIMVI	Skin Cance	Skin	Skin Cancer
ACH-0002	0.545968	TOLEDO	Lymphom	Lymphocy	Lymphoma
ACH-0026	0.641546	KP363T	Colon/Col	Colorectal	Colon/Colorectal Cancer
ACH-0018	1.500802	SSP25	Bile Duct (	Bile Duct	Bile Duct Cancer
ACH-0019	1.400538	ECC2	Bile Duct (	Bile Duct	Bile Duct Cancer
ACH-0000	1.182692	A673	Bone Canc	Bone	Bone Cancer
